# Pentose Phosphate Pathway Function Affects Tolerance to the G-Quadruplex Binder TMPyP4

**DOI:** 10.1371/journal.pone.0066242

**Published:** 2013-06-12

**Authors:** Elizabeth J. Andrew, Stephanie Merchan, Conor Lawless, A. Peter Banks, Darren J. Wilkinson, David Lydall

**Affiliations:** 1 Institute for Cell and Molecular Biosciences, Newcastle University Medical School, Newcastle Upon Tyne, United Kingdom; 2 School of Mathematics and Statistics, Newcastle University, Newcastle Upon Tyne, United Kingdom; Texas A&M University, United States of America

## Abstract

G-quadruplexes form in guanine-rich regions of DNA and the presence of these structures at telomeres prevents the activity of telomerase *in vitro*. Ligands such as the cationic porphyrin TMPyP4 stabilise G-quadruplexes and are therefore under investigation for their potential use as anti-cancer drugs. In order to investigate the mechanism of action of TMPyP4 *in vivo*, we carried out a genome-wide screen in the budding yeast *Saccharomyces cerevisiae*. We found that deletion of key pentose phosphate pathway (PPP) genes increased the sensitivity of yeast to the presence of TMPyP4. The PPP plays an important role in the oxidative stress response and sensitivity to TMPyP4 also increased when genes involved in the oxidative stress response, *CCS1* and *YAP1*, were deleted. For comparison we also report genome wide-screens using hydrogen peroxide, which causes oxidative stress, RHPS4, another G-quadruplex binder and hydroxyurea, an S phase poison. We found that a number of TMPyP4-sensitive strains are also sensitive to hydrogen peroxide in a genome-wide screen. Overall our results suggest that treatment with TMPyP4 results in light-dependent oxidative stress response in budding yeast, and that this, rather than G-quadruplex binding, is the major route to cytotoxicity. Our results have implications for the usefulness and mechanism of action of TMPyP4.

## Introduction

**Figure 1:**
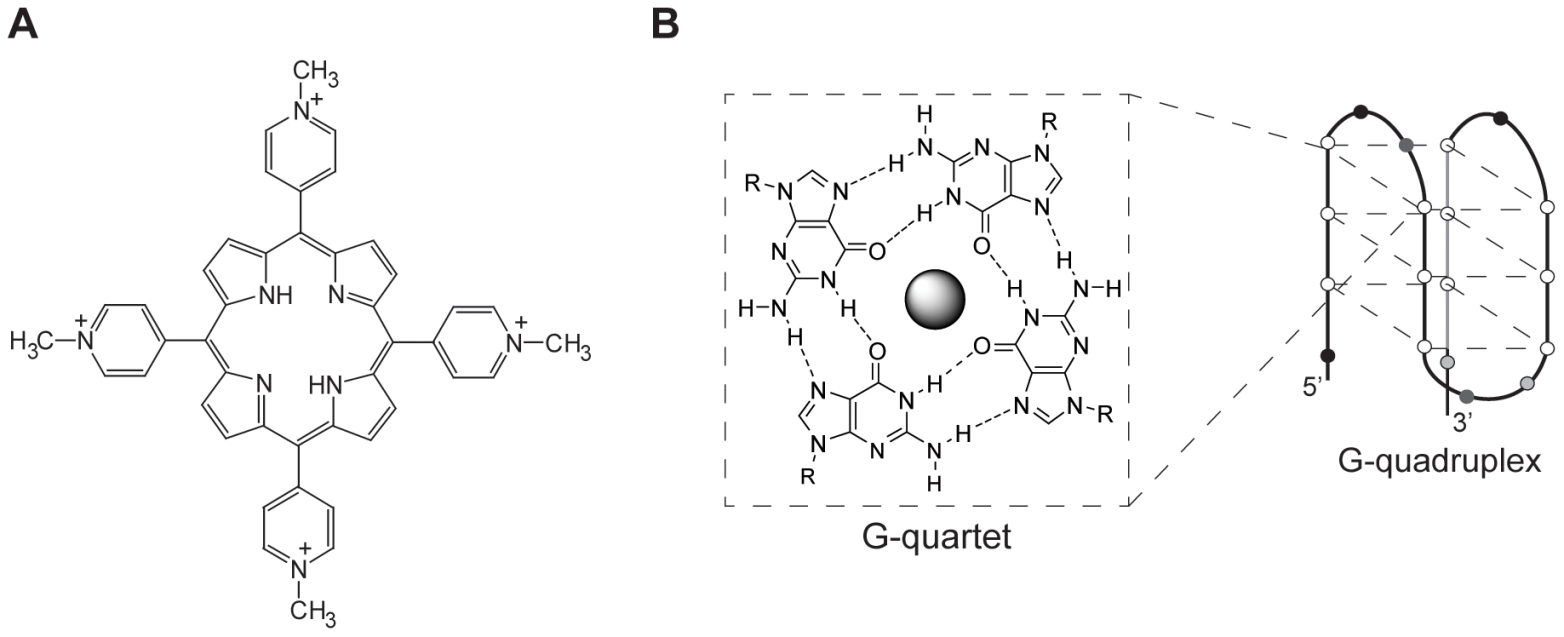
TMPyP4, G-quartet, and G-quadruplex structures (A) The structure of the porphyrin TMPyP4 (B) The structure of G-quartets, adapted from [51] and an example of a G-quadruplex structure. **The sphere in the centre of the G-quartet represents a central cation.**

TMPyP4 (5,10,15,20-tetratkis-(N-methyl-4-pyridyl)-21,23-H-porphyrin) (Figure 1a) is a widely used G-quadruplex binding molecule. The interaction between the porphyrin TMPyP4 and nucleic acid structures which form in guanine-rich regions of DNA and RNA, known as G-quadruplexes (Figure 1b), has been studied extensively [1], [2], [3], [4]. TMPyP4 exhibits the ability to bind and stabilise G-quadruplexes *in vitro*, binding to the exterior of the structure by end stacking [5], [6]. G-quadruplexes are predicted to form within telomeric regions due to their guanine-rich nature [7], [8]. Accordingly, it has been observed that TMPyP4 has the ability to inhibit telomerase activity *in vitro* and affect the c-MYC oncogene-dependent transcription of several genes in HeLa cells, including TERT, which encodes the human telomerase subunit [9], [10], [11]. This interaction with c-MYC suggests that the promoter region has G-quadruplex forming potential [12]. TMPyP4 interacts strongly with G-quadruplexes; however, the selectivity of TMPyP4 for these structures is comparatively poor versus duplex DNA [13], [14], [15]. In addition, the formation of G-quadruplexes *in vivo* is undetermined, and thus the capability of TMPyP4 to bind G-quadruplexes *in vivo* is also unknown [16].

TMPyP4 is a member of the porphyrin family of compounds. Porphyrins typically bind metal ions to form organometallic complexes such as heme, which contains a central iron atom and forms part of haemoglobin. TMPyP4 is able to form a number of different metal complexes; interestingly, the nature of the metal ion within the complex can influence the stacking interaction of TMPyP4 and the degree of telomerase inhibition [17]. Porphyrin derivatives are commonly used as photosensitizers in photodynamic therapy; porphyrins such as PHOTOFRIN® and Visudyne have been used in the treatment of age-related macular degeneration and cancer due to their ability to produce reactive oxygen species (ROS) upon exposure to light [18]. This ROS production can also lead to the cleavage of DNA, and photocleavage in this manner has been used in photodynamic cancer therapy to fragment DNA in malignant cells [19], [20], [21]. Therefore TMPyP4 may cause cytotoxicity either because of its effects on G-quadruplex structures, by catalysing ROS production, by both mechanisms or by alternative mechanisms.

To better understand the mechanism of TMPyP4 toxicity, we chose to study the effect of treating the budding yeast *Saccharomyces cerevisiae* with TMPyP4. Using a genomic single deletion library we identified 19 ORFs whose deletion lead to an increased TMPyP4-sensitivity in comparison to the wild type. Among these genes were *RPE1*, *TKL1* and *ZWF1*, which encode key pentose phosphate pathway (PPP) enzymes. The PPP has a role in the production of nucleotides and in protection against the presence of reactive oxygen species. Other gene deletions which displayed sensitivity to TMPyP4 are linked to the response to oxidative stress, for example *CCS1* and *YAP1*. Our data suggests that the presence of TMPyP4 induces the production of ROS. This theory is supported by results from parallel screens carried out on media containing hydrogen peroxide (H_2_O_2_) and media containing an alternative G-quadruplex binding ligand, RHPS4 [22]. A greater similarity in differential sensitivity was observed between TMPyP4- and H_2_O_2_- treatment than between the two G-quadruplex binding ligands. We also found that sensitivity to TMPyP4 substantially increases upon exposure to light, even at very low TMPyP4 concentration, consistent with the hypothesis that light-dependent ROS production is important in TMPyP4 treated cells.

## Materials and Methods

### Yeast Culture Conditions

All strains used in this study are listed in Table S1 in File S1. The single gene deletion collection was stored at −80°C in 384-well plates (Greiner BioOne) in 15% glycerol [23]. Yeast strains were cultured in complete synthetic media (CSM) with appropriate amino-acids and G418 (final concentration, 200 µg/ml) added. W303 genetic background strains were cultured in YEPD (ade). Plate filling and robotics were performed as described previously [24].

### High-throughput Culturing

Cultures were inoculated onto solid agar plates and photographed repeatedly to construct growth curves, as described previously [24]. Briefly, colonies were inoculated from the solid agar single gene deletion collection plates into 96-well plates containing 200 µl CSM supplemented with G418 media in each well. Cultures were grown to saturation for 3 days, without shaking, at 23°C. Cultures were resuspended, diluted approximately 1∶100 in 200 µl H_2_O and spotted in parallel onto solid CSM or CSM supplemented with 100 µM TMPyP4 (dissolved in H_2_O), 1.5 mM H_2_O_2_ (dissolved in H_2_O), 100 mM HU (dissolved in H_2_O) or 200 µM RHPS4 (dissolved in 1% DMSO) plates.

### Quantitative Fitness Analysis (QFA)

Plates were incubated at 30°C for 5 days in an S&P robotics automated, integrated imager and incubator and photographed at 6 hour intervals. The image analysis tool Colonyzer [25] was used to quantify cell density for each culture from captured photographs. The QFA R package [http://research.ncl.ac.uk/qfa] was used to assemble growth curves, fit a generalised logistic model to cell density dynamics and to generate fitness summaries for each strain as described in [26], [27].

### Manual Spot Tests

Serial dilutions of cultures grown to saturation in YEPD (ade) onto solid media containing the indicated compounds. The plates were incubated for 3 days at the indicated temperatures.

### Light Experiments

All plates were incubated in a SANYO MIR-153 cooled incubator fitted with a 15W×1 fluorescent lamp for 3 days at 30°C. Control (dark) plates were wrapped in aluminium foil and placed in the same incubator as light-exposed plates. The fluorescent lamp was on for the duration of the experiment.

## Results

### Identification of Gene Deletions which Affect Tolerance of TMPyP4

**Figure 2:**
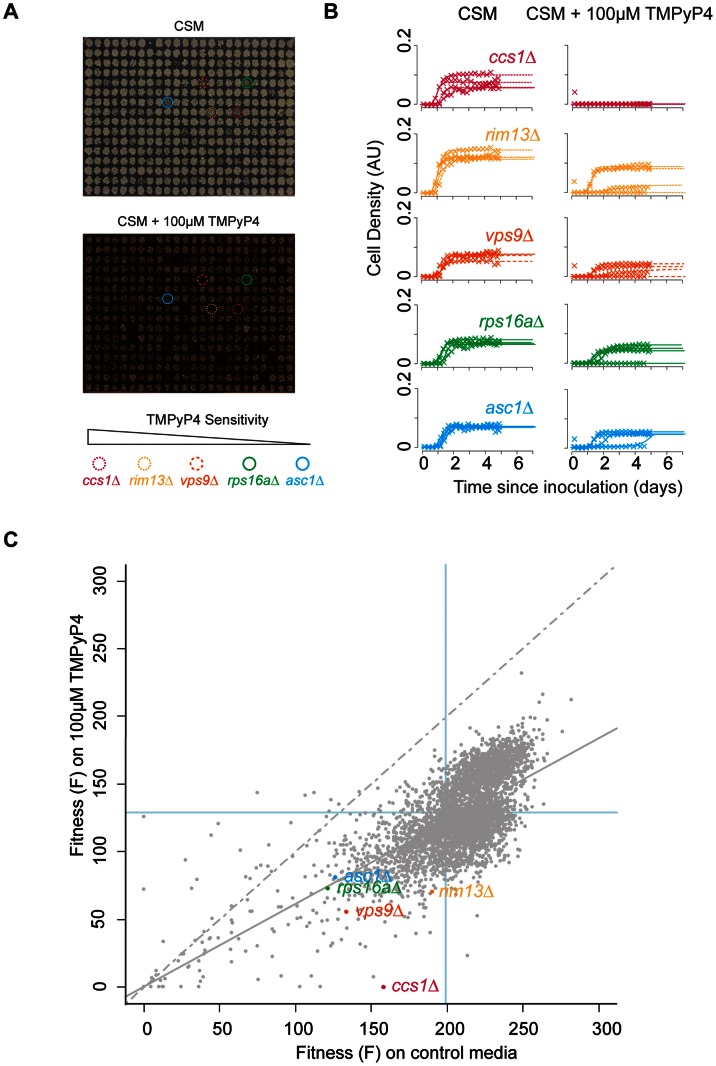
Fitness of *yfg*Δ strains on TMPyP4 (A) Plate 10 of the genome-wide screen grown on CSM and CSM containing 100 µM TMPyP4. Highlighted genes indicate five different deletion strains on the TMPyP4-containing plate. (B) Individual growth curves of the four repeats of the genes highlighted in (A) on CSM and CSM containing 100 µM TMPyP4. (C) The fitnesses of ∼4300 gene deletion mutants on media with or wihtout 100 µM TMPyP4. Each point on the graph represents a single deletion genotype. The dashed grey line indicates hypothetical 1∶1 growth under both conditions. The solid grey line indicates expected fitness based on a population model. Strains below this solid line display a reduced fitness on supplemented media than expected.

To understand the mechanism of action of TMPyP4, we carried out a genome-wide screen using a yeast single deletion library. We hypothesised that telomere-related and/or DNA repair genes might display differential sensitivity to the G-quadruplex binding ligand, since it has been demonstrated that TMPyP4 can inhibit telomerase *in vitro* [10], [11]. We chose to monitor growth at 30°C in the presence of 100 µM TMPyP4 as, under these conditions, fitness is around 30% inhibited. Preliminary studies established that this concentration was suitable to observe both increases and decreases in the fitness of strains relative to the wild type. We screened a genome-wide collection of around 4300 *Saccharomyces cerevisiae* gene deletion strains (*yfg*Δ, **y**our **f**avourite **g**ene deletion, which indicates any of the viable systematic gene deletions) for differential sensitivity to TMPyP4. Figure 2a is an example of one of 15 library plates (plate 10) used in the screen and demonstrates growth of strains in the presence and absence of 100 µM TMPyP4. The difference in colour between the control and treatment plates is caused by the deep purple colour of TMPyP4, a characteristic of many porphyrins. Four replicates of the screen were performed to identify the gene deletions affecting TMPyP4 sensitivity and quantitative fitness analysis (QFA) was performed as previously described [26].

Figure 2b displays growth curves of four replicates of specific strains highlighted in Figure 2a, with the most TMPyP4-sensitive genotype, *ccs1*Δ, at the top, and decreasing in sensitivity to *asc1*Δ at the bottom. The left-hand column shows growth over the time course on control media and the right-hand column shows growth on TMPyP4. As is clear from the growth curves, *ccs1*Δ strains grow well on control media, but upon incubation with TMPyP4, this strain ceases to grow. In comparison, the growth of *asc1*Δ strains decreases slightly when exposed to the G-quadruplex binding ligand. What is also clear from the growth curves is that variation in growth between the four replicates sometimes occur (visible, for instance, in the *vps9*Δ growth curves).

Maximum doubling rate (MDR) and maximum doubling potential (MDP) were estimated from the growth curves and culture fitness was defined as their product (Fitness, F, population doublings^2^/day). The mean fitness of each gene deletion strain on media supplemented with TMPyP4 was compared with mean fitness on media lacking the ligand, in each case from four replicates (Figure 2c). As is clear from the difference between the line of equal growth and the linear regression in Figure 2c, the growth of mutant strains on media supplemented with TMPyP4 is on average reduced compared to growth on media lacking the ligand (around 40% reduction in fitness, close to that expected). Vertical distance of each spot from the expected fitness linear regression model can be used to estimate TMPyP4-tolerance of each *yfg*Δ strain. We defined this value as the fitness differential (FD). Using an arbitrary cut-off, we classified those gene deletions which result in an FD of ≥0.5 as suppressing sensitivity to TMPyP4, and gene deletions which result in an FD of ≤−0.5 as enhancing sensitivity (entire data set available in Table S4 in File S1).

**Figure 3:**
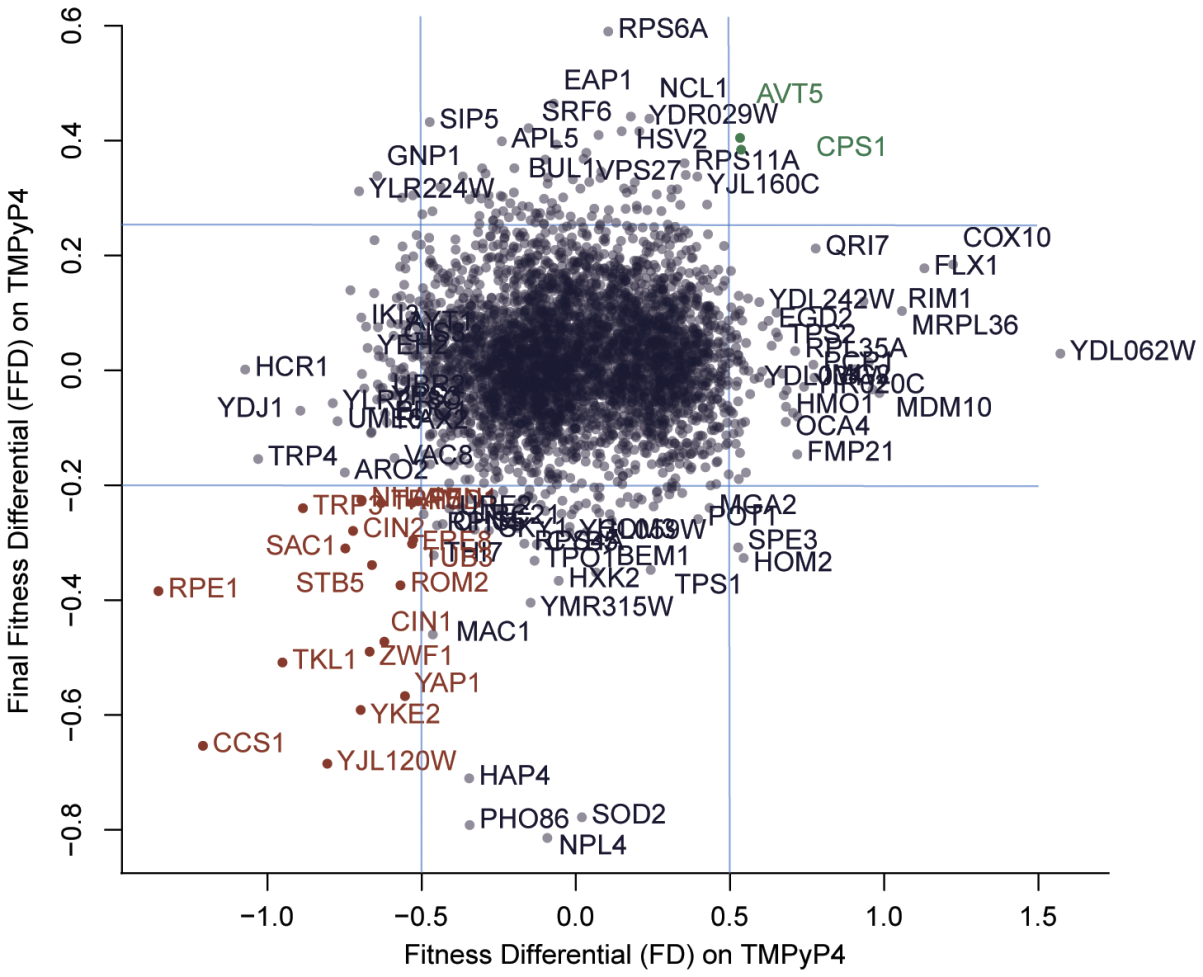
Comparison of TMPyP4 high-throughput screens. Correlation plot of fitness differential values from two screens (correlation = 0.066). Genes in red (bottom left-hand corner) were found to increase TMPyP4-sensitivity when deleted in both experiments (FD ≤−0.5 in the initial screen and FFD ≤−0.2 in the follow-up screen). Genes in green (top right-hand corner) were found to decrease TMPyP4-sensitivity when deleted according to both experiments (FD ≥0.5 in the initial screen and FFD ≥0.25 in the follow-up screen). Blue lines denote FD thresholds.

The results of the high-throughput screen were confirmed by repeating the screen using a simpler method of fitness analysis based on single time-point data. In the second screen we defined TMPyP4 tolerance as final fitness differential (FFD). An FFD of ≥0.25 indicates enhanced resistance to TMPyP4 and an FFD of ≤−0.2 indicates enhanced sensitivity (entire data set available in Table S5 in File S1). The results from the second screen were compared with the original screen (Figure 3). The comparison between the data sets identified 19 null mutations which reproducibly increased sensitivity to TMPyP4 in both screens (bottom left of the plot in Figure 3, and Table 1) and 2 which reduced sensitivity to the G-quadruplex binding ligand (top right of the same plot, and Table 1).

**Table 1:** Gene deletion strains with differential sensitivity to TMPyP4.

Sensitive		Resistant	
ORF	Gene	ORF	Gene
*YMR038C*	*CCS1*	*YBL089W*	*AVT5*
*YJL120W*	*YJL120W*	*YJL172W*	*CPS1*
*YPR074C*	*TKL1*		
*YJL121C*	*RPE1*		
*YLR200W*	*YKE2*		
*YNL241C*	*ZWF1*		
*YKL212W*	*SAC1*		
*YPL241C*	*CIN2*		
*YHR178W*	*STB5*		
*YOR349W*	*CIN1*		
*YKL211C*	*TRP3*		
*YLR371W*	*ROM2*		
*YML007W*	*YAP1*		
*YLR138W*	*NHA1*		
*YGL026C*	*TRP5*		
*YLR047C*	*FRE8*		
*YML124C*	*TUB3*		
*YML035C*	*AMD1*		
*YCR034W*	*FEN1*		

**Figure 4:**
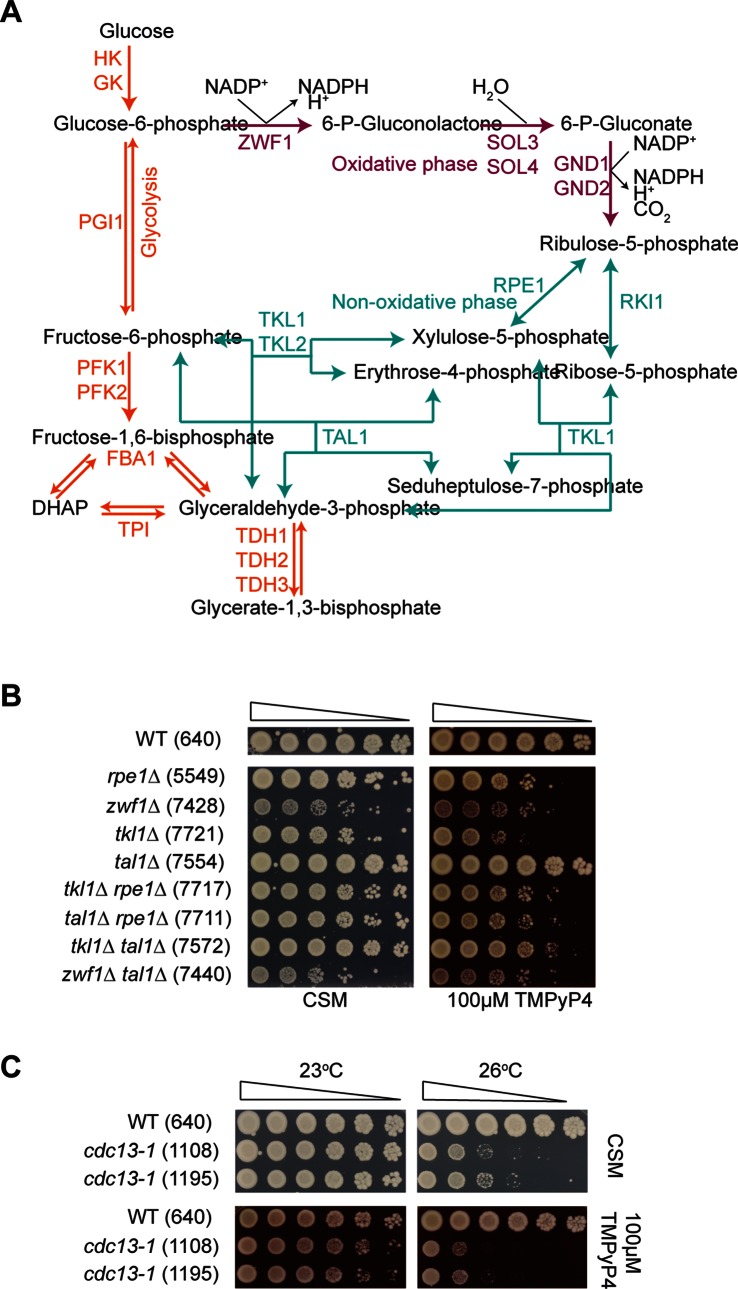
The pentose phosphate pathway protects against sensitivity to TMPyP4. (A) The pentose phosphate pathway. (B) Spot test for TMPyP4-sensitivity of *ppp*Δ strains in the W303 background. Strains were grown to saturation in YEPD before a 5-fold serial dilution and spotting onto plates with or without 100 µM TMPyP4. Incubation was carried out at 30°C for 3 days. (C) Spot test for *cdc13-1* interaction with TMPyP4. Strains were grown and spotted as in (B). Incubation was carried out at indicated temperatures for 3 days.

To identify pathways and mechanisms that affected cellular sensitivity to TMPyP4 we carried out Gene Ontology (GO) analysis of the 19 null mutations which caused an increase in TMPyP4-sensitivity. Over-represented terms include pentose phosphate shunt, nucleotide metabolic process and tryptophan biosynthetic process (Table S2 in File S1). 5 of the 19 TMPyP4-sensitive genes (*YJL120W, TKL1, RPE1, ZWF1* and *STB5*) are linked to the PPP (Figure 4a). The PPP plays an important role in the response to oxidative stress due to the production of NADPH, used to reduce antioxidants such as glutathione, in the oxidative phase of the pathway. Deletion of *AMD1* also results in sensitivity to TMPyP4– Amd1 catalyses the deamination of AMP to form IMP and ammonia, and therefore may be involved in regulation of intracellular adenine nucleotide pools. The precursor for nucleotide synthesis, ribose-5-phosphate, is produced by the PPP, and so the sensitivity caused by deletion of PPP-related genes may be linked to the nucleotide production process.

Other groups of functionally related single deletion strains demonstrate increased sensitivity to TMPyP4. Deletion of genes involved in tubulin folding and microtubule formation (*YKE2, TUB3, CIN1* and *CIN2*), tryptophan biosynthesis (*TRP3* and *TRP5*), and in the oxidative stress response (*CCS1* and YAP1) caused sensitivity to TMPyP4.

The remaining TMPyP4-sensitive genes in Table 1 encode proteins involved in phosphatidylinositol (PtdInsP) biosynthesis (*SAC1*), GDP/GTP exchange for Rho1 and Rho2 (*ROM2*), Na^+^/H^+^ transport (*NHA1*), iron homeostasis (*FRE8*) and fatty acid elongation (*FEN1*). Interestingly, we did not observe any telomere- or DNA damage response-related genes amongst those which displayed differential sensitivity to TMPyP4 in either screen.

### The Pentose Phosphate Pathway Provides Protection against TMPyP4 Treatment

Due to the number of pentose phosphate pathway (PPP) genes which demonstrated high sensitivity to TMPyP4 upon deletion, we were interested in investigating this pathway further. The main roles of the pentose phosphate pathway are in NADPH production and in the production of the nucleotide precursor ribose-5-phosphate. The results from our screen suggest that the PPP plays a role in the response to TMPyP4. Our screens were carried out in the S288C background, and so we wanted to confirm that PPP mutants are TMPyP4-sensitive in the *S. cerevisiae* W303 strain, which is very related but distinct from S288C [28]. Figure 4b demonstrates the sensitivity to TMPyP4 conferred by deletion of key PPP genes in W303, as well as the effect of deleting several PPP genes in the same strain. Deletion of *RPE1*, *TKL1* or *ZWF1* resulted in increased sensitivity to TMPyP4, consistent with the genome-wide screen. *TAL1* encodes a transaldolase which catalyses a reaction in the non-oxidative phase of the PPP, and in concordance with the screen results, deletion of this gene does not alter sensitivity to TMPyP4. This suggests that either the reaction Tal1 catalyses can be sufficiently carried out by a functional homologue (such as Nqm1 [29]) or that *TAL1* deletion does not result in metabolic changes that cause TMPyP4-sensitivity.

**Figure 5:**
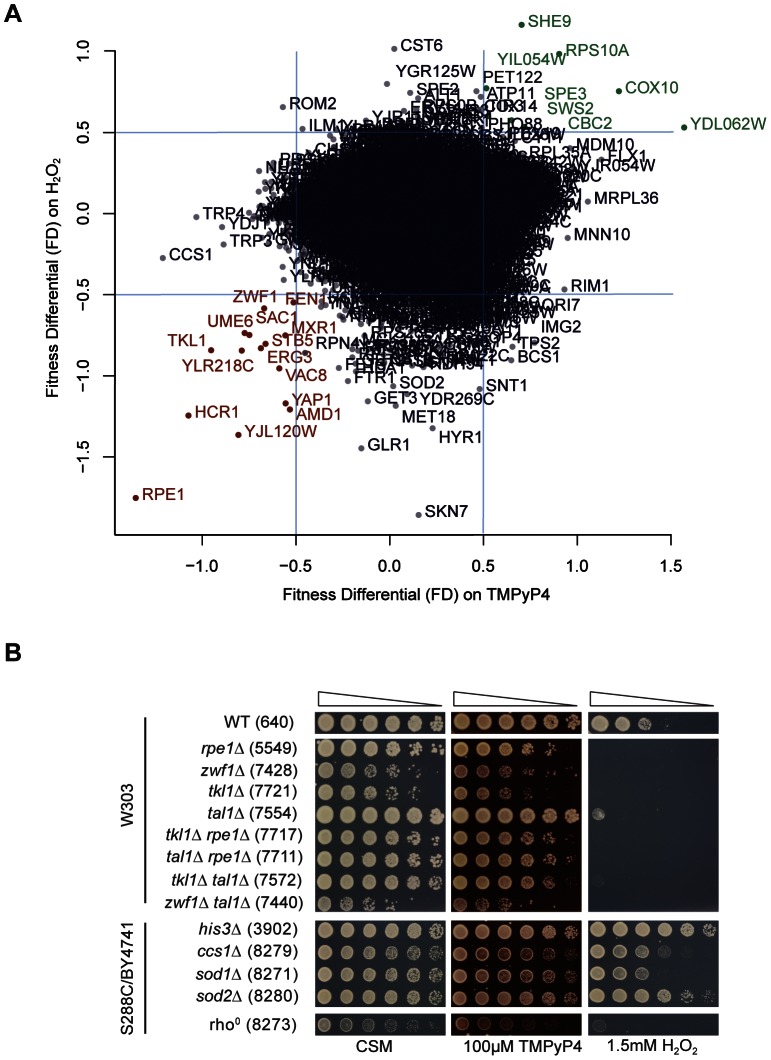
TMPyP4 and hydrogen peroxide treatment result in similar fitness changes. (A) Correlation plot of fitness differential values from the TMPyP4 screen and H_2_O_2_ screen. Genes in red (bottom left-hand corner) were found to increase sensitivity to both TMPyP4 and H_2_O_2_ when deleted (FD ≤−0.5). Genes in green (top right-hand corner) were found to decrease sensitivity to both TMPyP4 and H_2_O_2_ when deleted (FD ≥0.5). Blue lines denote FD thresholds. (B) Spot test for TMPyP4 and H_2_O_2_ sensitivity. Strains were grown to saturation in YEPD before a 5-fold serial dilution in water and spotting onto plates with or without 100 µM TMPyP4. Incubation was carried out at 30°C for 3 days. Strains from three different genetic backgrounds (W303, S288C and BY4741) were tested, as indicated on the left hand side and Table S1 in File S1.

**Figure 6:**
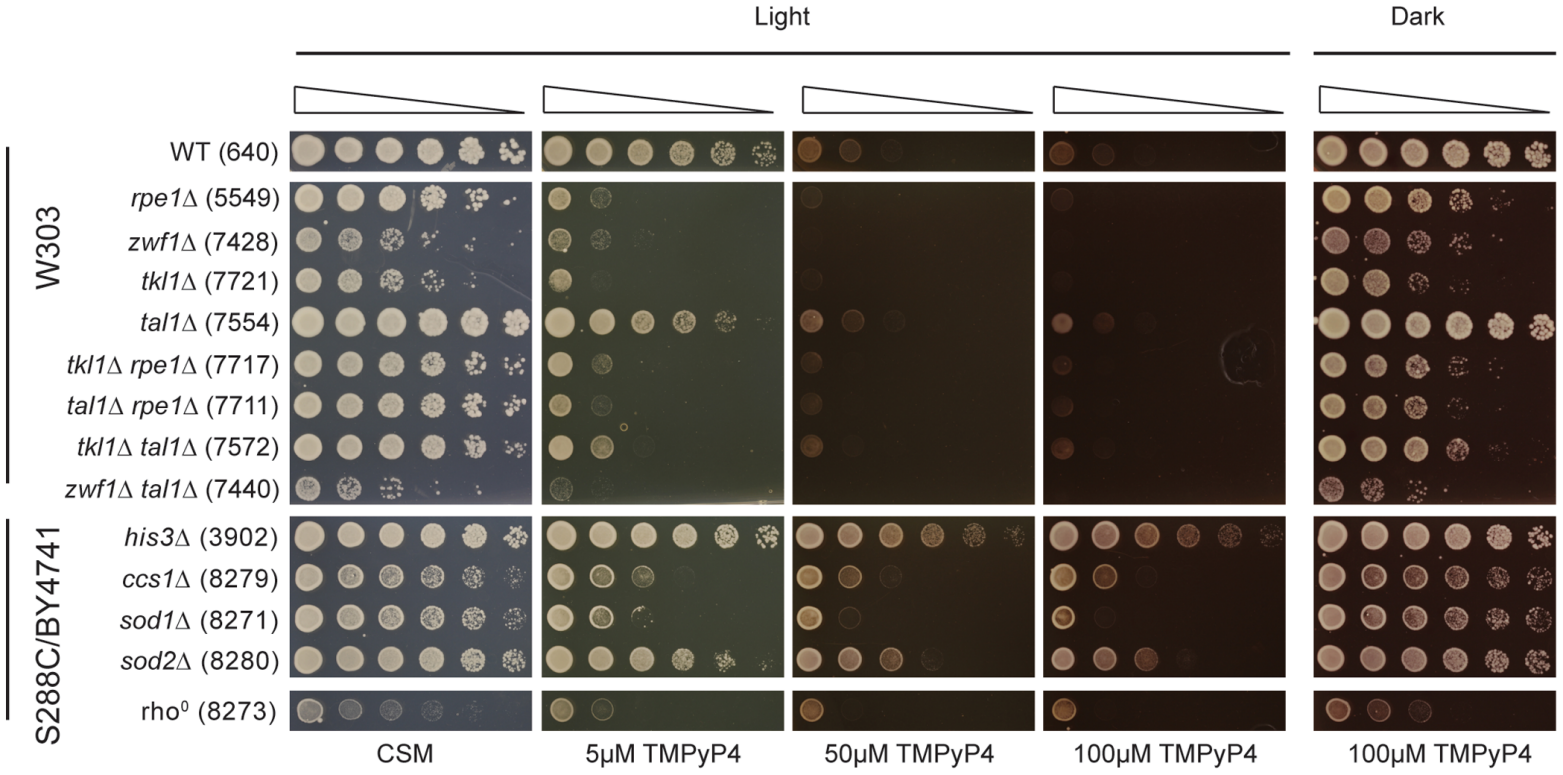
Exposure to light increases the sensitivity of yeast strains to TMPyP4. Strains were grown to saturation in YEPD before a 5-fold serial dilution in water and spotting onto plates containing 0 µM, 5 µM, 50 µM or 100 µM TMPyP4. Plates were incubated at 30°C for 3 days in an incubator fitted with a light. Plates labelled ‘Dark’ were wrapped in reflective foil. Strains from three different genetic backgrounds (W303, S288C and BY4741) were tested, as indicated on the left and in Table S1 in File S1. A control spot test on plates lacking TMPyP4 (CSM) was conducted in the dark; however, the growth of strains did not differ from control plates kept in the light.

We also investigated whether deletion of pairs of PPP genes in tandem would increase the sensitivity of yeast to the G-quadruplex binding ligand. Deletion of a single PPP gene can result in alteration in flux through other parts of the pathway in order to compensate for the deficiency. For instance, deletion of the G6PDH *zwf1* in *E. coli* causes a reversal of flux through the non-oxidative phase of the PPP, from glycolysis and towards production of erythrose-4-phosphate and ribose-5-phosphate (key for biosynthesis of amino acids and nucleic acids) [30]. Deletion of genes encoding important non-oxidative phase enzymes in strains lacking G6PDH can cause growth defects or even lethality, due to lack of ribose-5-phosphate production [31]. Krüger et al. observed that combining *ZWF1* and *TAL1* deletions in the same strain resulted in an increase in hydrogen peroxide sensitivity compared to both single deletion strains [32]. Therefore, we hypothesised that a *zwf1*Δ *tal1*Δ double mutant would be more sensitive to TMPyP4 than *zwf1*Δ and *tal1*Δ strains. In Figure 4b (in addition, see Figure 5b and Figure 6), we show that this hypothesis is correct, and the TMPyP4-sensitivity of a strain lacking both *ZWF1* and *TAL1* is higher than both single deletion strains. We also tested other double *ppp*Δ mutants, *tkl1*Δ *rpe1*Δ, *tal1*Δ *rpe1*Δ and *tkl1*Δ *tal1*Δ. The growth of *tkl1*Δ *rpe1*Δ and *tal1*Δ *rpe1*Δ strains resembled that of the *RPE1* null strain and the TMPyP4-sensitivity of *tkl1*Δ *tal1*Δ was not increased compared to *tkl1*Δ. As Tkl1, Rpe1 and Tal1 operate in the same phase of the pathway, it is unsurprising that *tkl1*Δ *rpe1*Δ, *tal1*Δ *rpe1*Δ and *tkl1*Δ *tal1*Δ strains do not exhibit altered TMPyP4-sensitivity. In addition, the similarity in sensitivity between *tkl1*Δ *tal1*Δ, *tkl1*Δ *rpe1*Δ and *tkl1*Δ strains may be due to the activity of the Tkl1 isoform Tkl2, in spite of minimal detectable transketolase activity in *tkl1*Δ strains [33]. We conclude that the PPP is important for protection against the effects of TMPyP4 treatment, and that *ppp*Δ strains are sensitive in both W303 and S288C backgrounds.

TMPyP4 can affect telomere biology upon binding to G-quadruplexes through prevention of the action of telomerase [10], [11]. Cdc13 is a telomere binding protein which prevents the recognition of telomeres as double strand breaks (DSBs). The temperature sensitive mutant *cdc13-1* is deficient in telomere capping at temperatures over 26°C, which results in the induction of the DNA damage response and eventual cell cycle arrest. Smith et al. recently demonstrated that stabilising G-quadruplexes can partially rescue the temperature sensitivity of *cdc13-1* strains [34]. To test whether TMPyP4 lessens the temperature sensitivity of *cdc13-1* strains, we carried out a spot test on with and without TMPyP4 and incubated the plates at permissive and non-permissive temperatures (Figure 4c). We found that the presence of TMPyP4 did not improve growth of *cdc13-1* strains at 26°C, but rather a slight reduction in growth was observed. This suggests that stabilising G-quadruplexes using TMPyP4 does not rescue the temperature sensitivity of *cdc13-1*, unlike G-quadruplex stabilisation by overexpression of the G4 DNA binding protein Stm1 or the HF1 single-chain antibody (scFv), which rescued growth defects caused by telomere uncapping in *cdc13-1* [34].

### Protection against the Effects of TMPyP4 Requires the Oxidative Stress Response

The pentose phosphate pathway plays a role in the protection against oxidative stress via the production of NADPH in the oxidative phase of the pathway [35], [36]. Of the 19 genes found to increase sensitivity to TMPyP4 upon deletion, 7 are linked with the oxidative stress response – the PPP-related genes, along with *CCS1* and *YAP1*. Ccs1 is the copper chaperone of the superoxide dismutase Sod1 and is thus involved in oxidative stress protection [37]. Yap1 is a basic leucine zipper (bZIP) transcription factor required for oxidative stress tolerance [38], [39]. This suggests that, in some capacity, TMPyP4 is promoting oxidative stress in yeast cells. Interestingly, a transcriptional study of the response of human cells to TMPyP4 identified differentially regulated genes which included a subset of genes related to the oxidative stress response [9], consistent with this hypothesis.

To further test the hypothesis that TMPyP4 is causing oxidative stress, we carried out a genome-wide screen identical to our TMPyP4 screen for gene deletions which affected tolerance to 1.5 mM H_2_O_2_ (Table S6 in File S1). We also investigated tolerance to 100 mM hydroxyurea (HU), an S phase poison and 200 µM RHPS4, another G-quadruplex binding ligand [22] (Tables S7 and S8 in File S1). We found the best correlation was between TMPyP4 and H_2_0_2_ (Figure 5a) rather than with RHPS4 or HU (data not shown). For example, those single deletion mutants most resistant to TMPyP4 tended to be resistant to hydrogen peroxide but less resistant to HU and RHPS4 (Figure S1 in File S1). All our data is available for download at our website to allow any pairwise comparisons to be made: http://research.ncl.ac.uk/qfa/AndrewGQuad/.

We found 15 genes whose deletion resulted in sensitivity to both TMPyP4 and hydrogen peroxide (Figure 5a and Table S3 in File S1), including the 5 pentose phosphate pathway-related genes, along with *YAP1, SAC1, AMD1* and *FEN1*. We confirmed the results in the W303 background by spot test on media containing 100 µM TMPyP4 and 1.5 mM H_2_O_2_ (Figure 5b). Consistent with our findings, previous studies have found *ppp*Δ strains to be sensitive to a range of oxidants [32], [40]. We also examined the TMPyP4- and H_2_O_2_-sensitivity of null mutants for *CCS1* and the superoxide dismutases *SOD1* and *SOD2*, as well as a rho^0^ strain (which is deficient in mitochondrial DNA and is sensitive to peroxides [41]). Here we used a *his3*Δ strain as a “wild type control”, as this strain was used as a control in the genome wide screen, and strains from the BY4741 background (isogenic to S288C, which was studied in the screen). The single gene deletion library lacks a *SOD1* null strain, but we hypothesised that since Sod1 activity relies on Ccs1 and *ccs1*Δ is TMPyP4-sensitve, a *sod1*Δ strain should also be sensitive to TMPyP4. As Figure 5b demonstrates, both *sod1*Δ and *sod2*Δ strains exhibit TMPyP4-sensitivity in comparison to *his3*Δ, but the phenotype is not as strong as that seen for *ccs1*Δ. The rho^0^ strain is also sensitive to the presence of TMPyP4. As predicted, rho^0^ is also highly sensitive to H_2_O_2_, as is the *sod1*Δ strain. This data supports the hypothesis that TMPyP4 is causing oxidative stress, since similar phenotypes are observed in the presence of TMPyP4 and hydrogen peroxide.

Interestingly, a recent study has demonstrated that TMPyP4 is toxic to *Staphylococcus aureus* (MRSA), enterohemorrhagic *Escherichia coli* (EHEC) and *Candida albicans* upon exposure to visible light [42]. Therefore to test whether light-dependent ROS formation was relevant to our studies, we carried out a spot test in which plates were either exposed to visible light or shielded. We found that the TMPyP4-sensitivies of all strains, in particular the *ppp*Δ strains, dramatically increased with exposure to light (Figure 6). This increase in sensitivity is also observable for *ccs1*Δ, *sod1*Δ, *sod2*Δ and rho^0^ strains. This, along with data previously described, strongly suggests that an oxidative stress response is occurring due to the presence of TMPyP4 and light, and that the production of ROS, rather than G-quadruplex binding, is causing toxicity in yeast cells.

## Discussion

In this study we carried out a genome-wide screen of yeast single deletion strains to better understand the mechanisms of action of TMPyP4, hypothesising that deletion of telomerase-, telomere-, or DNA damage response-associated genes would result in a change in sensitivity to TMPyP4 compared to wild type strains. However, we found no evidence of an over-representation of telomere associated genes amongst the strains found to be most sensitive to TMPyP4, instead observing that genes associated with the pentose phosphate pathway (PPP), the oxidative stress response and tubulin folding demonstrated highest TMPyP4-sensitivity upon deletion.

The PPP plays an important role in both nucleotide production and NADPH generation. However, the pathway is also significant in cancer cell metabolism, through the Warburg effect and the overexpression of a mutant form of the human transketolase (TKTL1) in various cancer cell lines [43], [44], [45]. Interestingly there may also exist a link between the oxidative phase of the PPP and the DNA damage response (DDR), through modulation of glucose-6-phosphate dehydrogenase activity by the DDR effector ATM [46]. The TMPyP4-sensitivity displayed by *ppp*Δ strains in all likelihood stems from a reduction in NADPH-generation. NADPH is a cofactor key for antioxidant function and therefore links the PPP to the oxidative stress response. Consequently, null mutants of PPP genes, including *tal1*Δ strains, are sensitive to a wide range of oxidative agents [32]. However, there may also be an NADPH-independent role for the PPP in the oxidative stress response, which is proposed to exert its effects through transcriptional alterations [32]. In addition to the *ppp*Δ strains, we also found several oxidative stress response-linked strains to be sensitive to TMPyP4, including null mutants for *CCS1*, *YAP1, SOD1* and *SOD2*. We also found that a number of TMPyP4-sensitive strains were also sensitive to hydrogen peroxide (H_2_O_2_). We hypothesise therefore that the sensitivity of the *ppp*Δ, *ccs1*Δ, *yap1*Δ and *sod*Δ strains to TMPyP4 is linked to a deficiency in the oxidative stress response.

It was previously noted through transcriptional studies that oxidative stress-linked genes were upregulated in response to TMPyP4 treatment in human cell lines, which suggested that ROS production is occurring due to the presence of TMPyP4 [9]. TMPyP4 is a member of the porphyrin family, a group of compounds historically used in photodynamic therapy, wherein reactive oxygen species are produced upon stimulation by light [18]. Interestingly, TMPyP4 has also been utilised in the photocleavage of DNA, which may also link to a potential reaction of the DDR [19], [20], [21]. The photoreactive property of TMPyP4 therefore provides a potential explanation for our observation that defects in the oxidative stress response cause TMPyP4-sensitivity. Indeed, we found that exposure to light dramatically increased the toxicity of TMPyP4. Our data is supported by a recent study investigating the photodynamic killing of human pathogens using TMPyP4 and exposure to visible light [42]. Therefore, we conclude that treatment of *S. cerevisiae* with TMPyP4 and exposure to light causes the production of ROS and, interestingly, the PPP is instrumental in protection against the phototoxic effects of the ligand.

Strains deficient in tubulin folding and microtubule formation (*cin1*Δ, *cin2*Δ, *yke2*Δ and *tub3*Δ) were also found to be TMPyP4-sensitive. Microtubules are targeted by certain anti-cancer drugs, which either inhibit tubulin polymerisation or cause stabilisation of microtubules [47]. TMPyP4 does not, as far as we are aware target microtubules; however, it has been demonstrated that TMPyP4, along with other G-quadruplex binding ligands, induces elongated chromosomes incapable of separating in anaphase [48]. Difficulties in chromosome segregation may therefore be exacerbated by deletion of key microtubule formation genes, resulting in increased sensitivity to TMPyP4. For that reason, the response of tubulin processing mechanisms to TMPyP4 could be an important area of study with regards to anti-cancer use of TMPyP4.

A study by Hershman et al. (2008) investigated the function of *N*-methyl mesoporphyrin (NMM), which selectively binds G-quadruplexes *in vitro* at a higher affinity than TMPyP4 [49]. Similar to the work described here, the authors screened for yeast mutants that enhance or suppress growth inhibition by NMM, finding that deletion of genes related to chromatin remodelling or modification, transcriptional regulation and those impacting upon telomere function led to increased sensitivity to the agent. This contrasts with our findings, dominated by genes related to the oxidative stress response, and suggests that the increased affinity for G-quadruplexes of NMM may make it a more reliable agent to use in the study of G-quadruplexes, at least in yeast.

There may be additional targets for TMPyP4 or effects of TMPyP4 treatment which remain to be identified. For example, Morris et al. recently demonstrated that TMPyP4 also has the ability to unfold G-quadruplexes in RNA and potentially affect translation in eukaryotes [50]. Our high-throughput data provides a resource to help identify other intracellular targets of TMPyP4, HU, RHPS4 and H_2_0_2_.

## Supporting Information

File S1 Figure S1: Fitness of yeast strain deletion library after treatment with TMPyP4, H_2_0_2_, HU and RHPS4. Fitness of yeast deletions strains after treatment with A) TMPyP4, B) H_2_0_2_, C) HU and d) RHPS4. Data is plotted as in Figure 2C and using data from Tables S4, S6, S7 and S8. All strains were cultured in parallel from the same initial starter cultures. Tables are also available for download from http://research.ncl.ac.uk/qfa/AndrewGQuad. Table S1. Strains of *Saccharomyces cerevisiae* used in this study. Table S2. GO analysis of 19 null mutations which increase TMPyP4 sensitivity. Table S3. Gene deletion strains with differential sensitivity to both TMPyP4 and H_2_O_2_. Table S4. TMPyP4 Screen 1. Table S5. TMPyP4 Screen 2. Table S6. H_2_O_2_ Screen. Table S7. RHPS4 Screen. Table S8. HU Screen.
(PDF)
