# Supplementary material for: Pentose Phosphate Pathway Function Affects Tolerance to the G-Quadruplex Binder TMPyP4
Source: PLoS One. 2013 Jun 12;8(6):e66242. doi: 10.1371/journal.pone.0066242 (PMC3680382; doi:10.1371/journal.pone.0066242)
Supplement: File S1 — Figure S1: Fitness of yeast strain deletion library after treatment with TMPyP4, H202, HU and RHPS4. Fitness of yeast deletions strains after treatment with A) TMPyP4, B) H202, C) HU and d) RHPS4. Data is plotted as in Figure 2C and using data from Tables S4, S6, S7 and S8. All strains were cultured in parallel from the same initial starter cultures. Tables are also available for download from http://research.ncl.ac.uk/qfa/AndrewGQuad. Table S1. Strains of Saccharomyces cerevisiae used in this study. Table S2. GO analysis of 19 null mutations which increase TMPyP4 sensitivity. Table S3. Gene deletion strains with differential sensitivity to both TMPyP4 and H2O2. Table S4. TMPyP4 Screen 1. Table S5. TMPyP4 Screen 2. Table S6. H2O2 Screen. Table S7. RHPS4 Screen. Table S8. HU Screen. (PDF) [file pone.0066242.s001.pdf]

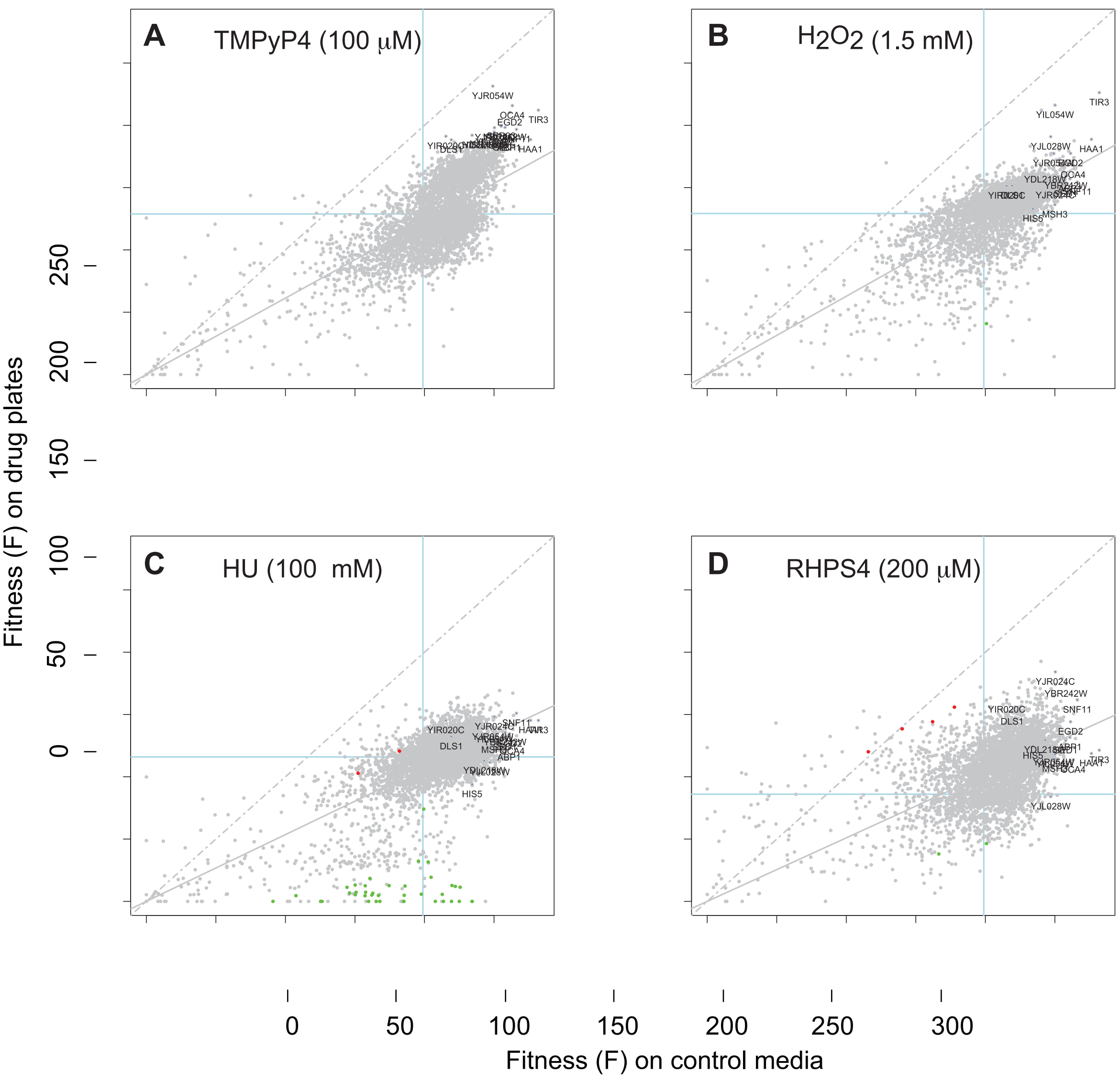
A
TMPyP4 (100 μM)
B
H2O2 (1.5 mM)
C
HU (100 mM)
D
RHPS4 (200 μM)
Fitness (F) on drug plates
Fitness (F) on control media
0
50
100
150
200
250
300
TIR3
YIL054W
HAA1
EGD2
OCA4
YJL028W
DLS1
YIR020C
HIS5
MSH3
SNF11
YJR024C
YBR242W
ABP1
YJR054W

Figure S1

**Table S1**

| **Name** | **Genotype** | **Background** | **Reference** |
|---|---|---|---|
| DLY640 | *MATa ade2-1 trp1-1 can1-100 leu2-3,112 his3-11,15 ura3 GAL+ psi+ ssd1-d2 RAD5* | W303 | Zubko *et al.(53)* |
| DLY5549 | *MATα rpe1::KanMX ade2-1 trp1-1can1-100 leu2-3,112 his3-11,15 ura3 GAL+ psi+ ssd1-d2 RAD5* | W303 | This study |
| DLY7428 | *MATa zwf1::NatMX ade2-1 trp1-1can1-100 leu2-3,112 his3-11,15 ura3 GAL+ psi+ ssd1-d2 RAD5* | W303 | This study |
| DLY7721 | *MATα tkl1::NatMX ade2-1 trp1-1can1-100 leu2-3,112 his3-11,15 ura3 GAL+ psi+ ssd1-d2 RAD5* | W303 | This study |
| DLY7554 | *MATa tal11::NatMX ade2-1 trp1-1can1-100 leu2-3,112 his3-11,15 ura3 GAL+ psi+ ssd1-d2 RAD5* | W303 | This study |
| DLY7717 | *MATa tkl1::NatMX rpe1::KanMX ade2-1 trp1-1can1-100 leu2-3,112 his3-11,15 ura3 GAL+ psi+ ssd1-d2 RAD5* | W303 | This study |
| DLY7711 | *MATα tal1::NatMX rpe1::KanMX ade2-1 trp1-1can1-100 leu2-3,112 his3-11,15 ura3 GAL+ psi+ ssd1-d2 RAD5* | W303 | This study |
| DLY7572 | *MATα tal11::NatMX tkl1::KanMX ade2-1 trp1-1can1-100 leu2-3,112 his3-11,15 ura3 GAL+ psi+ ssd1-d2 RAD5* | W303 | This study |
| DLY7440 | *MATa zwf1::NatMX tal11::KanMX ade2-1 trp1-1can1-100 leu2-3,112 his3-11,15 ura3 GAL+ psi+ ssd1-d2 RAD5* | W303 | This study |
| DLY1108 | *MATa ade2-1 trp1-1 can1-100 leu2-3,112 his3-11,15 ura3 GAL+ psi+ ssd1-d2 RAD5 cdc13-1-int* | W303 | Zubko *et al.(53)* |
| DLY1195 | *MATα trp1-1 can1-100 leu2-3,112 his3-11,15 ura3 GAL+ psi+ ssd1-d2 RAD5 cdc13-1-int LYS+ ade2-1* | W303 | Zubko *et al.(53)* |
| DLY7578 | *MATa tal11::NatMX cdc13-1-int ade2-1 trp1-1can1-100 leu2-3,112 his3-11,15 ura3 GAL+ psi+ ssd1-d2 RAD5* | W303 | This study |
| DLY7579 | *MATα tal11::NatMX cdc13-1-int ade2-1 trp1-1can1-100 leu2-3,112 his3-11,15 ura3 GAL+ psi+ ssd1-d2 RAD5* | W303 | This study |
| DLY5543 | *MATa tal11::KanMX cdc13-1-int ade2-1 trp1-1can1-100 leu2-3,112 his3-11,15 ura3 GAL+ psi+ ssd1-d2 RAD5* | W303 | This study |
| DLY5544 | *MATa tal11::KanMX cdc13-1-int ade2-1 trp1-1can1-100 leu2-3,112 his3-11,15 ura3 GAL+ psi+ ssd1-d2 RAD5* | W303 | This study |
| DLY7574 | *MATα tal11::NatMX tkl1::KanMX cdc13-1-int ade2-1 trp1-1can1-100 leu2-3,112 his3-11,15 ura3 GAL+ psi+ ssd1-d2 RAD5* | W303 | This study |
| DLY7575 | *MATa tal11::NatMX tkl1::KanMX cdc13-1-int ade2-1 trp1-1can1-100 leu2-3,112 his3-11,15 ura3 GAL+ psi+ ssd1-d2 RAD5* | W303 | This study |
| DLY3001 | *MATα ade2-1 trp1-1 can1-100 leu2-3,112 his3-11,15 ura3 GAL+ psi+ ssd1-d2 RAD5* | W303 | Zubko *et al.(53)* |
| DLY5550 | *MATα rpe1::KanMX ade2-1 trp1-1can1-100 leu2-3,112 his3-11,15 ura3 GAL+ psi+ ssd1-d2 RAD5* | W303 | This study |
| DLY7429 | *MATa zwf1::NatMX ade2-1 trp1-1can1-100 leu2-3,112 his3-11,15 ura3 GAL+ psi+ ssd1-d2 RAD5* | W303 | This study |
| DLY5541 | *MATa tkl1::KanMX ade2-1 trp1-1can1-100 leu2-3,112 his3-11,15 ura3 GAL+ psi+ ssd1-d2 RAD5* | W303 | This study |
| DLY3902 | *MATa his3::KanMX his3Δ leu2ΔO ura3ΔO met15ΔO* | S288C | Tong and Boone |
| DLY8271 | *MATa sod1::KanMX his3Δ leu2ΔO ura3ΔO met15ΔO* | | Euroscarf |
| DLY8273* | *MATa his3Δ leu2ΔO ura3ΔO met15ΔO* | | Tong and Boone |
| DLY8279 | *MATa ccs1::KanMX his3Δ leu2ΔO ura3ΔO met15ΔO* | | L Dirick |
| DLY8280 | *MATa sod2::KanMX his3Δ leu2ΔO ura3ΔO met15ΔO* | | Tong and Boone |

* created using ethidium bromide

**Table S2: GO analysis of 19 null mutations which increase TMPyP4-sensitivity**

| Gene Ontology Term | Genes annotated to the term | p-value |
|---|---|---|
| Pentose phosphate shunt | *RPE1, ZWF1, TKL1* | 0.00036 |
| Heterocycle metabolic process | *TRP5, RPE1, TRP3, ROM2, AMD1, ZWF1, TKL1* | 0.00052 |
| Nucleotide metabolic process | *RPE1, ROM2, AMD1, ZWF1, TKL1* | 0.00407 |
| Tryptophan biosynthetic process | *TRP5, TRP3* | 0.00921 |

**Table S3: Gene deletion strains with differential sensitivity to both TMPyP4 and $H_2O_2$**

| ORF | Gene | ORF | Gene |
|---|---|---|---|
| *Sensitive* | | *Resistant* | |
| *YJL121C* | *RPE1* | *YDL062W* | *YDL062W* |
| *YLR192C* | *HCR1* | *YPL172C* | *COX10* |
| *YJL120W* | *YJL120W* | *YOR293W* | *RPS10A* |
| *YPR074C* | *TKL1* | *YPL178W* | *CDC2* |
| *YLR218C* | *YLR218C* | *YDR393W* | *SHE9* |
| *YLR056W* | *ERG3* | *YNL081C* | *SWS2* |
| *YDR207C* | *UME6* | *YPR069C* | *SPE3* |
| *YKL212W* | *SAC1* | *YIL054W* | *YIL054W* |
| *YEL013W* | *VAC8* | | |
| *YHR178W* | *STB5* | | |
| *YML007W* | *YAP1* | | |
| *YML035C* | *AMD1* | | |
| *YER042W* | *MXR1* | | |
| *YNL241C* | *ZWF1* | | |
| *YCR034W* | *FEN1* | | |

**Table S4 Fitness on TMPyP4**

**Key**

| | |
|---|---|
| ORF: | Open reading frame Y-number for *yfg* deletion |
| Gene: | Standard human-readable gene name for *yfg* deletion |
| P: | p-value for the significance of difference between observed mean fitness of treated strains and predicted fitness estimated from mean fitness observed on CSM |
| Q: | False discovery rate corrected p-value (correcting for multiple testing) |
| FD: | Fitness differential (difference between observed mean fitness of treated strains and predicted fitness estimated from mean fitness observed on control media) |
| Mean_TMPyP4: | Mean fitness for all replicate strains following treatment with 100μM TMPyP4 |
| Mean_CSM: | Mean fitness for all replicate strains grown on CSM |

**Supplementary Table 4: TMPyP4 Screen 1 Data**

| ORF | Gene | P | Q | FD | Mean_TMPyP4 | Mean_CSM |
|---|---|---|---|---|---|---|
| YJL121C | RPE1 | 0.00010993 | 0.23063395 | -1.3547166 | 22.72973562 | 213.7510919 |
| YMR038C | CCS1 | 0.00097936 | 0.37922285 | -1.2103272 | 0 | 157.8643125 |
| YLR192C | HCR1 | 0.00074591 | 0.37922285 | -1.0733973 | 7.23209028 | 151.794114 |
| YDR354W | TRP4 | 0.08539408 | 0.89015714 | -1.0314142 | 52.3560035 | 219.8790162 |
| YPR074C | TKL1 | 0.15243205 | 1 | -0.9525929 | 39.83968383 | 189.1942143 |
| YNL064C | YDJ1 | 0.0011034 | 0.37922285 | -0.8943588 | 0 | 116.6522057 |
| YKL211C | TRP3 | 0.12922888 | 1 | -0.8861448 | 39.2990395 | 179.6459655 |
| YJL120W | YJL120W | 0.05835938 | 0.82030594 | -0.8071549 | 37.46570989 | 166.354538 |
| YLR218C | YLR218C | 0.07505711 | 0.85139088 | -0.7893853 | 0 | 102.9604009 |
| YDR207C | UME6 | 0.07074098 | 0.83733449 | -0.7739131 | 51.59403224 | 185.0506958 |
| YGL148W | ARO2 | 0.13349688 | 1 | -0.7504379 | 29.57030249 | 146.0858164 |
| YKL212W | SAC1 | 0.00165929 | 0.37922285 | -0.7487999 | 8.118129927 | 110.9009355 |
| YLR080W | EMP46 | 0.23584314 | 1 | -0.7322139 | 67.89182749 | 206.1804183 |
| YPL241C | CIN2 | 0.06941432 | 0.83733449 | -0.7237322 | 77.47237294 | 220.6922902 |
| YLR224W | YLR224W | 0.24508731 | 1 | -0.7040078 | 91.70002786 | 241.313483 |
| YLR200W | YKE2 | 0.22037408 | 1 | -0.698954 | 51.18851624 | 174.61263 |
| YLR384C | IKI3 | 0.19825907 | 1 | -0.6968266 | 58.29713159 | 185.9235752 |
| YLR138W | NHA1 | 0.17754812 | 1 | -0.69671 | 31.79905884 | 142.7113372 |
| YLR311C | YLR311C | 0.25234842 | 1 | -0.6948966 | 95.9934564 | 247.1242277 |
| YLR056W | ERG3 | 0.20243684 | 1 | -0.6872939 | 58.59179509 | 185.1605721 |
| YKR105C | YKR105C | 0.28874051 | 1 | -0.6813449 | 81.56753884 | 221.8395949 |
| YLR020C | YEH2 | 0.28973412 | 1 | -0.6765576 | 72.013982 | 205.6410216 |
| YNL241C | ZWF1 | 0.23273345 | 1 | -0.669929 | 51.90491612 | 171.9947411 |
| YGL152C | YGL152C | 0.33546258 | 1 | -0.6655087 | 80.30330591 | 217.7131153 |
| YLR236C | YLR236C | 0.23436507 | 1 | -0.663494 | 89.36605308 | 232.2243875 |
| YHR178W | STB5 | 0.2914769 | 1 | -0.6624256 | 54.55425096 | 175.3349887 |
| YER090W | TRP2 | 0.23154986 | 1 | -0.6551931 | 60.66259791 | 184.3494526 |
| YDR509W | YDR509W | 0.35348383 | 1 | -0.6543013 | 93.36018057 | 237.5365831 |
| YLR217W | YLR217W | 0.22170056 | 1 | -0.6455203 | 83.57017454 | 220.4316372 |
| YDR508C | GNP1 | 0.32066572 | 1 | -0.6450148 | 100.6749333 | 248.2498059 |
| YLR012C | YLR012C | 0.19799948 | 1 | -0.6436636 | 72.96228779 | 202.8965422 |
| YOL053C-A | YOL053C-A | 0.37792736 | 1 | -0.642681 | 84.9262981 | 222.272048 |
| YML029W | USA1 | 0.24201033 | 1 | -0.6421466 | 92.88477241 | 235.1762225 |
| YLR368W | MDM30 | 0.2439904 | 1 | -0.6399716 | 77.33489856 | 209.5432042 |
| YLR365W | YLR365W | 0.29821324 | 1 | -0.6353938 | 96.85963356 | 240.775246 |
| YGL026C | TRP5 | 0.19839135 | 1 | -0.6324262 | 61.171731 | 182.2099226 |
| YLR121C | YPS3 | 0.28980461 | 1 | -0.6314095 | 95.45000847 | 237.957599 |
| YLR024C | UBR2 | 0.30404086 | 1 | -0.6276664 | 101.2761711 | 246.9671661 |
| YLR221C | RSA3 | 0.2290061 | 1 | -0.6257006 | 84.42132751 | 219.234075 |
| YOR349W | CIN1 | 0.21025133 | 1 | -0.622254 | 88.38843857 | 225.2516989 |
| YLR044C | PDC1 | 0.23225669 | 1 | -0.6199046 | 48.11841764 | 159.2972649 |
| YLR444C | YLR444C | 0.20423393 | 1 | -0.6197338 | 64.13501348 | 185.3851712 |
| YLR084C | RAX2 | 0.24206582 | 1 | -0.6136729 | 97.07142996 | 238.2874301 |
| YLR392C | YLR392C | 0.29227977 | 1 | -0.6028163 | 92.35445109 | 229.1817973 |
| YLR349W | YLR349W | 0.28240558 | 1 | -0.5996295 | 89.93002436 | 224.8138518 |
| YLL002W | RTT109 | 0.00637044 | 0.5161257 | -0.5963361 | 8.125277115 | 91.02657253 |
| YMR161W | HLJ1 | 0.26602117 | 1 | -0.5953994 | 90.92794505 | 225.8889211 |

| | | | | | | |
|---|---|---|---|---|---|---|
| YLR094C | GIS3 | 0.27880686 | 1 | -0.5922693 | 89.85050243 | 223.7242132 |
| YEL013W | VAC8 | 0.32632319 | 1 | -0.5891244 | 68.73990837 | 188.8996222 |
| YKL047W | YKL047W | 0.29319459 | 1 | -0.5870262 | 96.63620257 | 234.1023736 |
| YLR287C | YLR287C | 0.30724135 | 1 | -0.5863867 | 92.06287246 | 226.5635305 |
| YMR154C | RIM13 | 0.33265877 | 1 | -0.582217 | 69.94022941 | 189.9554512 |
| YLR296W | YLR296W | 0.32970627 | 1 | -0.5813225 | 97.59860554 | 234.9273308 |
| YLL063C | AYT1 | 0.29277371 | 1 | -0.5780147 | 89.56264363 | 221.3957106 |
| YLR063W | YLR063W | 0.31251828 | 1 | -0.5718496 | 91.62909998 | 223.9603057 |
| YNL170W | YNL170W | 0.34376477 | 1 | -0.5711633 | 34.50121422 | 130.7411869 |
| YLR371W | ROM2 | 0.03789474 | 0.75834714 | -0.5699914 | 30.13178442 | 123.4653003 |
| YLR253W | YLR253W | 0.29529171 | 1 | -0.5690185 | 91.33126792 | 223.1055299 |
| YML003W | YML003W | 0.28900807 | 1 | -0.5680531 | 89.40530349 | 219.8399132 |
| YLR309C | IMH1 | 0.33906225 | 1 | -0.5676231 | 79.43750484 | 203.5343602 |
| YLR456W | YLR456W | 0.30088516 | 1 | -0.5655706 | 95.06341573 | 228.7399341 |
| YLR014C | PPR1 | 0.33167113 | 1 | -0.5649699 | 67.51175001 | 183.7469884 |
| YLR090W | XDJ1 | 0.33103663 | 1 | -0.5643088 | 98.28942304 | 233.8343892 |
| YLR146C | SPE4 | 0.30518596 | 1 | -0.5634446 | 73.01214519 | 192.5147619 |
| YML048W-A | YML048W-A | 0.31556326 | 1 | -0.5602119 | 92.40983518 | 223.7151406 |
| YGL195W | GCN1 | 0.3654622 | 1 | -0.5598333 | 91.01418048 | 221.3905679 |
| YGL153W | PEX14 | 0.35844762 | 1 | -0.5583668 | 99.50949531 | 235.0483157 |
| YML100W-A | YML100W-A | 0.31149287 | 1 | -0.5581094 | 94.03951739 | 226.0976151 |
| YER042W | MXR1 | 0.41087578 | 1 | -0.5570589 | 78.7042264 | 200.9610777 |
| YOL006C | TOP1 | 0.07908109 | 0.86626076 | -0.5562706 | 26.08499947 | 115.0786348 |
| YML007W | YAP1 | 0.2844526 | 1 | -0.5554166 | 80.72174283 | 204.035818 |
| YLR343W | GAS2 | 0.34601353 | 1 | -0.5538254 | 68.3517617 | 183.6627828 |
| YLR191W | PEX13 | 0.36442817 | 1 | -0.5536721 | 79.91153976 | 202.487494 |
| YLR231C | BNA5 | 0.27192022 | 1 | -0.5531351 | 100.7941868 | 236.4602452 |
| YLR312C | YLR312C | 0.29623396 | 1 | -0.5531019 | 86.1447616 | 212.5744798 |
| YBR175W | SWD3 | 0.27406918 | 1 | -0.5521722 | 32.39286331 | 124.8271311 |
| YLR306W | UBC12 | 0.29782898 | 1 | -0.5504913 | 82.22231383 | 205.8396191 |
| YLR053C | YLR053C | 0.36484201 | 1 | -0.539342 | 69.94106849 | 184.3645825 |
| YER071C | YER071C | 0.36994115 | 1 | -0.5387134 | 100.4045352 | 233.943999 |
| YLR137W | YLR137W | 0.320159 | 1 | -0.5386712 | 91.22234483 | 218.9697218 |
| YLR006C | SSK1 | 0.33641139 | 1 | -0.5371337 | 74.57529168 | 191.63124 |
| YLR363C | NMD4 | 0.25451337 | 1 | -0.5368944 | 96.73570395 | 227.7258323 |
| YLR023C | IZH3 | 0.31345197 | 1 | -0.5354466 | 84.22034689 | 207.1345137 |
| YLR279W | YLR279W | 0.29780293 | 1 | -0.5328934 | 103.259377 | 237.8388294 |
| YML035C | AMD1 | 0.22719742 | 1 | -0.5324463 | 57.79608037 | 163.666462 |
| YLR047C | FRE8 | 0.12757363 | 1 | -0.5322766 | 56.1311605 | 160.9301886 |
| YLR297W | YLR297W | 0.35076171 | 1 | -0.531047 | 92.14791193 | 219.4841459 |
| YLR280C | YLR280C | 0.34435981 | 1 | -0.5300565 | 90.24816833 | 216.258007 |
| YLR246W | ERF2 | 0.35723961 | 1 | -0.5298542 | 90.49055145 | 216.6267543 |
| YLR081W | GAL2 | 0.30346733 | 1 | -0.5287899 | 101.7083594 | 234.7751541 |
| YML124C | TUB3 | 0.29725699 | 1 | -0.5287232 | 85.90473985 | 209.0034606 |
| YLL048C | YBT1 | 0.29579979 | 1 | -0.5281924 | 79.49379319 | 198.4831338 |
| YLR345W | YLR345W | 0.3202062 | 1 | -0.526801 | 88.76858722 | 213.4213794 |
| YLL052C | AQY2 | 0.37637983 | 1 | -0.5219553 | 95.5424229 | 223.8320207 |
| YER096W | SHC1 | 0.41736781 | 1 | -0.5214129 | 108.8110992 | 245.3918069 |
| YLR257W | YLR257W | 0.30903886 | 1 | -0.5207829 | 105.3018137 | 239.5888235 |
| YLL046C | RNP1 | 0.38553948 | 1 | -0.5173955 | 70.54715523 | 182.490115 |
| YLR207W | HRD3 | 0.35547504 | 1 | -0.5163412 | 91.40883522 | 216.36121 |
| YLR283W | YLR283W | 0.36672079 | 1 | -0.5153444 | 95.83164204 | 223.4412448 |
| YGL252C | RTG2 | 0.38840232 | 1 | -0.513718 | 70.11454473 | 181.3052148 |
| YCR034W | FEN1 | 0.07722668 | 0.85916554 | -0.5133119 | 5.448742062 | 75.83435503 |
| YLR356W | YLR356W | 0.33862333 | 1 | -0.5085201 | 85.40145782 | 205.5479047 |
| YMR195W | ICY1 | 0.24280546 | 1 | -0.507666 | 101.6471617 | 231.9201672 |
| YMR152W | YIM1 | 0.35760061 | 1 | -0.5074617 | 96.90326255 | 224.16004 |
| YLR070C | XYL2 | 0.36968053 | 1 | -0.5060803 | 92.04285013 | 216.0564489 |
| YLR043C | TRX1 | 0.35553786 | 1 | -0.5052258 | 90.95419316 | 214.1702674 |
| YLR042C | YLR042C | 0.39471754 | 1 | -0.503676 | 69.57572933 | 179.1170482 |
| YLR093C | NYV1 | 0.38327084 | 1 | -0.4990598 | 67.0527388 | 174.4019877 |
| YLR353W | BUD8 | 0.37118192 | 1 | -0.4969926 | 92.30063322 | 215.2913669 |
| YLR118C | YLR118C | 0.37781764 | 1 | -0.4957185 | 92.13195458 | 214.8502019 |
| YDR537C | YDR537C | 0.35964503 | 1 | -0.4948919 | 92.56459157 | 215.4476642 |

| | | | | | | |
|---|---|---|---|---|---|---|
| YPL165C | SET6 | 0.33390712 | 1 | -0.4947291 | 96.32401269 | 221.5550305 |
| YLR412W | YLR412W | 0.42626308 | 1 | -0.4941689 | 73.40510077 | 184.1196544 |
| YDR127W | ARO1 | 0.34584058 | 1 | -0.4934418 | 56.35620506 | 156.2317848 |
| YDL119C | YDL119C | 0.43461919 | 1 | -0.4929841 | 74.36781461 | 185.5345264 |
| YER134C | YER134C | 0.41257597 | 1 | -0.4920495 | 99.12918224 | 225.7785022 |
| YLR128W | DCN1 | 0.33906538 | 1 | -0.4901606 | 82.3349385 | 198.1542217 |
| YLR352W | YLR352W | 0.35430107 | 1 | -0.4899909 | 88.7856093 | 208.6479422 |
| YLL060C | GTT2 | 0.36349071 | 1 | -0.4886007 | 90.37164761 | 211.0521682 |
| YLR332W | MID2 | 0.39104005 | 1 | -0.4883668 | 94.19218581 | 217.2498896 |
| YLR187W | SKG3 | 0.35843021 | 1 | -0.4877108 | 86.77249781 | 205.0687909 |
| YLR350W | ORM2 | 0.37424314 | 1 | -0.4874471 | 93.36390088 | 215.7796658 |
| YLR130C | ZRT2 | 0.38830455 | 1 | -0.4872013 | 92.17924236 | 213.8163773 |
| YLR401C | DUS3 | 0.39563952 | 1 | -0.4869712 | 97.08964183 | 221.7912739 |
| YLR273C | PIG1 | 0.31842182 | 1 | -0.4868817 | 100.7362797 | 227.7243362 |
| YLR250W | SSP120 | 0.40447521 | 1 | -0.4855163 | 97.23035789 | 221.83091 |
| YMR307W | GAS1 | 0.36176146 | 1 | -0.4854392 | 43.33195028 | 133.9559192 |
| YLR133W | CKI1 | 0.32700744 | 1 | -0.4822815 | 97.20954644 | 221.3750619 |
| YLR111W | YLR111W | 0.39138905 | 1 | -0.4818825 | 66.33324905 | 170.9886253 |
| YLR263W | RED1 | 0.37610767 | 1 | -0.4815445 | 87.66717823 | 205.7230115 |
| YLR092W | SUL2 | 0.3546401 | 1 | -0.480534 | 87.15917459 | 204.7630593 |
| YLR183C | TOS4 | 0.40955405 | 1 | -0.4775518 | 94.56356843 | 216.444699 |
| YLR194C | YLR194C | 0.37321663 | 1 | -0.4772117 | 88.14485399 | 205.93659 |
| YLR389C | STE23 | 0.39063483 | 1 | -0.4768559 | 92.95785875 | 213.7363208 |
| YLR346C | YLR346C | 0.33524878 | 1 | -0.4768407 | 91.50369244 | 211.3637633 |
| YFL043C | YFL043C | 0.39553408 | 1 | -0.4754037 | 89.17869963 | 207.3861397 |
| YPR129W | SCD6 | 0.51618007 | 1 | -0.4752598 | 86.01567502 | 202.2110152 |
| YMR140W | SIP5 | 0.29095974 | 1 | -0.4751245 | 103.8703162 | 231.2999194 |
| YKL136W | YKL136W | 0.36638531 | 1 | -0.4744491 | 104.598728 | 232.3992828 |
| YPR030W | CSR2 | 0.53063285 | 1 | -0.4742597 | 90.05587067 | 208.6668865 |
| YER007W | PAC2 | 0.15460856 | 1 | -0.4735544 | 106.0476438 | 234.6446028 |
| YLR210W | CLB4 | 0.39996564 | 1 | -0.4732792 | 94.82889199 | 216.3199534 |
| YLR126C | YLR126C | 0.38800274 | 1 | -0.4726455 | 94.33003763 | 215.4240686 |
| YMR182C | RGM1 | 0.28159279 | 1 | -0.4723384 | 104.3655174 | 231.7438076 |
| YLR227C | ADY4 | 0.54202819 | 1 | -0.4718961 | 91.24500175 | 210.2971162 |
| YER179W | DMC1 | 0.34933317 | 1 | -0.4707924 | 83.31862624 | 197.2316175 |
| YLR320W | MMS22 | 0.24556527 | 1 | -0.470705 | 51.74020797 | 145.7412263 |
| YLL061W | MMP1 | 0.38406106 | 1 | -0.4695607 | 89.14496419 | 206.5690324 |
| YML053C | YML053C | 0.32191704 | 1 | -0.4693385 | 105.9826953 | 233.9888401 |
| YDR463W | STP1 | 0.47217892 | 1 | -0.468375 | 85.80817894 | 200.9747633 |
| YLR348C | DIC1 | 0.40516326 | 1 | -0.4666215 | 92.98154572 | 212.4400468 |
| YLR015W | BRE2 | 0.371017 | 1 | -0.4662974 | 74.45176824 | 182.1906117 |
| YML117W | NAB6 | 0.35360503 | 1 | -0.4660677 | 89.68183245 | 206.9886292 |
| YLR327C | TMA10 | 0.41115285 | 1 | -0.4654735 | 93.97112155 | 213.9035144 |
| YJR118C | ILM1 | 0.32466748 | 1 | -0.4653115 | 62.97037533 | 163.3451037 |
| YKR012C | YKR012C | 0.4460848 | 1 | -0.4652595 | 81.7195698 | 193.9031737 |
| YMR021C | MAC1 | 0.22631393 | 1 | -0.4650953 | 67.24581498 | 170.2867121 |
| YML123C | PHO84 | 0.28051397 | 1 | -0.4639292 | 97.75744148 | 219.874526 |
| YMR269W | TMA23 | 0.29093967 | 1 | -0.4637836 | 49.72344148 | 141.5507389 |
| YMR137C | PSO2 | 0.2959336 | 1 | -0.4628603 | 104.5641076 | 230.8313034 |
| YMR144W | YMR144W | 0.29665014 | 1 | -0.4626129 | 101.6319878 | 226.0191095 |
| YLR281C | YLR281C | 0.39234114 | 1 | -0.4624284 | 79.28921794 | 189.5719702 |
| YLR237W | THI7 | 0.43307051 | 1 | -0.4620959 | 103.7160446 | 229.3490994 |
| YLR189C | ATG26 | 0.40526759 | 1 | -0.4619064 | 93.15872923 | 212.1138993 |
| YLR151C | PCD1 | 0.40358477 | 1 | -0.460703 | 93.95461674 | 213.2543801 |
| YMR214W | SCJ1 | 0.32541074 | 1 | -0.4590688 | 108.2305129 | 236.3137396 |
| YKL163W | PIR3 | 0.38264375 | 1 | -0.4578919 | 105.4459952 | 231.6209248 |
| YLR016C | PML1 | 0.40616409 | 1 | -0.4567435 | 92.78311043 | 210.8281557 |
| YLL055W | YLL055W | 0.39562939 | 1 | -0.4563207 | 90.44720562 | 206.9650355 |
| YLR096W | KIN2 | 0.35166215 | 1 | -0.4563015 | 95.60718367 | 215.3742963 |
| YLR135W | SLX4 | 0.37861794 | 1 | -0.4561861 | 84.52617315 | 197.2950302 |
| YLR031W | YLR031W | 0.39773455 | 1 | -0.4557141 | 91.16105775 | 208.0496311 |
| YLR303W | MET17 | 0.39907884 | 1 | -0.4555151 | 90.13479527 | 206.3506591 |
| YLR171W | YLR171W | 0.41367051 | 1 | -0.4539526 | 96.82979268 | 217.0610156 |
| YLR082C | SRL2 | 0.42832922 | 1 | -0.453421 | 92.49753173 | 209.9292465 |

| | | | | | | |
|---|---|---|---|---|---|---|
| YMR317W | YMR317W | 0.46381598 | 1 | -0.4531585 | 85.00922373 | 197.687602 |
| YLR381W | CTF3 | 0.39795367 | 1 | -0.4529688 | 89.11080293 | 204.3492376 |
| YLR036C | YLR036C | 0.39226836 | 1 | -0.45275 | 85.95964101 | 199.1836965 |
| YLR380W | CSR1 | 0.40909044 | 1 | -0.4527339 | 89.56573581 | 205.0602308 |
| YKR026C | GCN3 | 0.30369731 | 1 | -0.4518154 | 109.5809041 | 237.5690688 |
| YLR391W | YLR391W | 0.41643985 | 1 | -0.4517134 | 90.69628079 | 206.7701324 |
| YLR351C | NIT3 | 0.40324512 | 1 | -0.4516082 | 90.08259377 | 205.7559831 |
| YDL020C | RPN4 | 0.39202847 | 1 | -0.4514504 | 70.86005822 | 174.3989141 |
| YNL195C | YNL195C | 0.40452361 | 1 | -0.4497607 | 107.9776054 | 234.6873844 |
| YLR120C | YPS1 | 0.41025717 | 1 | -0.4490152 | 89.07052661 | 203.7679116 |
| YPR051W | MAK3 | 0.50233283 | 1 | -0.4480573 | 90.53945922 | 206.0376132 |
| YLR091W | YLR091W | 0.32742886 | 1 | -0.4475493 | 96.19075269 | 215.1840738 |
| YLR077W | FMP25 | 0.34180486 | 1 | -0.4474412 | 96.29582735 | 215.3412656 |
| YMR103C | YMR103C | 0.38448163 | 1 | -0.4459668 | 90.11226241 | 205.0685337 |
| YJR148W | BAT2 | 0.4375977 | 1 | -0.445319 | 118.7982162 | 251.7477501 |
| YMR035W | IMP2 | 0.43547788 | 1 | -0.444614 | 84.27128519 | 195.3701517 |
| YLR248W | RCK2 | 0.41385927 | 1 | -0.4430363 | 93.02481733 | 209.4343369 |
| YMR172W | HOT1 | 0.33303574 | 1 | -0.4416273 | 103.5698377 | 226.4410085 |
| YER111C | SWI4 | 0.47926361 | 1 | -0.4415758 | 86.96777619 | 199.3696867 |
| YLR073C | YLR073C | 0.34558747 | 1 | -0.4413214 | 95.84520124 | 213.8084394 |
| YER162C | RAD4 | 0.45029095 | 1 | -0.4401699 | 93.29576716 | 209.5021731 |
| YMR221C | FMP42 | 0.32610589 | 1 | -0.4390342 | 105.1893987 | 228.7429928 |
| YLR267W | BOP2 | 0.3473018 | 1 | -0.4367615 | 98.41664226 | 217.4056359 |
| YLR427W | MAG2 | 0.28081183 | 1 | -0.4367572 | 103.011188 | 224.8950843 |
| YLR385C | SWC7 | 0.42497871 | 1 | -0.435266 | 92.04936714 | 206.8306741 |
| YML012W | ERV25 | 0.43383218 | 1 | -0.4350921 | 97.20849695 | 215.2183892 |
| YLR354C | TAL1 | 0.38454278 | 1 | -0.4348583 | 86.72233195 | 198.0933962 |
| YMR199W | CLN1 | 0.39384991 | 1 | -0.4341033 | 90.37223532 | 203.944976 |
| YLR328W | NMA1 | 0.41940055 | 1 | -0.4339403 | 88.93649463 | 201.5831704 |
| YLR179C | YLR179C | 0.37957291 | 1 | -0.4338112 | 82.42113251 | 190.9450277 |
| YLR055C | SPT8 | 0.31550448 | 1 | -0.4329999 | 68.83924504 | 168.6980713 |
| YLR330W | CHS5 | 0.45355698 | 1 | -0.4329183 | 79.57331366 | 186.1860595 |
| YLR376C | PSY3 | 0.37623443 | 1 | -0.432736 | 100.9635449 | 221.0325357 |
| YMR316C-A | YMR316C-A | 0.4307447 | 1 | -0.4320643 | 111.7309248 | 238.4978624 |
| YMR055C | BUB2 | 0.31914295 | 1 | -0.4300318 | 102.8537416 | 223.7612191 |
| YLR408C | YLR408C | 0.3628696 | 1 | -0.4300232 | 93.95302801 | 209.2501928 |
| YLR377C | FBP1 | 0.43877018 | 1 | -0.429767 | 90.87272069 | 204.1952764 |
| YNL309W | STB1 | 0.39652256 | 1 | -0.4296852 | 99.61155155 | 218.4306116 |
| YLR329W | REC102 | 0.38057652 | 1 | -0.4294557 | 105.4301193 | 227.8860725 |
| YLR083C | EMP70 | 0.36189436 | 1 | -0.4276104 | 102.3320395 | 222.5949098 |
| YMR188C | MRPS17 | 0.31583652 | 1 | -0.4268356 | 99.04449754 | 217.1345282 |
| YLR152C | YLR152C | 0.46197564 | 1 | -0.4262187 | 99.08500929 | 217.1200976 |
| YLR087C | CSF1 | 0.33785307 | 1 | -0.4256093 | 77.61755437 | 182.0444699 |
| YLR206W | ENT2 | 0.42096633 | 1 | -0.4253449 | 83.7643665 | 192.0304958 |
| YLR097C | HRT3 | 0.42406289 | 1 | -0.4252732 | 91.64511618 | 204.8683047 |
| YER149C | PEA2 | 0.33715149 | 1 | -0.4252238 | 116.0417574 | 244.6331458 |
| YMR025W | CSI1 | 0.41567376 | 1 | -0.4246229 | 96.17752244 | 212.1721906 |
| YLR136C | TIS11 | 0.43491936 | 1 | -0.4243406 | 91.64309225 | 204.7433564 |
| YLR109W | AHP1 | 0.36853403 | 1 | -0.4231059 | 103.5188199 | 223.9420748 |
| YLR170C | APS1 | 0.46206491 | 1 | -0.4230315 | 93.41373135 | 207.4591057 |
| YLR035C | MLH2 | 0.43477674 | 1 | -0.4222869 | 92.31773501 | 205.5752943 |
| YLR315W | NKP2 | 0.37470198 | 1 | -0.4220456 | 83.23771747 | 190.7416219 |
| YFR011C | YFR011C | 0.45597266 | 1 | -0.4219944 | 90.80184958 | 203.0659489 |
| YLR032W | RAD5 | 0.43292752 | 1 | -0.4206353 | 78.6439853 | 183.0689917 |
| YLL062C | MHT1 | 0.40183304 | 1 | -0.4181226 | 100.3176112 | 218.0735013 |
| YLR017W | MEU1 | 0.42363014 | 1 | -0.4168771 | 104.7219618 | 225.0910042 |
| YEL008W | YEL008W | 0.48273891 | 1 | -0.4162727 | 101.219663 | 219.3027351 |
| YLR271W | YLR271W | 0.43202862 | 1 | -0.4151087 | 91.84892394 | 203.8747819 |
| YLR102C | APC9 | 0.42796786 | 1 | -0.4143161 | 107.5010491 | 229.2874149 |
| YNL087W | TCB2 | 0.45128743 | 1 | -0.4142031 | 112.3319355 | 237.1479683 |
| YMR096W | SNZ1 | 0.50336043 | 1 | -0.4141325 | 103.2926494 | 222.4029569 |
| YLR372W | SUR4 | 0.52856584 | 1 | -0.4139652 | 39.65010654 | 118.631365 |
| YLR037C | DAN2 | 0.43205193 | 1 | -0.4122472 | 90.81647699 | 201.8184656 |
| YLR313C | SPH1 | 0.46057591 | 1 | -0.4120743 | 95.51541815 | 209.4561018 |

| | | | | | | |
|---|---|---|---|---|---|---|
| YDR485C | VPS72 | 0.50515787 | 1 | -0.4120703 | 75.01370169 | 176.0337719 |
| YLL014W | YLL014W | 0.41978548 | 1 | -0.4118673 | 101.3513851 | 218.9428613 |
| YNL229C | URE2 | 0.45044062 | 1 | -0.4116986 | 111.577347 | 235.5911779 |
| YLR262C-A | TMA7 | 0.46234604 | 1 | -0.4115024 | 95.77349367 | 209.8022159 |
| YLR265C | NEJ1 | 0.45231282 | 1 | -0.4108339 | 90.38318832 | 200.9277715 |
| YLR286C | CTS1 | 0.46715822 | 1 | -0.4103307 | 97.16426494 | 211.9166284 |
| YMR127C | SAS2 | 0.47918686 | 1 | -0.4103169 | 101.8247675 | 219.5123593 |
| YNL253W | TEX1 | 0.41738086 | 1 | -0.4102194 | 99.82479146 | 216.2392782 |
| YLR124W | YLR124W | 0.42563457 | 1 | -0.4096254 | 104.7130496 | 224.1306194 |
| YLR216C | CPR6 | 0.40437871 | 1 | -0.407484 | 94.29939482 | 206.8750305 |
| YLL049W | LDB18 | 0.44846043 | 1 | -0.4074201 | 77.1498297 | 178.9095512 |
| YEL014C | YEL014C | 0.49914132 | 1 | -0.4072422 | 97.3452172 | 211.808771 |
| YER079W | YER079W | 0.45818547 | 1 | -0.4065075 | 97.09154927 | 211.2994151 |
| YLR219W | MSC3 | 0.42124979 | 1 | -0.4063505 | 85.5678523 | 192.4930693 |
| YER085C | YER085C | 0.30684486 | 1 | -0.4063336 | 112.387513 | 236.2121448 |
| YLR164W | YLR164W | 0.46989634 | 1 | -0.4062141 | 96.33771698 | 210.0322588 |
| YLR406C | RPL31B | 0.42975748 | 1 | -0.4060837 | 89.46352627 | 198.8089796 |
| YFR009W | GCN20 | 0.50638578 | 1 | -0.4057598 | 103.0940227 | 220.9871025 |
| YLR289W | GUF1 | 0.38051874 | 1 | -0.405093 | 96.01064449 | 209.3528395 |
| YKL077W | YKL077W | 0.46802471 | 1 | -0.4050135 | 116.1156292 | 242.1175166 |
| YGL045W | RIM8 | 0.38017919 | 1 | -0.4048788 | 101.3351585 | 218.0048988 |
| YLR064W | YLR064W | 0.43069936 | 1 | -0.4044835 | 86.52728129 | 193.8136023 |
| YLR341W | SPO77 | 0.41636246 | 1 | -0.4041964 | 95.51966493 | 208.4354982 |
| YLR364W | YLR364W | 0.39584672 | 1 | -0.4039406 | 72.76803181 | 171.3125321 |
| YMR054W | STV1 | 0.32896458 | 1 | -0.4037602 | 101.3435142 | 217.8726247 |
| YLR225C | YLR225C | 0.42757875 | 1 | -0.4013492 | 98.11918698 | 212.3018711 |
| YLR034C | SMF3 | 0.41722166 | 1 | -0.4005262 | 70.60096404 | 167.3344418 |
| YLR307W | CDA1 | 0.42228335 | 1 | -0.4002091 | 103.0948989 | 220.2645468 |
| YKL084W | HOT13 | 0.46873255 | 1 | -0.3998986 | 112.5549117 | 235.6457131 |
| YLR266C | PDR8 | 0.37427644 | 1 | -0.3997362 | 115.0081888 | 239.6238481 |
| YLR013W | GAT3 | 0.43141807 | 1 | -0.3995376 | 102.4902695 | 219.1913007 |
| YNL157W | IGO1 | 0.38887521 | 1 | -0.3993754 | 120.8477347 | 249.0963953 |
| YLR398C | SKI2 | 0.43282093 | 1 | -0.3989817 | 89.03758465 | 197.1882871 |
| YLR247C | IRC20 | 0.4966927 | 1 | -0.3980296 | 102.9952681 | 219.8178557 |
| YML013W | SEL1 | 0.45380794 | 1 | -0.3970758 | 63.75276357 | 155.7204966 |
| YKL131W | YKL131W | 0.46923859 | 1 | -0.3968629 | 107.9169883 | 227.6890419 |
| YMR017W | SPO20 | 0.40992135 | 1 | -0.3964979 | 86.67497114 | 193.0127939 |
| YMR088C | VBA1 | 0.42658987 | 1 | -0.3964031 | 116.1867976 | 241.1104711 |
| YNL063W | MTQ1 | 0.49170345 | 1 | -0.3962265 | 118.9587768 | 245.6063147 |
| YER113C | YER113C | 0.54842888 | 1 | -0.394339 | 110.0580856 | 230.8502539 |
| YLR018C | POM34 | 0.47387037 | 1 | -0.3927746 | 90.86940137 | 199.364905 |
| YLR003C | YLR003C | 0.39279728 | 1 | -0.3922985 | 96.6667564 | 208.7536318 |
| YDR480W | DIG2 | 0.45713568 | 1 | -0.3920841 | 92.91137854 | 202.6036658 |
| YLR361C | DCR2 | 0.42634677 | 1 | -0.3916542 | 82.56446366 | 185.6800916 |
| YLR169W | YLR169W | 0.42884799 | 1 | -0.3915367 | 102.3588003 | 217.9334094 |
| YER055C | HIS1 | 0.51674869 | 1 | -0.3908809 | 95.62116551 | 206.8642086 |
| YLR326W | YLR326W | 0.46567686 | 1 | -0.3892922 | 93.13966593 | 202.6116628 |
| YNL144C | YNL144C | 0.48061154 | 1 | -0.3892312 | 108.8297422 | 228.1816022 |
| YLR405W | DUS4 | 0.442943 | 1 | -0.3886882 | 103.8660579 | 220.0189982 |
| YMR003W | YMR003W | 0.33963924 | 1 | -0.3883314 | 104.6236167 | 221.2074299 |
| YLR404W | YLR404W | 0.45238132 | 1 | -0.3883053 | 74.75129444 | 172.506303 |
| YLR123C | YLR123C | 0.49365499 | 1 | -0.3878177 | 98.10031973 | 210.5061734 |
| YMR095C | SNO1 | 0.43781396 | 1 | -0.3877428 | 90.13908487 | 197.5180423 |
| YOR129C | YOR129C | 0.50094339 | 1 | -0.3873799 | 109.0818519 | 228.3511256 |
| YLR011W | LOT6 | 0.50274666 | 1 | -0.3870535 | 101.7612576 | 216.3745484 |
| YLR050C | YLR050C | 0.4304252 | 1 | -0.3869267 | 97.70108571 | 209.7391342 |
| YMR036C | MIH1 | 0.40908161 | 1 | -0.385427 | 113.1069661 | 234.6581287 |
| YMR119W-A | YMR119W-A | 0.34414498 | 1 | -0.3853833 | 102.093039 | 216.697575 |
| YMR065W | KAR5 | 0.36363001 | 1 | -0.3848319 | 105.73102 | 222.5562652 |
| YNL169C | PSD1 | 0.30381495 | 1 | -0.3844561 | 57.13989491 | 143.2942251 |
| YLL054C | YLL054C | 0.42903105 | 1 | -0.3842144 | 85.6606928 | 189.7571776 |
| YNL339C | YRF1-6 | 0.41482028 | 1 | -0.384087 | 116.8912456 | 240.6524567 |
| YLR057W | YLR057W | 0.50513424 | 1 | -0.3839688 | 97.93259136 | 209.73073 |
| YLR176C | RFX1 | 0.44986266 | 1 | -0.3830731 | 102.7655428 | 217.4925629 |

| | | | | | | |
|---|---|---|---|---|---|---|
| YLR415C | YLR415C | 0.42372333 | 1 | -0.3829903 | 113.5032719 | 234.9863547 |
| YOL062C | APM4 | 0.59828301 | 1 | -0.3820395 | 85.67243647 | 189.4926395 |
| YLR209C | PNP1 | 0.45731058 | 1 | -0.3816311 | 88.96623117 | 194.8089066 |
| YKR013W | PRY2 | 0.46313445 | 1 | -0.3814078 | 106.9610421 | 224.1148387 |
| YMR210W | YMR210W | 0.45039808 | 1 | -0.3808772 | 93.58266207 | 202.2362548 |
| YLR428C | YLR428C | 0.31711107 | 1 | -0.3806178 | 92.41404892 | 200.2973626 |
| YLR142W | PUT1 | 0.32996889 | 1 | -0.3804909 | 105.4820017 | 221.5841243 |
| YLR205C | HMX1 | 0.43011076 | 1 | -0.3803084 | 97.66562793 | 208.8180977 |
| YML099C | ARG81 | 0.39448742 | 1 | -0.3800219 | 103.6083051 | 218.4684628 |
| YLR058C | SHM2 | 0.47011489 | 1 | -0.3799895 | 102.0610515 | 215.9418993 |
| YLR001C | YLR001C | 0.41591575 | 1 | -0.3799618 | 98.51631571 | 210.1596784 |
| YKL127W | PGM1 | 0.48128685 | 1 | -0.3795534 | 110.7994164 | 230.1302701 |
| YLR062C | BUD28 | 0.34988195 | 1 | -0.3793898 | 89.84906693 | 195.9557705 |
| YLR375W | STP3 | 0.39376063 | 1 | -0.3793475 | 102.0593773 | 215.8554349 |
| YKL214C | YRA2 | 0.504232 | 1 | -0.3792855 | 117.4597372 | 240.9529454 |
| YOR027W | STI1 | 0.52382162 | 1 | -0.3789434 | 116.6352474 | 239.56425 |
| YML095C | RAD10 | 0.39244609 | 1 | -0.3787458 | 107.7289295 | 225.0194402 |
| YPL180W | TCO89 | 0.43897662 | 1 | -0.3781779 | 56.3547319 | 141.1953791 |
| YNL338W | YNL338W | 0.47918428 | 1 | -0.3775763 | 108.0164039 | 225.3355338 |
| YDR007W | TRP1 | 0.41878889 | 1 | -0.3772767 | 101.8913806 | 215.311477 |
| YLL059C | YLL059C | 0.46710837 | 1 | -0.3767635 | 86.60203676 | 190.3199246 |
| YDR475C | JIP4 | 0.38328123 | 1 | -0.3763332 | 114.3464483 | 235.4926094 |
| YLR335W | NUP2 | 0.45430064 | 1 | -0.3761291 | 88.80410658 | 193.8269767 |
| YMR300C | ADE4 | 0.47691425 | 1 | -0.3758095 | 106.680859 | 222.9278978 |
| YLR251W | SYM1 | 0.41086666 | 1 | -0.3756658 | 93.99447732 | 202.2278728 |
| YER139C | YER139C | 0.51718388 | 1 | -0.3741494 | 63.01456729 | 151.526779 |
| YLR400W | YLR400W | 0.45306595 | 1 | -0.3734074 | 87.1003912 | 190.6946013 |
| YLR402W | YLR402W | 0.4611753 | 1 | -0.3733566 | 22.30158389 | 85.05325942 |
| YMR086W | YMR086W | 0.39309484 | 1 | -0.3723825 | 109.049537 | 226.3423122 |
| YLR112W | YLR112W | 0.42052634 | 1 | -0.3722295 | 102.5140439 | 215.6682228 |
| YML017W | PSP2 | 0.3964733 | 1 | -0.3702864 | 103.2101424 | 216.5495594 |
| YKR096W | YKR096W | 0.49273312 | 1 | -0.3697779 | 110.8248508 | 228.8967033 |
| YLR041W | YLR041W | 0.53421782 | 1 | -0.369504 | 100.4016588 | 211.8691354 |
| YNL153C | GIM3 | 0.31545911 | 1 | -0.3691995 | 72.81353407 | 166.8553892 |
| YLR211C | YLR211C | 0.44194471 | 1 | -0.3690959 | 96.44644225 | 205.3681316 |
| YLR254C | NDL1 | 0.4547201 | 1 | -0.368156 | 97.69073922 | 207.2739802 |
| YKL079W | SMY1 | 0.50153562 | 1 | -0.3673632 | 106.5962965 | 221.6883762 |
| YNL320W | YNL320W | 0.47291606 | 1 | -0.3666775 | 104.6864905 | 218.4855831 |
| YMR266W | RSN1 | 0.51158611 | 1 | -0.366509 | 115.2583271 | 235.6977711 |
| YMR191W | SPG5 | 0.41557836 | 1 | -0.3663664 | 110.0900445 | 227.2538597 |
| YMR238W | DFG5 | 0.38134107 | 1 | -0.3663594 | 103.7711909 | 216.951981 |
| YNL305C | YNL305C | 0.50982334 | 1 | -0.3662762 | 66.79732162 | 156.6664979 |
| YLR149C | YLR149C | 0.50393732 | 1 | -0.3654139 | 93.66309623 | 200.3504799 |
| YER115C | SPR6 | 0.51163315 | 1 | -0.3653994 | 94.09064334 | 201.0455745 |
| YLR072W | YLR072W | 0.50734557 | 1 | -0.3645711 | 77.3242393 | 173.6050297 |
| YMR120C | ADE17 | 0.48571061 | 1 | -0.3641257 | 95.69463127 | 203.4942662 |
| YLR178C | TFS1 | 0.43422514 | 1 | -0.3640262 | 95.59903249 | 203.3254415 |
| YFR006W | YFR006W | 0.43612455 | 1 | -0.3639508 | 117.0558482 | 238.2944134 |
| YLR079W | SIC1 | 0.48840177 | 1 | -0.3637035 | 72.35293786 | 165.387672 |
| YPL213W | LEA1 | 0.48635022 | 1 | -0.3635467 | 73.28441313 | 166.8857072 |
| YLR173W | YLR173W | 0.46981073 | 1 | -0.3634667 | 88.25117789 | 191.2740289 |
| YLL026W | HSP104 | 0.51250426 | 1 | -0.3634015 | 114.3341327 | 233.785841 |
| YML104C | MDM1 | 0.3968313 | 1 | -0.362627 | 107.9739465 | 223.3164696 |
| YMR026C | PEX12 | 0.47195643 | 1 | -0.3622163 | 84.27574868 | 184.6302127 |
| YLR049C | YLR049C | 0.45137202 | 1 | -0.3621739 | 85.13876567 | 186.0315665 |
| YLR413W | YLR413W | 0.42449752 | 1 | -0.3618697 | 104.6423729 | 217.7865839 |
| YLR234W | TOP3 | 0.53223676 | 1 | -0.3618436 | 70.52068488 | 162.1581531 |
| YLR284C | ECI1 | 0.45140088 | 1 | -0.3613178 | 81.49497889 | 179.9798129 |
| YLR252W | YLR252W | 0.51920131 | 1 | -0.3607795 | 91.98493098 | 197.0102773 |
| YLR431C | ATG23 | 0.47251961 | 1 | -0.3606125 | 90.18885189 | 194.0605337 |
| YKL086W | SRX1 | 0.51800621 | 1 | -0.3603637 | 115.3293041 | 235.0119394 |
| YML119W | YML119W | 0.40889441 | 1 | -0.3595036 | 108.4702241 | 223.7181116 |
| YLR278C | YLR278C | 0.42294194 | 1 | -0.3594421 | 96.50639346 | 204.2067138 |
| YJR073C | OPI3 | 0.33064781 | 1 | -0.3593285 | 73.93823112 | 167.4013776 |

| | | | | | | |
|---|---|---|---|---|---|---|
| YML056C | IMD4 | 0.34739319 | 1 | -0.3589286 | 100.7412238 | 211.0433292 |
| YLR174W | IDP2 | 0.46354609 | 1 | -0.3588031 | 101.0853398 | 211.5879357 |
| YFL044C | OTU1 | 0.42439448 | 1 | -0.3579188 | 116.8367872 | 237.1505317 |
| YNL228W | YNL228W | 0.39969385 | 1 | -0.3575007 | 49.96667136 | 128.0846454 |
| YNL211C | YNL211C | 0.47351037 | 1 | -0.3573331 | 97.79763743 | 206.0366042 |
| YNL279W | PRM1 | 0.50622115 | 1 | -0.3572554 | 98.94929076 | 207.9038929 |
| YMR302C | YME2 | 0.4636945 | 1 | -0.3571415 | 121.8127326 | 245.1609171 |
| YKR069W | MET1 | 0.4182042 | 1 | -0.357077 | 120.0418008 | 242.2655351 |
| YJR135C | MCM22 | 0.42602682 | 1 | -0.3565813 | 112.3915728 | 229.7295133 |
| YKL197C | PEX1 | 0.42812511 | 1 | -0.3556947 | 111.6402196 | 228.3890226 |
| YMR006C | PLB2 | 0.37110235 | 1 | -0.3556017 | 101.6706244 | 212.1244992 |
| YMR041C | YMR041C | 0.43169213 | 1 | -0.3552394 | 114.6642975 | 233.2594774 |
| YFR025C | HIS2 | 0.49135407 | 1 | -0.3546598 | 78.48722558 | 174.2081764 |
| YLR046C | YLR046C | 0.46280531 | 1 | -0.3539703 | 105.697664 | 218.4765766 |
| YLR134W | PDC5 | 0.45230309 | 1 | -0.35307 | 97.29913399 | 204.6679073 |
| YMR173W-A | YMR173W-A | 0.42580147 | 1 | -0.351903 | 102.1566028 | 212.4343216 |
| YKL071W | YKL071W | 0.45668876 | 1 | -0.3516449 | 115.6282247 | 234.3620344 |
| YMR247C | RKR1 | 0.50967457 | 1 | -0.3506585 | 109.724049 | 224.6084091 |
| YGL046W | YGL046W | 0.54682634 | 1 | -0.3505097 | 99.7947026 | 208.4022249 |
| YML058W | SML1 | 0.41574702 | 1 | -0.3504909 | 96.05359799 | 202.3010484 |
| YKR061W | KTR2 | 0.5139578 | 1 | -0.3504902 | 112.3713921 | 228.9021551 |
| YKR046C | PET10 | 0.52006593 | 1 | -0.3502404 | 112.7360896 | 229.4640934 |
| YMR109W | MYO5 | 0.40615616 | 1 | -0.3493915 | 105.2180289 | 217.0974673 |
| YKL100C | YKL100C | 0.4555862 | 1 | -0.3482672 | 120.2176305 | 241.4030981 |
| YLR085C | ARP6 | 0.50690729 | 1 | -0.3474539 | 76.76925705 | 170.4676848 |
| YKL109W | HAP4 | 0.0361923 | 0.75809633 | -0.3471399 | 71.58442929 | 161.9744452 |
| YMR040W | YET2 | 0.42718257 | 1 | -0.3470731 | 108.4803208 | 222.1132399 |
| YNL022C | YNL022C | 0.52941195 | 1 | -0.3470383 | 115.8784256 | 234.1690641 |
| YML005W | TRM12 | 0.57016219 | 1 | -0.3465623 | 104.9015685 | 216.2125569 |
| YLR294C | YLR294C | 0.50364923 | 1 | -0.3463792 | 90.78333885 | 193.173198 |
| YLR394W | CST9 | 0.51018371 | 1 | -0.3463517 | 78.15100259 | 172.576437 |
| YMR320W | YMR320W | 0.54095967 | 1 | -0.3463205 | 116.5902645 | 235.2358709 |
| YMR042W | ARG80 | 0.50877628 | 1 | -0.345772 | 94.5818426 | 199.2863054 |
| YGL009C | LEU1 | 0.39892538 | 1 | -0.3457408 | 124.540378 | 248.1205089 |
| YML074C | FPR3 | 0.42278626 | 1 | -0.3457381 | 110.5703982 | 225.346353 |
| YJL117W | PHO86 | 0.46680299 | 1 | -0.3454963 | 90.97190419 | 193.3654414 |
| YER130C | YER130C | 0.38460751 | 1 | -0.3453655 | 118.2742473 | 237.8565366 |
| YLR193C | UPS1 | 0.37850066 | 1 | -0.3451733 | 68.06172064 | 155.9752384 |
| YKL040C | NFU1 | 0.51706417 | 1 | -0.3451379 | 110.584363 | 225.2908386 |
| YLR104W | YLR104W | 0.48261252 | 1 | -0.3450756 | 86.1055714 | 185.3774951 |
| YMR173W | DDR48 | 0.5102036 | 1 | -0.3440135 | 93.28484005 | 196.9425832 |
| YLR213C | CRR1 | 0.46714622 | 1 | -0.3436196 | 99.20343766 | 206.5396742 |
| YEL059W | YEL059W | 0.38128371 | 1 | -0.3435985 | 69.21550294 | 157.6507238 |
| YKL188C | PXA2 | 0.52441126 | 1 | -0.3434793 | 110.0244798 | 224.1617832 |
| YKL067W | YNK1 | 0.44702445 | 1 | -0.3433747 | 112.7948163 | 228.6643247 |
| YML019W | OST6 | 0.4221379 | 1 | -0.3433296 | 100.4105494 | 208.4696789 |
| YER080W | FMP29 | 0.37724762 | 1 | -0.3431713 | 114.8412767 | 231.9739312 |
| YNL130C | CPT1 | 0.54444807 | 1 | -0.3429537 | 116.5263766 | 234.6925879 |
| YNL196C | YNL196C | 0.5394709 | 1 | -0.3419195 | 114.0568429 | 230.5318721 |
| YNL322C | KRE1 | 0.51548784 | 1 | -0.3418279 | 108.8491089 | 222.0303085 |
| YMR283C | RIT1 | 0.45564443 | 1 | -0.3410815 | 118.5391072 | 237.7295449 |
| YMR196W | YMR196W | 0.4278047 | 1 | -0.3410238 | 106.4417178 | 218.0009024 |
| YFR019W | FAB1 | 0.33471212 | 1 | -0.3408521 | 120.8860931 | 241.5256634 |
| YMR232W | FUS2 | 0.47526531 | 1 | -0.3405448 | 107.2551325 | 219.264451 |
| YLR423C | ATG17 | 0.43370124 | 1 | -0.3402324 | 109.63494 | 223.1032591 |
| YML057W | CMP2 | 0.39573138 | 1 | -0.3401985 | 100.4607836 | 208.1431666 |
| YDL118W | YDL118W | 0.54744471 | 1 | -0.340195 | 82.62984121 | 179.0747902 |
| YLR367W | RPS22B | 0.44941571 | 1 | -0.3401065 | 91.63930227 | 193.7504356 |
| YLR241W | YLR241W | 0.46630483 | 1 | -0.339597 | 95.26983407 | 199.6024591 |
| YNL227C | JJJ1 | 0.36736655 | 1 | -0.3395413 | 70.58198428 | 159.3491671 |
| YLR258W | GSY2 | 0.53307164 | 1 | -0.3395412 | 92.47941543 | 195.0462524 |
| YKL178C | STE3 | 0.53726715 | 1 | -0.3394932 | 113.8251234 | 229.8376693 |
| YGL144C | ROG1 | 0.40219854 | 1 | -0.3389299 | 134.8495869 | 264.0381747 |
| YER185W | YER185W | 0.41467387 | 1 | -0.3387743 | 109.7949114 | 223.1738591 |

| | | | | | | |
|---|---|---|---|---|---|---|
| YLR420W | URA4 | 0.42838236 | 1 | -0.3386993 | 102.2513523 | 210.8666104 |
| YDR491C | YDR491C | 0.41365984 | 1 | -0.3382765 | 116.9285447 | 234.7381463 |
| YKL206C | ADD66 | 0.51389711 | 1 | -0.335763 | 107.293028 | 218.7025445 |
| YMR031C | YMR031C | 0.41894591 | 1 | -0.3357001 | 104.9251674 | 214.834254 |
| YMR073C | IRC21 | 0.3481107 | 1 | -0.3344341 | 84.02235312 | 180.5934567 |
| YMR011W | HXT2 | 0.40566547 | 1 | -0.3343878 | 104.2324139 | 213.5337693 |
| YLR285W | NNT1 | 0.46316683 | 1 | -0.3343228 | 98.4108283 | 204.0349759 |
| YMR251W | GTO3 | 0.53967178 | 1 | -0.3340642 | 111.7839267 | 225.8020046 |
| YMR106C | YKU80 | 0.44073576 | 1 | -0.3339 | 106.7947141 | 217.6471959 |
| YLR390W-A | CCW14 | 0.49330093 | 1 | -0.333646 | 80.66907445 | 175.0241673 |
| YMR101C | SRT1 | 0.43928261 | 1 | -0.3335113 | 112.0860847 | 226.2224706 |
| YMR010W | YMR010W | 0.50949779 | 1 | -0.3334725 | 93.31188221 | 195.6117923 |
| YLR344W | RPL26A | 0.48585219 | 1 | -0.3333238 | 83.26467186 | 179.2134734 |
| YLR319C | BUD6 | 0.60897935 | 1 | -0.3326141 | 102.9342134 | 211.1861053 |
| YNL173C | MDG1 | 0.54464512 | 1 | -0.3325339 | 111.8466051 | 225.7045888 |
| YMR023C | MSS1 | 0.44370602 | 1 | -0.3313892 | 109.7840237 | 222.1928724 |
| YMR070W | MOT3 | 0.42729801 | 1 | -0.331273 | 105.7100319 | 215.5363105 |
| YLL047W | YLL047W | 0.53047893 | 1 | -0.33127 | 94.27403593 | 196.8930045 |
| YLR342W | FKS1 | 0.38344162 | 1 | -0.3312145 | 67.25449342 | 152.8386327 |
| YML113W | DAT1 | 0.40223561 | 1 | -0.3310062 | 105.8566316 | 215.7404947 |
| YML058C-A | YML058C-A | 0.43784812 | 1 | -0.3306835 | 105.1599882 | 214.5627417 |
| YER066W | YER066W | 0.55574065 | 1 | -0.330604 | 95.10405367 | 198.15923 |
| YML097C | VPS9 | 0.47096929 | 1 | -0.330448 | 55.54463681 | 133.6493114 |
| YNL294C | RIM21 | 0.54556627 | 1 | -0.3300948 | 111.0316332 | 224.0578875 |
| YLR165C | PUS5 | 0.50392677 | 1 | -0.3298086 | 83.92205618 | 179.8266461 |
| YML002W | YML002W | 0.51681101 | 1 | -0.3297154 | 92.73451387 | 194.180525 |
| YOR209C | NPT1 | 0.47492265 | 1 | -0.3291968 | 112.0452708 | 225.5931848 |
| YLL005C | SPO75 | 0.4605932 | 1 | -0.3290847 | 111.059309 | 223.97125 |
| YMR019W | STB4 | 0.45916598 | 1 | -0.3282163 | 110.2639548 | 222.5613997 |
| YKL150W | MCR1 | 0.49421997 | 1 | -0.3281964 | 120.0495755 | 238.5112789 |
| YER163C | YER163C | 0.3801972 | 1 | -0.3280499 | 108.0269744 | 218.892989 |
| YMR160W | YMR160W | 0.55041939 | 1 | -0.3277541 | 97.55195225 | 201.778075 |
| YLR019W | PSR2 | 0.5565061 | 1 | -0.3273984 | 95.02897652 | 197.6187327 |
| YNL015W | PBI2 | 0.4977628 | 1 | -0.3268346 | 116.0117284 | 231.7511784 |
| YLR021W | IRC25 | 0.49626923 | 1 | -0.3267062 | 88.3641265 | 186.6634478 |
| YOR275C | RIM20 | 0.56339438 | 1 | -0.3258997 | 103.8807408 | 211.8533637 |
| YLR438W | CAR2 | 0.42673896 | 1 | -0.3257651 | 104.0460372 | 212.1052704 |
| YMR155W | YMR155W | 0.51795423 | 1 | -0.3254565 | 90.81126866 | 190.4897606 |
| YMR027W | YMR027W | 0.51517302 | 1 | -0.3252222 | 92.47598827 | 193.1730206 |
| YKL142W | MRP8 | 0.5146736 | 1 | -0.3246815 | 104.9365096 | 213.4155835 |
| YLR107W | REX3 | 0.53223742 | 1 | -0.3239208 | 91.46065648 | 191.3480933 |
| YMR034C | YMR034C | 0.46323485 | 1 | -0.3229923 | 105.7574606 | 214.53357 |
| YKL037W | YKL037W | 0.41226568 | 1 | -0.3226066 | 107.2027373 | 216.8393455 |
| YKL171W | YKL171W | 0.53202773 | 1 | -0.3221935 | 109.3241133 | 220.2437221 |
| YDR534C | FIT1 | 0.50568964 | 1 | -0.3219068 | 117.6353841 | 233.7553206 |
| YOR359W | VTS1 | 0.54571302 | 1 | -0.3218183 | 105.3318356 | 213.686593 |
| YER188W | YER188W | 0.42969394 | 1 | -0.3217322 | 115.6467993 | 230.4907702 |
| YLR287C-A | RPS30A | 0.45616059 | 1 | -0.3211303 | 77.37794776 | 168.0265531 |
| YLL038C | ENT4 | 0.53499464 | 1 | -0.3208148 | 107.2168575 | 216.6286545 |
| YDR423C | CAD1 | 0.39358085 | 1 | -0.3206506 | 114.0487814 | 227.7446094 |
| YMR175W | SIP18 | 0.47092861 | 1 | -0.3205195 | 109.223371 | 219.8611426 |
| YMR130W | YMR130W | 0.46849285 | 1 | -0.3197795 | 113.9492448 | 227.4687284 |
| YDR516C | EMI2 | 0.47115129 | 1 | -0.319735 | 117.6561715 | 233.5059377 |
| YKR043C | YKR043C | 0.47058433 | 1 | -0.3188186 | 112.3989353 | 224.816085 |
| YLR386W | VAC14 | 0.55366136 | 1 | -0.3182394 | 87.4178345 | 184.0164662 |
| YDR541C | YDR541C | 0.47883343 | 1 | -0.3176639 | 113.3032197 | 226.1396403 |
| YNL335W | DDI3 | 0.51501464 | 1 | -0.3172105 | 123.8717754 | 243.3093071 |
| YML051W | GAL80 | 0.43585796 | 1 | -0.3171499 | 105.6429345 | 213.58483 |
| YOR163W | DDP1 | 0.56946205 | 1 | -0.3170863 | 113.0352131 | 225.6274031 |
| YCR081W | SRB8 | 0.46305014 | 1 | -0.3166384 | 49.02621703 | 121.2218141 |
| YKL124W | SSH4 | 0.55391191 | 1 | -0.316169 | 110.5594898 | 221.4718485 |
| YMR132C | JLP2 | 0.52921881 | 1 | -0.3159418 | 94.40994239 | 195.1152857 |
| YNR001C | CIT1 | 0.54175249 | 1 | -0.315055 | 106.2564469 | 214.3117387 |
| YNR008W | LRO1 | 0.57915603 | 1 | -0.3141817 | 120.3960275 | 237.2481096 |

| | | | | | | |
|---|---|---|---|---|---|---|
| YNL028W | YNL028W | 0.51729219 | 1 | -0.3140326 | 124.5374868 | 243.9800521 |
| YNL314W | DAL82 | 0.56614146 | 1 | -0.313963 | 111.5731634 | 222.8365968 |
| YNR074C | AIF1 | 0.55701661 | 1 | -0.313693 | 107.338366 | 215.897828 |
| YNL168C | FMP41 | 0.54308823 | 1 | -0.3135313 | 109.1274964 | 218.793379 |
| YMR135C | GID8 | 0.53677982 | 1 | -0.3134883 | 105.7439249 | 213.2718902 |
| YGR097W | ASK10 | 0.48158997 | 1 | -0.3132638 | 118.9921707 | 234.8398303 |
| YER150W | SPI1 | 0.57989767 | 1 | -0.3132297 | 94.3795475 | 194.7120047 |
| YLR125W | YLR125W | 0.54152925 | 1 | -0.3129763 | 99.17570616 | 202.497617 |
| YDR469W | SDC1 | 0.52600519 | 1 | -0.3127162 | 74.05759845 | 161.5162757 |
| YLR122C | YLR122C | 0.51002958 | 1 | -0.3125348 | 100.5866069 | 204.7400759 |
| YML070W | DAK1 | 0.45823029 | 1 | -0.312446 | 103.0420248 | 208.7313051 |
| YLR426W | YLR426W | 0.50358188 | 1 | -0.3114303 | 90.71856588 | 188.5091904 |
| YFL026W | STE2 | 0.41756546 | 1 | -0.3114176 | 111.6585491 | 222.6437969 |
| YNL234W | YNL234W | 0.49215111 | 1 | -0.3112796 | 88.45844633 | 184.8050989 |
| YLL023C | YLL023C | 0.54740342 | 1 | -0.3112289 | 109.7241774 | 219.4657806 |
| YLR390W | ECM19 | 0.5594003 | 1 | -0.3108016 | 93.00746761 | 192.1585366 |
| YDR035W | ARO3 | 0.58719095 | 1 | -0.3107614 | 108.841424 | 217.9657448 |
| YFL053W | DAK2 | 0.51304672 | 1 | -0.3105585 | 120.2862579 | 236.5965858 |
| YKL200C | YKL200C | 0.53021886 | 1 | -0.3104808 | 113.2651209 | 225.1406338 |
| YMR233W | YMR233W | 0.49897558 | 1 | -0.3104082 | 114.0614217 | 226.4292857 |
| YLR453C | RIF2 | 0.56485565 | 1 | -0.3099836 | 99.8422539 | 203.1938831 |
| YLR039C | RIC1 | 0.51639936 | 1 | -0.3099234 | 86.30943278 | 181.1248919 |
| YLR181C | VTA1 | 0.48936328 | 1 | -0.3096033 | 97.303387 | 199.0054348 |
| YKL034W | TUL1 | 0.50494265 | 1 | -0.309127 | 123.2632291 | 241.2629237 |
| YLR199C | YLR199C | 0.40643632 | 1 | -0.3090676 | 106.7461767 | 214.3291566 |
| YMR141C | YMR141C | 0.44518297 | 1 | -0.3087825 | 95.52929951 | 196.0062603 |
| YLR180W | SAM1 | 0.4508643 | 1 | -0.308405 | 91.89077477 | 190.0255219 |
| YER175C | TMT1 | 0.44051855 | 1 | -0.3082907 | 113.5388778 | 225.3012573 |
| YNL237W | YTP1 | 0.58668139 | 1 | -0.3076215 | 117.0999755 | 231.0192463 |
| YJR108W | ABM1 | 0.55854909 | 1 | -0.3065839 | 110.457356 | 220.0551462 |
| YMR153W | NUP53 | 0.52232602 | 1 | -0.3063619 | 92.03106987 | 189.9877523 |
| YLR292C | SEC72 | 0.53000593 | 1 | -0.3056608 | 86.33849492 | 180.616289 |
| YNR012W | URK1 | 0.5835742 | 1 | -0.3056308 | 114.7117834 | 226.8663772 |
| YLR154C | RNH203 | 0.53582436 | 1 | -0.305085 | 81.83322726 | 173.1967287 |
| YNL009W | IDP3 | 0.61880705 | 1 | -0.3050786 | 130.6567457 | 252.7877585 |
| YKR032W | YKR032W | 0.59419045 | 1 | -0.3049808 | 120.3820666 | 236.0252784 |
| YNL032W | SIW14 | 0.59430161 | 1 | -0.3049197 | 118.2108829 | 232.4778545 |
| YJR124C | YJR124C | 0.5491106 | 1 | -0.3048339 | 107.5192172 | 215.0371531 |
| YKL133C | YKL133C | 0.56269618 | 1 | -0.304331 | 108.3910252 | 216.3927753 |
| YML054C | CYB2 | 0.48677099 | 1 | -0.3038066 | 107.6562682 | 215.1265845 |
| YMR056C | AAC1 | 0.52162345 | 1 | -0.3034427 | 116.2381588 | 229.0692792 |
| YLL051C | FRE6 | 0.54793207 | 1 | -0.3033735 | 91.06409149 | 188.0216004 |
| YLL042C | ATG10 | 0.57692243 | 1 | -0.3027752 | 112.511381 | 222.9068398 |
| YLR450W | HMG2 | 0.51673332 | 1 | -0.3026208 | 81.33635589 | 172.0653117 |
| YNL129W | NRK1 | 0.49785242 | 1 | -0.3018638 | 118.5481716 | 232.6291129 |
| YDR191W | HST4 | 0.63004528 | 1 | -0.3018101 | 133.0078955 | 256.194277 |
| YKL008C | LAC1 | 0.51821057 | 1 | -0.3015633 | 115.7846856 | 228.0848918 |
| YFL013W-A | YFL013W-A | 0.45929388 | 1 | -0.3012274 | 115.4865418 | 227.5550451 |
| YER084W | YER084W | 0.4816825 | 1 | -0.3010853 | 113.9283498 | 224.9963641 |
| YLL021W | SPA2 | 0.58225039 | 1 | -0.3010773 | 115.6958959 | 227.8767605 |
| YKR054C | DYN1 | 0.56009087 | 1 | -0.3008145 | 105.6741988 | 211.505159 |
| YLR220W | CCC1 | 0.50165901 | 1 | -0.3005785 | 98.46004689 | 199.7138981 |
| YLR110C | CCW12 | 0.52413752 | 1 | -0.3004525 | 82.55996864 | 173.7772359 |
| YGL175C | SAE2 | 0.41343456 | 1 | -0.300448 | 126.3259133 | 245.1236819 |
| YOR080W | DIA2 | 0.52949217 | 1 | -0.3004265 | 48.3125056 | 117.9437853 |
| YMR192W | GYL1 | 0.51585199 | 1 | -0.3000856 | 113.9972142 | 224.9782318 |
| YNL121C | TOM70 | 0.58529794 | 1 | -0.2994134 | 113.3683418 | 223.8653631 |
| YLR282C | YLR282C | 0.59295909 | 1 | -0.299319 | 93.74978131 | 191.8709648 |
| YLL056C | YLL056C | 0.54201486 | 1 | -0.2992882 | 101.198818 | 204.0103364 |
| YNL203C | YNL203C | 0.58435946 | 1 | -0.2992692 | 111.706253 | 221.1370252 |
| YKL056C | TMA19 | 0.55092526 | 1 | -0.2992627 | 97.79679868 | 198.4610546 |
| YEL015W | EDC3 | 0.59841654 | 1 | -0.2987995 | 100.0944707 | 202.1462877 |
| YFL018C | LPD1 | 0.60251897 | 1 | -0.2982336 | 84.75741265 | 177.0700821 |
| YNL319W | YNL319W | 0.5364253 | 1 | -0.2975134 | 100.6284212 | 202.8489913 |

| | | | | | | |
|---|---|---|---|---|---|---|
| YER124C | DSE1 | 0.4979618 | 1 | -0.2969232 | 113.2503161 | 223.3481692 |
| YML059C | NTE1 | 0.58331499 | 1 | -0.2965576 | 100.9926244 | 203.3180394 |
| YOR363C | PIP2 | 0.63162899 | 1 | -0.2965028 | 119.5879632 | 233.6249273 |
| YKR084C | HBS1 | 0.53442459 | 1 | -0.2964333 | 99.0247664 | 200.0938418 |
| YJR110W | YMR1 | 0.54308074 | 1 | -0.296418 | 102.9917965 | 206.5588797 |
| YKL001C | MET14 | 0.52360018 | 1 | -0.2961568 | 113.3243934 | 223.3689604 |
| YER078C | YER078C | 0.48124924 | 1 | -0.2959994 | 109.6689056 | 217.389276 |
| YKR101W | SIR1 | 0.5240832 | 1 | -0.2959562 | 123.5711183 | 240.0469594 |
| YEL071W | DLD3 | 0.49555876 | 1 | -0.2949083 | 120.454668 | 234.8298619 |
| YLR370C | ARC18 | 0.52548126 | 1 | -0.2946624 | 82.07519408 | 172.2317387 |
| YMR053C | STB2 | 0.48310191 | 1 | -0.2942165 | 105.7107926 | 210.7042308 |
| YJL024C | APS3 | 0.54811959 | 1 | -0.2941436 | 94.57526898 | 192.5416379 |
| YMR159C | ATG16 | 0.44531659 | 1 | -0.2934607 | 100.2382849 | 201.6843877 |
| YKL177W | YKL177W | 0.59579252 | 1 | -0.2931659 | 115.7394321 | 226.9158353 |
| YFL027C | GYP8 | 0.41143424 | 1 | -0.2930677 | 110.1104875 | 217.726754 |
| YLR059C | REX2 | 0.451188 | 1 | -0.2927158 | 88.07412945 | 181.757291 |
| YOR167C | RPS28A | 0.55016954 | 1 | -0.2915769 | 116.7479058 | 228.352597 |
| YLL053C | YLL053C | 0.55930661 | 1 | -0.2911079 | 87.98363508 | 181.4000468 |
| YFL011W | HXT10 | 0.49186281 | 1 | -0.2910324 | 119.058925 | 232.0489887 |
| YJR127C | RSF2 | 0.566131 | 1 | -0.2909809 | 106.7641098 | 211.9993226 |
| YNL183C | NPR1 | 0.64791481 | 1 | -0.2902548 | 123.0925496 | 238.5231632 |
| YER031C | YPT31 | 0.52471445 | 1 | -0.2901336 | 114.5444947 | 224.5723534 |
| YMR148W | YMR148W | 0.4422686 | 1 | -0.2900452 | 98.77898179 | 198.8599548 |
| YLL017W | YLL017W | 0.59748553 | 1 | -0.2898199 | 115.2520982 | 225.6849644 |
| YJR142W | YJR142W | 0.550961 | 1 | -0.289744 | 131.554289 | 252.2508284 |
| YMR189W | GCV2 | 0.48783782 | 1 | -0.2895595 | 108.5759841 | 214.7676374 |
| YKL096W-A | CWP2 | 0.53676432 | 1 | -0.2894483 | 123.3428399 | 238.8259838 |
| YFR020W | YFR020W | 0.40273426 | 1 | -0.2890201 | 126.5497499 | 243.9980238 |
| YNL117W | MLS1 | 0.53909225 | 1 | -0.2889336 | 121.5973228 | 235.9133219 |
| YKR048C | NAP1 | 0.56593587 | 1 | -0.288703 | 105.8642497 | 210.235268 |
| YGR100W | MDR1 | 0.43730431 | 1 | -0.2886844 | 125.4313005 | 242.1309487 |
| YKL222C | YKL222C | 0.57642464 | 1 | -0.2877365 | 108.5704667 | 214.5208592 |
| YLL057C | JLP1 | 0.51975717 | 1 | -0.2875 | 94.65831955 | 191.8104986 |
| YMR326C | YMR326C | 0.57259589 | 1 | -0.2867994 | 104.5765247 | 207.8877292 |
| YMR291W | YMR291W | 0.53057718 | 1 | -0.2867693 | 116.7486997 | 227.7268286 |
| YER002W | NOP16 | 0.59234338 | 1 | -0.2862698 | 97.92106488 | 196.9689508 |
| YER176W | ECM32 | 0.60612736 | 1 | -0.28626 | 93.06620205 | 189.0533074 |
| YLR143W | YLR143W | 0.56485442 | 1 | -0.2860432 | 85.8775325 | 177.3060909 |
| YDR528W | HLR1 | 0.54748403 | 1 | -0.2851357 | 121.3597455 | 235.0306596 |
| YMR172C-A | YMR172C-A | 0.49803846 | 1 | -0.2841973 | 105.5755708 | 209.1769821 |
| YLR214W | FRE1 | 0.59524221 | 1 | -0.2840804 | 80.31019454 | 167.974227 |
| YLR113W | HOG1 | 0.57440215 | 1 | -0.2838912 | 102.1971847 | 203.6296177 |
| YLR228C | ECM22 | 0.56611818 | 1 | -0.2835829 | 87.14009113 | 179.0434075 |
| YMR284W | YKU70 | 0.57162972 | 1 | -0.2835376 | 125.6582019 | 241.8295405 |
| YMR216C | SKY1 | 0.5154728 | 1 | -0.2832677 | 80.57081728 | 168.2930992 |
| YML116W | ATR1 | 0.48728379 | 1 | -0.2828136 | 102.7157802 | 204.3344845 |
| YLL015W | BPT1 | 0.62917764 | 1 | -0.2826063 | 121.4151371 | 234.7910515 |
| YLR108C | YLR108C | 0.60232754 | 1 | -0.2823294 | 107.1606534 | 211.5173418 |
| YOL110W | SHR5 | 0.61441868 | 1 | -0.2823218 | 111.4334625 | 218.4818577 |
| YER117W | RPL23B | 0.52303075 | 1 | -0.2822658 | 110.8904575 | 217.5893586 |
| YKL179C | COY1 | 0.54232719 | 1 | -0.2821186 | 116.5253624 | 226.7561453 |
| YLR300W | EXG1 | 0.6666162 | 1 | -0.2821114 | 122.9738219 | 237.2674578 |
| YPL208W | RKM1 | 0.70065257 | 1 | -0.2818326 | 97.04888416 | 194.9683801 |
| YML075C | HMG1 | 0.61983346 | 1 | -0.2816141 | 105.8814407 | 209.3386682 |
| YMR063W | RIM9 | 0.56623698 | 1 | -0.2815412 | 76.0080997 | 160.6297796 |
| YMR250W | GAD1 | 0.56304912 | 1 | -0.2812014 | 118.6838449 | 230.1552688 |
| YJR134C | SGM1 | 0.56395798 | 1 | -0.2809473 | 123.193112 | 237.4731088 |
| YER019C-A | SBH2 | 0.52279085 | 1 | -0.2799175 | 117.4247199 | 227.9351883 |
| YFL020C | PAU5 | 0.63410762 | 1 | -0.2797957 | 99.67124005 | 198.977653 |
| YJR103W | URA8 | 0.56504484 | 1 | -0.2794918 | 123.971388 | 238.552014 |
| YPR141C | KAR3 | 0.19677357 | 1 | -0.2789798 | 0 | 36.38764455 |
| YJR121W | ATP2 | 0.51917411 | 1 | -0.2785146 | 116.5233332 | 226.2827666 |
| YKR015C | YKR015C | 0.57517924 | 1 | -0.277834 | 103.0234924 | 204.1866138 |
| YER118C | SHO1 | 0.50117753 | 1 | -0.2773698 | 114.1493114 | 222.2633264 |

| | | | | | | |
|---|---|---|---|---|---|---|
| YKL096W | CWP1 | 0.55730548 | 1 | -0.2773234 | 125.8823359 | 241.3843992 |
| YLR362W | STE11 | 0.50643807 | 1 | -0.2772578 | 116.4996104 | 226.0801684 |
| YMR163C | INP2 | 0.57707134 | 1 | -0.2769272 | 89.77256207 | 182.4667405 |
| YKL164C | PIR1 | 0.60808516 | 1 | -0.2763243 | 113.8440635 | 221.6293453 |
| YMR147W | YMR147W | 0.52100658 | 1 | -0.276297 | 105.9806547 | 208.8068923 |
| YKL007W | CAP1 | 0.58606703 | 1 | -0.2757988 | 103.6608856 | 204.9602429 |
| YLR395C | COX8 | 0.51641589 | 1 | -0.2753761 | 97.66087206 | 195.1239114 |
| YMR068W | AVO2 | 0.49230568 | 1 | -0.2752037 | 103.3404539 | 204.36026 |
| YER177W | BMH1 | 0.43745377 | 1 | -0.2752024 | 103.0689237 | 203.917431 |
| YKL160W | ELF1 | 0.5757181 | 1 | -0.2750432 | 102.7234719 | 203.333513 |
| YMR164C | MSS11 | 0.52496316 | 1 | -0.275019 | 80.75776562 | 167.5219662 |
| YDR441C | APT2 | 0.44738455 | 1 | -0.2746723 | 108.8418097 | 213.2592278 |
| YGL236C | MTO1 | 0.4304268 | 1 | -0.2746703 | 130.5569708 | 248.6589227 |
| YJR109C | CPA2 | 0.42828673 | 1 | -0.2744672 | 111.3889976 | 217.3848949 |
| YNR025C | YNR025C | 0.62354834 | 1 | -0.274308 | 109.2763256 | 213.9200616 |
| YKR021W | ALY1 | 0.53227717 | 1 | -0.27426 | 111.7393099 | 217.9289456 |
| YNR002C | ATO2 | 0.55507564 | 1 | -0.2741341 | 98.04858239 | 195.5939605 |
| YKL183W | LOT5 | 0.5158741 | 1 | -0.2741229 | 110.6558273 | 216.1447744 |
| YKL064W | MNR2 | 0.59679411 | 1 | -0.2740873 | 106.7154067 | 209.7164738 |
| YNL127W | FAR11 | 0.58640945 | 1 | -0.2738999 | 122.9833179 | 236.2119033 |
| YFL034W | YFL034W | 0.4963849 | 1 | -0.2732254 | 108.6475762 | 212.7538743 |
| YGL019W | CKB1 | 0.55900245 | 1 | -0.2731493 | 82.56844731 | 170.229862 |
| YGL115W | SNF4 | 0.59923326 | 1 | -0.2730543 | 57.24326997 | 128.9324919 |
| YKR070W | YKR070W | 0.6181902 | 1 | -0.2727257 | 114.9693926 | 222.9944855 |
| YLR144C | ACF2 | 0.56134062 | 1 | -0.2726087 | 96.14642787 | 192.2941109 |
| YJR153W | PGU1 | 0.55508308 | 1 | -0.2725593 | 120.4517082 | 231.9100335 |
| YMR156C | TPP1 | 0.46486179 | 1 | -0.2721168 | 97.60711326 | 194.6111558 |
| YMR121C | RPL15B | 0.4836194 | 1 | -0.2719561 | 95.74220096 | 191.5500325 |
| YER019W | ISC1 | 0.50142921 | 1 | -0.2716219 | 115.8829143 | 224.3397393 |
| YNL298W | CLA4 | 0.52464584 | 1 | -0.2716114 | 85.06074464 | 174.0922115 |
| YMR058W | FET3 | 0.47303891 | 1 | -0.271582 | 101.1136957 | 200.2578195 |
| YKL033W-A | YKL033W-A | 0.57053971 | 1 | -0.270698 | 97.89376153 | 194.8933996 |
| YDR539W | YDR539W | 0.48819335 | 1 | -0.2706545 | 118.1031683 | 227.8330083 |
| YLR418C | CDC73 | 0.6267919 | 1 | -0.2706426 | 66.98747799 | 144.502887 |
| YNL332W | THI12 | 0.62534582 | 1 | -0.2706347 | 117.6229816 | 227.0476201 |
| YER143W | DDI1 | 0.50849866 | 1 | -0.2704755 | 116.0119749 | 224.4006057 |
| YOL116W | MSN1 | 0.63732888 | 1 | -0.2704734 | 112.9554156 | 219.4175439 |
| YKL066W | YKL066W | 0.54314296 | 1 | -0.2701154 | 119.0259109 | 229.2669397 |
| YKR056W | TRM2 | 0.53300098 | 1 | -0.2700247 | 116.018415 | 224.3523085 |
| YNL051W | COG5 | 0.57068578 | 1 | -0.2700171 | 116.5593215 | 225.2330927 |
| YLR290C | YLR290C | 0.56518921 | 1 | -0.2699534 | 87.59198346 | 178.0023735 |
| YNR062C | YNR062C | 0.61306152 | 1 | -0.2698881 | 110.6982599 | 215.6616014 |
| YMR138W | CIN4 | 0.59762464 | 1 | -0.269641 | 94.5256265 | 189.264819 |
| YML102C-A | YML102C-A | 0.4847877 | 1 | -0.2692976 | 106.2719424 | 208.3688152 |
| YGL082W | YGL082W | 0.43550997 | 1 | -0.269121 | 122.3764021 | 234.5991965 |
| YFL042C | YFL042C | 0.6029573 | 1 | -0.268821 | 90.04481256 | 181.8532586 |
| YDR506C | YDR506C | 0.58665193 | 1 | -0.2686221 | 85.92078707 | 175.1043456 |
| YMR104C | YPK2 | 0.50584569 | 1 | -0.2685764 | 104.57532 | 205.50892 |
| YPL161C | BEM4 | 0.55972363 | 1 | -0.2683968 | 90.6807655 | 182.8346629 |
| YMR007W | YMR007W | 0.49712326 | 1 | -0.2680891 | 100.4077147 | 198.6513531 |
| YML101C | CUE4 | 0.52543411 | 1 | -0.2678699 | 105.3560716 | 206.6895492 |
| YKR093W | PTR2 | 0.56959998 | 1 | -0.2677144 | 99.60016775 | 197.286017 |
| YKR099W | BAS1 | 0.57488379 | 1 | -0.267462 | 122.2619187 | 234.1961754 |
| YNL249C | MPA43 | 0.60674279 | 1 | -0.2668264 | 109.837896 | 213.8596897 |
| YMR209C | YMR209C | 0.56864821 | 1 | -0.266693 | 113.5331653 | 219.8662986 |
| YLR242C | ARV1 | 0.56143971 | 1 | -0.2665965 | 80.70612343 | 166.3392248 |
| YMR102C | YMR102C | 0.51595961 | 1 | -0.2664391 | 100.0830883 | 197.9069312 |
| YKL117W | SBA1 | 0.62089582 | 1 | -0.2664332 | 113.2019752 | 219.2925166 |
| YER059W | PCL6 | 0.46717515 | 1 | -0.2661565 | 116.2239064 | 224.1827567 |
| YGR153W | YGR153W | 0.49334035 | 1 | -0.2661217 | 114.5068349 | 221.3790547 |
| YLR337C | VRP1 | 0.5947852 | 1 | -0.2660501 | 44.69259174 | 107.5588653 |
| YNL202W | SPS19 | 0.59477373 | 1 | -0.2657263 | 133.0254297 | 251.5164165 |
| YNR056C | BIO5 | 0.67469364 | 1 | -0.2655144 | 126.9555772 | 241.5937282 |
| YKL026C | GPX1 | 0.57280806 | 1 | -0.2655129 | 122.9798135 | 235.1122617 |

| | | | | | | |
|---|---|---|---|---|---|---|
| YKR045C | YKR045C | 0.57608799 | 1 | -0.2654903 | 120.8015727 | 231.5583535 |
| YHR194W | MDM31 | 0.57866715 | 1 | -0.2653858 | 106.9301207 | 208.9315484 |
| YMR271C | URA10 | 0.64568529 | 1 | -0.2641629 | 120.4298102 | 230.7791734 |
| YOR156C | NFI1 | 0.65214347 | 1 | -0.2638129 | 121.6948615 | 232.795808 |
| YLR172C | DPH5 | 0.52972344 | 1 | -0.2631387 | 98.14739391 | 194.3208945 |
| YGR055W | MUP1 | 0.65463377 | 1 | -0.2630914 | 44.95418989 | 107.5994141 |
| YKR031C | SPO14 | 0.61364038 | 1 | -0.2630153 | 109.4574561 | 212.7424199 |
| YER137C | YER137C | 0.49632667 | 1 | -0.2628552 | 110.5895491 | 214.5670608 |
| YLR184W | YLR184W | 0.53459366 | 1 | -0.2625964 | 79.40041738 | 163.688934 |
| YDR524C | AGE1 | 0.60156134 | 1 | -0.2624444 | 92.02287116 | 184.2461732 |
| YMR225C | MRPL44 | 0.54087022 | 1 | -0.2623242 | 111.1413573 | 215.3973693 |
| YER041W | YEN1 | 0.5171171 | 1 | -0.2622361 | 116.2097266 | 223.6483012 |
| YLL058W | YLL058W | 0.503215 | 1 | -0.2621899 | 114.7863512 | 221.3218932 |
| YPL048W | CAM1 | 0.65949992 | 1 | -0.261941 | 107.8878957 | 210.0436085 |
| YMR204C | INP1 | 0.47851649 | 1 | -0.2618238 | 100.430635 | 197.8715201 |
| YDR533C | HSP31 | 0.52374546 | 1 | -0.2612303 | 119.7004924 | 229.2077469 |
| YKR067W | GPT2 | 0.57440442 | 1 | -0.2610451 | 119.5808979 | 228.9886292 |
| YNL293W | MSB3 | 0.63785256 | 1 | -0.2609983 | 118.0538683 | 226.4931678 |
| YLL044W | YLL044W | 0.60317979 | 1 | -0.2608352 | 127.8313297 | 242.4110733 |
| YOL111C | MDY2 | 0.68064453 | 1 | -0.2607729 | 126.0124025 | 239.4377312 |
| YKL157W | APE2 | 0.59332926 | 1 | -0.2605867 | 126.0556031 | 239.4838679 |
| YMR124W | YMR124W | 0.62707686 | 1 | -0.2603715 | 96.76732841 | 191.7101894 |
| YOL061W | PRS5 | 0.63270175 | 1 | -0.2600905 | 110.8329638 | 214.6032767 |
| YGR011W | YGR011W | 0.50361588 | 1 | -0.2600126 | 123.5563128 | 235.3346681 |
| YLR095C | IOC2 | 0.5395232 | 1 | -0.2598858 | 79.08720599 | 162.8247901 |
| YLR030W | YLR030W | 0.61557985 | 1 | -0.2596841 | 95.05499102 | 188.8290914 |
| YKL161C | YKL161C | 0.56329119 | 1 | -0.2596306 | 112.4687571 | 217.2099587 |
| YFL025C | BST1 | 0.49241778 | 1 | -0.2595615 | 103.3500402 | 202.3356481 |
| YLR333C | RPS25B | 0.60564138 | 1 | -0.2591968 | 89.99203889 | 180.511937 |
| YMR272C | SCS7 | 0.54129327 | 1 | -0.2588678 | 112.0057049 | 216.3555966 |
| YMR029C | FAR8 | 0.52499121 | 1 | -0.2587875 | 104.3706397 | 203.8984753 |
| YMR285C | NGL2 | 0.59850111 | 1 | -0.258769 | 126.5411957 | 240.0384038 |
| YKR030W | GMH1 | 0.56836169 | 1 | -0.2586409 | 96.57654327 | 191.1734597 |
| YKL114C | APN1 | 0.5702519 | 1 | -0.2585677 | 116.2301523 | 223.2031232 |
| YMR057C | YMR057C | 0.47721642 | 1 | -0.2582272 | 100.2558505 | 197.1174869 |
| YNL285W | YNL285W | 0.66986182 | 1 | -0.2581505 | 124.6318855 | 236.8451829 |
| YKR007W | MEH1 | 0.59724425 | 1 | -0.2578819 | 101.4606352 | 199.0364816 |
| YKL132C | RMA1 | 0.57841916 | 1 | -0.2575563 | 120.6503056 | 230.2769292 |
| YLR433C | CNA1 | 0.5559145 | 1 | -0.2572878 | 105.7460125 | 205.9449966 |
| YMR181C | YMR181C | 0.51224726 | 1 | -0.2563763 | 105.9865532 | 206.218237 |
| YJR147W | HMS2 | 0.55758285 | 1 | -0.2562703 | 113.2357171 | 218.021961 |
| YKL091C | YKL091C | 0.61044253 | 1 | -0.2560514 | 107.4455564 | 208.5543203 |
| YOL043C | NTG2 | 0.67166699 | 1 | -0.2559802 | 108.1443361 | 209.6841825 |
| YOL013C | HRD1 | 0.67406908 | 1 | -0.255966 | 123.5513723 | 234.7988068 |
| YMR157C | FMP39 | 0.56233679 | 1 | -0.2555866 | 105.9577138 | 206.0682168 |
| YKL187C | YKL187C | 0.58814854 | 1 | -0.2548866 | 119.8684918 | 228.654205 |
| YOL162W | YOL162W | 0.65878867 | 1 | -0.2546934 | 114.1058348 | 219.2347425 |
| YLR442C | SIR3 | 0.62027057 | 1 | -0.2544457 | 126.0525559 | 238.6779327 |
| YER098W | UBP9 | 0.64099424 | 1 | -0.2539951 | 91.79210344 | 182.7679253 |
| YNL280C | ERG24 | 0.55818145 | 1 | -0.2538468 | 110.4191061 | 213.1142398 |
| YMR008C | PLB1 | 0.48978926 | 1 | -0.253685 | 98.8226963 | 194.1887185 |
| YLR268W | SEC22 | 0.59571676 | 1 | -0.2536081 | 85.95222067 | 173.1972951 |
| YOR327C | SNC2 | 0.65779976 | 1 | -0.2534474 | 110.5681564 | 213.305124 |
| YDR494W | RSM28 | 0.56536191 | 1 | -0.2534113 | 117.680017 | 224.8941389 |
| YNL206C | RTT106 | 0.6360925 | 1 | -0.2533905 | 107.7003289 | 208.6225849 |
| YKL076C | PSY1 | 0.60828291 | 1 | -0.2533168 | 103.0156482 | 200.9760209 |
| YLL013C | PUF3 | 0.59582856 | 1 | -0.2532528 | 123.7565361 | 234.7793717 |
| YML020W | YML020W | 0.52839899 | 1 | -0.2531209 | 105.2385218 | 204.5741957 |
| YJL175W | YJL175W | 0.51173867 | 1 | -0.2529599 | 47.00699636 | 109.6244284 |
| YEL039C | CYC7 | 0.62463073 | 1 | -0.2524152 | 92.32329898 | 183.4278123 |
| YKR014C | YPT52 | 0.57062597 | 1 | -0.2521208 | 117.1632078 | 223.883311 |
| YKR023W | YKR023W | 0.58146082 | 1 | -0.2519911 | 97.3514532 | 191.5693685 |
| YKR044W | UIP5 | 0.54392055 | 1 | -0.2519536 | 117.5520413 | 224.495377 |
| YGL020C | GET1 | 0.46107162 | 1 | -0.25181 | 124.2599021 | 235.4117725 |

| | | | | | | |
|---|---|---|---|---|---|---|
| YML062C | MFT1 | 0.55388157 | 1 | -0.2515943 | 81.34479085 | 165.4236257 |
| YLR446W | YLR446W | 0.51909726 | 1 | -0.2503304 | 96.45941884 | 189.8985732 |
| YPL106C | SSE1 | 0.64631529 | 1 | -0.2501938 | 68.38153344 | 144.108319 |
| YNL049C | SFB2 | 0.58922417 | 1 | -0.2501238 | 120.420797 | 228.933352 |
| YOR364W | YOR364W | 0.67622344 | 1 | -0.2500778 | 108.0396007 | 208.7435765 |
| YOR136W | IDH2 | 0.6729285 | 1 | -0.2498043 | 120.8017147 | 229.5126446 |
| YML035C-A | YML035C-A | 0.61449984 | 1 | -0.2497394 | 89.17215415 | 177.9418162 |
| YLR004C | THI73 | 0.58651606 | 1 | -0.2494926 | 97.02152207 | 190.7056395 |
| YNL255C | GIS2 | 0.5761543 | 1 | -0.2494625 | 116.0370176 | 221.7006844 |
| YMR222C | FSH2 | 0.540837 | 1 | -0.2493649 | 108.8485457 | 209.969331 |
| YEL031W | SPF1 | 0.5783674 | 1 | -0.248216 | 80.46988046 | 163.5567118 |
| YEL001C | IRC22 | 0.56518477 | 1 | -0.2480372 | 116.2292294 | 221.8281219 |
| YKL050C | YKL050C | 0.63017816 | 1 | -0.2480184 | 109.0063503 | 210.0509686 |
| YKL098W | MTC2 | 0.61119673 | 1 | -0.2480089 | 103.9225677 | 201.7621622 |
| YLR232W | YLR232W | 0.59144362 | 1 | -0.2477924 | 99.64889254 | 194.767007 |
| YER109C | FLO8 | 0.51906013 | 1 | -0.247291 | 115.7615867 | 220.9684369 |
| YNL074C | MLF3 | 0.6347123 | 1 | -0.2472184 | 105.8454921 | 204.7937964 |
| YNL078W | NIS1 | 0.60035524 | 1 | -0.2468539 | 99.80332337 | 194.8963445 |
| YLR366W | YLR366W | 0.56406216 | 1 | -0.2466395 | 92.380695 | 182.7680455 |
| YDR452W | PPN1 | 0.54578048 | 1 | -0.2462102 | 123.9700077 | 234.2087983 |
| YJR149W | YJR149W | 0.65341653 | 1 | -0.2457093 | 116.9591552 | 222.7144084 |
| YJR100C | YJR100C | 0.56720576 | 1 | -0.2453983 | 110.7539306 | 212.5581186 |
| YDR538W | PAD1 | 0.54808121 | 1 | -0.2453566 | 113.453937 | 216.9542125 |
| YJR130C | STR2 | 0.59654575 | 1 | -0.2453397 | 107.3808293 | 207.0516621 |
| YJR039W | YJR039W | 0.71198834 | 1 | -0.2450196 | 87.36515783 | 174.380458 |
| YER075C | PTP3 | 0.53539857 | 1 | -0.2447305 | 108.085281 | 208.120599 |
| YNL175C | NOP13 | 0.64633692 | 1 | -0.2446313 | 112.5429305 | 215.3744997 |
| YML009C | MRPL39 | 0.58255675 | 1 | -0.2443289 | 111.2736098 | 213.2658152 |
| YER077C | YER077C | 0.65569983 | 1 | -0.244092 | 95.98966883 | 188.3190986 |
| YLR028C | ADE16 | 0.55424189 | 1 | -0.243966 | 96.41250012 | 188.9919646 |
| YOR038C | HIR2 | 0.6344993 | 1 | -0.2436341 | 121.5109067 | 229.86398 |
| YOR017W | PET127 | 0.67684346 | 1 | -0.2435816 | 119.4658241 | 226.5232571 |
| YKR103W | NFT1 | 0.68825476 | 1 | -0.2433869 | 125.2785786 | 235.9737855 |
| YMR115W | FMP24 | 0.60014901 | 1 | -0.2427807 | 87.57678631 | 174.4334316 |
| YGL002W | ERP6 | 0.52625874 | 1 | -0.2427409 | 123.4164529 | 232.8538932 |
| YER034W | YER034W | 0.69853704 | 1 | -0.2424075 | 83.08446658 | 167.061391 |
| YGR133W | PEX4 | 0.62412278 | 1 | -0.242307 | 106.8766258 | 205.8341559 |
| YDR476C | YDR476C | 0.58964287 | 1 | -0.2422931 | 120.2079868 | 227.5650664 |
| YNL162W | RPL42A | 0.6090613 | 1 | -0.2422416 | 100.396593 | 195.261903 |
| YGL156W | AMS1 | 0.48101198 | 1 | -0.242097 | 124.3683863 | 234.3217529 |
| YKL025C | PAN3 | 0.66184619 | 1 | -0.2417315 | 117.4819155 | 223.0477774 |
| YMR060C | SAM37 | 0.54305541 | 1 | -0.2414118 | 72.33682228 | 149.4107614 |
| YPL195W | APL5 | 0.64333123 | 1 | -0.2412114 | 104.6940617 | 202.1332464 |
| YNL099C | OCA1 | 0.63704106 | 1 | -0.2409183 | 94.31317305 | 185.1721453 |
| YMR122C | YMR122C | 0.53108607 | 1 | -0.240717 | 103.2164458 | 199.6599591 |
| YMR234W | RNH1 | 0.51982739 | 1 | -0.2406273 | 103.4141064 | 199.9704784 |
| YKR049C | FMP46 | 0.59023372 | 1 | -0.2394199 | 117.7335764 | 223.156541 |
| YOR040W | GLO4 | 0.69787955 | 1 | -0.2393401 | 126.0697924 | 236.7357914 |
| YDR525W-A | SNA2 | 0.56951296 | 1 | -0.2386269 | 117.4307572 | 222.5594528 |
| YNL143C | YNL143C | 0.51696404 | 1 | -0.2383988 | 90.30167973 | 178.3040051 |
| YLL012W | YEH1 | 0.59397226 | 1 | -0.2383479 | 117.0845444 | 221.9586696 |
| YKR017C | YKR017C | 0.57423495 | 1 | -0.2377767 | 107.6723154 | 206.5403906 |
| YLL024C | SSA2 | 0.58914059 | 1 | -0.2376682 | 113.0732113 | 215.3307566 |
| YFL052W | YFL052W | 0.59330333 | 1 | -0.2375181 | 119.1814905 | 225.2688695 |
| YMR304W | UBP15 | 0.5622864 | 1 | -0.2374796 | 110.9628405 | 211.865836 |
| YNL041C | COG6 | 0.61477093 | 1 | -0.2373339 | 100.4451769 | 194.7009809 |
| YKL159C | RCN1 | 0.56735377 | 1 | -0.2373136 | 109.2100105 | 208.986734 |
| YJR107W | YJR107W | 0.61946857 | 1 | -0.2370489 | 126.3247527 | 236.8525762 |
| YER095W | RAD51 | 0.71432425 | 1 | -0.2368758 | 80.92837341 | 162.8250341 |
| YMR110C | HFD1 | 0.5798171 | 1 | -0.2368514 | 108.9868137 | 208.5625968 |
| YMR100W | MUB1 | 0.61870385 | 1 | -0.2366379 | 87.16929889 | 172.9679321 |
| YOR025W | HST3 | 0.68642152 | 1 | -0.2366325 | 119.1472037 | 225.0974594 |
| YER001W | MNN1 | 0.56298639 | 1 | -0.2366274 | 117.9329233 | 223.1172775 |
| YPR070W | MED1 | 0.47376526 | 1 | -0.2366196 | 4.068637695 | 37.49522565 |

| | | | | | | |
|---|---|---|---|---|---|---|
| YOL019W | YOL019W | 0.68904814 | 1 | -0.2363901 | 120.0846362 | 226.5940378 |
| YOL104C | NDJ1 | 0.66843295 | 1 | -0.2362196 | 110.7651374 | 211.3792011 |
| YLR131C | ACE2 | 0.50317881 | 1 | -0.2361085 | 95.32794978 | 186.1990759 |
| YML037C | YML037C | 0.61436053 | 1 | -0.2356363 | 114.3352749 | 217.1231398 |
| YLR325C | RPL38 | 0.626303 | 1 | -0.235614 | 85.92687586 | 170.8089897 |
| YNL230C | ELA1 | 0.56860292 | 1 | -0.235591 | 114.9508488 | 218.120735 |
| YEL038W | UTR4 | 0.56900663 | 1 | -0.2353295 | 119.7435241 | 225.8996215 |
| YER056C | FCY2 | 0.58416986 | 1 | -0.235269 | 119.3595136 | 225.2657189 |
| YKL020C | SPT23 | 0.62381446 | 1 | -0.2347063 | 124.0262505 | 232.8000168 |
| YER067W | YER067W | 0.54642502 | 1 | -0.2345375 | 114.0524066 | 216.5186933 |
| YNR057C | BIO4 | 0.65687737 | 1 | -0.2338505 | 131.2138277 | 244.4055569 |
| YMR085W | YMR085W | 0.61826045 | 1 | -0.2338387 | 110.9876399 | 211.4313847 |
| YDR479C | PEX29 | 0.54891007 | 1 | -0.2337832 | 112.560669 | 213.9884842 |
| YML108W | YML108W | 0.53599257 | 1 | -0.2337277 | 103.3904209 | 199.031954 |
| YOR086C | TCB1 | 0.70377203 | 1 | -0.2334989 | 126.1521548 | 236.1081758 |
| YML013C-A | YML013C-A | 0.58980808 | 1 | -0.2334775 | 64.12767009 | 134.9933595 |
| YKR001C | VPS1 | 0.60684195 | 1 | -0.2334495 | 94.47820296 | 184.4670096 |
| YMR092C | AIP1 | 0.55013392 | 1 | -0.2331334 | 98.64753021 | 191.2225889 |
| YLR324W | PEX30 | 0.62390724 | 1 | -0.2329284 | 100.3561201 | 193.9811975 |
| YNR040W | YNR040W | 0.70623881 | 1 | -0.2325222 | 120.4866291 | 226.7448766 |
| YLR436C | ECM30 | 0.52527937 | 1 | -0.2324774 | 94.1413845 | 183.7911372 |
| YNL254C | RTC4 | 0.66126817 | 1 | -0.2324557 | 111.9207012 | 212.7720592 |
| YMR252C | YMR252C | 0.64505065 | 1 | -0.2323657 | 129.0919893 | 240.7528803 |
| YER184C | YER184C | 0.54617754 | 1 | -0.2322866 | 114.6184427 | 217.1478507 |
| YMR226C | TMA29 | 0.61747646 | 1 | -0.2322333 | 91.15472385 | 178.8904601 |
| YLR098C | CHA4 | 0.65774382 | 1 | -0.2321533 | 88.94398415 | 175.2760879 |
| YNL043C | YNL043C | 0.68314451 | 1 | -0.2320014 | 120.1030759 | 226.0516727 |
| YOR012W | YOR012W | 0.69676826 | 1 | -0.2317086 | 120.7852947 | 227.1256359 |
| YNL094W | APP1 | 0.63165913 | 1 | -0.231498 | 130.898972 | 243.5854338 |
| YFL028C | CAF16 | 0.56537819 | 1 | -0.2314032 | 108.547294 | 207.1354691 |
| YOL160W | YOL160W | 0.70246511 | 1 | -0.2312972 | 124.1788776 | 232.6041845 |
| YNR018W | YNR018W | 0.66383968 | 1 | -0.2312509 | 110.7814015 | 210.7576397 |
| YNL273W | TOF1 | 0.66107415 | 1 | -0.2312212 | 109.8901524 | 209.3008547 |
| YMR178W | YMR178W | 0.56791585 | 1 | -0.2307301 | 106.3642877 | 203.4889574 |
| YKR059W | TIF1 | 0.62564499 | 1 | -0.2307228 | 123.1090711 | 230.7852644 |
| YDR492W | IZH1 | 0.59566132 | 1 | -0.2302621 | 119.7355078 | 225.2256168 |
| YML128C | MSC1 | 0.61126079 | 1 | -0.2302316 | 79.59428552 | 159.7835925 |
| YMR279C | YMR279C | 0.66562836 | 1 | -0.2301875 | 111.5846635 | 211.9284159 |
| YOR123C | LEO1 | 0.69259449 | 1 | -0.2301643 | 113.8918538 | 215.6865543 |
| YLR177W | YLR177W | 0.61648785 | 1 | -0.2291708 | 68.93490868 | 142.2683669 |
| YLR119W | SRN2 | 0.63418927 | 1 | -0.2291652 | 85.13961517 | 168.684484 |
| YKR011C | YKR011C | 0.64476904 | 1 | -0.2287321 | 130.9903395 | 243.3736276 |
| YKL130C | SHE2 | 0.67747712 | 1 | -0.2282788 | 116.4080137 | 219.5424657 |
| YNL283C | WSC2 | 0.6130203 | 1 | -0.2282063 | 115.2132736 | 217.5853444 |
| YMR289W | ABZ2 | 0.66825325 | 1 | -0.2281207 | 111.2422187 | 211.100584 |
| YMR274C | RCE1 | 0.69278975 | 1 | -0.2278944 | 122.9352721 | 230.1330255 |
| YEL012W | UBC8 | 0.69333781 | 1 | -0.2276726 | 103.1506609 | 197.851315 |
| YFL049W | SWP82 | 0.55085955 | 1 | -0.227482 | 112.4057719 | 212.9141034 |
| YMR087W | YMR087W | 0.53576288 | 1 | -0.2271904 | 98.48357957 | 190.180177 |
| YER064C | YER064C | 0.57687236 | 1 | -0.2270189 | 114.7689634 | 216.7061596 |
| YGL249W | ZIP2 | 0.4908451 | 1 | -0.2269274 | 124.8692647 | 233.1596861 |
| YGL041C | YGL041C | 0.53573305 | 1 | -0.2267886 | 127.3566146 | 237.196448 |
| YML094W | GIM5 | 0.55564427 | 1 | -0.2266413 | 103.8977347 | 198.934677 |
| YML122C | YML122C | 0.58024323 | 1 | -0.2265154 | 108.3105446 | 206.1120068 |
| YOR164C | YOR164C | 0.69086957 | 1 | -0.2263316 | 116.4658394 | 219.3827505 |
| YKL069W | YKL069W | 0.66120381 | 1 | -0.2262862 | 67.33311081 | 139.2808792 |
| YKL113C | RAD27 | 0.53430453 | 1 | -0.2257917 | 76.41084032 | 154.0148626 |
| YKL168C | KKQ8 | 0.62358377 | 1 | -0.2257079 | 119.0104778 | 223.4496651 |
| YDR504C | SPG3 | 0.60348386 | 1 | -0.225645 | 119.4293743 | 224.1243437 |
| YOR109W | INP53 | 0.7024819 | 1 | -0.2255442 | 117.7611358 | 221.3916372 |
| YML096W | YML096W | 0.57073171 | 1 | -0.2254434 | 103.0231326 | 197.3526704 |
| YLR040C | YLR040C | 0.62595621 | 1 | -0.2249334 | 100.0933351 | 192.5100034 |
| YMR030W | RSF1 | 0.5881507 | 1 | -0.2249123 | 106.0271977 | 202.1806143 |
| YOR062C | YOR062C | 0.7006668 | 1 | -0.2245435 | 115.645343 | 217.8119579 |

| | | | | | | |
|---|---|---|---|---|---|---|
| YOL003C | PFA4 | 0.68361424 | 1 | -0.224188 | 115.0599096 | 216.8112273 |
| YKL202W | YKL202W | 0.64701842 | 1 | -0.2241169 | 104.7101446 | 199.9298152 |
| YER140W | YER140W | 0.56483806 | 1 | -0.2238698 | 117.1397107 | 220.1601956 |
| YKR016W | FMP13 | 0.65851429 | 1 | -0.2234089 | 107.5774616 | 204.5117477 |
| YHR086W | NAM8 | 0.7295597 | 1 | -0.223359 | 112.1899896 | 212.0245617 |
| YOR132W | VPS17 | 0.64701969 | 1 | -0.2232899 | 116.5152462 | 219.0665705 |
| YGL227W | VID30 | 0.61225803 | 1 | -0.2232773 | 134.5553122 | 248.4737567 |
| YER145C | FTR1 | 0.58476563 | 1 | -0.2230981 | 116.2163689 | 218.5543238 |
| YKR090W | PXL1 | 0.63819162 | 1 | -0.2230502 | 123.1742238 | 229.8907321 |
| YMR075W | RCO1 | 0.65360183 | 1 | -0.2229865 | 92.76066475 | 180.3023821 |
| YML100W | TSL1 | 0.57709439 | 1 | -0.2228142 | 106.4598396 | 202.6122395 |
| YOR142W | LSC1 | 0.68010062 | 1 | -0.2226807 | 104.4300481 | 199.2858679 |
| YOR172W | YRM1 | 0.71059183 | 1 | -0.2225435 | 122.9411305 | 229.4446589 |
| YFL032W | YFL032W | 0.57637789 | 1 | -0.2223995 | 120.3081248 | 225.133561 |
| YER167W | BCK2 | 0.63030992 | 1 | -0.2221097 | 124.5146256 | 231.9531878 |
| YOR084W | YOR084W | 0.70861811 | 1 | -0.2218975 | 117.5152744 | 220.5151977 |
| YKL030W | YKL030W | 0.70909857 | 1 | -0.2213202 | 126.1564441 | 234.5266942 |
| YMR297W | PRC1 | 0.68988036 | 1 | -0.2212853 | 118.0985575 | 221.3862067 |
| YMR044W | IOC4 | 0.56216047 | 1 | -0.221206 | 103.4415483 | 197.4820824 |
| YOL106W | YOL106W | 0.72014117 | 1 | -0.2209475 | 124.8631348 | 232.3697265 |
| YOR030W | DFG16 | 0.65551493 | 1 | -0.2208836 | 125.418051 | 233.266022 |
| YMR180C | CTL1 | 0.60003068 | 1 | -0.2193222 | 105.4014131 | 200.4313346 |
| YLR441C | RPS1A | 0.56416614 | 1 | -0.2186594 | 88.10035747 | 172.1407821 |
| YKR080W | MTD1 | 0.62010621 | 1 | -0.2185639 | 117.2612285 | 219.6662385 |
| YER174C | GRX4 | 0.69873934 | 1 | -0.2185255 | 137.9119967 | 253.3260262 |
| YOR165W | SEY1 | 0.7206055 | 1 | -0.2185004 | 124.5704419 | 231.5734056 |
| YML036W | CGI121 | 0.59915517 | 1 | -0.2184483 | 100.7360311 | 192.71187 |
| YOL025W | LAG2 | 0.68878284 | 1 | -0.2184162 | 129.9487173 | 240.330063 |
| YOR121C | YOR121C | 0.70655902 | 1 | -0.2183362 | 118.3575551 | 221.4237782 |
| YKL051W | SFK1 | 0.67237245 | 1 | -0.2182188 | 110.2432187 | 208.1804997 |
| YFL035C-B | YFL035C-B | 0.51070279 | 1 | -0.2178812 | 111.2734619 | 209.8159671 |
| YER129W | SAK1 | 0.60432485 | 1 | -0.2176497 | 119.3566177 | 222.9629028 |
| YKL092C | BUD2 | 0.67416268 | 1 | -0.2174572 | 111.507838 | 210.1427507 |
| YLR451W | LEU3 | 0.50142075 | 1 | -0.2174136 | 95.89817668 | 184.6902547 |
| YNR031C | SSK2 | 0.72172297 | 1 | -0.2171927 | 125.4074632 | 232.7673577 |
| YLR422W | YLR422W | 0.59196676 | 1 | -0.2168688 | 105.4715781 | 200.225711 |
| YKR051W | YKR051W | 0.62828064 | 1 | -0.2161514 | 122.3199847 | 227.5983362 |
| YMR177W | MMT1 | 0.58854528 | 1 | -0.2158048 | 104.1861168 | 197.9913849 |
| YDR451C | YHP1 | 0.61858315 | 1 | -0.2157472 | 127.2821898 | 235.6349894 |
| YMR037C | MSN2 | 0.66534631 | 1 | -0.2154093 | 118.4562271 | 221.2028725 |
| YMR052W | FAR3 | 0.59607271 | 1 | -0.2153367 | 107.7907441 | 203.8065826 |
| YOL113W | SKM1 | 0.73472053 | 1 | -0.2151833 | 127.0324981 | 235.1543849 |
| YLR182W | SWI6 | 0.36236172 | 1 | -0.2151148 | 18.50503143 | 58.22448181 |
| YOR022C | YOR022C | 0.70377001 | 1 | -0.2151131 | 114.6853717 | 215.0170091 |
| YML117W-A | YML117W-A | 0.51085806 | 1 | -0.2148165 | 88.45202516 | 172.2128327 |
| YOR263C | YOR263C | 0.72116459 | 1 | -0.2138519 | 124.5876747 | 230.9951867 |
| YML068W | ITT1 | 0.58924259 | 1 | -0.2137891 | 100.075253 | 191.0269613 |
| YOR008C-A | YOR008C-A | 0.65618294 | 1 | -0.2135304 | 127.1031159 | 235.0539146 |
| YOL057W | YOL057W | 0.72901624 | 1 | -0.2134603 | 121.6141106 | 226.0966189 |
| YOR112W | YOR112W | 0.7158217 | 1 | -0.2134005 | 116.9131305 | 218.4253061 |
| YKL073W | LHS1 | 0.65878324 | 1 | -0.2125168 | 103.4957498 | 196.4371005 |
| YMR022W | QRI8 | 0.5834321 | 1 | -0.2124766 | 103.4877954 | 196.4188818 |
| YJR133W | XPT1 | 0.67029609 | 1 | -0.2123262 | 105.7799009 | 200.1358502 |
| YLR357W | RSC2 | 0.63703077 | 1 | -0.2118574 | 74.43074164 | 148.9694401 |
| YNL008C | ASI3 | 0.6945368 | 1 | -0.2116095 | 113.7548404 | 213.0430738 |
| YNL271C | BNI1 | 0.64169642 | 1 | -0.211564 | 89.51898724 | 173.5279702 |
| YER004W | FMP52 | 0.61229384 | 1 | -0.2115493 | 115.6421599 | 216.1119302 |
| YER033C | ZRG8 | 0.63265407 | 1 | -0.2114824 | 121.3238052 | 225.3653922 |
| YLR416C | YLR416C | 0.59880469 | 1 | -0.2109907 | 107.9211441 | 203.452305 |
| YGL127C | SOH1 | 0.61839708 | 1 | -0.2108891 | 81.15743356 | 159.8089738 |
| YFR035C | YFR035C | 0.54673426 | 1 | -0.2106448 | 124.3054041 | 230.1167257 |
| YMR215W | GAS3 | 0.66353153 | 1 | -0.2102663 | 111.5437099 | 209.2633057 |
| YLR421C | RPN13 | 0.67207574 | 1 | -0.2100387 | 118.6064676 | 220.7472994 |
| YFL046W | FMP32 | 0.63443931 | 1 | -0.2098461 | 120.5456382 | 223.8834047 |

| | | | | | | |
|---|---|---|---|---|---|---|
| YMR086C-A | YMR086C-A | 0.64122522 | 1 | -0.2098406 | 112.720202 | 211.1256888 |
| YLR318W | EST2 | 0.54432149 | 1 | -0.20976 | 7.223148842 | 39.1343737 |
| YFR041C | ERJ5 | 0.57674003 | 1 | -0.2096966 | 126.9267374 | 234.2663362 |
| YLR264W | RPS28B | 0.67340588 | 1 | -0.2095862 | 87.84842411 | 170.5466487 |
| YER155C | BEM2 | 0.59412105 | 1 | -0.2092185 | 114.9152281 | 214.6228789 |
| YNL023C | FAP1 | 0.5760431 | 1 | -0.2090875 | 95.63626248 | 183.1773029 |
| YMR052C-A | YMR052C-A | 0.59884812 | 1 | -0.2089971 | 108.3739031 | 203.9303565 |
| YMR237W | BCH1 | 0.59510303 | 1 | -0.2088206 | 106.7360975 | 201.2373915 |
| YPL068C | YPL068C | 0.71385293 | 1 | -0.2085856 | 117.024686 | 217.979147 |
| YKR076W | ECM4 | 0.64490178 | 1 | -0.2084941 | 122.834611 | 227.438538 |
| YLL016W | SDC25 | 0.71226837 | 1 | -0.2084176 | 122.69707 | 227.2043359 |
| YML066C | SMA2 | 0.59653648 | 1 | -0.208351 | 98.35839721 | 187.5188559 |
| YJR119C | JHD2 | 0.68653018 | 1 | -0.2081938 | 111.8462476 | 209.4861877 |
| YER051W | JHD1 | 0.61298549 | 1 | -0.2081841 | 120.3591647 | 223.3626392 |
| YLR299W | ECM38 | 0.5733041 | 1 | -0.207943 | 108.7478227 | 204.4024278 |
| YNL044W | YIP3 | 0.68637243 | 1 | -0.2076677 | 109.9989566 | 206.4061129 |
| YNR071C | YNR071C | 0.74168562 | 1 | -0.2076109 | 126.8040249 | 233.794259 |
| YML052W | SUR7 | 0.59359303 | 1 | -0.2073386 | 102.7410762 | 194.5314279 |
| YOR019W | YOR019W | 0.74344269 | 1 | -0.2072956 | 128.8631427 | 237.109891 |
| YOR192C | THI72 | 0.75148187 | 1 | -0.2071468 | 130.4637104 | 239.6997231 |
| YLR414C | YLR414C | 0.63629346 | 1 | -0.2069054 | 79.77249186 | 157.0316445 |
| YEL064C | AVT2 | 0.61649291 | 1 | -0.2068226 | 117.4525704 | 218.4467403 |
| YKL075C | YKL075C | 0.68017977 | 1 | -0.2067982 | 103.4772291 | 195.6610238 |
| YNL101W | AVT4 | 0.67707173 | 1 | -0.2065348 | 127.0484928 | 234.0524304 |
| YOL037C | YOL037C | 0.73030351 | 1 | -0.2062734 | 123.8890727 | 228.8678672 |
| YEL020C | YEL020C | 0.60057062 | 1 | -0.2060327 | 111.9311144 | 209.3426627 |
| YER066C-A | YER066C-A | 0.55831587 | 1 | -0.2055461 | 108.2252405 | 203.2378929 |
| YMR107W | SPG4 | 0.65254309 | 1 | -0.2053846 | 108.9205316 | 204.3502881 |
| YFL021W | GAT1 | 0.62969809 | 1 | -0.2052779 | 121.916919 | 225.5230264 |
| YNL330C | RPD3 | 0.67115457 | 1 | -0.20509 | 120.8716705 | 223.7945586 |
| YOL075C | YOL075C | 0.74094255 | 1 | -0.2044897 | 126.5720633 | 233.0090069 |
| YMR304C-A | YMR304C-A | 0.69062672 | 1 | -0.2043985 | 109.2823808 | 204.8115493 |
| YGL140C | YGL140C | 0.56014722 | 1 | -0.2043328 | 129.4969515 | 237.7566885 |
| YKR091W | SRL3 | 0.65837743 | 1 | -0.2042232 | 122.9263195 | 227.0309806 |
| YGR292W | MAL12 | 0.74171818 | 1 | -0.2037591 | 127.0558079 | 233.7023153 |
| YML120C | NDI1 | 0.62873025 | 1 | -0.2037547 | 112.4225324 | 209.8466384 |
| YOR097C | YOR097C | 0.68396416 | 1 | -0.2034611 | 118.3397228 | 219.4545212 |
| YOR355W | GDS1 | 0.73429196 | 1 | -0.2033513 | 118.880341 | 220.3215235 |
| YLR374C | YLR374C | 0.65029857 | 1 | -0.202958 | 84.57544086 | 164.3465252 |
| YKL102C | YKL102C | 0.63452366 | 1 | -0.2028828 | 114.2431409 | 212.7008635 |
| YOR162C | YRR1 | 0.72719857 | 1 | -0.2028076 | 121.2396919 | 224.0968055 |
| YGL243W | TAD1 | 0.60289052 | 1 | -0.2027048 | 123.9812352 | 228.5526502 |
| YOL015W | IRC10 | 0.71776086 | 1 | -0.2027037 | 129.1363205 | 236.9563002 |
| YMR129W | POM152 | 0.59318877 | 1 | -0.2026903 | 104.162002 | 196.2415349 |
| YMR246W | FAA4 | 0.6528084 | 1 | -0.2026238 | 110.0210104 | 205.7841897 |
| YNL092W | YNL092W | 0.69427736 | 1 | -0.2025788 | 133.7734154 | 244.4993785 |
| YLR393W | ATP10 | 0.65389656 | 1 | -0.2023461 | 67.27886554 | 136.0699218 |
| YKL147C | YKL147C | 0.66559577 | 1 | -0.2021851 | 125.0754266 | 230.2686127 |
| YER114C | BOI2 | 0.6300669 | 1 | -0.2019272 | 114.1173291 | 212.3711244 |
| YKR089C | TGL4 | 0.65414937 | 1 | -0.2016019 | 117.1378714 | 217.2527754 |
| YFR018C | YFR018C | 0.5668803 | 1 | -0.2011745 | 127.1619796 | 233.5382793 |
| YNL128W | TEP1 | 0.70578646 | 1 | -0.2009923 | 113.2436519 | 210.8249317 |
| YPL183C | YPL183C | 0.69088578 | 1 | -0.2006764 | 91.79633162 | 175.8204014 |
| YEL017W | GTT3 | 0.59625253 | 1 | -0.2006573 | 119.0232314 | 220.2030749 |
| YNL040W | YNL040W | 0.68174735 | 1 | -0.200649 | 103.1760495 | 194.3679856 |
| YOR285W | YOR285W | 0.74491345 | 1 | -0.2005387 | 123.1419562 | 226.9019379 |
| YDR505C | PSP1 | 0.57743838 | 1 | -0.2001001 | 109.9084183 | 205.2714723 |
| YLR429W | CRN1 | 0.62453623 | 1 | -0.1999473 | 103.5527206 | 194.8905109 |
| YLR188W | MDL1 | 0.60523829 | 1 | -0.1999273 | 105.6497437 | 198.3064664 |
| YMR167W | MLH1 | 0.58914439 | 1 | -0.1999225 | 101.5529543 | 191.6272742 |
| YNL300W | YNL300W | 0.7010773 | 1 | -0.1996303 | 100.748092 | 190.2770698 |
| YNL067W | RPL9B | 0.66184021 | 1 | -0.1995489 | 94.61237059 | 180.2640336 |
| YER178W | PDA1 | 0.66210629 | 1 | -0.1995433 | 111.8930236 | 208.4341443 |
| YJR129C | YJR129C | 0.6738307 | 1 | -0.199252 | 102.0216215 | 192.3038356 |

| | | | | | | |
|---|---|---|---|---|---|---|
| YKL140W | TGL1 | 0.63138729 | 1 | -0.198895 | 112.3670183 | 209.122295 |
| YEL004W | YEA4 | 0.68300396 | 1 | -0.1987243 | 122.6917259 | 225.931319 |
| YKR087C | OMA1 | 0.66347604 | 1 | -0.1984117 | 124.451739 | 228.7597094 |
| YFR024C | YFR024C | 0.73831478 | 1 | -0.1983839 | 80.30140001 | 156.7824054 |
| YNL091W | NST1 | 0.65383573 | 1 | -0.1982757 | 54.17280942 | 114.1735788 |
| YGL004C | RPN14 | 0.5597343 | 1 | -0.1980592 | 125.1944423 | 229.9244883 |
| YNL270C | ALP1 | 0.68385447 | 1 | -0.1979582 | 127.3506225 | 233.4263049 |
| YMR174C | PAI3 | 0.56238303 | 1 | -0.1979317 | 90.78020736 | 173.8059193 |
| YER005W | YND1 | 0.66919399 | 1 | -0.1971306 | 120.5398861 | 222.2155215 |
| YMR259C | YMR259C | 0.67580781 | 1 | -0.1967109 | 122.5997661 | 225.5187898 |
| YMR039C | SUB1 | 0.60647195 | 1 | -0.1967098 | 101.8740782 | 191.7317225 |
| YNL072W | RNH201 | 0.68548201 | 1 | -0.1963036 | 126.750385 | 232.2319871 |
| YEL017C-A | PMP2 | 0.6552589 | 1 | -0.1962589 | 121.0272978 | 222.8964111 |
| YKR066C | CCP1 | 0.66964738 | 1 | -0.1960328 | 121.1728114 | 223.1041355 |
| YGL108C | YGL108C | 0.57060543 | 1 | -0.1958892 | 130.0322679 | 237.5280506 |
| YKL085W | MDH1 | 0.69968616 | 1 | -0.1957309 | 110.0464907 | 204.9266848 |
| YNL071W | LAT1 | 0.66579112 | 1 | -0.1954922 | 114.9667947 | 212.9166078 |
| YDL013W | HEX3 | 0.74346321 | 1 | -0.1952327 | 73.7844794 | 145.7475351 |
| YMR273C | ZDS1 | 0.68718784 | 1 | -0.1950871 | 124.7525775 | 228.8165074 |
| YNR032C-A | HUB1 | 0.74522724 | 1 | -0.1950039 | 122.8390439 | 225.6862127 |
| YKL081W | TEF4 | 0.67173689 | 1 | -0.1946621 | 91.43021575 | 174.4391112 |
| YNL323W | LEM3 | 0.65087994 | 1 | -0.1943951 | 116.3667664 | 215.055735 |
| YLL040C | VPS13 | 0.64221284 | 1 | -0.1942735 | 109.3722207 | 203.6373968 |
| YNR014W | YNR014W | 0.72416247 | 1 | -0.1941769 | 116.1065655 | 214.6030891 |
| YOR052C | YOR052C | 0.74820191 | 1 | -0.1940799 | 120.2468693 | 221.3399412 |
| YNL085W | MKT1 | 0.66633032 | 1 | -0.1938944 | 115.5607618 | 213.6764835 |
| YNL116W | DMA2 | 0.71574392 | 1 | -0.1937356 | 114.1853208 | 211.4135294 |
| YGR138C | TPO2 | 0.61623117 | 1 | -0.1937319 | 133.0813669 | 242.2172936 |
| YFR015C | GSY1 | 0.55910916 | 1 | -0.1936021 | 125.7750863 | 230.2897007 |
| YGL212W | VAM7 | 0.59283276 | 1 | -0.193044 | 30.3960829 | 74.73049039 |
| YER038W-A | FMP49 | 0.5584865 | 1 | -0.192982 | 109.8909564 | 204.3145885 |
| YKR003W | OSH6 | 0.70455158 | 1 | -0.1928993 | 109.6150461 | 203.8540091 |
| YFR014C | CMK1 | 0.63379306 | 1 | -0.1926656 | 139.9592198 | 253.2904607 |
| YNR063W | YNR063W | 0.76623996 | 1 | -0.1924353 | 132.0034095 | 240.2908986 |
| YKR050W | TRK2 | 0.65703353 | 1 | -0.1924001 | 119.3895082 | 219.7231811 |
| YLR443W | ECM7 | 0.63216687 | 1 | -0.1923975 | 100.9786361 | 189.7095242 |
| YOR304W | ISW2 | 0.73432915 | 1 | -0.1921121 | 113.8047646 | 210.5813939 |
| YMR207C | HFA1 | 0.686156 | 1 | -0.1920366 | 89.23256361 | 170.5140551 |
| YKL043W | PHD1 | 0.65430435 | 1 | -0.1918662 | 112.5155616 | 208.4476673 |
| YML109W | ZDS2 | 0.6506849 | 1 | -0.1917454 | 112.591442 | 208.5556103 |
| YKL120W | OAC1 | 0.64687179 | 1 | -0.1914999 | 117.9669248 | 217.2866768 |
| YPL139C | UME1 | 0.7572885 | 1 | -0.1913913 | 127.6021695 | 232.9798456 |
| YNL119W | NCS2 | 0.62723553 | 1 | -0.191327 | 81.50941717 | 157.831269 |
| YMR280C | CAT8 | 0.63478123 | 1 | -0.1912672 | 112.4163155 | 208.2077521 |
| YNR060W | FRE4 | 0.75556539 | 1 | -0.1911998 | 127.4811466 | 232.7575821 |
| YOR003W | YSP3 | 0.76332967 | 1 | -0.1911894 | 129.1493953 | 235.4757979 |
| YOR108W | LEU9 | 0.75229763 | 1 | -0.1911596 | 118.9078989 | 218.7762683 |
| YOR185C | GSP2 | 0.72034842 | 1 | -0.1909977 | 119.0312197 | 218.9561823 |
| YOL036W | YOL036W | 0.7469189 | 1 | -0.1909973 | 122.4851612 | 224.5867356 |
| YMR126C | DLT1 | 0.58425247 | 1 | -0.1907353 | 91.7966875 | 174.5243464 |
| YGR007W | MUQ1 | 0.54071566 | 1 | -0.1906972 | 117.1440344 | 215.840507 |
| YGR003W | CUL3 | 0.55500766 | 1 | -0.1906305 | 127.2047551 | 232.2327563 |
| YKL191W | DPH2 | 0.70760821 | 1 | -0.1904966 | 109.1665325 | 202.8094654 |
| YMR009W | ADI1 | 0.67734986 | 1 | -0.1904109 | 94.37228954 | 178.6807748 |
| YOL117W | RRI2 | 0.73508719 | 1 | -0.1903188 | 112.6599276 | 208.4811816 |
| YER132C | PMD1 | 0.61857314 | 1 | -0.189948 | 115.3062003 | 212.7467669 |
| YNL192W | CHS1 | 0.66695303 | 1 | -0.1897383 | 110.197906 | 204.3918975 |
| YDR519W | FPR2 | 0.6430563 | 1 | -0.1896773 | 118.3759175 | 217.7156929 |
| YKL185W | ASH1 | 0.66632565 | 1 | -0.1896436 | 116.0237605 | 213.8768199 |
| YNL142W | MEP2 | 0.70784451 | 1 | -0.1893532 | 105.8554202 | 197.2625581 |
| YNL328C | MDJ2 | 0.73136294 | 1 | -0.1893511 | 116.3064367 | 214.2994933 |
| YKR055W | RHO4 | 0.64943689 | 1 | -0.1893033 | 98.9117689 | 185.9365424 |
| YBR132C | AGP2 | 0.39100222 | 1 | -0.1891069 | 0 | 24.66542803 |
| YGR033C | TIM21 | 0.6009485 | 1 | -0.1890301 | 130.7167505 | 237.7492493 |

| | | | | | | |
|---|---|---|---|---|---|---|
| YOL068C | HST1 | 0.74726001 | 1 | -0.1886892 | 111.5803103 | 206.5086491 |
| YML038C | YMD8 | 0.66147801 | 1 | -0.1885936 | 81.00799152 | 156.65732 |
| YEL048C | YEL048C | 0.65553525 | 1 | -0.1884966 | 117.8387645 | 216.6860301 |
| YMR031W-A | YMR031W-A | 0.68625001 | 1 | -0.1879027 | 85.73862124 | 164.2790585 |
| YKL190W | CNB1 | 0.65451976 | 1 | -0.1875783 | 114.078354 | 210.4360467 |
| YNL289W | PCL1 | 0.75677588 | 1 | -0.1872416 | 128.343507 | 233.6471243 |
| YOR043W | WHI2 | 0.71678977 | 1 | -0.1872288 | 123.2697015 | 225.3741588 |
| YOL151W | GRE2 | 0.73737044 | 1 | -0.1867163 | 113.1900589 | 208.8755188 |
| YEL057C | YEL057C | 0.64323221 | 1 | -0.1866583 | 26.51404118 | 67.56911803 |
| YMR244C-A | YMR244C-A | 0.57306463 | 1 | -0.186436 | 96.71067226 | 181.9743443 |
| YKL174C | TPO5 | 0.68422982 | 1 | -0.1859568 | 121.2785223 | 221.9622402 |
| YPL101W | ELP4 | 0.69695185 | 1 | -0.1859144 | 92.6836952 | 175.3415576 |
| YPL008W | CHL1 | 0.71647526 | 1 | -0.1858903 | 105.2169854 | 195.7701169 |
| YMR264W | CUE1 | 0.70187523 | 1 | -0.1857818 | 95.24306347 | 179.4965263 |
| YOR051C | YOR051C | 0.7491061 | 1 | -0.1855712 | 120.6621781 | 220.9071885 |
| YNL187W | YNL187W | 0.68475614 | 1 | -0.1853681 | 117.3607988 | 215.498792 |
| YKL097C | YKL097C | 0.6618653 | 1 | -0.1853096 | 112.3865761 | 207.3822125 |
| YPL098C | MGR2 | 0.71922193 | 1 | -0.1844295 | 106.2131438 | 197.2035217 |
| YOL017W | ESC8 | 0.76318777 | 1 | -0.1843583 | 125.2181923 | 228.1761774 |
| YLR065C | YLR065C | 0.69802857 | 1 | -0.1842957 | 84.37800306 | 161.5905196 |
| YDR426C | YDR426C | 0.61181013 | 1 | -0.1840978 | 117.6071074 | 215.7346482 |
| YKL017C | HCS1 | 0.7184665 | 1 | -0.1839567 | 111.2824005 | 205.405735 |
| YOR024W | YOR024W | 0.76901824 | 1 | -0.183827 | 128.144088 | 232.8766609 |
| YML016C | PPZ1 | 0.74783957 | 1 | -0.1836757 | 102.4805257 | 191.0202991 |
| YOL064C | MET22 | 0.6795805 | 1 | -0.1834079 | 94.1880554 | 177.4670278 |
| YFL031W | HAC1 | 0.5845909 | 1 | -0.1831347 | 107.4999785 | 199.1324257 |
| YML018C | YML018C | 0.62942465 | 1 | -0.1828422 | 102.9155072 | 191.6206993 |
| YPL102C | YPL102C | 0.69767392 | 1 | -0.1828184 | 95.65936001 | 179.7886572 |
| YGL251C | HFM1 | 0.60669968 | 1 | -0.1827464 | 128.6426383 | 233.5484552 |
| YOL087C | YOL087C | 0.69776981 | 1 | -0.1824891 | 116.9139608 | 214.3948598 |
| YER083C | GET2 | 0.65448909 | 1 | -0.1821817 | 77.58108878 | 150.2344908 |
| YNL239W | LAP3 | 0.70982433 | 1 | -0.1819949 | 132.8891891 | 240.3731329 |
| YML008C | ERG6 | 0.56173307 | 1 | -0.1819529 | 88.42704916 | 167.8856885 |
| YDR488C | PAC11 | 0.68373603 | 1 | -0.1819082 | 120.0091317 | 219.364816 |
| YGL083W | SCY1 | 0.58728624 | 1 | -0.181716 | 132.4380843 | 239.6013701 |
| YGR044C | RME1 | 0.56735679 | 1 | -0.1815402 | 126.7552373 | 230.3142969 |
| YNL275W | BOR1 | 0.73259721 | 1 | -0.1814228 | 114.0911508 | 209.6540459 |
| YLR454W | FMP27 | 0.6252068 | 1 | -0.1813324 | 97.01515886 | 181.805047 |
| YGR037C | ACB1 | 0.63482688 | 1 | -0.1812643 | 131.672837 | 238.2949507 |
| YDR428C | YDR428C | 0.62468148 | 1 | -0.1812136 | 118.9469229 | 217.542619 |
| YGR136W | LSB1 | 0.57945696 | 1 | -0.1811393 | 124.6860216 | 226.8887777 |
| YOL007C | CSI2 | 0.75437322 | 1 | -0.1811154 | 118.1373484 | 216.210038 |
| YOR071C | THI71 | 0.72119182 | 1 | -0.1810906 | 119.8287177 | 218.964071 |
| YNL031C | HHT2 | 0.72835869 | 1 | -0.1809714 | 111.3754986 | 205.1681287 |
| YLR099C | ICT1 | 0.66675297 | 1 | -0.1808752 | 95.53395036 | 179.3307621 |
| YFR054C | YFR054C | 0.62995473 | 1 | -0.1805069 | 130.946181 | 237.0115768 |
| YLR168C | YLR168C | 0.71501294 | 1 | -0.1804197 | 89.3412543 | 169.1760363 |
| YNL021W | HDA1 | 0.61754866 | 1 | -0.1802768 | 94.63855807 | 177.7930487 |
| YKL205W | LOS1 | 0.65323802 | 1 | -0.1800652 | 85.18949101 | 162.361623 |
| YHR103W | SBE22 | 0.79195466 | 1 | -0.1798898 | 115.9022359 | 212.4065127 |
| YKL116C | PRR1 | 0.72824434 | 1 | -0.1798534 | 112.3446658 | 206.6022285 |
| YNL145W | MFA2 | 0.67109675 | 1 | -0.1798436 | 113.532211 | 208.5368846 |
| YML006C | GIS4 | 0.60914077 | 1 | -0.1796618 | 88.53919934 | 167.7696808 |
| YNL307C | MCK1 | 0.64975453 | 1 | -0.1793511 | 84.24465418 | 160.728206 |
| YKL216W | URA1 | 0.64953891 | 1 | -0.1787245 | 101.6048839 | 188.9470438 |
| YDR435C | PPM1 | 0.71637619 | 1 | -0.1782546 | 87.14446482 | 165.3124436 |
| YPL186C | UIP4 | 0.77445377 | 1 | -0.178181 | 128.8741663 | 233.3304191 |
| YPL110C | GDE1 | 0.77397752 | 1 | -0.1780411 | 126.8591385 | 230.0272755 |
| YEL011W | GLC3 | 0.67176641 | 1 | -0.1780086 | 118.8395809 | 216.9495963 |
| YNL004W | HRB1 | 0.69064857 | 1 | -0.1776399 | 116.5213231 | 213.122289 |
| YJR106W | ECM27 | 0.71112483 | 1 | -0.1775915 | 124.6461947 | 226.361102 |
| YGL209W | MIG2 | 0.59248405 | 1 | -0.1775317 | 110.4301363 | 203.1783539 |
| YPL056C | YPL056C | 0.78101851 | 1 | -0.1775135 | 129.2106063 | 233.7918136 |
| YKL218C | SRY1 | 0.69468535 | 1 | -0.1775005 | 116.0359374 | 212.3128309 |

| | | | | | | |
|---|---|---|---|---|---|---|
| YOR137C | SIA1 | 0.75181793 | 1 | -0.1768724 | 136.3776367 | 245.3918566 |
| YMR158C-B | YMR158C-B | 0.64247766 | 1 | -0.1768681 | 102.130566 | 189.5618838 |
| YOR064C | YNG1 | 0.75101478 | 1 | -0.1763693 | 131.1884061 | 236.866781 |
| YFL041W | FET5 | 0.7980809 | 1 | -0.1761563 | 119.2948875 | 217.4502366 |
| YKL062W | MSN4 | 0.6845502 | 1 | -0.1760229 | 116.4393554 | 212.7777657 |
| YGR143W | SKN1 | 0.67010142 | 1 | -0.1759433 | 129.1221739 | 233.4428501 |
| YGL242C | YGL242C | 0.63815583 | 1 | -0.1759379 | 132.4995535 | 238.9479352 |
| YDR453C | TSA2 | 0.65941097 | 1 | -0.175923 | 124.4857716 | 225.8819578 |
| YKR078W | YKR078W | 0.69417258 | 1 | -0.1758185 | 120.4048957 | 219.2157122 |
| YGL256W | ADH4 | 0.53285432 | 1 | -0.1757777 | 121.3823476 | 220.8038246 |
| YNR024W | YNR024W | 0.74020301 | 1 | -0.1754927 | 129.1332495 | 233.4021401 |
| YGL260W | YGL260W | 0.62286105 | 1 | -0.175421 | 124.4756714 | 225.8000242 |
| YOL053W | YOL053W | 0.77030528 | 1 | -0.1751589 | 124.9917826 | 226.6071895 |
| YJR146W | YJR146W | 0.69286045 | 1 | -0.1751587 | 124.5754749 | 225.9285114 |
| YLR388W | RPS29A | 0.59502067 | 1 | -0.1743436 | 81.50968861 | 155.6165402 |
| YKR100C | SKG1 | 0.6932501 | 1 | -0.1743061 | 123.4118269 | 223.9203258 |
| YKL106W | AAT1 | 0.74721687 | 1 | -0.1741216 | 116.5673846 | 212.7384894 |
| YMR319C | FET4 | 0.65891744 | 1 | -0.1740805 | 109.3141788 | 200.9089787 |
| YOL107W | YOL107W | 0.76769785 | 1 | -0.1739098 | 121.04409 | 220.0087686 |
| YMR048W | CSM3 | 0.65593934 | 1 | -0.1738456 | 106.5475236 | 196.3681488 |
| YOR079C | ATX2 | 0.77533812 | 1 | -0.1735115 | 123.5592124 | 224.0569505 |
| YMR139W | RIM11 | 0.67912736 | 1 | -0.173397 | 81.61013266 | 155.6568282 |
| YGR059W | SPR3 | 0.60106265 | 1 | -0.1729416 | 120.5692062 | 219.1083303 |
| YJR117W | STE24 | 0.75943914 | 1 | -0.1728013 | 120.1629358 | 218.4277282 |
| YOR100C | CRC1 | 0.74800355 | 1 | -0.1723997 | 111.7463651 | 204.6546927 |
| YDR517W | GRH1 | 0.67905492 | 1 | -0.1721165 | 116.8432522 | 212.9266696 |
| YDR512C | EMI1 | 0.70469111 | 1 | -0.172038 | 86.37148131 | 163.2414937 |
| YGR146C | YGR146C | 0.60590162 | 1 | -0.1719841 | 122.10271 | 221.4833449 |
| YER039C-A | YER039C-A | 0.68903206 | 1 | -0.1719101 | 119.2606137 | 216.8405213 |
| YGR088W | CTT1 | 0.61346164 | 1 | -0.1717908 | 122.3147637 | 221.8038332 |
| YOR099W | KTR1 | 0.77904897 | 1 | -0.1716911 | 125.3581871 | 226.7521914 |
| YMR201C | RAD14 | 0.66647216 | 1 | -0.1712545 | 105.0970369 | 193.6656169 |
| YOL084W | PHM7 | 0.75217762 | 1 | -0.1706867 | 130.0088921 | 234.2027524 |
| YMR318C | ADH6 | 0.69764201 | 1 | -0.1704248 | 124.0084744 | 224.3867314 |
| YML118W | NGL3 | 0.69854575 | 1 | -0.1703783 | 110.8888587 | 202.9931409 |
| YOR087W | YVC1 | 0.77324449 | 1 | -0.1700917 | 120.7882883 | 219.0937632 |
| YKL010C | UFD4 | 0.68023249 | 1 | -0.1699063 | 115.1747545 | 209.9184221 |
| YMR136W | GAT2 | 0.65439974 | 1 | -0.169891 | 103.428879 | 190.7683555 |
| YOL129W | VPS68 | 0.7790196 | 1 | -0.1693903 | 122.4682325 | 221.7409101 |
| YGR123C | PPT1 | 0.62685341 | 1 | -0.1692495 | 130.6376421 | 235.04028 |
| YPL017C | IRC15 | 0.77695659 | 1 | -0.1690899 | 114.7879367 | 209.1813457 |
| YGR139W | YGR139W | 0.63723542 | 1 | -0.16904 | 126.845529 | 228.8310704 |
| YKL217W | JEN1 | 0.72218061 | 1 | -0.1687674 | 102.3385799 | 188.8444001 |
| YLL007C | YLL007C | 0.69125575 | 1 | -0.1687534 | 116.3773929 | 211.728579 |
| YLR410W | VIP1 | 0.72121641 | 1 | -0.1686714 | 84.07488297 | 159.0584862 |
| YER072W | VTC1 | 0.66408265 | 1 | -0.1686447 | 122.6469219 | 221.9349545 |
| YNL274C | YNL274C | 0.71631877 | 1 | -0.1686063 | 123.4609312 | 223.2569466 |
| YGL013C | PDR1 | 0.61325201 | 1 | -0.1685909 | 121.8455155 | 220.6214922 |
| YGL006W | PMC1 | 0.62336874 | 1 | -0.1685029 | 123.4490948 | 223.2241635 |
| YJR145C | RPS4A | 0.68985622 | 1 | -0.1683142 | 118.9641805 | 215.8882645 |
| YLL001W | DNM1 | 0.73940818 | 1 | -0.1682326 | 110.5809009 | 202.2112435 |
| YNL096C | RPS7B | 0.74000135 | 1 | -0.1681766 | 101.3901969 | 187.2212957 |
| YKL107W | YKL107W | 0.70163588 | 1 | -0.1680122 | 116.5985003 | 211.9923548 |
| YPL052W | OAZ1 | 0.78230535 | 1 | -0.1677888 | 124.9388854 | 225.5596776 |
| YFL001W | DEG1 | 0.66800284 | 1 | -0.1675574 | 82.0058526 | 155.5402608 |
| YML021C | UNG1 | 0.70245615 | 1 | -0.1671107 | 82.59457996 | 156.4417333 |
| YLR261C | YLR261C | 0.69222512 | 1 | -0.1670447 | 79.07521005 | 150.6958658 |
| YOR101W | RAS1 | 0.75951891 | 1 | -0.1669978 | 127.8116683 | 230.1397001 |
| YOL070C | NBA1 | 0.77800726 | 1 | -0.1669195 | 120.7081648 | 218.549384 |
| YOL055C | THI20 | 0.77310239 | 1 | -0.1666819 | 122.6399435 | 221.6675711 |
| YLR114C | AVL9 | 0.67990043 | 1 | -0.1666673 | 95.87378118 | 178.0315899 |
| YMR020W | FMS1 | 0.63784818 | 1 | -0.166399 | 99.99404418 | 184.713438 |
| YKR058W | GLG1 | 0.72812478 | 1 | -0.1663925 | 125.1115454 | 225.659021 |
| YGL056C | SDS23 | 0.67607749 | 1 | -0.1663607 | 128.0093646 | 230.3788821 |

| | | | | | | |
|---|---|---|---|---|---|---|
| YPL253C | VIK1 | 0.78397399 | 1 | -0.1659979 | 125.7845624 | 226.7047043 |
| YLR255C | YLR255C | 0.69078395 | 1 | -0.16594 | 93.09667447 | 173.4095036 |
| YLR461W | PAU4 | 0.67635828 | 1 | -0.1656978 | 105.7171027 | 193.9516812 |
| YGL259W | YPS5 | 0.59371393 | 1 | -0.1656246 | 125.8176233 | 226.7099075 |
| YOR301W | RAX1 | 0.78802521 | 1 | -0.1653948 | 124.8471949 | 225.0979494 |
| YKL215C | YKL215C | 0.7636506 | 1 | -0.1653611 | 116.6638691 | 211.7531257 |
| YPR046W | MCM16 | 0.77824614 | 1 | -0.1647194 | 118.3710155 | 214.4524203 |
| YPL141C | YPL141C | 0.79021161 | 1 | -0.164667 | 121.9238635 | 220.2374196 |
| YMR016C | SOK2 | 0.70692667 | 1 | -0.1643491 | 101.589277 | 187.046602 |
| YLR407W | YLR407W | 0.69053798 | 1 | -0.1642241 | 93.84864026 | 174.4115509 |
| YOL101C | IZH4 | 0.7946069 | 1 | -0.164184 | 127.7723362 | 229.7085704 |
| YLR269C | YLR269C | 0.72880949 | 1 | -0.1636496 | 99.3603845 | 183.3218434 |
| YKL115C | YKL115C | 0.67487454 | 1 | -0.163346 | 109.0072337 | 199.0084882 |
| YML034W | SRC1 | 0.65381644 | 1 | -0.1629435 | 99.97429775 | 184.2305334 |
| YKL031W | YKL031W | 0.71167405 | 1 | -0.1629173 | 118.9401355 | 215.1451472 |
| YOL163W | YOL163W | 0.75919958 | 1 | -0.1625804 | 125.3523984 | 225.5544363 |
| YML131W | YML131W | 0.6990331 | 1 | -0.1620443 | 112.9336463 | 205.2395249 |
| YML048W | GSF2 | 0.69453326 | 1 | -0.1619196 | 97.44899555 | 179.9802607 |
| YKL220C | FRE2 | 0.72346423 | 1 | -0.1618236 | 114.5076423 | 207.776665 |
| YER016W | BIM1 | 0.66708217 | 1 | -0.1617804 | 115.9929057 | 210.1923 |
| YER101C | AST2 | 0.71770073 | 1 | -0.16144 | 113.6572417 | 206.3403027 |
| YOR009W | TIR4 | 0.79241229 | 1 | -0.1611284 | 127.4654355 | 228.8097226 |
| YNL219C | ALG9 | 0.72965908 | 1 | -0.161085 | 100.0954704 | 184.1856743 |
| YER024W | YAT2 | 0.71691863 | 1 | -0.1608743 | 129.6785727 | 232.3844275 |
| YKL199C | YKT9 | 0.72180052 | 1 | -0.160544 | 123.6843815 | 222.5696332 |
| YFR043C | IRC6 | 0.66043861 | 1 | -0.1601461 | 126.6746827 | 227.3925217 |
| YDL002C | NHP10 | 0.83716963 | 1 | -0.159519 | 45.92715816 | 95.67647629 |
| YMR306C-A | YMR306C-A | 0.76715559 | 1 | -0.1594985 | 114.5307373 | 207.5110412 |
| YGR109C | CLB6 | 0.64966115 | 1 | -0.1594052 | 128.6570627 | 230.5275484 |
| YOR216C | RUD3 | 0.78271451 | 1 | -0.1592395 | 118.7241593 | 214.3133642 |
| YOR233W | KIN4 | 0.79767792 | 1 | -0.1591902 | 123.3846995 | 221.9045214 |
| YNL056W | OCA2 | 0.72891077 | 1 | -0.1591078 | 99.2590113 | 182.5641922 |
| YKR077W | YKR077W | 0.74752262 | 1 | -0.1590963 | 106.8319862 | 194.9081154 |
| YGL090W | LIF1 | 0.6737489 | 1 | -0.1589113 | 126.7019703 | 227.2759418 |
| YKL048C | ELM1 | 0.72136829 | 1 | -0.1588874 | 92.49871718 | 171.5148444 |
| YDR440W | DOT1 | 0.67390264 | 1 | -0.15864 | 107.3119883 | 195.6310947 |
| YGL205W | POX1 | 0.64110883 | 1 | -0.1585286 | 119.1177799 | 214.8623185 |
| YER170W | ADK2 | 0.68650441 | 1 | -0.158088 | 120.9863295 | 217.8509495 |
| YNL123W | NMA111 | 0.73156049 | 1 | -0.1580064 | 110.3797374 | 200.549492 |
| YOR127W | RGA1 | 0.75757691 | 1 | -0.1579134 | 122.1512507 | 219.727229 |
| YLL032C | YLL032C | 0.68572587 | 1 | -0.1578238 | 121.7744231 | 219.1012296 |
| YKL090W | CUE2 | 0.76343013 | 1 | -0.1577287 | 114.8436368 | 207.7903005 |
| YMR018W | YMR018W | 0.66545443 | 1 | -0.1576618 | 100.2952277 | 184.0648261 |
| YOL105C | WSC3 | 0.78774798 | 1 | -0.1573305 | 118.111715 | 213.0659634 |
| YPL009C | YPL009C | 0.79242767 | 1 | -0.1572776 | 121.8720902 | 219.1892036 |
| YGL051W | MST27 | 0.66886051 | 1 | -0.1572053 | 134.5664456 | 239.8740543 |
| YMR166C | YMR166C | 0.77526113 | 1 | -0.1571302 | 50.93454224 | 103.5279235 |
| YKL063C | YKL063C | 0.7013356 | 1 | -0.1568441 | 118.9638754 | 214.3917115 |
| YLR185W | RPL37A | 0.62076961 | 1 | -0.1568091 | 64.71410693 | 125.9494238 |
| YOR001W | RRP6 | 0.76120966 | 1 | -0.1567847 | 105.3358383 | 192.1676017 |
| YOR091W | TMA46 | 0.7825048 | 1 | -0.156723 | 117.3451357 | 211.7370482 |
| YOR318C | YOR318C | 0.79838327 | 1 | -0.156536 | 127.1501337 | 227.6967265 |
| YGR019W | UGA1 | 0.67637208 | 1 | -0.1564872 | 137.4124574 | 244.4199504 |
| YER164W | CHD1 | 0.69561277 | 1 | -0.1558572 | 120.3060988 | 216.4510795 |
| YOL121C | RPS19A | 0.79177341 | 1 | -0.1556423 | 120.82606 | 217.2706761 |
| YML121W | GTR1 | 0.75859279 | 1 | -0.1555403 | 109.9634308 | 199.5491747 |
| YPR095C | SYT1 | 0.80401062 | 1 | -0.1552857 | 129.9253857 | 232.0578509 |
| YER180C | ISC10 | 0.66206883 | 1 | -0.1551849 | 111.2717785 | 201.6356796 |
| YKL029C | MAE1 | 0.7718593 | 1 | -0.1551528 | 117.0337627 | 211.0246463 |
| YNL204C | SPS18 | 0.71839049 | 1 | -0.155136 | 114.5061127 | 206.9018976 |
| YOL027C | MDM38 | 0.76289115 | 1 | -0.1551248 | 126.2143255 | 225.987112 |
| YNL179C | SRF6 | 0.74003575 | 1 | -0.1545427 | 123.631843 | 221.7012334 |
| YKL039W | PTM1 | 0.73638704 | 1 | -0.1542841 | 124.2961733 | 222.7504946 |
| YKL068W | NUP100 | 0.7557277 | 1 | -0.1540747 | 107.0689748 | 194.6394761 |

| | | | | | | |
|---|---|---|---|---|---|---|
| YNR049C | MSO1 | 0.74762943 | 1 | -0.1540287 | 113.8517599 | 205.6907423 |
| YLL020C | YLL020C | 0.72154887 | 1 | -0.1540255 | 121.3166109 | 217.859501 |
| YFR056C | YFR056C | 0.65489641 | 1 | -0.1539685 | 127.3868568 | 227.7477481 |
| YKL176C | LST4 | 0.69006619 | 1 | -0.1538955 | 106.4130712 | 193.5468601 |
| YLR338W | YLR338W | 0.67708609 | 1 | -0.153855 | 70.94401642 | 135.7200875 |
| YLR449W | FPR4 | 0.68875881 | 1 | -0.1537688 | 93.0063476 | 171.6747526 |
| YNL066W | SUN4 | 0.74571793 | 1 | -0.1532752 | 101.735744 | 185.8409952 |
| YPL232W | SSO1 | 0.77634632 | 1 | -0.1532661 | 124.5940219 | 223.1032604 |
| YGR105W | VMA21 | 0.78346132 | 1 | -0.1530611 | 56.40054673 | 111.9078371 |
| YGL132W | YGL132W | 0.65497803 | 1 | -0.1530479 | 116.775782 | 210.3295469 |
| YFL036W | RPO41 | 0.57988275 | 1 | -0.1529147 | 85.52838502 | 159.3728144 |
| YGR050C | YGR050C | 0.73064275 | 1 | -0.1526008 | 142.1971223 | 251.7129881 |
| YNL050C | YNL050C | 0.75435856 | 1 | -0.1525203 | 106.1203224 | 192.8902514 |
| YGL248W | PDE1 | 0.65960848 | 1 | -0.1522676 | 132.2245906 | 235.412346 |
| YOR131C | YOR131C | 0.80698631 | 1 | -0.1519236 | 129.8680528 | 231.5258682 |
| YMR258C | YMR258C | 0.73607025 | 1 | -0.1518704 | 120.1237409 | 215.6337917 |
| YPL091W | GLR1 | 0.81564761 | 1 | -0.1514149 | 122.6508991 | 219.6941392 |
| YGR087C | PDC6 | 0.68112943 | 1 | -0.1513994 | 117.7132601 | 211.6428089 |
| YGL263W | COS12 | 0.65083986 | 1 | -0.1513038 | 127.9366278 | 228.2964194 |
| YER045C | ACA1 | 0.73323461 | 1 | -0.1507258 | 122.6423075 | 219.5902496 |
| YOL013W-A | YOL013W-A | 0.76487223 | 1 | -0.1501301 | 117.903979 | 211.7881553 |
| YKR018C | YKR018C | 0.72597659 | 1 | -0.1501265 | 118.2878754 | 212.413512 |
| YGL229C | SAP4 | 0.68326727 | 1 | -0.149956 | 128.9919358 | 229.8409785 |
| YFR048W | RMD8 | 0.6612794 | 1 | -0.1498435 | 129.5279807 | 230.7001735 |
| YLL010C | PSR1 | 0.77404774 | 1 | -0.1496856 | 131.0654642 | 233.1859763 |
| YML071C | COG8 | 0.70880403 | 1 | -0.149667 | 104.9353591 | 190.5863687 |
| YPL221W | FLC1 | 0.79156051 | 1 | -0.1495441 | 131.6881554 | 234.1826205 |
| YPL136W | YPL136W | 0.80494904 | 1 | -0.1494807 | 120.9022171 | 216.5911675 |
| YOR081C | TGL5 | 0.78335543 | 1 | -0.1492563 | 128.4334651 | 228.8393039 |
| YOR196C | LIP5 | 0.73037113 | 1 | -0.1489195 | 50.25413883 | 101.347806 |
| YKR036C | CAF4 | 0.76062727 | 1 | -0.1487502 | 106.1628913 | 192.4679092 |
| YER047C | SAP1 | 0.71157388 | 1 | -0.1486902 | 121.7965208 | 217.9459497 |
| YBR131W | CCZ1 | 0.8264786 | 1 | -0.1486596 | 40.79055254 | 85.88640787 |
| YOL079W | YOL079W | 0.78327116 | 1 | -0.1486032 | 110.7789952 | 199.9738862 |
| YNL013C | YNL013C | 0.70769207 | 1 | -0.1484966 | 106.6609076 | 193.2466955 |
| YER108C | YER108C | 0.68850809 | 1 | -0.1482409 | 115.230892 | 207.1840894 |
| YPL099C | FMP14 | 0.85578458 | 1 | -0.1481055 | 95.86123536 | 175.5901017 |
| YGR052W | FMP48 | 0.68361226 | 1 | -0.1481004 | 113.1241156 | 203.7313161 |
| YMR315W | YMR315W | 0.77671548 | 1 | -0.1479042 | 111.4947464 | 201.0495285 |
| YGL121C | GPG1 | 0.6824477 | 1 | -0.1478984 | 108.5057632 | 196.1761479 |
| YKR060W | UTP30 | 0.71910443 | 1 | -0.1478942 | 110.543286 | 199.497162 |
| YOL018C | TLG2 | 0.76263217 | 1 | -0.1475818 | 101.7650138 | 185.1461155 |
| YOR177C | MPC54 | 0.81009772 | 1 | -0.1470602 | 124.9794965 | 222.9222163 |
| YOR092W | ECM3 | 0.79005157 | 1 | -0.1470539 | 127.5820264 | 227.1640316 |
| YLR445W | YLR445W | 0.70382322 | 1 | -0.1468992 | 96.35417258 | 176.2363553 |
| YMR241W | YHM2 | 0.7199175 | 1 | -0.1468529 | 110.8232041 | 199.817656 |
| YGR129W | SYF2 | 0.67504624 | 1 | -0.1465634 | 121.9125232 | 217.8576634 |
| YOL150C | YOL150C | 0.80718857 | 1 | -0.1465045 | 127.3829892 | 226.7679024 |
| YNL266W | YNL266W | 0.79428849 | 1 | -0.1464792 | 119.5213796 | 213.9486408 |
| YGR131W | YGR131W | 0.65041674 | 1 | -0.1462684 | 128.6972987 | 228.8796911 |
| YOR010C | TIR2 | 0.79543041 | 1 | -0.1460983 | 119.0853363 | 213.1881342 |
| YOR076C | SKI7 | 0.80639298 | 1 | -0.1458938 | 101.6513376 | 184.7406259 |
| YPL041C | YPL041C | 0.78087935 | 1 | -0.1458006 | 124.9371559 | 222.688908 |
| YKL110C | KTI12 | 0.73060031 | 1 | -0.1457386 | 92.138819 | 169.2131295 |
| YGL232W | TAN1 | 0.65307995 | 1 | -0.1457338 | 131.3311954 | 233.1037354 |
| YKL167C | MRP49 | 0.75446599 | 1 | -0.1449945 | 117.3432234 | 210.2041706 |
| YMR305C | SCW10 | 0.7611688 | 1 | -0.1449545 | 123.3263927 | 219.9526913 |
| YGL160W | YGL160W | 0.66992021 | 1 | -0.1447483 | 121.5479366 | 217.026569 |
| YKR041W | YKR041W | 0.76739869 | 1 | -0.1445813 | 129.5415048 | 230.0358633 |
| YOR113W | AZF1 | 0.79106321 | 1 | -0.1443033 | 115.1771335 | 206.5828744 |
| YGR117C | YGR117C | 0.69515318 | 1 | -0.1441251 | 128.5744672 | 228.3999043 |
| YFR012W | YFR012W | 0.70365908 | 1 | -0.1440139 | 120.9189616 | 215.9054195 |
| YNR072W | HXT17 | 0.81099035 | 1 | -0.1439197 | 120.2139608 | 214.7438526 |
| YGR093W | YGR093W | 0.66987523 | 1 | -0.1437783 | 106.189698 | 191.8631237 |

| | | | | | | |
|---|---|---|---|---|---|---|
| YOR015W | YOR015W | 0.81280091 | 1 | -0.1436208 | 125.695923 | 223.6415377 |
| YGL241W | KAP114 | 0.67340428 | 1 | -0.1432036 | 122.7394679 | 218.7675193 |
| YGL109W | YGL109W | 0.71347086 | 1 | -0.1428714 | 135.7421846 | 239.9211615 |
| YML072C | TCB3 | 0.73754337 | 1 | -0.14287 | 101.4625962 | 184.0385466 |
| YNL303W | YNL303W | 0.76609327 | 1 | -0.1427451 | 120.2221308 | 214.6039596 |
| YOL039W | RPP2A | 0.78209116 | 1 | -0.1426116 | 106.879189 | 192.8349468 |
| YOR247W | SRL1 | 0.81953872 | 1 | -0.1425724 | 129.8260022 | 230.2376258 |
| YER061C | CEM1 | 0.74635174 | 1 | -0.1422076 | 77.00531841 | 144.0820086 |
| YGL141W | HUL5 | 0.66308301 | 1 | -0.1421297 | 128.5442228 | 228.0903347 |
| YNL098C | RAS2 | 0.77565625 | 1 | -0.142063 | 126.0916312 | 224.0834341 |
| YOL047C | YOL047C | 0.8176525 | 1 | -0.1419666 | 125.6593599 | 223.3661645 |
| YKL121W | YKL121W | 0.71650313 | 1 | -0.1418273 | 105.6386423 | 190.7103153 |
| YBR267W | REI1 | 0.80365957 | 1 | -0.1417919 | 61.96654457 | 119.5116461 |
| YJR066W | TOR1 | 0.80817131 | 1 | -0.1416839 | 115.8932541 | 207.4086416 |
| YKR064W | OAF3 | 0.72446762 | 1 | -0.1416323 | 108.892933 | 195.9900131 |
| YNL325C | FIG4 | 0.72311101 | 1 | -0.1413279 | 112.081129 | 201.1476977 |
| YGL058W | RAD6 | 0.70140695 | 1 | -0.1412211 | 130.543016 | 231.230252 |
| YMR306W | FKS3 | 0.75857131 | 1 | -0.1408847 | 128.5366944 | 227.9156719 |
| YFL056C | AAD6 | 0.71249112 | 1 | -0.1408313 | 116.6986338 | 208.6103514 |
| YOR190W | SPR1 | 0.81576901 | 1 | -0.1407106 | 122.6685668 | 218.326774 |
| YGL089C | MF(ALPHA)2 | 0.71596517 | 1 | -0.1400621 | 135.517569 | 239.1885659 |
| YOL016C | CMK2 | 0.78391983 | 1 | -0.1399057 | 127.6998269 | 226.4237253 |
| YNL265C | IST1 | 0.77094053 | 1 | -0.1398817 | 128.6220988 | 227.9240804 |
| YNL082W | PMS1 | 0.77192733 | 1 | -0.13979 | 99.12094173 | 179.8194595 |
| YEL030W | ECM10 | 0.71817138 | 1 | -0.1397543 | 114.7260451 | 205.2541792 |
| YDR445C | YDR445C | 0.71270926 | 1 | -0.1396148 | 102.2635921 | 184.9197472 |
| YDR525W | API2 | 0.67307678 | 1 | -0.1394724 | 114.9248643 | 205.541526 |
| YGR026W | YGR026W | 0.69326843 | 1 | -0.1393583 | 132.8896606 | 234.8127697 |
| YMR133W | REC114 | 0.72520398 | 1 | -0.1392587 | 105.979545 | 190.9310309 |
| YMR187C | YMR187C | 0.73132418 | 1 | -0.1391341 | 100.5422573 | 182.0509364 |
| YMR179W | SPT21 | 0.75350329 | 1 | -0.1390791 | 85.06982998 | 156.8206801 |
| YKL128C | PMU1 | 0.7944811 | 1 | -0.1387846 | 114.2352559 | 204.327612 |
| YLR150W | STM1 | 0.73237427 | 1 | -0.1387844 | 96.92774191 | 176.1129609 |
| YGL202W | ARO8 | 0.65733728 | 1 | -0.1387756 | 127.424067 | 225.8267814 |
| YML055W | SPC2 | 0.71792189 | 1 | -0.1386278 | 101.7706961 | 183.9875009 |
| YDL006W | PTC1 | 0.71940461 | 1 | -0.1384963 | 83.64792222 | 154.4266829 |
| YPL018W | CTF19 | 0.82417887 | 1 | -0.137908 | 124.5675546 | 221.0569406 |
| YNR027W | BUD17 | 0.80413084 | 1 | -0.1378207 | 117.7466526 | 209.9261532 |
| YOR014W | RTS1 | 0.74603094 | 1 | -0.1373571 | 109.4090911 | 196.273835 |
| YKL094W | YJU3 | 0.74579747 | 1 | -0.137324 | 114.3446067 | 204.3153736 |
| YPR013C | YPR013C | 0.81272162 | 1 | -0.1368761 | 72.10578338 | 135.3994106 |
| YPL145C | KES1 | 0.81108399 | 1 | -0.1365565 | 114.5739437 | 204.5891284 |
| YML033W | YML033W | 0.73145635 | 1 | -0.1362277 | 107.5263914 | 193.0573542 |
| YGL067W | NPY1 | 0.6919188 | 1 | -0.1361402 | 128.6478744 | 227.4780898 |
| YOL032W | OPI10 | 0.81483041 | 1 | -0.13607 | 121.4790422 | 215.7823333 |
| YOR283W | YOR283W | 0.8253838 | 1 | -0.1359805 | 125.558851 | 222.421544 |
| YFR010W | UBP6 | 0.71352808 | 1 | -0.1358905 | 102.7062786 | 185.1556454 |
| YEL065W | SIT1 | 0.72147166 | 1 | -0.1358166 | 122.015164 | 216.6232664 |
| YGR053C | YGR053C | 0.74387042 | 1 | -0.1357743 | 127.8561932 | 226.1397661 |
| YOL046C | YOL046C | 0.81591859 | 1 | -0.1357307 | 121.1146559 | 215.1440567 |
| YOR055W | YOR055W | 0.79969767 | 1 | -0.1355552 | 133.1211663 | 234.6941205 |
| YPL197C | YPL197C | 0.82708347 | 1 | -0.1352929 | 126.7779256 | 224.3191942 |
| YER069W | ARG5 | 0.69367748 | 1 | -0.1352101 | 113.0892234 | 201.9931363 |
| YLL028W | TPO1 | 0.73558088 | 1 | -0.1352097 | 111.6439242 | 199.6369653 |
| YMR295C | YMR295C | 0.79371838 | 1 | -0.135208 | 134.380345 | 236.7015409 |
| YGR023W | MTL1 | 0.68825913 | 1 | -0.1351043 | 128.8217286 | 227.626385 |
| YGR127W | YGR127W | 0.7036312 | 1 | -0.1350785 | 137.5945253 | 241.9243947 |
| YFR055W | IRC7 | 0.70405133 | 1 | -0.1350543 | 124.8639887 | 221.1679842 |
| YPL205C | YPL205C | 0.67770701 | 1 | -0.1348945 | 12.03480847 | 37.21353359 |
| YMR004W | MVP1 | 0.797698 | 1 | -0.1348326 | 115.6536549 | 206.1244188 |
| YER131W | RPS26B | 0.77695024 | 1 | -0.1347487 | 85.5946478 | 157.111428 |
| YPL030W | TRM44 | 0.79955924 | 1 | -0.1347426 | 121.6334821 | 215.8609685 |
| YPL025C | YPL025C | 0.83580798 | 1 | -0.1347393 | 133.246963 | 234.7927767 |
| YOL137W | BSC6 | 0.81776221 | 1 | -0.1346232 | 117.7063602 | 209.4434174 |

| | | | | | | |
|---|---|---|---|---|---|---|
| YOR016C | ERP4 | 0.79947009 | 1 | -0.1345837 | 123.3983475 | 218.7173179 |
| YLR262C | YPT6 | 0.73400451 | 1 | -0.1345727 | 77.59703359 | 144.050786 |
| YPL032C | SVL3 | 0.83394012 | 1 | -0.1344034 | 126.8043734 | 224.2462847 |
| YOL126C | MDH2 | 0.83622012 | 1 | -0.1343936 | 131.0481016 | 231.163121 |
| YOR002W | ALG6 | 0.75943908 | 1 | -0.1343366 | 125.1098838 | 221.4752242 |
| YGL062W | PYC1 | 0.68253024 | 1 | -0.1342549 | 129.978489 | 229.4013518 |
| YFL050C | ALR2 | 0.72316727 | 1 | -0.1341964 | 112.4866862 | 200.8786667 |
| YMR190C | SGS1 | 0.71334607 | 1 | -0.1338902 | 92.1833136 | 167.7402627 |
| YGL258W | VEL1 | 0.71703314 | 1 | -0.1337856 | 126.8406791 | 224.2248985 |
| YPL071C | YPL071C | 0.78593381 | 1 | -0.1337215 | 124.1369279 | 219.808889 |
| YGR108W | CLB1 | 0.68778417 | 1 | -0.1336214 | 128.1131754 | 226.2778912 |
| YMR153C-A | YMR153C-A | 0.76225595 | 1 | -0.1336176 | 107.4864892 | 192.651875 |
| YOR049C | RSB1 | 0.81542938 | 1 | -0.1334943 | 118.6014112 | 210.7552848 |
| YGR066C | YGR066C | 0.74588358 | 1 | -0.1329653 | 137.8237365 | 242.0224354 |
| YMR114C | YMR114C | 0.74990293 | 1 | -0.1328149 | 107.9868652 | 193.3628799 |
| YOR153W | PDR5 | 0.80954129 | 1 | -0.132715 | 129.3335471 | 228.1491206 |
| YPR130C | YPR130C | 0.8217158 | 1 | -0.1325539 | 122.032622 | 216.2261735 |
| YLR448W | RPL6B | 0.76723514 | 1 | -0.1325198 | 94.88569998 | 171.9669283 |
| YGL039W | YGL039W | 0.72058736 | 1 | -0.1322489 | 134.4374278 | 236.4086492 |
| YLR460C | YLR460C | 0.72213345 | 1 | -0.1321933 | 99.53895676 | 179.5100663 |
| YOR050C | YOR050C | 0.81374397 | 1 | -0.1321518 | 134.0502936 | 235.7648699 |
| YGL032C | AGA2 | 0.72189055 | 1 | -0.1321297 | 131.1266739 | 230.9959222 |
| YGR014W | MSB2 | 0.73171301 | 1 | -0.131958 | 126.1100724 | 222.7954871 |
| YPL081W | RPS9A | 0.80181333 | 1 | -0.1316691 | 127.8971696 | 225.6711191 |
| YPR179C | HDA3 | 0.77232656 | 1 | -0.1315773 | 79.08351725 | 146.0833526 |
| YFL055W | AGP3 | 0.72944499 | 1 | -0.1315117 | 120.3151371 | 213.290395 |
| YOR035C | SHE4 | 0.73578463 | 1 | -0.1309329 | 81.52405793 | 149.977871 |
| YJR131W | MNS1 | 0.81625948 | 1 | -0.1307462 | 122.2996788 | 216.4257437 |
| YGR126W | YGR126W | 0.74484771 | 1 | -0.13069 | 132.2288492 | 232.6048963 |
| YGL177W | YGL177W | 0.72823721 | 1 | -0.1303779 | 130.5258037 | 229.7878945 |
| YER116C | SLX8 | 0.81203032 | 1 | -0.1302902 | 110.5172262 | 197.1585671 |
| YOL067C | RTG1 | 0.80531302 | 1 | -0.1302571 | 101.9236827 | 183.1450981 |
| YMR162C | DNF3 | 0.75599244 | 1 | -0.1302175 | 107.0223242 | 191.4517057 |
| YFR039C | YFR039C | 0.74491871 | 1 | -0.1300269 | 125.8766757 | 222.1631235 |
| YOR059C | YOR059C | 0.82023142 | 1 | -0.1299893 | 133.2958176 | 234.2528682 |
| YJR128W | YJR128W | 0.75457779 | 1 | -0.1296722 | 109.920069 | 196.1044733 |
| YNL156C | NSG2 | 0.77570348 | 1 | -0.1295032 | 124.7185409 | 220.2068305 |
| YPL248C | GAL4 | 0.84146143 | 1 | -0.1294473 | 130.1530549 | 229.0588629 |
| YOR322C | LDB19 | 0.76006408 | 1 | -0.1294059 | 108.8181069 | 194.2733348 |
| YGL166W | CUP2 | 0.70774568 | 1 | -0.1293946 | 131.5676074 | 231.3579821 |
| YLR435W | TSR2 | 0.76636832 | 1 | -0.1292406 | 83.71855215 | 153.3345957 |
| YML090W | YML090W | 0.78254427 | 1 | -0.1290356 | 91.33600838 | 165.7257973 |
| YHR004C | NEM1 | 0.70900741 | 1 | -0.1288697 | 147.6262068 | 257.4681781 |
| YML060W | OGG1 | 0.74029415 | 1 | -0.1287486 | 98.60464899 | 177.5376706 |
| YPL184C | MRN1 | 0.80144826 | 1 | -0.1287127 | 125.3448623 | 221.1247601 |
| YFR030W | MET10 | 0.72985377 | 1 | -0.1285927 | 117.4230787 | 208.1950449 |
| YFR017C | YFR017C | 0.70626933 | 1 | -0.1285099 | 127.6096326 | 224.7903251 |
| YGL164C | YRB30 | 0.72770943 | 1 | -0.1284354 | 133.5330931 | 234.4370003 |
| YDR465C | RMT2 | 0.77872527 | 1 | -0.1283391 | 122.435003 | 216.3323831 |
| YFR007W | YFR007W | 0.74602095 | 1 | -0.1282733 | 118.3366394 | 209.6426742 |
| YFR024C-A | LSB3 | 0.69676513 | 1 | -0.1281901 | 126.1418115 | 222.3557814 |
| YER186C | YER186C | 0.74752157 | 1 | -0.1281826 | 116.9981584 | 207.4488549 |
| YNL111C | CYB5 | 0.81958767 | 1 | -0.1280235 | 115.3365252 | 204.7193197 |
| YNR029C | YNR029C | 0.81413387 | 1 | -0.1280029 | 104.0501917 | 186.3177035 |
| YKR072C | SIS2 | 0.80878122 | 1 | -0.1278854 | 114.0484509 | 202.6014909 |
| YGR051C | YGR051C | 0.70452129 | 1 | -0.1278471 | 130.810924 | 229.922601 |
| YOR193W | PEX27 | 0.81773177 | 1 | -0.1272545 | 135.3042086 | 237.1702367 |
| YEL010W | YEL010W | 0.75410259 | 1 | -0.1271161 | 121.3756282 | 214.4458862 |
| YKR019C | IRS4 | 0.74436808 | 1 | -0.1269253 | 108.3230998 | 193.14283 |
| YGL224C | SDT1 | 0.72567534 | 1 | -0.1269048 | 123.1830469 | 217.364764 |
| YJR116W | YJR116W | 0.72642242 | 1 | -0.1268498 | 84.6126788 | 154.4803553 |
| YNL089C | YNL089C | 0.74888283 | 1 | -0.1267983 | 106.1719326 | 189.6194399 |
| YGR035C | YGR035C | 0.72123683 | 1 | -0.1265061 | 121.8606806 | 215.1570451 |
| YMR119W | ASI1 | 0.72177761 | 1 | -0.126335 | 121.8917923 | 215.1854442 |

| | | | | | | |
|---|---|---|---|---|---|---|
| YEL040W | UTR2 | 0.75038923 | 1 | -0.1263095 | 119.6226279 | 211.4829389 |
| YNL223W | ATG4 | 0.7892736 | 1 | -0.1262148 | 100.489017 | 180.2790676 |
| YPL256C | CLN2 | 0.82805249 | 1 | -0.1262104 | 122.2818044 | 215.8049939 |
| YOR298C-A | MBF1 | 0.84035155 | 1 | -0.1258 | 126.9094802 | 223.2954784 |
| YNR065C | YSN1 | 0.80507432 | 1 | -0.1255744 | 125.3813735 | 220.7749473 |
| YOR274W | MOD5 | 0.84981183 | 1 | -0.1255408 | 131.8612687 | 231.334055 |
| YGL257C | MNT2 | 0.74223527 | 1 | -0.1254036 | 137.6948883 | 240.826095 |
| YPL026C | SKS1 | 0.84176047 | 1 | -0.125375 | 125.1038386 | 220.2965005 |
| YMR261C | TPS3 | 0.80849984 | 1 | -0.1253271 | 109.9358826 | 195.5635161 |
| YPL058C | PDR12 | 0.83282023 | 1 | -0.1252664 | 120.0017359 | 211.9649064 |
| YNL069C | RPL16B | 0.7904961 | 1 | -0.1250074 | 109.5085477 | 194.8251763 |
| YOL031C | SIL1 | 0.82252424 | 1 | -0.1249691 | 131.1154065 | 230.043591 |
| YOL030W | GAS5 | 0.81777474 | 1 | -0.1248144 | 113.1985115 | 200.815374 |
| YGR107W | YGR107W | 0.69909195 | 1 | -0.1246741 | 111.3831539 | 197.8376902 |
| YOR041C | SRF5 | 0.83649635 | 1 | -0.1246178 | 122.8932263 | 216.5940068 |
| YOL118C | YOL118C | 0.82026885 | 1 | -0.1238749 | 130.5415192 | 228.9653238 |
| YMR303C | ADH2 | 0.82260349 | 1 | -0.1238673 | 118.4714552 | 209.2877686 |
| YFL040W | YFL040W | 0.76516302 | 1 | -0.1238284 | 109.6659694 | 194.9280347 |
| YOL054W | PSH1 | 0.82688088 | 1 | -0.1238209 | 114.6509035 | 203.0534752 |
| YFR016C | YFR016C | 0.72585432 | 1 | -0.1238017 | 127.2618156 | 223.6092216 |
| YOL059W | GPD2 | 0.84032102 | 1 | -0.1234166 | 125.0609308 | 219.9711094 |
| YGL158W | RCK1 | 0.71368539 | 1 | -0.1233789 | 128.4171234 | 225.4374501 |
| YNL327W | EGT2 | 0.80278305 | 1 | -0.1227472 | 128.1595957 | 224.9352401 |
| YGR049W | SCM4 | 0.72078651 | 1 | -0.122535 | 134.0247491 | 234.4688998 |
| YOL122C | SMF1 | 0.83029223 | 1 | -0.1225175 | 117.4250097 | 207.4057997 |
| YJR126C | VPS70 | 0.75584474 | 1 | -0.1220771 | 109.8914701 | 195.0672217 |
| YML027W | YOX1 | 0.74130052 | 1 | -0.1220719 | 92.71764579 | 167.0698532 |
| YOR107W | RGS2 | 0.79586667 | 1 | -0.1217886 | 115.085501 | 203.4968714 |
| YMR171C | YMR171C | 0.82190685 | 1 | -0.1215336 | 120.9837057 | 213.078841 |
| YKL032C | IXR1 | 0.83767214 | 1 | -0.1214643 | 113.0120407 | 200.0744272 |
| YOL044W | PEX15 | 0.83370867 | 1 | -0.1214212 | 120.7717414 | 212.7186345 |
| YDR459C | PFA5 | 0.76167554 | 1 | -0.1214166 | 114.8554933 | 203.0733898 |
| YER121W | YER121W | 0.7894671 | 1 | -0.1209434 | 125.5915976 | 220.5136299 |
| YGL210W | YPT32 | 0.72086964 | 1 | -0.120711 | 107.0479799 | 190.2535888 |
| YNL250W | RAD50 | 0.4511508 | 1 | -0.1204365 | 82.86254977 | 150.7908179 |
| YGL081W | YGL081W | 0.71685381 | 1 | -0.120292 | 132.9214432 | 232.3777475 |
| YGL216W | KIP3 | 0.72097687 | 1 | -0.120085 | 113.2106223 | 200.2182503 |
| YOR013W | YOR013W | 0.83688033 | 1 | -0.1200758 | 116.6758682 | 205.8660867 |
| YER035W | EDC2 | 0.75960547 | 1 | -0.1200454 | 118.5754052 | 208.9587265 |
| YOR280C | FSH3 | 0.81810097 | 1 | -0.119889 | 124.0169555 | 217.8091169 |
| YOR085W | OST3 | 0.82092603 | 1 | -0.1197747 | 108.9949883 | 193.3054734 |
| YER010C | YER010C | 0.77413393 | 1 | -0.1195644 | 125.9140574 | 220.8594257 |
| YGL021W | ALK1 | 0.72688332 | 1 | -0.1193511 | 128.6826987 | 225.3450343 |
| YKR088C | TVP38 | 0.77867199 | 1 | -0.1192564 | 115.6239013 | 204.0442897 |
| YFL019C | YFL019C | 0.78751901 | 1 | -0.119212 | 125.2607082 | 219.7483855 |
| YOR252W | TMA16 | 0.82940555 | 1 | -0.11873 | 103.7246395 | 184.5775072 |
| YEL068C | YEL068C | 0.75686347 | 1 | -0.1187297 | 113.734126 | 200.8948972 |
| YFL033C | RIM15 | 0.81586821 | 1 | -0.1187015 | 103.6073595 | 184.3826077 |
| YGL213C | SKI8 | 0.76445717 | 1 | -0.1184563 | 123.3568248 | 216.5461171 |
| YOR214C | YOR214C | 0.84912051 | 1 | -0.1182155 | 131.0918442 | 229.1243025 |
| YFL014W | HSP12 | 0.81349969 | 1 | -0.1181998 | 134.8181999 | 235.1969357 |
| YGR096W | TPC1 | 0.74627589 | 1 | -0.1178912 | 122.8967292 | 215.7223608 |
| YGR188C | BUB1 | 0.79512235 | 1 | -0.1177205 | 84.51923123 | 153.1372804 |
| YGL060W | YBP2 | 0.73114495 | 1 | -0.1175512 | 123.5074872 | 216.6736684 |
| YEL042W | GDA1 | 0.717601 | 1 | -0.1172724 | 109.5183528 | 193.8322835 |
| YOR367W | SCP1 | 0.83427111 | 1 | -0.1172309 | 126.8192362 | 222.030683 |
| YNR061C | YNR061C | 0.83611843 | 1 | -0.1171129 | 117.8113903 | 207.3307462 |
| YGR010W | NMA2 | 0.70547932 | 1 | -0.1170911 | 118.5241206 | 208.4897915 |
| YNL146W | YNL146W | 0.8416265 | 1 | -0.1170858 | 122.9200889 | 215.6553967 |
| YDL100C | GET3 | 0.38329792 | 1 | -0.1169566 | 123.1087197 | 215.9460503 |
| YOL004W | SIN3 | 0.78148358 | 1 | -0.1167807 | 85.72370003 | 154.9782096 |
| YOL119C | MCH4 | 0.81194772 | 1 | -0.116743 | 115.1887941 | 203.0071579 |
| YMR316C-B | YMR316C-B | 0.8089566 | 1 | -0.1165313 | 127.8342591 | 223.5941219 |
| YOL124C | TRM11 | 0.82600463 | 1 | -0.1164003 | 108.7741928 | 192.5054116 |

| | | | | | | |
|---|---|---|---|---|---|---|
| YGL139W | FLC3 | 0.76912543 | 1 | -0.1163315 | 130.4247894 | 227.7911394 |
| YKL221W | MCH2 | 0.77163468 | 1 | -0.1162416 | 107.7939314 | 190.8866896 |
| YFR045W | YFR045W | 0.74536317 | 1 | -0.1162143 | 132.6671266 | 231.4312946 |
| YPR148C | YPR148C | 0.84053675 | 1 | -0.1161618 | 122.8778895 | 215.4660801 |
| YLR360W | VPS38 | 0.79153711 | 1 | -0.1159359 | 94.84050396 | 169.7301969 |
| YER048C | CAJ1 | 0.78526511 | 1 | -0.1157533 | 127.3093209 | 222.6368987 |
| YGR016W | YGR016W | 0.73883683 | 1 | -0.1156186 | 131.6692424 | 229.7268488 |
| YFR053C | HXK1 | 0.74813739 | 1 | -0.1154129 | 134.9044673 | 234.9740767 |
| YMR198W | CIK1 | 0.75754228 | 1 | -0.1151771 | 88.66375297 | 159.5619133 |
| YGR084C | MRP13 | 0.75783737 | 1 | -0.1150697 | 126.1114974 | 220.59505 |
| YER187W | YER187W | 0.77397596 | 1 | -0.115068 | 118.6363343 | 208.4088545 |
| YKL061W | YKL061W | 0.81582642 | 1 | -0.1148198 | 107.718048 | 190.5775348 |
| YOR173W | DCS2 | 0.87724884 | 1 | -0.1146459 | 70.00246924 | 129.0710972 |
| YOR324C | FRT1 | 0.860675 | 1 | -0.1145691 | 131.7683469 | 229.7515198 |
| YMR311C | GLC8 | 0.81125166 | 1 | -0.1142856 | 130.6671241 | 227.9193446 |
| YNL214W | PEX17 | 0.82391903 | 1 | -0.1141603 | 126.7610303 | 221.5353036 |
| YER135C | YER135C | 0.80405495 | 1 | -0.1141603 | 122.8634432 | 215.1814716 |
| YDR382W | RPP2B | 0.78496689 | 1 | -0.1141329 | 42.92478399 | 84.86226328 |
| YPL176C | TRE1 | 0.85038557 | 1 | -0.1137323 | 119.3524846 | 209.4020949 |
| YOL103W | ITR2 | 0.83570115 | 1 | -0.1137033 | 114.8010697 | 201.9786182 |
| YOR246C | YOR246C | 0.84076822 | 1 | -0.113519 | 133.0202601 | 231.6554253 |
| YPL219W | PCL8 | 0.85664723 | 1 | -0.1133856 | 129.7418819 | 226.2936264 |
| YDR486C | VPS60 | 0.77890287 | 1 | -0.1133633 | 123.526126 | 216.1578151 |
| YPL112C | PEX25 | 0.85496673 | 1 | -0.1132446 | 125.7000895 | 219.6863187 |
| YOL080C | REX4 | 0.83412532 | 1 | -0.1130323 | 113.9907808 | 200.5701751 |
| YER011W | TIR1 | 0.80447681 | 1 | -0.1128783 | 127.9318358 | 223.2767311 |
| YOR309C | YOR309C | 0.68775543 | 1 | -0.1125776 | 96.49138889 | 171.9834349 |
| YLL043W | FPS1 | 0.81325105 | 1 | -0.1125405 | 103.7175957 | 183.7587239 |
| YGL180W | ATG1 | 0.77406339 | 1 | -0.1124384 | 136.612391 | 237.3703595 |
| YNL083W | SAL1 | 0.80803953 | 1 | -0.112361 | 99.69303701 | 177.1745037 |
| YOR070C | GYP1 | 0.82648782 | 1 | -0.1123415 | 109.4226594 | 193.0331436 |
| YGL226C-A | OST5 | 0.72683483 | 1 | -0.1121615 | 127.4609735 | 222.4156409 |
| YNR066C | YNR066C | 0.84402822 | 1 | -0.1119577 | 137.3405751 | 238.4947344 |
| YNR069C | BSC5 | 0.83613677 | 1 | -0.11165 | 111.7579214 | 196.7498799 |
| YNR058W | BIO3 | 0.83345814 | 1 | -0.1113754 | 133.7930362 | 232.6356068 |
| YPL047W | SGF11 | 0.83001494 | 1 | -0.1112988 | 105.3825265 | 186.3109378 |
| YGR151C | YGR151C | 0.7551161 | 1 | -0.1109862 | 125.6249571 | 219.2692832 |
| YGL234W | ADE5 | 0.69430565 | 1 | -0.1105719 | 118.662293 | 207.864743 |
| YMR251W-A | HOR7 | 0.80932534 | 1 | -0.1105619 | 127.6682709 | 222.5449357 |
| YOR273C | TPO4 | 0.84173572 | 1 | -0.1104138 | 113.2062628 | 198.9497271 |
| YDR430C | CYM1 | 0.80406776 | 1 | -0.1103437 | 123.2621772 | 215.3336776 |
| YPL073C | YPL073C | 0.84396318 | 1 | -0.110299 | 132.2366134 | 229.9579324 |
| YCR009C | RVS161 | 0.83411248 | 1 | -0.1100244 | 65.46534128 | 121.0718966 |
| YMR074C | YMR074C | 0.85867364 | 1 | -0.1097413 | 123.8914158 | 216.2808931 |
| YPL163C | SVS1 | 0.85950111 | 1 | -0.1096805 | 125.0885697 | 218.2245572 |
| YOR166C | SWT1 | 0.84620903 | 1 | -0.1092122 | 128.4071645 | 223.5734408 |
| YER049W | TPA1 | 0.78120946 | 1 | -0.1091075 | 120.4818766 | 210.64001 |
| YMR078C | CTF18 | 0.79507586 | 1 | -0.1090995 | 75.41700059 | 137.1744126 |
| YGR045C | YGR045C | 0.75992443 | 1 | -0.1088734 | 114.6325881 | 201.0739914 |
| YNL052W | COX5A | 0.84192106 | 1 | -0.1086544 | 113.6512753 | 199.4456929 |
| YNL302C | RPS19B | 0.78515943 | 1 | -0.1084891 | 76.28797556 | 138.5146624 |
| YNR055C | HOL1 | 0.85430312 | 1 | -0.1084333 | 122.6143624 | 214.0284413 |
| YNR019W | ARE2 | 0.83842457 | 1 | -0.1082569 | 111.4723425 | 195.841761 |
| YGR039W | YGR039W | 0.76947788 | 1 | -0.1081104 | 132.4878706 | 230.0820715 |
| YPR005C | HAL1 | 0.87157817 | 1 | -0.1080473 | 137.5750026 | 238.366861 |
| YGL138C | YGL138C | 0.77167621 | 1 | -0.108045 | 133.6655858 | 231.9934452 |
| YPL051W | ARL3 | 0.86189003 | 1 | -0.1080332 | 126.8679479 | 220.9104335 |
| YNL318C | HXT14 | 0.81201527 | 1 | -0.1077917 | 121.2275602 | 211.6839991 |
| YGL110C | CUE3 | 0.74424452 | 1 | -0.1076976 | 129.2428113 | 224.7381507 |
| YKL166C | TPK3 | 0.84363427 | 1 | -0.1075958 | 109.6370617 | 192.7636716 |
| YEL063C | CAN1 | 0.7913075 | 1 | -0.1075722 | 119.3682794 | 208.6243751 |
| YKL151C | YKL151C | 0.79663004 | 1 | -0.1075269 | 119.913318 | 209.5069943 |
| YOL115W | PAP2 | 0.8258448 | 1 | -0.107341 | 100.0691605 | 177.1328912 |
| YEL025C | YEL025C | 0.80174162 | 1 | -0.1072951 | 127.3892194 | 221.6639329 |

| | | | | | | |
|---|---|---|---|---|---|---|
| YNL134C | YNL134C | 0.8274655 | 1 | -0.1068163 | 106.4008371 | 187.3863257 |
| YKL103C | LAP4 | 0.81285005 | 1 | -0.106332 | 117.9900527 | 206.2158376 |
| YFL003C | MSH4 | 0.85401731 | 1 | -0.1062618 | 114.3528637 | 200.2773502 |
| YNL311C | SKP2 | 0.83314058 | 1 | -0.1061345 | 108.7703364 | 191.1601445 |
| YML067C | ERV41 | 0.82458327 | 1 | -0.1060562 | 112.8351523 | 197.7763768 |
| YMR183C | SSO2 | 0.7710761 | 1 | -0.105951 | 93.38726802 | 166.0588016 |
| YOL012C | HTZ1 | 0.83744169 | 1 | -0.1058187 | 109.206045 | 191.8292462 |
| YOR135C | YOR135C | 0.85566677 | 1 | -0.10579 | 119.6800668 | 208.900205 |
| YGL159W | YGL159W | 0.76538295 | 1 | -0.1057898 | 124.7614293 | 217.1837882 |
| YOL141W | PPM2 | 0.84876482 | 1 | -0.1056578 | 136.0378223 | 235.5492935 |
| YLR434C | YLR434C | 0.75994496 | 1 | -0.1049165 | 97.82968225 | 173.1658759 |
| YPL034W | YPL034W | 0.8693768 | 1 | -0.1048118 | 129.8866761 | 225.4113847 |
| YOR175C | YOR175C | 0.82898931 | 1 | -0.1043723 | 115.7780487 | 202.3542298 |
| YKR040C | YKR040C | 0.80469096 | 1 | -0.1043693 | 112.0548847 | 196.2843566 |
| YOR039W | CKB2 | 0.7728732 | 1 | -0.1041668 | 91.05336802 | 162.021365 |
| YFR021W | ATG18 | 0.75705871 | 1 | -0.1041511 | 123.3502746 | 214.6695908 |
| YKR098C | UBP11 | 0.78844342 | 1 | -0.1038258 | 105.6751481 | 185.8132566 |
| YPL257W | YPL257W | 0.86848404 | 1 | -0.103763 | 129.1220148 | 224.0280347 |
| YKL148C | SDH1 | 0.83052876 | 1 | -0.1037157 | 104.2794608 | 183.5236467 |
| YPL003W | ULA1 | 0.86216416 | 1 | -0.1036315 | 124.8817475 | 217.0984172 |
| YKL044W | YKL044W | 0.80406144 | 1 | -0.1035494 | 119.1336613 | 207.7172122 |
| YKL027W | YKL027W | 0.8101063 | 1 | -0.1034206 | 115.1361663 | 201.1837113 |
| YGL118C | YGL118C | 0.73213074 | 1 | -0.1031689 | 129.952843 | 225.3049582 |
| YGL228W | SHE10 | 0.79068513 | 1 | -0.1030727 | 131.3213128 | 227.5232841 |
| YGL015C | YGL015C | 0.77948783 | 1 | -0.1029851 | 131.4943497 | 227.7939452 |
| YDR436W | PPZ2 | 0.80224831 | 1 | -0.1028903 | 122.7386602 | 213.5080919 |
| YGR124W | ASN2 | 0.78107536 | 1 | -0.1027949 | 134.0407563 | 231.9202682 |
| YER119C-A | YER119C-A | 0.84479984 | 1 | -0.1024664 | 95.50414372 | 169.0552176 |
| YNL257C | SIP3 | 0.84980719 | 1 | -0.1023982 | 113.8406826 | 198.9384676 |
| YNL027W | CRZ1 | 0.78360906 | 1 | -0.1020205 | 132.4088419 | 229.1589322 |
| YNL029C | KTR5 | 0.80166519 | 1 | -0.1016299 | 86.63546738 | 154.4884352 |
| YER092W | IES5 | 0.1853229 | 1 | -0.1011331 | 0 | 13.19090227 |
| YOL024W | YOL024W | 0.84897831 | 1 | -0.1010865 | 126.8584047 | 219.9888093 |
| YEL028W | YEL028W | 0.79618949 | 1 | -0.1010576 | 116.3548499 | 202.8621983 |
| YNL321W | YNL321W | 0.84111026 | 1 | -0.100963 | 130.1662239 | 225.3650949 |
| YKL158W | YKL158W | 0.8274835 | 1 | -0.1008455 | 122.7249533 | 213.2190412 |
| YKL149C | DBR1 | 0.82948509 | 1 | -0.1008005 | 102.800339 | 180.7321538 |
| YOL155C | HPF1 | 0.84127307 | 1 | -0.1005145 | 116.5494201 | 203.108539 |
| YGR181W | TIM13 | 0.87163407 | 1 | -0.100503 | 134.4360914 | 232.2658185 |
| YOR005C | DNL4 | 0.8680596 | 1 | -0.1004837 | 125.7510087 | 218.1049138 |
| YBR288C | APM3 | 0.78457548 | 1 | -0.1004684 | 105.9067472 | 185.7528987 |
| YER062C | HOR2 | 0.79745354 | 1 | -0.1001186 | 109.096911 | 190.9078583 |
| YMR075C-A | YMR075C-A | 0.79942808 | 1 | -0.0999965 | 99.28396481 | 174.8949106 |
| YBR164C | ARL1 | 0.72726318 | 1 | -0.0998347 | 136.8859284 | 236.1723597 |
| YOR360C | PDE2 | 0.85416133 | 1 | -0.0997925 | 112.4891512 | 196.395347 |
| YML076C | WAR1 | 0.81194267 | 1 | -0.0997539 | 106.5374738 | 186.6879072 |
| YER046W-A | YER046W-A | 0.81056183 | 1 | -0.0996501 | 120.351786 | 209.1943966 |
| YOR006C | YOR006C | 0.83746849 | 1 | -0.0994555 | 114.1764085 | 199.1019484 |
| YNL024C | YNL024C | 0.83442946 | 1 | -0.0993862 | 123.3330552 | 214.0200316 |
| YKR095W | MLP1 | 0.78626748 | 1 | -0.099347 | 107.1866285 | 187.6930853 |
| YGR137W | YGR137W | 0.79252014 | 1 | -0.0993038 | 139.2464522 | 239.9512257 |
| YOR208W | PTP2 | 0.8492233 | 1 | -0.0992482 | 117.6919495 | 204.8059272 |
| YOR037W | CYC2 | 0.85930419 | 1 | -0.0991729 | 118.4160585 | 205.9765445 |
| YFR013W | IOC3 | 0.75602179 | 1 | -0.0990437 | 114.4522988 | 199.497994 |
| YOR292C | YOR292C | 0.8753656 | 1 | -0.0989431 | 131.0821241 | 226.5947299 |
| YMR002W | MIC17 | 0.8212447 | 1 | -0.0988597 | 111.0524615 | 193.9315834 |
| YOR133W | EFT1 | 0.85348575 | 1 | -0.0982165 | 124.842305 | 216.3278294 |
| YOL020W | TAT2 | 0.86316081 | 1 | -0.0981896 | 116.5848549 | 202.8630695 |
| YNR075W | COS10 | 0.84230956 | 1 | -0.0981712 | 115.137513 | 200.501215 |
| YML028W | TSA1 | 0.54266839 | 1 | -0.0981251 | 108.1188396 | 189.0534012 |
| YGR132C | PHB1 | 0.80144304 | 1 | -0.0979427 | 130.1246228 | 224.9033369 |
| YER032W | FIR1 | 0.79183261 | 1 | -0.0979082 | 118.9225667 | 206.6372898 |
| YPR118W | YPR118W | 0.86809323 | 1 | -0.0978942 | 122.7713968 | 212.9098139 |
| YDR497C | ITR1 | 0.81273432 | 1 | -0.0978918 | 120.6772417 | 209.4956195 |

| | | | | | | |
|---|---|---|---|---|---|---|
| YMR255W | GFD1 | 0.81987332 | 1 | -0.0978884 | 118.2111458 | 205.474955 |
| YNL296W | YNL296W | 0.85114978 | 1 | -0.0978483 | 105.5315426 | 184.7994918 |
| YJR140C | HIR3 | 0.76893147 | 1 | -0.0978476 | 92.34275343 | 163.2991056 |
| YDR536W | STL1 | 0.80865946 | 1 | -0.0976403 | 118.2506164 | 205.5069445 |
| YKR005C | YKR005C | 0.73245097 | 1 | -0.0975646 | 137.4021049 | 236.717736 |
| YDR490C | PKH1 | 0.82937393 | 1 | -0.097414 | 124.7773871 | 216.1173393 |
| YDR439W | LRS4 | 0.82832335 | 1 | -0.0973438 | 118.8376481 | 206.4252508 |
| YOL028C | YAP7 | 0.85874674 | 1 | -0.097318 | 134.2421481 | 231.5342277 |
| YML107C | PML39 | 0.80043037 | 1 | -0.0968739 | 100.667698 | 176.7433866 |
| YOL092W | YOL092W | 0.87253304 | 1 | -0.096787 | 120.3456678 | 208.8109868 |
| YNL295W | YNL295W | 0.84901223 | 1 | -0.0965854 | 111.5443516 | 194.4368283 |
| YDL052C | SLC1 | 0.82990186 | 1 | -0.0965689 | 27.5567412 | 57.51846215 |
| YGL053W | PRM8 | 0.7924331 | 1 | -0.0964316 | 133.2342145 | 229.7754793 |
| YKR042W | UTH1 | 0.81463382 | 1 | -0.0963825 | 120.2563756 | 208.6126651 |
| YEL023C | YEL023C | 0.81176932 | 1 | -0.096029 | 116.9963448 | 203.2520716 |
| YNL171C | 0 | 0.81008246 | 1 | -0.0959899 | 84.28006668 | 149.913042 |
| YKL207W | YKL207W | 0.83303732 | 1 | -0.0956641 | 118.1460795 | 205.0787734 |
| YNL212W | VID27 | 0.84462513 | 1 | -0.0956037 | 125.4619485 | 216.9971819 |
| YGL104C | VPS73 | 0.77636374 | 1 | -0.0955275 | 118.2373062 | 205.2096661 |
| YNL233W | BNI4 | 0.83326629 | 1 | -0.0954325 | 122.4845123 | 212.1210491 |
| YOR118W | RTC5 | 0.85670782 | 1 | -0.0954135 | 128.9908753 | 222.7252164 |
| YFR040W | SAP155 | 0.77607287 | 1 | -0.0952895 | 107.6818766 | 187.9712154 |
| YMR262W | YMR262W | 0.8258401 | 1 | -0.0948641 | 112.4426321 | 195.6766906 |
| YOR134W | BAG7 | 0.87396355 | 1 | -0.0947931 | 120.2152998 | 208.3383966 |
| YOL152W | FRE7 | 0.85392926 | 1 | -0.0947772 | 128.9716083 | 222.6108165 |
| YPL200W | CSM4 | 0.87751497 | 1 | -0.0945214 | 120.5761449 | 208.8912088 |
| YGL079W | YGL079W | 0.77592359 | 1 | -0.0944446 | 127.7072878 | 220.5063443 |
| YNL324W | YNL324W | 0.86340255 | 1 | -0.0943411 | 133.5552816 | 230.0262228 |
| YFR032C-A | RPL29 | 0.79641076 | 1 | -0.0942324 | 111.8213698 | 194.5815186 |
| YMR012W | CLU1 | 0.84560961 | 1 | -0.0941466 | 119.8542888 | 207.6655511 |
| YPL070W | MUK1 | 0.8578606 | 1 | -0.0937701 | 129.0486978 | 222.6051286 |
| YBR170C | NPL4 | 0.85267299 | 1 | -0.0937494 | 121.1785555 | 209.7725555 |
| YER007C-A | TMA20 | 0.79647791 | 1 | -0.09372 | 105.0386355 | 183.4574998 |
| YLR061W | RPL22A | 0.18327541 | 1 | -0.0933858 | 0 | 12.1804188 |
| YKR020W | VPS51 | 0.79554778 | 1 | -0.0933203 | 97.87758339 | 171.7314594 |
| YGL146C | YGL146C | 0.79074235 | 1 | -0.0932549 | 126.4147401 | 218.2440629 |
| YKR052C | MRS4 | 0.83587434 | 1 | -0.0932213 | 117.6746018 | 203.9915448 |
| YKR074W | YKR074W | 0.81842592 | 1 | -0.0931234 | 87.48912681 | 154.7705617 |
| YNL333W | SNZ2 | 0.82951781 | 1 | -0.0930623 | 119.1716756 | 206.4113287 |
| YKR009C | FOX2 | 0.85949161 | 1 | -0.0929666 | 114.0144628 | 197.9915917 |
| YDR522C | SPS2 | 0.79997274 | 1 | -0.0927945 | 104.0915683 | 181.7928857 |
| YER044C-A | MEI4 | 0.8260746 | 1 | -0.0922676 | 118.8892482 | 205.8472657 |
| YDR431W | YDR431W | 0.82251567 | 1 | -0.0921188 | 124.0508112 | 214.2422183 |
| YNL197C | WHI3 | 0.82562758 | 1 | -0.0920745 | 91.5039578 | 161.1787091 |
| YPL088W | YPL088W | 0.86232382 | 1 | -0.091834 | 121.8111192 | 210.5539384 |
| YPR077C | YPR077C | 0.86190615 | 1 | -0.0914981 | 135.1370046 | 232.233918 |
| YNR047W | YNR047W | 0.84959661 | 1 | -0.0912353 | 103.0282454 | 179.8560849 |
| YDR438W | THI74 | 0.82068084 | 1 | -0.0911983 | 122.5753394 | 211.7168507 |
| YKR039W | GAP1 | 0.83607373 | 1 | -0.0910726 | 119.1833534 | 206.1708549 |
| YPR167C | MET16 | 0.87247898 | 1 | -0.0906189 | 110.0551774 | 191.2309637 |
| YEL047C | YEL047C | 0.82099735 | 1 | -0.0904262 | 113.0916415 | 196.1558634 |
| YOR227W | YOR227W | 0.88329598 | 1 | -0.090364 | 126.6918759 | 218.3187838 |
| YPL170W | DAP1 | 0.89073387 | 1 | -0.0903151 | 134.7178436 | 231.3963024 |
| YLR437C | YLR437C | 0.86084512 | 1 | -0.0902894 | 126.1102342 | 217.3608685 |
| YFR033C | QCR6 | 0.80054363 | 1 | -0.0901507 | 135.6288952 | 232.8600528 |
| YML042W | CAT2 | 0.82595428 | 1 | -0.0898229 | 103.7086583 | 180.7810785 |
| YOR195W | SLK19 | 0.86910364 | 1 | -0.0897658 | 133.0295034 | 228.5723407 |
| YKR027W | BCH2 | 0.81472393 | 1 | -0.0895622 | 82.23669623 | 145.743576 |
| YPL155C | KIP2 | 0.89076547 | 1 | -0.0895394 | 135.5491632 | 232.6503363 |
| YKL015W | PUT3 | 0.83630424 | 1 | -0.0894537 | 121.8470583 | 210.3020536 |
| YDR481C | PHO8 | 0.83836259 | 1 | -0.089258 | 120.5906539 | 208.2283507 |
| YBR059C | AKL1 | 0.85478588 | 1 | -0.0892175 | 131.3324041 | 225.7342116 |
| YER183C | FAU1 | 0.7943886 | 1 | -0.0890927 | 123.4136708 | 212.8088521 |
| YGR038W | ORM1 | 0.820876 | 1 | -0.0884203 | 120.3850597 | 207.7839251 |

| | | | | | | |
|---|---|---|---|---|---|---|
| YGL114W | YGL114W | 0.82727862 | 1 | -0.0883075 | 129.4154416 | 222.490497 |
| YPR189W | SKI3 | 0.87517986 | 1 | -0.0882368 | 115.0630894 | 199.0841417 |
| YNL054W | VAC7 | 0.87263433 | 1 | -0.088223 | 136.8170578 | 234.54556 |
| YER081W | SER3 | 0.80991107 | 1 | -0.0879353 | 115.5196745 | 199.7891428 |
| YOL136C | PFK27 | 0.88982795 | 1 | -0.0875664 | 126.8425026 | 218.1994475 |
| YKR106W | YKR106W | 0.83878312 | 1 | -0.0874815 | 121.7967515 | 209.9628099 |
| YOR377W | ATF1 | 0.88451142 | 1 | -0.087468 | 128.0849549 | 220.212055 |
| YGL027C | CWH41 | 0.82018543 | 1 | -0.0873429 | 125.6626211 | 216.2468616 |
| YGR141W | VPS62 | 0.79870383 | 1 | -0.0873267 | 131.5273848 | 225.8054594 |
| YLR334C | YLR334C | 0.84991165 | 1 | -0.0869612 | 87.71772108 | 154.3394698 |
| YJR152W | DAL5 | 0.81555799 | 1 | -0.0868763 | 102.7407985 | 178.8189522 |
| YGL203C | KEX1 | 0.78413362 | 1 | -0.0865089 | 123.9100813 | 213.2810988 |
| YGR161C | RTS3 | 0.80979404 | 1 | -0.0864866 | 132.6704616 | 227.5593117 |
| YLR432W | IMD3 | 0.82684663 | 1 | -0.0861824 | 100.7789609 | 175.5302601 |
| YNL316C | PHA2 | 0.86192438 | 1 | -0.0859148 | 108.6161208 | 188.2714568 |
| YNL109W | YNL109W | 0.87470767 | 1 | -0.0855922 | 108.5721544 | 188.1577082 |
| YOR178C | GAC1 | 0.89319955 | 1 | -0.0853873 | 130.2351106 | 223.445831 |
| YDR477W | SNF1 | 0.87673057 | 1 | -0.0853084 | 109.1639669 | 189.0854624 |
| YKL023W | YKL023W | 0.83569184 | 1 | -0.0851385 | 114.9806478 | 198.5456341 |
| YGL196W | YGL196W | 0.77738626 | 1 | -0.085003 | 121.882308 | 209.7790173 |
| YGR152C | RSR1 | 0.83027992 | 1 | -0.0849926 | 142.60775 | 243.5641791 |
| YOR352W | YOR352W | 0.89292615 | 1 | -0.0849285 | 127.7938956 | 219.4063322 |
| YNL164C | IBD2 | 0.87936054 | 1 | -0.0847851 | 122.8804393 | 211.3777338 |
| YOR083W | WHI5 | 0.87943435 | 1 | -0.0847016 | 116.8445196 | 201.5271223 |
| YMR219W | ESC1 | 0.84913618 | 1 | -0.0846618 | 107.0963064 | 185.6304314 |
| YNR067C | DSE4 | 0.8636304 | 1 | -0.0845639 | 126.0970881 | 216.592652 |
| YGR080W | TWF1 | 0.83099324 | 1 | -0.084454 | 126.9307024 | 217.93727 |
| YOR124C | UBP2 | 0.88024879 | 1 | -0.0840553 | 116.0540748 | 200.1542425 |
| YOR082C | YOR082C | 0.86063478 | 1 | -0.0838425 | 121.2641119 | 208.6198592 |
| YOL159C | YOL159C | 0.88698426 | 1 | -0.0834744 | 121.0633806 | 208.2446159 |
| YOR186W | YOR186W | 0.87997308 | 1 | -0.0833586 | 117.2827072 | 202.0662787 |
| YPL246C | RBD2 | 0.89296655 | 1 | -0.0832834 | 124.0864441 | 213.1478946 |
| YMR111C | YMR111C | 0.86129313 | 1 | -0.0830728 | 112.3341579 | 193.9619081 |
| YOR188W | MSB1 | 0.88250175 | 1 | -0.0829972 | 134.6017853 | 230.25263 |
| YNL288W | CAF40 | 0.86163232 | 1 | -0.0827751 | 122.6425575 | 210.7277767 |
| YPL064C | CWC27 | 0.87349067 | 1 | -0.0827429 | 120.9155011 | 207.9081371 |
| YNR048W | YNR048W | 0.87177583 | 1 | -0.0825916 | 130.3864558 | 223.3279054 |
| YMR224C | MRE11 | 0.4890667 | 1 | -0.0822322 | 85.52786963 | 150.1527692 |
| YKR033C | YKR033C | 0.8482549 | 1 | -0.0821755 | 119.911491 | 206.1974065 |
| YNL106C | INP52 | 0.85117337 | 1 | -0.0821022 | 95.04134264 | 165.6446363 |
| YNR009W | NRM1 | 0.87596116 | 1 | -0.0814517 | 113.0952568 | 194.9912 |
| YNL208W | YNL208W | 0.83637685 | 1 | -0.0814325 | 106.1982373 | 183.7452008 |
| YBR078W | ECM33 | 0.87466496 | 1 | -0.0810845 | 121.6598175 | 208.9052182 |
| YPL138C | SPP1 | 0.88644408 | 1 | -0.0810196 | 112.8503599 | 194.5356168 |
| YOR381W | FRE3 | 0.88679315 | 1 | -0.0809927 | 125.3665749 | 214.9359779 |
| YPL123C | RNY1 | 0.8839759 | 1 | -0.0808499 | 134.6856073 | 230.1091924 |
| YNL035C | YNL035C | 0.8565312 | 1 | -0.0807345 | 122.2639497 | 209.8444217 |
| YML004C | GLO1 | 0.86680337 | 1 | -0.0807298 | 114.3787632 | 196.9894112 |
| YOR094W | ARF3 | 0.89303852 | 1 | -0.0806514 | 126.0978381 | 216.0835581 |
| YGL230C | YGL230C | 0.82089609 | 1 | -0.080619 | 126.3109133 | 216.4266979 |
| YEL037C | RAD23 | 0.80683099 | 1 | -0.0806077 | 117.7025109 | 202.3918429 |
| YPL249C | GYP5 | 0.89910418 | 1 | -0.0804239 | 132.3589866 | 226.2607892 |
| YOR171C | LCB4 | 0.89347888 | 1 | -0.0801826 | 123.4934592 | 211.7767709 |
| YEL006W | YEA6 | 0.86423024 | 1 | -0.0800391 | 128.5512787 | 220.0032836 |
| YOR044W | IRC23 | 0.88735809 | 1 | -0.0799273 | 134.8249576 | 230.2160241 |
| YPL113C | YPL113C | 0.87995049 | 1 | -0.0797417 | 116.2710617 | 199.9453483 |
| YGR017W | YGR017W | 0.82060606 | 1 | -0.0796661 | 126.8331417 | 217.1537395 |
| YOR242C | SSP2 | 0.90033819 | 1 | -0.079447 | 131.8007833 | 225.2233867 |
| YOL029C | YOL029C | 0.88684398 | 1 | -0.0793544 | 133.2525765 | 227.578022 |
| YMR205C | PFK2 | 0.82605089 | 1 | -0.0792994 | 96.20867699 | 167.1820517 |
| YNL205C | YNL205C | 0.8453486 | 1 | -0.0792448 | 105.5079423 | 182.3345432 |
| YOR028C | CIN5 | 0.88497138 | 1 | -0.0790345 | 135.764156 | 231.6306556 |
| YNR042W | YNR042W | 0.88000091 | 1 | -0.0788208 | 126.0568894 | 215.7780461 |
| YER181C | YER181C | 0.85964679 | 1 | -0.0788069 | 109.069835 | 188.0840095 |

| | | | | | | |
|---|---|---|---|---|---|---|
| YOR265W | RBL2 | 0.87243961 | 1 | -0.0785925 | 120.1921225 | 206.1875546 |
| YGL035C | MIG1 | 0.84588124 | 1 | -0.0784276 | 115.9092382 | 199.184107 |
| YOR314W | YOR314W | 0.89553756 | 1 | -0.0783565 | 122.9654879 | 210.6779011 |
| YNL115C | YNL115C | 0.88111938 | 1 | -0.0783316 | 114.9257436 | 197.568295 |
| YCL010C | SGF29 | 0.39100222 | 1 | -0.0782339 | 0 | 10.20413957 |
| YGR031W | YGR031W | 0.82708201 | 1 | -0.0781234 | 127.6223977 | 218.2391699 |
| YOR111W | YOR111W | 0.88199133 | 1 | -0.0780563 | 127.9714067 | 218.7993681 |
| YBR289W | SNF5 | 0.82376076 | 1 | -0.0780389 | 76.47167535 | 134.8424664 |
| YML032C | RAD52 | 0.58609784 | 1 | -0.078024 | 90.3085196 | 157.3972881 |
| YPL089C | RLM1 | 0.88796493 | 1 | -0.0780199 | 133.077666 | 227.1188131 |
| YNL291C | MID1 | 0.87783364 | 1 | -0.0777703 | 110.0810181 | 189.5972397 |
| YMR322C | SNO4 | 0.86288624 | 1 | -0.0773607 | 121.9921724 | 208.961322 |
| YEL016C | NPP2 | 0.82938443 | 1 | -0.0773062 | 111.3528653 | 191.6100661 |
| YNL176C | YNL176C | 0.87535283 | 1 | -0.0771589 | 132.8705624 | 226.6688985 |
| YER030W | CHZ1 | 0.83928233 | 1 | -0.0771361 | 126.4524187 | 216.2030954 |
| YJR137C | ECM17 | 0.85996416 | 1 | -0.0769294 | 110.0399423 | 189.4205985 |
| YGR032W | GSC2 | 0.81006931 | 1 | -0.0767954 | 121.3119849 | 207.7787441 |
| YOR105W | YOR105W | 0.88470766 | 1 | -0.0766097 | 127.1732152 | 217.309476 |
| YER088C | DOT6 | 0.86286285 | 1 | -0.0764393 | 121.0120312 | 207.2433144 |
| YGR092W | DBF2 | 0.71423416 | 1 | -0.0761073 | 99.69844097 | 172.4546958 |
| YOL099C | YOL099C | 0.88807082 | 1 | -0.0760891 | 135.1346348 | 230.2202457 |
| YOL085C | YOL085C | 0.88156807 | 1 | -0.0759794 | 127.7563446 | 218.1778828 |
| YGL208W | SIP2 | 0.81242988 | 1 | -0.0759161 | 112.8626647 | 193.8900188 |
| YKL065C | YET1 | 0.85237877 | 1 | -0.0758408 | 111.6517229 | 191.9061204 |
| YGL161C | YIP5 | 0.83669565 | 1 | -0.0756201 | 125.1823823 | 213.9349554 |
| YPL265W | DIP5 | 0.89437858 | 1 | -0.0755778 | 120.5289919 | 206.3435046 |
| YGL017W | ATE1 | 0.82111501 | 1 | -0.0754768 | 125.3213059 | 214.1427339 |
| YGL050W | TYW3 | 0.847791 | 1 | -0.0750995 | 129.8964745 | 221.5519462 |
| YPR115W | YPR115W | 0.89782146 | 1 | -0.0750835 | 142.279257 | 241.7362073 |
| YPL244C | HUT1 | 0.89305363 | 1 | -0.0750702 | 131.9104438 | 224.8312908 |
| YKL072W | STB6 | 0.87747135 | 1 | -0.0750639 | 124.2930998 | 212.4127099 |
| YOR189W | IES4 | 0.89987397 | 1 | -0.0750273 | 123.350906 | 210.8719709 |
| YNL224C | SQS1 | 0.89149867 | 1 | -0.0748135 | 113.2628288 | 194.3985491 |
| YPR117W | YPR117W | 0.90087286 | 1 | -0.0744931 | 120.9375616 | 206.8680663 |
| YOR386W | PHR1 | 0.90066555 | 1 | -0.0744883 | 122.2498414 | 209.0067175 |
| YNL326C | PFA3 | 0.87405941 | 1 | -0.0744762 | 100.0943529 | 172.8873704 |
| YLR425W | TUS1 | 0.83228712 | 1 | -0.0744751 | 98.49947302 | 170.2872583 |
| YDR461W | MFA1 | 0.89987199 | 1 | -0.0744152 | 152.4896848 | 258.2940382 |
| YMR194W | RPL36A | 0.85848392 | 1 | -0.0741125 | 101.1095539 | 174.4949053 |
| YML010C-B | YML010C-B | 0.87787541 | 1 | -0.074067 | 98.97423066 | 171.0079747 |
| YPL095C | EEB1 | 0.90844752 | 1 | -0.0740195 | 134.6616731 | 229.1792783 |
| YER166W | DNF1 | 0.841503 | 1 | -0.0736856 | 116.876807 | 200.1429287 |
| YGR043C | YGR043C | 0.82036796 | 1 | -0.0734373 | 124.8814166 | 213.1596215 |
| YER182W | FMP10 | 0.87362519 | 1 | -0.0733611 | 118.0328616 | 201.9851967 |
| YOR223W | YOR223W | 0.90624785 | 1 | -0.0732899 | 126.080761 | 215.0955573 |
| YKL204W | EAP1 | 0.85387247 | 1 | -0.0731418 | 106.7998854 | 183.6446516 |
| YGR110W | YGR110W | 0.83663725 | 1 | -0.0731099 | 122.1478455 | 208.6606636 |
| YNR039C | ZRG17 | 0.89589629 | 1 | -0.0730817 | 137.0500884 | 232.9505426 |
| YLL045C | RPL8B | 0.8336713 | 1 | -0.0730325 | 97.3628308 | 168.2461416 |
| YGR012W | YGR012W | 0.82550042 | 1 | -0.0730203 | 120.1653101 | 205.4170527 |
| YPL227C | ALG5 | 0.91875437 | 1 | -0.0727752 | 144.6756217 | 245.341684 |
| YGL262W | YGL262W | 0.8311641 | 1 | -0.0726372 | 129.9784395 | 221.3644007 |
| YER020W | GPA2 | 0.84461699 | 1 | -0.0725236 | 108.2684302 | 185.9580296 |
| YGL222C | EDC1 | 0.8408612 | 1 | -0.0725053 | 126.2925398 | 215.3384562 |
| YER119C | AVT6 | 0.85868097 | 1 | -0.0722161 | 120.2666388 | 205.4773427 |
| YOR245C | DGA1 | 0.90458544 | 1 | -0.0720457 | 123.6444432 | 210.9616028 |
| YJR150C | DAN1 | 0.88363407 | 1 | -0.0712717 | 102.4048485 | 176.2359636 |
| YOR093C | YOR093C | 0.89406486 | 1 | -0.0712309 | 132.0177081 | 224.5053855 |
| YPR154W | PIN3 | 0.90110894 | 1 | -0.0709174 | 119.3709677 | 203.8478287 |
| YMR312W | ELP6 | 0.84578507 | 1 | -0.0705057 | 87.59016844 | 151.9852263 |
| YPL260W | YPL260W | 0.90360325 | 1 | -0.0703863 | 124.7366707 | 212.5257064 |
| YPL092W | SSU1 | 0.90391483 | 1 | -0.0703254 | 139.2774702 | 236.222102 |
| YOL011W | PLB3 | 0.90238423 | 1 | -0.070139 | 119.1913393 | 203.4534815 |
| YFL006W | YFL006W | 0.86817093 | 1 | -0.0693966 | 122.0136368 | 207.9575429 |

| | | | | | | |
|---|---|---|---|---|---|---|
| YNL001W | DOM34 | 0.87064838 | 1 | -0.0692243 | 92.21755797 | 159.3616447 |
| YEL066W | HPA3 | 0.85963535 | 1 | -0.0689286 | 120.0529544 | 204.7002018 |
| YER106W | MAM1 | 0.8625741 | 1 | -0.0688051 | 114.4323756 | 195.5214556 |
| YOR239W | ABP140 | 0.91444532 | 1 | -0.0687312 | 133.093717 | 225.9334552 |
| YDR446W | ECM11 | 0.88604966 | 1 | -0.0686953 | 125.6675153 | 213.8226117 |
| YMR313C | TGL3 | 0.89009208 | 1 | -0.0686914 | 106.6720868 | 182.8558375 |
| YHL025W | SNF6 | 0.80129848 | 1 | -0.0686062 | 83.02932667 | 144.3024045 |
| YER091C-A | YER091C-A | 0.82026794 | 1 | -0.0684802 | 123.2470832 | 209.8487702 |
| YPL191C | YPL191C | 0.91119687 | 1 | -0.0682782 | 124.0979432 | 211.2094923 |
| YLR455W | YLR455W | 0.84555122 | 1 | -0.0678623 | 107.0652923 | 183.388701 |
| YMR080C | NAM7 | 0.88072068 | 1 | -0.0676679 | 113.1851101 | 193.3398397 |
| YER152C | YER152C | 0.87424594 | 1 | -0.0674863 | 122.2748311 | 208.1341822 |
| YER063W | THO1 | 0.8702077 | 1 | -0.0672637 | 122.7922488 | 208.9486308 |
| YNL246W | VPS75 | 0.87198667 | 1 | -0.067254 | 111.6230625 | 190.7394161 |
| YPL174C | NIP100 | 0.90180549 | 1 | -0.0672236 | 114.8119334 | 195.93393 |
| YOR170W | YOR170W | 0.91057018 | 1 | -0.0669755 | 122.9527077 | 209.17263 |
| YDR482C | CWC21 | 0.85008739 | 1 | -0.0669239 | 116.1460553 | 198.0697252 |
| YGL043W | DST1 | 0.84558751 | 1 | -0.0668866 | 118.1310461 | 201.300783 |
| YOL063C | CRT10 | 0.90163854 | 1 | -0.066625 | 115.4944439 | 196.9684796 |
| YGR071C | YGR071C | 0.8339075 | 1 | -0.0663526 | 111.5101426 | 190.4377595 |
| YOR115C | TRS33 | 0.91174766 | 1 | -0.066267 | 134.0768469 | 227.2147386 |
| YER128W | YER128W | 0.8686228 | 1 | -0.0660678 | 115.2740988 | 196.5366025 |
| YPL037C | EGD1 | 0.90440649 | 1 | -0.0656121 | 128.4266142 | 217.9183318 |
| YMR254C | YMR254C | 0.90181566 | 1 | -0.0653036 | 116.5015209 | 198.4378571 |
| YKR094C | RPL40B | 0.89290305 | 1 | -0.065294 | 105.7252272 | 180.8691488 |
| YPR106W | ISR1 | 0.91056727 | 1 | -0.0652408 | 111.0874707 | 189.6037108 |
| YMR123W | PKR1 | 0.87773676 | 1 | -0.0650729 | 99.6691163 | 170.9676653 |
| YFL054C | YFL054C | 0.86727807 | 1 | -0.0649396 | 122.573043 | 208.288156 |
| YDR501W | PLM2 | 0.87071111 | 1 | -0.0647806 | 126.8203194 | 215.1913033 |
| YOR088W | YOR088W | 0.91621952 | 1 | -0.0647697 | 115.5896738 | 196.8817349 |
| YGL165C | YGL165C | 0.84874558 | 1 | -0.0647328 | 116.9705248 | 199.1279758 |
| YOR317W | FAA1 | 0.91627089 | 1 | -0.0647232 | 127.6883727 | 216.598914 |
| YEL052W | AFG1 | 0.88333 | 1 | -0.0646896 | 122.0601717 | 207.4194669 |
| YMR275C | BUL1 | 0.88836354 | 1 | -0.0645341 | 117.5145181 | 199.988882 |
| YOR354C | MSC6 | 0.90564401 | 1 | -0.0644438 | 133.0523877 | 225.3068627 |
| YFL034C-A | RPL22B | 0.88075971 | 1 | -0.0643305 | 121.0720181 | 205.7617465 |
| YNL105W | YNL105W | 0.89145687 | 1 | -0.064115 | 103.2514788 | 176.6826685 |
| YOR328W | PDR10 | 0.90721824 | 1 | -0.0635083 | 137.2191757 | 231.9775203 |
| YOL002C | IZH2 | 0.90179719 | 1 | -0.0631955 | 130.3754076 | 220.7800476 |
| YNL141W | AAH1 | 0.88242087 | 1 | -0.0629862 | 84.34015009 | 145.7062835 |
| YFR022W | ROG3 | 0.85295839 | 1 | -0.0628163 | 125.0760383 | 212.0915842 |
| YGL007W | YGL007W | 0.753434 | 1 | -0.0627424 | 115.4788708 | 196.4366819 |
| YPL206C | YPL206C | 0.91685064 | 1 | -0.0626782 | 124.4437043 | 211.0427422 |
| YGR144W | THI4 | 0.87926847 | 1 | -0.0626487 | 133.166553 | 225.2588438 |
| YGR154C | GTO1 | 0.86964909 | 1 | -0.0626114 | 130.6357535 | 221.1282795 |
| YOR234C | RPL33B | 0.91192731 | 1 | -0.0624004 | 118.2728298 | 200.9467732 |
| YOL082W | ATG19 | 0.91473425 | 1 | -0.0623139 | 121.5852457 | 206.3353751 |
| YPL222W | FMP40 | 0.90839349 | 1 | -0.0619576 | 127.8342462 | 216.4759924 |
| YOR007C | SGT2 | 0.90692367 | 1 | -0.0619184 | 128.2568444 | 217.1598065 |
| YGR008C | STF2 | 0.85141418 | 1 | -0.0618204 | 126.4017682 | 214.1228764 |
| YPL192C | PRM3 | 0.92336692 | 1 | -0.061511 | 148.1341622 | 249.510578 |
| YGR149W | YGR149W | 0.82803784 | 1 | -0.0615048 | 127.3724986 | 215.6642044 |
| YPL236C | YPL236C | 0.93004017 | 1 | -0.0612692 | 145.8856456 | 245.8135206 |
| YGR018C | YGR018C | 0.87372641 | 1 | -0.061174 | 131.6352965 | 222.57024 |
| YKL038W | RGT1 | 0.87594289 | 1 | -0.0608089 | 111.7814937 | 190.1570423 |
| YPL061W | ALD6 | 0.9147482 | 1 | -0.0606895 | 117.4916713 | 199.4501772 |
| YGL131C | SNT2 | 0.87356869 | 1 | -0.0606823 | 133.3736215 | 225.3399263 |
| YOR058C | ASE1 | 0.92350762 | 1 | -0.0605701 | 128.0581738 | 216.6600742 |
| YNL077W | APJ1 | 0.8855332 | 1 | -0.0603611 | 114.955126 | 195.272274 |
| YKL053W | YKL053W | 0.9007036 | 1 | -0.0602554 | 106.0297917 | 180.7084589 |
| YGR025W | YGR025W | 0.85232708 | 1 | -0.0599621 | 123.2221544 | 208.6971107 |
| YOL014W | YOL014W | 0.92164738 | 1 | -0.0599404 | 126.1360445 | 213.4444849 |
| YLR235C | YLR235C | 0.9220203 | 1 | -0.059801 | 86.39034433 | 148.6330504 |
| YDR503C | LPP1 | 0.89027217 | 1 | -0.0596538 | 118.401562 | 200.7983905 |

| | | | | | | |
|---|---|---|---|---|---|---|
| YPR084W | YPR084W | 0.91863708 | 1 | -0.0593659 | 118.7959503 | 201.4037701 |
| YPR152C | URN1 | 0.91656133 | 1 | -0.0592174 | 117.3640643 | 199.0501516 |
| YOL058W | ARG1 | 0.91959755 | 1 | -0.0590377 | 123.6886379 | 209.3369994 |
| YKL009W | MRT4 | 0.81996081 | 1 | -0.0588373 | 81.14337597 | 139.9537669 |
| YOL060C | MAM3 | 0.91982118 | 1 | -0.0586281 | 122.6389055 | 207.5723022 |
| YPL119C | DBP1 | 0.92553175 | 1 | -0.0585178 | 129.6989825 | 219.0672199 |
| YDR424C | DYN2 | 0.88518911 | 1 | -0.0582898 | 121.1837763 | 205.1560344 |
| YML103C | NUP188 | 0.87531526 | 1 | -0.0582774 | 95.82677676 | 163.8175546 |
| YGL214W | YGL214W | 0.87236327 | 1 | -0.0582717 | 117.3328068 | 198.8758366 |
| YMR294W | JNM1 | 0.88478974 | 1 | -0.0580949 | 109.3440009 | 185.8294666 |
| YAL024C | LTE1 | 0.91588122 | 1 | -0.0580679 | 113.0724443 | 191.9040307 |
| YOR374W | ALD4 | 0.92616355 | 1 | -0.0580236 | 124.5099235 | 210.5435849 |
| YFR008W | FAR7 | 0.89143342 | 1 | -0.0577708 | 121.7115056 | 205.9486485 |
| YNL231C | PDR16 | 0.90309003 | 1 | -0.05773 | 103.4301908 | 176.1412099 |
| YNL242W | ATG2 | 0.90399307 | 1 | -0.057636 | 104.7891747 | 178.3443504 |
| YOR346W | REV1 | 0.91657522 | 1 | -0.0574638 | 131.5047122 | 221.8734446 |
| YER144C | UBP5 | 0.88272083 | 1 | -0.0572377 | 119.2294301 | 201.8328491 |
| YFR032C | YFR032C | 0.8805711 | 1 | -0.0572123 | 136.0358425 | 229.2272632 |
| YOR286W | FMP31 | 0.92131612 | 1 | -0.0570351 | 116.2358591 | 196.9263133 |
| YPL274W | SAM3 | 0.92191382 | 1 | -0.0569416 | 123.4282759 | 208.6391626 |
| YOL090W | MSH2 | 0.89979716 | 1 | -0.0568598 | 107.8406089 | 183.2175549 |
| YGL253W | HXK2 | 0.85455914 | 1 | -0.0568495 | 109.8963378 | 186.5674437 |
| YMR170C | ALD2 | 0.9118114 | 1 | -0.0563399 | 114.5837124 | 194.1423083 |
| YGL157W | YGL157W | 0.87585106 | 1 | -0.0562891 | 131.0060075 | 220.9072388 |
| YDR458C | HEH2 | 0.89265232 | 1 | -0.0561835 | 122.1544042 | 206.4636184 |
| YDR466W | PKH3 | 0.8923314 | 1 | -0.056096 | 121.4140458 | 205.2452822 |
| YFR046C | CNN1 | 0.88019043 | 1 | -0.0558725 | 133.4317901 | 224.8073949 |
| YKL129C | MYO3 | 0.88614419 | 1 | -0.0556784 | 115.0142442 | 194.7578818 |
| YOL088C | MPD2 | 0.90088855 | 1 | -0.0556291 | 111.2502003 | 188.6153338 |
| YGR182C | YGR182C | 0.92232913 | 1 | -0.0554498 | 125.244498 | 211.4053747 |
| YJR111C | YJR111C | 0.89630302 | 1 | -0.0554096 | 117.1834989 | 198.2591286 |
| YOR306C | MCH5 | 0.88174791 | 1 | -0.0551657 | 92.33035947 | 157.7118522 |
| YJR115W | YJR115W | 0.89571812 | 1 | -0.0550581 | 114.0848391 | 193.1618701 |
| YER073W | ALD5 | 0.91246402 | 1 | -0.0547706 | 131.7436002 | 221.9115963 |
| YNL180C | RHO5 | 0.93925587 | 1 | -0.0547592 | 86.17042907 | 147.6169439 |
| YPL019C | VTC3 | 0.93320616 | 1 | -0.0547353 | 135.4880853 | 228.0112417 |
| YKR053C | YSR3 | 0.91961269 | 1 | -0.0547271 | 118.9581039 | 201.0630629 |
| YFL015C | YFL015C | 0.89263441 | 1 | -0.054717 | 119.7145206 | 202.2948568 |
| YFL012W | YFL012W | 0.92375645 | 1 | -0.0545877 | 128.4069047 | 216.4482819 |
| YNL065W | AQR1 | 0.90307987 | 1 | -0.0539338 | 115.7697688 | 195.7619891 |
| YKR102W | FLO10 | 0.89480836 | 1 | -0.0538853 | 113.775688 | 192.5049267 |
| YGL014W | PUF4 | 0.85718256 | 1 | -0.053692 | 120.7409636 | 203.834464 |
| YGL005C | COG7 | 0.88287016 | 1 | -0.0535188 | 132.2581055 | 222.5870708 |
| YPL182C | YPL182C | 0.93288267 | 1 | -0.0534212 | 128.6010928 | 216.6126964 |
| YNR045W | PET494 | 0.91401553 | 1 | -0.0533643 | 120.1502782 | 202.8287927 |
| YGR142W | BTN2 | 0.88861618 | 1 | -0.0532987 | 139.3207963 | 234.0719298 |
| YNL057W | YNL057W | 0.8999735 | 1 | -0.0531667 | 115.4375144 | 195.12029 |
| YOR351C | MEK1 | 0.93320582 | 1 | -0.0530693 | 128.8162191 | 216.9174873 |
| YER067C-A | YER067C-A | 0.88355984 | 1 | -0.0528876 | 116.1967095 | 196.3215267 |
| YGR034W | RPL26B | 0.87100136 | 1 | -0.052741 | 108.2655609 | 183.3730834 |
| YMR067C | UBX4 | 0.87184842 | 1 | -0.0526385 | 95.98449565 | 163.339177 |
| YPL171C | OYE3 | 0.93381128 | 1 | -0.0525753 | 133.5067559 | 224.4995461 |
| YOR031W | CRS5 | 0.9162736 | 1 | -0.0523564 | 123.9766994 | 208.9351486 |
| YFR057W | YFR057W | 0.88818574 | 1 | -0.0523479 | 135.7303738 | 228.0948209 |
| YER151C | UBP3 | 0.77718939 | 1 | -0.0523047 | 86.29620454 | 147.5018392 |
| YFL051C | YFL051C | 0.89916998 | 1 | -0.0521675 | 121.1705944 | 204.3360032 |
| YOR291W | YOR291W | 0.92396144 | 1 | -0.0520159 | 132.0786144 | 222.0984323 |
| YLR233C | EST1 | 0.75952043 | 1 | -0.0519182 | 116.7180655 | 197.0450012 |
| YNR022C | MRPL50 | 0.91520085 | 1 | -0.0519061 | 125.668971 | 211.6351518 |
| YOR231W | MKK1 | 0.94685285 | 1 | -0.0514677 | 149.6934758 | 250.7425969 |
| YNL095C | YNL095C | 0.91770909 | 1 | -0.051396 | 106.5003283 | 180.3199856 |
| YNL058C | YNL058C | 0.91245678 | 1 | -0.0506229 | 99.71015886 | 169.1498369 |
| YOR072W | YOR072W | 0.91829529 | 1 | -0.0505049 | 131.3254138 | 220.6734882 |
| YOR222W | ODC2 | 0.92915324 | 1 | -0.0504634 | 132.7250941 | 222.9498269 |

| | | | | | | |
|---|---|---|---|---|---|---|
| YOR320C | GNT1 | 0.93340131 | 1 | -0.0502888 | 124.4820833 | 209.4893401 |
| YKR047W | YKR047W | 0.89796427 | 1 | -0.0502266 | 110.8271297 | 187.2209921 |
| YDR514C | YDR514C | 0.9113551 | 1 | -0.0499974 | 118.8583051 | 200.2834699 |
| YPL198W | RPL7B | 0.92770509 | 1 | -0.049689 | 126.6366133 | 212.9234187 |
| YNR030W | ALG12 | 0.92876927 | 1 | -0.0495819 | 118.3487116 | 199.3985473 |
| YNR015W | SMM1 | 0.90359861 | 1 | -0.0489961 | 111.9865865 | 188.9506349 |
| YGL136C | MRM2 | 0.87943549 | 1 | -0.0488028 | 121.31215 | 204.1279093 |
| YDR080W | VPS41 | 0.89042634 | 1 | -0.0486246 | 76.21975519 | 130.595251 |
| YNL215W | IES2 | 0.90871479 | 1 | -0.0485702 | 92.56969776 | 157.2417535 |
| YGL028C | SCW11 | 0.88561837 | 1 | -0.0485328 | 133.6310866 | 224.1749673 |
| YNL334C | SNO2 | 0.92361058 | 1 | -0.048389 | 105.8968613 | 178.9440091 |
| YOR021C | YOR021C | 0.92958803 | 1 | -0.0483362 | 133.1382913 | 223.3459753 |
| YNL154C | YCK2 | 0.91502856 | 1 | -0.0481553 | 96.63626129 | 163.8169344 |
| YFL010C | WWM1 | 0.93355059 | 1 | -0.0481333 | 148.6011185 | 248.5269418 |
| YEL067C | YEL067C | 0.90136322 | 1 | -0.0480882 | 118.989141 | 200.2477395 |
| YFL048C | EMP47 | 0.89230352 | 1 | -0.0480752 | 116.6839241 | 196.4880974 |
| YNR032W | PPG1 | 0.9279805 | 1 | -0.0476268 | 130.1299968 | 218.3493315 |
| YGR260W | TNA1 | 0.83004388 | 1 | -0.0474922 | 142.8136943 | 239.008687 |
| YPL137C | GIP3 | 0.93904547 | 1 | -0.0472198 | 126.1955987 | 211.8824159 |
| YGR130C | YGR130C | 0.87778546 | 1 | -0.0470957 | 132.7740192 | 222.5903429 |
| YPL144W | POC4 | 0.92611933 | 1 | -0.046988 | 122.8175243 | 206.345255 |
| YDR474C | YDR474C | 0.91410126 | 1 | -0.0468764 | 126.0620452 | 211.6199027 |
| YDL074C | BRE1 | 0.84603697 | 1 | -0.0468098 | 103.3016528 | 174.5073329 |
| YDR540C | IRC4 | 0.89389404 | 1 | -0.0467701 | 112.1756763 | 188.9685512 |
| YOR138C | RUP1 | 0.93334565 | 1 | -0.046698 | 139.2251859 | 233.0551285 |
| YEL056W | HAT2 | 0.91082339 | 1 | -0.0463986 | 120.4585723 | 202.4228265 |
| YGL250W | YGL250W | 0.88783762 | 1 | -0.046291 | 131.9735491 | 221.1804535 |
| YOL045W | PSK2 | 0.937165 | 1 | -0.0462525 | 122.0765144 | 205.0413371 |
| YOR067C | ALG8 | 0.91893474 | 1 | -0.0462242 | 116.9160548 | 196.6250896 |
| YGL162W | SUT1 | 0.89406656 | 1 | -0.0462234 | 114.020525 | 191.9047079 |
| YNL278W | CAF120 | 0.92477205 | 1 | -0.046193 | 104.4008259 | 176.2187526 |
| YGL194C | HOS2 | 0.89786877 | 1 | -0.0460268 | 120.12407 | 201.8290337 |
| YGR121C | MEP1 | 0.88214531 | 1 | -0.0455011 | 123.2402147 | 206.8403821 |
| YKR010C | TOF2 | 0.90389608 | 1 | -0.0454943 | 104.6985414 | 176.6129455 |
| YJL029C | VPS53 | 0.92652855 | 1 | -0.0454844 | 101.0930575 | 170.7340114 |
| YMR202W | ERG2 | 0.81273208 | 1 | -0.0452444 | 14.58727345 | 29.68138782 |
| YDL033C | SLM3 | 0.8334894 | 1 | -0.0451661 | 14.93749317 | 30.24209359 |
| YPR065W | ROX1 | 0.93969542 | 1 | -0.0448918 | 125.8799006 | 211.06412 |
| YOR297C | TIM18 | 0.9203754 | 1 | -0.0448799 | 113.5268243 | 190.9246333 |
| YPL074W | YTA6 | 0.93576644 | 1 | -0.0445021 | 136.5215622 | 228.361273 |
| YMR099C | YMR099C | 0.9109943 | 1 | -0.0443859 | 102.6770288 | 173.1729189 |
| YOR141C | ARP8 | 0.9235864 | 1 | -0.0442363 | 93.41800379 | 158.0593943 |
| YNL120C | YNL120C | 0.91538121 | 1 | -0.044225 | 91.45062071 | 154.8506962 |
| YNL097C | PHO23 | 0.92154531 | 1 | -0.0441766 | 116.8707546 | 196.2841631 |
| YLR190W | MMR1 | 0.90302452 | 1 | -0.0438526 | 84.6616173 | 143.7347256 |
| YGL231C | YGL231C | 0.87508053 | 1 | -0.0436761 | 113.3691446 | 190.5105717 |
| YJL092W | HPR5 | 0.93918003 | 1 | -0.0436618 | 119.1913665 | 200.0000709 |
| YOR213C | SAS5 | 0.93688367 | 1 | -0.0434891 | 139.1014142 | 232.4348147 |
| YML102W | CAC2 | 0.88974523 | 1 | -0.0434341 | 94.39062466 | 159.5403138 |
| YPL080C | YPL080C | 0.9295518 | 1 | -0.0432841 | 103.380274 | 174.1756345 |
| YGR040W | KSS1 | 0.91109859 | 1 | -0.0430865 | 116.1257894 | 194.9275473 |
| YEL033W | MTC7 | 0.89167005 | 1 | -0.0429674 | 96.93181144 | 163.6220779 |
| YNR021W | YNR021W | 0.93464872 | 1 | -0.0428017 | 130.6610645 | 218.5857306 |
| YMR253C | YMR253C | 0.92094835 | 1 | -0.042582 | 115.8342018 | 194.3863962 |
| YOR375C | GDH1 | 0.94004123 | 1 | -0.042473 | 132.6937747 | 221.856577 |
| YGL057C | YGL057C | 0.89106741 | 1 | -0.0424652 | 124.5345192 | 208.554383 |
| YNL030W | HHF2 | 0.91128387 | 1 | -0.0424497 | 102.9283921 | 173.3301531 |
| YPL121C | MEI5 | 0.94073793 | 1 | -0.0424437 | 129.3199741 | 216.3527995 |
| YOR255W | OSW1 | 0.93753917 | 1 | -0.0422525 | 137.2314909 | 229.2251791 |
| YDR036C | EHD3 | 0.94387529 | 1 | -0.0420353 | 124.5922887 | 208.5924863 |
| YDR515W | SLF1 | 0.94059388 | 1 | -0.0420183 | 116.2803817 | 195.0402361 |
| YKL156W | RPS27A | 0.93085393 | 1 | -0.0418356 | 106.0379165 | 178.3191821 |
| YDR496C | PUF6 | 0.78764353 | 1 | -0.0413577 | 99.72370928 | 167.9634593 |
| YOL041C | NOP12 | 0.90749744 | 1 | -0.0412707 | 86.62957421 | 146.6061204 |

| | | | | | | |
|---|---|---|---|---|---|---|
| YPL087W | YDC1 | 0.93403651 | 1 | -0.0411785 | 120.4486217 | 201.725741 |
| YGR077C | PEX8 | 0.92028485 | 1 | -0.0409958 | 113.5646662 | 190.4797197 |
| YOR128C | ADE2 | 0.93715795 | 1 | -0.0407914 | 115.3880938 | 193.4256054 |
| YPL038W | MET31 | 0.94749313 | 1 | -0.0405719 | 128.2664345 | 214.3911814 |
| YML041C | VPS71 | 0.92874432 | 1 | -0.0400917 | 102.6326717 | 172.5405083 |
| YER158C | YER158C | 0.91866379 | 1 | -0.0398661 | 115.6565144 | 193.7424983 |
| YEL041W | YEF1 | 0.92308768 | 1 | -0.0391264 | 123.9992049 | 207.246228 |
| YOR344C | TYE7 | 0.94731646 | 1 | -0.0390536 | 117.273633 | 196.2727435 |
| YKL198C | PTK1 | 0.81943975 | 1 | -0.0390135 | 130.6890095 | 218.1371931 |
| YPL062W | YPL062W | 0.94517265 | 1 | -0.0387355 | 115.8446784 | 193.9017764 |
| YDR425W | SNX41 | 0.91843379 | 1 | -0.0384708 | 117.3102768 | 196.2564593 |
| YPL225W | YPL225W | 0.9508808 | 1 | -0.0383332 | 117.1460328 | 195.9707621 |
| YER039C | HVG1 | 0.92081053 | 1 | -0.0382828 | 119.8795483 | 200.4203562 |
| YCR053W | THR4 | 0.8596321 | 1 | -0.0378569 | 19.29658067 | 36.3949173 |
| YOR357C | SNX3 | 0.95075193 | 1 | -0.0374571 | 141.7487249 | 235.9636819 |
| YEL043W | YEL043W | 0.91449461 | 1 | -0.0372407 | 123.5255044 | 206.2280563 |
| YMR165C | PAH1 | 0.93859911 | 1 | -0.0369101 | 35.54087832 | 62.75280377 |
| YOL132W | GAS4 | 0.94990056 | 1 | -0.0366705 | 121.470501 | 202.8036228 |
| YPL230W | YPL230W | 0.95232622 | 1 | -0.0365924 | 127.5054976 | 212.6316602 |
| YDR520C | YDR520C | 0.93405974 | 1 | -0.0363597 | 120.2587552 | 200.7877057 |
| YEL060C | PRB1 | 0.92143932 | 1 | -0.0363449 | 117.6657211 | 196.5586222 |
| YJL079C | PRY1 | 0.94537356 | 1 | -0.0362458 | 128.7690596 | 214.6463031 |
| YOL073C | YOL073C | 0.95088022 | 1 | -0.0360932 | 119.5574915 | 199.6097447 |
| YOR152C | YOR152C | 0.94768303 | 1 | -0.035707 | 132.2089773 | 220.1837642 |
| YFL004W | VTC2 | 0.95573802 | 1 | -0.0355999 | 135.2997285 | 225.2083249 |
| YOR313C | SPS4 | 0.94704013 | 1 | -0.0354585 | 132.6153521 | 220.8138274 |
| YOL138C | RTC1 | 0.94584193 | 1 | -0.0353927 | 111.5177646 | 186.4120543 |
| YER028C | MIG3 | 0.93167816 | 1 | -0.035258 | 127.9797341 | 213.2307059 |
| YER087C-A | YER087C-A | 0.94465438 | 1 | -0.0350878 | 128.7914792 | 214.5318126 |
| YNL259C | ATX1 | 0.93921985 | 1 | -0.0350072 | 122.4900325 | 204.2487162 |
| YOR271C | FSF1 | 0.95114686 | 1 | -0.0349333 | 120.3069511 | 200.6802274 |
| YER065C | ICL1 | 0.92355443 | 1 | -0.0348726 | 116.4234347 | 194.3414179 |
| YML111W | BUL2 | 0.39100222 | 1 | -0.0348477 | 0 | 4.545219816 |
| YKR082W | NUP133 | 0.9291643 | 1 | -0.034786 | 84.61193308 | 142.4711549 |
| YOR139C | YOR139C | 0.73227367 | 1 | -0.0347355 | 5.855712125 | 14.07654881 |
| YGL197W | MDS3 | 0.90851803 | 1 | -0.0346254 | 125.4995276 | 209.1049844 |
| YKR029C | SET3 | 0.92753515 | 1 | -0.0343485 | 107.214633 | 179.2609067 |
| YPL127C | HHO1 | 0.95620097 | 1 | -0.0342849 | 129.8346387 | 216.1276448 |
| YOL083W | YOL083W | 0.95321649 | 1 | -0.034097 | 119.8054021 | 199.753519 |
| YER060W | FCY21 | 0.93016169 | 1 | -0.0335561 | 118.8097564 | 198.0598812 |
| YNL292W | PUS4 | 0.94050876 | 1 | -0.0332795 | 124.2454307 | 206.8850158 |
| YDR455C | :::GUK1 | 0.94189353 | 1 | -0.0331988 | 102.1518026 | 170.8575468 |
| YIL007C | NAS2 | 0.93760737 | 1 | -0.0331015 | 108.0239218 | 180.4175701 |
| YML106W | URA5 | 0.94478682 | 1 | -0.0330667 | 117.9284635 | 196.5593669 |
| YJL115W | ASF1 | 0.95117175 | 1 | -0.0329436 | 59.5978949 | 101.4530798 |
| YKL093W | MBR1 | 0.93244307 | 1 | -0.0328448 | 112.7316795 | 188.0586466 |
| YHR097C | YHR097C | 0.90557429 | 1 | -0.0326845 | 146.1527564 | 242.5206302 |
| YGL085W | YGL085W | 0.92533022 | 1 | -0.0326445 | 114.6095734 | 191.0938576 |
| YNR051C | BRE5 | 0.95158772 | 1 | -0.0320977 | 111.0822731 | 185.2723483 |
| YKL074C | MUD2 | 0.93620714 | 1 | -0.0317164 | 108.6691409 | 181.2887338 |
| YOR289W | YOR289W | 0.96059952 | 1 | -0.0316022 | 135.1325125 | 224.4143057 |
| YGL133W | ITC1 | 0.92446238 | 1 | -0.0315192 | 115.5676777 | 192.5089831 |
| YOR311C | HSD1 | 0.95892353 | 1 | -0.0314216 | 125.3624119 | 208.4635808 |
| YGL255W | ZRT1 | 0.93647797 | 1 | -0.0313549 | 127.3788158 | 211.7420121 |
| YBR223C | TDP1 | 0.91907151 | 1 | -0.0312617 | 151.7746499 | 251.4998285 |
| YER027C | GAL83 | 0.92202433 | 1 | -0.031109 | 114.9650448 | 191.4730649 |
| YKR035C | YKR035C | 0.93515144 | 1 | -0.0307872 | 108.2043969 | 180.4099189 |
| YNL200C | YNL200C | 0.94550792 | 1 | -0.0306756 | 125.6736803 | 208.8737048 |
| YOR307C | SLY41 | 0.95827422 | 1 | -0.0302509 | 137.4838942 | 228.0712736 |
| YPR040W | TIP41 | 0.95844809 | 1 | -0.0301705 | 123.9790878 | 206.0453022 |
| YER097W | YER097W | 0.93873694 | 1 | -0.0300535 | 122.7139281 | 203.9675859 |
| YGR028W | MSP1 | 0.94338789 | 1 | -0.03002 | 127.1342357 | 211.1691883 |
| YNL201C | PSY2 | 0.94433726 | 1 | -0.0298194 | 118.523162 | 197.1052931 |
| YDR444W | YDR444W | 0.96980722 | 1 | -0.029765 | 96.80449058 | 161.6925197 |

| | | | | | | |
|---|---|---|---|---|---|---|
| YKL146W | AVT3 | 0.90582277 | 1 | -0.0297545 | 132.6573321 | 220.138277 |
| YMR176W | ECM5 | 0.86769898 | 1 | -0.0297523 | 127.7059156 | 212.0662179 |
| YNR010W | CSE2 | 0.7087244 | 1 | -0.0297013 | 4.509410132 | 11.22519173 |
| YDR447C | RPS17B | 0.92549092 | 1 | -0.0295973 | 103.5851289 | 172.7244077 |
| YNL053W | MSG5 | 0.9610396 | 1 | -0.0295796 | 123.2315537 | 204.7496145 |
| YFR049W | YMR31 | 0.92476715 | 1 | -0.0292703 | 125.2958122 | 208.0744104 |
| YGL034C | YGL034C | 0.94121367 | 1 | -0.0291307 | 135.9656489 | 225.4501229 |
| YLR452C | SST2 | 0.94669059 | 1 | -0.0289788 | 106.085691 | 176.7201417 |
| YMR158W-A | YMR158W-A | 0.93902274 | 1 | -0.0288492 | 101.4057031 | 169.0739394 |
| YAL013W | DEP1 | 0.95786828 | 1 | -0.0287714 | 118.1921073 | 196.4289116 |
| YDR535C | YDR535C | 0.94173105 | 1 | -0.0285126 | 124.9156378 | 207.355822 |
| YPR171W | BSP1 | 0.95592168 | 1 | -0.0283488 | 129.9760394 | 215.5839102 |
| YMR135W-A | YMR135W-A | 0.9481643 | 1 | -0.0281068 | 82.12302448 | 137.5425816 |
| YGL254W | FZF1 | 0.94139212 | 1 | -0.0279492 | 121.9535558 | 202.4535626 |
| YDR483W | KRE2 | 0.9268922 | 1 | -0.0278581 | 121.1530579 | 201.1367165 |
| YGR200C | ELP2 | 0.89292977 | 1 | -0.0275056 | 113.7109752 | 188.9586984 |
| YHL031C | GOS1 | 0.91052284 | 1 | -0.027448 | 151.0244733 | 249.7794726 |
| YER056C-A | RPL34A | 0.93552201 | 1 | -0.0269313 | 124.6369348 | 206.6952387 |
| YMR278W | YMR278W | 0.95231634 | 1 | -0.0269048 | 120.252379 | 199.5440955 |
| YOR230W | WTM1 | 0.96211496 | 1 | -0.0268975 | 135.7451851 | 224.7994507 |
| YKL005C | BYE1 | 0.95410087 | 1 | -0.0268195 | 123.4856041 | 204.8037594 |
| YKL105C | YKL105C | 0.94450022 | 1 | -0.0266393 | 114.9759375 | 190.9078396 |
| YPL207W | TYW1 | 0.96615402 | 1 | -0.0264379 | 132.5542923 | 219.5377203 |
| YHR077C | NMD2 | 0.87153581 | 1 | -0.0262453 | 129.0505833 | 213.8008688 |
| YPL196W | OXR1 | 0.96298866 | 1 | -0.0262302 | 138.6614375 | 229.4664676 |
| YEL007W | YEL007W | 0.9511112 | 1 | -0.0256072 | 84.08073477 | 140.408002 |
| YOR303W | CPA1 | 0.96860897 | 1 | -0.0254817 | 134.0201897 | 221.8027003 |
| YOL112W | MSB4 | 0.95818735 | 1 | -0.024932 | 121.4324445 | 201.2105166 |
| YNL190W | YNL190W | 0.95493436 | 1 | -0.024898 | 93.49148607 | 155.6568658 |
| YGL077C | HNM1 | 0.9414206 | 1 | -0.0247 | 128.7871159 | 213.1698098 |
| YGR041W | BUD9 | 0.94734882 | 1 | -0.0245885 | 131.0524865 | 216.8482644 |
| YOR090C | PTC5 | 0.96533418 | 1 | -0.0244903 | 118.1152497 | 195.745229 |
| YDR513W | GRX2 | 0.95296618 | 1 | -0.0244504 | 122.933605 | 203.5948847 |
| YKR035W-A | DID2 | 0.95158471 | 1 | -0.0242655 | 113.2728895 | 187.8219129 |
| YGR079W | YGR079W | 0.93180045 | 1 | -0.0242278 | 106.8983712 | 177.42529 |
| YGL170C | SPO74 | 0.9471767 | 1 | -0.0238028 | 133.5528003 | 220.8217812 |
| YGR254W | ENO1 | 0.96883079 | 1 | -0.0237637 | 124.8576349 | 206.6418634 |
| YOR126C | IAH1 | 0.9630939 | 1 | -0.0234611 | 127.6098978 | 211.0891245 |
| YGL221C | NIF3 | 0.94639454 | 1 | -0.023423 | 110.6913467 | 183.5036124 |
| YOR018W | ROD1 | 0.96429139 | 1 | -0.0233538 | 120.1029234 | 198.8372931 |
| YGL016W | KAP122 | 0.94512584 | 1 | -0.0233378 | 112.4511148 | 186.3612627 |
| YOR182C | RPS30B | 0.95438251 | 1 | -0.0232478 | 83.56261098 | 139.255618 |
| YIR004W | DJP1 | 0.96870405 | 1 | -0.023227 | 117.6718855 | 194.8576862 |
| YJL106W | IME2 | 0.96794581 | 1 | -0.0231006 | 120.9540629 | 200.1917949 |
| YGL215W | CLG1 | 0.9448142 | 1 | -0.0229152 | 135.619021 | 224.0743608 |
| YOR042W | CUE5 | 0.96548571 | 1 | -0.0227865 | 127.2437652 | 210.4042663 |
| YPL269W | KAR9 | 0.96970845 | 1 | -0.0226099 | 122.9998227 | 203.4627785 |
| YFL023W | BUD27 | 0.92415498 | 1 | -0.0225144 | 105.9639957 | 175.6785999 |
| YPL103C | FMP30 | 0.97212039 | 1 | -0.0221738 | 131.6893705 | 217.5715588 |
| YMR186W | HSC82 | 0.95899317 | 1 | -0.0221305 | 102.5709194 | 170.0971403 |
| YGL059W | YGL059W | 0.95886111 | 1 | -0.0220126 | 149.1987873 | 246.0942959 |
| YPR121W | THI22 | 0.97267828 | 1 | -0.0219156 | 135.7889314 | 224.2209662 |
| YOR266W | PNT1 | 0.9729502 | 1 | -0.0219061 | 133.9583466 | 221.2355191 |
| YOL158C | ENB1 | 0.9670808 | 1 | -0.0218509 | 131.6547558 | 217.4730107 |
| YGL226W | MTC3 | 0.95068425 | 1 | -0.0217968 | 117.9787892 | 195.1714625 |
| YER123W | YCK3 | 0.95200032 | 1 | -0.0216192 | 111.3711735 | 184.3765893 |
| YGR001C | YGR001C | 0.9541425 | 1 | -0.0216164 | 137.5662966 | 227.0793908 |
| YHR092C | HXT4 | 0.94307763 | 1 | -0.0214648 | 151.3275835 | 249.4932003 |
| YMR143W | RPS16A | 0.95166789 | 1 | -0.0214437 | 72.65253924 | 121.2347645 |
| YMR015C | ERG5 | 0.95462858 | 1 | -0.0213791 | 99.67185607 | 165.2731047 |
| YOL098C | YOL098C | 0.9715613 | 1 | -0.0208541 | 123.4380478 | 203.9481559 |
| YLL029W | YLL029W | 0.96902481 | 1 | -0.0207318 | 134.3758973 | 221.7630373 |
| YGR027C | RPS25A | 0.92422603 | 1 | -0.0206566 | 104.0949957 | 172.3894386 |
| YNL167C | SKO1 | 0.95745229 | 1 | -0.0205347 | 115.6399143 | 191.1940229 |

| | | | | | | |
|---|---|---|---|---|---|---|
| YPL159C | PET20 | 0.95706182 | 1 | -0.020388 | 104.9964975 | 173.8240383 |
| YKR034W | DAL80 | 0.95889005 | 1 | -0.0201157 | 110.4162932 | 182.6238505 |
| YNL034W | YNL034W | 0.96903626 | 1 | -0.0200934 | 109.4150694 | 180.9887494 |
| YGR189C | CRH1 | 0.94660705 | 1 | -0.0199591 | 147.2370836 | 242.6284969 |
| YPL185W | YPL185W | 0.97148471 | 1 | -0.0195789 | 133.4989345 | 220.1830392 |
| YOR366W | YOR366W | 0.97211784 | 1 | -0.0193599 | 137.8246146 | 227.2061895 |
| YOL035C | YOL035C | 0.97335037 | 1 | -0.0193574 | 120.594942 | 199.1181219 |
| YPL046C | ELC1 | 0.97417117 | 1 | -0.0192363 | 120.4721603 | 198.9021646 |
| YPR158W | YPR158W | 0.9702652 | 1 | -0.0190303 | 131.6432081 | 217.086287 |
| YDR471W | RPL27B | 0.95156293 | 1 | -0.0189448 | 104.6043558 | 172.9965331 |
| YGR070W | ROM1 | 0.95482525 | 1 | -0.0188987 | 131.4315126 | 216.7240219 |
| YOR321W | PMT3 | 0.97340052 | 1 | -0.0183859 | 140.8899855 | 232.0763011 |
| YGR235C | YGR235C | 0.96467913 | 1 | -0.0181859 | 133.224847 | 219.5545395 |
| YOR384W | FRE5 | 0.97958494 | 1 | -0.0180848 | 144.1282869 | 237.3160829 |
| YFR026C | YFR026C | 0.95634629 | 1 | -0.0180802 | 132.7545259 | 218.7740294 |
| YAL065C | YAL065C | 0.9819244 | 1 | -0.0179535 | 116.7713806 | 192.7018587 |
| YGL094C | PAN2 | 0.97427823 | 1 | -0.0179317 | 138.5522292 | 228.2060607 |
| YPR132W | RPS23B | 0.97507203 | 1 | -0.0179282 | 117.7600411 | 194.3102647 |
| YMR244W | YMR244W | 0.97487672 | 1 | -0.0171779 | 118.8025591 | 195.9119217 |
| YPL140C | MKK2 | 0.97477001 | 1 | -0.0171117 | 135.4176653 | 222.9891473 |
| YGL036W | YGL036W | 0.96338796 | 1 | -0.0168263 | 116.1034315 | 191.465955 |
| YGL125W | MET13 | 0.95412703 | 1 | -0.0167783 | 117.5087987 | 193.7507118 |
| YNL010W | YNL010W | 0.97127632 | 1 | -0.0158249 | 118.1735568 | 194.7100451 |
| YOR029W | YOR029W | 0.9790001 | 1 | -0.0153061 | 121.0752757 | 199.372749 |
| YHR030C | SLT2 | 0.97662467 | 1 | -0.0152857 | 132.631297 | 218.2086575 |
| YOR045W | TOM6 | 0.97918739 | 1 | -0.0151643 | 121.0424616 | 199.3007635 |
| YPL247C | YPL247C | 0.9831567 | 1 | -0.0151566 | 143.5196727 | 235.9420031 |
| YOR225W | YOR225W | 0.97874523 | 1 | -0.0148555 | 132.3022821 | 217.6161871 |
| YGR125W | YGR125W | 0.97237881 | 1 | -0.0147923 | 146.2373807 | 240.324884 |
| YPL166W | ATG29 | 0.98231413 | 1 | -0.0146944 | 130.9667765 | 215.418041 |
| YDR031W | MIC14 | 0.98197542 | 1 | -0.0144377 | 130.3815777 | 214.4305747 |
| YGR067C | YGR067C | 0.97821811 | 1 | -0.0142832 | 119.3106211 | 196.3625935 |
| YNL093W | YPT53 | 0.98317253 | 1 | -0.0140895 | 122.5464298 | 201.6123357 |
| YGL176C | YGL176C | 0.96931191 | 1 | -0.0139778 | 135.8884704 | 223.3478987 |
| YOR342C | YOR342C | 0.98067895 | 1 | -0.0137381 | 138.6878212 | 227.880126 |
| YNR004W | YNR004W | 0.97307587 | 1 | -0.0136931 | 103.0035548 | 169.7019219 |
| YOR253W | NAT5 | 0.97881199 | 1 | -0.0135079 | 123.5974391 | 203.2498273 |
| YGL261C | PAU11 | 0.97116262 | 1 | -0.0134193 | 118.4630568 | 194.8682234 |
| YGL181W | GTS1 | 0.96976958 | 1 | -0.0132644 | 134.8817615 | 221.6137115 |
| YNR013C | PHO91 | 0.97877512 | 1 | -0.0119684 | 117.5338719 | 193.1642272 |
| YDR530C | APA2 | 0.97892521 | 1 | -0.0119446 | 127.6638874 | 209.6750266 |
| YPR195C | YPR195C | 0.98378677 | 1 | -0.0119244 | 125.7543819 | 206.5595242 |
| YOR268C | YOR268C | 0.98223476 | 1 | -0.0118877 | 132.3533758 | 217.3123902 |
| YNL068C | FKH2 | 0.97836711 | 1 | -0.0118374 | 115.2809311 | 189.4744116 |
| YGL084C | GUP1 | 0.97042217 | 1 | -0.0117641 | 113.3450917 | 186.309046 |
| YOR243C | PUS7 | 0.98147281 | 1 | -0.0111945 | 123.2312196 | 202.351075 |
| YMR223W | UBP8 | 0.97429588 | 1 | -0.0111752 | 100.2670262 | 164.9124355 |
| YHR047C | AAP1 | 0.97177795 | 1 | -0.0110223 | 140.7788727 | 230.9347237 |
| YNL193W | YNL193W | 0.98030017 | 1 | -0.0108458 | 121.7975253 | 199.9683884 |
| YPR037C | ERV2 | 0.98467071 | 1 | -0.0108218 | 145.8685149 | 239.2056759 |
| YGL163C | RAD54 | 0.97727665 | 1 | -0.0105509 | 111.9674132 | 183.9049201 |
| YNL086W | YNL086W | 0.98626512 | 1 | -0.0105021 | 122.7692695 | 201.5076989 |
| YNL297C | MON2 | 0.97106126 | 1 | -0.0104642 | 89.20112078 | 146.7801084 |
| YOR371C | GPB1 | 0.98566454 | 1 | -0.010269 | 132.4558859 | 217.2683649 |
| YLR054C | OSW2 | 0.9883281 | 1 | -0.0100285 | 112.245247 | 184.2897152 |
| YOL048C | YOL048C | 0.9864375 | 1 | -0.0097895 | 120.2893204 | 197.3719447 |
| YIL040W | APQ12 | 0.9843746 | 1 | -0.0097145 | 79.93828702 | 131.5820951 |
| YPR096C | YPR096C | 0.98792561 | 1 | -0.0096968 | 134.5783619 | 220.6537885 |
| YPR014C | YPR014C | 0.98787912 | 1 | -0.0092858 | 129.1651554 | 211.7755991 |
| YPL223C | GRE1 | 0.9889739 | 1 | -0.0086554 | 131.6073059 | 215.6745555 |
| YOR034C | AKR2 | 0.98665643 | 1 | -0.0083906 | 123.6545509 | 202.6754727 |
| YFR038W | IRC5 | 0.98294548 | 1 | -0.0078675 | 129.0118649 | 211.340711 |
| YPL150W | YPL150W | 0.98867953 | 1 | -0.0077982 | 135.9694414 | 222.6738895 |
| YGR058W | YGR058W | 0.97877275 | 1 | -0.0076099 | 120.2497467 | 197.0231543 |

| | | | | | | |
|---|---|---|---|---|---|---|
| YDL176W | YDL176W | 0.97479409 | 1 | -0.0074574 | 141.3078188 | 231.3320255 |
| YOR069W | VPS5 | 0.98621352 | 1 | -0.0073747 | 109.6241466 | 179.6706693 |
| YKL201C | MNN4 | 0.98795018 | 1 | -0.0070367 | 123.7573198 | 202.6664107 |
| YNL238W | KEX2 | 0.9903097 | 1 | -0.0070059 | 78.39758896 | 128.7171768 |
| YPR140W | TAZ1 | 0.99090283 | 1 | -0.0069331 | 126.1015896 | 206.4745255 |
| YIL136W | OM45 | 0.99132078 | 1 | -0.0067082 | 125.3579177 | 205.2328586 |
| YPL024W | RMI1 | 0.98958129 | 1 | -0.0064625 | 105.8412324 | 173.3848034 |
| YPR164W | MMS1 | 0.99260345 | 1 | -0.0063466 | 79.59237851 | 130.578922 |
| YML047C | PRM6 | 0.98856534 | 1 | -0.0061546 | 99.59512895 | 163.1622729 |
| YMR230W | RPS10B | 0.98396886 | 1 | -0.006108 | 89.15359741 | 146.134454 |
| YGL023C | PIB2 | 0.98362255 | 1 | -0.0060368 | 132.7168198 | 217.141732 |
| YOL091W | SPO21 | 0.99247737 | 1 | -0.0054048 | 121.3127401 | 198.4684173 |
| YBR095C | RXT2 | 0.98896953 | 1 | -0.0052351 | 124.5321057 | 203.694487 |
| YNR073C | YNR073C | 0.99236746 | 1 | -0.0050597 | 129.3865165 | 211.585245 |
| YNL135C | FPR1 | 0.99156701 | 1 | -0.0049852 | 101.4980862 | 166.1119331 |
| YJL107C | YJL107C | 0.99410134 | 1 | -0.0049171 | 134.1269271 | 219.294446 |
| YGL235W | YGL235W | 0.98791194 | 1 | -0.0047281 | 113.0190651 | 184.859847 |
| YBR118W | TEF2 | 0.99317795 | 1 | -0.0045664 | 111.4550181 | 182.2890611 |
| YNR028W | CPR8 | 0.99331767 | 1 | -0.0044453 | 129.9463973 | 212.4178192 |
| YER161C | SPT2 | 0.99340762 | 1 | -0.0040037 | 130.428132 | 213.1455407 |
| YPL100W | ATG21 | 0.99535354 | 1 | -0.0034321 | 139.6193911 | 228.054535 |
| YNL284C | MRPL10 | 0.99599557 | 1 | -0.0032832 | 135.4033518 | 221.1621559 |
| YOR288C | MPD1 | 0.99559501 | 1 | -0.0031923 | 135.4562024 | 221.2364449 |
| YKL123W | YKL123W | 0.99462984 | 1 | -0.0031076 | 125.2509531 | 204.5888482 |
| YOL056W | GPM3 | 0.99568053 | 1 | -0.0028913 | 127.059191 | 207.5084217 |
| YGR106C | YGR106C | 0.99352449 | 1 | -0.0027848 | 129.939135 | 212.1894057 |
| YOR184W | SER1 | 0.99554849 | 1 | -0.0026304 | 116.8591823 | 190.8463821 |
| YDR046C | BAP3 | 0.99702613 | 1 | -0.0020872 | 118.7710404 | 193.8922349 |
| YGR163W | GTR2 | 0.99790165 | 1 | -0.0018211 | 96.76445473 | 157.9824998 |
| YNR020C | YNR020C | 0.99710841 | 1 | -0.0018058 | 106.6216396 | 174.0496325 |
| YGL086W | MAD1 | 0.99590805 | 1 | -0.0017895 | 134.0619238 | 218.7805349 |
| YOR338W | YOR338W | 0.99818681 | 1 | -0.0017668 | 149.8556692 | 244.5244674 |
| YPR007C | REC8 | 0.99845242 | 1 | -0.0011617 | 122.9696183 | 200.6160295 |
| YPL194W | DDC1 | 0.99829564 | 1 | -0.0011568 | 130.9600402 | 213.6413317 |
| YLR238W | FAR10 | 0.99853631 | 1 | -0.0011263 | 128.655663 | 209.8807664 |
| YGL126W | SCS3 | 0.99813281 | 1 | -0.00095 | 133.8994261 | 218.406138 |
| YDR511W | ACN9 | 0.99971378 | 1 | -0.0001278 | 111.3038247 | 181.4636444 |
| YER142C | MAG1 | 0.99982436 | 1 | -0.0000922 | 121.9685104 | 198.844525 |
| YOR238W | YOR238W | 0.99996567 | 1 | -0.0000248 | 142.9980578 | 233.1180034 |
| YGL151W | NUT1 | 1 | 1 | 0 | 0 | 0 |
| YKL101W | HSL1 | 1 | 1 | 0 | 0 | 0 |
| YOR212W | STE4 | 0.99997801 | 1 | 0.0000165 | 146.0314461 | 238.0576343 |
| YOR219C | STE13 | 0.99989973 | 1 | 0.0000689 | 127.5621218 | 207.9421969 |
| YHR167W | THP2 | 0.99880703 | 1 | 0.00042349 | 142.3456231 | 231.9959416 |
| YGR089W | NNF2 | 0.99884062 | 1 | 0.00044905 | 108.8653182 | 177.4131694 |
| YGR015C | YGR015C | 0.99897696 | 1 | 0.00049512 | 129.848704 | 211.6141784 |
| YPR054W | SMK1 | 0.99920194 | 1 | 0.00060915 | 121.9874004 | 198.7838453 |
| YGL117W | YGL117W | 0.99721201 | 1 | 0.00101063 | 137.4513463 | 223.9407328 |
| YOR237W | HES1 | 0.99858457 | 1 | 0.00103921 | 140.8093514 | 229.4112087 |
| YGL042C | YGL042C | 0.99709771 | 1 | 0.0016959 | 116.7339182 | 190.0778968 |
| YGR069W | YGR069W | 0.9962184 | 1 | 0.00173944 | 130.1929777 | 212.0131145 |
| YPR093C | ASR1 | 0.9976746 | 1 | 0.00178444 | 126.7397934 | 206.3778788 |
| YOR296W | YOR296W | 0.99742498 | 1 | 0.00191895 | 139.2929298 | 226.8244002 |
| YNL199C | GCR2 | 0.99624226 | 1 | 0.00193202 | 98.17465803 | 159.7918783 |
| YPR024W | YME1 | 0.9941348 | 1 | 0.00212042 | 86.23295361 | 140.2999938 |
| YPL245W | YPL245W | 0.99698328 | 1 | 0.00214286 | 137.3990209 | 223.7077532 |
| YKR024C | DBP7 | 0.98202996 | 1 | 0.00226415 | 24.5100822 | 39.66090747 |
| YPR063C | YPR063C | 0.99653973 | 1 | 0.00238898 | 133.1377286 | 216.7289134 |
| YPL111W | CAR1 | 0.99648945 | 1 | 0.00243528 | 131.1846617 | 213.538993 |
| YNL304W | YPT11 | 0.99535212 | 1 | 0.00243965 | 115.3738875 | 187.7637717 |
| YEL049W | PAU2 | 0.99479092 | 1 | 0.00256595 | 119.7743901 | 194.9209776 |
| YGR255C | COQ6 | 0.99612779 | 1 | 0.00273081 | 117.1934384 | 190.6920198 |
| YPR023C | EAF3 | 0.99613055 | 1 | 0.00282866 | 113.4431532 | 184.5655591 |
| YOR011W | AUS1 | 0.99569267 | 1 | 0.00289307 | 132.8880711 | 216.2561729 |

| | | | | | | |
|---|---|---|---|---|---|---|
| YER053C | PIC2 | 0.99480784 | 1 | 0.00290704 | 128.0562778 | 208.377584 |
| YPL115C | BEM3 | 0.9958241 | 1 | 0.00303269 | 138.6580807 | 225.6442055 |
| YOR104W | PIN2 | 0.9954679 | 1 | 0.00306966 | 130.5727633 | 212.4587372 |
| YPL067C | YPL067C | 0.99491466 | 1 | 0.0033838 | 128.7208071 | 209.398712 |
| YKR065C | PAM17 | 0.99373557 | 1 | 0.00342915 | 118.1975721 | 192.2378687 |
| YOR376W | YOR376W | 0.99459045 | 1 | 0.00386212 | 142.4431076 | 231.7063551 |
| YOR155C | ISN1 | 0.99473657 | 1 | 0.00403499 | 130.6385745 | 212.4401132 |
| YGR042W | YGR042W | 0.99169471 | 1 | 0.0040561 | 132.0549221 | 214.7462821 |
| YJL119C | YJL119C | 0.99373305 | 1 | 0.00463954 | 114.815305 | 186.5662402 |
| YPL220W | RPL1A | 0.99342914 | 1 | 0.00471505 | 138.1559807 | 224.6062519 |
| YPL168W | YPL168W | 0.99282257 | 1 | 0.00518805 | 121.9343475 | 198.1001271 |
| YOL065C | INP54 | 0.99166514 | 1 | 0.00535882 | 127.2447763 | 206.7348903 |
| YKL046C | DCW1 | 0.98973864 | 1 | 0.00561996 | 119.8979659 | 194.7240921 |
| YPL109C | YPL109C | 0.99080841 | 1 | 0.00623084 | 127.464184 | 206.9788293 |
| YPL021W | ECM23 | 0.99162154 | 1 | 0.00625441 | 124.0220594 | 201.3644193 |
| YPL035C | YPL035C | 0.99092549 | 1 | 0.00631141 | 116.2833264 | 188.7413375 |
| YOR300W | YOR300W | 0.98510342 | 1 | 0.00635407 | 130.574687 | 212.0334832 |
| YOR023C | AHC1 | 0.99043355 | 1 | 0.0068144 | 136.5641373 | 221.7374163 |
| YMR299C | DYN3 | 0.98676833 | 1 | 0.00684737 | 114.4272704 | 185.6456973 |
| YPL130W | SPO19 | 0.98969791 | 1 | 0.00716381 | 138.2103244 | 224.3754496 |
| YOR383C | FIT3 | 0.98984821 | 1 | 0.00746514 | 138.3945626 | 224.6364905 |
| YNR059W | MNT4 | 0.96523329 | 1 | 0.00752356 | 127.4553745 | 206.7958564 |
| YGR273C | YGR273C | 0.99032278 | 1 | 0.00763063 | 129.6335678 | 210.3327715 |
| YDR493W | FMP36 | 0.98991568 | 1 | 0.0082138 | 130.3182027 | 211.3727976 |
| YPL116W | HOS3 | 0.98891299 | 1 | 0.00825846 | 137.4573422 | 223.0051649 |
| YOR356W | YOR356W | 0.98847115 | 1 | 0.00826565 | 137.0015482 | 222.2611936 |
| YPR038W | YPR038W | 0.98947978 | 1 | 0.00830267 | 131.1347786 | 212.6923843 |
| YOL050C | :::GAL11 | 0.98546157 | 1 | 0.00837384 | 115.0962446 | 186.5371573 |
| YPR123C | YPR123C | 0.98602853 | 1 | 0.00844214 | 122.6313664 | 198.81197 |
| YGR022C | YGR022C | 0.97832679 | 1 | 0.00864356 | 112.456339 | 182.1984156 |
| YOR078W | BUD21 | 0.97840253 | 1 | 0.00875881 | 68.5433861 | 110.5966902 |
| YPR020W | ATP20 | 0.98721119 | 1 | 0.00882622 | 110.6551601 | 179.2383173 |
| YKL162C | YKL162C | 0.98436153 | 1 | 0.00911899 | 124.579916 | 201.9002035 |
| YNL194C | YNL194C | 0.98443978 | 1 | 0.00952025 | 125.8880439 | 203.9803708 |
| YGR054W | YGR054W | 0.98168816 | 1 | 0.00961783 | 135.3858796 | 219.4509714 |
| YPL125W | KAP120 | 0.98694398 | 1 | 0.00991453 | 123.1994215 | 199.5459655 |
| YFR047C | BNA6 | 0.97596945 | 1 | 0.01033829 | 134.5903696 | 218.0601642 |
| YOR302W | YOR302W | 0.98387643 | 1 | 0.01137067 | 137.0314993 | 221.9050285 |
| YPR064W | YPR064W | 0.98447272 | 1 | 0.01145919 | 122.514419 | 198.2278052 |
| YNR034W | SOL1 | 0.98381416 | 1 | 0.01186233 | 118.8423038 | 192.1889583 |
| YPL179W | PPQ1 | 0.98185549 | 1 | 0.01190495 | 111.5116957 | 180.2330756 |
| YPL199C | YPL199C | 0.98124729 | 1 | 0.01205778 | 129.8840384 | 210.1636505 |
| YPL057C | SUR1 | 0.98203804 | 1 | 0.01222272 | 117.476698 | 189.915748 |
| YPL203W | TPK2 | 0.98381286 | 1 | 0.01271261 | 148.2466904 | 240.0129505 |
| YOR264W | DSE3 | 0.98169369 | 1 | 0.01271446 | 138.3956167 | 223.9535346 |
| YOL131W | YOL131W | 0.98185172 | 1 | 0.01329162 | 120.0665136 | 193.9982353 |
| YGL087C | MMS2 | 0.97088606 | 1 | 0.01352116 | 137.1231695 | 221.7739772 |
| YGR247W | CPD1 | 0.96089628 | 1 | 0.01367437 | 147.3854818 | 238.4835682 |
| YOR304C-A | YOR304C-A | 0.98047876 | 1 | 0.01372782 | 138.7101349 | 224.3340862 |
| YOR315W | SFG1 | 0.98227575 | 1 | 0.0137286 | 142.9348223 | 231.2210502 |
| YOR226C | ISU2 | 0.98225892 | 1 | 0.01411157 | 135.9067232 | 219.7139254 |
| YOL089C | HAL9 | 0.97573097 | 1 | 0.01422379 | 119.1941945 | 192.4546003 |
| YOR061W | CKA2 | 0.97104754 | 1 | 0.01433665 | 109.7185582 | 176.9927421 |
| YOR144C | ELG1 | 0.97554113 | 1 | 0.01459558 | 124.2337938 | 200.6216387 |
| YDR369C | XRS2 | 0.92956005 | 1 | 0.01465387 | 103.968377 | 167.5774479 |
| YNL140C | YNL140C | 0.97403223 | 1 | 0.01465834 | 115.245869 | 185.9613801 |
| YPL105C | YPL105C | 0.97735127 | 1 | 0.01520803 | 138.4278632 | 223.6808633 |
| YNL079C | TPM1 | 0.97238085 | 1 | 0.01526573 | 95.52708078 | 153.7366803 |
| YOR348C | PUT4 | 0.98081577 | 1 | 0.01530825 | 130.6599626 | 211.004596 |
| YDR467C | YDR467C | 0.97305371 | 1 | 0.01540833 | 132.6699427 | 214.268203 |
| YPR122W | AXL1 | 0.97924655 | 1 | 0.01565791 | 120.6438781 | 194.6308146 |
| YOR347C | PYK2 | 0.98229216 | 1 | 0.01567067 | 141.9934952 | 229.433197 |
| YOR298W | MUM3 | 0.97809523 | 1 | 0.01579588 | 139.666907 | 225.6240727 |
| YIL049W | DFG10 | 0.94581468 | 1 | 0.01582829 | 132.8983783 | 214.5858213 |

| | | | | | | |
|---|---|---|---|---|---|---|
| YDR290W | :::RTT103 | 0.97776403 | 1 | 0.01583531 | 116.9214501 | 188.5393917 |
| YGR004W | PEX31 | 0.96624696 | 1 | 0.01604203 | 132.0563939 | 213.1853435 |
| YPL114W | YPL114W | 0.98059671 | 1 | 0.01615255 | 141.2571993 | 228.1700393 |
| YJR120W | YJR120W | 0.97330471 | 1 | 0.01625031 | 102.2311496 | 164.5372026 |
| YJL053W | PEP8 | 0.92026232 | 1 | 0.01640939 | 140.5448949 | 226.9753433 |
| YEL005C | VAB2 | 0.969811 | 1 | 0.01644828 | 127.0262109 | 204.932175 |
| YER054C | GIP2 | 0.97015202 | 1 | 0.01656643 | 129.6701806 | 209.2269517 |
| YOR161C | PNS1 | 0.97263866 | 1 | 0.01662632 | 124.6053793 | 200.9625251 |
| YNR064C | YNR064C | 0.97484265 | 1 | 0.01706282 | 118.8661375 | 191.5495064 |
| YPL147W | PXA1 | 0.97484759 | 1 | 0.01813884 | 139.3248514 | 224.7608598 |
| YPR120C | CLB5 | 0.96966666 | 1 | 0.01817753 | 120.0248817 | 193.293092 |
| YKL006W | RPL14A | 0.83830666 | 1 | 0.01821338 | 5.490070172 | 6.57429619 |
| YHR008C | SOD2 | 0.96682045 | 1 | 0.0184035 | 119.524013 | 192.4471055 |
| YMR245W | YMR245W | 0.95488927 | 1 | 0.01885036 | 100.5008579 | 161.3773608 |
| YFR044C | DUG1 | 0.95860827 | 1 | 0.0195356 | 128.8408179 | 207.487657 |
| YPR185W | ATG13 | 0.97394117 | 1 | 0.01980007 | 114.5584223 | 184.1700662 |
| YOR229W | WTM2 | 0.97272023 | 1 | 0.01983135 | 143.4899061 | 231.3299575 |
| YPL226W | NEW1 | 0.95667564 | 1 | 0.02051566 | 97.98869023 | 157.0648307 |
| YPL023C | MET12 | 0.97112879 | 1 | 0.02065619 | 139.8767655 | 225.3322475 |
| YLL025W | PAU17 | 0.95161242 | 1 | 0.02092982 | 132.8840989 | 213.8971449 |
| YNL100W | YNL100W | 0.95899152 | 1 | 0.02104905 | 117.2133155 | 188.3351553 |
| YML001W | YPT7 | 0.95161201 | 1 | 0.02110213 | 90.21903524 | 144.3222805 |
| YEL062W | NPR2 | 0.9524747 | 1 | 0.02118178 | 112.7858417 | 181.1001955 |
| YGL217C | YGL217C | 0.89862964 | 1 | 0.02119971 | 123.037819 | 197.8105822 |
| YPL214C | THI6 | 0.9741892 | 1 | 0.02121103 | 129.3796855 | 208.1475878 |
| YPL187W | MF(ALPHA)1 | 0.97336752 | 1 | 0.02143979 | 131.3038968 | 211.254591 |
| YPL022W | RAD1 | 0.97393522 | 1 | 0.02198962 | 155.1163482 | 250.0018244 |
| YNL107W | YAF9 | 0.95890299 | 1 | 0.02251527 | 119.2728513 | 191.5013596 |
| YOR220W | WSP1 | 0.97130584 | 1 | 0.02256615 | 125.9781179 | 202.4256185 |
| YGR237C | YGR237C | 0.93875076 | 1 | 0.0227266 | 152.4163284 | 245.5041385 |
| YOR343C | YOR343C | 0.96871798 | 1 | 0.02360059 | 141.8275107 | 228.1283015 |
| YPR149W | NCE102 | 0.96829475 | 1 | 0.0236161 | 116.1245414 | 186.2254176 |
| YPR058W | YMC1 | 0.96912606 | 1 | 0.02379078 | 126.0652663 | 202.4079577 |
| YJR044C | VPS55 | 0.96331118 | 1 | 0.0239522 | 120.8103091 | 193.820296 |
| YGR068C | YGR068C | 0.96916598 | 1 | 0.02396622 | 129.9708266 | 208.7519012 |
| YIL036W | CST6 | 0.92886854 | 1 | 0.02413182 | 26.77650097 | 40.50338886 |
| YPR018W | RLF2 | 0.95820396 | 1 | 0.02437819 | 118.6736985 | 190.2816406 |
| YOR235W | YOR235W | 0.95698581 | 1 | 0.02453211 | 113.5906392 | 181.9751846 |
| YDR326C | YSP2 | 0.96098873 | 1 | 0.02461759 | 127.4584021 | 204.5711991 |
| YPR193C | HPA2 | 0.96750022 | 1 | 0.02514304 | 142.7950827 | 229.5044498 |
| YJR139C | HOM6 | 0.95546854 | 1 | 0.02532605 | 95.65027974 | 152.6253394 |
| YPR076W | YPR076W | 0.9671502 | 1 | 0.02554215 | 130.902806 | 210.0656595 |
| YKR028W | SAP190 | 0.95258281 | 1 | 0.0256572 | 92.99738018 | 148.2574023 |
| YPL039W | YPL039W | 0.96298954 | 1 | 0.02633068 | 143.0398441 | 229.7485532 |
| YOR228C | YOR228C | 0.96395856 | 1 | 0.02642465 | 137.6099008 | 220.8844283 |
| YNL155W | YNL155W | 0.94590886 | 1 | 0.02644265 | 108.8292814 | 173.9640476 |
| YMR263W | SAP30 | 0.94230015 | 1 | 0.02697617 | 104.6604287 | 167.0984159 |
| YOL042W | NGL1 | 0.95825439 | 1 | 0.02724828 | 134.3037663 | 215.3873557 |
| YDL172C | YDL172C | 0.90872295 | 1 | 0.02732531 | 147.8418136 | 237.4469716 |
| YBR270C | BIT2 | 0.91255403 | 1 | 0.02772235 | 151.2322748 | 242.9222988 |
| YNL226W | YNL226W | 0.95463189 | 1 | 0.02853092 | 88.94397116 | 141.2747323 |
| YGR122C-A | YGR122C-A | 0.96276795 | 1 | 0.02866837 | 125.5012753 | 200.8523524 |
| YBL103C | RTG3 | 0.92906696 | 1 | 0.02911666 | 125.9685981 | 201.5557085 |
| YNL012W | SPO1 | 0.95952979 | 1 | 0.02926034 | 148.7189473 | 238.6244823 |
| YHR059W | FYV4 | 0.92984367 | 1 | 0.02939601 | 129.8161585 | 207.7915478 |
| YPL270W | MDL2 | 0.95935923 | 1 | 0.02955494 | 121.6979435 | 194.5365419 |
| YFR023W | PES4 | 0.94496249 | 1 | 0.0297665 | 138.6981657 | 222.2226313 |
| YBR028C | YBR028C | 0.84278896 | 1 | 0.03051323 | 127.6012469 | 204.0350894 |
| YDR271C | YDR271C | 0.95912617 | 1 | 0.03052314 | 122.4741125 | 195.6755662 |
| YGL167C | PMR1 | 0.93885161 | 1 | 0.03057189 | 79.30828448 | 125.300474 |
| YGL063W | PUS2 | 0.95793936 | 1 | 0.03066007 | 121.4841763 | 194.0439165 |
| YFR034C | PHO4 | 0.93848694 | 1 | 0.03067714 | 136.7806165 | 218.9778757 |
| YPL258C | THI21 | 0.9621788 | 1 | 0.03077084 | 136.0420046 | 217.7615733 |
| YPL202C | AFT2 | 0.95593206 | 1 | 0.03100893 | 139.8555844 | 223.9473987 |

| | | | | | | |
|---|---|---|---|---|---|---|
| YPR022C | YPR022C | 0.95695226 | 1 | 0.03153186 | 122.6337136 | 195.8041787 |
| YGR134W | CAF130 | 0.92614483 | 1 | 0.03157577 | 138.2423133 | 221.2435181 |
| YIL128W | MET18 | 0.8489107 | 1 | 0.03176885 | 115.6230491 | 184.3445149 |
| YGR288W | MAL13 | 0.91451955 | 1 | 0.03186675 | 149.7368747 | 239.9439467 |
| YOR267C | HRK1 | 0.95312792 | 1 | 0.03209345 | 114.4744449 | 182.4297278 |
| YOR120W | GCY1 | 0.95171362 | 1 | 0.03237445 | 131.4260442 | 210.0274956 |
| YMR276W | DSK2 | 0.95418587 | 1 | 0.03237969 | 122.6865052 | 195.7796552 |
| YNR006W | VPS27 | 0.93749448 | 1 | 0.03269561 | 91.80688129 | 145.3986288 |
| YOR378W | YOR378W | 0.96127888 | 1 | 0.03290574 | 141.5525223 | 226.466336 |
| YIL053W | RHR2 | 0.90619603 | 1 | 0.03307682 | 134.6397509 | 215.1748536 |
| YPL158C | YPL158C | 0.95229565 | 1 | 0.03314925 | 113.3017135 | 180.3802364 |
| YPL177C | CUP9 | 0.95643751 | 1 | 0.03364055 | 145.2640859 | 232.4210672 |
| YMR193C-A | YMR193C-A | 0.92123379 | 1 | 0.03366607 | 96.10062068 | 152.2716854 |
| YIL043C | CBR1 | 0.92912393 | 1 | 0.03386793 | 155.9749459 | 249.8522046 |
| YML112W | CTK3 | 0.95475559 | 1 | 0.03392341 | 125.9776634 | 200.9435369 |
| YMR316W | DIA1 | 0.9375471 | 1 | 0.03394363 | 117.4877157 | 187.1006263 |
| YER052C | HOM3 | 0.92343999 | 1 | 0.03427784 | 105.4447199 | 167.424599 |
| YGR086C | PIL1 | 0.92368528 | 1 | 0.0343405 | 118.0704443 | 187.9988242 |
| YOR270C | VPH1 | 0.94720357 | 1 | 0.03451909 | 110.0591803 | 174.9156045 |
| YPL033C | YPL033C | 0.95280903 | 1 | 0.03458874 | 134.6870732 | 215.0547974 |
| YHR046C | INM1 | 0.90239397 | 1 | 0.03468199 | 152.5832725 | 244.2169363 |
| YDR274C | YDR274C | 0.95657532 | 1 | 0.03473574 | 134.205738 | 214.2509529 |
| YML011C | RAD33 | 0.94769958 | 1 | 0.0350581 | 96.01550719 | 151.9513692 |
| YOR325W | YOR325W | 0.95161781 | 1 | 0.03527247 | 119.8038526 | 190.7030604 |
| YOR215C | YOR215C | 0.94853131 | 1 | 0.03541999 | 122.798538 | 195.5657414 |
| YER074W | RPS24A | 0.89097671 | 1 | 0.03575594 | 107.2866684 | 170.2345442 |
| YDL019C | OSH2 | 0.8821957 | 1 | 0.03585756 | 152.7458068 | 244.328567 |
| YPR045C | MNI2 | 0.93511448 | 1 | 0.03725499 | 95.74983151 | 151.2317237 |
| YGL010W | YGL010W | 0.91234982 | 1 | 0.03726495 | 133.954323 | 213.5112104 |
| YHR043C | DOG2 | 0.88564295 | 1 | 0.03741611 | 153.3609714 | 245.1281227 |
| YPR027C | YPR027C | 0.95110239 | 1 | 0.03767174 | 127.1792044 | 202.4133856 |
| YER156C | YER156C | 0.92218662 | 1 | 0.03790593 | 125.4738156 | 199.6027231 |
| YBR299W | MAL32 | 0.95158679 | 1 | 0.03805389 | 130.4944018 | 207.76796 |
| YPL135W | ISU1 | 0.95318273 | 1 | 0.03824384 | 146.3914696 | 233.6585114 |
| YCL045C | YCL045C | 0.85004227 | 1 | 0.03853827 | 152.581499 | 243.7110661 |
| YMR243C | ZRC1 | 0.93428376 | 1 | 0.03857292 | 110.6476453 | 175.3461713 |
| YMR294W-A | YMR294W-A | 0.92755194 | 1 | 0.03874845 | 119.4965877 | 189.7487821 |
| YOR053W | YOR053W | 0.93259647 | 1 | 0.03898912 | 123.2657551 | 195.8618703 |
| YNL235C | :::SIN4 | 0.68416459 | 1 | 0.04078326 | 6.686555336 | 5.580986059 |
| YPR153W | YPR153W | 0.94491378 | 1 | 0.04080028 | 122.1014479 | 193.7275908 |
| YJL177W | RPL17B | 0.94141244 | 1 | 0.04104948 | 140.9691001 | 224.4530465 |
| YPL216W | YPL216W | 0.95144074 | 1 | 0.04107816 | 153.1107503 | 244.2425679 |
| YGL211W | NCS6 | 0.90370557 | 1 | 0.04134037 | 105.8532792 | 167.1694566 |
| YPR068C | HOS1 | 0.94349465 | 1 | 0.04178306 | 126.8983898 | 201.4193584 |
| YKL213C | DOA1 | 0.91232725 | 1 | 0.04194993 | 102.1418441 | 161.0395873 |
| YJL101C | GSH1 | 0.95432886 | 1 | 0.04203381 | 58.88987529 | 90.51948601 |
| YPR050C | YPR050C | 0.92921079 | 1 | 0.04226015 | 109.0621332 | 172.2805476 |
| YPR150W | YPR150W | 0.93773623 | 1 | 0.04245485 | 137.7474138 | 219.0177651 |
| YNL122C | YNL122C | 0.93596014 | 1 | 0.04263091 | 115.6152845 | 182.9151061 |
| YIL029C | YIL029C | 0.85955972 | 1 | 0.04279496 | 134.6634313 | 213.9459097 |
| YPL224C | MMT2 | 0.94529531 | 1 | 0.04299496 | 127.4561217 | 202.1705006 |
| YPL053C | KTR6 | 0.94070173 | 1 | 0.04370106 | 142.4221494 | 226.4759521 |
| YPR011C | YPR011C | 0.94753478 | 1 | 0.04412602 | 138.3749208 | 219.8227516 |
| YOR066W | YOR066W | 0.92838906 | 1 | 0.04414073 | 129.7231108 | 205.7166923 |
| YPL229W | YPL229W | 0.93785612 | 1 | 0.04436539 | 134.3127535 | 213.1694029 |
| YGL101W | YGL101W | 0.90328266 | 1 | 0.04453823 | 120.349847 | 190.3845941 |
| YPL066W | YPL066W | 0.92544059 | 1 | 0.04454985 | 129.1555315 | 204.738065 |
| YNR007C | ATG3 | 0.91447181 | 1 | 0.04469548 | 115.4570629 | 182.3878896 |
| YKL070W | YKL070W | 0.91955794 | 1 | 0.04521258 | 128.3778234 | 203.3838078 |
| YGR072W | UPF3 | 0.886629 | 1 | 0.04522394 | 122.0469507 | 193.0617674 |
| YGR241C | YAP1802 | 0.88318662 | 1 | 0.04529679 | 144.5365203 | 229.7146573 |
| YHR032W | YHR032W | 0.88157437 | 1 | 0.0453569 | 156.3315077 | 248.9349519 |
| YCR063W | BUD31 | 0.73633425 | 1 | 0.04540204 | 8.784953876 | 8.399353583 |
| YPR012W | YPR012W | 0.93423015 | 1 | 0.04544558 | 129.5583583 | 205.2779207 |

| | | | | | | |
|---|---|---|---|---|---|---|
| YNL011C | YNL011C | 0.78713544 | 1 | 0.04559415 | 130.4318599 | 206.6825207 |
| YOR339C | UBC11 | 0.93458744 | 1 | 0.04578971 | 123.4643481 | 195.2986081 |
| YOR382W | FIT2 | 0.79948952 | 1 | 0.04586903 | 127.7171329 | 202.2211315 |
| YMR118C | YMR118C | 0.94039801 | 1 | 0.04598924 | 129.8687521 | 205.7130123 |
| YPR119W | CLB2 | 0.78451204 | 1 | 0.04613464 | 129.8884214 | 205.7261129 |
| YML081C-A | ATP18 | 0.90572814 | 1 | 0.04619035 | 99.37135394 | 155.9700641 |
| YOR154W | SLP1 | 0.92605755 | 1 | 0.04639387 | 128.463358 | 203.3691686 |
| YPR184W | GDB1 | 0.93668898 | 1 | 0.04701842 | 124.0404402 | 196.0774877 |
| YPR071W | YPR071W | 0.94173321 | 1 | 0.04792492 | 136.561934 | 216.3717341 |
| YPL134C | ODC1 | 0.93176563 | 1 | 0.04849566 | 134.1401919 | 212.3493793 |
| YPR174C | YPR174C | 0.92665664 | 1 | 0.04859143 | 136.009076 | 215.3835343 |
| YMR116C | ASC1 | 0.86175809 | 1 | 0.04924871 | 81.44939355 | 126.3548617 |
| YOR251C | YOR251C | 0.92503873 | 1 | 0.04929771 | 127.8243394 | 201.9486927 |
| YEL053C | MAK10 | 0.88626726 | 1 | 0.04930549 | 125.5070809 | 198.1700946 |
| YOR337W | TEA1 | 0.93276804 | 1 | 0.05018771 | 125.9851081 | 198.8343026 |
| YPL079W | RPL21B | 0.91645168 | 1 | 0.05064486 | 123.2105937 | 194.2516753 |
| YPR021C | AGC1 | 0.9310153 | 1 | 0.05065334 | 123.8055237 | 195.2204213 |
| YJL206C | YJL206C | 0.77798804 | 1 | 0.05072846 | 150.647536 | 238.9683481 |
| YNL336W | COS1 | 0.91299985 | 1 | 0.05083623 | 118.7376195 | 186.9348927 |
| YOR284W | HUA2 | 0.93532463 | 1 | 0.05085608 | 134.0164011 | 211.8397029 |
| YIL094C | LYS12 | 0.92806179 | 1 | 0.0509876 | 114.2689773 | 179.6303897 |
| YNL016W | PUB1 | 0.91891041 | 1 | 0.0510952 | 108.8301559 | 170.7500142 |
| YHR039C | MSC7 | 0.87533072 | 1 | 0.05119546 | 161.3847818 | 256.4112454 |
| YJL134W | LCB3 | 0.77092045 | 1 | 0.05145655 | 147.4721853 | 233.6969407 |
| YNL047C | SLM2 | 0.92585145 | 1 | 0.05175422 | 130.1317168 | 205.3897633 |
| YOR008C | SLG1 | 0.90722474 | 1 | 0.05176559 | 96.15226105 | 149.9951287 |
| YFL030W | AGX1 | 0.9120506 | 1 | 0.05210204 | 125.6149521 | 197.9811878 |
| YPR042C | PUF2 | 0.93474907 | 1 | 0.05229152 | 126.9886985 | 200.1959485 |
| YMR169C | ALD3 | 0.74738974 | 1 | 0.05243087 | 129.7576599 | 204.6917218 |
| YLL009C | COX17 | 0.89498372 | 1 | 0.05291059 | 87.27730091 | 135.3778663 |
| YOR089C | VPS21 | 0.9132739 | 1 | 0.0529781 | 117.5538431 | 184.7257402 |
| YPR199C | ARR1 | 0.93093207 | 1 | 0.05393122 | 128.4807033 | 202.414341 |
| YMR256C | COX7 | 0.88993784 | 1 | 0.05420695 | 84.60842436 | 130.8579904 |
| YCR105W | ADH7 | 0.83140747 | 1 | 0.0542157 | 157.0259154 | 248.911509 |
| YPL107W | YPL107W | 0.9357093 | 1 | 0.05424214 | 135.425353 | 213.6949232 |
| YPL164C | MLH3 | 0.91390259 | 1 | 0.05432129 | 124.7059978 | 196.2099557 |
| YPR146C | YPR146C | 0.93587149 | 1 | 0.05454375 | 139.9597639 | 221.047559 |
| YNL014W | HEF3 | 0.92831521 | 1 | 0.05513335 | 125.5090556 | 197.413179 |
| YGL049C | TIF4632 | 0.92817437 | 1 | 0.05519031 | 128.0854747 | 201.6058162 |
| YDL232W | OST4 | 0.84840922 | 1 | 0.05535648 | 111.5465525 | 174.6224677 |
| YJL211C | YJL211C | 0.78660742 | 1 | 0.05544279 | 139.0970116 | 219.5238421 |
| YHR041C | SRB2 | 0.88578609 | 1 | 0.05561724 | 99.22032699 | 154.4943007 |
| YBR108W | YBR108W | 0.85128707 | 1 | 0.05596198 | 143.7240504 | 226.9991004 |
| YPL014W | YPL014W | 0.91604629 | 1 | 0.05597838 | 135.1028416 | 212.9427064 |
| YHL033C | RPL8A | 0.8635148 | 1 | 0.05599461 | 124.5712714 | 195.7720722 |
| YPR109W | YPR109W | 0.92944101 | 1 | 0.05652239 | 124.6128237 | 195.7709717 |
| YGR166W | KRE11 | 0.82508376 | 1 | 0.05665819 | 142.5092109 | 224.9278673 |
| YOR114W | YOR114W | 0.91394905 | 1 | 0.05689585 | 132.5276409 | 208.6249598 |
| YGL096W | TOS8 | 0.92471396 | 1 | 0.05701928 | 125.3930944 | 196.9781551 |
| YMR265C | YMR265C | 0.89819685 | 1 | 0.05739605 | 123.8338626 | 194.3871608 |
| YHR116W | COX23 | 0.9054844 | 1 | 0.05750748 | 103.6137645 | 161.4099162 |
| YKL041W | VPS24 | 0.86013115 | 1 | 0.05758198 | 95.10716048 | 147.5327727 |
| YIL112W | HOS4 | 0.75935883 | 1 | 0.05779941 | 147.8132985 | 233.4257155 |
| YPR001W | CIT3 | 0.91636926 | 1 | 0.05804528 | 136.5262077 | 214.9934826 |
| YNL165W | YNL165W | 0.89906848 | 1 | 0.05826324 | 120.6249606 | 189.042915 |
| YGR170W | PSD2 | 0.85681094 | 1 | 0.05835043 | 140.1900956 | 220.9265359 |
| YPL036W | PMA2 | 0.92739206 | 1 | 0.05845192 | 144.2456503 | 227.5246446 |
| YOR191W | RIS1 | 0.90601238 | 1 | 0.05849025 | 122.7260992 | 192.4385724 |
| YJL144W | YJL144W | 0.93700695 | 1 | 0.05850851 | 121.5347415 | 190.4940444 |
| YHR025W | THR1 | 0.94523982 | 1 | 0.05853835 | 59.1715082 | 88.82589775 |
| YOR032C | HMS1 | 0.90613718 | 1 | 0.05858265 | 125.3281938 | 196.6684426 |
| YGR268C | HUA1 | 0.85095428 | 1 | 0.05904587 | 167.1585048 | 264.7996044 |
| YOL009C | MDM12 | 0.92847946 | 1 | 0.05906234 | 40.21012706 | 57.84679775 |
| YIL158W | YIL158W | 0.91645902 | 1 | 0.05909457 | 117.1174803 | 183.2166061 |

| | | | | | | |
|---|---|---|---|---|---|---|
| YNL198C | YNL198C | 0.88098045 | 1 | 0.05919055 | 85.1768293 | 131.1345851 |
| YAL042W | ERV46 | 0.85311716 | 1 | 0.06004153 | 154.7689248 | 244.4723034 |
| YMR292W | GOT1 | 0.89081215 | 1 | 0.06062793 | 115.5863948 | 180.5206386 |
| YBR274W | CHK1 | 0.74604013 | 1 | 0.06108098 | 145.2698044 | 228.8513051 |
| YNL159C | ASI2 | 0.89421117 | 1 | 0.06109843 | 123.2903784 | 193.01827 |
| YGL071W | AFT1 | 0.91420764 | 1 | 0.06119228 | 137.6919706 | 216.4834373 |
| YBR213W | MET8 | 0.83385978 | 1 | 0.06132167 | 159.1003114 | 251.3663394 |
| YHR129C | ARP1 | 0.83728193 | 1 | 0.06230345 | 137.6318759 | 216.24054 |
| YPL015C | HST2 | 0.92502902 | 1 | 0.06287298 | 135.0735086 | 211.9956183 |
| YPR200C | ARR2 | 0.92480913 | 1 | 0.06345161 | 141.0536254 | 221.6689057 |
| YOR299W | BUD7 | 0.91854997 | 1 | 0.06353398 | 128.4116247 | 201.0492302 |
| YDR297W | SUR2 | 0.7472555 | 1 | 0.0635595 | 159.5331267 | 251.7800311 |
| YER120W | SCS2 | 0.83459968 | 1 | 0.06377979 | 119.7667896 | 186.9243991 |
| YER173W | RAD24 | 0.72059357 | 1 | 0.06388807 | 129.4147653 | 202.6383605 |
| YPR145W | ASN1 | 0.91573786 | 1 | 0.06390689 | 122.8104985 | 191.8696618 |
| YPR059C | YPR059C | 0.91340442 | 1 | 0.06421866 | 123.9033118 | 193.6104953 |
| YBR200W | BEM1 | 0.88832983 | 1 | 0.06427842 | 108.8507755 | 169.0641268 |
| YFL063W | YFL063W | 0.91724386 | 1 | 0.0644699 | 130.4139841 | 204.1913942 |
| YHL017W | YHL017W | 0.8055453 | 1 | 0.06483855 | 144.0625913 | 226.3932068 |
| YGL037C | PNC1 | 0.90381119 | 1 | 0.0649411 | 136.9112714 | 214.7217826 |
| YGR157W | CHO2 | 0.75547225 | 1 | 0.06496072 | 19.24870897 | 22.90625845 |
| YCL046W | YCL046W | 0.71732485 | 1 | 0.06502772 | 154.7723153 | 243.827476 |
| YPL201C | YIG1 | 0.90559959 | 1 | 0.06526676 | 139.4432324 | 218.8068972 |
| YIL085C | KTR7 | 0.90247073 | 1 | 0.06527002 | 132.6457687 | 207.7252792 |
| YPR157W | YPR157W | 0.91311243 | 1 | 0.06535661 | 126.8660627 | 198.2919344 |
| YMR310C | YMR310C | 0.88694752 | 1 | 0.06561793 | 120.7218507 | 188.2415846 |
| YPR009W | SUT2 | 0.91324121 | 1 | 0.06568867 | 124.4383053 | 194.2909042 |
| YKL175W | ZRT3 | 0.67039673 | 1 | 0.06622692 | 134.646984 | 210.8628407 |
| YGL149W | YGL149W | 0.78744864 | 1 | 0.06705301 | 113.0299207 | 175.5150568 |
| YLR308W | CDA2 | 0.90003487 | 1 | 0.06730145 | 131.6666184 | 205.8641103 |
| YPL086C | ELP3 | 0.89931174 | 1 | 0.06746588 | 120.9408517 | 188.3575692 |
| YML030W | YML030W | 0.66561044 | 1 | 0.06753736 | 112.5208114 | 174.6219349 |
| YML026C | RPS18B | 0.87166987 | 1 | 0.06754173 | 100.7555697 | 155.4417215 |
| YGL024W | YGL024W | 0.84381925 | 1 | 0.06756989 | 95.91480662 | 147.5466591 |
| YJR125C | ENT3 | 0.86830746 | 1 | 0.06789249 | 114.422296 | 177.6754062 |
| YBL098W | BNA4 | 0.81532252 | 1 | 0.06807592 | 130.1917233 | 203.3587284 |
| YDR242W | AMD2 | 0.89769212 | 1 | 0.06816189 | 128.4786702 | 200.5549044 |
| YLL039C | UBI4 | 0.8705303 | 1 | 0.06828909 | 121.6812856 | 189.4572484 |
| YOL128C | YGK3 | 0.9012552 | 1 | 0.06831908 | 132.7404444 | 207.4819264 |
| YKL208W | CBT1 | 0.87549819 | 1 | 0.06832593 | 95.19452772 | 146.2738528 |
| YPR075C | OPY2 | 0.90762505 | 1 | 0.06848177 | 121.8545513 | 189.7145737 |
| YKL139W | CTK1 | 0.89636927 | 1 | 0.06921439 | 65.79602925 | 98.23267408 |
| YHR057C | CPR2 | 0.78197036 | 1 | 0.06937927 | 149.8966551 | 235.3116197 |
| YGR243W | FMP43 | 0.82441639 | 1 | 0.06955076 | 149.4374754 | 234.5406993 |
| YPR066W | UBA3 | 0.90732666 | 1 | 0.06976273 | 117.1126333 | 181.8172436 |
| YGR174C | CBP4 | 0.76863952 | 1 | 0.06979355 | 142.5003621 | 223.2001824 |
| YHR111W | UBA4 | 0.78219238 | 1 | 0.07026053 | 117.8205407 | 182.9063424 |
| YFL007W | BLM10 | 0.91382144 | 1 | 0.07052659 | 140.0493812 | 219.1089932 |
| YNL277W | MET2 | 0.88965674 | 1 | 0.07068948 | 134.0663034 | 209.3341618 |
| YIL122W | POG1 | 0.9077132 | 1 | 0.07073649 | 127.7856515 | 199.0893409 |
| YOR197W | MCA1 | 0.91814389 | 1 | 0.07090233 | 142.3903038 | 222.8761459 |
| YOL147C | PEX11 | 0.9019015 | 1 | 0.07152208 | 123.7899393 | 192.4730833 |
| YHL032C | GUT1 | 0.83323301 | 1 | 0.07168772 | 159.0005467 | 249.8516486 |
| YPL181W | CTI6 | 0.902092 | 1 | 0.07192098 | 144.978819 | 226.9630676 |
| YPL149W | ATG5 | 0.91886819 | 1 | 0.07216464 | 161.3489936 | 253.6178704 |
| YGR223C | HSV2 | 0.81083618 | 1 | 0.07247993 | 118.5404892 | 183.7905203 |
| YBR188C | NTC20 | 0.79836625 | 1 | 0.07260049 | 145.4947436 | 227.7154964 |
| YAL017W | PSK1 | 0.81346589 | 1 | 0.07283359 | 148.2931652 | 232.2470679 |
| YER089C | PTC2 | 0.90423386 | 1 | 0.0729492 | 128.4365826 | 199.8618787 |
| YHR209W | CRG1 | 0.7822689 | 1 | 0.0730292 | 129.4597367 | 201.5193861 |
| YOR068C | VAM10 | 0.87022267 | 1 | 0.07312897 | 96.40319523 | 147.617751 |
| YPR029C | APL4 | 0.89568575 | 1 | 0.07321955 | 138.7750542 | 216.6803453 |
| YNR005C | YNR005C | 0.86781026 | 1 | 0.07375317 | 115.463447 | 178.6082708 |
| YGR228W | YGR228W | 0.75346066 | 1 | 0.07378368 | 147.9338406 | 231.5373763 |

| | | | | | | |
|---|---|---|---|---|---|---|
| YGR061C | ADE6 | 0.88940429 | 1 | 0.07382234 | 132.4988482 | 206.3702814 |
| YBR036C | CSG2 | 0.86429382 | 1 | 0.07451989 | 113.2872001 | 174.9605583 |
| YBL025W | RRN10 | 0.75600106 | 1 | 0.07454412 | 146.24996 | 228.6931379 |
| YOR350C | MNE1 | 0.88782901 | 1 | 0.07463211 | 112.2606878 | 173.2725061 |
| YKL184W | SPE1 | 0.90369461 | 1 | 0.07476935 | 36.33123545 | 49.47476343 |
| YOR026W | BUB3 | 0.88945995 | 1 | 0.07548206 | 100.8640716 | 154.5829357 |
| YPL162C | YPL162C | 0.912496 | 1 | 0.07560507 | 151.2864113 | 236.7651565 |
| YGL054C | ERV14 | 0.79335752 | 1 | 0.0756527 | 120.7934998 | 187.0495403 |
| YOR158W | PET123 | 0.90143049 | 1 | 0.07600902 | 126.4725003 | 196.2609448 |
| YMR105C | PGM2 | 0.65392317 | 1 | 0.07610747 | 127.6311817 | 198.1369821 |
| YIL027C | KRE27 | 0.77476498 | 1 | 0.07615784 | 151.2895359 | 236.6981514 |
| YPR083W | MDM36 | 0.905822 | 1 | 0.07627441 | 133.3626535 | 207.458626 |
| YJL204C | RCY1 | 0.58691631 | 1 | 0.07630344 | 92.1453432 | 140.2625705 |
| YIL113W | SDP1 | 0.65280186 | 1 | 0.0763755 | 151.4910761 | 236.9983119 |
| YNL070W | TOM7 | 0.86000737 | 1 | 0.07647388 | 93.93944858 | 143.1650826 |
| YLL041C | SDH2 | 0.85159723 | 1 | 0.07647456 | 110.3344272 | 169.8920129 |
| YCR107W | AAD3 | 0.88040969 | 1 | 0.07649565 | 129.5778918 | 201.2598683 |
| YPL027W | SMA1 | 0.9090081 | 1 | 0.0765017 | 150.3806794 | 235.1716878 |
| YOR308C | SNU66 | 0.87115377 | 1 | 0.07671232 | 129.4298255 | 200.9902306 |
| YPR128C | ANT1 | 0.89802814 | 1 | 0.0769366 | 124.5789238 | 193.0530607 |
| YPR160W | GPH1 | 0.61045548 | 1 | 0.07699194 | 108.2994409 | 166.5071052 |
| YPL212C | PUS1 | 0.90114977 | 1 | 0.07720516 | 145.8061086 | 227.6224899 |
| YPL072W | UBP16 | 0.90373775 | 1 | 0.07750434 | 133.4267201 | 207.4026463 |
| YDL186W | YDL186W | 0.90793143 | 1 | 0.07754842 | 118.7236083 | 183.4279518 |
| YPR201W | ARR3 | 0.88751643 | 1 | 0.07782394 | 128.8652069 | 199.9248032 |
| YJR154W | YJR154W | 0.62898691 | 1 | 0.07782922 | 133.5376795 | 207.5411566 |
| YER046W | SPO73 | 0.86094814 | 1 | 0.07793207 | 129.2650784 | 200.5625684 |
| YOL093W | TRM10 | 0.86289624 | 1 | 0.07851108 | 114.8277344 | 176.9513551 |
| YGR111W | YGR111W | 0.78636023 | 1 | 0.07852216 | 109.1964794 | 167.7698641 |
| YNL268W | LYP1 | 0.66027434 | 1 | 0.07868213 | 133.753942 | 207.7824615 |
| YJR011C | YJR011C | 0.68634771 | 1 | 0.07874912 | 140.6945418 | 219.0882579 |
| YPR079W | MRL1 | 0.89658394 | 1 | 0.07919828 | 127.9835337 | 198.3082462 |
| YNL136W | EAF7 | 0.83226069 | 1 | 0.07943834 | 101.092296 | 154.438964 |
| YIL016W | SNL1 | 0.81725559 | 1 | 0.07962094 | 150.284618 | 234.6082434 |
| YBR178W | YBR178W | 0.79447431 | 1 | 0.0796928 | 158.7183836 | 248.3475562 |
| YPL261C | YPL261C | 0.87262561 | 1 | 0.07977668 | 130.5586501 | 202.4307482 |
| YPL167C | REV3 | 0.88224862 | 1 | 0.08045441 | 138.1068694 | 214.6474229 |
| YOR180C | DCI1 | 0.89232827 | 1 | 0.08090693 | 123.971443 | 191.5448934 |
| YBR227C | MCX1 | 0.60645775 | 1 | 0.08117754 | 154.05303 | 240.5484623 |
| YPR126C | YPR126C | 0.88625787 | 1 | 0.08146465 | 148.9960194 | 232.2670981 |
| YOL001W | PHO80 | 0.86499749 | 1 | 0.08204952 | 101.3124666 | 154.4573056 |
| YPR147C | YPR147C | 0.86810795 | 1 | 0.08229781 | 125.7808879 | 194.3132277 |
| YAL011W | SWC3 | 0.8101757 | 1 | 0.08253746 | 133.4267336 | 206.7461924 |
| YPR097W | YPR097W | 0.87590322 | 1 | 0.08293477 | 135.7075125 | 210.4124864 |
| YNR033W | ABZ1 | 0.87861917 | 1 | 0.08304812 | 133.6907442 | 207.1099757 |
| YPR053C | YPR053C | 0.86859348 | 1 | 0.08327224 | 128.4958418 | 198.6120379 |
| YGR193C | PDX1 | 0.76099596 | 1 | 0.08341768 | 162.3646072 | 253.8057729 |
| YOL008W | COQ10 | 0.87006236 | 1 | 0.08379411 | 108.0644594 | 165.2368244 |
| YIL111W | COX5B | 0.89055028 | 1 | 0.08439544 | 129.1950103 | 199.6053186 |
| YOR047C | STD1 | 0.62092242 | 1 | 0.08498869 | 132.7562415 | 205.3334436 |
| YOR248W | YOR248W | 0.89293166 | 1 | 0.08503485 | 133.6794752 | 206.8324733 |
| YHR079C | IRE1 | 0.79941008 | 1 | 0.08576275 | 163.8089136 | 255.8544048 |
| YOR202W | HIS3 | 0.43760347 | 1 | 0.08601388 | 128.8683841 | 198.8617596 |
| YPR151C | SUE1 | 0.86694719 | 1 | 0.08689029 | 129.6424189 | 200.0092762 |
| YGL179C | TOS3 | 0.62760941 | 1 | 0.08689696 | 127.8314054 | 197.0561005 |
| YPL250C | ICY2 | 0.88447191 | 1 | 0.08707858 | 124.2491795 | 191.1926827 |
| YDR159W | SAC3 | 0.4159074 | 1 | 0.08712523 | 12.23247765 | 8.577488451 |
| YHR204W | MNL1 | 0.78470743 | 1 | 0.08753988 | 153.8297076 | 239.3545551 |
| YHL043W | ECM34 | 0.71617961 | 1 | 0.08800191 | 143.0465935 | 221.7157103 |
| YIL152W | YIL152W | 0.66639959 | 1 | 0.08823152 | 143.5084447 | 222.4386685 |
| YDL109C | YDL109C | 0.59539274 | 1 | 0.08849083 | 133.9256878 | 206.7830808 |
| YHR012W | VPS29 | 0.71170087 | 1 | 0.08852439 | 125.3799416 | 192.8474675 |
| YJL023C | PET130 | 0.59917208 | 1 | 0.08888083 | 138.9283541 | 214.887536 |
| YHR050W | SMF2 | 0.76172279 | 1 | 0.0889941 | 155.7022838 | 242.2175451 |

| | | | | | | |
|---|---|---|---|---|---|---|
| YPR198W | SGE1 | 0.8713036 | 1 | 0.08915009 | 131.964699 | 203.5002981 |
| YDR026C | YDR026C | 0.88356888 | 1 | 0.08980775 | 127.5095801 | 196.1518039 |
| YGR271W | SLH1 | 0.86268287 | 1 | 0.09000903 | 128.0908139 | 197.0730764 |
| YPL004C | LSP1 | 0.87772199 | 1 | 0.09034935 | 142.5742861 | 220.6395772 |
| YIL028W | YIL028W | 0.73062463 | 1 | 0.0903551 | 148.2855914 | 229.9493701 |
| YIR027C | DAL1 | 0.64469575 | 1 | 0.09036677 | 149.3218487 | 231.6371509 |
| YDR032C | PST2 | 0.85517507 | 1 | 0.09044483 | 128.3219545 | 197.3930388 |
| YHR200W | RPN10 | 0.66869069 | 1 | 0.09046887 | 127.008444 | 195.2486247 |
| YGL031C | RPL24A | 0.74135867 | 1 | 0.09056329 | 104.1127225 | 157.911809 |
| YOR368W | RAD17 | 0.58747308 | 1 | 0.09064953 | 127.2525396 | 195.6229835 |
| YPR125W | YLH47 | 0.87679534 | 1 | 0.0908666 | 140.5602414 | 217.2888251 |
| YIL125W | KGD1 | 0.89614438 | 1 | 0.09103818 | 169.1386285 | 263.8548008 |
| YMR145C | NDE1 | 0.77234937 | 1 | 0.09252327 | 94.13989907 | 141.3985169 |
| YIR039C | YPS6 | 0.53997681 | 1 | 0.09275328 | 131.6550726 | 202.525578 |
| YAL002W | VPS8 | 0.8568849 | 1 | 0.09331175 | 110.2472074 | 167.5537333 |
| YLR038C | COX12 | 0.76373571 | 1 | 0.09337516 | 82.75230252 | 122.723396 |
| YBR119W | MUD1 | 0.7641341 | 1 | 0.09338989 | 155.994289 | 242.1202234 |
| YDR269C | YDR269C | 0.58451198 | 1 | 0.09375296 | 130.676815 | 200.8004379 |
| YHR093W | AHT1 | 0.7574077 | 1 | 0.09394588 | 158.1570607 | 245.5734448 |
| YIL050W | PCL7 | 0.73458261 | 1 | 0.09448986 | 150.2663778 | 232.6391388 |
| YGR269W | YGR269W | 0.73888416 | 1 | 0.09476814 | 139.9261255 | 215.7462096 |
| YHR180W | YHR180W | 0.68317722 | 1 | 0.09531562 | 155.4359808 | 240.9588957 |
| YDR217C | RAD9 | 0.56541235 | 1 | 0.09534342 | 129.9763267 | 199.4510594 |
| YER091C | MET6 | 0.7599142 | 1 | 0.0956873 | 115.2708356 | 175.4333833 |
| YBL091C-A | SCS22 | 0.77621637 | 1 | 0.09580119 | 146.2918966 | 225.9889185 |
| YPR173C | VPS4 | 0.85124884 | 1 | 0.09604276 | 109.0777642 | 165.2911037 |
| YGR286C | BIO2 | 0.69727285 | 1 | 0.09604908 | 140.3938416 | 216.3416039 |
| YGL199C | YGL199C | 0.78363185 | 1 | 0.09611539 | 131.674237 | 202.1182959 |
| YOR380W | RDR1 | 0.8698391 | 1 | 0.09725768 | 141.5528209 | 218.0733277 |
| YIL045W | PIG2 | 0.74011398 | 1 | 0.09782744 | 153.679508 | 237.7678825 |
| YER093C-A | YER093C-A | 0.78757937 | 1 | 0.0982583 | 116.6445483 | 177.3374654 |
| YNL108C | YNL108C | 0.85254666 | 1 | 0.09861016 | 130.9751997 | 200.6533328 |
| YBR098W | MMS4 | 0.75980345 | 1 | 0.09865463 | 146.296852 | 225.6248206 |
| YKL055C | OAR1 | 0.8143289 | 1 | 0.09895926 | 113.2482431 | 171.7093969 |
| YGR085C | RPL11B | 0.78970249 | 1 | 0.09907507 | 98.81052403 | 148.1579887 |
| YHR130C | YHR130C | 0.78003448 | 1 | 0.09916812 | 159.8300617 | 247.6196209 |
| YHR124W | NDT80 | 0.62935123 | 1 | 0.09933071 | 141.115777 | 217.0904734 |
| YLL019C | KNS1 | 0.56004191 | 1 | 0.09982965 | 128.3501192 | 196.2148804 |
| YIL102C | YIL102C | 0.86823799 | 1 | 0.10005052 | 127.5073469 | 194.8121882 |
| YBR040W | FIG1 | 0.75174852 | 1 | 0.10007578 | 162.0188042 | 251.0693115 |
| YOL081W | IRA2 | 0.83555252 | 1 | 0.10030946 | 103.0083892 | 154.8403262 |
| YAL046C | YAL046C | 0.83828895 | 1 | 0.10053966 | 126.8714297 | 193.7117193 |
| YCR102C | YCR102C | 0.71096682 | 1 | 0.10068047 | 156.507756 | 242.0063566 |
| YPR114W | YPR114W | 0.86797025 | 1 | 0.10076103 | 124.4343007 | 189.7098501 |
| YJL094C | KHA1 | 0.51188328 | 1 | 0.10086681 | 131.6948276 | 201.5321303 |
| YGR249W | MGA1 | 0.76198128 | 1 | 0.10118814 | 162.7016815 | 252.0374491 |
| YHL009C | YAP3 | 0.74263995 | 1 | 0.1015522 | 149.0250565 | 229.6943932 |
| YHR151C | MTC6 | 0.75558859 | 1 | 0.10176018 | 143.982975 | 221.4476886 |
| YGR261C | APL6 | 0.76491265 | 1 | 0.10191718 | 140.47541 | 215.7091935 |
| YDR532C | KRE28 | 0.5258809 | 1 | 0.10212142 | 24.41222574 | 26.47688776 |
| YDR043C | NRG1 | 0.86478327 | 1 | 0.10228565 | 122.0121229 | 185.5623687 |
| YPR032W | SRO7 | 0.85120495 | 1 | 0.10302787 | 132.5760965 | 202.6869011 |
| YPL090C | RPS6A | 0.80656082 | 1 | 0.10377357 | 113.8738371 | 172.1013013 |
| YPL240C | HSP82 | 0.87391689 | 1 | 0.10385998 | 138.2433475 | 211.8170939 |
| YPL108W | YPL108W | 0.86950012 | 1 | 0.10426018 | 134.1794401 | 205.139933 |
| YDR335W | MSN5 | 0.4485853 | 1 | 0.10457077 | 149.3142694 | 229.7721514 |
| YHR061C | GIC1 | 0.66798326 | 1 | 0.1053429 | 138.2835963 | 211.6892891 |
| YHR136C | SPL2 | 0.75091281 | 1 | 0.10540429 | 167.876697 | 259.9238174 |
| YOL109W | ZEO1 | 0.83709766 | 1 | 0.10578834 | 119.7341584 | 181.3919817 |
| YPR170C | YPR170C | 0.86569507 | 1 | 0.1058242 | 127.2273388 | 193.6026521 |
| YNL125C | ESBP6 | 0.58854894 | 1 | 0.1060445 | 146.420847 | 224.8630872 |
| YPL001W | HAT1 | 0.53308847 | 1 | 0.10640093 | 129.6968679 | 197.5532446 |
| YHR155W | YSP1 | 0.77441889 | 1 | 0.10680687 | 158.5153746 | 244.4800937 |
| YBR134W | YBR134W | 0.73217296 | 1 | 0.1068444 | 152.4062278 | 234.5160953 |

| | | | | | | |
|---|---|---|---|---|---|---|
| YJL210W | PEX2 | 0.5472607 | 1 | 0.10696697 | 141.8194586 | 217.2416058 |
| YHR176W | FMO1 | 0.70992487 | 1 | 0.10796216 | 142.6662322 | 218.4922088 |
| YPR052C | NHP6A | 0.85434042 | 1 | 0.10818825 | 142.4301963 | 218.0779346 |
| YGL029W | CGR1 | 0.86101733 | 1 | 0.10844976 | 132.0089085 | 201.0550905 |
| YPL272C | YPL272C | 0.86699856 | 1 | 0.10863291 | 127.6711098 | 193.9597432 |
| YBR278W | DPB3 | 0.47903966 | 1 | 0.10872425 | 144.3652289 | 221.162505 |
| YOR240W | YOR240W | 0.82923162 | 1 | 0.10874893 | 131.3184257 | 199.8904485 |
| YIL023C | YKE4 | 0.69166635 | 1 | 0.10882973 | 123.3165838 | 186.8353429 |
| YKL087C | CYT2 | 0.84151082 | 1 | 0.10887828 | 116.0678642 | 175.0121817 |
| YPR015C | YPR015C | 0.86819242 | 1 | 0.10889958 | 139.2566531 | 212.8116604 |
| YAL048C | GEM1 | 0.85296272 | 1 | 0.10895867 | 35.81744855 | 44.17783901 |
| YOL052C | SPE2 | 0.75816998 | 1 | 0.1090272 | 35.21216306 | 43.18216694 |
| YIL057C | YIL057C | 0.75862888 | 1 | 0.10906056 | 172.0037564 | 266.1748403 |
| YIL035C | CKA1 | 0.86961157 | 1 | 0.10915435 | 102.8997504 | 153.5095753 |
| YIL008W | URM1 | 0.77222282 | 1 | 0.10930402 | 125.4706944 | 190.2851029 |
| YBR197C | YBR197C | 0.70336635 | 1 | 0.10945142 | 148.2242441 | 227.358608 |
| YHR104W | GRE3 | 0.68705964 | 1 | 0.10982523 | 137.6137244 | 210.0126324 |
| YHL027W | RIM101 | 0.74837845 | 1 | 0.10984251 | 147.6324249 | 226.3428173 |
| YBR280C | SAF1 | 0.60714483 | 1 | 0.11057263 | 156.1689042 | 240.163716 |
| YGR289C | MAL11 | 0.8428246 | 1 | 0.11064676 | 133.7896867 | 203.6715503 |
| YIL041W | GVP36 | 0.68498369 | 1 | 0.11084284 | 141.4141651 | 216.0753647 |
| YHR117W | TOM71 | 0.6871184 | 1 | 0.1109491 | 144.5066706 | 221.1028932 |
| YGR203W | YGR203W | 0.7417823 | 1 | 0.11094967 | 166.5420135 | 257.0247335 |
| YHR005C | GPA1 | 0.67449072 | 1 | 0.11115066 | 139.4550262 | 212.8414368 |
| YNL076W | MKS1 | 0.78726284 | 1 | 0.11124708 | 110.9430181 | 166.3487157 |
| YOR385W | YOR385W | 0.87176431 | 1 | 0.11152627 | 152.8122042 | 234.567255 |
| YML022W | APT1 | 0.80727634 | 1 | 0.11156796 | 92.11350254 | 135.6110743 |
| YHR157W | REC104 | 0.67803764 | 1 | 0.11160657 | 137.1381953 | 209.0050838 |
| YOR183W | FYV12 | 0.76511931 | 1 | 0.11187369 | 97.9543785 | 145.0929661 |
| YNL329C | PEX6 | 0.8145346 | 1 | 0.11196965 | 117.6480199 | 177.1849332 |
| YBL056W | PTC3 | 0.75291612 | 1 | 0.11238113 | 149.6376015 | 229.2805315 |
| YIL065C | FIS1 | 0.52193107 | 1 | 0.11274102 | 125.2020218 | 189.3988212 |
| YHR143W | DSE2 | 0.69335546 | 1 | 0.11275021 | 166.0248342 | 255.9467839 |
| YHR131C | YHR131C | 0.71222646 | 1 | 0.11300222 | 153.7526002 | 235.9077736 |
| YJL060W | BNA3 | 0.41243116 | 1 | 0.11309838 | 150.1955344 | 230.0965195 |
| YOR269W | PAC1 | 0.8208552 | 1 | 0.11313056 | 131.9131526 | 200.2884686 |
| YOR140W | SFL1 | 0.76635775 | 1 | 0.11348305 | 24.50353564 | 25.1438304 |
| YHR184W | SSP1 | 0.72314937 | 1 | 0.11360531 | 147.6696806 | 225.9127645 |
| YPR002W | PDH1 | 0.85264331 | 1 | 0.11408039 | 130.563551 | 197.9644671 |
| YOL086C | ADH1 | 0.85197373 | 1 | 0.11410151 | 124.1166514 | 187.4520063 |
| YGL219C | MDM34 | 0.71491909 | 1 | 0.11484803 | 113.5213122 | 170.0821633 |
| YCR086W | CSM1 | 0.62124421 | 1 | 0.11498337 | 129.8928956 | 196.7533904 |
| YPL096W | PNG1 | 0.49725642 | 1 | 0.11520119 | 134.1567301 | 203.675863 |
| YHR133C | NSG1 | 0.68520965 | 1 | 0.1153271 | 151.9697219 | 232.6980971 |
| YJL196C | ELO1 | 0.2809553 | 1 | 0.11538257 | 156.9604199 | 240.8266753 |
| YHL037C | YHL037C | 0.65099207 | 1 | 0.11557747 | 139.5320322 | 212.3895779 |
| YDR121W | DPB4 | 0.53167419 | 1 | 0.11573984 | 156.5572671 | 240.1228585 |
| YGL244W | RTF1 | 0.76991208 | 1 | 0.11602036 | 116.9239564 | 175.4762299 |
| YFL010W-A | AUA1 | 0.8252519 | 1 | 0.11609028 | 130.0238342 | 196.8224698 |
| YNL042W | BOP3 | 0.82610867 | 1 | 0.11624934 | 131.7453695 | 199.6081623 |
| YDR202C | RAV2 | 0.46953394 | 1 | 0.11689896 | 153.5331677 | 235.0418006 |
| YHR105W | YPT35 | 0.63700063 | 1 | 0.11698169 | 148.3755047 | 226.6230109 |
| YKR097W | PCK1 | 0.78287416 | 1 | 0.11733362 | 110.7000653 | 165.1587807 |
| YGR164W | YGR164W | 0.71736821 | 1 | 0.11746805 | 158.4665931 | 243.0100207 |
| YBR189W | RPS9B | 0.75971628 | 1 | 0.11774183 | 33.13183432 | 38.65416575 |
| YBR054W | YRO2 | 0.68614641 | 1 | 0.1184745 | 153.6021787 | 234.9488027 |
| YFL047W | RGD2 | 0.8083267 | 1 | 0.1185795 | 141.4555844 | 215.133785 |
| YOR365C | YOR365C | 0.84429128 | 1 | 0.11861995 | 135.7638354 | 205.8498465 |
| YJR091C | JSN1 | 0.49024707 | 1 | 0.11928487 | 150.9746155 | 230.5596636 |
| YAL028W | FRT2 | 0.63882246 | 1 | 0.11929071 | 145.406236 | 221.4813547 |
| YGL078C | DBP3 | 0.49648567 | 1 | 0.11938212 | 104.1693975 | 154.2453285 |
| YPR026W | ATH1 | 0.86241101 | 1 | 0.11957837 | 143.3552012 | 218.1002483 |
| YGR208W | SER2 | 0.63849853 | 1 | 0.11966914 | 135.2000592 | 204.7939343 |
| YIR035C | YIR035C | 0.60492072 | 1 | 0.11967856 | 146.9092552 | 223.8809829 |

| | | | | | | |
|---|---|---|---|---|---|---|
| YIL044C | AGE2 | 0.66454017 | 1 | 0.1197049 | 130.1041428 | 196.4819301 |
| YPL133C | RDS2 | 0.85113857 | 1 | 0.11975461 | 149.4273387 | 227.9760318 |
| YAL026C | DRS2 | 0.69182508 | 1 | 0.11985969 | 119.1543317 | 178.6114093 |
| YDL149W | ATG9 | 0.73687148 | 1 | 0.11988745 | 147.5145239 | 224.8404431 |
| YGL124C | MON1 | 0.66794118 | 1 | 0.12018114 | 94.99380353 | 139.1831003 |
| YBR012C | YBR012C | 0.78582392 | 1 | 0.12036487 | 137.6297665 | 208.6640871 |
| YGR244C | LSC2 | 0.65589971 | 1 | 0.12069434 | 144.3929527 | 219.6464288 |
| YBR255W | MTC4 | 0.39259516 | 1 | 0.12146021 | 154.3102611 | 235.7136849 |
| YNL191W | DUG3 | 0.77308205 | 1 | 0.12150374 | 120.0022563 | 179.7792566 |
| YDR034C | LYS14 | 0.46731944 | 1 | 0.12193805 | 124.6230964 | 187.2554815 |
| YNL281W | HCH1 | 0.80164474 | 1 | 0.12225157 | 124.7387635 | 187.4031487 |
| YHL042W | YHL042W | 0.67814058 | 1 | 0.12227317 | 134.4064705 | 203.1605827 |
| YBR186W | PCH2 | 0.67239751 | 1 | 0.12232648 | 152.5899061 | 232.7961816 |
| YIR031C | DAL7 | 0.57496701 | 1 | 0.12259625 | 153.731573 | 234.622136 |
| YPR196W | YPR196W | 0.84994244 | 1 | 0.12360666 | 136.8637889 | 206.9925637 |
| YOR312C | RPL20B | 0.81223306 | 1 | 0.12387884 | 133.2960305 | 201.1409199 |
| YJR060W | CBF1 | 0.44855039 | 1 | 0.1239025 | 149.66407 | 227.8209351 |
| YDR092W | UBC13 | 0.26032469 | 1 | 0.1239295 | 144.2171683 | 218.9378995 |
| YMR206W | YMR206W | 0.46593459 | 1 | 0.1244886 | 131.9165847 | 198.8126211 |
| YER060W-A | FCY22 | 0.72375647 | 1 | 0.1247054 | 114.1714769 | 169.8563517 |
| YOR333C | YOR333C | 0.83431987 | 1 | 0.12486507 | 125.5648697 | 188.4089812 |
| YNL090W | RHO2 | 0.82636376 | 1 | 0.12531152 | 124.1967687 | 186.1204782 |
| YPL263C | KEL3 | 0.81192688 | 1 | 0.12553533 | 122.7506232 | 183.7337872 |
| YHL036W | MUP3 | 0.67474388 | 1 | 0.12565786 | 156.1784359 | 238.2116707 |
| YKR104W | YKR104W | 0.82154227 | 1 | 0.1257943 | 136.1954923 | 205.6177731 |
| YGR279C | SCW4 | 0.67256672 | 1 | 0.12609987 | 158.5666536 | 242.0472804 |
| YBR199W | KTR4 | 0.64373457 | 1 | 0.126395 | 143.2281991 | 217.0041097 |
| YGL154C | LYS5 | 0.69495383 | 1 | 0.12660564 | 119.1670485 | 177.7522579 |
| YDR030C | RAD28 | 0.80146895 | 1 | 0.12737896 | 131.4175058 | 197.6220323 |
| YDR033W | MRH1 | 0.82143599 | 1 | 0.12747253 | 137.0295717 | 206.7585922 |
| YGR227W | DIE2 | 0.66883883 | 1 | 0.12840762 | 153.9682599 | 234.2499981 |
| YBR105C | VID24 | 0.77664307 | 1 | 0.12907788 | 155.6596695 | 236.9199038 |
| YCL075W | YCL075W | 0.44622099 | 1 | 0.1293896 | 156.7602907 | 238.6734734 |
| YDR387C | YDR387C | 0.52421842 | 1 | 0.12947462 | 157.5150161 | 239.8927336 |
| YBL088C | TEL1 | 0.70249854 | 1 | 0.12950645 | 154.5865834 | 235.1146646 |
| YBR157C | ICS2 | 0.68179606 | 1 | 0.12951601 | 158.876885 | 242.1074478 |
| YOL153C | YOL153C | 0.81688054 | 1 | 0.12955483 | 135.8346794 | 204.539087 |
| YBR145W | ADH5 | 0.67554276 | 1 | 0.12957408 | 157.8450637 | 240.4178031 |
| YJL082W | IML2 | 0.80051408 | 1 | 0.12967304 | 125.6561678 | 187.930707 |
| YJR033C | RAV1 | 0.59502911 | 1 | 0.12982673 | 145.7791024 | 220.714976 |
| YOL114C | YOL114C | 0.80313665 | 1 | 0.13002293 | 131.5677496 | 197.5221032 |
| YPR003C | YPR003C | 0.79843656 | 1 | 0.13012173 | 129.7373331 | 194.5252797 |
| YBR042C | YBR042C | 0.65584016 | 1 | 0.13069451 | 142.8857534 | 215.8850655 |
| YHR009C | YHR009C | 0.59042728 | 1 | 0.13091354 | 129.3924231 | 193.8597332 |
| YIL090W | ICE2 | 0.55442733 | 1 | 0.13134764 | 120.5373111 | 179.3675502 |
| YGR187C | HGH1 | 0.61016178 | 1 | 0.13188798 | 153.8299551 | 233.5705871 |
| YBR219C | YBR219C | 0.67875886 | 1 | 0.13190316 | 155.6786312 | 236.5823104 |
| YGR287C | YGR287C | 0.66750467 | 1 | 0.13204733 | 151.9838504 | 230.5402914 |
| YBR008C | FLR1 | 0.67182569 | 1 | 0.13215565 | 150.4090338 | 227.9589043 |
| YHR075C | PPE1 | 0.59179114 | 1 | 0.13268662 | 144.2561664 | 217.859273 |
| YGR194C | XKS1 | 0.62367085 | 1 | 0.13282717 | 143.0436526 | 215.8643066 |
| YBL021C | HAP3 | 0.67455172 | 1 | 0.1332596 | 130.0388934 | 194.6076069 |
| YGR176W | YGR176W | 0.60779092 | 1 | 0.13400023 | 138.017061 | 207.516977 |
| YHL007C | STE20 | 0.60032459 | 1 | 0.13410181 | 137.4142348 | 206.5210038 |
| YDR500C | RPL37B | 0.62011357 | 1 | 0.13432212 | 109.7581366 | 161.4074253 |
| YHR137W | ARO9 | 0.59075715 | 1 | 0.1347182 | 143.8347391 | 216.9072836 |
| YGR148C | RPL24B | 0.66512692 | 1 | 0.134775 | 113.0815271 | 166.7661306 |
| YBR013C | YBR013C | 0.66536511 | 1 | 0.13486105 | 156.4544038 | 237.4611703 |
| YDL089W | YDL089W | 0.85317639 | 1 | 0.13499401 | 118.8627609 | 176.1621058 |
| YBR086C | IST2 | 0.80149124 | 1 | 0.13509498 | 127.1511525 | 189.660634 |
| YHR108W | GGA2 | 0.64574516 | 1 | 0.13511508 | 124.1153458 | 184.7090546 |
| YHL045W | YHL045W | 0.59710229 | 1 | 0.13535207 | 135.320332 | 202.9444606 |
| YOR279C | RFM1 | 0.84464098 | 1 | 0.1353835 | 154.4910118 | 234.1923147 |
| YHL044W | YHL044W | 0.5412428 | 1 | 0.13567531 | 162.2944199 | 246.8753335 |

| | | | | | | |
|---|---|---|---|---|---|---|
| YDL113C | ATG20 | 0.50335209 | 1 | 0.13600121 | 148.9389091 | 225.0607332 |
| YJL093C | TOK1 | 0.59146864 | 1 | 0.13672094 | 149.8999253 | 226.5335019 |
| YHL019C | APM2 | 0.62937364 | 1 | 0.13740351 | 142.9636804 | 215.1370399 |
| YJR035W | RAD26 | 0.48852728 | 1 | 0.13750563 | 149.4297014 | 225.6645977 |
| YDR371W | CTS2 | 0.44937739 | 1 | 0.13754527 | 160.7270146 | 244.0762551 |
| YPL189W | GUP2 | 0.8522908 | 1 | 0.13756307 | 166.6188515 | 253.6787789 |
| YAL061W | YAL061W | 0.66031308 | 1 | 0.13761836 | 155.8008449 | 236.0361029 |
| YLR387C | REH1 | 0.39891794 | 1 | 0.13778513 | 137.9076688 | 206.8449782 |
| YGR232W | NAS6 | 0.63268084 | 1 | 0.13817237 | 150.3364358 | 227.0557878 |
| YFR031C-A | RPL2A | 0.68162977 | 1 | 0.13846998 | 116.7913657 | 172.3319518 |
| YPR188C | MLC2 | 0.79878878 | 1 | 0.13856879 | 135.4067739 | 202.6658164 |
| YPR073C | LTP1 | 0.82642894 | 1 | 0.13868089 | 130.19037 | 194.1474377 |
| YOR334W | MRS2 | 0.8167058 | 1 | 0.13871008 | 128.9471374 | 192.1169182 |
| YBR162W-A | YSY6 | 0.68507798 | 1 | 0.13905219 | 156.7834184 | 237.4508742 |
| YBR023C | CHS3 | 0.62291762 | 1 | 0.13930216 | 155.0410409 | 234.5778542 |
| YIL039W | YIL039W | 0.5754823 | 1 | 0.13988138 | 130.9350359 | 195.204808 |
| YHR082C | KSP1 | 0.55532376 | 1 | 0.14036055 | 150.5136927 | 227.0593445 |
| YHR202W | YHR202W | 0.61498624 | 1 | 0.14055136 | 153.4230723 | 231.7773143 |
| YHR066W | SSF1 | 0.46391737 | 1 | 0.1406674 | 117.2843592 | 172.8490158 |
| YDL077C | VAM6 | 0.49791699 | 1 | 0.14069194 | 110.1225585 | 161.170681 |
| YHR033W | YHR033W | 0.65244417 | 1 | 0.14082355 | 151.6361879 | 228.8288417 |
| YNL046W | YNL046W | 0.68781753 | 1 | 0.14098874 | 146.2538169 | 220.0329799 |
| YJL191W | RPS14B | 0.3852769 | 1 | 0.14138059 | 151.8567664 | 229.1157725 |
| YJL057C | IKS1 | 0.51516512 | 1 | 0.14158709 | 156.7304562 | 237.0339055 |
| YIL141W | YIL141W | 0.33102261 | 1 | 0.14185126 | 151.0634015 | 227.7610423 |
| YHR199C | FMP34 | 0.6031832 | 1 | 0.14185757 | 147.5668121 | 222.0600957 |
| YLR048W | RPS0B | 0.77556952 | 1 | 0.14209437 | 47.43829975 | 58.80017081 |
| YBR061C | TRM7 | 0.67146705 | 1 | 0.14227757 | 144.3047873 | 216.6875766 |
| YHR044C | DOG1 | 0.59484075 | 1 | 0.14252961 | 150.8612566 | 227.3430295 |
| YGR231C | PHB2 | 0.32359176 | 1 | 0.14279191 | 123.0263494 | 181.9324794 |
| YBR165W | UBS1 | 0.63117851 | 1 | 0.14281877 | 134.1514264 | 200.0650256 |
| YNL020C | ARK1 | 0.78910171 | 1 | 0.1431051 | 98.4389518 | 141.8093686 |
| YOR345C | YOR345C | 0.82572008 | 1 | 0.14313555 | 137.0342775 | 204.7233175 |
| YGR259C | YGR259C | 0.59538445 | 1 | 0.14336243 | 144.494936 | 216.8560566 |
| YHL040C | ARN1 | 0.65459284 | 1 | 0.14351 | 152.8873333 | 230.5180572 |
| YDR502C | SAM2 | 0.75233609 | 1 | 0.14360326 | 155.5202382 | 234.7980425 |
| YIL020C | HIS6 | 0.68667447 | 1 | 0.14375581 | 125.8686908 | 186.4403297 |
| YGL198W | YIP4 | 0.76305652 | 1 | 0.1437886 | 145.4126972 | 218.2966013 |
| YHR014W | SPO13 | 0.63051064 | 1 | 0.14395193 | 150.6262484 | 226.7744046 |
| YDL194W | SNF3 | 0.42368353 | 1 | 0.14424014 | 156.5838468 | 236.4488632 |
| YIL095W | PRK1 | 0.46232488 | 1 | 0.14434394 | 155.7127728 | 235.0153039 |
| YIL170W | HXT12 | 0.37988948 | 1 | 0.14448785 | 160.6963283 | 243.1207023 |
| YJL128C | PBS2 | 0.3763604 | 1 | 0.14454274 | 165.7963877 | 251.4276362 |
| YAR040C | YAR040C | 0.59530773 | 1 | 0.14510886 | 134.9686515 | 201.0985629 |
| YDR333C | YDR333C | 0.3049944 | 1 | 0.14534189 | 155.3200584 | 234.2449389 |
| YOR179C | SYC1 | 0.77186441 | 1 | 0.14537608 | 125.3565405 | 185.3940899 |
| YBR072W | HSP26 | 0.63506141 | 1 | 0.14539896 | 164.686485 | 249.5065995 |
| YDL123W | SNA4 | 0.53001782 | 1 | 0.14564358 | 157.1948416 | 237.261852 |
| YBR150C | TBS1 | 0.6629027 | 1 | 0.14575066 | 141.0962622 | 211.0040552 |
| YNL217W | YNL217W | 0.68805427 | 1 | 0.14577494 | 136.4925761 | 203.4959798 |
| YKR057W | RPS21A | 0.81194405 | 1 | 0.14591339 | 121.4417639 | 178.9421574 |
| YBR166C | TYR1 | 0.62587443 | 1 | 0.14596837 | 157.5686625 | 237.8288897 |
| YBL063W | KIP1 | 0.66360091 | 1 | 0.14612934 | 157.6588068 | 237.9548467 |
| YHR029C | YHI9 | 0.62665129 | 1 | 0.14628963 | 158.5005661 | 239.3061728 |
| YBR075W | YBR075W | 0.60820863 | 1 | 0.14636301 | 143.3271035 | 214.5608926 |
| YGR229C | SMI1 | 0.81072709 | 1 | 0.14713471 | 89.83658751 | 127.2602473 |
| YDR029W | YDR029W | 0.79633055 | 1 | 0.14734998 | 146.4733684 | 219.5611884 |
| YHR142W | CHS7 | 0.64150095 | 1 | 0.14795341 | 150.3841203 | 225.8577718 |
| YJR074W | MOG1 | 0.39688525 | 1 | 0.14796587 | 132.7324771 | 197.0805187 |
| YBR019C | GAL10 | 0.60107486 | 1 | 0.14821246 | 147.5576606 | 221.2163015 |
| YBR151W | APD1 | 0.6550344 | 1 | 0.14838692 | 152.226388 | 228.8044849 |
| YAL020C | ATS1 | 0.59718783 | 1 | 0.14840253 | 137.283937 | 204.4433341 |
| YHL046C | PAU13 | 0.59307228 | 1 | 0.14872342 | 140.1187804 | 209.0228282 |
| YNL286W | CUS2 | 0.73648398 | 1 | 0.14878374 | 121.5740057 | 178.7833554 |

| | | | | | | |
|---|---|---|---|---|---|---|
| YAL053W | FLC2 | 0.64946009 | 1 | 0.14891579 | 150.6259989 | 226.1265567 |
| YKR092C | SRP40 | 0.7590878 | 1 | 0.14904016 | 81.76706869 | 113.8568254 |
| YCL047C | YCL047C | 0.21868373 | 1 | 0.1491377 | 156.1661157 | 235.1290852 |
| YGR021W | YGR021W | 0.59711501 | 1 | 0.14925756 | 135.0363345 | 200.6677809 |
| YPL262W | FUM1 | 0.74612634 | 1 | 0.1493407 | 122.5557956 | 180.3112198 |
| YDL131W | LYS21 | 0.48934882 | 1 | 0.14936481 | 147.9326496 | 221.6773046 |
| YJL187C | SWE1 | 0.41750471 | 1 | 0.14996579 | 159.2633375 | 240.0701529 |
| YIL032C | YIL032C | 0.69265885 | 1 | 0.15069569 | 164.4358027 | 248.4070804 |
| YPR039W | YPR039W | 0.79422672 | 1 | 0.15074781 | 145.2567782 | 217.1347256 |
| YJL131C | YJL131C | 0.39462311 | 1 | 0.15087601 | 156.478258 | 235.4112084 |
| YLR089C | ALT1 | 0.80721336 | 1 | 0.15114723 | 55.79946471 | 71.24972949 |
| YPR156C | TPO3 | 0.78870425 | 1 | 0.15146248 | 134.7702829 | 199.9464739 |
| YBR262C | FMP51 | 0.50207606 | 1 | 0.15155679 | 154.7686571 | 232.5354302 |
| YHR210C | YHR210C | 0.56571505 | 1 | 0.15172847 | 140.5170222 | 209.2800885 |
| YJL051W | IRC8 | 0.47224555 | 1 | 0.15178993 | 159.7659233 | 240.6515413 |
| YHR034C | PIH1 | 0.49162373 | 1 | 0.15195773 | 110.5811778 | 160.4489108 |
| YNR044W | AGA1 | 0.78916208 | 1 | 0.15198321 | 133.4801501 | 197.7753861 |
| YBR141C | YBR141C | 0.69888341 | 1 | 0.15204159 | 151.4390582 | 227.0443011 |
| YBL064C | PRX1 | 0.6521511 | 1 | 0.15231117 | 156.3080994 | 234.9466278 |
| YJR087W | YJR087W | 0.76922138 | 1 | 0.15252435 | 114.0799114 | 166.0786254 |
| YPR004C | YPR004C | 0.81448692 | 1 | 0.15272844 | 133.5180939 | 197.7400413 |
| YDR253C | MET32 | 0.39705297 | 1 | 0.15310875 | 150.3454275 | 225.1222781 |
| YPR062W | FCY1 | 0.78611231 | 1 | 0.15323066 | 130.5233843 | 192.7925736 |
| YEL061C | CIN8 | 0.68227746 | 1 | 0.15331103 | 110.9571738 | 160.8853452 |
| YHR015W | MIP6 | 0.57090903 | 1 | 0.15336924 | 137.5505659 | 204.2301782 |
| YDL040C | NAT1 | 0.34129848 | 1 | 0.15351008 | 136.8194459 | 203.0199395 |
| YNL299W | TRF5 | 0.79744912 | 1 | 0.15353736 | 117.2716513 | 171.1496568 |
| YDR146C | SWI5 | 0.29836789 | 1 | 0.15368169 | 146.1917192 | 218.2761929 |
| YHR001W-A | QCR10 | 0.61884225 | 1 | 0.15369455 | 145.3808703 | 216.9526736 |
| YHR206W | SKN7 | 0.50701627 | 1 | 0.15379267 | 165.3066333 | 249.422762 |
| YIL015C-A | YIL015C-A | 0.58826321 | 1 | 0.15404487 | 135.2343834 | 200.366225 |
| YPL267W | ACM1 | 0.7931389 | 1 | 0.15407289 | 140.3976543 | 208.7797107 |
| YNR050C | LYS9 | 0.73146609 | 1 | 0.15420221 | 118.6553589 | 173.3186534 |
| YHL029C | OCA5 | 0.48333407 | 1 | 0.15424288 | 152.6900306 | 228.7965133 |
| YLR373C | VID22 | 0.78585881 | 1 | 0.15455396 | 55.67420275 | 70.60118392 |
| YOL108C | INO4 | 0.71293281 | 1 | 0.15464783 | 108.0071862 | 155.9019288 |
| YIL005W | EPS1 | 0.64641298 | 1 | 0.15482976 | 144.97686 | 216.1459906 |
| YJL021C | YJL021C | 0.35511397 | 1 | 0.15484392 | 163.6336849 | 246.5584136 |
| YPR127W | YPR127W | 0.78876632 | 1 | 0.15506989 | 135.8972543 | 201.3131394 |
| YML063W | RPS1B | 0.76648461 | 1 | 0.15515124 | 96.76796658 | 137.5141437 |
| YPR194C | OPT2 | 0.75087373 | 1 | 0.15535121 | 104.1315429 | 149.4921305 |
| YIR043C | YIR043C | 0.38703264 | 1 | 0.15556423 | 156.7673278 | 235.2709589 |
| YPL156C | PRM4 | 0.4069975 | 1 | 0.15557701 | 133.865595 | 197.9349926 |
| YBR084W | MIS1 | 0.67217078 | 1 | 0.15558397 | 157.3845497 | 236.2745768 |
| YDR241W | BUD26 | 0.44749244 | 1 | 0.15561112 | 116.9171861 | 170.3013262 |
| YCR106W | RDS1 | 0.34550305 | 1 | 0.15572526 | 152.9301251 | 228.9945652 |
| YCR100C | YCR100C | 0.36483616 | 1 | 0.15581389 | 155.020797 | 232.3912088 |
| YGR276C | RNH70 | 0.76608089 | 1 | 0.15641926 | 130.224259 | 191.8890487 |
| YBR068C | BAP2 | 0.6914616 | 1 | 0.15650957 | 150.0787983 | 224.2440478 |
| YGR272C | YGR272C | 0.76777438 | 1 | 0.15655313 | 135.1323009 | 199.8726551 |
| YHL047C | ARN2 | 0.51344341 | 1 | 0.1566227 | 147.0939506 | 219.3634067 |
| YGR213C | RTA1 | 0.61019826 | 1 | 0.15690304 | 146.5870416 | 218.5004816 |
| YPL154C | PEP4 | 0.81843174 | 1 | 0.15711262 | 142.4399148 | 211.712518 |
| YAL034C | FUN19 | 0.6068164 | 1 | 0.15768785 | 160.8894237 | 241.7137945 |
| YPL259C | APM1 | 0.80988971 | 1 | 0.15789886 | 154.3909976 | 231.0925684 |
| YBR043C | QDR3 | 0.63095017 | 1 | 0.15797138 | 139.1211725 | 206.190311 |
| YDR358W | GGA1 | 0.39719537 | 1 | 0.15805364 | 161.0222999 | 241.882699 |
| YHR121W | LSM12 | 0.5663339 | 1 | 0.1581178 | 142.3913707 | 211.5022753 |
| YEL003W | GIM4 | 0.54790777 | 1 | 0.15819554 | 101.4277007 | 144.7133506 |
| YIL079C | AIR1 | 0.39822367 | 1 | 0.15833884 | 157.6688389 | 236.3787026 |
| YGL066W | SGF73 | 0.58575643 | 1 | 0.15841267 | 110.9414933 | 160.1943705 |
| YDR320C | SWA2 | 0.33302163 | 1 | 0.15871646 | 158.8919564 | 238.3233705 |
| YGR248W | SOL4 | 0.59345753 | 1 | 0.15898559 | 143.6218938 | 213.3950821 |
| YJR053W | BFA1 | 0.23629421 | 1 | 0.159346 | 150.8298944 | 225.0985223 |

| | | | | | | |
|---|---|---|---|---|---|---|
| YCR006C | YCR006C | 0.27906582 | 1 | 0.15936655 | 158.9454659 | 238.3258084 |
| YOR316C | COT1 | 0.82309679 | 1 | 0.15938359 | 158.2295213 | 237.1564572 |
| YGR242W | YGR242W | 0.61319871 | 1 | 0.15957068 | 152.0877737 | 227.119806 |
| YPR134W | MSS18 | 0.75200624 | 1 | 0.15957131 | 123.4155464 | 180.3783916 |
| YBR064W | YBR064W | 0.62882723 | 1 | 0.15962832 | 152.6111136 | 227.9654336 |
| YCL057W | PRD1 | 0.52417181 | 1 | 0.15987312 | 159.5913225 | 239.3126094 |
| YGR201C | YGR201C | 0.57762001 | 1 | 0.16009876 | 156.4549161 | 234.1702228 |
| YPL264C | YPL264C | 0.80593415 | 1 | 0.16018991 | 138.2584628 | 204.4945604 |
| YPR111W | DBF20 | 0.77251124 | 1 | 0.1602968 | 138.0943715 | 204.2131177 |
| YHR181W | SVP26 | 0.57500042 | 1 | 0.16035469 | 148.8007504 | 221.6590571 |
| YGR184C | UBR1 | 0.47955662 | 1 | 0.16038307 | 142.1416525 | 210.7997248 |
| YCR098C | GIT1 | 0.54974358 | 1 | 0.1604468 | 154.188575 | 230.4302488 |
| YNL045W | YNL045W | 0.69819954 | 1 | 0.16095478 | 114.0040221 | 164.8553204 |
| YBR090C | YBR090C | 0.60778972 | 1 | 0.16105273 | 161.3235584 | 241.9826336 |
| YDL226C | GCS1 | 0.49015756 | 1 | 0.16107211 | 127.4284276 | 186.7244203 |
| YBR283C | SSH1 | 0.29773024 | 1 | 0.16175598 | 150.141755 | 223.662384 |
| YNL104C | LEU4 | 0.74704518 | 1 | 0.16176348 | 129.0812341 | 189.3286422 |
| YJL058C | BIT61 | 0.30886457 | 1 | 0.16213669 | 156.4503348 | 233.896946 |
| YGR173W | RBG2 | 0.49706275 | 1 | 0.16219389 | 154.8388901 | 231.2625155 |
| YBR204C | YBR204C | 0.60318321 | 1 | 0.16223928 | 153.0733788 | 228.3784659 |
| YAL045C | YAL045C | 0.56431309 | 1 | 0.16231605 | 151.513247 | 225.8251339 |
| YGR270W | YTA7 | 0.47519201 | 1 | 0.16250401 | 120.2728098 | 174.8726008 |
| YIL139C | REV7 | 0.44704454 | 1 | 0.16271809 | 153.9436792 | 229.7347745 |
| YHR003C | YHR003C | 0.54093588 | 1 | 0.16300003 | 152.0068767 | 226.5406339 |
| YPL055C | LGE1 | 0.78438214 | 1 | 0.16331804 | 118.1777706 | 171.3511029 |
| YFL013C | IES1 | 0.73704001 | 1 | 0.16338733 | 34.06724951 | 34.22548242 |
| YNL301C | RPL18B | 0.82862933 | 1 | 0.16338898 | 138.4955084 | 204.4637328 |
| YGR122W | YGR122W | 0.76032101 | 1 | 0.1633899 | 133.8589282 | 196.9050813 |
| YBR030W | YBR030W | 0.62871449 | 1 | 0.16354186 | 156.3200387 | 233.501259 |
| YHL012W | YHL012W | 0.57964804 | 1 | 0.16361368 | 144.9069317 | 214.8862972 |
| YOR276W | CAF20 | 0.76820776 | 1 | 0.1636575 | 137.884105 | 203.4320028 |
| YDR257C | SET7 | 0.34143368 | 1 | 0.16366842 | 149.5139089 | 222.3894303 |
| YOR106W | VAM3 | 0.76474694 | 1 | 0.16377643 | 85.44332128 | 117.9277664 |
| YHR198C | FMP22 | 0.52208647 | 1 | 0.16392456 | 142.4996518 | 210.9214121 |
| YDR414C | ERD1 | 0.32742608 | 1 | 0.16440484 | 157.2272397 | 234.8676154 |
| YBR077C | SLM4 | 0.52114215 | 1 | 0.16479118 | 157.3296838 | 234.9842278 |
| YAR035W | YAT1 | 0.60714591 | 1 | 0.16546713 | 154.7467972 | 230.6854539 |
| YGR283C | YGR283C | 0.50030098 | 1 | 0.16580547 | 155.1134377 | 231.2390188 |
| YBR111C | YSA1 | 0.65657464 | 1 | 0.16614293 | 157.038329 | 234.3329526 |
| YDL025C | YDL025C | 0.39027084 | 1 | 0.16622553 | 158.5826569 | 236.8397356 |
| YHL006C | SHU1 | 0.57463184 | 1 | 0.16675922 | 143.5548777 | 212.2719086 |
| YNL166C | BNI5 | 0.7219074 | 1 | 0.16691144 | 146.2890337 | 216.7092632 |
| YBL049W | MOH1 | 0.6493631 | 1 | 0.16711683 | 161.0469903 | 240.7408269 |
| YJL112W | MDV1 | 0.39991704 | 1 | 0.16742109 | 155.7803123 | 232.1154273 |
| YPL069C | BTS1 | 0.76635642 | 1 | 0.16753648 | 49.19323024 | 58.3426077 |
| YDL134C | PPH21 | 0.55596248 | 1 | 0.16769101 | 145.6840428 | 215.6213299 |
| YHR159W | YHR159W | 0.57403117 | 1 | 0.16770618 | 149.7139838 | 222.1889418 |
| YAL040C | CLN3 | 0.60612813 | 1 | 0.1677161 | 154.4865624 | 229.9678845 |
| YHR161C | YAP1801 | 0.64476658 | 1 | 0.16803799 | 152.9391644 | 227.4033389 |
| YHR006W | STP2 | 0.58608948 | 1 | 0.16810258 | 145.1106054 | 214.6328336 |
| YIL017C | VID28 | 0.61999317 | 1 | 0.16831226 | 131.0075323 | 191.6147198 |
| YBR006W | UGA2 | 0.59526771 | 1 | 0.16849202 | 134.0046192 | 196.4771105 |
| YIR032C | DAL3 | 0.41327384 | 1 | 0.16849628 | 162.754834 | 243.3450225 |
| YGR169C | PUS6 | 0.59729213 | 1 | 0.16854407 | 160.5821483 | 239.7968868 |
| YAR028W | YAR028W | 0.63109659 | 1 | 0.16862332 | 141.6163282 | 208.8685583 |
| YOR221C | MCT1 | 0.72922876 | 1 | 0.16893102 | 121.8573881 | 176.6174915 |
| YAL008W | FUN14 | 0.62556789 | 1 | 0.16953521 | 155.2569554 | 230.9865068 |
| YGR291C | YGR291C | 0.74058031 | 1 | 0.16965364 | 134.7694507 | 197.5724243 |
| YGR256W | GND2 | 0.58602521 | 1 | 0.16977578 | 151.1952778 | 224.3338013 |
| YBR203W | COS111 | 0.58184818 | 1 | 0.17013701 | 160.3155201 | 239.154462 |
| YGR078C | PAC10 | 0.61317822 | 1 | 0.17026738 | 119.5785003 | 172.7281566 |
| YBR083W | TEC1 | 0.58654005 | 1 | 0.17036628 | 146.5900597 | 216.7493755 |
| YDR277C | MTH1 | 0.34609223 | 1 | 0.17110412 | 157.6378975 | 234.6632735 |
| YIL066C | RNR3 | 0.34433165 | 1 | 0.17150077 | 157.3665259 | 234.1691481 |

| | | | | | | |
|---|---|---|---|---|---|---|
| YHR135C | YCK1 | 0.5412676 | 1 | 0.17166735 | 151.5300653 | 224.6328499 |
| YBR020W | GAL1 | 0.61356829 | 1 | 0.17200557 | 143.5954394 | 211.6537456 |
| YDR095C | YDR095C | 0.28503506 | 1 | 0.1720138 | 172.0357759 | 258.0159771 |
| YJL095W | BCK1 | 0.2939491 | 1 | 0.17207803 | 155.2611335 | 230.6616545 |
| YDR265W | PEX10 | 0.43824071 | 1 | 0.17223494 | 154.2931896 | 229.0632511 |
| YBR182C | SMP1 | 0.5972582 | 1 | 0.17249542 | 150.4920588 | 222.8326907 |
| YBR084C-A | RPL19A | 0.65055462 | 1 | 0.17303864 | 142.7931985 | 210.2111913 |
| YIL131C | FKH1 | 0.80114733 | 1 | 0.17308711 | 139.786801 | 205.3038538 |
| YBR272C | HSM3 | 0.33445332 | 1 | 0.17312328 | 163.9044069 | 244.6155466 |
| YHR114W | BZZ1 | 0.61215126 | 1 | 0.17334792 | 156.3544366 | 232.2783199 |
| YCR082W | AHC2 | 0.48376555 | 1 | 0.17371629 | 168.6026347 | 252.1972289 |
| YHR191C | CTF8 | 0.5352512 | 1 | 0.1737345 | 124.1520798 | 179.7317636 |
| YDR048C | YDR048C | 0.21623797 | 1 | 0.17477697 | 150.7753784 | 222.9969722 |
| YBR041W | FAT1 | 0.48601458 | 1 | 0.17519103 | 124.0452991 | 179.3677144 |
| YJR020W | YJR020W | 0.3844182 | 1 | 0.17651626 | 151.7338711 | 224.3326448 |
| YGR212W | SLI1 | 0.53803712 | 1 | 0.17700326 | 152.4807782 | 225.4867293 |
| YIL153W | RRD1 | 0.35583171 | 1 | 0.17700816 | 142.1347678 | 208.6200725 |
| YGL174W | BUD13 | 0.33411731 | 1 | 0.1772423 | 88.87567799 | 121.7668108 |
| YBR245C | ISW1 | 0.43241027 | 1 | 0.17786933 | 164.8705721 | 245.5715513 |
| YIL056W | VHR1 | 0.53913747 | 1 | 0.17793128 | 150.8355772 | 222.6836873 |
| YHL021C | FMP12 | 0.53363922 | 1 | 0.1781891 | 146.9600799 | 216.3322417 |
| YNL037C | IDH1 | 0.68232176 | 1 | 0.17834795 | 115.4047058 | 164.8700983 |
| YIR009W | MSL1 | 0.33146346 | 1 | 0.17834886 | 160.4395359 | 238.285551 |
| YBR177C | EHT1 | 0.61932013 | 1 | 0.17858651 | 156.8815633 | 232.4543643 |
| YNL147W | LSM7 | 0.69804195 | 1 | 0.17886618 | 103.9131305 | 146.0689921 |
| YDR385W | EFT2 | 0.38711379 | 1 | 0.17921863 | 148.6400179 | 218.9365863 |
| YAR042W | SWH1 | 0.53154256 | 1 | 0.17924959 | 149.7280674 | 220.7062817 |
| YHR087W | RTC3 | 0.48033782 | 1 | 0.17970414 | 144.1620359 | 211.5732758 |
| YHR094C | HXT1 | 0.50665087 | 1 | 0.17981183 | 149.8031807 | 220.7553979 |
| YBR073W | RDH54 | 0.60020792 | 1 | 0.1799095 | 139.3853959 | 203.7596337 |
| YDR289C | RTT103 | 0.39723813 | 1 | 0.17995401 | 152.8121846 | 225.6421164 |
| YAL068C | PAU8 | 0.5392426 | 1 | 0.18007252 | 151.0809639 | 222.8044314 |
| YCL051W | LRE1 | 0.25138804 | 1 | 0.18043887 | 162.2890103 | 241.0279543 |
| YIL067C | YIL067C | 0.35763054 | 1 | 0.18071652 | 144.2592282 | 211.5996716 |
| YJL068C | YJL068C | 0.26787257 | 1 | 0.18076993 | 162.1745363 | 240.7981582 |
| YKL137W | YKL137W | 0.68047048 | 1 | 0.18082706 | 122.0954235 | 175.4539225 |
| YHR109W | CTM1 | 0.53152741 | 1 | 0.18103292 | 154.0701295 | 227.5520899 |
| YPR089W | YPR089W | 0.69248569 | 1 | 0.18151215 | 113.634904 | 161.5722655 |
| YBR024W | SCO2 | 0.61963798 | 1 | 0.1815553 | 158.7537521 | 235.1191745 |
| YHL039W | YHL039W | 0.5340043 | 1 | 0.18168173 | 151.9404529 | 223.9956754 |
| YIL073C | SPO22 | 0.1950934 | 1 | 0.18176058 | 151.2765961 | 222.9031744 |
| YAR044W | OSH1 | 0.50095676 | 1 | 0.18186232 | 152.2770003 | 224.5207596 |
| YPR192W | AQY1 | 0.78328963 | 1 | 0.1819048 | 141.5367096 | 207.006446 |
| YJL217W | YJL217W | 0.18572246 | 1 | 0.18201825 | 155.9621945 | 230.5080076 |
| YHL041W | YHL041W | 0.58434653 | 1 | 0.18239789 | 146.3914139 | 214.8562479 |
| YCL022C | YCL022C | 0.23682078 | 1 | 0.18244007 | 167.5860398 | 249.4021273 |
| YBR218C | PYC2 | 0.62437181 | 1 | 0.18283488 | 160.4969033 | 237.7939553 |
| YCR076C | YCR076C | 0.26156442 | 1 | 0.18296028 | 166.6994158 | 247.8889063 |
| YGR199W | PMT6 | 0.56331546 | 1 | 0.18326703 | 151.3527304 | 222.8308009 |
| YDL190C | UFD2 | 0.12990023 | 1 | 0.18384339 | 155.033879 | 228.7566169 |
| YJR049C | UTR1 | 0.31005577 | 1 | 0.18418683 | 157.6802792 | 233.0259714 |
| YBR207W | FTH1 | 0.55565785 | 1 | 0.18420388 | 158.3113628 | 234.0525367 |
| YJL216C | YJL216C | 0.33202266 | 1 | 0.18430886 | 157.9479589 | 233.4464251 |
| YBL100C | YBL100C | 0.5892994 | 1 | 0.18443747 | 151.5100802 | 222.93465 |
| YDR209C | YDR209C | 0.27310089 | 1 | 0.18464881 | 161.4776952 | 239.1562442 |
| YJL171C | YJL171C | 0.31008893 | 1 | 0.18487304 | 161.6916411 | 239.4757716 |
| YAL029C | MYO4 | 0.59052346 | 1 | 0.185211 | 162.3482438 | 240.5020814 |
| YBL001C | ECM15 | 0.60499486 | 1 | 0.18548624 | 159.1911539 | 235.3195087 |
| YAL015C | NTG1 | 0.52231626 | 1 | 0.1854945 | 152.2196766 | 223.9535615 |
| YDR157W | YDR157W | 0.17374141 | 1 | 0.18589108 | 162.3350889 | 240.3919327 |
| YAL007C | ERP2 | 0.5113013 | 1 | 0.18617468 | 152.3968449 | 224.1536634 |
| YPR061C | JID1 | 0.72760494 | 1 | 0.18618227 | 132.732133 | 192.0953508 |
| YCR026C | NPP1 | 0.29111547 | 1 | 0.18627699 | 145.3725929 | 212.6894152 |
| YBR301W | DAN3 | 0.20055888 | 1 | 0.18633757 | 164.5465983 | 243.9388895 |

| | | | | | | |
|---|---|---|---|---|---|---|
| YDR221W | GTB1 | 0.37665873 | 1 | 0.186577 | 161.0453113 | 238.1998781 |
| YDL133W | YDL133W | 0.19421417 | 1 | 0.18662284 | 170.8500188 | 254.177488 |
| YBL053W | YBL053W | 0.59491634 | 1 | 0.18683836 | 148.50229 | 217.7182139 |
| YDL037C | BSC1 | 0.47038594 | 1 | 0.1870077 | 156.1836103 | 230.218179 |
| YAR031W | PRM9 | 0.59532094 | 1 | 0.18707019 | 158.7737159 | 234.4324066 |
| YBR050C | REG2 | 0.59057408 | 1 | 0.18727366 | 139.6874079 | 203.2914562 |
| YDR144C | MKC7 | 0.29919968 | 1 | 0.18727777 | 158.3689481 | 233.7454813 |
| YIL013C | PDR11 | 0.49670372 | 1 | 0.18777348 | 158.1075736 | 233.2547341 |
| YBL060W | YBL060W | 0.610593 | 1 | 0.18812325 | 154.7349061 | 227.7110063 |
| YBR216C | YBP1 | 0.57523215 | 1 | 0.18818293 | 150.4127408 | 220.6572474 |
| YGR239C | PEX21 | 0.46642442 | 1 | 0.18868658 | 147.3793601 | 215.6465529 |
| YPR197C | YPR197C | 0.73841344 | 1 | 0.18916853 | 144.106953 | 210.2490288 |
| YDR042C | YDR042C | 0.72133343 | 1 | 0.18937665 | 130.3345084 | 187.7701074 |
| YOR277C | YOR277C | 0.64931647 | 1 | 0.18982992 | 138.7885748 | 201.4927668 |
| YDL171C | GLT1 | 0.2125842 | 1 | 0.18993597 | 150.4677542 | 220.5182787 |
| YHR126C | YHR126C | 0.47800088 | 1 | 0.1900705 | 146.5790938 | 214.1614561 |
| YDL122W | UBP1 | 0.36571094 | 1 | 0.19015332 | 160.8793752 | 237.4629062 |
| YDR247W | VHS1 | 0.30357908 | 1 | 0.19036924 | 150.1228905 | 219.8995716 |
| YIL092W | YIL092W | 0.2557857 | 1 | 0.19077308 | 150.0890784 | 219.7917788 |
| YIL002C | INP51 | 0.51493378 | 1 | 0.19103897 | 155.9438236 | 229.3014775 |
| YBL102W | SFT2 | 0.58369547 | 1 | 0.19140911 | 159.1778594 | 234.5253098 |
| YHR127W | YHR127W | 0.51192559 | 1 | 0.19179155 | 153.2411206 | 224.7973826 |
| YBR147W | RTC2 | 0.65920029 | 1 | 0.19181526 | 100.9432297 | 139.538511 |
| YHR150W | PEX28 | 0.5212944 | 1 | 0.19185153 | 145.8106473 | 212.6764369 |
| YBR001C | NTH2 | 0.5137834 | 1 | 0.19211203 | 154.029872 | 226.0414018 |
| YBL107C | YBL107C | 0.51961197 | 1 | 0.19212922 | 144.7929213 | 210.9811245 |
| YGR135W | PRE9 | 0.42204704 | 1 | 0.19217969 | 120.9952032 | 172.1796124 |
| YDR067C | OCA6 | 0.47896097 | 1 | 0.19252965 | 140.8060881 | 204.4295799 |
| YER057C | HMF1 | 0.66498289 | 1 | 0.19283591 | 117.2161101 | 165.9333609 |
| YAR037W | YAR037W | 0.52334256 | 1 | 0.19285749 | 162.1181071 | 239.129574 |
| YPL157W | TGS1 | 0.61052217 | 1 | 0.19372937 | 80.95546693 | 106.7048918 |
| YDR415C | YDR415C | 0.20129246 | 1 | 0.19406063 | 163.2090131 | 240.7510365 |
| YBR052C | RFS1 | 0.61917382 | 1 | 0.19420176 | 159.7261215 | 235.054835 |
| YBR287W | ZSP1 | 0.29966123 | 1 | 0.19420236 | 171.252829 | 253.8455421 |
| YGL237C | HAP2 | 0.44312083 | 1 | 0.19423515 | 105.5363961 | 146.7106394 |
| YBR250W | YBR250W | 0.15843784 | 1 | 0.19430207 | 160.1209468 | 235.6853947 |
| YAL019W | FUN30 | 0.55739934 | 1 | 0.19430986 | 151.9699127 | 222.3965993 |
| YIL006W | YIA6 | 0.57437751 | 1 | 0.19435813 | 154.077103 | 225.8254358 |
| YHR139C-A | YHR139C-A | 0.51182151 | 1 | 0.1943885 | 159.5209531 | 234.696014 |
| YBR056W | YBR056W | 0.59250345 | 1 | 0.19443729 | 171.368162 | 254.0029149 |
| YDR389W | SAC7 | 0.12100106 | 1 | 0.19445103 | 155.1777698 | 227.6076203 |
| YIL093C | RSM25 | 0.30345457 | 1 | 0.19452201 | 152.4331542 | 223.1241032 |
| YPR172W | YPR172W | 0.72708187 | 1 | 0.19452884 | 131.7826783 | 189.4589017 |
| YBL095W | YBL095W | 0.54761658 | 1 | 0.19455786 | 148.7639946 | 217.1379799 |
| YAL066W | YAL066W | 0.57704436 | 1 | 0.19476651 | 153.1919749 | 224.3292391 |
| YJL027C | YJL027C | 0.36040522 | 1 | 0.19493747 | 165.1941385 | 243.8728124 |
| YHL026C | YHL026C | 0.4882052 | 1 | 0.19504756 | 141.896728 | 205.8791215 |
| YHL008C | YHL008C | 0.52868568 | 1 | 0.19505197 | 147.6663133 | 215.2840975 |
| YGR284C | ERV29 | 0.53077409 | 1 | 0.19511083 | 148.0993995 | 215.9824362 |
| YGR196C | FYV8 | 0.53344872 | 1 | 0.1952408 | 158.934947 | 233.6295425 |
| YMR194C-A | YMR194C-A | 0.72576023 | 1 | 0.19526803 | 137.7611875 | 199.1086263 |
| YBR201W | DER1 | 0.49945338 | 1 | 0.19545003 | 158.7568732 | 233.3119572 |
| YDR345C | HXT3 | 0.36460304 | 1 | 0.19563278 | 164.9782814 | 243.4302321 |
| YDL214C | PRR2 | 0.21189359 | 1 | 0.19573742 | 158.2585779 | 232.4621547 |
| YHR031C | RRM3 | 0.43788099 | 1 | 0.19601897 | 141.5877427 | 205.2487131 |
| YIL064W | YIL064W | 0.27796093 | 1 | 0.19605175 | 151.3715209 | 221.1939079 |
| YDL222C | FMP45 | 0.17183264 | 1 | 0.19649003 | 156.4573932 | 229.4277074 |
| YGR183C | QCR9 | 0.51760861 | 1 | 0.19658732 | 138.3903549 | 199.9622164 |
| YIR036C | IRC24 | 0.27611998 | 1 | 0.19662911 | 158.6665253 | 233.0108851 |
| YAL051W | OAF1 | 0.56616383 | 1 | 0.19665904 | 169.9730528 | 251.4388296 |
| YCR065W | HCM1 | 0.29770024 | 1 | 0.1966768 | 142.7288328 | 207.0231114 |
| YBR046C | ZTA1 | 0.57412706 | 1 | 0.19690897 | 160.8120949 | 236.472079 |
| YPL054W | LEE1 | 0.77565924 | 1 | 0.19716938 | 137.4834538 | 198.4078716 |
| YJL185C | YJL185C | 0.2640301 | 1 | 0.19749937 | 167.425285 | 247.1758649 |

| | | | | | | |
|---|---|---|---|---|---|---|
| YDR401W | YDR401W | 0.34567007 | 1 | 0.19774015 | 170.5777353 | 252.2835694 |
| YHR115C | DMA1 | 0.52170644 | 1 | 0.19784995 | 160.4747159 | 235.7993535 |
| YIL030C | SSM4 | 0.54380702 | 1 | 0.19801228 | 154.3970127 | 225.8703369 |
| YHR203C | RPS4B | 0.48053744 | 1 | 0.19803341 | 117.9151967 | 166.3950922 |
| YHL002W | HSE1 | 0.52129454 | 1 | 0.19804044 | 150.1922509 | 219.0120799 |
| YDR422C | SIP1 | 0.27211857 | 1 | 0.19828526 | 164.1253307 | 241.6937899 |
| YHR158C | KEL1 | 0.44477496 | 1 | 0.19841288 | 141.6243307 | 204.9961188 |
| YDL066W | IDP1 | 0.16573346 | 1 | 0.19850223 | 169.6966359 | 250.747807 |
| YHR207C | SET5 | 0.58427679 | 1 | 0.19883367 | 167.7254317 | 247.4911281 |
| YJR090C | GRR1 | 0.75092086 | 1 | 0.19911071 | 40.57068051 | 40.1679054 |
| YCR059C | YIH1 | 0.40331658 | 1 | 0.19982307 | 156.6135207 | 229.2474945 |
| YJL078C | PRY3 | 0.24168177 | 1 | 0.2000992 | 156.0092775 | 228.2264437 |
| YPR098C | YPR098C | 0.72454359 | 1 | 0.2001449 | 134.77332 | 193.6017236 |
| YBR018C | GAL7 | 0.47360546 | 1 | 0.20030132 | 151.3979073 | 220.6826469 |
| YGR263C | SAY1 | 0.482762 | 1 | 0.20038537 | 148.6508576 | 216.1934559 |
| YBR057C | MUM2 | 0.52138517 | 1 | 0.20044728 | 148.0569262 | 215.2171573 |
| YBR161W | CSH1 | 0.47293459 | 1 | 0.20047034 | 153.1886695 | 223.5798933 |
| YCR037C | PHO87 | 0.13862228 | 1 | 0.20065712 | 170.9147242 | 252.4524639 |
| YPR006C | ICL2 | 0.71878439 | 1 | 0.20106138 | 141.4817224 | 204.418192 |
| YJL103C | GSM1 | 0.7229219 | 1 | 0.20111896 | 140.4000144 | 202.6472871 |
| YJL213W | YJL213W | 0.288996 | 1 | 0.20130911 | 146.9924117 | 213.3693804 |
| YBR082C | UBC4 | 0.39107577 | 1 | 0.20158009 | 125.1622562 | 177.7466182 |
| YBR015C | MNN2 | 0.51026827 | 1 | 0.20169484 | 145.1266964 | 210.2775893 |
| YCL062W | YCL062W | 0.40129748 | 1 | 0.20219909 | 139.4246293 | 200.9163364 |
| YHL028W | WSC4 | 0.49325313 | 1 | 0.20233879 | 163.8559338 | 240.7259138 |
| YDR281C | PHM6 | 0.19619914 | 1 | 0.20258438 | 157.3088777 | 230.0209009 |
| YDR058C | TGL2 | 0.17618111 | 1 | 0.20271218 | 144.8714009 | 209.728715 |
| YGR118W | RPS23A | 0.39417956 | 1 | 0.20297524 | 110.4763598 | 153.6237659 |
| YBL066C | SEF1 | 0.58993558 | 1 | 0.20302733 | 152.1484988 | 221.5507011 |
| YJL152W | YJL152W | 0.25735622 | 1 | 0.20309117 | 161.3424566 | 236.5303214 |
| YAR027W | UIP3 | 0.51314824 | 1 | 0.20319393 | 149.1760171 | 216.6832457 |
| YJL007C | YJL007C | 0.23403539 | 1 | 0.2033723 | 151.8045404 | 220.9449863 |
| YJL212C | OPT1 | 0.15605226 | 1 | 0.20356561 | 148.7857238 | 215.9985121 |
| YBR010W | HHT1 | 0.48972805 | 1 | 0.20370679 | 137.6293548 | 197.7930362 |
| YHL003C | LAG1 | 0.48418332 | 1 | 0.20418024 | 119.4756035 | 168.1371226 |
| YCR016W | YCR016W | 0.17880215 | 1 | 0.20419149 | 157.718794 | 230.4795273 |
| YIL154C | IMP2' | 0.36934715 | 1 | 0.20455707 | 136.9044825 | 196.5004507 |
| YAR014C | BUD14 | 0.424335 | 1 | 0.20458522 | 129.3042867 | 184.1069744 |
| YBR101C | FES1 | 0.57639506 | 1 | 0.20558649 | 159.935251 | 233.9108341 |
| YHR160C | PEX18 | 0.50271404 | 1 | 0.20611945 | 157.9170295 | 230.5512241 |
| YCR014C | POL4 | 0.1933513 | 1 | 0.20653693 | 163.1644825 | 239.0511461 |
| YDR352W | YDR352W | 0.30345243 | 1 | 0.20665104 | 155.4990487 | 226.5401077 |
| YPR090W | YPR090W | 0.71805227 | 1 | 0.20667627 | 142.7812316 | 205.8042902 |
| YGR290W | YGR290W | 0.51097639 | 1 | 0.20670173 | 152.8942683 | 222.2871944 |
| YCL039W | GID7 | 0.32845365 | 1 | 0.20698842 | 159.7184512 | 233.3745536 |
| YIL001W | YIL001W | 0.4583652 | 1 | 0.20711911 | 157.5228442 | 229.7782389 |
| YGR230W | BNS1 | 0.56160557 | 1 | 0.2072994 | 166.1464372 | 243.8128639 |
| YNL218W | MGS1 | 0.68159556 | 1 | 0.20731408 | 131.5585926 | 187.4260059 |
| YHR022C | YHR022C | 0.52862101 | 1 | 0.20743 | 143.1388684 | 206.2889972 |
| YHR016C | YSC84 | 0.6228387 | 1 | 0.20759435 | 159.0587212 | 232.2200321 |
| YOR358W | HAP5 | 0.69647596 | 1 | 0.20802917 | 111.7833076 | 155.0951568 |
| YGR234W | YHB1 | 0.50637381 | 1 | 0.2080584 | 153.2914574 | 222.7577386 |
| YJL020C | BBC1 | 0.29230183 | 1 | 0.20883253 | 164.6618557 | 241.1927391 |
| YBR053C | YBR053C | 0.54293245 | 1 | 0.20891975 | 152.7638761 | 221.7853309 |
| YDL081C | RPP1A | 0.05288317 | 0.80700955 | 0.20906143 | 97.98211041 | 132.4618708 |
| YGL105W | ARC1 | 0.42287939 | 1 | 0.20924547 | 70.96339339 | 88.39207869 |
| YIR019C | MUC1 | 0.17914859 | 1 | 0.21054699 | 149.1575034 | 215.6939962 |
| YGR221C | TOS2 | 0.542125 | 1 | 0.21069578 | 148.2149199 | 214.1379944 |
| YDR075W | PPH3 | 0.20491371 | 1 | 0.21069839 | 165.3382429 | 242.0520153 |
| YAL060W | BDH1 | 0.561625 | 1 | 0.21084004 | 160.7167823 | 234.4996571 |
| YGR209C | TRX2 | 0.39792986 | 1 | 0.21084898 | 141.1846808 | 202.6573487 |
| YBR128C | ATG14 | 0.49455594 | 1 | 0.21105471 | 153.1065204 | 222.0654428 |
| YIR023W | DAL81 | 0.24370515 | 1 | 0.21119285 | 147.7754296 | 213.3567053 |
| YAL023C | PMT2 | 0.45387138 | 1 | 0.21141394 | 151.803032 | 219.893647 |

| | | | | | | |
|---|---|---|---|---|---|---|
| YGR238C | KEL2 | 0.20111117 | 1 | 0.21145137 | 164.3589007 | 240.3572843 |
| YDL054C | MCH1 | 0.24825069 | 1 | 0.21179736 | 153.2829773 | 222.2562371 |
| YHR110W | ERP5 | 0.50322309 | 1 | 0.21185773 | 137.9443527 | 197.2434086 |
| YBL104C | YBL104C | 0.58973299 | 1 | 0.21196285 | 170.000626 | 249.4876865 |
| YIL146C | ECM37 | 0.26110409 | 1 | 0.21196834 | 146.1470853 | 210.6010383 |
| YBL028C | YBL028C | 0.48250218 | 1 | 0.21307879 | 152.6577588 | 221.0698707 |
| YDR305C | HNT2 | 0.39165856 | 1 | 0.21382795 | 141.5264288 | 202.825915 |
| YBL094C | YBL094C | 0.39070541 | 1 | 0.21449928 | 147.4871769 | 212.4555358 |
| YDR330W | UBX5 | 0.2260276 | 1 | 0.21465924 | 157.7332744 | 229.1378138 |
| YIL163C | YIL163C | 0.22770463 | 1 | 0.21507265 | 164.9405944 | 240.8332315 |
| YHR123W | EPT1 | 0.56929679 | 1 | 0.21525917 | 155.2314463 | 224.9810947 |
| YIL086C | YIL086C | 0.17003616 | 1 | 0.2153418 | 145.6525067 | 209.3547751 |
| YCR091W | KIN82 | 0.36322651 | 1 | 0.21546343 | 156.0001643 | 226.2076144 |
| YIL123W | SIM1 | 0.21433492 | 1 | 0.21558457 | 167.0187895 | 244.1543256 |
| YCL074W | YCL074W | 0.41529913 | 1 | 0.21602849 | 146.6814283 | 210.9425516 |
| YJR099W | YUH1 | 0.15073139 | 1 | 0.21639706 | 167.9399288 | 245.549988 |
| YIL042C | PKP1 | 0.55563834 | 1 | 0.21655866 | 151.9326233 | 219.4338756 |
| YIL124W | AYR1 | 0.19471028 | 1 | 0.2170888 | 154.7327747 | 223.9295217 |
| YDR270W | CCC2 | 0.31738727 | 1 | 0.21740638 | 169.2718464 | 247.5896281 |
| YGR214W | RPS0A | 0.21405851 | 1 | 0.217543 | 121.218729 | 169.2358379 |
| YDR321W | ASP1 | 0.21686726 | 1 | 0.21774725 | 162.8182275 | 237.0245079 |
| YHL010C | YHL010C | 0.51353661 | 1 | 0.2179765 | 153.720747 | 222.1639352 |
| YHL024W | RIM4 | 0.47060919 | 1 | 0.21804793 | 146.5546457 | 210.472474 |
| YDR388W | RVS167 | 0.2241994 | 1 | 0.21830218 | 132.6105841 | 187.7077669 |
| YGR226C | YGR226C | 0.53489043 | 1 | 0.21887758 | 163.969886 | 238.754506 |
| YBR130C | SHE3 | 0.47175833 | 1 | 0.21891286 | 153.526416 | 221.7250074 |
| YPL183W-A | RTC6 | 0.60821317 | 1 | 0.21908908 | 109.7304396 | 150.3060252 |
| YJR034W | PET191 | 0.71602036 | 1 | 0.21939785 | 147.447067 | 211.7512228 |
| YPR138C | MEP3 | 0.69470962 | 1 | 0.21986391 | 138.8240394 | 197.6332145 |
| YIR024C | YIR024C | 0.23249541 | 1 | 0.21988718 | 166.6571086 | 243.0035211 |
| YDL012C | YDL012C | 0.23146426 | 1 | 0.21990496 | 164.2822516 | 239.1297208 |
| YHR154W | RTT107 | 0.40963373 | 1 | 0.22012383 | 152.3898299 | 219.7142024 |
| YIL089W | YIL089W | 0.18401468 | 1 | 0.22014954 | 148.4657724 | 213.3138684 |
| YJL190C | RPS22A | 0.30562788 | 1 | 0.22027078 | 167.7653515 | 244.7601394 |
| YBL085W | BOI1 | 0.48947804 | 1 | 0.2202778 | 165.2639372 | 240.6814297 |
| YBL048W | YBL048W | 0.48264384 | 1 | 0.22029137 | 167.6117606 | 244.5070713 |
| YBR007C | DSF2 | 0.53992721 | 1 | 0.22061733 | 152.1013239 | 219.179513 |
| YBR085W | AAC3 | 0.69181841 | 1 | 0.22071353 | 137.3243572 | 195.0776226 |
| YHR132C | ECM14 | 0.46019947 | 1 | 0.22121277 | 156.9538206 | 227.0123667 |
| YMR282C | AEP2 | 0.56111614 | 1 | 0.22168364 | 103.0241152 | 139.0349941 |
| YKL053C-A | MDM35 | 0.60901293 | 1 | 0.22178277 | 101.8414614 | 137.0941076 |
| YOR379C | YOR379C | 0.71594805 | 1 | 0.2225181 | 148.0636619 | 212.3494164 |
| YDR318W | MCM21 | 0.16272205 | 1 | 0.22277578 | 160.6449562 | 232.8257748 |
| YCR021C | HSP30 | 0.03060751 | 0.74675374 | 0.22307349 | 149.6257051 | 214.8234114 |
| YBR222C | PCS60 | 0.48056933 | 1 | 0.22345763 | 164.8899536 | 239.657015 |
| YJL126W | NIT2 | 0.27394556 | 1 | 0.22361401 | 161.8404707 | 234.6653655 |
| YJR092W | BUD4 | 0.3133384 | 1 | 0.22375024 | 164.8151231 | 239.4968618 |
| YAR020C | PAU7 | 0.50933937 | 1 | 0.22376395 | 155.7094392 | 224.651029 |
| YBR129C | OPY1 | 0.45531062 | 1 | 0.22396085 | 141.1995969 | 200.9714689 |
| YCR073C | SSK22 | 0.11939711 | 0.99917458 | 0.2241584 | 158.6808097 | 229.4434948 |
| YDR112W | YDR112W | 0.20450091 | 1 | 0.22473501 | 163.6702835 | 237.5021047 |
| YJL165C | HAL5 | 0.25165774 | 1 | 0.22514095 | 143.1547999 | 204.00491 |
| YBR291C | CTP1 | 0.13312621 | 1 | 0.22525184 | 156.682235 | 226.0428092 |
| YBL061C | SKT5 | 0.50880941 | 1 | 0.22526389 | 152.1562222 | 218.6629527 |
| YDR162C | NBP2 | 0.07403027 | 0.85139088 | 0.22527443 | 139.8800906 | 198.6490839 |
| YBR162C | TOS1 | 0.50427042 | 1 | 0.22536289 | 155.1363261 | 223.5081916 |
| YDL128W | VCX1 | 0.09491277 | 0.90972195 | 0.22554956 | 171.8734677 | 250.7686542 |
| YBL008W | HIR1 | 0.48472353 | 1 | 0.22556893 | 145.9438409 | 208.4957697 |
| YBR062C | YBR062C | 0.47272664 | 1 | 0.22585955 | 148.9689694 | 213.3894152 |
| YBR212W | NGR1 | 0.50324045 | 1 | 0.22623542 | 147.9488918 | 211.677464 |
| YJR009C | TDH2 | 0.21844897 | 1 | 0.22674713 | 158.6641089 | 229.0786182 |
| YBR149W | ARA1 | 0.21011509 | 1 | 0.22691441 | 137.019679 | 193.7721508 |
| YBL101C | ECM21 | 0.45057271 | 1 | 0.22730114 | 154.7061301 | 222.5540797 |
| YOL071W | EMI5 | 0.14452242 | 1 | 0.22759334 | 130.9662072 | 183.8152551 |

| | | | | | | |
|---|---|---|---|---|---|---|
| YPR091C | YPR091C | 0.68098514 | 1 | 0.22764736 | 145.052271 | 206.7712453 |
| YDL161W | ENT1 | 0.09876341 | 0.91993506 | 0.22768272 | 153.0359288 | 219.7815553 |
| YHR171W | ATG7 | 0.3994009 | 1 | 0.22768539 | 138.9627616 | 196.8391944 |
| YDL160C | DHH1 | 0.08531751 | 0.89015714 | 0.22806877 | 175.3501125 | 256.1076823 |
| YHL014C | YLF2 | 0.42625773 | 1 | 0.22810444 | 137.8068414 | 194.9001626 |
| YIR016W | YIR016W | 0.13187241 | 1 | 0.22817292 | 156.4439123 | 225.2732964 |
| YIR037W | HYR1 | 0.13576308 | 1 | 0.22835634 | 160.328864 | 231.5826031 |
| YHR106W | TRR2 | 0.46976384 | 1 | 0.22844304 | 166.2449473 | 241.2156673 |
| YDR098C | GRX3 | 0.10943865 | 0.96297041 | 0.22880623 | 165.2220258 | 239.5007333 |
| YNL080C | EOS1 | 0.68412542 | 1 | 0.2291592 | 108.5710665 | 147.1025634 |
| YDL082W | RPL13A | 0.03477468 | 0.75034314 | 0.22960872 | 123.3748187 | 171.1769399 |
| YDR307W | YDR307W | 0.2065201 | 1 | 0.23025598 | 163.6617706 | 236.7681201 |
| YDR252W | BTT1 | 0.2226469 | 1 | 0.23028714 | 162.4738259 | 234.8274739 |
| YBL047C | EDE1 | 0.44199016 | 1 | 0.23039411 | 149.4434308 | 213.571432 |
| YBR022W | POA1 | 0.49638897 | 1 | 0.23042425 | 144.2187861 | 205.0503095 |
| YAL067C | SEO1 | 0.50624024 | 1 | 0.23083775 | 154.8583743 | 222.340984 |
| YGR295C | COS6 | 0.44272214 | 1 | 0.23094136 | 153.7477837 | 220.5169905 |
| YBR016W | YBR016W | 0.44379255 | 1 | 0.23112328 | 153.0416955 | 219.3422005 |
| YBR093C | PHO5 | 0.28843682 | 1 | 0.23145458 | 116.4829738 | 159.7011295 |
| YBR092C | PHO3 | 0.4960199 | 1 | 0.23162098 | 170.9949514 | 248.5445992 |
| YDR155C | CPR1 | 0.14390003 | 1 | 0.23175053 | 160.8288707 | 231.9550042 |
| YBR099C | YBR099C | 0.4355661 | 1 | 0.23237728 | 149.1064729 | 212.7634574 |
| YDL133C-A | RPL41B | 0.05094244 | 0.80681531 | 0.23248203 | 142.772354 | 202.4239439 |
| YPR017C | DSS4 | 0.68355476 | 1 | 0.23272245 | 138.9468148 | 196.1562091 |
| YBR074W | YBR074W | 0.4886937 | 1 | 0.23283628 | 170.4756599 | 247.5395398 |
| YBL091C | MAP2 | 0.49757883 | 1 | 0.23338488 | 167.6753984 | 242.9030115 |
| YBL043W | ECM13 | 0.48728619 | 1 | 0.23364014 | 172.7024973 | 251.0648719 |
| YBR090C-A | YBR090C-A | 0.45918491 | 1 | 0.23373628 | 151.9129569 | 217.1613191 |
| YJR025C | BNA1 | 0.22106886 | 1 | 0.23379969 | 145.0990527 | 206.045054 |
| YDR097C | MSH6 | 0.25538572 | 1 | 0.23382866 | 140.7313766 | 198.9211094 |
| YBR113W | YBR113W | 0.52583043 | 1 | 0.23390081 | 157.7489333 | 226.6536407 |
| YDR107C | YDR107C | 0.1686861 | 1 | 0.23396318 | 162.1664864 | 233.8469803 |
| YDR223W | CRF1 | 0.20579017 | 1 | 0.23424122 | 164.1645059 | 237.0678785 |
| YJL146W | IDS2 | 0.19191016 | 1 | 0.23531336 | 168.9818853 | 244.7813072 |
| YJL062W | LAS21 | 0.25242351 | 1 | 0.23582441 | 146.3508148 | 207.8215832 |
| YGL147C | RPL9A | 0.44184395 | 1 | 0.23588393 | 111.9516735 | 151.7364994 |
| YBL037W | APL3 | 0.58395555 | 1 | 0.23631185 | 175.6991571 | 255.6015381 |
| YIL133C | RPL16A | 0.25606894 | 1 | 0.23640245 | 129.9515054 | 181.0121113 |
| YDR149C | YDR149C | 0.24396409 | 1 | 0.23647549 | 147.5443327 | 209.6823301 |
| YHR156C | LIN1 | 0.46204021 | 1 | 0.23657439 | 141.6552306 | 200.0690437 |
| YDL056W | MBP1 | 0.26783829 | 1 | 0.23695753 | 157.9524837 | 226.5867764 |
| YBL024W | NCL1 | 0.49883189 | 1 | 0.23710521 | 154.5898128 | 221.0857038 |
| YBR107C | IML3 | 0.57239181 | 1 | 0.23730442 | 157.1747459 | 225.2736664 |
| YCL029C | BIK1 | 0.07688506 | 0.85916554 | 0.23764959 | 130.1032772 | 181.0968631 |
| YHR113W | YHR113W | 0.37191263 | 1 | 0.23794827 | 151.2364017 | 215.5090275 |
| YDR262W | YDR262W | 0.09173807 | 0.89876254 | 0.23802765 | 139.4164156 | 196.2297873 |
| YAL030W | SNC1 | 0.48522098 | 1 | 0.23814506 | 160.2121841 | 230.1156386 |
| YBR195C | MSI1 | 0.38631473 | 1 | 0.23827335 | 146.3213051 | 207.4540592 |
| YPR008W | HAA1 | 0.74666982 | 1 | 0.23838082 | 188.5515414 | 276.2835781 |
| YGR236C | SPG1 | 0.43975525 | 1 | 0.23854112 | 155.9278418 | 223.0796649 |
| YJR078W | BNA2 | 0.18751399 | 1 | 0.23858053 | 169.4289581 | 245.0839827 |
| YHR076W | PTC7 | 0.36237706 | 1 | 0.23867638 | 146.77602 | 208.1427656 |
| YBL075C | SSA3 | 0.58342679 | 1 | 0.23884195 | 166.3243681 | 239.9887973 |
| YGR192C | TDH3 | 0.26846343 | 1 | 0.23888769 | 144.1036836 | 203.7587734 |
| YAR023C | YAR023C | 0.54860724 | 1 | 0.23909078 | 164.9194055 | 237.6659779 |
| YBR233W | PBP2 | 0.25772043 | 1 | 0.23934539 | 157.5112805 | 225.5560775 |
| YPR028W | YOP1 | 0.7191572 | 1 | 0.23949296 | 139.7544672 | 196.5897554 |
| YDL210W | UGA4 | 0.17961696 | 1 | 0.2394997 | 159.1519969 | 228.2106399 |
| YPL239W | YAR1 | 0.55743529 | 1 | 0.23993235 | 96.2565143 | 125.6222831 |
| YAR018C | KIN3 | 0.44096415 | 1 | 0.23997509 | 158.5833476 | 227.221625 |
| YCR099C | YCR099C | 0.21722772 | 1 | 0.240766 | 163.5879245 | 235.2769028 |
| YBL051C | PIN4 | 0.52470053 | 1 | 0.24077388 | 130.9277691 | 182.0334413 |
| YJL188C | BUD19 | 0.22841164 | 1 | 0.2407801 | 156.6264127 | 223.9264396 |
| YDR128W | MTC5 | 0.22877338 | 1 | 0.24082733 | 158.634595 | 227.1940085 |

| | | | | | | |
|---|---|---|---|---|---|---|
| YBR063C | YBR063C | 0.45993344 | 1 | 0.24111455 | 151.2108635 | 215.0544136 |
| YBR126C | TPS1 | 0.45224147 | 1 | 0.24149068 | 131.4681656 | 182.8208997 |
| YPL273W | SAM4 | 0.67147373 | 1 | 0.24207877 | 134.794147 | 188.1661937 |
| YBL027W | RPL19B | 0.4686427 | 1 | 0.24251341 | 127.1189089 | 175.5973661 |
| YDL080C | THI3 | 0.08814603 | 0.89597255 | 0.24258539 | 134.8545158 | 188.1985282 |
| YCL011C | GBP2 | 0.05920301 | 0.82538218 | 0.24333594 | 166.0888812 | 239.0187518 |
| YJL153C | INO1 | 0.17340067 | 1 | 0.24347324 | 154.0020036 | 219.2968714 |
| YBR215W | HPC2 | 0.52839563 | 1 | 0.24377209 | 164.3819413 | 236.179218 |
| YDR100W | TVP15 | 0.1981338 | 1 | 0.24390831 | 173.8688965 | 251.6270408 |
| YJR021C | REC107 | 0.20298663 | 1 | 0.24405018 | 158.8993661 | 227.2052778 |
| YJL154C | VPS35 | 0.2036075 | 1 | 0.24422362 | 151.2800753 | 214.761723 |
| YDR259C | YAP6 | 0.21808067 | 1 | 0.24452734 | 160.7028652 | 230.0830977 |
| YBL070C | YBL070C | 0.47369028 | 1 | 0.24473598 | 159.7465196 | 228.4968536 |
| YBL067C | UBP13 | 0.44895844 | 1 | 0.2449451 | 152.3904626 | 216.4777682 |
| YJL043W | YJL043W | 0.17302367 | 1 | 0.24521931 | 153.890909 | 218.8880227 |
| YBL052C | SAS3 | 0.44107965 | 1 | 0.24553951 | 150.5870885 | 213.4603859 |
| YBR224W | YBR224W | 0.03834764 | 0.75834714 | 0.24630415 | 153.7622963 | 218.5368622 |
| YDL238C | GUD1 | 0.08826185 | 0.89597255 | 0.24645721 | 171.555458 | 247.5232291 |
| YHR095W | YHR095W | 0.48679858 | 1 | 0.24647393 | 164.4763537 | 235.9807242 |
| YIL084C | SDS3 | 0.22318932 | 1 | 0.24668924 | 149.6878798 | 211.8445403 |
| YDR249C | YDR249C | 0.18185549 | 1 | 0.2467464 | 159.4189556 | 227.7006394 |
| YDR357C | YDR357C | 0.43321804 | 1 | 0.24681131 | 148.2369398 | 209.4633029 |
| YBR094W | PBY1 | 0.46166342 | 1 | 0.24701886 | 147.5974946 | 208.3938109 |
| YDR372C | VPS74 | 0.0878187 | 0.89597255 | 0.24708662 | 158.7666381 | 226.5928593 |
| YBL046W | PSY4 | 0.4588159 | 1 | 0.24733731 | 157.0919462 | 223.8300859 |
| YBL019W | APN2 | 0.44628184 | 1 | 0.24760998 | 149.4046437 | 211.2627169 |
| YHR139C | SPS100 | 0.43507593 | 1 | 0.24766773 | 153.208781 | 217.4566718 |
| YBR194W | SOY1 | 0.19136717 | 1 | 0.24769138 | 101.061626 | 132.4435346 |
| YDR276C | PMP3 | 0.0856606 | 0.89015714 | 0.24820709 | 155.4283225 | 221.0046085 |
| YJR062C | NTA1 | 0.15302335 | 1 | 0.2484256 | 151.603693 | 214.7412147 |
| YHR096C | HXT5 | 0.37370099 | 1 | 0.24902867 | 152.3128225 | 215.818575 |
| YBR205W | KTR3 | 0.43019877 | 1 | 0.24913729 | 148.9125231 | 210.2612547 |
| YAL049C | YAL049C | 0.3903032 | 1 | 0.24923014 | 153.2213086 | 217.2733068 |
| YDL219W | DTD1 | 0.19482181 | 1 | 0.24929061 | 170.4965172 | 245.4273851 |
| YJL013C | MAD3 | 0.16501258 | 1 | 0.25004239 | 156.5740068 | 222.6329171 |
| YDR131C | YDR131C | 0.17964734 | 1 | 0.25052278 | 162.9680067 | 232.993728 |
| YGR281W | YOR1 | 0.36426872 | 1 | 0.25097075 | 147.8198027 | 208.2407673 |
| YCR022C | YCR022C | 0.12310082 | 1 | 0.25106154 | 172.7538435 | 248.8762829 |
| YBL054W | YBL054W | 0.53297316 | 1 | 0.2512137 | 166.723463 | 239.0257389 |
| YBR246W | YBR246W | 0.26215573 | 1 | 0.25140184 | 143.0630279 | 200.430067 |
| YBR065C | ECM2 | 0.50686432 | 1 | 0.25140983 | 153.0770251 | 216.7537971 |
| YBR076W | ECM8 | 0.45283607 | 1 | 0.25143363 | 136.0010468 | 188.9135122 |
| YBR225W | YBR225W | 0.26114082 | 1 | 0.25170182 | 152.0729374 | 215.078853 |
| YCL012W | YCL012W | 0.1291555 | 1 | 0.25196019 | 162.187769 | 231.5343058 |
| YDL042C | SIR2 | 0.1693652 | 1 | 0.25200907 | 152.1219027 | 215.1186009 |
| YDL203C | ACK1 | 0.1575273 | 1 | 0.25210558 | 164.2692989 | 234.9086422 |
| YCR083W | TRX3 | 0.1484009 | 1 | 0.25210988 | 155.2889831 | 220.268412 |
| YBL096C | YBL096C | 0.44214983 | 1 | 0.25222357 | 157.6609545 | 224.1203608 |
| YHR078W | YHR078W | 0.42934796 | 1 | 0.25229894 | 161.8551156 | 230.9478322 |
| YIL024C | YIL024C | 0.4446627 | 1 | 0.25234173 | 159.9442885 | 227.8272291 |
| YIL052C | RPL34B | 0.33810803 | 1 | 0.25244366 | 145.3973449 | 204.0995747 |
| YBL086C | YBL086C | 0.48182404 | 1 | 0.25253087 | 167.6355572 | 240.3408309 |
| YCR024C-A | PMP1 | 0.14725009 | 1 | 0.25264942 | 154.0545094 | 218.1856066 |
| YCL069W | VBA3 | 0.21259572 | 1 | 0.25295012 | 156.6413038 | 222.3633662 |
| YAL043C-A | YAL043C-A | 0.40920333 | 1 | 0.25309378 | 146.107082 | 205.1717884 |
| YCL006C | YCL006C | 0.14348112 | 1 | 0.25312321 | 170.3920053 | 244.7571199 |
| YDR134C | YDR134C | 0.3265633 | 1 | 0.25314033 | 164.6305821 | 235.3626412 |
| YCL049C | YCL049C | 0.0911167 | 0.89876254 | 0.25343275 | 153.0517046 | 216.4486687 |
| YIL014W | MNT3 | 0.49237166 | 1 | 0.25359321 | 167.5147456 | 240.0053219 |
| YDL239C | ADY3 | 0.11960311 | 0.99917458 | 0.25407057 | 165.4179865 | 236.5249319 |
| YAL004W | YAL004W | 0.41892954 | 1 | 0.25421556 | 148.0754807 | 208.2343485 |
| YNR041C | COQ2 | 0.5947023 | 1 | 0.25426433 | 98.47512897 | 127.3697228 |
| YAL022C | FUN26 | 0.43815389 | 1 | 0.25427191 | 152.3009288 | 215.1153039 |
| YDR096W | GIS1 | 0.14610126 | 1 | 0.25447368 | 161.2389379 | 229.6596889 |

| | | | | | | |
|---|---|---|---|---|---|---|
| YDR395W | SXM1 | 0.06675422 | 0.83733449 | 0.25473154 | 164.5301633 | 234.9913962 |
| YDL110C | TMA17 | 0.18495802 | 1 | 0.25484143 | 165.231801 | 236.1208686 |
| YDL200C | MGT1 | 0.13371416 | 1 | 0.25575126 | 172.6978656 | 248.1733436 |
| YBR067C | TIP1 | 0.47509248 | 1 | 0.25593561 | 165.7914714 | 236.890526 |
| YHL005C | YHL005C | 0.33045681 | 1 | 0.25626558 | 140.5078067 | 195.6301749 |
| YPR092W | YPR092W | 0.66386872 | 1 | 0.25637539 | 144.819493 | 202.644743 |
| YJR040W | GEF1 | 0.09626597 | 0.91297781 | 0.25643435 | 156.3911536 | 221.5011208 |
| YCL035C | GRX1 | 0.39206185 | 1 | 0.25663516 | 165.808045 | 236.8263011 |
| YCR010C | ADY2 | 0.12087155 | 1 | 0.25669002 | 159.5221945 | 226.5719811 |
| YDR293C | SSD1 | 0.69712826 | 1 | 0.25675232 | 119.9050141 | 161.9801117 |
| YIL132C | CSM2 | 0.25679154 | 1 | 0.25736579 | 149.884074 | 210.7718209 |
| YDR409W | SIZ1 | 0.19307396 | 1 | 0.25752914 | 161.0451129 | 228.945189 |
| YBL065W | YBL065W | 0.49423814 | 1 | 0.25779306 | 159.984503 | 227.1817635 |
| YCR008W | SAT4 | 0.12245532 | 1 | 0.25803474 | 154.8479762 | 218.7766997 |
| YJL193W | YJL193W | 0.20300945 | 1 | 0.25837715 | 158.0324876 | 223.923414 |
| YJL042W | MHP1 | 0.11916295 | 0.99917458 | 0.25860697 | 148.5276422 | 208.3986826 |
| YBR025C | YBR025C | 0.35372235 | 1 | 0.258677 | 127.9237539 | 174.8011859 |
| YDR006C | SOK1 | 0.22015011 | 1 | 0.25874163 | 175.9336827 | 253.0583198 |
| YDR014W | RAD61 | 0.11809927 | 0.99917458 | 0.25892737 | 147.2311124 | 206.2432968 |
| YDR250C | YDR250C | 0.41675627 | 1 | 0.25909941 | 171.1569656 | 245.2246727 |
| YIL167W | SDL1 | 0.25052297 | 1 | 0.259164 | 151.8101279 | 213.6771222 |
| YBR069C | TAT1 | 0.43997739 | 1 | 0.25929744 | 155.8440081 | 220.2357306 |
| YDL053C | PBP4 | 0.24597111 | 1 | 0.25939639 | 164.5432617 | 234.4043077 |
| YDL135C | RDI1 | 0.08399364 | 0.89015714 | 0.25944774 | 164.1311159 | 233.7257319 |
| YGR250C | YGR250C | 0.45655418 | 1 | 0.25992533 | 159.882554 | 226.737453 |
| YHL035C | VMR1 | 0.37581114 | 1 | 0.26027363 | 149.4258301 | 209.645521 |
| YPR031W | NTO1 | 0.64248217 | 1 | 0.26038749 | 136.4200816 | 188.4287583 |
| YDR384C | ATO3 | 0.13708023 | 1 | 0.2604418 | 164.1623689 | 233.6470238 |
| YDR349C | YPS7 | 0.71399549 | 1 | 0.26051818 | 126.7441359 | 172.6380312 |
| YBR104W | YMC2 | 0.46211688 | 1 | 0.26063471 | 142.4869975 | 198.2867724 |
| YBR034C | HMT1 | 0.33571855 | 1 | 0.26073257 | 138.3822265 | 191.5824289 |
| YJL164C | TPK1 | 0.18042582 | 1 | 0.26096025 | 146.0962134 | 204.1280385 |
| YHR163W | SOL3 | 0.44729221 | 1 | 0.26099631 | 145.7701785 | 203.5918344 |
| YGR282C | BGL2 | 0.54911752 | 1 | 0.26099718 | 174.3632259 | 250.2039743 |
| YPR078C | YPR078C | 0.62750498 | 1 | 0.26150021 | 132.9693754 | 182.6583002 |
| YHL016C | DUR3 | 0.38780119 | 1 | 0.2615825 | 149.4984922 | 209.5932571 |
| YDR256C | CTA1 | 0.23687297 | 1 | 0.26169194 | 157.3605177 | 222.3956194 |
| YBR260C | RGD1 | 0.08913796 | 0.89743999 | 0.26174403 | 161.3792927 | 228.9402139 |
| YDR351W | SBE2 | 0.19280462 | 1 | 0.26218896 | 166.7493016 | 237.6363452 |
| YGL168W | HUR1 | 0.58885823 | 1 | 0.26284193 | 89.93548157 | 112.3296429 |
| YHR185C | PFS1 | 0.3877046 | 1 | 0.26300599 | 155.5610105 | 219.2906792 |
| YJL030W | MAD2 | 0.0788818 | 0.86626076 | 0.26308133 | 157.5723443 | 222.5597188 |
| YDR214W | AHA1 | 0.1426791 | 1 | 0.2632734 | 163.8947728 | 232.8414607 |
| YDL136W | RPL35B | 0.24694779 | 1 | 0.26339029 | 140.5326759 | 194.741433 |
| YCR011C | ADP1 | 0.09129164 | 0.89876254 | 0.26351121 | 169.6610024 | 242.2105242 |
| YDR151C | CTH1 | 0.18811405 | 1 | 0.26356233 | 160.3010617 | 226.9453247 |
| YGR205W | YGR205W | 0.41844181 | 1 | 0.26389645 | 157.898886 | 222.9857282 |
| YIR002C | MPH1 | 0.17054459 | 1 | 0.26420664 | 140.4758405 | 194.5423024 |
| YGR081C | SLX9 | 0.42633057 | 1 | 0.26453814 | 112.3574058 | 148.6605216 |
| YAL054C | ACS1 | 0.41257476 | 1 | 0.264696 | 182.816184 | 263.5015065 |
| YGR202C | PCT1 | 0.57079932 | 1 | 0.26475072 | 163.4676823 | 231.9525311 |
| YAL055W | PEX22 | 0.53094625 | 1 | 0.26475459 | 174.7693903 | 250.3760193 |
| YAR002W | NUP60 | 0.41949306 | 1 | 0.2648288 | 153.9249304 | 216.385797 |
| YIL025C | YIL025C | 0.4240258 | 1 | 0.26488851 | 155.6801336 | 219.2393341 |
| YDR420W | HKR1 | 0.08434741 | 0.89015714 | 0.26491561 | 158.485557 | 223.8091869 |
| YJR080C | FMP26 | 0.31045916 | 1 | 0.265489 | 156.4259485 | 220.3768348 |
| YBR176W | ECM31 | 0.3984932 | 1 | 0.2655788 | 155.4424678 | 218.7618569 |
| YOR054C | VHS3 | 0.67777854 | 1 | 0.26583497 | 129.9610833 | 177.1888085 |
| YDR001C | NTH1 | 0.10485579 | 0.94141418 | 0.26630059 | 172.2340922 | 246.0413412 |
| YHR162W | YHR162W | 0.3210607 | 1 | 0.26637147 | 150.6411766 | 210.8314254 |
| YJR005W | APL1 | 0.2040929 | 1 | 0.26676026 | 168.0014829 | 239.0814056 |
| YJL178C | ATG27 | 0.2839672 | 1 | 0.26701088 | 141.4913657 | 195.8320458 |
| YHR195W | NVJ1 | 0.44676111 | 1 | 0.26758584 | 158.7928794 | 223.9619022 |
| YJL157C | FAR1 | 0.16065998 | 1 | 0.26780114 | 163.4861761 | 231.5848107 |

| | | | | | | |
|---|---|---|---|---|---|---|
| YHR028C | DAP2 | 0.35849444 | 1 | 0.26813065 | 142.0719537 | 196.632465 |
| YHR079C-B | YHR079C-B | 0.36360097 | 1 | 0.26866037 | 154.8781169 | 217.4399219 |
| YBR027C | YBR027C | 0.40424427 | 1 | 0.26886031 | 157.4047105 | 221.532685 |
| YIL037C | PRM2 | 0.35817361 | 1 | 0.26904093 | 143.8793074 | 199.4600761 |
| YDR019C | GCV1 | 0.17213769 | 1 | 0.2691042 | 159.1702413 | 224.3790336 |
| YIL038C | NOT3 | 0.37025294 | 1 | 0.26926408 | 148.883069 | 207.5880801 |
| YDR206W | EBS1 | 0.15360326 | 1 | 0.2692892 | 170.860134 | 243.4117128 |
| YJR010W | MET3 | 0.02678473 | 0.70990463 | 0.26944199 | 178.9914819 | 256.6474697 |
| YBR005W | RCR1 | 0.3998548 | 1 | 0.26948712 | 156.1262379 | 219.3667684 |
| YBL029W | YBL029W | 0.4600283 | 1 | 0.26974636 | 164.2129249 | 232.5158356 |
| YBR009C | HHF1 | 0.48574047 | 1 | 0.26977076 | 141.1612842 | 194.9339753 |
| YIL162W | SUC2 | 0.07805779 | 0.86415424 | 0.26988714 | 159.8131758 | 225.3250223 |
| YAR030C | YAR030C | 0.38731654 | 1 | 0.2699553 | 159.8155252 | 225.3199624 |
| YBR032W | YBR032W | 0.44968844 | 1 | 0.269959 | 150.1953207 | 209.6366667 |
| YBR158W | AMN1 | 0.44689755 | 1 | 0.27045024 | 161.3902731 | 227.8225536 |
| YDR248C | YDR248C | 0.17314854 | 1 | 0.27097305 | 175.7263235 | 251.1249258 |
| YLR239C | LIP2 | 0.64878773 | 1 | 0.2713127 | 63.92718226 | 68.82618228 |
| YBL106C | SRO77 | 0.43999895 | 1 | 0.27135396 | 152.933825 | 213.9190183 |
| YCR023C | YCR023C | 0.063907 | 0.83733449 | 0.27147205 | 161.3337303 | 227.5971024 |
| YDR291W | HRQ1 | 0.18196579 | 1 | 0.27164984 | 174.7828238 | 249.4985625 |
| YGR266W | YGR266W | 0.39387611 | 1 | 0.27176909 | 167.9449074 | 238.3358692 |
| YIL159W | BNR1 | 0.0968912 | 0.91297781 | 0.27179243 | 174.3245232 | 248.7328459 |
| YDR399W | HPT1 | 0.05228604 | 0.80700955 | 0.27181312 | 166.8889345 | 236.6086845 |
| YIL012W | YIL012W | 0.34255094 | 1 | 0.27189716 | 152.2777603 | 212.778654 |
| YDR011W | SNQ2 | 0.13557739 | 1 | 0.2720707 | 163.7452194 | 231.4502176 |
| YDL216C | RRI1 | 0.09783469 | 0.9152552 | 0.27280645 | 168.102196 | 238.4569762 |
| YBR051W | YBR051W | 0.42890099 | 1 | 0.27322828 | 160.0936187 | 225.3464112 |
| YJL089W | SIP4 | 0.15811921 | 1 | 0.27329404 | 175.7408048 | 250.8458045 |
| YIL145C | PAN6 | 0.13182046 | 1 | 0.27341598 | 153.0830123 | 213.8932713 |
| YDL227C | HO | 0.11185343 | 0.96890919 | 0.27357844 | 158.8772639 | 223.3178433 |
| YHR021W-A | ECM12 | 0.31802992 | 1 | 0.27381711 | 144.9689456 | 200.6134373 |
| YJL099W | CHS6 | 0.14483047 | 1 | 0.27395574 | 156.270749 | 219.0195036 |
| YDR055W | PST1 | 0.10123016 | 0.92623613 | 0.27415341 | 170.0316407 | 241.4266635 |
| YBR031W | RPL4A | 0.29311316 | 1 | 0.27416354 | 133.3039247 | 181.5519884 |
| YDL192W | ARF1 | 0.15347975 | 1 | 0.27440269 | 125.6233224 | 168.9999137 |
| YDL071C | YDL071C | 0.20800199 | 1 | 0.27453471 | 160.2004709 | 225.3502017 |
| YDR015C | YDR015C | 0.1606714 | 1 | 0.27468137 | 164.0670074 | 231.6342827 |
| YBL055C | YBL055C | 0.35720059 | 1 | 0.2747247 | 163.9859456 | 231.4964841 |
| YBR033W | EDS1 | 0.41822311 | 1 | 0.27473124 | 153.3480797 | 214.1538323 |
| YJR032W | CPR7 | 0.23436059 | 1 | 0.27474737 | 124.1680056 | 166.5825059 |
| YCL044C | MGR1 | 0.26468266 | 1 | 0.27487403 | 152.5191526 | 212.7838938 |
| YHR140W | YHR140W | 0.39057011 | 1 | 0.27492508 | 160.6133318 | 225.9723299 |
| YBL010C | YBL010C | 0.41841604 | 1 | 0.27503703 | 160.4784472 | 225.7378396 |
| YBR045C | GIP1 | 0.32648302 | 1 | 0.27506665 | 153.8212078 | 214.8813749 |
| YDL234C | GYP7 | 0.21891972 | 1 | 0.27509443 | 168.4244514 | 238.6838925 |
| YIL034C | CAP2 | 0.35826655 | 1 | 0.27520305 | 147.0202066 | 203.7766237 |
| YBR221C | PDB1 | 0.41591729 | 1 | 0.27558341 | 161.1377425 | 226.741355 |
| YDL134C-A | YDL134C-A | 0.21760967 | 1 | 0.27579488 | 177.7122999 | 253.7335397 |
| YDR260C | SWM1 | 0.17126852 | 1 | 0.27579564 | 165.1782084 | 233.3004218 |
| YBL036C | YBL036C | 0.53313725 | 1 | 0.27598934 | 154.5423708 | 215.9366645 |
| YCR032W | BPH1 | 0.11369042 | 0.97713092 | 0.27610332 | 166.4643424 | 235.3569405 |
| YBR138C | YBR138C | 0.39873579 | 1 | 0.27633133 | 165.1325203 | 233.1560704 |
| YIL130W | ASG1 | 0.13058325 | 1 | 0.2764447 | 158.2589034 | 221.935945 |
| YJL147C | YJL147C | 0.1988236 | 1 | 0.27644676 | 153.6826481 | 214.475487 |
| YDR126W | SWF1 | 0.15610259 | 1 | 0.27650596 | 116.8457993 | 154.4165037 |
| YDL011C | YDL011C | 0.09004522 | 0.89876254 | 0.27744713 | 164.9790436 | 232.7603394 |
| YIL101C | XBP1 | 0.09852869 | 0.91974388 | 0.27752336 | 167.2939611 | 236.5241637 |
| YDR003W | RCR2 | 0.08520858 | 0.89015714 | 0.27752987 | 176.6657707 | 251.801196 |
| YIL110W | MNI1 | 0.33019919 | 1 | 0.27760988 | 124.77807 | 167.2036692 |
| YDR156W | RPA14 | 0.10317246 | 0.93121481 | 0.27762506 | 144.4310674 | 199.2399153 |
| YCL013W | YCL013W | 0.25455441 | 1 | 0.27767653 | 165.6991291 | 233.9042984 |
| YCR051W | YCR051W | 0.28022459 | 1 | 0.27768936 | 158.4268235 | 222.0473455 |
| YJR097W | JJJ3 | 0.16835922 | 1 | 0.27801124 | 154.1878904 | 215.0950732 |
| YAL056W | GPB2 | 0.4185938 | 1 | 0.2783485 | 152.6924659 | 212.6132499 |

| | | | | | | |
|---|---|---|---|---|---|---|
| YDR368W | YPR1 | 0.06604286 | 0.83733449 | 0.27835711 | 163.1848799 | 229.7168117 |
| YDR312W | SSF2 | 0.14536184 | 1 | 0.27877288 | 168.7918149 | 238.8029817 |
| YJR010C-A | SPC1 | 0.14419444 | 1 | 0.27885214 | 175.1418424 | 249.1444295 |
| YJR058C | APS2 | 0.03675352 | 0.75834714 | 0.27886475 | 150.2068056 | 208.4938031 |
| YDR419W | RAD30 | 0.43493644 | 1 | 0.27895547 | 157.953516 | 221.1106214 |
| YPR135W | CTF4 | 0.58703405 | 1 | 0.27900368 | 120.8912548 | 160.6856063 |
| YJL045W | YJL045W | 0.1050157 | 0.94141418 | 0.27952553 | 165.8461554 | 233.9028121 |
| YCR001W | YCR001W | 0.09583044 | 0.91297781 | 0.27954889 | 165.6351622 | 233.5558052 |
| YBR184W | YBR184W | 0.41546514 | 1 | 0.27959184 | 165.1524849 | 232.7633456 |
| YJR079W | YJR079W | 0.09157272 | 0.89876254 | 0.27970333 | 164.0038787 | 230.8763507 |
| YAL058C-A | YAL058C-A | 0.37260813 | 1 | 0.27984223 | 153.6811925 | 214.0302383 |
| YDL173W | YDL173W | 0.06228816 | 0.82808418 | 0.27995616 | 154.9053317 | 216.010964 |
| YDL184C | RPL41A | 0.00806469 | 0.56297129 | 0.28009516 | 151.7121049 | 210.7872512 |
| YDL024C | DIA3 | 0.05007499 | 0.80592814 | 0.28023668 | 171.1650205 | 242.480846 |
| YJL162C | JJJ2 | 0.18251624 | 1 | 0.28041941 | 167.4076726 | 236.331801 |
| YJL122W | ALB1 | 0.03395863 | 0.75034314 | 0.28050857 | 168.7389525 | 238.4904175 |
| YJR070C | LIA1 | 0.18313287 | 1 | 0.28053196 | 151.4711913 | 210.3375432 |
| YDR203W | YDR203W | 0.09414547 | 0.9084509 | 0.28057735 | 150.8789062 | 209.3660824 |
| YDR072C | IPT1 | 0.23983469 | 1 | 0.28093248 | 156.3720004 | 218.2745782 |
| YBR058C | UBP14 | 0.33911501 | 1 | 0.28112033 | 157.4871637 | 220.068011 |
| YJL137C | GLG2 | 0.06707679 | 0.83733449 | 0.281245 | 167.3313702 | 236.09973 |
| YAR003W | SWD1 | 0.62163896 | 1 | 0.28128452 | 106.3228229 | 136.6387239 |
| YDL241W | YDL241W | 0.17168074 | 1 | 0.28138803 | 157.4730554 | 220.0100958 |
| YJL198W | PHO90 | 0.12551917 | 1 | 0.28139646 | 170.5604323 | 241.3439772 |
| YBL069W | AST1 | 0.41772656 | 1 | 0.28144728 | 161.6051705 | 226.738522 |
| YDR198C | RKM2 | 0.12545143 | 1 | 0.28158634 | 166.6054906 | 234.8718834 |
| YBR208C | DUR1 | 0.38307038 | 1 | 0.28159296 | 158.6517032 | 221.9047931 |
| YOL049W | GSH2 | 0.67255495 | 1 | 0.28168058 | 110.2396161 | 142.9722038 |
| YAL064C-A | YAL064C-A | 0.35368737 | 1 | 0.28169857 | 156.8943112 | 219.0261257 |
| YDL039C | PRM7 | 0.22414787 | 1 | 0.28186811 | 157.5792065 | 220.1205258 |
| YBR048W | RPS11B | 0.16211158 | 1 | 0.28197851 | 117.6899475 | 155.0788399 |
| YIL149C | MLP2 | 0.14248258 | 1 | 0.28221389 | 163.7629625 | 230.1561547 |
| YGL218W | YGL218W | 0.61441214 | 1 | 0.28222703 | 50.39177459 | 45.33725509 |
| YDR178W | SDH4 | 0.24086633 | 1 | 0.28283845 | 151.2784602 | 209.7225156 |
| YBL042C | FUI1 | 0.4651345 | 1 | 0.28301606 | 165.4909999 | 232.8685662 |
| YDL095W | PMT1 | 0.05676575 | 0.81796023 | 0.28309741 | 161.2971044 | 226.0210862 |
| YIL010W | DOT5 | 0.5358173 | 1 | 0.28325891 | 165.6292825 | 233.0623188 |
| YGR206W | MVB12 | 0.29920026 | 1 | 0.28326586 | 143.4004191 | 196.8240218 |
| YDR378C | LSM6 | 0.6306 | 1 | 0.28342068 | 58.78747261 | 58.86819414 |
| YDL046W | NPC2 | 0.35687413 | 1 | 0.28350392 | 174.6302802 | 247.7037466 |
| YDR063W | YDR063W | 0.11551679 | 0.98810576 | 0.28367257 | 166.8275551 | 234.9617828 |
| YDL036C | PUS9 | 0.18286827 | 1 | 0.28384666 | 158.3797166 | 221.1674483 |
| YJL124C | LSM1 | 0.16532411 | 1 | 0.28439796 | 120.3488147 | 159.0977413 |
| YIR007W | YIR007W | 0.09775105 | 0.9152552 | 0.28445253 | 157.9758697 | 220.4300752 |
| YJL148W | RPA34 | 0.0866165 | 0.89192147 | 0.28456445 | 131.7067971 | 177.5917569 |
| YBR226C | YBR226C | 0.12076956 | 1 | 0.28478829 | 163.6072969 | 229.5666085 |
| YGR225W | AMA1 | 0.32690254 | 1 | 0.28479334 | 142.752151 | 195.5679875 |
| YBL083C | YBL083C | 0.43023895 | 1 | 0.28496234 | 160.4608938 | 224.4146548 |
| YBL082C | ALG3 | 0.37220524 | 1 | 0.28498336 | 153.8989291 | 213.7146285 |
| YDR057W | YOS9 | 0.15385899 | 1 | 0.2851194 | 179.513657 | 255.4538961 |
| YDR210W | YDR210W | 0.0959224 | 0.91297781 | 0.28515927 | 174.6112444 | 247.4568053 |
| YHL034C | SBP1 | 0.41148002 | 1 | 0.28561436 | 151.5754353 | 209.8445777 |
| YDR103W | STE5 | 0.1338952 | 1 | 0.28582027 | 170.4840795 | 240.6425051 |
| YPR057W | BRR1 | 0.46536445 | 1 | 0.28600148 | 105.0903415 | 134.0142998 |
| YCL048W | SPS22 | 0.1306981 | 1 | 0.286048 | 174.3081098 | 246.8467183 |
| YDR275W | BSC2 | 0.12565694 | 1 | 0.28626607 | 158.4783809 | 221.0127246 |
| YHR035W | YHR035W | 0.33584134 | 1 | 0.28634158 | 149.3856077 | 206.1798784 |
| YHR017W | YSC83 | 0.46304622 | 1 | 0.28637746 | 168.7202327 | 237.6944147 |
| YDL023C | SRF4 | 0.02887319 | 0.72930271 | 0.28642414 | 164.0255875 | 230.0351383 |
| YHR048W | YHR048W | 0.32904744 | 1 | 0.28664239 | 154.1898374 | 213.9724767 |
| YDR402C | DIT2 | 0.14922103 | 1 | 0.28672183 | 160.4801618 | 224.2165736 |
| YCR075C | ERS1 | 0.13258367 | 1 | 0.28682113 | 158.4606124 | 220.9113618 |
| YJL135W | YJL135W | 0.23207176 | 1 | 0.28706485 | 153.9416687 | 213.5128117 |
| YDR171W | HSP42 | 0.03016473 | 0.74243573 | 0.28708304 | 165.598476 | 232.5133121 |

| | | | | | | |
|---|---|---|---|---|---|---|
| YGR210C | YGR210C | 0.30259196 | 1 | 0.28723517 | 150.0903747 | 207.2122348 |
| YHR018C | ARG4 | 0.33939545 | 1 | 0.28755514 | 153.955654 | 213.4716607 |
| YCR062W | YCR062W | 0.07078967 | 0.83733449 | 0.28761107 | 159.1084206 | 221.8643821 |
| YHR146W | CRP1 | 0.38366963 | 1 | 0.287986 | 163.1307117 | 228.3726005 |
| YBR026C | ETR1 | 0.37197085 | 1 | 0.28811882 | 142.1427497 | 194.1407974 |
| YIR014W | YIR014W | 0.12941852 | 1 | 0.28834267 | 162.5236126 | 227.3363895 |
| YIL058W | YIL058W | 0.30809978 | 1 | 0.28857595 | 145.037991 | 198.8009826 |
| YHR049C-A | YHR049C-A | 0.4155985 | 1 | 0.2887083 | 161.8143327 | 226.1324353 |
| YDR010C | YDR010C | 0.1363506 | 1 | 0.2895663 | 165.2484432 | 231.6187966 |
| YDR255C | RMD5 | 0.11212909 | 0.96890919 | 0.2899915 | 166.7489772 | 234.0095009 |
| YDL130W | RPP1B | 0.05993548 | 0.82753359 | 0.29035821 | 121.0030853 | 159.3869284 |
| YPR044C | YPR044C | 0.45914479 | 1 | 0.29049973 | 101.0598477 | 126.8570965 |
| YDR084C | TVP23 | 0.14396265 | 1 | 0.29050278 | 167.6481896 | 235.4087054 |
| YDL112W | TRM3 | 0.03685812 | 0.75834714 | 0.29053905 | 171.2750673 | 241.3164947 |
| YCL001W | RER1 | 0.11854037 | 0.99917458 | 0.2906731 | 164.4881055 | 230.2349357 |
| YDL188C | PPH22 | 0.15291399 | 1 | 0.29069103 | 163.3873817 | 228.4382019 |
| YDR287W | YDR287W | 0.11333383 | 0.97713092 | 0.29070079 | 165.5266039 | 231.9242802 |
| YDR315C | IPK1 | 0.12182909 | 1 | 0.29106101 | 133.8069286 | 180.1680274 |
| YAL059W | ECM1 | 0.34640391 | 1 | 0.29147225 | 143.7684057 | 196.353543 |
| YJL215C | YJL215C | 0.09920709 | 0.92031276 | 0.291698 | 150.0737311 | 206.6030111 |
| YIL103W | DPH1 | 0.13872645 | 1 | 0.29199761 | 156.0659287 | 216.332385 |
| YJR026W | YJR026W | 0.07947037 | 0.86830979 | 0.29227736 | 161.546198 | 225.2298064 |
| YAR047C | YAR047C | 0.31684381 | 1 | 0.29251786 | 146.2072203 | 200.1929068 |
| YPL060W | LPE10 | 0.59419588 | 1 | 0.29264243 | 136.1527176 | 183.7858552 |
| YIR003W | YIR003W | 0.13774156 | 1 | 0.29289063 | 156.4772957 | 216.8865157 |
| YCR102W-A | YCR102W-A | 0.11171631 | 0.96890919 | 0.29315459 | 165.508026 | 231.5739417 |
| YJR038C | YJR038C | 0.06593869 | 0.83733449 | 0.29363752 | 156.3103721 | 216.5169797 |
| YDR099W | BMH2 | 0.03238929 | 0.75034314 | 0.29370849 | 160.2061562 | 222.8586124 |
| YIR001C | SGN1 | 0.17251317 | 1 | 0.29463369 | 155.3470921 | 214.8167143 |
| YIR020W-B | YIR020W-B | 0.12112817 | 1 | 0.29499563 | 163.9039002 | 228.7187751 |
| YJR059W | PTK2 | 0.01623272 | 0.64284628 | 0.29528784 | 154.497724 | 213.3467565 |
| YIL155C | GUT2 | 0.15337435 | 1 | 0.29563885 | 156.15372 | 216.0005713 |
| YCL033C | YCL033C | 0.04702548 | 0.79308159 | 0.29598447 | 166.7451704 | 233.2216243 |
| YDR348C | YDR348C | 0.0835091 | 0.89015714 | 0.29622593 | 156.2622928 | 216.1009923 |
| YBR220C | YBR220C | 0.37916008 | 1 | 0.29648614 | 156.1111549 | 215.8206682 |
| YBR261C | YBR261C | 0.06406057 | 0.83733449 | 0.29682852 | 173.4996549 | 244.1226635 |
| YAL036C | RBG1 | 0.42540073 | 1 | 0.29690924 | 160.1001463 | 222.2683188 |
| YCR089W | FIG2 | 0.06202112 | 0.82808418 | 0.2972042 | 177.4324823 | 250.4849407 |
| YIL076W | SEC28 | 0.16167596 | 1 | 0.29767615 | 139.6428567 | 188.81891 |
| YJL037W | IRC18 | 0.12647786 | 1 | 0.2976932 | 165.8031213 | 231.463029 |
| YBR168W | PEX32 | 0.2039801 | 1 | 0.2978059 | 139.4992934 | 188.5679511 |
| YDR116C | MRPL1 | 0.06736051 | 0.83733449 | 0.29796416 | 153.7272622 | 211.7416781 |
| YDL144C | YDL144C | 0.0789393 | 0.86626076 | 0.29836314 | 163.8959099 | 228.2665205 |
| YCR068W | ATG15 | 0.19385631 | 1 | 0.2985071 | 156.635481 | 216.4118262 |
| YJL168C | SET2 | 0.18917477 | 1 | 0.29900655 | 149.5286963 | 204.7612355 |
| YCL030C | HIS4 | 0.21821748 | 1 | 0.2990373 | 158.0391501 | 218.6309272 |
| YDL061C | RPS29B | 0.00900966 | 0.56297129 | 0.29931843 | 104.7138264 | 131.6635627 |
| YBR137W | YBR137W | 0.30999105 | 1 | 0.29946027 | 157.2993299 | 217.3697071 |
| YHR037W | PUT2 | 0.43716647 | 1 | 0.30022538 | 152.34305 | 209.1902084 |
| YDR085C | AFR1 | 0.13138152 | 1 | 0.30030814 | 176.1250313 | 247.94869 |
| YHL020C | OPI1 | 0.57613963 | 1 | 0.30067233 | 59.52841075 | 57.82591536 |
| YGL080W | FMP37 | 0.13938913 | 1 | 0.30068613 | 120.5951195 | 157.3747828 |
| YDR338C | YDR338C | 0.1069065 | 0.95239943 | 0.30078604 | 166.4254443 | 232.0741353 |
| YBR047W | FMP23 | 0.41178349 | 1 | 0.30079116 | 164.1931395 | 228.4343746 |
| YDR272W | GLO2 | 0.17049559 | 1 | 0.30093537 | 171.8830788 | 240.9516684 |
| YCR048W | ARE1 | 0.09066887 | 0.89876254 | 0.30099696 | 168.4828506 | 235.4005986 |
| YBR294W | SUL1 | 0.21059369 | 1 | 0.30116609 | 179.4559651 | 253.2668588 |
| YJR001W | AVT1 | 0.1478411 | 1 | 0.3011734 | 176.7586343 | 248.8687298 |
| YBR258C | SHG1 | 0.01631818 | 0.64284628 | 0.30156123 | 181.0502557 | 255.8143256 |
| YBR116C | YBR116C | 0.400198 | 1 | 0.30196036 | 159.576061 | 220.7551343 |
| YDR215C | YDR215C | 0.13744377 | 1 | 0.30202957 | 165.9168952 | 231.0829052 |
| YBR159W | IFA38 | 0.31459177 | 1 | 0.30205186 | 154.4307703 | 212.3553702 |
| YJR084W | CSN12 | 0.06968863 | 0.83733449 | 0.30219455 | 156.1096156 | 215.0736048 |
| YBR249C | ARO4 | 0.14825235 | 1 | 0.302313 | 161.7912593 | 224.3203443 |

| | | | | | | |
|---|---|---|---|---|---|---|
| YAR050W | FLO1 | 0.37863527 | 1 | 0.30242219 | 174.7257411 | 245.3918349 |
| YBL032W | HEK2 | 0.3395304 | 1 | 0.30289914 | 165.4309928 | 230.17737 |
| YDR374C | YDR374C | 0.23774521 | 1 | 0.30312093 | 158.4279557 | 218.7321233 |
| YBR125C | PTC4 | 0.39211957 | 1 | 0.30314439 | 161.9263548 | 224.432137 |
| YJR094W-A | RPL43B | 0.05848468 | 0.82030594 | 0.30338409 | 136.4009149 | 182.789419 |
| YDR093W | DNF2 | 0.13876936 | 1 | 0.30377977 | 174.1227259 | 244.231732 |
| YIL015W | BAR1 | 0.40115678 | 1 | 0.30382162 | 176.0071614 | 247.2982723 |
| YBR230C | OM14 | 0.09329364 | 0.90839656 | 0.30420188 | 159.4109203 | 220.1935589 |
| YGR217W | CCH1 | 0.38091581 | 1 | 0.30473121 | 163.3159516 | 226.4904814 |
| YCR087C-A | LUG1 | 0.17285555 | 1 | 0.30489624 | 147.1941652 | 200.1872949 |
| YBL087C | RPL23A | 0.16370351 | 1 | 0.30519365 | 121.507365 | 158.2740004 |
| YDR199W | YDR199W | 0.20189884 | 1 | 0.30522268 | 170.9142581 | 238.8131027 |
| YJL207C | LAA1 | 0.02139896 | 0.70561357 | 0.30550959 | 162.8544513 | 225.63662 |
| YDR120C | TRM1 | 0.09106525 | 0.89876254 | 0.30551932 | 162.1549823 | 224.4950811 |
| YDR020C | YDR020C | 0.12302086 | 1 | 0.30563394 | 162.6237032 | 225.2442369 |
| YBR300C | YBR300C | 0.07179271 | 0.8399943 | 0.30620816 | 165.6138238 | 230.0438223 |
| YCL063W | VAC17 | 0.21433103 | 1 | 0.306322 | 146.9073111 | 199.5337022 |
| YDR332W | IRC3 | 0.13899726 | 1 | 0.30683669 | 164.4194846 | 228.0148358 |
| YJR096W | YJR096W | 0.10102884 | 0.92623613 | 0.30728437 | 164.1870773 | 227.5775743 |
| YIR030C | DCG1 | 0.06903847 | 0.83733449 | 0.3077091 | 177.1066573 | 248.583616 |
| YBR144C | YBR144C | 0.44456014 | 1 | 0.30784926 | 160.8755581 | 222.1054729 |
| YDR135C | YCF1 | 0.02553244 | 0.70990463 | 0.30788518 | 156.608309 | 215.1443374 |
| YBL007C | SLA1 | 0.25733407 | 1 | 0.30841664 | 135.3345608 | 180.3946531 |
| YJL163C | YJL163C | 0.14377897 | 1 | 0.30843273 | 156.4894628 | 214.8791775 |
| YIL120W | QDR1 | 0.12180184 | 1 | 0.30845813 | 162.3973999 | 224.5069568 |
| YAL037W | YAL037W | 0.45327669 | 1 | 0.30850888 | 169.963943 | 236.8352809 |
| YDL018C | ERP3 | 0.30607403 | 1 | 0.30871159 | 155.3949603 | 213.0585523 |
| YIL009C-A | EST3 | 0.35881195 | 1 | 0.30880345 | 159.4391769 | 219.6394348 |
| YDL156W | YDL156W | 0.07101907 | 0.83733449 | 0.30883455 | 160.9712149 | 222.1328989 |
| YDR392W | SPT3 | 0.0780838 | 0.86415424 | 0.30916596 | 129.1683127 | 170.2447284 |
| YCR007C | YCR007C | 0.07562665 | 0.85233814 | 0.30935593 | 167.9332735 | 233.4144108 |
| YDR266C | YDR266C | 0.16444164 | 1 | 0.30977446 | 158.5233892 | 218.0198717 |
| YDR056C | YDR056C | 0.15244671 | 1 | 0.30981681 | 174.9227911 | 244.7485772 |
| YJL110C | GZF3 | 0.06156872 | 0.82808418 | 0.30983085 | 165.5933597 | 229.5379499 |
| YGR207C | YGR207C | 0.30768939 | 1 | 0.31009667 | 161.6109081 | 223.0111038 |
| YAR043C | YAR043C | 0.36095625 | 1 | 0.31046574 | 169.4303915 | 235.7102519 |
| YDR143C | SAN1 | 0.30119478 | 1 | 0.31063727 | 182.1006333 | 256.3428493 |
| YIL087C | YIL087C | 0.08804537 | 0.89597255 | 0.31068935 | 163.9282589 | 226.7115344 |
| YDL129W | YDL129W | 0.0488952 | 0.80391719 | 0.31078582 | 170.5145421 | 237.4358808 |
| YPR060C | ARO7 | 0.43527922 | 1 | 0.31121986 | 104.7156975 | 130.1142964 |
| YCL009C | ILV6 | 0.00622089 | 0.5161257 | 0.31159568 | 159.6600825 | 219.6353597 |
| YIR044C | YIR044C | 0.08344171 | 0.89015714 | 0.31202471 | 160.5329046 | 221.0022716 |
| YIL088C | AVT7 | 0.19172419 | 1 | 0.31235755 | 153.5120613 | 209.5135132 |
| YBR281C | DUG2 | 0.19406393 | 1 | 0.31280491 | 151.2730085 | 205.8050699 |
| YDL154W | MSH5 | 0.0744086 | 0.85139088 | 0.31289732 | 186.7537491 | 263.6335562 |
| YDR061W | YDR061W | 0.20486228 | 1 | 0.31290853 | 171.2788402 | 238.404968 |
| YIL157C | FMP35 | 0.09267375 | 0.90441152 | 0.31340813 | 166.0156364 | 229.7597541 |
| YJL138C | TIF2 | 0.04427702 | 0.77602255 | 0.31387015 | 144.8485214 | 195.1929604 |
| YBR290W | BSD2 | 0.14210962 | 1 | 0.31404703 | 153.671372 | 209.5528589 |
| YDL201W | TRM8 | 0.02152664 | 0.70561357 | 0.31416294 | 173.678621 | 242.1534659 |
| YDL076C | RXT3 | 0.04918073 | 0.80391719 | 0.31417311 | 182.03314 | 255.7716373 |
| YDL204W | RTN2 | 0.05384424 | 0.80700955 | 0.314328 | 170.1775047 | 236.4244334 |
| YCR027C | RHB1 | 0.13869044 | 1 | 0.31477413 | 153.4227851 | 209.0527776 |
| YJR015W | YJR015W | 0.06983538 | 0.83733449 | 0.3152444 | 161.8029423 | 222.6527332 |
| YPR155C | NCA2 | 0.46029758 | 1 | 0.31526013 | 151.960377 | 206.6053771 |
| YNL236W | SIN4 | 0.50593771 | 1 | 0.31527428 | 33.31711368 | 13.19183114 |
| YIL161W | YIL161W | 0.23293089 | 1 | 0.31544642 | 176.0775932 | 245.8968531 |
| YCL050C | APA1 | 0.02351165 | 0.70990463 | 0.31568946 | 170.3718546 | 236.5636852 |
| YJL158C | CIS3 | 0.08533858 | 0.89015714 | 0.31571167 | 169.5728592 | 235.2582691 |
| YDR181C | SAS4 | 0.09958833 | 0.92031276 | 0.31579688 | 184.1922559 | 259.0796282 |
| YBL057C | PTH2 | 0.34897249 | 1 | 0.31583082 | 159.3275943 | 218.5409452 |
| YBR231C | SWC5 | 0.08978671 | 0.89876254 | 0.31604535 | 158.7240779 | 217.5291136 |
| YDL038C | YDL038C | 0.03378672 | 0.75034314 | 0.3162513 | 179.2721413 | 250.9996105 |
| YDR009W | GAL3 | 0.14616989 | 1 | 0.31638033 | 166.6124232 | 230.3449663 |

| | | | | | | |
|---|---|---|---|---|---|---|
| YDL157C | YDL157C | 0.25872851 | 1 | 0.31639285 | 169.4475853 | 234.9652021 |
| YDL090C | RAM1 | 0.18088893 | 1 | 0.31651863 | 146.260267 | 197.148937 |
| YJL218W | YJL218W | 0.06889744 | 0.83733449 | 0.3168976 | 176.2364437 | 245.966532 |
| YJL064W | YJL064W | 0.19615497 | 1 | 0.31719838 | 149.1314265 | 201.7408276 |
| YGR197C | SNG1 | 0.35126233 | 1 | 0.31738377 | 156.3939795 | 213.5560266 |
| YIL055C | YIL055C | 0.30309963 | 1 | 0.31751716 | 162.7792618 | 223.9478863 |
| YDL223C | HBT1 | 0.10290424 | 0.93121481 | 0.31753332 | 169.0818829 | 234.2202818 |
| YIR018W | YAP5 | 0.05675231 | 0.81796023 | 0.31779459 | 162.9404235 | 224.1744264 |
| YCL056C | YCL056C | 0.04338247 | 0.77602255 | 0.3179928 | 172.2920576 | 239.3935638 |
| YDL162C | YDL162C | 0.05571323 | 0.81725117 | 0.31819253 | 168.5649618 | 233.2916179 |
| YDR163W | CWC15 | 0.09449425 | 0.90866306 | 0.31834693 | 165.0843878 | 227.5974645 |
| YIL140W | AXL2 | 0.24008813 | 1 | 0.31853222 | 178.938453 | 250.1581294 |
| YML050W | YML050W | 0.60966796 | 1 | 0.31854431 | 150.4723346 | 203.7512186 |
| YBR044C | TCM62 | 0.30672836 | 1 | 0.31898037 | 134.3910983 | 177.4787858 |
| YDR344C | YDR344C | 0.04145484 | 0.77009646 | 0.31906355 | 171.2030792 | 237.4786568 |
| YBR071W | YBR071W | 0.29202137 | 1 | 0.31923388 | 155.1336031 | 211.2600549 |
| YDL026W | YDL026W | 0.12422855 | 1 | 0.31935895 | 162.1446058 | 222.6730463 |
| YBL013W | FMT1 | 0.33024474 | 1 | 0.31978871 | 155.6609126 | 212.0473055 |
| YDR251W | PAM1 | 0.11899792 | 0.99917458 | 0.32000957 | 176.865759 | 246.5865414 |
| YHR153C | SPO16 | 0.31137183 | 1 | 0.32038196 | 153.898851 | 209.0974221 |
| YBR115C | LYS2 | 0.2078621 | 1 | 0.32040636 | 130.850245 | 171.5205092 |
| YJL169W | YJL169W | 0.11651475 | 0.99259248 | 0.32051157 | 158.5364311 | 216.6406794 |
| YBR293W | VBA2 | 0.02502102 | 0.70990463 | 0.32074079 | 171.2658041 | 237.3621471 |
| YDR049W | YDR049W | 0.08642986 | 0.89192147 | 0.32091418 | 136.974132 | 181.4374054 |
| YIL098C | FMC1 | 0.1213382 | 1 | 0.32096303 | 149.6764332 | 202.1382678 |
| YDR263C | DIN7 | 0.16280412 | 1 | 0.32145329 | 171.6897671 | 237.9603577 |
| YJR036C | HUL4 | 0.10544528 | 0.94226883 | 0.32186797 | 159.7271136 | 218.404807 |
| YBL079W | NUP170 | 0.29458445 | 1 | 0.3218774 | 129.9095446 | 169.7951144 |
| YBR181C | RPS6B | 0.212679 | 1 | 0.32224058 | 120.8959629 | 155.0538448 |
| YHR179W | OYE2 | 0.48824985 | 1 | 0.32274696 | 164.1830073 | 225.5541375 |
| YBR139W | YBR139W | 0.31380881 | 1 | 0.32277181 | 168.1246032 | 231.9764678 |
| YDR005C | MAF1 | 0.10322735 | 0.93121481 | 0.32284996 | 159.2091189 | 217.4322924 |
| YCL026C | YCL026C | 0.15443891 | 1 | 0.32285441 | 159.4132405 | 217.76447 |
| YDR279W | RNH202 | 0.14085003 | 1 | 0.32325422 | 163.8144145 | 224.8870964 |
| YBR210W | ERV15 | 0.49959263 | 1 | 0.32329437 | 176.1496407 | 244.9906884 |
| YCL038C | ATG22 | 0.04556499 | 0.78262426 | 0.32339895 | 172.8018964 | 239.5195704 |
| YJL151C | SNA3 | 0.0502648 | 0.80592814 | 0.32369656 | 163.7229027 | 224.6802192 |
| YDL229W | SSB1 | 0.14514471 | 1 | 0.32409601 | 160.0408943 | 218.6257256 |
| YCR087W | YCR087W | 0.09035809 | 0.89876254 | 0.32503573 | 152.4092714 | 206.0621203 |
| YBL068W | PRS4 | 0.39978741 | 1 | 0.32524596 | 166.7663828 | 229.4395967 |
| YBL009W | ALK2 | 0.40605348 | 1 | 0.32534262 | 170.2633273 | 235.1276919 |
| YDL027C | YDL027C | 0.07117538 | 0.83733449 | 0.32537536 | 172.1208864 | 238.1516065 |
| YDL035C | GPR1 | 0.08583409 | 0.89015714 | 0.32657664 | 162.967962 | 223.0738672 |
| YAL058W | CNE1 | 0.24061733 | 1 | 0.32681339 | 143.3873602 | 191.1227803 |
| YDR024W | FYV1 | 0.10897355 | 0.96084682 | 0.32729668 | 141.6585814 | 188.2414975 |
| YPL120W | VPS30 | 0.59170115 | 1 | 0.32740415 | 142.797364 | 190.0839177 |
| YHR067W | HTD2 | 0.65178263 | 1 | 0.32751457 | 127.4775859 | 165.0952836 |
| YIL151C | YIL151C | 0.45876677 | 1 | 0.32761419 | 153.6463975 | 207.7425667 |
| YDR225W | HTA1 | 0.06507235 | 0.83733449 | 0.32836516 | 172.3145345 | 238.0773272 |
| YJL214W | HXT8 | 0.05662535 | 0.81796023 | 0.32839211 | 173.8643667 | 240.6003419 |
| YBR156C | SLI15 | 0.29038609 | 1 | 0.32851833 | 158.8163978 | 216.0527489 |
| YGR177C | ATF2 | 0.31386428 | 1 | 0.3285431 | 163.8614487 | 224.2739369 |
| YDR411C | DFM1 | 0.13437299 | 1 | 0.32872209 | 174.3116993 | 241.2865409 |
| YCR079W | PTC6 | 0.02730844 | 0.70990463 | 0.32873641 | 178.0341556 | 247.3530049 |
| YCL026C-A | FRM2 | 0.29333398 | 1 | 0.32898018 | 151.2014572 | 203.5786682 |
| YDR400W | URH1 | 0.12492054 | 1 | 0.32912673 | 180.1953828 | 250.8253168 |
| YJL208C | NUC1 | 0.06992509 | 0.83733449 | 0.32935143 | 165.0043666 | 226.0316854 |
| YDL091C | UBX3 | 0.0581687 | 0.82030594 | 0.32947448 | 175.0098337 | 242.3265017 |
| YBR209W | YBR209W | 0.34507652 | 1 | 0.32974977 | 149.5868233 | 200.8461217 |
| YBR187W | GDT1 | 0.40421601 | 1 | 0.33009331 | 158.8880503 | 215.9641303 |
| YIL165C | YIL165C | 0.06146529 | 0.82808418 | 0.33055077 | 163.6047267 | 223.5935676 |
| YIL173W | VTH1 | 0.11053225 | 0.96702536 | 0.3311759 | 161.5188221 | 220.1115988 |
| YAL027W | YAL027W | 0.48374023 | 1 | 0.33131336 | 175.7426049 | 243.281215 |
| YIL137C | TMA108 | 0.1789997 | 1 | 0.3315494 | 157.3741127 | 213.3061963 |

| | | | | | | |
|---|---|---|---|---|---|---|
| YBL072C | RPS8A | 0.32283309 | 1 | 0.33239248 | 148.9261311 | 199.424372 |
| YDL180W | YDL180W | 0.09459068 | 0.90866306 | 0.33240894 | 171.2727753 | 235.8516211 |
| YDL088C | ASM4 | 0.11057509 | 0.96702536 | 0.33265453 | 167.9165859 | 230.3483443 |
| YBR244W | GPX2 | 0.07047556 | 0.83733449 | 0.33271261 | 166.6264306 | 228.2375635 |
| YCL016C | DCC1 | 0.02543288 | 0.70990463 | 0.33318596 | 133.7630939 | 174.6021648 |
| YDL185W | TFP1 | 0.09413889 | 0.9084509 | 0.33326138 | 172.9916461 | 238.5425327 |
| YDR051C | YDR051C | 0.04925245 | 0.80391719 | 0.33328712 | 159.6553003 | 216.7983258 |
| YCR017C | CWH43 | 0.04202805 | 0.77009646 | 0.33364118 | 172.0263666 | 236.9194 |
| YDL048C | STP4 | 0.11804626 | 0.99917458 | 0.3340117 | 165.9864094 | 227.0247622 |
| YDR379W | RGA2 | 0.08159497 | 0.88254103 | 0.33420656 | 164.839205 | 225.1291789 |
| YDL197C | ASF2 | 0.03427484 | 0.75034314 | 0.33463748 | 164.4462711 | 224.432415 |
| YDL099W | BUG1 | 0.03119029 | 0.75034314 | 0.33482101 | 164.5743933 | 224.6173408 |
| YJL161W | FMP33 | 0.13827287 | 1 | 0.33492869 | 166.1078137 | 227.1030712 |
| YGR275W | RTT102 | 0.44449881 | 1 | 0.33508316 | 171.8180154 | 236.3916686 |
| YHR134W | WSS1 | 0.35023795 | 1 | 0.33547275 | 147.3736282 | 196.4917268 |
| YAL005C | SSA1 | 0.28889031 | 1 | 0.33555913 | 148.1721314 | 197.7821762 |
| YDR184C | ATC1 | 0.05221355 | 0.80700955 | 0.33579135 | 154.1191945 | 207.4467618 |
| YBR103W | SIF2 | 0.25087307 | 1 | 0.33580184 | 136.9089491 | 179.389331 |
| YCR044C | PER1 | 0.07428031 | 0.85139088 | 0.33599733 | 148.9585037 | 199.0069616 |
| YDR152W | GIR2 | 0.14904031 | 1 | 0.3365457 | 163.3058104 | 222.3243503 |
| YJL139C | YUR1 | 0.0898764 | 0.89876254 | 0.337231 | 164.074209 | 223.4876052 |
| YCR015C | YCR015C | 0.00804489 | 0.56297129 | 0.33783362 | 172.4318709 | 237.0336268 |
| YDL124W | YDL124W | 0.06221167 | 0.82808418 | 0.3378419 | 166.8821231 | 227.985373 |
| YIL071C | PCI8 | 0.05826029 | 0.82030594 | 0.33785717 | 168.6571797 | 230.8770708 |
| YDR186C | YDR186C | 0.05295023 | 0.80700955 | 0.33815713 | 167.5257273 | 228.9934579 |
| YDR346C | SVF1 | 0.07396869 | 0.85139088 | 0.33821962 | 162.4996545 | 220.7918266 |
| YDR254W | CHL4 | 0.11377864 | 0.97713092 | 0.33852252 | 168.279529 | 230.1746444 |
| YDL174C | DLD1 | 0.048504 | 0.80391719 | 0.33865124 | 153.574822 | 206.1863091 |
| YJR048W | CYC1 | 0.06096894 | 0.82808418 | 0.33883607 | 167.2117429 | 228.3930474 |
| YPR043W | RPL43A | 0.53689217 | 1 | 0.33920552 | 108.1906705 | 132.1289805 |
| YBR171W | SEC66 | 0.30149113 | 1 | 0.33938684 | 165.1920239 | 225.0286735 |
| YAL031C | GIP4 | 0.36977316 | 1 | 0.33971265 | 163.5528222 | 222.3139591 |
| YDR261C | EXG2 | 0.01757361 | 0.66607965 | 0.34022958 | 163.8638109 | 222.7535076 |
| YPL152W | RRD2 | 0.55949619 | 1 | 0.34042669 | 109.0032284 | 133.2943295 |
| YJR082C | EAF6 | 0.0392206 | 0.75861823 | 0.34047186 | 144.144783 | 190.5760388 |
| YDL096C | YDL096C | 0.00722189 | 0.56297129 | 0.34055848 | 165.5959617 | 225.5343523 |
| YIL164C | NIT1 | 0.21171039 | 1 | 0.34075323 | 172.5402 | 236.8294168 |
| YBR275C | RIF1 | 0.0961083 | 0.91297781 | 0.34095884 | 153.328207 | 205.4832966 |
| YJL071W | ARG2 | 0.00943967 | 0.56297129 | 0.34117886 | 165.2539343 | 224.8958641 |
| YCR031C | RPS14A | 0.14766666 | 1 | 0.34168936 | 141.9963396 | 186.9148555 |
| YDL094C | YDL094C | 0.02216558 | 0.70990463 | 0.34169214 | 173.6186028 | 238.4649606 |
| YBR285W | YBR285W | 0.07447278 | 0.85139088 | 0.34192538 | 174.8878806 | 240.5037094 |
| YDL199C | YDL199C | 0.17437238 | 1 | 0.34212817 | 180.2043365 | 249.1441216 |
| YBL031W | SHE1 | 0.34377857 | 1 | 0.34229608 | 157.450885 | 212.0296497 |
| YDR018C | YDR018C | 0.11673479 | 0.99259248 | 0.34248311 | 173.7051741 | 238.5029211 |
| YBL059W | YBL059W | 0.31786167 | 1 | 0.342541 | 158.9441249 | 214.4319762 |
| YDL175C | AIR2 | 0.0541264 | 0.80700955 | 0.34309949 | 172.7299653 | 236.8327454 |
| YJR031C | GEA1 | 0.07690885 | 0.85916554 | 0.34340721 | 165.6818599 | 225.3028205 |
| YHL013C | OTU2 | 0.22877785 | 1 | 0.34353288 | 145.3085826 | 192.0740068 |
| YCR061W | YCR061W | 0.03996171 | 0.75927246 | 0.34359446 | 146.3379508 | 193.7440455 |
| YBR066C | NRG2 | 0.3374059 | 1 | 0.34389001 | 159.731141 | 215.539014 |
| YBR284W | YBR284W | 0.13274862 | 1 | 0.34401473 | 157.5392973 | 211.9496131 |
| YJL159W | HSP150 | 0.13487039 | 1 | 0.34429402 | 162.6564965 | 220.2552199 |
| YJR014W | TMA22 | 0.07139998 | 0.83768171 | 0.34457744 | 163.5753655 | 221.7161885 |
| YDR119W | YDR119W | 0.09966126 | 0.92031276 | 0.34462721 | 163.1313373 | 220.9858446 |
| YDR273W | DON1 | 0.06743352 | 0.83733449 | 0.34492594 | 166.798533 | 226.9251263 |
| YBR217W | ATG12 | 0.30063966 | 1 | 0.34500481 | 164.5810296 | 223.2998754 |
| YJL108C | PRM10 | 0.02329873 | 0.70990463 | 0.3450486 | 170.161402 | 232.3912612 |
| YCR101C | YCR101C | 0.05945127 | 0.82616104 | 0.34531439 | 159.6474002 | 215.2167163 |
| YBR292C | YBR292C | 0.01612275 | 0.64284628 | 0.34538563 | 165.4418703 | 224.6535428 |
| YDR233C | RTN1 | 0.06392456 | 0.83733449 | 0.34588337 | 163.4514255 | 221.3438079 |
| YDL138W | RGT2 | 0.0809811 | 0.87926513 | 0.34596105 | 169.0221508 | 230.4150466 |
| YJL155C | FBP26 | 0.05057536 | 0.80623471 | 0.34606587 | 169.7190121 | 231.5373956 |
| YJL149W | YJL149W | 0.06486283 | 0.83733449 | 0.34643314 | 164.7838385 | 223.4441951 |

| | | | | | | |
|---|---|---|---|---|---|---|
| YBR148W | YSW1 | 0.40542182 | 1 | 0.3468765 | 156.8875109 | 210.51381 |
| YBR295W | PCA1 | 0.11131293 | 0.96890919 | 0.34721615 | 153.3904582 | 204.7686295 |
| YBR172C | SMY2 | 0.32188651 | 1 | 0.34774191 | 140.6916918 | 183.998584 |
| YJL066C | MPM1 | 0.02579126 | 0.70990463 | 0.34777454 | 159.2684324 | 214.2780445 |
| YJR047C | ANB1 | 0.06584092 | 0.83733449 | 0.34784535 | 170.4909686 | 232.5637356 |
| YDR150W | NUM1 | 0.02737146 | 0.70990463 | 0.34828126 | 150.4469165 | 199.8311588 |
| YDL187C | YDL187C | 0.1245923 | 1 | 0.34849923 | 172.5156964 | 235.7791515 |
| YDR122W | KIN1 | 0.10186129 | 0.9266788 | 0.34867807 | 168.7820547 | 229.66926 |
| YIL138C | TPM2 | 0.07060926 | 0.83733449 | 0.34871096 | 176.4320954 | 242.1360305 |
| YCL076W | YCL076W | 0.03478106 | 0.75034314 | 0.34887713 | 176.8198867 | 242.7465322 |
| YIR005W | IST3 | 0.08328339 | 0.89015714 | 0.34972031 | 135.4699646 | 175.2281038 |
| YDL050C | YDL050C | 0.03276207 | 0.75034314 | 0.35029213 | 175.8614978 | 240.999612 |
| YBL003C | HTA2 | 0.31252437 | 1 | 0.35043457 | 165.4769918 | 224.0522597 |
| YBR169C | SSE2 | 0.30842202 | 1 | 0.35046926 | 163.4357997 | 220.720192 |
| YBR206W | YBR206W | 0.3209261 | 1 | 0.35048052 | 175.0560418 | 239.6619893 |
| YDR294C | DPL1 | 0.13792594 | 1 | 0.35062988 | 164.3444397 | 222.1805031 |
| YHR112C | YHR112C | 0.29417468 | 1 | 0.35096992 | 171.391935 | 233.6249458 |
| YDR025W | RPS11A | 0.03908822 | 0.75861823 | 0.35106075 | 140.249384 | 182.8446565 |
| YBL005W | PDR3 | 0.32013227 | 1 | 0.3510987 | 162.7744037 | 219.5598892 |
| YDL206W | YDL206W | 0.08907248 | 0.89743999 | 0.35146479 | 175.455819 | 240.185324 |
| YHR182W | YHR182W | 0.21820837 | 1 | 0.35180135 | 155.9678931 | 208.3723001 |
| YDL237W | YDL237W | 0.06728709 | 0.83733449 | 0.35222638 | 160.7592188 | 216.1276593 |
| YDL083C | RPS16B | 0.00253611 | 0.43208972 | 0.35254106 | 114.2094596 | 140.2014137 |
| YBL017C | PEP1 | 0.30293771 | 1 | 0.35283818 | 163.7221802 | 220.8780683 |
| YJR083C | ACF4 | 0.09656756 | 0.91297781 | 0.352889 | 175.6012821 | 240.2366964 |
| YJL084C | ALY2 | 0.02744391 | 0.70990463 | 0.35316999 | 153.5817739 | 204.303946 |
| YAR029W | YAR029W | 0.31529208 | 1 | 0.35411479 | 175.1009018 | 239.2610978 |
| YDL167C | NRP1 | 0.05399036 | 0.80700955 | 0.35415696 | 176.4590851 | 241.4697018 |
| YDR070C | FMP16 | 0.08735551 | 0.89597255 | 0.35423325 | 179.2021196 | 245.9314327 |
| YDL168W | SFA1 | 0.00829533 | 0.56297129 | 0.3545727 | 163.7581883 | 220.7105332 |
| YAL014C | SYN8 | 0.30550691 | 1 | 0.35485961 | 169.0529658 | 229.3046321 |
| YDR183W | PLP1 | 0.05439802 | 0.80825291 | 0.35491826 | 172.0032806 | 234.1065719 |
| YJL182C | YJL182C | 0.07723286 | 0.85916554 | 0.35522754 | 159.3869277 | 213.4991129 |
| YBL011W | SCT1 | 0.34606258 | 1 | 0.35537758 | 151.7403727 | 201.0141639 |
| YIL108W | YIL108W | 0.05030013 | 0.80592814 | 0.35566467 | 161.1278658 | 216.2801658 |
| YJL017W | YJL017W | 0.04942574 | 0.80391719 | 0.35585585 | 163.0047351 | 219.3148937 |
| YDR406W | PDR15 | 0.10554991 | 0.94226883 | 0.355873 | 184.4424029 | 254.2602441 |
| YBL078C | ATG8 | 0.33409058 | 1 | 0.35603613 | 160.6473814 | 215.4484324 |
| YDL213C | NOP6 | 0.06110933 | 0.82808418 | 0.35624614 | 167.6685965 | 226.8669941 |
| YIL072W | HOP1 | 0.03330043 | 0.75034314 | 0.35648113 | 170.5029977 | 231.4569716 |
| YBR228W | SLX1 | 0.08191168 | 0.88374059 | 0.35678215 | 167.2486198 | 226.1124376 |
| YHL023C | RMD11 | 0.13055427 | 1 | 0.35725451 | 124.7931445 | 156.8401066 |
| YHR021C | RPS27B | 0.12416518 | 1 | 0.35733608 | 105.9025988 | 126.034187 |
| YDR370C | YDR370C | 0.08603056 | 0.89015714 | 0.35859547 | 173.7257971 | 236.4349878 |
| YJL012C | VTC4 | 0.04075932 | 0.76763392 | 0.35967705 | 168.0725688 | 227.07805 |
| YIL059C | YIL059C | 0.45076038 | 1 | 0.35976187 | 173.6628794 | 236.1802854 |
| YHR045W | YHR045W | 0.29403414 | 1 | 0.35989561 | 158.7355923 | 211.8284464 |
| YDR022C | CIS1 | 0.05634607 | 0.81796023 | 0.35994746 | 170.4269057 | 230.8808094 |
| YCL014W | BUD3 | 0.59516579 | 1 | 0.36015186 | 135.2192662 | 173.458818 |
| YHR073W | OSH3 | 0.45080558 | 1 | 0.36030427 | 166.7333746 | 224.8130929 |
| YDR154C | YDR154C | 0.0918856 | 0.89876254 | 0.36066831 | 170.5348964 | 230.9628332 |
| YIL047C | SYG1 | 0.378151 | 1 | 0.3615109 | 168.0758292 | 226.8441744 |
| YBL015W | ACH1 | 0.40854999 | 1 | 0.36184208 | 167.1931835 | 225.3620928 |
| YJL142C | YJL142C | 0.06704439 | 0.83733449 | 0.36191608 | 162.6653791 | 217.9712354 |
| YJR094C | IME1 | 0.03007706 | 0.74243573 | 0.36229686 | 166.292566 | 223.8345934 |
| YDR071C | PAA1 | 0.05238938 | 0.80700955 | 0.36251099 | 164.9107514 | 221.5540361 |
| YIL168W | YIL168W | 0.15660273 | 1 | 0.36290225 | 154.3700142 | 204.3195421 |
| YDL231C | BRE4 | 0.02724526 | 0.70990463 | 0.36295812 | 161.2214968 | 215.4815103 |
| YGR224W | AZR1 | 0.33855967 | 1 | 0.36328512 | 168.7119835 | 227.6498164 |
| YHR080C | YHR080C | 0.39051511 | 1 | 0.3635262 | 163.3572099 | 218.8890442 |
| YBR180W | DTR1 | 0.31829811 | 1 | 0.36353444 | 168.5857029 | 227.4114347 |
| YIR029W | DAL2 | 0.05860922 | 0.82030594 | 0.36356597 | 168.4415132 | 227.1722652 |
| YDR306C | YDR306C | 0.07028586 | 0.83733449 | 0.3648449 | 166.4140059 | 223.7002195 |
| YDL215C | GDH2 | 0.11846549 | 0.99917458 | 0.36489178 | 171.3467525 | 231.7354466 |

| | | | | | | |
|---|---|---|---|---|---|---|
| YHR138C | YHR138C | 0.44536834 | 1 | 0.36553465 | 157.3713607 | 208.8689767 |
| YDR068W | DOS2 | 0.08753545 | 0.89597255 | 0.3657585 | 165.7533478 | 222.5040563 |
| YDR222W | YDR222W | 0.06629073 | 0.83733449 | 0.36603964 | 170.7191733 | 230.5626525 |
| YDR403W | DIT1 | 0.0969534 | 0.91297781 | 0.36641282 | 173.3812512 | 234.853686 |
| YCL034W | LSB5 | 0.01726519 | 0.66607965 | 0.36641933 | 182.3875429 | 249.5348522 |
| YBL016W | FUS3 | 0.19439476 | 1 | 0.36712055 | 136.2834783 | 174.2847571 |
| YBR264C | YPT10 | 0.05719939 | 0.82030594 | 0.3673382 | 177.2495604 | 241.0390866 |
| YBR114W | RAD16 | 0.3744773 | 1 | 0.36810542 | 150.2248065 | 196.8833887 |
| YDR153C | ENT5 | 0.12369611 | 1 | 0.36838078 | 174.5265476 | 236.4640597 |
| YAR015W | ADE1 | 0.26204065 | 1 | 0.3684468 | 159.6331226 | 212.1762561 |
| YDL146W | LDB17 | 0.09156366 | 0.89876254 | 0.36889276 | 166.789046 | 223.7836413 |
| YDL010W | YDL010W | 0.07467452 | 0.85139088 | 0.36911135 | 168.3252044 | 226.2593688 |
| YDL189W | RBS1 | 0.06228952 | 0.82808418 | 0.36922357 | 166.4313318 | 223.1573493 |
| YDR102C | YDR102C | 0.13391386 | 1 | 0.37040501 | 174.3313037 | 235.8817515 |
| YDR408C | ADE8 | 0.0641942 | 0.83733449 | 0.3705361 | 164.383244 | 219.6473724 |
| YDR363W | ESC2 | 0.0892424 | 0.89743999 | 0.37105711 | 136.1788812 | 173.6007938 |
| YJL004C | SYS1 | 0.02448599 | 0.70990463 | 0.37122003 | 154.6743559 | 203.7307813 |
| YJL044C | GYP6 | 0.03189013 | 0.75034314 | 0.37134218 | 180.5249643 | 245.856391 |
| YDR336W | YDR336W | 0.12468579 | 1 | 0.37172734 | 165.5220214 | 221.348426 |
| YDR319C | YDR319C | 0.01889777 | 0.67199032 | 0.37211589 | 168.5712822 | 226.268638 |
| YDR234W | LYS4 | 0.08480003 | 0.89015714 | 0.3722432 | 156.1222657 | 205.9577044 |
| YBR229C | ROT2 | 0.01613832 | 0.64284628 | 0.37224594 | 172.248804 | 232.2467544 |
| YJL070C | YJL070C | 0.03543145 | 0.75034314 | 0.37225756 | 144.4011131 | 186.8480627 |
| YJL077C | ICS3 | 0.03868473 | 0.75834714 | 0.37225834 | 178.5884794 | 242.5800472 |
| YCR088W | ABP1 | 0.0144333 | 0.64109615 | 0.37238738 | 189.7979047 | 260.8367698 |
| YDR359C | VID21 | 0.4170405 | 1 | 0.37274593 | 111.431601 | 133.0376179 |
| YDR383C | NKP1 | 0.0357014 | 0.75147951 | 0.37363726 | 164.1449035 | 218.854343 |
| YBR239C | YBR239C | 0.13370384 | 1 | 0.37372232 | 182.7686931 | 249.2036641 |
| YCL060C | :::MRC1 | 0.01225907 | 0.61929729 | 0.37379565 | 166.1900824 | 222.167725 |
| YDL114W | YDL114W | 0.01557468 | 0.64284628 | 0.37392714 | 173.4202493 | 233.9371588 |
| YBR241C | YBR241C | 0.04703968 | 0.79308159 | 0.37417013 | 172.7362969 | 232.7904893 |
| YDR110W | FOB1 | 0.08271359 | 0.89015576 | 0.37441139 | 168.9041544 | 226.5118806 |
| YDL117W | CYK3 | 0.09395389 | 0.9084509 | 0.37455185 | 153.1156292 | 200.7551795 |
| YCR067C | SED4 | 0.02635232 | 0.70990463 | 0.37461057 | 165.1550224 | 220.3740834 |
| YDR117C | TMA64 | 0.10981378 | 0.96429522 | 0.37479509 | 177.0353917 | 239.71734 |
| YIR042C | YIR042C | 0.04652454 | 0.79308159 | 0.3750265 | 168.5453852 | 225.8467877 |
| YJL067W | YJL067W | 0.04255845 | 0.77009646 | 0.37531482 | 166.16989 | 221.9366598 |
| YDR205W | MSC2 | 0.10157206 | 0.92623613 | 0.37540331 | 173.3121365 | 233.5683753 |
| YCL002C | YCL002C | 0.05291391 | 0.80700955 | 0.3758403 | 161.6052611 | 214.4268848 |
| YDR179C | CSN9 | 0.1373539 | 1 | 0.37640197 | 163.2233646 | 216.9914505 |
| YDR219C | MFB1 | 0.00304545 | 0.43208972 | 0.37644854 | 155.1631477 | 203.8456472 |
| YJL047C | RTT101 | 0.05129497 | 0.80681531 | 0.37723153 | 144.2643264 | 185.9763125 |
| YIL166C | YIL166C | 0.01130956 | 0.59954617 | 0.37755702 | 165.4353741 | 220.4468031 |
| YDR239C | YDR239C | 0.04194904 | 0.77009646 | 0.37853139 | 161.4193954 | 213.7728852 |
| YDR185C | YDR185C | 0.05752193 | 0.82030594 | 0.37913636 | 177.6893478 | 240.2171787 |
| YDL224C | WHI4 | 0.09940384 | 0.92031276 | 0.37964308 | 178.828803 | 242.0086222 |
| YBR121C | GRS1 | 0.37249603 | 1 | 0.37968563 | 158.4695806 | 208.8135614 |
| YCL064C | CHA1 | 0.04247379 | 0.77009646 | 0.37987936 | 162.6748644 | 215.6437273 |
| YBR100W | YBR100W | 0.31584265 | 1 | 0.38124608 | 144.2285278 | 185.3943319 |
| YCR069W | CPR4 | 0.10819732 | 0.95793669 | 0.381921 | 169.7049094 | 226.8377807 |
| YDL051W | LHP1 | 0.02525827 | 0.70990463 | 0.38192974 | 158.6026493 | 208.7377876 |
| YHR152W | SPO12 | 0.26630658 | 1 | 0.38306566 | 163.081221 | 215.8905756 |
| YAR002C-A | ERP1 | 0.25147531 | 1 | 0.38307241 | 165.1051738 | 219.1891337 |
| YCL040W | GLK1 | 0.03741762 | 0.75834714 | 0.38322351 | 162.1183255 | 214.3002792 |
| YDR310C | SUM1 | 0.00428518 | 0.47636479 | 0.38381593 | 158.0119644 | 207.528838 |
| YCR036W | RBK1 | 0.14703847 | 1 | 0.38414527 | 151.493668 | 196.8597853 |
| YJR061W | YJR061W | 0.17568187 | 1 | 0.38419586 | 182.3090975 | 247.0883583 |
| YDL086W | YDL086W | 0.06099205 | 0.82808418 | 0.38455026 | 170.5233913 | 227.8291286 |
| YJL133W | MRS3 | 0.00016934 | 0.23063395 | 0.38557885 | 178.0393723 | 239.947486 |
| YDL127W | PCL2 | 0.01531094 | 0.64284628 | 0.38584578 | 175.5236668 | 235.8115791 |
| YIL099W | SGA1 | 0.06819918 | 0.83733449 | 0.38616166 | 165.7836343 | 219.8922222 |
| YDL142C | CRD1 | 0.00563584 | 0.5161257 | 0.38653492 | 164.7573161 | 218.1704385 |
| YDL155W | CLB3 | 0.04915025 | 0.80391719 | 0.38668847 | 167.2640952 | 222.236951 |
| YJL036W | SNX4 | 0.03276096 | 0.75034314 | 0.38764333 | 171.2019528 | 228.5318849 |

| | | | | | | |
|---|---|---|---|---|---|---|
| YBR174C | :::SWD3 | 0.31389521 | 1 | 0.38830985 | 106.1373121 | 122.3768725 |
| YDR133C | YDR133C | 0.11548844 | 0.98810576 | 0.38869039 | 183.9579153 | 249.1900259 |
| YDL211C | YDL211C | 0.05015556 | 0.80592814 | 0.38873602 | 167.9123658 | 223.0266938 |
| YBR235W | YBR235W | 0.02106176 | 0.70107895 | 0.38882149 | 166.9729649 | 221.4841393 |
| YJR088C | YJR088C | 0.04896239 | 0.80391719 | 0.38931207 | 177.707019 | 238.9187583 |
| YIL077C | YIL077C | 0.05591276 | 0.81725117 | 0.38937711 | 159.7558727 | 209.6463982 |
| YCR025C | YCR025C | 0.06887665 | 0.83733449 | 0.38944838 | 160.4962581 | 210.8440759 |
| YCL042W | YCL042W | 0.05595525 | 0.81725117 | 0.38979697 | 168.3161576 | 223.5465722 |
| YDR216W | ADR1 | 0.03015194 | 0.74243573 | 0.39013949 | 173.035937 | 231.1960604 |
| YBR214W | SDS24 | 0.3237173 | 1 | 0.39048378 | 164.3788885 | 217.0384731 |
| YBL039C | URA7 | 0.21721416 | 1 | 0.39052108 | 148.2249515 | 190.6995354 |
| YCL037C | SRO9 | 0.00443749 | 0.47636479 | 0.39074164 | 137.6811344 | 173.4822845 |
| YDR316W | OMS1 | 0.07705958 | 0.85916554 | 0.39106508 | 162.5002529 | 213.9001105 |
| YDR213W | UPC2 | 0.08108733 | 0.87926513 | 0.39132387 | 180.0424617 | 242.4635838 |
| YCR043C | YCR043C | 0.07033158 | 0.83733449 | 0.39139949 | 164.0790167 | 216.4301863 |
| YHL022C | SPO11 | 0.36837602 | 1 | 0.3916807 | 159.8487177 | 209.4972943 |
| YIR013C | GAT4 | 0.0785004 | 0.86626076 | 0.39169266 | 166.7564139 | 220.7566291 |
| YBR183W | YPC1 | 0.28471757 | 1 | 0.39170798 | 169.9688011 | 225.99145 |
| YCR020C-A | MAK31 | 0.05356412 | 0.80700955 | 0.39179533 | 163.688496 | 215.7419325 |
| YGR233C | PHO81 | 0.40223307 | 1 | 0.39191473 | 170.6006814 | 226.9945708 |
| YDR317W | HIM1 | 0.10159693 | 0.92623613 | 0.39230084 | 164.1763033 | 216.471219 |
| YBR296C | PHO89 | 0.03302657 | 0.75034314 | 0.39233605 | 176.2879439 | 236.210967 |
| YJL160C | YJL160C | 0.03854847 | 0.75834714 | 0.39271491 | 169.1561166 | 224.5352803 |
| YBR146W | MRPS9 | 0.40584899 | 1 | 0.39361594 | 172.396649 | 229.700459 |
| YAL062W | GDH3 | 0.27595576 | 1 | 0.39391495 | 162.4824102 | 213.4993116 |
| YIL100W | YIL100W | 0.0707166 | 0.83733449 | 0.39402498 | 181.2205691 | 244.0318208 |
| YDR104C | SPO71 | 0.03336412 | 0.75034314 | 0.3942589 | 169.0758835 | 224.2030996 |
| YDL125C | HNT1 | 0.00297008 | 0.43208972 | 0.394752 | 168.7131747 | 223.5474977 |
| YIL160C | POT1 | 0.06608418 | 0.83733449 | 0.39504307 | 159.4237586 | 208.3659709 |
| YJL136C | RPS21B | 0.04676353 | 0.79308159 | 0.39515655 | 158.5011175 | 206.847084 |
| YDL230W | PTP1 | 0.28324119 | 1 | 0.39560271 | 151.332329 | 195.1023649 |
| YDR004W | RAD57 | 0.01763244 | 0.66607965 | 0.39586707 | 162.7086504 | 213.6135108 |
| YJL145W | SFH5 | 0.02061604 | 0.69704923 | 0.3962608 | 170.8176162 | 226.7813542 |
| YDL130W-A | STF1 | 0.02253255 | 0.70990463 | 0.39693864 | 164.664871 | 216.6627666 |
| YJR030C | YJR030C | 0.07401359 | 0.85139088 | 0.39751032 | 171.3978648 | 227.5642964 |
| YJL116C | NCA3 | 0.00319987 | 0.43208972 | 0.39815214 | 184.4491255 | 248.7566891 |
| YBR232C | YBR232C | 0.02372223 | 0.70990463 | 0.3984928 | 175.0213761 | 233.3431821 |
| YDR142C | PEX7 | 0.10716721 | 0.95274538 | 0.39893234 | 165.6567406 | 218.0196673 |
| YDL178W | DLD2 | 0.01532247 | 0.64284628 | 0.39984656 | 174.5991689 | 232.478329 |
| YDL121C | YDL121C | 0.01904913 | 0.67199032 | 0.40019207 | 186.1019393 | 251.185028 |
| YDR077W | SED1 | 0.02593807 | 0.70990463 | 0.40055112 | 190.1508936 | 257.7387833 |
| YDR139C | RUB1 | 0.03976881 | 0.75896559 | 0.40064335 | 177.7159204 | 237.4553169 |
| YJL083W | TAX4 | 0.07497806 | 0.85139088 | 0.40098721 | 173.4435567 | 230.4456791 |
| YDR108W | GSG1 | 0.02461415 | 0.70990463 | 0.40115504 | 150.7153283 | 193.372336 |
| YBR259W | YBR259W | 0.06769734 | 0.83733449 | 0.4018491 | 164.9559605 | 216.496823 |
| YCL032W | STE50 | 0.0024149 | 0.43206596 | 0.40336835 | 178.2896676 | 238.0352125 |
| YDR090C | YDR090C | 0.11191919 | 0.96890919 | 0.40339643 | 177.8290244 | 237.2806122 |
| YJL150W | YJL150W | 0.06537131 | 0.83733449 | 0.40381065 | 159.3674792 | 207.1306584 |
| YBR185C | MBA1 | 0.19815024 | 1 | 0.40453185 | 149.985655 | 191.7423849 |
| YDR059C | UBC5 | 0.03368017 | 0.75034314 | 0.40486839 | 162.384611 | 211.9112115 |
| YDR334W | SWR1 | 0.10403816 | 0.93656155 | 0.40631614 | 169.1181077 | 222.699295 |
| YJR051W | OSM1 | 0.04377262 | 0.77602255 | 0.40710357 | 173.3286031 | 229.4605193 |
| YJL197W | UBP12 | 0.00668452 | 0.53154321 | 0.40741119 | 187.427849 | 252.4049215 |
| YDL137W | ARF2 | 0.02045354 | 0.69704376 | 0.40803427 | 168.2002276 | 220.9788732 |
| YDL170W | UGA3 | 0.06778586 | 0.83733449 | 0.40811373 | 164.7539303 | 215.3503703 |
| YDL243C | AAD4 | 0.05367167 | 0.80700955 | 0.40894479 | 173.6574554 | 229.75646 |
| YDR282C | YDR282C | 0.05580398 | 0.81725117 | 0.40929769 | 177.6918105 | 236.287219 |
| YCR045C | YCR045C | 0.00592123 | 0.5161257 | 0.40939337 | 151.2751292 | 193.210387 |
| YHR049W | FSH1 | 0.30176047 | 1 | 0.40948779 | 178.4699522 | 237.5309461 |
| YJL048C | UBX6 | 0.0273841 | 0.70990463 | 0.40960161 | 173.0854157 | 228.7382535 |
| YDL240W | LRG1 | 0.01888286 | 0.67199032 | 0.40998201 | 183.2143812 | 245.2008312 |
| YDR309C | GIC2 | 0.02652445 | 0.70990463 | 0.41008683 | 166.9214932 | 218.6265685 |
| YBL062W | YBL062W | 0.24519025 | 1 | 0.41071119 | 165.979496 | 217.0094929 |
| YJL049W | YJL049W | 0.00309614 | 0.43208972 | 0.41113475 | 162.5040763 | 211.2886342 |

| | | | | | | |
|---|---|---|---|---|---|---|
| YJL141C | YAK1 | 0.11214436 | 0.96890919 | 0.41157972 | 172.3560722 | 227.2912752 |
| YJR052W | RAD7 | 0.05474664 | 0.81062778 | 0.41226207 | 170.1771348 | 223.6501816 |
| YDR284C | DPP1 | 0.02948479 | 0.74039574 | 0.41251255 | 166.2534685 | 217.221168 |
| YDR083W | RRP8 | 0.00150455 | 0.37922285 | 0.41368344 | 126.1962426 | 151.7673429 |
| YDR218C | SPR28 | 0.08427454 | 0.89015714 | 0.41371825 | 178.4874197 | 237.0076389 |
| YDR285W | ZIP1 | 0.04150342 | 0.77009646 | 0.41419162 | 170.8353821 | 224.4715802 |
| YDR161W | YDR161W | 0.06857302 | 0.83733449 | 0.41452849 | 170.4118744 | 223.7372409 |
| YDR193W | YDR193W | 0.03645924 | 0.75834714 | 0.41490852 | 177.1704074 | 234.7054025 |
| YDL169C | UGX2 | 0.02313051 | 0.70990463 | 0.41508533 | 180.0972586 | 239.4536807 |
| YCR060W | TAH1 | 0.08579121 | 0.89015714 | 0.41511994 | 150.7096021 | 191.5415443 |
| YBR263W | SHM1 | 0.04401723 | 0.77602255 | 0.4151637 | 160.7002546 | 207.8225524 |
| YJL098W | SAP185 | 0.01622282 | 0.64284628 | 0.41647457 | 181.5080252 | 241.5723054 |
| YJL170C | ASG7 | 0.04275415 | 0.77009646 | 0.41699841 | 173.5412708 | 228.5166139 |
| YDR073W | SNF11 | 0.10862254 | 0.95972263 | 0.41872144 | 196.7551956 | 266.135111 |
| YJR069C | HAM1 | 0.02204693 | 0.70990463 | 0.41903771 | 168.0147555 | 219.2413265 |
| YCL028W | RNQ1 | 0.03331449 | 0.75034314 | 0.41921419 | 179.4819579 | 237.9120887 |
| YDR410C | STE14 | 0.04789238 | 0.80331985 | 0.41991575 | 176.116191 | 232.3337254 |
| YDR123C | INO2 | 0.02405724 | 0.70990463 | 0.42052555 | 140.4928508 | 174.1811834 |
| YJL046W | YJL046W | 0.22029995 | 1 | 0.42069179 | 85.27195159 | 84.13864617 |
| YDR380W | ARO10 | 0.0751702 | 0.85139088 | 0.42111155 | 167.4096363 | 217.9843708 |
| YDR124W | YDR124W | 0.05864786 | 0.82030594 | 0.42179894 | 173.4698883 | 227.774108 |
| YJL132W | YJL132W | 0.04448173 | 0.77613727 | 0.42185231 | 164.7840844 | 213.6075905 |
| YIL148W | RPL40A | 0.01438953 | 0.64109615 | 0.4222169 | 157.0932673 | 201.0225017 |
| YCR020C | PET18 | 0.00322004 | 0.43208972 | 0.42431151 | 164.694502 | 213.1407963 |
| YDR094W | YDR094W | 0.08891953 | 0.89743999 | 0.42450286 | 178.2416028 | 235.2002604 |
| YIL114C | POR2 | 0.01799377 | 0.66607965 | 0.4246244 | 181.9257519 | 241.1902908 |
| YDR363W-A | SEM1 | 0.00437342 | 0.47636479 | 0.42489898 | 150.1068512 | 189.2834511 |
| YDR169C | STB3 | 0.03547267 | 0.75034314 | 0.4253198 | 170.6686923 | 222.748382 |
| YCR030C | SYP1 | 0.00233822 | 0.43206596 | 0.42534433 | 164.4340693 | 212.5815294 |
| YBR286W | APE3 | 0.0470973 | 0.79308159 | 0.42557906 | 172.4121008 | 225.5566634 |
| YIL060W | YIL060W | 0.00607697 | 0.5161257 | 0.42561349 | 165.1009657 | 213.6335931 |
| YCR077C | PAT1 | 0.02334532 | 0.70990463 | 0.42628303 | 129.8300531 | 156.0477859 |
| YJL183W | MNN11 | 0.03431017 | 0.75034314 | 0.42636256 | 157.469332 | 201.0948369 |
| YIL119C | RPI1 | 0.02801061 | 0.72022488 | 0.4266439 | 164.5134627 | 212.5414511 |
| YDR008C | YDR008C | 0.05296735 | 0.80700955 | 0.42678637 | 164.4837598 | 212.4744474 |
| YDL106C | PHO2 | 0.03166427 | 0.75034314 | 0.42716423 | 179.5775764 | 237.0310326 |
| YJR095W | SFC1 | 0.01951782 | 0.68137812 | 0.42726723 | 144.3383332 | 179.5707466 |
| YDL078C | MDH3 | 0.03947775 | 0.75896559 | 0.42738379 | 169.4865985 | 220.5521291 |
| YCR090C | YCR090C | 0.00932462 | 0.56297129 | 0.42753665 | 188.119393 | 250.9072868 |
| YJL186W | MNN5 | 0.01841406 | 0.67064278 | 0.42838886 | 168.9509826 | 219.5478782 |
| YDR220C | YDR220C | 0.10281947 | 0.93121481 | 0.42857284 | 175.1178351 | 229.5770567 |
| YOR125C | CAT5 | 0.44232085 | 1 | 0.42879163 | 127.6183451 | 152.11507 |
| YDL236W | PHO13 | 0.0608167 | 0.82808418 | 0.42881433 | 161.8393959 | 207.8991085 |
| YIR017C | MET28 | 0.04233682 | 0.77009646 | 0.42976717 | 181.5470548 | 239.9021632 |
| YDR391C | YDR391C | 0.04234923 | 0.77009646 | 0.43019595 | 171.6179696 | 223.6598883 |
| YDR125C | ECM18 | 0.0184294 | 0.67064278 | 0.43028379 | 175.4026432 | 229.8181881 |
| YDL070W | BDF2 | 0.03401602 | 0.75034314 | 0.4303394 | 151.538325 | 190.9074342 |
| YHR125W | YHR125W | 0.29257858 | 1 | 0.43179384 | 154.7073463 | 195.8838537 |
| YDR179W-A | YDR179W-A | 0.06460615 | 0.83733449 | 0.43193141 | 169.0699752 | 219.279801 |
| YDR340W | YDR340W | 0.04392715 | 0.77602255 | 0.43201053 | 169.6167199 | 220.1607825 |
| YIR033W | MGA2 | 0.09757563 | 0.9152552 | 0.43222928 | 163.9840581 | 210.9499117 |
| YDR105C | TMS1 | 0.03386587 | 0.75034314 | 0.43227195 | 164.8475006 | 212.351925 |
| YGR168C | YGR168C | 0.16065914 | 1 | 0.43231207 | 135.5295731 | 164.5527424 |
| YJL055W | YJL055W | 0.02025003 | 0.69562886 | 0.43292975 | 165.2358182 | 212.899161 |
| YIL096C | YIL096C | 0.00154477 | 0.37922285 | 0.4331436 | 168.4863981 | 218.1703485 |
| YBR271W | YBR271W | 0.00858606 | 0.56297129 | 0.43323309 | 167.6139044 | 216.7363415 |
| YJL192C | SOP4 | 0.0084991 | 0.56297129 | 0.43366457 | 165.7021707 | 213.5635632 |
| YDR386W | MUS81 | 0.0286041 | 0.72930271 | 0.43377088 | 178.3863896 | 234.2274532 |
| YJR050W | ISY1 | 0.04104484 | 0.76963549 | 0.4349056 | 172.0003571 | 223.668969 |
| YCL027W | FUS1 | 0.10072635 | 0.92616478 | 0.43501992 | 179.3706707 | 235.6691091 |
| YGR178C | PBP1 | 0.19714582 | 1 | 0.43572466 | 160.2277085 | 204.3704213 |
| YDL093W | PMT5 | 0.0598286 | 0.82753359 | 0.43637334 | 162.3999911 | 207.8270574 |
| YDR229W | IVY1 | 0.04016999 | 0.75986753 | 0.43648838 | 169.9528058 | 220.1246161 |
| YJR043C | POL32 | 0.06723034 | 0.83733449 | 0.43677519 | 145.2470368 | 179.8119771 |

| | | | | | | |
|---|---|---|---|---|---|---|
| YHR132W-A | IGO2 | 0.18552517 | 1 | 0.43705617 | 165.7609051 | 213.216942 |
| YHR189W | PTH1 | 0.03758717 | 0.75834714 | 0.43742726 | 142.0209482 | 174.4677721 |
| YCR085W | YCR085W | 0.00905306 | 0.56297129 | 0.43929906 | 157.7487721 | 199.8630577 |
| YDR147W | EKI1 | 0.03195413 | 0.75034314 | 0.44194564 | 183.3664852 | 241.2797384 |
| YCR049C | YCR049C | 0.01391664 | 0.63572394 | 0.44219079 | 175.7666556 | 228.8585558 |
| YBL081W | YBL081W | 0.31209829 | 1 | 0.44240017 | 161.2962709 | 205.2416926 |
| YDL179W | PCL9 | 0.00495709 | 0.48411182 | 0.44241486 | 182.0200443 | 239.0235753 |
| YDR313C | PIB1 | 0.04853017 | 0.80391719 | 0.44243347 | 160.4970488 | 203.9344611 |
| YCL025C | AGP1 | 0.00167798 | 0.37922285 | 0.44326514 | 176.3676075 | 229.698097 |
| YDR278C | YDR278C | 0.0250723 | 0.70990463 | 0.44414839 | 169.9832067 | 219.1750727 |
| YDL183C | YDL183C | 0.01519024 | 0.64284628 | 0.4450617 | 130.644953 | 154.9269092 |
| YAL018C | YAL018C | 0.35053597 | 1 | 0.44793519 | 170.9036911 | 220.1817257 |
| YJL059W | YHC3 | 0.2092549 | 1 | 0.44800166 | 177.7777891 | 231.379179 |
| YDL001W | RMD1 | 0.01121141 | 0.59954617 | 0.44900539 | 182.8566898 | 239.5278613 |
| YPR191W | QCR2 | 0.4040405 | 1 | 0.44958425 | 96.34382822 | 98.41949236 |
| YDR192C | NUP42 | 0.03025763 | 0.74243573 | 0.44983855 | 169.307487 | 217.331344 |
| YDL085W | NDE2 | 0.01381496 | 0.63572394 | 0.45028308 | 182.78779 | 239.2488907 |
| YCL036W | GFD2 | 0.01182854 | 0.61929729 | 0.45382116 | 171.572493 | 220.5042897 |
| YDR360W | YDR360W | 0.03885337 | 0.75834714 | 0.45464035 | 164.2006657 | 208.3799232 |
| YBR014C | YBR014C | 0.361129 | 1 | 0.45595892 | 183.60771 | 239.8452143 |
| YDL181W | INH1 | 0.19932309 | 1 | 0.45640379 | 150.0019058 | 185.0031635 |
| YDR227W | SIR4 | 0.06735377 | 0.83733449 | 0.45719219 | 165.9502995 | 210.8993291 |
| YCL023C | YCL023C | 0.00957614 | 0.56328667 | 0.45755023 | 180.9194182 | 235.2552172 |
| YDL182W | LYS20 | 0.00121926 | 0.37922285 | 0.45774481 | 136.0398029 | 162.0672969 |
| YCR050C | YCR050C | 0.00819253 | 0.56297129 | 0.45813631 | 160.6252576 | 202.0953278 |
| YHR081W | LRP1 | 0.10761295 | 0.95473146 | 0.45875543 | 122.8589255 | 140.4480741 |
| YDL079C | MRK1 | 0.01463145 | 0.64109615 | 0.45999057 | 166.1331119 | 210.8323523 |
| YCL001W-A | YCL001W-A | 0.01236638 | 0.61929729 | 0.45999872 | 163.4894606 | 206.5216218 |
| YCL024W | KCC4 | 0.00376949 | 0.47188494 | 0.46046045 | 166.7121122 | 211.7149499 |
| YJR003C | YJR003C | 0.01316678 | 0.62820154 | 0.46060488 | 170.4492402 | 217.7883602 |
| YBR240C | THI2 | 0.02876742 | 0.72930271 | 0.46174226 | 172.7454831 | 221.3833346 |
| YER153C | PET122 | 0.38113521 | 1 | 0.46212478 | 79.98349832 | 70.11328281 |
| YDR165W | TRM82 | 0.06078461 | 0.82808418 | 0.46291832 | 178.1124901 | 229.9792096 |
| YJL201W | ECM25 | 0.00485126 | 0.48411182 | 0.46298096 | 188.9504514 | 247.6390341 |
| YDL233W | YDL233W | 0.05847002 | 0.82030594 | 0.46303511 | 178.9897506 | 231.3940836 |
| YBR277C | YBR277C | 0.01054622 | 0.58812289 | 0.4642555 | 145.9660942 | 177.3998943 |
| YJR075W | HOC1 | 0.03834902 | 0.75834714 | 0.4651031 | 166.5109932 | 210.7815403 |
| YDR421W | ARO80 | 0.09369572 | 0.9084509 | 0.46513721 | 186.9339258 | 244.0704611 |
| YJR077C | MIR1 | 0.01448262 | 0.64109615 | 0.46524781 | 133.2639627 | 156.5635103 |
| YJL016W | YJL016W | 0.03106064 | 0.75034314 | 0.46652 | 182.7208728 | 237.0220033 |
| YDL177C | YDL177C | 0.01209468 | 0.61929729 | 0.46812851 | 170.2619855 | 216.5017834 |
| YBL071C | YBL071C | 0.17226894 | 1 | 0.46917918 | 163.227634 | 204.8973752 |
| YML129C | COX14 | 0.43272056 | 1 | 0.46977867 | 107.8222148 | 114.4975249 |
| YBR238C | YBR238C | 0.01043281 | 0.58812289 | 0.47040355 | 179.5673807 | 231.3746597 |
| YDR109C | YDR109C | 0.00932417 | 0.56297129 | 0.47111927 | 170.7883462 | 216.9697651 |
| YDL041W | YDL041W | 0.01387896 | 0.63572394 | 0.47234664 | 165.9442662 | 208.9128814 |
| YDL159W | STE7 | 0.05345539 | 0.80700955 | 0.47329356 | 187.4453341 | 243.8403148 |
| YDR132C | YDR132C | 0.03729432 | 0.75834714 | 0.47374579 | 168.2979475 | 212.5673486 |
| YDR258C | HSP78 | 0.05243236 | 0.80700955 | 0.4761284 | 167.0990259 | 210.3021067 |
| YOL095C | HMI1 | 0.40697075 | 1 | 0.47827681 | 104.4050769 | 107.8185013 |
| YDR089W | YDR089W | 0.07996274 | 0.87147213 | 0.47838953 | 169.4683649 | 213.8696704 |
| YCR092C | MSH3 | 0.01798088 | 0.66607965 | 0.47878614 | 191.659184 | 249.9933111 |
| YDL059C | RAD59 | 0.00294292 | 0.43208972 | 0.47923555 | 172.9322218 | 219.4060858 |
| YCR033W | SNT1 | 0.04416419 | 0.77602255 | 0.47925998 | 154.0414414 | 188.6072369 |
| YDR101C | ARX1 | 0.01086501 | 0.59813257 | 0.47953077 | 144.0689605 | 172.3148246 |
| YJR098C | YJR098C | 0.06629857 | 0.83733449 | 0.4811999 | 167.8160803 | 210.8095644 |
| YJL130C | URA2 | 0.00387543 | 0.47188494 | 0.48164636 | 174.0882623 | 220.9762136 |
| YJL123C | MTC1 | 0.00821257 | 0.56297129 | 0.48175268 | 179.0770648 | 229.0950686 |
| YBR266C | YBR266C | 0.37775327 | 1 | 0.48298479 | 128.3745855 | 146.2794158 |
| YIR028W | DAL4 | 0.00833836 | 0.56297129 | 0.48374306 | 188.1188753 | 243.5753789 |
| YNL315C | ATP11 | 0.31526473 | 1 | 0.48529858 | 89.24572919 | 82.18994455 |
| YBR297W | MAL33 | 0.00559826 | 0.5161257 | 0.48585803 | 159.4899843 | 196.628836 |
| YJL105W | SET4 | 0.00756222 | 0.56297129 | 0.48654226 | 161.2017451 | 199.3300949 |
| YJR037W | YJR037W | 0.03513365 | 0.75034314 | 0.48882994 | 182.3533301 | 233.5129272 |

| | | | | | | |
|---|---|---|---|---|---|---|
| YIL156W | UBP7 | 0.01305827 | 0.62820154 | 0.48928217 | 174.4178072 | 220.5174892 |
| YBR106W | PHO88 | 0.10019244 | 0.9232325 | 0.49020216 | 126.7847078 | 142.7462346 |
| YIL011W | TIR3 | 0.37524282 | 1 | 0.49050596 | 212.1846043 | 281.9251267 |
| YDR329C | PEX3 | 0.03451862 | 0.75034314 | 0.4918702 | 178.8261495 | 227.3663892 |
| YCR019W | MAK32 | 0.02297473 | 0.70990463 | 0.49369053 | 178.7663497 | 227.0314758 |
| YIL070C | MAM33 | 0.04485281 | 0.77613727 | 0.49436289 | 189.0488821 | 243.7063163 |
| YIL135C | VHS2 | 0.00815855 | 0.56297129 | 0.49436737 | 188.1249952 | 242.1996154 |
| YDR076W | RAD55 | 0.07534433 | 0.85139088 | 0.49604226 | 160.9591668 | 197.6955499 |
| YIL105C | SLM1 | 0.00336069 | 0.43729742 | 0.49612386 | 181.8046878 | 231.6671788 |
| YLL006W | MMM1 | 0.56152329 | 1 | 0.49729156 | 81.2729244 | 67.62845704 |
| YDR130C | FIN1 | 0.03865648 | 0.75834714 | 0.49838485 | 174.1668773 | 218.9211522 |
| YBR242W | YBR242W | 0.00209337 | 0.40858765 | 0.50153303 | 198.3505831 | 257.9346966 |
| YCL055W | KAR4 | 0.01377755 | 0.63572394 | 0.50182768 | 172.6059892 | 215.927547 |
| YBR298C | MAL31 | 0.00056707 | 0.34785756 | 0.50204793 | 163.2112336 | 200.5835319 |
| YCL061C | MRC1 | 0.00395619 | 0.47188494 | 0.50247596 | 147.694435 | 175.2322903 |
| YDR111C | ALT2 | 0.01122897 | 0.59954617 | 0.5028122 | 175.5125856 | 220.5374555 |
| YJL038C | YJL038C | 0.00284528 | 0.43208972 | 0.50456913 | 172.4412591 | 215.3014349 |
| YJL206C-A | YJL206C-A | 0.02570569 | 0.70990463 | 0.50833614 | 181.5951475 | 229.7327253 |
| YJL088W | ARG3 | 0.03801555 | 0.75834714 | 0.51018556 | 171.6074206 | 213.209557 |
| YIL054W | YIL054W | 0.35623127 | 1 | 0.51498254 | 194.6108064 | 250.083896 |
| YDR286C | YDR286C | 0.02698861 | 0.70990463 | 0.51540862 | 170.7331591 | 211.1030912 |
| YJL100W | LSB6 | 0.07368255 | 0.85139088 | 0.51622099 | 185.1928077 | 234.5691845 |
| YJL028W | YJL028W | 0.12740595 | 1 | 0.51740007 | 192.9149912 | 247.0040646 |
| YCR005C | CIT2 | 0.01796804 | 0.66607965 | 0.51793673 | 165.7277836 | 202.6136065 |
| YIL117C | PRM5 | 0.01909241 | 0.67199032 | 0.51823471 | 175.391957 | 218.3292315 |
| YDR314C | RAD34 | 0.02552183 | 0.70990463 | 0.51866081 | 179.9280221 | 225.6683271 |
| YBR248C | HIS7 | 0.04500656 | 0.77613727 | 0.52165259 | 161.3539328 | 194.9987121 |
| YJL176C | SWI3 | 0.01305396 | 0.62820154 | 0.52248622 | 167.5859373 | 205.0493657 |
| YJL181W | YJL181W | 0.06129763 | 0.82808418 | 0.52345385 | 178.5840203 | 222.8521808 |
| YDR066C | YDR066C | 0.02616322 | 0.70990463 | 0.52373068 | 175.04166 | 217.0413333 |
| YJL073W | JEM1 | 0.01575919 | 0.64284628 | 0.52379843 | 170.6145441 | 209.8154329 |
| YPR069C | SPE3 | 0.37916201 | 1 | 0.52484434 | 87.68290667 | 74.48424095 |
| YGL173C | KEM1 | 0.08530412 | 0.89015714 | 0.52485717 | 89.52974737 | 77.4932799 |
| YIR038C | GTT1 | 0.02096865 | 0.70107895 | 0.52606586 | 175.5182565 | 217.5136985 |
| YIR025W | MND2 | 0.00456311 | 0.47790275 | 0.52708971 | 168.2306167 | 205.4998798 |
| YIL097W | FYV10 | 0.00139715 | 0.37922285 | 0.52713643 | 159.18967 | 190.7552774 |
| YBR273C | UBX7 | 0.02579054 | 0.70990463 | 0.52749645 | 183.5685351 | 230.450632 |
| YIL107C | PFK26 | 0.00129303 | 0.37922285 | 0.52767736 | 174.9977817 | 216.4550337 |
| YHL030W | ECM29 | 0.26832695 | 1 | 0.53096436 | 176.1231957 | 217.8609518 |
| YDR244W | PEX5 | 0.03327276 | 0.75034314 | 0.53097535 | 182.9053212 | 228.9157077 |
| YBL089W | AVT5 | 0.25639332 | 1 | 0.53116121 | 144.9779883 | 167.0625028 |
| YIL074C | SER33 | 0.0200056 | 0.69277445 | 0.53453942 | 199.2580698 | 255.1090175 |
| YJL172W | CPS1 | 0.00106948 | 0.37922285 | 0.53473085 | 149.4297039 | 173.8540769 |
| YDL218W | YDL218W | 0.02579648 | 0.70990463 | 0.53605433 | 191.9231275 | 242.9540372 |
| YBR276C | PPS1 | 0.0061635 | 0.5161257 | 0.53818477 | 172.9384484 | 211.7274245 |
| YDR158W | HOM2 | 0.00011504 | 0.23063395 | 0.54287703 | 147.1421045 | 169.0623272 |
| YJR019C | TES1 | 0.00941374 | 0.56297129 | 0.54328485 | 172.3268765 | 210.0652342 |
| YCR073W-A | SOL2 | 0.01295636 | 0.62820154 | 0.54390965 | 173.5605025 | 211.9947926 |
| YDR148C | KGD2 | 0.0101901 | 0.58341717 | 0.54751453 | 160.1181421 | 189.6109302 |
| YDR173C | ARG82 | 0.07420979 | 0.85139088 | 0.55032076 | 127.5696663 | 136.1845355 |
| YOR033C | EXO1 | 0.00196044 | 0.40086403 | 0.55081732 | 115.13022 | 115.841041 |
| YJR024C | YJR024C | 0.01008596 | 0.58341717 | 0.55705862 | 198.1223477 | 250.3203647 |
| YJL199C | MBB1 | 0.00496062 | 0.48411182 | 0.55902632 | 181.743648 | 223.3632338 |
| YIR034C | LYS1 | 0.0397119 | 0.75896559 | 0.56401343 | 151.1683889 | 172.8691136 |
| YDL034W | YDL034W | 0.00567809 | 0.5161257 | 0.57810306 | 183.5643728 | 223.8431705 |
| YDR304C | CPR5 | 0.01540844 | 0.64284628 | 0.59344627 | 173.4638714 | 205.3761486 |
| YDL242W | YDL242W | 0.01727333 | 0.66607965 | 0.59355622 | 166.9195965 | 194.6933602 |
| YDL065C | PEX19 | 0.32274451 | 1 | 0.60257788 | 168.3535881 | 195.8543424 |
| YIL116W | HIS5 | 0.00865412 | 0.56297129 | 0.60437402 | 192.0348346 | 234.2251279 |
| YHR193C | EGD2 | 0.14227654 | 1 | 0.62368163 | 210.1692214 | 261.2694089 |
| YJR008W | YJR008W | 0.00628545 | 0.5161257 | 0.62549152 | 178.5362328 | 209.4653912 |
| YJL022W | YJL022W | 0.00095762 | 0.37922285 | 0.63373025 | 186.5781879 | 221.5007627 |
| YDR375C | BCS1 | 0.00037864 | 0.3251748 | 0.6479615 | 146.7940084 | 154.7885794 |
| YNL081C | SWS2 | 0.2190325 | 1 | 0.6496228 | 125.9490317 | 120.5905089 |

| | | | | | | |
|---|---|---|---|---|---|---|
| YDR074W | TPS2 | 0.03533335 | 0.75034314 | 0.65418609 | 170.0210238 | 191.8412722 |
| YOR065W | CYT1 | 0.11952389 | 0.99917458 | 0.66791949 | 72.78379803 | 31.53433357 |
| YJL065C | DLS1 | 0.03843103 | 0.75834714 | 0.67307721 | 188.3540481 | 219.2636943 |
| YCR095C | OCA4 | 0.00021484 | 0.23063395 | 0.67973317 | 215.800523 | 263.1386667 |
| YDR174W | HMO1 | 0.02442254 | 0.70990463 | 0.68020062 | 129.4424503 | 122.2971656 |
| YNL003C | PET8 | 0.23568266 | 1 | 0.68236207 | 105.1012631 | 82.33435343 |
| YDR393W | SHE9 | 0.24008838 | 1 | 0.70308061 | 135.1887929 | 128.6805634 |
| YDL191W | RPL35A | 0.14617387 | 1 | 0.70969019 | 144.0061359 | 142.1924604 |
| YBR269C | FMP21 | 0.02183997 | 0.70990463 | 0.71667739 | 175.113689 | 191.9925027 |
| YPL178W | CBC2 | 0.14603177 | 1 | 0.71807524 | 78.98231906 | 35.09726312 |
| YIR020C | YIR020C | 0.00048583 | 0.34768968 | 0.73857529 | 191.2143969 | 215.3836341 |
| YGR101W | PCP1 | 0.22938708 | 1 | 0.76895464 | 100.63008 | 63.75108729 |
| YCR071C | IMG2 | 0.05387774 | 0.80700955 | 0.77232192 | 121.3547763 | 97.09719338 |
| YDL104C | QRI7 | 0.09677472 | 0.91297781 | 0.77640569 | 138.0395742 | 123.7640231 |
| YOR293W | RPS10A | 0.1164577 | 0.99259248 | 0.90355863 | 72.29326784 | 0 |
| YCR028C-A | RIM1 | 0.05121593 | 0.80681531 | 0.93143694 | 143.5269231 | 112.4885759 |
| YDR245W | MNN10 | 0.04286284 | 0.77009646 | 0.94890379 | 141.3599179 | 106.6777144 |
| YAL010C | MDM10 | 0.01240325 | 0.61929729 | 0.96066 | 93.98412975 | 27.91254735 |
| YJR054W | YJR054W | 0.00184548 | 0.39622473 | 0.98290067 | 231.4465469 | 249.1022695 |
| YBR122C | MRPL36 | 0.04500492 | 0.77613727 | 1.05629868 | 111.6997326 | 44.31818219 |
| YIL134W | FLX1 | 0.05069478 | 0.80623471 | 1.12881975 | 120.6273465 | 49.41293443 |
| YPL172C | COX10 | 0.19830538 | 1 | 1.22185684 | 143.6971086 | 74.88622338 |
| YDL062W | YDL062W | 0.00424882 | 0.47636479 | 1.56993602 | 125.6097853 | 0 |

**Table S5 Fitness on TMPyP4**

**Key**

| | |
|---|---|
| ORF: | Open reading frame Y-number for *yfg* deletion |
| Gene: | Standard human-readable gene name for *yfg* deletion |
| Std Error: | Standard error for strain fitness (in CSM and TMPyP4 screen) |
| t-value: | t- statistic for the significance of difference between observed mean fitness of TMPyP4 treated strains and predicted fitness estimated from mean fitness observed on CSM |
| P: | p-value for the significance of difference between observed mean fitness of TMPyP4 treated strains and predicted fitness estimated from mean fitness observed on CSM |
| Q: | False discovery rate corrected p-value (correcting for multiple testing) |
| FFD: | Final fitness differential (difference between observed mean fitness of TMPyP4 treated strains and predicted fitness estimated from mean fitness observed on CSM) |

**Supplementary Table 5: TMPyP4 Screen 2**

| ORF | Gene | Std Error | t-value | P | Q | FFD |
|---|---|---|---|---|---|---|
| YBR170C | NPL4 | 0.15269937 | -5.3650175 | 8.16E-08 | 0.000350297 | -0.819234768 |
| YJL117W | PHO86 | 0.15269937 | -5.2182845 | 1.82E-07 | 0.000390433 | -0.796828738 |
| YHR008C | SOD2 | 0.15269937 | -5.1296013 | 2.92E-07 | 0.000418298 | -0.783286861 |
| YKL109W | HAP4 | 0.15269937 | -4.6852422 | 2.81E-06 | 0.003014947 | -0.71543352 |
| YJL120W | YJL120W | 0.15269937 | -4.5181633 | 6.27E-06 | 0.005376948 | -0.689920676 |
| YMR038C | CCS1 | 0.15269937 | -4.3144223 | 1.61E-05 | 0.011486509 | -0.658809545 |
| YLR200W | YKE2 | 0.15269937 | -3.9056844 | 9.42E-05 | 0.057742965 | -0.596395526 |
| YML007W | YAP1 | 0.15269937 | -3.7475477 | 0.00017896 | 0.085323839 | -0.572248153 |
| YPR074C | TKL1 | 0.15269937 | -3.3639886 | 0.00076936 | 0.330132615 | -0.513678921 |
| YNL241C | ZWF1 | 0.15269937 | -3.2409051 | 0.00119301 | 0.46538339 | -0.494884148 |
| YOR349W | CIN1 | 0.15269937 | -3.1272083 | 0.0017667 | 0.631743128 | -0.477522718 |
| YMR021C | MAC1 | 0.15269937 | -3.0451384 | 0.00232807 | 0.768441252 | -0.464990707 |
| YMR315W | YMR315W | 0.15269937 | -2.6826005 | 0.00730986 | 0.999976173 | -0.409631389 |
| YJL121C | RPE1 | 0.15269937 | -2.5501054 | 0.01077474 | 0.999976173 | -0.389399473 |
| YLR371W | ROM2 | 0.15269937 | -2.4844979 | 0.01297969 | 0.999976173 | -0.379381253 |
| YGL253W | HXK2 | 0.15269937 | -2.432436 | 0.01500437 | 0.999976173 | -0.371431434 |
| YBR200W | BEM1 | 0.15269937 | -2.3384525 | 0.01937135 | 0.999976173 | -0.357080221 |
| YBR126C | TPS1 | 0.15269937 | -2.3089815 | 0.02095244 | 0.999976173 | -0.352580013 |
| YHR178W | STB5 | 0.15269937 | -2.2531284 | 0.02425938 | 0.999976173 | -0.344051281 |
| YLL028W | TPO1 | 0.15269937 | -2.2036911 | 0.02755484 | 0.999976173 | -0.336502238 |
| YDR158W | HOM2 | 0.15269937 | -2.1727142 | 0.02981086 | 0.999976173 | -0.331772086 |
| YLR237W | THI7 | 0.15269937 | -2.1412216 | 0.03226547 | 0.999976173 | -0.326963179 |
| YKL212W | SAC1 | 0.15269937 | -2.0640601 | 0.03902195 | 0.999976173 | -0.315180666 |
| YPR069C | SPE3 | 0.15269937 | -2.0531108 | 0.04007185 | 0.999976173 | -0.313508717 |
| YNL111C | CYB5 | 0.15269937 | -2.016892 | 0.04371708 | 0.999976173 | -0.307978128 |
| YLR047C | FRE8 | 0.15269937 | -2.0111259 | 0.0443225 | 0.999976173 | -0.307097648 |
| YJR145C | RPS4A | 0.15269937 | -2.0092401 | 0.04452203 | 0.999976173 | -0.306809684 |
| YML124C | TUB3 | 0.15269937 | -1.9658794 | 0.04932321 | 0.999976173 | -0.300188531 |
| YGL059W | PKP2 | 0.15269937 | -1.8835618 | 0.05963547 | 0.999976173 | -0.287618689 |
| YER052C | HOM3 | 0.15269937 | -1.8725979 | 0.06113519 | 0.999976173 | -0.28594451 |
| YPL241C | CIN2 | 0.15269937 | -1.8638801 | 0.06234985 | 0.999976173 | -0.284613305 |
| YMR216C | SKY1 | 0.15269937 | -1.8608921 | 0.06277073 | 0.999976173 | -0.284157049 |
| YOR209C | NPT1 | 0.15269937 | -1.8509847 | 0.06418313 | 0.999976173 | -0.282644184 |
| YML038C | YMD8 | 0.15269937 | -1.8310255 | 0.06710821 | 0.999976173 | -0.279596435 |
| YMR015C | ERG5 | 0.15269937 | -1.8183157 | 0.06902743 | 0.999976173 | -0.277655662 |
| YDL020C | RPN4 | 0.15269937 | -1.801159 | 0.07168944 | 0.999976173 | -0.275035839 |
| YLR330W | CHS5 | 0.15269937 | -1.7861153 | 0.07409228 | 0.999976173 | -0.272738671 |
| YMR086C-A | YMR086C-A | 0.15269937 | -1.7490036 | 0.08030224 | 0.999976173 | -0.267071735 |
| YCR086W | CSM1 | 0.15269937 | -1.7447926 | 0.08103283 | 0.999976173 | -0.266428726 |
| YIL160C | POT1 | 0.15269937 | -1.7289531 | 0.08382939 | 0.999976173 | -0.264010048 |
| YMR073C | IRC21 | 0.15269937 | -1.7106287 | 0.08716166 | 0.999976173 | -0.261211911 |
| YHR030C | SLT2 | 0.15269937 | -1.6756164 | 0.09382542 | 0.999976173 | -0.255865564 |
| YPL165C | SET6 | 0.15269937 | -1.6737998 | 0.09418202 | 0.999976173 | -0.255588174 |
| YOL052C | SPE2 | 0.15269937 | -1.6725023 | 0.09443739 | 0.999976173 | -0.255390037 |
| YGR260W | TNA1 | 0.15269937 | -1.6685752 | 0.09521368 | 0.999976173 | -0.254790374 |
| YKL184W | SPE1 | 0.15269937 | -1.6593448 | 0.09705843 | 0.999976173 | -0.253380901 |

| | | | | | | |
|---|---|---|---|---|---|---|
| YBR164C | ARL1 | 0.15269937 | -1.6366943 | 0.10170651 | 0.999976173 | -0.249922179 |
| YNL099C | OCA1 | 0.15269937 | -1.6250652 | 0.10416083 | 0.999976173 | -0.248146426 |
| YNL238W | KEX2 | 0.15269937 | -1.6182264 | 0.10562598 | 0.999976173 | -0.247102151 |
| YLL044W | YLL044W | 0.15269937 | -1.6169071 | 0.10591051 | 0.999976173 | -0.246900686 |
| YGR157W | CHO2 | 0.15269937 | -1.6114294 | 0.10709835 | 0.999976173 | -0.246064247 |
| YNL229C | URE2 | 0.15269937 | -1.6090465 | 0.10761836 | 0.999976173 | -0.245700377 |
| YKL211C | TRP3 | 0.15269937 | -1.6047614 | 0.10855852 | 0.999976173 | -0.245046045 |
| YIR033W | MGA2 | 0.15269937 | -1.6012537 | 0.10933293 | 0.999976173 | -0.244510425 |
| YLR402W | YLR402W | 0.15269937 | -1.5972972 | 0.11021167 | 0.999976173 | -0.24390627 |
| YMR009W | ADI1 | 0.15269937 | -1.5968958 | 0.11030113 | 0.999976173 | -0.243844977 |
| YGR281W | YOR1 | 0.15269937 | -1.5845665 | 0.11307707 | 0.999976173 | -0.2419623 |
| YOR300W | YOR300W | 0.10797476 | -2.2279529 | 0.02589221 | 0.999976173 | -0.240562668 |
| YMR198W | CIK1 | 0.15269937 | -1.5667786 | 0.11717866 | 0.999976173 | -0.239246101 |
| YIL013C | PDR11 | 0.15269937 | -1.5552551 | 0.11989755 | 0.999976173 | -0.237486461 |
| YIL128W | MET18 | 0.15269937 | -1.5502625 | 0.12109074 | 0.999976173 | -0.236724099 |
| YMR031W-A | YMR031W-A | 0.15269937 | -1.5493613 | 0.1213071 | 0.999976173 | -0.236586493 |
| YNL032W | SIW14 | 0.15269937 | -1.5491865 | 0.1213491 | 0.999976173 | -0.236559799 |
| YGL026C | TRP5 | 0.15269937 | -1.5470565 | 0.12186182 | 0.999976173 | -0.236234554 |
| YML035C | AMD1 | 0.15269937 | -1.5440001 | 0.12260051 | 0.999976173 | -0.235767842 |
| YGR259C | YGR259C | 0.15269937 | -1.5425022 | 0.12296383 | 0.999976173 | -0.235539101 |
| YCR034W | FEN1 | 0.15269937 | -1.5256843 | 0.12710072 | 0.999976173 | -0.232971019 |
| YOR297C | TIM18 | 0.15269937 | -1.5200182 | 0.1285186 | 0.999976173 | -0.232105819 |
| YLR138W | NHA1 | 0.15269937 | -1.5125859 | 0.13039707 | 0.999976173 | -0.23097091 |
| YBL104C | YBL104C | 0.15269937 | -1.507169 | 0.13177954 | 0.999976173 | -0.230143747 |
| YJR117W | STE24 | 0.15269937 | -1.5040787 | 0.1325733 | 0.999976173 | -0.229671862 |
| YMR048W | CSM3 | 0.15269937 | -1.503746 | 0.13265896 | 0.999976173 | -0.229621066 |
| YNL130C | CPT1 | 0.15269937 | -1.5021518 | 0.13307011 | 0.999976173 | -0.229377621 |
| YAL055W | PEX22 | 0.15269937 | -1.4932637 | 0.13538035 | 0.999976173 | -0.228020422 |
| YKL121W | DGR2 | 0.15269937 | -1.4904654 | 0.13611408 | 0.999976173 | -0.227593124 |
| YCR037C | PHO87 | 0.15269937 | -1.4820461 | 0.13834018 | 0.999976173 | -0.226307506 |
| YIL027C | KRE27 | 0.15269937 | -1.4804262 | 0.13877171 | 0.999976173 | -0.226060137 |
| YNL166C | BNI5 | 0.15269937 | -1.4773434 | 0.13959578 | 0.999976173 | -0.225589394 |
| YML121W | GTR1 | 0.15269937 | -1.4751035 | 0.14019688 | 0.999976173 | -0.225247365 |
| YPR095C | SYT1 | 0.15269937 | -1.466189 | 0.14260894 | 0.999976173 | -0.223886132 |
| YFL007W | BLM10 | 0.15269937 | -1.4617584 | 0.14381956 | 0.999976173 | -0.223209578 |
| YMR007W | YMR007W | 0.15269937 | -1.4479821 | 0.14763415 | 0.999976173 | -0.221105953 |
| YBR116C | YBR116C | 0.15269937 | -1.4478162 | 0.14768056 | 0.999976173 | -0.221080616 |
| YOR360C | PDE2 | 0.15269937 | -1.4438139 | 0.14880343 | 0.999976173 | -0.220469461 |
| YCR061W | YCR061W | 0.15269937 | -1.4319563 | 0.15216839 | 0.999976173 | -0.218658817 |
| YGR282C | BGL2 | 0.15269937 | -1.4242461 | 0.15438728 | 0.999976173 | -0.21748148 |
| YDR151C | CTH1 | 0.15269937 | -1.410375 | 0.158441 | 0.999976173 | -0.215363365 |
| YJR107W | YJR107W | 0.15269937 | -1.4069976 | 0.1594401 | 0.999976173 | -0.214847641 |
| YPL091W | GLR1 | 0.15269937 | -1.3957993 | 0.16278691 | 0.999976173 | -0.213137664 |
| YPL123C | RNY1 | 0.15269937 | -1.3946975 | 0.16311904 | 0.999976173 | -0.212969421 |
| YDR266C | YDR266C | 0.15269937 | -1.3801261 | 0.16755973 | 0.999976173 | -0.210744383 |
| YGL023C | PIB2 | 0.15269937 | -1.3764903 | 0.16868179 | 0.999976173 | -0.210189192 |
| YGL054C | ERV14 | 0.15269937 | -1.3687584 | 0.17108668 | 0.999976173 | -0.209008538 |
| YHR025W | THR1 | 0.15269937 | -1.3678535 | 0.1713698 | 0.999976173 | -0.208870361 |
| YJR059W | PTK2 | 0.15269937 | -1.3646696 | 0.17236878 | 0.999976173 | -0.208384178 |
| YJL110C | GZF3 | 0.15269937 | -1.3569591 | 0.17480604 | 0.999976173 | -0.207206788 |
| YCL026C | NA | 0.15269937 | -1.3545393 | 0.17557618 | 0.999976173 | -0.206837299 |
| YGR178C | PBP1 | 0.15269937 | -1.3454067 | 0.17850571 | 0.999976173 | -0.205442751 |
| YGL007W | BRP1 | 0.15269937 | -1.3336467 | 0.18233141 | 0.999976173 | -0.203646999 |
| YPL262W | FUM1 | 0.15269937 | -1.3303054 | 0.18342939 | 0.999976173 | -0.203136787 |
| YCL036W | GFD2 | 0.15269937 | -1.3300786 | 0.18350407 | 0.999976173 | -0.203102166 |
| YLL014W | EMC6 | 0.15269937 | -1.3215608 | 0.18632611 | 0.999976173 | -0.201801497 |
| YPR120C | CLB5 | 0.15269937 | -1.3208645 | 0.18655822 | 0.999976173 | -0.201695171 |
| YKL056C | TMA19 | 0.15269937 | -1.317522 | 0.18767537 | 0.999976173 | -0.201184771 |
| YPR060C | ARO7 | 0.15269937 | -1.3149664 | 0.18853283 | 0.999976173 | -0.200794537 |
| YDR500C | RPL37B | 0.15269937 | -1.3023167 | 0.19281973 | 0.999976173 | -0.198862929 |
| YMR171C | EAR1 | 0.15269937 | -1.3008295 | 0.19332841 | 0.999976173 | -0.198635834 |
| YKL062W | MSN4 | 0.15269937 | -1.2963135 | 0.19487905 | 0.999976173 | -0.197946248 |
| YJR073C | OPI3 | 0.15269937 | -1.2789145 | 0.20093862 | 0.999976173 | -0.195289435 |
| YGL009C | LEU1 | 0.15269937 | -1.2777449 | 0.20135084 | 0.999976173 | -0.195110836 |

| | | | | | | |
|---|---|---|---|---|---|---|
| YGR092W | DBF2 | 0.15269937 | -1.2769182 | 0.20164258 | 0.999976173 | -0.194984598 |
| YIL097W | FYV10 | 0.15269937 | -1.27283 | 0.20308981 | 0.999976173 | -0.194360327 |
| YGR055W | MUP1 | 0.15269937 | -1.2705256 | 0.20390887 | 0.999976173 | -0.194008455 |
| YOR058C | ASE1 | 0.15269937 | -1.2701239 | 0.20405191 | 0.999976173 | -0.193947112 |
| YKR027W | BCH2 | 0.15269937 | -1.2645949 | 0.20602794 | 0.999976173 | -0.193102845 |
| YDR263C | DIN7 | 0.15269937 | -1.2641859 | 0.20617468 | 0.999976173 | -0.193040385 |
| YPL046C | ELC1 | 0.15269937 | -1.2598239 | 0.20774425 | 0.999976173 | -0.192374307 |
| YPL037C | EGD1 | 0.15269937 | -1.2523103 | 0.21046813 | 0.999976173 | -0.191226985 |
| YGL020C | GET1 | 0.15269937 | -1.2508655 | 0.21099486 | 0.999976173 | -0.191006362 |
| YIL032C | YIL032C | 0.15269937 | -1.2398312 | 0.21504906 | 0.999976173 | -0.189321438 |
| YBR141C | YBR141C | 0.15269937 | -1.2366372 | 0.21623302 | 0.999976173 | -0.188833712 |
| YNL125C | ESBP6 | 0.15269937 | -1.2361381 | 0.21641843 | 0.999976173 | -0.188757508 |
| YLR190W | MMR1 | 0.15269937 | -1.2254523 | 0.22041599 | 0.999976173 | -0.18712579 |
| YNL106C | INP52 | 0.15269937 | -1.2228079 | 0.22141339 | 0.999976173 | -0.186721988 |
| YGR161C | RTS3 | 0.15269937 | -1.2204694 | 0.2222981 | 0.999976173 | -0.186364901 |
| YIL135C | VHS2 | 0.15269937 | -1.2195054 | 0.22266352 | 0.999976173 | -0.186217704 |
| YGR018C | YGR018C | 0.15269937 | -1.2152984 | 0.22426337 | 0.999976173 | -0.185575296 |
| YNL121C | TOM70 | 0.15269937 | -1.2036402 | 0.22873967 | 0.999976173 | -0.183795095 |
| YHR006W | STP2 | 0.15269937 | -1.2035474 | 0.22877555 | 0.999976173 | -0.183780926 |
| YKR041W | YKR041W | 0.15269937 | -1.2018986 | 0.2294138 | 0.999976173 | -0.183529154 |
| YDR072C | IPT1 | 0.15269937 | -1.2016308 | 0.22951759 | 0.999976173 | -0.183488261 |
| YPR013C | YPR013C | 0.15269937 | -1.1993154 | 0.2304163 | 0.999976173 | -0.183134701 |
| YKR055W | RHO4 | 0.15269937 | -1.1991911 | 0.23046463 | 0.999976173 | -0.183115713 |
| YDR173C | ARG82 | 0.15269937 | -1.1988104 | 0.23061265 | 0.999976173 | -0.183057586 |
| YGL148W | ARO2 | 0.15269937 | -1.1981154 | 0.23088307 | 0.999976173 | -0.182951455 |
| YOR322C | LDB19 | 0.15269937 | -1.1939267 | 0.23251756 | 0.999976173 | -0.182311854 |
| YBR059C | AKL1 | 0.15269937 | -1.1923305 | 0.23314262 | 0.999976173 | -0.182068104 |
| YJR147W | HMS2 | 0.15269937 | -1.1883753 | 0.23469646 | 0.999976173 | -0.181464161 |
| YFR031C-A | RPL2A | 0.15269937 | -1.1781233 | 0.23875829 | 0.999976173 | -0.179898676 |
| YOL061W | PRS5 | 0.15269937 | -1.1779779 | 0.23881623 | 0.999976173 | -0.17987648 |
| YDR247W | VHS1 | 0.15269937 | -1.1658223 | 0.24369701 | 0.999976173 | -0.178020333 |
| YDR408C | ADE8 | 0.15269937 | -1.1616962 | 0.24536959 | 0.999976173 | -0.177390273 |
| YLR451W | LEU3 | 0.15269937 | -1.1596841 | 0.24618815 | 0.999976173 | -0.177083021 |
| YKL023W | YKL023W | 0.15269937 | -1.1538489 | 0.24857279 | 0.999976173 | -0.176191988 |
| YKL176C | LST4 | 0.15269937 | -1.1411672 | 0.25381089 | 0.999976173 | -0.174255511 |
| YJR069C | HAM1 | 0.15269937 | -1.1387884 | 0.25480196 | 0.999976173 | -0.173892264 |
| YBL032W | HEK2 | 0.15269937 | -1.1385405 | 0.25490538 | 0.999976173 | -0.173854415 |
| YCL060C | NA | 0.15269937 | -1.1382641 | 0.25502076 | 0.999976173 | -0.173812202 |
| YNL168C | FMP41 | 0.15269937 | -1.1377338 | 0.25524218 | 0.999976173 | -0.173731229 |
| YER002W | NOP16 | 0.15269937 | -1.1372897 | 0.25542773 | 0.999976173 | -0.17366341 |
| YIL125W | KGD1 | 0.15269937 | -1.1277035 | 0.2594556 | 0.999976173 | -0.172199608 |
| YMR016C | SOK2 | 0.15269937 | -1.1269688 | 0.25976611 | 0.999976173 | -0.172087422 |
| YML013W | UBX2 | 0.15269937 | -1.1159506 | 0.26445361 | 0.999976173 | -0.170404955 |
| YBR147W | RTC2 | 0.15269937 | -1.1124494 | 0.26595528 | 0.999976173 | -0.169870324 |
| YIL047C | SYG1 | 0.15269937 | -1.1103145 | 0.26687382 | 0.999976173 | -0.169544324 |
| YDR127W | ARO1 | 0.15269937 | -1.1082843 | 0.26774936 | 0.999976173 | -0.169234303 |
| YER007W | PAC2 | 0.15269937 | -1.1049051 | 0.26921098 | 0.999976173 | -0.168718306 |
| YBL067C | UBP13 | 0.15269937 | -1.1040051 | 0.26960117 | 0.999976173 | -0.168580882 |
| YIL039W | TED1 | 0.15269937 | -1.0987373 | 0.27189286 | 0.999976173 | -0.167776495 |
| YHR134W | WSS1 | 0.15269937 | -1.0985868 | 0.27195854 | 0.999976173 | -0.167753511 |
| YOL118C | YOL118C | 0.15269937 | -1.0967497 | 0.27276103 | 0.999976173 | -0.16747298 |
| YOL128C | YGK3 | 0.15269937 | -1.0961247 | 0.27303439 | 0.999976173 | -0.167377547 |
| YHR194W | MDM31 | 0.15269937 | -1.0957759 | 0.27318705 | 0.999976173 | -0.167324282 |
| YER016W | BIM1 | 0.15269937 | -1.09379 | 0.27405727 | 0.999976173 | -0.167021036 |
| YLR085C | ARP6 | 0.15269937 | -1.0937009 | 0.27409637 | 0.999976173 | -0.167007428 |
| YIL159W | BNR1 | 0.15269937 | -1.0918739 | 0.27489871 | 0.999976173 | -0.166728445 |
| YGL175C | SAE2 | 0.15269937 | -1.0823831 | 0.2790924 | 0.999976173 | -0.165279217 |
| YDR360W | OPI7 | 0.15269937 | -1.0823533 | 0.27910564 | 0.999976173 | -0.165274664 |
| YHL029C | OCA5 | 0.15269937 | -1.0813052 | 0.27957145 | 0.999976173 | -0.165114613 |
| YAL059W | ECM1 | 0.15269937 | -1.0788523 | 0.28066363 | 0.999976173 | -0.164740057 |
| YNL180C | RHO5 | 0.15269937 | -1.0770827 | 0.28145333 | 0.999976173 | -0.164469851 |
| YLR372W | SUR4 | 0.15269937 | -1.0749435 | 0.28241005 | 0.999976173 | -0.164143185 |
| YBR028C | YBR028C | 0.15269937 | -1.0741237 | 0.28277724 | 0.999976173 | -0.16401801 |
| YGR292W | MAL12 | 0.15269937 | -1.0727615 | 0.28338812 | 0.999976173 | -0.163810005 |

| | | | | | | |
|---|---|---|---|---|---|---|
| YDR156W | RPA14 | 0.15269937 | -1.0713649 | 0.28401535 | 0.999976173 | -0.163596747 |
| YCR050C | YCR050C | 0.15269937 | -1.0697416 | 0.28474561 | 0.999976173 | -0.163348861 |
| YGR037C | ACB1 | 0.15269937 | -1.0621383 | 0.28818279 | 0.999976173 | -0.162187851 |
| YAR050W | FLO1 | 0.15269937 | -1.0596357 | 0.28932026 | 0.999976173 | -0.161805693 |
| YDR007W | TRP1 | 0.15269937 | -1.0584112 | 0.28987787 | 0.999976173 | -0.161618724 |
| YHR004C | NEM1 | 0.15269937 | -1.0537059 | 0.29202743 | 0.999976173 | -0.160900218 |
| YMR126C | DLT1 | 0.15269937 | -1.0505595 | 0.29347075 | 0.999976173 | -0.160419768 |
| YOR371C | GPB1 | 0.15269937 | -1.0488858 | 0.29424047 | 0.999976173 | -0.160164194 |
| YLR065C | YLR065C | 0.15269937 | -1.046044 | 0.29555047 | 0.999976173 | -0.159730259 |
| YBR105C | VID24 | 0.15269937 | -1.0455794 | 0.29576504 | 0.999976173 | -0.159659305 |
| YDR354W | TRP4 | 0.15269937 | -1.0440525 | 0.29647083 | 0.999976173 | -0.159426157 |
| YLR324W | PEX30 | 0.15269937 | -1.0393918 | 0.29863226 | 0.999976173 | -0.158714466 |
| YOR113W | AZF1 | 0.15269937 | -1.0368865 | 0.29979844 | 0.999976173 | -0.158331908 |
| YKR084C | HBS1 | 0.15269937 | -1.0349249 | 0.30071364 | 0.999976173 | -0.158032379 |
| YEL013W | VAC8 | 0.15269937 | -1.0323728 | 0.30190713 | 0.999976173 | -0.157642677 |
| YML106W | URA5 | 0.15269937 | -1.027655 | 0.30412173 | 0.999976173 | -0.156922268 |
| YBR058C | UBP14 | 0.15269937 | -1.0274695 | 0.30420902 | 0.999976173 | -0.156893946 |
| YFL033C | RIM15 | 0.15269937 | -1.0247498 | 0.30549082 | 0.999976173 | -0.156478648 |
| YCL050C | APA1 | 0.15269937 | -1.0237879 | 0.30594503 | 0.999976173 | -0.156331763 |
| YNL268W | LYP1 | 0.15269937 | -1.0231549 | 0.30624417 | 0.999976173 | -0.156235102 |
| YGL126W | SCS3 | 0.15269937 | -1.0214879 | 0.3070329 | 0.999976173 | -0.155980547 |
| YER142C | MAG1 | 0.15269937 | -1.0213655 | 0.30709082 | 0.999976173 | -0.155961872 |
| YAL005C | SSA1 | 0.15269937 | -1.0203884 | 0.30755384 | 0.999976173 | -0.155812654 |
| YCR068W | ATG15 | 0.15269937 | -1.0155711 | 0.30984317 | 0.999976173 | -0.155077058 |
| YOL044W | PEX15 | 0.15269937 | -1.0095935 | 0.31269954 | 0.999976173 | -0.15416428 |
| YOL117W | RRI2 | 0.15269937 | -1.0077662 | 0.31357614 | 0.999976173 | -0.153885255 |
| YMR209C | YMR209C | 0.15269937 | -1.0073637 | 0.31376944 | 0.999976173 | -0.153823798 |
| YDR406W | PDR15 | 0.15269937 | -1.005521 | 0.31465542 | 0.999976173 | -0.153542424 |
| YMR244C-A | YMR244C-A | 0.15269937 | -1.0051979 | 0.31481095 | 0.999976173 | -0.153493083 |
| YNL255C | GIS2 | 0.15269937 | -1.003226 | 0.3157612 | 0.999976173 | -0.153191974 |
| YIL161W | YIL161W | 0.15269937 | -0.9995645 | 0.31753063 | 0.999976173 | -0.15263287 |
| YNL153C | GIM3 | 0.15269937 | -0.9965654 | 0.3189848 | 0.999976173 | -0.152174909 |
| YCR045C | RRT12 | 0.15269937 | -0.9932768 | 0.32058434 | 0.999976173 | -0.151672743 |
| YPL161C | BEM4 | 0.15269937 | -0.9918854 | 0.32126271 | 0.999976173 | -0.151460267 |
| YBR269C | FMP21 | 0.15269937 | -0.9918727 | 0.3212689 | 0.999976173 | -0.151458331 |
| YCR060W | TAH1 | 0.15269937 | -0.9914544 | 0.321473 | 0.999976173 | -0.15139446 |
| YGR125W | YGR125W | 0.15269937 | -0.9890147 | 0.32266518 | 0.999976173 | -0.151021918 |
| YMR175w | NA | 0.15269937 | -0.9884694 | 0.32293204 | 0.999976173 | -0.150938652 |
| YLR350W | ORM2 | 0.15269937 | -0.9883427 | 0.32299405 | 0.999976173 | -0.150919309 |
| YDR363W | ESC2 | 0.15269937 | -0.9870667 | 0.32361914 | 0.999976173 | -0.150724466 |
| YLR308W | CDA2 | 0.15269937 | -0.9853347 | 0.32446887 | 0.999976173 | -0.150459991 |
| YDR359C | EAF1 | 0.15269937 | -0.9850209 | 0.324623 | 0.999976173 | -0.150412068 |
| YAR043C | NA | 0.15269937 | -0.9834292 | 0.32540544 | 0.999976173 | -0.150169012 |
| YFR023W | PES4 | 0.15269937 | -0.9821663 | 0.32602708 | 0.999976173 | -0.149976177 |
| YPL027W | SMA1 | 0.15269937 | -0.9810595 | 0.32657255 | 0.999976173 | -0.149807168 |
| YDR541C | YDR541C | 0.15269937 | -0.9803943 | 0.32690068 | 0.999976173 | -0.149705585 |
| YHR106W | TRR2 | 0.15269937 | -0.9791245 | 0.32752761 | 0.999976173 | -0.149511691 |
| YBR148W | YSW1 | 0.15269937 | -0.9765389 | 0.32880662 | 0.999976173 | -0.149116863 |
| YGR163W | GTR2 | 0.15269937 | -0.9760734 | 0.32903722 | 0.999976173 | -0.149045785 |
| YJR148W | BAT2 | 0.15269937 | -0.9755225 | 0.32931025 | 0.999976173 | -0.14896167 |
| YHL030W | ECM29 | 0.15269937 | -0.9743035 | 0.32991495 | 0.999976173 | -0.148775532 |
| YFL013C | IES1 | 0.15269937 | -0.9725461 | 0.33078803 | 0.999976173 | -0.14850717 |
| YOR379C | YOR379C | 0.15269937 | -0.969976 | 0.33206753 | 0.999976173 | -0.148114713 |
| YGR188C | BUB1 | 0.15269937 | -0.9676692 | 0.33321865 | 0.999976173 | -0.147762469 |
| YKL053W | YKL053W | 0.15269937 | -0.9661434 | 0.33398146 | 0.999976173 | -0.14752948 |
| YBR121C | GRS1 | 0.15269937 | -0.962505 | 0.33580499 | 0.999976173 | -0.1469739 |
| YGL176C | YGL176C | 0.15269937 | -0.9583196 | 0.33791057 | 0.999976173 | -0.146334795 |
| YNL202W | SPS19 | 0.15269937 | -0.9545279 | 0.33982541 | 0.999976173 | -0.145755802 |
| YOR084W | LPX1 | 0.15269937 | -0.9530265 | 0.34058553 | 0.999976173 | -0.145526544 |
| YPL170W | DAP1 | 0.15269937 | -0.950713 | 0.34175897 | 0.999976173 | -0.145173267 |
| YDR386W | MUS81 | 0.15269937 | -0.9504825 | 0.341876 | 0.999976173 | -0.145138078 |
| YKL074C | MUD2 | 0.15269937 | -0.9500521 | 0.34209464 | 0.999976173 | -0.145072353 |
| YER019W | ISC1 | 0.15269937 | -0.9498327 | 0.34220614 | 0.999976173 | -0.145038848 |
| YJR116W | YJR116W | 0.15269937 | -0.948983 | 0.3426381 | 0.999976173 | -0.144909106 |

| | | | | | | |
|---|---|---|---|---|---|---|
| YKL200C | NA | 0.15269937 | -0.9485417 | 0.34286258 | 0.999976173 | -0.144841722 |
| YJL083W | TAX4 | 0.15269937 | -0.9462327 | 0.34403873 | 0.999976173 | -0.144489135 |
| YGL041C | YGL041C | 0.15269937 | -0.9430942 | 0.34564151 | 0.999976173 | -0.144009888 |
| YBR216C | YBP1 | 0.15269937 | -0.9398903 | 0.34728259 | 0.999976173 | -0.143520655 |
| YMR207C | HFA1 | 0.15269937 | -0.9387649 | 0.34786024 | 0.999976173 | -0.143348798 |
| YCL016C | DCC1 | 0.15269937 | -0.937728 | 0.34839296 | 0.999976173 | -0.143190467 |
| YER116C | SLX8 | 0.15269937 | -0.9373465 | 0.34858907 | 0.999976173 | -0.143132223 |
| YPL032C | SVL3 | 0.15269937 | -0.9370541 | 0.34873946 | 0.999976173 | -0.143087568 |
| YBR090C | YBR090C | 0.15269937 | -0.9365326 | 0.34900779 | 0.999976173 | -0.143007927 |
| YPR096C | YPR096C | 0.15269937 | -0.9358843 | 0.34934149 | 0.999976173 | -0.142908936 |
| YKR072C | SIS2 | 0.15269937 | -0.935882 | 0.34934266 | 0.999976173 | -0.14290859 |
| YGL243W | TAD1 | 0.15269937 | -0.935078 | 0.34975679 | 0.999976173 | -0.142785825 |
| YOR055W | YOR055W | 0.15269937 | -0.9343248 | 0.35014509 | 0.999976173 | -0.142670801 |
| YNL321W | VNX1 | 0.15269937 | -0.9337511 | 0.35044099 | 0.999976173 | -0.142583204 |
| YDR515W | SLF1 | 0.15269937 | -0.9333459 | 0.35065012 | 0.999976173 | -0.142521322 |
| YGR232W | NAS6 | 0.15269937 | -0.9295543 | 0.35261056 | 0.999976173 | -0.14194235 |
| YDR395W | SXM1 | 0.15269937 | -0.9185307 | 0.35834965 | 0.999976173 | -0.140259053 |
| YNL078W | NIS1 | 0.15269937 | -0.914936 | 0.36023374 | 0.999976173 | -0.139710147 |
| YGL115W | SNF4 | 0.15269937 | -0.9144273 | 0.36050086 | 0.999976173 | -0.139632472 |
| YCL001W-A | YCL001W-A | 0.15269937 | -0.9139167 | 0.3607691 | 0.999976173 | -0.139554507 |
| YKR007W | MEH1 | 0.15269937 | -0.9063963 | 0.36473456 | 0.999976173 | -0.13840614 |
| YEL049W | PAU2 | 0.15269937 | -0.9057265 | 0.36508903 | 0.999976173 | -0.138303869 |
| YIL123W | SIM1 | 0.15269937 | -0.9029838 | 0.36654288 | 0.999976173 | -0.137885056 |
| YMR031C | YMR031C | 0.15269937 | -0.902636 | 0.36672751 | 0.999976173 | -0.137831942 |
| YGR106C | VOA1 | 0.15269937 | -0.9003546 | 0.36793994 | 0.999976173 | -0.137483576 |
| YBL072C | RPS8A | 0.15269937 | -0.9002256 | 0.36800859 | 0.999976173 | -0.137463872 |
| YIL059C | YIL059C | 0.15269937 | -0.8986977 | 0.36882203 | 0.999976173 | -0.137230576 |
| YBL075C | SSA3 | 0.15269937 | -0.8963727 | 0.37006206 | 0.999976173 | -0.136875547 |
| YHR159W | YHR159W | 0.15269937 | -0.8949439 | 0.37082539 | 0.999976173 | -0.136657366 |
| YPR153W | YPR153W | 0.15269937 | -0.8948601 | 0.37087018 | 0.999976173 | -0.136644574 |
| YOR001W | RRP6 | 0.15269937 | -0.8944172 | 0.37110701 | 0.999976173 | -0.136576942 |
| YDL172C | YDL172C | 0.15269937 | -0.8916824 | 0.37257147 | 0.999976173 | -0.136159337 |
| YOR008C | SLG1 | 0.15269937 | -0.8893288 | 0.37383468 | 0.999976173 | -0.135799936 |
| YNL206C | RTT106 | 0.15269937 | -0.8885064 | 0.37427666 | 0.999976173 | -0.135674365 |
| YGR021W | YGR021W | 0.15269937 | -0.8879423 | 0.37458002 | 0.999976173 | -0.135588229 |
| YBR093C | PHO5 | 0.15269937 | -0.8875987 | 0.37476491 | 0.999976173 | -0.135535753 |
| YCR036W | RBK1 | 0.15269937 | -0.8855692 | 0.37585793 | 0.999976173 | -0.135225858 |
| YBR297W | MAL33 | 0.15269937 | -0.8834065 | 0.37702488 | 0.999976173 | -0.134895613 |
| YBR286W | APE3 | 0.15269937 | -0.8819087 | 0.37783435 | 0.999976173 | -0.134666903 |
| YLR182W | SWI6 | 0.15269937 | -0.881163 | 0.37823779 | 0.999976173 | -0.134553027 |
| YOR144C | ELG1 | 0.15269937 | -0.8806503 | 0.37851531 | 0.999976173 | -0.134474738 |
| YGR226C | YGR226C | 0.15269937 | -0.8799691 | 0.3788842 | 0.999976173 | -0.134370727 |
| YER140W | YER140W | 0.15269937 | -0.8797318 | 0.37901277 | 0.999976173 | -0.134334491 |
| YML032C | RAD52 | 0.15269937 | -0.8772002 | 0.38038603 | 0.999976173 | -0.133947918 |
| YOR059C | YOR059C | 0.15269937 | -0.8752383 | 0.38145237 | 0.999976173 | -0.133648334 |
| YJL028W | YJL028W | 0.15269937 | -0.8714837 | 0.38349815 | 0.999976173 | -0.133075015 |
| YHL020C | OPI1 | 0.15269937 | -0.8707672 | 0.38388933 | 0.999976173 | -0.132965603 |
| YNL117W | MLS1 | 0.15269937 | -0.8692616 | 0.3847121 | 0.999976173 | -0.1327357 |
| YML051W | GAL80 | 0.15269937 | -0.8692563 | 0.38471504 | 0.999976173 | -0.132734879 |
| YDR251W | PAM1 | 0.15269937 | -0.8683608 | 0.38520486 | 0.999976173 | -0.132598151 |
| YOL015W | IRC10 | 0.15269937 | -0.8666792 | 0.38612583 | 0.999976173 | -0.132341365 |
| YLR267W | BOP2 | 0.15269937 | -0.8637566 | 0.38772961 | 0.999976173 | -0.131895086 |
| YIL011W | TIR3 | 0.15269937 | -0.8631116 | 0.38808408 | 0.999976173 | -0.131796598 |
| YGL199C | YGL199C | 0.15269937 | -0.8627589 | 0.38827801 | 0.999976173 | -0.131742742 |
| YOR039W | CKB2 | 0.15269937 | -0.86271 | 0.38830493 | 0.999976173 | -0.131735267 |
| YHR045W | YHR045W | 0.15269937 | -0.8626267 | 0.38835074 | 0.999976173 | -0.131722549 |
| YBR071W | YBR071W | 0.15269937 | -0.8612753 | 0.38909441 | 0.999976173 | -0.13151619 |
| YHR021C | RPS27B | 0.15269937 | -0.8599567 | 0.38982085 | 0.999976173 | -0.131314848 |
| YGR242W | YGR242W | 0.15269937 | -0.8595516 | 0.39004422 | 0.999976173 | -0.131252985 |
| YOL016C | CMK2 | 0.15269937 | -0.8594635 | 0.39009282 | 0.999976173 | -0.131239526 |
| YML048W | GSF2 | 0.15269937 | -0.8583609 | 0.39070113 | 0.999976173 | -0.131071171 |
| YML107C | PML39 | 0.15269937 | -0.8583098 | 0.39072934 | 0.999976173 | -0.131063369 |
| YHR189W | PTH1 | 0.15269937 | -0.8575396 | 0.3911547 | 0.999976173 | -0.130945746 |
| YNL136W | EAF7 | 0.15269937 | -0.8566244 | 0.39166039 | 0.999976173 | -0.130806009 |

| | | | | | | |
|---|---|---|---|---|---|---|
| YDR399W | HPT1 | 0.15269937 | -0.8553502 | 0.39236523 | 0.999976173 | -0.130611428 |
| YEL061C | CIN8 | 0.15269937 | -0.8552746 | 0.39240703 | 0.999976173 | -0.130599896 |
| YCR025C | YCR025C | 0.15269937 | -0.8545971 | 0.39278216 | 0.999976173 | -0.130496428 |
| YJL148W | RPA34 | 0.15269937 | -0.8508214 | 0.39487646 | 0.999976173 | -0.129919883 |
| YMR258C | YMR258C | 0.15269937 | -0.8497324 | 0.39548175 | 0.999976173 | -0.129753593 |
| YMR099C | YMR099C | 0.15269937 | -0.8485627 | 0.39613253 | 0.999976173 | -0.129574981 |
| YKL037W | AIM26 | 0.15269937 | -0.8478147 | 0.39654901 | 0.999976173 | -0.129460766 |
| YER046W | SPO73 | 0.15269937 | -0.8470886 | 0.39695358 | 0.999976173 | -0.129349889 |
| YGR132C | PHB1 | 0.15269937 | -0.8456461 | 0.39775798 | 0.999976173 | -0.12912963 |
| YOL059W | GPD2 | 0.15269937 | -0.8445363 | 0.39837758 | 0.999976173 | -0.12896016 |
| YGR268C | HUA1 | 0.15269937 | -0.842981 | 0.39924684 | 0.999976173 | -0.128722668 |
| YMR135C | GID8 | 0.15269937 | -0.8425539 | 0.39948576 | 0.999976173 | -0.128657447 |
| YFL019C | YFL019C | 0.15269937 | -0.8413923 | 0.40013598 | 0.999976173 | -0.128480066 |
| YCL009C | ILV6 | 0.15269937 | -0.8381031 | 0.40198054 | 0.999976173 | -0.127977809 |
| YCR053W | THR4 | 0.15269937 | -0.837037 | 0.40257947 | 0.999976173 | -0.127815024 |
| YPR121W | THI22 | 0.15269937 | -0.834573 | 0.40396587 | 0.999976173 | -0.127438766 |
| YKL008C | LAC1 | 0.15269937 | -0.8326243 | 0.40506431 | 0.999976173 | -0.127141205 |
| YER045C | ACA1 | 0.15269937 | -0.8316178 | 0.4056324 | 0.999976173 | -0.126987505 |
| YDR057W | YOS9 | 0.15269937 | -0.8296183 | 0.40676228 | 0.999976173 | -0.126682185 |
| YKL123W | YKL123W | 0.15269937 | -0.8271293 | 0.40817142 | 0.999976173 | -0.126302112 |
| YMR304C-A | YMR304C-A | 0.15269937 | -0.8260436 | 0.40878697 | 0.999976173 | -0.126136332 |
| YOR112W | CEX1 | 0.15269937 | -0.824677 | 0.40956259 | 0.999976173 | -0.125927655 |
| YJL092W | SRS2 | 0.15269937 | -0.8242601 | 0.40979936 | 0.999976173 | -0.125863998 |
| YGL029W | CGR1 | 0.15269937 | -0.8232229 | 0.41038883 | 0.999976173 | -0.125705614 |
| YJR021C | REC107 | 0.15269937 | -0.8217523 | 0.41122545 | 0.999976173 | -0.125481056 |
| YGL215W | CLG1 | 0.15269937 | -0.8146896 | 0.41525748 | 0.999976173 | -0.12440259 |
| YNL098C | RAS2 | 0.15269937 | -0.8129113 | 0.41627639 | 0.999976173 | -0.124131038 |
| YOL126C | MDH2 | 0.15269937 | -0.8119219 | 0.41684391 | 0.999976173 | -0.123979959 |
| YBR270C | BIT2 | 0.15269937 | -0.8110549 | 0.41734159 | 0.999976173 | -0.123847571 |
| YJR080C | AIM24 | 0.15269937 | -0.809764 | 0.41808328 | 0.999976173 | -0.123650445 |
| YPR125W | YLH47 | 0.15269937 | -0.806132 | 0.42017416 | 0.999976173 | -0.123095841 |
| YKL098W | MTC2 | 0.15269937 | -0.8042992 | 0.42123157 | 0.999976173 | -0.122815979 |
| YNL234W | YNL234W | 0.15269937 | -0.8039591 | 0.42142796 | 0.999976173 | -0.122764047 |
| YNL227C | JJJ1 | 0.15269937 | -0.8021435 | 0.42247729 | 0.999976173 | -0.12248681 |
| YNR072W | HXT17 | 0.15269937 | -0.8017044 | 0.42273133 | 0.999976173 | -0.122419751 |
| YLR087C | CSF1 | 0.15269937 | -0.8001658 | 0.42362209 | 0.999976173 | -0.122184807 |
| YPR065W | ROX1 | 0.15269937 | -0.8001638 | 0.42362324 | 0.999976173 | -0.122184505 |
| YPL157W | TGS1 | 0.15269937 | -0.7978089 | 0.42498872 | 0.999976173 | -0.121824911 |
| YGL163C | RAD54 | 0.15269937 | -0.7972724 | 0.42530013 | 0.999976173 | -0.121742996 |
| YJR100C | AIM25 | 0.15269937 | -0.7946475 | 0.42682588 | 0.999976173 | -0.121342164 |
| YMR052W | FAR3 | 0.15269937 | -0.7920073 | 0.42836365 | 0.999976173 | -0.120939018 |
| YCL011C | GBP2 | 0.15269937 | -0.7903153 | 0.42935086 | 0.999976173 | -0.120680651 |
| YPR135W | CTF4 | 0.15269937 | -0.7896469 | 0.42974122 | 0.999976173 | -0.120578586 |
| YOR083W | WHI5 | 0.15269937 | -0.7889741 | 0.43013437 | 0.999976173 | -0.120475843 |
| YDR461W | MFA1 | 0.15269937 | -0.7889563 | 0.43014476 | 0.999976173 | -0.120473129 |
| YKL136W | YKL136W | 0.15269937 | -0.7859516 | 0.43190305 | 0.999976173 | -0.120014306 |
| YLR418C | CDC73 | 0.15269937 | -0.7850102 | 0.43245479 | 0.999976173 | -0.119870554 |
| YJL136C | RPS21B | 0.15269937 | -0.7830467 | 0.43360686 | 0.999976173 | -0.11957073 |
| YPR151C | SUE1 | 0.15269937 | -0.7826665 | 0.43383013 | 0.999976173 | -0.119512678 |
| YJL048C | UBX6 | 0.15269937 | -0.7822568 | 0.43407084 | 0.999976173 | -0.119450112 |
| YLR334C | YLR334C | 0.15269937 | -0.7819132 | 0.43427271 | 0.999976173 | -0.119397655 |
| YGR208W | SER2 | 0.15269937 | -0.7810008 | 0.43480915 | 0.999976173 | -0.11925833 |
| YBR001C | NTH2 | 0.15269937 | -0.7784034 | 0.43633832 | 0.999976173 | -0.118861713 |
| YGL045W | RIM8 | 0.15269937 | -0.7779711 | 0.43659316 | 0.999976173 | -0.118795694 |
| YPL272C | YPL272C | 0.15269937 | -0.7768493 | 0.43725477 | 0.999976173 | -0.118624398 |
| YER032W | FIR1 | 0.15269937 | -0.7759591 | 0.4377802 | 0.999976173 | -0.118488467 |
| YJR079W | YJR079W | 0.15269937 | -0.775822 | 0.43786115 | 0.999976173 | -0.118467534 |
| YCL032W | STE50 | 0.15269937 | -0.7755073 | 0.43804706 | 0.999976173 | -0.118419466 |
| YKR092C | SRP40 | 0.15269937 | -0.7749133 | 0.43839799 | 0.999976173 | -0.118328762 |
| YMR147W | YMR147W | 0.15269937 | -0.7744803 | 0.43865391 | 0.999976173 | -0.118262644 |
| YJL105W | SET4 | 0.15269937 | -0.7741668 | 0.43883924 | 0.999976173 | -0.118214774 |
| YNL224C | SQS1 | 0.15269937 | -0.771438 | 0.44045438 | 0.999976173 | -0.117798097 |
| YAL004W | YAL004W | 0.15269937 | -0.7708174 | 0.44082221 | 0.999976173 | -0.117703326 |
| YGR154C | GTO1 | 0.15269937 | -0.7704903 | 0.44101612 | 0.999976173 | -0.117653384 |

| | | | | | | |
|---|---|---|---|---|---|---|
| YOR365C | YOR365C | 0.15269937 | -0.7701131 | 0.44123984 | 0.999976173 | -0.117595778 |
| YKL207W | AIM27 | 0.15269937 | -0.7684986 | 0.44219803 | 0.999976173 | -0.117349248 |
| YKL140W | TGL1 | 0.15269937 | -0.7684474 | 0.44222843 | 0.999976173 | -0.117341432 |
| YIL043C | CBR1 | 0.15269937 | -0.7670128 | 0.44308089 | 0.999976173 | -0.117122369 |
| YKR048C | NAP1 | 0.15269937 | -0.7662081 | 0.44355947 | 0.999976173 | -0.116999492 |
| YHR191C | CTF8 | 0.15269937 | -0.7641725 | 0.44477142 | 0.999976173 | -0.116688654 |
| YPR030W | CSR2 | 0.15269937 | -0.7629639 | 0.44549188 | 0.999976173 | -0.116504101 |
| YDR155C | CPR1 | 0.15269937 | -0.7585173 | 0.44814824 | 0.999976173 | -0.115825116 |
| YNL323W | LEM3 | 0.15269937 | -0.7582238 | 0.44832389 | 0.999976173 | -0.1157803 |
| YGL078C | DBP3 | 0.15269937 | -0.7581912 | 0.44834343 | 0.999976173 | -0.115775315 |
| YJL088W | ARG3 | 0.15269937 | -0.7549021 | 0.45031461 | 0.999976173 | -0.115273066 |
| YMR316C-A | YMR316C-A | 0.15269937 | -0.754533 | 0.45053607 | 0.999976173 | -0.115216717 |
| YNL052W | COX5A | 0.15269937 | -0.753673 | 0.45105247 | 0.999976173 | -0.115085385 |
| YJR118C | ILM1 | 0.15269937 | -0.7534787 | 0.45116913 | 0.999976173 | -0.115055725 |
| YJL159W | HSP150 | 0.15269937 | -0.7532661 | 0.45129684 | 0.999976173 | -0.115023262 |
| YMR283C | RIT1 | 0.15269937 | -0.7526623 | 0.45165969 | 0.999976173 | -0.114931062 |
| YLR234W | TOP3 | 0.15269937 | -0.7511461 | 0.45257154 | 0.999976173 | -0.114699538 |
| YJR097W | JJJ3 | 0.15269937 | -0.7500128 | 0.45325382 | 0.999976173 | -0.114526475 |
| YEL059W | YEL059W | 0.15269937 | -0.7490847 | 0.45381295 | 0.999976173 | -0.114384761 |
| YGR025W | YGR025W | 0.15269937 | -0.748861 | 0.45394781 | 0.999976173 | -0.114350595 |
| YNL339C | YRF1-6 | 0.15269937 | -0.7483206 | 0.45427358 | 0.999976173 | -0.114268086 |
| YHL003C | LAG1 | 0.15269937 | -0.7479853 | 0.45447581 | 0.999976173 | -0.114216881 |
| YGL152C | YGL152C | 0.15269937 | -0.7478387 | 0.45456421 | 0.999976173 | -0.114194503 |
| YEL031W | SPF1 | 0.15269937 | -0.7477969 | 0.45458949 | 0.999976173 | -0.114188106 |
| YFR048W | RMD8 | 0.15269937 | -0.7474146 | 0.45482015 | 0.999976173 | -0.114129728 |
| YGR007W | MUQ1 | 0.15269937 | -0.7464818 | 0.45538321 | 0.999976173 | -0.113987294 |
| YNL056W | OCA2 | 0.15269937 | -0.7452721 | 0.45611403 | 0.999976173 | -0.113802572 |
| YGR043C | NQM1 | 0.15269937 | -0.7417528 | 0.45824384 | 0.999976173 | -0.113265184 |
| YFL054C | YFL054C | 0.15269937 | -0.741662 | 0.45829886 | 0.999976173 | -0.11325132 |
| YER019C-A | SBH2 | 0.15269937 | -0.7416087 | 0.45833117 | 0.999976173 | -0.113243179 |
| YGL058W | RAD6 | 0.15269937 | -0.7412428 | 0.45855296 | 0.999976173 | -0.113187304 |
| YMR303C | ADH2 | 0.15269937 | -0.7393559 | 0.45969759 | 0.999976173 | -0.112899184 |
| YBR099C | YBR099C | 0.15269937 | -0.7392649 | 0.45975287 | 0.999976173 | -0.112885279 |
| YIL107C | PFK26 | 0.15269937 | -0.7380168 | 0.46051091 | 0.999976173 | -0.112694701 |
| YBL052C | SAS3 | 0.15269937 | -0.7373177 | 0.46093584 | 0.999976173 | -0.112587947 |
| YKL073W | LHS1 | 0.15269937 | -0.7371038 | 0.46106593 | 0.999976173 | -0.112555277 |
| YLR236C | YLR236C | 0.15269937 | -0.7363503 | 0.46152419 | 0.999976173 | -0.112440229 |
| YGR270W | YTA7 | 0.15269937 | -0.7362486 | 0.46158611 | 0.999976173 | -0.112424688 |
| YBR104W | YMC2 | 0.15269937 | -0.7361585 | 0.4616409 | 0.999976173 | -0.112410938 |
| YGL046W | NA | 0.15269937 | -0.7342644 | 0.46279427 | 0.999976173 | -0.112121705 |
| YOR031W | CRS5 | 0.15269937 | -0.7336088 | 0.46319381 | 0.999976173 | -0.112021604 |
| YOR164C | GET4 | 0.15269937 | -0.7329674 | 0.46358496 | 0.999976173 | -0.111923653 |
| YDR149C | YDR149C | 0.15269937 | -0.7329143 | 0.46361731 | 0.999976173 | -0.111915555 |
| YHR017W | YSC83 | 0.15269937 | -0.7316101 | 0.46441321 | 0.999976173 | -0.111716395 |
| YGR027C | RPS25A | 0.15269937 | -0.7311851 | 0.4646727 | 0.999976173 | -0.111651505 |
| YLL049W | LDB18 | 0.15269937 | -0.7295786 | 0.46565439 | 0.999976173 | -0.111406193 |
| YNL080C | EOS1 | 0.15269937 | -0.7295555 | 0.4656685 | 0.999976173 | -0.11140267 |
| YDR438W | THI74 | 0.15269937 | -0.7283361 | 0.46641444 | 0.999976173 | -0.111216465 |
| YNL183C | NPR1 | 0.15269937 | -0.7260189 | 0.46783371 | 0.999976173 | -0.110862633 |
| YOR193W | PEX27 | 0.15269937 | -0.7241271 | 0.46899424 | 0.999976173 | -0.11057375 |
| YNL307C | MCK1 | 0.15269937 | -0.7240159 | 0.46906253 | 0.999976173 | -0.110556765 |
| YDR439W | LRS4 | 0.15269937 | -0.7231809 | 0.46957527 | 0.999976173 | -0.110429267 |
| YHR151C | MTC6 | 0.15269937 | -0.7213201 | 0.47071911 | 0.999976173 | -0.11014512 |
| YER135C | YER135C | 0.15269937 | -0.7198884 | 0.47160019 | 0.999976173 | -0.109926505 |
| YBR103W | SIF2 | 0.15269937 | -0.7180737 | 0.47271831 | 0.999976173 | -0.109649401 |
| YLR049C | YLR049C | 0.15269937 | -0.7164595 | 0.47371412 | 0.999976173 | -0.109402912 |
| YOL086C | ADH1 | 0.15269937 | -0.7158608 | 0.47408374 | 0.999976173 | -0.109311494 |
| YAL018C | YAL018C | 0.15269937 | -0.7156314 | 0.47422546 | 0.999976173 | -0.109276454 |
| YDR369C | 32 | 0.15269937 | -0.7155625 | 0.47426801 | 0.999976173 | -0.109265933 |
| YCR085W | YCR085W | 0.15269937 | -0.7149493 | 0.47464679 | 0.999976173 | -0.10917231 |
| YNR048W | YNR048W | 0.15269937 | -0.7089708 | 0.47834899 | 0.999976173 | -0.108259388 |
| YGL084C | GUP1 | 0.15269937 | -0.7076687 | 0.47915736 | 0.999976173 | -0.108060567 |
| YDR297W | SUR2 | 0.15269937 | -0.7063992 | 0.47994629 | 0.999976173 | -0.107866705 |
| YML103C | NUP188 | 0.15269937 | -0.7019922 | 0.48269034 | 0.999976173 | -0.107193763 |

| | | | | | | |
|---|---|---|---|---|---|---|
| YKL010C | UFD4 | 0.15269937 | -0.7013025 | 0.48312055 | 0.999976173 | -0.107088448 |
| YBR215W | HPC2 | 0.15269937 | -0.7012347 | 0.48316286 | 0.999976173 | -0.107078093 |
| YOL056W | GPM3 | 0.15269937 | -0.6995852 | 0.48419264 | 0.999976173 | -0.106826223 |
| YBL015W | ACH1 | 0.15269937 | -0.6985938 | 0.48481217 | 0.999976173 | -0.106674835 |
| YIL045W | PIG2 | 0.15269937 | -0.6983524 | 0.48496308 | 0.999976173 | -0.106637974 |
| YNL199C | GCR2 | 0.15269937 | -0.697392 | 0.48556376 | 0.999976173 | -0.106491318 |
| YNL127W | FAR11 | 0.15269937 | -0.6969088 | 0.48586612 | 0.999976173 | -0.106417533 |
| YPL024W | RMI1 | 0.15269937 | -0.6956783 | 0.48663654 | 0.999976173 | -0.106229639 |
| YNL235C | YNL235C | 0.15269937 | -0.695611 | 0.48667872 | 0.999976173 | -0.106219358 |
| YNL332W | THI12 | 0.15269937 | -0.6947838 | 0.48719701 | 0.999976173 | -0.106093051 |
| YAL036C | RBG1 | 0.15269937 | -0.6942295 | 0.48754449 | 0.999976173 | -0.106008412 |
| YDR146C | SWI5 | 0.15269937 | -0.6921008 | 0.48888025 | 0.999976173 | -0.105683348 |
| YOR184W | SER1 | 0.15269937 | -0.691669 | 0.48915142 | 0.999976173 | -0.105617416 |
| YNR063W | YNR063W | 0.15269937 | -0.6906287 | 0.48980511 | 0.999976173 | -0.105458562 |
| YNL284C | MRPL10 | 0.15269937 | -0.6897119 | 0.49038156 | 0.999976173 | -0.105318569 |
| YKL077W | YKL077W | 0.15269937 | -0.6891554 | 0.49073167 | 0.999976173 | -0.105233589 |
| YJL201W | ECM25 | 0.15269937 | -0.6874218 | 0.49182309 | 0.999976173 | -0.10496888 |
| YIL035C | CKA1 | 0.15269937 | -0.6872778 | 0.49191382 | 0.999976173 | -0.104946888 |
| YBR195C | MSI1 | 0.15269937 | -0.6848825 | 0.49342418 | 0.999976173 | -0.104581129 |
| YJL095W | BCK1 | 0.15269937 | -0.6846918 | 0.49354457 | 0.999976173 | -0.104551999 |
| YOL080C | REX4 | 0.15269937 | -0.6845326 | 0.49364503 | 0.999976173 | -0.104527696 |
| YGL131C | SNT2 | 0.15269937 | -0.6820436 | 0.4952175 | 0.999976173 | -0.104147619 |
| YJR075W | HOC1 | 0.15269937 | -0.6814178 | 0.49561324 | 0.999976173 | -0.10405207 |
| YNL233W | BNI4 | 0.15269937 | -0.6802604 | 0.49634566 | 0.999976173 | -0.103875335 |
| YML011C | RAD33 | 0.15269937 | -0.6796871 | 0.49670865 | 0.999976173 | -0.103787795 |
| YNL230C | ELA1 | 0.15269937 | -0.6762594 | 0.49888201 | 0.999976173 | -0.103264379 |
| YIL089W | YIL089W | 0.15269937 | -0.6756025 | 0.4992991 | 0.999976173 | -0.103164071 |
| YNL319W | YNL319W | 0.15269937 | -0.6749668 | 0.4997029 | 0.999976173 | -0.103066998 |
| YBR014C | GRX7 | 0.15269937 | -0.6743797 | 0.50007597 | 0.999976173 | -0.102977351 |
| YNL146W | YNL146W | 0.15269937 | -0.6731445 | 0.50086136 | 0.999976173 | -0.102788741 |
| YNL162W | RPL42A | 0.15269937 | -0.6706311 | 0.50246154 | 0.999976173 | -0.102404945 |
| YGR105W | VMA21 | 0.15269937 | -0.6692127 | 0.5033658 | 0.999976173 | -0.102188349 |
| YKL166C | TPK3 | 0.15269937 | -0.6690898 | 0.50344416 | 0.999976173 | -0.102169587 |
| YCL010C | SGF29 | 0.15269937 | -0.667772 | 0.50428509 | 0.999976173 | -0.101968362 |
| YMR088C | VBA1 | 0.15269937 | -0.6673597 | 0.50454837 | 0.999976173 | -0.101905397 |
| YIL149C | MLP2 | 0.15269937 | -0.6673234 | 0.5045715 | 0.999976173 | -0.101899866 |
| YCR026C | NPP1 | 0.15269937 | -0.6666501 | 0.50500159 | 0.999976173 | -0.101797049 |
| YCL025C | AGP1 | 0.15269937 | -0.6661392 | 0.50532805 | 0.999976173 | -0.101719036 |
| YKL034W | TUL1 | 0.15269937 | -0.6645784 | 0.5063261 | 0.999976173 | -0.1014807 |
| YGL263W | COS12 | 0.15269937 | -0.6644868 | 0.50638473 | 0.999976173 | -0.101466708 |
| YNL129W | NRK1 | 0.15269937 | -0.6641501 | 0.50660016 | 0.999976173 | -0.1014153 |
| YGR010W | NMA2 | 0.15269937 | -0.663655 | 0.50691707 | 0.999976173 | -0.101339694 |
| YGR071C | YGR071C | 0.15269937 | -0.663519 | 0.50700413 | 0.999976173 | -0.101318928 |
| YJR154W | YJR154W | 0.15269937 | -0.663467 | 0.50703743 | 0.999976173 | -0.101310985 |
| YNL016W | PUB1 | 0.15269937 | -0.6618282 | 0.50808725 | 0.999976173 | -0.10106074 |
| YOR008C-A | YOR008C-A | 0.15269937 | -0.6607749 | 0.50876258 | 0.999976173 | -0.100899902 |
| YPL189W | GUP2 | 0.15269937 | -0.6586349 | 0.51013611 | 0.999976173 | -0.100573129 |
| YML120C | NDI1 | 0.15269937 | -0.6581659 | 0.51043737 | 0.999976173 | -0.100501518 |
| YDR128W | MTC5 | 0.15269937 | -0.6581211 | 0.51046617 | 0.999976173 | -0.100494674 |
| YOL087C | YOL087C | 0.15269937 | -0.6577462 | 0.51070709 | 0.999976173 | -0.100437423 |
| YKL027W | YKL027W | 0.15269937 | -0.6564528 | 0.51153868 | 0.999976173 | -0.10023992 |
| YBR284W | YBR284W | 0.15269937 | -0.6560993 | 0.51176606 | 0.999976173 | -0.100185947 |
| YLR266C | PDR8 | 0.15269937 | -0.6540347 | 0.51309528 | 0.999976173 | -0.099870678 |
| YAL031C | GIP4 | 0.15269937 | -0.6505046 | 0.51537213 | 0.999976173 | -0.099331632 |
| YAL064C-A | YAL064C-A | 0.15269937 | -0.6503996 | 0.51543992 | 0.999976173 | -0.099315603 |
| YER120W | SCS2 | 0.15269937 | -0.6502487 | 0.51553738 | 0.999976173 | -0.099292558 |
| YKL064W | MNR2 | 0.15269937 | -0.6496447 | 0.51592751 | 0.999976173 | -0.099200334 |
| YFL006W | NA | 0.15269937 | -0.6492409 | 0.51618844 | 0.999976173 | -0.099138672 |
| YJR103W | URA8 | 0.15269937 | -0.6483445 | 0.51676793 | 0.999976173 | -0.099001789 |
| YNL330C | RPD3 | 0.15269937 | -0.6479835 | 0.51700135 | 0.999976173 | -0.098946673 |
| YBR086C | IST2 | 0.15269937 | -0.6474312 | 0.51735862 | 0.999976173 | -0.098862341 |
| YBL029W | YBL029W | 0.15269937 | -0.6471406 | 0.51754669 | 0.999976173 | -0.098817959 |
| YBL094C | YBL094C | 0.15269937 | -0.6461695 | 0.51817532 | 0.999976173 | -0.098669672 |
| YEL011W | GLC3 | 0.15269937 | -0.6457245 | 0.51846349 | 0.999976173 | -0.098601727 |

| | | | | | | |
|---|---|---|---|---|---|---|
| YNL021W | HDA1 | 0.15269937 | -0.645503 | 0.51860697 | 0.999976173 | -0.098567904 |
| YIL054W | YIL054W | 0.15269937 | -0.642429 | 0.52060033 | 0.999976173 | -0.098098507 |
| YDR157W | YDR157W | 0.15269937 | -0.6415829 | 0.52114972 | 0.999976173 | -0.097969301 |
| YIR002C | MPH1 | 0.15269937 | -0.6414762 | 0.521219 | 0.999976173 | -0.097953012 |
| YDL002C | NHP10 | 0.15269937 | -0.6407022 | 0.52172186 | 0.999976173 | -0.097834818 |
| YAL054C | ACS1 | 0.15269937 | -0.6396361 | 0.52241486 | 0.999976173 | -0.097672028 |
| YNL187W | SWT21 | 0.15269937 | -0.6395188 | 0.52249116 | 0.999976173 | -0.097654111 |
| YER091C-A | YER091C-A | 0.15269937 | -0.6394498 | 0.522536 | 0.999976173 | -0.097643583 |
| YML019W | OST6 | 0.15269937 | -0.6391084 | 0.52275807 | 0.999976173 | -0.097591446 |
| YGR078C | PAC10 | 0.15269937 | -0.6379406 | 0.52351799 | 0.999976173 | -0.097413123 |
| YOR221C | MCT1 | 0.15269937 | -0.6378743 | 0.52356116 | 0.999976173 | -0.097402998 |
| YER083C | GET2 | 0.15269937 | -0.6371007 | 0.52406487 | 0.999976173 | -0.097284875 |
| YGL222C | EDC1 | 0.15269937 | -0.6362524 | 0.52461755 | 0.999976173 | -0.097155336 |
| YDL218W | YDL218W | 0.15269937 | -0.6361112 | 0.52470955 | 0.999976173 | -0.097133781 |
| YOL055C | THI20 | 0.15269937 | -0.6353098 | 0.52523201 | 0.999976173 | -0.0970114 |
| YML022W | APT1 | 0.15269937 | -0.6349595 | 0.52546042 | 0.999976173 | -0.096957916 |
| YDR530C | APA2 | 0.15269937 | -0.6345527 | 0.52572577 | 0.999976173 | -0.096895799 |
| YMR259C | YMR259C | 0.15269937 | -0.6319889 | 0.52739971 | 0.999976173 | -0.096504305 |
| YPL158C | AIM44 | 0.15269937 | -0.6308821 | 0.52812317 | 0.999976173 | -0.096335298 |
| YGR210C | YGR210C | 0.15269937 | -0.6297891 | 0.52883811 | 0.999976173 | -0.0961684 |
| YBR074W | YBR074W | 0.15269937 | -0.6297187 | 0.52888421 | 0.999976173 | -0.096157644 |
| YGL090W | LIF1 | 0.15269937 | -0.6297092 | 0.52889042 | 0.999976173 | -0.096156195 |
| YIL156W | UBP7 | 0.15269937 | -0.6296547 | 0.52892611 | 0.999976173 | -0.096147867 |
| YDR374C | YDR374C | 0.15269937 | -0.6295594 | 0.52898845 | 0.999976173 | -0.09613332 |
| YOR026W | BUB3 | 0.15269937 | -0.628455 | 0.52971149 | 0.999976173 | -0.095964673 |
| YAR018C | KIN3 | 0.15269937 | -0.6278751 | 0.53009129 | 0.999976173 | -0.095876132 |
| YOR086C | TCB1 | 0.15269937 | -0.6272644 | 0.53049148 | 0.999976173 | -0.095782872 |
| YOR027W | STI1 | 0.15269937 | -0.6271766 | 0.53054904 | 0.999976173 | -0.095769462 |
| YLR062C | BUD28 | 0.15269937 | -0.6269519 | 0.53069627 | 0.999976173 | -0.095735162 |
| YHR181W | SVP26 | 0.15269937 | -0.6266309 | 0.53090674 | 0.999976173 | -0.095686139 |
| YJR111C | YJR111C | 0.15269937 | -0.626428 | 0.53103977 | 0.999976173 | -0.095655158 |
| YLR084C | RAX2 | 0.15269937 | -0.6257752 | 0.53146794 | 0.999976173 | -0.09555547 |
| YOL159C | YOL159C | 0.15269937 | -0.6256123 | 0.53157478 | 0.999976173 | -0.095530601 |
| YHR092C | HXT4 | 0.15269937 | -0.6248719 | 0.53206062 | 0.999976173 | -0.095417547 |
| YJR070C | LIA1 | 0.15269937 | -0.6243617 | 0.53239559 | 0.999976173 | -0.095339631 |
| YHL039W | YHL039W | 0.15269937 | -0.622771 | 0.53344049 | 0.999976173 | -0.09509674 |
| YHR044C | DOG1 | 0.15269937 | -0.6219475 | 0.53398189 | 0.999976173 | -0.094970982 |
| YCR095C | OCA4 | 0.15269937 | -0.6216818 | 0.53415662 | 0.999976173 | -0.09493041 |
| YOR155C | ISN1 | 0.15269937 | -0.6214827 | 0.53428752 | 0.999976173 | -0.09490002 |
| YDR148C | KGD2 | 0.15269937 | -0.6208665 | 0.53469292 | 0.999976173 | -0.094805922 |
| YDL200C | MGT1 | 0.15269937 | -0.6206374 | 0.53484367 | 0.999976173 | -0.094770941 |
| YMR294W | JNM1 | 0.15269937 | -0.6204434 | 0.53497139 | 0.999976173 | -0.094741307 |
| YGL262W | YGL262W | 0.15269937 | -0.6202962 | 0.53506827 | 0.999976173 | -0.094718833 |
| YGR058W | PEF1 | 0.15269937 | -0.6191569 | 0.53581842 | 0.999976173 | -0.094544872 |
| YNL071W | LAT1 | 0.15269937 | -0.618061 | 0.53654058 | 0.999976173 | -0.094377519 |
| YER030W | CHZ1 | 0.15269937 | -0.6177957 | 0.53671542 | 0.999976173 | -0.094337018 |
| YER080W | AIM9 | 0.15269937 | -0.6175323 | 0.5368891 | 0.999976173 | -0.094296794 |
| YER028C | MIG3 | 0.15269937 | -0.6165274 | 0.53755192 | 0.999976173 | -0.094143341 |
| YKR026C | GCN3 | 0.15269937 | -0.6161909 | 0.53777392 | 0.999976173 | -0.094091966 |
| YHR029C | YHI9 | 0.15269937 | -0.6157337 | 0.53807569 | 0.999976173 | -0.094022147 |
| YDR207C | UME6 | 0.15269937 | -0.6135423 | 0.53952324 | 0.999976173 | -0.093687513 |
| YKR080W | MTD1 | 0.15269937 | -0.6129966 | 0.53988394 | 0.999976173 | -0.093604199 |
| YER178W | PDA1 | 0.15269937 | -0.6129587 | 0.53990901 | 0.999976173 | -0.093598409 |
| YHR155W | YSP1 | 0.15269937 | -0.6127143 | 0.54007062 | 0.999976173 | -0.093561089 |
| YHR135C | YCK1 | 0.15269937 | -0.612208 | 0.54040555 | 0.999976173 | -0.093483767 |
| YGL110C | CUE3 | 0.15269937 | -0.611817 | 0.54066421 | 0.999976173 | -0.093424067 |
| YER048C | CAJ1 | 0.15269937 | -0.6108858 | 0.54128053 | 0.999976173 | -0.093281878 |
| YJL112W | MDV1 | 0.15269937 | -0.6104885 | 0.54154363 | 0.999976173 | -0.093221204 |
| YBR090C-A | NA | 0.15269937 | -0.610333 | 0.54164658 | 0.999976173 | -0.093197467 |
| YDR133C | YDR133C | 0.15269937 | -0.6085138 | 0.54285212 | 0.999976173 | -0.092919665 |
| YNL274C | GOR1 | 0.15269937 | -0.6078966 | 0.54326139 | 0.999976173 | -0.092825425 |
| YPL239W | YAR1 | 0.15269937 | -0.6062763 | 0.5443366 | 0.999976173 | -0.092578008 |
| YGR109C | CLB6 | 0.15269937 | -0.6055387 | 0.54482641 | 0.999976173 | -0.092465378 |
| YGL231C | EMC4 | 0.15269937 | -0.6041739 | 0.54573335 | 0.999976173 | -0.092256965 |

| | | | | | | |
|---|---|---|---|---|---|---|
| YIL034C | CAP2 | 0.15269937 | -0.6038288 | 0.54596276 | 0.999976173 | -0.092204273 |
| YMR036C | MIH1 | 0.15269937 | -0.6034252 | 0.54623111 | 0.999976173 | -0.092142653 |
| YMR122C | YMR122C | 0.15269937 | -0.6030177 | 0.54650218 | 0.999976173 | -0.092080423 |
| YMR006C | PLB2 | 0.15269937 | -0.6014555 | 0.5475419 | 0.999976173 | -0.091841875 |
| YKL001C | MET14 | 0.15269937 | -0.6009097 | 0.54790542 | 0.999976173 | -0.091758524 |
| YOR081C | TGL5 | 0.15269937 | -0.6001831 | 0.54838947 | 0.999976173 | -0.091647577 |
| YER069W | ARG5,6 | 0.15269937 | -0.5997992 | 0.54864534 | 0.999976173 | -0.09158895 |
| YJL047C | RTT101 | 0.15269937 | -0.5990883 | 0.54911925 | 0.999976173 | -0.091480401 |
| YGR004W | PEX31 | 0.15269937 | -0.597944 | 0.54988251 | 0.999976173 | -0.091305674 |
| YBR026C | ETR1 | 0.15269937 | -0.59761 | 0.55010541 | 0.999976173 | -0.091254668 |
| YPR141C | KAR3 | 0.15269937 | -0.5969501 | 0.5505459 | 0.999976173 | -0.091153904 |
| YML034W | SRC1 | 0.15269937 | -0.5961459 | 0.55108298 | 0.999976173 | -0.091031099 |
| YBL082C | ALG3 | 0.15269937 | -0.5959836 | 0.55119136 | 0.999976173 | -0.091006324 |
| YBL079W | NUP170 | 0.15269937 | -0.5952233 | 0.5516994 | 0.999976173 | -0.090890224 |
| YLR329W | REC102 | 0.15269937 | -0.594953 | 0.55188007 | 0.999976173 | -0.09084895 |
| YPR119W | CLB2 | 0.15269937 | -0.5949031 | 0.55191344 | 0.999976173 | -0.090841325 |
| YGL227W | VID30 | 0.15269937 | -0.5942411 | 0.55235604 | 0.999976173 | -0.090740242 |
| YMR078C | CTF18 | 0.15269937 | -0.5939784 | 0.55253177 | 0.999976173 | -0.090700121 |
| YGR144W | THI4 | 0.15269937 | -0.592783 | 0.55333159 | 0.999976173 | -0.090517582 |
| YML028W | TSA1 | 0.15269937 | -0.5923914 | 0.5535937 | 0.999976173 | -0.090457789 |
| YDR514C | YDR514C | 0.15269937 | -0.5922797 | 0.55366847 | 0.999976173 | -0.090440735 |
| YML004C | GLO1 | 0.15269937 | -0.5917068 | 0.5540521 | 0.999976173 | -0.090353254 |
| YBR098W | MMS4 | 0.15269937 | -0.589014 | 0.555857 | 0.999976173 | -0.089942067 |
| YNL218W | MGS1 | 0.15269937 | -0.5878851 | 0.55661453 | 0.999976173 | -0.089769684 |
| YHR041C | SRB2 | 0.15269937 | -0.5875561 | 0.55683537 | 0.999976173 | -0.089719451 |
| YGR168C | YGR168C | 0.15269937 | -0.5866695 | 0.55743078 | 0.999976173 | -0.089584067 |
| YFL004W | VTC2 | 0.15269937 | -0.5852082 | 0.55841281 | 0.999976173 | -0.089360924 |
| YAL056W | GPB2 | 0.15269937 | -0.5841888 | 0.55909839 | 0.999976173 | -0.089205258 |
| YNL203C | YNL203C | 0.15269937 | -0.582912 | 0.55995762 | 0.999976173 | -0.089010293 |
| YMR225C | MRPL44 | 0.15269937 | -0.5813381 | 0.56101764 | 0.999976173 | -0.088769965 |
| YNL140C | YNL140C | 0.15269937 | -0.58005 | 0.56188593 | 0.999976173 | -0.08857327 |
| YNL001W | DOM34 | 0.15269937 | -0.5777825 | 0.56341601 | 0.999976173 | -0.088227018 |
| YKL055C | OAR1 | 0.15269937 | -0.5775362 | 0.56358233 | 0.999976173 | -0.088189408 |
| YIL014W | MNT3 | 0.15269937 | -0.5774895 | 0.56361384 | 0.999976173 | -0.088182281 |
| YPR071W | YPR071W | 0.15269937 | -0.5769184 | 0.5639996 | 0.999976173 | -0.088095073 |
| YDR067C | OCA6 | 0.15269937 | -0.5768225 | 0.56406437 | 0.999976173 | -0.088080433 |
| YFL051C | YFL051C | 0.15269937 | -0.5752377 | 0.56513554 | 0.999976173 | -0.087838432 |
| YDR161W | YDR161W | 0.15269937 | -0.574747 | 0.56546738 | 0.999976173 | -0.087763509 |
| YPL092W | SSU1 | 0.15269937 | -0.5743406 | 0.56574231 | 0.999976173 | -0.087701449 |
| YGL257C | MNT2 | 0.15269937 | -0.5741081 | 0.56589964 | 0.999976173 | -0.087665943 |
| YDR191W | HST4 | 0.15269937 | -0.5737407 | 0.56614828 | 0.999976173 | -0.087609838 |
| YJR146W | YJR146W | 0.15269937 | -0.5722462 | 0.56716018 | 0.999976173 | -0.087381627 |
| YJR099W | YUH1 | 0.15269937 | -0.570971 | 0.56802423 | 0.999976173 | -0.087186916 |
| YBR183W | YPC1 | 0.15269937 | -0.5702401 | 0.56851981 | 0.999976173 | -0.087075301 |
| YNL298W | CLA4 | 0.15269937 | -0.5697296 | 0.56886605 | 0.999976173 | -0.086997348 |
| YPL108W | YPL108W | 0.15269937 | -0.5692303 | 0.56920483 | 0.999976173 | -0.086921099 |
| YOL111C | MDY2 | 0.15269937 | -0.568297 | 0.56983825 | 0.999976173 | -0.08677859 |
| YPL226W | NEW1 | 0.15269937 | -0.5676553 | 0.57027395 | 0.999976173 | -0.086680609 |
| YLR391W | NA | 0.15269937 | -0.5672614 | 0.57054148 | 0.999976173 | -0.086620464 |
| YBL013W | FMT1 | 0.15269937 | -0.5672051 | 0.57057975 | 0.999976173 | -0.086611861 |
| YMR180C | CTL1 | 0.15269937 | -0.5663885 | 0.57113461 | 0.999976173 | -0.086487166 |
| YOR291W | YPK9 | 0.15269937 | -0.5653518 | 0.57183937 | 0.999976173 | -0.086328868 |
| YMR261C | TPS3 | 0.15269937 | -0.564851 | 0.57218001 | 0.999976173 | -0.086252388 |
| YBR118W | TEF2 | 0.15269937 | -0.5645757 | 0.57236732 | 0.999976173 | -0.086210344 |
| YPR164W | MMS1 | 0.15269937 | -0.5641857 | 0.57263264 | 0.999976173 | -0.086150799 |
| YHR114W | BZZ1 | 0.15269937 | -0.5641344 | 0.57266757 | 0.999976173 | -0.086142961 |
| YLR390W | ECM19 | 0.15269937 | -0.5630441 | 0.57340974 | 0.999976173 | -0.085976474 |
| YML108W | YML108W | 0.15269937 | -0.5628166 | 0.57356464 | 0.999976173 | -0.085941739 |
| YLR064W | YLR064W | 0.15269937 | -0.5625147 | 0.57377027 | 0.999976173 | -0.085895634 |
| YPL178W | CBC2 | 0.15269937 | -0.5620763 | 0.5740689 | 0.999976173 | -0.085828694 |
| YOR011W | AUS1 | 0.15269937 | -0.5614312 | 0.57450847 | 0.999976173 | -0.08573019 |
| YGL089C | MF(ALPHA)2 | 0.15269937 | -0.5586673 | 0.57639364 | 0.999976173 | -0.08530814 |
| YDR252W | BTT1 | 0.15269937 | -0.5581451 | 0.57675015 | 0.999976173 | -0.085228397 |
| YDR262W | YDR262W | 0.15269937 | -0.5577781 | 0.57700074 | 0.999976173 | -0.085172362 |

| | | | | | | |
|---|---|---|---|---|---|---|
| YGR187C | HGH1 | 0.15269937 | -0.5577418 | 0.57702554 | 0.999976173 | -0.085166817 |
| YGL180W | ATG1 | 0.15269937 | -0.5573462 | 0.5772957 | 0.999976173 | -0.085106418 |
| YDR090C | YDR090C | 0.15269937 | -0.5572768 | 0.57734311 | 0.999976173 | -0.085095821 |
| YEL063C | CAN1 | 0.15269937 | -0.5572181 | 0.57738326 | 0.999976173 | -0.085086845 |
| YLR282C | YLR282C | 0.15269937 | -0.5569118 | 0.57759248 | 0.999976173 | -0.085040083 |
| YFL012W | YFL012W | 0.15269937 | -0.5548402 | 0.57900875 | 0.999976173 | -0.084723748 |
| YDR147W | EKI1 | 0.15269937 | -0.5546244 | 0.57915636 | 0.999976173 | -0.0846908 |
| YDR387C | YDR387C | 0.15269937 | -0.5541643 | 0.57947118 | 0.999976173 | -0.084620539 |
| YKL038W | RGT1 | 0.15269937 | -0.553592 | 0.57986285 | 0.999976173 | -0.084533153 |
| YML063W | RPS1B | 0.15269937 | -0.5518943 | 0.58102554 | 0.999976173 | -0.084273909 |
| YNL253W | TEX1 | 0.15269937 | -0.5516362 | 0.58120239 | 0.999976173 | -0.084234496 |
| YJR061W | YJR061W | 0.15269937 | -0.5516086 | 0.58122131 | 0.999976173 | -0.084230281 |
| YML035C-A | NA | 0.15269937 | -0.551558 | 0.58125597 | 0.999976173 | -0.084222559 |
| YOL071W | EMI5 | 0.15269937 | -0.5513589 | 0.58139245 | 0.999976173 | -0.084192149 |
| YGL219C | MDM34 | 0.15269937 | -0.5508365 | 0.58175054 | 0.999976173 | -0.084112379 |
| YGR181W | TIM13 | 0.15269937 | -0.5502841 | 0.58212924 | 0.999976173 | -0.08402804 |
| YGL241W | KAP114 | 0.15269937 | -0.5489294 | 0.58305865 | 0.999976173 | -0.083821167 |
| YNR040W | YNR040W | 0.15269937 | -0.5488377 | 0.58312155 | 0.999976173 | -0.083807172 |
| YGL242C | YGL242C | 0.15269937 | -0.5483352 | 0.58346646 | 0.999976173 | -0.083730444 |
| YGR142W | BTN2 | 0.15269937 | -0.5481506 | 0.5835932 | 0.999976173 | -0.083702254 |
| YNL296W | YNL296W | 0.15269937 | -0.5477163 | 0.58389143 | 0.999976173 | -0.083635934 |
| YMR034C | YMR034C | 0.15269937 | -0.5470978 | 0.58431623 | 0.999976173 | -0.083541492 |
| YAR002W | NUP60 | 0.15269937 | -0.5462513 | 0.58489788 | 0.999976173 | -0.083412233 |
| YCR009C | RVS161 | 0.15269937 | -0.545753 | 0.58524046 | 0.999976173 | -0.08333613 |
| YKL039W | PTM1 | 0.15269937 | -0.5455149 | 0.58540413 | 0.999976173 | -0.08329978 |
| YGR237C | YGR237C | 0.15269937 | -0.5453196 | 0.58553839 | 0.999976173 | -0.083269964 |
| YPR066W | UBA3 | 0.15269937 | -0.5445087 | 0.58609616 | 0.999976173 | -0.083146131 |
| YNR032W | PPG1 | 0.15269937 | -0.5401417 | 0.58910394 | 0.999976173 | -0.082479299 |
| YGR031W | YGR031W | 0.15269937 | -0.5388185 | 0.59001675 | 0.999976173 | -0.082277239 |
| YJR110W | YMR1 | 0.15269937 | -0.5384427 | 0.59027609 | 0.999976173 | -0.082219857 |
| YMR085W | YMR085W | 0.15269937 | -0.5383347 | 0.59035062 | 0.999976173 | -0.082203369 |
| YLR099C | ICT1 | 0.15269937 | -0.5373318 | 0.59104303 | 0.999976173 | -0.082050231 |
| YLR001C | YLR001C | 0.15269937 | -0.5373212 | 0.59105038 | 0.999976173 | -0.082048607 |
| YLR284C | ECI1 | 0.15269937 | -0.5370188 | 0.59125922 | 0.999976173 | -0.082002435 |
| YMR244W | YMR244W | 0.15269937 | -0.5366643 | 0.59150414 | 0.999976173 | -0.081948296 |
| YFL040W | YFL040W | 0.15269937 | -0.5356907 | 0.59217693 | 0.999976173 | -0.08179963 |
| YKR031C | SPO14 | 0.15269937 | -0.5344 | 0.59306942 | 0.999976173 | -0.081602537 |
| YGR017W | YGR017W | 0.15269937 | -0.5337608 | 0.59351159 | 0.999976173 | -0.081504942 |
| YER111C | SWI4 | 0.15269937 | -0.5335362 | 0.59366704 | 0.999976173 | -0.081470638 |
| YGL252C | RTG2 | 0.15269937 | -0.5323315 | 0.59450099 | 0.999976173 | -0.081286681 |
| YBR094W | PBY1 | 0.15269937 | -0.5314172 | 0.59513428 | 0.999976173 | -0.081147065 |
| YFR011C | AIM13 | 0.15269937 | -0.5311528 | 0.59531742 | 0.999976173 | -0.081106702 |
| YHR158C | KEL1 | 0.15269937 | -0.531138 | 0.59532772 | 0.999976173 | -0.081104433 |
| YOR380W | RDR1 | 0.15269937 | -0.5310985 | 0.59535505 | 0.999976173 | -0.081098411 |
| YMR169c | NA | 0.15269937 | -0.5305339 | 0.59574638 | 0.999976173 | -0.081012185 |
| YKL033W-A | YKL033W-A | 0.15269937 | -0.530335 | 0.59588423 | 0.999976173 | -0.080981818 |
| YMR123W | PKR1 | 0.15269937 | -0.5300537 | 0.59607922 | 0.999976173 | -0.080938869 |
| YLR044C | PDC1 | 0.15269937 | -0.5296412 | 0.59636525 | 0.999976173 | -0.080875878 |
| YER155C | BEM2 | 0.15269937 | -0.5287633 | 0.59697419 | 0.999976173 | -0.080741821 |
| YGR152C | RSR1 | 0.15269937 | -0.5286094 | 0.59708098 | 0.999976173 | -0.080718318 |
| YMR120C | ADE17 | 0.15269937 | -0.5275255 | 0.59783321 | 0.999976173 | -0.080552813 |
| YOL109W | ZEO1 | 0.15269937 | -0.5274552 | 0.59788202 | 0.999976173 | -0.080542078 |
| YNR071C | YNR071C | 0.15269937 | -0.5269273 | 0.5982486 | 0.999976173 | -0.080461462 |
| YDR055W | PST1 | 0.15269937 | -0.5255321 | 0.59921782 | 0.999976173 | -0.080248424 |
| YGR177C | ATF2 | 0.15269937 | -0.5253572 | 0.59933941 | 0.999976173 | -0.080221709 |
| YIR017C | MET28 | 0.15269937 | -0.5251874 | 0.59945743 | 0.999976173 | -0.080195781 |
| YJR094C | IME1 | 0.15269937 | -0.5247705 | 0.59974721 | 0.999976173 | -0.080132128 |
| YAL030W | SNC1 | 0.15269937 | -0.5243873 | 0.60001366 | 0.999976173 | -0.080073611 |
| YGR193C | PDX1 | 0.15269937 | -0.5242517 | 0.60010798 | 0.999976173 | -0.0800529 |
| YFR017C | YFR017C | 0.15269937 | -0.5238652 | 0.60037682 | 0.999976173 | -0.079993876 |
| YJL206C-A | NA | 0.15269937 | -0.5233801 | 0.60071426 | 0.999976173 | -0.079919806 |
| YJR140C | HIR3 | 0.15269937 | -0.5216071 | 0.60194836 | 0.999976173 | -0.079649077 |
| YOR154W | SLP1 | 0.15269937 | -0.5212933 | 0.60216695 | 0.999976173 | -0.07960115 |
| YBR295W | PCA1 | 0.15269937 | -0.5212315 | 0.60220996 | 0.999976173 | -0.079591722 |

| | | | | | | |
|---|---|---|---|---|---|---|
| YGR263C | SAY1 | 0.15269937 | -0.5209966 | 0.60237358 | 0.999976173 | -0.079555852 |
| YNL144C | YNL144C | 0.15269937 | -0.5209171 | 0.60242898 | 0.999976173 | -0.079543709 |
| YDR270W | CCC2 | 0.15269937 | -0.5203889 | 0.60279699 | 0.999976173 | -0.079463055 |
| YML066C | SMA2 | 0.15269937 | -0.5200812 | 0.60301143 | 0.999976173 | -0.079416069 |
| YOL041C | NOP12 | 0.15269937 | -0.5196576 | 0.60330666 | 0.999976173 | -0.079351391 |
| YOR195W | SLK19 | 0.15269937 | -0.5189168 | 0.60382318 | 0.999976173 | -0.079238269 |
| YDR393W | SHE9 | 0.15269937 | -0.5183489 | 0.60421926 | 0.999976173 | -0.079151553 |
| YEL001C | IRC22 | 0.15269937 | -0.518151 | 0.60435734 | 0.999976173 | -0.07912133 |
| YDR186C | YDR186C | 0.15269937 | -0.5180394 | 0.60443522 | 0.999976173 | -0.079104285 |
| YDL042C | SIR2 | 0.15269937 | -0.517856 | 0.60456315 | 0.999976173 | -0.079076285 |
| YLR342W | FKS1 | 0.15269937 | -0.5174658 | 0.60483545 | 0.999976173 | -0.0790167 |
| YCL033C | MXR2 | 0.15269937 | -0.5173408 | 0.60492269 | 0.999976173 | -0.078997612 |
| YJR098C | YJR098C | 0.15269937 | -0.5168206 | 0.60528578 | 0.999976173 | -0.078918185 |
| YNL167C | SKO1 | 0.15269937 | -0.5157156 | 0.60605743 | 0.999976173 | -0.07874945 |
| YLR152C | YLR152C | 0.15269937 | -0.5151912 | 0.60642383 | 0.999976173 | -0.078669366 |
| YLL007C | YLL007C | 0.15269937 | -0.5148122 | 0.60668867 | 0.999976173 | -0.078611493 |
| YLL041C | SDH2 | 0.15269937 | -0.5145903 | 0.60684376 | 0.999976173 | -0.078577607 |
| YMR237W | BCH1 | 0.15269937 | -0.5137082 | 0.60746038 | 0.999976173 | -0.07844292 |
| YKL137W | CMC1 | 0.15269937 | -0.5135992 | 0.60753664 | 0.999976173 | -0.078426268 |
| YNL226W | YNL226W | 0.15269937 | -0.513387 | 0.60768502 | 0.999976173 | -0.078393869 |
| YLR055C | SPT8 | 0.15269937 | -0.5123103 | 0.60843824 | 0.999976173 | -0.078229455 |
| YOL057W | YOL057W | 0.15269937 | -0.5118811 | 0.6087386 | 0.999976173 | -0.078163917 |
| YLR315W | NKP2 | 0.15269937 | -0.5118464 | 0.60876286 | 0.999976173 | -0.078158625 |
| YGR141W | VPS62 | 0.15269937 | -0.5113253 | 0.60912768 | 0.999976173 | -0.078079043 |
| YMR111C | YMR111C | 0.15269937 | -0.510736 | 0.60954028 | 0.999976173 | -0.077989063 |
| YLR079W | SIC1 | 0.15269937 | -0.5104831 | 0.60971738 | 0.999976173 | -0.077950451 |
| YDR458C | HEH2 | 0.15269937 | -0.5103342 | 0.60982167 | 0.999976173 | -0.077927714 |
| YNL115C | YNL115C | 0.15269937 | -0.5098302 | 0.61017475 | 0.999976173 | -0.077850752 |
| YKL157W | APE2 | 0.15269937 | -0.5094739 | 0.61042446 | 0.999976173 | -0.077796335 |
| YNL302C | RPS19B | 0.15269937 | -0.509146 | 0.61065421 | 0.999976173 | -0.077746274 |
| YIL093C | RSM25 | 0.15269937 | -0.5090522 | 0.61071994 | 0.999976173 | -0.077731954 |
| YPR026W | ATH1 | 0.15269937 | -0.5082561 | 0.61127805 | 0.999976173 | -0.077610391 |
| YLR054C | OSW2 | 0.15269937 | -0.5070322 | 0.61213655 | 0.999976173 | -0.077423494 |
| YGR269W | YGR269W | 0.15269937 | -0.5067692 | 0.6123211 | 0.999976173 | -0.077383332 |
| YNR058W | BIO3 | 0.15269937 | -0.5051034 | 0.61349051 | 0.999976173 | -0.077128969 |
| YLR031W | YLR031W | 0.15269937 | -0.5042094 | 0.61411851 | 0.999976173 | -0.076992459 |
| YGR129W | SYF2 | 0.15269937 | -0.5034995 | 0.61461742 | 0.999976173 | -0.076884054 |
| YJR153W | PGU1 | 0.15269937 | -0.5032008 | 0.61482736 | 0.999976173 | -0.076838448 |
| YAR023C | YAR023C | 0.15269937 | -0.5027017 | 0.61517829 | 0.999976173 | -0.076762229 |
| YHR179W | OYE2 | 0.15269937 | -0.5025704 | 0.61527062 | 0.999976173 | -0.07674218 |
| YGL194C | HOS2 | 0.15269937 | -0.5015183 | 0.61601067 | 0.999976173 | -0.076581526 |
| YNR008W | LRO1 | 0.15269937 | -0.5009234 | 0.61642927 | 0.999976173 | -0.076490689 |
| YGR051C | YGR051C | 0.15269937 | -0.5007011 | 0.61658572 | 0.999976173 | -0.076456748 |
| YML104C | MDM1 | 0.15269937 | -0.5002046 | 0.61693523 | 0.999976173 | -0.076380933 |
| YNL116W | DMA2 | 0.15269937 | -0.5001439 | 0.61697797 | 0.999976173 | -0.076371663 |
| YNL142W | MEP2 | 0.15269937 | -0.4992587 | 0.6176014 | 0.999976173 | -0.076236483 |
| YBR298C | MAL31 | 0.15269937 | -0.4990373 | 0.61775735 | 0.999976173 | -0.076202679 |
| YLR172C | DPH5 | 0.15269937 | -0.4979642 | 0.61851354 | 0.999976173 | -0.07603881 |
| YMR119W | ASI1 | 0.10797476 | -0.7035066 | 0.48174643 | 0.999976173 | -0.075960952 |
| YKL175W | ZRT3 | 0.15269937 | -0.4957245 | 0.62009298 | 0.999976173 | -0.075696821 |
| YNL072W | RNH201 | 0.15269937 | -0.4956679 | 0.62013295 | 0.999976173 | -0.075688172 |
| YNL069C | RPL16B | 0.15269937 | -0.4946287 | 0.62086645 | 0.999976173 | -0.075529486 |
| YNL064C | YDJ1 | 0.15269937 | -0.493553 | 0.62162607 | 0.999976173 | -0.075365234 |
| YMR285C | NGL2 | 0.15269937 | -0.4927015 | 0.62222773 | 0.999976173 | -0.075235199 |
| YDL173W | PAR32 | 0.15269937 | -0.4920372 | 0.62269719 | 0.999976173 | -0.075133774 |
| YER011W | TIR1 | 0.15269937 | -0.4910716 | 0.62337997 | 0.999976173 | -0.07498632 |
| YGR192C | TDH3 | 0.15269937 | -0.4909451 | 0.62346945 | 0.999976173 | -0.074967003 |
| YFR041C | ERJ5 | 0.15269937 | -0.490927 | 0.62348223 | 0.999976173 | -0.074964242 |
| YDL231C | BRE4 | 0.15269937 | -0.4875385 | 0.62588093 | 0.999976173 | -0.074446813 |
| YJR121W | ATP2 | 0.15269937 | -0.4859466 | 0.62700915 | 0.999976173 | -0.074203734 |
| YIL066C | RNR3 | 0.15269937 | -0.4853463 | 0.62743482 | 0.999976173 | -0.074112072 |
| YML094W | GIM5 | 0.15269937 | -0.4853185 | 0.62745456 | 0.999976173 | -0.074107823 |
| YDR076W | RAD55 | 0.15269937 | -0.4851739 | 0.6275571 | 0.999976173 | -0.074085747 |
| YMR273C | ZDS1 | 0.15269937 | -0.4829406 | 0.62914197 | 0.999976173 | -0.073744729 |

| | | | | | | |
|---|---|---|---|---|---|---|
| YOR014W | RTS1 | 0.15269937 | -0.4824613 | 0.62948236 | 0.999976173 | -0.073671535 |
| YER177W | BMH1 | 0.15269937 | -0.4817825 | 0.62996451 | 0.999976173 | -0.073567887 |
| YNL246W | VPS75 | 0.15269937 | -0.479848 | 0.63133955 | 0.999976173 | -0.073272482 |
| YHR016C | YSC84 | 0.15269937 | -0.4795449 | 0.63155509 | 0.999976173 | -0.073226202 |
| YNL147W | LSM7 | 0.15269937 | -0.479084 | 0.63188291 | 0.999976173 | -0.073155824 |
| YDR477W | SNF1 | 0.15269937 | -0.4787384 | 0.63212881 | 0.999976173 | -0.073103044 |
| YLR143W | YLR143W | 0.15269937 | -0.4783585 | 0.6323991 | 0.999976173 | -0.07304504 |
| YGL049C | TIF4632 | 0.15269937 | -0.4779851 | 0.63266484 | 0.999976173 | -0.072988023 |
| YCL029C | BIK1 | 0.15269937 | -0.4777521 | 0.63283068 | 0.999976173 | -0.072952445 |
| YOR288C | MPD1 | 0.15269937 | -0.4770895 | 0.6333024 | 0.999976173 | -0.072851267 |
| YCL045C | EMC1 | 0.15269937 | -0.4768197 | 0.63349455 | 0.999976173 | -0.072810063 |
| YLR413W | YLR413W | 0.15269937 | -0.4767989 | 0.63350937 | 0.999976173 | -0.072806885 |
| YLR319C | BUD6 | 0.15269937 | -0.4764366 | 0.63376738 | 0.999976173 | -0.072751567 |
| YMR245W | YMR245W | 0.15269937 | -0.4757616 | 0.63424825 | 0.999976173 | -0.072648493 |
| YDR125C | ECM18 | 0.15269937 | -0.4733157 | 0.63599194 | 0.999976173 | -0.072275009 |
| YEL056W | HAT2 | 0.15269937 | -0.4730754 | 0.63616334 | 0.999976173 | -0.072238321 |
| YKL029C | MAE1 | 0.15269937 | -0.4714494 | 0.63732384 | 0.999976173 | -0.07199002 |
| YPL140C | MKK2 | 0.15269937 | -0.470759 | 0.63781681 | 0.999976173 | -0.071884601 |
| YJL214W | HXT8 | 0.15269937 | -0.4705439 | 0.63797043 | 0.999976173 | -0.071851757 |
| YMR153C-A | YMR153C-A | 0.15269937 | -0.4699261 | 0.63841175 | 0.999976173 | -0.071757422 |
| YBL064C | PRX1 | 0.15269937 | -0.4695965 | 0.63864731 | 0.999976173 | -0.071707082 |
| YFR057W | YFR057W | 0.15269937 | -0.4695396 | 0.63868796 | 0.999976173 | -0.071698394 |
| YDL076C | RXT3 | 0.15269937 | -0.4693373 | 0.63883249 | 0.999976173 | -0.071667512 |
| YER061C | CEM1 | 0.15269937 | -0.4690999 | 0.6390022 | 0.999976173 | -0.071631252 |
| YOL158C | ENB1 | 0.15269937 | -0.4688336 | 0.63919249 | 0.999976173 | -0.071590601 |
| YDL240W | LRG1 | 0.15269937 | -0.4682619 | 0.63960124 | 0.999976173 | -0.071503296 |
| YDR291W | HRQ1 | 0.15269937 | -0.4679279 | 0.63984009 | 0.999976173 | -0.071452293 |
| YOR222W | ODC2 | 0.15269937 | -0.4677715 | 0.63995194 | 0.999976173 | -0.071428411 |
| YMR302C | YME2 | 0.15269937 | -0.4671876 | 0.6403696 | 0.999976173 | -0.071339249 |
| YML102W | CAC2 | 0.15269937 | -0.4643008 | 0.64243612 | 0.999976173 | -0.070898445 |
| YLL029W | FRA1 | 0.15269937 | -0.4613989 | 0.64451631 | 0.999976173 | -0.070455321 |
| YCR092C | MSH3 | 0.15269937 | -0.4608998 | 0.64487439 | 0.999976173 | -0.070379101 |
| YKL032C | IXR1 | 0.15269937 | -0.4608682 | 0.64489703 | 0.999976173 | -0.070374284 |
| YJR052W | RAD7 | 0.15269937 | -0.4607973 | 0.6449479 | 0.999976173 | -0.070363457 |
| YMR019W | STB4 | 0.15269937 | -0.4595634 | 0.64583348 | 0.999976173 | -0.070175042 |
| YGL094C | PAN2 | 0.15269937 | -0.4593847 | 0.64596177 | 0.999976173 | -0.070147755 |
| YJR134C | SGM1 | 0.15269937 | -0.4589723 | 0.64625788 | 0.999976173 | -0.070084785 |
| YBR294W | SUL1 | 0.15269937 | -0.4589435 | 0.64627857 | 0.999976173 | -0.070080386 |
| YKR051W | YKR051W | 0.15269937 | -0.4582352 | 0.64678729 | 0.999976173 | -0.069972231 |
| YBR208C | DUR1,2 | 0.15269937 | -0.4560827 | 0.64833434 | 0.999976173 | -0.069643537 |
| YOR030W | DFG16 | 0.15269937 | -0.4558921 | 0.6484714 | 0.999976173 | -0.069614433 |
| YBR157C | ICS2 | 0.15269937 | -0.455256 | 0.64892889 | 0.999976173 | -0.069517303 |
| YGL036W | YGL036W | 0.15269937 | -0.4546782 | 0.64934456 | 0.999976173 | -0.069429077 |
| YMR271C | URA10 | 0.15269937 | -0.4544312 | 0.64952229 | 0.999976173 | -0.06939136 |
| YOR128C | ADE2 | 0.15269937 | -0.4540258 | 0.64981404 | 0.999976173 | -0.069329455 |
| YDL243C | AAD4 | 0.15269937 | -0.4531565 | 0.65043986 | 0.999976173 | -0.069196708 |
| YDR255C | RMD5 | 0.15269937 | -0.4515484 | 0.65159816 | 0.999976173 | -0.068951147 |
| YOR078W | BUD21 | 0.15269937 | -0.4510219 | 0.65197751 | 0.999976173 | -0.068870765 |
| YEL048C | TCA17 | 0.15269937 | -0.4506205 | 0.65226685 | 0.999976173 | -0.068809467 |
| YOR079C | ATX2 | 0.15269937 | -0.4490399 | 0.65340666 | 0.999976173 | -0.068568102 |
| YGR042W | YGR042W | 0.15269937 | -0.4487553 | 0.65361191 | 0.999976173 | -0.068524657 |
| YPL212C | PUS1 | 0.15269937 | -0.4485175 | 0.65378353 | 0.999976173 | -0.068488335 |
| YDR174W | HMO1 | 0.15269937 | -0.4482485 | 0.6539776 | 0.999976173 | -0.068447265 |
| YOR049C | RSB1 | 0.15269937 | -0.4482464 | 0.65397914 | 0.999976173 | -0.068446938 |
| YGL161C | YIP5 | 0.15269937 | -0.4471246 | 0.65478882 | 0.999976173 | -0.068275647 |
| YLR185W | RPL37A | 0.15269937 | -0.4462057 | 0.65545242 | 0.999976173 | -0.068135322 |
| YOL012C | HTZ1 | 0.15269937 | -0.445617 | 0.65587768 | 0.999976173 | -0.068045427 |
| YGR214W | RPS0A | 0.15269937 | -0.4435595 | 0.65736478 | 0.999976173 | -0.067731258 |
| YJL067W | YJL067W | 0.15269937 | -0.4432415 | 0.65759477 | 0.999976173 | -0.067682695 |
| YNL004W | HRB1 | 0.15269937 | -0.4432043 | 0.65762164 | 0.999976173 | -0.067677021 |
| YBR213W | MET8 | 0.15269937 | -0.4427712 | 0.65793492 | 0.999976173 | -0.067610883 |
| YDR452W | PPN1 | 0.15269937 | -0.442679 | 0.65800161 | 0.999976173 | -0.067596807 |
| YMR039C | SUB1 | 0.15269937 | -0.4422954 | 0.65827915 | 0.999976173 | -0.067538228 |
| YGL031C | RPL24A | 0.15269937 | -0.442086 | 0.65843068 | 0.999976173 | -0.067506249 |

| | | | | | | |
|---|---|---|---|---|---|---|
| YNL338W | YNL338W | 0.15269937 | -0.4420197 | 0.65847862 | 0.999976173 | -0.067496133 |
| YKL051W | SFK1 | 0.15269937 | -0.4417199 | 0.65869562 | 0.999976173 | -0.067450343 |
| YDR513W | GRX2 | 0.15269937 | -0.4417032 | 0.65870765 | 0.999976173 | -0.067447805 |
| YJL062W | LAS21 | 0.15269937 | -0.4414739 | 0.65887364 | 0.999976173 | -0.067412785 |
| YMR008C | PLB1 | 0.15269937 | -0.4411252 | 0.65912605 | 0.999976173 | -0.067359537 |
| YFR032C-A | RPL29 | 0.15269937 | -0.4402888 | 0.65973161 | 0.999976173 | -0.067231823 |
| YAR020C | PAU7 | 0.15269937 | -0.4400178 | 0.65992791 | 0.999976173 | -0.067190434 |
| YJR149W | YJR149W | 0.15269937 | -0.4395334 | 0.66027874 | 0.999976173 | -0.067116474 |
| YJR050W | ISY1 | 0.15269937 | -0.439025 | 0.66064711 | 0.999976173 | -0.067038833 |
| YOR018W | ROD1 | 0.15269937 | -0.4384661 | 0.66105213 | 0.999976173 | -0.066953488 |
| YGL071W | AFT1 | 0.15269937 | -0.4382285 | 0.66122431 | 0.999976173 | -0.066917213 |
| YBR106W | PHO88 | 0.15269937 | -0.4358703 | 0.66293448 | 0.999976173 | -0.066557115 |
| YFR053C | HXK1 | 0.15269937 | -0.4358661 | 0.66293749 | 0.999976173 | -0.066556482 |
| YNL293W | MSB3 | 0.15269937 | -0.4352322 | 0.66339753 | 0.999976173 | -0.066459678 |
| YHR095W | YHR095W | 0.15269937 | -0.4349642 | 0.66359203 | 0.999976173 | -0.066418759 |
| YLR180W | SAM1 | 0.15269937 | -0.4347296 | 0.66376234 | 0.999976173 | -0.066382932 |
| YFR049W | YMR31 | 0.15269937 | -0.4342416 | 0.66411659 | 0.999976173 | -0.066308423 |
| YDR422C | SIP1 | 0.15269937 | -0.4340346 | 0.66426694 | 0.999976173 | -0.066276805 |
| YFR032C | RRT5 | 0.15269937 | -0.4338341 | 0.66441254 | 0.999976173 | -0.066246189 |
| YOR024W | YOR024W | 0.15269937 | -0.4333817 | 0.66474112 | 0.999976173 | -0.066177106 |
| YLR414C | YLR414C | 0.15269937 | -0.4320213 | 0.66572955 | 0.999976173 | -0.065969374 |
| YJR106W | ECM27 | 0.15269937 | -0.4319339 | 0.66579308 | 0.999976173 | -0.065956026 |
| YBR008C | FLR1 | 0.15269937 | -0.4315373 | 0.66608133 | 0.999976173 | -0.065895472 |
| YDL134C-A | NA | 0.15269937 | -0.4304109 | 0.66690036 | 0.999976173 | -0.06572347 |
| YML067C | ERV41 | 0.15269937 | -0.4302486 | 0.66701836 | 0.999976173 | -0.065698695 |
| YGL056C | SDS23 | 0.15269937 | -0.4291175 | 0.66784132 | 0.999976173 | -0.065525963 |
| YHL027W | RIM101 | 0.15269937 | -0.4288598 | 0.66802881 | 0.999976173 | -0.065486622 |
| YLR253W | YLR253W | 0.15269937 | -0.4271904 | 0.66924418 | 0.999976173 | -0.065231707 |
| YLR354C | TAL1 | 0.15269937 | -0.426967 | 0.66940692 | 0.999976173 | -0.065197588 |
| YGL147C | RPL9A | 0.15269937 | -0.4262066 | 0.66996087 | 0.999976173 | -0.065081473 |
| YOR070C | GYP1 | 0.15269937 | -0.4259653 | 0.67013669 | 0.999976173 | -0.065044627 |
| YMR305C | SCW10 | 0.15269937 | -0.4253379 | 0.67059387 | 0.999976173 | -0.064948835 |
| YDR528W | HLR1 | 0.15269937 | -0.4251749 | 0.67071273 | 0.999976173 | -0.064923934 |
| YBL078C | ATG8 | 0.15269937 | -0.4244028 | 0.6712756 | 0.999976173 | -0.064806039 |
| YGL170C | SPO74 | 0.15269937 | -0.4239307 | 0.6716199 | 0.999976173 | -0.064733943 |
| YDR162C | NBP2 | 0.15269937 | -0.4236082 | 0.67185512 | 0.999976173 | -0.064684697 |
| YML053C | YML053C | 0.15269937 | -0.4227516 | 0.67247999 | 0.999976173 | -0.064553906 |
| YPL247C | YPL247C | 0.15269937 | -0.4214413 | 0.67343635 | 0.999976173 | -0.064353822 |
| YPL248C | GAL4 | 0.15269937 | -0.4207513 | 0.67394016 | 0.999976173 | -0.064248462 |
| YGR207C | YGR207C | 0.15269937 | -0.420169 | 0.67436551 | 0.999976173 | -0.064159534 |
| YGR279C | SCW4 | 0.15269937 | -0.4195003 | 0.67485401 | 0.999976173 | -0.06405743 |
| YDR018C | YDR018C | 0.15269937 | -0.417355 | 0.67642222 | 0.999976173 | -0.063729844 |
| YAR047C | YAR047C | 0.15269937 | -0.4164355 | 0.67709481 | 0.999976173 | -0.063589437 |
| YOL098C | YOL098C | 0.15269937 | -0.4162668 | 0.67721827 | 0.999976173 | -0.063563669 |
| YLL013C | PUF3 | 0.15269937 | -0.4161958 | 0.67727016 | 0.999976173 | -0.063552838 |
| YJR131W | MNS1 | 0.15269937 | -0.4161538 | 0.67730089 | 0.999976173 | -0.063546425 |
| YKR045C | YKR045C | 0.15269937 | -0.4158719 | 0.67750716 | 0.999976173 | -0.063503381 |
| YCL056C | YCL056C | 0.15269937 | -0.4154947 | 0.67778323 | 0.999976173 | -0.063445778 |
| YGL250W | RMR1 | 0.15269937 | -0.4150787 | 0.67808773 | 0.999976173 | -0.063382254 |
| YBR283C | SSH1 | 0.15269937 | -0.4132787 | 0.67940587 | 0.999976173 | -0.063107391 |
| YGR153W | YGR153W | 0.15269937 | -0.4113223 | 0.68083962 | 0.999976173 | -0.062808655 |
| YMR037C | MSN2 | 0.15269937 | -0.4109287 | 0.68112818 | 0.999976173 | -0.062748559 |
| YOL141W | PPM2 | 0.15269937 | -0.4100866 | 0.68174582 | 0.999976173 | -0.062619962 |
| YMR272C | SCS7 | 0.15269937 | -0.4097654 | 0.68198142 | 0.999976173 | -0.06257092 |
| YKR078W | YKR078W | 0.15269937 | -0.4090488 | 0.68250722 | 0.999976173 | -0.062461494 |
| YLR218C | YLR218C | 0.15269937 | -0.4077026 | 0.68349538 | 0.999976173 | -0.062255933 |
| YOR162C | YRR1 | 0.15269937 | -0.407426 | 0.68369852 | 0.999976173 | -0.062213688 |
| YDR525W | API2 | 0.15269937 | -0.4073615 | 0.68374585 | 0.999976173 | -0.062203845 |
| YJL077C | ICS3 | 0.15269937 | -0.4070662 | 0.68396271 | 0.999976173 | -0.062158755 |
| YNR056C | BIO5 | 0.15269937 | -0.4068562 | 0.68411698 | 0.999976173 | -0.062126682 |
| YML090W | YML090W | 0.15269937 | -0.4066764 | 0.68424907 | 0.999976173 | -0.062099222 |
| YLR406C | RPL31B | 0.15269937 | -0.4059807 | 0.68476012 | 0.999976173 | -0.061992998 |
| YAR029W | YAR029W | 0.15269937 | -0.4057303 | 0.68494414 | 0.999976173 | -0.061954757 |
| YNL292W | PUS4 | 0.15269937 | -0.4057137 | 0.68495632 | 0.999976173 | -0.061952226 |

| | | | | | | |
|---|---|---|---|---|---|---|
| YOL002C | IZH2 | 0.15269937 | -0.4054299 | 0.68516487 | 0.999976173 | -0.061908893 |
| YNL242W | ATG2 | 0.15269937 | -0.4043194 | 0.68598119 | 0.999976173 | -0.061739317 |
| YDR338C | YDR338C | 0.15269937 | -0.4041905 | 0.68607601 | 0.999976173 | -0.061719627 |
| YGR199W | PMT6 | 0.15269937 | -0.4039715 | 0.68623699 | 0.999976173 | -0.061686196 |
| YPL183C | RTT10 | 0.15269937 | -0.4034399 | 0.686628 | 0.999976173 | -0.061605011 |
| YFL047W | RGD2 | 0.15269937 | -0.4033422 | 0.68669986 | 0.999976173 | -0.061590091 |
| YBR238C | YBR238C | 0.15269937 | -0.4033272 | 0.68671088 | 0.999976173 | -0.061587804 |
| YKL103C | LAP4 | 0.15269937 | -0.4031899 | 0.68681186 | 0.999976173 | -0.061566842 |
| YMR010W | YMR010W | 0.15269937 | -0.4029766 | 0.68696874 | 0.999976173 | -0.061534276 |
| YDR537C | YDR537C | 0.15269937 | -0.4029175 | 0.68701225 | 0.999976173 | -0.061525245 |
| YOL084W | PHM7 | 0.15269937 | -0.4029111 | 0.68701698 | 0.999976173 | -0.061524263 |
| YIL072W | HOP1 | 0.15269937 | -0.4024144 | 0.6873824 | 0.999976173 | -0.061448422 |
| YOL160W | YOL160W | 0.15269937 | -0.4022512 | 0.68750249 | 0.999976173 | -0.061423503 |
| YAL068C | PAU8 | 0.15269937 | -0.4015281 | 0.68803468 | 0.999976173 | -0.061313084 |
| YNL020C | ARK1 | 0.15269937 | -0.4013732 | 0.68814869 | 0.999976173 | -0.061289433 |
| YMR172C-A | YMR172C-A | 0.15269937 | -0.3999045 | 0.68923017 | 0.999976173 | -0.061065161 |
| YPL014W | YPL014W | 0.15269937 | -0.3995116 | 0.68951955 | 0.999976173 | -0.061005172 |
| YMR326C | YMR326C | 0.15269937 | -0.3994546 | 0.68956155 | 0.999976173 | -0.060996468 |
| YGL081W | YGL081W | 0.15269937 | -0.399089 | 0.68983089 | 0.999976173 | -0.060940643 |
| YJL068C | YJL068C | 0.15269937 | -0.3987439 | 0.69008523 | 0.999976173 | -0.060887936 |
| YNL027W | CRZ1 | 0.15269937 | -0.3973003 | 0.69114933 | 0.999976173 | -0.060667497 |
| YGR127W | YGR127W | 0.15269937 | -0.3971691 | 0.69124604 | 0.999976173 | -0.060647469 |
| YLR013W | GAT3 | 0.15269937 | -0.3967531 | 0.69155282 | 0.999976173 | -0.060583943 |
| YBR205W | KTR3 | 0.15269937 | -0.3966481 | 0.69163026 | 0.999976173 | -0.060567909 |
| YOR166C | SWT1 | 0.15269937 | -0.3958752 | 0.69220039 | 0.999976173 | -0.060449885 |
| YBR290W | BSD2 | 0.15269937 | -0.3952115 | 0.69269008 | 0.999976173 | -0.06034854 |
| YER092W | IES5 | 0.15269937 | -0.3951364 | 0.6927455 | 0.999976173 | -0.060337074 |
| YCL035C | GRX1 | 0.15269937 | -0.3931039 | 0.69424598 | 0.999976173 | -0.060026716 |
| YBR018C | GAL7 | 0.15269937 | -0.3930452 | 0.69428935 | 0.999976173 | -0.06001775 |
| YPL164C | MLH3 | 0.15269937 | -0.392951 | 0.69435893 | 0.999976173 | -0.060003364 |
| YOR235W | IRC13 | 0.15269937 | -0.3927313 | 0.69452116 | 0.999976173 | -0.059969824 |
| YBR138C | YBR138C | 0.15269937 | -0.3924241 | 0.69474811 | 0.999976173 | -0.059922912 |
| YER084W | YER084W | 0.15269937 | -0.3918105 | 0.69520148 | 0.999976173 | -0.059829211 |
| YDR015C | YDR015C | 0.15269937 | -0.3914471 | 0.69547001 | 0.999976173 | -0.059773723 |
| YDR276C | PMP3 | 0.15269937 | -0.3909245 | 0.69585623 | 0.999976173 | -0.059693929 |
| YCR024C-A | PMP1 | 0.15269937 | -0.3907295 | 0.69600038 | 0.999976173 | -0.059664152 |
| YGL144C | ROG1 | 0.15269937 | -0.3903972 | 0.69624608 | 0.999976173 | -0.059613402 |
| YBR189W | RPS9B | 0.15269937 | -0.3893239 | 0.69703974 | 0.999976173 | -0.059449517 |
| YMR262W | YMR262W | 0.15269937 | -0.389175 | 0.69714989 | 0.999976173 | -0.059426777 |
| YOR182C | RPS30B | 0.15269937 | -0.3890135 | 0.69726937 | 0.999976173 | -0.059402112 |
| YLR194C | YLR194C | 0.15269937 | -0.3878147 | 0.69815637 | 0.999976173 | -0.059219055 |
| YGL173C | KEM1 | 0.15269937 | -0.3869854 | 0.69877016 | 0.999976173 | -0.059092432 |
| YBR054W | YRO2 | 0.15269937 | -0.3864952 | 0.69913312 | 0.999976173 | -0.059017575 |
| YMR306W | FKS3 | 0.15269937 | -0.3862964 | 0.69928032 | 0.999976173 | -0.058987219 |
| YDL054C | MCH1 | 0.15269937 | -0.3857988 | 0.69964882 | 0.999976173 | -0.058911238 |
| YBR293W | VBA2 | 0.15269937 | -0.3855056 | 0.69986601 | 0.999976173 | -0.058866464 |
| YPR018W | RLF2 | 0.15269937 | -0.3845449 | 0.70057781 | 0.999976173 | -0.058719756 |
| YGL214W | YGL214W | 0.15269937 | -0.3844087 | 0.7006787 | 0.999976173 | -0.058698967 |
| YOR099W | KTR1 | 0.15269937 | -0.3831465 | 0.70161428 | 0.999976173 | -0.058506229 |
| YHR003C | YHR003C | 0.15269937 | -0.3830797 | 0.70166379 | 0.999976173 | -0.058496031 |
| YGR213C | RTA1 | 0.15269937 | -0.3830392 | 0.70169381 | 0.999976173 | -0.05848985 |
| YBR258C | SHG1 | 0.15269937 | -0.3829695 | 0.70174549 | 0.999976173 | -0.058479206 |
| YGL228W | SHE10 | 0.15269937 | -0.3816545 | 0.7027208 | 0.999976173 | -0.058278396 |
| YOR034C | AKR2 | 0.15269937 | -0.3813033 | 0.70298135 | 0.999976173 | -0.058224768 |
| YMR311C | GLC8 | 0.15269937 | -0.380992 | 0.7032123 | 0.999976173 | -0.058177238 |
| YBR176W | ECM31 | 0.15269937 | -0.3809366 | 0.70325342 | 0.999976173 | -0.058168776 |
| YKL190W | CNB1 | 0.15269937 | -0.3796155 | 0.70423397 | 0.999976173 | -0.057967044 |
| YDR126W | SWF1 | 0.15269937 | -0.3795128 | 0.70431021 | 0.999976173 | -0.057951365 |
| YMR276W | DSK2 | 0.15269937 | -0.3788836 | 0.70477743 | 0.999976173 | -0.05785528 |
| YBR197C | YBR197C | 0.15269937 | -0.3779433 | 0.70547583 | 0.999976173 | -0.057711699 |
| YBL107C | YBL107C | 0.15269937 | -0.3778477 | 0.70554686 | 0.999976173 | -0.057697098 |
| YGR224W | AZR1 | 0.15269937 | -0.3776118 | 0.70572207 | 0.999976173 | -0.057661087 |
| YMR018W | YMR018W | 0.15269937 | -0.3771442 | 0.70606957 | 0.999976173 | -0.057589674 |
| YPL026C | SKS1 | 0.15269937 | -0.376164 | 0.70679807 | 0.999976173 | -0.057440002 |

| | | | | | | |
|---|---|---|---|---|---|---|
| YLR048W | RPS0B | 0.15269937 | -0.3755261 | 0.70727234 | 0.999976173 | -0.057342593 |
| YOR093C | YOR093C | 0.15269937 | -0.3754599 | 0.70732151 | 0.999976173 | -0.057332494 |
| YGL043W | DST1 | 0.15269937 | -0.3731749 | 0.70902137 | 0.999976173 | -0.056983563 |
| YJR032W | CPR7 | 0.15269937 | -0.3724745 | 0.70954264 | 0.999976173 | -0.05687662 |
| YBR278W | DPB3 | 0.15269937 | -0.37232 | 0.70965767 | 0.999976173 | -0.056853025 |
| YJR043C | POL32 | 0.15269937 | -0.3720992 | 0.70982203 | 0.999976173 | -0.056819313 |
| YER033C | ZRG8 | 0.15269937 | -0.3715252 | 0.71024946 | 0.999976173 | -0.056731655 |
| YHR049C-A | YHR049C-A | 0.15269937 | -0.3711475 | 0.71053071 | 0.999976173 | -0.056673987 |
| YMR316C-B | YMR316C-B | 0.15269937 | -0.3707464 | 0.71082945 | 0.999976173 | -0.056612743 |
| YGL140C | YGL140C | 0.15269937 | -0.3697707 | 0.71155638 | 0.999976173 | -0.056463749 |
| YER139C | RTR1 | 0.15269937 | -0.36977 | 0.71155687 | 0.999976173 | -0.05646365 |
| YNR060W | FRE4 | 0.15269937 | -0.369727 | 0.71158897 | 0.999976173 | -0.056457072 |
| YHR009C | YHR009C | 0.15269937 | -0.3683286 | 0.71263124 | 0.999976173 | -0.056243543 |
| YDR278C | YDR278C | 0.15269937 | -0.3681205 | 0.71278642 | 0.999976173 | -0.056211762 |
| YER060W-A | FCY22 | 0.15269937 | -0.3680649 | 0.71282786 | 0.999976173 | -0.056203276 |
| YKL096W-A | CWP2 | 0.15269937 | -0.3665332 | 0.71397026 | 0.999976173 | -0.055969382 |
| YLR217W | YLR217W | 0.15269937 | -0.3659612 | 0.71439699 | 0.999976173 | -0.055882048 |
| YKR082W | NUP133 | 0.15269937 | -0.3659025 | 0.71444084 | 0.999976173 | -0.055873075 |
| YLR426W | YLR426W | 0.15269937 | -0.365205 | 0.71496138 | 0.999976173 | -0.05576657 |
| YLR006C | SSK1 | 0.15269937 | -0.3651419 | 0.71500849 | 0.999976173 | -0.055756931 |
| YDL216C | RRI1 | 0.15269937 | -0.364813 | 0.71525399 | 0.999976173 | -0.055706712 |
| YBR169C | SSE2 | 0.15269937 | -0.3643938 | 0.71556693 | 0.999976173 | -0.055642704 |
| YAL015C | NTG1 | 0.15269937 | -0.3643288 | 0.71561549 | 0.999976173 | -0.055632774 |
| YOL064C | MET22 | 0.15269937 | -0.3641551 | 0.71574514 | 0.999976173 | -0.055606258 |
| YDL199C | YDL199C | 0.15269937 | -0.3635573 | 0.71619155 | 0.999976173 | -0.055514975 |
| YLR193C | UPS1 | 0.15269937 | -0.3630392 | 0.71657855 | 0.999976173 | -0.055435858 |
| YOL116W | MSN1 | 0.15269937 | -0.3629481 | 0.71664665 | 0.999976173 | -0.055421938 |
| YBR174C | YBR174C | 0.15269937 | -0.3625166 | 0.716969 | 0.999976173 | -0.05535605 |
| YGR230W | BNS1 | 0.15269937 | -0.3618004 | 0.71750417 | 0.999976173 | -0.055246686 |
| YDL182W | LYS20 | 0.15269937 | -0.361286 | 0.71788863 | 0.999976173 | -0.055168138 |
| YGL085W | YGL085W | 0.15269937 | -0.3612058 | 0.71794852 | 0.999976173 | -0.055155903 |
| YER117W | RPL23B | 0.15269937 | -0.3611653 | 0.71797885 | 0.999976173 | -0.055149707 |
| YGL015C | YGL015C | 0.15269937 | -0.3607647 | 0.71827831 | 0.999976173 | -0.055088537 |
| YGL205W | POX1 | 0.15269937 | -0.3606464 | 0.71836675 | 0.999976173 | -0.055070475 |
| YLR073C | RFU1 | 0.15269937 | -0.360384 | 0.71856296 | 0.999976173 | -0.055030401 |
| YLR184W | YLR184W | 0.15269937 | -0.3602066 | 0.71869555 | 0.999976173 | -0.055003324 |
| YJR062C | NTA1 | 0.15269937 | -0.3601934 | 0.71870542 | 0.999976173 | -0.055001309 |
| YFL023W | BUD27 | 0.15269937 | -0.3596322 | 0.71912517 | 0.999976173 | -0.054915601 |
| YER005W | YND1 | 0.15269937 | -0.3593831 | 0.71931142 | 0.999976173 | -0.054877578 |
| YLR121C | YPS3 | 0.15269937 | -0.3593706 | 0.71932078 | 0.999976173 | -0.054875666 |
| YDR318W | MCM21 | 0.15269937 | -0.359302 | 0.71937214 | 0.999976173 | -0.054865181 |
| YNL304W | YPT11 | 0.15269937 | -0.3592566 | 0.71940603 | 0.999976173 | -0.054858261 |
| YMR063W | RIM9 | 0.15269937 | -0.3581688 | 0.7202199 | 0.999976173 | -0.054692151 |
| YDL181W | INH1 | 0.15269937 | -0.3575243 | 0.72070228 | 0.999976173 | -0.054593727 |
| YOR192C | THI72 | 0.15269937 | -0.3569607 | 0.72112417 | 0.999976173 | -0.054507665 |
| YOR080W | DIA2 | 0.15269937 | -0.3553019 | 0.72236631 | 0.999976173 | -0.054254377 |
| YOR165W | SEY1 | 0.15269937 | -0.3549393 | 0.72263796 | 0.999976173 | -0.054199006 |
| YKL040C | NFU1 | 0.15269937 | -0.3546482 | 0.72285608 | 0.999976173 | -0.054154548 |
| YGL165C | YGL165C | 0.15269937 | -0.3544553 | 0.72300056 | 0.999976173 | -0.054125104 |
| YKL043W | PHD1 | 0.15269937 | -0.35439 | 0.72304949 | 0.999976173 | -0.054115133 |
| YDR185C | UPS3 | 0.15269937 | -0.3542587 | 0.72314793 | 0.999976173 | -0.054095073 |
| YOR274W | MOD5 | 0.15269937 | -0.3540191 | 0.72332748 | 0.999976173 | -0.054058486 |
| YPR057W | BRR1 | 0.15269937 | -0.3539869 | 0.72335161 | 0.999976173 | -0.054053568 |
| YHR198C | AIM18 | 0.15269937 | -0.3527925 | 0.72424689 | 0.999976173 | -0.053871189 |
| YOL029C | YOL029C | 0.15269937 | -0.3524219 | 0.72452471 | 0.999976173 | -0.053814608 |
| YFL015C | YFL015C | 0.15269937 | -0.3519025 | 0.72491426 | 0.999976173 | -0.053735287 |
| YOR050C | YOR050C | 0.15269937 | -0.3516894 | 0.72507409 | 0.999976173 | -0.053702745 |
| YKL020C | SPT23 | 0.15269937 | -0.3514921 | 0.72522208 | 0.999976173 | -0.053672616 |
| YDR334W | SWR1 | 0.15269937 | -0.3513578 | 0.72532281 | 0.999976173 | -0.05365211 |
| YOL043C | NTG2 | 0.15269937 | -0.3508091 | 0.72573442 | 0.999976173 | -0.053568327 |
| YDL211C | YDL211C | 0.15269937 | -0.3503091 | 0.72610959 | 0.999976173 | -0.053491975 |
| YKL007W | CAP1 | 0.15269937 | -0.3496358 | 0.72661486 | 0.999976173 | -0.053389169 |
| YAR044W | NA | 0.15269937 | -0.348306 | 0.7276132 | 0.999976173 | -0.053186109 |
| YKL132C | RMA1 | 0.15269937 | -0.3479337 | 0.72789277 | 0.999976173 | -0.053129261 |

| | | | | | | |
|---|---|---|---|---|---|---|
| YPR115W | YPR115W | 0.15269937 | -0.3475894 | 0.72815139 | 0.999976173 | -0.053076682 |
| YGR202C | PCT1 | 0.15269937 | -0.3470547 | 0.72855308 | 0.999976173 | -0.052995026 |
| YMR202W | ERG2 | 0.15269937 | -0.3469128 | 0.72865968 | 0.999976173 | -0.052973359 |
| YHR153C | SPO16 | 0.15269937 | -0.3467943 | 0.72874866 | 0.999976173 | -0.052955274 |
| YLR444C | YLR444C | 0.15269937 | -0.3450752 | 0.73004061 | 0.999976173 | -0.05269277 |
| YMR319C | FET4 | 0.15269937 | -0.3447456 | 0.73028841 | 0.999976173 | -0.052642439 |
| YDR179C | CSN9 | 0.15269937 | -0.3445959 | 0.73040102 | 0.999976173 | -0.052619569 |
| YKR057W | RPS21A | 0.15269937 | -0.3439558 | 0.73088234 | 0.999976173 | -0.052521829 |
| YNR066C | YNR066C | 0.15269937 | -0.3436275 | 0.73112926 | 0.999976173 | -0.052471694 |
| YNL279W | PRM1 | 0.15269937 | -0.343436 | 0.73127327 | 0.999976173 | -0.05244246 |
| YBR069C | TAT1 | 0.15269937 | -0.3433934 | 0.73130531 | 0.999976173 | -0.052435955 |
| YPL187W | MF(ALPHA)1 | 0.15269937 | -0.3433602 | 0.73133031 | 0.999976173 | -0.052430879 |
| YER041W | YEN1 | 0.15269937 | -0.343214 | 0.73144028 | 0.999976173 | -0.052408556 |
| YDL137W | ARF2 | 0.15269937 | -0.3431658 | 0.73147651 | 0.999976173 | -0.052401201 |
| YBR007C | DSF2 | 0.15269937 | -0.3422831 | 0.73214064 | 0.999976173 | -0.052266411 |
| YDR294C | DPL1 | 0.15269937 | -0.3412711 | 0.73290224 | 0.999976173 | -0.052111887 |
| YLR407W | YLR407W | 0.15269937 | -0.3406846 | 0.73334381 | 0.999976173 | -0.052022319 |
| YGR182C | YGR182C | 0.15269937 | -0.3398919 | 0.73394069 | 0.999976173 | -0.051901279 |
| YGR023W | MTL1 | 0.15269937 | -0.3396705 | 0.73410746 | 0.999976173 | -0.051867465 |
| YJR036C | HUL4 | 0.15269937 | -0.3395564 | 0.73419338 | 0.999976173 | -0.051850045 |
| YGR015C | YGR015C | 0.15269937 | -0.3395102 | 0.73422816 | 0.999976173 | -0.051842993 |
| YMR114C | YMR114C | 0.15269937 | -0.3394694 | 0.73425893 | 0.999976173 | -0.051836756 |
| YER113C | TMN3 | 0.15269937 | -0.3388827 | 0.73470083 | 0.999976173 | -0.051747177 |
| YER121W | YER121W | 0.15269937 | -0.3385083 | 0.73498294 | 0.999976173 | -0.051689999 |
| YBL048W | RRT1 | 0.15269937 | -0.3382079 | 0.73520924 | 0.999976173 | -0.051644137 |
| YDR476C | YDR476C | 0.15269937 | -0.3376389 | 0.73563803 | 0.999976173 | -0.051557252 |
| YMR182C | RGM1 | 0.15269937 | -0.3371982 | 0.73597023 | 0.999976173 | -0.05148995 |
| YMR195W | ICY1 | 0.15269937 | -0.3362327 | 0.73669814 | 0.999976173 | -0.051342516 |
| YKL187C | YKL187C | 0.15269937 | -0.3360556 | 0.7368317 | 0.999976173 | -0.05131547 |
| YKL113C | RAD27 | 0.15269937 | -0.3350276 | 0.73760696 | 0.999976173 | -0.051158506 |
| YOR101W | RAS1 | 0.15269937 | -0.3346922 | 0.73786001 | 0.999976173 | -0.051107284 |
| YMR170C | ALD2 | 0.15269937 | -0.3343278 | 0.73813493 | 0.999976173 | -0.051051641 |
| YNL012W | SPO1 | 0.15269937 | -0.3332676 | 0.73893502 | 0.999976173 | -0.050889745 |
| YPR063C | YPR063C | 0.15269937 | -0.333084 | 0.73907357 | 0.999976173 | -0.050861715 |
| YHR154W | RTT107 | 0.15269937 | -0.3325215 | 0.73949819 | 0.999976173 | -0.050775825 |
| YMR158C-B | NA | 0.15269937 | -0.3324083 | 0.73958368 | 0.999976173 | -0.050758533 |
| YNL173C | MDG1 | 0.15269937 | -0.3315502 | 0.74023162 | 0.999976173 | -0.050627504 |
| YJL036W | SNX4 | 0.15269937 | -0.3310745 | 0.74059086 | 0.999976173 | -0.050554872 |
| YJR127C | RSF2 | 0.15269937 | -0.3307879 | 0.74080741 | 0.999976173 | -0.050511096 |
| YGR054W | YGR054W | 0.15269937 | -0.3301406 | 0.74129641 | 0.999976173 | -0.050412258 |
| YHR073W | OSH3 | 0.15269937 | -0.3301115 | 0.74131838 | 0.999976173 | -0.050407817 |
| YGR118W | RPS23A | 0.15269937 | -0.3299481 | 0.74144181 | 0.999976173 | -0.050382873 |
| YDR497C | ITR1 | 0.15269937 | -0.3298378 | 0.74152521 | 0.999976173 | -0.050366019 |
| YOR167C | RPS28A | 0.15269937 | -0.3291428 | 0.74205039 | 0.999976173 | -0.050259902 |
| YHR132W-A | YHR132W-A | 0.15269937 | -0.3286699 | 0.74240785 | 0.999976173 | -0.050187688 |
| YMR144W | YMR144W | 0.15269937 | -0.328534 | 0.74251058 | 0.999976173 | -0.050166936 |
| YLR442C | SIR3 | 0.15269937 | -0.3276611 | 0.74317054 | 0.999976173 | -0.050033647 |
| YJL190C | RPS22A | 0.15269937 | -0.3275854 | 0.74322781 | 0.999976173 | -0.050022082 |
| YNL024C | YNL024C | 0.15269937 | -0.3274276 | 0.74334713 | 0.999976173 | -0.049997988 |
| YJL218W | YJL218W | 0.15269937 | -0.3272564 | 0.74347659 | 0.999976173 | -0.049971848 |
| YOL048C | RRT8 | 0.15269937 | -0.3270856 | 0.74360576 | 0.999976173 | -0.049945769 |
| YLL057C | JLP1 | 0.15269937 | -0.3264914 | 0.74405524 | 0.999976173 | -0.049855028 |
| YDL006W | PTC1 | 0.15269937 | -0.3256797 | 0.74466936 | 0.999976173 | -0.049731079 |
| YGR001C | YGR001C | 0.15269937 | -0.3251582 | 0.74506399 | 0.999976173 | -0.049651446 |
| YGL021W | ALK1 | 0.15269937 | -0.3242981 | 0.745715 | 0.999976173 | -0.04952011 |
| YHR079C | IRE1 | 0.15269937 | -0.3239375 | 0.74598797 | 0.999976173 | -0.04946505 |
| YJR040W | GEF1 | 0.15269937 | -0.3237334 | 0.74614253 | 0.999976173 | -0.049433878 |
| YPR045C | YPR045C | 0.15269937 | -0.323647 | 0.74620795 | 0.999976173 | -0.049420685 |
| YBR281C | DUG2 | 0.15269937 | -0.3233405 | 0.74644003 | 0.999976173 | -0.049373882 |
| YFR024C-A | LSB3 | 0.15269937 | -0.3231181 | 0.74660843 | 0.999976173 | -0.049339926 |
| YOR053W | YOR053W | 0.15269937 | -0.3229992 | 0.74669844 | 0.999976173 | -0.049321777 |
| YGL042C | YGL042C | 0.15269937 | -0.3226241 | 0.74698257 | 0.999976173 | -0.04926449 |
| YDL230W | PTP1 | 0.15269937 | -0.3204317 | 0.74864365 | 0.999976173 | -0.048929723 |
| YLR235C | YLR235C | 0.15269937 | -0.3201315 | 0.74887123 | 0.999976173 | -0.048883877 |

| | | | | | | |
|---|---|---|---|---|---|---|
| YDL174C | DLD1 | 0.15269937 | -0.3198487 | 0.74908559 | 0.999976173 | -0.048840697 |
| YLR265C | NEJ1 | 0.15269937 | -0.3188757 | 0.74982335 | 0.999976173 | -0.048692116 |
| YLR296W | YLR296W | 0.15269937 | -0.3186647 | 0.74998337 | 0.999976173 | -0.048659895 |
| YMR106C | 34 | 0.15269937 | -0.3186135 | 0.75002222 | 0.999976173 | -0.048652073 |
| YGR131W | YGR131W | 0.15269937 | -0.3185215 | 0.75009194 | 0.999976173 | -0.048638033 |
| YJL065C | DLS1 | 0.15269937 | -0.3177004 | 0.75071474 | 0.999976173 | -0.048512655 |
| YER010C | YER010C | 0.15269937 | -0.3174694 | 0.75089004 | 0.999976173 | -0.04847737 |
| YLR363C | NMD4 | 0.15269937 | -0.3172658 | 0.75104451 | 0.999976173 | -0.048446282 |
| YLL002W | RTT109 | 0.15269937 | -0.3169978 | 0.75124785 | 0.999976173 | -0.048405359 |
| YBR221C | PDB1 | 0.15269937 | -0.3168253 | 0.7513787 | 0.999976173 | -0.048379027 |
| YOL115W | PAP2 | 0.15269937 | -0.3164286 | 0.75167974 | 0.999976173 | -0.048318451 |
| YBR204C | YBR204C | 0.15269937 | -0.3161979 | 0.75185488 | 0.999976173 | -0.048283212 |
| YBR199W | KTR4 | 0.15269937 | -0.3161461 | 0.75189413 | 0.999976173 | -0.048275316 |
| YCR105W | ADH7 | 0.15269937 | -0.3160415 | 0.75197356 | 0.999976173 | -0.048259336 |
| YDR116C | MRPL1 | 0.15269937 | -0.315627 | 0.75228822 | 0.999976173 | -0.048196035 |
| YIL085C | KTR7 | 0.15269937 | -0.3147299 | 0.75296931 | 0.999976173 | -0.048059049 |
| YLR388W | RPS29A | 0.15269937 | -0.3137305 | 0.75372829 | 0.999976173 | -0.047906443 |
| YHR012W | VPS29 | 0.15269937 | -0.3132586 | 0.75408676 | 0.999976173 | -0.047834382 |
| YIL101C | XBP1 | 0.15269937 | -0.3130703 | 0.75422979 | 0.999976173 | -0.047805632 |
| YOR285W | YOR285W | 0.15269937 | -0.3126689 | 0.75453474 | 0.999976173 | -0.047744344 |
| YDR131C | YDR131C | 0.15269937 | -0.3124375 | 0.75471058 | 0.999976173 | -0.047709006 |
| YGL124C | MON1 | 0.15269937 | -0.3124325 | 0.75471436 | 0.999976173 | -0.047708247 |
| YDR279W | RNH202 | 0.15269937 | -0.3117369 | 0.75524299 | 0.999976173 | -0.047602028 |
| YBR218C | PYC2 | 0.15269937 | -0.3113609 | 0.7555288 | 0.999976173 | -0.047544608 |
| YDL124W | YDL124W | 0.15269937 | -0.3113227 | 0.7555578 | 0.999976173 | -0.047538782 |
| YBR005W | RCR1 | 0.15269937 | -0.3104481 | 0.75622269 | 0.999976173 | -0.047405233 |
| YGR164W | YGR164W | 0.15269937 | -0.3104314 | 0.75623545 | 0.999976173 | -0.047402672 |
| YLR228C | ECM22 | 0.15269937 | -0.31033 | 0.75631255 | 0.999976173 | -0.047387187 |
| YIL173W | VTH1 | 0.15269937 | -0.3102662 | 0.75636104 | 0.999976173 | -0.04737745 |
| YMR280C | CAT8 | 0.15269937 | -0.3100543 | 0.75652215 | 0.999976173 | -0.047345098 |
| YEL066W | HPA3 | 0.15269937 | -0.3099512 | 0.75660057 | 0.999976173 | -0.047329349 |
| YDR056C | YDR056C | 0.15269937 | -0.3099243 | 0.75662105 | 0.999976173 | -0.047325237 |
| YLR199C | PBA1 | 0.15269937 | -0.308634 | 0.75760245 | 0.999976173 | -0.047128213 |
| YJR074W | MOG1 | 0.15269937 | -0.3085705 | 0.75765076 | 0.999976173 | -0.047118518 |
| YPR179C | HDA3 | 0.15269937 | -0.3084198 | 0.75776537 | 0.999976173 | -0.047095513 |
| YOL031C | SIL1 | 0.15269937 | -0.3074478 | 0.75850499 | 0.999976173 | -0.046947091 |
| YOL001W | PHO80 | 0.15269937 | -0.3070506 | 0.75880731 | 0.999976173 | -0.046886436 |
| YMR105C | PGM2 | 0.15269937 | -0.3064585 | 0.75925804 | 0.999976173 | -0.046796019 |
| YBR134W | YBR134W | 0.15269937 | -0.3061655 | 0.75948109 | 0.999976173 | -0.04675128 |
| YLR401C | DUS3 | 0.15269937 | -0.3057524 | 0.75979562 | 0.999976173 | -0.046688199 |
| YAL020C | ATS1 | 0.15269937 | -0.3053945 | 0.7600682 | 0.999976173 | -0.046633539 |
| YIR034C | LYS1 | 0.15269937 | -0.3043491 | 0.76086441 | 0.999976173 | -0.046473911 |
| YIR004W | DJP1 | 0.15269937 | -0.303705 | 0.76135509 | 0.999976173 | -0.046375561 |
| YMR119W-A | YMR119W-A | 0.15269937 | -0.3026154 | 0.76218539 | 0.999976173 | -0.046209182 |
| YNL217W | YNL217W | 0.15269937 | -0.3022534 | 0.76246131 | 0.999976173 | -0.046153905 |
| YDR139C | RUB1 | 0.15269937 | -0.3020945 | 0.76258248 | 0.999976173 | -0.046129632 |
| YGL236C | MTO1 | 0.15269937 | -0.3014722 | 0.76305684 | 0.999976173 | -0.04603462 |
| YJR119C | JHD2 | 0.15269937 | -0.3011722 | 0.76328559 | 0.999976173 | -0.045988806 |
| YOR137C | SIA1 | 0.15269937 | -0.2998991 | 0.7642565 | 0.999976173 | -0.045794408 |
| YOR006C | TSR3 | 0.15269937 | -0.2997469 | 0.76437262 | 0.999976173 | -0.045771162 |
| YFL041W | FET5 | 0.15269937 | -0.2994596 | 0.7645918 | 0.999976173 | -0.045727289 |
| YAR037W | NA | 0.15269937 | -0.2988222 | 0.7650781 | 0.999976173 | -0.045629963 |
| YGR044C | RME1 | 0.15269937 | -0.2982041 | 0.76554978 | 0.999976173 | -0.045535579 |
| YNL329C | PEX6 | 0.15269937 | -0.2981776 | 0.76557 | 0.999976173 | -0.045531532 |
| YMR104C | YPK2 | 0.15269937 | -0.2981439 | 0.76559572 | 0.999976173 | -0.045526387 |
| YDR154C | YDR154C | 0.15269937 | -0.2971184 | 0.76637853 | 0.999976173 | -0.045369785 |
| YNR064C | YNR064C | 0.15269937 | -0.2967251 | 0.76667878 | 0.999976173 | -0.045309734 |
| YGL109W | YGL109W | 0.15269937 | -0.2961735 | 0.76709999 | 0.999976173 | -0.045225499 |
| YDL236W | PHO13 | 0.15269937 | -0.296021 | 0.7672164 | 0.999976173 | -0.045202222 |
| YMR086W | YMR086W | 0.15269937 | -0.2955331 | 0.76758905 | 0.999976173 | -0.045127715 |
| YJR015W | YJR015W | 0.15269937 | -0.2949979 | 0.76799785 | 0.999976173 | -0.045045992 |
| YOR013W | IRC11 | 0.15269937 | -0.2939983 | 0.76876152 | 0.999976173 | -0.044893361 |
| YJR054W | YJR054W | 0.15269937 | -0.29328 | 0.7693105 | 0.999976173 | -0.044783669 |
| YGL039W | YGL039W | 0.15269937 | -0.2929848 | 0.76953612 | 0.999976173 | -0.044738592 |

| | | | | | | |
|---|---|---|---|---|---|---|
| YML118W | NGL3 | 0.15269937 | -0.2925538 | 0.76986558 | 0.999976173 | -0.044672779 |
| YIR024C | YIR024C | 0.15269937 | -0.2921249 | 0.77019347 | 0.999976173 | -0.044607286 |
| YBR072W | HSP26 | 0.15269937 | -0.2907734 | 0.77122698 | 0.999976173 | -0.04440091 |
| YHR139C | SPS100 | 0.15269937 | -0.2904502 | 0.77147417 | 0.999976173 | -0.044351562 |
| YNL176C | YNL176C | 0.15269937 | -0.2898786 | 0.77191146 | 0.999976173 | -0.044264272 |
| YER167W | BCK2 | 0.15269937 | -0.2892029 | 0.77242845 | 0.999976173 | -0.044161095 |
| YBL096C | YBL096C | 0.15269937 | -0.2891327 | 0.77248217 | 0.999976173 | -0.044150375 |
| YDR385W | EFT2 | 0.15269937 | -0.2881453 | 0.77323781 | 0.999976173 | -0.043999607 |
| YLR063W | YLR063W | 0.15269937 | -0.2863447 | 0.77461638 | 0.999976173 | -0.043724661 |
| YER059W | PCL6 | 0.15269937 | -0.2860862 | 0.77481436 | 0.999976173 | -0.043685187 |
| YOR289W | YOR289W | 0.15269937 | -0.2859742 | 0.77490018 | 0.999976173 | -0.043668076 |
| YAL051W | OAF1 | 0.15269937 | -0.2856186 | 0.77517251 | 0.999976173 | -0.043613785 |
| YBR184W | YBR184W | 0.15269937 | -0.2854208 | 0.77532408 | 0.999976173 | -0.04358357 |
| YBR015C | MNN2 | 0.15269937 | -0.285011 | 0.77563797 | 0.999976173 | -0.043521004 |
| YDR421W | ARO80 | 0.15269937 | -0.2849862 | 0.77565698 | 0.999976173 | -0.043517214 |
| YOL124C | TRM11 | 0.15269937 | -0.2849538 | 0.77568182 | 0.999976173 | -0.043512264 |
| YDL050C | YDL050C | 0.15269937 | -0.2838044 | 0.77656252 | 0.999976173 | -0.043336758 |
| YCL063W | VAC17 | 0.15269937 | -0.2836231 | 0.77670147 | 0.999976173 | -0.043309073 |
| YOR347C | PYK2 | 0.15269937 | -0.2827566 | 0.77736567 | 0.999976173 | -0.043176756 |
| YOR307C | SLY41 | 0.15269937 | -0.2825363 | 0.7775346 | 0.999976173 | -0.043143108 |
| YLR144C | ACF2 | 0.15269937 | -0.2823819 | 0.77765298 | 0.999976173 | -0.043119532 |
| YJR092W | BUD4 | 0.15269937 | -0.2820843 | 0.77788112 | 0.999976173 | -0.043074095 |
| YPR195C | YPR195C | 0.15269937 | -0.2816352 | 0.77822547 | 0.999976173 | -0.043005522 |
| YDL001W | RMD1 | 0.15269937 | -0.2814497 | 0.77836775 | 0.999976173 | -0.042977191 |
| YAL043C-A | NA | 0.15269937 | -0.2813883 | 0.77841487 | 0.999976173 | -0.042967809 |
| YDR459C | PFA5 | 0.15269937 | -0.2813263 | 0.7784624 | 0.999976173 | -0.042958345 |
| YDR150W | NUM1 | 0.15269937 | -0.2799595 | 0.7795108 | 0.999976173 | -0.04274964 |
| YLR112W | YLR112W | 0.15269937 | -0.2792815 | 0.78003107 | 0.999976173 | -0.042646101 |
| YER123W | YCK3 | 0.15269937 | -0.2787247 | 0.78045831 | 0.999976173 | -0.042561089 |
| YBR036C | CSG2 | 0.15269937 | -0.2783761 | 0.78072591 | 0.999976173 | -0.042507849 |
| YPR001W | CIT3 | 0.15269937 | -0.2782453 | 0.78082632 | 0.999976173 | -0.042487874 |
| YOL099C | YOL099C | 0.15269937 | -0.2777693 | 0.78119165 | 0.999976173 | -0.042415202 |
| YPL144W | POC4 | 0.15269937 | -0.2771133 | 0.78169531 | 0.999976173 | -0.042315028 |
| YOR066W | MSA1 | 0.15269937 | -0.2770782 | 0.78172227 | 0.999976173 | -0.042309665 |
| YOR299W | BUD7 | 0.15269937 | -0.2769159 | 0.78184689 | 0.999976173 | -0.042284883 |
| YOL108C | INO4 | 0.15269937 | -0.2768974 | 0.7818611 | 0.999976173 | -0.042282058 |
| YOR097C | YOR097C | 0.15269937 | -0.276889 | 0.78186752 | 0.999976173 | -0.04228078 |
| YDR004W | RAD57 | 0.15269937 | -0.2768733 | 0.78187959 | 0.999976173 | -0.042278379 |
| YFL013W-A | YFL013W-A | 0.15269937 | -0.2760596 | 0.78250453 | 0.999976173 | -0.042154119 |
| YOL065C | INP54 | 0.15269937 | -0.2756851 | 0.78279215 | 0.999976173 | -0.042096937 |
| YKL048C | ELM1 | 0.15269937 | -0.2756831 | 0.78279371 | 0.999976173 | -0.042096628 |
| YLR028C | ADE16 | 0.15269937 | -0.2745278 | 0.7836812 | 0.999976173 | -0.041920228 |
| YMR206W | YMR206W | 0.15269937 | -0.2739422 | 0.78413119 | 0.999976173 | -0.041830807 |
| YNL090W | RHO2 | 0.15269937 | -0.273608 | 0.78438806 | 0.999976173 | -0.041779769 |
| YBR212W | NGR1 | 0.15269937 | -0.27248 | 0.78525515 | 0.999976173 | -0.04160752 |
| YBR016W | YBR016W | 0.15269937 | -0.2722456 | 0.78543537 | 0.999976173 | -0.041571727 |
| YDL215C | GDH2 | 0.15269937 | -0.2722213 | 0.78545406 | 0.999976173 | -0.041568014 |
| YBL091C-A | SCS22 | 0.15269937 | -0.2707809 | 0.78656168 | 0.999976173 | -0.041348077 |
| YGR137W | YGR137W | 0.15269937 | -0.2703672 | 0.7868799 | 0.999976173 | -0.041284904 |
| YPL138C | SPP1 | 0.15269937 | -0.2702197 | 0.78699342 | 0.999976173 | -0.04126237 |
| YKL006W | RPL14A | 0.15269937 | -0.2689967 | 0.78793434 | 0.999976173 | -0.041075629 |
| YGL127C | SOH1 | 0.15269937 | -0.268822 | 0.78806878 | 0.999976173 | -0.041048953 |
| YOR132W | VPS17 | 0.15269937 | -0.2682608 | 0.78850071 | 0.999976173 | -0.040963255 |
| YJL057C | IKS1 | 0.15269937 | -0.2678192 | 0.78884064 | 0.999976173 | -0.040895819 |
| YNL051W | COG5 | 0.15269937 | -0.2677551 | 0.78888996 | 0.999976173 | -0.040886034 |
| YDR424C | DYN2 | 0.15269937 | -0.2677098 | 0.78892484 | 0.999976173 | -0.040879117 |
| YGR235C | YGR235C | 0.15269937 | -0.2674043 | 0.78916001 | 0.999976173 | -0.040832469 |
| YMR056C | AAC1 | 0.15269937 | -0.2670053 | 0.7894672 | 0.999976173 | -0.040771542 |
| YJR014W | TMA22 | 0.15269937 | -0.2662612 | 0.79004021 | 0.999976173 | -0.04065791 |
| YHR160C | PEX18 | 0.15269937 | -0.2661668 | 0.79011287 | 0.999976173 | -0.040643501 |
| YGL060W | YBP2 | 0.15269937 | -0.2657043 | 0.79046906 | 0.999976173 | -0.04057288 |
| YNL249C | MPA43 | 0.15269937 | -0.2657001 | 0.79047227 | 0.999976173 | -0.040572242 |
| YPR046W | MCM16 | 0.15269937 | -0.2650123 | 0.7910021 | 0.999976173 | -0.04046721 |
| YDL136W | RPL35B | 0.15269937 | -0.2645872 | 0.79132959 | 0.999976173 | -0.040402298 |

| | | | | | | |
|---|---|---|---|---|---|---|
| YGL050W | TYW3 | 0.15269937 | -0.2645476 | 0.7913601 | 0.999976173 | -0.040396249 |
| YEL012W | UBC8 | 0.15269937 | -0.2644715 | 0.79141873 | 0.999976173 | -0.04038463 |
| YNL254C | RTC4 | 0.15269937 | -0.2633294 | 0.79229877 | 0.999976173 | -0.040210238 |
| YNL123W | NMA111 | 0.15269937 | -0.2632245 | 0.79237961 | 0.999976173 | -0.04019422 |
| YBR219C | YBR219C | 0.15269937 | -0.2626199 | 0.79284569 | 0.999976173 | -0.040101885 |
| YOR021C | YOR021C | 0.15269937 | -0.2625926 | 0.79286669 | 0.999976173 | -0.040097724 |
| YGL136C | MRM2 | 0.15269937 | -0.2622323 | 0.79314441 | 0.999976173 | -0.040042712 |
| YMR158W-A | NA | 0.15269937 | -0.2622086 | 0.79316268 | 0.999976173 | -0.040039093 |
| YHR046C | INM1 | 0.15269937 | -0.2620906 | 0.79325369 | 0.999976173 | -0.040021066 |
| YDR046C | BAP3 | 0.15269937 | -0.2609814 | 0.79410895 | 0.999976173 | -0.039851689 |
| YIL138C | TPM2 | 0.15269937 | -0.2608808 | 0.79418648 | 0.999976173 | -0.039836338 |
| YKL167C | MRP49 | 0.15269937 | -0.2607512 | 0.79428647 | 0.999976173 | -0.039816539 |
| YKL160W | ELF1 | 0.15269937 | -0.2603758 | 0.79457596 | 0.999976173 | -0.039759223 |
| YNL003C | PET8 | 0.15269937 | -0.2596614 | 0.79512707 | 0.999976173 | -0.039650125 |
| YMR133W | REC114 | 0.15269937 | -0.2594995 | 0.79525196 | 0.999976173 | -0.039625402 |
| YGR143W | SKN1 | 0.15269937 | -0.2593324 | 0.79538087 | 0.999976173 | -0.039599888 |
| YKR011C | YKR011C | 0.15269937 | -0.2585056 | 0.79601878 | 0.999976173 | -0.039473642 |
| YDL157C | YDL157C | 0.15269937 | -0.2581221 | 0.79631472 | 0.999976173 | -0.039415082 |
| YLR374C | YLR374C | 0.15269937 | -0.2580509 | 0.79636965 | 0.999976173 | -0.039404214 |
| YLR269C | YLR269C | 0.15269937 | -0.2577693 | 0.79658697 | 0.999976173 | -0.039361215 |
| YGR139W | YGR139W | 0.15269937 | -0.2566255 | 0.79746996 | 0.999976173 | -0.039186545 |
| YPR089W | YPR089W | 0.15269937 | -0.2565251 | 0.79754748 | 0.999976173 | -0.039171213 |
| YBR207W | FTH1 | 0.15269937 | -0.2564279 | 0.79762249 | 0.999976173 | -0.039156377 |
| YIR031C | DAL7 | 0.15269937 | -0.2561186 | 0.7978613 | 0.999976173 | -0.039109146 |
| YNL285W | YNL285W | 0.15269937 | -0.2560154 | 0.79794094 | 0.999976173 | -0.039093396 |
| YDR315C | IPK1 | 0.15269937 | -0.2547042 | 0.79895359 | 0.999976173 | -0.038893171 |
| YJL216C | YJL216C | 0.15269937 | -0.2544439 | 0.79915465 | 0.999976173 | -0.038853424 |
| YHR143W | DSE2 | 0.15269937 | -0.2531816 | 0.80012988 | 0.999976173 | -0.038660672 |
| YKL085W | MDH1 | 0.15269937 | -0.2526448 | 0.80054473 | 0.999976173 | -0.038578697 |
| YHR096C | HXT5 | 0.15269937 | -0.2516573 | 0.80130797 | 0.999976173 | -0.038427908 |
| YDR482C | CWC21 | 0.15269937 | -0.2513981 | 0.80150833 | 0.999976173 | -0.038388331 |
| YAL010C | MDM10 | 0.15269937 | -0.2513335 | 0.80155828 | 0.999976173 | -0.038378463 |
| YIL124W | AYR1 | 0.15269937 | -0.2500081 | 0.80258308 | 0.999976173 | -0.038176075 |
| YCL046W | YCL046W | 0.15269937 | -0.2495852 | 0.80291014 | 0.999976173 | -0.038111498 |
| YMR012W | CLU1 | 0.15269937 | -0.2494327 | 0.8030281 | 0.999976173 | -0.03808821 |
| YGR061C | ADE6 | 0.15269937 | -0.2491241 | 0.80326679 | 0.999976173 | -0.038041087 |
| YBR031W | RPL4A | 0.15269937 | -0.2485225 | 0.80373213 | 0.999976173 | -0.03794923 |
| YDR068W | DOS2 | 0.15269937 | -0.2476438 | 0.80441199 | 0.999976173 | -0.03781505 |
| YLR428C | YLR428C | 0.15269937 | -0.2473831 | 0.8046137 | 0.999976173 | -0.037775244 |
| YHR066W | SSF1 | 0.15269937 | -0.2472072 | 0.80474984 | 0.999976173 | -0.037748381 |
| YAR015W | ADE1 | 0.15269937 | -0.2465423 | 0.80526445 | 0.999976173 | -0.037646847 |
| YNL198C | YNL198C | 0.15269937 | -0.2464477 | 0.80533765 | 0.999976173 | -0.037632406 |
| YIL095W | PRK1 | 0.15269937 | -0.2463399 | 0.80542105 | 0.999976173 | -0.037615952 |
| YER065C | ICL1 | 0.15269937 | -0.2446872 | 0.80670056 | 0.999976173 | -0.037363584 |
| YNL191W | DUG3 | 0.15269937 | -0.2440522 | 0.80719235 | 0.999976173 | -0.037266612 |
| YPL192C | PRM3 | 0.15269937 | -0.243725 | 0.80744575 | 0.999976173 | -0.037216651 |
| YGL032C | AGA2 | 0.15269937 | -0.2435627 | 0.80757147 | 0.999976173 | -0.037191865 |
| YER188W | YER188W | 0.15269937 | -0.2432225 | 0.80783499 | 0.999976173 | -0.037139917 |
| YFR047C | BNA6 | 0.15269937 | -0.242596 | 0.8083203 | 0.999976173 | -0.037044256 |
| YDR479C | PEX29 | 0.15269937 | -0.242525 | 0.80837529 | 0.999976173 | -0.037033417 |
| YBR181C | RPS6B | 0.15269937 | -0.2420181 | 0.80876806 | 0.999976173 | -0.036956008 |
| YML095C | RAD10 | 0.15269937 | -0.2417386 | 0.80898466 | 0.999976173 | -0.036913326 |
| YPL008W | CHL1 | 0.15269937 | -0.2407376 | 0.80976041 | 0.999976173 | -0.036760477 |
| YPL270W | MDL2 | 0.15269937 | -0.2404884 | 0.80995354 | 0.999976173 | -0.03672243 |
| YDR095C | YDR095C | 0.15269937 | -0.2403069 | 0.81009427 | 0.999976173 | -0.036694707 |
| YJR020W | YJR020W | 0.15269937 | -0.2402877 | 0.81010911 | 0.999976173 | -0.036691783 |
| YLL053C | YLL053C | 0.15269937 | -0.2399311 | 0.81038554 | 0.999976173 | -0.036637332 |
| YLR056W | ERG3 | 0.15269937 | -0.2397002 | 0.8105646 | 0.999976173 | -0.036602064 |
| YBR061C | TRM7 | 0.10797476 | -0.3385851 | 0.73492506 | 0.999976173 | -0.036558642 |
| YLR365W | YLR365W | 0.15269937 | -0.2390237 | 0.81108909 | 0.999976173 | -0.036498768 |
| YOL132W | GAS4 | 0.15269937 | -0.236245 | 0.81324446 | 0.999976173 | -0.036074456 |
| YMR224C | 11 | 0.15269937 | -0.2361604 | 0.81331007 | 0.999976173 | -0.036061544 |
| YPL096W | PNG1 | 0.15269937 | -0.2348091 | 0.81435877 | 0.999976173 | -0.035855198 |
| YIL037C | PRM2 | 0.15269937 | -0.2344314 | 0.81465192 | 0.999976173 | -0.03579753 |

| | | | | | | |
|---|---|---|---|---|---|---|
| YMR115W | MGR3 | 0.15269937 | -0.2338238 | 0.81512365 | 0.999976173 | -0.035704741 |
| YOR247W | SRL1 | 0.15269937 | -0.2334055 | 0.81544841 | 0.999976173 | -0.035640867 |
| YBR277C | YBR277C | 0.15269937 | -0.2333717 | 0.81547461 | 0.999976173 | -0.035635714 |
| YGR247W | CPD1 | 0.15269937 | -0.2333023 | 0.81552854 | 0.999976173 | -0.035625109 |
| YLR216C | CPR6 | 0.15269937 | -0.233254 | 0.81556598 | 0.999976173 | -0.035617745 |
| YLR024C | UBR2 | 0.15269937 | -0.2320777 | 0.81647948 | 0.999976173 | -0.035438122 |
| YBR041W | FAT1 | 0.15269937 | -0.2316729 | 0.81679393 | 0.999976173 | -0.035376302 |
| YNL022C | YNL022C | 0.15269937 | -0.2316047 | 0.81684688 | 0.999976173 | -0.035365891 |
| YLR257W | YLR257W | 0.15269937 | -0.2314568 | 0.8169618 | 0.999976173 | -0.035343301 |
| YOL085C | YOL085C | 0.15269937 | -0.2306659 | 0.81757615 | 0.999976173 | -0.035222544 |
| YBR223C | TDP1 | 0.15269937 | -0.2303348 | 0.81783343 | 0.999976173 | -0.03517198 |
| YKL158W | NA | 0.15269937 | -0.2297114 | 0.81831785 | 0.999976173 | -0.035076784 |
| YKR032W | YKR032W | 0.15269937 | -0.2295748 | 0.81842404 | 0.999976173 | -0.035055919 |
| YOL020W | TAT2 | 0.15269937 | -0.2295218 | 0.81846518 | 0.999976173 | -0.035047835 |
| YLR239C | LIP2 | 0.15269937 | -0.2291921 | 0.8187214 | 0.999976173 | -0.034997494 |
| YPL205C | YPL205C | 0.15269937 | -0.2285158 | 0.81924709 | 0.999976173 | -0.034894216 |
| YIL163C | YIL163C | 0.15269937 | -0.2280598 | 0.81960153 | 0.999976173 | -0.034824591 |
| YOR044W | IRC23 | 0.15269937 | -0.2278764 | 0.81974413 | 0.999976173 | -0.034796582 |
| YGR126W | YGR126W | 0.15269937 | -0.2276337 | 0.81993279 | 0.999976173 | -0.034759527 |
| YJL187C | SWE1 | 0.15269937 | -0.2276262 | 0.81993865 | 0.999976173 | -0.034758376 |
| YJL211C | YJL211C | 0.15269937 | -0.2275012 | 0.8200358 | 0.999976173 | -0.034739296 |
| YMR210W | YMR210W | 0.15269937 | -0.2273157 | 0.82018002 | 0.999976173 | -0.03471097 |
| YOL138C | RTC1 | 0.15269937 | -0.2272801 | 0.82020775 | 0.999976173 | -0.034705525 |
| YOR108W | LEU9 | 0.15269937 | -0.2271157 | 0.82033557 | 0.999976173 | -0.034680424 |
| YCR065W | HCM1 | 0.15269937 | -0.2262393 | 0.82101713 | 0.999976173 | -0.034546591 |
| YDR540C | IRC4 | 0.15269937 | -0.2251487 | 0.8218654 | 0.999976173 | -0.034380057 |
| YNR039C | ZRG17 | 0.15269937 | -0.2251054 | 0.82189904 | 0.999976173 | -0.034373455 |
| YOR196C | LIP5 | 0.15269937 | -0.224698 | 0.822216 | 0.999976173 | -0.034311241 |
| YGR069W | YGR069W | 0.15269937 | -0.2245543 | 0.82232776 | 0.999976173 | -0.034289306 |
| YFL025C | BST1 | 0.15269937 | -0.2243232 | 0.82250762 | 0.999976173 | -0.034254006 |
| YDR293C | SSD1 | 0.15269937 | -0.2240651 | 0.82270844 | 0.999976173 | -0.034214595 |
| YOL054W | PSH1 | 0.15269937 | -0.2240111 | 0.82275042 | 0.999976173 | -0.034206355 |
| YBR119W | MUD1 | 0.15269937 | -0.2239932 | 0.82276433 | 0.999976173 | -0.034203625 |
| YGR183C | QCR9 | 0.15269937 | -0.2231589 | 0.82341362 | 0.999976173 | -0.034076217 |
| YGL157W | YGL157W | 0.15269937 | -0.2230491 | 0.82349909 | 0.999976173 | -0.034059449 |
| YOR091W | TMA46 | 0.15269937 | -0.2229719 | 0.82355911 | 0.999976173 | -0.034047672 |
| YJL207C | LAA1 | 0.15269937 | -0.2224065 | 0.82399917 | 0.999976173 | -0.033961338 |
| YGL057C | GEP7 | 0.15269937 | -0.2223458 | 0.82404648 | 0.999976173 | -0.033952057 |
| YDR003W | RCR2 | 0.15269937 | -0.2222457 | 0.82412434 | 0.999976173 | -0.033936784 |
| YJR010W | MET3 | 0.15269937 | -0.222066 | 0.82426429 | 0.999976173 | -0.03390933 |
| YMR130W | YMR130W | 0.15269937 | -0.2219555 | 0.82435027 | 0.999976173 | -0.033892465 |
| YJR120W | YJR120W | 0.15269937 | -0.2215421 | 0.82467214 | 0.999976173 | -0.033829332 |
| YMR246W | FAA4 | 0.15269937 | -0.2213986 | 0.82478383 | 0.999976173 | -0.033807425 |
| YIR020C | YIR020C | 0.15269937 | -0.2211438 | 0.82498219 | 0.999976173 | -0.033768523 |
| YOR005C | DNL4 | 0.15269937 | -0.2211252 | 0.82499672 | 0.999976173 | -0.033765672 |
| YKL044W | YKL044W | 0.15269937 | -0.2198122 | 0.82601919 | 0.999976173 | -0.033565176 |
| YDR048C | YDR048C | 0.15269937 | -0.2195994 | 0.82618488 | 0.999976173 | -0.033532692 |
| YHR142W | CHS7 | 0.15269937 | -0.2194473 | 0.82630339 | 0.999976173 | -0.033509457 |
| YIL162W | SUC2 | 0.15269937 | -0.2194153 | 0.82632824 | 0.999976173 | -0.033504584 |
| YDR159W | SAC3 | 0.15269937 | -0.2192245 | 0.82647686 | 0.999976173 | -0.033475449 |
| YMR002W | MIC17 | 0.15269937 | -0.2189672 | 0.82667729 | 0.999976173 | -0.033436159 |
| YLL059C | YLL059C | 0.15269937 | -0.2170383 | 0.82818018 | 0.999976173 | -0.033141616 |
| YDR392W | SPT3 | 0.15269937 | -0.2169372 | 0.828259 | 0.999976173 | -0.033126173 |
| YBR210W | ERV15 | 0.15269937 | -0.2147539 | 0.82996087 | 0.999976173 | -0.032792788 |
| YAR027W | UIP3 | 0.15269937 | -0.2145409 | 0.83012697 | 0.999976173 | -0.032760259 |
| YOL129W | VPS68 | 0.15269937 | -0.2142929 | 0.83032037 | 0.999976173 | -0.032722385 |
| YJR047C | ANB1 | 0.15269937 | -0.2140204 | 0.83053285 | 0.999976173 | -0.032680775 |
| YGR130C | YGR130C | 0.15269937 | -0.2126802 | 0.8315781 | 0.999976173 | -0.032476129 |
| YER039C-A | YER039C-A | 0.15269937 | -0.212621 | 0.83162428 | 0.999976173 | -0.032467088 |
| YLR188W | MDL1 | 0.15269937 | -0.2125324 | 0.83169334 | 0.999976173 | -0.032453569 |
| YHR206W | SKN7 | 0.15269937 | -0.2125121 | 0.83170923 | 0.999976173 | -0.03245046 |
| YDR123C | INO2 | 0.15269937 | -0.212295 | 0.83187861 | 0.999976173 | -0.032417304 |
| YDR415C | YDR415C | 0.15269937 | -0.2118762 | 0.83220527 | 0.999976173 | -0.032353364 |
| YNR021W | YNR021W | 0.15269937 | -0.2118008 | 0.83226412 | 0.999976173 | -0.032341846 |

| | | | | | | |
|---|---|---|---|---|---|---|
| YLR174W | IDP2 | 0.15269937 | -0.2116528 | 0.83237959 | 0.999976173 | -0.032319246 |
| YBR267W | REI1 | 0.15269937 | -0.2115635 | 0.83244926 | 0.999976173 | -0.032305611 |
| YFL027C | GYP8 | 0.15269937 | -0.2101441 | 0.83355687 | 0.999976173 | -0.032088868 |
| YDR314C | RAD34 | 0.15269937 | -0.2100967 | 0.83359389 | 0.999976173 | -0.032081626 |
| YJL170C | ASG7 | 0.15269937 | -0.2099183 | 0.83373312 | 0.999976173 | -0.032054385 |
| YMR157C | AIM36 | 0.15269937 | -0.2092922 | 0.83422175 | 0.999976173 | -0.031958792 |
| YOR009W | TIR4 | 0.15269937 | -0.2091389 | 0.83434144 | 0.999976173 | -0.031935379 |
| YOR313C | SPS4 | 0.15269937 | -0.2086993 | 0.83468465 | 0.999976173 | -0.031868246 |
| YOR038C | HIR2 | 0.15269937 | -0.2084835 | 0.83485314 | 0.999976173 | -0.031835292 |
| YGL198W | YIP4 | 0.15269937 | -0.2084834 | 0.83485322 | 0.999976173 | -0.031835276 |
| YLR377C | FBP1 | 0.15269937 | -0.2083498 | 0.83495751 | 0.999976173 | -0.031814878 |
| YGL197W | MDS3 | 0.15269937 | -0.2080311 | 0.83520635 | 0.999976173 | -0.031766211 |
| YBR107C | IML3 | 0.15269937 | -0.2076197 | 0.83552757 | 0.999976173 | -0.031703393 |
| YDL197C | ASF2 | 0.15269937 | -0.2074379 | 0.83566953 | 0.999976173 | -0.031675632 |
| YFR024C | NA | 0.15269937 | -0.2074252 | 0.8356794 | 0.999976173 | -0.031673703 |
| YBL062W | YBL062W | 0.15269937 | -0.2069971 | 0.83601373 | 0.999976173 | -0.031608331 |
| YGL256W | ADH4 | 0.15269937 | -0.2068228 | 0.83614988 | 0.999976173 | -0.031581709 |
| YHR032W | YHR032W | 0.15269937 | -0.206723 | 0.83622782 | 0.999976173 | -0.031566471 |
| YFR021W | ATG18 | 0.15269937 | -0.2066448 | 0.83628892 | 0.999976173 | -0.031554525 |
| YNL070W | TOM7 | 0.15269937 | -0.2063795 | 0.83649612 | 0.999976173 | -0.031514015 |
| YFL001W | DEG1 | 0.15269937 | -0.2061239 | 0.83669578 | 0.999976173 | -0.031474983 |
| YGR148C | RPL24B | 0.15269937 | -0.2059459 | 0.83683483 | 0.999976173 | -0.031447801 |
| YDL061C | RPS29B | 0.15269937 | -0.2059243 | 0.8368517 | 0.999976173 | -0.031444503 |
| YHR018C | ARG4 | 0.15269937 | -0.2055966 | 0.83710765 | 0.999976173 | -0.03139447 |
| YPR017C | DSS4 | 0.15269937 | -0.2051419 | 0.83746287 | 0.999976173 | -0.031325039 |
| YJL058C | BIT61 | 0.15269937 | -0.2048224 | 0.83771253 | 0.999976173 | -0.031276243 |
| YOL079W | YOL079W | 0.15269937 | -0.2047835 | 0.83774288 | 0.999976173 | -0.031270312 |
| YIR013C | GAT4 | 0.15269937 | -0.2043032 | 0.8381182 | 0.999976173 | -0.031196963 |
| YDR382W | RPP2B | 0.15269937 | -0.2035489 | 0.83870766 | 0.999976173 | -0.031081782 |
| YDR120C | TRM1 | 0.15269937 | -0.2032867 | 0.83891252 | 0.999976173 | -0.031041755 |
| YKL066W | YKL066W | 0.15269937 | -0.2028081 | 0.83928659 | 0.999976173 | -0.030968675 |
| YLR070C | XYL2 | 0.15269937 | -0.2025756 | 0.83946838 | 0.999976173 | -0.030933161 |
| YGL006W | PMC1 | 0.15269937 | -0.2016086 | 0.84022432 | 0.999976173 | -0.030785503 |
| YDR199W | YDR199W | 0.15269937 | -0.2007508 | 0.84089503 | 0.999976173 | -0.030654516 |
| YER056C-A | RPL34A | 0.15269937 | -0.2003922 | 0.84117542 | 0.999976173 | -0.030599764 |
| YDR431W | YDR431W | 0.15269937 | -0.2001249 | 0.8413845 | 0.999976173 | -0.030558939 |
| YGL244W | RTF1 | 0.15269937 | -0.2000518 | 0.8414416 | 0.999976173 | -0.03054779 |
| YNR009W | NRM1 | 0.15269937 | -0.1999397 | 0.84152927 | 0.999976173 | -0.030530673 |
| YJR037W | YJR037W | 0.15269937 | -0.1998926 | 0.84156618 | 0.999976173 | -0.030523466 |
| YDR336W | YDR336W | 0.15269937 | -0.199652 | 0.84175436 | 0.999976173 | -0.030486727 |
| YDR282C | YDR282C | 0.15269937 | -0.1996317 | 0.84177018 | 0.999976173 | -0.030483638 |
| YBR246W | RRT2 | 0.15269937 | -0.199318 | 0.84201555 | 0.999976173 | -0.030435735 |
| YNL214W | PEX17 | 0.15269937 | -0.1992475 | 0.84207074 | 0.999976173 | -0.03042496 |
| YER124C | DSE1 | 0.15269937 | -0.1992236 | 0.84208939 | 0.999976173 | -0.030421319 |
| YER057C | HMF1 | 0.15269937 | -0.1988362 | 0.8423924 | 0.999976173 | -0.030362169 |
| YCR015C | YCR015C | 0.15269937 | -0.1985961 | 0.8425803 | 0.999976173 | -0.030325491 |
| YOR202W | HIS3 | 0.07634968 | -0.3945759 | 0.69315913 | 0.999976173 | -0.030125747 |
| YMR040W | YET2 | 0.15269937 | -0.1970501 | 0.84378987 | 0.999976173 | -0.030089426 |
| YOR022C | YOR022C | 0.15269937 | -0.1968586 | 0.84393971 | 0.999976173 | -0.030060188 |
| YDR032C | PST2 | 0.15269937 | -0.1967932 | 0.84399095 | 0.999976173 | -0.03005019 |
| YOL114C | YOL114C | 0.15269937 | -0.1961166 | 0.84452047 | 0.999976173 | -0.029946875 |
| YKL061W | YKL061W | 0.15269937 | -0.1955107 | 0.8449947 | 0.999976173 | -0.029854359 |
| YGL230C | YGL230C | 0.15269937 | -0.1953191 | 0.84514471 | 0.999976173 | -0.029825096 |
| YFL046W | FMP32 | 0.15269937 | -0.1951974 | 0.84523993 | 0.999976173 | -0.029806522 |
| YOR072W | YOR072W | 0.15269937 | -0.1951663 | 0.84526431 | 0.999976173 | -0.029801766 |
| YGR019W | UGA1 | 0.15269937 | -0.1949151 | 0.84546092 | 0.999976173 | -0.029763416 |
| YLR012C | YLR012C | 0.15269937 | -0.1943716 | 0.84588642 | 0.999976173 | -0.029680426 |
| YER007C-A | TMA20 | 0.15269937 | -0.1935574 | 0.84652401 | 0.999976173 | -0.029556086 |
| YNR024W | MPP6 | 0.15269937 | -0.1930564 | 0.84691634 | 0.999976173 | -0.029479584 |
| YPL197C | YPL197C | 0.15269937 | -0.1930284 | 0.84693824 | 0.999976173 | -0.029475315 |
| YKL025C | PAN3 | 0.15269937 | -0.1924556 | 0.84738683 | 0.999976173 | -0.029387852 |
| YOR269W | PAC1 | 0.15269937 | -0.1921861 | 0.84759792 | 0.999976173 | -0.0293467 |
| YKR076W | ECM4 | 0.15269937 | -0.1915878 | 0.84806659 | 0.999976173 | -0.029255337 |
| YMR020W | FMS1 | 0.15269937 | -0.1900737 | 0.84925291 | 0.999976173 | -0.029024126 |

| | | | | | | |
|---|---|---|---|---|---|---|
| YDL041W | YDL041W | 0.15269937 | -0.1892513 | 0.84989731 | 0.999976173 | -0.028898561 |
| YAR002C-A | ERP1 | 0.15269937 | -0.1887133 | 0.85031903 | 0.999976173 | -0.028816397 |
| YER119C-A | YER119C-A | 0.15269937 | -0.1885478 | 0.85044874 | 0.999976173 | -0.028791127 |
| YMR030W | RSF1 | 0.15269937 | -0.1883475 | 0.85060576 | 0.999976173 | -0.028760538 |
| YMR041C | ARA2 | 0.15269937 | -0.1880529 | 0.85083665 | 0.999976173 | -0.02871556 |
| YCR087W | YCR087W | 0.15269937 | -0.1876376 | 0.85116223 | 0.999976173 | -0.02865214 |
| YKL069W | YKL069W | 0.15269937 | -0.1875196 | 0.85125472 | 0.999976173 | -0.028634125 |
| YNL273W | TOF1 | 0.15269937 | -0.1874031 | 0.85134606 | 0.999976173 | -0.028616335 |
| YLR320W | MMS22 | 0.15269937 | -0.1870087 | 0.85165529 | 0.999976173 | -0.028556108 |
| YDR011W | SNQ2 | 0.15269937 | -0.1859461 | 0.85248847 | 0.999976173 | -0.028393855 |
| YPR156C | TPO3 | 0.15269937 | -0.1856876 | 0.85269124 | 0.999976173 | -0.028354373 |
| YGR034W | RPL26B | 0.15269937 | -0.1851028 | 0.85314988 | 0.999976173 | -0.028265074 |
| YNL097C | PHO23 | 0.15269937 | -0.1848844 | 0.85332117 | 0.999976173 | -0.028231727 |
| YMR165C | PAH1 | 0.15269937 | -0.1844862 | 0.85363349 | 0.999976173 | -0.028170926 |
| YNL009W | IDP3 | 0.15269937 | -0.1843516 | 0.85373905 | 0.999976173 | -0.028150379 |
| YBL053W | YBL053W | 0.15269937 | -0.1839984 | 0.85401613 | 0.999976173 | -0.028096444 |
| YEL033W | MTC7 | 0.15269937 | -0.1835257 | 0.85438698 | 0.999976173 | -0.028024261 |
| YGR003W | CUL3 | 0.15269937 | -0.1832646 | 0.85459182 | 0.999976173 | -0.027984392 |
| YBL055C | YBL055C | 0.15269937 | -0.1831148 | 0.85470936 | 0.999976173 | -0.027961517 |
| YOR104W | PIN2 | 0.15269937 | -0.1827834 | 0.85496938 | 0.999976173 | -0.027910915 |
| YJR001W | AVT1 | 0.15269937 | -0.1819715 | 0.8556065 | 0.999976173 | -0.027786936 |
| YGR136W | LSB1 | 0.15269937 | -0.1818255 | 0.85572113 | 0.999976173 | -0.027764632 |
| YGL125W | MET13 | 0.15269937 | -0.1812331 | 0.85618603 | 0.999976173 | -0.02767418 |
| YDR234W | LYS4 | 0.15269937 | -0.1808676 | 0.85647294 | 0.999976173 | -0.027618364 |
| YHR140W | YHR140W | 0.15269937 | -0.1805138 | 0.85675065 | 0.999976173 | -0.02756434 |
| YER027C | GAL83 | 0.15269937 | -0.1799731 | 0.8571751 | 0.999976173 | -0.027481777 |
| YJR008W | YJR008W | 0.15269937 | -0.1799499 | 0.85719329 | 0.999976173 | -0.027478239 |
| YKR059W | TIF1 | 0.15269937 | -0.1794483 | 0.85758713 | 0.999976173 | -0.027401639 |
| YBR209W | YBR209W | 0.15269937 | -0.1793281 | 0.85768152 | 0.999976173 | -0.02738328 |
| YGR236C | SPG1 | 0.15269937 | -0.178457 | 0.8583655 | 0.999976173 | -0.027250269 |
| YLR176C | RFX1 | 0.15269937 | -0.1783928 | 0.85841589 | 0.999976173 | -0.027240469 |
| YOR045W | TOM6 | 0.15269937 | -0.1783216 | 0.85847179 | 0.999976173 | -0.0272296 |
| YGR049W | SCM4 | 0.15269937 | -0.1783138 | 0.85847793 | 0.999976173 | -0.027228405 |
| YBR050C | REG2 | 0.15269937 | -0.1783131 | 0.8584785 | 0.999976173 | -0.027228296 |
| YER066C-A | YER066C-A | 0.15269937 | -0.1776095 | 0.85903108 | 0.999976173 | -0.027120853 |
| YPL039W | YPL039W | 0.15269937 | -0.1771259 | 0.85941091 | 0.999976173 | -0.027047007 |
| YGL138C | YGL138C | 0.15269937 | -0.1770756 | 0.85945036 | 0.999976173 | -0.027039339 |
| YBR076W | ECM8 | 0.15269937 | -0.1766826 | 0.85975907 | 0.999976173 | -0.026979325 |
| YAR003W | SWD1 | 0.15269937 | -0.1758698 | 0.86039758 | 0.999976173 | -0.026855212 |
| YKL221W | MCH2 | 0.15269937 | -0.1758145 | 0.86044102 | 0.999976173 | -0.026846769 |
| YLR387C | REH1 | 0.15269937 | -0.1753109 | 0.86083671 | 0.999976173 | -0.026769864 |
| YOR219C | STE13 | 0.15269937 | -0.1751449 | 0.86096719 | 0.999976173 | -0.026744508 |
| YGR209C | TRX2 | 0.15269937 | -0.1747747 | 0.86125801 | 0.999976173 | -0.026687991 |
| YDR165W | TRM82 | 0.15269937 | -0.173711 | 0.86209393 | 0.999976173 | -0.026525562 |
| YOR226C | ISU2 | 0.15269937 | -0.1735693 | 0.86220533 | 0.999976173 | -0.026503919 |
| YDR073W | SNF11 | 0.15269937 | -0.173353 | 0.86237531 | 0.999976173 | -0.026470896 |
| YPR158W | CUR1 | 0.15269937 | -0.1726685 | 0.86291337 | 0.999976173 | -0.026366368 |
| YIL023C | YKE4 | 0.15269937 | -0.1724708 | 0.86306878 | 0.999976173 | -0.026336178 |
| YGL249W | ZIP2 | 0.15269937 | -0.1719617 | 0.86346901 | 0.999976173 | -0.026258438 |
| YGL179C | TOS3 | 0.15269937 | -0.1715446 | 0.86379691 | 0.999976173 | -0.02619475 |
| YDL123W | SNA4 | 0.15269937 | -0.1711003 | 0.86414625 | 0.999976173 | -0.026126904 |
| YGL218W | YGL218W | 0.15269937 | -0.1710532 | 0.86418324 | 0.999976173 | -0.026119722 |
| YLR040C | YLR040C | 0.15269937 | -0.1707772 | 0.86440032 | 0.999976173 | -0.026077565 |
| YOR141C | ARP8 | 0.15269937 | -0.1707502 | 0.86442149 | 0.999976173 | -0.026073454 |
| YGL248W | PDE1 | 0.15269937 | -0.1704761 | 0.86463703 | 0.999976173 | -0.026031598 |
| YMR297W | PRC1 | 0.15269937 | -0.1701708 | 0.86487714 | 0.999976173 | -0.025984974 |
| YER130C | YER130C | 0.15269937 | -0.1698288 | 0.86514609 | 0.999976173 | -0.025932753 |
| YBR108W | AIM3 | 0.15269937 | -0.1690168 | 0.86578471 | 0.999976173 | -0.025808765 |
| YPL196W | OXR1 | 0.15269937 | -0.1688441 | 0.86592063 | 0.999976173 | -0.02578238 |
| YNR014W | YNR014W | 0.15269937 | -0.1684706 | 0.8662144 | 0.999976173 | -0.025725352 |
| YLR279W | YLR279W | 0.15269937 | -0.1683684 | 0.86629474 | 0.999976173 | -0.025709755 |
| YKL146W | AVT3 | 0.15269937 | -0.1681555 | 0.86646228 | 0.999976173 | -0.025677234 |
| YNL087W | TCB2 | 0.15269937 | -0.1679255 | 0.86664317 | 0.999976173 | -0.025642123 |
| YBR045C | GIP1 | 0.15269937 | -0.1677554 | 0.86677699 | 0.999976173 | -0.025616149 |

| | | | | | | |
|---|---|---|---|---|---|---|
| YGR249W | MGA1 | 0.15269937 | -0.1675748 | 0.8669191 | 0.999976173 | -0.025588567 |
| YGL224C | SDT1 | 0.15269937 | -0.1675267 | 0.86695695 | 0.999976173 | -0.02558122 |
| YIL119C | RPI1 | 0.15269937 | -0.1672522 | 0.86717295 | 0.999976173 | -0.0255393 |
| YHR146W | CRP1 | 0.15269937 | -0.1672026 | 0.86721192 | 0.999976173 | -0.025531736 |
| YIL111W | COX5B | 0.15269937 | -0.1669286 | 0.86742756 | 0.999976173 | -0.025489885 |
| YOL004W | SIN3 | 0.15269937 | -0.166429 | 0.86782063 | 0.999976173 | -0.025413606 |
| YER143W | DDI1 | 0.15269937 | -0.1662555 | 0.86795721 | 0.999976173 | -0.025387105 |
| YBR131W | CCZ1 | 0.15269937 | -0.1659629 | 0.86818742 | 0.999976173 | -0.025342433 |
| YGR033C | TIM21 | 0.15269937 | -0.1653228 | 0.86869122 | 0.999976173 | -0.025244684 |
| YMR136W | GAT2 | 0.15269937 | -0.1642163 | 0.86956212 | 0.999976173 | -0.02507573 |
| YPR075C | OPY2 | 0.15269937 | -0.1641844 | 0.86958728 | 0.999976173 | -0.025070851 |
| YLL026W | HSP104 | 0.15269937 | -0.1640493 | 0.86969364 | 0.999976173 | -0.025050219 |
| YNR020C | ATP23 | 0.15269937 | -0.1639968 | 0.86973492 | 0.999976173 | -0.025042212 |
| YMR204C | INP1 | 0.15269937 | -0.1636934 | 0.86997377 | 0.999976173 | -0.024995883 |
| YDL156W | YDL156W | 0.15269937 | -0.1634239 | 0.87018599 | 0.999976173 | -0.02495472 |
| YBR280C | SAF1 | 0.15269937 | -0.1633577 | 0.8702381 | 0.999976173 | -0.024944613 |
| YJL046W | AIM22 | 0.15269937 | -0.1620468 | 0.8712703 | 0.999976173 | -0.024744437 |
| YJL176C | SWI3 | 0.15269937 | -0.1619887 | 0.87131599 | 0.999976173 | -0.024735576 |
| YIL155C | GUT2 | 0.15269937 | -0.1617412 | 0.87151095 | 0.999976173 | -0.024697773 |
| YLR177W | YLR177W | 0.15269937 | -0.1613788 | 0.87179632 | 0.999976173 | -0.02464244 |
| YNL101W | AVT4 | 0.15269937 | -0.161278 | 0.87187571 | 0.999976173 | -0.024627047 |
| YOL006C | TOP1 | 0.15269937 | -0.1606307 | 0.87238552 | 0.999976173 | -0.024528207 |
| YNL120C | YNL120C | 0.15269937 | -0.1604873 | 0.87249846 | 0.999976173 | -0.024506312 |
| YIL108W | YIL108W | 0.15269937 | -0.1582266 | 0.87427947 | 0.999976173 | -0.024161104 |
| YHL031C | GOS1 | 0.15269937 | -0.1582238 | 0.8742817 | 0.999976173 | -0.02416067 |
| YBR084W | MIS1 | 0.15269937 | -0.1578156 | 0.87460334 | 0.999976173 | -0.024098341 |
| YLR089C | ALT1 | 0.15269937 | -0.1577781 | 0.87463285 | 0.999976173 | -0.024092622 |
| YLL060C | GTT2 | 0.15269937 | -0.1570066 | 0.87524085 | 0.999976173 | -0.023974812 |
| YBR162C | TOS1 | 0.15269937 | -0.1566619 | 0.87551252 | 0.999976173 | -0.023922177 |
| YMR035W | IMP2 | 0.15269937 | -0.1564785 | 0.87565709 | 0.999976173 | -0.023894167 |
| YML129C | COX14 | 0.15269937 | -0.1559264 | 0.87609221 | 0.999976173 | -0.02380987 |
| YLR018C | POM34 | 0.15269937 | -0.1553894 | 0.87651554 | 0.999976173 | -0.023727865 |
| YLR083C | EMP70 | 0.15269937 | -0.155363 | 0.87653636 | 0.999976173 | -0.023723833 |
| YOR076C | SKI7 | 0.15269937 | -0.1553097 | 0.87657836 | 0.999976173 | -0.023715696 |
| YKL188C | PXA2 | 0.15269937 | -0.1548766 | 0.87691977 | 0.999976173 | -0.023649565 |
| YCR043C | YCR043C | 0.15269937 | -0.1546614 | 0.87708944 | 0.999976173 | -0.023616702 |
| YDR329C | PEX3 | 0.15269937 | -0.1545271 | 0.87719535 | 0.999976173 | -0.023596191 |
| YMR060C | SAM37 | 0.15269937 | -0.1543596 | 0.87732738 | 0.999976173 | -0.023570618 |
| YNL275W | BOR1 | 0.15269937 | -0.153742 | 0.87781438 | 0.999976173 | -0.023476302 |
| YMR205C | PFK2 | 0.15269937 | -0.1536642 | 0.87787569 | 0.999976173 | -0.023464429 |
| YOR183W | FYV12 | 0.15269937 | -0.1536318 | 0.87790127 | 0.999976173 | -0.023459477 |
| YFR008W | FAR7 | 0.15269937 | -0.1534214 | 0.87806716 | 0.999976173 | -0.023427351 |
| YLR333C | RPS25B | 0.15269937 | -0.1525786 | 0.87873177 | 0.999976173 | -0.023298659 |
| YDR248C | YDR248C | 0.15269937 | -0.1521282 | 0.879087 | 0.999976173 | -0.023229882 |
| YLR191W | PEX13 | 0.15269937 | -0.1515028 | 0.87958029 | 0.999976173 | -0.02313438 |
| YPL213W | LEA1 | 0.15269937 | -0.1514663 | 0.87960911 | 0.999976173 | -0.023128801 |
| YDR265W | PEX10 | 0.15269937 | -0.1513559 | 0.87969612 | 0.999976173 | -0.023111956 |
| YOL014W | YOL014W | 0.15269937 | -0.1511727 | 0.87984065 | 0.999976173 | -0.023083978 |
| YOR216C | RUD3 | 0.15269937 | -0.1508575 | 0.88008928 | 0.999976173 | -0.023035849 |
| YHR152W | SPO12 | 0.15269937 | -0.1505284 | 0.88034894 | 0.999976173 | -0.022985587 |
| YER089C | PTC2 | 0.15269937 | -0.1503322 | 0.8805037 | 0.999976173 | -0.022955632 |
| YDR409W | SIZ1 | 0.15269937 | -0.1498449 | 0.88088819 | 0.999976173 | -0.022881214 |
| YOR292C | YOR292C | 0.15269937 | -0.1491397 | 0.88144455 | 0.999976173 | -0.02277354 |
| YBR168W | PEX32 | 0.15269937 | -0.1480421 | 0.8823107 | 0.999976173 | -0.022605935 |
| YOR002W | ALG6 | 0.15269937 | -0.1475978 | 0.88266135 | 0.999976173 | -0.022538088 |
| YBR151W | APD1 | 0.15269937 | -0.1474421 | 0.8827842 | 0.999976173 | -0.022514321 |
| YLR435W | TSR2 | 0.15269937 | -0.1472745 | 0.88291649 | 0.999976173 | -0.022488726 |
| YMR156C | TPP1 | 0.15269937 | -0.1469097 | 0.88320441 | 0.999976173 | -0.022433023 |
| YHR079C-B | NA | 0.15269937 | -0.1467961 | 0.88329409 | 0.999976173 | -0.022415675 |
| YGL260W | YGL260W | 0.15269937 | -0.1467015 | 0.8833688 | 0.999976173 | -0.022401222 |
| YKL093W | MBR1 | 0.15269937 | -0.146609 | 0.8834418 | 0.999976173 | -0.022387099 |
| YLR090W | XDJ1 | 0.15269937 | -0.1457852 | 0.8840921 | 0.999976173 | -0.022261307 |
| YJL044C | GYP6 | 0.15269937 | -0.1449647 | 0.88473984 | 0.999976173 | -0.022136023 |
| YPR058W | YMC1 | 0.15269937 | -0.1447064 | 0.88494384 | 0.999976173 | -0.02209657 |

| | | | | | | |
|---|---|---|---|---|---|---|
| YOR007C | SGT2 | 0.15269937 | -0.1444683 | 0.88513181 | 0.999976173 | -0.022060218 |
| YHR049W | FSH1 | 0.15269937 | -0.1439848 | 0.88551356 | 0.999976173 | -0.021986394 |
| YER044C-A | MEI4 | 0.15269937 | -0.1439457 | 0.88554451 | 0.999976173 | -0.02198041 |
| YDR178W | SDH4 | 0.15269937 | -0.1437406 | 0.88570643 | 0.999976173 | -0.021949099 |
| YLR398C | SKI2 | 0.15269937 | -0.1424503 | 0.88672546 | 0.999976173 | -0.021752066 |
| YDR233C | RTN1 | 0.15269937 | -0.1421569 | 0.88695717 | 0.999976173 | -0.02170727 |
| YNL215W | IES2 | 0.15269937 | -0.1419396 | 0.88712878 | 0.999976173 | -0.021674094 |
| YPL112C | PEX25 | 0.15269937 | -0.1417276 | 0.88729624 | 0.999976173 | -0.021641721 |
| YOR123C | LEO1 | 0.15269937 | -0.1414602 | 0.88750747 | 0.999976173 | -0.021600889 |
| YGL202W | ARO8 | 0.15269937 | -0.1413176 | 0.88762015 | 0.999976173 | -0.021579106 |
| YBR180W | DTR1 | 0.15269937 | -0.1408983 | 0.88795142 | 0.999976173 | -0.021515074 |
| YLR104W | YLR104W | 0.15269937 | -0.1408463 | 0.88799248 | 0.999976173 | -0.021507137 |
| YCL038C | ATG22 | 0.15269937 | -0.140829 | 0.88800614 | 0.999976173 | -0.021504497 |
| YOL063C | CRT10 | 0.15269937 | -0.1408122 | 0.88801943 | 0.999976173 | -0.021501929 |
| YOR115C | TRS33 | 0.15269937 | -0.1407465 | 0.88807133 | 0.999976173 | -0.021491896 |
| YMR310C | YMR310C | 0.15269937 | -0.1402331 | 0.88847694 | 0.999976173 | -0.021413499 |
| YKL174C | TPO5 | 0.15269937 | -0.1399489 | 0.88870144 | 0.999976173 | -0.021370111 |
| YAR030C | YAR030C | 0.15269937 | -0.139535 | 0.88902848 | 0.999976173 | -0.021306907 |
| YDR352W | YDR352W | 0.15269937 | -0.1395204 | 0.88904001 | 0.999976173 | -0.021304679 |
| YBL103C | RTG3 | 0.10797476 | -0.197241 | 0.84364053 | 0.999976173 | -0.021297044 |
| YJR049C | UTR1 | 0.15269937 | -0.1382724 | 0.89002622 | 0.999976173 | -0.021114106 |
| YEL005C | VAB2 | 0.15269937 | -0.1381404 | 0.89013053 | 0.999976173 | -0.021093951 |
| YJR133W | XPT1 | 0.15269937 | -0.1380905 | 0.89016994 | 0.999976173 | -0.021086338 |
| YLR415C | YLR415C | 0.15269937 | -0.1377267 | 0.89045751 | 0.999976173 | -0.021030775 |
| YGR108W | CLB1 | 0.15269937 | -0.1371436 | 0.89091839 | 0.999976173 | -0.020941734 |
| YDR333C | YDR333C | 0.15269937 | -0.1369923 | 0.89103798 | 0.999976173 | -0.020918631 |
| YMR022W | UBC7 | 0.15269937 | -0.1369558 | 0.89106679 | 0.999976173 | -0.020913065 |
| YKR050W | TRK2 | 0.15269937 | -0.1367585 | 0.89122276 | 0.999976173 | -0.020882934 |
| YML113W | DAT1 | 0.15269937 | -0.1366783 | 0.89128614 | 0.999976173 | -0.020870691 |
| YBL088C | 25 | 0.15269937 | -0.1366256 | 0.89132782 | 0.999976173 | -0.020862639 |
| YIL057C | YIL057C | 0.15269937 | -0.1365729 | 0.89136946 | 0.999976173 | -0.020854595 |
| YOL070C | NBA1 | 0.15269937 | -0.1360597 | 0.89177517 | 0.999976173 | -0.020776225 |
| YFL026W | STE2 | 0.15269937 | -0.1357815 | 0.89199508 | 0.999976173 | -0.020733747 |
| YMR243C | ZRC1 | 0.15269937 | -0.1354298 | 0.89227308 | 0.999976173 | -0.020680051 |
| YMR269W | TMA23 | 0.15269937 | -0.1353895 | 0.89230497 | 0.999976173 | -0.020673892 |
| YDR260C | SWM1 | 0.15269937 | -0.1353454 | 0.89233983 | 0.999976173 | -0.020667158 |
| YBR217W | ATG12 | 0.15269937 | -0.1348249 | 0.89275133 | 0.999976173 | -0.020587683 |
| YMR316W | DIA1 | 0.15269937 | -0.1344712 | 0.89303101 | 0.999976173 | -0.020533671 |
| YLR433C | CNA1 | 0.15269937 | -0.1343494 | 0.89312732 | 0.999976173 | -0.020515071 |
| YMR052C-A | YMR052C-A | 0.15269937 | -0.1342558 | 0.89320136 | 0.999976173 | -0.020500773 |
| YBR222C | PCS60 | 0.15269937 | -0.1342064 | 0.89324037 | 0.999976173 | -0.020493239 |
| YIL157C | COA1 | 0.15269937 | -0.133486 | 0.8938101 | 0.999976173 | -0.020383223 |
| YDR223W | CRF1 | 0.15269937 | -0.1331771 | 0.89405435 | 0.999976173 | -0.02033606 |
| YER075C | PTP3 | 0.15269937 | -0.1330309 | 0.89416995 | 0.999976173 | -0.020313739 |
| YDL090C | RAM1 | 0.15269937 | -0.1325378 | 0.89455993 | 0.999976173 | -0.020238443 |
| YDR451C | YHP1 | 0.15269937 | -0.1324126 | 0.89465897 | 0.999976173 | -0.020219322 |
| YJR135C | MCM22 | 0.15269937 | -0.1321045 | 0.89490265 | 0.999976173 | -0.020172275 |
| YOR366W | YOR366W | 0.15269937 | -0.1312868 | 0.89554945 | 0.999976173 | -0.020047413 |
| YDL034W | YDL034W | 0.15269937 | -0.1306347 | 0.89606527 | 0.999976173 | -0.019947843 |
| YLL001W | DNM1 | 0.15269937 | -0.129773 | 0.89674704 | 0.999976173 | -0.019816254 |
| YOR286W | YOR286W | 0.15269937 | -0.1286445 | 0.89763993 | 0.999976173 | -0.019643938 |
| YOR003W | YSP3 | 0.15269937 | -0.1285274 | 0.89773264 | 0.999976173 | -0.019626048 |
| YBR115C | LYS2 | 0.10797476 | -0.1815179 | 0.85596249 | 0.999976173 | -0.019599353 |
| YOR243C | PUS7 | 0.15269937 | -0.128259 | 0.89794501 | 0.999976173 | -0.019585067 |
| YKL015W | PUT3 | 0.15269937 | -0.128128 | 0.8980487 | 0.999976173 | -0.019565059 |
| YJL175W | YJL175W | 0.15269937 | -0.1279645 | 0.89817807 | 0.999976173 | -0.019540097 |
| YJR033C | RAV1 | 0.15269937 | -0.1278513 | 0.89826765 | 0.999976173 | -0.019522812 |
| YOR023C | AHC1 | 0.15269937 | -0.1274489 | 0.89858607 | 0.999976173 | -0.019461373 |
| YMR201C | RAD14 | 0.15269937 | -0.1274328 | 0.89859885 | 0.999976173 | -0.019458907 |
| YHR081W | LRP1 | 0.15269937 | -0.1272682 | 0.89872909 | 0.999976173 | -0.019433779 |
| YEL065W | SIT1 | 0.15269937 | -0.1266172 | 0.8992444 | 0.999976173 | -0.019334359 |
| YJR011C | YJR011C | 0.15269937 | -0.1265603 | 0.89928943 | 0.999976173 | -0.019325673 |
| YCR091W | KIN82 | 0.15269937 | -0.126482 | 0.89935141 | 0.999976173 | -0.019313715 |
| YCL074W | YCL074W | 0.15269937 | -0.126125 | 0.89963394 | 0.999976173 | -0.019259211 |

| | | | | | | |
|---|---|---|---|---|---|---|
| YJL124C | LSM1 | 0.15269937 | -0.1260148 | 0.89972121 | 0.999976173 | -0.019242376 |
| YFR038W | IRC5 | 0.15269937 | -0.1255124 | 0.90011886 | 0.999976173 | -0.019165668 |
| YDL183C | YDL183C | 0.15269937 | -0.1251516 | 0.90040452 | 0.999976173 | -0.019110567 |
| YJL108C | PRM10 | 0.15269937 | -0.1244773 | 0.90093832 | 0.999976173 | -0.019007609 |
| YNR018W | AIM38 | 0.15269937 | -0.1233382 | 0.90184026 | 0.999976173 | -0.018833664 |
| YGR066C | YGR066C | 0.15269937 | -0.1229311 | 0.90216265 | 0.999976173 | -0.018771494 |
| YMR254C | YMR254C | 0.15269937 | -0.1227975 | 0.90226838 | 0.999976173 | -0.018751105 |
| YGR290W | YGR290W | 0.15269937 | -0.1209337 | 0.90374452 | 0.999976173 | -0.018466494 |
| YLR061W | RPL22A | 0.15269937 | -0.1198615 | 0.9045938 | 0.999976173 | -0.018302774 |
| YMR017W | SPO20 | 0.15269937 | -0.1195447 | 0.90484472 | 0.999976173 | -0.018254406 |
| YDR501W | PLM2 | 0.15269937 | -0.1190765 | 0.90521569 | 0.999976173 | -0.018182903 |
| YNL109W | YNL109W | 0.15269937 | -0.118867 | 0.90538169 | 0.999976173 | -0.018150908 |
| YHR115C | DMA1 | 0.15269937 | -0.1188361 | 0.9054061 | 0.999976173 | -0.018146203 |
| YJL204C | RCY1 | 0.15269937 | -0.1188324 | 0.90540907 | 0.999976173 | -0.018145631 |
| YDL180W | YDL180W | 0.15269937 | -0.118766 | 0.90546165 | 0.999976173 | -0.018135495 |
| YJL155C | FBP26 | 0.15269937 | -0.1187525 | 0.9054724 | 0.999976173 | -0.018133424 |
| YDR005C | MAF1 | 0.15269937 | -0.1185399 | 0.9056408 | 0.999976173 | -0.018100968 |
| YOR133W | EFT1 | 0.15269937 | -0.1181676 | 0.90593581 | 0.999976173 | -0.01804411 |
| YAR042W | SWH1 | 0.15269937 | -0.117546 | 0.90642828 | 0.999976173 | -0.017949204 |
| YDR493W | AIM8 | 0.15269937 | -0.1170303 | 0.90683698 | 0.999976173 | -0.017870447 |
| YGL154C | LYS5 | 0.15269937 | -0.1159807 | 0.90766876 | 0.999976173 | -0.017710174 |
| YCR071C | IMG2 | 0.15269937 | -0.1157912 | 0.90781895 | 0.999976173 | -0.017681238 |
| YLR046C | YLR046C | 0.15269937 | -0.1145578 | 0.90879653 | 0.999976173 | -0.017492902 |
| YBR042C | CST26 | 0.15269937 | -0.1144584 | 0.90887529 | 0.999976173 | -0.017477728 |
| YLR381W | CTF3 | 0.15269937 | -0.1143775 | 0.90893946 | 0.999976173 | -0.017465367 |
| YGR070W | ROM1 | 0.15269937 | -0.1139781 | 0.90925603 | 0.999976173 | -0.017404385 |
| YIL130W | ASG1 | 0.15269937 | -0.1137876 | 0.90940705 | 0.999976173 | -0.017375295 |
| YDR304C | CPR5 | 0.15269937 | -0.1136482 | 0.90951754 | 0.999976173 | -0.017354011 |
| YER067C-A | YER067C-A | 0.15269937 | -0.113466 | 0.90966201 | 0.999976173 | -0.017326184 |
| YKL047W | YKL047W | 0.15269937 | -0.1133244 | 0.90977425 | 0.999976173 | -0.017304564 |
| YKL124W | SSH4 | 0.15269937 | -0.1120889 | 0.91075377 | 0.999976173 | -0.017115908 |
| YDR469W | SDC1 | 0.15269937 | -0.1119822 | 0.91083841 | 0.999976173 | -0.017099607 |
| YER109C | FLO8 | 0.15269937 | -0.1113113 | 0.91137034 | 0.999976173 | -0.016997169 |
| YMR161W | HLJ1 | 0.15269937 | -0.1113033 | 0.91137671 | 0.999976173 | -0.016995941 |
| YML102C-A | NA | 0.15269937 | -0.1108165 | 0.9117627 | 0.999976173 | -0.016921613 |
| YBL001C | ECM15 | 0.15269937 | -0.1104653 | 0.91204124 | 0.999976173 | -0.016867979 |
| YLL045C | RPL8B | 0.15269937 | -0.1100467 | 0.91237316 | 0.999976173 | -0.016804067 |
| YBR125C | PTC4 | 0.15269937 | -0.1091257 | 0.91310363 | 0.999976173 | -0.016663426 |
| YML116W | ATR1 | 0.15269937 | -0.1085691 | 0.91354508 | 0.999976173 | -0.016578438 |
| YCR087C-A | YCR087C-A | 0.15269937 | -0.1084855 | 0.91361142 | 0.999976173 | -0.016565667 |
| YJL089W | SIP4 | 0.15269937 | -0.1080767 | 0.91393572 | 0.999976173 | -0.016503238 |
| YNL037C | IDH1 | 0.15269937 | -0.1079108 | 0.91406727 | 0.999976173 | -0.016477914 |
| YDR022C | CIS1 | 0.15269937 | -0.1068591 | 0.91490156 | 0.999976173 | -0.016317322 |
| YPL260W | YPL260W | 0.15269937 | -0.106477 | 0.91520472 | 0.999976173 | -0.016258971 |
| YHR059W | FYV4 | 0.15269937 | -0.1063891 | 0.91527445 | 0.999976173 | -0.016245552 |
| YDL033C | SLM3 | 0.15269937 | -0.1062305 | 0.91540031 | 0.999976173 | -0.016221327 |
| YOR186W | YOR186W | 0.15269937 | -0.1061936 | 0.91542956 | 0.999976173 | -0.016215698 |
| YBR271W | YBR271W | 0.15269937 | -0.1058476 | 0.91570412 | 0.999976173 | -0.016162856 |
| YOR041C | YOR041C | 0.15269937 | -0.1050439 | 0.91634182 | 0.999976173 | -0.016040132 |
| YNL283C | WSC2 | 0.15269937 | -0.1047496 | 0.91657529 | 0.999976173 | -0.015995203 |
| YMR135W-A | YMR135W-A | 0.15269937 | -0.1041361 | 0.91706215 | 0.999976173 | -0.015901516 |
| YBL056W | PTC3 | 0.15269937 | -0.1032577 | 0.91775926 | 0.999976173 | -0.015767382 |
| YER156C | YER156C | 0.15269937 | -0.1030437 | 0.91792911 | 0.999976173 | -0.015734703 |
| YPR078C | YPR078C | 0.15269937 | -0.1028379 | 0.91809245 | 0.999976173 | -0.015703276 |
| YLL043W | FPS1 | 0.15269937 | -0.1025076 | 0.91835459 | 0.999976173 | -0.015652843 |
| YNR031C | SSK2 | 0.15269937 | -0.1023083 | 0.91851274 | 0.999976173 | -0.015622416 |
| YJL169W | YJL169W | 0.15269937 | -0.1021577 | 0.91863228 | 0.999976173 | -0.015599419 |
| YLR344W | RPL26A | 0.15269937 | -0.1017869 | 0.91892663 | 0.999976173 | -0.015542791 |
| YCL047C | YCL047C | 0.15269937 | -0.1017383 | 0.91896518 | 0.999976173 | -0.015535376 |
| YOR127W | RGA1 | 0.15269937 | -0.1016886 | 0.91900465 | 0.999976173 | -0.015527782 |
| YDL130W-A | STF1 | 0.15269937 | -0.1011862 | 0.91940346 | 0.999976173 | -0.015451064 |
| YHR078W | YHR078W | 0.15269937 | -0.1006522 | 0.91982732 | 0.999976173 | -0.015369531 |
| YBL054W | TOD6 | 0.15269937 | -0.1006475 | 0.91983108 | 0.999976173 | -0.015368806 |
| YLR081W | GAL2 | 0.15269937 | -0.1005859 | 0.91987999 | 0.999976173 | -0.015359399 |

| | | | | | | |
|---|---|---|---|---|---|---|
| YBR262C | AIM5 | 0.15269937 | -0.1005437 | 0.91991348 | 0.999976173 | -0.015352958 |
| YLR400W | YLR400W | 0.15269937 | -0.1001483 | 0.92022735 | 0.999976173 | -0.015292585 |
| YJL045W | YJL045W | 0.15269937 | -0.0998085 | 0.92049712 | 0.999976173 | -0.015240698 |
| YDL177C | YDL177C | 0.15269937 | -0.0995353 | 0.92071401 | 0.999976173 | -0.015198981 |
| YKR058W | GLG1 | 0.15269937 | -0.099242 | 0.92094689 | 0.999976173 | -0.015154191 |
| YKR054C | DYN1 | 0.15269937 | -0.0991299 | 0.92103587 | 0.999976173 | -0.015137079 |
| YDL232W | OST4 | 0.15269937 | -0.0984861 | 0.92154711 | 0.999976173 | -0.015038758 |
| YHL033C | RPL8A | 0.15269937 | -0.0983897 | 0.92162362 | 0.999976173 | -0.015024043 |
| YMR127C | SAS2 | 0.15269937 | -0.0982584 | 0.92172783 | 0.999976173 | -0.015004002 |
| YGL114W | YGL114W | 0.15269937 | -0.0980617 | 0.92188403 | 0.999976173 | -0.014973964 |
| YGL004C | RPN14 | 0.15269937 | -0.0979443 | 0.92197726 | 0.999976173 | -0.014956037 |
| YNL328C | MDJ2 | 0.15269937 | -0.0964435 | 0.92316908 | 0.999976173 | -0.014726864 |
| YKL191W | DPH2 | 0.15269937 | -0.0962178 | 0.92334831 | 0.999976173 | -0.014692402 |
| YIL146C | ATG32 | 0.15269937 | -0.0961016 | 0.92344066 | 0.999976173 | -0.014674646 |
| YDR257C | RKM4 | 0.15269937 | -0.0956026 | 0.92383696 | 0.999976173 | -0.014598452 |
| YBR046C | ZTA1 | 0.15269937 | -0.0954493 | 0.92395869 | 0.999976173 | -0.014575048 |
| YDR229W | IVY1 | 0.15269937 | -0.0948955 | 0.9243986 | 0.999976173 | -0.014490476 |
| YGR138C | TPO2 | 0.15269937 | -0.0948903 | 0.9244027 | 0.999976173 | -0.014489687 |
| YMR215W | GAS3 | 0.15269937 | -0.0948632 | 0.92442419 | 0.999976173 | -0.014485557 |
| YGR243W | FMP43 | 0.15269937 | -0.0948453 | 0.92443842 | 0.999976173 | -0.014482821 |
| YDL187C | YDL187C | 0.15269937 | -0.0948253 | 0.92445431 | 0.999976173 | -0.014479767 |
| YDL233W | YDL233W | 0.15269937 | -0.0948019 | 0.92447289 | 0.999976173 | -0.014476194 |
| YAL037W | YAL037W | 0.15269937 | -0.094591 | 0.92464045 | 0.999976173 | -0.014443981 |
| YJL152W | YJL152W | 0.15269937 | -0.094428 | 0.92476988 | 0.999976173 | -0.014419101 |
| YLR133W | CKI1 | 0.15269937 | -0.094332 | 0.92484618 | 0.999976173 | -0.014404432 |
| YFR040W | SAP155 | 0.15269937 | -0.0942852 | 0.92488332 | 0.999976173 | -0.014397294 |
| YFR015C | GSY1 | 0.15269937 | -0.0937278 | 0.9253261 | 0.999976173 | -0.014312178 |
| YKR024C | DBP7 | 0.15269937 | -0.0934421 | 0.92555307 | 0.999976173 | -0.014268549 |
| YFL036W | RPO41 | 0.15269937 | -0.0933261 | 0.92564519 | 0.999976173 | -0.014250842 |
| YGL066W | SGF73 | 0.15269937 | -0.0923933 | 0.9263863 | 0.999976173 | -0.014108393 |
| YMR241W | YHM2 | 0.15269937 | -0.0922982 | 0.92646187 | 0.999976173 | -0.014093869 |
| YNR067C | DSE4 | 0.15269937 | -0.0921958 | 0.92654318 | 0.999976173 | -0.014078242 |
| YGL196W | DSD1 | 0.15269937 | -0.0919048 | 0.9267744 | 0.999976173 | -0.014033803 |
| YER145C | FTR1 | 0.15269937 | -0.0918489 | 0.92681878 | 0.999976173 | -0.014025273 |
| YGR234W | YHB1 | 0.15269937 | -0.0916511 | 0.92697592 | 0.999976173 | -0.013995072 |
| YNL043C | YNL043C | 0.15269937 | -0.0910755 | 0.9274333 | 0.999976173 | -0.013907173 |
| YGR287C | YGR287C | 0.15269937 | -0.0908874 | 0.92758277 | 0.999976173 | -0.013878448 |
| YKL151C | YKL151C | 0.15269937 | -0.0894882 | 0.92869465 | 0.999976173 | -0.013664787 |
| YOR170W | YOR170W | 0.15269937 | -0.0883925 | 0.92956539 | 0.999976173 | -0.013497482 |
| YDR070C | FMP16 | 0.15269937 | -0.0882397 | 0.92968683 | 0.999976173 | -0.013474149 |
| YJL171C | YJL171C | 0.15269937 | -0.0879384 | 0.92992634 | 0.999976173 | -0.013428133 |
| YER034W | YER034W | 0.15269937 | -0.087162 | 0.93054339 | 0.999976173 | -0.013309588 |
| YMR291W | YMR291W | 0.15269937 | -0.0867939 | 0.93083604 | 0.999976173 | -0.013253366 |
| YOL073C | YOL073C | 0.15269937 | -0.0860373 | 0.93143743 | 0.999976173 | -0.013137841 |
| YMR214W | SCJ1 | 0.15269937 | -0.0854512 | 0.93190336 | 0.999976173 | -0.013048342 |
| YNL011C | YNL011C | 0.15269937 | -0.0841454 | 0.93294147 | 0.999976173 | -0.012848952 |
| YLL017W | YLL017W | 0.15269937 | -0.0827337 | 0.93406395 | 0.999976173 | -0.01263338 |
| YFL052W | YFL052W | 0.15269937 | -0.082683 | 0.93410426 | 0.999976173 | -0.012625639 |
| YIL139C | REV7 | 0.15269937 | -0.0821396 | 0.93453638 | 0.999976173 | -0.012542657 |
| YPR197C | YPR197C | 0.15269937 | -0.0815727 | 0.93498714 | 0.999976173 | -0.0124561 |
| YKL009W | MRT4 | 0.15269937 | -0.0806636 | 0.93571011 | 0.999976173 | -0.01231728 |
| YGR201C | YGR201C | 0.15269937 | -0.0805891 | 0.93576935 | 0.999976173 | -0.012305906 |
| YKL133C | YKL133C | 0.15269937 | -0.0800656 | 0.93618573 | 0.999976173 | -0.012225961 |
| YBR231C | SWC5 | 0.15269937 | -0.0797903 | 0.93640464 | 0.999976173 | -0.012183932 |
| YNR057C | BIO4 | 0.15269937 | -0.0796555 | 0.93651184 | 0.999976173 | -0.01216335 |
| YKR049C | FMP46 | 0.15269937 | -0.0784756 | 0.93745037 | 0.999976173 | -0.011983169 |
| YGR256W | GND2 | 0.15269937 | -0.0774648 | 0.93825439 | 0.999976173 | -0.011828827 |
| YGR026W | YGR026W | 0.15269937 | -0.076905 | 0.93869975 | 0.999976173 | -0.011743338 |
| YBR075W | NA | 0.10797476 | -0.108722 | 0.91342381 | 0.999976173 | -0.011739235 |
| YPL250C | ICY2 | 0.15269937 | -0.0765633 | 0.93897158 | 0.999976173 | -0.011691161 |
| YPL053C | KTR6 | 0.15269937 | -0.0762971 | 0.9391833 | 0.999976173 | -0.011650522 |
| YOR311C | DGK1 | 0.15269937 | -0.0761019 | 0.93933859 | 0.999976173 | -0.011620717 |
| YER114C | BOI2 | 0.15269937 | -0.0759161 | 0.93948642 | 0.999976173 | -0.011592344 |
| YBR266C | SLM6 | 0.15269937 | -0.075662 | 0.93968857 | 0.999976173 | -0.011553544 |

| | | | | | | |
|---|---|---|---|---|---|---|
| YLR173W | YLR173W | 0.15269937 | -0.0755255 | 0.93979717 | 0.999976173 | -0.011532701 |
| YOR338W | YOR338W | 0.15269937 | -0.0754962 | 0.93982047 | 0.999976173 | -0.011528228 |
| YPL159C | PET20 | 0.15269937 | -0.0754175 | 0.93988313 | 0.999976173 | -0.011516202 |
| YDR281C | PHM6 | 0.15269937 | -0.0749333 | 0.9402684 | 0.999976173 | -0.011442261 |
| YLR311C | YLR311C | 0.15269937 | -0.0748707 | 0.94031814 | 0.999976173 | -0.011432713 |
| YHR195W | NVJ1 | 0.15269937 | -0.0743803 | 0.94070837 | 0.999976173 | -0.011357822 |
| YHR182W | YHR182W | 0.15269937 | -0.0739974 | 0.94101305 | 0.999976173 | -0.011299351 |
| YGL051W | MST27 | 0.15269937 | -0.0735882 | 0.94133865 | 0.999976173 | -0.011236867 |
| YIL016W | SNL1 | 0.15269937 | -0.0734002 | 0.94148824 | 0.999976173 | -0.01120816 |
| YOR142W | LSC1 | 0.15269937 | -0.073377 | 0.94150665 | 0.999976173 | -0.011204628 |
| YLR111W | YLR111W | 0.15269937 | -0.0731275 | 0.94170522 | 0.999976173 | -0.011166524 |
| YIL090W | ICE2 | 0.15269937 | -0.0715528 | 0.94295833 | 0.999976173 | -0.010926073 |
| YOR065W | CYT1 | 0.15269937 | -0.071025 | 0.94337843 | 0.999976173 | -0.010845468 |
| YPL081W | RPS9A | 0.15269937 | -0.0709457 | 0.94344152 | 0.999976173 | -0.010833363 |
| YLR109W | AHP1 | 0.15269937 | -0.0703928 | 0.94388154 | 0.999976173 | -0.010748941 |
| YHR103W | SBE22 | 0.15269937 | -0.0696545 | 0.94446922 | 0.999976173 | -0.010636193 |
| YAL067C | SEO1 | 0.15269937 | -0.0686185 | 0.94529383 | 0.999976173 | -0.010477999 |
| YDR153C | ENT5 | 0.15269937 | -0.068556 | 0.94534357 | 0.999976173 | -0.010468458 |
| YER149C | PEA2 | 0.15269937 | -0.0684796 | 0.94540437 | 0.999976173 | -0.010456795 |
| YDR043C | NRG1 | 0.15269937 | -0.0682263 | 0.94560604 | 0.999976173 | -0.010418108 |
| YER042W | MXR1 | 0.15269937 | -0.0680437 | 0.94575134 | 0.999976173 | -0.010390237 |
| YOR032C | HMS1 | 0.15269937 | -0.0679267 | 0.94584452 | 0.999976173 | -0.010372362 |
| YOR114W | YOR114W | 0.15269937 | -0.0679052 | 0.94586159 | 0.999976173 | -0.010369088 |
| YLR151C | PCD1 | 0.15269937 | -0.0657509 | 0.94757664 | 0.999976173 | -0.010040124 |
| YNL045W | LAP2 | 0.15269937 | -0.0649789 | 0.94819132 | 0.999976173 | -0.009922233 |
| YKR016W | FCJ1 | 0.15269937 | -0.064799 | 0.94833453 | 0.999976173 | -0.009894769 |
| YDL080C | THI3 | 0.15269937 | -0.0647252 | 0.94839333 | 0.999976173 | -0.009883491 |
| YFL056C | AAD6 | 0.15269937 | -0.064453 | 0.94861 | 0.999976173 | -0.009841938 |
| YGR241C | YAP1802 | 0.15269937 | -0.0641066 | 0.94888582 | 0.999976173 | -0.009789042 |
| YDR001C | NTH1 | 0.15269937 | -0.0641016 | 0.94888979 | 0.999976173 | -0.00978828 |
| YDR083W | RRP8 | 0.15269937 | -0.0640873 | 0.9489012 | 0.999976173 | -0.009786091 |
| YBR156C | SLI15 | 0.15269937 | -0.0640589 | 0.94892384 | 0.999976173 | -0.009781749 |
| YNR001C | CIT1 | 0.15269937 | -0.0628625 | 0.94987645 | 0.999976173 | -0.009599071 |
| YJR066W | TOR1 | 0.15269937 | -0.0622116 | 0.95039483 | 0.999976173 | -0.009499668 |
| YDR104C | SPO71 | 0.15269937 | -0.062193 | 0.95040965 | 0.999976173 | -0.009496824 |
| YKL164C | PIR1 | 0.15269937 | -0.0611824 | 0.95121438 | 0.999976173 | -0.00934252 |
| YLR287C-A | RPS30A | 0.15269937 | -0.061143 | 0.95124578 | 0.999976173 | -0.0093365 |
| YGL149W | YGL149W | 0.15269937 | -0.0600883 | 0.95208577 | 0.999976173 | -0.009175446 |
| YNL046W | YNL046W | 0.15269937 | -0.0582142 | 0.95357843 | 0.999976173 | -0.008889278 |
| YOL053W | AIM39 | 0.15269937 | -0.0578001 | 0.95390831 | 0.999976173 | -0.008826038 |
| YDR313C | PIB1 | 0.15269937 | -0.0577296 | 0.95396444 | 0.999976173 | -0.008815277 |
| YPL167C | REV3 | 0.15269937 | -0.0568614 | 0.95465603 | 0.999976173 | -0.008682702 |
| YBR132C | AGP2 | 0.15269937 | -0.0568329 | 0.95467874 | 0.999976173 | -0.008678349 |
| YLR292C | SEC72 | 0.15269937 | -0.0556763 | 0.95560009 | 0.999976173 | -0.008501739 |
| YLR178C | TFS1 | 0.15269937 | -0.0556412 | 0.95562803 | 0.999976173 | -0.008496383 |
| YPR152C | URN1 | 0.15269937 | -0.0555895 | 0.95566926 | 0.999976173 | -0.008488481 |
| YMR189W | GCV2 | 0.15269937 | -0.0551243 | 0.9560399 | 0.999976173 | -0.008417439 |
| YAL024C | LTE1 | 0.15269937 | -0.054922 | 0.95620106 | 0.999976173 | -0.008386549 |
| YDL023C | YDL023C | 0.15269937 | -0.0548413 | 0.95626528 | 0.999976173 | -0.008374239 |
| YIL079C | AIR1 | 0.15269937 | -0.0536686 | 0.95719963 | 0.999976173 | -0.008195159 |
| YML008C | ERG6 | 0.15269937 | -0.0530867 | 0.95766325 | 0.999976173 | -0.008106303 |
| YDR169C | STB3 | 0.15269937 | -0.0529985 | 0.95773348 | 0.999976173 | -0.008092844 |
| YPL206C | PGC1 | 0.15269937 | -0.0519873 | 0.95853925 | 0.999976173 | -0.007938422 |
| YDL035C | GPR1 | 0.15269937 | -0.0516877 | 0.95877791 | 0.999976173 | -0.007892685 |
| YAL029C | MYO4 | 0.15269937 | -0.0513744 | 0.95902756 | 0.999976173 | -0.007844841 |
| YER129W | SAK1 | 0.15269937 | -0.0511788 | 0.9591834 | 0.999976173 | -0.007814978 |
| YPR044C | OPI11 | 0.15269937 | -0.0505562 | 0.95967953 | 0.999976173 | -0.007719905 |
| YGR284C | ERV29 | 0.15269937 | -0.0503604 | 0.95983556 | 0.999976173 | -0.007690004 |
| YPL232W | SSO1 | 0.15269937 | -0.0501807 | 0.95997878 | 0.999976173 | -0.007662562 |
| YIR032C | DAL3 | 0.15269937 | -0.0500772 | 0.96006122 | 0.999976173 | -0.007646763 |
| YBL071C | YBL071C | 0.15269937 | -0.0499263 | 0.9601815 | 0.999976173 | -0.007623716 |
| YOR090C | PTC5 | 0.15269937 | -0.049614 | 0.96043035 | 0.999976173 | -0.007576031 |
| YJR035W | RAD26 | 0.15269937 | -0.0494148 | 0.96058911 | 0.999976173 | -0.00754561 |
| YGL232W | TAN1 | 0.15269937 | -0.0492398 | 0.96072859 | 0.999976173 | -0.007518883 |

| | | | | | | |
|---|---|---|---|---|---|---|
| YGL177W | YGL177W | 0.15269937 | -0.0491907 | 0.96076774 | 0.999976173 | -0.007511382 |
| YDR261C | EXG2 | 0.15269937 | -0.0489021 | 0.96099765 | 0.999976173 | -0.007467327 |
| YPL263C | KEL3 | 0.15269937 | -0.0488796 | 0.96101563 | 0.999976173 | -0.007463882 |
| YDL074C | BRE1 | 0.10797476 | -0.0688579 | 0.94510329 | 0.999976173 | -0.007434911 |
| YPL208W | RKM1 | 0.15269937 | -0.0483905 | 0.96140537 | 0.999976173 | -0.007389205 |
| YLL058W | YLL058W | 0.15269937 | -0.0482045 | 0.96155367 | 0.999976173 | -0.00736079 |
| YDL065C | PEX19 | 0.15269937 | -0.0476963 | 0.96195864 | 0.999976173 | -0.007283197 |
| YDR471W | RPL27B | 0.15269937 | -0.0468397 | 0.96264134 | 0.999976173 | -0.007152395 |
| YOL028C | YAP7 | 0.15269937 | -0.0459108 | 0.96338168 | 0.999976173 | -0.007010553 |
| YHR067W | HTD2 | 0.15269937 | -0.0458522 | 0.96342843 | 0.999976173 | -0.007001598 |
| YDL168W | SFA1 | 0.15269937 | -0.0457682 | 0.96349536 | 0.999976173 | -0.006988775 |
| YNL288W | CAF40 | 0.15269937 | -0.0453205 | 0.96385223 | 0.999976173 | -0.006920406 |
| YJR115W | YJR115W | 0.15269937 | -0.0451808 | 0.96396355 | 0.999976173 | -0.006899079 |
| YPR053C | YPR053C | 0.15269937 | -0.0448124 | 0.96425717 | 0.999976173 | -0.006842829 |
| YOL007C | CSI2 | 0.15269937 | -0.0442253 | 0.96472513 | 0.999976173 | -0.00675318 |
| YGR122C-A | YGR122C-A | 0.15269937 | -0.0441024 | 0.96482308 | 0.999976173 | -0.006734415 |
| YKR009C | FOX2 | 0.15269937 | -0.0429204 | 0.96576534 | 0.999976173 | -0.006553913 |
| YPL216W | YPL216W | 0.15269937 | -0.0427042 | 0.96593766 | 0.999976173 | -0.006520905 |
| YOR037W | CYC2 | 0.15269937 | -0.0426994 | 0.96594148 | 0.999976173 | -0.006520172 |
| YOL045W | PSK2 | 0.15269937 | -0.0422894 | 0.96626833 | 0.999976173 | -0.006457563 |
| YDR306C | YDR306C | 0.15269937 | -0.0422767 | 0.96627848 | 0.999976173 | -0.00645562 |
| YOL025W | LAG2 | 0.15269937 | -0.0419563 | 0.96653386 | 0.999976173 | -0.0064067 |
| YJR048W | CYC1 | 0.15269937 | -0.0414031 | 0.96697484 | 0.999976173 | -0.006322231 |
| YMR266W | RSN1 | 0.15269937 | -0.0409494 | 0.96733654 | 0.999976173 | -0.006252949 |
| YJL199C | MBB1 | 0.15269937 | -0.0401447 | 0.96797811 | 0.999976173 | -0.006130064 |
| YLR389C | STE23 | 0.15269937 | -0.0397721 | 0.96827512 | 0.999976173 | -0.006073176 |
| YMR223W | UBP8 | 0.15269937 | -0.0392797 | 0.96866772 | 0.999976173 | -0.00599798 |
| YAL022C | FUN26 | 0.15269937 | -0.0389373 | 0.96894071 | 0.999976173 | -0.005945696 |
| YOL090W | MSH2 | 0.15269937 | -0.0384629 | 0.96931888 | 0.999976173 | -0.005873267 |
| YLR289W | GUF1 | 0.15269937 | -0.0384203 | 0.96935291 | 0.999976173 | -0.005866749 |
| YOL152W | FRE7 | 0.15269937 | -0.0375384 | 0.970056 | 0.999976173 | -0.005732092 |
| YMR124W | YMR124W | 0.15269937 | -0.036807 | 0.97063917 | 0.999976173 | -0.005620407 |
| YGR151C | YGR151C | 0.15269937 | -0.0366672 | 0.97075067 | 0.999976173 | -0.005599054 |
| YLR404W | FLD1 | 0.15269937 | -0.0349954 | 0.97208371 | 0.999976173 | -0.005343769 |
| YOR234C | RPL33B | 0.15269937 | -0.0347961 | 0.97224261 | 0.999976173 | -0.00531334 |
| YMR148W | OSW5 | 0.15269937 | -0.0345533 | 0.97243618 | 0.999976173 | -0.005276273 |
| YGR124W | ASN2 | 0.15269937 | -0.0344729 | 0.97250031 | 0.999976173 | -0.005263991 |
| YDR453C | TSA2 | 0.15269937 | -0.03446 | 0.97251064 | 0.999976173 | -0.005262013 |
| YLL016W | SDC25 | 0.15269937 | -0.0344252 | 0.97253837 | 0.999976173 | -0.005256703 |
| YBR056W | YBR056W | 0.15269937 | -0.0342443 | 0.97268262 | 0.999976173 | -0.00522908 |
| YPL156C | PRM4 | 0.15269937 | -0.0338661 | 0.97298415 | 0.999976173 | -0.005171339 |
| YLR113W | HOG1 | 0.15269937 | -0.0334567 | 0.97331062 | 0.999976173 | -0.005108824 |
| YGL108C | YGL108C | 0.15269937 | -0.0328559 | 0.97378976 | 0.999976173 | -0.005017073 |
| YCR073W-A | SOL2 | 0.15269937 | -0.0325186 | 0.97405871 | 0.999976173 | -0.004965574 |
| YKL094W | YJU3 | 0.15269937 | -0.0323687 | 0.97417829 | 0.999976173 | -0.004942676 |
| YLR037C | PAU23 | 0.15269937 | -0.0322655 | 0.97426054 | 0.999976173 | -0.004926928 |
| YNR015W | SMM1 | 0.15269937 | -0.0312909 | 0.97503778 | 0.999976173 | -0.004778102 |
| YPL009C | YPL009C | 0.15269937 | -0.0312511 | 0.9750695 | 0.999976173 | -0.00477203 |
| YLR427W | MAG2 | 0.15269937 | -0.0304812 | 0.97568356 | 0.999976173 | -0.004654452 |
| YGL024W | YGL024W | 0.15269937 | -0.0303798 | 0.97576442 | 0.999976173 | -0.00463897 |
| YPL055C | LGE1 | 0.15269937 | -0.0301415 | 0.97595446 | 0.999976173 | -0.004602584 |
| YMR159C | ATG16 | 0.15269937 | -0.0297014 | 0.97630545 | 0.999976173 | -0.00453538 |
| YGR276C | RNH70 | 0.15269937 | -0.0295721 | 0.97640857 | 0.999976173 | -0.004515637 |
| YGR041W | BUD9 | 0.15269937 | -0.0294912 | 0.97647309 | 0.999976173 | -0.004503282 |
| YJR124C | YJR124C | 0.15269937 | -0.0292412 | 0.97667243 | 0.999976173 | -0.004465117 |
| YMR192W | GYL1 | 0.15269937 | -0.0291233 | 0.9767665 | 0.999976173 | -0.004447104 |
| YMR167W | MLH1 | 0.15269937 | -0.0286709 | 0.97712726 | 0.999976173 | -0.004378034 |
| YGR081C | SLX9 | 0.15269937 | -0.0286551 | 0.9771399 | 0.999976173 | -0.004375613 |
| YHR082C | KSP1 | 0.15269937 | -0.0282628 | 0.97745273 | 0.999976173 | -0.004315718 |
| YLR287C | YLR287C | 0.15269937 | -0.0275834 | 0.97799463 | 0.999976173 | -0.004211969 |
| YDR239C | YDR239C | 0.15269937 | -0.0269065 | 0.97853454 | 0.999976173 | -0.004108601 |
| YPR062W | FCY1 | 0.15269937 | -0.0268885 | 0.9785489 | 0.999976173 | -0.004105852 |
| YKR094C | RPL40B | 0.15269937 | -0.0249347 | 0.98010727 | 0.999976173 | -0.003807507 |
| YCR089W | FIG2 | 0.15269937 | -0.0248671 | 0.98016116 | 0.999976173 | -0.003797192 |

| | | | | | | |
|---|---|---|---|---|---|---|
| YJR060W | CBF1 | 0.15269937 | -0.0245089 | 0.98044689 | 0.999976173 | -0.003742491 |
| YDL013W | SLX5 | 0.15269937 | -0.0242747 | 0.98063369 | 0.999976173 | -0.003706729 |
| YLR192C | HCR1 | 0.15269937 | -0.0239463 | 0.98089564 | 0.999976173 | -0.003656582 |
| YDR102C | YDR102C | 0.15269937 | -0.0237804 | 0.98102799 | 0.999976173 | -0.003631246 |
| YBR113W | YBR113W | 0.15269937 | -0.0234252 | 0.9813113 | 0.999976173 | -0.003577011 |
| YMR143W | RPS16A | 0.15269937 | -0.022893 | 0.9817358 | 0.999976173 | -0.003495746 |
| YMR101C | SRT1 | 0.15269937 | -0.0228133 | 0.98179934 | 0.999976173 | -0.003483583 |
| YER051W | JHD1 | 0.15269937 | -0.0227694 | 0.98183438 | 0.999976173 | -0.003476875 |
| YGR275W | RTT102 | 0.15269937 | -0.0227316 | 0.98186456 | 0.999976173 | -0.003471098 |
| YEL068C | YEL068C | 0.15269937 | -0.0219383 | 0.98249731 | 0.999976173 | -0.00334997 |
| YGL002W | ERP6 | 0.15269937 | -0.0216553 | 0.9827231 | 0.999976173 | -0.003306748 |
| YHR202W | YHR202W | 0.15269937 | -0.0215446 | 0.98281137 | 0.999976173 | -0.003289852 |
| YOR293W | RPS10A | 0.15269937 | -0.0213562 | 0.9829617 | 0.999976173 | -0.003261073 |
| YLR318W | 6 | 0.15269937 | -0.0211238 | 0.98314705 | 0.999976173 | -0.003225594 |
| YGL014W | PUF4 | 0.15269937 | -0.0210174 | 0.98323195 | 0.999976173 | -0.003209341 |
| YDL039C | PRM7 | 0.15269937 | -0.0205269 | 0.98362324 | 0.999976173 | -0.003134438 |
| YDL213C | NOP6 | 0.15269937 | -0.0203749 | 0.98374448 | 0.999976173 | -0.003111231 |
| YIL094C | LYS12 | 0.15269937 | -0.0200146 | 0.9840319 | 0.999976173 | -0.003056213 |
| YOR308C | SNU66 | 0.15269937 | -0.0199975 | 0.98404554 | 0.999976173 | -0.003053602 |
| YMR054W | STV1 | 0.15269937 | -0.0199159 | 0.98411062 | 0.999976173 | -0.003041144 |
| YML054C | CYB2 | 0.15269937 | -0.0197349 | 0.984255 | 0.999976173 | -0.003013507 |
| YKL031W | YKL031W | 0.15269937 | -0.0188811 | 0.98493614 | 0.999976173 | -0.002883124 |
| YLR032W | RAD5 | 0.15269937 | -0.0185042 | 0.98523675 | 0.999976173 | -0.002825584 |
| YOR111W | YOR111W | 0.15269937 | -0.0182243 | 0.98546004 | 0.999976173 | -0.002782842 |
| YGR133W | PEX4 | 0.15269937 | -0.0178539 | 0.98575552 | 0.999976173 | -0.002726285 |
| YOR303W | CPA1 | 0.15269937 | -0.0174349 | 0.98608978 | 0.999976173 | -0.002662302 |
| YNL270C | ALP1 | 0.15269937 | -0.0173253 | 0.98617722 | 0.999976173 | -0.002645565 |
| YLR309C | IMH1 | 0.15269937 | -0.0172374 | 0.98624734 | 0.999976173 | -0.002632144 |
| YGR077C | PEX8 | 0.15269937 | -0.0166908 | 0.98668343 | 0.999976173 | -0.002548672 |
| YDL159W | STE7 | 0.15269937 | -0.0165148 | 0.9868238 | 0.999976173 | -0.002521804 |
| YMR070W | MOT3 | 0.15269937 | -0.0163109 | 0.98698648 | 0.999976173 | -0.002490666 |
| YAL066W | YAL066W | 0.15269937 | -0.0157956 | 0.98739759 | 0.999976173 | -0.002411975 |
| YAR028W | YAR028W | 0.15269937 | -0.0155047 | 0.98762962 | 0.999976173 | -0.002367565 |
| YGR254W | ENO1 | 0.15269937 | -0.0154693 | 0.98765792 | 0.999976173 | -0.002362148 |
| YDR256C | CTA1 | 0.15269937 | -0.0151008 | 0.98795191 | 0.999976173 | -0.002305878 |
| YER047C | SAP1 | 0.15269937 | -0.0149635 | 0.98806144 | 0.999976173 | -0.002284912 |
| YEL038W | UTR4 | 0.15269937 | -0.0141167 | 0.98873702 | 0.999976173 | -0.002155605 |
| YBR052C | RFS1 | 0.15269937 | -0.0137184 | 0.98905472 | 0.999976173 | -0.002094798 |
| YDR490C | PKH1 | 0.15269937 | -0.0136215 | 0.98913209 | 0.999976173 | -0.002079989 |
| YAL034C | FUN19 | 0.15269937 | -0.0135808 | 0.98916452 | 0.999976173 | -0.002073781 |
| YLR038C | COX12 | 0.15269937 | -0.0135103 | 0.98922079 | 0.999976173 | -0.002063011 |
| YPL041C | YPL041C | 0.15269937 | -0.0134985 | 0.98923022 | 0.999976173 | -0.002061207 |
| YOR284W | HUA2 | 0.15269937 | -0.0127312 | 0.98984233 | 0.999976173 | -0.001944048 |
| YIL096C | YIL096C | 0.15269937 | -0.0121023 | 0.99034409 | 0.999976173 | -0.001848014 |
| YKR033C | YKR033C | 0.15269937 | -0.0118684 | 0.99053072 | 0.999976173 | -0.001812294 |
| YIR019C | MUC1 | 0.15269937 | -0.0111912 | 0.99107099 | 0.999976173 | -0.001708888 |
| YIL012W | YIL012W | 0.15269937 | -0.0107017 | 0.9914615 | 0.999976173 | -0.001634148 |
| YJL208C | NUC1 | 0.15269937 | -0.0104557 | 0.99165778 | 0.999976173 | -0.001596581 |
| YMR068W | AVO2 | 0.15269937 | -0.0103637 | 0.99173122 | 0.999976173 | -0.001582525 |
| YML109W | ZDS2 | 0.15269937 | -0.0101761 | 0.99188089 | 0.999976173 | -0.001553879 |
| YNR050C | LYS9 | 0.15269937 | -0.0093778 | 0.99251776 | 0.999976173 | -0.001431988 |
| YGR039W | YGR039W | 0.15269937 | -0.0090875 | 0.99274941 | 0.999976173 | -0.001387652 |
| YOR017W | PET127 | 0.15269937 | -0.0088262 | 0.99295786 | 0.999976173 | -0.001347757 |
| YLR348C | DIC1 | 0.15269937 | -0.0084609 | 0.99324929 | 0.999976173 | -0.001291981 |
| YFR022W | ROG3 | 0.15269937 | -0.0081746 | 0.99347778 | 0.999976173 | -0.00124825 |
| YJL122W | ALB1 | 0.15269937 | -0.0080509 | 0.99357642 | 0.999976173 | -0.001229373 |
| YOR088W | NA | 0.15269937 | -0.0074263 | 0.99407479 | 0.999976173 | -0.00113399 |
| YIR023W | DAL81 | 0.15269937 | -0.0073744 | 0.99411623 | 0.999976173 | -0.001126059 |
| YNL294C | RIM21 | 0.15269937 | -0.0062987 | 0.99497442 | 0.999976173 | -0.000961813 |
| YOR068C | VAM10 | 0.15269937 | -0.0052436 | 0.99581626 | 0.999976173 | -0.000800696 |
| YOL062C | APM4 | 0.15269937 | -0.0047535 | 0.99620731 | 0.999976173 | -0.000725856 |
| YBR062C | YBR062C | 0.15269937 | -0.0045122 | 0.99639981 | 0.999976173 | -0.000689015 |
| YBR249C | ARO4 | 0.15269937 | -0.0044618 | 0.99644006 | 0.999976173 | -0.000681311 |
| YKL097C | YKL097C | 0.15269937 | -0.0042989 | 0.99657006 | 0.999976173 | -0.000656432 |

| | | | | | | |
|---|---|---|---|---|---|---|
| YDR410C | STE14 | 0.15269937 | -0.0038845 | 0.99690065 | 0.999976173 | -0.000593162 |
| YKR013W | PRY2 | 0.15269937 | -0.0037932 | 0.99697348 | 0.999976173 | -0.000579224 |
| YBL095W | YBL095W | 0.15269937 | -0.0036586 | 0.9970809 | 0.999976173 | -0.000558666 |
| YNL212W | VID27 | 0.15269937 | -0.0033117 | 0.99735768 | 0.999976173 | -0.000505694 |
| YPL152W | RRD2 | 0.15269937 | -0.0032878 | 0.99737674 | 0.999976173 | -0.000502045 |
| YNL200C | YNL200C | 0.15269937 | -0.0031223 | 0.99750879 | 0.999976173 | -0.000476775 |
| YIL112W | HOS4 | 0.15269937 | -0.0029081 | 0.99767969 | 0.999976173 | -0.000444067 |
| YOL009C | MDM12 | 0.15269937 | -0.0023795 | 0.99810144 | 0.999976173 | -0.000363352 |
| YHR047C | AAP1 | 0.15269937 | -0.0016003 | 0.99872319 | 0.999976173 | -0.000244358 |
| YBR289W | SNF5 | 0.15269937 | -0.001446 | 0.99884625 | 0.999976173 | -0.000220808 |
| YJL066C | MPM1 | 0.15269937 | -0.0012672 | 0.99898893 | 0.999976173 | -0.000193501 |
| YER004W | FMP52 | 0.15269937 | -0.0012146 | 0.99903089 | 0.999976173 | -0.00018547 |
| YER079W | YER079W | 0.15269937 | -0.0011414 | 0.99908928 | 0.999976173 | -0.000174295 |
| YHR113W | YHR113W | 0.15269937 | -0.0008897 | 0.99929011 | 0.999976173 | -0.00013586 |
| YJL197W | UBP12 | 0.15269937 | -0.000714 | 0.99943035 | 0.999976173 | -0.000109022 |
| YNL236W | SIN4 | 0.15269937 | -0.0005423 | 0.99956734 | 0.999976173 | -8.28E-05 |
| YJR090C | GRR1 | 0.15269937 | -0.0002791 | 0.99977728 | 0.999976173 | -4.26E-05 |
| YOR298W | MUM3 | 0.15269937 | -0.0002653 | 0.99978833 | 0.999976173 | -4.05E-05 |
| YKL101W | HSL1 | 0.15269937 | -0.0002494 | 0.99980101 | 0.999976173 | -3.81E-05 |
| YBR067C | TIP1 | 0.15269937 | -0.000238 | 0.99981014 | 0.999976173 | -3.63E-05 |
| YHL025W | SNF6 | NA | NA | NA | NA | 0 |
| YML111W | BUL2 | NA | NA | NA | NA | 0 |
| YPL162C | YPL162C | 0.15269937 | 2.99E-05 | 0.99997617 | 0.999976173 | 4.56E-06 |
| YPL225W | YPL225W | 0.15269937 | 0.00021302 | 0.99983004 | 0.999976173 | 3.25E-05 |
| YLR248W | RCK2 | 0.15269937 | 0.00022285 | 0.9998222 | 0.999976173 | 3.40E-05 |
| YDL114W | YDL114W | 0.15269937 | 0.00028587 | 0.99977191 | 0.999976173 | 4.37E-05 |
| YJL107C | YJL107C | 0.15269937 | 0.00041203 | 0.99967125 | 0.999976173 | 6.29E-05 |
| YIR028W | DAL4 | 0.15269937 | 0.00042671 | 0.99965954 | 0.999976173 | 6.52E-05 |
| YMR247C | RKR1 | 0.15269937 | 0.00057731 | 0.99953938 | 0.999976173 | 8.82E-05 |
| YOR309C | YOR309C | 0.10797476 | 0.00112995 | 0.99909844 | 0.999976173 | 0.000122006 |
| YOL035C | YOL035C | 0.15269937 | 0.00091204 | 0.99927231 | 0.999976173 | 0.000139268 |
| YBR019C | GAL10 | 0.15269937 | 0.00130327 | 0.99896016 | 0.999976173 | 0.000199008 |
| YKL177W | YKL177W | 0.15269937 | 0.00151604 | 0.99879039 | 0.999976173 | 0.000231498 |
| YPR076W | YPR076W | 0.15269937 | 0.0020348 | 0.99837648 | 0.999976173 | 0.000310712 |
| YGR135W | PRE9 | 0.15269937 | 0.00214411 | 0.99828926 | 0.999976173 | 0.000327405 |
| YMR132C | JLP2 | 0.15269937 | 0.00217387 | 0.99826552 | 0.999976173 | 0.000331948 |
| YGL195W | GCN1 | 0.15269937 | 0.00234588 | 0.99812828 | 0.999976173 | 0.000358214 |
| YEL010W | YEL010W | 0.15269937 | 0.00390002 | 0.99688827 | 0.999976173 | 0.00059553 |
| YFR007W | YFH7 | 0.15269937 | 0.00391331 | 0.99687767 | 0.999976173 | 0.000597559 |
| YDR110W | FOB1 | 0.15269937 | 0.00412083 | 0.9967121 | 0.999976173 | 0.000629247 |
| YGL226C-A | OST5 | 0.15269937 | 0.00414904 | 0.99668959 | 0.999976173 | 0.000633555 |
| YER158C | YER158C | 0.15269937 | 0.00430729 | 0.99656333 | 0.999976173 | 0.00065772 |
| YOL122C | SMF1 | 0.15269937 | 0.00453179 | 0.9963842 | 0.999976173 | 0.000692002 |
| YOR279C | RFM1 | 0.15269937 | 0.00471631 | 0.99623698 | 0.999976173 | 0.000720177 |
| YGR134W | CAF130 | 0.15269937 | 0.00476262 | 0.99620003 | 0.999976173 | 0.000727249 |
| YEL016C | NPP2 | 0.15269937 | 0.00482514 | 0.99615015 | 0.999976173 | 0.000736796 |
| YPL106C | SSE1 | 0.15269937 | 0.00482854 | 0.99614743 | 0.999976173 | 0.000737316 |
| YDR534C | FIT1 | 0.15269937 | 0.00484643 | 0.99613316 | 0.999976173 | 0.000740047 |
| YLR102C | APC9 | 0.15269937 | 0.00535337 | 0.99572869 | 0.999976173 | 0.000817455 |
| YGL005C | COG7 | 0.15269937 | 0.00555307 | 0.99556935 | 0.999976173 | 0.000847951 |
| YKR044W | UIP5 | 0.15269937 | 0.00558535 | 0.9955436 | 0.999976173 | 0.000852879 |
| YER049W | TPA1 | 0.15269937 | 0.00565894 | 0.99548488 | 0.999976173 | 0.000864117 |
| YBR291C | CTP1 | 0.15269937 | 0.00645883 | 0.99484668 | 0.999976173 | 0.00098626 |
| YOR042W | CUE5 | 0.15269937 | 0.00669653 | 0.99465703 | 0.999976173 | 0.001022556 |
| YGR229C | SMI1 | 0.15269937 | 0.00767836 | 0.99387367 | 0.999976173 | 0.001172481 |
| YNL193W | YNL193W | 0.15269937 | 0.00956961 | 0.99236475 | 0.999976173 | 0.001461273 |
| YJL013C | MAD3 | 0.15269937 | 0.01031848 | 0.99176727 | 0.999976173 | 0.001575625 |
| YDR135C | YCF1 | 0.15269937 | 0.01036005 | 0.9917341 | 0.999976173 | 0.001581973 |
| YBR068C | BAP2 | 0.15269937 | 0.01082518 | 0.99136301 | 0.999976173 | 0.001652997 |
| YPL261C | YPL261C | 0.15269937 | 0.01091671 | 0.99128998 | 0.999976173 | 0.001666975 |
| YPL073C | YPL073C | 0.15269937 | 0.01155044 | 0.99078438 | 0.999976173 | 0.001763744 |
| YPR043W | RPL43A | 0.15269937 | 0.01199318 | 0.99043115 | 0.999976173 | 0.00183135 |
| YPL025C | YPL025C | 0.15269937 | 0.01204479 | 0.99038997 | 0.999976173 | 0.001839232 |
| YLR395C | COX8 | 0.15269937 | 0.01253087 | 0.99000217 | 0.999976173 | 0.001913455 |

| | | | | | | |
|---|---|---|---|---|---|---|
| YHL035C | VMR1 | 0.15269937 | 0.01336467 | 0.98933696 | 0.999976173 | 0.002040776 |
| YKR043C | YKR043C | 0.15269937 | 0.01376043 | 0.98902122 | 0.999976173 | 0.002101208 |
| YDL201W | TRM8 | 0.15269937 | 0.01378516 | 0.98900149 | 0.999976173 | 0.002104985 |
| YJL161W | FMP33 | 0.15269937 | 0.01452656 | 0.98841 | 0.999976173 | 0.002218197 |
| YDR485C | VPS72 | 0.15269937 | 0.01514582 | 0.98791596 | 0.999976173 | 0.002312757 |
| YER128W | YER128W | 0.15269937 | 0.01652797 | 0.98681332 | 0.999976173 | 0.00252381 |
| YNL091W | NST1 | 0.15269937 | 0.01657832 | 0.98677315 | 0.999976173 | 0.002531499 |
| YER137C | YER137C | 0.15269937 | 0.01673549 | 0.98664776 | 0.999976173 | 0.002555499 |
| YOL046C | YOL046C | 0.15269937 | 0.01682183 | 0.98657889 | 0.999976173 | 0.002568683 |
| YCR102C | YCR102C | 0.15269937 | 0.01722199 | 0.98625965 | 0.999976173 | 0.002629788 |
| YPL017C | IRC15 | 0.15269937 | 0.0180668 | 0.9855857 | 0.999976173 | 0.002758789 |
| YLL015W | BPT1 | 0.15269937 | 0.01905898 | 0.9847942 | 0.999976173 | 0.002910294 |
| YLR352W | YLR352W | 0.15269937 | 0.01920958 | 0.98467406 | 0.999976173 | 0.002933291 |
| YML052W | SUR7 | 0.15269937 | 0.01933551 | 0.9845736 | 0.999976173 | 0.002952521 |
| YJR108W | ABM1 | 0.15269937 | 0.01956277 | 0.98439232 | 0.999976173 | 0.002987222 |
| YMR322C | SNO4 | 0.15269937 | 0.02048595 | 0.98365588 | 0.999976173 | 0.003128191 |
| YOL042W | NGL1 | 0.15269937 | 0.02056293 | 0.98359447 | 0.999976173 | 0.003139946 |
| YHR163W | SOL3 | 0.15269937 | 0.02057044 | 0.98358848 | 0.999976173 | 0.003141093 |
| YGR212W | SLI1 | 0.15269937 | 0.02304157 | 0.9816173 | 0.999976173 | 0.003518432 |
| YLR126C | YLR126C | 0.15269937 | 0.02340126 | 0.98133038 | 0.999976173 | 0.003573357 |
| YBR065C | ECM2 | 0.15269937 | 0.02438784 | 0.98054344 | 0.999976173 | 0.003724007 |
| YLR189C | ATG26 | 0.15269937 | 0.02442247 | 0.98051582 | 0.999976173 | 0.003729295 |
| YFR045W | YFR045W | 0.15269937 | 0.02444678 | 0.98049643 | 0.999976173 | 0.003733007 |
| YOR328W | PDR10 | 0.15269937 | 0.02456277 | 0.9804039 | 0.999976173 | 0.00375072 |
| YML074C | FPR3 | 0.15269937 | 0.02476027 | 0.98024637 | 0.999976173 | 0.003780878 |
| YNL239W | LAP3 | 0.15269937 | 0.02601582 | 0.97924492 | 0.999976173 | 0.003972599 |
| YOR324C | FRT1 | 0.15269937 | 0.02611853 | 0.979163 | 0.999976173 | 0.003988282 |
| YGL019W | CKB1 | 0.15269937 | 0.02622628 | 0.97907706 | 0.999976173 | 0.004004736 |
| YBL068W | PRS4 | 0.15269937 | 0.0270073 | 0.97845412 | 0.999976173 | 0.004123998 |
| YDR539W | YDR539W | 0.15269937 | 0.02708051 | 0.97839573 | 0.999976173 | 0.004135177 |
| YJR019C | TES1 | 0.15269937 | 0.02710887 | 0.97837311 | 0.999976173 | 0.004139508 |
| YOL150C | YOL150C | 0.15269937 | 0.02734106 | 0.97818792 | 0.999976173 | 0.004174962 |
| YNL316C | PHA2 | 0.15269937 | 0.02849308 | 0.97726911 | 0.999976173 | 0.004350875 |
| YDR272W | GLO2 | 0.15269937 | 0.02907528 | 0.97680478 | 0.999976173 | 0.004439777 |
| YPR171W | BSP1 | 0.15269937 | 0.02909105 | 0.9767922 | 0.999976173 | 0.004442184 |
| YOR019W | YOR019W | 0.15269937 | 0.02938923 | 0.97655439 | 0.999976173 | 0.004487717 |
| YFL063W | YFL063W | 0.15269937 | 0.0302469 | 0.97587038 | 0.999976173 | 0.004618683 |
| YGR101W | PCP1 | 0.15269937 | 0.03124457 | 0.97507473 | 0.999976173 | 0.004771026 |
| YMR250W | GAD1 | 0.15269937 | 0.03138482 | 0.97496289 | 0.999976173 | 0.004792442 |
| YGR014W | MSB2 | 0.15269937 | 0.0319925 | 0.97447828 | 0.999976173 | 0.004885234 |
| YNL077W | APJ1 | 0.15269937 | 0.03261213 | 0.97398415 | 0.999976173 | 0.004979851 |
| YGR088W | CTT1 | 0.15269937 | 0.03277446 | 0.9738547 | 0.999976173 | 0.005004639 |
| YDR132C | YDR132C | 0.15269937 | 0.03301163 | 0.97366557 | 0.999976173 | 0.005040856 |
| YLL021W | SPA2 | 0.15269937 | 0.03327548 | 0.97345516 | 0.999976173 | 0.005081145 |
| YPR193C | HPA2 | 0.15269937 | 0.03345596 | 0.97331124 | 0.999976173 | 0.005108704 |
| YOL027C | MDM38 | 0.15269937 | 0.03418409 | 0.97273061 | 0.999976173 | 0.005219889 |
| YOL089C | HAL9 | 0.15269937 | 0.03454052 | 0.9724464 | 0.999976173 | 0.005274316 |
| YNL165W | YNL165W | 0.15269937 | 0.03477196 | 0.97226185 | 0.999976173 | 0.005309656 |
| YLR373C | VID22 | 0.15269937 | 0.03483003 | 0.97221554 | 0.999976173 | 0.005318524 |
| YMR300C | ADE4 | 0.15269937 | 0.03520256 | 0.97191849 | 0.999976173 | 0.005375408 |
| YDR481C | PHO8 | 0.15269937 | 0.03533757 | 0.97181084 | 0.999976173 | 0.005396024 |
| YGL258W | VEL1 | 0.15269937 | 0.03544413 | 0.97172587 | 0.999976173 | 0.005412295 |
| YBR225W | YBR225W | 0.15269937 | 0.03555873 | 0.97163449 | 0.999976173 | 0.005429795 |
| YKR061W | KTR2 | 0.15269937 | 0.03606724 | 0.97122902 | 0.999976173 | 0.005507445 |
| YBR030W | RKM3 | 0.15269937 | 0.03629794 | 0.97104508 | 0.999976173 | 0.005542672 |
| YCR075C | ERS1 | 0.15269937 | 0.03657128 | 0.97082712 | 0.999976173 | 0.005584412 |
| YNL008C | ASI3 | 0.15269937 | 0.03712146 | 0.97038845 | 0.999976173 | 0.005668423 |
| YBL049W | MOH1 | 0.15269937 | 0.03713545 | 0.97037729 | 0.999976173 | 0.005670559 |
| YFL021W | GAT1 | 0.15269937 | 0.03728092 | 0.97026131 | 0.999976173 | 0.005692773 |
| YER182W | FMP10 | 0.15269937 | 0.03733803 | 0.97021577 | 0.999976173 | 0.005701493 |
| YGR012W | YGR012W | 0.15269937 | 0.03789142 | 0.96977455 | 0.999976173 | 0.005785995 |
| YBL085W | BOI1 | 0.15269937 | 0.03851715 | 0.96927565 | 0.999976173 | 0.005881545 |
| YER046W-A | YER046W-A | 0.15269937 | 0.03861871 | 0.96919468 | 0.999976173 | 0.005897053 |
| YMR255W | GFD1 | 0.15269937 | 0.03896505 | 0.96891855 | 0.999976173 | 0.005949939 |

| | | | | | | |
|---|---|---|---|---|---|---|
| YGL174W | BUD13 | 0.15269937 | 0.03926455 | 0.96867977 | 0.999976173 | 0.005995672 |
| YDR122W | KIN1 | 0.15269937 | 0.03955416 | 0.96844888 | 0.999976173 | 0.006039894 |
| YNR032C-A | HUB1 | 0.15269937 | 0.04034822 | 0.96781582 | 0.999976173 | 0.006161148 |
| YKL050C | YKL050C | 0.15269937 | 0.04055251 | 0.96765296 | 0.999976173 | 0.006192342 |
| YNR007C | ATG3 | 0.15269937 | 0.04058614 | 0.96762614 | 0.999976173 | 0.006197478 |
| YOR071C | NRT1 | 0.15269937 | 0.04075706 | 0.96748989 | 0.999976173 | 0.006223576 |
| YLR285W | NNT1 | 0.15269937 | 0.04131678 | 0.96704368 | 0.999976173 | 0.006309045 |
| YKL105C | YKL105C | 0.15269937 | 0.04237014 | 0.96620396 | 0.999976173 | 0.006469894 |
| YIL170W | HXT12 | 0.15269937 | 0.04263096 | 0.96599604 | 0.999976173 | 0.006509721 |
| YOR190W | SPR1 | 0.15269937 | 0.04327249 | 0.96548465 | 0.999976173 | 0.006607682 |
| YER174C | GRX4 | 0.15269937 | 0.0434322 | 0.96535734 | 0.999976173 | 0.00663207 |
| YDR019C | GCV1 | 0.15269937 | 0.04430757 | 0.96465958 | 0.999976173 | 0.006765738 |
| YBR264C | YPT10 | 0.15269937 | 0.04451643 | 0.9644931 | 0.999976173 | 0.00679763 |
| YDL083C | RPS16B | 0.15269937 | 0.04489736 | 0.96418947 | 0.999976173 | 0.006855799 |
| YCR044C | PER1 | 0.15269937 | 0.04490633 | 0.96418232 | 0.999976173 | 0.006857168 |
| YOL131W | YOL131W | 0.15269937 | 0.04493577 | 0.96415885 | 0.999976173 | 0.006861663 |
| YER067W | YER067W | 0.15269937 | 0.04587765 | 0.96340812 | 0.999976173 | 0.007005489 |
| YMR181C | YMR181C | 0.15269937 | 0.0462244 | 0.96313175 | 0.999976173 | 0.007058437 |
| YFR034C | PHO4 | 0.15269937 | 0.04637988 | 0.96300783 | 0.999976173 | 0.007082178 |
| YBR250W | SPO23 | 0.15269937 | 0.04675095 | 0.96271208 | 0.999976173 | 0.007138841 |
| YIL105C | SLM1 | 0.15269937 | 0.0471891 | 0.96236288 | 0.999976173 | 0.007205745 |
| YPL168W | YPL168W | 0.15269937 | 0.04814877 | 0.96159805 | 0.999976173 | 0.007352287 |
| YML075C | HMG1 | 0.15269937 | 0.04821814 | 0.96154277 | 0.999976173 | 0.007362879 |
| YGR266W | YGR266W | 0.15269937 | 0.04927915 | 0.96069722 | 0.999976173 | 0.007524895 |
| YNL335W | DDI3 | 0.15269937 | 0.05005468 | 0.9600792 | 0.999976173 | 0.007643317 |
| YOR378W | YOR378W | 0.15269937 | 0.05039961 | 0.95980434 | 0.999976173 | 0.007695989 |
| YNL049C | SFB2 | 0.15269937 | 0.05165792 | 0.95880167 | 0.999976173 | 0.007888131 |
| YGL053W | PRM8 | 0.15269937 | 0.05177689 | 0.95870687 | 0.999976173 | 0.007906298 |
| YKL065C | YET1 | 0.15269937 | 0.05263958 | 0.95801949 | 0.999976173 | 0.00803803 |
| YPR140W | TAZ1 | 0.15269937 | 0.05280989 | 0.95788379 | 0.999976173 | 0.008064037 |
| YMR003W | AIM34 | 0.15269937 | 0.05340995 | 0.95740569 | 0.999976173 | 0.008155666 |
| YJL135W | YJL135W | 0.15269937 | 0.05359486 | 0.95725837 | 0.999976173 | 0.008183901 |
| YOR152C | YOR152C | 0.15269937 | 0.05359526 | 0.95725805 | 0.999976173 | 0.008183962 |
| YNL289W | PCL1 | 0.15269937 | 0.05371786 | 0.95716037 | 0.999976173 | 0.008202683 |
| YFL010C | WWM1 | 0.15269937 | 0.05399491 | 0.95693964 | 0.999976173 | 0.008244988 |
| YDR213W | UPC2 | 0.15269937 | 0.05445321 | 0.95657451 | 0.999976173 | 0.00831497 |
| YHR039C | MSC7 | 0.15269937 | 0.05459288 | 0.95646323 | 0.999976173 | 0.008336299 |
| YDR271C | YDR271C | 0.15269937 | 0.05476612 | 0.95632522 | 0.999976173 | 0.008362751 |
| YBR101C | FES1 | 0.15269937 | 0.05545927 | 0.95577301 | 0.999976173 | 0.008468595 |
| YGL213C | SKI8 | 0.15269937 | 0.05580798 | 0.95549521 | 0.999976173 | 0.008521843 |
| YOL058W | ARG1 | 0.15269937 | 0.05607622 | 0.95528153 | 0.999976173 | 0.008562802 |
| YMR191W | SPG5 | 0.15269937 | 0.05638903 | 0.95503233 | 0.999976173 | 0.008610569 |
| YEL057C | YEL057C | 0.15269937 | 0.05657529 | 0.95488395 | 0.999976173 | 0.008639011 |
| YBR027C | YBR027C | 0.15269937 | 0.05667804 | 0.9548021 | 0.999976173 | 0.008654701 |
| YLR392C | YLR392C | 0.15269937 | 0.0567867 | 0.95471555 | 0.999976173 | 0.008671293 |
| YJL070C | YJL070C | 0.15269937 | 0.05694019 | 0.95459328 | 0.999976173 | 0.008694731 |
| YNL035C | YNL035C | 0.15269937 | 0.05695539 | 0.95458117 | 0.999976173 | 0.008697051 |
| YPL155C | KIP2 | 0.15269937 | 0.05700635 | 0.95454058 | 0.999976173 | 0.008704834 |
| YDR245W | MNN10 | 0.15269937 | 0.05730947 | 0.95429912 | 0.999976173 | 0.00875112 |
| YKL168C | KKQ8 | 0.15269937 | 0.05762644 | 0.95404663 | 0.999976173 | 0.008799521 |
| YJL020C | BBC1 | 0.15269937 | 0.05787289 | 0.95385032 | 0.999976173 | 0.008837154 |
| YKR012C | YKR012C | 0.15269937 | 0.05789893 | 0.95382958 | 0.999976173 | 0.00884113 |
| YOR082C | YOR082C | 0.15269937 | 0.05804286 | 0.95371494 | 0.999976173 | 0.008863108 |
| YJR109C | CPA2 | 0.10797476 | 0.08229559 | 0.9344123 | 0.999976173 | 0.008885846 |
| YMR178W | YMR178W | 0.15269937 | 0.05880393 | 0.95310873 | 0.999976173 | 0.008979322 |
| YER081W | SER3 | 0.15269937 | 0.05983306 | 0.95228906 | 0.999976173 | 0.00913647 |
| YDR222W | YDR222W | 0.15269937 | 0.06027129 | 0.95194004 | 0.999976173 | 0.009203387 |
| YJL153C | INO1 | 0.15269937 | 0.06048959 | 0.95176618 | 0.999976173 | 0.009236721 |
| YJR078W | BNA2 | 0.15269937 | 0.06067436 | 0.95161902 | 0.999976173 | 0.009264936 |
| YFL030W | AGX1 | 0.15269937 | 0.06080023 | 0.95151878 | 0.999976173 | 0.009284156 |
| YMR295C | YMR295C | 0.15269937 | 0.06109913 | 0.95128074 | 0.999976173 | 0.009329798 |
| YDR059C | UBC5 | 0.15269937 | 0.06116446 | 0.9512287 | 0.999976173 | 0.009339775 |
| YOR233W | KIN4 | 0.15269937 | 0.06129151 | 0.95112752 | 0.999976173 | 0.009359175 |
| YDL037C | BSC1 | 0.15269937 | 0.06175189 | 0.95076089 | 0.999976173 | 0.009429475 |

| | | | | | | |
|---|---|---|---|---|---|---|
| YDR093W | DNF2 | 0.15269937 | 0.06183353 | 0.95069588 | 0.999976173 | 0.00944194 |
| YKL156W | RPS27A | 0.15269937 | 0.06209725 | 0.95048587 | 0.999976173 | 0.00948221 |
| YDR219C | MFB1 | 0.15269937 | 0.06300899 | 0.94975984 | 0.999976173 | 0.009621432 |
| YJL073W | JEM1 | 0.15269937 | 0.06301647 | 0.94975388 | 0.999976173 | 0.009622575 |
| YJR142W | YJR142W | 0.15269937 | 0.06351807 | 0.94935446 | 0.999976173 | 0.009699168 |
| YPL274W | SAM3 | 0.15269937 | 0.0635206 | 0.94935245 | 0.999976173 | 0.009699555 |
| YDL125C | HNT1 | 0.15269937 | 0.06393088 | 0.94902576 | 0.999976173 | 0.009762205 |
| YGR197C | SNG1 | 0.15269937 | 0.06479305 | 0.94833928 | 0.999976173 | 0.009893857 |
| YJR031C | GEA1 | 0.15269937 | 0.06533128 | 0.94791075 | 0.999976173 | 0.009976044 |
| YOR029W | YOR029W | 0.15269937 | 0.06544129 | 0.94782315 | 0.999976173 | 0.009992844 |
| YHR111W | UBA4 | 0.15269937 | 0.06550119 | 0.94777546 | 0.999976173 | 0.01000199 |
| YML020W | YML020W | 0.15269937 | 0.0667731 | 0.94676285 | 0.999976173 | 0.010196211 |
| YIL030C | SSM4 | 0.15269937 | 0.06792688 | 0.94584437 | 0.999976173 | 0.010372391 |
| YDL127W | PCL2 | 0.15269937 | 0.06798761 | 0.94579603 | 0.999976173 | 0.010381664 |
| YOR367W | SCP1 | 0.15269937 | 0.06798761 | 0.94579602 | 0.999976173 | 0.010381665 |
| YKR067W | GPT2 | 0.15269937 | 0.06838585 | 0.94547901 | 0.999976173 | 0.010442476 |
| YLR096W | KIN2 | 0.15269937 | 0.06869575 | 0.94523233 | 0.999976173 | 0.010489798 |
| YKL218C | SRY1 | 0.15269937 | 0.07004477 | 0.94415857 | 0.999976173 | 0.010695791 |
| YBR100W | NA | 0.15269937 | 0.07042965 | 0.94385223 | 0.999976173 | 0.010754563 |
| YOR188W | MSB1 | 0.15269937 | 0.0715829 | 0.9429344 | 0.999976173 | 0.010930663 |
| YBR048W | RPS11B | 0.15269937 | 0.07206169 | 0.94255337 | 0.999976173 | 0.011003774 |
| YPL176C | TRE1 | 0.15269937 | 0.0723305 | 0.94233945 | 0.999976173 | 0.011044821 |
| YOR025W | HST3 | 0.15269937 | 0.07252041 | 0.94218832 | 0.999976173 | 0.011073821 |
| YMR193C-A | YMR193C-A | 0.15269937 | 0.07309993 | 0.94172717 | 0.999976173 | 0.011162312 |
| YNR051C | BRE5 | 0.15269937 | 0.0731814 | 0.94166233 | 0.999976173 | 0.011174754 |
| YJR150C | DAN1 | 0.15269937 | 0.07319933 | 0.94164807 | 0.999976173 | 0.011177491 |
| YLR021W | IRC25 | 0.15269937 | 0.07320679 | 0.94164213 | 0.999976173 | 0.01117863 |
| YDR371W | CTS2 | 0.15269937 | 0.07323701 | 0.94161808 | 0.999976173 | 0.011183245 |
| YOR240W | NA | 0.15269937 | 0.07344974 | 0.9414488 | 0.999976173 | 0.011215729 |
| YBL063W | KIP1 | 0.15269937 | 0.07368783 | 0.94125935 | 0.999976173 | 0.011252085 |
| YDR254W | CHL4 | 0.15269937 | 0.07412652 | 0.94091029 | 0.999976173 | 0.011319072 |
| YJR010C-A | SPC1 | 0.15269937 | 0.07483217 | 0.94034882 | 0.999976173 | 0.011426825 |
| YIL009C-A | 7 | 0.15269937 | 0.07533692 | 0.93994723 | 0.999976173 | 0.0115039 |
| YLR238W | FAR10 | 0.15269937 | 0.0755599 | 0.93976982 | 0.999976173 | 0.011537949 |
| YDL119C | YDL119C | 0.15269937 | 0.07580681 | 0.93957339 | 0.999976173 | 0.011575651 |
| YGR085C | RPL11B | 0.15269937 | 0.07596956 | 0.9394439 | 0.999976173 | 0.011600503 |
| YJR030C | YJR030C | 0.15269937 | 0.07632543 | 0.93916078 | 0.999976173 | 0.011654845 |
| YIL074C | SER33 | 0.15269937 | 0.0763545 | 0.93913766 | 0.999976173 | 0.011659283 |
| YNL192W | CHS1 | 0.15269937 | 0.07685435 | 0.93874001 | 0.999976173 | 0.011735611 |
| YMR294W-A | YMR294W-A | 0.15269937 | 0.07777072 | 0.93801104 | 0.999976173 | 0.01187554 |
| YDR317W | HIM1 | 0.15269937 | 0.07809856 | 0.93775026 | 0.999976173 | 0.0119256 |
| YMR190C | SGS1 | 0.15269937 | 0.07863085 | 0.93732685 | 0.999976173 | 0.012006882 |
| YDR491C | YDR491C | 0.15269937 | 0.07877248 | 0.93721421 | 0.999976173 | 0.012028507 |
| YCR027C | RHB1 | 0.15269937 | 0.07916219 | 0.93690423 | 0.999976173 | 0.012088017 |
| YBR239C | YBR239C | 0.15269937 | 0.07924403 | 0.93683914 | 0.999976173 | 0.012100513 |
| YFR046C | CNN1 | 0.15269937 | 0.08017914 | 0.9360954 | 0.999976173 | 0.012243303 |
| YNL271C | BNI1 | 0.15269937 | 0.08024689 | 0.93604152 | 0.999976173 | 0.012253649 |
| YFR014C | CMK1 | 0.15269937 | 0.08037187 | 0.93594212 | 0.999976173 | 0.012272733 |
| YDL091C | UBX3 | 0.15269937 | 0.08054437 | 0.93580493 | 0.999976173 | 0.012299074 |
| YJL181W | YJL181W | 0.15269937 | 0.08115366 | 0.93532038 | 0.999976173 | 0.012392112 |
| YJL165C | HAL5 | 0.15269937 | 0.081965 | 0.93467518 | 0.999976173 | 0.012516003 |
| YHR080C | YHR080C | 0.15269937 | 0.08251974 | 0.93423406 | 0.999976173 | 0.012600713 |
| YDR441C | APT2 | 0.15269937 | 0.08272389 | 0.93407173 | 0.999976173 | 0.012631885 |
| YKL185W | ASH1 | 0.15269937 | 0.0830577 | 0.9338063 | 0.999976173 | 0.012682858 |
| YPL098C | MGR2 | 0.15269937 | 0.08347781 | 0.93347227 | 0.999976173 | 0.012747008 |
| YLR082C | SRL2 | 0.15269937 | 0.08381519 | 0.93320402 | 0.999976173 | 0.012798527 |
| YER119C | AVT6 | 0.15269937 | 0.08408958 | 0.93298586 | 0.999976173 | 0.012840426 |
| YJL123C | MTC1 | 0.15269937 | 0.08416023 | 0.93292969 | 0.999976173 | 0.012851214 |
| YFR026C | ULI1 | 0.15269937 | 0.08427908 | 0.9328352 | 0.999976173 | 0.012869362 |
| YJL023C | PET130 | 0.15269937 | 0.08531744 | 0.93200968 | 0.999976173 | 0.013027919 |
| YOL106W | YOL106W | 0.15269937 | 0.0854172 | 0.93193038 | 0.999976173 | 0.013043152 |
| YMR096W | SNZ1 | 0.15269937 | 0.08643141 | 0.93112414 | 0.999976173 | 0.013198022 |
| YNL042W | BOP3 | 0.15269937 | 0.08652609 | 0.93104888 | 0.999976173 | 0.013212479 |
| YLR405W | DUS4 | 0.15269937 | 0.08653226 | 0.93104398 | 0.999976173 | 0.013213422 |

| | | | | | | |
|---|---|---|---|---|---|---|
| YGL016W | KAP122 | 0.15269937 | 0.08688831 | 0.93076096 | 0.999976173 | 0.01326779 |
| YML119W | YML119W | 0.15269937 | 0.0870188 | 0.93065724 | 0.999976173 | 0.013287715 |
| YDR285W | ZIP1 | 0.15269937 | 0.08717659 | 0.93053182 | 0.999976173 | 0.01331181 |
| YJR025C | BNA1 | 0.15269937 | 0.08769204 | 0.93012212 | 0.999976173 | 0.013390519 |
| YBR214W | SDS24 | 0.15269937 | 0.08823466 | 0.92969085 | 0.999976173 | 0.013473377 |
| YOL163W | YOL163W | 0.15269937 | 0.08910198 | 0.92900155 | 0.999976173 | 0.013605816 |
| YMR067C | UBX4 | 0.15269937 | 0.0894025 | 0.92876273 | 0.999976173 | 0.013651705 |
| YBL043W | ECM13 | 0.15269937 | 0.09035565 | 0.9280053 | 0.999976173 | 0.01379725 |
| YOL049W | GSH2 | 0.15269937 | 0.09121306 | 0.927324 | 0.999976173 | 0.013928176 |
| YIR005W | IST3 | 0.15269937 | 0.09124107 | 0.92730174 | 0.999976173 | 0.013932454 |
| YOR381W | FRE3 | 0.15269937 | 0.09150122 | 0.92709504 | 0.999976173 | 0.013972178 |
| YGR096W | TPC1 | 0.15269937 | 0.09218709 | 0.92655011 | 0.999976173 | 0.01407691 |
| YBR299W | MAL32 | 0.15269937 | 0.09258049 | 0.92623756 | 0.999976173 | 0.014136981 |
| YAL026C | DRS2 | 0.15269937 | 0.09298168 | 0.92591883 | 0.999976173 | 0.014198244 |
| YIL164C | NIT1 | 0.15269937 | 0.09303914 | 0.92587319 | 0.999976173 | 0.014207017 |
| YOR161C | PNS1 | 0.15269937 | 0.09322842 | 0.92572282 | 0.999976173 | 0.01423592 |
| YOL013C | HRD1 | 0.15269937 | 0.09346341 | 0.92553614 | 0.999976173 | 0.014271803 |
| YEL017C-A | PMP2 | 0.15269937 | 0.09367171 | 0.92537067 | 0.999976173 | 0.01430361 |
| YGR196C | FYV8 | 0.15269937 | 0.09401693 | 0.92509643 | 0.999976173 | 0.014356326 |
| YBR137W | YBR137W | 0.15269937 | 0.0947732 | 0.9244957 | 0.999976173 | 0.014471808 |
| YPR126C | YPR126C | 0.15269937 | 0.09552387 | 0.92389947 | 0.999976173 | 0.014586434 |
| YPL061W | ALD6 | 0.15269937 | 0.09624051 | 0.9233303 | 0.999976173 | 0.014695865 |
| YER093C-A | AIM11 | 0.15269937 | 0.09732198 | 0.92247146 | 0.999976173 | 0.014861004 |
| YNL054W | VAC7 | 0.15269937 | 0.09769773 | 0.92217308 | 0.999976173 | 0.014918381 |
| YMR053C | STB2 | 0.15269937 | 0.09805448 | 0.9218898 | 0.999976173 | 0.014972856 |
| YFL053W | DAK2 | 0.15269937 | 0.09807208 | 0.92187581 | 0.999976173 | 0.014975545 |
| YCL069W | VBA3 | 0.15269937 | 0.0981931 | 0.92177972 | 0.999976173 | 0.014994025 |
| YML096W | YML096W | 0.15269937 | 0.09839005 | 0.92162334 | 0.999976173 | 0.015024098 |
| YLR209C | PNP1 | 0.15269937 | 0.0983908 | 0.92162275 | 0.999976173 | 0.015024212 |
| YNL318C | HXT14 | 0.15269937 | 0.09849048 | 0.9215436 | 0.999976173 | 0.015039433 |
| YDL106C | PHO2 | 0.15269937 | 0.09918451 | 0.92099254 | 0.999976173 | 0.015145412 |
| YJL198W | PHO90 | 0.15269937 | 0.09958152 | 0.92067734 | 0.999976173 | 0.015206034 |
| YDR488C | PAC11 | 0.15269937 | 0.0999809 | 0.92036026 | 0.999976173 | 0.01526702 |
| YDR379W | RGA2 | 0.15269937 | 0.10014849 | 0.92022722 | 0.999976173 | 0.01529261 |
| YGR205W | YGR205W | 0.15269937 | 0.10035381 | 0.92006422 | 0.999976173 | 0.015323963 |
| YER085C | YER085C | 0.15269937 | 0.10041824 | 0.92001307 | 0.999976173 | 0.015333801 |
| YML101C | CUE4 | 0.15269937 | 0.10132877 | 0.91929027 | 0.999976173 | 0.015472838 |
| YHR021W-A | ECM12 | 0.15269937 | 0.10136158 | 0.91926422 | 0.999976173 | 0.015477848 |
| YCL026C-A | FRM2 | 0.15269937 | 0.10349964 | 0.91756724 | 0.999976173 | 0.01580433 |
| YDR535C | YDR535C | 0.15269937 | 0.10382983 | 0.9173052 | 0.999976173 | 0.01585475 |
| YDR525W-A | SNA2 | 0.15269937 | 0.10424793 | 0.9169734 | 0.999976173 | 0.015918593 |
| YOR171C | LCB4 | 0.15269937 | 0.1043851 | 0.91686456 | 0.999976173 | 0.015939538 |
| YJL030W | MAD2 | 0.15269937 | 0.10450207 | 0.91677174 | 0.999976173 | 0.015957399 |
| YCL013W | NA | 0.15269937 | 0.10510226 | 0.91629548 | 0.999976173 | 0.016049048 |
| YJR130C | STR2 | 0.15269937 | 0.10601743 | 0.91556934 | 0.999976173 | 0.016188794 |
| YPL273W | SAM4 | 0.15269937 | 0.10605842 | 0.91553682 | 0.999976173 | 0.016195053 |
| YGR227W | DIE2 | 0.15269937 | 0.10647436 | 0.91520682 | 0.999976173 | 0.016258567 |
| YOR237W | HES1 | 0.15269937 | 0.10648007 | 0.91520229 | 0.999976173 | 0.01625944 |
| YDR538W | PAD1 | 0.15269937 | 0.10668381 | 0.91504066 | 0.999976173 | 0.016290549 |
| YPR014C | YPR014C | 0.15269937 | 0.10736547 | 0.91449988 | 0.999976173 | 0.016394639 |
| YPR097W | YPR097W | 0.15269937 | 0.10740527 | 0.91446831 | 0.999976173 | 0.016400716 |
| YCR010C | ADY2 | 0.15269937 | 0.10765552 | 0.91426979 | 0.999976173 | 0.01643893 |
| YGR203W | YGR203W | 0.15269937 | 0.10865905 | 0.91347377 | 0.999976173 | 0.016592167 |
| YGL146C | RRT6 | 0.15269937 | 0.1088782 | 0.91329994 | 0.999976173 | 0.016625632 |
| YGL217C | YGL217C | 0.15269937 | 0.10973149 | 0.91262318 | 0.999976173 | 0.016755929 |
| YBR047W | FMP23 | 0.15269937 | 0.10987935 | 0.91250591 | 0.999976173 | 0.016778507 |
| YJR137C | MET5 | 0.15269937 | 0.11027829 | 0.91218953 | 0.999976173 | 0.016839425 |
| YJL059W | YHC3 | 0.15269937 | 0.11036447 | 0.91212118 | 0.999976173 | 0.016852585 |
| YLR150W | STM1 | 0.15269937 | 0.11044478 | 0.9120575 | 0.999976173 | 0.016864847 |
| YPL111W | CAR1 | 0.15269937 | 0.11078726 | 0.91178591 | 0.999976173 | 0.016917144 |
| YFR035C | YFR035C | 0.15269937 | 0.1108324 | 0.91175011 | 0.999976173 | 0.016924038 |
| YMR284W | 33 | 0.15269937 | 0.1110449 | 0.9115816 | 0.999976173 | 0.016956485 |
| YNR042W | YNR042W | 0.15269937 | 0.11106064 | 0.91156912 | 0.999976173 | 0.016958889 |
| YNR065C | YNR065C | 0.15269937 | 0.11148868 | 0.91122971 | 0.999976173 | 0.01702425 |

| | | | | | | |
|---|---|---|---|---|---|---|
| YDR511W | ACN9 | 0.15269937 | 0.11165253 | 0.91109979 | 0.999976173 | 0.01704927 |
| YER060W | FCY21 | 0.15269937 | 0.11180631 | 0.91097785 | 0.999976173 | 0.017072753 |
| YLR097C | HRT3 | 0.15269937 | 0.1120471 | 0.91078693 | 0.999976173 | 0.017109521 |
| YKR021W | ALY1 | 0.15269937 | 0.11224332 | 0.91063135 | 0.999976173 | 0.017139484 |
| YEL060C | PRB1 | 0.15269937 | 0.11266693 | 0.9102955 | 0.999976173 | 0.017204168 |
| YNL297C | MON2 | 0.15269937 | 0.11273519 | 0.91024138 | 0.999976173 | 0.017214592 |
| YOR348C | PUT4 | 0.15269937 | 0.11293499 | 0.91008297 | 0.999976173 | 0.017245101 |
| YJR087W | YJR087W | 0.15269937 | 0.11299642 | 0.91003427 | 0.999976173 | 0.017254482 |
| YKR035C | OPI8 | 0.15269937 | 0.11317005 | 0.90989661 | 0.999976173 | 0.017280995 |
| YGR059W | SPR3 | 0.15269937 | 0.11369729 | 0.90947864 | 0.999976173 | 0.017361504 |
| YCL001W | RER1 | 0.15269937 | 0.11370622 | 0.90947156 | 0.999976173 | 0.017362868 |
| YCL062W | NA | 0.15269937 | 0.11436528 | 0.90894912 | 0.999976173 | 0.017463506 |
| YHR204W | MNL1 | 0.15269937 | 0.11469631 | 0.90868673 | 0.999976173 | 0.017514054 |
| YEL004W | YEA4 | 0.15269937 | 0.11472419 | 0.90866463 | 0.999976173 | 0.017518311 |
| YHR034C | PIH1 | 0.15269937 | 0.11512092 | 0.90835017 | 0.999976173 | 0.017578891 |
| YOL147C | PEX11 | 0.15269937 | 0.11528112 | 0.9082232 | 0.999976173 | 0.017603354 |
| YMR166C | YMR166C | 0.15269937 | 0.11560246 | 0.90796851 | 0.999976173 | 0.017652422 |
| YLR286C | CTS1 | 0.15269937 | 0.1156148 | 0.90795873 | 0.999976173 | 0.017654306 |
| YGL034C | YGL034C | 0.15269937 | 0.11580302 | 0.90780955 | 0.999976173 | 0.017683048 |
| YJL038C | LOH1 | 0.15269937 | 0.11632324 | 0.90739727 | 0.999976173 | 0.017762485 |
| YGL105W | ARC1 | 0.15269937 | 0.11688238 | 0.90695416 | 0.999976173 | 0.017847866 |
| YLR221C | RSA3 | 0.15269937 | 0.1169932 | 0.90686635 | 0.999976173 | 0.017864787 |
| YEL041W | YEF1 | 0.15269937 | 0.11739653 | 0.90654674 | 0.999976173 | 0.017926376 |
| YLR422W | YLR422W | 0.15269937 | 0.11767912 | 0.90632282 | 0.999976173 | 0.017969528 |
| YCL023C | YCL023C | 0.15269937 | 0.11779992 | 0.9062271 | 0.999976173 | 0.017987973 |
| YLR280C | YLR280C | 0.15269937 | 0.1179943 | 0.90607309 | 0.999976173 | 0.018017654 |
| YEL037C | RAD23 | 0.15269937 | 0.11850242 | 0.90567049 | 0.999976173 | 0.018095245 |
| YDR274C | YDR274C | 0.15269937 | 0.1191215 | 0.90518002 | 0.999976173 | 0.018189777 |
| YER073W | ALD5 | 0.15269937 | 0.11931436 | 0.90502723 | 0.999976173 | 0.018219227 |
| YDR215C | YDR215C | 0.15269937 | 0.11945934 | 0.90491238 | 0.999976173 | 0.018241366 |
| YDR214W | AHA1 | 0.15269937 | 0.11956846 | 0.90482593 | 0.999976173 | 0.018258028 |
| YDL053C | PBP4 | 0.15269937 | 0.11978911 | 0.90465114 | 0.999976173 | 0.018291722 |
| YKL053C-A | MDM35 | 0.15269937 | 0.1201017 | 0.90440351 | 0.999976173 | 0.018339454 |
| YHR043C | DOG2 | 0.15269937 | 0.12021237 | 0.90431586 | 0.999976173 | 0.018356352 |
| YMR230W | RPS10B | 0.15269937 | 0.12035034 | 0.90420656 | 0.999976173 | 0.01837742 |
| YDR351W | SBE2 | 0.15269937 | 0.12169739 | 0.90313963 | 0.999976173 | 0.018583114 |
| YGR122W | YGR122W | 0.15269937 | 0.12220989 | 0.90273374 | 0.999976173 | 0.018661373 |
| YMR029C | FAR8 | 0.15269937 | 0.12224874 | 0.90270298 | 0.999976173 | 0.018667304 |
| YGR289C | MAL11 | 0.15269937 | 0.12410893 | 0.90122999 | 0.999976173 | 0.018951355 |
| YDR402C | DIT2 | 0.15269937 | 0.1250545 | 0.90048137 | 0.999976173 | 0.019095743 |
| YDR198C | RKM2 | 0.15269937 | 0.12525124 | 0.90032562 | 0.999976173 | 0.019125785 |
| YBR165W | UBS1 | 0.10797476 | 0.17734763 | 0.85923673 | 0.999976173 | 0.019149067 |
| YKL026C | GPX1 | 0.15269937 | 0.12541839 | 0.9001933 | 0.999976173 | 0.019151309 |
| YGL159W | YGL159W | 0.15269937 | 0.12563874 | 0.90001887 | 0.999976173 | 0.019184956 |
| YDL010W | GRX6 | 0.15269937 | 0.12590733 | 0.89980626 | 0.999976173 | 0.019225969 |
| YIL060W | YIL060W | 0.15269937 | 0.12710317 | 0.89885973 | 0.999976173 | 0.019408574 |
| YMR320W | YMR320W | 0.15269937 | 0.12733797 | 0.8986739 | 0.999976173 | 0.019444426 |
| YDL238C | GUD1 | 0.15269937 | 0.12750475 | 0.89854191 | 0.999976173 | 0.019469894 |
| YLR368W | MDM30 | 0.15269937 | 0.1277569 | 0.89834235 | 0.999976173 | 0.019508397 |
| YEL053C | MAK10 | 0.15269937 | 0.12781602 | 0.89829556 | 0.999976173 | 0.019517426 |
| YFL011W | HXT10 | 0.15269937 | 0.12785195 | 0.89826713 | 0.999976173 | 0.019522911 |
| YDR378C | LSM6 | 0.15269937 | 0.12810823 | 0.89806432 | 0.999976173 | 0.019562045 |
| YOL121C | RPS19A | 0.15269937 | 0.12833903 | 0.89788168 | 0.999976173 | 0.019597288 |
| YDR400W | URH1 | 0.15269937 | 0.1287401 | 0.8975643 | 0.999976173 | 0.019658532 |
| YMR152W | YIM1 | 0.15269937 | 0.12919516 | 0.89720423 | 0.999976173 | 0.019728019 |
| YEL047C | YEL047C | 0.15269937 | 0.12922032 | 0.89718432 | 0.999976173 | 0.019731861 |
| YOR064C | YNG1 | 0.15269937 | 0.13038541 | 0.89626253 | 0.999976173 | 0.019909769 |
| YCR014C | POL4 | 0.15269937 | 0.1306761 | 0.89603256 | 0.999976173 | 0.019954157 |
| YOR047C | STD1 | 0.15269937 | 0.13072036 | 0.89599754 | 0.999976173 | 0.019960915 |
| YOR263C | YOR263C | 0.15269937 | 0.13072742 | 0.89599196 | 0.999976173 | 0.019961994 |
| YKR035W-A | DID2 | 0.15269937 | 0.13134941 | 0.89549993 | 0.999976173 | 0.020056972 |
| YNL141W | AAH1 | 0.15269937 | 0.13278922 | 0.89436111 | 0.999976173 | 0.020276829 |
| YIL165C | YIL165C | 0.15269937 | 0.13282446 | 0.89433324 | 0.999976173 | 0.020282211 |
| YML058C-A | NA | 0.15269937 | 0.13419535 | 0.89324914 | 0.999976173 | 0.020491545 |

| | | | | | | |
|---|---|---|---|---|---|---|
| YMR282C | AEP2 | 0.15269937 | 0.13422676 | 0.89322431 | 0.999976173 | 0.02049634 |
| YOR178C | GAC1 | 0.15269937 | 0.13503263 | 0.89258713 | 0.999976173 | 0.020619397 |
| YHR031C | RRM3 | 0.15269937 | 0.13536395 | 0.89232518 | 0.999976173 | 0.020669989 |
| YJR038C | YJR038C | 0.15269937 | 0.13541328 | 0.89228617 | 0.999976173 | 0.020677523 |
| YPL021W | ECM23 | 0.15269937 | 0.13611162 | 0.8917341 | 0.999976173 | 0.020784158 |
| YGL259W | YPS5 | 0.15269937 | 0.1370582 | 0.89098586 | 0.999976173 | 0.0209287 |
| YGL160W | AIM14 | 0.15269937 | 0.1372548 | 0.89083047 | 0.999976173 | 0.02095872 |
| YHR087W | RTC3 | 0.15269937 | 0.13765856 | 0.89051134 | 0.999976173 | 0.021020375 |
| YMR274C | RCE1 | 0.15269937 | 0.13799195 | 0.89024785 | 0.999976173 | 0.021071283 |
| YEL064C | AVT2 | 0.15269937 | 0.13815244 | 0.89012102 | 0.999976173 | 0.02109579 |
| YKL005C | BYE1 | 0.15269937 | 0.13816161 | 0.89011377 | 0.999976173 | 0.02109719 |
| YER096W | SHC1 | 0.15269937 | 0.13859873 | 0.88976832 | 0.999976173 | 0.021163938 |
| YPL227C | ALG5 | 0.15269937 | 0.13860674 | 0.88976199 | 0.999976173 | 0.021165161 |
| YNL128W | TEP1 | 0.15269937 | 0.13903633 | 0.88942253 | 0.999976173 | 0.021230759 |
| YML099C | ARG81 | 0.15269937 | 0.13924122 | 0.88926062 | 0.999976173 | 0.021262046 |
| YBR066C | NRG2 | 0.15269937 | 0.13950497 | 0.88905221 | 0.999976173 | 0.02130232 |
| YDR440W | DOT1 | 0.15269937 | 0.14002307 | 0.88864286 | 0.999976173 | 0.021381433 |
| YIR038C | GTT1 | 0.15269937 | 0.14015579 | 0.888538 | 0.999976173 | 0.0214017 |
| YGL203C | KEX1 | 0.15269937 | 0.14031697 | 0.88841065 | 0.999976173 | 0.021426312 |
| YLR385C | SWC7 | 0.15269937 | 0.14038627 | 0.8883559 | 0.999976173 | 0.021436894 |
| YBL036C | YBL036C | 0.15269937 | 0.14046879 | 0.8882907 | 0.999976173 | 0.021449495 |
| YPL030W | TRM44 | 0.15269937 | 0.1410079 | 0.8878648 | 0.999976173 | 0.021531817 |
| YFL034W | YFL034W | 0.15269937 | 0.14188174 | 0.88717452 | 0.999976173 | 0.021665252 |
| YKL017C | HCS1 | 0.15269937 | 0.14190781 | 0.88715393 | 0.999976173 | 0.021669232 |
| YDL052C | SLC1 | 0.15269937 | 0.14203726 | 0.88705168 | 0.999976173 | 0.021689 |
| YDR066C | RTR2 | 0.15269937 | 0.14208596 | 0.88701321 | 0.999976173 | 0.021696436 |
| YOL018C | TLG2 | 0.15269937 | 0.14229148 | 0.88685089 | 0.999976173 | 0.021727818 |
| YHR129C | ARP1 | 0.15269937 | 0.14269424 | 0.88653278 | 0.999976173 | 0.02178932 |
| YMR183C | SSO2 | 0.15269937 | 0.14319151 | 0.88614005 | 0.999976173 | 0.021865253 |
| YBR044C | TCM62 | 0.15269937 | 0.14339569 | 0.88597881 | 0.999976173 | 0.02189643 |
| YDR117C | TMA64 | 0.15269937 | 0.14379272 | 0.88566527 | 0.999976173 | 0.021957056 |
| YFL018C | LPD1 | 0.15269937 | 0.14425373 | 0.88530124 | 0.999976173 | 0.022027453 |
| YAL065C | YAL065C | 0.15269937 | 0.14448962 | 0.88511497 | 0.999976173 | 0.022063474 |
| YKL092C | BUD2 | 0.15269937 | 0.14470692 | 0.8849434 | 0.999976173 | 0.022096655 |
| YDR532C | YDR532C | 0.15269937 | 0.14476369 | 0.88489858 | 0.999976173 | 0.022105324 |
| YKR090W | PXL1 | 0.15269937 | 0.14548693 | 0.88432756 | 0.999976173 | 0.022215762 |
| YDL117W | CYK3 | 0.15269937 | 0.1458177 | 0.88406643 | 0.999976173 | 0.022266271 |
| YCR073C | SSK22 | 0.15269937 | 0.14624173 | 0.8837317 | 0.999976173 | 0.022331019 |
| YLR283W | YLR283W | 0.15269937 | 0.14631029 | 0.88367758 | 0.999976173 | 0.022341488 |
| YDL099W | BUG1 | 0.15269937 | 0.14641752 | 0.88359294 | 0.999976173 | 0.022357863 |
| YLR351C | NIT3 | 0.15269937 | 0.14644513 | 0.88357114 | 0.999976173 | 0.022362079 |
| YMR251W | GTO3 | 0.15269937 | 0.14653046 | 0.88350378 | 0.999976173 | 0.022375109 |
| YNL277W | MET2 | 0.15269937 | 0.14672645 | 0.88334909 | 0.999976173 | 0.022405035 |
| YLR313C | SPH1 | 0.15269937 | 0.14685616 | 0.8832467 | 0.999976173 | 0.022424843 |
| YPL099C | AIM43 | 0.15269937 | 0.14726938 | 0.88292055 | 0.999976173 | 0.022487941 |
| YHR014W | SPO13 | 0.15269937 | 0.1475286 | 0.88271596 | 0.999976173 | 0.022527524 |
| YPR020W | ATP20 | 0.15269937 | 0.14945131 | 0.88119869 | 0.999976173 | 0.022821121 |
| YJL055W | YJL055W | 0.15269937 | 0.14996627 | 0.88079239 | 0.999976173 | 0.022899755 |
| YDR524C | AGE1 | 0.15269937 | 0.1504059 | 0.88044556 | 0.999976173 | 0.022966885 |
| YDL118W | YDL118W | 0.15269937 | 0.15052198 | 0.88035398 | 0.999976173 | 0.022984611 |
| YMR075W | RCO1 | 0.15269937 | 0.15096796 | 0.88000217 | 0.999976173 | 0.023052712 |
| YPR093C | ASR1 | 0.15269937 | 0.15159563 | 0.87950706 | 0.999976173 | 0.023148557 |
| YKL148C | SDH1 | 0.15269937 | 0.15253119 | 0.87876918 | 0.999976173 | 0.023291416 |
| YER179W | DMC1 | 0.15269937 | 0.15275933 | 0.87858926 | 0.999976173 | 0.023326252 |
| YJL154C | VPS35 | 0.15269937 | 0.15334273 | 0.8781292 | 0.999976173 | 0.023415338 |
| YJL145W | SFH5 | 0.15269937 | 0.15402655 | 0.87759 | 0.999976173 | 0.023519757 |
| YHR093W | AHT1 | 0.15269937 | 0.15408337 | 0.8775452 | 0.999976173 | 0.023528432 |
| YPL115C | BEM3 | 0.15269937 | 0.15521754 | 0.87665103 | 0.999976173 | 0.02370162 |
| YMR141C | YMR141C | 0.15269937 | 0.15556785 | 0.87637488 | 0.999976173 | 0.023755112 |
| YJL016W | YJL016W | 0.15269937 | 0.15563992 | 0.87631807 | 0.999976173 | 0.023766117 |
| YOL030W | GAS5 | 0.15269937 | 0.156021 | 0.87601768 | 0.999976173 | 0.023824308 |
| YGR016W | YGR016W | 0.15269937 | 0.15613541 | 0.87592751 | 0.999976173 | 0.023841778 |
| YBR185C | MBA1 | 0.15269937 | 0.15635401 | 0.87575521 | 0.999976173 | 0.023875157 |
| YFR006W | YFR006W | 0.15269937 | 0.15647864 | 0.87565698 | 0.999976173 | 0.023894189 |

| | | | | | | |
|---|---|---|---|---|---|---|
| YDL169C | UGX2 | 0.15269937 | 0.15660404 | 0.87555814 | 0.999976173 | 0.023913337 |
| YDL234C | GYP7 | 0.15269937 | 0.15673453 | 0.87545529 | 0.999976173 | 0.023933264 |
| YDL027C | YDL027C | 0.15269937 | 0.15716754 | 0.87511403 | 0.999976173 | 0.023999384 |
| YMR110C | HFD1 | 0.15269937 | 0.15729115 | 0.87501662 | 0.999976173 | 0.024018259 |
| YDL062W | YDL062W | 0.15269937 | 0.15731496 | 0.87499786 | 0.999976173 | 0.024021894 |
| YKL116C | PRR1 | 0.15269937 | 0.15736486 | 0.87495853 | 0.999976173 | 0.024029515 |
| YBR158W | AMN1 | 0.15269937 | 0.1577103 | 0.87468631 | 0.999976173 | 0.024082263 |
| YEL008W | YEL008W | 0.15269937 | 0.15778677 | 0.87462605 | 0.999976173 | 0.02409394 |
| YLR017W | MEU1 | 0.15269937 | 0.15788921 | 0.87454533 | 0.999976173 | 0.024109583 |
| YDR058C | TGL2 | 0.15269937 | 0.15814754 | 0.87434177 | 0.999976173 | 0.024149029 |
| YOL036W | YOL036W | 0.15269937 | 0.15866041 | 0.87393767 | 0.999976173 | 0.024227344 |
| YKL114C | APN1 | 0.15269937 | 0.15958557 | 0.87320879 | 0.999976173 | 0.024368616 |
| YMR196W | YMR196W | 0.15269937 | 0.15963626 | 0.87316886 | 0.999976173 | 0.024376355 |
| YBR301W | PAU24 | 0.15269937 | 0.15988952 | 0.87296935 | 0.999976173 | 0.024415029 |
| YCR062W | NA | 0.15269937 | 0.16011702 | 0.87279015 | 0.999976173 | 0.024449767 |
| YDL154W | MSH5 | 0.15269937 | 0.16052615 | 0.87246788 | 0.999976173 | 0.024512241 |
| YCR088W | ABP1 | 0.15269937 | 0.16057051 | 0.87243294 | 0.999976173 | 0.024519015 |
| YHR048W | YHK8 | 0.15269937 | 0.16082541 | 0.87223217 | 0.999976173 | 0.024557938 |
| YDR071C | PAA1 | 0.15269937 | 0.16099361 | 0.87209969 | 0.999976173 | 0.024583622 |
| YDR321W | ASP1 | 0.15269937 | 0.16208857 | 0.87123737 | 0.999976173 | 0.024750822 |
| YGL010W | YGL010W | 0.15269937 | 0.16239176 | 0.87099863 | 0.999976173 | 0.024797118 |
| YPL240C | HSP82 | 0.15269937 | 0.16267838 | 0.87077294 | 0.999976173 | 0.024840885 |
| YDL078C | MDH3 | 0.15269937 | 0.16272458 | 0.87073656 | 0.999976173 | 0.024847941 |
| YMR256C | COX7 | 0.15269937 | 0.1627892 | 0.87068568 | 0.999976173 | 0.024857808 |
| YDR101C | ARX1 | 0.15269937 | 0.16337279 | 0.8702262 | 0.999976173 | 0.024946921 |
| YLR135W | SLX4 | 0.15269937 | 0.16345387 | 0.87016237 | 0.999976173 | 0.024959301 |
| YOR280C | FSH3 | 0.15269937 | 0.164426 | 0.86939708 | 0.999976173 | 0.025107746 |
| YKR046C | PET10 | 0.15269937 | 0.16445461 | 0.86937456 | 0.999976173 | 0.025112115 |
| YOL081W | IRA2 | 0.15269937 | 0.16503857 | 0.86891491 | 0.999976173 | 0.025201286 |
| YHR131C | YHR131C | 0.15269937 | 0.16509567 | 0.86886997 | 0.999976173 | 0.025210003 |
| YMR289W | ABZ2 | 0.15269937 | 0.16514816 | 0.86882866 | 0.999976173 | 0.025218019 |
| YOR136W | IDH2 | 0.15269937 | 0.16527314 | 0.86873029 | 0.999976173 | 0.025237103 |
| YDR411C | DFM1 | 0.15269937 | 0.16566378 | 0.86842284 | 0.999976173 | 0.025296754 |
| YGL096W | TOS8 | 0.15269937 | 0.16594494 | 0.86820158 | 0.999976173 | 0.025339687 |
| YDR218C | SPR28 | 0.15269937 | 0.16612469 | 0.86806012 | 0.999976173 | 0.025367134 |
| YCR017C | CWH43 | 0.15269937 | 0.16702498 | 0.8673517 | 0.999976173 | 0.025504609 |
| YLR211C | YLR211C | 0.15269937 | 0.16739573 | 0.86705999 | 0.999976173 | 0.025561222 |
| YLL032C | YLL032C | 0.15269937 | 0.16771994 | 0.86680492 | 0.999976173 | 0.025610728 |
| YJR128W | YJR128W | 0.15269937 | 0.16779219 | 0.86674808 | 0.999976173 | 0.025621761 |
| YBR232C | YBR232C | 0.15269937 | 0.1679327 | 0.86663753 | 0.999976173 | 0.025643217 |
| YJR051W | OSM1 | 0.15269937 | 0.16833755 | 0.86631905 | 0.999976173 | 0.025705037 |
| YAL019W | FUN30 | 0.15269937 | 0.16834283 | 0.86631489 | 0.999976173 | 0.025705844 |
| YPR154W | PIN3 | 0.15269937 | 0.16837358 | 0.86629071 | 0.999976173 | 0.025710539 |
| YOR120W | GCY1 | 0.15269937 | 0.16856823 | 0.86613759 | 0.999976173 | 0.025740262 |
| YMR162C | DNF3 | 0.15269937 | 0.16890792 | 0.86587039 | 0.999976173 | 0.025792133 |
| YIL070C | MAM33 | 0.15269937 | 0.16904457 | 0.8657629 | 0.999976173 | 0.025812999 |
| YOL050C | YOL050C | 0.15269937 | 0.16906703 | 0.86574524 | 0.999976173 | 0.025816428 |
| YDR220C | YDR220C | 0.15269937 | 0.16967008 | 0.86527093 | 0.999976173 | 0.025908513 |
| YDL019C | OSH2 | 0.15269937 | 0.16973222 | 0.86522206 | 0.999976173 | 0.025918002 |
| YDR503C | LPP1 | 0.15269937 | 0.16975528 | 0.86520393 | 0.999976173 | 0.025921523 |
| YGL229C | SAP4 | 0.15269937 | 0.16990806 | 0.86508377 | 0.999976173 | 0.025944853 |
| YOL037C | YOL037C | 0.15269937 | 0.17050676 | 0.86461295 | 0.999976173 | 0.026036275 |
| YLL006W | MMM1 | 0.15269937 | 0.17076606 | 0.86440905 | 0.999976173 | 0.026075869 |
| YHL019C | APM2 | 0.15269937 | 0.1711638 | 0.86409631 | 0.999976173 | 0.026136603 |
| YOR334W | MRS2 | 0.15269937 | 0.1712508 | 0.86402791 | 0.999976173 | 0.026149888 |
| YKR017C | YKR017C | 0.15269937 | 0.17268546 | 0.86290003 | 0.999976173 | 0.026368959 |
| YJL183W | MNN11 | 0.15269937 | 0.17337193 | 0.86236044 | 0.999976173 | 0.026473783 |
| YDR202C | RAV2 | 0.15269937 | 0.17359852 | 0.86218235 | 0.999976173 | 0.026508384 |
| YBR034C | HMT1 | 0.15269937 | 0.17408832 | 0.86179741 | 0.999976173 | 0.026583177 |
| YBR292C | YBR292C | 0.15269937 | 0.17417764 | 0.86172722 | 0.999976173 | 0.026596814 |
| YDL160C | DHH1 | 0.15269937 | 0.17422045 | 0.86169358 | 0.999976173 | 0.026603352 |
| YOR153W | PDR5 | 0.15269937 | 0.17490704 | 0.86115405 | 0.999976173 | 0.026708194 |
| YHR180W | YHR180W | 0.15269937 | 0.17536341 | 0.86079546 | 0.999976173 | 0.026777881 |
| YLL012W | YEH1 | 0.15269937 | 0.175982 | 0.86030946 | 0.999976173 | 0.026872339 |

| | | | | | | |
|---|---|---|---|---|---|---|
| YGL086W | MAD1 | 0.15269937 | 0.17603561 | 0.86026734 | 0.999976173 | 0.026880526 |
| YOR092W | ECM3 | 0.15269937 | 0.17626399 | 0.86008793 | 0.999976173 | 0.026915399 |
| YMR306C-A | YMR306C-A | 0.15269937 | 0.17637914 | 0.85999747 | 0.999976173 | 0.026932983 |
| YEL025C | YEL025C | 0.15269937 | 0.17658793 | 0.85983345 | 0.999976173 | 0.026964865 |
| YHL024W | RIM4 | 0.15269937 | 0.17701276 | 0.85949975 | 0.999976173 | 0.027029736 |
| YGL251C | HFM1 | 0.15269937 | 0.17749354 | 0.85912213 | 0.999976173 | 0.027103151 |
| YML005W | TRM12 | 0.15269937 | 0.17788112 | 0.85881773 | 0.999976173 | 0.027162334 |
| YDR193W | YDR193W | 0.15269937 | 0.17873619 | 0.85814625 | 0.999976173 | 0.027292903 |
| YIL092W | YIL092W | 0.15269937 | 0.17907388 | 0.85788109 | 0.999976173 | 0.027344468 |
| YOR242C | SSP2 | 0.15269937 | 0.17920444 | 0.85777858 | 0.999976173 | 0.027364404 |
| YDL175C | AIR2 | 0.15269937 | 0.17938957 | 0.85763323 | 0.999976173 | 0.027392673 |
| YJL093C | TOK1 | 0.15269937 | 0.17948035 | 0.85756195 | 0.999976173 | 0.027406535 |
| YPR098C | YPR098C | 0.15269937 | 0.17995804 | 0.85718692 | 0.999976173 | 0.027479479 |
| YGL067W | NPY1 | 0.15269937 | 0.18008111 | 0.8570903 | 0.999976173 | 0.027498271 |
| YMR194W | RPL36A | 0.15269937 | 0.18027993 | 0.85693422 | 0.999976173 | 0.027528631 |
| YCR008W | SAT4 | 0.15269937 | 0.18035133 | 0.85687818 | 0.999976173 | 0.027539533 |
| YOR010C | TIR2 | 0.15269937 | 0.18119897 | 0.85621282 | 0.999976173 | 0.027668968 |
| YFL031W | HAC1 | 0.15269937 | 0.18183782 | 0.85571143 | 0.999976173 | 0.02776652 |
| YGR225W | AMA1 | 0.15269937 | 0.18208293 | 0.85551907 | 0.999976173 | 0.027803949 |
| YFR009W | GCN20 | 0.15269937 | 0.18254483 | 0.8551566 | 0.999976173 | 0.02787448 |
| YIR020W-B | NA | 0.15269937 | 0.18261157 | 0.85510423 | 0.999976173 | 0.027884671 |
| YLL054C | YLL054C | 0.15269937 | 0.18410621 | 0.85393158 | 0.999976173 | 0.028112901 |
| YGR217W | CCH1 | 0.15269937 | 0.18426272 | 0.8538088 | 0.999976173 | 0.028136801 |
| YDR144C | MKC7 | 0.15269937 | 0.18461272 | 0.85353426 | 0.999976173 | 0.028190245 |
| YHR167W | THP2 | 0.15269937 | 0.18472222 | 0.85344837 | 0.999976173 | 0.028206965 |
| YHR077C | 30 | 0.15269937 | 0.18481126 | 0.85337852 | 0.999976173 | 0.028220562 |
| YPL259C | APM1 | 0.15269937 | 0.18515947 | 0.85310541 | 0.999976173 | 0.028273734 |
| YBR182C | SMP1 | 0.15269937 | 0.18530915 | 0.85298801 | 0.999976173 | 0.02829659 |
| YDR512C | EMI1 | 0.15269937 | 0.18539536 | 0.8529204 | 0.999976173 | 0.028309755 |
| YLR252W | YLR252W | 0.15269937 | 0.18578354 | 0.85261597 | 0.999976173 | 0.028369029 |
| YOL107W | YOL107W | 0.15269937 | 0.18607369 | 0.85238843 | 0.999976173 | 0.028413335 |
| YDL191W | RPL35A | 0.15269937 | 0.18652094 | 0.85203772 | 0.999976173 | 0.028481629 |
| YGR121C | MEP1 | 0.15269937 | 0.18676691 | 0.85184485 | 0.999976173 | 0.028519189 |
| YHR139C-A | YHR139C-A | 0.15269937 | 0.1874894 | 0.8512784 | 0.999976173 | 0.028629512 |
| YCR106W | RDS1 | 0.15269937 | 0.18755554 | 0.85122655 | 0.999976173 | 0.028639612 |
| YMR219W | ESC1 | 0.15269937 | 0.18762236 | 0.85117417 | 0.999976173 | 0.028649815 |
| YLR149C | YLR149C | 0.15269937 | 0.18959018 | 0.84963177 | 0.999976173 | 0.0289503 |
| YLL019C | KNS1 | 0.15269937 | 0.19019651 | 0.84915664 | 0.999976173 | 0.029042886 |
| YDL185W | TFP1 | 0.15269937 | 0.19025426 | 0.84911139 | 0.999976173 | 0.029051705 |
| YDL135C | RDI1 | 0.15269937 | 0.19078189 | 0.84869797 | 0.999976173 | 0.029132274 |
| YLR306W | UBC12 | 0.15269937 | 0.19145486 | 0.84817074 | 0.999976173 | 0.029235036 |
| YLR041W | YLR041W | 0.15269937 | 0.1923339 | 0.84748217 | 0.999976173 | 0.029369264 |
| YOL019W | YOL019W | 0.15269937 | 0.19247329 | 0.84737299 | 0.999976173 | 0.02939055 |
| YNL303W | YNL303W | 0.15269937 | 0.19255114 | 0.84731202 | 0.999976173 | 0.029402437 |
| YPL154C | PEP4 | 0.15269937 | 0.19348831 | 0.84657808 | 0.999976173 | 0.029545542 |
| YOL067C | RTG1 | 0.15269937 | 0.19359378 | 0.84649549 | 0.999976173 | 0.029561647 |
| YKR103W | NFT1 | 0.15269937 | 0.19359554 | 0.84649411 | 0.999976173 | 0.029561917 |
| YOR246C | YOR246C | 0.15269937 | 0.19366791 | 0.84643744 | 0.999976173 | 0.029572967 |
| YHR137W | ARO9 | 0.15269937 | 0.19390275 | 0.84625355 | 0.999976173 | 0.029608827 |
| YGR166W | KRE11 | 0.15269937 | 0.19401986 | 0.84616185 | 0.999976173 | 0.02962671 |
| YDL214C | PRR2 | 0.15269937 | 0.1942429 | 0.84598722 | 0.999976173 | 0.029660768 |
| YPR150W | YPR150W | 0.15269937 | 0.19462414 | 0.84568873 | 0.999976173 | 0.029718983 |
| YNL228W | YNL228W | 0.15269937 | 0.19462846 | 0.84568534 | 0.999976173 | 0.029719643 |
| YNL265C | IST1 | 0.15269937 | 0.19512314 | 0.84529808 | 0.999976173 | 0.029795179 |
| YOR337W | TEA1 | 0.15269937 | 0.19536825 | 0.8451062 | 0.999976173 | 0.029832608 |
| YGL156W | AMS1 | 0.15269937 | 0.19544391 | 0.84504697 | 0.999976173 | 0.029844162 |
| YBL037W | APL3 | 0.15269937 | 0.1957 | 0.84484652 | 0.999976173 | 0.029883266 |
| YDL142C | CRD1 | 0.15269937 | 0.19579987 | 0.84476835 | 0.999976173 | 0.029898515 |
| YLR364W | GRX8 | 0.15269937 | 0.19694079 | 0.84387542 | 0.999976173 | 0.030072733 |
| YJL177W | RPL17B | 0.15269937 | 0.19763769 | 0.8433301 | 0.999976173 | 0.03017915 |
| YML029W | USA1 | 0.15269937 | 0.19772064 | 0.84326519 | 0.999976173 | 0.030191816 |
| YKL067W | YNK1 | 0.15269937 | 0.19804606 | 0.84301058 | 0.999976173 | 0.030241508 |
| YER118C | SHO1 | 0.15269937 | 0.19889891 | 0.84234339 | 0.999976173 | 0.030371737 |
| YGL083W | SCY1 | 0.15269937 | 0.19914351 | 0.84215205 | 0.999976173 | 0.030409088 |

| | | | | | | |
|---|---|---|---|---|---|---|
| YML041C | VPS71 | 0.15269937 | 0.19923481 | 0.84208064 | 0.999976173 | 0.030423029 |
| YJR082C | EAF6 | 0.15269937 | 0.1999025 | 0.8415584 | 0.999976173 | 0.030524985 |
| YOR229W | WTM2 | 0.15269937 | 0.20001111 | 0.84147346 | 0.999976173 | 0.03054157 |
| YML071C | COG8 | 0.15269937 | 0.20009642 | 0.84140675 | 0.999976173 | 0.030554596 |
| YNR004W | YNR004W | 0.15269937 | 0.20151425 | 0.84029807 | 0.999976173 | 0.030771098 |
| YJL017W | NA | 0.15269937 | 0.20151873 | 0.84029457 | 0.999976173 | 0.030771782 |
| YDR344C | YDR344C | 0.15269937 | 0.20157392 | 0.84025142 | 0.999976173 | 0.03078021 |
| YPL194W | 3 | 0.15269937 | 0.2021299 | 0.83981676 | 0.999976173 | 0.030865107 |
| YGR228W | YGR228W | 0.15269937 | 0.20253336 | 0.83950138 | 0.999976173 | 0.030926715 |
| YJL128C | PBS2 | 0.15269937 | 0.20256648 | 0.83947548 | 0.999976173 | 0.030931773 |
| YDR034C | LYS14 | 0.15269937 | 0.20315856 | 0.8390127 | 0.999976173 | 0.031022183 |
| YDR494W | RSM28 | 0.15269937 | 0.20319251 | 0.83898616 | 0.999976173 | 0.031027367 |
| YDL133W | YDL133W | 0.15269937 | 0.20321355 | 0.83896972 | 0.999976173 | 0.031030581 |
| YBR040W | FIG1 | 0.15269937 | 0.20364872 | 0.83862962 | 0.999976173 | 0.03109703 |
| YNL314W | DAL82 | 0.15269937 | 0.20405693 | 0.83831061 | 0.999976173 | 0.031159364 |
| YGR170W | PSD2 | 0.15269937 | 0.20426689 | 0.83814655 | 0.999976173 | 0.031191424 |
| YOR315W | SFG1 | 0.15269937 | 0.20445213 | 0.8380018 | 0.999976173 | 0.03121971 |
| YLR250W | SSP120 | 0.15269937 | 0.20557519 | 0.83712437 | 0.999976173 | 0.031391201 |
| YMR278W | PGM3 | 0.15269937 | 0.20606429 | 0.83674231 | 0.999976173 | 0.031465887 |
| YJL217W | REE1 | 0.15269937 | 0.20708989 | 0.83594128 | 0.999976173 | 0.031622495 |
| YDR031W | MIC14 | 0.15269937 | 0.20710227 | 0.83593162 | 0.999976173 | 0.031624385 |
| YLR179C | YLR179C | 0.15269937 | 0.20713637 | 0.83590499 | 0.999976173 | 0.031629593 |
| YKL041W | VPS24 | 0.15269937 | 0.20798519 | 0.83524217 | 0.999976173 | 0.031759207 |
| YDR492W | IZH1 | 0.15269937 | 0.20914856 | 0.83433391 | 0.999976173 | 0.031936852 |
| YOR121C | YOR121C | 0.15269937 | 0.20955909 | 0.83401346 | 0.999976173 | 0.031999539 |
| YGR008C | STF2 | 0.15269937 | 0.20985953 | 0.83377896 | 0.999976173 | 0.032045417 |
| YMR318C | ADH6 | 0.15269937 | 0.2099509 | 0.83370764 | 0.999976173 | 0.03205937 |
| YCR099C | YCR099C | 0.15269937 | 0.20995409 | 0.83370515 | 0.999976173 | 0.032059857 |
| YLR370C | ARC18 | 0.15269937 | 0.21026581 | 0.83346187 | 0.999976173 | 0.032107457 |
| YER035W | EDC2 | 0.15269937 | 0.21045694 | 0.83331272 | 0.999976173 | 0.032136641 |
| YLR134W | PDC5 | 0.15269937 | 0.21061774 | 0.83318723 | 0.999976173 | 0.032161195 |
| YDL128W | VCX1 | 0.15269937 | 0.21101826 | 0.83287468 | 0.999976173 | 0.032222354 |
| YLR297W | YLR297W | 0.15269937 | 0.21140668 | 0.8325716 | 0.999976173 | 0.032281667 |
| YEL030W | ECM10 | 0.15269937 | 0.21174253 | 0.83230957 | 0.999976173 | 0.032332951 |
| YGL132W | YGL132W | 0.15269937 | 0.21197154 | 0.83213091 | 0.999976173 | 0.032367919 |
| YMR026C | PEX12 | 0.15269937 | 0.2124102 | 0.8317887 | 0.999976173 | 0.032434902 |
| YER180C | ISC10 | 0.15269937 | 0.21251954 | 0.8317034 | 0.999976173 | 0.032451599 |
| YKL205W | LOS1 | 0.15269937 | 0.21315276 | 0.83120949 | 0.999976173 | 0.032548291 |
| YDR533C | HSP31 | 0.15269937 | 0.21327446 | 0.83111457 | 0.999976173 | 0.032566874 |
| YDR330W | UBX5 | 0.15269937 | 0.21350095 | 0.83093793 | 0.999976173 | 0.032601459 |
| YLR408C | YLR408C | 0.15269937 | 0.21422803 | 0.83037093 | 0.999976173 | 0.032712484 |
| YAL011W | SWC3 | 0.15269937 | 0.21452316 | 0.83014079 | 0.999976173 | 0.032757551 |
| YER108C | NA | 0.15269937 | 0.21478627 | 0.82993565 | 0.999976173 | 0.032797728 |
| YOR094W | ARF3 | 0.15269937 | 0.21524973 | 0.82957432 | 0.999976173 | 0.032868497 |
| YNL327W | EGT2 | 0.15269937 | 0.21561906 | 0.8292864 | 0.999976173 | 0.032924894 |
| YDR171W | HSP42 | 0.15269937 | 0.21613971 | 0.82888055 | 0.999976173 | 0.033004396 |
| YNR002C | ATO2 | 0.15269937 | 0.21660829 | 0.82851533 | 0.999976173 | 0.033075948 |
| YKR077W | MSA2 | 0.15269937 | 0.21673723 | 0.82841484 | 0.999976173 | 0.033095638 |
| YER053C | PIC2 | 0.15269937 | 0.21741589 | 0.82788597 | 0.999976173 | 0.033199268 |
| YML018C | YML018C | 0.15269937 | 0.21793193 | 0.82748387 | 0.999976173 | 0.033278067 |
| YNL135C | FPR1 | 0.15269937 | 0.21813313 | 0.8273271 | 0.999976173 | 0.033308791 |
| YKR099W | BAS1 | 0.15269937 | 0.21876597 | 0.82683408 | 0.999976173 | 0.033405425 |
| YOL082W | ATG19 | 0.15269937 | 0.21988923 | 0.82595916 | 0.999976173 | 0.033576946 |
| YER166W | DNF1 | 0.15269937 | 0.21996552 | 0.82589974 | 0.999976173 | 0.033588596 |
| YLL023C | YLL023C | 0.15269937 | 0.22020207 | 0.82571552 | 0.999976173 | 0.033624717 |
| YOR191W | ULS1 | 0.15269937 | 0.22108798 | 0.82502567 | 0.999976173 | 0.033759995 |
| YMR226C | YMR226C | 0.15269937 | 0.22124916 | 0.82490018 | 0.999976173 | 0.033784607 |
| YNL157W | YNL157W | 0.15269937 | 0.22130193 | 0.82485909 | 0.999976173 | 0.033792664 |
| YLR020C | YEH2 | 0.15269937 | 0.22187319 | 0.82441435 | 0.999976173 | 0.033879896 |
| YER097W | YER097W | 0.15269937 | 0.22194071 | 0.82436179 | 0.999976173 | 0.033890205 |
| YLR326W | YLR326W | 0.15269937 | 0.22221657 | 0.82414705 | 0.999976173 | 0.033932329 |
| YJL101C | GSH1 | 0.15269937 | 0.22276212 | 0.82372241 | 0.999976173 | 0.034015634 |
| YDL138W | RGT2 | 0.15269937 | 0.22303302 | 0.82351157 | 0.999976173 | 0.034057001 |
| YPL186C | UIP4 | 0.15269937 | 0.22304424 | 0.82350283 | 0.999976173 | 0.034058714 |

| | | | | | | |
|---|---|---|---|---|---|---|
| YLR165C | PUS5 | 0.15269937 | 0.22353611 | 0.82312005 | 0.999976173 | 0.034133822 |
| YDR372C | VPS74 | 0.15269937 | 0.22411085 | 0.82267282 | 0.999976173 | 0.034221585 |
| YPR189W | SKI3 | 0.15269937 | 0.22496103 | 0.82201137 | 0.999976173 | 0.034351407 |
| YCR005C | CIT2 | 0.15269937 | 0.22525251 | 0.82178462 | 0.999976173 | 0.034395915 |
| YJR125C | ENT3 | 0.15269937 | 0.22527368 | 0.82176815 | 0.999976173 | 0.034399148 |
| YDR227W | SIR4 | 0.15269937 | 0.22549679 | 0.8215946 | 0.999976173 | 0.034433216 |
| YJL049W | YJL049W | 0.15269937 | 0.22647891 | 0.82083075 | 0.999976173 | 0.034583186 |
| YKL159C | RCN1 | 0.15269937 | 0.22669064 | 0.82066609 | 0.999976173 | 0.034615517 |
| YML117W | NAB6 | 0.15269937 | 0.22731306 | 0.82018211 | 0.999976173 | 0.03471056 |
| YDR253C | MET32 | 0.15269937 | 0.22737705 | 0.82013236 | 0.999976173 | 0.034720331 |
| YHR203C | RPS4B | 0.15269937 | 0.22774181 | 0.81984877 | 0.999976173 | 0.034776029 |
| YJR129C | YJR129C | 0.15269937 | 0.22775927 | 0.81983519 | 0.999976173 | 0.034778696 |
| YOL017W | ESC8 | 0.15269937 | 0.2279188 | 0.81971117 | 0.999976173 | 0.034803056 |
| YDR250C | YDR250C | 0.15269937 | 0.22886168 | 0.81897823 | 0.999976173 | 0.034947033 |
| YHR076W | PTC7 | 0.15269937 | 0.22927407 | 0.81865772 | 0.999976173 | 0.035010005 |
| YEL071W | DLD3 | 0.15269937 | 0.22998505 | 0.81810521 | 0.999976173 | 0.035118571 |
| YOR304C-A | YOR304C-A | 0.15269937 | 0.23006814 | 0.81804064 | 0.999976173 | 0.03513126 |
| YDL051W | LHP1 | 0.15269937 | 0.23103369 | 0.81729046 | 0.999976173 | 0.035278698 |
| YOR067C | ALG8 | 0.15269937 | 0.23160218 | 0.81684884 | 0.999976173 | 0.035365506 |
| YDR089W | YDR089W | 0.15269937 | 0.23249826 | 0.81615288 | 0.999976173 | 0.035502336 |
| YKR069W | MET1 | 0.15269937 | 0.23304714 | 0.81572664 | 0.999976173 | 0.035586151 |
| YPR015C | YPR015C | 0.15269937 | 0.23358064 | 0.8153124 | 0.999976173 | 0.035667616 |
| YDR332W | IRC3 | 0.15269937 | 0.23368165 | 0.81523398 | 0.999976173 | 0.035683039 |
| YPL069C | BTS1 | 0.15269937 | 0.23500923 | 0.81420343 | 0.999976173 | 0.03588576 |
| YER020W | GPA2 | 0.15269937 | 0.2357972 | 0.8135919 | 0.999976173 | 0.036006084 |
| YPL109C | YPL109C | 0.15269937 | 0.23628235 | 0.81321545 | 0.999976173 | 0.036080164 |
| YDR480W | DIG2 | 0.15269937 | 0.23633604 | 0.81317379 | 0.999976173 | 0.036088363 |
| YMR177W | MMT1 | 0.15269937 | 0.23721833 | 0.81248929 | 0.999976173 | 0.036223089 |
| YDR033W | MRH1 | 0.15269937 | 0.23723104 | 0.81247943 | 0.999976173 | 0.036225029 |
| YLR438W | CAR2 | 0.15269937 | 0.23802757 | 0.8118616 | 0.999976173 | 0.036346658 |
| YLR268W | SEC22 | 0.15269937 | 0.23810156 | 0.81180421 | 0.999976173 | 0.036357958 |
| YGL164C | YRB30 | 0.15269937 | 0.2381151 | 0.81179371 | 0.999976173 | 0.036360024 |
| YDL130W | RPP1B | 0.15269937 | 0.23884276 | 0.8112294 | 0.999976173 | 0.036471138 |
| YHL006C | SHU1 | 0.15269937 | 0.23941334 | 0.81078698 | 0.999976173 | 0.036558266 |
| YDR163W | CWC15 | 0.15269937 | 0.23942208 | 0.8107802 | 0.999976173 | 0.0365596 |
| YEL052W | AFG1 | 0.15269937 | 0.23968939 | 0.81057296 | 0.999976173 | 0.036600418 |
| YLR281C | YLR281C | 0.15269937 | 0.24017676 | 0.81019513 | 0.999976173 | 0.036674839 |
| YDR436W | PPZ2 | 0.15269937 | 0.24034829 | 0.81006217 | 0.999976173 | 0.036701031 |
| YPL110C | GDE1 | 0.15269937 | 0.24183182 | 0.8089124 | 0.999976173 | 0.036927565 |
| YGL181W | GTS1 | 0.15269937 | 0.24264952 | 0.80827884 | 0.999976173 | 0.037052428 |
| YIL148W | RPL40A | 0.15269937 | 0.24277583 | 0.80818098 | 0.999976173 | 0.037071715 |
| YPR123C | YPR123C | 0.15269937 | 0.24285682 | 0.80811824 | 0.999976173 | 0.037084083 |
| YDR217C | 17 | 0.15269937 | 0.24286304 | 0.80811343 | 0.999976173 | 0.037085031 |
| YJL178C | ATG27 | 0.15269937 | 0.24288931 | 0.80809307 | 0.999976173 | 0.037089044 |
| YIL099W | SGA1 | 0.15269937 | 0.24289267 | 0.80809047 | 0.999976173 | 0.037089556 |
| YHR133C | NSG1 | 0.15269937 | 0.24322396 | 0.80783383 | 0.999976173 | 0.037140145 |
| YMR299C | DYN3 | 0.15269937 | 0.24355644 | 0.8075763 | 0.999976173 | 0.037190914 |
| YER087C-A | YER087C-A | 0.15269937 | 0.24359967 | 0.80754281 | 0.999976173 | 0.037197515 |
| YGL166W | CUP2 | 0.15269937 | 0.24372465 | 0.80744601 | 0.999976173 | 0.0372166 |
| YPL114W | YPL114W | 0.15269937 | 0.24377948 | 0.80740355 | 0.999976173 | 0.037224972 |
| YHR200W | RPN10 | 0.15269937 | 0.24400727 | 0.80722712 | 0.999976173 | 0.037259755 |
| YJL012C | VTC4 | 0.15269937 | 0.24413781 | 0.80712603 | 0.999976173 | 0.037279688 |
| YCL044C | MGR1 | 0.15269937 | 0.24435292 | 0.80695944 | 0.999976173 | 0.037312536 |
| YAL014C | SYN8 | 0.15269937 | 0.24559744 | 0.80599582 | 0.999976173 | 0.037502573 |
| YER106W | MAM1 | 0.15269937 | 0.2464113 | 0.80536581 | 0.999976173 | 0.037626849 |
| YER187W | YER187W | 0.15269937 | 0.24658573 | 0.8052308 | 0.999976173 | 0.037653485 |
| YJL022W | YJL022W | 0.15269937 | 0.24740887 | 0.80459377 | 0.999976173 | 0.037779178 |
| YKR056W | TRM2 | 0.15269937 | 0.24866988 | 0.80361812 | 0.999976173 | 0.037971732 |
| YLR441C | RPS1A | 0.15269937 | 0.24896272 | 0.8033916 | 0.999976173 | 0.038016449 |
| YDL088C | ASM4 | 0.15269937 | 0.24935011 | 0.80309195 | 0.999976173 | 0.038075603 |
| YLR036C | YLR036C | 0.15269937 | 0.24977439 | 0.80276381 | 0.999976173 | 0.038140391 |
| YOR012W | YOR012W | 0.15269937 | 0.25000794 | 0.80258319 | 0.999976173 | 0.038176054 |
| YLR137W | YLR137W | 0.15269937 | 0.2501657 | 0.80246119 | 0.999976173 | 0.038200144 |
| YKR039W | GAP1 | 0.15269937 | 0.25035438 | 0.80231529 | 0.999976173 | 0.038228955 |

| | | | | | | |
|---|---|---|---|---|---|---|
| YHL047C | ARN2 | 0.15269937 | 0.25086659 | 0.80191924 | 0.999976173 | 0.038307169 |
| YLR043C | TRX1 | 0.15269937 | 0.25093507 | 0.8018663 | 0.999976173 | 0.038317626 |
| YGL208W | SIP2 | 0.15269937 | 0.25112491 | 0.80171953 | 0.999976173 | 0.038346614 |
| YMR023C | MSS1 | 0.15269937 | 0.25152183 | 0.80141268 | 0.999976173 | 0.038407224 |
| YKL030W | YKL030W | 0.15269937 | 0.2517025 | 0.80127302 | 0.999976173 | 0.038434812 |
| YJL142C | IRC9 | 0.15269937 | 0.25178982 | 0.80120553 | 0.999976173 | 0.038448145 |
| YHL005C | YHL005C | 0.15269937 | 0.25180266 | 0.8011956 | 0.999976173 | 0.038450106 |
| YPR008W | HAA1 | 0.15269937 | 0.25329495 | 0.80004231 | 0.999976173 | 0.038677979 |
| YDR403W | DIT1 | 0.15269937 | 0.25362129 | 0.79979016 | 0.999976173 | 0.03872781 |
| YCR067C | SED4 | 0.15269937 | 0.25439175 | 0.79919495 | 0.999976173 | 0.038845458 |
| YOL011W | PLB3 | 0.15269937 | 0.25499342 | 0.79873021 | 0.999976173 | 0.038937333 |
| YLR328W | NMA1 | 0.15269937 | 0.2554528 | 0.79837542 | 0.999976173 | 0.039007481 |
| YPR024W | YME1 | 0.15269937 | 0.25627831 | 0.79773798 | 0.999976173 | 0.039133535 |
| YCR077C | PAT1 | 0.15269937 | 0.25648349 | 0.79757956 | 0.999976173 | 0.039164867 |
| YOR052C | YOR052C | 0.15269937 | 0.25659419 | 0.7974941 | 0.999976173 | 0.03918177 |
| YCL049C | YCL049C | 0.15269937 | 0.25672256 | 0.797395 | 0.999976173 | 0.039201372 |
| YNL086W | YNL086W | 0.15269937 | 0.25677767 | 0.79735245 | 0.999976173 | 0.039209787 |
| YHR086W | NAM8 | 0.15269937 | 0.25710532 | 0.79709952 | 0.999976173 | 0.039259819 |
| YOR355W | GDS1 | 0.15269937 | 0.25729418 | 0.79695373 | 0.999976173 | 0.039288659 |
| YPL149W | ATG5 | 0.15269937 | 0.25779543 | 0.79656684 | 0.999976173 | 0.039365199 |
| YDL040C | NAT1 | 0.15269937 | 0.25978271 | 0.79503346 | 0.999976173 | 0.039668655 |
| YGL158W | RCK1 | 0.15269937 | 0.2598314 | 0.79499589 | 0.999976173 | 0.039676091 |
| YDR099W | BMH2 | 0.15269937 | 0.25987008 | 0.79496606 | 0.999976173 | 0.039681996 |
| YER088C | DOT6 | 0.15269937 | 0.26000002 | 0.79486582 | 0.999976173 | 0.039701839 |
| YDR063W | AIM7 | 0.15269937 | 0.26028906 | 0.79464288 | 0.999976173 | 0.039745974 |
| YFR033C | QCR6 | 0.15269937 | 0.26102206 | 0.79407757 | 0.999976173 | 0.039857903 |
| YDR203W | YDR203W | 0.15269937 | 0.26126709 | 0.79388862 | 0.999976173 | 0.039895319 |
| YPL105C | SYH1 | 0.15269937 | 0.26165142 | 0.79359228 | 0.999976173 | 0.039954005 |
| YKL120W | OAC1 | 0.15269937 | 0.26186723 | 0.79342589 | 0.999976173 | 0.039986959 |
| YPR002W | PDH1 | 0.15269937 | 0.2624104 | 0.79300714 | 0.999976173 | 0.040069902 |
| YDR446W | ECM11 | 0.15269937 | 0.26253572 | 0.79291054 | 0.999976173 | 0.040089038 |
| YPL125W | KAP120 | 0.15269937 | 0.26260801 | 0.79285481 | 0.999976173 | 0.040100076 |
| YDL093W | PMT5 | 0.15269937 | 0.26343176 | 0.79221991 | 0.999976173 | 0.040225862 |
| YDR061W | YDR061W | 0.15269937 | 0.26344104 | 0.79221276 | 0.999976173 | 0.04022728 |
| YDR107C | TMN2 | 0.15269937 | 0.26380109 | 0.7919353 | 0.999976173 | 0.040282259 |
| YGL167C | PMR1 | 0.15269937 | 0.26431028 | 0.79154294 | 0.999976173 | 0.040360012 |
| YKL130C | SHE2 | 0.15269937 | 0.26518207 | 0.79087132 | 0.999976173 | 0.040493134 |
| YBL039C | URA7 | 0.15269937 | 0.26519261 | 0.7908632 | 0.999976173 | 0.040494743 |
| YPR172W | YPR172W | 0.15269937 | 0.26536938 | 0.79072703 | 0.999976173 | 0.040521736 |
| YKR005C | YKR005C | 0.15269937 | 0.26539081 | 0.79071052 | 0.999976173 | 0.040525009 |
| YLR233C | 5 | 0.15269937 | 0.26639342 | 0.78993835 | 0.999976173 | 0.040678107 |
| YDR216W | ADR1 | 0.15269937 | 0.26652963 | 0.78983347 | 0.999976173 | 0.040698905 |
| YKR070W | YKR070W | 0.15269937 | 0.26677633 | 0.78964351 | 0.999976173 | 0.040736576 |
| YGR146C | YGR146C | 0.15269937 | 0.2668321 | 0.78960056 | 0.999976173 | 0.040745092 |
| YIR035C | YIR035C | 0.15269937 | 0.26733141 | 0.78921614 | 0.999976173 | 0.040821336 |
| YJL186W | MNN5 | 0.15269937 | 0.26742431 | 0.78914461 | 0.999976173 | 0.040835523 |
| YDR097C | MSH6 | 0.15269937 | 0.2675554 | 0.78904369 | 0.999976173 | 0.04085554 |
| YPL244C | HUT1 | 0.15269937 | 0.26785386 | 0.78881394 | 0.999976173 | 0.040901115 |
| YIL120W | QDR1 | 0.15269937 | 0.26792275 | 0.78876091 | 0.999976173 | 0.040911634 |
| YLR030W | YLR030W | 0.15269937 | 0.26813076 | 0.7886008 | 0.999976173 | 0.040943397 |
| YGR255C | COQ6 | 0.15269937 | 0.26838937 | 0.78840176 | 0.999976173 | 0.040982886 |
| YLL055W | YCT1 | 0.15269937 | 0.26841635 | 0.78838099 | 0.999976173 | 0.040987006 |
| YEL015W | EDC3 | 0.15269937 | 0.26854246 | 0.78828394 | 0.999976173 | 0.041006263 |
| YJL191W | RPS14B | 0.15269937 | 0.26854452 | 0.78828235 | 0.999976173 | 0.041006578 |
| YBR032W | YBR032W | 0.15269937 | 0.26934212 | 0.78766857 | 0.999976173 | 0.04112837 |
| YGL082W | YGL082W | 0.15269937 | 0.26944593 | 0.78758869 | 0.999976173 | 0.041144223 |
| YCL039W | GID7 | 0.15269937 | 0.27054624 | 0.78674221 | 0.999976173 | 0.041312238 |
| YLR128W | DCN1 | 0.15269937 | 0.27072075 | 0.78660798 | 0.999976173 | 0.041338886 |
| YHR104W | GRE3 | 0.15269937 | 0.27161704 | 0.78591866 | 0.999976173 | 0.04147575 |
| YHR127W | YHR127W | 0.15269937 | 0.27191686 | 0.78568812 | 0.999976173 | 0.041521531 |
| YNL211C | YNL211C | 0.15269937 | 0.27229391 | 0.78539821 | 0.999976173 | 0.041579108 |
| YOR134W | BAG7 | 0.15269937 | 0.27278685 | 0.78501924 | 0.999976173 | 0.041654379 |
| YBR012C | YBR012C | 0.15269937 | 0.27303291 | 0.78483009 | 0.999976173 | 0.041691953 |
| YDR242W | AMD2 | 0.15269937 | 0.27330302 | 0.78462247 | 0.999976173 | 0.041733198 |

| | | | | | | |
|---|---|---|---|---|---|---|
| YIL058W | YIL058W | 0.15269937 | 0.27348937 | 0.78447924 | 0.999976173 | 0.041761654 |
| YOR283W | YOR283W | 0.15269937 | 0.27367287 | 0.78433821 | 0.999976173 | 0.041789673 |
| YBR043C | QDR3 | 0.15269937 | 0.2739588 | 0.78411846 | 0.999976173 | 0.041833336 |
| YOR383C | FIT3 | 0.15269937 | 0.27405467 | 0.78404479 | 0.999976173 | 0.041847974 |
| YHR207C | SET5 | 0.15269937 | 0.27423811 | 0.78390383 | 0.999976173 | 0.041875986 |
| YER063W | THO1 | 0.15269937 | 0.27465858 | 0.78358074 | 0.999976173 | 0.041940192 |
| YFL050C | ALR2 | 0.15269937 | 0.27466431 | 0.78357635 | 0.999976173 | 0.041941066 |
| YJL116C | NCA3 | 0.15269937 | 0.27491432 | 0.78338426 | 0.999976173 | 0.041979242 |
| YDL079C | MRK1 | 0.15269937 | 0.27511462 | 0.78323037 | 0.999976173 | 0.042009829 |
| YKR042W | UTH1 | 0.15269937 | 0.27531589 | 0.78307575 | 0.999976173 | 0.042040562 |
| YDR312W | SSF2 | 0.15269937 | 0.27597731 | 0.7825677 | 0.999976173 | 0.04214156 |
| YJL157C | FAR1 | 0.15269937 | 0.27615211 | 0.78243344 | 0.999976173 | 0.042168252 |
| YKL072W | STB6 | 0.15269937 | 0.27647736 | 0.78218365 | 0.999976173 | 0.042217917 |
| YBR194W | AIM4 | 0.15269937 | 0.27667647 | 0.78203075 | 0.999976173 | 0.042248321 |
| YKL127W | PGM1 | 0.15269937 | 0.27724683 | 0.7815928 | 0.999976173 | 0.042335415 |
| YCL034W | LSB5 | 0.15269937 | 0.2780867 | 0.78094802 | 0.999976173 | 0.042463663 |
| YKR052C | MRS4 | 0.15269937 | 0.27814438 | 0.78090375 | 0.999976173 | 0.04247247 |
| YER134C | YER134C | 0.15269937 | 0.27833112 | 0.78076041 | 0.999976173 | 0.042500985 |
| YPL119C | DBP1 | 0.15269937 | 0.27836153 | 0.78073707 | 0.999976173 | 0.042505629 |
| YLR375W | STP3 | 0.15269937 | 0.27862333 | 0.78053613 | 0.999976173 | 0.042545606 |
| YMR233W | TRI1 | 0.15269937 | 0.27883543 | 0.78037335 | 0.999976173 | 0.042577993 |
| YOR033C | 8 | 0.15269937 | 0.27899352 | 0.78025202 | 0.999976173 | 0.042602134 |
| YOR316C | COT1 | 0.15269937 | 0.2798584 | 0.77958838 | 0.999976173 | 0.042734201 |
| YJL037W | IRC18 | 0.15269937 | 0.27990145 | 0.77955535 | 0.999976173 | 0.042740774 |
| YER163C | YER163C | 0.15269937 | 0.27991976 | 0.7795413 | 0.999976173 | 0.042743569 |
| YJL132W | YJL132W | 0.15269937 | 0.2802557 | 0.77928358 | 0.999976173 | 0.042794867 |
| YDL237W | AIM6 | 0.15269937 | 0.28068361 | 0.77895533 | 0.999976173 | 0.042860209 |
| YOL032W | OPI10 | 0.15269937 | 0.28077758 | 0.77888324 | 0.999976173 | 0.042874559 |
| YCR063W | BUD31 | 0.15269937 | 0.2813954 | 0.77840939 | 0.999976173 | 0.0429689 |
| YLR019W | PSR2 | 0.15269937 | 0.28163722 | 0.77822395 | 0.999976173 | 0.043005825 |
| YER175C | TMT1 | 0.15269937 | 0.2817509 | 0.77813677 | 0.999976173 | 0.043023184 |
| YPR106W | ISR1 | 0.15269937 | 0.28267139 | 0.77743101 | 0.999976173 | 0.043163743 |
| YEL020C | YEL020C | 0.15269937 | 0.28283737 | 0.77730377 | 0.999976173 | 0.043189087 |
| YPL249C | GYP5 | 0.15269937 | 0.28317525 | 0.77704476 | 0.999976173 | 0.043240681 |
| YFL032W | YFL032W | 0.15269937 | 0.28322509 | 0.77700656 | 0.999976173 | 0.043248291 |
| YAL013W | DEP1 | 0.15269937 | 0.28368141 | 0.77665681 | 0.999976173 | 0.043317971 |
| YDL025C | YDL025C | 0.15269937 | 0.28385685 | 0.77652235 | 0.999976173 | 0.043344762 |
| YKL075C | YKL075C | 0.15269937 | 0.28395138 | 0.77644991 | 0.999976173 | 0.043359195 |
| YCR059C | YIH1 | 0.15269937 | 0.28414128 | 0.77630438 | 0.999976173 | 0.043388193 |
| YER186C | YER186C | 0.15269937 | 0.2848987 | 0.77572403 | 0.999976173 | 0.04350385 |
| YIL151C | YIL151C | 0.15269937 | 0.28531347 | 0.77540628 | 0.999976173 | 0.043567186 |
| YDR316W | OMS1 | 0.15269937 | 0.28537708 | 0.77535755 | 0.999976173 | 0.043576899 |
| YDR179W-A | YDR179W-A | 0.15269937 | 0.28562944 | 0.77516423 | 0.999976173 | 0.043615435 |
| YDR520C | URC2 | 0.15269937 | 0.2865881 | 0.77443002 | 0.999976173 | 0.043761822 |
| YBR166C | TYR1 | 0.15269937 | 0.28710796 | 0.77403196 | 0.999976173 | 0.043841203 |
| YHR112C | YHR112C | 0.15269937 | 0.28763075 | 0.7736317 | 0.999976173 | 0.043921033 |
| YDL038C | NA | 0.15269937 | 0.28766865 | 0.77360269 | 0.999976173 | 0.043926821 |
| YBR255W | MTC4 | 0.15269937 | 0.28779287 | 0.7735076 | 0.999976173 | 0.043945789 |
| YLR077W | FMP25 | 0.15269937 | 0.28792568 | 0.77340593 | 0.999976173 | 0.043966068 |
| YFL028C | CAF16 | 0.15269937 | 0.28810681 | 0.77326729 | 0.999976173 | 0.043993727 |
| YGR295C | COS6 | 0.15269937 | 0.28839798 | 0.77304442 | 0.999976173 | 0.044038189 |
| YDL056W | MBP1 | 0.15269937 | 0.2887498 | 0.77277516 | 0.999976173 | 0.044091911 |
| YJL193W | YJL193W | 0.15269937 | 0.28901146 | 0.77257492 | 0.999976173 | 0.044131866 |
| YPL267W | ACM1 | 0.15269937 | 0.28917772 | 0.7724477 | 0.999976173 | 0.044157254 |
| YDL121C | YDL121C | 0.15269937 | 0.28932742 | 0.77233314 | 0.999976173 | 0.044180114 |
| YKL091C | YKL091C | 0.15269937 | 0.28963859 | 0.77209506 | 0.999976173 | 0.044227628 |
| YLR278C | YLR278C | 0.15269937 | 0.29004574 | 0.77178356 | 0.999976173 | 0.044289801 |
| YJL099W | CHS6 | 0.15269937 | 0.29024051 | 0.77163457 | 0.999976173 | 0.044319542 |
| YPL066W | YPL066W | 0.15269937 | 0.29034959 | 0.77155113 | 0.999976173 | 0.044336199 |
| YDR401W | YDR401W | 0.15269937 | 0.29087442 | 0.77114969 | 0.999976173 | 0.04441634 |
| YIL001W | YIL001W | 0.15269937 | 0.29130725 | 0.77081868 | 0.999976173 | 0.044482432 |
| YOR172W | YRM1 | 0.15269937 | 0.29161499 | 0.77058335 | 0.999976173 | 0.044529424 |
| YEL062W | NPR2 | 0.15269937 | 0.29223861 | 0.77010653 | 0.999976173 | 0.04462465 |
| YPL001W | HAT1 | 0.15269937 | 0.29228377 | 0.77007201 | 0.999976173 | 0.044631547 |

| | | | | | | |
|---|---|---|---|---|---|---|
| YER024W | YAT2 | 0.15269937 | 0.29303973 | 0.76949413 | 0.999976173 | 0.044746981 |
| YBL028C | YBL028C | 0.15269937 | 0.29326775 | 0.76931985 | 0.999976173 | 0.044781799 |
| YLR220W | CCC1 | 0.15269937 | 0.29331592 | 0.76928303 | 0.999976173 | 0.044789155 |
| YDR249C | YDR249C | 0.15269937 | 0.2933779 | 0.76923567 | 0.999976173 | 0.044798619 |
| YPL064C | CWC27 | 0.15269937 | 0.29372466 | 0.76897066 | 0.999976173 | 0.04485157 |
| YGR283C | YGR283C | 0.15269937 | 0.29383144 | 0.76888906 | 0.999976173 | 0.044867874 |
| YBR240C | THI2 | 0.15269937 | 0.29417533 | 0.76862629 | 0.999976173 | 0.044920386 |
| YLR290C | YLR290C | 0.15269937 | 0.29452009 | 0.76836288 | 0.999976173 | 0.04497303 |
| YMR252C | YMR252C | 0.15269937 | 0.2946099 | 0.76829426 | 0.999976173 | 0.044986744 |
| YDR289C | RTT103 | 0.15269937 | 0.29494504 | 0.76803823 | 0.999976173 | 0.04503792 |
| YGR273C | YGR273C | 0.15269937 | 0.29496824 | 0.76802051 | 0.999976173 | 0.045041462 |
| YMR188C | MRPS17 | 0.15269937 | 0.29646474 | 0.76687757 | 0.999976173 | 0.045269978 |
| YCR022C | YCR022C | 0.15269937 | 0.29661248 | 0.76676476 | 0.999976173 | 0.045292537 |
| YAL017W | PSK1 | 0.15269937 | 0.29661458 | 0.76676315 | 0.999976173 | 0.045292859 |
| YKL163W | PIR3 | 0.15269937 | 0.29696466 | 0.76649587 | 0.999976173 | 0.045346315 |
| YER077C | YER077C | 0.15269937 | 0.29697024 | 0.76649161 | 0.999976173 | 0.045347167 |
| YFR016C | YFR016C | 0.15269937 | 0.29700775 | 0.76646297 | 0.999976173 | 0.045352895 |
| YAL008W | FUN14 | 0.15269937 | 0.29737501 | 0.7661826 | 0.999976173 | 0.045408976 |
| YOL095C | HMI1 | 0.15269937 | 0.29767066 | 0.76595692 | 0.999976173 | 0.045454121 |
| YDR384C | ATO3 | 0.15269937 | 0.29796117 | 0.76573519 | 0.999976173 | 0.045498481 |
| YNR027W | BUD17 | 0.15269937 | 0.2980409 | 0.76567434 | 0.999976173 | 0.045510657 |
| YKR102W | FLO10 | 0.15269937 | 0.29843335 | 0.76537484 | 0.999976173 | 0.045570583 |
| YNL175C | NOP13 | 0.15269937 | 0.29853826 | 0.76529478 | 0.999976173 | 0.045586603 |
| YBR206W | YBR206W | 0.15269937 | 0.29877516 | 0.76511401 | 0.999976173 | 0.045622777 |
| YFL048C | EMP47 | 0.15269937 | 0.29905392 | 0.7649013 | 0.999976173 | 0.045665344 |
| YPL246C | RBD2 | 0.15269937 | 0.29963363 | 0.76445903 | 0.999976173 | 0.045753866 |
| YDL149W | ATG9 | 0.15269937 | 0.29992234 | 0.7642388 | 0.999976173 | 0.045797951 |
| YIL040W | APQ12 | 0.15269937 | 0.30001864 | 0.76416535 | 0.999976173 | 0.045812656 |
| YPR031W | NTO1 | 0.15269937 | 0.3014897 | 0.76304353 | 0.999976173 | 0.046037286 |
| YML033W | NA | 0.15269937 | 0.30166636 | 0.76290884 | 0.999976173 | 0.046064262 |
| YHR094C | HXT1 | 0.15269937 | 0.30320326 | 0.7617374 | 0.999976173 | 0.046298945 |
| YMR222C | FSH2 | 0.15269937 | 0.30324632 | 0.76170459 | 0.999976173 | 0.04630552 |
| YDL226C | GCS1 | 0.15269937 | 0.30329258 | 0.76166933 | 0.999976173 | 0.046312585 |
| YPR028W | YOP1 | 0.15269937 | 0.30337682 | 0.76160514 | 0.999976173 | 0.046325449 |
| YCR098C | GIT1 | 0.15269937 | 0.30349817 | 0.76151268 | 0.999976173 | 0.046343978 |
| YGR084C | MRP13 | 0.15269937 | 0.30371381 | 0.76134838 | 0.999976173 | 0.046376906 |
| YGR176W | YGR176W | 0.15269937 | 0.30399278 | 0.76113583 | 0.999976173 | 0.046419505 |
| YKR074W | AIM29 | 0.15269937 | 0.3046609 | 0.76062688 | 0.999976173 | 0.046521527 |
| YOR140W | SFL1 | 0.15269937 | 0.30470056 | 0.76059667 | 0.999976173 | 0.046527582 |
| YMR304W | UBP15 | 0.15269937 | 0.30557423 | 0.7599313 | 0.999976173 | 0.046660991 |
| YMR263W | SAP30 | 0.15269937 | 0.30579568 | 0.75976268 | 0.999976173 | 0.046694806 |
| YJL126W | NIT2 | 0.15269937 | 0.30584723 | 0.75972343 | 0.999976173 | 0.046702678 |
| YBR220C | YBR220C | 0.15269937 | 0.30588707 | 0.75969309 | 0.999976173 | 0.046708762 |
| YOR180C | DCI1 | 0.15269937 | 0.30641045 | 0.75929462 | 0.999976173 | 0.046788681 |
| YDR423C | CAD1 | 0.15269937 | 0.30662221 | 0.75913341 | 0.999976173 | 0.046821017 |
| YPL207W | TYW1 | 0.15269937 | 0.30696514 | 0.75887237 | 0.999976173 | 0.046873382 |
| YPR122W | AXL1 | 0.15269937 | 0.30705313 | 0.7588054 | 0.999976173 | 0.046886818 |
| YNR061C | YNR061C | 0.15269937 | 0.30833304 | 0.75783141 | 0.999976173 | 0.047082259 |
| YOR087W | YVC1 | 0.15269937 | 0.30836607 | 0.75780628 | 0.999976173 | 0.047087303 |
| YLR187W | SKG3 | 0.15269937 | 0.3084964 | 0.75770712 | 0.999976173 | 0.047107205 |
| YCR031C | RPS14A | 0.15269937 | 0.30859199 | 0.7576344 | 0.999976173 | 0.047121801 |
| YBL100C | YBL100C | 0.15269937 | 0.30920142 | 0.7571708 | 0.999976173 | 0.047214861 |
| YHR097C | YHR097C | 0.15269937 | 0.30979843 | 0.75671674 | 0.999976173 | 0.047306024 |
| YOR351C | MEK1 | 0.15269937 | 0.31141826 | 0.75548518 | 0.999976173 | 0.047553371 |
| YPL214C | THI6 | 0.15269937 | 0.31156372 | 0.75537462 | 0.999976173 | 0.047575582 |
| YML128C | MSC1 | 0.15269937 | 0.31200848 | 0.75503659 | 0.999976173 | 0.047643497 |
| YDR275W | BSC2 | 0.15269937 | 0.31218959 | 0.75489896 | 0.999976173 | 0.047671152 |
| YML009c | NA | 0.15269937 | 0.31295845 | 0.75431475 | 0.999976173 | 0.047788557 |
| YKR066C | CCP1 | 0.15269937 | 0.31385648 | 0.75363257 | 0.999976173 | 0.047925686 |
| YLL009C | COX17 | 0.15269937 | 0.3143092 | 0.75328874 | 0.999976173 | 0.047994816 |
| YHL040C | ARN1 | 0.15269937 | 0.31448782 | 0.7531531 | 0.999976173 | 0.04802209 |
| YNL305C | YNL305C | 0.15269937 | 0.31481072 | 0.75290791 | 0.999976173 | 0.048071397 |
| YPL253C | VIK1 | 0.15269937 | 0.31486815 | 0.7528643 | 0.999976173 | 0.048080167 |
| YLL024C | SSA2 | 0.15269937 | 0.31561852 | 0.75229462 | 0.999976173 | 0.048194748 |

| | | | | | | |
|---|---|---|---|---|---|---|
| YBR025C | OLA1 | 0.15269937 | 0.31594276 | 0.7520485 | 0.999976173 | 0.048244259 |
| YJR083C | ACF4 | 0.15269937 | 0.31675209 | 0.75143428 | 0.999976173 | 0.048367843 |
| YKR106W | YKR106W | 0.15269937 | 0.31708161 | 0.75118423 | 0.999976173 | 0.048418161 |
| YDL086W | YDL086W | 0.15269937 | 0.3178105 | 0.75063125 | 0.999976173 | 0.048529462 |
| YPR194C | OPT2 | 0.15269937 | 0.31781269 | 0.75062958 | 0.999976173 | 0.048529797 |
| YGL168W | HUR1 | 0.15269937 | 0.31823934 | 0.75030596 | 0.999976173 | 0.048594945 |
| YGL017W | ATE1 | 0.15269937 | 0.31922267 | 0.74956024 | 0.999976173 | 0.048745099 |
| YLR232W | YLR232W | 0.15269937 | 0.31985622 | 0.74907991 | 0.999976173 | 0.048841841 |
| YOR062C | YOR062C | 0.15269937 | 0.3203023 | 0.74874177 | 0.999976173 | 0.048909958 |
| YKL171W | YKL171W | 0.15269937 | 0.32093925 | 0.74825902 | 0.999976173 | 0.04900722 |
| YFR054C | YFR054C | 0.15269937 | 0.32100136 | 0.74821195 | 0.999976173 | 0.049016703 |
| YDR357C | YDR357C | 0.15269937 | 0.32159199 | 0.7477644 | 0.999976173 | 0.049106894 |
| YER064C | YER064C | 0.15269937 | 0.32323545 | 0.74651955 | 0.999976173 | 0.049357847 |
| YPL038W | MET31 | 0.15269937 | 0.32403459 | 0.74591446 | 0.999976173 | 0.049479877 |
| YDR075W | PPH3 | 0.15269937 | 0.32461551 | 0.74547471 | 0.999976173 | 0.049568583 |
| YER144C | UBP5 | 0.15269937 | 0.32479575 | 0.74533828 | 0.999976173 | 0.049596106 |
| YOR135C | IRC14 | 0.15269937 | 0.3251761 | 0.74505042 | 0.999976173 | 0.049654185 |
| YMR292W | GOT1 | 0.15269937 | 0.32572247 | 0.74463697 | 0.999976173 | 0.049737615 |
| YOL136C | PFK27 | 0.15269937 | 0.32649128 | 0.74405532 | 0.999976173 | 0.049855012 |
| YJR005W | APL1 | 0.15269937 | 0.32671439 | 0.74388656 | 0.999976173 | 0.04988908 |
| YBL042C | FUI1 | 0.15269937 | 0.32717289 | 0.74353977 | 0.999976173 | 0.049959092 |
| YDL011C | YDL011C | 0.15269937 | 0.32722085 | 0.7435035 | 0.999976173 | 0.049966416 |
| YBR248C | HIS7 | 0.15269937 | 0.32724489 | 0.74348532 | 0.999976173 | 0.049970087 |
| YMR173W-A | YMR173W-A | 0.15269937 | 0.3277803 | 0.74308043 | 0.999976173 | 0.050051844 |
| YIR042C | YIR042C | 0.15269937 | 0.32781456 | 0.74305453 | 0.999976173 | 0.050057075 |
| YMR186W | HSC82 | 0.15269937 | 0.32783839 | 0.74303651 | 0.999976173 | 0.050060714 |
| YBR177C | EHT1 | 0.15269937 | 0.32846346 | 0.74256392 | 0.999976173 | 0.050156162 |
| YKL086W | SRX1 | 0.15269937 | 0.32896386 | 0.74218567 | 0.999976173 | 0.050232572 |
| YCL055W | KAR4 | 0.15269937 | 0.32980402 | 0.74155072 | 0.999976173 | 0.050360865 |
| YNL145W | MFA2 | 0.15269937 | 0.33092425 | 0.74070438 | 0.999976173 | 0.050531923 |
| YGL153W | PEX14 | 0.15269937 | 0.33218425 | 0.73975282 | 0.999976173 | 0.050724324 |
| YKL090W | CUE2 | 0.15269937 | 0.3324881 | 0.73952342 | 0.999976173 | 0.050770722 |
| YKL100C | YKL100C | 0.15269937 | 0.33253439 | 0.73948846 | 0.999976173 | 0.050777791 |
| YKL150W | MCR1 | 0.15269937 | 0.33277638 | 0.73930578 | 0.999976173 | 0.050814742 |
| YGL212W | VAM7 | 0.15269937 | 0.33303042 | 0.73911402 | 0.999976173 | 0.050853533 |
| YNL325C | FIG4 | 0.15269937 | 0.33305645 | 0.73909437 | 0.999976173 | 0.050857509 |
| YBR296C | PHO89 | 0.15269937 | 0.33341578 | 0.73882315 | 0.999976173 | 0.050912378 |
| YDR284C | DPP1 | 0.15269937 | 0.33397672 | 0.73839983 | 0.999976173 | 0.050998033 |
| YKL197C | PEX1 | 0.15269937 | 0.333996 | 0.73838528 | 0.999976173 | 0.051000978 |
| YOR304W | ISW2 | 0.15269937 | 0.33451337 | 0.73799491 | 0.999976173 | 0.051079979 |
| YML081C-A | ATP18 | 0.15269937 | 0.33530815 | 0.73739536 | 0.999976173 | 0.051201342 |
| YLL039C | UBI4 | 0.15269937 | 0.33534553 | 0.73736717 | 0.999976173 | 0.05120705 |
| YKL161C | YKL161C | 0.15269937 | 0.33593823 | 0.73692017 | 0.999976173 | 0.051297555 |
| YGR173W | RBG2 | 0.15269937 | 0.3373919 | 0.73582422 | 0.999976173 | 0.051519529 |
| YJL146W | IDS2 | 0.15269937 | 0.33771443 | 0.73558114 | 0.999976173 | 0.051568779 |
| YDR430C | CYM1 | 0.15269937 | 0.33803523 | 0.73533938 | 0.999976173 | 0.051617765 |
| YPR073C | LTP1 | 0.15269937 | 0.33834756 | 0.73510403 | 0.999976173 | 0.051665458 |
| YDR516C | EMI2 | 0.15269937 | 0.33841101 | 0.73505622 | 0.999976173 | 0.051675146 |
| YBR175W | SWD3 | 0.15269937 | 0.33854286 | 0.73495688 | 0.999976173 | 0.05169528 |
| YNL044W | YIP3 | 0.15269937 | 0.33889525 | 0.73469139 | 0.999976173 | 0.05174909 |
| YPL245W | YPL245W | 0.15269937 | 0.33971129 | 0.73407671 | 0.999976173 | 0.051873699 |
| YGR244C | LSC2 | 0.15269937 | 0.34003355 | 0.73383402 | 0.999976173 | 0.051922908 |
| YNL280C | ERG24 | 0.15269937 | 0.34118197 | 0.73296936 | 0.999976173 | 0.05209827 |
| YPL256C | CLN2 | 0.15269937 | 0.34227542 | 0.73214641 | 0.999976173 | 0.052265239 |
| YKL149C | DBR1 | 0.15269937 | 0.34270518 | 0.73182304 | 0.999976173 | 0.052330864 |
| YPR003C | YPR003C | 0.15269937 | 0.34321416 | 0.73144014 | 0.999976173 | 0.052408585 |
| YOR368W | 13 | 0.15269937 | 0.34328332 | 0.73138811 | 0.999976173 | 0.052419145 |
| YML016C | PPZ1 | 0.15269937 | 0.34374781 | 0.73103874 | 0.999976173 | 0.052490072 |
| YDL186W | YDL186W | 0.15269937 | 0.34381743 | 0.73098638 | 0.999976173 | 0.052500704 |
| YDR006C | SOK1 | 0.15269937 | 0.34433261 | 0.73059896 | 0.999976173 | 0.052579371 |
| YMR194C-A | YMR194C-A | 0.15269937 | 0.34458045 | 0.7304126 | 0.999976173 | 0.052617216 |
| YDL071C | YDL071C | 0.15269937 | 0.34535233 | 0.72983231 | 0.999976173 | 0.052735082 |
| YDL170W | UGA3 | 0.15269937 | 0.3458394 | 0.72946622 | 0.999976173 | 0.052809457 |
| YLL052C | AQY2 | 0.15269937 | 0.34594113 | 0.72938977 | 0.999976173 | 0.052824991 |

| | | | | | | |
|---|---|---|---|---|---|---|
| YHR022C | YHR022C | 0.15269937 | 0.34665118 | 0.7288562 | 0.999976173 | 0.052933416 |
| YPL221W | FLC1 | 0.15269937 | 0.34666703 | 0.7288443 | 0.999976173 | 0.052935835 |
| YKL070W | YKL070W | 0.15269937 | 0.34743866 | 0.72826462 | 0.999976173 | 0.053053662 |
| YDR074W | TPS2 | 0.15269937 | 0.34821298 | 0.72768307 | 0.999976173 | 0.0531719 |
| YML057W | CMP2 | 0.15269937 | 0.34844757 | 0.72750692 | 0.999976173 | 0.053207722 |
| YNL063W | MTQ1 | 0.15269937 | 0.34892503 | 0.72714843 | 0.999976173 | 0.053280631 |
| YFR044C | DUG1 | 0.15269937 | 0.34901519 | 0.72708075 | 0.999976173 | 0.053294398 |
| YLR011W | LOT6 | 0.15269937 | 0.34986401 | 0.7264436 | 0.999976173 | 0.053424012 |
| YIL158W | AIM20 | 0.15269937 | 0.350288 | 0.72612542 | 0.999976173 | 0.053488756 |
| YMR173W | DDR48 | 0.15269937 | 0.35053754 | 0.72593817 | 0.999976173 | 0.05352686 |
| YHR116W | COX23 | 0.15269937 | 0.35062046 | 0.72587596 | 0.999976173 | 0.053539522 |
| YDR111C | ALT2 | 0.15269937 | 0.35096741 | 0.72561565 | 0.999976173 | 0.053592502 |
| YGL013C | PDR1 | 0.15269937 | 0.35130962 | 0.72535893 | 0.999976173 | 0.053644757 |
| YPL229W | YPL229W | 0.15269937 | 0.35250797 | 0.72446021 | 0.999976173 | 0.053827744 |
| YOR231W | MKK1 | 0.15269937 | 0.35275213 | 0.72427714 | 0.999976173 | 0.053865027 |
| YNL171C | YNL171C | 0.15269937 | 0.35315059 | 0.72397842 | 0.999976173 | 0.053925872 |
| YDR221W | GTB1 | 0.15269937 | 0.35343058 | 0.72376854 | 0.999976173 | 0.053968625 |
| YOR314W | YOR314W | 0.15269937 | 0.35346073 | 0.72374594 | 0.999976173 | 0.053973229 |
| YBR009C | HHF1 | 0.15269937 | 0.35367469 | 0.72358557 | 0.999976173 | 0.054005901 |
| YIL055C | YIL055C | 0.15269937 | 0.3543833 | 0.72305453 | 0.999976173 | 0.054114105 |
| YLR170C | APS1 | 0.15269937 | 0.35461601 | 0.72288016 | 0.999976173 | 0.054149641 |
| YOR100C | CRC1 | 0.15269937 | 0.35506113 | 0.72254668 | 0.999976173 | 0.05421761 |
| YLR332W | MID2 | 0.15269937 | 0.35538754 | 0.72230218 | 0.999976173 | 0.054267451 |
| YBL005W | PDR3 | 0.15269937 | 0.35596106 | 0.72187262 | 0.999976173 | 0.054355029 |
| YCR079W | PTC6 | 0.15269937 | 0.35670756 | 0.72131365 | 0.999976173 | 0.054469018 |
| YOR163W | DDP1 | 0.15269937 | 0.35713539 | 0.72099336 | 0.999976173 | 0.054534347 |
| YDL176W | YDL176W | 0.15269937 | 0.35720971 | 0.72093773 | 0.999976173 | 0.054545696 |
| YDR391C | YDR391C | 0.15269937 | 0.35816545 | 0.72022242 | 0.999976173 | 0.054691638 |
| YMR172W | HOT1 | 0.15269937 | 0.35870002 | 0.71982244 | 0.999976173 | 0.054773265 |
| YDR152W | GIR2 | 0.15269937 | 0.35879212 | 0.71975353 | 0.999976173 | 0.05478733 |
| YPL203W | TPK2 | 0.15269937 | 0.35957353 | 0.71916901 | 0.999976173 | 0.054906649 |
| YLR110C | CCW12 | 0.15269937 | 0.36017 | 0.71872294 | 0.999976173 | 0.054997731 |
| YIL122W | POG1 | 0.15269937 | 0.36071524 | 0.71831527 | 0.999976173 | 0.055080989 |
| YDR100W | TVP15 | 0.15269937 | 0.36093149 | 0.7181536 | 0.999976173 | 0.05511401 |
| YLR094C | GIS3 | 0.15269937 | 0.36097453 | 0.71812143 | 0.999976173 | 0.055120582 |
| YJR096W | YJR096W | 0.15269937 | 0.36168458 | 0.7175907 | 0.999976173 | 0.055229005 |
| YJL188C | BUD19 | 0.15269937 | 0.36211058 | 0.71727235 | 0.999976173 | 0.055294055 |
| YOR016C | ERP4 | 0.15269937 | 0.36260486 | 0.71690304 | 0.999976173 | 0.055369532 |
| YNR022C | MRPL50 | 0.15269937 | 0.36345331 | 0.71626925 | 0.999976173 | 0.055499091 |
| YLR337C | VRP1 | 0.15269937 | 0.36405359 | 0.71582097 | 0.999976173 | 0.055590752 |
| YHR138C | YHR138C | 0.15269937 | 0.36435731 | 0.71559418 | 0.999976173 | 0.055637131 |
| YNL169C | PSD1 | 0.15269937 | 0.36494238 | 0.7151574 | 0.999976173 | 0.05572647 |
| YLR264W | RPS28B | 0.15269937 | 0.36506137 | 0.71506858 | 0.999976173 | 0.055744639 |
| YML026C | RPS18B | 0.15269937 | 0.36529765 | 0.71489222 | 0.999976173 | 0.055780719 |
| YMR265C | YMR265C | 0.15269937 | 0.36539315 | 0.71482093 | 0.999976173 | 0.055795303 |
| YLR122C | YLR122C | 0.15269937 | 0.36647162 | 0.71401618 | 0.999976173 | 0.055959984 |
| YIR044C | YIR044C | 0.15269937 | 0.36659509 | 0.71392407 | 0.999976173 | 0.055978837 |
| YFR055W | IRC7 | 0.15269937 | 0.36693609 | 0.71366968 | 0.999976173 | 0.056030909 |
| YMR080C | 29 | 0.15269937 | 0.36704789 | 0.71358629 | 0.999976173 | 0.056047981 |
| YIR003W | AIM21 | 0.15269937 | 0.36796731 | 0.71290061 | 0.999976173 | 0.056188375 |
| YDL109C | YDL109C | 0.15269937 | 0.36812054 | 0.71278637 | 0.999976173 | 0.056211773 |
| YGR238C | KEL2 | 0.15269937 | 0.36816131 | 0.71275597 | 0.999976173 | 0.056217999 |
| YER074W | RPS24A | 0.15269937 | 0.3687322 | 0.71233036 | 0.999976173 | 0.056305173 |
| YIL098C | FMC1 | 0.15269937 | 0.36945344 | 0.71179279 | 0.999976173 | 0.056415306 |
| YOL024W | YOL024W | 0.15269937 | 0.36956736 | 0.71170789 | 0.999976173 | 0.056432702 |
| YPL113C | YPL113C | 0.15269937 | 0.37001899 | 0.71137137 | 0.999976173 | 0.056501665 |
| YMR279C | YMR279C | 0.15269937 | 0.37125791 | 0.71044848 | 0.999976173 | 0.056690848 |
| YNR012W | URK1 | 0.15269937 | 0.37126627 | 0.71044226 | 0.999976173 | 0.056692124 |
| YIL117C | PRM5 | 0.15269937 | 0.3713976 | 0.71034445 | 0.999976173 | 0.056712177 |
| YER078C | ICP55 | 0.15269937 | 0.37227841 | 0.70968862 | 0.999976173 | 0.056846677 |
| YNL300W | TOS6 | 0.15269937 | 0.37261752 | 0.70943618 | 0.999976173 | 0.056898459 |
| YMR129W | POM152 | 0.15269937 | 0.37299192 | 0.70915751 | 0.999976173 | 0.056955629 |
| YFR043C | IRC6 | 0.15269937 | 0.37307482 | 0.70909581 | 0.999976173 | 0.056968288 |
| YGR221C | TOS2 | 0.15269937 | 0.37483431 | 0.70778676 | 0.999976173 | 0.057236962 |

| | | | | | | |
|---|---|---|---|---|---|---|
| YOL068C | HST1 | 0.15269937 | 0.37507297 | 0.70760927 | 0.999976173 | 0.057273404 |
| YJL133W | MRS3 | 0.15269937 | 0.37518571 | 0.70752543 | 0.999976173 | 0.05729062 |
| YGR272C | NA | 0.15269937 | 0.37588871 | 0.70700271 | 0.999976173 | 0.057397968 |
| YML017W | PSP2 | 0.15269937 | 0.37606455 | 0.70687199 | 0.999976173 | 0.057424818 |
| YCR107W | AAD3 | 0.15269937 | 0.37696568 | 0.7062022 | 0.999976173 | 0.057562421 |
| YCR020C-A | MAK31 | 0.15269937 | 0.37723407 | 0.70600276 | 0.999976173 | 0.057603402 |
| YJL053W | PEP8 | 0.15269937 | 0.37797833 | 0.70544979 | 0.999976173 | 0.057717051 |
| YPR167C | MET16 | 0.15269937 | 0.37849931 | 0.70506281 | 0.999976173 | 0.057796604 |
| YKL206C | ADD66 | 0.15269937 | 0.37852871 | 0.70504097 | 0.999976173 | 0.057801094 |
| YDL089W | NUR1 | 0.15269937 | 0.37945967 | 0.70434966 | 0.999976173 | 0.057943251 |
| YBR272C | HSM3 | 0.15269937 | 0.37990932 | 0.70401585 | 0.999976173 | 0.058011912 |
| YLR410W | VIP1 | 0.15269937 | 0.37995479 | 0.70398209 | 0.999976173 | 0.058018856 |
| YBR130C | SHE3 | 0.15269937 | 0.38015744 | 0.70383167 | 0.999976173 | 0.0580498 |
| YIR043C | YIR043C | 0.15269937 | 0.38080181 | 0.70335344 | 0.999976173 | 0.058148195 |
| YDR108W | GSG1 | 0.15269937 | 0.38086287 | 0.70330813 | 0.999976173 | 0.058157519 |
| YLR357W | RSC2 | 0.15269937 | 0.38097036 | 0.70322837 | 0.999976173 | 0.058173932 |
| YML055W | SPC2 | 0.15269937 | 0.38103977 | 0.70317687 | 0.999976173 | 0.05818453 |
| YOR040W | GLO4 | 0.15269937 | 0.38166981 | 0.70270943 | 0.999976173 | 0.058280738 |
| YFR056C | YFR056C | 0.15269937 | 0.38200186 | 0.70246312 | 0.999976173 | 0.058331442 |
| YKL178C | STE3 | 0.15269937 | 0.38242363 | 0.7021503 | 0.999976173 | 0.058395846 |
| YCL014W | BUD3 | 0.15269937 | 0.38242905 | 0.70214628 | 0.999976173 | 0.058396674 |
| YBR013C | YBR013C | 0.15269937 | 0.38273523 | 0.70191923 | 0.999976173 | 0.058443427 |
| YIL100W | YIL100W | 0.15269937 | 0.38299397 | 0.70172738 | 0.999976173 | 0.058482936 |
| YOR223W | YOR223W | 0.15269937 | 0.38327105 | 0.70152195 | 0.999976173 | 0.058525247 |
| YJL094C | KHA1 | 0.15269937 | 0.3841361 | 0.70088073 | 0.999976173 | 0.058657339 |
| YNL257C | SIP3 | 0.15269937 | 0.38417148 | 0.70085451 | 0.999976173 | 0.058662741 |
| YDR241W | BUD26 | 0.15269937 | 0.38522883 | 0.70007105 | 0.999976173 | 0.058824198 |
| YAL060W | BDH1 | 0.15269937 | 0.38569763 | 0.69972378 | 0.999976173 | 0.058895784 |
| YJL158C | CIS3 | 0.15269937 | 0.38592982 | 0.69955181 | 0.999976173 | 0.058931239 |
| YAL062W | GDH3 | 0.15269937 | 0.38594011 | 0.6995442 | 0.999976173 | 0.058932809 |
| YJL131C | AIM23 | 0.15269937 | 0.38680517 | 0.69890363 | 0.999976173 | 0.059064904 |
| YER164W | CHD1 | 0.15269937 | 0.38730814 | 0.69853128 | 0.999976173 | 0.059141707 |
| YIR039C | YPS6 | 0.15269937 | 0.38797155 | 0.69804027 | 0.999976173 | 0.05924301 |
| YPL147W | PXA1 | 0.15269937 | 0.38940349 | 0.69698089 | 0.999976173 | 0.059461666 |
| YLR142W | PUT1 | 0.15269937 | 0.3896872 | 0.69677107 | 0.999976173 | 0.059504988 |
| YLR171W | YLR171W | 0.15269937 | 0.38988213 | 0.69662692 | 0.999976173 | 0.059534753 |
| YNR028W | CPR8 | 0.15269937 | 0.39000656 | 0.6965349 | 0.999976173 | 0.059553754 |
| YER151C | UBP3 | 0.15269937 | 0.3902792 | 0.69633331 | 0.999976173 | 0.059595386 |
| YOR318C | YOR318C | 0.15269937 | 0.39095989 | 0.6958301 | 0.999976173 | 0.059699327 |
| YKR015C | YKR015C | 0.15269937 | 0.39117325 | 0.6956724 | 0.999976173 | 0.059731908 |
| YNL324W | YNL324W | 0.15269937 | 0.39219935 | 0.69491415 | 0.999976173 | 0.059888592 |
| YJL150W | YJL150W | 0.15269937 | 0.39255146 | 0.69465402 | 0.999976173 | 0.05994236 |
| YGL077C | HNM1 | 0.15269937 | 0.39500343 | 0.69284361 | 0.999976173 | 0.060316774 |
| YMR187C | YMR187C | 0.15269937 | 0.39530197 | 0.6926233 | 0.999976173 | 0.06036236 |
| YLR380W | CSR1 | 0.15269937 | 0.39581551 | 0.69224439 | 0.999976173 | 0.060440778 |
| YML100W | TSL1 | 0.15269937 | 0.39600734 | 0.69210288 | 0.999976173 | 0.060470069 |
| YIL131C | FKH1 | 0.15269937 | 0.39628155 | 0.6919006 | 0.999976173 | 0.060511941 |
| YGR194C | XKS1 | 0.15269937 | 0.39641039 | 0.69180557 | 0.999976173 | 0.060531615 |
| YBL102W | SFT2 | 0.15269937 | 0.39656421 | 0.69169212 | 0.999976173 | 0.060555103 |
| YDR310C | SUM1 | 0.15269937 | 0.3968714 | 0.69146557 | 0.999976173 | 0.060602011 |
| YDR375C | BCS1 | 0.15269937 | 0.39713412 | 0.69127183 | 0.999976173 | 0.060642128 |
| YLR125W | YLR125W | 0.15269937 | 0.39767981 | 0.6908695 | 0.999976173 | 0.060725454 |
| YOR179C | SYC1 | 0.15269937 | 0.39785121 | 0.69074314 | 0.999976173 | 0.060751628 |
| YLR412W | BER1 | 0.15269937 | 0.39817367 | 0.69050546 | 0.999976173 | 0.060800867 |
| YML037C | YML037C | 0.15269937 | 0.39864484 | 0.6901582 | 0.999976173 | 0.060872814 |
| YPL171C | OYE3 | 0.15269937 | 0.39898662 | 0.68990635 | 0.999976173 | 0.060925004 |
| YER091C | MET6 | 0.15269937 | 0.3991435 | 0.68979076 | 0.999976173 | 0.060948959 |
| YMR121C | RPL15B | 0.15269937 | 0.40177501 | 0.68785293 | 0.999976173 | 0.061350789 |
| YLL010C | PSR1 | 0.15269937 | 0.40318526 | 0.68681527 | 0.999976173 | 0.061566133 |
| YPR012W | YPR012W | 0.15269937 | 0.4042866 | 0.68600532 | 0.999976173 | 0.061734307 |
| YML131W | YML131W | 0.15269937 | 0.40432989 | 0.68597349 | 0.999976173 | 0.061740918 |
| YIR037W | HYR1 | 0.15269937 | 0.40450776 | 0.68584271 | 0.999976173 | 0.061768079 |
| YBR057C | MUM2 | 0.15269937 | 0.40488346 | 0.68556652 | 0.999976173 | 0.061825447 |
| YDL081C | RPP1A | 0.15269937 | 0.40527698 | 0.68527727 | 0.999976173 | 0.061885538 |

| | | | | | | |
|---|---|---|---|---|---|---|
| YNL013C | YNL013C | 0.15269937 | 0.40534888 | 0.68522443 | 0.999976173 | 0.061896517 |
| YDR348C | YDR348C | 0.15269937 | 0.40556951 | 0.68506229 | 0.999976173 | 0.061930207 |
| YDR307W | YDR307W | 0.15269937 | 0.40585417 | 0.68485311 | 0.999976173 | 0.061973674 |
| YDL059C | RAD59 | 0.15269937 | 0.40609823 | 0.68467378 | 0.999976173 | 0.062010942 |
| YMR103C | YMR103C | 0.15269937 | 0.40623111 | 0.68457616 | 0.999976173 | 0.062031232 |
| YDR447C | RPS17B | 0.15269937 | 0.40663831 | 0.68427702 | 0.999976173 | 0.062093412 |
| YLR231C | BNA5 | 0.15269937 | 0.40685753 | 0.68411599 | 0.999976173 | 0.062126887 |
| YLR114C | AVL9 | 0.15269937 | 0.40741855 | 0.68370397 | 0.999976173 | 0.062212554 |
| YDL048C | STP4 | 0.15269937 | 0.40808949 | 0.68321135 | 0.999976173 | 0.062315007 |
| YDR467C | YDR467C | 0.15269937 | 0.40850628 | 0.6829054 | 0.999976173 | 0.062378649 |
| YNL195C | YNL195C | 0.15269937 | 0.40908134 | 0.68248335 | 0.999976173 | 0.062466461 |
| YKL162C | YKL162C | 0.15269937 | 0.40950491 | 0.68217255 | 0.999976173 | 0.062531141 |
| YBL059W | YBL059W | 0.15269937 | 0.40962148 | 0.68208702 | 0.999976173 | 0.062548941 |
| YAL040C | CLN3 | 0.15269937 | 0.41027215 | 0.68160971 | 0.999976173 | 0.062648297 |
| YGL079W | YGL079W | 0.15269937 | 0.41070836 | 0.68128979 | 0.999976173 | 0.062714907 |
| YGL062W | PYC1 | 0.15269937 | 0.41233651 | 0.6800962 | 0.999976173 | 0.062963524 |
| YCR090C | YCR090C | 0.15269937 | 0.41262179 | 0.67988715 | 0.999976173 | 0.063007085 |
| YER039C | HVG1 | 0.15269937 | 0.41404096 | 0.67884754 | 0.999976173 | 0.063223792 |
| YCR076C | YCR076C | 0.15269937 | 0.41456902 | 0.67846087 | 0.999976173 | 0.063304426 |
| YFL049W | SWP82 | 0.15269937 | 0.41540951 | 0.67784559 | 0.999976173 | 0.063432768 |
| YAL027W | SAW1 | 0.15269937 | 0.41655623 | 0.67700648 | 0.999976173 | 0.063607872 |
| YPR042C | PUF2 | 0.15269937 | 0.41675605 | 0.67686031 | 0.999976173 | 0.063638384 |
| YEL067C | YEL067C | 0.15269937 | 0.4185423 | 0.67555414 | 0.999976173 | 0.063911144 |
| YPR023C | EAF3 | 0.15269937 | 0.41855148 | 0.67554743 | 0.999976173 | 0.063912545 |
| YER031C | YPT31 | 0.15269937 | 0.41876558 | 0.67539093 | 0.999976173 | 0.063945239 |
| YOR035C | SHE4 | 0.15269937 | 0.41944917 | 0.67489137 | 0.999976173 | 0.064049622 |
| YMR095C | SNO1 | 0.15269937 | 0.42002296 | 0.67447216 | 0.999976173 | 0.06413724 |
| YDR130C | FIN1 | 0.15269937 | 0.42026187 | 0.67429765 | 0.999976173 | 0.06417372 |
| YOR177C | MPC54 | 0.15269937 | 0.42041076 | 0.6741889 | 0.999976173 | 0.064196456 |
| YLR057W | YLR057W | 0.15269937 | 0.42123669 | 0.67358575 | 0.999976173 | 0.064322575 |
| YOR225W | YOR225W | 0.15269937 | 0.42143379 | 0.67344185 | 0.999976173 | 0.064352672 |
| YMR317W | YMR317W | 0.15269937 | 0.42164397 | 0.67328841 | 0.999976173 | 0.064384766 |
| YIL113W | SDP1 | 0.15269937 | 0.4218326 | 0.67315071 | 0.999976173 | 0.06441357 |
| YLR035C | MLH2 | 0.15269937 | 0.42187134 | 0.67312243 | 0.999976173 | 0.064419486 |
| YPR022C | YPR022C | 0.15269937 | 0.42264065 | 0.67256097 | 0.999976173 | 0.064536959 |
| YHR185C | PFS1 | 0.15269937 | 0.42332102 | 0.67206457 | 0.999976173 | 0.064640852 |
| YDL227C | HO | 0.15269937 | 0.42363517 | 0.67183542 | 0.999976173 | 0.064688822 |
| YIL036W | CST6 | 0.15269937 | 0.42414887 | 0.67146077 | 0.999976173 | 0.064767264 |
| YOL162W | YOL162W | 0.15269937 | 0.42429784 | 0.67135214 | 0.999976173 | 0.064790011 |
| YDR181C | SAS4 | 0.15269937 | 0.42518346 | 0.67070647 | 0.999976173 | 0.064925244 |
| YPR192W | AQY1 | 0.15269937 | 0.42532738 | 0.67060157 | 0.999976173 | 0.064947221 |
| YDR426C | YDR426C | 0.15269937 | 0.42562811 | 0.67038239 | 0.999976173 | 0.064993143 |
| YBR022W | POA1 | 0.15269937 | 0.42574684 | 0.67029586 | 0.999976173 | 0.065011273 |
| YAR035W | YAT1 | 0.15269937 | 0.42579439 | 0.67026121 | 0.999976173 | 0.065018533 |
| YEL040W | UTR2 | 0.15269937 | 0.42758041 | 0.66896018 | 0.999976173 | 0.065291258 |
| YBL017C | PEP1 | 0.15269937 | 0.42769508 | 0.66887669 | 0.999976173 | 0.065308767 |
| YFL035C-B | NA | 0.15269937 | 0.42993614 | 0.66724567 | 0.999976173 | 0.065650976 |
| YCR082W | AHC2 | 0.15269937 | 0.43008334 | 0.6671386 | 0.999976173 | 0.065673453 |
| YJR077C | MIR1 | 0.15269937 | 0.43036559 | 0.66693331 | 0.999976173 | 0.065716552 |
| YJL106W | IME2 | 0.15269937 | 0.43068043 | 0.66670433 | 0.999976173 | 0.065764629 |
| YBL009W | ALK2 | 0.15269937 | 0.43104626 | 0.66643832 | 0.999976173 | 0.06582049 |
| YKL106W | AAT1 | 0.15269937 | 0.43155879 | 0.66606571 | 0.999976173 | 0.065898753 |
| YPR184W | GDB1 | 0.15269937 | 0.43174541 | 0.66593005 | 0.999976173 | 0.065927251 |
| YLR225C | YLR225C | 0.15269937 | 0.43179204 | 0.66589616 | 0.999976173 | 0.065934371 |
| YDL224C | WHI4 | 0.15269937 | 0.43228829 | 0.6655355 | 0.999976173 | 0.066010147 |
| YPL181W | CTI6 | 0.15269937 | 0.43236868 | 0.66547708 | 0.999976173 | 0.066022423 |
| YPR149W | NCE102 | 0.15269937 | 0.43257392 | 0.66532794 | 0.999976173 | 0.066053763 |
| YOR118W | RTC5 | 0.15269937 | 0.43268595 | 0.66524654 | 0.999976173 | 0.06607087 |
| YKR010C | TOF2 | 0.15269937 | 0.43288869 | 0.66509924 | 0.999976173 | 0.066101829 |
| YPL060W | LPE10 | 0.15269937 | 0.43454296 | 0.66389783 | 0.999976173 | 0.066354434 |
| YLL051C | FRE6 | 0.15269937 | 0.43455682 | 0.66388776 | 0.999976173 | 0.066356551 |
| YPL201C | YIG1 | 0.15269937 | 0.43484145 | 0.66368114 | 0.999976173 | 0.066400014 |
| YLR362W | STE11 | 0.15269937 | 0.43519398 | 0.66342526 | 0.999976173 | 0.066453845 |
| YNL334C | SNO2 | 0.15269937 | 0.43530683 | 0.66334335 | 0.999976173 | 0.066471077 |

| | | | | | | |
|---|---|---|---|---|---|---|
| YCR051W | YCR051W | 0.15269937 | 0.43610456 | 0.66276451 | 0.999976173 | 0.066592889 |
| YIL024C | YIL024C | 0.15269937 | 0.43613781 | 0.66274038 | 0.999976173 | 0.066597967 |
| YDR112W | IRC2 | 0.15269937 | 0.43623791 | 0.66266776 | 0.999976173 | 0.066613251 |
| YMR160W | YMR160W | 0.15269937 | 0.43626745 | 0.66264633 | 0.999976173 | 0.066617764 |
| YGL234W | ADE5,7 | 0.15269937 | 0.43626983 | 0.66264461 | 0.999976173 | 0.066618126 |
| YIL103W | DPH1 | 0.15269937 | 0.43685255 | 0.66222193 | 0.999976173 | 0.066707107 |
| YGL216W | KIP3 | 0.15269937 | 0.43687135 | 0.66220829 | 0.999976173 | 0.066709978 |
| YLR004C | THI73 | 0.15269937 | 0.43696483 | 0.66214049 | 0.999976173 | 0.066724253 |
| YLR307W | CDA1 | 0.15269937 | 0.43701537 | 0.66210385 | 0.999976173 | 0.06673197 |
| YJL147C | YJL147C | 0.15269937 | 0.43712747 | 0.66202255 | 0.999976173 | 0.066749087 |
| YCL064C | CHA1 | 0.15269937 | 0.43736792 | 0.66184819 | 0.999976173 | 0.066785804 |
| YGR149W | YGR149W | 0.15269937 | 0.43748167 | 0.66176571 | 0.999976173 | 0.066803173 |
| YLR107W | REX3 | 0.15269937 | 0.43757763 | 0.66169614 | 0.999976173 | 0.066817826 |
| YPL052W | OAZ1 | 0.15269937 | 0.43773263 | 0.66158376 | 0.999976173 | 0.066841495 |
| YCR011C | ADP1 | 0.15269937 | 0.43807609 | 0.66133478 | 0.999976173 | 0.066893941 |
| YIL076W | SEC28 | 0.15269937 | 0.43811183 | 0.66130887 | 0.999976173 | 0.066899399 |
| YNL326C | PFA3 | 0.15269937 | 0.43817233 | 0.66126502 | 0.999976173 | 0.066908638 |
| YKL139W | CTK1 | 0.15269937 | 0.43835673 | 0.66113136 | 0.999976173 | 0.066936795 |
| YKL142W | MRP8 | 0.15269937 | 0.43877711 | 0.66082671 | 0.999976173 | 0.067000986 |
| YDR209C | YDR209C | 0.15269937 | 0.43885386 | 0.66077109 | 0.999976173 | 0.067012706 |
| YBR235W | YBR235W | 0.15269937 | 0.43953183 | 0.66027989 | 0.999976173 | 0.067116232 |
| YPR117W | YPR117W | 0.15269937 | 0.43980498 | 0.66008203 | 0.999976173 | 0.067157941 |
| YHL009C | YAP3 | 0.15269937 | 0.44005947 | 0.65989771 | 0.999976173 | 0.067196802 |
| YKR047W | YKR047W | 0.15269937 | 0.44019084 | 0.65980256 | 0.999976173 | 0.067216863 |
| YMR234W | RNH1 | 0.15269937 | 0.44146441 | 0.65888051 | 0.999976173 | 0.067411335 |
| YDL095W | PMT1 | 0.15269937 | 0.44156103 | 0.65881058 | 0.999976173 | 0.067426089 |
| YEL006W | YEA6 | 0.15269937 | 0.44190837 | 0.6585592 | 0.999976173 | 0.067479128 |
| YOR275C | RIM20 | 0.15269937 | 0.44259728 | 0.65806075 | 0.999976173 | 0.067584323 |
| YCL048W | SPS22 | 0.15269937 | 0.44268271 | 0.65799895 | 0.999976173 | 0.067597369 |
| YPL163C | SVS1 | 0.15269937 | 0.44362234 | 0.65731936 | 0.999976173 | 0.06774085 |
| YNL164C | IBD2 | 0.15269937 | 0.44430323 | 0.65682708 | 0.999976173 | 0.067844821 |
| YPL056C | YPL056C | 0.15269937 | 0.44430663 | 0.65682462 | 0.999976173 | 0.06784534 |
| YHL028W | WSC4 | 0.15269937 | 0.44694762 | 0.65491662 | 0.999976173 | 0.068248617 |
| YDL134C | PPH21 | 0.15269937 | 0.4475296 | 0.65449646 | 0.999976173 | 0.068337487 |
| YPL100W | ATG21 | 0.15269937 | 0.44806451 | 0.65411039 | 0.999976173 | 0.068419166 |
| YOL112W | MSB4 | 0.15269937 | 0.44856512 | 0.65374915 | 0.999976173 | 0.068495609 |
| YEL028W | YEL028W | 0.15269937 | 0.44878249 | 0.65359232 | 0.999976173 | 0.068528802 |
| YKL102C | YKL102C | 0.15269937 | 0.44959663 | 0.65300508 | 0.999976173 | 0.06865312 |
| YNL315C | ATP11 | 0.15269937 | 0.44972678 | 0.65291122 | 0.999976173 | 0.068672995 |
| YIL087C | AIM19 | 0.15269937 | 0.45110123 | 0.65192037 | 0.999976173 | 0.068882872 |
| YHL016C | DUR3 | 0.15269937 | 0.45119731 | 0.65185112 | 0.999976173 | 0.068897543 |
| YGR189C | CRH1 | 0.15269937 | 0.4518024 | 0.65141512 | 0.999976173 | 0.06898994 |
| YHL034C | SBP1 | 0.15269937 | 0.45180265 | 0.65141494 | 0.999976173 | 0.068989979 |
| YHR124W | NDT80 | 0.15269937 | 0.45235528 | 0.65101684 | 0.999976173 | 0.069074365 |
| YCL002C | YCL002C | 0.15269937 | 0.45276047 | 0.65072502 | 0.999976173 | 0.069136236 |
| YCL040W | GLK1 | 0.15269937 | 0.45331024 | 0.65032916 | 0.999976173 | 0.069220186 |
| YLR219W | MSC3 | 0.15269937 | 0.45354468 | 0.65016038 | 0.999976173 | 0.069255985 |
| YDR536W | STL1 | 0.15269937 | 0.45561227 | 0.64867264 | 0.999976173 | 0.069571705 |
| YDR192C | NUP42 | 0.15269937 | 0.45592141 | 0.64845031 | 0.999976173 | 0.069618911 |
| YMR065W | KAR5 | 0.15269937 | 0.45597538 | 0.64841151 | 0.999976173 | 0.069627151 |
| YMR238W | DFG5 | 0.15269937 | 0.45598468 | 0.64840481 | 0.999976173 | 0.069628572 |
| YDR008C | YDR008C | 0.15269937 | 0.45615498 | 0.64828236 | 0.999976173 | 0.069654576 |
| YCR006C | YCR006C | 0.15269937 | 0.45660914 | 0.64795584 | 0.999976173 | 0.069723926 |
| YPL180W | TCO89 | 0.15269937 | 0.45698519 | 0.64768552 | 0.999976173 | 0.069781349 |
| YDL012C | YDL012C | 0.15269937 | 0.45757607 | 0.64726087 | 0.999976173 | 0.069871576 |
| YDL144C | YDL144C | 0.15269937 | 0.45776111 | 0.64712791 | 0.999976173 | 0.069899832 |
| YOL053C-A | NA | 0.15269937 | 0.45801103 | 0.64694836 | 0.999976173 | 0.069937994 |
| YNL311C | YNL311C | 0.15269937 | 0.45820317 | 0.64681033 | 0.999976173 | 0.069967333 |
| YDR287W | INM2 | 0.15269937 | 0.45829398 | 0.64674509 | 0.999976173 | 0.069981201 |
| YOR359W | VTS1 | 0.15269937 | 0.45863979 | 0.64649671 | 0.999976173 | 0.070034004 |
| YHR210C | YHR210C | 0.15269937 | 0.45865929 | 0.6464827 | 0.999976173 | 0.070036983 |
| YHR136C | SPL2 | 0.15269937 | 0.45868629 | 0.64646331 | 0.999976173 | 0.070041106 |
| YDR425W | SNX41 | 0.15269937 | 0.45880809 | 0.64637583 | 0.999976173 | 0.070059704 |
| YML047C | PRM6 | 0.15269937 | 0.45888144 | 0.64632316 | 0.999976173 | 0.070070904 |

| | | | | | | |
|---|---|---|---|---|---|---|
| YLR207W | HRD3 | 0.15269937 | 0.45898591 | 0.64624814 | 0.999976173 | 0.070086857 |
| YJL027C | YJL027C | 0.15269937 | 0.45921848 | 0.64608113 | 0.999976173 | 0.070122371 |
| YHR132C | ECM14 | 0.15269937 | 0.45944109 | 0.6459213 | 0.999976173 | 0.070156364 |
| YHR050W | SMF2 | 0.15269937 | 0.46079438 | 0.64495 | 0.999976173 | 0.070363009 |
| YDR119W | VBA4 | 0.15269937 | 0.46136564 | 0.64454017 | 0.999976173 | 0.070450241 |
| YDR517W | GRH1 | 0.15269937 | 0.46150122 | 0.64444292 | 0.999976173 | 0.070470943 |
| YNR030W | ALG12 | 0.15269937 | 0.46206174 | 0.64404092 | 0.999976173 | 0.070556535 |
| YBR186W | PCH2 | 0.15269937 | 0.46235853 | 0.64382812 | 0.999976173 | 0.070601854 |
| YIL110W | MNI1 | 0.15269937 | 0.46278056 | 0.64352556 | 0.999976173 | 0.070666297 |
| YNR045W | PET494 | 0.15269937 | 0.4630981 | 0.64329794 | 0.999976173 | 0.070714786 |
| YCR102W-A | YCR102W-A | 0.15269937 | 0.46337867 | 0.64309686 | 0.999976173 | 0.070757629 |
| YKR105C | VBA5 | 0.15269937 | 0.4652459 | 0.64175929 | 0.999976173 | 0.071042753 |
| YIL140W | AXL2 | 0.15269937 | 0.46529806 | 0.64172193 | 0.999976173 | 0.071050719 |
| YHR130C | YHR130C | 0.15269937 | 0.46540237 | 0.64164725 | 0.999976173 | 0.071066647 |
| YML062C | MFT1 | 0.15269937 | 0.46562903 | 0.64148498 | 0.999976173 | 0.071101257 |
| YPR079W | MRL1 | 0.15269937 | 0.46589452 | 0.64129492 | 0.999976173 | 0.071141798 |
| YPR021C | AGC1 | 0.15269937 | 0.46664043 | 0.64076108 | 0.999976173 | 0.071255698 |
| YMR075C-A | YMR075C-A | 0.15269937 | 0.46742327 | 0.64020101 | 0.999976173 | 0.071375238 |
| YGR011W | YGR011W | 0.15269937 | 0.46903574 | 0.63904804 | 0.999976173 | 0.07162146 |
| YOR107W | RGS2 | 0.15269937 | 0.46904718 | 0.63903986 | 0.999976173 | 0.071623207 |
| YBL019W | APN2 | 0.15269937 | 0.46915907 | 0.63895989 | 0.999976173 | 0.071640292 |
| YBR063C | YBR063C | 0.15269937 | 0.46916344 | 0.63895677 | 0.999976173 | 0.071640959 |
| YDL161W | ENT1 | 0.15269937 | 0.46932646 | 0.63884026 | 0.999976173 | 0.071665852 |
| YIL116W | HIS5 | 0.15269937 | 0.46941148 | 0.6387795 | 0.999976173 | 0.071678835 |
| YDR326C | YSP2 | 0.15269937 | 0.46958915 | 0.63865253 | 0.999976173 | 0.071705965 |
| YML056C | IMD4 | 0.15269937 | 0.47011717 | 0.63827526 | 0.999976173 | 0.071786594 |
| YPR032W | SRO7 | 0.15269937 | 0.4701899 | 0.63822331 | 0.999976173 | 0.0717977 |
| YLR168C | UPS2 | 0.15269937 | 0.47248822 | 0.63658233 | 0.999976173 | 0.072148652 |
| YOR358W | HAP5 | 0.15269937 | 0.4730115 | 0.63620896 | 0.999976173 | 0.072228556 |
| YOL060C | MAM3 | 0.15269937 | 0.47374881 | 0.63568304 | 0.999976173 | 0.072341143 |
| YOL075C | YOL075C | 0.15269937 | 0.47428415 | 0.63530129 | 0.999976173 | 0.072422888 |
| YPR064W | YPR064W | 0.15269937 | 0.47533623 | 0.63455134 | 0.999976173 | 0.072583542 |
| YPL198W | RPL7B | 0.15269937 | 0.47544717 | 0.63447229 | 0.999976173 | 0.072600482 |
| YHL041W | YHL041W | 0.15269937 | 0.47598523 | 0.63408892 | 0.999976173 | 0.072682643 |
| YLL056C | YLL056C | 0.15269937 | 0.47650916 | 0.6337157 | 0.999976173 | 0.072762647 |
| YDR290W | YDR290W | 0.15269937 | 0.47721771 | 0.63321113 | 0.999976173 | 0.072870841 |
| YKL131W | YKL131W | 0.15269937 | 0.47767203 | 0.63288769 | 0.999976173 | 0.072940216 |
| YGL118C | YGL118C | 0.15269937 | 0.47820771 | 0.63250641 | 0.999976173 | 0.073022014 |
| YIL025C | YIL025C | 0.15269937 | 0.47864481 | 0.63219537 | 0.999976173 | 0.07308876 |
| YPR191W | QCR2 | 0.15269937 | 0.47897855 | 0.63195793 | 0.999976173 | 0.073139721 |
| YLR416C | YLR416C | 0.15269937 | 0.47902518 | 0.63192476 | 0.999976173 | 0.073146841 |
| YCL027W | FUS1 | 0.15269937 | 0.47916401 | 0.631826 | 0.999976173 | 0.07316804 |
| YDR010C | YDR010C | 0.15269937 | 0.47934066 | 0.63170035 | 0.999976173 | 0.073195015 |
| YDR349C | YPS7 | 0.15269937 | 0.47960816 | 0.63151009 | 0.999976173 | 0.073235861 |
| YLR299W | ECM38 | 0.15269937 | 0.48001332 | 0.63122197 | 0.999976173 | 0.07329773 |
| YDL046W | NPC2 | 0.15269937 | 0.48015163 | 0.63112364 | 0.999976173 | 0.073318849 |
| YGL104C | VPS73 | 0.15269937 | 0.48028151 | 0.63103129 | 0.999976173 | 0.073338683 |
| YDR210W | YDR210W | 0.15269937 | 0.4805529 | 0.63083836 | 0.999976173 | 0.073380122 |
| YML070W | DAK1 | 0.15269937 | 0.48062278 | 0.63078868 | 0.999976173 | 0.073390794 |
| YDR109C | YDR109C | 0.15269937 | 0.4813981 | 0.63023765 | 0.999976173 | 0.073509185 |
| YLR050C | YLR050C | 0.15269937 | 0.48147754 | 0.6301812 | 0.999976173 | 0.073521315 |
| YMR232W | FUS2 | 0.15269937 | 0.48229 | 0.62960402 | 0.999976173 | 0.073645377 |
| YPR029C | APL4 | 0.15269937 | 0.48263504 | 0.62935898 | 0.999976173 | 0.073698064 |
| YMR011W | HXT2 | 0.15269937 | 0.48272775 | 0.62929314 | 0.999976173 | 0.073712221 |
| YPL136W | YPL136W | 0.15269937 | 0.48293703 | 0.62914453 | 0.999976173 | 0.073744178 |
| YOR385W | YOR385W | 0.15269937 | 0.48361256 | 0.62866494 | 0.999976173 | 0.073847332 |
| YMR004W | MVP1 | 0.15269937 | 0.48369399 | 0.62860714 | 0.999976173 | 0.073859766 |
| YML048W-A | NA | 0.15269937 | 0.48405759 | 0.62834909 | 0.999976173 | 0.073915288 |
| YIR018W | YAP5 | 0.15269937 | 0.48440553 | 0.62810219 | 0.999976173 | 0.073968417 |
| YGL028C | SCW11 | 0.15269937 | 0.48462459 | 0.62794677 | 0.999976173 | 0.074001867 |
| YLR453C | 23 | 0.15269937 | 0.48498057 | 0.62769423 | 0.999976173 | 0.074056225 |
| YNR033W | ABZ1 | 0.15269937 | 0.48520239 | 0.62753689 | 0.999976173 | 0.074090097 |
| YNL031C | HHT2 | 0.15269937 | 0.48602389 | 0.62695434 | 0.999976173 | 0.074215539 |
| YNR019W | ARE2 | 0.15269937 | 0.48753377 | 0.62588424 | 0.999976173 | 0.074446097 |

| | | | | | | |
|---|---|---|---|---|---|---|
| YHR109W | CTM1 | 0.15269937 | 0.48792366 | 0.62560804 | 0.999976173 | 0.074505634 |
| YKL081W | TEF4 | 0.15269937 | 0.48848308 | 0.62521185 | 0.999976173 | 0.074591056 |
| YPR128C | ANT1 | 0.15269937 | 0.48930265 | 0.62463159 | 0.999976173 | 0.074716205 |
| YLR120C | YPS1 | 0.15269937 | 0.48945264 | 0.62452542 | 0.999976173 | 0.074739107 |
| YOR346W | REV1 | 0.15269937 | 0.48965854 | 0.62437969 | 0.999976173 | 0.074770548 |
| YML122C | YML122C | 0.15269937 | 0.49058301 | 0.62372556 | 0.999976173 | 0.074911714 |
| YDL082W | RPL13A | 0.15269937 | 0.49078841 | 0.62358026 | 0.999976173 | 0.074943079 |
| YBR188C | NTC20 | 0.15269937 | 0.49080829 | 0.6235662 | 0.999976173 | 0.074946115 |
| YDR496C | PUF6 | 0.15269937 | 0.49095209 | 0.6234645 | 0.999976173 | 0.074968072 |
| YOR296W | YOR296W | 0.15269937 | 0.49113912 | 0.62333222 | 0.999976173 | 0.074996632 |
| YOL008W | COQ10 | 0.15269937 | 0.49129809 | 0.62321979 | 0.999976173 | 0.075020906 |
| YJL043W | YJL043W | 0.15269937 | 0.49218184 | 0.62259497 | 0.999976173 | 0.075155855 |
| YBR233W | PBP2 | 0.15269937 | 0.49265275 | 0.62226214 | 0.999976173 | 0.075227763 |
| YMR199W | CLN1 | 0.15269937 | 0.49275093 | 0.62219276 | 0.999976173 | 0.075242755 |
| YBR287W | YBR287W | 0.15269937 | 0.49279639 | 0.62216064 | 0.999976173 | 0.075249697 |
| YLL063C | AYT1 | 0.15269937 | 0.49362028 | 0.62157856 | 0.999976173 | 0.075375504 |
| YLR335W | NUP2 | 0.15269937 | 0.4946763 | 0.62083283 | 0.999976173 | 0.075536757 |
| YAL053W | FLC2 | 0.15269937 | 0.49477734 | 0.62076149 | 0.999976173 | 0.075552187 |
| YPL068C | YPL068C | 0.15269937 | 0.49759843 | 0.61877133 | 0.999976173 | 0.075982965 |
| YPL182C | YPL182C | 0.15269937 | 0.49849367 | 0.61814036 | 0.999976173 | 0.076119667 |
| YLL025W | PAU17 | 0.15269937 | 0.49895472 | 0.61781552 | 0.999976173 | 0.07619007 |
| YFR018C | YFR018C | 0.15269937 | 0.50026587 | 0.61689213 | 0.999976173 | 0.076390281 |
| YOR377W | ATF1 | 0.15269937 | 0.50067983 | 0.61660072 | 0.999976173 | 0.076453493 |
| YMR058W | FET3 | 0.15269937 | 0.50073138 | 0.61656444 | 0.999976173 | 0.076461364 |
| YNL204C | SPS18 | 0.15269937 | 0.50095304 | 0.61640843 | 0.999976173 | 0.076495212 |
| YNL094W | APP1 | 0.15269937 | 0.50219592 | 0.61553398 | 0.999976173 | 0.076684999 |
| YBL057C | PTH2 | 0.15269937 | 0.5032976 | 0.61475933 | 0.999976173 | 0.076853225 |
| YML012W | ERV25 | 0.15269937 | 0.50372854 | 0.61445643 | 0.999976173 | 0.076919029 |
| YLR349W | YLR349W | 0.15269937 | 0.50464373 | 0.61381338 | 0.999976173 | 0.077058778 |
| YJL115W | ASF1 | 0.15269937 | 0.50577383 | 0.61301973 | 0.999976173 | 0.077231344 |
| YPL130W | SPO19 | 0.15269937 | 0.50614606 | 0.61275842 | 0.999976173 | 0.077288182 |
| YPL183W-A | RTC6 | 0.15269937 | 0.50658067 | 0.61245339 | 0.999976173 | 0.077354546 |
| YJL212C | OPT1 | 0.15269937 | 0.5072993 | 0.61194915 | 0.999976173 | 0.077464281 |
| YPL018W | CTF19 | 0.15269937 | 0.50814121 | 0.61135864 | 0.999976173 | 0.07759284 |
| YDR502C | SAM2 | 0.15269937 | 0.50824921 | 0.61128292 | 0.999976173 | 0.077609331 |
| YGR072W | 31 | 0.15269937 | 0.50831162 | 0.61123915 | 0.999976173 | 0.077618862 |
| YFL055W | AGP3 | 0.15269937 | 0.50883242 | 0.61087403 | 0.999976173 | 0.077698387 |
| YOR245C | DGA1 | 0.15269937 | 0.50980642 | 0.61019143 | 0.999976173 | 0.077847116 |
| YPL174C | NIP100 | 0.15269937 | 0.51014706 | 0.60995278 | 0.999976173 | 0.077899133 |
| YBR128C | ATG14 | 0.15269937 | 0.51026742 | 0.60986848 | 0.999976173 | 0.077917511 |
| YNL309W | STB1 | 0.15269937 | 0.51032726 | 0.60982656 | 0.999976173 | 0.077926649 |
| YNL333W | SNZ2 | 0.15269937 | 0.51034105 | 0.6098169 | 0.999976173 | 0.077928755 |
| YNL301C | RPL18B | 0.15269937 | 0.51070596 | 0.60956132 | 0.999976173 | 0.077984476 |
| YOR356W | YOR356W | 0.15269937 | 0.51086204 | 0.60945202 | 0.999976173 | 0.078008309 |
| YJL021C | NA | 0.15269937 | 0.51103534 | 0.60933067 | 0.999976173 | 0.078034772 |
| YJL134W | LCB3 | 0.15269937 | 0.51142942 | 0.60905476 | 0.999976173 | 0.078094947 |
| YHR110W | ERP5 | 0.15269937 | 0.51148477 | 0.60901601 | 0.999976173 | 0.078103401 |
| YLR367W | RPS22B | 0.15269937 | 0.51149467 | 0.60900908 | 0.999976173 | 0.078104911 |
| YHR075C | PPE1 | 0.15269937 | 0.51159633 | 0.60893792 | 0.999976173 | 0.078120435 |
| YEL017W | GTT3 | 0.15269937 | 0.51208942 | 0.6085928 | 0.999976173 | 0.078195729 |
| YIL114C | POR2 | 0.15269937 | 0.51233619 | 0.60842011 | 0.999976173 | 0.078233411 |
| YPL258C | THI21 | 0.15269937 | 0.5130074 | 0.60795052 | 0.999976173 | 0.078335905 |
| YAL049C | AIM2 | 0.15269937 | 0.51367167 | 0.60748594 | 0.999976173 | 0.078437339 |
| YGR086C | PIL1 | 0.15269937 | 0.51668526 | 0.6053803 | 0.999976173 | 0.078897511 |
| YBL083C | YBL083C | 0.15269937 | 0.51807872 | 0.60440777 | 0.999976173 | 0.079110292 |
| YPR174C | YPR174C | 0.15269937 | 0.51907068 | 0.60371589 | 0.999976173 | 0.079261763 |
| YBR053C | YBR053C | 0.15269937 | 0.51937898 | 0.60350092 | 0.999976173 | 0.079308841 |
| YIL038C | NOT3 | 0.15269937 | 0.51961443 | 0.60333678 | 0.999976173 | 0.079344793 |
| YOR363C | PIP2 | 0.15269937 | 0.5198856 | 0.60314776 | 0.999976173 | 0.079386201 |
| YOL083W | YOL083W | 0.15269937 | 0.52108378 | 0.60231286 | 0.999976173 | 0.079569163 |
| YFL003C | MSH4 | 0.15269937 | 0.52147129 | 0.60204296 | 0.999976173 | 0.079628335 |
| YLR023C | IZH3 | 0.15269937 | 0.52158658 | 0.60196267 | 0.999976173 | 0.07964594 |
| YBL101C | ECM21 | 0.15269937 | 0.52189876 | 0.60174528 | 0.999976173 | 0.07969361 |
| YPR077C | YPR077C | 0.15269937 | 0.52398228 | 0.60029535 | 0.999976173 | 0.080011761 |

| | | | | | | |
|---|---|---|---|---|---|---|
| YDR184C | ATC1 | 0.15269937 | 0.5243429 | 0.60004455 | 0.999976173 | 0.080066828 |
| YOL047C | YOL047C | 0.15269937 | 0.52452046 | 0.59992108 | 0.999976173 | 0.080093941 |
| YLR263W | RED1 | 0.15269937 | 0.52503138 | 0.59956587 | 0.999976173 | 0.080171959 |
| YER184C | YER184C | 0.15269937 | 0.5286514 | 0.59705183 | 0.999976173 | 0.080724733 |
| YPR146C | YPR146C | 0.15269937 | 0.52874001 | 0.59699035 | 0.999976173 | 0.080738264 |
| YKL208W | CBT1 | 0.15269937 | 0.52905965 | 0.5967686 | 0.999976173 | 0.080787074 |
| YOR051C | YOR051C | 0.15269937 | 0.52979498 | 0.59625862 | 0.999976173 | 0.080899358 |
| YDR466W | PKH3 | 0.15269937 | 0.52987 | 0.59620661 | 0.999976173 | 0.080910812 |
| YER170W | ADK2 | 0.15269937 | 0.53000621 | 0.59611217 | 0.999976173 | 0.080931612 |
| YCL028W | RNQ1 | 0.15269937 | 0.53021531 | 0.5959672 | 0.999976173 | 0.080963542 |
| YNR041C | COQ2 | 0.15269937 | 0.53135736 | 0.59517572 | 0.999976173 | 0.081137932 |
| YHR193C | EGD2 | 0.15269937 | 0.53154417 | 0.5950463 | 0.999976173 | 0.081166457 |
| YCR019W | MAK32 | 0.15269937 | 0.53345928 | 0.59372027 | 0.999976173 | 0.081458894 |
| YHR035W | YHR035W | 0.15269937 | 0.53387352 | 0.59343362 | 0.999976173 | 0.081522148 |
| YJL210W | PEX2 | 0.15269937 | 0.53446211 | 0.59302644 | 0.999976173 | 0.081612026 |
| YBR276C | PPS1 | 0.15269937 | 0.5346593 | 0.59289006 | 0.999976173 | 0.081642137 |
| YBL070C | YBL070C | 0.15269937 | 0.53779906 | 0.5907204 | 0.999976173 | 0.082121575 |
| YPL219W | PCL8 | 0.15269937 | 0.53815585 | 0.59047408 | 0.999976173 | 0.082176057 |
| YPR027C | YPR027C | 0.15269937 | 0.53896726 | 0.58991407 | 0.999976173 | 0.08229996 |
| YNL058C | YNL058C | 0.15269937 | 0.53921787 | 0.58974116 | 0.999976173 | 0.082338227 |
| YKL084W | HOT13 | 0.15269937 | 0.54014279 | 0.5891032 | 0.999976173 | 0.082479462 |
| YHR123W | EPT1 | 0.15269937 | 0.54023591 | 0.58903899 | 0.999976173 | 0.082493681 |
| YCL030C | HIS4 | 0.15269937 | 0.54082057 | 0.58863591 | 0.999976173 | 0.082582958 |
| YMR251W-A | HOR7 | 0.15269937 | 0.5432321 | 0.58697468 | 0.999976173 | 0.082951197 |
| YDR077W | SED1 | 0.15269937 | 0.54395631 | 0.58647622 | 0.999976173 | 0.083061783 |
| YDR094W | YDR094W | 0.15269937 | 0.54457899 | 0.5860478 | 0.999976173 | 0.083156866 |
| YAL061W | BDH2 | 0.15269937 | 0.54480976 | 0.58588905 | 0.999976173 | 0.083192105 |
| YMR107W | SPG4 | 0.15269937 | 0.54483694 | 0.58587036 | 0.999976173 | 0.083196255 |
| YLR181C | VTA1 | 0.15269937 | 0.54512861 | 0.58566976 | 0.999976173 | 0.083240793 |
| YAL045C | YAL045C | 0.15269937 | 0.54560452 | 0.58534252 | 0.999976173 | 0.083313464 |
| YIL167W | SDL1 | 0.15269937 | 0.54573861 | 0.58525033 | 0.999976173 | 0.08333394 |
| YIL020C | HIS6 | 0.15269937 | 0.54603642 | 0.58504561 | 0.999976173 | 0.083379415 |
| YLR247C | IRC20 | 0.15269937 | 0.54613839 | 0.58497551 | 0.999976173 | 0.083394986 |
| YMR312W | ELP6 | 0.15269937 | 0.5466274 | 0.58463945 | 0.999976173 | 0.083469657 |
| YJL004C | SYS1 | 0.15269937 | 0.54770936 | 0.5838962 | 0.999976173 | 0.083634872 |
| YKL096W | CWP1 | 0.15269937 | 0.54894973 | 0.58304468 | 0.999976173 | 0.083824275 |
| YMR057C | YMR057C | 0.15269937 | 0.54943933 | 0.58270873 | 0.999976173 | 0.083899037 |
| YCL006C | NA | 0.15269937 | 0.54965877 | 0.58255818 | 0.999976173 | 0.083932546 |
| YDR206W | 4 | 0.15269937 | 0.54978425 | 0.58247211 | 0.999976173 | 0.083951706 |
| YDL162C | YDL162C | 0.15269937 | 0.55064112 | 0.58188447 | 0.999976173 | 0.084082549 |
| YDL210W | UGA4 | 0.15269937 | 0.55126065 | 0.58145976 | 0.999976173 | 0.084177152 |
| YIR027C | DAL1 | 0.15269937 | 0.55180363 | 0.58108766 | 0.999976173 | 0.084260064 |
| YOR265W | RBL2 | 0.15269937 | 0.55196557 | 0.58097671 | 0.999976173 | 0.084284792 |
| YLR345W | YLR345W | 0.15269937 | 0.55273096 | 0.58045242 | 0.999976173 | 0.084401668 |
| YDL094C | YDL094C | 0.15269937 | 0.55353343 | 0.57990298 | 0.999976173 | 0.084524203 |
| YJR094W-A | RPL43B | 0.15269937 | 0.55354982 | 0.57989175 | 0.999976173 | 0.084526707 |
| YBR285W | YBR285W | 0.15269937 | 0.55364054 | 0.57982965 | 0.999976173 | 0.08454056 |
| YMR025W | CSI1 | 0.15269937 | 0.55371766 | 0.57977687 | 0.999976173 | 0.084552335 |
| YMR307W | GAS1 | 0.15269937 | 0.55384377 | 0.57969055 | 0.999976173 | 0.084571592 |
| YHR057C | CPR2 | 0.15269937 | 0.55410254 | 0.57951346 | 0.999976173 | 0.084611106 |
| YKR060W | UTP30 | 0.15269937 | 0.55447339 | 0.5792597 | 0.999976173 | 0.084667735 |
| YBR085W | AAC3 | 0.15269937 | 0.55510662 | 0.57882653 | 0.999976173 | 0.084764429 |
| YPL058C | PDR12 | 0.15269937 | 0.55537546 | 0.57864267 | 0.999976173 | 0.084805481 |
| YAL042W | ERV46 | 0.15269937 | 0.55580114 | 0.57835161 | 0.999976173 | 0.084870482 |
| YML059C | NTE1 | 0.15269937 | 0.55614919 | 0.57811367 | 0.999976173 | 0.084923629 |
| YBR073W | RDH54 | 0.15269937 | 0.55621083 | 0.57807154 | 0.999976173 | 0.084933042 |
| YMR044W | IOC4 | 0.15269937 | 0.55764714 | 0.57709018 | 0.999976173 | 0.085152365 |
| YCR049C | YCR049C | 0.15269937 | 0.55859862 | 0.57644051 | 0.999976173 | 0.085297655 |
| YFL034C-A | RPL22B | 0.15269937 | 0.55861244 | 0.57643108 | 0.999976173 | 0.085299766 |
| YCR023C | YCR023C | 0.15269937 | 0.55891628 | 0.57622369 | 0.999976173 | 0.085346161 |
| YIR036C | IRC24 | 0.15269937 | 0.55903124 | 0.57614524 | 0.999976173 | 0.085363715 |
| YBR024W | SCO2 | 0.15269937 | 0.55924396 | 0.57600007 | 0.999976173 | 0.085396198 |
| YBR092C | PHO3 | 0.15269937 | 0.56077995 | 0.57495241 | 0.999976173 | 0.085630742 |
| YOR214C | YOR214C | 0.15269937 | 0.56111915 | 0.57472117 | 0.999976173 | 0.085682539 |

| | | | | | | |
|---|---|---|---|---|---|---|
| YDL024C | DIA3 | 0.15269937 | 0.56112954 | 0.57471408 | 0.999976173 | 0.085684125 |
| YPR059C | YPR059C | 0.15269937 | 0.56211026 | 0.57404576 | 0.999976173 | 0.08583388 |
| YLR241W | YLR241W | 0.15269937 | 0.56236156 | 0.57387457 | 0.999976173 | 0.085872253 |
| YGR271W | SLH1 | 0.15269937 | 0.56299655 | 0.5734421 | 0.999976173 | 0.085969216 |
| YBR145W | ADH5 | 0.15269937 | 0.56673335 | 0.57090027 | 0.999976173 | 0.086539824 |
| YOR227W | HER1 | 0.15269937 | 0.56725942 | 0.57054286 | 0.999976173 | 0.086620153 |
| YLR384C | IKI3 | 0.15269937 | 0.5691078 | 0.56928792 | 0.999976173 | 0.0869024 |
| YBL091C | MAP2 | 0.15269937 | 0.56913385 | 0.56927024 | 0.999976173 | 0.086906378 |
| YPL095C | EEB1 | 0.15269937 | 0.56921436 | 0.56921561 | 0.999976173 | 0.086918672 |
| YKR014C | YPT52 | 0.15269937 | 0.57075942 | 0.56816769 | 0.999976173 | 0.087154601 |
| YPL121C | MEI5 | 0.15269937 | 0.5709509 | 0.56803788 | 0.999976173 | 0.08718384 |
| YPR090W | NA | 0.15269937 | 0.57135201 | 0.56776601 | 0.999976173 | 0.087245089 |
| YDR035W | ARO3 | 0.15269937 | 0.57149031 | 0.56767228 | 0.999976173 | 0.087266208 |
| YNL197C | WHI3 | 0.15269937 | 0.57267362 | 0.56687067 | 0.999976173 | 0.087446898 |
| YNR073C | YNR073C | 0.15269937 | 0.57303817 | 0.56662382 | 0.999976173 | 0.087502566 |
| YOR215C | AIM41 | 0.15269937 | 0.57319362 | 0.56651858 | 0.999976173 | 0.087526303 |
| YBL106C | SRO77 | 0.15269937 | 0.57458138 | 0.56557944 | 0.999976173 | 0.087738213 |
| YLR461W | PAU4 | 0.15269937 | 0.57463179 | 0.56554534 | 0.999976173 | 0.08774591 |
| YGR050C | YGR050C | 0.15269937 | 0.57464071 | 0.56553931 | 0.999976173 | 0.087747272 |
| YNL299W | TRF5 | 0.15269937 | 0.57513021 | 0.56520823 | 0.999976173 | 0.087822018 |
| YKL063C | YKL063C | 0.15269937 | 0.57573989 | 0.56479601 | 0.999976173 | 0.087915116 |
| YGR200C | ELP2 | 0.15269937 | 0.5760081 | 0.56461471 | 0.999976173 | 0.087956071 |
| YMR042W | ARG80 | 0.15269937 | 0.57619479 | 0.56448853 | 0.999976173 | 0.087984579 |
| YNL194C | YNL194C | 0.15269937 | 0.57718469 | 0.56381971 | 0.999976173 | 0.088135737 |
| YLR327C | TMA10 | 0.15269937 | 0.57762023 | 0.56352556 | 0.999976173 | 0.088202243 |
| YOR302W | YOR302W | 0.15269937 | 0.57773151 | 0.56345043 | 0.999976173 | 0.088219235 |
| YLR039C | RIC1 | 0.15269937 | 0.57794974 | 0.56330308 | 0.999976173 | 0.088252559 |
| YER185W | PUG1 | 0.15269937 | 0.57844678 | 0.56296754 | 0.999976173 | 0.088328457 |
| YIL056W | VHR1 | 0.15269937 | 0.57872458 | 0.56278006 | 0.999976173 | 0.088370877 |
| YDL036C | PUS9 | 0.15269937 | 0.57889393 | 0.56266578 | 0.999976173 | 0.088396736 |
| YPR199C | ARR1 | 0.15269937 | 0.57907247 | 0.56254531 | 0.999976173 | 0.088423999 |
| YPL199C | YPL199C | 0.15269937 | 0.57977536 | 0.56207115 | 0.999976173 | 0.088531331 |
| YDL122W | UBP1 | 0.15269937 | 0.58001205 | 0.56191154 | 0.999976173 | 0.088567472 |
| YBR139W | YBR139W | 0.15269937 | 0.58069356 | 0.56145205 | 0.999976173 | 0.088671539 |
| YDR286C | YDR286C | 0.15269937 | 0.58093171 | 0.56129153 | 0.999976173 | 0.088707904 |
| YHR157W | REC104 | 0.15269937 | 0.58170914 | 0.56076767 | 0.999976173 | 0.088826617 |
| YPR061C | JID1 | 0.15269937 | 0.58176987 | 0.56072677 | 0.999976173 | 0.08883589 |
| YHR028C | DAP2 | 0.15269937 | 0.58216945 | 0.56045762 | 0.999976173 | 0.088896905 |
| YJR152W | DAL5 | 0.15269937 | 0.58274966 | 0.56006691 | 0.999976173 | 0.088985504 |
| YOR321W | PMT3 | 0.15269937 | 0.58315609 | 0.55979331 | 0.999976173 | 0.089047564 |
| YHR176W | FMO1 | 0.15269937 | 0.58418128 | 0.55910344 | 0.999976173 | 0.089204111 |
| YBR244W | GPX2 | 0.15269937 | 0.58424469 | 0.55906078 | 0.999976173 | 0.089213794 |
| YPL034W | YPL034W | 0.15269937 | 0.5843549 | 0.55898665 | 0.999976173 | 0.089230622 |
| YJR034W | PET191 | 0.15269937 | 0.58436631 | 0.55897898 | 0.999976173 | 0.089232365 |
| YGR174C | CBP4 | 0.15269937 | 0.58563116 | 0.5581285 | 0.999976173 | 0.089425507 |
| YMR055C | BUB2 | 0.15269937 | 0.58633546 | 0.55765522 | 0.999976173 | 0.089533053 |
| YGR123C | PPT1 | 0.15269937 | 0.58676797 | 0.55736466 | 0.999976173 | 0.089599097 |
| YIL136W | OM45 | 0.15269937 | 0.58741769 | 0.55692834 | 0.999976173 | 0.089698308 |
| YPL135W | ISU1 | 0.15269937 | 0.5877348 | 0.55671544 | 0.999976173 | 0.089746731 |
| YJL185C | YJL185C | 0.15269937 | 0.5878237 | 0.55665576 | 0.999976173 | 0.089760306 |
| YGR100W | MDR1 | 0.15269937 | 0.58841478 | 0.55625905 | 0.999976173 | 0.089850564 |
| YLL047W | YLL047W | 0.15269937 | 0.58884461 | 0.55597065 | 0.999976173 | 0.089916199 |
| YGR053C | YGR053C | 0.15269937 | 0.59069253 | 0.55473161 | 0.999976173 | 0.090198375 |
| YDR346C | SVF1 | 0.15269937 | 0.59071677 | 0.55471536 | 0.999976173 | 0.090202076 |
| YIR001C | SGN1 | 0.15269937 | 0.59148078 | 0.55420349 | 0.999976173 | 0.09031874 |
| YOL153C | YOL153C | 0.15269937 | 0.59170394 | 0.55405402 | 0.999976173 | 0.090352816 |
| YER161C | SPT2 | 0.15269937 | 0.59210012 | 0.55378871 | 0.999976173 | 0.090413313 |
| YER153C | PET122 | 0.15269937 | 0.59283188 | 0.55329885 | 0.999976173 | 0.090525052 |
| YDR505C | PSP1 | 0.15269937 | 0.59291484 | 0.55324332 | 0.999976173 | 0.090537721 |
| YKL128C | PMU1 | 0.15269937 | 0.59298566 | 0.55319593 | 0.999976173 | 0.090548534 |
| YER071C | YER071C | 0.15269937 | 0.59328732 | 0.55299406 | 0.999976173 | 0.090594597 |
| YLR205C | HMX1 | 0.15269937 | 0.59487095 | 0.55193494 | 0.999976173 | 0.090836416 |
| YHR037W | PUT2 | 0.15269937 | 0.59573779 | 0.55135561 | 0.999976173 | 0.090968782 |
| YLR423C | ATG17 | 0.15269937 | 0.59618694 | 0.55105556 | 0.999976173 | 0.091037367 |

| | | | | | | |
|---|---|---|---|---|---|---|
| YPL062W | YPL062W | 0.15269937 | 0.59652949 | 0.55082677 | 0.999976173 | 0.091089674 |
| YGR040W | KSS1 | 0.15269937 | 0.59683106 | 0.5506254 | 0.999976173 | 0.091135724 |
| YJL100W | LSB6 | 0.15269937 | 0.59811928 | 0.54976557 | 0.999976173 | 0.091332435 |
| YDR309C | GIC2 | 0.15269937 | 0.59864495 | 0.54941491 | 0.999976173 | 0.091412705 |
| YER115C | SPR6 | 0.15269937 | 0.59888158 | 0.54925709 | 0.999976173 | 0.091448838 |
| YDR042C | YDR042C | 0.15269937 | 0.59931474 | 0.54896826 | 0.999976173 | 0.09151498 |
| YNL322C | KRE1 | 0.15269937 | 0.59936452 | 0.54893508 | 0.999976173 | 0.091522582 |
| YPL257W | YPL257W | 0.15269937 | 0.59961561 | 0.54876769 | 0.999976173 | 0.091560923 |
| YJL051W | IRC8 | 0.15269937 | 0.60096729 | 0.54786703 | 0.999976173 | 0.091767324 |
| YMR102C | YMR102C | 0.15269937 | 0.60105072 | 0.54781147 | 0.999976173 | 0.091780063 |
| YNL286W | CUS2 | 0.15269937 | 0.60180614 | 0.54730846 | 0.999976173 | 0.091895416 |
| YJR009C | TDH2 | 0.15269937 | 0.60375966 | 0.54600873 | 0.999976173 | 0.092193717 |
| YOR384W | FRE5 | 0.15269937 | 0.60399709 | 0.54585087 | 0.999976173 | 0.092229972 |
| YDR105C | TMS1 | 0.15269937 | 0.60453012 | 0.54549654 | 0.999976173 | 0.092311366 |
| YJL149W | YJL149W | 0.15269937 | 0.60485563 | 0.54528023 | 0.999976173 | 0.092361071 |
| YLR242C | ARV1 | 0.15269937 | 0.6055253 | 0.54483533 | 0.999976173 | 0.092463329 |
| YKL179C | COY1 | 0.15269937 | 0.60757988 | 0.54347147 | 0.999976173 | 0.092777063 |
| YDL179W | PCL9 | 0.15269937 | 0.6090528 | 0.54249478 | 0.999976173 | 0.093001976 |
| YBR010W | HHT1 | 0.15269937 | 0.60916646 | 0.54241945 | 0.999976173 | 0.093019332 |
| YBR300C | YBR300C | 0.15269937 | 0.61118263 | 0.54108404 | 0.999976173 | 0.0933272 |
| YAL007C | ERP2 | 0.15269937 | 0.61125239 | 0.54103787 | 0.999976173 | 0.093337852 |
| YBR273C | UBX7 | 0.15269937 | 0.61430176 | 0.53902134 | 0.999976173 | 0.093803489 |
| YGR035C | YGR035C | 0.15269937 | 0.61505686 | 0.53852258 | 0.999976173 | 0.093918792 |
| YPL070W | MUK1 | 0.15269937 | 0.61533869 | 0.53833648 | 0.999976173 | 0.093961827 |
| YDL146W | LDB17 | 0.15269937 | 0.61562162 | 0.53814969 | 0.999976173 | 0.094005031 |
| YML123C | PHO84 | 0.15269937 | 0.6158879 | 0.53797392 | 0.999976173 | 0.094045692 |
| YLR262C-A | TMA7 | 0.15269937 | 0.61682981 | 0.53735241 | 0.999976173 | 0.094189521 |
| YKL202W | YKL202W | 0.15269937 | 0.61731478 | 0.53703255 | 0.999976173 | 0.094263575 |
| YBR203W | COS111 | 0.15269937 | 0.61824375 | 0.53642011 | 0.999976173 | 0.094405428 |
| YOR028C | CIN5 | 0.15269937 | 0.61880501 | 0.53605026 | 0.999976173 | 0.094491132 |
| YLR341W | SPO77 | 0.15269937 | 0.61893684 | 0.53596341 | 0.999976173 | 0.094511263 |
| YMR100W | MUB1 | 0.15269937 | 0.61954237 | 0.53556457 | 0.999976173 | 0.094603726 |
| YBR274W | 2 | 0.15269937 | 0.61977964 | 0.53540833 | 0.999976173 | 0.094639958 |
| YJL138C | TIF2 | 0.15269937 | 0.62131139 | 0.53440023 | 0.999976173 | 0.094873855 |
| YNR034W | SOL1 | 0.15269937 | 0.62144592 | 0.53431174 | 0.999976173 | 0.094894398 |
| YOR173W | DCS2 | 0.15269937 | 0.62284653 | 0.53339086 | 0.999976173 | 0.09510827 |
| YLL061W | MMP1 | 0.15269937 | 0.62321246 | 0.5331504 | 0.999976173 | 0.095164147 |
| YOR105W | YOR105W | 0.15269937 | 0.62325373 | 0.53312329 | 0.999976173 | 0.095170449 |
| YPL185W | YPL185W | 0.15269937 | 0.62386671 | 0.53272062 | 0.999976173 | 0.09526405 |
| YNR044W | AGA1 | 0.15269937 | 0.62405549 | 0.53259664 | 0.999976173 | 0.095292877 |
| YNL081C | SWS2 | 0.15269937 | 0.62467057 | 0.5321928 | 0.999976173 | 0.095386799 |
| YDR020C | DAS2 | 0.15269937 | 0.62469787 | 0.53217487 | 0.999976173 | 0.095390969 |
| YMR264W | CUE1 | 0.15269937 | 0.62514497 | 0.53188142 | 0.999976173 | 0.095459241 |
| YKL107W | YKL107W | 0.15269937 | 0.62540674 | 0.53170965 | 0.999976173 | 0.095499212 |
| YJL071W | ARG2 | 0.15269937 | 0.62576574 | 0.53147412 | 0.999976173 | 0.095554031 |
| YPL003W | ULA1 | 0.15269937 | 0.62592499 | 0.53136966 | 0.999976173 | 0.095578349 |
| YPL116W | HOS3 | 0.15269937 | 0.62768909 | 0.53021317 | 0.999976173 | 0.095847726 |
| YAR014C | BUD14 | 0.15269937 | 0.62832816 | 0.52979452 | 0.999976173 | 0.095945312 |
| YBL025W | RRN10 | 0.15269937 | 0.62976343 | 0.52885493 | 0.999976173 | 0.096164477 |
| YPL051W | ARL3 | 0.15269937 | 0.63062715 | 0.5282899 | 0.999976173 | 0.096296366 |
| YIL007C | NAS2 | 0.15269937 | 0.63063212 | 0.52828666 | 0.999976173 | 0.096297124 |
| YJL098W | SAP185 | 0.15269937 | 0.63102056 | 0.52803265 | 0.999976173 | 0.096356439 |
| YBR083W | TEC1 | 0.15269937 | 0.6312721 | 0.52786819 | 0.999976173 | 0.09639485 |
| YNL156C | NSG2 | 0.15269937 | 0.6314319 | 0.52776374 | 0.999976173 | 0.09641925 |
| YNR055C | HOL1 | 0.15269937 | 0.63245374 | 0.52709601 | 0.999976173 | 0.096575284 |
| YOR352W | YOR352W | 0.15269937 | 0.63322719 | 0.52659088 | 0.999976173 | 0.09669339 |
| YPR084W | YPR084W | 0.15269937 | 0.63345211 | 0.52644404 | 0.999976173 | 0.096727735 |
| YER038W-A | YER038W-A | 0.15269937 | 0.63347181 | 0.52643118 | 0.999976173 | 0.096730743 |
| YKL217W | JEN1 | 0.15269937 | 0.63468631 | 0.52563863 | 0.999976173 | 0.096916198 |
| YOL113W | SKM1 | 0.15269937 | 0.63474138 | 0.52560271 | 0.999976173 | 0.096924606 |
| YJR053W | BFA1 | 0.15269937 | 0.63504068 | 0.52540749 | 0.999976173 | 0.09697031 |
| YFL044C | OTU1 | 0.15269937 | 0.63562966 | 0.52502345 | 0.999976173 | 0.097060246 |
| YBR033W | EDS1 | 0.15269937 | 0.63803789 | 0.52345465 | 0.999976173 | 0.097427981 |
| YNL014W | HEF3 | 0.15269937 | 0.63889771 | 0.52289512 | 0.999976173 | 0.097559276 |

| | | | | | | |
|---|---|---|---|---|---|---|
| YER152C | YER152C | 0.15269937 | 0.63977262 | 0.5223261 | 0.999976173 | 0.097692873 |
| YEL023C | YEL023C | 0.15269937 | 0.64199838 | 0.52087991 | 0.999976173 | 0.098032746 |
| YPL074W | YTA6 | 0.15269937 | 0.64206184 | 0.52083871 | 0.999976173 | 0.098042436 |
| YOR208W | PTP2 | 0.15269937 | 0.64235635 | 0.52064752 | 0.999976173 | 0.098087407 |
| YJL196C | ELO1 | 0.15269937 | 0.64264571 | 0.5204597 | 0.999976173 | 0.098131592 |
| YBR122C | MRPL36 | 0.15269937 | 0.64361211 | 0.51983269 | 0.999976173 | 0.098279161 |
| YOR239W | ABP140 | 0.15269937 | 0.6438446 | 0.51968191 | 0.999976173 | 0.098314662 |
| YLR123C | YLR123C | 0.15269937 | 0.64385759 | 0.51967348 | 0.999976173 | 0.098316646 |
| YJL007C | YJL007C | 0.15269937 | 0.64401441 | 0.51957179 | 0.999976173 | 0.098340591 |
| YBL046W | PSY4 | 0.15269937 | 0.64460293 | 0.51919024 | 0.999976173 | 0.098430459 |
| YPR198W | SGE1 | 0.15269937 | 0.64466242 | 0.51915168 | 0.999976173 | 0.098439543 |
| YNL041C | COG6 | 0.15269937 | 0.64584065 | 0.51838828 | 0.999976173 | 0.098619457 |
| YKL117W | SBA1 | 0.15269937 | 0.64788976 | 0.51706199 | 0.999976173 | 0.098932356 |
| YDL066W | IDP1 | 0.15269937 | 0.64794217 | 0.51702809 | 0.999976173 | 0.098940359 |
| YDR244W | PEX5 | 0.15269937 | 0.64847078 | 0.51668625 | 0.999976173 | 0.099021077 |
| YIL132C | CSM2 | 0.15269937 | 0.64849426 | 0.51667107 | 0.999976173 | 0.099024662 |
| YLR130C | ZRT2 | 0.15269937 | 0.64858769 | 0.51661066 | 0.999976173 | 0.099038929 |
| YNL053W | MSG5 | 0.15269937 | 0.64892084 | 0.5163953 | 0.999976173 | 0.099089801 |
| YDR358W | GGA1 | 0.15269937 | 0.64895902 | 0.51637062 | 0.999976173 | 0.09909563 |
| YDL085W | NDE2 | 0.15269937 | 0.64958976 | 0.51596301 | 0.999976173 | 0.099191944 |
| YGL139W | FLC3 | 0.15269937 | 0.64993426 | 0.51574045 | 0.999976173 | 0.099244549 |
| YKL071W | YKL071W | 0.15269937 | 0.6503376 | 0.51547994 | 0.999976173 | 0.099306139 |
| YBL061C | SKT5 | 0.15269937 | 0.65041548 | 0.51542965 | 0.999976173 | 0.099318031 |
| YKR093W | PTR2 | 0.15269937 | 0.65113983 | 0.514962 | 0.999976173 | 0.099428639 |
| YML006C | GIS4 | 0.15269937 | 0.65153048 | 0.51470989 | 0.999976173 | 0.099488291 |
| YDR414C | ERD1 | 0.15269937 | 0.65179158 | 0.51454142 | 0.999976173 | 0.099528161 |
| YPL071C | YPL071C | 0.15269937 | 0.65202675 | 0.5143897 | 0.999976173 | 0.099564072 |
| YHL037C | YHL037C | 0.15269937 | 0.65204371 | 0.51437876 | 0.999976173 | 0.099566661 |
| YOR301W | RAX1 | 0.15269937 | 0.65248612 | 0.51409342 | 0.999976173 | 0.099634216 |
| YKL215C | YKL215C | 0.15269937 | 0.6529303 | 0.51380701 | 0.999976173 | 0.099702043 |
| YDL070W | BDF2 | 0.15269937 | 0.6532864 | 0.51357746 | 0.999976173 | 0.099756419 |
| YPR155C | NCA2 | 0.15269937 | 0.6548219 | 0.51258824 | 0.999976173 | 0.09999089 |
| YPL047W | SGF11 | 0.15269937 | 0.65712685 | 0.51110519 | 0.999976173 | 0.100342854 |
| YFR030W | MET10 | 0.15269937 | 0.65723765 | 0.51103396 | 0.999976173 | 0.100359772 |
| YLR325C | RPL38 | 0.15269937 | 0.65789297 | 0.51061276 | 0.999976173 | 0.100459839 |
| YOR156C | NFI1 | 0.15269937 | 0.65945294 | 0.50961082 | 0.999976173 | 0.100698046 |
| YPR147C | YPR147C | 0.15269937 | 0.65946084 | 0.50960575 | 0.999976173 | 0.100699251 |
| YPR188C | MLC2 | 0.15269937 | 0.65986982 | 0.50934324 | 0.999976173 | 0.100761702 |
| YNL219C | ALG9 | 0.15269937 | 0.66031077 | 0.50906029 | 0.999976173 | 0.100829036 |
| YLR251W | SYM1 | 0.15269937 | 0.66042128 | 0.50898939 | 0.999976173 | 0.10084591 |
| YCL076W | YCL076W | 0.15269937 | 0.6604746 | 0.50895518 | 0.999976173 | 0.100854053 |
| YGL221C | NIF3 | 0.15269937 | 0.66175428 | 0.50813459 | 0.999976173 | 0.101049459 |
| YNL281W | HCH1 | 0.15269937 | 0.66196537 | 0.5079993 | 0.999976173 | 0.101081693 |
| YPR118W | MRI1 | 0.15269937 | 0.66219137 | 0.50785447 | 0.999976173 | 0.101116202 |
| YOR255W | OSW1 | 0.15269937 | 0.66412006 | 0.50661938 | 0.999976173 | 0.101410712 |
| YJL103C | GSM1 | 0.15269937 | 0.66438049 | 0.50645273 | 0.999976173 | 0.10145048 |
| YNL100W | AIM37 | 0.15269937 | 0.66499515 | 0.50605951 | 0.999976173 | 0.101544338 |
| YIR009W | MSL1 | 0.15269937 | 0.66503685 | 0.50603285 | 0.999976173 | 0.101550705 |
| YBL087C | RPL23A | 0.15269937 | 0.6651119 | 0.50598484 | 0.999976173 | 0.101562166 |
| YKL079W | SMY1 | 0.15269937 | 0.66589149 | 0.50548639 | 0.999976173 | 0.101681209 |
| YPR004C | AIM45 | 0.15269937 | 0.66628402 | 0.50523551 | 0.999976173 | 0.101741147 |
| YGL237C | HAP2 | 0.15269937 | 0.66650881 | 0.50509187 | 0.999976173 | 0.101775472 |
| YMR027W | YMR027W | 0.15269937 | 0.66756613 | 0.50441653 | 0.999976173 | 0.101936925 |
| YDL223C | HBT1 | 0.15269937 | 0.66966589 | 0.50307677 | 0.999976173 | 0.102257556 |
| YDR380W | ARO10 | 0.15269937 | 0.66971703 | 0.50304416 | 0.999976173 | 0.102265366 |
| YLR164W | YLR164W | 0.15269937 | 0.67228589 | 0.50140771 | 0.999976173 | 0.102657628 |
| YDL204W | RTN2 | 0.15269937 | 0.67245523 | 0.50129993 | 0.999976173 | 0.102683487 |
| YBR111C | YSA1 | 0.15269937 | 0.67455418 | 0.49996507 | 0.999976173 | 0.103003995 |
| YBR171W | SEC66 | 0.15269937 | 0.67679753 | 0.49854047 | 0.999976173 | 0.103346554 |
| YDR335W | MSN5 | 0.15269937 | 0.67735895 | 0.49818429 | 0.999976173 | 0.103432281 |
| YPL191C | YPL191C | 0.15269937 | 0.67775955 | 0.49793022 | 0.999976173 | 0.103493453 |
| YDR098C | GRX3 | 0.15269937 | 0.67797233 | 0.49779529 | 0.999976173 | 0.103525945 |
| YEL042W | GDA1 | 0.15269937 | 0.67870778 | 0.4973291 | 0.999976173 | 0.103638247 |
| YHL023C | NPR3 | 0.15269937 | 0.67932559 | 0.49693765 | 0.999976173 | 0.103732587 |

| | | | | | | |
|---|---|---|---|---|---|---|
| YDR143C | SAN1 | 0.15269937 | 0.67985748 | 0.49660078 | 0.999976173 | 0.103813805 |
| YAR031W | PRM9 | 0.15269937 | 0.68057488 | 0.49614661 | 0.999976173 | 0.103923352 |
| YJR139C | HOM6 | 0.15269937 | 0.68103008 | 0.49585854 | 0.999976173 | 0.103992862 |
| YDL026W | YDL026W | 0.15269937 | 0.68133233 | 0.49566732 | 0.999976173 | 0.104039014 |
| YNL336W | COS1 | 0.15269937 | 0.68134143 | 0.49566156 | 0.999976173 | 0.104040405 |
| YOR131C | YOR131C | 0.15269937 | 0.68332332 | 0.49440867 | 0.999976173 | 0.104343038 |
| YDR455C | YDR455C | 0.15269937 | 0.68361298 | 0.4942257 | 0.999976173 | 0.104387269 |
| YCL061C | MRC1 | 0.15269937 | 0.68498374 | 0.49336031 | 0.999976173 | 0.104596583 |
| YKL087C | CYT2 | 0.15269937 | 0.6860597 | 0.4926816 | 0.999976173 | 0.104760881 |
| YOR125C | CAT5 | 0.15269937 | 0.68651864 | 0.49239226 | 0.999976173 | 0.104830961 |
| YKR101W | SIR1 | 0.15269937 | 0.68662779 | 0.49232346 | 0.999976173 | 0.104847628 |
| YDL131W | LYS21 | 0.15269937 | 0.68720546 | 0.49195942 | 0.999976173 | 0.104935838 |
| YDR465C | RMT2 | 0.15269937 | 0.68910265 | 0.49076484 | 0.999976173 | 0.105225538 |
| YGL211W | NCS6 | 0.15269937 | 0.69007896 | 0.49015071 | 0.999976173 | 0.10537462 |
| YJL082W | IML2 | 0.15269937 | 0.69138406 | 0.48933042 | 0.999976173 | 0.105573907 |
| YJL192C | SOP4 | 0.15269937 | 0.69223353 | 0.48879689 | 0.999976173 | 0.105703621 |
| YOR350C | MNE1 | 0.15269937 | 0.69224568 | 0.48878926 | 0.999976173 | 0.105705476 |
| YAL058C-A | NA | 0.15269937 | 0.69229273 | 0.48875972 | 0.999976173 | 0.105712661 |
| YLR456W | YLR456W | 0.15269937 | 0.69234153 | 0.48872908 | 0.999976173 | 0.105720113 |
| YBR178W | YBR178W | 0.15269937 | 0.69256106 | 0.48859126 | 0.999976173 | 0.105753635 |
| YCR083W | TRX3 | 0.15269937 | 0.693804 | 0.48781136 | 0.999976173 | 0.10594343 |
| YOR189W | IES4 | 0.15269937 | 0.69408515 | 0.48763503 | 0.999976173 | 0.105986363 |
| YGR067C | YGR067C | 0.15269937 | 0.69430124 | 0.48749954 | 0.999976173 | 0.10601936 |
| YPL048W | CAM1 | 0.15269937 | 0.69451155 | 0.48736769 | 0.999976173 | 0.106051474 |
| YHR005C | GPA1 | 0.15269937 | 0.696093 | 0.48637683 | 0.999976173 | 0.10629296 |
| YNL076W | MKS1 | 0.15269937 | 0.69619009 | 0.48631604 | 0.999976173 | 0.106307786 |
| YBR020W | GAL1 | 0.10797476 | 0.98563664 | 0.32432066 | 0.999976173 | 0.106423876 |
| YKL198C | PTK1 | 0.15269937 | 0.69787607 | 0.48526096 | 0.999976173 | 0.106565234 |
| YDR124W | YDR124W | 0.15269937 | 0.69808778 | 0.48512857 | 0.999976173 | 0.106597561 |
| YLR258W | GSY2 | 0.15269937 | 0.69818452 | 0.48506808 | 0.999976173 | 0.106612333 |
| YDR363W-A | SEM1 | 0.15269937 | 0.69864928 | 0.48477751 | 0.999976173 | 0.106683302 |
| YDR142C | PEX7 | 0.15269937 | 0.69971932 | 0.48410889 | 0.999976173 | 0.106846696 |
| YLR213C | CRR1 | 0.15269937 | 0.70061507 | 0.48354956 | 0.999976173 | 0.106983477 |
| YDR085C | AFR1 | 0.15269937 | 0.70125463 | 0.48315042 | 0.999976173 | 0.107081137 |
| YLR183C | TOS4 | 0.15269937 | 0.70205969 | 0.48264825 | 0.999976173 | 0.10720407 |
| YGL162W | SUT1 | 0.15269937 | 0.70209259 | 0.48262773 | 0.999976173 | 0.107209093 |
| YDL113C | ATG20 | 0.15269937 | 0.70252421 | 0.48235862 | 0.999976173 | 0.107275002 |
| YML030W | AIM31 | 0.15269937 | 0.7029299 | 0.48210575 | 0.999976173 | 0.10733695 |
| YIL005W | EPS1 | 0.15269937 | 0.70309866 | 0.48200058 | 0.999976173 | 0.10736272 |
| YBL065W | YBL065W | 0.15269937 | 0.70471529 | 0.48099377 | 0.999976173 | 0.107609579 |
| YDR340W | YDR340W | 0.15269937 | 0.70499776 | 0.48081797 | 0.999976173 | 0.107652711 |
| YCL037C | SRO9 | 0.15269937 | 0.70529228 | 0.48063471 | 0.999976173 | 0.107697683 |
| YLR300W | EXG1 | 0.15269937 | 0.70785173 | 0.4790437 | 0.999976173 | 0.108088511 |
| YIL088C | AVT7 | 0.15269937 | 0.70822991 | 0.47880887 | 0.999976173 | 0.108146258 |
| YFR019W | FAB1 | 0.15269937 | 0.70921587 | 0.47819691 | 0.999976173 | 0.108296814 |
| YFR020W | YFR020W | 0.15269937 | 0.71017765 | 0.47760037 | 0.999976173 | 0.108443677 |
| YNL295W | YNL295W | 0.15269937 | 0.71043532 | 0.47744062 | 0.999976173 | 0.108483023 |
| YPL015C | HST2 | 0.15269937 | 0.71302684 | 0.47583557 | 0.999976173 | 0.108878746 |
| YJL079C | PRY1 | 0.15269937 | 0.71494918 | 0.47464689 | 0.999976173 | 0.109172287 |
| YLR118C | YLR118C | 0.15269937 | 0.7151224 | 0.47453986 | 0.999976173 | 0.109198737 |
| YMR153W | NUP53 | 0.15269937 | 0.71516332 | 0.47451458 | 0.999976173 | 0.109204985 |
| YNL082W | PMS1 | 0.15269937 | 0.71529995 | 0.47443016 | 0.999976173 | 0.109225849 |
| YER176W | ECM32 | 0.15269937 | 0.71577985 | 0.47413375 | 0.999976173 | 0.109299129 |
| YDL203C | ACK1 | 0.15269937 | 0.7177857 | 0.47289591 | 0.999976173 | 0.109605421 |
| YIR029W | DAL2 | 0.15269937 | 0.71779382 | 0.4728909 | 0.999976173 | 0.109606661 |
| YKR053C | YSR3 | 0.15269937 | 0.718445 | 0.47248943 | 0.999976173 | 0.109706096 |
| YHR126C | ANS1 | 0.15269937 | 0.71865034 | 0.47236287 | 0.999976173 | 0.109737451 |
| YHL008C | YHL008C | 0.15269937 | 0.71869385 | 0.47233606 | 0.999976173 | 0.109744095 |
| YPR148C | YPR148C | 0.15269937 | 0.71946693 | 0.47185976 | 0.999976173 | 0.109862145 |
| YNL034W | YNL034W | 0.15269937 | 0.72092843 | 0.47096006 | 0.999976173 | 0.110085314 |
| YGR248W | SOL4 | 0.15269937 | 0.72148821 | 0.4706157 | 0.999976173 | 0.110170792 |
| YJL137C | GLG2 | 0.15269937 | 0.72178186 | 0.47043512 | 0.999976173 | 0.110215632 |
| YBR129C | OPY1 | 0.15269937 | 0.72210699 | 0.47023522 | 0.999976173 | 0.110265279 |
| YNR069C | BSC5 | 0.15269937 | 0.72255946 | 0.46995711 | 0.999976173 | 0.110334371 |

| | | | | | | |
|---|---|---|---|---|---|---|
| YAL046C | AIM1 | 0.15269937 | 0.72347209 | 0.46939643 | 0.999976173 | 0.110473729 |
| YOR333C | YOR333C | 0.15269937 | 0.72385845 | 0.46915917 | 0.999976173 | 0.110532727 |
| YNL231C | PDR16 | 0.15269937 | 0.72491905 | 0.46850824 | 0.999976173 | 0.110694679 |
| YLR058C | SHM2 | 0.15269937 | 0.7249908 | 0.46846422 | 0.999976173 | 0.110705635 |
| YIL006W | YIA6 | 0.15269937 | 0.72500296 | 0.46845676 | 0.999976173 | 0.110707492 |
| YPR200C | ARR2 | 0.15269937 | 0.72528848 | 0.46828162 | 0.999976173 | 0.110751091 |
| YLR303W | MET17 | 0.15269937 | 0.72555937 | 0.46811549 | 0.999976173 | 0.110792455 |
| YGR028W | MSP1 | 0.15269937 | 0.72687147 | 0.46731126 | 0.999976173 | 0.110992813 |
| YLR294C | YLR294C | 0.15269937 | 0.72745039 | 0.46695667 | 0.999976173 | 0.111081214 |
| YGR250C | YGR250C | 0.15269937 | 0.72789338 | 0.46668543 | 0.999976173 | 0.111148858 |
| YDL155W | CLB3 | 0.15269937 | 0.72829081 | 0.46644217 | 0.999976173 | 0.111209545 |
| YPL035C | YPL035C | 0.15269937 | 0.72853595 | 0.46629215 | 0.999976173 | 0.111246978 |
| YOR138C | RUP1 | 0.15269937 | 0.73041006 | 0.46514617 | 0.999976173 | 0.111533154 |
| YOR382W | FIT2 | 0.15269937 | 0.73071991 | 0.46495686 | 0.999976173 | 0.111580467 |
| YLR092W | SUL2 | 0.15269937 | 0.73657174 | 0.4613895 | 0.999976173 | 0.112474038 |
| YOR266W | PNT1 | 0.15269937 | 0.73740463 | 0.460883 | 0.999976173 | 0.112601219 |
| YKR065C | PAM17 | 0.15269937 | 0.73823923 | 0.46037577 | 0.999976173 | 0.112728663 |
| YMR221C | YMR221C | 0.15269937 | 0.73836236 | 0.46030097 | 0.999976173 | 0.112747465 |
| YDR444W | YDR444W | 0.15269937 | 0.73880629 | 0.46003132 | 0.999976173 | 0.112815252 |
| YNL079C | TPM1 | 0.15269937 | 0.7401419 | 0.4592206 | 0.999976173 | 0.113019199 |
| YLR429W | CRN1 | 0.15269937 | 0.74126206 | 0.45854128 | 0.999976173 | 0.113190246 |
| YJR084W | CSN12 | 0.15269937 | 0.74216279 | 0.45799544 | 0.999976173 | 0.113327787 |
| YJR058C | APS2 | 0.15269937 | 0.74399778 | 0.45688456 | 0.999976173 | 0.113607989 |
| YDL242W | YDL242W | 0.15269937 | 0.74487006 | 0.45635703 | 0.999976173 | 0.113741186 |
| YIL017C | VID28 | 0.15269937 | 0.74704541 | 0.45504293 | 0.999976173 | 0.114073361 |
| YDL112W | TRM3 | 0.15269937 | 0.74788922 | 0.45453377 | 0.999976173 | 0.114202209 |
| YFR010W | UBP6 | 0.15269937 | 0.7479147 | 0.4545184 | 0.999976173 | 0.114206101 |
| YOL003C | PFA4 | 0.15269937 | 0.74825347 | 0.45431407 | 0.999976173 | 0.114257831 |
| YPL222W | FMP40 | 0.15269937 | 0.74867833 | 0.4540579 | 0.999976173 | 0.114322706 |
| YOR185C | GSP2 | 0.15269937 | 0.74981486 | 0.45337303 | 0.999976173 | 0.114496253 |
| YPL134C | ODC1 | 0.15269937 | 0.75024654 | 0.45311305 | 0.999976173 | 0.114562171 |
| YJL168C | SET2 | 0.15269937 | 0.75043445 | 0.4529999 | 0.999976173 | 0.114590865 |
| YOL013W-A | YOL013W-A | 0.15269937 | 0.75209204 | 0.45200255 | 0.999976173 | 0.114843977 |
| YCR028C-A | RIM1 | 0.15269937 | 0.75226053 | 0.45190124 | 0.999976173 | 0.114869705 |
| YJR026W | YJR026W | 0.15269937 | 0.75235176 | 0.45184638 | 0.999976173 | 0.114883637 |
| YKR018C | YKR018C | 0.15269937 | 0.75313679 | 0.45137456 | 0.999976173 | 0.11500351 |
| YOR015W | YOR015W | 0.15269937 | 0.75317649 | 0.45135071 | 0.999976173 | 0.115009572 |
| YLR356W | YLR356W | 0.15269937 | 0.75337271 | 0.45123282 | 0.999976173 | 0.115039535 |
| YDR036C | EHD3 | 0.15269937 | 0.75468075 | 0.45044742 | 0.999976173 | 0.115239271 |
| YHL032C | GUT1 | 0.15269937 | 0.75556892 | 0.44991457 | 0.999976173 | 0.115374894 |
| YPL184C | MRN1 | 0.15269937 | 0.75576129 | 0.4497992 | 0.999976173 | 0.11540427 |
| YHL022C | SPO11 | 0.15269937 | 0.7562634 | 0.44949816 | 0.999976173 | 0.115480941 |
| YOR248W | YOR248W | 0.15269937 | 0.75639958 | 0.44941654 | 0.999976173 | 0.115501736 |
| YDR419W | RAD30 | 0.15269937 | 0.75708555 | 0.44900549 | 0.999976173 | 0.115606484 |
| YDR389W | SAC7 | 0.15269937 | 0.75712111 | 0.44898419 | 0.999976173 | 0.115611914 |
| YOR139C | YOR139C | 0.15269937 | 0.75841461 | 0.44820971 | 0.999976173 | 0.11580943 |
| YIL002C | INP51 | 0.15269937 | 0.75859395 | 0.44810239 | 0.999976173 | 0.115836815 |
| YPR006C | ICL2 | 0.15269937 | 0.75957718 | 0.44751427 | 0.999976173 | 0.115986954 |
| YGR184C | UBR1 | 0.15269937 | 0.7595838 | 0.44751032 | 0.999976173 | 0.115987964 |
| YGL151W | NUT1 | 0.15269937 | 0.76100199 | 0.44666281 | 0.999976173 | 0.116204521 |
| YNL201C | PSY2 | 0.15269937 | 0.7614003 | 0.44642494 | 0.999976173 | 0.116265343 |
| YER101C | AST2 | 0.15269937 | 0.76142784 | 0.44640849 | 0.999976173 | 0.116269548 |
| YIL071C | PCI8 | 0.15269937 | 0.76172114 | 0.44623339 | 0.999976173 | 0.116314335 |
| YBL011W | SCT1 | 0.15269937 | 0.76213893 | 0.44598403 | 0.999976173 | 0.116378132 |
| YDR428C | BNA7 | 0.15269937 | 0.76235998 | 0.44585213 | 0.999976173 | 0.116411885 |
| YBR149W | ARA1 | 0.15269937 | 0.76321467 | 0.44534233 | 0.999976173 | 0.116542395 |
| YML097C | VPS9 | 0.15269937 | 0.76529092 | 0.4441053 | 0.999976173 | 0.116859438 |
| YDL229W | SSB1 | 0.15269937 | 0.76538306 | 0.44405045 | 0.999976173 | 0.116873508 |
| YDR519W | FPR2 | 0.15269937 | 0.76548765 | 0.44398819 | 0.999976173 | 0.116889479 |
| YMR116C | ASC1 | 0.15269937 | 0.76590983 | 0.44373693 | 0.999976173 | 0.116953946 |
| YCL051W | LRE1 | 0.15269937 | 0.76664351 | 0.44330048 | 0.999976173 | 0.117065978 |
| YPR070W | MED1 | 0.15269937 | 0.7690358 | 0.44187907 | 0.999976173 | 0.117431279 |
| YBL003C | HTA2 | 0.15269937 | 0.77014402 | 0.44122149 | 0.999976173 | 0.117600503 |
| YIL041W | GVP36 | 0.15269937 | 0.77099445 | 0.44071725 | 0.999976173 | 0.117730363 |

| | | | | | | |
|---|---|---|---|---|---|---|
| YGL027C | CWH41 | 0.15269937 | 0.77140571 | 0.44047353 | 0.999976173 | 0.117793162 |
| YDL167C | NRP1 | 0.15269937 | 0.77532038 | 0.43815744 | 0.999976173 | 0.118390931 |
| YDR103W | STE5 | 0.15269937 | 0.77578144 | 0.43788513 | 0.999976173 | 0.118461333 |
| YPL230W | USV1 | 0.15269937 | 0.77583908 | 0.43785109 | 0.999976173 | 0.118470136 |
| YIR030C | DCG1 | 0.15269937 | 0.77773781 | 0.4367307 | 0.999976173 | 0.11876007 |
| YKL068W | NUP100 | 0.15269937 | 0.77779405 | 0.43669754 | 0.999976173 | 0.118768657 |
| YBR172C | SMY2 | 0.15269937 | 0.77787824 | 0.4366479 | 0.999976173 | 0.118781513 |
| YOL105C | WSC3 | 0.15269937 | 0.78027674 | 0.43523513 | 0.999976173 | 0.119147763 |
| YNL095C | YNL095C | 0.15269937 | 0.78062616 | 0.43502953 | 0.999976173 | 0.11920112 |
| YDR096W | GIS1 | 0.15269937 | 0.78074173 | 0.43496155 | 0.999976173 | 0.119218767 |
| YGR233C | PHO81 | 0.15269937 | 0.78186432 | 0.43430146 | 0.999976173 | 0.119390186 |
| YER066W | RRT13 | 0.15269937 | 0.78330759 | 0.43345367 | 0.999976173 | 0.119610572 |
| YHR199C | AIM46 | 0.15269937 | 0.78384288 | 0.43313947 | 0.999976173 | 0.119692311 |
| YBR006W | UGA2 | 0.15269937 | 0.78477448 | 0.43259298 | 0.999976173 | 0.119834565 |
| YLR053C | YLR053C | 0.15269937 | 0.78531995 | 0.43227318 | 0.999976173 | 0.119917858 |
| YML076C | WAR1 | 0.15269937 | 0.78559623 | 0.43211126 | 0.999976173 | 0.119960047 |
| YJR024C | MDE1 | 0.15269937 | 0.78632736 | 0.43168292 | 0.999976173 | 0.120071689 |
| YER150W | SPI1 | 0.15269937 | 0.78652267 | 0.43156854 | 0.999976173 | 0.120101512 |
| YNL320W | YNL320W | 0.15269937 | 0.78843359 | 0.43045035 | 0.999976173 | 0.120393309 |
| YIL102C | YIL102C | 0.15269937 | 0.78855497 | 0.43037938 | 0.999976173 | 0.120411843 |
| YKL183W | LOT5 | 0.15269937 | 0.78858904 | 0.43035945 | 0.999976173 | 0.120417047 |
| YJR088C | EMC2 | 0.15269937 | 0.78859233 | 0.43035753 | 0.999976173 | 0.120417549 |
| YMR074C | YMR074C | 0.15269937 | 0.78897018 | 0.43013665 | 0.999976173 | 0.120475246 |
| YIL067C | YIL067C | 0.15269937 | 0.78988166 | 0.42960411 | 0.999976173 | 0.120614428 |
| YMR154C | RIM13 | 0.15269937 | 0.7912231 | 0.42882106 | 0.999976173 | 0.120819265 |
| YHR001W-A | QCR10 | 0.15269937 | 0.79222244 | 0.42823824 | 0.999976173 | 0.120971864 |
| YOR085W | OST3 | 0.15269937 | 0.79230639 | 0.4281893 | 0.999976173 | 0.120984683 |
| YJL144W | YJL144W | 0.15269937 | 0.79241726 | 0.42812467 | 0.999976173 | 0.121001613 |
| YJR126C | VPS70 | 0.15269937 | 0.79305631 | 0.42775228 | 0.999976173 | 0.121099196 |
| YNL074C | MLF3 | 0.15269937 | 0.79418932 | 0.4270925 | 0.999976173 | 0.121272206 |
| YDR009W | GAL3 | 0.15269937 | 0.79485528 | 0.42670497 | 0.999976173 | 0.121373898 |
| YCR069W | CPR4 | 0.15269937 | 0.79514709 | 0.42653523 | 0.999976173 | 0.121418456 |
| YGR288W | MAL13 | 0.15269937 | 0.79515006 | 0.4265335 | 0.999976173 | 0.12141891 |
| YLR072W | YLR072W | 0.15269937 | 0.79549039 | 0.42633558 | 0.999976173 | 0.121470879 |
| YMR164C | MSS11 | 0.15269937 | 0.79601181 | 0.42603246 | 0.999976173 | 0.121550499 |
| YNR049C | MSO1 | 0.15269937 | 0.79602989 | 0.42602195 | 0.999976173 | 0.12155326 |
| YPR007C | REC8 | 0.15269937 | 0.79611995 | 0.42596961 | 0.999976173 | 0.121567011 |
| YHR156C | LIN1 | 0.15269937 | 0.80032581 | 0.42352939 | 0.999976173 | 0.122209243 |
| YDL219W | DTD1 | 0.15269937 | 0.80060065 | 0.42337021 | 0.999976173 | 0.122251212 |
| YLR210W | CLB4 | 0.15269937 | 0.8009288 | 0.42318021 | 0.999976173 | 0.12230132 |
| YHR105W | YPT35 | 0.15269937 | 0.80122003 | 0.42301163 | 0.999976173 | 0.12234579 |
| YDR030C | RAD28 | 0.15269937 | 0.80169661 | 0.42273583 | 0.999976173 | 0.122418565 |
| YDR273W | DON1 | 0.15269937 | 0.80186821 | 0.42263655 | 0.999976173 | 0.122444767 |
| YGR169C | PUS6 | 0.15269937 | 0.80237183 | 0.42234526 | 0.999976173 | 0.122521669 |
| YDR259C | YAP6 | 0.15269937 | 0.80414689 | 0.42131952 | 0.999976173 | 0.122792721 |
| YDR277C | MTH1 | 0.15269937 | 0.8052604 | 0.42067682 | 0.999976173 | 0.122962752 |
| YNL259C | ATX1 | 0.15269937 | 0.80546279 | 0.42056006 | 0.999976173 | 0.122993657 |
| YOR197W | MCA1 | 0.15269937 | 0.80623676 | 0.42011375 | 0.999976173 | 0.123111842 |
| YPL141C | YPL141C | 0.15269937 | 0.80849263 | 0.41881447 | 0.999976173 | 0.123456313 |
| YDR370C | YDR370C | 0.15269937 | 0.80954759 | 0.41820768 | 0.999976173 | 0.123617403 |
| YPR011C | YPR011C | 0.15269937 | 0.80956114 | 0.41819989 | 0.999976173 | 0.123619473 |
| YPR109W | YPR109W | 0.15269937 | 0.81084948 | 0.41745956 | 0.999976173 | 0.123816201 |
| YNL067W | RPL9B | 0.15269937 | 0.81239166 | 0.41657439 | 0.999976173 | 0.124051691 |
| YGL141W | HUL5 | 0.15269937 | 0.81298586 | 0.41623364 | 0.999976173 | 0.124142425 |
| YNL108C | YNL108C | 0.15269937 | 0.81333046 | 0.41603609 | 0.999976173 | 0.124195045 |
| YGR022C | YGR022C | 0.15269937 | 0.8135721 | 0.41589761 | 0.999976173 | 0.124231943 |
| YML003W | YML003W | 0.15269937 | 0.81507297 | 0.41503804 | 0.999976173 | 0.124461125 |
| YPR083W | MDM36 | 0.15269937 | 0.81562613 | 0.4147215 | 0.999976173 | 0.124545594 |
| YIR025W | MND2 | 0.15269937 | 0.81662824 | 0.41414842 | 0.999976173 | 0.124698614 |
| YNL134C | YNL134C | 0.15269937 | 0.81716193 | 0.41384341 | 0.999976173 | 0.124780108 |
| YPR138C | MEP3 | 0.15269937 | 0.81808341 | 0.41331709 | 0.999976173 | 0.124920818 |
| YBR161W | CSH1 | 0.15269937 | 0.82055821 | 0.41190551 | 0.999976173 | 0.125298718 |
| YAL058W | CNE1 | 0.15269937 | 0.82079853 | 0.41176859 | 0.999976173 | 0.125335416 |
| YLR436C | ECM30 | 0.15269937 | 0.82082899 | 0.41175124 | 0.999976173 | 0.125340066 |

| | | | | | | |
|---|---|---|---|---|---|---|
| YHR121W | LSM12 | 0.15269937 | 0.82192641 | 0.41112635 | 0.999976173 | 0.125507641 |
| YMR176W | ECM5 | 0.15269937 | 0.8235887 | 0.41018088 | 0.999976173 | 0.125761472 |
| YKR019C | IRS4 | 0.15269937 | 0.82373733 | 0.41009641 | 0.999976173 | 0.125784167 |
| YNL278W | CAF120 | 0.15269937 | 0.82395627 | 0.40997199 | 0.999976173 | 0.1258176 |
| YPR134W | MSS18 | 0.15269937 | 0.82408625 | 0.40989814 | 0.999976173 | 0.125837447 |
| YPL145C | KES1 | 0.15269937 | 0.82446241 | 0.40968445 | 0.999976173 | 0.125894888 |
| YGL235W | YGL235W | 0.15269937 | 0.82477766 | 0.40950543 | 0.999976173 | 0.125943025 |
| YGL261C | PAU11 | 0.15269937 | 0.82511166 | 0.4093158 | 0.999976173 | 0.125994027 |
| YLR273C | PIG1 | 0.15269937 | 0.82654775 | 0.40850105 | 0.999976173 | 0.126213318 |
| YMR179W | SPT21 | 0.15269937 | 0.8266935 | 0.40841842 | 0.999976173 | 0.126235573 |
| YHR117W | TOM71 | 0.15269937 | 0.8274767 | 0.40797454 | 0.999976173 | 0.126355167 |
| YKR029C | SET3 | 0.15269937 | 0.82778694 | 0.4077988 | 0.999976173 | 0.126402541 |
| YAL048C | GEM1 | 0.15269937 | 0.8287642 | 0.40724549 | 0.999976173 | 0.126551768 |
| YBR146W | MRPS9 | 0.15269937 | 0.83115808 | 0.40589199 | 0.999976173 | 0.126917311 |
| YLR255C | YLR255C | 0.15269937 | 0.83318052 | 0.40475061 | 0.999976173 | 0.127226137 |
| YKL201C | MNN4 | 0.15269937 | 0.83359021 | 0.40451963 | 0.999976173 | 0.127288696 |
| YIL010W | DOT5 | 0.15269937 | 0.83576728 | 0.40329355 | 0.999976173 | 0.127621133 |
| YKR003W | OSH6 | 0.15269937 | 0.83711024 | 0.40253833 | 0.999976173 | 0.127826202 |
| YNL030W | HHF2 | 0.15269937 | 0.83966498 | 0.401104 | 0.999976173 | 0.12821631 |
| YPR092W | YPR092W | 0.15269937 | 0.84000497 | 0.40091335 | 0.999976173 | 0.128268227 |
| YPR114W | YPR114W | 0.15269937 | 0.84002364 | 0.40090289 | 0.999976173 | 0.128271077 |
| YKR087C | OMA1 | 0.15269937 | 0.8400303 | 0.40089915 | 0.999976173 | 0.128272094 |
| YJL084C | ALY2 | 0.15269937 | 0.84091933 | 0.40040089 | 0.999976173 | 0.128407848 |
| YLR124W | YLR124W | 0.15269937 | 0.84276574 | 0.39936725 | 0.999976173 | 0.128689793 |
| YGR231C | PHB2 | 0.15269937 | 0.84311109 | 0.3991741 | 0.999976173 | 0.128742529 |
| YDR026C | YDR026C | 0.15269937 | 0.8435908 | 0.3989059 | 0.999976173 | 0.12881578 |
| YIR014W | YIR014W | 0.15269937 | 0.84537474 | 0.39790945 | 0.999976173 | 0.129088187 |
| YPL022W | RAD1 | 0.15269937 | 0.8461267 | 0.39748988 | 0.999976173 | 0.12920301 |
| YER090W | TRP2 | 0.15269937 | 0.84627726 | 0.39740591 | 0.999976173 | 0.129226 |
| YBR224W | YBR224W | 0.15269937 | 0.84697785 | 0.39701529 | 0.999976173 | 0.12933298 |
| YLL038C | ENT4 | 0.15269937 | 0.84790357 | 0.39649951 | 0.999976173 | 0.129474338 |
| YHR162W | YHR162W | 0.15269937 | 0.84809936 | 0.39639047 | 0.999976173 | 0.129504235 |
| YCL057W | PRD1 | 0.15269937 | 0.84818151 | 0.39634473 | 0.999976173 | 0.129516778 |
| YCR101C | YCR101C | 0.15269937 | 0.84826383 | 0.39629889 | 0.999976173 | 0.129529349 |
| YBL008W | HIR1 | 0.15269937 | 0.8499312 | 0.39537119 | 0.999976173 | 0.129783955 |
| YIL029C | YIL029C | 0.15269937 | 0.8506804 | 0.39495477 | 0.999976173 | 0.129898358 |
| YOL103W | ITR2 | 0.15269937 | 0.85237308 | 0.39401494 | 0.999976173 | 0.130156828 |
| YHL045W | YHL045W | 0.15269937 | 0.85294353 | 0.3936985 | 0.999976173 | 0.130243937 |
| YKL046C | DCW1 | 0.15269937 | 0.85344539 | 0.39342025 | 0.999976173 | 0.13032057 |
| YML058W | SML1 | 0.15269937 | 0.85380117 | 0.39322306 | 0.999976173 | 0.130374897 |
| YEL003W | GIM4 | 0.15269937 | 0.85576955 | 0.39213317 | 0.999976173 | 0.130675468 |
| YJL119C | YJL119C | 0.15269937 | 0.85603273 | 0.39198759 | 0.999976173 | 0.130715655 |
| YOR251C | TUM1 | 0.15269937 | 0.85766176 | 0.39108719 | 0.999976173 | 0.130964407 |
| YER054C | GIP2 | 0.15269937 | 0.85800151 | 0.39089957 | 0.999976173 | 0.131016286 |
| YGR117C | YGR117C | 0.15269937 | 0.85841889 | 0.39066914 | 0.999976173 | 0.13108002 |
| YPL067C | YPL067C | 0.15269937 | 0.86332241 | 0.38796822 | 0.999976173 | 0.131828784 |
| YNL029C | KTR5 | 0.15269937 | 0.86417746 | 0.38749841 | 0.999976173 | 0.131959351 |
| YOR126C | IAH1 | 0.15269937 | 0.86645906 | 0.38624649 | 0.999976173 | 0.132307749 |
| YCR016W | YCR016W | 0.15269937 | 0.86802171 | 0.38539049 | 0.999976173 | 0.132546364 |
| YGR291C | YGR291C | 0.15269937 | 0.8700569 | 0.38427737 | 0.999976173 | 0.132857137 |
| YPR052C | NHP6A | 0.15269937 | 0.87031648 | 0.38413554 | 0.999976173 | 0.132896774 |
| YGL101W | YGL101W | 0.15269937 | 0.87041505 | 0.38408169 | 0.999976173 | 0.132911827 |
| YML010C-B | NA | 0.15269937 | 0.87102682 | 0.38374758 | 0.999976173 | 0.133005243 |
| YNR010W | CSE2 | 0.15269937 | 0.87106426 | 0.38372714 | 0.999976173 | 0.133010959 |
| YKR088C | TVP38 | 0.15269937 | 0.87204064 | 0.38319429 | 0.999976173 | 0.133160053 |
| YCL024W | KCC4 | 0.15269937 | 0.87324587 | 0.38253717 | 0.999976173 | 0.133344091 |
| YPL103C | FMP30 | 0.15269937 | 0.87355556 | 0.38236843 | 0.999976173 | 0.13339138 |
| YGL226W | MTC3 | 0.15269937 | 0.87618811 | 0.3809359 | 0.999976173 | 0.133793369 |
| YOR342C | YOR342C | 0.15269937 | 0.87774875 | 0.38008822 | 0.999976173 | 0.134031678 |
| YLR080W | EMP46 | 0.15269937 | 0.88005523 | 0.37883755 | 0.999976173 | 0.134383875 |
| YPR196W | YPR196W | 0.15269937 | 0.88006062 | 0.37883464 | 0.999976173 | 0.134384698 |
| YKL214C | YRA2 | 0.15269937 | 0.88119476 | 0.37822059 | 0.999976173 | 0.134557882 |
| YDR319C | YDR319C | 0.15269937 | 0.88243268 | 0.37755106 | 0.999976173 | 0.13474691 |
| YJL162C | JJJ2 | 0.15269937 | 0.88398315 | 0.37671351 | 0.999976173 | 0.134983666 |

| | | | | | | |
|---|---|---|---|---|---|---|
| YCR001W | YCR001W | 0.15269937 | 0.88420106 | 0.37659589 | 0.999976173 | 0.135016941 |
| YNL047C | SLM2 | 0.15269937 | 0.88506898 | 0.37612765 | 0.999976173 | 0.135149471 |
| YBL031W | SHE1 | 0.15269937 | 0.88528084 | 0.3760134 | 0.999976173 | 0.135181823 |
| YPL202C | AFT2 | 0.15269937 | 0.88636308 | 0.37543014 | 0.999976173 | 0.13534708 |
| YLR460C | YLR460C | 0.15269937 | 0.88651451 | 0.37534857 | 0.999976173 | 0.135370203 |
| YDR205W | MSC2 | 0.15269937 | 0.88986083 | 0.37354888 | 0.999976173 | 0.135881184 |
| YDL241W | YDL241W | 0.15269937 | 0.89115778 | 0.37285281 | 0.999976173 | 0.136079227 |
| YIL084C | SDS3 | 0.15269937 | 0.89131236 | 0.3727699 | 0.999976173 | 0.136102832 |
| YBR260C | RGD1 | 0.15269937 | 0.89244509 | 0.3721627 | 0.999976173 | 0.136275799 |
| YPL223C | GRE1 | 0.15269937 | 0.8933251 | 0.3716914 | 0.999976173 | 0.136410177 |
| YOR230W | WTM1 | 0.15269937 | 0.89542486 | 0.37056834 | 0.999976173 | 0.136730808 |
| YDL192W | ARF1 | 0.15269937 | 0.89579057 | 0.37037295 | 0.999976173 | 0.136786652 |
| YOL088C | MPD2 | 0.15269937 | 0.89676316 | 0.36985364 | 0.999976173 | 0.136935165 |
| YER183C | FAU1 | 0.15269937 | 0.89765091 | 0.36938003 | 0.999976173 | 0.137070724 |
| YOR317W | FAA1 | 0.15269937 | 0.89766212 | 0.36937405 | 0.999976173 | 0.137072437 |
| YNL107W | YAF9 | 0.15269937 | 0.8985744 | 0.36888775 | 0.999976173 | 0.137211742 |
| YHL042W | YHL042W | 0.15269937 | 0.89910529 | 0.36860494 | 0.999976173 | 0.137292808 |
| YLR034C | SMF3 | 0.15269937 | 0.89953145 | 0.36837801 | 0.999976173 | 0.137357882 |
| YGL210W | YPT32 | 0.15269937 | 0.90047195 | 0.36787752 | 0.999976173 | 0.137501496 |
| YNL068C | FKH2 | 0.15269937 | 0.90122182 | 0.36747877 | 0.999976173 | 0.137616 |
| YGR032W | GSC2 | 0.15269937 | 0.90129523 | 0.36743975 | 0.999976173 | 0.13762721 |
| YBL021C | HAP3 | 0.15269937 | 0.90131318 | 0.3674302 | 0.999976173 | 0.137629952 |
| YJL060W | BNA3 | 0.15269937 | 0.9016843 | 0.36723298 | 0.999976173 | 0.13768662 |
| YLR432W | IMD3 | 0.15269937 | 0.90183843 | 0.36715109 | 0.999976173 | 0.137710156 |
| YBL069W | AST1 | 0.15269937 | 0.90350093 | 0.36626849 | 0.999976173 | 0.137964019 |
| YFL014W | HSP12 | 0.15269937 | 0.90379368 | 0.36611322 | 0.999976173 | 0.138008722 |
| YLR353W | BUD8 | 0.15269937 | 0.90500898 | 0.36546905 | 0.999976173 | 0.138194297 |
| YDR474C | NA | 0.15269937 | 0.9056597 | 0.36512442 | 0.999976173 | 0.138293662 |
| YBR150C | TBS1 | 0.15269937 | 0.90679792 | 0.3645221 | 0.999976173 | 0.138467468 |
| YDR014W | RAD61 | 0.15269937 | 0.90714747 | 0.36433726 | 0.999976173 | 0.138520843 |
| YHR125W | YHR125W | 0.15269937 | 0.90734311 | 0.36423382 | 0.999976173 | 0.138550718 |
| YDR504C | SPG3 | 0.15269937 | 0.90760498 | 0.3640954 | 0.999976173 | 0.138590705 |
| YDR383C | NKP1 | 0.15269937 | 0.91160661 | 0.36198432 | 0.999976173 | 0.139201752 |
| YOL039W | RPP2A | 0.15269937 | 0.91304583 | 0.36122694 | 0.999976173 | 0.139421519 |
| YKR001C | VPS1 | 0.15269937 | 0.91458368 | 0.36041874 | 0.999976173 | 0.139656347 |
| YDR121W | DPB4 | 0.15269937 | 0.91498996 | 0.36020542 | 0.999976173 | 0.139718387 |
| YBR114W | RAD16 | 0.15269937 | 0.91749347 | 0.35889266 | 0.999976173 | 0.14010067 |
| YOR306C | MCH5 | 0.10797476 | 1.29864355 | 0.19407784 | 0.999976173 | 0.140220722 |
| YEL007W | YEL007W | 0.15269937 | 0.92049803 | 0.35732113 | 0.999976173 | 0.140559465 |
| YDL018C | ERP3 | 0.15269937 | 0.92054765 | 0.35729522 | 0.999976173 | 0.140567042 |
| YFR013W | IOC3 | 0.15269937 | 0.92118982 | 0.35695991 | 0.999976173 | 0.140665102 |
| YHR150W | PEX28 | 0.15269937 | 0.9215884 | 0.35675189 | 0.999976173 | 0.140725964 |
| YGL037C | PNC1 | 0.15269937 | 0.9235349 | 0.35573712 | 0.999976173 | 0.141023194 |
| YPL088W | YPL088W | 0.15269937 | 0.92566643 | 0.35462798 | 0.999976173 | 0.141348677 |
| YDL189W | RBS1 | 0.15269937 | 0.92569296 | 0.35461419 | 0.999976173 | 0.141352728 |
| YBR078W | ECM33 | 0.15269937 | 0.92680753 | 0.35403511 | 0.999976173 | 0.141522922 |
| YGL254W | FZF1 | 0.15269937 | 0.9277232 | 0.35355981 | 0.999976173 | 0.141662744 |
| YDR258C | HSP78 | 0.15269937 | 0.92803244 | 0.35339939 | 0.999976173 | 0.141709965 |
| YOR238W | YOR238W | 0.15269937 | 0.92957081 | 0.35260201 | 0.999976173 | 0.141944873 |
| YIL065C | FIS1 | 0.15269937 | 0.93033291 | 0.35220741 | 0.999976173 | 0.142061246 |
| YGR080W | TWF1 | 0.15269937 | 0.93125246 | 0.35173167 | 0.999976173 | 0.14220166 |
| YHL044W | YHL044W | 0.15269937 | 0.93191002 | 0.35139171 | 0.999976173 | 0.14230207 |
| YBR259W | YBR259W | 0.15269937 | 0.93288884 | 0.35088606 | 0.999976173 | 0.142451534 |
| YOR277C | YOR277C | 0.15269937 | 0.93319753 | 0.35072668 | 0.999976173 | 0.142498672 |
| YNL085W | MKT1 | 0.15269937 | 0.93768667 | 0.3484142 | 0.999976173 | 0.14318416 |
| YBR051W | YBR051W | 0.15269937 | 0.94024336 | 0.34710151 | 0.999976173 | 0.143574564 |
| YML100W-A | YML100W-A | 0.15269937 | 0.94205327 | 0.34617415 | 0.999976173 | 0.143850936 |
| YKL147C | YKL147C | 0.15269937 | 0.94225159 | 0.34607263 | 0.999976173 | 0.14388122 |
| YGR206W | MVB12 | 0.15269937 | 0.94254032 | 0.34592487 | 0.999976173 | 0.143925309 |
| YOR069W | VPS5 | 0.15269937 | 0.94377157 | 0.34529519 | 0.999976173 | 0.14411332 |
| YGR111W | YGR111W | 0.15269937 | 0.94380785 | 0.34527665 | 0.999976173 | 0.14411886 |
| YGR097W | ASK10 | 0.15269937 | 0.94471926 | 0.34481103 | 0.999976173 | 0.144258032 |
| YCR021C | HSP30 | 0.15269937 | 0.94485672 | 0.34474084 | 0.999976173 | 0.144279021 |
| YPR145W | ASN1 | 0.15269937 | 0.94631253 | 0.34399803 | 0.999976173 | 0.144501322 |

| | | | | | | |
|---|---|---|---|---|---|---|
| YPR005C | HAL1 | 0.15269937 | 0.94658886 | 0.34385715 | 0.999976173 | 0.144543519 |
| YPL133C | RDS2 | 0.15269937 | 0.94698839 | 0.34365353 | 0.999976173 | 0.144604527 |
| YGR045C | YGR045C | 0.15269937 | 0.94749557 | 0.34339515 | 0.999976173 | 0.144681973 |
| YMR109W | MYO5 | 0.15269937 | 0.95015403 | 0.34204285 | 0.999976173 | 0.145087918 |
| YLR016C | PML1 | 0.15269937 | 0.95029423 | 0.34197163 | 0.999976173 | 0.145109327 |
| YNL291C | MID1 | 0.15269937 | 0.95263421 | 0.34078433 | 0.999976173 | 0.14546664 |
| YLR338W | OPI9 | 0.15269937 | 0.95275927 | 0.34072094 | 0.999976173 | 0.145485737 |
| YDR183W | PLP1 | 0.15269937 | 0.95321642 | 0.34048932 | 0.999976173 | 0.145555543 |
| YOR228C | YOR228C | 0.15269937 | 0.95487353 | 0.33965056 | 0.999976173 | 0.145808583 |
| YAL023C | PMT2 | 0.15269937 | 0.95527174 | 0.33944921 | 0.999976173 | 0.145869388 |
| YMR118C | YMR118C | 0.15269937 | 0.95647429 | 0.33884159 | 0.999976173 | 0.146053017 |
| YKR064W | OAF3 | 0.15269937 | 0.95756359 | 0.3382918 | 0.999976173 | 0.146219353 |
| YHL017W | YHL017W | 0.15269937 | 0.95756689 | 0.33829014 | 0.999976173 | 0.146219857 |
| YOR212W | STE4 | 0.15269937 | 0.95995911 | 0.33708475 | 0.999976173 | 0.146585147 |
| YHL046C | PAU13 | 0.15269937 | 0.96047096 | 0.3368272 | 0.999976173 | 0.146663306 |
| YCL012W | NA | 0.15269937 | 0.96138672 | 0.33636673 | 0.999976173 | 0.146803143 |
| YPR038W | IRC16 | 0.15269937 | 0.96232942 | 0.33589314 | 0.999976173 | 0.146947092 |
| YLR425W | TUS1 | 0.15269937 | 0.96363008 | 0.33524042 | 0.999976173 | 0.147145702 |
| YJL206C | YJL206C | 0.15269937 | 0.96479327 | 0.33465737 | 0.999976173 | 0.147323321 |
| YPL236C | YPL236C | 0.15269937 | 0.96717982 | 0.33346318 | 0.999976173 | 0.147687746 |
| YGR079W | YGR079W | 0.15269937 | 0.96925047 | 0.33242928 | 0.999976173 | 0.148003933 |
| YHR161C | YAP1801 | 0.15269937 | 0.97014988 | 0.33198084 | 0.999976173 | 0.148141272 |
| YLR136C | TIS11 | 0.15269937 | 0.97060383 | 0.33175466 | 0.999976173 | 0.148210589 |
| YGL133W | ITC1 | 0.15269937 | 0.97087289 | 0.33162064 | 0.999976173 | 0.148251675 |
| YLR003C | CMS1 | 0.15269937 | 0.97291882 | 0.33060273 | 0.999976173 | 0.148564087 |
| YGR087C | PDC6 | 0.15269937 | 0.97302468 | 0.33055012 | 0.999976173 | 0.148580251 |
| YOR061W | CKA2 | 0.15269937 | 0.97422467 | 0.3299541 | 0.999976173 | 0.148763489 |
| YNL083W | SAL1 | 0.15269937 | 0.97463425 | 0.32975082 | 0.999976173 | 0.148826032 |
| YNL190W | YNL190W | 0.15269937 | 0.9749725 | 0.32958301 | 0.999976173 | 0.148877682 |
| YJL151C | SNA3 | 0.15269937 | 0.97666467 | 0.32874431 | 0.999976173 | 0.149136076 |
| YBR064W | YBR064W | 0.15269937 | 0.97816506 | 0.32800183 | 0.999976173 | 0.149365185 |
| YBL010C | YBL010C | 0.15269937 | 0.98012827 | 0.32703196 | 0.999976173 | 0.149664965 |
| YPL089C | RLM1 | 0.15269937 | 0.9804032 | 0.32689629 | 0.999976173 | 0.149706946 |
| YLR271W | YLR271W | 0.15269937 | 0.98409764 | 0.3250767 | 0.999976173 | 0.150271085 |
| YPL269W | KAR9 | 0.15269937 | 0.98432728 | 0.32496381 | 0.999976173 | 0.150306151 |
| YLR452C | SST2 | 0.15269937 | 0.98633697 | 0.323977 | 0.999976173 | 0.150613029 |
| YJL130C | URA2 | 0.15269937 | 0.98822023 | 0.32305403 | 0.999976173 | 0.150900602 |
| YDR320C | SWA2 | 0.15269937 | 0.98850733 | 0.32291348 | 0.999976173 | 0.150944443 |
| YPL127C | HHO1 | 0.15269937 | 0.98868217 | 0.3228279 | 0.999976173 | 0.15097114 |
| YNL104C | LEU4 | 0.15269937 | 0.98938055 | 0.32248623 | 0.999976173 | 0.151077782 |
| YKR023W | YKR023W | 0.15269937 | 0.99154053 | 0.32143097 | 0.999976173 | 0.15140761 |
| YKR089C | TGL4 | 0.15269937 | 0.99193029 | 0.3212408 | 0.999976173 | 0.151467126 |
| YOR354C | MSC6 | 0.15269937 | 0.99212985 | 0.32114345 | 0.999976173 | 0.151497599 |
| YNL266W | YNL266W | 0.15269937 | 0.99266334 | 0.32088332 | 0.999976173 | 0.151579063 |
| YPR111W | DBF20 | 0.15269937 | 0.99419732 | 0.3201361 | 0.999976173 | 0.1518133 |
| YJL141C | YAK1 | 0.15269937 | 0.99446406 | 0.32000628 | 0.999976173 | 0.151854031 |
| YML002W | YML002W | 0.15269937 | 0.99450424 | 0.31998673 | 0.999976173 | 0.151860167 |
| YJL064W | YJL064W | 0.15269937 | 0.99453393 | 0.31997228 | 0.999976173 | 0.151864701 |
| YHR033W | YHR033W | 0.15269937 | 0.996337 | 0.31909574 | 0.999976173 | 0.152140028 |
| YPL200W | CSM4 | 0.15269937 | 0.99836964 | 0.31810947 | 0.999976173 | 0.152450411 |
| YDR420W | HKR1 | 0.15269937 | 0.99852538 | 0.31803399 | 0.999976173 | 0.152474192 |
| YDR475C | JIP4 | 0.15269937 | 0.99885779 | 0.31787291 | 0.999976173 | 0.152524952 |
| YOR264W | DSE3 | 0.15269937 | 1.00110425 | 0.31678576 | 0.999976173 | 0.152867984 |
| YCR030C | SYP1 | 0.15269937 | 1.00115125 | 0.31676304 | 0.999976173 | 0.152875161 |
| YER072W | VTC1 | 0.15269937 | 1.00153272 | 0.31657868 | 0.999976173 | 0.152933411 |
| YCR100C | YCR100C | 0.15269937 | 1.00314032 | 0.31580253 | 0.999976173 | 0.153178891 |
| YMR174C | PAI3 | 0.15269937 | 1.00367521 | 0.31554457 | 0.999976173 | 0.153260567 |
| YML072C | TCB3 | 0.15269937 | 1.00481199 | 0.31499677 | 0.999976173 | 0.153434154 |
| YOR213C | SAS5 | 0.15269937 | 1.00704501 | 0.31392255 | 0.999976173 | 0.153775135 |
| YBR162W-A | YSY6 | 0.15269937 | 1.00822938 | 0.31335377 | 0.999976173 | 0.153955987 |
| YIR007W | YIR007W | 0.15269937 | 1.0085701 | 0.31319027 | 0.999976173 | 0.154008015 |
| YLR420W | URA4 | 0.15269937 | 1.01112172 | 0.31196762 | 0.999976173 | 0.154397645 |
| YIL044C | AGE2 | 0.15269937 | 1.01142264 | 0.31182363 | 0.999976173 | 0.154443596 |
| YEL043W | YEL043W | 0.15269937 | 1.01167596 | 0.31170246 | 0.999976173 | 0.154482278 |

| | | | | | | |
|---|---|---|---|---|---|---|
| YNL205C | YNL205C | 0.15269937 | 1.01252046 | 0.31129873 | 0.999976173 | 0.154611232 |
| YJR044C | VPS55 | 0.15269937 | 1.01319177 | 0.31097803 | 0.999976173 | 0.154713741 |
| YDL129W | YDL129W | 0.15269937 | 1.01373031 | 0.31072092 | 0.999976173 | 0.154795975 |
| YLR206W | ENT2 | 0.15269937 | 1.01579377 | 0.30973709 | 0.999976173 | 0.155111064 |
| YPR091C | YPR091C | 0.15269937 | 1.01825982 | 0.308564 | 0.999976173 | 0.155487629 |
| YDL184C | RPL41A | 0.15269937 | 1.01850244 | 0.30844875 | 0.999976173 | 0.155524676 |
| YLR091W | GEP5 | 0.15269937 | 1.01899013 | 0.30821717 | 0.999976173 | 0.155599147 |
| YOL104C | NDJ1 | 0.15269937 | 1.01910685 | 0.30816176 | 0.999976173 | 0.15561697 |
| YBR226C | YBR226C | 0.15269937 | 1.02031564 | 0.30758832 | 0.999976173 | 0.155801551 |
| YKL115C | YKL115C | 0.15269937 | 1.02135665 | 0.30709503 | 0.999976173 | 0.155960513 |
| YHR184W | SSP1 | 0.15269937 | 1.02158947 | 0.30698478 | 0.999976173 | 0.155996064 |
| YOR273C | TPO4 | 0.15269937 | 1.0230155 | 0.30631007 | 0.999976173 | 0.156213817 |
| YML042W | CAT2 | 0.15269937 | 1.02342989 | 0.30611419 | 0.999976173 | 0.156277096 |
| YLR437C | DIF1 | 0.15269937 | 1.02368338 | 0.30599441 | 0.999976173 | 0.156315803 |
| YDR134C | YDR134C | 0.15269937 | 1.0264492 | 0.30468948 | 0.999976173 | 0.156738141 |
| YNL159C | ASI2 | 0.15269937 | 1.03095736 | 0.30257044 | 0.999976173 | 0.157426535 |
| YJL213W | YJL213W | 0.15269937 | 1.03107435 | 0.30251558 | 0.999976173 | 0.157444399 |
| YBR201W | DER1 | 0.15269937 | 1.0316105 | 0.30226425 | 0.999976173 | 0.157526269 |
| YER173W | 14 | 0.15269937 | 1.03241209 | 0.30188875 | 0.999976173 | 0.157648672 |
| YIL015C-A | NA | 0.15269937 | 1.03261359 | 0.30179441 | 0.999976173 | 0.15767944 |
| YPL023C | MET12 | 0.15269937 | 1.03368796 | 0.30129171 | 0.999976173 | 0.157843496 |
| YPR039W | YPR039W | 0.15269937 | 1.03382037 | 0.3012298 | 0.999976173 | 0.157863714 |
| YDR225W | HTA1 | 0.15269937 | 1.03389315 | 0.30119577 | 0.999976173 | 0.157874828 |
| YFL010W-A | AUA1 | 0.15269937 | 1.03390292 | 0.3011912 | 0.999976173 | 0.15787632 |
| YER132C | PMD1 | 0.15269937 | 1.03409572 | 0.30110107 | 0.999976173 | 0.157905761 |
| YBR187W | GDT1 | 0.15269937 | 1.03704258 | 0.29972569 | 0.999976173 | 0.158355745 |
| YDL194W | SNF3 | 0.15269937 | 1.03738709 | 0.29956517 | 0.999976173 | 0.158408351 |
| YGR286C | BIO2 | 0.15269937 | 1.0415688 | 0.29762136 | 0.999976173 | 0.159046895 |
| YOR271C | FSF1 | 0.15269937 | 1.04200855 | 0.29741744 | 0.999976173 | 0.159114044 |
| YDL178W | DLD2 | 0.15269937 | 1.04433614 | 0.29633964 | 0.999976173 | 0.159469466 |
| YLL062C | MHT1 | 0.15269937 | 1.04692018 | 0.29514616 | 0.999976173 | 0.159864048 |
| YGL117W | YGL117W | 0.15269937 | 1.04943536 | 0.29398758 | 0.999976173 | 0.160248113 |
| YOR043W | WHI2 | 0.15269937 | 1.05272534 | 0.29247671 | 0.999976173 | 0.160750492 |
| YMR253C | YMR253C | 0.15269937 | 1.05680713 | 0.29060947 | 0.999976173 | 0.161373779 |
| YLR262C | YPT6 | 0.15269937 | 1.0585061 | 0.28983464 | 0.999976173 | 0.16163321 |
| YGR052W | FMP48 | 0.15269937 | 1.05976773 | 0.28926016 | 0.999976173 | 0.16182586 |
| YFR039C | YFR039C | 0.15269937 | 1.06095241 | 0.28872142 | 0.999976173 | 0.162006761 |
| YBL081W | YBL081W | 0.15269937 | 1.06130938 | 0.28855922 | 0.999976173 | 0.162061269 |
| YHL021C | AIM17 | 0.15269937 | 1.0627183 | 0.28791963 | 0.999976173 | 0.16227641 |
| YOR374W | ALD4 | 0.15269937 | 1.06569512 | 0.28657141 | 0.999976173 | 0.162730969 |
| YOR320C | GNT1 | 0.15269937 | 1.06572418 | 0.28655827 | 0.999976173 | 0.162735407 |
| YEL039C | CYC7 | 0.15269937 | 1.06729429 | 0.28584891 | 0.999976173 | 0.162975161 |
| YAL028W | FRT2 | 0.15269937 | 1.06810131 | 0.28548477 | 0.999976173 | 0.163098392 |
| YOR252W | TMA16 | 0.15269937 | 1.06874125 | 0.28519624 | 0.999976173 | 0.163196111 |
| YOR270C | VPH1 | 0.15269937 | 1.06875221 | 0.2851913 | 0.999976173 | 0.163197784 |
| YIL050W | PCL7 | 0.15269937 | 1.07240169 | 0.28354964 | 0.999976173 | 0.163755058 |
| YHL036W | MUP3 | 0.15269937 | 1.07268568 | 0.28342216 | 0.999976173 | 0.163798423 |
| YLR376C | PSY3 | 0.15269937 | 1.07322152 | 0.28318173 | 0.999976173 | 0.163880246 |
| YHL012W | YHL012W | 0.15269937 | 1.0734704 | 0.28307011 | 0.999976173 | 0.163918249 |
| YEL014C | YEL014C | 0.15269937 | 1.07470636 | 0.28251622 | 0.999976173 | 0.16410698 |
| YER181C | YER181C | 0.15269937 | 1.0752148 | 0.28228859 | 0.999976173 | 0.164184617 |
| YLR390W-A | CCW14 | 0.15269937 | 1.07528651 | 0.28225649 | 0.999976173 | 0.164195568 |
| YBL016W | FUS3 | 0.15269937 | 1.07606423 | 0.28190855 | 0.999976173 | 0.164314326 |
| YOR344C | TYE7 | 0.15269937 | 1.07631961 | 0.28179437 | 0.999976173 | 0.164353321 |
| YML060W | OGG1 | 0.15269937 | 1.07661748 | 0.28166121 | 0.999976173 | 0.164398807 |
| YMR087W | YMR087W | 0.15269937 | 1.07757905 | 0.28123169 | 0.999976173 | 0.164545637 |
| YLR455W | YLR455W | 0.10797476 | 1.52425375 | 0.12745754 | 0.999976173 | 0.164580928 |
| YFR012W | YFR012W | 0.15269937 | 1.07856462 | 0.2807919 | 0.999976173 | 0.164696134 |
| YGL255W | ZRT1 | 0.15269937 | 1.07881156 | 0.28068178 | 0.999976173 | 0.164733842 |
| YKR097W | PCK1 | 0.15269937 | 1.0788838 | 0.28064957 | 0.999976173 | 0.164744873 |
| YBR245C | ISW1 | 0.15269937 | 1.08118869 | 0.27962325 | 0.999976173 | 0.165096828 |
| YGR239C | PEX21 | 0.15269937 | 1.08401536 | 0.27836808 | 0.999976173 | 0.165528457 |
| YBR159W | IFA38 | 0.15269937 | 1.08832873 | 0.27646013 | 0.999976173 | 0.166187108 |
| YCL042W | YCL042W | 0.15269937 | 1.08957815 | 0.27590915 | 0.999976173 | 0.166377893 |

| | | | | | | |
|---|---|---|---|---|---|---|
| YHR015W | MIP6 | 0.15269937 | 1.09067484 | 0.27542613 | 0.999976173 | 0.166545356 |
| YHR061C | GIC1 | 0.15269937 | 1.09122524 | 0.27518393 | 0.999976173 | 0.166629403 |
| YBL027W | RPL19B | 0.15269937 | 1.09419178 | 0.27388105 | 0.999976173 | 0.167082391 |
| YDL096C | OPI6 | 0.15269937 | 1.09677293 | 0.27275086 | 0.999976173 | 0.16747653 |
| YKL199C | NA | 0.15269937 | 1.09745046 | 0.27245472 | 0.999976173 | 0.16757999 |
| YHL043W | ECM34 | 0.15269937 | 1.09769357 | 0.27234852 | 0.999976173 | 0.167617112 |
| YPR160W | GPH1 | 0.15269937 | 1.09801828 | 0.27220671 | 0.999976173 | 0.167666695 |
| YDR445C | YDR445C | 0.15269937 | 1.09827732 | 0.27209362 | 0.999976173 | 0.16770625 |
| YKL213C | DOA1 | 0.15269937 | 1.09850863 | 0.27199266 | 0.999976173 | 0.167741571 |
| YOR327C | SNC2 | 0.15269937 | 1.09882494 | 0.27185464 | 0.999976173 | 0.167789872 |
| YLR443W | ECM7 | 0.15269937 | 1.09967687 | 0.27148315 | 0.999976173 | 0.167919961 |
| YBR242W | YBR242W | 0.15269937 | 1.09982334 | 0.27141932 | 0.999976173 | 0.167942327 |
| YCR081W | SRB8 | 0.15269937 | 1.10079446 | 0.27099635 | 0.999976173 | 0.168090616 |
| YDR486C | VPS60 | 0.15269937 | 1.10455667 | 0.26936199 | 0.999976173 | 0.168665103 |
| YLR421C | RPN13 | 0.15269937 | 1.10601708 | 0.2687294 | 0.999976173 | 0.168888106 |
| YHL002W | HSE1 | 0.15269937 | 1.10720016 | 0.26821768 | 0.999976173 | 0.169068763 |
| YKR096W | YKR096W | 0.15269937 | 1.10881678 | 0.26751952 | 0.999976173 | 0.16931562 |
| YOR109W | INP53 | 0.15269937 | 1.11150481 | 0.26636144 | 0.999976173 | 0.16972608 |
| YOR276W | CAF20 | 0.15269937 | 1.112544 | 0.26591465 | 0.999976173 | 0.169884763 |
| YOR312C | RPL20B | 0.15269937 | 1.11274187 | 0.26582963 | 0.999976173 | 0.169914978 |
| YIL042C | PKP1 | 0.15269937 | 1.11277141 | 0.26581694 | 0.999976173 | 0.169919488 |
| YNR013C | PHO91 | 0.15269937 | 1.11325382 | 0.26560976 | 0.999976173 | 0.169993152 |
| YJR003C | YJR003C | 0.15269937 | 1.11449043 | 0.26507919 | 0.999976173 | 0.170181981 |
| YOL091W | SPO21 | 0.15269937 | 1.11629528 | 0.26430611 | 0.999976173 | 0.170457581 |
| YBR263W | SHM1 | 0.15269937 | 1.11807472 | 0.26354545 | 0.999976173 | 0.170729301 |
| YPR051W | MAK3 | 0.15269937 | 1.12201119 | 0.26186808 | 0.999976173 | 0.171330398 |
| YLL005C | SPO75 | 0.15269937 | 1.12212688 | 0.2618189 | 0.999976173 | 0.171348063 |
| YIL141W | YIL141W | 0.15269937 | 1.12279835 | 0.26153355 | 0.999976173 | 0.171450597 |
| YOR158W | PET123 | 0.15269937 | 1.12423687 | 0.26092297 | 0.999976173 | 0.171670257 |
| YOR339C | UBC11 | 0.15269937 | 1.12730302 | 0.25962483 | 0.999976173 | 0.172138456 |
| YLR343W | GAS2 | 0.15269937 | 1.12757608 | 0.25950944 | 0.999976173 | 0.172180152 |
| YOR345C | YOR345C | 0.15269937 | 1.12762126 | 0.25949035 | 0.999976173 | 0.172187051 |
| YIL028W | YIL028W | 0.15269937 | 1.12853408 | 0.25910488 | 0.999976173 | 0.172326439 |
| YMR145C | NDE1 | 0.15269937 | 1.13026638 | 0.25837446 | 0.999976173 | 0.172590959 |
| YIL134W | FLX1 | 0.15269937 | 1.13054309 | 0.25825792 | 0.999976173 | 0.172633213 |
| YGR110W | CLD1 | 0.15269937 | 1.13462645 | 0.25654236 | 0.999976173 | 0.173256739 |
| YBR082C | UBC4 | 0.10797476 | 1.60523956 | 0.10845328 | 0.999976173 | 0.173325351 |
| YJL042W | MHP1 | 0.15269937 | 1.13689163 | 0.25559411 | 0.999976173 | 0.17360263 |
| YML112W | CTK3 | 0.15269937 | 1.13809963 | 0.25508941 | 0.999976173 | 0.173787092 |
| YLR059C | REX2 | 0.15269937 | 1.1382616 | 0.25502179 | 0.999976173 | 0.173811825 |
| YOR386W | PHR1 | 0.15269937 | 1.1386247 | 0.25487025 | 0.999976173 | 0.173867269 |
| YPR157W | YPR157W | 0.15269937 | 1.13893938 | 0.25473897 | 0.999976173 | 0.173915321 |
| YGL209W | MIG2 | 0.15269937 | 1.14358925 | 0.25280459 | 0.999976173 | 0.174625353 |
| YJL078C | PRY3 | 0.15269937 | 1.14409827 | 0.25259346 | 0.999976173 | 0.17470308 |
| YHL026C | YHL026C | 0.15269937 | 1.14420745 | 0.25254819 | 0.999976173 | 0.174719751 |
| YJL163C | YJL163C | 0.15269937 | 1.14450725 | 0.25242391 | 0.999976173 | 0.174765531 |
| YBL098W | BNA4 | 0.15269937 | 1.14681493 | 0.25146871 | 0.999976173 | 0.175117913 |
| YNL170W | YNL170W | 0.15269937 | 1.14953717 | 0.25034516 | 0.999976173 | 0.175533596 |
| YHR108W | GGA2 | 0.15269937 | 1.15093175 | 0.24977094 | 0.999976173 | 0.175746549 |
| YGR089W | NNF2 | 0.15269937 | 1.15136291 | 0.24959359 | 0.999976173 | 0.175812386 |
| YOR129C | AFI1 | 0.15269937 | 1.15242518 | 0.24915703 | 0.999976173 | 0.175974594 |
| YKR034W | DAL80 | 0.15269937 | 1.15252887 | 0.24911445 | 0.999976173 | 0.175990427 |
| YDR345C | HXT3 | 0.15269937 | 1.15327666 | 0.24880749 | 0.999976173 | 0.176104615 |
| YOR325W | YOR325W | 0.15269937 | 1.15563765 | 0.24784006 | 0.999976173 | 0.176465136 |
| YMR137C | PSO2 | 0.15269937 | 1.15620473 | 0.24760809 | 0.999976173 | 0.176551729 |
| YOR267C | HRK1 | 0.15269937 | 1.1584749 | 0.24668097 | 0.999976173 | 0.176898383 |
| YIL008W | URM1 | 0.15269937 | 1.16054335 | 0.24583835 | 0.999976173 | 0.177214233 |
| YJL029C | VPS53 | 0.15269937 | 1.16099431 | 0.24565491 | 0.999976173 | 0.177283095 |
| YDL133C-A | RPL41B | 0.15269937 | 1.16809325 | 0.2427799 | 0.999976173 | 0.178367099 |
| YKR104W | YKR104W | 0.15269937 | 1.16861932 | 0.24256779 | 0.999976173 | 0.17844743 |
| YBL086C | YBL086C | 0.15269937 | 1.17014675 | 0.24195268 | 0.999976173 | 0.178680667 |
| YNL155W | YNL155W | 0.15269937 | 1.17271855 | 0.24091947 | 0.999976173 | 0.179073378 |
| YOL155C | HPF1 | 0.15269937 | 1.1733688 | 0.24065873 | 0.999976173 | 0.179172672 |
| YHL014C | YLF2 | 0.15269937 | 1.17363836 | 0.2405507 | 0.999976173 | 0.179213834 |

| | | | | | | |
|---|---|---|---|---|---|---|
| YPL172C | COX10 | 0.15269937 | 1.17374073 | 0.24050968 | 0.999976173 | 0.179229466 |
| YLR366W | YLR366W | 0.15269937 | 1.17564069 | 0.23974929 | 0.999976173 | 0.179519588 |
| YDR435C | PPM1 | 0.15269937 | 1.17825291 | 0.23870662 | 0.999976173 | 0.179918472 |
| YPL101W | ELP4 | 0.15269937 | 1.17907851 | 0.23837774 | 0.999976173 | 0.18004454 |
| YBR144C | YBR144C | 0.15269937 | 1.18235754 | 0.23707472 | 0.999976173 | 0.180545246 |
| YGR038W | ORM1 | 0.15269937 | 1.18710686 | 0.23519636 | 0.999976173 | 0.181270465 |
| YDR084C | TVP23 | 0.15269937 | 1.18813209 | 0.23479227 | 0.999976173 | 0.181427016 |
| YLR169W | YLR169W | 0.15269937 | 1.18847168 | 0.23465853 | 0.999976173 | 0.181478871 |
| YOL101C | IZH4 | 0.15269937 | 1.18918515 | 0.23437771 | 0.999976173 | 0.181587819 |
| YKR036C | CAF4 | 0.15269937 | 1.18943839 | 0.2342781 | 0.999976173 | 0.181626488 |
| YFL043C | NA | 0.15269937 | 1.1906869 | 0.23378743 | 0.999976173 | 0.181817134 |
| YLR445W | YLR445W | 0.15269937 | 1.19134926 | 0.23352742 | 0.999976173 | 0.181918276 |
| YDR463W | STP1 | 0.15269937 | 1.19206513 | 0.23324663 | 0.999976173 | 0.182027589 |
| YPL220W | RPL1A | 0.15269937 | 1.19296426 | 0.2328943 | 0.999976173 | 0.182164885 |
| YNR059W | MNT4 | 0.15269937 | 1.19452375 | 0.23228409 | 0.999976173 | 0.18240302 |
| YDR092W | UBC13 | 0.15269937 | 1.19578703 | 0.23179062 | 0.999976173 | 0.182595921 |
| YOR376W | YOR376W | 0.15269937 | 1.1965988 | 0.23147392 | 0.999976173 | 0.182719878 |
| YOR253W | NAT5 | 0.15269937 | 1.19927402 | 0.23043238 | 0.999976173 | 0.183128382 |
| YKR020W | VPS51 | 0.15269937 | 1.1997095 | 0.23026315 | 0.999976173 | 0.18319488 |
| YNL096C | RPS7B | 0.15269937 | 1.20136174 | 0.22962189 | 0.999976173 | 0.183447177 |
| YDR305C | HNT2 | 0.15269937 | 1.2014086 | 0.22960372 | 0.999976173 | 0.183454331 |
| YBR095C | RXT2 | 0.10797476 | 1.69989572 | 0.08916254 | 0.999976173 | 0.183545828 |
| YNL050C | YNL050C | 0.15269937 | 1.20964203 | 0.22642732 | 0.999976173 | 0.184711571 |
| YDR024W | FYV1 | 0.15269937 | 1.21040615 | 0.22613412 | 0.999976173 | 0.184828252 |
| YBL066C | SEF1 | 0.15269937 | 1.2121177 | 0.22547838 | 0.999976173 | 0.185089605 |
| YER131W | RPS26B | 0.15269937 | 1.21570359 | 0.22410893 | 0.999976173 | 0.185637168 |
| YPL057C | SUR1 | 0.15269937 | 1.21608349 | 0.2239642 | 0.999976173 | 0.185695178 |
| YPL150W | YPL150W | 0.15269937 | 1.21612227 | 0.22394943 | 0.999976173 | 0.1857011 |
| YDL222C | FMP45 | 0.15269937 | 1.21654591 | 0.22378812 | 0.999976173 | 0.185765789 |
| YBL060W | YEL1 | 0.15269937 | 1.21667407 | 0.22373933 | 0.999976173 | 0.185785358 |
| YOL151W | GRE2 | 0.15269937 | 1.21760376 | 0.22338567 | 0.999976173 | 0.185927322 |
| YFR025C | HIS2 | 0.15269937 | 1.22014956 | 0.22241929 | 0.999976173 | 0.186316065 |
| YKR095W | MLP1 | 0.15269937 | 1.22141607 | 0.22193964 | 0.999976173 | 0.18650946 |
| YLR227C | ADY4 | 0.15269937 | 1.22642548 | 0.22004975 | 0.999976173 | 0.187274393 |
| YPL224C | MMT2 | 0.15269937 | 1.22935249 | 0.21895084 | 0.999976173 | 0.187721346 |
| YBR023C | CHS3 | 0.15269937 | 1.23105626 | 0.218313 | 0.999976173 | 0.18798151 |
| YLR312C | YLR312C | 0.15269937 | 1.23632637 | 0.21634848 | 0.999976173 | 0.188786252 |
| YER001W | MNN1 | 0.15269937 | 1.23721894 | 0.21601703 | 0.999976173 | 0.188922547 |
| YKL222C | YKL222C | 0.15269937 | 1.23842171 | 0.21557096 | 0.999976173 | 0.189106209 |
| YHL010C | ETP1 | 0.15269937 | 1.23960963 | 0.21513105 | 0.999976173 | 0.189287604 |
| YNL196C | SLZ1 | 0.15269937 | 1.24046431 | 0.21481494 | 0.999976173 | 0.189418113 |
| YPL087W | YDC1 | 0.15269937 | 1.24081189 | 0.21468648 | 0.999976173 | 0.189471188 |
| YNL122C | YNL122C | 0.15269937 | 1.24163522 | 0.21438242 | 0.999976173 | 0.189596911 |
| YKL220C | FRE2 | 0.15269937 | 1.24283344 | 0.21394047 | 0.999976173 | 0.189779877 |
| YKL076C | PSY1 | 0.15269937 | 1.2438546 | 0.21356433 | 0.999976173 | 0.189935809 |
| YPL086C | ELP3 | 0.15269937 | 1.24558906 | 0.21292656 | 0.999976173 | 0.19020066 |
| YBR241C | YBR241C | 0.15269937 | 1.24632231 | 0.21265736 | 0.999976173 | 0.190312626 |
| YLL020C | YLL020C | 0.15269937 | 1.24654248 | 0.21257657 | 0.999976173 | 0.190346247 |
| YDR080W | VPS41 | 0.15269937 | 1.25016671 | 0.21124995 | 0.999976173 | 0.190899663 |
| YIL166C | YIL166C | 0.15269937 | 1.25179759 | 0.21065493 | 0.999976173 | 0.191148699 |
| YDL077C | VAM6 | 0.15269937 | 1.25309678 | 0.2101818 | 0.999976173 | 0.191347083 |
| YKL110C | KTI12 | 0.15269937 | 1.25412594 | 0.20980755 | 0.999976173 | 0.191504235 |
| YPR068C | HOS1 | 0.15269937 | 1.25550726 | 0.209306 | 0.999976173 | 0.191715163 |
| YLR108C | YLR108C | 0.15269937 | 1.25564701 | 0.2092553 | 0.999976173 | 0.191736502 |
| YNR025C | YNR025C | 0.15269937 | 1.25754616 | 0.20856727 | 0.999976173 | 0.192026502 |
| YLR446W | YLR446W | 0.15269937 | 1.25768148 | 0.20851831 | 0.999976173 | 0.192047164 |
| YLR431C | ATG23 | 0.15269937 | 1.2611496 | 0.20726631 | 0.999976173 | 0.192576744 |
| YNR005C | YNR005C | 0.15269937 | 1.26425898 | 0.20614846 | 0.999976173 | 0.193051544 |
| YGL063W | PUS2 | 0.15269937 | 1.26463129 | 0.20601491 | 0.999976173 | 0.193108395 |
| YLL048C | YBT1 | 0.15269937 | 1.2667235 | 0.20526556 | 0.999976173 | 0.193427875 |
| YML117W-A | NA | 0.15269937 | 1.26957318 | 0.20424811 | 0.999976173 | 0.193863019 |
| YOR343C | YOR343C | 0.15269937 | 1.26998785 | 0.20410037 | 0.999976173 | 0.193926339 |
| YML001W | YPT7 | 0.15269937 | 1.27214911 | 0.20333156 | 0.999976173 | 0.194256362 |
| YGR107W | YGR107W | 0.15269937 | 1.27444912 | 0.20251572 | 0.999976173 | 0.194607573 |

| | | | | | | |
|---|---|---|---|---|---|---|
| YNL223W | ATG4 | 0.15269937 | 1.2802209 | 0.20047892 | 0.999976173 | 0.195488919 |
| YKL129C | MYO3 | 0.15269937 | 1.28031205 | 0.20044687 | 0.999976173 | 0.195502838 |
| YJR091C | JSN1 | 0.15269937 | 1.28091919 | 0.20023352 | 0.999976173 | 0.195595548 |
| YPL107W | YPL107W | 0.15269937 | 1.28274021 | 0.19959458 | 0.999976173 | 0.195873616 |
| YCR048W | ARE1 | 0.15269937 | 1.28301754 | 0.19949741 | 0.999976173 | 0.195915965 |
| YBR275C | 22 | 0.15269937 | 1.28558524 | 0.19859934 | 0.999976173 | 0.196308051 |
| YPL004C | LSP1 | 0.15269937 | 1.29355783 | 0.19582973 | 0.999976173 | 0.197525461 |
| YKR028W | SAP190 | 0.15269937 | 1.29784292 | 0.19435287 | 0.999976173 | 0.198179792 |
| YNL066W | SUN4 | 0.15269937 | 1.30117954 | 0.19320858 | 0.999976173 | 0.198689291 |
| YNL057W | YNL057W | 0.15269937 | 1.30724519 | 0.19114105 | 0.999976173 | 0.199615511 |
| YNL023C | FAP1 | 0.15269937 | 1.31086106 | 0.18991633 | 0.999976173 | 0.200167653 |
| YIL133C | RPL16A | 0.15269937 | 1.31109647 | 0.18983679 | 0.999976173 | 0.200203599 |
| YMR092C | AIP1 | 0.15269937 | 1.31177751 | 0.18960684 | 0.999976173 | 0.200307594 |
| YNL154C | YCK2 | 0.15269937 | 1.31361952 | 0.18898591 | 0.999976173 | 0.200588867 |
| YDR483W | KRE2 | 0.10797476 | 1.86599593 | 0.06205323 | 0.999976173 | 0.201480458 |
| YHR171W | ATG7 | 0.15269937 | 1.32055906 | 0.18666009 | 0.999976173 | 0.201648532 |
| YOR089C | VPS21 | 0.15269937 | 1.32640802 | 0.18471627 | 0.999976173 | 0.202541664 |
| YPL179W | PPQ1 | 0.15269937 | 1.32701004 | 0.18451706 | 0.999976173 | 0.202633592 |
| YBR228W | SLX1 | 0.15269937 | 1.33477505 | 0.18196171 | 0.999976173 | 0.203819304 |
| YOL092W | YOL092W | 0.15269937 | 1.33635235 | 0.18144587 | 0.999976173 | 0.204060156 |
| YPR129W | SCD6 | 0.15269937 | 1.33922875 | 0.18050796 | 0.999976173 | 0.204499381 |
| YBR229C | ROT2 | 0.15269937 | 1.34008898 | 0.18022817 | 0.999976173 | 0.204630737 |
| YMR313C | TGL3 | 0.15269937 | 1.34031888 | 0.18015345 | 0.999976173 | 0.204665843 |
| YLR360W | VPS38 | 0.15269937 | 1.34063372 | 0.18005116 | 0.999976173 | 0.204713919 |
| YER055C | HIS1 | 0.15269937 | 1.34351215 | 0.17911796 | 0.999976173 | 0.205153453 |
| YDR368W | YPR1 | 0.15269937 | 1.34791908 | 0.1776962 | 0.999976173 | 0.205826389 |
| YHL007C | STE20 | 0.15269937 | 1.35218019 | 0.17632948 | 0.999976173 | 0.206477057 |
| YDL104C | QRI7 | 0.15269937 | 1.35603249 | 0.17510065 | 0.999976173 | 0.207065301 |
| YLR450W | HMG2 | 0.15269937 | 1.35759446 | 0.17460423 | 0.999976173 | 0.207303813 |
| YKR091W | SRL3 | 0.15269937 | 1.35874471 | 0.17423933 | 0.999976173 | 0.207479456 |
| YPR009W | SUT2 | 0.15269937 | 1.35909272 | 0.17412904 | 0.999976173 | 0.207532596 |
| YNL119W | NCS2 | 0.15269937 | 1.36173856 | 0.17329225 | 0.999976173 | 0.207936614 |
| YPL177C | CUP9 | 0.15269937 | 1.37275849 | 0.16983932 | 0.999976173 | 0.20961935 |
| YIL077C | YIL077C | 0.15269937 | 1.37490615 | 0.16917243 | 0.999976173 | 0.209947297 |
| YDL239C | ADY3 | 0.15269937 | 1.37512376 | 0.16910497 | 0.999976173 | 0.209980526 |
| YBL051C | PIN4 | 0.15269937 | 1.37832734 | 0.16811415 | 0.999976173 | 0.210469711 |
| YNL092W | YNL092W | 0.15269937 | 1.37965299 | 0.16770542 | 0.999976173 | 0.210672137 |
| YLR146C | SPE4 | 0.15269937 | 1.37981333 | 0.16765603 | 0.999976173 | 0.210696621 |
| YPR054W | SMK1 | 0.15269937 | 1.38135415 | 0.16718201 | 0.999976173 | 0.210931903 |
| YOR268C | YOR268C | 0.15269937 | 1.38142673 | 0.1671597 | 0.999976173 | 0.210942986 |
| YFL042C | YFL042C | 0.15269937 | 1.38155872 | 0.16711915 | 0.999976173 | 0.210963141 |
| YCR033W | SNT1 | 0.15269937 | 1.38494123 | 0.16608236 | 0.999976173 | 0.211479647 |
| YMR155W | YMR155W | 0.15269937 | 1.39278084 | 0.16369803 | 0.999976173 | 0.212676751 |
| YNR075W | COS10 | 0.15269937 | 1.39456744 | 0.16315828 | 0.999976173 | 0.212949564 |
| YIL015W | BAR1 | 0.15269937 | 1.39466039 | 0.16313023 | 0.999976173 | 0.212963757 |
| YLR131C | ACE2 | 0.15269937 | 1.39755465 | 0.16225881 | 0.999976173 | 0.213405708 |
| YNL089C | YNL089C | 0.15269937 | 1.39818435 | 0.16206968 | 0.999976173 | 0.213501864 |
| YIR016W | YIR016W | 0.15269937 | 1.40033305 | 0.16142558 | 0.999976173 | 0.213829969 |
| YER098W | UBP9 | 0.15269937 | 1.40046129 | 0.1613872 | 0.999976173 | 0.21384955 |
| YLR042C | YLR042C | 0.15269937 | 1.40117952 | 0.16117238 | 0.999976173 | 0.213959224 |
| YPL137C | GIP3 | 0.15269937 | 1.40525816 | 0.15995652 | 0.999976173 | 0.21458203 |
| YGR093W | YGR093W | 0.15269937 | 1.40720279 | 0.15937926 | 0.999976173 | 0.214878973 |
| YNL010W | YNL010W | 0.15269937 | 1.41115506 | 0.1582109 | 0.999976173 | 0.215482483 |
| YLL042C | ATG10 | 0.15269937 | 1.41152605 | 0.15810157 | 0.999976173 | 0.215539133 |
| YML021C | UNG1 | 0.15269937 | 1.41311156 | 0.15763494 | 0.999976173 | 0.215781239 |
| YOL137W | BSC6 | 0.15269937 | 1.41738674 | 0.15638191 | 0.999976173 | 0.216434056 |
| YOR054C | VHS3 | 0.15269937 | 1.419768 | 0.15568727 | 0.999976173 | 0.216797673 |
| YKR098C | UBP11 | 0.15269937 | 1.42083257 | 0.15537747 | 0.999976173 | 0.216960232 |
| YOR298C-A | MBF1 | 0.15269937 | 1.42507998 | 0.15414613 | 0.999976173 | 0.217608809 |
| YOR357C | SNX3 | 0.15269937 | 1.42875483 | 0.15308677 | 0.999976173 | 0.218169956 |
| YDR388W | RVS167 | 0.15269937 | 1.43480844 | 0.15135376 | 0.999976173 | 0.219094339 |
| YAL002W | VPS8 | 0.15269937 | 1.43585398 | 0.15105597 | 0.999976173 | 0.219253992 |
| YAR040C | NA | 0.15269937 | 1.44236817 | 0.14921063 | 0.999976173 | 0.220248704 |
| YDR509W | YDR509W | 0.15269937 | 1.45111294 | 0.14676052 | 0.999976173 | 0.221584025 |

| | | | | | | |
|---|---|---|---|---|---|---|
| YIL152W | YIL152W | 0.15269937 | 1.45658595 | 0.14524283 | 0.999976173 | 0.222419752 |
| YLR361C | DCR2 | 0.15269937 | 1.459255 | 0.14450706 | 0.999976173 | 0.222827313 |
| YDL188C | PPH22 | 0.15269937 | 1.46334039 | 0.14338639 | 0.999976173 | 0.223451149 |
| YML050W | AIM32 | 0.15269937 | 1.46898902 | 0.14184791 | 0.999976173 | 0.224313692 |
| YDL190C | UFD2 | 0.15269937 | 1.4699224 | 0.14159491 | 0.999976173 | 0.224456219 |
| YDR269C | YDR269C | 0.15269937 | 1.47169041 | 0.14111664 | 0.999976173 | 0.224726193 |
| YML013C-A | NA | 0.15269937 | 1.47414758 | 0.14045401 | 0.999976173 | 0.225101401 |
| YLR098C | CHA4 | 0.15269937 | 1.47599492 | 0.13995741 | 0.999976173 | 0.225383488 |
| YOL119C | MCH4 | 0.15269937 | 1.47612897 | 0.13992143 | 0.999976173 | 0.225403958 |
| YNL065W | AQR1 | 0.15269937 | 1.47738696 | 0.13958409 | 0.999976173 | 0.225596052 |
| YLR346C | YLR346C | 0.15269937 | 1.47832387 | 0.13933327 | 0.999976173 | 0.225739118 |
| YHL013C | OTU2 | 0.15269937 | 1.47918007 | 0.13910435 | 0.999976173 | 0.225869859 |
| YIL137C | TMA108 | 0.15269937 | 1.48100631 | 0.13861705 | 0.999976173 | 0.226148725 |
| YJL215C | YJL215C | 0.15269937 | 1.48161195 | 0.13845574 | 0.999976173 | 0.226241206 |
| YNL093W | YPT53 | 0.15269937 | 1.48241051 | 0.13824326 | 0.999976173 | 0.226363145 |
| YPL102C | YPL102C | 0.15269937 | 1.48782567 | 0.13680904 | 0.999976173 | 0.227190036 |
| YLR261C | VPS63 | 0.15269937 | 1.49319978 | 0.13539708 | 0.999976173 | 0.22801066 |
| YMR163C | INP2 | 0.15269937 | 1.49377129 | 0.13524758 | 0.999976173 | 0.228097929 |
| YFL020C | PAU5 | 0.15269937 | 1.49583251 | 0.13470949 | 0.999976173 | 0.228412676 |
| YPL033C | SRL4 | 0.15269937 | 1.49777499 | 0.1342039 | 0.999976173 | 0.228709291 |
| YLR454W | FMP27 | 0.15269937 | 1.50272082 | 0.13292324 | 0.999976173 | 0.229464516 |
| YIL145C | PAN6 | 0.15269937 | 1.50440241 | 0.13248997 | 0.999976173 | 0.229721293 |
| YLL046C | RNP1 | 0.15269937 | 1.5085051 | 0.1314375 | 0.999976173 | 0.230347772 |
| YDL110C | TMA17 | 0.15269937 | 1.51135285 | 0.13071077 | 0.999976173 | 0.230782622 |
| YJR095W | SFC1 | 0.15269937 | 1.51846466 | 0.1289095 | 0.999976173 | 0.231868591 |
| YLR119W | SRN2 | 0.15269937 | 1.5214469 | 0.12815993 | 0.999976173 | 0.232323977 |
| YIL053W | RHR2 | 0.15269937 | 1.52148182 | 0.12815117 | 0.999976173 | 0.232329309 |
| YIL168W | YIL168W | 0.15269937 | 1.52870912 | 0.12634878 | 0.999976173 | 0.233432913 |
| YPR185W | ATG13 | 0.15269937 | 1.53308013 | 0.12526832 | 0.999976173 | 0.234100363 |
| YER062C | HOR2 | 0.15269937 | 1.5353446 | 0.12471141 | 0.999976173 | 0.234446147 |
| YMR139W | RIM11 | 0.15269937 | 1.53713886 | 0.12427152 | 0.999976173 | 0.23472013 |
| YPL139C | UME1 | 0.15269937 | 1.53775543 | 0.12412064 | 0.999976173 | 0.234814278 |
| YDR051C | DET1 | 0.15269937 | 1.53809829 | 0.1240368 | 0.999976173 | 0.234866634 |
| YLR386W | VAC14 | 0.15269937 | 1.5423955 | 0.12298973 | 0.999976173 | 0.235522815 |
| YPR173C | VPS4 | 0.15269937 | 1.54343405 | 0.12273771 | 0.999976173 | 0.235681401 |
| YER056C | FCY2 | 0.15269937 | 1.55954708 | 0.11887916 | 0.999976173 | 0.238141851 |
| YDL206W | YDL206W | 0.15269937 | 1.5607388 | 0.11859761 | 0.999976173 | 0.238323824 |
| YBR084C-A | RPL19A | 0.15269937 | 1.56177784 | 0.11835255 | 0.999976173 | 0.238482486 |
| YGL080W | FMP37 | 0.15269937 | 1.5643037 | 0.11775848 | 0.999976173 | 0.238868182 |
| YPL265W | DIP5 | 0.15269937 | 1.56767641 | 0.11696888 | 0.999976173 | 0.239383193 |
| YHR209W | CRG1 | 0.15269937 | 1.5725008 | 0.11584666 | 0.999976173 | 0.240119875 |
| YKR030W | GMH1 | 0.15269937 | 1.57496241 | 0.11527733 | 0.999976173 | 0.240495762 |
| YBR230C | OM14 | 0.15269937 | 1.57832491 | 0.11450319 | 0.999976173 | 0.241009213 |
| YPL080C | YPL080C | 0.15269937 | 1.58791796 | 0.11231709 | 0.999976173 | 0.242474065 |
| YMR138W | CIN4 | 0.15269937 | 1.59421322 | 0.11090049 | 0.999976173 | 0.243435347 |
| YIL073C | SPO22 | 0.15269937 | 1.59494081 | 0.11073767 | 0.999976173 | 0.243546451 |
| YIL086C | YIL086C | 0.15269937 | 1.59532415 | 0.11065196 | 0.999976173 | 0.243604987 |
| YOR220W | RCN2 | 0.15269937 | 1.59705009 | 0.11026674 | 0.999976173 | 0.243868537 |
| YIL052C | RPL34B | 0.15269937 | 1.60060586 | 0.10947644 | 0.999976173 | 0.2444115 |
| YDR049W | YDR049W | 0.15269937 | 1.60398076 | 0.10873048 | 0.999976173 | 0.244926845 |
| YPR040W | TIP41 | 0.15269937 | 1.60864836 | 0.10770544 | 0.999976173 | 0.245639585 |
| YBR077C | SLM4 | 0.15269937 | 1.61463004 | 0.10640301 | 0.999976173 | 0.246552983 |
| YPL072W | UBP16 | 0.15269937 | 1.61712345 | 0.10586381 | 0.999976173 | 0.246933725 |
| YPL166W | ATG29 | 0.15269937 | 1.62910956 | 0.10330198 | 0.999976173 | 0.248763997 |
| YBR261C | TAE1 | 0.15269937 | 1.62957974 | 0.10320251 | 0.999976173 | 0.248835793 |
| YPR201W | ARR3 | 0.15269937 | 1.63358588 | 0.10235798 | 0.999976173 | 0.249447528 |
| YLR434C | YLR434C | 0.15269937 | 1.6348269 | 0.10209749 | 0.999976173 | 0.24963703 |
| YNL028W | YNL028W | 0.15269937 | 1.63532958 | 0.10199212 | 0.999976173 | 0.24971379 |
| YOR364W | YOR364W | 0.15269937 | 1.6353822 | 0.1019811 | 0.999976173 | 0.249721826 |
| YKR040C | YKR040C | 0.15269937 | 1.63876279 | 0.10127481 | 0.999976173 | 0.250238038 |
| YCL075W | YCL075W | 0.15269937 | 1.64998207 | 0.09895872 | 0.999976173 | 0.251951216 |
| YLR448W | RPL6B | 0.15269937 | 1.65306945 | 0.09832885 | 0.999976173 | 0.252422657 |
| YIL049W | DFG10 | 0.15269937 | 1.65740649 | 0.09744943 | 0.999976173 | 0.253084921 |
| YNR062C | YNR062C | 0.15269937 | 1.67301921 | 0.09433559 | 0.999976173 | 0.255468973 |

| | | | | | | |
|---|---|---|---|---|---|---|
| YGL121C | GPG1 | 0.15269937 | 1.67738604 | 0.09347908 | 0.999976173 | 0.256135784 |
| YDR506C | YDR506C | 0.15269937 | 1.67994328 | 0.09298041 | 0.999976173 | 0.256526274 |
| YGR261C | APL6 | 0.15269937 | 1.69430439 | 0.09021948 | 0.999976173 | 0.258719206 |
| YML027W | YOX1 | 0.15269937 | 1.7081354 | 0.0876232 | 0.999976173 | 0.260831193 |
| YML036W | CGI121 | 0.15269937 | 1.71210128 | 0.08688997 | 0.999976173 | 0.26143678 |
| YPR132W | RPS23B | 0.15269937 | 1.72145261 | 0.08518066 | 0.999976173 | 0.262864721 |
| YKL216W | URA1 | 0.15269937 | 1.72444255 | 0.08463991 | 0.999976173 | 0.263321283 |
| YPL079W | RPL21B | 0.15269937 | 1.72553443 | 0.08444313 | 0.999976173 | 0.263488013 |
| YNR074C | AIF1 | 0.15269937 | 1.72744887 | 0.084099 | 0.999976173 | 0.263780347 |
| YOR124C | UBP2 | 0.15269937 | 1.73277838 | 0.08314697 | 0.999976173 | 0.26459416 |
| YPL019C | VTC3 | 0.15269937 | 1.74015319 | 0.081844 | 0.999976173 | 0.265720288 |
| YLR093C | NYV1 | 0.15269937 | 1.74642413 | 0.08074913 | 0.999976173 | 0.266677857 |
| YNR029C | YNR029C | 0.15269937 | 1.75508482 | 0.07925662 | 0.999976173 | 0.268000339 |
| YLR095C | IOC2 | 0.15269937 | 1.75700027 | 0.07892957 | 0.999976173 | 0.268292828 |
| YPR130C | YPR130C | 0.15269937 | 1.7587573 | 0.07863054 | 0.999976173 | 0.268561124 |
| YDL100C | GET3 | 0.15269937 | 1.75986495 | 0.07844251 | 0.999976173 | 0.268730262 |
| YLR015W | BRE2 | 0.15269937 | 1.78084364 | 0.07494971 | 0.999976173 | 0.271933694 |
| YCR032W | BPH1 | 0.15269937 | 1.78253626 | 0.07467353 | 0.999976173 | 0.272192157 |
| YER095W | RAD51 | 0.15269937 | 1.78280155 | 0.07463032 | 0.999976173 | 0.272232667 |
| YLL040C | VPS13 | 0.15269937 | 1.78392485 | 0.07444758 | 0.999976173 | 0.272404193 |
| YNL208W | YNL208W | 0.15269937 | 1.78534507 | 0.07421706 | 0.999976173 | 0.27262106 |
| YPR170C | YPR170C | 0.15269937 | 1.79417353 | 0.07279713 | 0.999976173 | 0.273969161 |
| YBL047C | EDE1 | 0.15269937 | 1.79456535 | 0.07273463 | 0.999976173 | 0.274028991 |
| YPL054W | LEE1 | 0.15269937 | 1.80505143 | 0.07107824 | 0.999976173 | 0.275630209 |
| YPL264C | YPL264C | 0.15269937 | 1.80817374 | 0.07059106 | 0.999976173 | 0.276106983 |
| YPL036W | PMA2 | 0.15269937 | 1.81020141 | 0.07027614 | 0.999976173 | 0.276416608 |
| YNL105W | RRT16 | 0.15269937 | 1.81062414 | 0.07021063 | 0.999976173 | 0.276481157 |
| YLR449W | FPR4 | 0.15269937 | 1.83276747 | 0.06684863 | 0.999976173 | 0.27986243 |
| YKR100C | SKG1 | 0.15269937 | 1.83970486 | 0.06582301 | 0.999976173 | 0.280921765 |
| YIL064W | SEE1 | 0.15269937 | 1.84267017 | 0.0653886 | 0.999976173 | 0.281374566 |
| YJL139C | YUR1 | 0.15269937 | 1.85291171 | 0.06390638 | 0.999976173 | 0.282938443 |
| YCR020C | PET18 | 0.15269937 | 1.85716195 | 0.06329946 | 0.999976173 | 0.283587453 |
| YOL110W | SHR5 | 0.15269937 | 1.86357389 | 0.06239287 | 0.999976173 | 0.284566552 |
| YML068W | ITT1 | 0.15269937 | 1.8717359 | 0.06125441 | 0.999976173 | 0.285812885 |
| YGL035C | MIG1 | 0.15269937 | 1.87485195 | 0.06082433 | 0.999976173 | 0.286288704 |
| YLR394W | CST9 | 0.15269937 | 1.91731904 | 0.0552084 | 0.999976173 | 0.292773401 |
| YOR175C | ALE1 | 0.15269937 | 1.92267429 | 0.05453192 | 0.999976173 | 0.293591144 |
| YNL237W | YTP1 | 0.15269937 | 1.9264045 | 0.05406481 | 0.999976173 | 0.294160745 |
| YLR014C | PPR1 | 0.15269937 | 1.93569015 | 0.05291653 | 0.999976173 | 0.295578658 |
| YBL007C | SLA1 | 0.15269937 | 1.93844134 | 0.05258025 | 0.999976173 | 0.295998763 |
| YOR375C | GDH1 | 0.15269937 | 1.93847649 | 0.05257596 | 0.999976173 | 0.296004131 |
| YNL143C | YNL143C | 0.15269937 | 1.94522945 | 0.05175817 | 0.999976173 | 0.297035303 |
| YJR039W | YJR039W | 0.15269937 | 1.95624325 | 0.05044723 | 0.999976173 | 0.298717104 |
| YLR214W | FRE1 | 0.15269937 | 1.96051505 | 0.04994632 | 0.999976173 | 0.299369405 |
| YLR246W | ERF2 | 0.15269937 | 1.96207207 | 0.04976478 | 0.999976173 | 0.299607161 |
| YJL164C | TPK1 | 0.15269937 | 1.97338022 | 0.04846287 | 0.999976173 | 0.301333908 |
| YNL015W | PBI2 | 0.15269937 | 1.98933842 | 0.04667436 | 0.999976173 | 0.303770715 |
| YOR106W | VAM3 | 0.15269937 | 1.99257302 | 0.0463187 | 0.999976173 | 0.304264637 |
| YLR393W | ATP10 | 0.15269937 | 1.99459669 | 0.04609734 | 0.999976173 | 0.304573649 |
| YLR224W | YLR224W | 0.15269937 | 2.00897255 | 0.0445504 | 0.999976173 | 0.306768835 |
| YCL022C | YCL022C | 0.15269937 | 2.02648159 | 0.04272568 | 0.999976173 | 0.309442453 |
| YCR007C | YCR007C | 0.15269937 | 2.02870608 | 0.04249844 | 0.999976173 | 0.309782132 |
| YPL120W | VPS30 | 0.15269937 | 2.03153865 | 0.04221057 | 0.999976173 | 0.310214663 |
| YGL087C | MMS2 | 0.15269937 | 2.03920034 | 0.04144016 | 0.999976173 | 0.311384599 |
| YPR127W | YPR127W | 0.15269937 | 2.04043473 | 0.04131716 | 0.999976173 | 0.31157309 |
| YER162C | RAD4 | 0.15269937 | 2.05905292 | 0.03949914 | 0.999976173 | 0.314416075 |
| YDR522C | SPS2 | 0.15269937 | 2.09481136 | 0.03619745 | 0.999976173 | 0.319876366 |
| YDL171C | GLT1 | 0.15269937 | 2.110417 | 0.03483201 | 0.999976173 | 0.322259337 |
| YPR050C | YPR050C | 0.15269937 | 2.11430037 | 0.03449915 | 0.999976173 | 0.322852326 |
| YPR037C | ERV2 | 0.15269937 | 2.14473972 | 0.03198293 | 0.999976173 | 0.327500395 |
| YOL093W | TRM10 | 0.15269937 | 2.16077297 | 0.03072202 | 0.999976173 | 0.329948662 |
| YNR047W | FPK1 | 0.15269937 | 2.16451054 | 0.03043429 | 0.999976173 | 0.330519387 |
| YJL160C | YJL160C | 0.15269937 | 2.17620451 | 0.02954897 | 0.999976173 | 0.332305048 |
| YLR254C | NDL1 | 0.15269937 | 2.17763989 | 0.02944185 | 0.999976173 | 0.33252423 |

| | | | | | | |
|---|---|---|---|---|---|---|
| YDR508C | GNP1 | 0.15269937 | 2.18281361 | 0.02905848 | 0.999976173 | 0.333314254 |
| YJL182C | YJL182C | 0.15269937 | 2.19425489 | 0.02822594 | 0.999976173 | 0.335061331 |
| YJL024C | APS3 | 0.15269937 | 2.21031382 | 0.0270921 | 0.999976173 | 0.337513519 |
| YBR227C | MCX1 | 0.15269937 | 2.2316008 | 0.02564988 | 0.999976173 | 0.340764027 |
| YLR154C | RNH203 | 0.15269937 | 2.27064847 | 0.02317644 | 0.999976173 | 0.346726582 |
| YNL040W | YNL040W | 0.15269937 | 2.27175552 | 0.02310944 | 0.999976173 | 0.346895627 |
| YDR025W | RPS11A | 0.15269937 | 2.32878648 | 0.01987803 | 0.999976173 | 0.355604219 |
| YBR288C | APM3 | 0.15269937 | 2.36989738 | 0.0178003 | 0.999976173 | 0.361881827 |
| YGR068C | ART5 | 0.15269937 | 2.37854426 | 0.01738835 | 0.999976173 | 0.363202201 |
| YNR006W | VPS27 | 0.15269937 | 2.44780083 | 0.0143797 | 0.999976173 | 0.373777634 |
| YJL172W | CPS1 | 0.15269937 | 2.48244072 | 0.01305486 | 0.999976173 | 0.379067123 |
| YMR275C | BUL1 | 0.15269937 | 2.54071566 | 0.01106837 | 0.999976173 | 0.38796567 |
| YPL195W | APL5 | 0.15269937 | 2.57843926 | 0.00993024 | 0.999976173 | 0.393726039 |
| YBL089W | AVT5 | 0.15269937 | 2.6169345 | 0.00887749 | 0.999976173 | 0.399604239 |
| YGR223C | HSV2 | 0.15269937 | 2.6494372 | 0.00806749 | 0.999976173 | 0.40456738 |
| YIL154C | IMP2' | 0.15269937 | 2.69233532 | 0.00709992 | 0.999976173 | 0.411117896 |
| YDR029W | YDR029W | 0.15269937 | 2.69300438 | 0.00708569 | 0.999976173 | 0.411220061 |
| YNL179C | YNL179C | 0.15269937 | 2.72789779 | 0.00637823 | 0.999976173 | 0.416548263 |
| YMR140W | SIP5 | 0.15269937 | 2.79838604 | 0.00513966 | 0.999976173 | 0.427311774 |
| YBL024W | NCL1 | 0.15269937 | 2.83689068 | 0.00455906 | 0.999976173 | 0.433191408 |
| YIL153W | RRD1 | 0.15269937 | 2.86039761 | 0.00423448 | 0.999976173 | 0.436780901 |
| YKL204W | EAP1 | 0.15269937 | 3.00853934 | 0.00262759 | 0.805356193 | 0.459402049 |
| YPL090C | RPS6A | 0.15269937 | 3.83011535 | 0.00012839 | 0.068862439 | 0.584856185 |

**Table S6  Fitness on H202**

**Key**

| | |
|---|---|
| ORF: | Open reading frame Y-number for *yfg* deletion |
| Gene: | Standard human-readable gene name for *yfg* deletion |
| P: | p-value for the significance of difference between observed mean fitness of treated strains and predicted fitness estimated from mean fitness observed on CSM |
| Q: | False discovery rate corrected p-value (correcting for multiple testing) |
| FD: | Fitness differential (difference between observed mean fitness of treated strains and predicted fitness estimated from mean fitness observed on control media) |
| Mean_$H_2O_2$: | Mean fitness for all replicate strains following treatment with 1.5mM $H_2O_2$ |
| Mean_CSM: | Mean fitness for all replicate strains grown on CSM |

**Supplementary Table 6: $H_2O_2$ Screen Data**

| ORF | Gene | P | Q | FD | Mean_$H_2O_2$ | Mean_CSM |
|---|---|---|---|---|---|---|
| YHR206W | SKN7 | 0.0003758 | 0.33960437 | -1.8583709 | 14.36071281 | 249.422762 |
| YJL121C | RPE1 | 0.00070477 | 0.33960437 | -1.7533416 | 0 | 213.7510919 |
| YPL091W | GLR1 | 0.01798241 | 0.87709187 | -1.4473525 | 27.1586233 | 219.6941392 |
| YJL120W | YJL120W | 0.00879452 | 0.84351098 | -1.3645607 | 0 | 166.354538 |
| YIR037W | HYR1 | 0.10349205 | 0.94753174 | -1.3237546 | 44.08714622 | 231.5826031 |
| YLR192C | HCR1 | 0.0028167 | 0.60474514 | -1.2451255 | 0 | 151.794114 |
| YML035C | AMD1 | 0.00087443 | 0.33960437 | -1.2078607 | 10.3087833 | 163.666462 |
| YIL128W | MET18 | 0.00126521 | 0.38805922 | -1.1850011 | 25.04466963 | 184.3445149 |
| YML007W | YAP1 | 0.09825232 | 0.94753174 | -1.1701398 | 38.54852392 | 204.035818 |
| YDL100C | GET3 | 0.04375869 | 0.94753174 | -1.1575011 | 46.99573623 | 215.9460503 |
| YDR269C | YDR269C | 2.47E-09 | 1.06E-05 | -1.1136107 | 40.84455805 | 200.8004379 |
| YCR033W | SNT1 | 0.05217858 | 0.94753174 | -1.0816454 | 35.6345171 | 188.6072369 |
| YHR008C | SOD2 | 0.12880147 | 0.97616733 | -1.0643454 | 39.37042258 | 192.4471055 |
| YER145C | FTR1 | 0.02940256 | 0.94753174 | -1.0325037 | 58.2034747 | 218.5543238 |
| YNL021W | HDA1 | 0.03668724 | 0.94753174 | -0.9734507 | 37.12657798 | 177.7930487 |
| YEL013W | VAC8 | 0.13968762 | 0.97853666 | -0.954402 | 45.55983561 | 188.8996222 |
| YMR058W | FET3 | 0.02976978 | 0.94753174 | -0.951908 | 52.88369399 | 200.2578195 |
| YBR073W | RDH54 | 0.14780907 | 0.98328895 | -0.9469926 | 55.45914337 | 203.7596337 |
| YJR091C | JSN1 | 0.07753075 | 0.94753174 | -0.937046 | 73.0509972 | 230.5596636 |
| YIL007C | NAS2 | 0.05740251 | 0.94753174 | -0.9284039 | 42.22353147 | 180.4175701 |
| YER083C | GET2 | 0.05646554 | 0.94753174 | -0.9255558 | 23.4866881 | 150.2344908 |
| YBR275C | RIF1 | 0.14196431 | 0.97853666 | -0.9251534 | 58.21359936 | 205.4832966 |
| YLR452C | SST2 | 0.11035807 | 0.95531631 | -0.9174378 | 40.74111838 | 176.7201417 |
| YBL008W | HIR1 | 0.09677952 | 0.94753174 | -0.9167064 | 60.75212271 | 208.4957697 |
| YIL152W | YIL152W | 0.07102707 | 0.94753174 | -0.9061142 | 70.31915985 | 222.4386685 |
| YDR375C | BCS1 | 0.04507219 | 0.94753174 | -0.9044374 | 27.96345952 | 154.7885794 |
| YHR022C | YHR022C | 0.16135253 | 0.99381655 | -0.8972631 | 60.85484656 | 206.2889972 |
| YNL067W | RPL9B | 0.02767822 | 0.94311309 | -0.886581 | 45.32905898 | 180.2640336 |
| YDR455C | :::GUK1 | 0.12790413 | 0.97616733 | -0.8698116 | 40.70566332 | 170.8575468 |
| YDL020C | RPN4 | 0.17466975 | 0.99917121 | -0.8591948 | 43.74245467 | 174.3989141 |
| YCL075W | YCL075W | 0.05057824 | 0.94753174 | -0.8481476 | 84.95247566 | 238.6734734 |
| YNL096C | RPS7B | 0.03008711 | 0.94753174 | -0.8452241 | 52.86446801 | 187.2212957 |
| YLR218C | YLR218C | 0.07505711 | 0.94753174 | -0.8445559 | 0 | 102.9604009 |
| YPR074C | TKL1 | 0.18089028 | 0.99917121 | -0.8426283 | 54.30218837 | 189.1942143 |
| YGR089W | NNF2 | 0.09872635 | 0.94753174 | -0.8396108 | 47.13473588 | 177.4131694 |
| YJR129C | YJR129C | 0.15731702 | 0.98919321 | -0.8361148 | 56.75369435 | 192.3038356 |
| YCR009C | RVS161 | 0.0875426 | 0.94753174 | -0.8350695 | 12.10025281 | 121.0718966 |
| YLR056W | ERG3 | 0.05238737 | 0.94753174 | -0.830271 | 52.71514092 | 185.1605721 |
| YIL132C | CSM2 | 0.10945257 | 0.95531631 | -0.8268757 | 69.05887856 | 210.7718209 |
| YDR074W | TPS2 | 0.22866804 | 0.99917121 | -0.8211216 | 57.61107393 | 191.8412722 |
| YIL145C | PAN6 | 0.10242326 | 0.94753174 | -0.8121873 | 72.14367986 | 213.8932713 |
| YIL098C | FMC1 | 0.07927136 | 0.94753174 | -0.8104725 | 64.89285028 | 202.1382678 |
| YHR111W | UBA4 | 0.07223279 | 0.94753174 | -0.8073207 | 53.05655361 | 182.9063424 |
| YHR178W | STB5 | 0.24502603 | 0.99917121 | -0.8029864 | 48.63359667 | 175.3349887 |
| YCR071C | IMG2 | 0.05997623 | 0.94753174 | -0.7964617 | 0 | 97.09719338 |
| YNL307C | MCK1 | 0.07511641 | 0.94753174 | -0.7948517 | 40.08336165 | 160.728206 |
| YJL192C | SOP4 | 0.13175257 | 0.97853666 | -0.7852689 | 73.99748406 | 213.5635632 |

| | | | | | | |
|---|---|---|---|---|---|---|
| YGR209C | TRX2 | 0.21340062 | 0.99917121 | -0.765607 | 68.65372148 | 202.6573487 |
| YER131W | RPS26B | 0.08740654 | 0.94753174 | -0.7544988 | 40.9014377 | 157.111428 |
| YER042W | MXR1 | 0.10071832 | 0.94753174 | -0.7510458 | 68.70326701 | 200.9610777 |
| YKL212W | SAC1 | 0.00102815 | 0.33960437 | -0.7484526 | 12.34426913 | 110.9009355 |
| YHL022C | SPO11 | 0.17437284 | 0.99917121 | -0.7423319 | 74.73111434 | 209.4972943 |
| YDR207C | UME6 | 0.23966959 | 0.99917121 | -0.7363352 | 59.83782716 | 185.0506958 |
| YIL133C | RPL16A | 0.24570892 | 0.99917121 | -0.7289656 | 57.86581934 | 181.0121113 |
| YHR184W | SSP1 | 0.09683497 | 0.94753174 | -0.7258938 | 86.29848586 | 225.9127645 |
| YHR199C | FMP34 | 0.17889773 | 0.99917121 | -0.7151656 | 84.70036238 | 222.0600957 |
| YDR080W | VPS41 | 0.07067986 | 0.94753174 | -0.7077654 | 27.82724245 | 130.595251 |
| YJL206C | YJL206C | 0.01796964 | 0.87709187 | -0.7075313 | 95.90317723 | 238.9683481 |
| YHL041W | YHL041W | 0.1686027 | 0.99636201 | -0.6958387 | 81.65602926 | 214.8562479 |
| YKR010C | TOF2 | 0.08019322 | 0.94753174 | -0.6940641 | 57.77519385 | 176.6129455 |
| YIR023W | DAL81 | 0.00978005 | 0.84351098 | -0.6929762 | 80.93347137 | 213.3567053 |
| YBR114W | RAD16 | 0.13675082 | 0.97853666 | -0.687883 | 70.97820149 | 196.8833887 |
| YLR449W | FPR4 | 0.22532123 | 0.99917121 | -0.6810804 | 55.66805485 | 171.6747526 |
| YIL042C | PKP1 | 0.23838255 | 0.99917121 | -0.6791226 | 85.81054375 | 219.4338756 |
| YHR123W | EPT1 | 0.20734579 | 0.99917121 | -0.6723686 | 89.81126393 | 224.9810947 |
| YPL080C | YPL080C | 0.07930117 | 0.94753174 | -0.6705827 | 58.04229849 | 174.1756345 |
| YJL078C | PRY3 | 0.03662324 | 0.94753174 | -0.6689793 | 92.1088211 | 228.2264437 |
| YOR273C | TPO4 | 0.18742002 | 0.99917121 | -0.6672169 | 73.85804972 | 198.9497271 |
| YCL076W | YCL076W | 0.09384263 | 0.94753174 | -0.6661243 | 101.4459749 | 242.7465322 |
| YIL064W | YIL064W | 0.22477309 | 0.99917121 | -0.6644385 | 88.04004783 | 221.1939079 |
| YDR071C | PAA1 | 0.00665908 | 0.84351098 | -0.6625598 | 88.41004171 | 221.5540361 |
| YHL024W | RIM4 | 0.18181098 | 0.99917121 | -0.6581142 | 81.79120202 | 210.472474 |
| YIL154C | IMP2' | 0.19085136 | 0.99917121 | -0.6578333 | 73.03831343 | 196.5004507 |
| YIR016W | YIR016W | 0.03144488 | 0.94753174 | -0.6576321 | 91.12299032 | 225.2732964 |
| YNL078W | NIS1 | 0.14489304 | 0.98328895 | -0.6542162 | 72.30785857 | 194.8963445 |
| YPL088W | YPL088W | 0.19224775 | 0.99917121 | -0.6535295 | 82.19336241 | 210.5539384 |
| YJR069C | HAM1 | 0.19948038 | 0.99917121 | -0.6452173 | 88.28539708 | 219.2413265 |
| YOR271C | FSF1 | 0.16037657 | 0.99381655 | -0.6426569 | 76.82510267 | 200.6802274 |
| YNR006W | VPS27 | 0.07167004 | 0.94753174 | -0.6404237 | 42.27937235 | 145.3986288 |
| YOR196C | LIP5 | 0.0333211 | 0.94753174 | -0.6381754 | 14.78770161 | 101.347806 |
| YGL020C | GET1 | 0.07301376 | 0.94753174 | -0.6374152 | 99.03772063 | 235.4117725 |
| YBR009C | HHF1 | 0.18650903 | 0.99917121 | -0.6359151 | 73.73261676 | 194.9339753 |
| YHR125W | YHR125W | 0.2343149 | 0.99917121 | -0.6308644 | 74.71581926 | 195.8838537 |
| YLR242C | ARV1 | 0.08790105 | 0.94753174 | -0.6292942 | 56.28208227 | 166.3392248 |
| YMR312W | ELP6 | 0.1072003 | 0.94910942 | -0.6292733 | 47.26941215 | 151.9852263 |
| YDR273W | DON1 | 0.01243838 | 0.84351098 | -0.6270185 | 94.50409794 | 226.9251263 |
| YDL188C | PPH22 | 0.01408117 | 0.84351098 | -0.6230205 | 95.76039152 | 228.4382019 |
| YMR282C | AEP2 | 0.12852365 | 0.97616733 | -0.6229228 | 39.62289177 | 139.0349941 |
| YDL194W | SNF3 | 0.03485245 | 0.94753174 | -0.6198508 | 101.0337366 | 236.4488632 |
| YIL153W | RRD1 | 0.23950807 | 0.99917121 | -0.6195482 | 83.58049677 | 208.6200725 |
| YCR007C | YCR007C | 0.00689601 | 0.84351098 | -0.6159866 | 99.42394844 | 233.4144108 |
| YNL082W | PMS1 | 0.38161661 | 0.99917121 | -0.6153851 | 65.81250793 | 179.8194595 |
| YJL095W | BCK1 | 0.09684275 | 0.94753174 | -0.6104984 | 98.11539839 | 230.6616545 |
| YDR244W | PEX5 | 0.01497333 | 0.84351098 | -0.6032032 | 97.57746665 | 228.9157077 |
| YDL133C-A | RPL41B | 0.11765068 | 0.95851884 | -0.5964262 | 81.45955213 | 202.4239439 |
| YNL041C | COG6 | 0.01865434 | 0.87709187 | -0.5963124 | 76.61826787 | 194.7009809 |
| YBR100W | YBR100W | 0.18269053 | 0.99917121 | -0.5945398 | 70.90942586 | 185.3943319 |
| YDR277C | MTH1 | 0.02128727 | 0.87709187 | -0.5892392 | 102.2560025 | 234.6632735 |
| YDL104C | QRI7 | 0.33389609 | 0.99917121 | -0.5855532 | 32.89376712 | 123.7640231 |
| YNL241C | ZWF1 | 0.1411987 | 0.97853666 | -0.5847029 | 63.24762628 | 171.9947411 |
| YBL051C | PIN4 | 0.1555149 | 0.98492771 | -0.5831031 | 69.67438586 | 182.0334413 |
| YML028W | TSA1 | 0.00019952 | 0.28557955 | -0.5775158 | 74.51066313 | 189.0534012 |
| YNL320W | YNL320W | 0.01935868 | 0.87709187 | -0.5750504 | 93.18273935 | 218.4855831 |
| YBR023C | CHS3 | 0.01898457 | 0.87709187 | -0.5716711 | 103.5473672 | 234.5778542 |
| YIL110W | MNI1 | 0.18948766 | 0.99917121 | -0.5706018 | 61.31842141 | 167.2036692 |
| YJR095W | SFC1 | 0.18740307 | 0.99917121 | -0.570443 | 69.09706719 | 179.5707466 |
| YJR088C | YJR088C | 0.01881362 | 0.87709187 | -0.5695046 | 106.4393103 | 238.9187583 |
| YIL073C | SPO22 | 0.11158742 | 0.95531631 | -0.5660522 | 96.64587708 | 222.9031744 |
| YBL007C | SLA1 | 0.18135865 | 0.99917121 | -0.5654454 | 69.99709737 | 180.3946531 |
| YNL196C | YNL196C | 0.05835735 | 0.94753174 | -0.5647762 | 101.5343717 | 230.5318721 |
| YBR227C | MCX1 | 0.0207352 | 0.87709187 | -0.5629972 | 107.9609625 | 240.5484623 |

| | | | | | | |
|---|---|---|---|---|---|---|
| YBL098W | BNA4 | 0.29060034 | 0.99917121 | -0.562834 | 84.61840131 | 203.3587284 |
| YLR448W | RPL6B | 0.33739346 | 0.99917121 | -0.5615371 | 65.00372275 | 171.9669283 |
| YOR360C | PDE2 | 0.24067482 | 0.99917121 | -0.5595315 | 80.49825251 | 196.395347 |
| YDL222C | FMP45 | 0.01540826 | 0.84351098 | -0.5566131 | 101.4659167 | 229.4277074 |
| YML013C-A | YML013C-A | 0.21077704 | 0.99917121 | -0.5555768 | 42.24073897 | 134.9933595 |
| YJR058C | APS2 | 0.01061195 | 0.84351098 | -0.5501021 | 88.8179596 | 208.4938031 |
| YBR216C | YBP1 | 0.01060366 | 0.84351098 | -0.5497184 | 96.48595216 | 220.6572474 |
| YHR180W | YHR180W | 0.05205158 | 0.94753174 | -0.5487198 | 109.3117801 | 240.9588957 |
| YCR034W | FEN1 | 0.07767742 | 0.94753174 | -0.5476062 | 5.699267638 | 75.83435503 |
| YBR255W | MTC4 | 0.07164566 | 0.94753174 | -0.5474971 | 106.1114165 | 235.7136849 |
| YJL139C | YUR1 | 0.08393035 | 0.94753174 | -0.5460106 | 98.54727425 | 223.4876052 |
| YIL040W | APQ12 | 0.30725628 | 0.99917121 | -0.5454451 | 40.8741497 | 131.5820951 |
| YBR261C | YBR261C | 0.02613733 | 0.92955221 | -0.5439178 | 111.6662637 | 244.1226635 |
| YLL026W | HSP104 | 0.03581989 | 0.94753174 | -0.5415537 | 105.3557557 | 233.785841 |
| YHR117W | TOM71 | 0.14859478 | 0.98328895 | -0.5411378 | 97.42274263 | 221.1028932 |
| YKR055W | RHO4 | 0.31960211 | 0.99917121 | -0.5405388 | 75.3842217 | 185.9365424 |
| YJL212C | OPT1 | 0.01328899 | 0.84351098 | -0.5384601 | 94.42220952 | 215.9985121 |
| YML008C | ERG6 | 0.16649255 | 0.99381655 | -0.5310891 | 64.77179907 | 167.8856885 |
| YBL060W | YBL060W | 0.00956954 | 0.84351098 | -0.5289142 | 102.5084494 | 227.7110063 |
| YDR134C | YDR134C | 0.35119333 | 0.99917121 | -0.5272152 | 107.4437374 | 235.3626412 |
| YKL199C | YKT9 | 0.01700589 | 0.87709187 | -0.5269913 | 99.42689792 | 222.5696332 |
| YPR170C | YPR170C | 0.13563669 | 0.97853666 | -0.521229 | 81.6768736 | 193.6026521 |
| YDL190C | UFD2 | 0.15123565 | 0.98328895 | -0.5166868 | 104.1012185 | 228.7566169 |
| YDL219W | DTD1 | 0.04439853 | 0.94753174 | -0.5160385 | 114.6200523 | 245.4273851 |
| YBR224W | YBR224W | 0.05938374 | 0.94753174 | -0.5151964 | 97.79734398 | 218.5368622 |
| YDL110C | TMA17 | 0.05027965 | 0.94753174 | -0.5141239 | 108.9221664 | 236.1208686 |
| YGL226W | MTC3 | 0.04401176 | 0.94753174 | -0.5140536 | 83.21142333 | 195.1714625 |
| YHL040C | ARN1 | 0.40453991 | 0.99917121 | -0.5125142 | 105.5268465 | 230.5180572 |
| YHL044W | YHL044W | 0.05712334 | 0.94753174 | -0.5114617 | 115.8797551 | 246.8753335 |
| YDR305C | HNT2 | 0.08522192 | 0.94753174 | -0.5101411 | 88.31794051 | 202.825915 |
| YIR001C | SGN1 | 0.08099754 | 0.94753174 | -0.5077331 | 96.03249138 | 214.8167143 |
| YNL074C | MLF3 | 0.07432753 | 0.94753174 | -0.5073899 | 89.76439941 | 204.7937964 |
| YJR039W | YJR039W | 0.28967369 | 0.99917121 | -0.5053772 | 70.81898977 | 174.380458 |
| YHR005C | GPA1 | 0.08369203 | 0.94753174 | -0.4930355 | 95.91726085 | 212.8414368 |
| YGR110W | YGR110W | 0.06283689 | 0.94753174 | -0.4920113 | 93.37015795 | 208.6606636 |
| YNL010W | YNL010W | 0.02335503 | 0.88749104 | -0.491554 | 84.64422026 | 194.7100451 |
| YOL091W | SPO21 | 0.35645263 | 0.99917121 | -0.4902197 | 87.10661935 | 198.4684173 |
| YML050W | YML050W | 0.12145986 | 0.96405745 | -0.4887517 | 90.5365974 | 203.7512186 |
| YIL092W | YIL092W | 0.35985609 | 0.99917121 | -0.487121 | 100.7348655 | 219.7917788 |
| YJR137C | ECM17 | 0.15238199 | 0.98492771 | -0.4836013 | 81.93131519 | 189.4205985 |
| YNL089C | YNL089C | 0.04330069 | 0.94753174 | -0.4794188 | 82.37639517 | 189.6194399 |
| YCR102W-A | YCR102W-A | 0.03170541 | 0.94753174 | -0.4772309 | 108.8912183 | 231.5739417 |
| YNL085W | MKT1 | 0.0739876 | 0.94753174 | -0.4771853 | 97.65515078 | 213.6764835 |
| YLR193C | UPS1 | 0.18990401 | 0.99917121 | -0.4756561 | 61.53600037 | 155.9752384 |
| YLL042C | ATG10 | 0.0644221 | 0.94753174 | -0.4716536 | 103.875293 | 222.9068398 |
| YBR076W | ECM8 | 0.35517056 | 0.99917121 | -0.4709013 | 82.58517227 | 188.9135122 |
| YNL257C | SIP3 | 0.01281853 | 0.84351098 | -0.4693051 | 89.00302406 | 198.9384676 |
| YCR028C-A | RIM1 | 0.35306256 | 0.99917121 | -0.4676415 | 34.84008356 | 112.4885759 |
| YDR220C | YDR220C | 0.41525506 | 0.99917121 | -0.4667082 | 108.4427946 | 229.5770567 |
| YDL006W | PTC1 | 0.19307265 | 0.99917121 | -0.4649004 | 61.38696536 | 154.4266829 |
| YPL224C | MMT2 | 0.1606519 | 0.99381655 | -0.4647702 | 91.37991856 | 202.1705006 |
| YIL028W | YIL028W | 0.05395308 | 0.94753174 | -0.4645607 | 108.841013 | 229.9493701 |
| YOR125C | CAT5 | 0.46577614 | 0.99917121 | -0.4622542 | 60.13786742 | 152.11507 |
| YNL029C | KTR5 | 0.20606998 | 0.99917121 | -0.4615463 | 61.68252962 | 154.4884352 |
| YDR293C | SSD1 | 0.51913414 | 0.99917121 | -0.4610202 | 66.42756488 | 161.9801117 |
| YPL107W | YPL107W | 0.10211658 | 0.94753174 | -0.4602189 | 98.96567327 | 213.6949232 |
| YOL090W | MSH2 | 0.31019804 | 0.99917121 | -0.4581853 | 79.98165636 | 183.2175549 |
| YAL028W | FRT2 | 0.03423211 | 0.94753174 | -0.4571779 | 104.0883505 | 221.4813547 |
| YCR008W | SAT4 | 0.11293208 | 0.95531631 | -0.4569688 | 102.4058417 | 218.7766997 |
| YGR238C | KEL2 | 0.06796693 | 0.94753174 | -0.4560918 | 116.0255311 | 240.3572843 |
| YNR041C | COQ2 | 0.17872633 | 0.99917121 | -0.4548667 | 45.1634367 | 127.3697228 |
| YHL017W | YHL017W | 0.05494614 | 0.94753174 | -0.4532193 | 107.4760504 | 226.3932068 |
| YDL179W | PCL9 | 0.05482426 | 0.94753174 | -0.4522947 | 115.4786713 | 239.0235753 |
| YIL029C | YIL029C | 0.07057342 | 0.94753174 | -0.4513119 | 99.80521142 | 213.9459097 |

| | | | | | | |
|---|---|---|---|---|---|---|
| YHL029C | OCA5 | 0.11165577 | 0.95531631 | -0.4512929 | 109.1328004 | 228.7965133 |
| YJL213W | YJL213W | 0.07455744 | 0.94753174 | -0.451269 | 99.4464323 | 213.3693804 |
| YJL042W | MHP1 | 0.23593856 | 0.99917121 | -0.4508893 | 96.35392201 | 208.3986826 |
| YOR220W | WSP1 | 0.32157439 | 0.99917121 | -0.4494449 | 92.71343327 | 202.4256185 |
| YOR364W | YOR364W | 0.14147749 | 0.97853666 | -0.4485676 | 96.74825704 | 208.7435765 |
| YIL065C | FIS1 | 0.0660883 | 0.94753174 | -0.4483566 | 84.61595515 | 189.3988212 |
| YPL087W | YDC1 | 0.10416267 | 0.94753174 | -0.4473515 | 92.43418231 | 201.725741 |
| YJL007C | YJL007C | 0.07220625 | 0.94753174 | -0.4465694 | 104.5636916 | 220.9449863 |
| YCL006C | YCL006C | 0.06088368 | 0.94753174 | -0.4459385 | 119.5659552 | 244.7571199 |
| YIL052C | RPL34B | 0.15546822 | 0.98492771 | -0.4443128 | 94.15758835 | 204.0995747 |
| YNL093W | YPT53 | 0.08506509 | 0.94753174 | -0.4438226 | 92.63313685 | 201.6123357 |
| YJL160C | YJL160C | 0.08622293 | 0.94753174 | -0.4433253 | 107.0667581 | 224.5352803 |
| YGR071C | YGR071C | 0.01798153 | 0.87709187 | -0.4428454 | 85.69034582 | 190.4377595 |
| YDR058C | TGL2 | 0.21627788 | 0.99917121 | -0.4427677 | 97.81095952 | 209.728715 |
| YER176W | ECM32 | 0.27807496 | 0.99917121 | -0.4424425 | 84.85175599 | 189.0533074 |
| YNL107W | YAF9 | 0.04322625 | 0.94753174 | -0.4422905 | 86.40076473 | 191.5013596 |
| YBL069W | AST1 | 0.03878844 | 0.94753174 | -0.4422104 | 108.5357395 | 226.738522 |
| YNR029C | YNR029C | 0.19061351 | 0.99917121 | -0.4411375 | 83.23371811 | 186.3177035 |
| YBL102W | SFT2 | 0.03573595 | 0.94753174 | -0.4409948 | 113.5188893 | 234.5253098 |
| YIL168W | YIL168W | 0.12993773 | 0.97616733 | -0.4386067 | 94.73258232 | 204.3195421 |
| YNL065W | AQR1 | 0.05636609 | 0.94753174 | -0.4373728 | 89.45292489 | 195.7619891 |
| YOL095C | HMI1 | 0.28785092 | 0.99917121 | -0.4357496 | 34.34892252 | 107.8185013 |
| YJR126C | VPS70 | 0.0089362 | 0.84351098 | -0.4349457 | 89.20243267 | 195.0672217 |
| YNL291C | MID1 | 0.38880376 | 0.99917121 | -0.434231 | 85.82201825 | 189.5972397 |
| YNL201C | PSY2 | 0.10073456 | 0.94753174 | -0.434195 | 90.5398057 | 197.1052931 |
| YPL036W | PMA2 | 0.15067892 | 0.98328895 | -0.4321084 | 109.8028297 | 227.5246446 |
| YFR011C | YFR011C | 0.10152836 | 0.94753174 | -0.4319098 | 94.45803597 | 203.0659489 |
| YNL333W | SNZ2 | 0.0422696 | 0.94753174 | -0.4317163 | 96.57374413 | 206.4113287 |
| YHR108W | GGA2 | 0.36850431 | 0.99917121 | -0.430201 | 83.06078205 | 184.7090546 |
| YMR166C | YMR166C | 0.40450367 | 0.99917121 | -0.4285003 | 32.20945203 | 103.5279235 |
| YPL090C | RPS6A | 0.02384697 | 0.89823574 | -0.4240779 | 75.61192726 | 172.1013013 |
| YHR104W | GRE3 | 0.13733347 | 0.97853666 | -0.4221446 | 99.56815844 | 210.0126324 |
| YNL301C | RPL18B | 0.02252076 | 0.88145277 | -0.4208138 | 96.18534528 | 204.4637328 |
| YPL183C | YPL183C | 0.2929714 | 0.99917121 | -0.4202357 | 78.24167532 | 175.8204014 |
| YJL117W | PHO86 | 0.11421212 | 0.95531631 | -0.4170689 | 89.50235576 | 193.3654414 |
| YHR067W | HTD2 | 0.5900757 | 0.99917121 | -0.4162109 | 71.81446044 | 165.0952836 |
| YJL163C | YJL163C | 0.07480045 | 0.94753174 | -0.4153417 | 103.1451646 | 214.8791775 |
| YDR253C | MET32 | 0.04698214 | 0.94753174 | -0.4152015 | 109.5885343 | 225.1222781 |
| YHR121W | LSM12 | 0.15051927 | 0.98328895 | -0.4149161 | 101.0570578 | 211.5022753 |
| YJR094W-A | RPL43B | 0.0346909 | 0.94753174 | -0.4137445 | 83.11515969 | 182.789419 |
| YKR029C | SET3 | 0.05400979 | 0.94753174 | -0.4126228 | 80.98513783 | 179.2609067 |
| YCR081W | SRB8 | 0.24942142 | 0.99917121 | -0.4106474 | 44.68798321 | 121.2218141 |
| YNL119W | NCS2 | 0.04427615 | 0.94753174 | -0.4093407 | 67.77865998 | 157.831269 |
| YLR456W | YLR456W | 0.05865857 | 0.94753174 | -0.4090174 | 112.3338691 | 228.7399341 |
| YNR062C | YNR062C | 0.32174635 | 0.99917121 | -0.407673 | 104.2236322 | 215.6616014 |
| YHL037C | YHL037C | 0.06609748 | 0.94753174 | -0.4067758 | 102.2375 | 212.3895779 |
| YIL005W | EPS1 | 0.23056967 | 0.99917121 | -0.4066084 | 104.6093378 | 216.1459906 |
| YOR253W | NAT5 | 0.13913442 | 0.97853666 | -0.4058037 | 96.57218481 | 203.2498273 |
| YKL214C | YRA2 | 0.0514541 | 0.94753174 | -0.4049714 | 120.3133654 | 240.9529454 |
| YDR522C | SPS2 | 0.37205949 | 0.99917121 | -0.4041794 | 83.22163628 | 181.7928857 |
| YOR299W | BUD7 | 0.04297386 | 0.94753174 | -0.4040005 | 95.32827051 | 201.0492302 |
| YBR042C | YBR042C | 0.04551329 | 0.94753174 | -0.4038402 | 104.6574037 | 215.8850655 |
| YKL217W | JEN1 | 0.01454463 | 0.84351098 | -0.4038006 | 87.67897275 | 188.8444001 |
| YKR028W | SAP190 | 0.27344692 | 0.99917121 | -0.4037638 | 62.19326603 | 148.2574023 |
| YHL008C | YHL008C | 0.08749579 | 0.94753174 | -0.4033371 | 104.3185166 | 215.2840975 |
| YDR496C | PUF6 | 0.0009526 | 0.33960437 | -0.4023116 | 74.67979687 | 167.9634593 |
| YHR033W | YHR033W | 0.04986869 | 0.94753174 | -0.4022449 | 112.9082002 | 228.8288417 |
| YPL179W | PPQ1 | 0.0647191 | 0.94753174 | -0.4021045 | 82.40093308 | 180.2330756 |
| YJR073C | OPI3 | 0.05449465 | 0.94753174 | -0.4012142 | 74.41082314 | 167.4013776 |
| YOL151W | GRE2 | 0.11206032 | 0.95531631 | -0.4011574 | 100.4608281 | 208.8755188 |
| YFL034W | YFL034W | 0.47081979 | 0.99917121 | -0.3993452 | 103.0351629 | 212.7538743 |
| YKR003W | OSH6 | 0.02157577 | 0.87709187 | -0.399017 | 97.47119697 | 203.8540091 |
| YJL204C | RCY1 | 0.2632375 | 0.99917121 | -0.398458 | 57.57873959 | 140.2625705 |
| YHR031C | RRM3 | 0.11870323 | 0.96075443 | -0.3975429 | 98.45992514 | 205.2487131 |

| | | | | | | |
|---|---|---|---|---|---|---|
| YGR085C | RPL11B | 0.46607777 | 0.99917121 | -0.3970514 | 62.64473414 | 148.1579887 |
| YHR130C | YHR130C | 0.30342775 | 0.99917121 | -0.3967316 | 125.1308541 | 247.6196209 |
| YKR023W | YKR023W | 0.00531121 | 0.84351098 | -0.3966346 | 89.93887166 | 191.5693685 |
| YJL151C | SNA3 | 0.03310678 | 0.94753174 | -0.3963927 | 110.7509215 | 224.6802192 |
| YHR151C | MTC6 | 0.08985442 | 0.94753174 | -0.395647 | 108.7779911 | 221.4476886 |
| YOR106W | VAM3 | 0.51961117 | 0.99917121 | -0.3939272 | 43.89941557 | 117.9277664 |
| YHL009C | YAP3 | 0.1186825 | 0.96075443 | -0.3909948 | 114.3130651 | 229.6943932 |
| YJL071W | ARG2 | 0.08652643 | 0.94753174 | -0.3896567 | 111.402051 | 224.8958641 |
| YHL013C | OTU2 | 0.04197207 | 0.94753174 | -0.3880898 | 90.90997325 | 192.0740068 |
| YHR103W | SBE22 | 0.53405921 | 0.99917121 | -0.3870663 | 103.7570884 | 212.4065127 |
| YPR051W | MAK3 | 0.45886136 | 0.99917121 | -0.3869427 | 99.76690203 | 206.0376132 |
| YIL137C | TMA108 | 0.12725173 | 0.97616733 | -0.3855835 | 104.4356128 | 213.3061963 |
| YFR012W | YFR012W | 0.04349642 | 0.94753174 | -0.3853825 | 106.0833018 | 215.9054195 |
| YHL036W | MUP3 | 0.13968174 | 0.97853666 | -0.3851626 | 120.1084038 | 238.2116707 |
| YNR074C | AIF1 | 0.33720657 | 0.99917121 | -0.3832109 | 106.2447917 | 215.897828 |
| YHL028W | WSC4 | 0.08072775 | 0.94753174 | -0.3822252 | 121.9122281 | 240.7259138 |
| YJR083C | ACF4 | 0.25479259 | 0.99917121 | -0.3818935 | 121.6303981 | 240.2366964 |
| YGR248W | SOL4 | 0.30001649 | 0.99917121 | -0.3816084 | 104.7957623 | 213.3950821 |
| YIR033W | MGA2 | 0.34217761 | 0.99917121 | -0.3801147 | 103.3745605 | 210.9499117 |
| YHL019C | APM2 | 0.12676942 | 0.97616733 | -0.3800355 | 106.0101299 | 215.1370399 |
| YJL215C | YJL215C | 0.30040382 | 0.99917121 | -0.3774066 | 100.8520487 | 206.6030111 |
| YKR064W | OAF3 | 0.31677112 | 0.99917121 | -0.3763323 | 94.26936314 | 195.9900131 |
| YIL050W | PCL7 | 0.08912219 | 0.94753174 | -0.3763187 | 117.2859573 | 232.6391388 |
| YBR150C | TBS1 | 0.26438735 | 0.99917121 | -0.3751755 | 103.7867005 | 211.0040552 |
| YOR068C | VAM10 | 0.2735174 | 0.99917121 | -0.3750318 | 63.99127671 | 147.617751 |
| YDR274C | YDR274C | 0.1384534 | 0.97853666 | -0.3738782 | 105.9250686 | 214.2509529 |
| YNL077W | APJ1 | 0.05414475 | 0.94753174 | -0.3732616 | 94.05371409 | 195.272274 |
| YBR289W | SNF5 | 0.35842357 | 0.99917121 | -0.3714825 | 56.24016249 | 134.8424664 |
| YHR126C | YHR126C | 0.14027109 | 0.97853666 | -0.3699029 | 106.1732077 | 214.1614561 |
| YJL046W | YJL046W | 0.30611975 | 0.99917121 | -0.3678344 | 24.67759866 | 84.13864617 |
| YIL038C | NOT3 | 0.10613944 | 0.94753174 | -0.3675151 | 102.227959 | 207.5880801 |
| YHR198C | FMP22 | 0.00681718 | 0.84351098 | -0.3648177 | 104.5277901 | 210.9214121 |
| YML032C | RAD52 | 0.00081345 | 0.33960437 | -0.3647546 | 70.91961705 | 157.3972881 |
| YDR092W | UBC13 | 0.05464456 | 0.94753174 | -0.3645367 | 109.5836426 | 218.9378995 |
| YLR261C | YLR261C | 0.33516455 | 0.99917121 | -0.3644482 | 66.73460154 | 150.6958658 |
| YHL003C | LAG1 | 0.30766552 | 0.99917121 | -0.3637472 | 77.74132645 | 168.1371226 |
| YNL211C | YNL211C | 0.37732199 | 0.99917121 | -0.3617721 | 101.6933156 | 206.0366042 |
| YPR129W | SCD6 | 0.50534769 | 0.99917121 | -0.3602838 | 99.40479966 | 202.2110152 |
| YLR134W | PDC5 | 0.49785727 | 0.99917121 | -0.3597977 | 100.9849341 | 204.6679073 |
| YGR045C | YGR045C | 0.05094815 | 0.94753174 | -0.3588404 | 98.80125972 | 201.0739914 |
| YOR298C-A | MBF1 | 0.25075573 | 0.99917121 | -0.3584981 | 112.7824977 | 223.2954784 |
| YBR043C | QDR3 | 0.12567319 | 0.97616733 | -0.3580178 | 102.0772717 | 206.190311 |
| YPL046C | ELC1 | 0.13469809 | 0.97853666 | -0.3565232 | 97.61476365 | 198.9021646 |
| YBL066C | SEF1 | 0.12384764 | 0.97578308 | -0.3551084 | 111.946298 | 221.5507011 |
| YJL175W | YJL175W | 0.24280196 | 0.99917121 | -0.3542892 | 41.71961401 | 109.6244284 |
| YCL024W | KCC4 | 0.01199567 | 0.84351098 | -0.3535909 | 105.8856481 | 211.7149499 |
| YOR306C | MCH5 | 0.38451648 | 0.99917121 | -0.3532015 | 72.00165835 | 157.7118522 |
| YNR002C | ATO2 | 0.21360631 | 0.99917121 | -0.3523226 | 95.85881366 | 195.5939605 |
| YDR369C | XRS2 | 0.12963517 | 0.97616733 | -0.3516245 | 78.31796517 | 167.5774479 |
| YGR040W | KSS1 | 0.49886734 | 0.99917121 | -0.3502514 | 95.59887868 | 194.9275473 |
| YIL008W | URM1 | 0.44386118 | 0.99917121 | -0.3496369 | 92.73048515 | 190.2851029 |
| YNL259C | ATX1 | 0.05877941 | 0.94753174 | -0.3493949 | 101.5181179 | 204.2487162 |
| YDL171C | GLT1 | 0.06566533 | 0.94753174 | -0.3482235 | 111.8250421 | 220.5182787 |
| YHR171W | ATG7 | 0.21151473 | 0.99917121 | -0.3473334 | 97.02278747 | 196.8391944 |
| YER095W | RAD51 | 0.37400964 | 0.99917121 | -0.3468608 | 75.69816595 | 162.8250341 |
| YNL205C | YNL205C | 0.31346489 | 0.99917121 | -0.3464454 | 87.98189206 | 182.3345432 |
| YGR163W | GTR2 | 0.57850937 | 0.99917121 | -0.3433914 | 72.92268704 | 157.9824998 |
| YHR150W | PEX28 | 0.25362714 | 0.99917121 | -0.3430167 | 107.2990226 | 212.6764369 |
| YKR040C | YKR040C | 0.01370878 | 0.84351098 | -0.3423608 | 97.05504826 | 196.2843566 |
| YDR007W | TRP1 | 0.15488398 | 0.98492771 | -0.3415824 | 109.0636305 | 215.311477 |
| YFR030W | MET10 | 0.0898039 | 0.94753174 | -0.3409578 | 104.642348 | 208.1950449 |
| YMR087W | YMR087W | 0.00093589 | 0.33960437 | -0.3409335 | 93.33091697 | 190.180177 |
| YBL091C-A | SCS22 | 0.09327058 | 0.94753174 | -0.3400049 | 115.8898063 | 225.9889185 |
| YML021C | UNG1 | 0.39031766 | 0.99917121 | -0.3388555 | 72.30235516 | 156.4417333 |

| | | | | | | |
|---|---|---|---|---|---|---|
| YNL023C | FAP1 | 0.0881842 | 0.94753174 | -0.338496 | 89.11974289 | 183.1773029 |
| YLR443W | ECM7 | 0.01080485 | 0.84351098 | -0.3367525 | 93.35544109 | 189.7095242 |
| YPL033C | YPL033C | 0.29265794 | 0.99917121 | -0.3359209 | 109.3358757 | 215.0547974 |
| YKL220C | FRE2 | 0.27090171 | 0.99917121 | -0.3356919 | 104.7827602 | 207.776665 |
| YKR036C | CAF4 | 0.01564281 | 0.84351098 | -0.335491 | 95.18428244 | 192.4679092 |
| YKL222C | YKL222C | 0.19620494 | 0.99917121 | -0.333325 | 109.1993018 | 214.5208592 |
| YHR105W | YPT35 | 0.0929845 | 0.94753174 | -0.3294549 | 117.0957171 | 226.6230109 |
| YNL170W | YNL170W | 0.58647765 | 0.99917121 | -0.3285456 | 56.95180143 | 130.7411869 |
| YPL141C | YPL141C | 0.29537169 | 0.99917121 | -0.3278334 | 113.2097239 | 220.2374196 |
| YPR185W | ATG13 | 0.30769217 | 0.99917121 | -0.3277976 | 90.56226214 | 184.1700662 |
| YPR089W | YPR089W | 0.47740565 | 0.99917121 | -0.3270891 | 76.42514644 | 161.5722655 |
| YJL154C | VPS35 | 0.16715524 | 0.99551259 | -0.3263022 | 109.8882327 | 214.761723 |
| YNR075W | COS10 | 0.21244332 | 0.99917121 | -0.3255948 | 100.9868289 | 200.501215 |
| YIL086C | YIL086C | 0.20418186 | 0.99917121 | -0.3239705 | 106.6711945 | 209.3547751 |
| YNL058C | YNL058C | 0.02051496 | 0.87709187 | -0.3230412 | 81.49374853 | 169.1498369 |
| YDR270W | CCC2 | 0.10596234 | 0.94753174 | -0.3229975 | 130.7570724 | 247.5896281 |
| YAL005C | SSA1 | 0.30947797 | 0.99917121 | -0.3226425 | 99.50530101 | 197.7821762 |
| YHR001W-A | QCR10 | 0.22247834 | 0.99917121 | -0.3216633 | 111.6192959 | 216.9526736 |
| YGL132W | YGL132W | 0.09685052 | 0.94753174 | -0.3214925 | 107.4730596 | 210.3295469 |
| YIL041W | GVP36 | 0.09813679 | 0.94753174 | -0.3200176 | 111.1943364 | 216.0753647 |
| YNL183C | NPR1 | 0.32431982 | 0.99917121 | -0.3190826 | 125.3630837 | 238.5231632 |
| YOL113W | SKM1 | 0.18567575 | 0.99917121 | -0.318498 | 123.2922496 | 235.1543849 |
| YHR030C | SLT2 | 0.38824399 | 0.99917121 | -0.3145689 | 112.9511905 | 218.2086575 |
| YDL241W | YDL241W | 0.10440572 | 0.94753174 | -0.314508 | 114.0871495 | 220.0100958 |
| YNL105W | YNL105W | 0.26850882 | 0.99917121 | -0.3142788 | 86.89518731 | 176.6826685 |
| YPR160W | GPH1 | 0.05465717 | 0.94753174 | -0.3137167 | 80.54799609 | 166.5071052 |
| YIL015C-A | YIL015C-A | 0.21862376 | 0.99917121 | -0.3111615 | 102.0070596 | 200.366225 |
| YHL016C | DUR3 | 0.09321035 | 0.94753174 | -0.3107644 | 107.8320146 | 209.5932571 |
| YCR006C | YCR006C | 0.10503786 | 0.94753174 | -0.3100255 | 125.9325485 | 238.3258084 |
| YDR051C | YDR051C | 0.06783633 | 0.94753174 | -0.3093794 | 112.4628098 | 216.7983258 |
| YPL219W | PCL8 | 0.16028321 | 0.99381655 | -0.3091526 | 118.4432017 | 226.2936264 |
| YGR107W | YGR107W | 0.20482491 | 0.99917121 | -0.3082488 | 100.642145 | 197.8376902 |
| YMR163C | INP2 | 0.02333754 | 0.88749104 | -0.3073676 | 91.05669001 | 182.4667405 |
| YDR183W | PLP1 | 0.23243161 | 0.99917121 | -0.3071749 | 123.5011235 | 234.1065719 |
| YPR032W | SRO7 | 0.2835241 | 0.99917121 | -0.3057704 | 103.8771824 | 202.6869011 |
| YBL089W | AVT5 | 0.20030038 | 0.99917121 | -0.3056476 | 81.5145533 | 167.0625028 |
| YHR124W | NDT80 | 0.09800206 | 0.94753174 | -0.304827 | 112.9948079 | 217.0904734 |
| YDR205W | MSC2 | 0.03574912 | 0.94753174 | -0.3045248 | 123.3660291 | 233.5683753 |
| YJL182C | YJL182C | 0.37163208 | 0.99917121 | -0.303559 | 110.8365216 | 213.4991129 |
| YLR429W | CRN1 | 0.03733302 | 0.94753174 | -0.3034485 | 99.15882897 | 194.8905109 |
| YNL154C | YCK2 | 0.09784106 | 0.94753174 | -0.303257 | 79.65936493 | 163.8169344 |
| YOR225W | YOR225W | 0.21789575 | 0.99917121 | -0.3028663 | 113.4750695 | 217.6161871 |
| YER098W | UBP9 | 0.54131563 | 0.99917121 | -0.3027322 | 91.6007187 | 182.7679253 |
| YGR221C | TOS2 | 0.28517542 | 0.99917121 | -0.3018979 | 111.3649148 | 214.1379944 |
| YIL023C | YKE4 | 0.2358228 | 0.99917121 | -0.3017825 | 94.22775442 | 186.8353429 |
| YKL086W | SRX1 | 0.16985403 | 0.99636201 | -0.3004441 | 124.584995 | 235.0119394 |
| YJL153C | INO1 | 0.40676005 | 0.99917121 | -0.3000331 | 114.7474401 | 219.2968714 |
| YGR239C | PEX21 | 0.14982085 | 0.98328895 | -0.2994978 | 112.4960309 | 215.6465529 |
| YHL007C | STE20 | 0.16663901 | 0.99381655 | -0.29748 | 106.9196963 | 206.5210038 |
| YNL228W | YNL228W | 0.33782227 | 0.99917121 | -0.2951818 | 57.8378229 | 128.0846454 |
| YHR043C | DOG2 | 0.14803788 | 0.98328895 | -0.2951095 | 131.3463442 | 245.1281227 |
| YNL250W | RAD50 | 0.03869157 | 0.94753174 | -0.2946515 | 72.13783261 | 150.7908179 |
| YGL009C | LEU1 | 0.15045448 | 0.98328895 | -0.2945307 | 133.2698683 | 248.1205089 |
| YOR043W | WHI2 | 0.28608084 | 0.99917121 | -0.2937384 | 119.0458842 | 225.3741588 |
| YPR145W | ASN1 | 0.53792631 | 0.99917121 | -0.2929565 | 98.06500455 | 191.8696618 |
| YDR126W | SWF1 | 0.44667348 | 0.99917121 | -0.2926587 | 74.56732609 | 154.4165037 |
| YML006C | GIS4 | 0.02081469 | 0.87709187 | -0.292454 | 82.96875435 | 167.7696808 |
| YDR127W | ARO1 | 0.55517815 | 0.99917121 | -0.2919364 | 75.76261296 | 156.2317848 |
| YIL049W | DFG10 | 0.18222884 | 0.99917121 | -0.2908122 | 112.4948635 | 214.5858213 |
| YBL024W | NCL1 | 0.16189276 | 0.99381655 | -0.2907965 | 116.5779702 | 221.0857038 |
| YHR037W | PUT2 | 0.27636116 | 0.99917121 | -0.2881491 | 109.3103171 | 209.1902084 |
| YHR161C | YAP1801 | 0.2092212 | 0.99917121 | -0.2880052 | 120.7591335 | 227.4033389 |
| YNR047W | YNR047W | 0.41170982 | 0.99917121 | -0.2877706 | 90.91754579 | 179.8560849 |
| YGR087C | PDC6 | 0.41443398 | 0.99917121 | -0.2858633 | 111.0255421 | 211.6428089 |

| | | | | | | |
|---|---|---|---|---|---|---|
| YHL012W | YHL012W | 0.39896148 | 0.99917121 | -0.2857335 | 113.0723832 | 214.8862972 |
| YNL281W | HCH1 | 0.14231297 | 0.97931394 | -0.2854177 | 95.83721901 | 187.4031487 |
| YNR059W | MNT4 | 0.00162561 | 0.43627345 | -0.2848536 | 108.0589774 | 206.7958564 |
| YNR049C | MSO1 | 0.22323932 | 0.99917121 | -0.2836214 | 107.4593025 | 205.6907423 |
| YNL100W | YNL100W | 0.20760006 | 0.99917121 | -0.2834831 | 96.57062944 | 188.3351553 |
| YJL138C | TIF2 | 0.39983135 | 0.99917121 | -0.2826666 | 100.9398199 | 195.1929604 |
| YGR072W | UPF3 | 0.60324312 | 0.99917121 | -0.282292 | 99.63012082 | 193.0617674 |
| YNL227C | JJJ1 | 0.23009623 | 0.99917121 | -0.2822321 | 78.46327694 | 159.3491671 |
| YGL212W | VAM7 | 0.42709789 | 0.99917121 | -0.281921 | 25.34679447 | 74.73049039 |
| YGL216W | KIP3 | 0.14566687 | 0.98328895 | -0.2811316 | 104.2132058 | 200.2182503 |
| YOR275C | RIM20 | 0.32249644 | 0.99917121 | -0.2806012 | 111.5606352 | 211.8533637 |
| YLR227C | ADY4 | 0.49116952 | 0.99917121 | -0.2805618 | 110.586332 | 210.2971162 |
| YGR053C | YGR053C | 0.13298177 | 0.97853666 | -0.2800805 | 120.5723213 | 226.1397661 |
| YLR337C | VRP1 | 0.61140925 | 0.99917121 | -0.2798243 | 46.12344568 | 107.5588653 |
| YOR268C | YOR268C | 0.16947423 | 0.99636201 | -0.278724 | 115.1326055 | 217.3123902 |
| YDR014W | RAD61 | 0.24524588 | 0.99917121 | -0.2778194 | 108.2505019 | 206.2432968 |
| YNL234W | YNL234W | 0.1040399 | 0.94753174 | -0.2764784 | 94.89004114 | 184.8050989 |
| YHR210C | YHR210C | 0.18885422 | 0.99917121 | -0.2761221 | 110.2875486 | 209.2800885 |
| YLR338W | YLR338W | 0.40802813 | 0.99917121 | -0.2756611 | 64.12735013 | 135.7200875 |
| YOR327C | SNC2 | 0.40742611 | 0.99917121 | -0.2743336 | 112.9521846 | 213.305124 |
| YMR038C | CCS1 | 0.494142 | 0.99917121 | -0.2738696 | 78.17102181 | 157.8643125 |
| YKR030W | GMH1 | 0.15540863 | 0.98492771 | -0.2727122 | 99.17768997 | 191.1734597 |
| YKL216W | URA1 | 0.40319984 | 0.99917121 | -0.2725733 | 97.79014123 | 188.9470438 |
| YNL068C | FKH2 | 0.35392863 | 0.99917121 | -0.2720327 | 98.16271765 | 189.4744116 |
| YNL015W | PBI2 | 0.19417677 | 0.99917121 | -0.2709344 | 124.7964971 | 231.7511784 |
| YDR313C | PIB1 | 0.19049669 | 0.99917121 | -0.2702396 | 107.3808651 | 203.9344611 |
| YNL208W | YNL208W | 0.09321732 | 0.94753174 | -0.2701938 | 94.70556999 | 183.7452008 |
| YCR092C | MSH3 | 0.03926713 | 0.94753174 | -0.2700764 | 136.3181997 | 249.9933111 |
| YBR006W | UGA2 | 0.32996358 | 0.99917121 | -0.2699159 | 102.7224503 | 196.4771105 |
| YPL054W | LEE1 | 0.04694863 | 0.94753174 | -0.2696964 | 103.9517718 | 198.4078716 |
| YAR040C | YAR040C | 0.40936419 | 0.99917121 | -0.268338 | 105.745516 | 201.0985629 |
| YMR155W | YMR155W | 0.09600339 | 0.94753174 | -0.2674137 | 99.15397998 | 190.4897606 |
| YIL077C | YIL077C | 0.410574 | 0.99917121 | -0.2673259 | 111.1910192 | 209.6463982 |
| YGR055W | MUP1 | 0.60942207 | 0.99917121 | -0.2671613 | 47.1183878 | 107.5994141 |
| YPR044C | YPR044C | 0.62534482 | 0.99917121 | -0.2670087 | 59.22384004 | 126.8570965 |
| YGL255W | ZRT1 | 0.13927909 | 0.97853666 | -0.2666866 | 112.556005 | 211.7420121 |
| YIL166C | YIL166C | 0.04495132 | 0.94753174 | -0.2663921 | 118.0451395 | 220.4468031 |
| YML117W-A | YML117W-A | 0.13262544 | 0.97853666 | -0.2646408 | 87.88841296 | 172.2128327 |
| YOL155C | HPF1 | 0.33728864 | 0.99917121 | -0.2640029 | 107.3396688 | 203.108539 |
| YKR093W | PTR2 | 0.33390533 | 0.99917121 | -0.2635656 | 103.7166174 | 197.286017 |
| YJL185C | YJL185C | 0.18111685 | 0.99917121 | -0.2634832 | 135.0536182 | 247.1758649 |
| YOR317W | FAA1 | 0.20429384 | 0.99917121 | -0.2624618 | 115.9295719 | 216.598914 |
| YDR247W | VHS1 | 0.36394154 | 0.99917121 | -0.2623786 | 118.0087473 | 219.8995716 |
| YBL031W | SHE1 | 0.34765179 | 0.99917121 | -0.2615458 | 113.1302151 | 212.0296497 |
| YHL043W | ECM34 | 0.23684592 | 0.99917121 | -0.2613937 | 119.2246829 | 221.7157103 |
| YDR162C | NBP2 | 0.27227727 | 0.99917121 | -0.2605248 | 104.8054279 | 198.6490839 |
| YGR097W | ASK10 | 0.25232875 | 0.99917121 | -0.2597659 | 127.5912208 | 234.8398303 |
| YOL119C | MCH4 | 0.29065718 | 0.99917121 | -0.2595607 | 107.6160936 | 203.0071579 |
| YDR420W | HKR1 | 0.34901062 | 0.99917121 | -0.2595111 | 120.6835093 | 223.8091869 |
| YCR016W | YCR016W | 0.11059344 | 0.95531631 | -0.2586598 | 124.9376453 | 230.4795273 |
| YML036W | CGI121 | 0.12506504 | 0.97616733 | -0.2584656 | 101.2345226 | 192.71187 |
| YGL133W | ITC1 | 0.48565733 | 0.99917121 | -0.2567634 | 101.2374284 | 192.5089831 |
| YPR050C | YPR050C | 0.34170205 | 0.99917121 | -0.2551668 | 88.65626073 | 172.2805476 |
| YMR137C | PSO2 | 0.24324452 | 0.99917121 | -0.2551638 | 125.4262107 | 230.8313034 |
| YJL164C | TPK1 | 0.23462449 | 0.99917121 | -0.2550907 | 108.662229 | 204.1280385 |
| YHL033C | RPL8A | 0.58579233 | 0.99917121 | -0.2544734 | 103.4619602 | 195.7720722 |
| YML038C | YMD8 | 0.52943125 | 0.99917121 | -0.2542492 | 78.91516465 | 156.65732 |
| YOR128C | ADE2 | 0.64040338 | 0.99917121 | -0.2539474 | 102.0286553 | 193.4256054 |
| YMR253C | YMR253C | 0.1338662 | 0.97853666 | -0.2537301 | 102.6486648 | 194.3863962 |
| YBR172C | SMY2 | 0.61091904 | 0.99917121 | -0.2535717 | 96.13727876 | 183.998584 |
| YJL112W | MDV1 | 0.25938398 | 0.99917121 | -0.2533681 | 126.3701152 | 232.1154273 |
| YGR050C | YGR050C | 0.44040429 | 0.99917121 | -0.2531007 | 138.6978052 | 251.7129881 |
| YOR325W | YOR325W | 0.37834903 | 0.99917121 | -0.2524541 | 100.4332354 | 190.7030604 |
| YOR008C-A | YOR008C-A | 0.10159099 | 0.94753174 | -0.2524502 | 128.2857479 | 235.0539146 |

| | | | | | | |
|---|---|---|---|---|---|---|
| YLR032W | RAD5 | 0.15600289 | 0.98575602 | -0.2524356 | 95.64047663 | 183.0689917 |
| YNL134C | YNL134C | 0.36313936 | 0.99917121 | -0.2523129 | 98.36114253 | 187.3863257 |
| YDR076W | RAD55 | 0.08218764 | 0.94753174 | -0.2523073 | 104.8357342 | 197.6955499 |
| YJR124C | YJR124C | 0.04568126 | 0.94753174 | -0.251866 | 115.7600019 | 215.0371531 |
| YKL136W | YKL136W | 0.23851707 | 0.99917121 | -0.2518005 | 126.6683929 | 232.3992828 |
| YNL190W | YNL190W | 0.10054389 | 0.94753174 | -0.2513352 | 78.50997878 | 155.6568658 |
| YLR460C | YLR460C | 0.06503265 | 0.94753174 | -0.2512796 | 93.49398446 | 179.5100663 |
| YBR171W | SEC66 | 0.15734023 | 0.98919321 | -0.2506302 | 122.1292612 | 225.0286735 |
| YBR086C | IST2 | 0.48763227 | 0.99917121 | -0.2502854 | 99.94462693 | 189.660634 |
| YMR246W | FAA4 | 0.39096438 | 0.99917121 | -0.250017 | 110.0907251 | 205.7841897 |
| YGR100W | MDR1 | 0.08958382 | 0.94753174 | -0.2492964 | 132.9715646 | 242.1309487 |
| YNL028W | YNL028W | 0.27041334 | 0.99917121 | -0.2488393 | 134.1677882 | 243.9800521 |
| YKL163W | PIR3 | 0.17770384 | 0.99917121 | -0.2486587 | 126.4201178 | 231.6209248 |
| YLR450W | HMG2 | 0.02555573 | 0.92955221 | -0.2483551 | 89.04259232 | 172.0653117 |
| YJL079C | PRY1 | 0.26293347 | 0.99917121 | -0.2483543 | 115.7834027 | 214.6463031 |
| YNL083W | SAL1 | 0.16058541 | 0.99381655 | -0.2481659 | 92.26563566 | 177.1745037 |
| YDL033C | SLM3 | 0.27220539 | 0.99917121 | -0.2480676 | 0 | 30.24209359 |
| YIL002C | INP51 | 0.26116399 | 0.99917121 | -0.2480613 | 125.0092471 | 229.3014775 |
| YBR182C | SMP1 | 0.08847274 | 0.94753174 | -0.247639 | 120.9791962 | 222.8326907 |
| YNR044W | AGA1 | 0.34095937 | 0.99917121 | -0.2473319 | 105.2667904 | 197.7753861 |
| YKL202W | YKL202W | 0.12807323 | 0.97616733 | -0.2472354 | 106.6271519 | 199.9298152 |
| YDL176W | YDL176W | 0.24061083 | 0.99917121 | -0.2463051 | 126.4188778 | 231.3320255 |
| YDR297W | SUR2 | 0.26368605 | 0.99917121 | -0.2459789 | 139.2851459 | 251.7800311 |
| YLR446W | YLR446W | 0.35096665 | 0.99917121 | -0.2459552 | 100.42557 | 189.8985732 |
| YGR286C | BIO2 | 0.40620125 | 0.99917121 | -0.2457395 | 117.0482354 | 216.3416039 |
| YMR284W | YKU70 | 0.27992401 | 0.99917121 | -0.245492 | 133.0735402 | 241.8295405 |
| YNL159C | ASI2 | 0.14687487 | 0.98328895 | -0.2451068 | 102.4496864 | 193.01827 |
| YKR019C | IRS4 | 0.14432458 | 0.98328895 | -0.2444399 | 102.5789635 | 193.14283 |
| YGR105W | VMA21 | 0.69514361 | 0.99917121 | -0.243968 | 51.59973277 | 111.9078371 |
| YBR010W | HHT1 | 0.39614985 | 0.99917121 | -0.2438713 | 105.5428177 | 197.7930362 |
| YLR402W | YLR402W | 0.63734457 | 0.99917121 | -0.2431427 | 34.79831597 | 85.05325942 |
| YHL046C | PAU13 | 0.2286321 | 0.99917121 | -0.2421426 | 112.7274443 | 209.0228282 |
| YHL031C | GOS1 | 0.31244237 | 0.99917121 | -0.2405042 | 138.4479435 | 249.7794726 |
| YER056C | FCY2 | 0.2092822 | 0.99917121 | -0.2391892 | 123.1540451 | 225.2657189 |
| YNL230C | ELA1 | 0.06217602 | 0.94753174 | -0.2358738 | 118.9208374 | 218.120735 |
| YKL115C | YKL115C | 0.1982892 | 0.99917121 | -0.2347053 | 107.0078582 | 199.0084882 |
| YAL029C | MYO4 | 0.21183099 | 0.99917121 | -0.2346467 | 133.0702096 | 240.5020814 |
| YCL061C | MRC1 | 0.15320519 | 0.98492771 | -0.2339126 | 92.13716261 | 175.2322903 |
| YKR026C | GCN3 | 0.22610861 | 0.99917121 | -0.233897 | 131.2856832 | 237.5690688 |
| YGR194C | XKS1 | 0.31421364 | 0.99917121 | -0.2334578 | 117.688777 | 215.8643066 |
| YMR036C | MIH1 | 0.46858328 | 0.99917121 | -0.2330666 | 129.5211958 | 234.6581287 |
| YOL137W | BSC6 | 0.38624691 | 0.99917121 | -0.2329897 | 113.6923141 | 209.4434174 |
| YGL209W | MIG2 | 0.23429344 | 0.99917121 | -0.2324428 | 109.7997437 | 203.1783539 |
| YBR270C | BIT2 | 0.20457163 | 0.99917121 | -0.2322556 | 134.7731683 | 242.9222988 |
| YKR027W | BCH2 | 0.69228503 | 0.99917121 | -0.2311215 | 73.832013 | 145.743576 |
| YBR272C | HSM3 | 0.20143494 | 0.99917121 | -0.2305637 | 135.9660496 | 244.6155466 |
| YJL093C | TOK1 | 0.41628485 | 0.99917121 | -0.2305008 | 124.6153913 | 226.5335019 |
| YFR039C | YFR039C | 0.34360341 | 0.99917121 | -0.2285862 | 122.0173888 | 222.1631235 |
| YER071C | YER071C | 0.26937108 | 0.99917121 | -0.2280187 | 129.4591918 | 233.943999 |
| YCL022C | YCL022C | 0.18380666 | 0.99917121 | -0.226882 | 139.2538826 | 249.4021273 |
| YBR025C | YBR025C | 0.17379205 | 0.99917121 | -0.2268545 | 92.40679296 | 174.8011859 |
| YHL006C | SHU1 | 0.29059847 | 0.99917121 | -0.2266492 | 115.9540271 | 212.2719086 |
| YPL213W | LEA1 | 0.31426655 | 0.99917121 | -0.2259878 | 87.50224783 | 166.8857072 |
| YBR147W | RTC2 | 0.66355612 | 0.99917121 | -0.2257011 | 70.35022694 | 139.538511 |
| YDR266C | YDR266C | 0.3391093 | 0.99917121 | -0.2255402 | 119.6486359 | 218.0198717 |
| YGR183C | QCR9 | 0.34150383 | 0.99917121 | -0.2252124 | 108.3335681 | 199.9622164 |
| YIL044C | AGE2 | 0.15232465 | 0.98492771 | -0.2242841 | 106.2190339 | 196.4819301 |
| YIR043C | YIR043C | 0.22688507 | 0.99917121 | -0.2237615 | 130.6184488 | 235.2709589 |
| YAL065C | YAL065C | 0.76287616 | 0.99917121 | -0.2234814 | 103.9066074 | 192.7018587 |
| YOL105C | WSC3 | 0.4148814 | 0.99917121 | -0.2231677 | 116.719235 | 213.0659634 |
| YNL286W | CUS2 | 0.11731592 | 0.9579757 | -0.2223518 | 95.25230787 | 178.7833554 |
| YHL047C | ARN2 | 0.24506825 | 0.99917121 | -0.2222023 | 120.7479171 | 219.3634067 |
| YAL026C | DRS2 | 0.38457014 | 0.99917121 | -0.2220398 | 95.16821238 | 178.6114093 |
| YMR304W | UBP15 | 0.20169983 | 0.99917121 | -0.2214055 | 116.1004651 | 211.865836 |

| | | | | | | |
|---|---|---|---|---|---|---|
| YPR070W | MED1 | 0.53575165 | 0.99917121 | -0.2193713 | 6.751928478 | 37.49522565 |
| YKR095W | MLP1 | 0.40603334 | 0.99917121 | -0.2190468 | 101.1006253 | 187.6930853 |
| YHR157W | REC104 | 0.31289507 | 0.99917121 | -0.2187923 | 114.503986 | 209.0050838 |
| YLR394W | CST9 | 0.04001369 | 0.94753174 | -0.2186302 | 91.63930549 | 172.576437 |
| YPL127C | HHO1 | 0.41610995 | 0.99917121 | -0.217696 | 119.0608679 | 216.1276448 |
| YGR028W | MSP1 | 0.48584307 | 0.99917121 | -0.2176872 | 115.9476433 | 211.1691883 |
| YHR156C | LIN1 | 0.25474275 | 0.99917121 | -0.2173475 | 109.002789 | 200.0690437 |
| YNL271C | BNI1 | 0.46894042 | 0.99917121 | -0.2159923 | 92.43881938 | 173.5279702 |
| YPL041C | YPL041C | 0.39092826 | 0.99917121 | -0.2144503 | 123.4298151 | 222.688908 |
| YLR431C | ATG23 | 0.4071713 | 0.99917121 | -0.213558 | 105.5195867 | 194.0605337 |
| YNL087W | TCB2 | 0.31844465 | 0.99917121 | -0.213543 | 132.5795293 | 237.1479683 |
| YPR127W | YPR127W | 0.52602912 | 0.99917121 | -0.2135164 | 110.0773916 | 201.3131394 |
| YKL215C | YKL215C | 0.06890402 | 0.94753174 | -0.2123072 | 116.7262489 | 211.7531257 |
| YLR015W | BRE2 | 0.16546203 | 0.99381655 | -0.211665 | 98.21023725 | 182.1906117 |
| YBL081W | YBL081W | 0.29152394 | 0.99917121 | -0.2107286 | 112.7579431 | 205.2416926 |
| YMR274C | RCE1 | 0.38989847 | 0.99917121 | -0.2105517 | 128.4031763 | 230.1330255 |
| YDR388W | RVS167 | 0.6835458 | 0.99917121 | -0.2098502 | 101.8139345 | 187.7077669 |
| YPR135W | CTF4 | 0.68058557 | 0.99917121 | -0.2096314 | 84.86084031 | 160.6856063 |
| YBR175W | SWD3 | 0.54153441 | 0.99917121 | -0.209557 | 62.34750837 | 124.8271311 |
| YJL172W | CPS1 | 0.30067755 | 0.99917121 | -0.2094145 | 93.14720993 | 173.8540769 |
| YFR010W | UBP6 | 0.32723751 | 0.99917121 | -0.2091111 | 100.2677898 | 185.1556454 |
| YDL230W | PTP1 | 0.71515851 | 0.99917121 | -0.2083397 | 106.5733619 | 195.1023649 |
| YBL021C | HAP3 | 0.31677979 | 0.99917121 | -0.2078947 | 106.2967259 | 194.6076069 |
| YOL062C | APM4 | 0.70149138 | 0.99917121 | -0.2078931 | 103.0846674 | 189.4926395 |
| YIL066C | RNR3 | 0.24603985 | 0.99917121 | -0.207491 | 131.1721793 | 234.1691481 |
| YML121W | GTR1 | 0.21566072 | 0.99917121 | -0.2074697 | 109.4325602 | 199.5491747 |
| YHL045W | YHL045W | 0.30228076 | 0.99917121 | -0.2069475 | 111.6047628 | 202.9444606 |
| YOR140W | SFL1 | 0.39100222 | 0.99917121 | -0.2062479 | 0 | 25.1438304 |
| YGR241C | YAP1802 | 0.3650711 | 0.99917121 | -0.2060083 | 128.4882841 | 229.7146573 |
| YPR172W | YPR172W | 0.41982092 | 0.99917121 | -0.205879 | 103.2176777 | 189.4589017 |
| YIL079C | AIR1 | 0.29513175 | 0.99917121 | -0.2055972 | 132.704763 | 236.3787026 |
| YOR209C | NPT1 | 0.28354855 | 0.99917121 | -0.2055479 | 125.9352591 | 225.5931848 |
| YEL057C | YEL057C | 0.68002221 | 0.99917121 | -0.2052815 | 26.7169606 | 67.56911803 |
| YMR092C | AIP1 | 0.20067374 | 0.99917121 | -0.2050462 | 104.3890234 | 191.2225889 |
| YMR186W | HSC82 | 0.04423183 | 0.94753174 | -0.2046113 | 91.15559138 | 170.0971403 |
| YJR048W | CYC1 | 0.30338681 | 0.99917121 | -0.2043412 | 127.7859451 | 228.3930474 |
| YMR164C | MSS11 | 0.09889816 | 0.94753174 | -0.2038706 | 89.59510083 | 167.5219662 |
| YDR098C | GRX3 | 0.35311117 | 0.99917121 | -0.2037377 | 134.8077496 | 239.5007333 |
| YOL110W | SHR5 | 0.47364345 | 0.99917121 | -0.2034428 | 121.6305283 | 218.4818577 |
| YGR035C | YGR035C | 0.19905825 | 0.99917121 | -0.2026056 | 119.6066546 | 215.1570451 |
| YIL043C | CBR1 | 0.27156101 | 0.99917121 | -0.2026031 | 141.3953145 | 249.8522046 |
| YDR414C | ERD1 | 0.3623961 | 0.99917121 | -0.2021175 | 132.0222082 | 234.8676154 |
| YHR086W | NAM8 | 0.60058717 | 0.99917121 | -0.2018809 | 117.6949404 | 212.0245617 |
| YMR078C | CTF18 | 0.57499197 | 0.99917121 | -0.2016788 | 70.70471786 | 137.1744126 |
| YKL047W | YKL047W | 0.32832165 | 0.99917121 | -0.2016064 | 131.5807666 | 234.1023736 |
| YHR061C | GIC1 | 0.48296271 | 0.99917121 | -0.2004791 | 117.5917106 | 211.6892891 |
| YJL187C | SWE1 | 0.28391273 | 0.99917121 | -0.1996098 | 135.4813741 | 240.0701529 |
| YGR202C | PCT1 | 0.21708485 | 0.99917121 | -0.1992868 | 130.4082569 | 231.9525311 |
| YHR167W | THP2 | 0.35605127 | 0.99917121 | -0.1991337 | 130.4472422 | 231.9959416 |
| YCR048W | ARE1 | 0.76967741 | 0.99917121 | -0.1989952 | 132.5959618 | 235.4005986 |
| YOR027W | STI1 | 0.24997378 | 0.99917121 | -0.1989792 | 135.2119492 | 239.56425 |
| YNL206C | RTT106 | 0.42946428 | 0.99917121 | -0.1974632 | 115.8967261 | 208.6225849 |
| YML072C | TCB3 | 0.50664118 | 0.99917121 | -0.1974059 | 100.4624021 | 184.0385466 |
| YNR025C | YNR025C | 0.49848432 | 0.99917121 | -0.1971546 | 119.2471575 | 213.9200616 |
| YOL002C | IZH2 | 0.31756066 | 0.99917121 | -0.1965391 | 123.6023308 | 220.7800476 |
| YJR128W | YJR128W | 0.35077305 | 0.99917121 | -0.1965064 | 108.1086374 | 196.1044733 |
| YNL155W | YNL155W | 0.32208171 | 0.99917121 | -0.1953011 | 94.29678958 | 173.9640476 |
| YDL206W | YDL206W | 0.48801579 | 0.99917121 | -0.1950157 | 135.9054274 | 240.185324 |
| YHL030W | ECM29 | 0.4106291 | 0.99917121 | -0.1945111 | 121.9244055 | 217.8609518 |
| YCR020C | PET18 | 0.33625818 | 0.99917121 | -0.1941979 | 118.9841399 | 213.1407963 |
| YBR012C | YBR012C | 0.70735869 | 0.99917121 | -0.1936551 | 116.2143402 | 208.6640871 |
| YDR257C | SET7 | 0.31329434 | 0.99917121 | -0.1936515 | 124.8340944 | 222.3894303 |
| YDL094C | YDL094C | 0.18866164 | 0.99917121 | -0.1935308 | 134.9387256 | 238.4649606 |
| YAR029W | YAR029W | 0.24481278 | 0.99917121 | -0.1933014 | 135.4562595 | 239.2610978 |

| | | | | | | |
|---|---|---|---|---|---|---|
| YBL106C | SRO77 | 0.34085393 | 0.99917121 | -0.1932645 | 119.5443252 | 213.9190183 |
| YHL023C | RMD11 | 0.615682 | 0.99917121 | -0.1926955 | 83.74248251 | 156.8401066 |
| YOR129C | YOR129C | 0.5393188 | 0.99917121 | -0.1926893 | 128.6516867 | 228.3511256 |
| YIL039W | YIL039W | 0.47681051 | 0.99917121 | -0.1925195 | 107.8488857 | 195.204808 |
| YPR179C | HDA3 | 0.19793428 | 0.99917121 | -0.1922704 | 77.01981745 | 146.0833526 |
| YKR007W | MEH1 | 0.46764237 | 0.99917121 | -0.1913038 | 110.3482448 | 199.0364816 |
| YJL146W | IDS2 | 0.27564499 | 0.99917121 | -0.1911586 | 139.0869833 | 244.7813072 |
| YOR088W | YOR088W | 0.66227117 | 0.99917121 | -0.1906222 | 109.0472502 | 196.8817349 |
| YKL211C | TRP3 | 0.31006059 | 0.99917121 | -0.1903629 | 98.24308412 | 179.6459655 |
| YGL235W | YGL235W | 0.27969513 | 0.99917121 | -0.1897023 | 101.5679602 | 184.859847 |
| YJL053W | PEP8 | 0.32931988 | 0.99917121 | -0.1893072 | 128.0466301 | 226.9753433 |
| YIR035C | YIR035C | 0.4230916 | 0.99917121 | -0.1888586 | 126.1377295 | 223.8809829 |
| YLL039C | UBI4 | 0.03582819 | 0.94753174 | -0.1887295 | 104.5295957 | 189.4572484 |
| YGR079W | YGR079W | 0.27637854 | 0.99917121 | -0.1882753 | 97.00832787 | 177.42529 |
| YNL080C | EOS1 | 0.366535 | 0.99917121 | -0.1879849 | 77.98796853 | 147.1025634 |
| YNL069C | RPL16B | 0.6481606 | 0.99917121 | -0.1874842 | 107.9959822 | 194.8251763 |
| YMR224C | MRE11 | 0.09716644 | 0.94753174 | -0.1870594 | 79.9743457 | 150.1527692 |
| YNL168C | FMP41 | 0.11972886 | 0.96096398 | -0.187016 | 123.0837909 | 218.793379 |
| YGR229C | SMI1 | 0.67372219 | 0.99917121 | -0.186959 | 65.60559409 | 127.2602473 |
| YBR131W | CCZ1 | 0.7905736 | 0.99917121 | -0.1867023 | 39.64258317 | 85.88640787 |
| YLR432W | IMD3 | 0.01287633 | 0.84351098 | -0.1866186 | 95.9450933 | 175.5302601 |
| YOR033C | EXO1 | 0.24190717 | 0.99917121 | -0.1865364 | 58.46671812 | 115.841041 |
| YLR441C | RPS1A | 0.55862877 | 0.99917121 | -0.1861638 | 93.85133072 | 172.1407821 |
| YCL011C | GBP2 | 0.178261 | 0.99917121 | -0.1856868 | 135.8870338 | 239.0187518 |
| YKR104W | YKR104W | 0.45068597 | 0.99917121 | -0.1855339 | 114.9230188 | 205.6177731 |
| YIL124W | AYR1 | 0.33157758 | 0.99917121 | -0.1849122 | 126.470339 | 223.9295217 |
| YIL116W | HIS5 | 0.34234066 | 0.99917121 | -0.1845599 | 132.9629279 | 234.2251279 |
| YIL057C | YIL057C | 0.07788902 | 0.94753174 | -0.1840429 | 153.066845 | 266.1748403 |
| YJR066W | TOR1 | 0.47441689 | 0.99917121 | -0.1834423 | 116.2078103 | 207.4086416 |
| YOL104C | NDJ1 | 0.50682783 | 0.99917121 | -0.1823516 | 118.7848122 | 211.3792011 |
| YJR115W | YJR115W | 0.55737401 | 0.99917121 | -0.1823419 | 107.345117 | 193.1618701 |
| YIL161W | YIL161W | 0.40277542 | 0.99917121 | -0.1823245 | 140.4638817 | 245.8968531 |
| YGR011W | YGR011W | 0.28503768 | 0.99917121 | -0.1818468 | 133.8674283 | 235.3346681 |
| YDR477W | SNF1 | 0.52608379 | 0.99917121 | -0.1816157 | 104.840744 | 189.0854624 |
| YGL261C | PAU11 | 0.35087952 | 0.99917121 | -0.1813163 | 108.4952242 | 194.8682234 |
| YDR508C | GNP1 | 0.38020064 | 0.99917121 | -0.1806763 | 142.067715 | 248.2498059 |
| YER096W | SHC1 | 0.28728189 | 0.99917121 | -0.1803773 | 140.2957912 | 245.3918069 |
| YOR339C | UBC11 | 0.51669364 | 0.99917121 | -0.1798231 | 108.8798214 | 195.2986081 |
| YJL217W | YJL217W | 0.34155387 | 0.99917121 | -0.1792301 | 131.0366349 | 230.5080076 |
| YBR283C | SSH1 | 0.28057107 | 0.99917121 | -0.1789365 | 126.7600806 | 223.662384 |
| YML048W-A | YML048W-A | 0.53853699 | 0.99917121 | -0.1785511 | 126.8227134 | 223.7151406 |
| YOR328W | PDR10 | 0.36509486 | 0.99917121 | -0.1776648 | 132.0793218 | 231.9775203 |
| YBR287W | ZSP1 | 0.36963476 | 0.99917121 | -0.1776494 | 145.8135638 | 253.8455421 |
| YOL152W | FRE7 | 0.52709747 | 0.99917121 | -0.1771399 | 126.2372458 | 222.6108165 |
| YNL040W | YNL040W | 0.70330943 | 0.99917121 | -0.1756819 | 108.6124475 | 194.3679856 |
| YDR401W | YDR401W | 0.19450687 | 0.99917121 | -0.1752076 | 145.0195873 | 252.2835694 |
| YNL179C | SRF6 | 0.53658001 | 0.99917121 | -0.1751473 | 125.8185811 | 221.7012334 |
| YMR016C | SOK2 | 0.6320131 | 0.99917121 | -0.1744203 | 104.1112215 | 187.046602 |
| YJR020W | YJR020W | 0.38671417 | 0.99917121 | -0.1741152 | 127.5501159 | 224.3326448 |
| YMR214W | SCJ1 | 0.389555 | 0.99917121 | -0.1737772 | 135.1000916 | 236.3137396 |
| YDR004W | RAD57 | 0.4400139 | 0.99917121 | -0.1733327 | 120.8784359 | 213.6135108 |
| YJL144W | YJL144W | 0.79798351 | 0.99917121 | -0.1725903 | 106.4163113 | 190.4940444 |
| YKR065C | PAM17 | 0.08380728 | 0.94753174 | -0.17259 | 107.5114515 | 192.2378687 |
| YGR080W | TWF1 | 0.26233127 | 0.99917121 | -0.1716927 | 123.719305 | 217.93727 |
| YCR032W | BPH1 | 0.34505454 | 0.99917121 | -0.1715896 | 134.666703 | 235.3569405 |
| YIL053W | RHR2 | 0.55063195 | 0.99917121 | -0.1715437 | 121.9959246 | 215.1748536 |
| YJR142W | YJR142W | 0.28003557 | 0.99917121 | -0.1710635 | 145.3163008 | 252.2508284 |
| YIL141W | YIL141W | 0.27070171 | 0.99917121 | -0.1709196 | 129.9477957 | 227.7610423 |
| YJL128C | PBS2 | 0.3331119 | 0.99917121 | -0.1708051 | 144.8191187 | 251.4276362 |
| YOR371C | GPB1 | 0.41830613 | 0.99917121 | -0.1707942 | 123.3680195 | 217.2683649 |
| YDL112W | TRM3 | 0.20940618 | 0.99917121 | -0.1704049 | 138.4999893 | 241.3164947 |
| YHR015W | MIP6 | 0.4770152 | 0.99917121 | -0.1702575 | 115.2211662 | 204.2301782 |
| YKL062W | MSN4 | 0.40909029 | 0.99917121 | -0.168702 | 120.7081126 | 212.7777657 |
| YNL128W | TEP1 | 0.61398491 | 0.99917121 | -0.1683668 | 119.5074069 | 210.8249317 |

| | | | | | | |
|---|---|---|---|---|---|---|
| YOL031C | SIL1 | 0.43347817 | 0.99917121 | -0.168043 | 131.6014641 | 230.043591 |
| YMR196W | YMR196W | 0.3822563 | 0.99917121 | -0.1680402 | 124.0388981 | 218.0009024 |
| YLR454W | FMP27 | 0.56713287 | 0.99917121 | -0.1680401 | 101.3080044 | 181.805047 |
| YHR110W | ERP5 | 0.62696766 | 0.99917121 | -0.167092 | 111.0758453 | 197.2434086 |
| YJR084W | CSN12 | 0.48415735 | 0.99917121 | -0.1669472 | 122.284241 | 215.0736048 |
| YOL111C | MDY2 | 0.49053194 | 0.99917121 | -0.1664573 | 137.6223593 | 239.4377312 |
| YGL121C | GPG1 | 0.29389669 | 0.99917121 | -0.1662981 | 110.4663859 | 196.1761479 |
| YBR129C | OPY1 | 0.58500129 | 0.99917121 | -0.1659961 | 113.5009527 | 200.9714689 |
| YOR124C | UBP2 | 0.43927428 | 0.99917121 | -0.1657973 | 113.0029618 | 200.1542425 |
| YNL108C | YNL108C | 0.45871849 | 0.99917121 | -0.1652932 | 113.354977 | 200.6533328 |
| YNR069C | BSC5 | 0.50610229 | 0.99917121 | -0.1650358 | 110.9233255 | 196.7498799 |
| YJL027C | YJL027C | 0.43965208 | 0.99917121 | -0.1647448 | 140.5386823 | 243.8728124 |
| YDR400W | URH1 | 0.38758297 | 0.99917121 | -0.1645849 | 144.9170808 | 250.8253168 |
| YIL112W | HOS4 | 0.4669368 | 0.99917121 | -0.1645041 | 133.9963668 | 233.4257155 |
| YDR073W | SNF11 | 0.26198203 | 0.99917121 | -0.1644425 | 154.5424909 | 266.135111 |
| YGL046W | YGL046W | 0.314719 | 0.99917121 | -0.1642381 | 118.3020417 | 208.4022249 |
| YGR174C | CBP4 | 0.3221173 | 0.99917121 | -0.1638964 | 127.6212842 | 223.2001824 |
| YLR334C | YLR334C | 0.43521823 | 0.99917121 | -0.1635335 | 84.40472156 | 154.3394698 |
| YKR091W | SRL3 | 0.31142408 | 0.99917121 | -0.1635122 | 130.0564287 | 227.0309806 |
| YOR269W | PAC1 | 0.49150044 | 0.99917121 | -0.1634807 | 113.2646094 | 200.2884686 |
| YCR037C | PHO87 | 0.36534915 | 0.99917121 | -0.1631173 | 146.0512852 | 252.4524639 |
| YLR415C | YLR415C | 0.33900417 | 0.99917121 | -0.1629638 | 135.0943673 | 234.9863547 |
| YDL133W | YDL133W | 0.31606993 | 0.99917121 | -0.162846 | 147.1553666 | 254.177488 |
| YNL298W | CLA4 | 0.5288522 | 0.99917121 | -0.1627455 | 96.86971576 | 174.0922115 |
| YNL094W | APP1 | 0.38454993 | 0.99917121 | -0.1620627 | 140.5635483 | 243.5854338 |
| YNL063W | MTQ1 | 0.42810538 | 0.99917121 | -0.1615972 | 141.8683 | 245.6063147 |
| YJR040W | GEF1 | 0.25698207 | 0.99917121 | -0.1613659 | 126.7480072 | 221.5011208 |
| YNL130C | CPT1 | 0.41571534 | 0.99917121 | -0.1613144 | 135.0361576 | 234.6925879 |
| YDR075W | PPH3 | 0.29102534 | 0.99917121 | -0.1612756 | 139.6608296 | 242.0520153 |
| YJL047C | RTT101 | 0.39752051 | 0.99917121 | -0.1609909 | 104.467232 | 185.9763125 |
| YBR258C | SHG1 | 0.30918001 | 0.99917121 | -0.1607571 | 148.3432183 | 255.8143256 |
| YDL002C | NHP10 | 0.82900367 | 0.99917121 | -0.1603698 | 47.80672488 | 95.67647629 |
| YBL094C | YBL094C | 0.46101441 | 0.99917121 | -0.1602393 | 121.1536596 | 212.4555358 |
| YDR528W | HLR1 | 0.49519761 | 0.99917121 | -0.1600356 | 135.3463729 | 235.0306596 |
| YCL001W | RER1 | 0.41653263 | 0.99917121 | -0.1597623 | 132.355595 | 230.2349357 |
| YOR039W | CKB2 | 0.37146509 | 0.99917121 | -0.1596342 | 89.52745758 | 162.021365 |
| YBL011W | SCT1 | 0.59984494 | 0.99917121 | -0.1596091 | 114.0167575 | 201.0141639 |
| YOL088C | MPD2 | 0.4528679 | 0.99917121 | -0.1595421 | 106.2354495 | 188.6153338 |
| YLR320W | MMS22 | 0.40781599 | 0.99917121 | -0.1594897 | 79.3146375 | 145.7412263 |
| YOL086C | ADH1 | 0.52621402 | 0.99917121 | -0.1592337 | 105.528497 | 187.4520063 |
| YNL143C | YNL143C | 0.3073012 | 0.99917121 | -0.159003 | 99.80123608 | 178.3040051 |
| YNL030W | HHF2 | 0.3340372 | 0.99917121 | -0.1589226 | 96.68382769 | 173.3301531 |
| YOL027C | MDM38 | 0.40256326 | 0.99917121 | -0.1581862 | 129.8086354 | 225.987112 |
| YNL294C | RIM21 | 0.50144542 | 0.99917121 | -0.1564345 | 128.7311973 | 224.0578875 |
| YPL123C | RNY1 | 0.43312175 | 0.99917121 | -0.156336 | 132.5389432 | 230.1091924 |
| YOL087C | YOL087C | 0.48166718 | 0.99917121 | -0.1562977 | 122.6733171 | 214.3948598 |
| YJL162C | JJJ2 | 0.45456967 | 0.99917121 | -0.1557289 | 136.4931991 | 236.331801 |
| YNL051W | COG5 | 0.41210071 | 0.99917121 | -0.155441 | 129.5452881 | 225.2330927 |
| YOR267C | HRK1 | 0.62227891 | 0.99917121 | -0.155069 | 102.6933643 | 182.4297278 |
| YML060W | OGG1 | 0.61221662 | 0.99917121 | -0.1548906 | 99.63482735 | 177.5376706 |
| YKL066W | YKL066W | 0.23016459 | 0.99917121 | -0.1546674 | 132.1377571 | 229.2669397 |
| YDR386W | MUS81 | 0.33185319 | 0.99917121 | -0.1543183 | 135.2796701 | 234.2274532 |
| YKL129C | MYO3 | 0.36034485 | 0.99917121 | -0.154126 | 110.507607 | 194.7578818 |
| YOR363C | PIP2 | 0.5943227 | 0.99917121 | -0.1533345 | 134.9766085 | 233.6249273 |
| YBR301W | DAN3 | 0.33437076 | 0.99917121 | -0.1531813 | 141.4654772 | 243.9388895 |
| YNL253W | TEX1 | 0.47819753 | 0.99917121 | -0.1529415 | 124.0885565 | 216.2392782 |
| YOR219C | STE13 | 0.45598151 | 0.99917121 | -0.1528392 | 118.8858428 | 207.9421969 |
| YAL042W | ERV46 | 0.36193851 | 0.99917121 | -0.1527528 | 141.8332657 | 244.4723034 |
| YJL165C | HAL5 | 0.49849552 | 0.99917121 | -0.152377 | 116.4486206 | 204.00491 |
| YJR092W | BUD4 | 0.42795139 | 0.99917121 | -0.1517776 | 138.7833651 | 239.4968618 |
| YER055C | HIS1 | 0.57025712 | 0.99917121 | -0.1517365 | 118.2932919 | 206.8642086 |
| YDR146C | SWI5 | 0.38839174 | 0.99917121 | -0.151657 | 125.4660738 | 218.2761929 |
| YBR098W | MMS4 | 0.4214833 | 0.99917121 | -0.1514466 | 130.0970965 | 225.6248206 |
| YJL057C | IKS1 | 0.47678257 | 0.99917121 | -0.1514115 | 137.2646626 | 237.0339055 |

| | | | | | | |
|---|---|---|---|---|---|---|
| YPL092W | SSU1 | 0.43713958 | 0.99917121 | -0.1506387 | 136.8140134 | 236.222102 |
| YHR028C | DAP2 | 0.52186397 | 0.99917121 | -0.1505842 | 111.9560007 | 196.632465 |
| YDR245W | MNN10 | 0.7740956 | 0.99917121 | -0.1500993 | 55.50178182 | 106.6777144 |
| YBR083W | TEC1 | 0.56455032 | 0.99917121 | -0.1500372 | 124.6312488 | 216.7493755 |
| YJL115W | ASF1 | 0.70401853 | 0.99917121 | -0.1499039 | 52.23568385 | 101.4530798 |
| YAL058C-A | YAL058C-A | 0.44083987 | 0.99917121 | -0.1498565 | 122.9374676 | 214.0302383 |
| YOR093C | YOR093C | 0.48563092 | 0.99917121 | -0.1496526 | 129.531447 | 224.5053855 |
| YEL003W | GIM4 | 0.74871848 | 0.99917121 | -0.1495055 | 79.43351763 | 144.7133506 |
| YDR209C | YDR209C | 0.44766938 | 0.99917121 | -0.1492629 | 138.7619787 | 239.1562442 |
| YNL092W | YNL092W | 0.46611279 | 0.99917121 | -0.1492252 | 142.1203376 | 244.4993785 |
| YJL084C | ALY2 | 0.48016524 | 0.99917121 | -0.1492238 | 116.8778221 | 204.303946 |
| YKR105C | YKR105C | 0.668159 | 0.99917121 | -0.1483846 | 127.9544087 | 221.8395949 |
| YDR151C | CTH1 | 0.40757087 | 0.99917121 | -0.1482974 | 131.1674725 | 226.9453247 |
| YGR189C | CRH1 | 0.43626935 | 0.99917121 | -0.1480577 | 141.0348128 | 242.6284969 |
| YBR026C | ETR1 | 0.49331902 | 0.99917121 | -0.1478332 | 110.6018592 | 194.1407974 |
| YOL013W-A | YOL013W-A | 0.61652986 | 0.99917121 | -0.1476786 | 121.6961857 | 211.7881553 |
| YCR022C | YCR022C | 0.34874138 | 0.99917121 | -0.1466903 | 145.0630965 | 248.8762829 |
| YNL214W | PEX17 | 0.48129149 | 0.99917121 | -0.1466652 | 127.8949549 | 221.5353036 |
| YNL285W | YNL285W | 0.51270489 | 0.99917121 | -0.1465214 | 137.5205274 | 236.8451829 |
| YDR320C | SWA2 | 0.4066934 | 0.99917121 | -0.1463057 | 138.4653358 | 238.3233705 |
| YOR357C | SNX3 | 0.48984642 | 0.99917121 | -0.1460873 | 137.0001836 | 235.9636819 |
| YJR107W | YJR107W | 0.35584022 | 0.99917121 | -0.1460368 | 137.5622708 | 236.8525762 |
| YBR092C | PHO3 | 0.44653053 | 0.99917121 | -0.1457851 | 144.9241025 | 248.5445992 |
| YPL186C | UIP4 | 0.30636599 | 0.99917121 | -0.1454536 | 135.3950178 | 233.3304191 |
| YOR163W | DDP1 | 0.54085014 | 0.99917121 | -0.1446211 | 130.6212787 | 225.6274031 |
| YLR390W-A | CCW14 | 0.14429302 | 0.98328895 | -0.14341 | 98.93530258 | 175.0241673 |
| YDR541C | YDR541C | 0.57343631 | 0.99917121 | -0.1429556 | 131.0704735 | 226.1396403 |
| YNL339C | YRF1-6 | 0.42071566 | 0.99917121 | -0.1429214 | 140.1871026 | 240.6524567 |
| YOR167C | RPS28A | 0.56016142 | 0.99917121 | -0.1424635 | 132.4978797 | 228.352597 |
| YGR003W | CUL3 | 0.47865209 | 0.99917121 | -0.1420953 | 134.9627983 | 232.2327563 |
| YHL034C | SBP1 | 0.55341086 | 0.99917121 | -0.1420695 | 120.9050517 | 209.8445777 |
| YJL190C | RPS22A | 0.36539235 | 0.99917121 | -0.1417782 | 142.8542387 | 244.7601394 |
| YMR135W-A | YMR135W-A | 0.71981072 | 0.99917121 | -0.1417282 | 75.52572495 | 137.5425816 |
| YDR097C | MSH6 | 0.61420539 | 0.99917121 | -0.1416192 | 114.0796212 | 198.9211094 |
| YMR140W | SIP5 | 0.53952985 | 0.99917121 | -0.1413938 | 134.4306847 | 231.2999194 |
| YKL096W-A | CWP2 | 0.39998851 | 0.99917121 | -0.1412146 | 139.1707546 | 238.8259838 |
| YLR034C | SMF3 | 0.15128409 | 0.98328895 | -0.1411115 | 94.28214586 | 167.3344418 |
| YNR040W | YNR040W | 0.55471509 | 0.99917121 | -0.1411063 | 131.5921395 | 226.7448766 |
| YCR026C | NPP1 | 0.345241 | 0.99917121 | -0.1410566 | 122.7691489 | 212.6894152 |
| YIL095W | PRK1 | 0.49210418 | 0.99917121 | -0.1407714 | 136.8115852 | 235.0153039 |
| YNL095C | YNL095C | 0.52715629 | 0.99917121 | -0.140449 | 102.4877575 | 180.3199856 |
| YIL101C | XBP1 | 0.31455382 | 0.99917121 | -0.1403701 | 137.7898672 | 236.5241637 |
| YNL141W | AAH1 | 0.56087997 | 0.99917121 | -0.1397357 | 80.80504822 | 145.7062835 |
| YIR009W | MSL1 | 0.45748031 | 0.99917121 | -0.1397303 | 138.9450002 | 238.285551 |
| YFR025C | HIS2 | 0.51895325 | 0.99917121 | -0.1391484 | 98.7491258 | 174.2081764 |
| YPL030W | TRM44 | 0.55022581 | 0.99917121 | -0.1390859 | 124.911753 | 215.8609685 |
| YJR049C | UTR1 | 0.44053351 | 0.99917121 | -0.1390783 | 135.6919146 | 233.0259714 |
| YGL082W | YGL082W | 0.49722258 | 0.99917121 | -0.1390595 | 136.6813367 | 234.5991965 |
| YGL002W | ERP6 | 0.51854062 | 0.99917121 | -0.1382771 | 135.6451852 | 232.8538932 |
| YIL139C | REV7 | 0.53778642 | 0.99917121 | -0.1381703 | 133.6945678 | 229.7347745 |
| YBR013C | YBR013C | 0.34290871 | 0.99917121 | -0.1381699 | 138.5467552 | 237.4611703 |
| YKR011C | YKR011C | 0.35968052 | 0.99917121 | -0.1380417 | 142.2695761 | 243.3736276 |
| YDL189W | RBS1 | 0.28195938 | 0.99917121 | -0.1380317 | 129.5745742 | 223.1573493 |
| YMR305C | SCW10 | 0.48315464 | 0.99917121 | -0.1380209 | 127.5628832 | 219.9526913 |
| YJL051W | IRC8 | 0.54374582 | 0.99917121 | -0.1376483 | 140.5902332 | 240.6515413 |
| YDR348C | YDR348C | 0.51722004 | 0.99917121 | -0.1370501 | 125.2183529 | 216.1009923 |
| YNR031C | SSK2 | 0.44662881 | 0.99917121 | -0.1369627 | 135.691475 | 232.7673577 |
| YIL097W | FYV10 | 0.49593209 | 0.99917121 | -0.1367858 | 109.3215448 | 190.7552774 |
| YNL175C | NOP13 | 0.52010998 | 0.99917121 | -0.136567 | 124.7990979 | 215.3744997 |
| YNL194C | YNL194C | 0.80346994 | 0.99917121 | -0.136506 | 117.6482841 | 203.9803708 |
| YOR136W | IDH2 | 0.51654796 | 0.99917121 | -0.1364775 | 133.68467 | 229.5126446 |
| YKL084W | HOT13 | 0.49449687 | 0.99917121 | -0.1363496 | 137.5460096 | 235.6457131 |
| YLR346C | YLR346C | 0.49754283 | 0.99917121 | -0.1362855 | 122.3019201 | 211.3637633 |
| YDR225W | HTA1 | 0.48474361 | 0.99917121 | -0.1357172 | 139.1214776 | 238.0773272 |

| | | | | | | |
|---|---|---|---|---|---|---|
| YDR255C | RMD5 | 0.44168791 | 0.99917121 | -0.1355787 | 136.5774956 | 234.0095009 |
| YDR371W | CTS2 | 0.45325715 | 0.99917121 | -0.1355117 | 142.904517 | 244.0762551 |
| YHL032C | GUT1 | 0.69304512 | 0.99917121 | -0.1352375 | 146.5524448 | 249.8516486 |
| YBR267W | REI1 | 0.82995034 | 0.99917121 | -0.1350222 | 64.7157534 | 119.5116461 |
| YMR103C | YMR103C | 0.41470563 | 0.99917121 | -0.1347076 | 118.4693355 | 205.0685337 |
| YGR282C | BGL2 | 0.41170266 | 0.99917121 | -0.1335068 | 146.9062042 | 250.2039743 |
| YOR113W | AZF1 | 0.74168259 | 0.99917121 | -0.1333038 | 119.5278116 | 206.5828744 |
| YMR174C | PAI3 | 0.60340566 | 0.99917121 | -0.1331917 | 98.95255472 | 173.8059193 |
| YPR005C | HAL1 | 0.50228275 | 0.99917121 | -0.1331587 | 139.4991775 | 238.366861 |
| YOR367W | SCP1 | 0.65919379 | 0.99917121 | -0.1331357 | 129.241865 | 222.030683 |
| YDR265W | PEX10 | 0.53711037 | 0.99917121 | -0.1330572 | 133.6643104 | 229.0632511 |
| YDR085C | AFR1 | 0.43543944 | 0.99917121 | -0.1330006 | 145.5286464 | 247.94869 |
| YJL218W | YJL218W | 0.50768873 | 0.99917121 | -0.132771 | 144.3014344 | 245.966532 |
| YHR012W | VPS29 | 0.69608101 | 0.99917121 | -0.1326642 | 110.9509835 | 192.8474675 |
| YBR040W | FIG1 | 0.50863493 | 0.99917121 | -0.1321718 | 147.5518447 | 251.0693115 |
| YDL056W | MBP1 | 0.58816081 | 0.99917121 | -0.1321182 | 132.1809796 | 226.5867764 |
| YKL213C | DOA1 | 0.06106324 | 0.94753174 | -0.1321162 | 91.0176698 | 161.0395873 |
| YJL178C | ATG27 | 0.43001762 | 0.99917121 | -0.1320383 | 112.873208 | 195.8320458 |
| YBR022W | POA1 | 0.40661378 | 0.99917121 | -0.1317152 | 118.686987 | 205.0503095 |
| YDL227C | HO | 0.53558334 | 0.99917121 | -0.1316002 | 130.1677582 | 223.3178433 |
| YJR011C | YJR011C | 0.45127385 | 0.99917121 | -0.1313841 | 127.5281334 | 219.0882579 |
| YBR291C | CTP1 | 0.39906014 | 0.99917121 | -0.1312372 | 131.9068174 | 226.0428092 |
| YOR252W | TMA16 | 0.60954939 | 0.99917121 | -0.1312033 | 105.8693173 | 184.5775072 |
| YMR173W | DDR48 | 0.55012499 | 0.99917121 | -0.1311382 | 113.6395298 | 196.9425832 |
| YKR066C | CCP1 | 0.46766545 | 0.99917121 | -0.1310052 | 130.0790971 | 223.1041355 |
| YNL066W | SUN4 | 0.41501677 | 0.99917121 | -0.1307127 | 106.7003415 | 185.8409952 |
| YOR246C | YOR246C | 0.60762141 | 0.99917121 | -0.130234 | 135.5083323 | 231.6554253 |
| YOL159C | YOL159C | 0.56646198 | 0.99917121 | -0.1302262 | 120.8069993 | 208.2446159 |
| YHR209W | CRG1 | 0.66484696 | 0.99917121 | -0.1299104 | 116.6077509 | 201.5193861 |
| YGR143W | SKN1 | 0.5594281 | 0.99917121 | -0.1299007 | 136.656345 | 233.4428501 |
| YDR358W | GGA1 | 0.48669115 | 0.99917121 | -0.1297222 | 141.9702132 | 241.882699 |
| YHL014C | YLF2 | 0.64540055 | 0.99917121 | -0.1294654 | 112.4849638 | 194.9001626 |
| YFL032W | YFL032W | 0.39827442 | 0.99917121 | -0.1292376 | 131.4889016 | 225.133561 |
| YBR078W | ECM33 | 0.51191894 | 0.99917121 | -0.1289809 | 121.3171986 | 208.9052182 |
| YCL045C | YCL045C | 0.40420279 | 0.99917121 | -0.1282993 | 143.2273614 | 243.7110661 |
| YIL170W | HXT12 | 0.43944154 | 0.99917121 | -0.1281273 | 142.8697849 | 243.1207023 |
| YJR062C | NTA1 | 0.45254126 | 0.99917121 | -0.1274272 | 125.1011412 | 214.7412147 |
| YKL188C | PXA2 | 0.53941732 | 0.99917121 | -0.12737 | 131.0216087 | 224.1617832 |
| YIR027C | DAL1 | 0.5486889 | 0.99917121 | -0.1271713 | 135.7313342 | 231.6371509 |
| YIL032C | YIL032C | 0.47283389 | 0.99917121 | -0.126968 | 146.2783657 | 248.4070804 |
| YJR096W | YJR096W | 0.61921176 | 0.99917121 | -0.1267829 | 133.2116672 | 227.5775743 |
| YNL223W | ATG4 | 0.40750635 | 0.99917121 | -0.1264724 | 103.5320988 | 180.2790676 |
| YCR053W | THR4 | 0.59300778 | 0.99917121 | -0.1264192 | 13.17730764 | 36.3949173 |
| YLR047C | FRE8 | 0.60687657 | 0.99917121 | -0.1264014 | 91.38649536 | 160.9301886 |
| YML029W | USA1 | 0.56548205 | 0.99917121 | -0.1262286 | 138.0260302 | 235.1762225 |
| YAL004W | YAL004W | 0.37727645 | 0.99917121 | -0.1260449 | 121.1206706 | 208.2343485 |
| YMR195W | ICY1 | 0.55949449 | 0.99917121 | -0.1258949 | 136.0067846 | 231.9201672 |
| YHR143W | DSE2 | 0.60274977 | 0.99917121 | -0.125758 | 151.1059218 | 255.9467839 |
| YDR387C | YDR387C | 0.48322626 | 0.99917121 | -0.1257576 | 141.0240473 | 239.8927336 |
| YDR335W | MSN5 | 0.44913596 | 0.99917121 | -0.1256382 | 134.6774895 | 229.7721514 |
| YJL021C | YJL021C | 0.47848614 | 0.99917121 | -0.1255909 | 145.2228441 | 246.5584136 |
| YCL026C | YCL026C | 0.53229845 | 0.99917121 | -0.1254933 | 127.1477922 | 217.76447 |
| YCL056C | YCL056C | 0.40605462 | 0.99917121 | -0.1254832 | 140.7315817 | 239.3935638 |
| YGR292W | MAL12 | 0.51672529 | 0.99917121 | -0.1254159 | 137.1626453 | 233.7023153 |
| YDR392W | SPT3 | 0.43091246 | 0.99917121 | -0.1253546 | 97.31614008 | 170.2447284 |
| YPL018W | CTF19 | 0.62526109 | 0.99917121 | -0.1253439 | 129.2268921 | 221.0569406 |
| YOL049W | GSH2 | 0.8533941 | 0.99917121 | -0.125126 | 80.20656558 | 142.9722038 |
| YOR344C | TYE7 | 0.57827919 | 0.99917121 | -0.1249497 | 113.692664 | 196.2727435 |
| YKR034W | DAL80 | 0.25489988 | 0.99917121 | -0.1248626 | 105.1278671 | 182.6238505 |
| YDR120C | TRM1 | 0.35021172 | 0.99917121 | -0.1247551 | 131.4311176 | 224.4950811 |
| YIR031C | DAL7 | 0.58627931 | 0.99917121 | -0.1245861 | 137.8038141 | 234.622136 |
| YGR111W | YGR111W | 0.79069859 | 0.99917121 | -0.1244215 | 95.83337231 | 167.7698641 |
| YPL236C | YPL236C | 0.48160506 | 0.99917121 | -0.1241907 | 144.862253 | 245.8135206 |
| YGL208W | SIP2 | 0.10337255 | 0.94753174 | -0.1234636 | 112.3100923 | 193.8900188 |

| | | | | | | |
|---|---|---|---|---|---|---|
| YPL037C | EGD1 | 0.6053348 | 0.99917121 | -0.1221895 | 127.4973569 | 217.9183318 |
| YPR201W | ARR3 | 0.69138853 | 0.99917121 | -0.1221742 | 116.1986366 | 199.9248032 |
| YJL020C | BBC1 | 0.56192332 | 0.99917121 | -0.1220858 | 142.1215613 | 241.1927391 |
| YDR003W | RCR2 | 0.49364872 | 0.99917121 | -0.121806 | 148.8050683 | 251.801196 |
| YJR005W | APL1 | 0.55776095 | 0.99917121 | -0.1217523 | 140.8211832 | 239.0814056 |
| YOR028C | CIN5 | 0.55144569 | 0.99917121 | -0.1215337 | 136.1588664 | 231.6306556 |
| YOL101C | IZH4 | 0.59469748 | 0.99917121 | -0.1214965 | 134.9546509 | 229.7085704 |
| YNL338W | YNL338W | 0.57979397 | 0.99917121 | -0.1214625 | 132.211 | 225.3355338 |
| YIL167W | SDL1 | 0.55436697 | 0.99917121 | -0.1210259 | 124.9229685 | 213.6771222 |
| YDR221W | GTB1 | 0.45691985 | 0.99917121 | -0.1209699 | 140.3274855 | 238.1998781 |
| YDR181C | SAS4 | 0.39932037 | 0.99917121 | -0.1209531 | 153.4412018 | 259.0796282 |
| YDR259C | YAP6 | 0.44502972 | 0.99917121 | -0.1208384 | 135.2402369 | 230.0830977 |
| YDR502C | SAM2 | 0.54071984 | 0.99917121 | -0.1204929 | 138.2276632 | 234.7980425 |
| YPL205C | YPL205C | 0.73047388 | 0.99917121 | -0.1202597 | 14.16296387 | 37.21353359 |
| YPR030W | CSR2 | 0.45909542 | 0.99917121 | -0.1200971 | 121.8476674 | 208.6668865 |
| YNL157W | IGO1 | 0.36420759 | 0.99917121 | -0.1200358 | 147.2419823 | 249.0963953 |
| YDR406W | PDR15 | 0.3969935 | 0.99917121 | -0.1198872 | 150.4962422 | 254.2602441 |
| YBR280C | SAF1 | 0.43157668 | 0.99917121 | -0.1195109 | 141.6724711 | 240.163716 |
| YIL113W | SDP1 | 0.56112139 | 0.99917121 | -0.1190771 | 139.7178145 | 236.9983119 |
| YOR165W | SEY1 | 0.60751329 | 0.99917121 | -0.1189936 | 136.3173829 | 231.5734056 |
| YOL001W | PHO80 | 0.46969449 | 0.99917121 | -0.1188996 | 87.89587431 | 154.4573056 |
| YPL081W | RPS9A | 0.58366111 | 0.99917121 | -0.1187932 | 132.6261073 | 225.6711191 |
| YOR231W | MKK1 | 0.51418166 | 0.99917121 | -0.1186022 | 148.3855524 | 250.7425969 |
| YHR044C | DOG1 | 0.51448662 | 0.99917121 | -0.11851 | 133.6977454 | 227.3430295 |
| YDR077W | SED1 | 0.31034031 | 0.99917121 | -0.1182643 | 152.8050086 | 257.7387833 |
| YGL254W | FZF1 | 0.60475784 | 0.99917121 | -0.118058 | 118.1018268 | 202.4535626 |
| YFL033C | RIM15 | 0.6419317 | 0.99917121 | -0.1179999 | 106.7577666 | 184.3826077 |
| YMR057C | YMR057C | 0.49573957 | 0.99917121 | -0.1179835 | 114.7564927 | 197.1174869 |
| YKL071W | YKL071W | 0.57653856 | 0.99917121 | -0.1177273 | 138.165581 | 234.3620344 |
| YIL164C | NIT1 | 0.53644818 | 0.99917121 | -0.1173158 | 139.7465939 | 236.8294168 |
| YOR288C | MPD1 | 0.47316685 | 0.99917121 | -0.1171289 | 129.9685569 | 221.2364449 |
| YCL035C | GRX1 | 0.62475318 | 0.99917121 | -0.1170442 | 139.7654348 | 236.8263011 |
| YPL133C | RDS2 | 0.50030725 | 0.99917121 | -0.1169101 | 134.2177501 | 227.9760318 |
| YNL127W | FAR11 | 0.6070702 | 0.99917121 | -0.1165883 | 139.4144964 | 236.2119033 |
| YMR317W | YMR317W | 0.76450016 | 0.99917121 | -0.1164459 | 115.2322383 | 197.687602 |
| YNL129W | NRK1 | 0.61510238 | 0.99917121 | -0.1161621 | 137.1971428 | 232.6291129 |
| YJL043W | YJL043W | 0.46365361 | 0.99917121 | -0.1161064 | 128.5720349 | 218.8880227 |
| YLR343W | GAS2 | 0.42255482 | 0.99917121 | -0.1160284 | 106.4566534 | 183.6627828 |
| YOR139C | YOR139C | 0.18710166 | 0.99917121 | -0.1154661 | 0 | 14.07654881 |
| YOR285W | YOR285W | 0.48415467 | 0.99917121 | -0.1153321 | 133.664036 | 226.9019379 |
| YBR164C | ARL1 | 0.47071969 | 0.99917121 | -0.1150671 | 139.5061272 | 236.1723597 |
| YLR093C | NYV1 | 0.47294167 | 0.99917121 | -0.1149061 | 100.7268248 | 174.4019877 |
| YKR020W | VPS51 | 0.32290459 | 0.99917121 | -0.1148803 | 99.05171065 | 171.7314594 |
| YBR177C | EHT1 | 0.54112439 | 0.99917121 | -0.1146593 | 137.2024527 | 232.4543643 |
| YOR083W | WHI5 | 0.46858126 | 0.99917121 | -0.1145926 | 117.785334 | 201.5271223 |
| YOR238W | YOR238W | 0.56984338 | 0.99917121 | -0.1144181 | 137.6376863 | 233.1180034 |
| YDR095C | YDR095C | 0.46681055 | 0.99917121 | -0.1137192 | 153.3270516 | 258.0159771 |
| YFR020W | YFR020W | 0.46274849 | 0.99917121 | -0.1136481 | 144.5292605 | 243.9980238 |
| YJL126W | NIT2 | 0.53740141 | 0.99917121 | -0.1134882 | 138.6806156 | 234.6653655 |
| YDR022C | CIS1 | 0.50189026 | 0.99917121 | -0.1132908 | 136.3190365 | 230.8808094 |
| YIR024C | YIR024C | 0.57853044 | 0.99917121 | -0.1130683 | 143.9491019 | 243.0035211 |
| YOR156C | NFI1 | 0.52058308 | 0.99917121 | -0.1127618 | 137.5621486 | 232.795808 |
| YOR376W | YOR376W | 0.57913623 | 0.99917121 | -0.1122135 | 136.9199585 | 231.7063551 |
| YFL012W | YFL012W | 0.71242896 | 0.99917121 | -0.1120393 | 127.351263 | 216.4482819 |
| YMR283C | RIT1 | 0.59796749 | 0.99917121 | -0.1116409 | 140.7463436 | 237.7295449 |
| YDL090C | RAM1 | 0.71338664 | 0.99917121 | -0.1115695 | 115.2672927 | 197.148937 |
| YAL051W | OAF1 | 0.51586252 | 0.99917121 | -0.1115406 | 149.3634135 | 251.4388296 |
| YPL137C | GIP3 | 0.5025185 | 0.99917121 | -0.1113382 | 124.5375905 | 211.8824159 |
| YNL334C | SNO2 | 0.78286971 | 0.99917121 | -0.1110988 | 103.8706816 | 178.9440091 |
| YLR059C | REX2 | 0.37552677 | 0.99917121 | -0.1106413 | 105.6724463 | 181.757291 |
| YDL187C | YDL187C | 0.63608675 | 0.99917121 | -0.110623 | 139.5994286 | 235.7791515 |
| YFR013W | IOC3 | 0.70736243 | 0.99917121 | -0.1104444 | 116.8286291 | 199.497994 |
| YOR318C | YOR318C | 0.58112693 | 0.99917121 | -0.1101782 | 134.5577429 | 227.6967265 |
| YDR252W | BTT1 | 0.49549847 | 0.99917121 | -0.1096757 | 139.0743053 | 234.8274739 |

| | | | | | | |
|---|---|---|---|---|---|---|
| YOR107W | RGS2 | 0.58188317 | 0.99917121 | -0.1095712 | 119.4067662 | 203.4968714 |
| YMR300C | ADE4 | 0.62093633 | 0.99917121 | -0.1094533 | 131.6184299 | 222.9278978 |
| YDR415C | YDR415C | 0.40594773 | 0.99917121 | -0.1094021 | 142.8152313 | 240.7510365 |
| YNR057C | BIO4 | 0.40171308 | 0.99917121 | -0.1093525 | 145.1140569 | 244.4055569 |
| YMR247C | RKR1 | 0.56613032 | 0.99917121 | -0.1093238 | 132.683698 | 224.6084091 |
| YOL013C | HRD1 | 0.50132098 | 0.99917121 | -0.1093141 | 139.0839818 | 234.7988068 |
| YMR285C | NGL2 | 0.58841644 | 0.99917121 | -0.1092779 | 142.3772094 | 240.0384038 |
| YIR036C | IRC24 | 0.54532402 | 0.99917121 | -0.1091236 | 137.9757614 | 233.0108851 |
| YBR072W | HSP26 | 0.29966416 | 0.99917121 | -0.1086397 | 148.3720727 | 249.5065995 |
| YJL134W | LCB3 | 0.59381617 | 0.99917121 | -0.1085756 | 138.4485544 | 233.6969407 |
| YGL042C | YGL042C | 0.69696657 | 0.99917121 | -0.1085126 | 111.0607379 | 190.0778968 |
| YER031C | YPT31 | 0.69522797 | 0.99917121 | -0.1083579 | 132.7350089 | 224.5723534 |
| YKL037W | YKL037W | 0.30841876 | 0.99917121 | -0.1083383 | 127.8801992 | 216.8393455 |
| YOR242C | SSP2 | 0.41475642 | 0.99917121 | -0.1082613 | 133.1512476 | 225.2233867 |
| YIR007W | YIR007W | 0.64911342 | 0.99917121 | -0.1079609 | 130.1640574 | 220.4300752 |
| YKR059W | TIF1 | 0.49946114 | 0.99917121 | -0.1075938 | 136.6951923 | 230.7852644 |
| YJR078W | BNA2 | 0.5582099 | 0.99917121 | -0.1070721 | 145.7146927 | 245.0839827 |
| YMR259C | YMR259C | 0.63611 | 0.99917121 | -0.1067525 | 133.4522742 | 225.5187898 |
| YNL123W | NMA111 | 0.71185813 | 0.99917121 | -0.106741 | 117.7725037 | 200.549492 |
| YLR306W | UBC12 | 0.64465693 | 0.99917121 | -0.1062973 | 121.1286588 | 205.8396191 |
| YKR024C | DBP7 | 0.43454387 | 0.99917121 | -0.1059432 | 16.79597376 | 39.66090747 |
| YDR483W | KRE2 | 0.55777655 | 0.99917121 | -0.1059178 | 118.2042974 | 201.1367165 |
| YER124C | DSE1 | 0.6282678 | 0.99917121 | -0.1056523 | 132.1733625 | 223.3481692 |
| YLR146C | SPE4 | 0.61787289 | 0.99917121 | -0.105367 | 112.8319045 | 192.5147619 |
| YFR024C | YFR024C | 0.84504763 | 0.99917121 | -0.1046404 | 90.44770616 | 156.7824054 |
| YPL099C | FMP14 | 0.88166955 | 0.99917121 | -0.1043989 | 102.2773802 | 175.5901017 |
| YMR302C | YME2 | 0.45784646 | 0.99917121 | -0.1043484 | 145.9715352 | 245.1609171 |
| YDR214W | AHA1 | 0.55274393 | 0.99917121 | -0.1041154 | 138.2527886 | 232.8414607 |
| YKR061W | KTR2 | 0.60204843 | 0.99917121 | -0.1041072 | 135.7795446 | 228.9021551 |
| YKR001C | VPS1 | 0.45629349 | 0.99917121 | -0.1040004 | 107.8825627 | 184.4670096 |
| YOR228C | YOR228C | 0.55016676 | 0.99917121 | -0.1037379 | 130.7727053 | 220.8844283 |
| YJL068C | YJL068C | 0.59386328 | 0.99917121 | -0.1037185 | 143.2799567 | 240.7981582 |
| YDR155C | CPR1 | 0.49065536 | 0.99917121 | -0.1037085 | 137.7272429 | 231.9550042 |
| YOR313C | SPS4 | 0.56102914 | 0.99917121 | -0.1035477 | 130.7429277 | 220.8138274 |
| YML053C | YML053C | 0.60084349 | 0.99917121 | -0.1033167 | 139.0344861 | 233.9888401 |
| YGR086C | PIL1 | 0.45533197 | 0.99917121 | -0.1031894 | 110.162623 | 187.9988242 |
| YKL127W | PGM1 | 0.56054477 | 0.99917121 | -0.1031592 | 136.6233698 | 230.1302701 |
| YNL098C | RAS2 | 0.52916046 | 0.99917121 | -0.103038 | 132.8352532 | 224.0834341 |
| YCL057W | PRD1 | 0.46926266 | 0.99917121 | -0.1028043 | 142.4170258 | 239.3126094 |
| YKL008C | LAC1 | 0.58944618 | 0.99917121 | -0.1027714 | 135.3685683 | 228.0848918 |
| YKR098C | UBP11 | 0.36618493 | 0.99917121 | -0.1024695 | 108.8452112 | 185.8132566 |
| YHR047C | AAP1 | 0.62808824 | 0.99917121 | -0.1024108 | 137.1858648 | 230.9347237 |
| YBR284W | YBR284W | 0.61296374 | 0.99917121 | -0.1021836 | 125.2806633 | 211.9496131 |
| YNL054W | VAC7 | 0.59738698 | 0.99917121 | -0.1020381 | 139.4819956 | 234.54556 |
| YKR101W | SIR1 | 0.48291138 | 0.99917121 | -0.1018581 | 142.9506363 | 240.0469594 |
| YCL046W | YCL046W | 0.55681807 | 0.99917121 | -0.101791 | 145.3299296 | 243.827476 |
| YML054C | CYB2 | 0.56316072 | 0.99917121 | -0.1017328 | 127.3103025 | 215.1265845 |
| YOL117W | RRI2 | 0.65782698 | 0.99917121 | -0.1015952 | 123.1475422 | 208.4811816 |
| YHL025W | SNF6 | 0.81606322 | 0.99917121 | -0.1015805 | 82.84456371 | 144.3024045 |
| YDL226C | GCS1 | 0.69640663 | 0.99917121 | -0.1015363 | 109.4888589 | 186.7244203 |
| YKL200C | YKL200C | 0.65978323 | 0.99917121 | -0.1015087 | 133.6162547 | 225.1406338 |
| YGR260W | TNA1 | 0.59152714 | 0.99917121 | -0.1012115 | 142.3481062 | 239.008687 |
| YLL013C | PUF3 | 0.54765374 | 0.99917121 | -0.1011747 | 139.6949308 | 234.7793717 |
| YNL238W | KEX2 | 0.85044213 | 0.99917121 | -0.1006461 | 73.12862308 | 128.7171768 |
| YJR135C | MCM22 | 0.53658663 | 0.99917121 | -0.1005267 | 136.5732389 | 229.7295133 |
| YDL054C | MCH1 | 0.58446343 | 0.99917121 | -0.1004734 | 131.8841224 | 222.2562371 |
| YBR132C | AGP2 | 0.67882334 | 0.99917121 | -0.1002854 | 7.812016206 | 24.66542803 |
| YMR320W | YMR320W | 0.63754472 | 0.99917121 | -0.1001964 | 140.0565064 | 235.2358709 |
| YPR155C | NCA2 | 0.71232205 | 0.99917121 | -0.1000695 | 122.0863467 | 206.6053771 |
| YBR178W | YBR178W | 0.59564614 | 0.99917121 | -0.1000387 | 148.3026822 | 248.3475562 |
| YDL172C | YDL172C | 0.53428507 | 0.99917121 | -0.1000253 | 141.4581706 | 237.4469716 |
| YHR050W | SMF2 | 0.63658445 | 0.99917121 | -0.0999393 | 144.4606671 | 242.2175451 |
| YOR279C | RFM1 | 0.57768124 | 0.99917121 | -0.0998108 | 139.4306785 | 234.1923147 |
| YPL015C | HST2 | 0.7174635 | 0.99917121 | -0.0994664 | 125.5175765 | 211.9956183 |

| | | | | | | |
|---|---|---|---|---|---|---|
| YHL026C | YHL026C | 0.76378979 | 0.99917121 | -0.0994288 | 121.6793139 | 205.8791215 |
| YIL159W | BNR1 | 0.6133084 | 0.99917121 | -0.0993469 | 148.5976103 | 248.7328459 |
| YJL216C | YJL216C | 0.55084449 | 0.99917121 | -0.099213 | 139.0080319 | 233.4464251 |
| YOR062C | YOR062C | 0.69370161 | 0.99917121 | -0.0989217 | 129.2119252 | 217.8119579 |
| YCL016C | DCC1 | 0.49711048 | 0.99917121 | -0.0989098 | 102.0772057 | 174.6021648 |
| YHL027W | RIM101 | 0.59972629 | 0.99917121 | -0.0987128 | 134.5852737 | 226.3428173 |
| YBR020W | GAL1 | 0.6325603 | 0.99917121 | -0.0986756 | 125.3634273 | 211.6537456 |
| YHL021C | FMP12 | 0.75417816 | 0.99917121 | -0.0983018 | 128.3301302 | 216.3322417 |
| YJL193W | YJL193W | 0.7110572 | 0.99917121 | -0.0981311 | 133.1104334 | 223.923414 |
| YJL024C | APS3 | 0.68769921 | 0.99917121 | -0.0980839 | 113.4063772 | 192.5416379 |
| YIR032C | DAL3 | 0.64742106 | 0.99917121 | -0.0975441 | 145.3520911 | 243.3450225 |
| YMR221C | FMP42 | 0.64851607 | 0.99917121 | -0.0973279 | 136.1986094 | 228.7429928 |
| YNL043C | YNL043C | 0.60168349 | 0.99917121 | -0.0967314 | 134.5541335 | 226.0516727 |
| YDL200C | MGT1 | 0.56406636 | 0.99917121 | -0.0960372 | 148.4996285 | 248.1733436 |
| YNL203C | YNL203C | 0.68909294 | 0.99917121 | -0.0959602 | 131.5267897 | 221.1370252 |
| YER134C | YER134C | 0.64651201 | 0.99917121 | -0.0958246 | 134.4520112 | 225.7785022 |
| YBR223C | TDP1 | 0.57425562 | 0.99917121 | -0.0957071 | 150.6139259 | 251.4998285 |
| YER078C | YER078C | 0.65294566 | 0.99917121 | -0.0956362 | 129.1980219 | 217.389276 |
| YDR334W | SWR1 | 0.63465086 | 0.99917121 | -0.0953974 | 132.5509824 | 222.699295 |
| YKR069W | MET1 | 0.54774949 | 0.99917121 | -0.0953069 | 144.8454571 | 242.2655351 |
| YFL014W | HSP12 | 0.56366623 | 0.99917121 | -0.0952866 | 140.4079478 | 235.1969357 |
| YMR182C | RGM1 | 0.65501102 | 0.99917121 | -0.0951978 | 138.246191 | 231.7438076 |
| YGR288W | MAL13 | 0.6147815 | 0.99917121 | -0.0951748 | 143.397619 | 239.9439467 |
| YDR161W | YDR161W | 0.59741253 | 0.99917121 | -0.0951144 | 133.2244707 | 223.7372409 |
| YPL100W | ATG21 | 0.70055733 | 0.99917121 | -0.0945884 | 135.9759941 | 228.054535 |
| YAR043C | YAR043C | 0.57271173 | 0.99917121 | -0.0945717 | 140.7850412 | 235.7102519 |
| YJL062W | LAS21 | 0.61494956 | 0.99917121 | -0.0945211 | 123.2749093 | 207.8215832 |
| YKL150W | MCR1 | 0.60615347 | 0.99917121 | -0.0943836 | 142.5584821 | 238.5112789 |
| YDL046W | NPC2 | 0.43032475 | 0.99917121 | -0.0938901 | 148.3691079 | 247.7037466 |
| YPL024W | RMI1 | 0.65368468 | 0.99917121 | -0.0938594 | 101.6993558 | 173.3848034 |
| YBR016W | YBR016W | 0.49648218 | 0.99917121 | -0.0937572 | 130.568305 | 219.3422005 |
| YJR035W | RAD26 | 0.65642719 | 0.99917121 | -0.0937396 | 134.5401057 | 225.6645977 |
| YGL175C | SAE2 | 0.48759291 | 0.99917121 | -0.0935602 | 146.7740918 | 245.1236819 |
| YLR427W | MAG2 | 0.61285367 | 0.99917121 | -0.0935266 | 134.0731562 | 224.8950843 |
| YDL082W | RPL13A | 0.36819369 | 0.99917121 | -0.0934475 | 100.3443605 | 171.1769399 |
| YOR283W | YOR283W | 0.60197424 | 0.99917121 | -0.0933984 | 132.5295978 | 222.421544 |
| YGL243W | TAD1 | 0.61054369 | 0.99917121 | -0.0933317 | 136.3850222 | 228.5526502 |
| YDR345C | HXT3 | 0.55817112 | 0.99917121 | -0.0931993 | 145.7382396 | 243.4302321 |
| YJR060W | CBF1 | 0.56695931 | 0.99917121 | -0.0930393 | 135.9478949 | 227.8209351 |
| YDL204W | RTN2 | 0.80044183 | 0.99917121 | -0.0927965 | 141.3694536 | 236.4244334 |
| YOL108C | INO4 | 0.49554017 | 0.99917121 | -0.0927322 | 90.80645945 | 155.9019288 |
| YOL122C | SMF1 | 0.6428405 | 0.99917121 | -0.0925542 | 123.164383 | 207.4057997 |
| YDL160C | DHH1 | 0.43366175 | 0.99917121 | -0.0924318 | 153.7584026 | 256.1076823 |
| YDR279W | RNH202 | 0.5945646 | 0.99917121 | -0.092101 | 134.1772832 | 224.8870964 |
| YOR185C | GSP2 | 0.7607395 | 0.99917121 | -0.091775 | 130.4776435 | 218.9561823 |
| YFR019W | FAB1 | 0.63575522 | 0.99917121 | -0.0915301 | 144.6699693 | 241.5256634 |
| YOL043C | NTG2 | 0.78811911 | 0.99917121 | -0.0914556 | 124.6793066 | 209.6841825 |
| YKR096W | YKR096W | 0.50318162 | 0.99917121 | -0.0913689 | 136.7513579 | 228.8967033 |
| YCR082W | AHC2 | 0.59031871 | 0.99917121 | -0.0912543 | 151.3927962 | 252.1972289 |
| YOL124C | TRM11 | 0.67560679 | 0.99917121 | -0.0910514 | 113.9220302 | 192.5054116 |
| YDL019C | OSH2 | 0.7053624 | 0.99917121 | -0.0909201 | 146.4768889 | 244.328567 |
| YKL040C | NFU1 | 0.60346439 | 0.99917121 | -0.0907237 | 134.5362767 | 225.2908386 |
| YDR359C | VID21 | 0.88302855 | 0.99917121 | -0.0905541 | 76.6144872 | 133.0376179 |
| YPL253C | VIK1 | 0.6161556 | 0.99917121 | -0.0904809 | 135.4427746 | 226.7047043 |
| YML057W | CMP2 | 0.50163619 | 0.99917121 | -0.0904644 | 123.787442 | 208.1431666 |
| YKL100C | YKL100C | 0.57969228 | 0.99917121 | -0.0902575 | 144.6904242 | 241.4030981 |
| YGR044C | RME1 | 0.47037341 | 0.99917121 | -0.0900436 | 137.743067 | 230.3142969 |
| YPR037C | ERV2 | 0.48278717 | 0.99917121 | -0.0900067 | 143.3296513 | 239.2056759 |
| YMR188C | MRPS17 | 0.66400508 | 0.99917121 | -0.089992 | 129.470157 | 217.1345282 |
| YJR033C | RAV1 | 0.65840243 | 0.99917121 | -0.0897387 | 131.7380608 | 220.714976 |
| YPL038W | MET31 | 0.63954699 | 0.99917121 | -0.0897224 | 127.7679848 | 214.3911814 |
| YNL022C | YNL022C | 0.55107338 | 0.99917121 | -0.0895077 | 140.2048742 | 234.1690641 |
| YML047C | PRM6 | 0.74790947 | 0.99917121 | -0.0893127 | 95.62772761 | 163.1622729 |
| YJL152W | YJL152W | 0.61142147 | 0.99917121 | -0.0891776 | 141.7130141 | 236.5303214 |

| | | | | | | |
|---|---|---|---|---|---|---|
| YLL015W | BPT1 | 0.59146679 | 0.99917121 | -0.0891692 | 140.6213973 | 234.7910515 |
| YJR099W | YUH1 | 0.59940993 | 0.99917121 | -0.088954 | 147.3944618 | 245.549988 |
| YMR232W | FUS2 | 0.68342537 | 0.99917121 | -0.0887117 | 130.9057605 | 219.264451 |
| YDR321W | ASP1 | 0.553425 | 0.99917121 | -0.0886298 | 142.0653034 | 237.0245079 |
| YDR157W | YDR157W | 0.60737311 | 0.99917121 | -0.0886283 | 144.180151 | 240.3919327 |
| YGL067W | NPY1 | 0.59037049 | 0.99917121 | -0.0886088 | 136.0717846 | 227.4780898 |
| YOL099C | YOL099C | 0.65380738 | 0.99917121 | -0.0885388 | 137.7992075 | 230.2202457 |
| YCR088W | ABP1 | 0.55387348 | 0.99917121 | -0.0885131 | 157.0282754 | 260.8367698 |
| YFL043C | YFL043C | 0.71097429 | 0.99917121 | -0.0884979 | 123.4625848 | 207.3861397 |
| YMR266W | RSN1 | 0.67491123 | 0.99917121 | -0.08803 | 141.2780322 | 235.6977711 |
| YIL160C | POT1 | 0.63527007 | 0.99917121 | -0.0879093 | 124.122975 | 208.3659709 |
| YBR278W | DPB3 | 0.60715201 | 0.99917121 | -0.0876687 | 132.1775883 | 221.162505 |
| YLR053C | YLR053C | 0.3219552 | 0.99917121 | -0.0873442 | 109.0934333 | 184.3645825 |
| YMR313C | TGL3 | 0.74557835 | 0.99917121 | -0.0872172 | 108.1556637 | 182.8558375 |
| YKR017C | YKR017C | 0.71127212 | 0.99917121 | -0.0871852 | 123.0319533 | 206.5403906 |
| YJR009C | TDH2 | 0.56100448 | 0.99917121 | -0.087084 | 137.1936453 | 229.0786182 |
| YCR036W | RBK1 | 0.74020955 | 0.99917121 | -0.0870808 | 116.9605522 | 196.8597853 |
| YDR143C | SAN1 | 0.50278716 | 0.99917121 | -0.086515 | 154.3590767 | 256.3428493 |
| YJR070C | LIA1 | 0.6457883 | 0.99917121 | -0.0864912 | 125.4696882 | 210.3375432 |
| YPL025C | YPL025C | 0.57510416 | 0.99917121 | -0.0864888 | 140.8276922 | 234.7927767 |
| YDR444W | YDR444W | 0.89300111 | 0.99917121 | -0.0862078 | 94.94244185 | 161.6925197 |
| YJR001W | AVT1 | 0.56664375 | 0.99917121 | -0.0861922 | 149.6900624 | 248.8687298 |
| YBR281C | DUG2 | 0.71577595 | 0.99917121 | -0.0860141 | 122.659832 | 205.8050699 |
| YNL117W | MLS1 | 0.70755354 | 0.99917121 | -0.0859582 | 141.5720181 | 235.9133219 |
| YJL045W | YJL045W | 0.6116093 | 0.99917121 | -0.0858664 | 140.3164505 | 233.9028121 |
| YGR022C | YGR022C | 0.53106832 | 0.99917121 | -0.0857263 | 107.8569526 | 182.1984156 |
| YFR035C | YFR035C | 0.63762763 | 0.99917121 | -0.0854864 | 137.9678897 | 230.1167257 |
| YCR068W | ATG15 | 0.70489581 | 0.99917121 | -0.085358 | 129.3710792 | 216.4118262 |
| YGR270W | YTA7 | 0.72420864 | 0.99917121 | -0.0851157 | 103.3031023 | 174.8726008 |
| YOR322C | LDB19 | 0.72777561 | 0.99917121 | -0.0849924 | 115.4961587 | 194.2733348 |
| YHR092C | HXT4 | 0.72361552 | 0.99917121 | -0.0849793 | 150.1750861 | 249.4932003 |
| YLR128W | DCN1 | 0.70359788 | 0.99917121 | -0.0849537 | 117.9363049 | 198.1542217 |
| YOR099W | KTR1 | 0.68190392 | 0.99917121 | -0.0849198 | 135.8983484 | 226.7521914 |
| YOR214C | YOR214C | 0.61856584 | 0.99917121 | -0.084779 | 137.3988114 | 229.1243025 |
| YPL135W | ISU1 | 0.65513494 | 0.99917121 | -0.084388 | 140.2762161 | 233.6585114 |
| YCL010C | SGF29 | 0.39100222 | 0.99917121 | -0.0837018 | 0 | 10.20413957 |
| YDR403W | DIT1 | 0.57282506 | 0.99917121 | -0.0829399 | 141.1376429 | 234.853686 |
| YNL231C | PDR16 | 0.67155854 | 0.99917121 | -0.0829323 | 104.2669514 | 176.1412099 |
| YOL054W | PSH1 | 0.70341298 | 0.99917121 | -0.0829065 | 121.1697589 | 203.0534752 |
| YPL195W | APL5 | 0.70152084 | 0.99917121 | -0.0828934 | 120.5928564 | 202.1332464 |
| YNL064C | YDJ1 | 0.4095507 | 0.99917121 | -0.0828689 | 66.91286455 | 116.6522057 |
| YNL009W | IDP3 | 0.62877277 | 0.99917121 | -0.0828637 | 152.4060321 | 252.7877585 |
| YPL115C | BEM3 | 0.64950944 | 0.99917121 | -0.0825753 | 135.3820322 | 225.6442055 |
| YPR114W | YPR114W | 0.68311221 | 0.99917121 | -0.0823061 | 112.8359628 | 189.7098501 |
| YNL305C | YNL305C | 0.61454267 | 0.99917121 | -0.0820155 | 92.10707529 | 156.6664979 |
| YNL032W | SIW14 | 0.67368464 | 0.99917121 | -0.0815745 | 139.7501657 | 232.4778545 |
| YBR285W | YBR285W | 0.60240299 | 0.99917121 | -0.0814226 | 144.802008 | 240.5037094 |
| YDR282C | YDR282C | 0.65195382 | 0.99917121 | -0.0810305 | 142.1840831 | 236.287219 |
| YOR342C | YOR342C | 0.69813914 | 0.99917121 | -0.0808044 | 136.9217628 | 227.880126 |
| YER066W | YER066W | 0.49813635 | 0.99917121 | -0.0806855 | 118.2662251 | 198.15923 |
| YPL240C | HSP82 | 0.67694714 | 0.99917121 | -0.0806089 | 126.8491913 | 211.8170939 |
| YDR307W | YDR307W | 0.66513868 | 0.99917121 | -0.08051 | 142.5259376 | 236.7681201 |
| YMR307W | GAS1 | 0.87023965 | 0.99917121 | -0.0803855 | 77.96968082 | 133.9559192 |
| YKR045C | YKR045C | 0.67157726 | 0.99917121 | -0.080331 | 139.2679226 | 231.5583535 |
| YDR144C | MKC7 | 0.62709266 | 0.99917121 | -0.0801361 | 140.6563551 | 233.7454813 |
| YKL020C | SPT23 | 0.614154 | 0.99917121 | -0.0797962 | 140.0886251 | 232.8000168 |
| YFL023W | BUD27 | 0.79767497 | 0.99917121 | -0.0797252 | 104.2219622 | 175.6785999 |
| YJR153W | PGU1 | 0.63899729 | 0.99917121 | -0.0795349 | 139.5497233 | 231.9100335 |
| YGL041C | YGL041C | 0.53391105 | 0.99917121 | -0.0792044 | 142.8948867 | 237.196448 |
| YBL055C | YBL055C | 0.52303499 | 0.99917121 | -0.0789754 | 139.3328567 | 231.4964841 |
| YCL034W | LSB5 | 0.63011034 | 0.99917121 | -0.0789642 | 150.6617583 | 249.5348522 |
| YPL101W | ELP4 | 0.75094819 | 0.99917121 | -0.0784796 | 104.1056691 | 175.3415576 |
| YNL195C | YNL195C | 0.70498842 | 0.99917121 | -0.0783293 | 141.386194 | 234.6873844 |
| YDL203C | ACK1 | 0.63129106 | 0.99917121 | -0.0782631 | 141.5302158 | 234.9086422 |

| | | | | | | |
|---|---|---|---|---|---|---|
| YJR108W | ABM1 | 0.60718986 | 0.99917121 | -0.0781514 | 132.2108066 | 220.0551462 |
| YOR343C | YOR343C | 0.80199431 | 0.99917121 | -0.0780767 | 137.28645 | 228.1283015 |
| YIL006W | YIA6 | 0.83435412 | 0.99917121 | -0.0777333 | 135.8665442 | 225.8254358 |
| YBL043W | ECM13 | 0.61951561 | 0.99917121 | -0.077494 | 151.7351661 | 251.0648719 |
| YDR516C | EMI2 | 0.74035584 | 0.99917121 | -0.0773783 | 140.7170592 | 233.5059377 |
| YPR123C | YPR123C | 0.69278433 | 0.99917121 | -0.0773762 | 118.9294962 | 198.81197 |
| YIL100W | YIL100W | 0.65841213 | 0.99917121 | -0.0770922 | 147.3491854 | 244.0318208 |
| YDR384C | ATO3 | 0.66000244 | 0.99917121 | -0.0770656 | 140.8296004 | 233.6470238 |
| YBL056W | PTC3 | 0.70538889 | 0.99917121 | -0.0769352 | 138.0974382 | 229.2805315 |
| YPL086C | ELP3 | 0.67746584 | 0.99917121 | -0.0768125 | 112.4073204 | 188.3575692 |
| YJR080C | FMP26 | 0.76699723 | 0.99917121 | -0.0766892 | 132.5247744 | 220.3768348 |
| YDR475C | JIP4 | 0.74201194 | 0.99917121 | -0.0758096 | 142.0847806 | 235.4926094 |
| YOR222W | ODC2 | 0.67488589 | 0.99917121 | -0.0757671 | 134.2112016 | 222.9498269 |
| YEL071W | DLD3 | 0.6943161 | 0.99917121 | -0.075735 | 141.6742882 | 234.8298619 |
| YKR021W | ALY1 | 0.6352871 | 0.99917121 | -0.0756281 | 131.0687402 | 217.9289456 |
| YHR191C | CTF8 | 0.65414598 | 0.99917121 | -0.075456 | 107.0941899 | 179.7317636 |
| YOL118C | YOL118C | 0.6722778 | 0.99917121 | -0.0752786 | 138.0263153 | 228.9653238 |
| YKL124W | SSH4 | 0.6696544 | 0.99917121 | -0.0748018 | 133.3569414 | 221.4718485 |
| YPR173C | VPS4 | 0.62035131 | 0.99917121 | -0.0747587 | 98.07887634 | 165.2911037 |
| YGL089C | MF(ALPHA)2 | 0.66194679 | 0.99917121 | -0.074627 | 144.4963756 | 239.1885659 |
| YGR052W | FMP48 | 0.63445012 | 0.99917121 | -0.0745471 | 122.2354337 | 203.7313161 |
| YOR071C | THI71 | 0.70306115 | 0.99917121 | -0.0744204 | 131.8112635 | 218.964071 |
| YDR057W | YOS9 | 0.69600637 | 0.99917121 | -0.0744097 | 154.7275926 | 255.4538961 |
| YKL067W | YNK1 | 0.66113716 | 0.99917121 | -0.074204 | 137.9195647 | 228.6643247 |
| YPL227C | ALG5 | 0.63706322 | 0.99917121 | -0.0740703 | 148.4031314 | 245.341684 |
| YDR534C | FIT1 | 0.7336799 | 0.99917121 | -0.0740632 | 141.1274688 | 233.7553206 |
| YPL262W | FUM1 | 0.67900531 | 0.99917121 | -0.0739463 | 107.5736717 | 180.3112198 |
| YNL050C | YNL050C | 0.74327301 | 0.99917121 | -0.0735644 | 115.5025037 | 192.8902514 |
| YJR109C | CPA2 | 0.65384512 | 0.99917121 | -0.0735218 | 130.8883405 | 217.3848949 |
| YLR451W | LEU3 | 0.59219217 | 0.99917121 | -0.0734189 | 110.3640653 | 184.6902547 |
| YCR027C | RHB1 | 0.73394968 | 0.99917121 | -0.073362 | 125.668023 | 209.0527776 |
| YBR145W | ADH5 | 0.64475396 | 0.99917121 | -0.0733397 | 145.3668833 | 240.4178031 |
| YPL003W | ULA1 | 0.69093115 | 0.99917121 | -0.0732322 | 130.7306054 | 217.0984172 |
| YER185W | YER185W | 0.7501405 | 0.99917121 | -0.0731722 | 134.5505591 | 223.1738591 |
| YPL113C | YPL113C | 0.81095714 | 0.99917121 | -0.0731316 | 119.9662221 | 199.9453483 |
| YKL034W | TUL1 | 0.59128261 | 0.99917121 | -0.0731083 | 145.9153368 | 241.2629237 |
| YLR354C | TAL1 | 0.74706583 | 0.99917121 | -0.0730982 | 118.8057597 | 198.0933962 |
| YDR084C | TVP23 | 0.64912327 | 0.99917121 | -0.0729599 | 142.2502639 | 235.4087054 |
| YHR046C | INM1 | 0.66903231 | 0.99917121 | -0.0729296 | 147.7841265 | 244.2169363 |
| YDL131W | LYS21 | 0.68030052 | 0.99917121 | -0.0726274 | 133.6524327 | 221.6773046 |
| YGR247W | CPD1 | 0.74838795 | 0.99917121 | -0.0724798 | 144.2180213 | 238.4835682 |
| YBR226C | YBR226C | 0.6950531 | 0.99917121 | -0.0723363 | 138.6291839 | 229.5666085 |
| YDR056C | YDR056C | 0.66288104 | 0.99917121 | -0.0721346 | 148.1788644 | 244.7485772 |
| YJL101C | GSH1 | 0.92826673 | 0.99917121 | -0.0719739 | 51.33570174 | 90.51948601 |
| YOL019W | YOL019W | 0.70085752 | 0.99917121 | -0.0719525 | 136.7917974 | 226.5940378 |
| YDR351W | SBE2 | 0.65075541 | 0.99917121 | -0.0718708 | 143.7325932 | 237.6363452 |
| YDL128W | VCX1 | 0.61994748 | 0.99917121 | -0.0718665 | 151.979979 | 250.7686542 |
| YJL060W | BNA3 | 0.66166612 | 0.99917121 | -0.0717908 | 139.0037307 | 230.0965195 |
| YJR024C | YJR024C | 0.67383858 | 0.99917121 | -0.0715863 | 151.7199088 | 250.3203647 |
| YJL141C | YAK1 | 0.61876774 | 0.99917121 | -0.0712846 | 137.2807968 | 227.2912752 |
| YJL036W | SNX4 | 0.59857907 | 0.99917121 | -0.0712794 | 138.0602916 | 228.5318849 |
| YCR065W | HCM1 | 0.69267177 | 0.99917121 | -0.0712466 | 124.5553536 | 207.0231114 |
| YDR263C | DIN7 | 0.67054779 | 0.99917121 | -0.0711746 | 143.9893766 | 237.9603577 |
| YBR288C | APM3 | 0.67627973 | 0.99917121 | -0.0709563 | 111.2199434 | 185.7528987 |
| YKL157W | APE2 | 0.6881101 | 0.99917121 | -0.0706671 | 144.9849855 | 239.4838679 |
| YDR198C | RKM2 | 0.5766321 | 0.99917121 | -0.0706244 | 142.0919422 | 234.8718834 |
| YNL099C | OCA1 | 0.8406117 | 0.99917121 | -0.0705037 | 110.8898837 | 185.1721453 |
| YDL149W | ATG9 | 0.80580204 | 0.99917121 | -0.0704539 | 135.8052831 | 224.8404431 |
| YDR422C | SIP1 | 0.68391099 | 0.99917121 | -0.0703387 | 146.3979584 | 241.6937899 |
| YDL050C | YDL050C | 0.69834666 | 0.99917121 | -0.0702817 | 145.9663809 | 240.999612 |
| YOL016C | CMK2 | 0.73936845 | 0.99917121 | -0.0701551 | 136.8224544 | 226.4237253 |
| YBR107C | IML3 | 0.70606374 | 0.99917121 | -0.0698336 | 136.1248293 | 225.2736664 |
| YHR004C | NEM1 | 0.68361905 | 0.99917121 | -0.0696876 | 156.3540802 | 257.4681781 |
| YPL197C | YPL197C | 0.70423547 | 0.99917121 | -0.0696264 | 135.5412917 | 224.3191942 |

| | | | | | | |
|---|---|---|---|---|---|---|
| YBL104C | YBL104C | 0.69616002 | 0.99917121 | -0.0694295 | 151.3621097 | 249.4876865 |
| YDR272W | GLO2 | 0.62569142 | 0.99917121 | -0.0694176 | 146.0024213 | 240.9516684 |
| YPR189W | SKI3 | 0.76752474 | 0.99917121 | -0.0693822 | 119.7124384 | 199.0841417 |
| YDR019C | GCV1 | 0.67998827 | 0.99917121 | -0.0693754 | 135.5980874 | 224.3790336 |
| YDR537C | YDR537C | 0.74123048 | 0.99917121 | -0.0693015 | 129.9948658 | 215.4476642 |
| YNL255C | GIS2 | 0.68123673 | 0.99917121 | -0.069282 | 133.9232363 | 221.7006844 |
| YPL165C | SET6 | 0.72188357 | 0.99917121 | -0.069011 | 133.8525142 | 221.5550305 |
| YOR384W | FRE5 | 0.69677033 | 0.99917121 | -0.0689613 | 143.7542239 | 237.3160829 |
| YGR249W | MGA1 | 0.77038173 | 0.99917121 | -0.0689167 | 153.0026119 | 252.0374491 |
| YCR063W | BUD31 | 0.39100222 | 0.99917121 | -0.0688976 | 0 | 8.399353583 |
| YJL214W | HXT8 | 0.68192912 | 0.99917121 | -0.0687653 | 145.8317343 | 240.6003419 |
| YKL114C | APN1 | 0.70427407 | 0.99917121 | -0.0687366 | 134.9085217 | 223.2031232 |
| YOR050C | YOR050C | 0.73636238 | 0.99917121 | -0.0684667 | 142.8179263 | 235.7648699 |
| YGR184C | UBR1 | 0.71271123 | 0.99917121 | -0.0683609 | 127.1479858 | 210.7997248 |
| YCL050C | APA1 | 0.5739384 | 0.99917121 | -0.0683457 | 143.328849 | 236.5636852 |
| YNR014W | YNR014W | 0.72785686 | 0.99917121 | -0.0682388 | 129.545837 | 214.6030891 |
| YOR002W | ALG6 | 0.75307462 | 0.99917121 | -0.0681714 | 133.8666763 | 221.4752242 |
| YKR041W | YKR041W | 0.66635804 | 0.99917121 | -0.0677891 | 139.2720095 | 230.0358633 |
| YGR014W | MSB2 | 0.67211675 | 0.99917121 | -0.0677837 | 134.725483 | 222.7954871 |
| YDR178W | SDH4 | 0.76944668 | 0.99917121 | -0.0677428 | 126.51882 | 209.7225156 |
| YMR037C | MSN2 | 0.7140739 | 0.99917121 | -0.0676781 | 133.7334119 | 221.2028725 |
| YOR017W | PET127 | 0.75252602 | 0.99917121 | -0.0676142 | 137.0794916 | 226.5232571 |
| YGR193C | PDX1 | 0.74268218 | 0.99917121 | -0.0675173 | 154.2202543 | 253.8057729 |
| YOR348C | PUT4 | 0.80973398 | 0.99917121 | -0.0672556 | 127.3612685 | 211.004596 |
| YOR001W | RRP6 | 0.72966591 | 0.99917121 | -0.067254 | 115.5318069 | 192.1676017 |
| YDR491C | YDR491C | 0.71841645 | 0.99917121 | -0.0672405 | 142.2670271 | 234.7381463 |
| YNL121C | TOM70 | 0.73242721 | 0.99917121 | -0.0670026 | 135.4571629 | 223.8653631 |
| YKR052C | MRS4 | 0.73423085 | 0.99917121 | -0.0669187 | 122.9828799 | 203.9915448 |
| YPL250C | ICY2 | 0.69639908 | 0.99917121 | -0.0668067 | 114.9538027 | 191.1926827 |
| YDR463W | STP1 | 0.79126057 | 0.99917121 | -0.0667929 | 121.0979803 | 200.9747633 |
| YDR287W | YDR287W | 0.7128484 | 0.99917121 | -0.0667791 | 140.5352537 | 231.9242802 |
| YNL071W | LAT1 | 0.73435463 | 0.99917121 | -0.0667644 | 128.5996094 | 212.9166078 |
| YIL163C | YIL163C | 0.72409553 | 0.99917121 | -0.0667065 | 146.1356064 | 240.8332315 |
| YBR295W | PCA1 | 0.76821509 | 0.99917121 | -0.066602 | 123.495131 | 204.7686295 |
| YLL012W | YEH1 | 0.6670459 | 0.99917121 | -0.0665857 | 134.2916841 | 221.9586696 |
| YKR103W | NFT1 | 0.68021694 | 0.99917121 | -0.0665189 | 143.0982487 | 235.9737855 |
| YDR199W | YDR199W | 0.68380475 | 0.99917121 | -0.0663742 | 144.892412 | 238.8131027 |
| YOR133W | EFT1 | 0.76047352 | 0.99917121 | -0.0662444 | 130.7816616 | 216.3278294 |
| YOR061W | CKA2 | 0.54819636 | 0.99917121 | -0.0660732 | 106.0924347 | 176.9927421 |
| YBL029W | YBL029W | 0.72205284 | 0.99917121 | -0.06606 | 140.9617987 | 232.5158356 |
| YMR135C | GID8 | 0.74133781 | 0.99917121 | -0.0660511 | 128.8773357 | 213.2718902 |
| YDL130W | RPP1B | 0.62135043 | 0.99917121 | -0.0659468 | 95.04570885 | 159.3869284 |
| YCR024C-A | PMP1 | 0.72923533 | 0.99917121 | -0.0652983 | 132.0207702 | 218.1856066 |
| YER085C | YER085C | 0.68649855 | 0.99917121 | -0.0652555 | 143.3446688 | 236.2121448 |
| YHL010C | YHL010C | 0.84713628 | 0.99917121 | -0.0651241 | 134.5324869 | 222.1639352 |
| YNL229C | URE2 | 0.75300511 | 0.99917121 | -0.0648584 | 142.9851033 | 235.5911779 |
| YDR072C | IPT1 | 0.71508341 | 0.99917121 | -0.0646705 | 132.1247067 | 218.2745782 |
| YDL074C | BRE1 | 0.6732392 | 0.99917121 | -0.0645648 | 104.6470882 | 174.5073329 |
| YBR203W | COS111 | 0.67011961 | 0.99917121 | -0.0643035 | 145.2653163 | 239.154462 |
| YPL052W | OAZ1 | 0.72492189 | 0.99917121 | -0.0639791 | 136.7526621 | 225.5596776 |
| YGR250C | YGR250C | 0.82068789 | 0.99917121 | -0.0639447 | 137.4949358 | 226.737453 |
| YJL013C | MAD3 | 0.69084461 | 0.99917121 | -0.0637488 | 134.9322995 | 222.6329171 |
| YDR370C | YDR370C | 0.67451995 | 0.99917121 | -0.0636819 | 143.6050853 | 236.4349878 |
| YNL056W | OCA2 | 0.57810982 | 0.99917121 | -0.0636126 | 109.779671 | 182.5641922 |
| YGL210W | YPT32 | 0.53798482 | 0.99917121 | -0.063407 | 114.6243373 | 190.2535888 |
| YPL004C | LSP1 | 0.77311642 | 0.99917121 | -0.0632405 | 133.7194017 | 220.6395772 |
| YGL221C | NIF3 | 0.81440042 | 0.99917121 | -0.0631609 | 110.4042118 | 183.5036124 |
| YOL109W | ZEO1 | 0.63087569 | 0.99917121 | -0.0631348 | 109.080106 | 181.3919817 |
| YOL046C | YOL046C | 0.73151067 | 0.99917121 | -0.0628129 | 130.3009649 | 215.1440567 |
| YMR291W | YMR291W | 0.79286748 | 0.99917121 | -0.0627226 | 138.2098248 | 227.7268286 |
| YER129W | SAK1 | 0.75764161 | 0.99917121 | -0.0625355 | 135.2324175 | 222.9629028 |
| YIL010W | DOT5 | 0.77281904 | 0.99917121 | -0.0625093 | 141.5768337 | 233.0623188 |
| YGR213C | RTA1 | 0.74540953 | 0.99917121 | -0.0624129 | 132.4394164 | 218.5004816 |
| YOL116W | MSN1 | 0.80161653 | 0.99917121 | -0.0624109 | 133.0154819 | 219.4175439 |

| | | | | | | |
|---|---|---|---|---|---|---|
| YAL040C | CLN3 | 0.73695022 | 0.99917121 | -0.062306 | 139.6490987 | 229.9678845 |
| YLL038C | ENT4 | 0.72170314 | 0.99917121 | -0.0621064 | 131.2873798 | 216.6286545 |
| YAL054C | ACS1 | 0.66697308 | 0.99917121 | -0.0620848 | 160.7250622 | 263.5015065 |
| YFL053W | DAK2 | 0.72779034 | 0.99917121 | -0.0618702 | 143.8452684 | 236.5965858 |
| YDL210W | UGA4 | 0.71651924 | 0.99917121 | -0.0618028 | 138.5840759 | 228.2106399 |
| YMR295C | YMR295C | 0.77807834 | 0.99917121 | -0.0616945 | 143.9246324 | 236.7015409 |
| YNR008W | LRO1 | 0.67489935 | 0.99917121 | -0.0616783 | 144.2691182 | 237.2481096 |
| YAR003W | SWD1 | 0.91037713 | 0.99917121 | -0.0616433 | 81.08937162 | 136.6387239 |
| YIL108W | YIL108W | 0.65219511 | 0.99917121 | -0.0616114 | 131.1064268 | 216.2801658 |
| YGR157W | CHO2 | 0.77636896 | 0.99917121 | -0.0613922 | 9.684910834 | 22.90625845 |
| YOR302W | YOR302W | 0.7336065 | 0.99917121 | -0.0613503 | 134.6588152 | 221.9050285 |
| YDR191W | HST4 | 0.61956096 | 0.99917121 | -0.0613203 | 156.1946709 | 256.194277 |
| YJL207C | LAA1 | 0.58121512 | 0.99917121 | -0.0610284 | 137.0268925 | 225.63662 |
| YIR028W | DAL4 | 0.59310155 | 0.99917121 | -0.06101 | 148.2937938 | 243.5753789 |
| YOR212W | STE4 | 0.6793137 | 0.99917121 | -0.0608644 | 144.8398089 | 238.0576343 |
| YJL132W | YJL132W | 0.74420958 | 0.99917121 | -0.0602503 | 129.5322627 | 213.6075905 |
| YMR252C | YMR252C | 0.78262288 | 0.99917121 | -0.0599315 | 146.6038349 | 240.7528803 |
| YOR311C | HSD1 | 0.76791047 | 0.99917121 | -0.0598418 | 126.3331136 | 208.4635808 |
| YPL027W | SMA1 | 0.7206491 | 0.99917121 | -0.0598268 | 143.1068839 | 235.1716878 |
| YAL019W | FUN30 | 0.74946574 | 0.99917121 | -0.0597938 | 135.0866828 | 222.3965993 |
| YLR094C | GIS3 | 0.74006645 | 0.99917121 | -0.0596652 | 135.9302691 | 223.7242132 |
| YLR376C | PSY3 | 0.73788368 | 0.99917121 | -0.0595902 | 134.2456444 | 221.0325357 |
| YDR005C | MAF1 | 0.73312166 | 0.99917121 | -0.0595136 | 131.990569 | 217.4322924 |
| YER061C | CEM1 | 0.71190853 | 0.99917121 | -0.0593309 | 85.94076916 | 144.0820086 |
| YER150W | SPI1 | 0.79977721 | 0.99917121 | -0.0591654 | 117.7489398 | 194.7120047 |
| YOR301W | RAX1 | 0.765252 | 0.99917121 | -0.0590632 | 136.8390598 | 225.0979494 |
| YEL038W | UTR4 | 0.76914082 | 0.99917121 | -0.0587643 | 137.3653939 | 225.8996215 |
| YLR231C | BNA5 | 0.78526353 | 0.99917121 | -0.0587512 | 143.9984396 | 236.4602452 |
| YOR022C | YOR022C | 0.79069369 | 0.99917121 | -0.0582088 | 130.5736665 | 215.0170091 |
| YDR333C | YDR333C | 0.72919404 | 0.99917121 | -0.0580446 | 142.6613268 | 234.2449389 |
| YDR067C | OCA6 | 0.79860376 | 0.99917121 | -0.05804 | 123.9377129 | 204.4295799 |
| YDR332W | IRC3 | 0.74079206 | 0.99917121 | -0.0578634 | 138.7627144 | 228.0148358 |
| YCR076C | YCR076C | 0.72854228 | 0.99917121 | -0.0578332 | 151.245887 | 247.8889063 |
| YDL224C | WHI4 | 0.70656788 | 0.99917121 | -0.0576043 | 147.5706115 | 242.0086222 |
| YNL101W | AVT4 | 0.78105807 | 0.99917121 | -0.0573908 | 142.5904909 | 234.0524304 |
| YJL168C | SET2 | 0.81227289 | 0.99917121 | -0.057302 | 124.2024957 | 204.7612355 |
| YER181C | YER181C | 0.5791 | 0.99917121 | -0.0571848 | 113.7382127 | 188.0840095 |
| YLL029W | YLL029W | 0.60993967 | 0.99917121 | -0.0570566 | 134.8983699 | 221.7630373 |
| YJL171C | YJL171C | 0.75050402 | 0.99917121 | -0.0569483 | 146.0302132 | 239.4757716 |
| YOR197W | MCA1 | 0.72692548 | 0.99917121 | -0.0568243 | 135.6151829 | 222.8761459 |
| YLL014W | YLL014W | 0.79392953 | 0.99917121 | -0.0567498 | 133.1507926 | 218.9428613 |
| YGL110C | CUE3 | 0.77968535 | 0.99917121 | -0.0566847 | 136.7952047 | 224.7381507 |
| YJL191W | RPS14B | 0.76219744 | 0.99917121 | -0.0565541 | 139.5543369 | 229.1157725 |
| YPL139C | UME1 | 0.75196733 | 0.99917121 | -0.0564271 | 141.9906921 | 232.9798456 |
| YDR539W | YDR539W | 0.74328889 | 0.99917121 | -0.0563998 | 138.760581 | 227.8330083 |
| YCL051W | LRE1 | 0.71006781 | 0.99917121 | -0.0563565 | 147.050286 | 241.0279543 |
| YDR001C | NTH1 | 0.63580287 | 0.99917121 | -0.0563323 | 150.2005319 | 246.0413412 |
| YOR019W | YOR019W | 0.7754854 | 0.99917121 | -0.0563233 | 144.592297 | 237.109891 |
| YOL129W | VPS68 | 0.7829385 | 0.99917121 | -0.0560026 | 134.9651674 | 221.7409101 |
| YCL032W | STE50 | 0.67614306 | 0.99917121 | -0.0558898 | 145.2065786 | 238.0352125 |
| YDR509W | YDR509W | 0.74276789 | 0.99917121 | -0.0558303 | 144.8979988 | 237.5365831 |
| YMR040W | YET2 | 0.73192484 | 0.99917121 | -0.0557826 | 135.2158309 | 222.1132399 |
| YDL199C | YDL199C | 0.729201 | 0.99917121 | -0.0556858 | 152.1985639 | 249.1441216 |
| YJL198W | PHO90 | 0.7724937 | 0.99917121 | -0.0556311 | 147.3042861 | 241.3439772 |
| YDR312W | SSF2 | 0.76177042 | 0.99917121 | -0.0552577 | 145.7371299 | 238.8029817 |
| YGR225W | AMA1 | 0.75369898 | 0.99917121 | -0.0551305 | 118.5954103 | 195.5679875 |
| YNL049C | SFB2 | 0.77009557 | 0.99917121 | -0.0550724 | 139.553219 | 228.933352 |
| YMR139W | RIM11 | 0.64161737 | 0.99917121 | -0.0546642 | 93.56700594 | 155.6568282 |
| YPL272C | YPL272C | 0.82627177 | 0.99917121 | -0.0545857 | 117.6271481 | 193.9597432 |
| YHR204W | MNL1 | 0.79570904 | 0.99917121 | -0.0543332 | 146.1542968 | 239.3545551 |
| YNL265C | IST1 | 0.81056499 | 0.99917121 | -0.0543251 | 138.9766078 | 227.9240804 |
| YLR183C | TOS4 | 0.7698728 | 0.99917121 | -0.0541704 | 131.7794316 | 216.444699 |
| YOR040W | GLO4 | 0.7650414 | 0.99917121 | -0.0541603 | 144.5229605 | 236.7357914 |
| YDL186W | YDL186W | 0.91227372 | 0.99917121 | -0.0535381 | 111.093414 | 183.4279518 |

| | | | | | | |
|---|---|---|---|---|---|---|
| YOR164C | YOR164C | 0.79794126 | 0.99917121 | -0.053523 | 133.6740836 | 219.3827505 |
| YOR195W | SLK19 | 0.79968276 | 0.99917121 | -0.0535186 | 139.4454584 | 228.5723407 |
| YNL300W | YNL300W | 0.86216792 | 0.99917121 | -0.0535043 | 115.3972243 | 190.2770698 |
| YKL132C | RMA1 | 0.7834982 | 0.99917121 | -0.0534513 | 140.521087 | 230.2769292 |
| YPL068C | YPL068C | 0.8083139 | 0.99917121 | -0.053184 | 132.8185813 | 217.979147 |
| YGL263W | COS12 | 0.72974724 | 0.99917121 | -0.0531497 | 139.3004292 | 228.2964194 |
| YFL003C | MSH4 | 0.83540663 | 0.99917121 | -0.0530411 | 121.7128415 | 200.2773502 |
| YHR176W | FMO1 | 0.82440338 | 0.99917121 | -0.0529955 | 133.155214 | 218.4922088 |
| YNL091W | NST1 | 0.81646042 | 0.99917121 | -0.0528513 | 67.65443097 | 114.1735788 |
| YKL079W | SMY1 | 0.83270934 | 0.99917121 | -0.052789 | 135.17821 | 221.6883762 |
| YOR084W | YOR084W | 0.8042342 | 0.99917121 | -0.0527054 | 134.4478528 | 220.5151977 |
| YOR291W | YOR291W | 0.77069939 | 0.99917121 | -0.0526852 | 135.4436696 | 222.0984323 |
| YDR352W | YDR352W | 0.73780843 | 0.99917121 | -0.0526315 | 138.2371369 | 226.5401077 |
| YPL194W | DDC1 | 0.76047067 | 0.99917121 | -0.0525777 | 130.1408602 | 213.6413317 |
| YBR056W | YBR056W | 0.75025563 | 0.99917121 | -0.0525389 | 155.4907965 | 254.0029149 |
| YOR192C | THI72 | 0.76615064 | 0.99917121 | -0.0524898 | 146.5121921 | 239.6997231 |
| YMR273C | ZDS1 | 0.78790137 | 0.99917121 | -0.0521388 | 139.7044341 | 228.8165074 |
| YJL089W | SIP4 | 0.70825597 | 0.99917121 | -0.0520559 | 153.5451182 | 250.8458045 |
| YBR032W | YBR032W | 0.67517803 | 0.99917121 | -0.0518264 | 127.6834642 | 209.6366667 |
| YOR076C | SKI7 | 0.85524367 | 0.99917121 | -0.0517705 | 112.0530978 | 184.7406259 |
| YIL093C | RSM25 | 0.79026762 | 0.99917121 | -0.0517423 | 136.1599766 | 223.1241032 |
| YIL017C | VID28 | 0.85720773 | 0.99917121 | -0.0513707 | 116.4006135 | 191.6147198 |
| YLR081W | GAL2 | 0.80659476 | 0.99917121 | -0.0511817 | 143.5197237 | 234.7751541 |
| YKL171W | YKL171W | 0.68099531 | 0.99917121 | -0.0510544 | 134.4037725 | 220.2437221 |
| YDR210W | YDR210W | 0.75793578 | 0.99917121 | -0.051022 | 151.4959957 | 247.4568053 |
| YPL110C | GDE1 | 0.72568793 | 0.99917121 | -0.0508305 | 140.5649591 | 230.0272755 |
| YGL056C | SDS23 | 0.82057413 | 0.99917121 | -0.050702 | 140.7955994 | 230.3788821 |
| YNL270C | ALP1 | 0.77120668 | 0.99917121 | -0.0505502 | 142.7209945 | 233.4263049 |
| YFR006W | YFR006W | 0.81605782 | 0.99917121 | -0.0504734 | 145.7840326 | 238.2944134 |
| YJL082W | IML2 | 0.86719045 | 0.99917121 | -0.0504592 | 114.1568531 | 187.930707 |
| YKL146W | AVT3 | 0.68931354 | 0.99917121 | -0.0502965 | 134.3955779 | 220.138277 |
| YKL179C | COY1 | 0.75987158 | 0.99917121 | -0.0502681 | 138.5537505 | 226.7561453 |
| YDR330W | UBX5 | 0.7499506 | 0.99917121 | -0.0502404 | 140.0515518 | 229.1378138 |
| YDR234W | LYS4 | 0.84494272 | 0.99917121 | -0.0502136 | 125.4965588 | 205.9577044 |
| YGR235C | YGR235C | 0.88903952 | 0.99917121 | -0.0498833 | 134.0606207 | 219.5545395 |
| YDR193W | YDR193W | 0.74670734 | 0.99917121 | -0.0497952 | 143.5820703 | 234.7054025 |
| YIL140W | AXL2 | 0.73785802 | 0.99917121 | -0.0497155 | 153.2924424 | 250.1581294 |
| YDR103W | STE5 | 0.73983239 | 0.99917121 | -0.0493731 | 147.3428726 | 240.6425051 |
| YDR055W | PST1 | 0.67880059 | 0.99917121 | -0.0493462 | 147.8373785 | 241.4266635 |
| YLR438W | CAR2 | 0.77947132 | 0.99917121 | -0.0492127 | 129.4338431 | 212.1052704 |
| YGL045W | RIM8 | 0.73741285 | 0.99917121 | -0.0491942 | 133.1402104 | 218.0048988 |
| YCL039W | GID7 | 0.79273338 | 0.99917121 | -0.0490495 | 142.8033935 | 233.3745536 |
| YLR428C | YLR428C | 0.86408549 | 0.99917121 | -0.0490081 | 122.0341713 | 200.2973626 |
| YJL116C | NCA3 | 0.69933948 | 0.99917121 | -0.0488538 | 152.4783142 | 248.7566891 |
| YDR389W | SAC7 | 0.74417228 | 0.99917121 | -0.0488208 | 139.1992793 | 227.6076203 |
| YDR395W | SXM1 | 0.79274322 | 0.99917121 | -0.0488101 | 143.8370923 | 234.9913962 |
| YNR067C | DSE4 | 0.67166648 | 0.99917121 | -0.0486698 | 132.2934668 | 216.592652 |
| YPL196W | OXR1 | 0.75189817 | 0.99917121 | -0.0483969 | 140.3990886 | 229.4664676 |
| YDR149C | YDR149C | 0.79631423 | 0.99917121 | -0.0482271 | 127.9877029 | 209.6823301 |
| YDR249C | YDR249C | 0.71692885 | 0.99917121 | -0.0481812 | 139.3066632 | 227.7006394 |
| YOR337W | TEA1 | 0.88972826 | 0.99917121 | -0.04804 | 121.1894918 | 198.8343026 |
| YDL025C | YDL025C | 0.80227894 | 0.99917121 | -0.0479721 | 145.0619989 | 236.8397356 |
| YFR032C-A | RPL29 | 0.86371688 | 0.99917121 | -0.0478913 | 118.5301354 | 194.5815186 |
| YMR039C | SUB1 | 0.60310143 | 0.99917121 | -0.0478876 | 116.7407603 | 191.7317225 |
| YKL174C | TPO5 | 0.79549112 | 0.99917121 | -0.0477967 | 135.7324025 | 221.9622402 |
| YKL187C | YKL187C | 0.78887798 | 0.99917121 | -0.0475816 | 139.9514054 | 228.654205 |
| YDL214C | PRR2 | 0.74371949 | 0.99917121 | -0.0474557 | 142.3524257 | 232.4621547 |
| YKR013W | PRY2 | 0.75297518 | 0.99917121 | -0.0474145 | 137.113487 | 224.1148387 |
| YHR016C | YSC84 | 0.81022891 | 0.99917121 | -0.0472329 | 142.2174334 | 232.2200321 |
| YOR346W | REV1 | 0.73725221 | 0.99917121 | -0.0471707 | 135.7245619 | 221.8734446 |
| YDL066W | IDP1 | 0.82735456 | 0.99917121 | -0.0468947 | 153.8787167 | 250.747807 |
| YGR287C | YGR287C | 0.82502711 | 0.99917121 | -0.0468862 | 141.1890998 | 230.5402914 |
| YJL122W | ALB1 | 0.75661927 | 0.99917121 | -0.0468569 | 146.1840067 | 238.4904175 |
| YPL130W | SPO19 | 0.77844212 | 0.99917121 | -0.0466358 | 137.3367666 | 224.3754496 |

| | | | | | | |
|---|---|---|---|---|---|---|
| YDR011W | SNQ2 | 0.78662891 | 0.99917121 | -0.0464748 | 141.7920287 | 231.4502176 |
| YDR186C | YDR186C | 0.73773607 | 0.99917121 | -0.0463587 | 140.258079 | 228.9934579 |
| YIL056W | VHR1 | 0.77747162 | 0.99917121 | -0.0461223 | 136.3136569 | 222.6836873 |
| YKR046C | PET10 | 0.79669892 | 0.99917121 | -0.0460299 | 140.5788096 | 229.4640934 |
| YBL063W | KIP1 | 0.76767123 | 0.99917121 | -0.0460174 | 145.9119395 | 237.9548467 |
| YDL077C | VAM6 | 0.83231753 | 0.99917121 | -0.04595 | 97.69684374 | 161.170681 |
| YDL229W | SSB1 | 0.77669183 | 0.99917121 | -0.0458587 | 133.7854506 | 218.6257256 |
| YNL235C | :::SIN4 | 0.39100222 | 0.99917121 | -0.0457793 | 0 | 5.580986059 |
| YDR318W | MCM21 | 0.74566051 | 0.99917121 | -0.0457352 | 142.7125042 | 232.8257748 |
| YNL237W | YTP1 | 0.81985976 | 0.99917121 | -0.0456978 | 141.5808709 | 231.0192463 |
| YER109C | FLO8 | 0.81763487 | 0.99917121 | -0.0455406 | 135.2810199 | 220.9684369 |
| YLL006W | MMM1 | 0.94361342 | 0.99917121 | -0.0454582 | 38.99023039 | 67.62845704 |
| YBL009W | ALK2 | 0.82906132 | 0.99917121 | -0.0453701 | 144.1860474 | 235.1276919 |
| YOL075C | YOL075C | 0.77073315 | 0.99917121 | -0.0452584 | 142.8640723 | 233.0090069 |
| YOL160W | YOL160W | 0.76725889 | 0.99917121 | -0.0452463 | 142.610771 | 232.6041845 |
| YDR206W | EBS1 | 0.79876219 | 0.99917121 | -0.0449698 | 149.4190376 | 243.4117128 |
| YJL210W | PEX2 | 0.80551034 | 0.99917121 | -0.0447279 | 133.0028014 | 217.2416058 |
| YNL322C | KRE1 | 0.79208463 | 0.99917121 | -0.0446593 | 136.0153489 | 222.0303085 |
| YDR254W | CHL4 | 0.80684044 | 0.99917121 | -0.0445709 | 141.1367372 | 230.1746444 |
| YAL024C | LTE1 | 0.84856403 | 0.99917121 | -0.0442278 | 117.129162 | 191.9040307 |
| YEL039C | CYC7 | 0.62151712 | 0.99917121 | -0.0441819 | 111.809632 | 183.4278123 |
| YBR007C | DSF2 | 0.73686192 | 0.99917121 | -0.0439263 | 134.2811691 | 219.179513 |
| YER164W | CHD1 | 0.79026265 | 0.99917121 | -0.0436774 | 132.5867762 | 216.4510795 |
| YAL020C | ATS1 | 0.82038682 | 0.99917121 | -0.0436712 | 125.0464205 | 204.4433341 |
| YKL117W | SBA1 | 0.80514819 | 0.99917121 | -0.0436693 | 134.3718108 | 219.2925166 |
| YJL110C | GZF3 | 0.78900814 | 0.99917121 | -0.0436033 | 140.8109746 | 229.5379499 |
| YBR052C | RFS1 | 0.80158048 | 0.99917121 | -0.043335 | 144.2960994 | 235.054835 |
| YMR086W | YMR086W | 0.79068202 | 0.99917121 | -0.0432894 | 138.828151 | 226.3423122 |
| YDR291W | HRQ1 | 0.80031379 | 0.99917121 | -0.0432768 | 153.3711837 | 249.4985625 |
| YHL042W | YHL042W | 0.8767066 | 0.99917121 | -0.043242 | 124.2737142 | 203.1605827 |
| YOR359W | VTS1 | 0.8533332 | 0.99917121 | -0.0429427 | 130.9069352 | 213.686593 |
| YPL112C | PEX25 | 0.82197468 | 0.99917121 | -0.0427543 | 134.6891751 | 219.6863187 |
| YER034W | YER034W | 0.95061516 | 0.99917121 | -0.0427245 | 101.6431434 | 167.061391 |
| YER090W | TRP2 | 0.60494637 | 0.99917121 | -0.0426908 | 112.5025786 | 184.3494526 |
| YJL077C | ICS3 | 0.79483713 | 0.99917121 | -0.0426042 | 149.0778661 | 242.5800472 |
| YPL017C | IRC15 | 0.85494369 | 0.99917121 | -0.0425202 | 128.1099978 | 209.1813457 |
| YDL185W | TFP1 | 0.75405027 | 0.99917121 | -0.0424811 | 146.5517442 | 238.5425327 |
| YCR011C | ADP1 | 0.76614581 | 0.99917121 | -0.0423411 | 148.8659506 | 242.2105242 |
| YDR202C | RAV2 | 0.78607015 | 0.99917121 | -0.0421878 | 144.3757439 | 235.0418006 |
| YBR099C | YBR099C | 0.77766047 | 0.99917121 | -0.0421165 | 130.390464 | 212.7634574 |
| YBR104W | YMC2 | 0.85592094 | 0.99917121 | -0.0417111 | 121.3301786 | 198.2867724 |
| YER175C | TMT1 | 0.80322247 | 0.99917121 | -0.0416036 | 138.3034353 | 225.3012573 |
| YKL001C | MET14 | 0.84791095 | 0.99917121 | -0.0412686 | 137.115606 | 223.3689604 |
| YOR131C | YOR131C | 0.82561098 | 0.99917121 | -0.04122 | 142.2418427 | 231.5258682 |
| YDR009W | GAL3 | 0.80937542 | 0.99917121 | -0.0407712 | 141.5346027 | 230.3449663 |
| YDL216C | RRI1 | 0.81016102 | 0.99917121 | -0.0403358 | 146.6622523 | 238.4569762 |
| YGR130C | YGR130C | 0.79854042 | 0.99917121 | -0.040294 | 136.7012501 | 222.5903429 |
| YOR324C | FRT1 | 0.82399456 | 0.99917121 | -0.0402635 | 141.2007886 | 229.7515198 |
| YML042W | CAT2 | 0.79195955 | 0.99917121 | -0.0402587 | 110.4478448 | 180.7810785 |
| YNL176C | YNL176C | 0.83182096 | 0.99917121 | -0.0401659 | 139.2723849 | 226.6688985 |
| YDR215C | YDR215C | 0.81864282 | 0.99917121 | -0.0400989 | 142.0494971 | 231.0829052 |
| YDR018C | YDR018C | 0.76933137 | 0.99917121 | -0.0400152 | 146.7156503 | 238.5029211 |
| YOR292C | YOR292C | 0.83023542 | 0.99917121 | -0.0395734 | 139.2711628 | 226.5947299 |
| YBR101C | FES1 | 0.82581651 | 0.99917121 | -0.0395608 | 143.8666255 | 233.9108341 |
| YKL178C | STE3 | 0.83277794 | 0.99917121 | -0.0394847 | 141.3145106 | 229.8376693 |
| YCR099C | YCR099C | 0.81830204 | 0.99917121 | -0.0391505 | 144.7559249 | 235.2769028 |
| YIR002C | MPH1 | 0.82295571 | 0.99917121 | -0.0391323 | 119.176095 | 194.5423024 |
| YNL289W | PCL1 | 0.85182258 | 0.99917121 | -0.0389361 | 143.7488432 | 233.6471243 |
| YCL026C-A | FRM2 | 0.89741843 | 0.99917121 | -0.0386193 | 124.8901851 | 203.5786682 |
| YGR231C | PHB2 | 0.63762509 | 0.99917121 | -0.0384831 | 111.3068633 | 181.9324794 |
| YKR014C | YPT52 | 0.80453721 | 0.99917121 | -0.0384213 | 137.6566087 | 223.883311 |
| YDR421W | ARO80 | 0.75532822 | 0.99917121 | -0.0383961 | 150.3360141 | 244.0704611 |
| YEL001C | IRC22 | 0.82211179 | 0.99917121 | -0.0382458 | 136.3793886 | 221.8281219 |
| YML027W | YOX1 | 0.88704924 | 0.99917121 | -0.0380519 | 102.0061878 | 167.0698532 |

| | | | | | | |
|---|---|---|---|---|---|---|
| YNL156C | NSG2 | 0.8432568 | 0.99917121 | -0.0380358 | 135.3772989 | 220.2068305 |
| YOR134W | BAG7 | 0.85887959 | 0.99917121 | -0.0379069 | 127.9338258 | 208.3383966 |
| YOL003C | PFA4 | 0.83075958 | 0.99917121 | -0.0378764 | 133.2570718 | 216.8112273 |
| YDR372C | VPS74 | 0.82966862 | 0.99917121 | -0.0377834 | 139.4070346 | 226.5928593 |
| YNR064C | YNR064C | 0.89244941 | 0.99917121 | -0.0375744 | 117.4159007 | 191.5495064 |
| YKR005C | YKR005C | 0.79516505 | 0.99917121 | -0.0374373 | 145.7919283 | 236.717736 |
| YDL234C | GYP7 | 0.82115657 | 0.99917121 | -0.03733 | 147.0348839 | 238.6838925 |
| YML111W | BUL2 | 0.39100222 | 0.99917121 | -0.0372832 | 0 | 4.545219816 |
| YDR165W | TRM82 | 0.82352907 | 0.99917121 | -0.037213 | 141.5773182 | 229.9792096 |
| YJR010C-A | SPC1 | 0.83346392 | 0.99917121 | -0.0372102 | 153.6132405 | 249.1444295 |
| YOR321W | PMT3 | 0.82297394 | 0.99917121 | -0.0370166 | 142.9093268 | 232.0763011 |
| YNL309W | STB1 | 0.85888427 | 0.99917121 | -0.0369175 | 134.3474556 | 218.4306116 |
| YOR255W | OSW1 | 0.78124839 | 0.99917121 | -0.0368908 | 141.1284612 | 229.2251791 |
| YLR090W | XDJ1 | 0.85950984 | 0.99917121 | -0.0368283 | 144.0278171 | 233.8343892 |
| YOR101W | RAS1 | 0.87167938 | 0.99917121 | -0.0368245 | 141.7078498 | 230.1397001 |
| YKR051W | YKR051W | 0.8390666 | 0.99917121 | -0.0367886 | 140.1146271 | 227.5983362 |
| YGR093W | YGR093W | 0.83224166 | 0.99917121 | -0.0366908 | 117.6804967 | 191.8631237 |
| YDR048C | YDR048C | 0.79835969 | 0.99917121 | -0.0366365 | 137.2366308 | 222.9969722 |
| YBL048W | YBL048W | 0.8378864 | 0.99917121 | -0.0365914 | 150.7483715 | 244.5070713 |
| YPL220W | RPL1A | 0.81882555 | 0.99917121 | -0.0365607 | 138.253059 | 224.6062519 |
| YPL185W | YPL185W | 0.8080198 | 0.99917121 | -0.0363223 | 135.4935407 | 220.1830392 |
| YDR411C | DFM1 | 0.77930327 | 0.99917121 | -0.0363013 | 148.7480937 | 241.2865409 |
| YOR162C | YRR1 | 0.85781433 | 0.99917121 | -0.0362822 | 137.9544475 | 224.0968055 |
| YDR100W | TVP15 | 0.81145325 | 0.99917121 | -0.0362007 | 155.2496058 | 251.6270408 |
| YDR063W | YDR063W | 0.82777831 | 0.99917121 | -0.0361927 | 144.7844788 | 234.9617828 |
| YOR378W | YOR378W | 0.82426259 | 0.99917121 | -0.0360723 | 139.458581 | 226.466336 |
| YGR027C | RPS25A | 0.74435441 | 0.99917121 | -0.0359828 | 105.5052822 | 172.3894386 |
| YDR525W-A | SNA2 | 0.86850396 | 0.99917121 | -0.0354957 | 137.0492061 | 222.5594528 |
| YPL145C | KES1 | 0.8542026 | 0.99917121 | -0.0354907 | 125.7642757 | 204.5891284 |
| YPL102C | YPL102C | 0.84557038 | 0.99917121 | -0.0354478 | 110.1929289 | 179.7886572 |
| YPL189W | GUP2 | 0.82335568 | 0.99917121 | -0.0354199 | 156.5978641 | 253.6787789 |
| YBR050C | REG2 | 0.86737636 | 0.99917121 | -0.0354024 | 124.9561003 | 203.2914562 |
| YDR150W | NUM1 | 0.84522642 | 0.99917121 | -0.0351763 | 122.800354 | 199.8311588 |
| YOR377W | ATF1 | 0.83339121 | 0.99917121 | -0.0351567 | 135.601005 | 220.212055 |
| YDR461W | MFA1 | 0.84987065 | 0.99917121 | -0.0350728 | 159.522814 | 258.2940382 |
| YNL280C | ERG24 | 0.86529875 | 0.99917121 | -0.0348064 | 131.170415 | 213.1142398 |
| YPL216W | YPL216W | 0.81927061 | 0.99917121 | -0.0347607 | 150.7224203 | 244.2425679 |
| YMR175W | SIP18 | 0.82606564 | 0.99917121 | -0.0347542 | 135.4114485 | 219.8611426 |
| YFL044C | OTU1 | 0.85268893 | 0.99917121 | -0.0347289 | 146.271076 | 237.1505317 |
| YNR055C | HOL1 | 0.8810315 | 0.99917121 | -0.034718 | 131.751299 | 214.0284413 |
| YDR419W | RAD30 | 0.90533035 | 0.99917121 | -0.0346668 | 136.2028088 | 221.1106214 |
| YPR013C | YPR013C | 0.9276338 | 0.99917121 | -0.0346346 | 82.37885814 | 135.3994106 |
| YGL115W | SNF4 | 0.94205943 | 0.99917121 | -0.0345881 | 78.32121375 | 128.9324919 |
| YBR094W | PBY1 | 0.85481026 | 0.99917121 | -0.0344338 | 128.234523 | 208.3938109 |
| YNR001C | CIT1 | 0.85711843 | 0.99917121 | -0.0343361 | 131.9584431 | 214.3117387 |
| YBR242W | YBR242W | 0.86717071 | 0.99917121 | -0.0342164 | 159.3627112 | 257.9346966 |
| YMR316C-A | YMR316C-A | 0.84675621 | 0.99917121 | -0.0342074 | 147.1571227 | 238.4978624 |
| YMR172W | HOT1 | 0.89382601 | 0.99917121 | -0.0340537 | 139.5972187 | 226.4410085 |
| YDR128W | MTC5 | 0.85005448 | 0.99917121 | -0.0339891 | 140.0750421 | 227.1940085 |
| YDR399W | HPT1 | 0.81378216 | 0.99917121 | -0.0339499 | 145.9904389 | 236.6086845 |
| YAR035W | YAT1 | 0.81741429 | 0.99917121 | -0.0337438 | 142.286438 | 230.6854539 |
| YLR080W | EMP46 | 0.88301966 | 0.99917121 | -0.0337075 | 126.9001229 | 206.1804183 |
| YOL057W | YOL057W | 0.89355294 | 0.99917121 | -0.0336937 | 139.4084981 | 226.0966189 |
| YNL330C | RPD3 | 0.87985791 | 0.99917121 | -0.0336677 | 137.9648029 | 223.7945586 |
| YBR144C | YBR144C | 0.86257773 | 0.99917121 | -0.0334096 | 136.9238257 | 222.1054729 |
| YOL068C | HST1 | 0.89789742 | 0.99917121 | -0.0333997 | 127.1298184 | 206.5086491 |
| YER062C | HOR2 | 0.74782512 | 0.99917121 | -0.0333593 | 117.3356519 | 190.9078583 |
| YAL066W | YAL066W | 0.86472103 | 0.99917121 | -0.033319 | 138.3272745 | 224.3292391 |
| YJR087W | YJR087W | 0.94664588 | 0.99917121 | -0.033301 | 101.7474228 | 166.0786254 |
| YOL006C | TOP1 | 0.89510942 | 0.99917121 | -0.0332383 | 69.72436727 | 115.0786348 |
| YCR086W | CSM1 | 0.89745668 | 0.99917121 | -0.0330327 | 121.0316351 | 196.7533904 |
| YHR109W | CTM1 | 0.86659249 | 0.99917121 | -0.0327279 | 140.3964747 | 227.5520899 |
| YLL010C | PSR1 | 0.8454665 | 0.99917121 | -0.0325182 | 143.9505972 | 233.1859763 |
| YNL120C | YNL120C | 0.77199584 | 0.99917121 | -0.032502 | 94.7574826 | 154.8506962 |

| | | | | | | |
|---|---|---|---|---|---|---|
| YNL335W | DDI3 | 0.8692287 | 0.99917121 | -0.0322581 | 150.3279307 | 243.3093071 |
| YJL099W | CHS6 | 0.85773265 | 0.99917121 | -0.0322253 | 135.0765074 | 219.0195036 |
| YER173W | RAD24 | 0.76946481 | 0.99917121 | -0.0321077 | 124.7982005 | 202.6383605 |
| YHR131C | YHR131C | 0.87537807 | 0.99917121 | -0.0320683 | 145.6943164 | 235.9077736 |
| YPR115W | YPR115W | 0.81503734 | 0.99917121 | -0.0320069 | 149.3592632 | 241.7362073 |
| YJR131W | MNS1 | 0.77453917 | 0.99917121 | -0.0318269 | 133.4781353 | 216.4257437 |
| YOL107W | YOL107W | 0.87029686 | 0.99917121 | -0.0317991 | 135.7303982 | 220.0087686 |
| YBR090C | YBR090C | 0.86399118 | 0.99917121 | -0.0317549 | 149.5333084 | 241.9826336 |
| YKR084C | HBS1 | 0.83641691 | 0.99917121 | -0.0315645 | 123.2418332 | 200.0938418 |
| YKR015C | YKR015C | 0.8568968 | 0.99917121 | -0.0315335 | 125.8144619 | 204.1866138 |
| YDR154C | YDR154C | 0.83450378 | 0.99917121 | -0.0313554 | 142.6434897 | 230.9628332 |
| YDR251W | PAM1 | 0.83105049 | 0.99917121 | -0.0312794 | 152.4609568 | 246.5865414 |
| YBL061C | SKT5 | 0.84173181 | 0.99917121 | -0.0312579 | 134.9266634 | 218.6629527 |
| YDR476C | YDR476C | 0.87226048 | 0.99917121 | -0.0312237 | 140.5197861 | 227.5650664 |
| YOR349W | CIN1 | 0.8680239 | 0.99917121 | -0.0311171 | 139.0751581 | 225.2516989 |
| YMR107W | SPG4 | 0.90429226 | 0.99917121 | -0.0310181 | 125.9567069 | 204.3502881 |
| YKR054C | DYN1 | 0.87791949 | 0.99917121 | -0.0310029 | 130.4511084 | 211.505159 |
| YMR144W | YMR144W | 0.87950943 | 0.99917121 | -0.0308717 | 139.5758733 | 226.0191095 |
| YNL278W | CAF120 | 0.90606145 | 0.99917121 | -0.0308672 | 108.3017263 | 176.2187526 |
| YPL257W | YPL257W | 0.86244803 | 0.99917121 | -0.0308416 | 138.327794 | 224.0280347 |
| YBR044C | TCM62 | 0.79825088 | 0.99917121 | -0.0308136 | 109.097133 | 177.4787858 |
| YFR041C | ERJ5 | 0.85193832 | 0.99917121 | -0.0308124 | 144.7596522 | 234.2663362 |
| YBL047C | EDE1 | 0.83466886 | 0.99917121 | -0.0307786 | 131.7658954 | 213.571432 |
| YDR061W | YDR061W | 0.86225684 | 0.99917121 | -0.0306334 | 147.372409 | 238.404968 |
| YDR153C | ENT5 | 0.82242633 | 0.99917121 | -0.0306099 | 146.1553226 | 236.4640597 |
| YDR385W | EFT2 | 0.87361245 | 0.99917121 | -0.0305031 | 135.1562911 | 218.9365863 |
| YOR298W | MUM3 | 0.85211279 | 0.99917121 | -0.0303794 | 139.3654864 | 225.6240727 |
| YJL196C | ELO1 | 0.74330286 | 0.99917121 | -0.0302567 | 148.9220695 | 240.8266753 |
| YOR152C | YOR152C | 0.85594326 | 0.99917121 | -0.0300264 | 135.9760125 | 220.1837642 |
| YIL138C | TPM2 | 0.87598235 | 0.99917121 | -0.0300223 | 149.7622877 | 242.1360305 |
| YNL044W | YIP3 | 0.83882897 | 0.99917121 | -0.0299236 | 127.331556 | 206.4061129 |
| YMR161W | HLJ1 | 0.8776158 | 0.99917121 | -0.0294353 | 139.6040847 | 225.8889211 |
| YPL105C | YPL105C | 0.8866509 | 0.99917121 | -0.0290017 | 138.2506285 | 223.6808633 |
| YDL099W | BUG1 | 0.83934546 | 0.99917121 | -0.0289349 | 138.8438483 | 224.6173408 |
| YDL238C | GUD1 | 0.80541479 | 0.99917121 | -0.028766 | 153.2416162 | 247.5232291 |
| YDL170W | UGA3 | 0.88952817 | 0.99917121 | -0.0285982 | 133.0499941 | 215.3503703 |
| YDL071C | YDL071C | 0.86705288 | 0.99917121 | -0.0285462 | 139.3338428 | 225.3502017 |
| YNL321W | YNL321W | 0.88374595 | 0.99917121 | -0.0283803 | 139.3558947 | 225.3650949 |
| YFR015C | GSY1 | 0.87079129 | 0.99917121 | -0.0283293 | 142.4524375 | 230.2897007 |
| YJR134C | SGM1 | 0.87673841 | 0.99917121 | -0.0281194 | 146.9796713 | 237.4731088 |
| YGL004C | RPN14 | 0.86554116 | 0.99917121 | -0.0280419 | 142.2450915 | 229.9244883 |
| YKL023W | YKL023W | 0.82137303 | 0.99917121 | -0.027748 | 122.5617585 | 198.5456341 |
| YHR032W | YHR032W | 0.89616569 | 0.99917121 | -0.0277458 | 154.2062843 | 248.9349519 |
| YML074C | FPR3 | 0.85562832 | 0.99917121 | -0.0276562 | 139.3995639 | 225.346353 |
| YOR109W | INP53 | 0.90463043 | 0.99917121 | -0.0275907 | 136.9210274 | 221.3916372 |
| YBL085W | BOI1 | 0.85789036 | 0.99917121 | -0.0275176 | 149.0405637 | 240.6814297 |
| YDR329C | PEX3 | 0.89498692 | 0.99917121 | -0.0274875 | 140.681054 | 227.3663892 |
| YPL249C | GYP5 | 0.84735766 | 0.99917121 | -0.0274442 | 139.9900612 | 226.2607892 |
| YOR178C | GAC1 | 0.88288653 | 0.99917121 | -0.0273479 | 138.2296424 | 223.445831 |
| YDL184C | RPL41A | 0.87140506 | 0.99917121 | -0.0272732 | 130.2858127 | 210.7872512 |
| YOR126C | IAH1 | 0.83737655 | 0.99917121 | -0.027063 | 130.49148 | 211.0891245 |
| YGR233C | PHO81 | 0.86690085 | 0.99917121 | -0.0267891 | 140.5010288 | 226.9945708 |
| YKL039W | PTM1 | 0.83374705 | 0.99917121 | -0.0266236 | 137.8484279 | 222.7504946 |
| YDR256C | CTA1 | 0.88457697 | 0.99917121 | -0.0265813 | 137.6288043 | 222.3956194 |
| YCR100C | YCR100C | 0.89890873 | 0.99917121 | -0.0265168 | 143.9109497 | 232.3912088 |
| YMR250W | GAD1 | 0.89833189 | 0.99917121 | -0.0265096 | 142.5073309 | 230.1552688 |
| YMR152W | YIM1 | 0.88688402 | 0.99917121 | -0.0265061 | 138.7426178 | 224.16004 |
| YBR054W | YRO2 | 0.87115561 | 0.99917121 | -0.0263709 | 145.5282754 | 234.9488027 |
| YOR155C | ISN1 | 0.8422211 | 0.99917121 | -0.0262701 | 131.4006025 | 212.4401132 |
| YJL017W | YJL017W | 0.85235921 | 0.99917121 | -0.0260226 | 135.7368879 | 219.3148937 |
| YDL134C-A | YDL134C-A | 0.87786358 | 0.99917121 | -0.0260195 | 157.3519468 | 253.7335397 |
| YCL038C | ATG22 | 0.84267464 | 0.99917121 | -0.0258502 | 148.4385719 | 239.5195704 |
| YDL036C | PUS9 | 0.88364955 | 0.99917121 | -0.0256792 | 136.9265786 | 221.1674483 |
| YOR213C | SAS5 | 0.86277167 | 0.99917121 | -0.0254883 | 144.0170734 | 232.4348147 |

| | | | | | | |
|---|---|---|---|---|---|---|
| YGL162W | SUT1 | 0.90494258 | 0.99917121 | -0.0254281 | 118.5688785 | 191.9047079 |
| YDL223C | HBT1 | 0.86101668 | 0.99917121 | -0.0252793 | 145.1543409 | 234.2202818 |
| YNR060W | FRE4 | 0.86287072 | 0.99917121 | -0.0252484 | 144.2381397 | 232.7575821 |
| YLR199C | YLR199C | 0.89110483 | 0.99917121 | -0.0251088 | 132.6758201 | 214.3291566 |
| YLR384C | IKI3 | 0.89353302 | 0.99917121 | -0.0249589 | 114.8486691 | 185.9235752 |
| YJR044C | VPS55 | 0.92538114 | 0.99917121 | -0.0249078 | 119.8117029 | 193.820296 |
| YDL201W | TRM8 | 0.86332118 | 0.99917121 | -0.0248617 | 150.1683317 | 242.1534659 |
| YNR032C-A | HUB1 | 0.89908449 | 0.99917121 | -0.0248431 | 139.8283636 | 225.6862127 |
| YBR067C | TIP1 | 0.90268635 | 0.99917121 | -0.0247257 | 146.8736317 | 236.890526 |
| YFL020C | PAU5 | 0.90818876 | 0.99917121 | -0.0247203 | 123.0648651 | 198.977653 |
| YKL005C | BYE1 | 0.83963092 | 0.99917121 | -0.0244198 | 126.7466506 | 204.8037594 |
| YOR270C | VPH1 | 0.90548684 | 0.99917121 | -0.0243849 | 107.9796366 | 174.9156045 |
| YNL027W | CRZ1 | 0.88994851 | 0.99917121 | -0.0240767 | 142.0678966 | 229.1589322 |
| YIL120W | QDR1 | 0.89934543 | 0.99917121 | -0.0238458 | 139.1641477 | 224.5069568 |
| YNR034W | SOL1 | 0.90820224 | 0.99917121 | -0.0236954 | 118.8800418 | 192.1889583 |
| YJR130C | STR2 | 0.91879291 | 0.99917121 | -0.0236499 | 128.2172652 | 207.0516621 |
| YDR357C | YDR357C | 0.92984024 | 0.99917121 | -0.0235257 | 129.7412809 | 209.4633029 |
| YOL067C | RTG1 | 0.93031225 | 0.99917121 | -0.0234011 | 113.2230593 | 183.1450981 |
| YPR156C | TPO3 | 0.92576785 | 0.99917121 | -0.0233197 | 123.7805084 | 199.9464739 |
| YJL037W | IRC18 | 0.88561491 | 0.99917121 | -0.0232907 | 143.5750412 | 231.463029 |
| YAR028W | YAR028W | 0.9074666 | 0.99917121 | -0.0230456 | 129.4045347 | 208.8685583 |
| YBR201W | DER1 | 0.87369449 | 0.99917121 | -0.022968 | 144.7608692 | 233.3119572 |
| YKL026C | GPX1 | 0.89366492 | 0.99917121 | -0.0229056 | 145.896231 | 235.1122617 |
| YPL225W | YPL225W | 0.94420719 | 0.99917121 | -0.0228952 | 121.3162751 | 195.9707621 |
| YJL044C | GYP6 | 0.85214614 | 0.99917121 | -0.0228657 | 152.646571 | 245.856391 |
| YLR224W | YLR224W | 0.91135478 | 0.99917121 | -0.0228496 | 149.7948726 | 241.313483 |
| YCR025C | YCR025C | 0.90985429 | 0.99917121 | -0.0225314 | 130.6845226 | 210.8440759 |
| YER004W | FMP52 | 0.89827111 | 0.99917121 | -0.0225186 | 133.9937032 | 216.1119302 |
| YKR050W | TRK2 | 0.86506747 | 0.99917121 | -0.0223898 | 136.2714214 | 219.7231811 |
| YDR121W | DPB4 | 0.892172 | 0.99917121 | -0.0223383 | 149.0863034 | 240.1228585 |
| YKR099W | BAS1 | 0.89467656 | 0.99917121 | -0.0222969 | 145.3675352 | 234.1961754 |
| YJL098W | SAP185 | 0.88977289 | 0.99917121 | -0.0222121 | 150.0062159 | 241.5723054 |
| YDR122W | KIN1 | 0.85043258 | 0.99917121 | -0.0221572 | 142.5353386 | 229.66926 |
| YDR260C | SWM1 | 0.90459158 | 0.99917121 | -0.022071 | 144.8223014 | 233.3004218 |
| YPL103C | FMP30 | 0.89991988 | 0.99917121 | -0.0220475 | 134.9464147 | 217.5715588 |
| YBR046C | ZTA1 | 0.9040393 | 0.99917121 | -0.022039 | 146.8165393 | 236.472079 |
| YLR349W | YLR349W | 0.90536169 | 0.99917121 | -0.0220361 | 139.4954243 | 224.8138518 |
| YHR097C | YHR097C | 0.91915941 | 0.99917121 | -0.0219969 | 150.6182397 | 242.5206302 |
| YDR096W | GIS1 | 0.90438098 | 0.99917121 | -0.0218518 | 142.552712 | 229.6596889 |
| YGL117W | YGL117W | 0.88302363 | 0.99917121 | -0.0218273 | 138.9630962 | 223.9407328 |
| YOR375C | GDH1 | 0.898209 | 0.99917121 | -0.0216878 | 137.6649315 | 221.856577 |
| YMR261C | TPS3 | 0.88609083 | 0.99917121 | -0.0215578 | 121.1629089 | 195.5635161 |
| YLR206W | ENT2 | 0.93561829 | 0.99917121 | -0.0213839 | 118.9575015 | 192.0304958 |
| YDR070C | FMP16 | 0.85466553 | 0.99917121 | -0.0213737 | 152.8079238 | 245.9314327 |
| YOR320C | GNT1 | 0.87059174 | 0.99917121 | -0.0213129 | 129.92704 | 209.4893401 |
| YDR354W | TRP4 | 0.90906335 | 0.99917121 | -0.0212164 | 136.4591221 | 219.8790162 |
| YNL162W | RPL42A | 0.87694223 | 0.99917121 | -0.0210873 | 121.0095255 | 195.261903 |
| YGR126W | YGR126W | 0.87386465 | 0.99917121 | -0.0208574 | 144.4784212 | 232.6048963 |
| YOR064C | YNG1 | 0.8894613 | 0.99917121 | -0.0208101 | 147.1584961 | 236.866781 |
| YDR171W | HSP42 | 0.89873338 | 0.99917121 | -0.0207904 | 144.4260384 | 232.5133121 |
| YLR098C | CHA4 | 0.9373601 | 0.99917121 | -0.020505 | 108.5030656 | 175.2760879 |
| YPL032C | SVL3 | 0.91304447 | 0.99917121 | -0.0204942 | 139.2570459 | 224.2462847 |
| YGR223C | HSV2 | 0.92062861 | 0.99917121 | -0.0204784 | 113.8521395 | 183.7905203 |
| YDR423C | CAD1 | 0.91679508 | 0.99917121 | -0.0202887 | 141.469712 | 227.7446094 |
| YPL055C | LGE1 | 0.91567629 | 0.99917121 | -0.0202615 | 106.0568292 | 171.3511029 |
| YHR155W | YSP1 | 0.92839526 | 0.99917121 | -0.0201899 | 151.9871215 | 244.4800937 |
| YIL016W | SNL1 | 0.9309161 | 0.99917121 | -0.0201852 | 145.7879801 | 234.6082434 |
| YDR216W | ADR1 | 0.90298651 | 0.99917121 | -0.0200622 | 143.6545553 | 231.1960604 |
| YMR154C | RIM13 | 0.86526432 | 0.99917121 | -0.02002 | 117.7587926 | 189.9554512 |
| YDR102C | YDR102C | 0.89204849 | 0.99917121 | -0.0198713 | 146.6117764 | 235.8817515 |
| YER084W | YER084W | 0.91533055 | 0.99917121 | -0.0196837 | 139.7901441 | 224.9963641 |
| YBL086C | YBL086C | 0.8991372 | 0.99917121 | -0.0196735 | 149.4272096 | 240.3408309 |
| YKL096W | CWP1 | 0.90827038 | 0.99917121 | -0.0196725 | 150.0826436 | 241.3843992 |
| YAR050W | FLO1 | 0.88465326 | 0.99917121 | -0.019617 | 152.6035478 | 245.3918349 |

| | | | | | | |
|---|---|---|---|---|---|---|
| YHR142W | CHS7 | 0.9322032 | 0.99917121 | -0.0196165 | 140.3362485 | 225.8577718 |
| YDR294C | DPL1 | 0.9049665 | 0.99917121 | -0.0195593 | 138.0313127 | 222.1805031 |
| YNR061C | YNR061C | 0.88278728 | 0.99917121 | -0.0195591 | 128.7057217 | 207.3307462 |
| YOR216C | RUD3 | 0.90742803 | 0.99917121 | -0.0194927 | 133.0958743 | 214.3133642 |
| YGR230W | BNS1 | 0.90272298 | 0.99917121 | -0.0191857 | 151.6449797 | 243.8128639 |
| YAL017W | PSK1 | 0.91065079 | 0.99917121 | -0.0191848 | 144.3817624 | 232.2470679 |
| YPL191C | YPL191C | 0.92377387 | 0.99917121 | -0.0191486 | 131.1729895 | 211.2094923 |
| YER149C | PEA2 | 0.91904575 | 0.99917121 | -0.0187415 | 152.1941261 | 244.6331458 |
| YOR051C | YOR051C | 0.91673751 | 0.99917121 | -0.0186597 | 137.3005474 | 220.9071885 |
| YBL065W | YBL065W | 0.89736539 | 0.99917121 | -0.0186574 | 141.2411388 | 227.1817635 |
| YOR381W | FRE3 | 0.94266279 | 0.99917121 | -0.0185886 | 133.556089 | 214.9359779 |
| YPR077C | YPR077C | 0.92243899 | 0.99917121 | -0.0185665 | 144.4208385 | 232.233918 |
| YPL247C | YPL247C | 0.87929906 | 0.99917121 | -0.0185663 | 146.7495206 | 235.9420031 |
| YDL011C | YDL011C | 0.90130643 | 0.99917121 | -0.0185631 | 144.7516912 | 232.7603394 |
| YMR027W | YMR027W | 0.84671098 | 0.99917121 | -0.0181616 | 119.9217006 | 193.1730206 |
| YOR115C | TRS33 | 0.92566339 | 0.99917121 | -0.0181497 | 141.3007154 | 227.2147386 |
| YDR107C | YDR107C | 0.91507058 | 0.99917121 | -0.0178842 | 145.4860761 | 233.8469803 |
| YGR139W | YGR139W | 0.900695 | 0.99917121 | -0.0177981 | 142.3426868 | 228.8310704 |
| YPL009C | YPL009C | 0.92008255 | 0.99917121 | -0.0177779 | 136.2891717 | 219.1892036 |
| YGR010W | NMA2 | 0.89339995 | 0.99917121 | -0.0177504 | 129.5720732 | 208.4897915 |
| YCR083W | TRX3 | 0.90451318 | 0.99917121 | -0.017519 | 136.9867283 | 220.268412 |
| YGR164W | YGR164W | 0.89619421 | 0.99917121 | -0.0172705 | 151.2874256 | 243.0100207 |
| YPL095C | EEB1 | 0.90984356 | 0.99917121 | -0.0169745 | 142.6244194 | 229.1792783 |
| YOR082C | YOR082C | 0.94163689 | 0.99917121 | -0.0168265 | 129.7244874 | 208.6198592 |
| YKL010C | UFD4 | 0.89728762 | 0.99917121 | -0.016731 | 130.5472901 | 209.9184221 |
| YNR012W | URK1 | 0.94363499 | 0.99917121 | -0.0167264 | 141.1909157 | 226.8663772 |
| YDL156W | YDL156W | 0.90674138 | 0.99917121 | -0.0167247 | 138.2184335 | 222.1328989 |
| YMR326C | YMR326C | 0.93175207 | 0.99917121 | -0.0165985 | 129.2821668 | 207.8877292 |
| YIL146C | ECM37 | 0.91920847 | 0.99917121 | -0.0165916 | 130.9866436 | 210.6010383 |
| YHR087W | RTC3 | 0.94140175 | 0.99917121 | -0.0165794 | 131.5981472 | 211.5732758 |
| YIL087C | YIL087C | 0.91820628 | 0.99917121 | -0.0164703 | 141.1132839 | 226.7115344 |
| YFR048W | RMD8 | 0.92828446 | 0.99917121 | -0.0163615 | 143.6264686 | 230.7001735 |
| YPL223C | GRE1 | 0.91986859 | 0.99917121 | -0.0163311 | 134.192749 | 215.6745555 |
| YKL221W | MCH2 | 0.8947103 | 0.99917121 | -0.0163099 | 118.6276563 | 190.8866896 |
| YEL014C | YEL014C | 0.94181859 | 0.99917121 | -0.0160337 | 131.7878146 | 211.808771 |
| YDR271C | YDR271C | 0.95948561 | 0.99917121 | -0.0159303 | 121.6641181 | 195.6755662 |
| YBR001C | NTH2 | 0.93272717 | 0.99917121 | -0.0157993 | 140.7438106 | 226.0414018 |
| YLL046C | RNP1 | 0.89363369 | 0.99917121 | -0.0157902 | 113.3944164 | 182.490115 |
| YPL241C | CIN2 | 0.94485055 | 0.99917121 | -0.015644 | 137.3964739 | 220.6922902 |
| YBR024W | SCO2 | 0.9358857 | 0.99917121 | -0.0154496 | 146.4714038 | 235.1191745 |
| YJL147C | YJL147C | 0.93768522 | 0.99917121 | -0.0153833 | 133.5122978 | 214.475487 |
| YBR170C | NPL4 | 0.94403303 | 0.99917121 | -0.015319 | 130.5637889 | 209.7725555 |
| YDR517W | GRH1 | 0.94087933 | 0.99917121 | -0.0152392 | 132.5506786 | 212.9266696 |
| YKR056W | TRM2 | 0.92436578 | 0.99917121 | -0.0152054 | 139.7285339 | 224.3523085 |
| YDL042C | SIR2 | 0.90140499 | 0.99917121 | -0.0151209 | 133.9362621 | 215.1186009 |
| YMR271C | URA10 | 0.94460419 | 0.99917121 | -0.0149597 | 143.7833965 | 230.7791734 |
| YLR420W | URA4 | 0.93342324 | 0.99917121 | -0.0149539 | 131.2788108 | 210.8666104 |
| YDR010C | YDR010C | 0.92580846 | 0.99917121 | -0.0148779 | 144.3169455 | 231.6187966 |
| YNL202W | SPS19 | 0.92666016 | 0.99917121 | -0.0148246 | 156.8166792 | 251.5164165 |
| YDR090C | YDR090C | 0.91477189 | 0.99917121 | -0.0148193 | 147.8770382 | 237.2806122 |
| YER059W | PCL6 | 0.91706687 | 0.99917121 | -0.0147303 | 139.6584291 | 224.1827567 |
| YDR152W | GIR2 | 0.93250755 | 0.99917121 | -0.0147123 | 138.4927337 | 222.3243503 |
| YDR131C | YDR131C | 0.93147028 | 0.99917121 | -0.0145665 | 145.2042406 | 232.993728 |
| YGR077C | PEX8 | 0.95465487 | 0.99917121 | -0.0145428 | 118.5073659 | 190.4797197 |
| YPL180W | TCO89 | 0.88360088 | 0.99917121 | -0.014505 | 87.55982679 | 141.1953791 |
| YLL048C | YBT1 | 0.91306564 | 0.99917121 | -0.0143815 | 123.5458361 | 198.4831338 |
| YGL053W | PRM8 | 0.92759734 | 0.99917121 | -0.0142641 | 143.2063364 | 229.7754793 |
| YPL163C | SVS1 | 0.94136575 | 0.99917121 | -0.0142025 | 135.9571065 | 218.2245572 |
| YKR087C | OMA1 | 0.93429683 | 0.99917121 | -0.0141716 | 142.5755185 | 228.7597094 |
| YGR268C | HUA1 | 0.88612497 | 0.99917121 | -0.0141172 | 165.21264 | 264.7996044 |
| YNL047C | SLM2 | 0.96072065 | 0.99917121 | -0.0140971 | 127.9049547 | 205.3897633 |
| YBL001C | ECM15 | 0.93523627 | 0.99917121 | -0.0140703 | 146.702811 | 235.3195087 |
| YML003W | YML003W | 0.95511737 | 0.99917121 | -0.0139893 | 136.9878666 | 219.8399132 |
| YLR217W | YLR217W | 0.94100453 | 0.99917121 | -0.0138295 | 137.3717024 | 220.4316372 |

| | | | | | | |
|---|---|---|---|---|---|---|
| YDR409W | SIZ1 | 0.93745871 | 0.99917121 | -0.0137699 | 142.7227534 | 228.945189 |
| YER092W | IES5 | 0.90821436 | 0.99917121 | -0.0137477 | 7.231336294 | 13.19090227 |
| YJL158C | CIS3 | 0.93720398 | 0.99917121 | -0.0137321 | 146.6902413 | 235.2582691 |
| YFL040W | YFL040W | 0.9516708 | 0.99917121 | -0.0137316 | 121.3629998 | 194.9280347 |
| YOL061W | PRS5 | 0.94453485 | 0.99917121 | -0.0136846 | 133.7226027 | 214.6032767 |
| YNR065C | YSN1 | 0.94151297 | 0.99917121 | -0.0136713 | 137.5994122 | 220.7749473 |
| YPL177C | CUP9 | 0.94282208 | 0.99917121 | -0.0135073 | 144.9256997 | 232.4210672 |
| YMR251W | GTO3 | 0.94808219 | 0.99917121 | -0.0135061 | 140.7690399 | 225.8020046 |
| YJL157C | FAR1 | 0.93264085 | 0.99917121 | -0.0134993 | 144.401143 | 231.5848107 |
| YJL137C | GLG2 | 0.93286041 | 0.99917121 | -0.0134125 | 147.2431454 | 236.09973 |
| YML058W | SML1 | 0.95778407 | 0.99917121 | -0.0133345 | 126.0236363 | 202.3010484 |
| YDR093W | DNF2 | 0.934959 | 0.99917121 | -0.0132784 | 152.3602915 | 244.231732 |
| YOR289W | YOR289W | 0.93206499 | 0.99917121 | -0.0131827 | 139.9223266 | 224.4143057 |
| YPL171C | OYE3 | 0.93052968 | 0.99917121 | -0.0130996 | 139.9822163 | 224.4995461 |
| YIL090W | ICE2 | 0.95881525 | 0.99917121 | -0.0129055 | 111.6543022 | 179.3675502 |
| YMR258C | YMR258C | 0.91758891 | 0.99917121 | -0.0127566 | 134.4408076 | 215.6337917 |
| YAR031W | PRM9 | 0.93819084 | 0.99917121 | -0.0126987 | 146.250719 | 234.4324066 |
| YDL089W | YDL089W | 0.98614826 | 0.99917121 | -0.0123875 | 109.6809495 | 176.1621058 |
| YOR086C | TCB1 | 0.94981012 | 0.99917121 | -0.01203 | 147.354293 | 236.1081758 |
| YMR096W | SNZ1 | 0.93311444 | 0.99917121 | -0.0120112 | 138.7488908 | 222.4029569 |
| YDL239C | ADY3 | 0.93099652 | 0.99917121 | -0.0119775 | 147.6200374 | 236.5249319 |
| YJR037W | YJR037W | 0.93004694 | 0.99917121 | -0.0118176 | 145.7407501 | 233.5129272 |
| YPL159C | PET20 | 0.95109164 | 0.99917121 | -0.0115565 | 108.276276 | 173.8240383 |
| YJR059W | PTK2 | 0.91828539 | 0.99917121 | -0.0114612 | 133.1037303 | 213.3467565 |
| YPL232W | SSO1 | 0.95286083 | 0.99917121 | -0.0110989 | 139.2585311 | 223.1032604 |
| YNL173C | MDG1 | 0.96860088 | 0.99917121 | -0.0110229 | 140.8979754 | 225.7045888 |
| YPL022W | RAD1 | 0.93970856 | 0.99917121 | -0.0109552 | 156.1617592 | 250.0018244 |
| YOR303W | CPA1 | 0.94368228 | 0.99917121 | -0.010886 | 138.4580772 | 221.8027003 |
| YDL215C | GDH2 | 0.94697382 | 0.99917121 | -0.0107702 | 144.7046817 | 231.7354466 |
| YDR290W | :::RTT103 | 0.96622118 | 0.99917121 | -0.0106907 | 117.5837598 | 188.5393917 |
| YIL035C | CKA1 | 0.98641929 | 0.99917121 | -0.010618 | 95.59069536 | 153.5095753 |
| YKL098W | MTC2 | 0.93880553 | 0.99917121 | -0.0106047 | 125.8942082 | 201.7621622 |
| YGR243W | FMP43 | 0.95885531 | 0.99917121 | -0.0105766 | 146.481198 | 234.5406993 |
| YDL178W | DLD2 | 0.94871627 | 0.99917121 | -0.0105634 | 145.1870453 | 232.478329 |
| YDR147W | EKI1 | 0.93511022 | 0.99917121 | -0.0104396 | 150.7237825 | 241.2797384 |
| YLR300W | EXG1 | 0.94484528 | 0.99917121 | -0.0103595 | 148.2102158 | 237.2674578 |
| YFR031C-A | RPL2A | 0.97335278 | 0.99917121 | -0.0103181 | 107.4340576 | 172.3319518 |
| YPL008W | CHL1 | 0.96138445 | 0.99917121 | -0.0100194 | 122.1760325 | 195.7701169 |
| YOR230W | WTM1 | 0.95782431 | 0.99917121 | -0.0099933 | 140.4083787 | 224.7994507 |
| YDR185C | YDR185C | 0.95138368 | 0.99917121 | -0.0099631 | 150.0929764 | 240.2171787 |
| YOR239W | ABP140 | 0.95180108 | 0.99917121 | -0.0099091 | 141.1269756 | 225.9334552 |
| YPL039W | YPL039W | 0.94750874 | 0.99917121 | -0.0096356 | 143.5437828 | 229.7485532 |
| YPL161C | BEM4 | 0.96808953 | 0.99917121 | -0.0094263 | 114.0980071 | 182.8346629 |
| YIL130W | ASG1 | 0.95621797 | 0.99917121 | -0.0094176 | 138.6541764 | 221.935945 |
| YOR018W | ROD1 | 0.96791852 | 0.99917121 | -0.0093934 | 124.1501341 | 198.8372931 |
| YBL054W | YBL054W | 0.94746144 | 0.99917121 | -0.0092583 | 149.3987155 | 239.0257389 |
| YOL025W | LAG2 | 0.95973274 | 0.99917121 | -0.0092523 | 150.2182873 | 240.330063 |
| YER188W | YER188W | 0.96059068 | 0.99917121 | -0.0092491 | 144.0394867 | 230.4907702 |
| YGL224C | SDT1 | 0.96465091 | 0.99917121 | -0.0092449 | 135.7967075 | 217.364764 |
| YDL197C | ASF2 | 0.94856107 | 0.99917121 | -0.0092028 | 140.2383966 | 224.432415 |
| YNL145W | MFA2 | 0.95821252 | 0.99917121 | -0.0091964 | 130.2565364 | 208.5368846 |
| YJL136C | RPS21B | 0.95696583 | 0.99917121 | -0.0089968 | 129.2106296 | 206.847084 |
| YNR071C | YNR071C | 0.96417713 | 0.99917121 | -0.0088599 | 146.1438651 | 233.794259 |
| YDR163W | CWC15 | 0.95600384 | 0.99917121 | -0.0088263 | 142.2548679 | 227.5974645 |
| YDL129W | YDL129W | 0.96415553 | 0.99917121 | -0.008788 | 148.4363003 | 237.4358808 |
| YNL288W | CAF40 | 0.97126554 | 0.99917121 | -0.0084198 | 131.6918688 | 210.7277767 |
| YDR363W-A | SEM1 | 0.95008002 | 0.99917121 | -0.0083431 | 118.2307616 | 189.2834511 |
| YMR041C | YMR041C | 0.94643743 | 0.99917121 | -0.0083339 | 145.8482961 | 233.2594774 |
| YGR256W | GND2 | 0.96554679 | 0.99917121 | -0.008316 | 140.244366 | 224.3338013 |
| YDR015C | YDR015C | 0.96708182 | 0.99917121 | -0.0081777 | 144.8396333 | 231.6342827 |
| YGL140C | YGL140C | 0.95610255 | 0.99917121 | -0.0081445 | 148.68703 | 237.7566885 |
| YGR117C | YGR117C | 0.95892066 | 0.99917121 | -0.0080135 | 142.821021 | 228.3999043 |
| YKL030W | YKL030W | 0.95870455 | 0.99917121 | -0.0079495 | 146.6735299 | 234.5266942 |
| YLR137W | YLR137W | 0.96571063 | 0.99917121 | -0.0079177 | 136.9062262 | 218.9697218 |

| | | | | | | |
|---|---|---|---|---|---|---|
| YDL037C | BSC1 | 0.95691331 | 0.99917121 | -0.0078723 | 143.9737057 | 230.218179 |
| YOR245C | DGA1 | 0.96353939 | 0.99917121 | -0.0078578 | 131.88174 | 210.9616028 |
| YBR222C | PCS60 | 0.96370025 | 0.99917121 | -0.0078492 | 149.9030402 | 239.657015 |
| YPL226W | NEW1 | 0.98720927 | 0.99917121 | -0.0077223 | 98.04508276 | 157.0648307 |
| YOL085C | YOL085C | 0.97092661 | 0.99917121 | -0.007667 | 136.4281477 | 218.1778828 |
| YGR237C | YGR237C | 0.97210664 | 0.99917121 | -0.0075099 | 153.6009895 | 245.5041385 |
| YPL089C | RLM1 | 0.96959755 | 0.99917121 | -0.0074712 | 142.0580234 | 227.1188131 |
| YPL181W | CTI6 | 0.9637575 | 0.99917121 | -0.0074667 | 141.9605601 | 226.9630676 |
| YJR103W | URA8 | 0.96849344 | 0.99917121 | -0.0073871 | 149.2444788 | 238.552014 |
| YBL091C | MAP2 | 0.96571385 | 0.99917121 | -0.0073634 | 151.9787072 | 242.9030115 |
| YER155C | BEM2 | 0.97239058 | 0.99917121 | -0.0069602 | 134.2497308 | 214.6228789 |
| YOR118W | RTC5 | 0.97189256 | 0.99917121 | -0.0068118 | 139.3493354 | 222.7252164 |
| YIL114C | POR2 | 0.97401116 | 0.99917121 | -0.0067376 | 150.9510331 | 241.1902908 |
| YNL106C | INP52 | 0.94620476 | 0.99917121 | -0.0065958 | 103.5194194 | 165.6446363 |
| YNR056C | BIO5 | 0.96676719 | 0.99917121 | -0.0064553 | 151.2260028 | 241.5937282 |
| YLR077W | FMP25 | 0.97520153 | 0.99917121 | -0.0063396 | 134.7483839 | 215.3412656 |
| YGL144C | ROG1 | 0.94994647 | 0.99917121 | -0.0062585 | 165.3361272 | 264.0381747 |
| YKR089C | TGL4 | 0.9701265 | 0.99917121 | -0.0061333 | 135.9646043 | 217.2527754 |
| YMR216C | SKY1 | 0.92854766 | 0.99917121 | -0.0059573 | 105.2315286 | 168.2930992 |
| YOR188W | MSB1 | 0.98183549 | 0.99917121 | -0.0057996 | 144.1540227 | 230.25263 |
| YCL014W | BUD3 | 0.99356303 | 0.99917121 | -0.0057492 | 108.4915189 | 173.458818 |
| YKL025C | PAN3 | 0.97450077 | 0.99917121 | -0.0057174 | 139.635688 | 223.0477774 |
| YOL081W | IRA2 | 0.98632889 | 0.99917121 | -0.0057042 | 96.80260086 | 154.8403262 |
| YKL131W | YKL131W | 0.98205985 | 0.99917121 | -0.0056826 | 142.5530528 | 227.6890419 |
| YOL037C | YOL037C | 0.97764493 | 0.99917121 | -0.005481 | 143.3087938 | 228.8678672 |
| YKL164C | PIR1 | 0.97189679 | 0.99917121 | -0.0054184 | 138.7678072 | 221.6293453 |
| YDL213C | NOP6 | 0.97801354 | 0.99917121 | -0.0052761 | 142.0679346 | 226.8669941 |
| YNR039C | ZRG17 | 0.9793478 | 0.99917121 | -0.0052218 | 145.8925403 | 232.9505426 |
| YFL018C | LPD1 | 0.98553779 | 0.99917121 | -0.0051543 | 110.8049274 | 177.0700821 |
| YDR281C | PHM6 | 0.97107246 | 0.99917121 | -0.0049688 | 144.0721073 | 230.0209009 |
| YGL138C | YGL138C | 0.973458 | 0.99917121 | -0.0049666 | 145.3110243 | 231.9934452 |
| YBR008C | FLR1 | 0.97707786 | 0.99917121 | -0.0049433 | 142.7791328 | 227.9589043 |
| YKL183W | LOT5 | 0.97661215 | 0.99917121 | -0.0048098 | 135.3701112 | 216.1447744 |
| YBL042C | FUI1 | 0.97954904 | 0.99917121 | -0.0046586 | 145.8841815 | 232.8685662 |
| YKR043C | YKR043C | 0.98025565 | 0.99917121 | -0.0046011 | 140.8316433 | 224.816085 |
| YDL024C | DIA3 | 0.97586001 | 0.99917121 | -0.0045398 | 151.9297635 | 242.480846 |
| YFR016C | YFR016C | 0.97651836 | 0.99917121 | -0.0043267 | 140.0947438 | 223.6092216 |
| YBL027W | RPL19B | 0.9893502 | 0.99917121 | -0.0043051 | 109.9450841 | 175.5973661 |
| YMR289W | ABZ2 | 0.98193639 | 0.99917121 | -0.0042783 | 132.2430583 | 211.100584 |
| YGR108W | CLB1 | 0.97525322 | 0.99917121 | -0.0042554 | 141.7761227 | 226.2778912 |
| YBR134W | YBR134W | 0.98100265 | 0.99917121 | -0.0040718 | 146.9637515 | 234.5160953 |
| YOR092W | ECM3 | 0.98437302 | 0.99917121 | -0.0040148 | 142.3510384 | 227.1640316 |
| YLR221C | RSA3 | 0.98408307 | 0.99917121 | -0.0038131 | 137.3864879 | 219.234075 |
| YMR145C | NDE1 | 0.97868344 | 0.99917121 | -0.0037379 | 88.51172252 | 141.3985169 |
| YOL018C | TLG2 | 0.98102025 | 0.99917121 | -0.0036957 | 115.9883305 | 185.1461155 |
| YOL084W | PHM7 | 0.98811757 | 0.99917121 | -0.0034024 | 146.8182176 | 234.2027524 |
| YCR010C | ADY2 | 0.98374007 | 0.99917121 | -0.0032744 | 142.0359136 | 226.5719811 |
| YLR142W | PUT1 | 0.98670831 | 0.99917121 | -0.0030781 | 138.9185814 | 221.5841243 |
| YJR121W | ATP2 | 0.98130565 | 0.99917121 | -0.0030653 | 141.8703002 | 226.2827666 |
| YML076C | WAR1 | 0.98805665 | 0.99917121 | -0.0030026 | 117.0096308 | 186.6879072 |
| YLR368W | MDM30 | 0.99356638 | 0.99917121 | -0.0029496 | 131.3667578 | 209.5432042 |
| YER019W | ISC1 | 0.98890871 | 0.99917121 | -0.0027295 | 140.6757941 | 224.3397393 |
| YOR172W | YRM1 | 0.98919025 | 0.99917121 | -0.0026871 | 143.884917 | 229.4446589 |
| YOL032W | OPI10 | 0.98830631 | 0.99917121 | -0.0023921 | 135.3275964 | 215.7823333 |
| YML100W-A | YML100W-A | 0.99006009 | 0.99917121 | -0.0023491 | 141.8088594 | 226.0976151 |
| YMR060C | SAM37 | 0.98100011 | 0.99917121 | -0.0022348 | 93.65846667 | 149.4107614 |
| YCR015C | YCR015C | 0.98747872 | 0.99917121 | -0.0022177 | 148.6867047 | 237.0336268 |
| YJL059W | YHC3 | 0.99055102 | 0.99917121 | -0.0021573 | 145.1403498 | 231.379179 |
| YCL060C | :::MRC1 | 0.98810589 | 0.99917121 | -0.002138 | 139.3570572 | 222.167725 |
| YNL249C | MPA43 | 0.98846234 | 0.99917121 | -0.0021001 | 134.142535 | 213.8596897 |
| YPR118W | YPR118W | 0.99171126 | 0.99917121 | -0.0018547 | 133.5648012 | 212.9098139 |
| YNL116W | DMA2 | 0.9896806 | 0.99917121 | -0.0017995 | 132.6293662 | 211.4135294 |
| YJL161W | FMP33 | 0.99320941 | 0.99917121 | -0.0017914 | 142.4829791 | 227.1030712 |
| YAR023C | YAR023C | 0.99320671 | 0.99917121 | -0.0017127 | 149.1224793 | 237.6659779 |

| | | | | | | |
|---|---|---|---|---|---|---|
| YMR315W | YMR315W | 0.99277102 | 0.99917121 | -0.0014906 | 126.1444515 | 201.0495285 |
| YPL051W | ARL3 | 0.99297336 | 0.99917121 | -0.0014869 | 138.6173303 | 220.9104335 |
| YER113C | YER113C | 0.99365063 | 0.99917121 | -0.0014377 | 144.8632802 | 230.8502539 |
| YOR316C | COT1 | 0.99352285 | 0.99917121 | -0.0013132 | 148.8330887 | 237.1564572 |
| YHR093W | AHT1 | 0.99252199 | 0.99917121 | -0.0012084 | 154.1269604 | 245.5734448 |
| YLR426W | YLR426W | 0.99013307 | 0.99917121 | -0.0012051 | 118.2910105 | 188.5091904 |
| YPR007C | REC8 | 0.99655051 | 1 | -0.0011315 | 125.8997062 | 200.6160295 |
| YDR112W | YDR112W | 0.99417035 | 0.99917121 | -0.0011234 | 149.0646868 | 237.5021047 |
| YJL016W | YJL016W | 0.99484531 | 0.99926684 | -0.0011133 | 148.7639579 | 237.0220033 |
| YJR148W | BAT2 | 0.99412222 | 0.99917121 | -0.0009906 | 158.0210793 | 251.7477501 |
| YMR019W | STB4 | 0.99528454 | 0.99940883 | -0.0008848 | 139.7002281 | 222.5613997 |
| YDR346C | SVF1 | 0.99428472 | 0.99917121 | -0.0006852 | 138.6042211 | 220.7918266 |
| YDL027C | YDL027C | 0.99832108 | 1 | -0.0003608 | 149.5309505 | 238.1516065 |
| YOL138C | RTC1 | 0.99854485 | 1 | -0.0002507 | 117.0470825 | 186.4120543 |
| YPL098C | MGR2 | 0.99932101 | 1 | -0.0001661 | 123.830572 | 197.2035217 |
| YLR154C | RNH203 | 0.99855854 | 1 | -0.0001566 | 108.7550932 | 173.1967287 |
| YGR088W | CTT1 | 0.99962578 | 1 | -0.0000725 | 139.2866667 | 221.8038332 |
| YGL151W | NUT1 | 1 | 1 | 0 | 0 | 0 |
| YKL101W | HSL1 | 1 | 1 | 0 | 0 | 0 |
| YDR020C | YDR020C | 0.99976028 | 1 | 0.0000616 | 141.4575 | 225.2442369 |
| YIR038C | GTT1 | 0.99960416 | 1 | 0.00010185 | 136.6058226 | 217.5136985 |
| YMR034C | YMR034C | 0.99823591 | 1 | 0.00037137 | 134.7549446 | 214.53357 |
| YER116C | SLX8 | 0.99863525 | 1 | 0.0003742 | 123.8437057 | 197.1585671 |
| YPL109C | YPL109C | 0.99807266 | 1 | 0.00041665 | 130.0140537 | 206.9788293 |
| YKL133C | YKL133C | 0.99760266 | 1 | 0.00048525 | 135.9312391 | 216.3927753 |
| YLR363C | NMD4 | 0.99677222 | 1 | 0.0007715 | 143.0702841 | 227.7258323 |
| YGR266W | YGR266W | 0.99709536 | 1 | 0.0008419 | 149.7387485 | 238.3358692 |
| YLL007C | YLL007C | 0.99560587 | 0.99940883 | 0.00111573 | 133.0504051 | 211.728579 |
| YGR068C | YGR068C | 0.99376402 | 0.99917121 | 0.00121192 | 131.1884238 | 208.7519012 |
| YKR044W | UIP5 | 0.99395186 | 0.99917121 | 0.00132131 | 141.0836601 | 224.495377 |
| YDR344C | YDR344C | 0.98995187 | 0.99917121 | 0.00132334 | 149.2372804 | 237.4786568 |
| YDR506C | YDR506C | 0.99337037 | 0.99917121 | 0.00135697 | 110.0689529 | 175.1043456 |
| YBR212W | NGR1 | 0.99380263 | 0.99917121 | 0.0014269 | 133.0421283 | 211.677464 |
| YKL063C | YKL063C | 0.99160306 | 0.99917121 | 0.00146381 | 134.7494944 | 214.3917115 |
| YHR073W | OSH3 | 0.9945184 | 0.9991722 | 0.00158031 | 141.3030136 | 224.8130929 |
| YPL147W | PXA1 | 0.99195232 | 0.99917121 | 0.0016428 | 141.2749952 | 224.7608598 |
| YPL208W | RKM1 | 0.9956849 | 0.99940883 | 0.00169162 | 122.569135 | 194.9683801 |
| YIL149C | MLP2 | 0.99008731 | 0.99917121 | 0.00185679 | 144.6796091 | 230.1561547 |
| YCR098C | GIT1 | 0.99248775 | 0.99917121 | 0.00197669 | 144.8609195 | 230.4302488 |
| YGL156W | AMS1 | 0.99015314 | 0.99917121 | 0.00201282 | 147.3075399 | 234.3217529 |
| YGL197W | MDS3 | 0.98467879 | 0.99917121 | 0.00210847 | 131.4787987 | 209.1049844 |
| YKR076W | ECM4 | 0.98877297 | 0.99917121 | 0.00217051 | 142.9969718 | 227.438538 |
| YER179W | DMC1 | 0.99097077 | 0.99917121 | 0.0022323 | 124.0318362 | 197.2316175 |
| YNR058W | BIO3 | 0.98933854 | 0.99917121 | 0.00227978 | 146.2690828 | 232.6356068 |
| YER178W | PDA1 | 0.99069297 | 0.99917121 | 0.00243943 | 131.0828512 | 208.4341443 |
| YDR336W | YDR336W | 0.98778262 | 0.99917121 | 0.00255205 | 139.2016077 | 221.348426 |
| YOR354C | MSC6 | 0.98941624 | 0.99917121 | 0.00257135 | 141.6889729 | 225.3068627 |
| YOL163W | YOL163W | 0.98801006 | 0.99917121 | 0.00265154 | 141.8505877 | 225.5544363 |
| YDR159W | SAC3 | 0.97572046 | 0.99917121 | 0.00268515 | 5.592213852 | 8.577488451 |
| YDR125C | ECM18 | 0.97772718 | 0.99917121 | 0.00271681 | 144.5332099 | 229.8181881 |
| YJL159W | HSP150 | 0.98899439 | 0.99917121 | 0.00277872 | 138.532431 | 220.2552199 |
| YOL036W | YOL036W | 0.9879151 | 0.99917121 | 0.00282889 | 141.2564524 | 224.5867356 |
| YHR129C | ARP1 | 0.98883917 | 0.99917121 | 0.00289732 | 136.0203031 | 216.24054 |
| YCR102C | YCR102C | 0.98598115 | 0.99917121 | 0.00292604 | 152.2033663 | 242.0063566 |
| YOL106W | YOL106W | 0.98635794 | 0.99917121 | 0.00293975 | 146.1526375 | 232.3697265 |
| YJL197W | UBP12 | 0.97810092 | 0.99917121 | 0.00320928 | 158.7553217 | 252.4049215 |
| YNL283C | WSC2 | 0.98580042 | 0.99917121 | 0.00322419 | 136.8898612 | 217.5853444 |
| YKL147C | YKL147C | 0.98370207 | 0.99917121 | 0.00325192 | 144.8570435 | 230.2686127 |
| YNL332W | THI12 | 0.98269 | 0.99917121 | 0.00342149 | 142.8472506 | 227.0476201 |
| YAL030W | SNC1 | 0.98302028 | 0.99917121 | 0.00351145 | 144.7808452 | 230.1156386 |
| YOL017W | ESC8 | 0.98431518 | 0.99917121 | 0.00368263 | 143.5759743 | 228.1761774 |
| YJL058C | BIT61 | 0.9752051 | 0.99917121 | 0.00400537 | 147.1933108 | 233.896946 |
| YPL244C | HUT1 | 0.98032148 | 0.99917121 | 0.00416443 | 141.512281 | 224.8312908 |
| YHR160C | PEX18 | 0.97897299 | 0.99917121 | 0.00421358 | 145.1081468 | 230.5512241 |

| | | | | | | |
|---|---|---|---|---|---|---|
| YMR053C | STB2 | 0.98281105 | 0.99917121 | 0.0042382 | 132.6461723 | 210.7042308 |
| YGR161C | RTS3 | 0.97678284 | 0.99917121 | 0.00425462 | 143.232376 | 227.5593117 |
| YBR186W | PCH2 | 0.9790014 | 0.99917121 | 0.00425909 | 146.5214579 | 232.7961816 |
| YAL060W | BDH1 | 0.98276158 | 0.99917121 | 0.00426455 | 147.5916541 | 234.4996571 |
| YBR233W | PBP2 | 0.97238118 | 0.99917121 | 0.00437978 | 141.9839324 | 225.5560775 |
| YHL039W | YHL039W | 0.97797025 | 0.99917121 | 0.00439441 | 141.0051238 | 223.9956754 |
| YMR055C | BUB2 | 0.98178512 | 0.99917121 | 0.00441148 | 140.859193 | 223.7612191 |
| YGR132C | PHB1 | 0.97298978 | 0.99917121 | 0.00449963 | 141.5831884 | 224.9033369 |
| YDR250C | YDR250C | 0.97446495 | 0.99917121 | 0.00454815 | 154.3486481 | 245.2246727 |
| YNL187W | YNL187W | 0.97634733 | 0.99917121 | 0.00471836 | 135.6939054 | 215.498792 |
| YJR038C | YJR038C | 0.97293592 | 0.99917121 | 0.00472037 | 136.3334783 | 216.5169797 |
| YLR442C | SIR3 | 0.96117941 | 0.99917121 | 0.00479099 | 150.2559048 | 238.6779327 |
| YOL121C | RPS19A | 0.97641046 | 0.99917121 | 0.00482649 | 136.8149218 | 217.2706761 |
| YOR296W | YOR296W | 0.97740268 | 0.99917121 | 0.00484611 | 142.8161374 | 226.8244002 |
| YPL121C | MEI5 | 0.98374765 | 0.99917121 | 0.0048782 | 136.2424564 | 216.3527995 |
| YPL070W | MUK1 | 0.97277775 | 0.99917121 | 0.00500148 | 140.1783408 | 222.6051286 |
| YPL203W | TPK2 | 0.9719019 | 0.99917121 | 0.00501584 | 151.1115066 | 240.0129505 |
| YPL119C | DBP1 | 0.97638515 | 0.99917121 | 0.00505222 | 137.9604285 | 219.0672199 |
| YBR219C | YBR219C | 0.97747219 | 0.99917121 | 0.00505811 | 148.96031 | 236.5823104 |
| YOL028C | YAP7 | 0.97639017 | 0.99917121 | 0.00530667 | 145.8091566 | 231.5342277 |
| YOR215C | YOR215C | 0.98424652 | 0.99917121 | 0.00531177 | 123.2214333 | 195.5657414 |
| YOR385W | YOR385W | 0.97087746 | 0.99917121 | 0.00541028 | 147.7218219 | 234.567255 |
| YGL148W | ARO2 | 0.99010418 | 0.99917121 | 0.00554871 | 92.16631167 | 146.0858164 |
| YGL194C | HOS2 | 0.96281542 | 0.99917121 | 0.00569682 | 127.1842438 | 201.8290337 |
| YDR275W | BSC2 | 0.97356502 | 0.99917121 | 0.00573431 | 139.2344207 | 221.0127246 |
| YIR030C | DCG1 | 0.97798118 | 0.99917121 | 0.00577782 | 156.5521985 | 248.583616 |
| YCR014C | POL4 | 0.96258878 | 0.99917121 | 0.00588716 | 150.5742039 | 239.0511461 |
| YDR289C | RTT103 | 0.9787602 | 0.99917121 | 0.00589219 | 142.1537539 | 225.6421164 |
| YKL093W | MBR1 | 0.92915432 | 0.99917121 | 0.00598548 | 118.5585764 | 188.0586466 |
| YBR207W | FTH1 | 0.97100414 | 0.99917121 | 0.00618917 | 147.4582119 | 234.0525367 |
| YOR234C | RPL33B | 0.97543422 | 0.99917121 | 0.00625138 | 126.6726434 | 200.9467732 |
| YBR231C | SWC5 | 0.96768365 | 0.99917121 | 0.0064666 | 137.1027858 | 217.5291136 |
| YBR246W | YBR246W | 0.98102019 | 0.99917121 | 0.00653258 | 126.3696817 | 200.430067 |
| YJL145W | SFH5 | 0.96702467 | 0.99917121 | 0.00674642 | 142.9345918 | 226.7813542 |
| YNL275W | BOR1 | 0.9620086 | 0.99917121 | 0.00698981 | 132.1973216 | 209.6540459 |
| YML051W | GAL80 | 0.96259405 | 0.99917121 | 0.00707539 | 134.6723949 | 213.58483 |
| YCR106W | RDS1 | 0.9678113 | 0.99917121 | 0.0070944 | 144.3511234 | 228.9945652 |
| YAL053W | FLC2 | 0.97140436 | 0.99917121 | 0.00710455 | 142.5507988 | 226.1265567 |
| YNL279W | PRM1 | 0.97876055 | 0.99917121 | 0.00723337 | 131.1168769 | 207.9038929 |
| YKL068W | NUP100 | 0.96179981 | 0.99917121 | 0.0072367 | 122.7871127 | 194.6394761 |
| YJR047C | ANB1 | 0.96303473 | 0.99917121 | 0.00734688 | 146.6118827 | 232.5637356 |
| YBR248C | HIS7 | 0.9063031 | 0.99917121 | 0.00737528 | 123.023322 | 194.9987121 |
| YJR026W | YJR026W | 0.95961926 | 0.99917121 | 0.00749128 | 142.01725 | 225.2298064 |
| YLL040C | VPS13 | 0.96147489 | 0.99917121 | 0.00756525 | 128.462937 | 203.6373968 |
| YOR229W | WTM2 | 0.96268443 | 0.99917121 | 0.00761026 | 145.8572373 | 231.3299575 |
| YDR104C | SPO71 | 0.95424777 | 0.99917121 | 0.00766331 | 141.3856509 | 224.2030996 |
| YMR104C | YPK2 | 0.93263455 | 0.99917121 | 0.00767734 | 129.6468305 | 205.50892 |
| YDR099W | BMH2 | 0.95280549 | 0.99917121 | 0.00784172 | 140.5549755 | 222.8586124 |
| YER019C-A | SBH2 | 0.96548647 | 0.99917121 | 0.00791851 | 143.7489313 | 227.9351883 |
| YOR087W | YVC1 | 0.96940589 | 0.99917121 | 0.00831577 | 138.2269538 | 219.0937632 |
| YJL208C | NUC1 | 0.95890232 | 0.99917121 | 0.00832731 | 142.5848342 | 226.0316854 |
| YDR217C | RAD9 | 0.93126535 | 0.99917121 | 0.00839313 | 125.8973104 | 199.4510594 |
| YHR134W | WSS1 | 0.96384872 | 0.99917121 | 0.00844116 | 124.0425348 | 196.4917268 |
| YCL044C | MGR1 | 0.96962901 | 0.99917121 | 0.00859386 | 134.2856631 | 212.7838938 |
| YPL221W | FLC1 | 0.96255217 | 0.99917121 | 0.00863025 | 147.7267921 | 234.1826205 |
| YOL055C | THI20 | 0.95148618 | 0.99917121 | 0.00865427 | 139.8692137 | 221.6675711 |
| YOL070C | NBA1 | 0.96801891 | 0.99917121 | 0.00876122 | 137.919188 | 218.549384 |
| YMR165C | PAH1 | 0.98680768 | 0.99917121 | 0.00877168 | 40.08015012 | 62.75280377 |
| YOL053C-A | YOL053C-A | 0.95699595 | 0.99917121 | 0.00888245 | 140.2662928 | 222.272048 |
| YPL164C | MLH3 | 0.95076466 | 0.99917121 | 0.00902393 | 123.9101996 | 196.2099557 |
| YOR264W | DSE3 | 0.95245582 | 0.99917121 | 0.00909699 | 141.338687 | 223.9535346 |
| YPL230W | YPL230W | 0.9584299 | 0.99917121 | 0.0091537 | 134.2329217 | 212.6316602 |
| YHR114W | BZZ1 | 0.96772636 | 0.99917121 | 0.00916159 | 146.5715758 | 232.2783199 |
| YPL071C | YPL071C | 0.96401815 | 0.99917121 | 0.00950119 | 138.7668057 | 219.808889 |

| | | | | | | |
|---|---|---|---|---|---|---|
| YBR064W | YBR064W | 0.95633248 | 0.99917121 | 0.00954102 | 143.8921441 | 227.9654336 |
| YBR069C | TAT1 | 0.93663326 | 0.99917121 | 0.00963349 | 139.0449902 | 220.2357306 |
| YGR066C | YGR066C | 0.94393149 | 0.99917121 | 0.00973844 | 152.7350178 | 242.0224354 |
| YGR124W | ASN2 | 0.95588366 | 0.99917121 | 0.0097667 | 146.3930475 | 231.9202682 |
| YGR153W | YGR153W | 0.96598175 | 0.99917121 | 0.00988437 | 139.7822023 | 221.3790547 |
| YNL254C | RTC4 | 0.95420541 | 0.99917121 | 0.01026082 | 134.4058522 | 212.7720592 |
| YHR127W | YHR127W | 0.93444984 | 0.99917121 | 0.01027311 | 141.9586644 | 224.7973826 |
| YGR200C | ELP2 | 0.95999384 | 0.99917121 | 0.01031201 | 119.4550443 | 188.9586984 |
| YKR070W | YKR070W | 0.95418183 | 0.99917121 | 0.01031987 | 140.8300302 | 222.9944855 |
| YPL023C | MET12 | 0.9493105 | 0.99917121 | 0.01079952 | 142.3348598 | 225.3322475 |
| YNR018W | YNR018W | 0.95568373 | 0.99917121 | 0.01099649 | 133.1971247 | 210.7576397 |
| YKR082W | NUP133 | 0.90266294 | 0.99917121 | 0.01110701 | 90.32185504 | 142.4711549 |
| YNL328C | MDJ2 | 0.9540367 | 0.99917121 | 0.01120329 | 135.4372323 | 214.2994933 |
| YML095C | RAD10 | 0.94699115 | 0.99917121 | 0.0112648 | 142.1740393 | 225.0194402 |
| YBR221C | PDB1 | 0.94904073 | 0.99917121 | 0.01128699 | 143.2570962 | 226.741355 |
| YKL087C | CYT2 | 0.93570349 | 0.99917121 | 0.01152429 | 110.7894806 | 175.0121817 |
| YPL202C | AFT2 | 0.94346705 | 0.99917121 | 0.011739 | 141.537105 | 223.9473987 |
| YJL048C | UBX6 | 0.92674275 | 0.99917121 | 0.01176431 | 144.5476864 | 228.7382535 |
| YPL212C | PUS1 | 0.94774561 | 0.99917121 | 0.0118501 | 143.853558 | 227.6224899 |
| YLR329W | REC102 | 0.95127269 | 0.99917121 | 0.01185538 | 144.0194913 | 227.8860725 |
| YIL155C | GUT2 | 0.95724106 | 0.99917121 | 0.01203096 | 136.5688709 | 216.0005713 |
| YLR332W | MID2 | 0.95876777 | 0.99917121 | 0.01208636 | 137.3576804 | 217.2498896 |
| YOL126C | MDH2 | 0.94030226 | 0.99917121 | 0.01211831 | 146.0975989 | 231.163121 |
| YLR365W | YLR365W | 0.94135248 | 0.99917121 | 0.01225771 | 152.1446606 | 240.775246 |
| YDR241W | BUD26 | 0.95994073 | 0.99917121 | 0.01229526 | 107.8901007 | 170.3013262 |
| YGR269W | YGR269W | 0.94212673 | 0.99917121 | 0.01232237 | 136.4314427 | 215.7462096 |
| YMR127C | SAS2 | 0.93273033 | 0.99917121 | 0.01250165 | 138.8103001 | 219.5123593 |
| YOR382W | FIT2 | 0.84349621 | 0.99917121 | 0.0125652 | 127.9563205 | 202.2211315 |
| YNL142W | MEP2 | 0.94340056 | 0.99917121 | 0.01258293 | 124.8437066 | 197.2625581 |
| YJR032W | CPR7 | 0.93442244 | 0.99917121 | 0.01260069 | 105.5780755 | 166.5825059 |
| YDR533C | HSP31 | 0.94843707 | 0.99917121 | 0.01262976 | 144.9087856 | 229.2077469 |
| YAL008W | FUN14 | 0.93390206 | 0.99917121 | 0.01280338 | 146.0391344 | 230.9865068 |
| YGL177W | YGL177W | 0.92195795 | 0.99917121 | 0.01299564 | 145.3011287 | 229.7878945 |
| YNL323W | LEM3 | 0.93971328 | 0.99917121 | 0.01301908 | 136.0511662 | 215.055735 |
| YLR133W | CKI1 | 0.94214462 | 0.99917121 | 0.01330772 | 140.0417851 | 221.3750619 |
| YBR238C | YBR238C | 0.92341021 | 0.99917121 | 0.013383 | 146.327269 | 231.3746597 |
| YDL091C | UBX3 | 0.91794291 | 0.99917121 | 0.01341419 | 153.2073844 | 242.3265017 |
| YDR029W | YDR029W | 0.95462576 | 0.99917121 | 0.01344748 | 138.9133771 | 219.5611884 |
| YJL155C | FBP26 | 0.92352009 | 0.99917121 | 0.01349613 | 146.4381279 | 231.5373956 |
| YJR021C | REC107 | 0.93804132 | 0.99917121 | 0.01352467 | 143.7197542 | 227.2052778 |
| YDR223W | CRF1 | 0.93709287 | 0.99917121 | 0.0136716 | 149.9246905 | 237.0678785 |
| YOR015W | YOR015W | 0.94434846 | 0.99917121 | 0.01373161 | 141.4975782 | 223.6415377 |
| YAR037W | YAR037W | 0.9344463 | 0.99917121 | 0.01383417 | 151.2318763 | 239.129574 |
| YPL073C | YPL073C | 0.94124095 | 0.99917121 | 0.01393867 | 145.4801097 | 229.9579324 |
| YBL064C | PRX1 | 0.93363154 | 0.99917121 | 0.01396011 | 148.6146392 | 234.9466278 |
| YMR225C | MRPL44 | 0.92460051 | 0.99917121 | 0.01411142 | 136.3493405 | 215.3973693 |
| YJR015W | YJR015W | 0.92081211 | 0.99917121 | 0.01423764 | 140.915353 | 222.6527332 |
| YPL014W | YPL014W | 0.92093976 | 0.99917121 | 0.01430936 | 134.8229731 | 212.9427064 |
| YJR025C | BNA1 | 0.93050786 | 0.99917121 | 0.01443329 | 130.5007541 | 206.045054 |
| YBL049W | MOH1 | 0.93515341 | 0.99917121 | 0.01443788 | 152.2899581 | 240.7408269 |
| YGR176W | YGR176W | 0.89437993 | 0.99917121 | 0.01449791 | 131.4300647 | 207.516977 |
| YAR042W | SWH1 | 0.93295843 | 0.99917121 | 0.01456752 | 139.7182425 | 220.7062817 |
| YHL020C | OPI1 | 0.96380083 | 0.99917121 | 0.01459699 | 37.43206075 | 57.82591536 |
| YBR119W | MUD1 | 0.93990701 | 0.99917121 | 0.01469839 | 153.1761604 | 242.1202234 |
| YER130C | YER130C | 0.92939465 | 0.99917121 | 0.01493429 | 150.5166368 | 237.8565366 |
| YJL133W | MRS3 | 0.89508277 | 0.99917121 | 0.01495574 | 151.8313898 | 239.947486 |
| YHR041C | SRB2 | 0.95221971 | 0.99917121 | 0.01499372 | 98.16992392 | 154.4943007 |
| YIR042C | YIR042C | 0.92942772 | 0.99917121 | 0.01513876 | 142.9902004 | 225.8467877 |
| YGR122W | YGR122W | 0.95345111 | 0.99917121 | 0.01517403 | 124.8175858 | 196.9050813 |
| YJL177W | RPL17B | 0.93373661 | 0.99917121 | 0.01518252 | 142.118285 | 224.4530465 |
| YLR014C | PPR1 | 0.93787304 | 0.99917121 | 0.01551098 | 116.5801351 | 183.7469884 |
| YJR043C | POL32 | 0.95443333 | 0.99917121 | 0.01559909 | 114.115704 | 179.8119771 |
| YGL158W | RCK1 | 0.9159427 | 0.99917121 | 0.01586105 | 142.7884361 | 225.4374501 |
| YJR152W | DAL5 | 0.91576368 | 0.99917121 | 0.01604118 | 113.5259333 | 178.8189522 |

| | | | | | | |
|---|---|---|---|---|---|---|
| YBR210W | ERV15 | 0.92739301 | 0.99917121 | 0.01622406 | 155.0956091 | 244.9906884 |
| YOR223W | YOR223W | 0.92032863 | 0.99917121 | 0.01623863 | 136.3226618 | 215.0955573 |
| YER121W | YER121W | 0.93564644 | 0.99917121 | 0.01624467 | 139.7256592 | 220.5136299 |
| YBR014C | YBR014C | 0.9265232 | 0.99917121 | 0.01632676 | 151.8721282 | 239.8452143 |
| YKL081W | TEF4 | 0.95183004 | 0.99917121 | 0.01637613 | 110.8010482 | 174.4391112 |
| YCL048W | SPS22 | 0.92092669 | 0.99917121 | 0.01648624 | 156.2812639 | 246.8467183 |
| YDR504C | SPG3 | 0.92273318 | 0.99917121 | 0.01657449 | 142.0184291 | 224.1243437 |
| YOR383C | FIT3 | 0.9256826 | 0.99917121 | 0.01660384 | 142.3423027 | 224.6364905 |
| YLR084C | RAX2 | 0.93385621 | 0.99917121 | 0.01666066 | 150.9194068 | 238.2874301 |
| YCL036W | GFD2 | 0.89681576 | 0.99917121 | 0.01676577 | 139.7596891 | 220.5042897 |
| YIL162W | SUC2 | 0.90690233 | 0.99917121 | 0.01681042 | 142.7905144 | 225.3250223 |
| YPL140C | MKK2 | 0.92186997 | 0.99917121 | 0.01685332 | 141.326876 | 222.9891473 |
| YIL173W | VTH1 | 0.93363592 | 0.99917121 | 0.01688996 | 139.5225881 | 220.1115988 |
| YER045C | ACA1 | 0.92183625 | 0.99917121 | 0.01710273 | 139.2114717 | 219.5902496 |
| YIL001W | YIL001W | 0.91844657 | 0.99917121 | 0.01716555 | 145.6143117 | 229.7782389 |
| YJR052W | RAD7 | 0.92666745 | 0.99917121 | 0.01747787 | 141.7898192 | 223.6501816 |
| YNL327W | EGT2 | 0.9262116 | 0.99917121 | 0.01751529 | 142.5996968 | 224.9352401 |
| YIR029W | DAL2 | 0.90602935 | 0.99917121 | 0.01780473 | 144.0267023 | 227.1722652 |
| YOL060C | MAM3 | 0.88481122 | 0.99917121 | 0.01786601 | 131.7226692 | 207.5723022 |
| YDR117C | TMA64 | 0.90833658 | 0.99917121 | 0.01797672 | 151.9181437 | 239.71734 |
| YAL061W | YAL061W | 0.91069184 | 0.99917121 | 0.01807845 | 149.6141251 | 236.0361029 |
| YGL160W | YGL160W | 0.92008622 | 0.99917121 | 0.01825293 | 137.689546 | 217.026569 |
| YGR187C | HGH1 | 0.91784606 | 0.99917121 | 0.01832168 | 148.0844089 | 233.5705871 |
| YIR004W | DJP1 | 0.94598861 | 0.99917121 | 0.01848399 | 123.785236 | 194.8576862 |
| YBR166C | TYR1 | 0.906197 | 0.99917121 | 0.01862647 | 150.7819461 | 237.8288897 |
| YJL211C | YJL211C | 0.92418622 | 0.99917121 | 0.018736 | 139.2948103 | 219.5238421 |
| YNL035C | YNL035C | 0.91732701 | 0.99917121 | 0.01879739 | 133.2208606 | 209.8444217 |
| YKR067W | GPT2 | 0.90866753 | 0.99917121 | 0.01915027 | 145.2703878 | 228.9886292 |
| YDR184C | ATC1 | 0.87827474 | 0.99917121 | 0.01927405 | 131.7516291 | 207.4467618 |
| YMR173W-A | YMR173W-A | 0.93203986 | 0.99917121 | 0.01943804 | 134.8963585 | 212.4343216 |
| YOR035C | SHE4 | 0.90647626 | 0.99917121 | 0.01945127 | 95.67488585 | 149.977871 |
| YCR075C | ERS1 | 0.90053541 | 0.99917121 | 0.01945472 | 140.2211942 | 220.9113618 |
| YDL113C | ATG20 | 0.91713883 | 0.99917121 | 0.01967072 | 142.8435255 | 225.0607332 |
| YAR044W | OSH1 | 0.89137905 | 0.99917121 | 0.0198064 | 142.514811 | 224.5207596 |
| YOR037W | CYC2 | 0.92287671 | 0.99917121 | 0.01999123 | 130.8832437 | 205.9765445 |
| YGR067C | YGR067C | 0.91582709 | 0.99917121 | 0.02000006 | 124.8463835 | 196.3625935 |
| YDR110W | FOB1 | 0.85664259 | 0.99917121 | 0.02001027 | 143.7808375 | 226.5118806 |
| YOR366W | YOR366W | 0.89622075 | 0.99917121 | 0.02018717 | 144.2304053 | 227.2061895 |
| YGR170W | PSD2 | 0.91810935 | 0.99917121 | 0.02021476 | 140.2889122 | 220.9265359 |
| YPL246C | RBD2 | 0.91993229 | 0.99917121 | 0.02021832 | 135.4042186 | 213.1478946 |
| YKR090W | PXL1 | 0.88720825 | 0.99917121 | 0.02032399 | 145.9267662 | 229.8907321 |
| YGL118C | YGL118C | 0.8946856 | 0.99917121 | 0.02033495 | 143.0477513 | 225.3049582 |
| YNL193W | YNL193W | 0.8737742 | 0.99917121 | 0.02039842 | 127.1413115 | 199.9683884 |
| YNL233W | BNI4 | 0.92021192 | 0.99917121 | 0.02098496 | 134.8180558 | 212.1210491 |
| YGR101W | PCP1 | 0.9787013 | 0.99917121 | 0.02099901 | 41.64318945 | 63.75108729 |
| YJL030W | MAD2 | 0.92080918 | 0.99917121 | 0.02109349 | 141.3818216 | 222.5597188 |
| YER137C | YER137C | 0.91303604 | 0.99917121 | 0.02126524 | 136.3756027 | 214.5670608 |
| YOR221C | MCT1 | 0.90218892 | 0.99917121 | 0.02128101 | 112.5445812 | 176.6174915 |
| YNL204C | SPS18 | 0.87556905 | 0.99917121 | 0.02160267 | 131.5877343 | 206.9018976 |
| YOR108W | LEU9 | 0.92155214 | 0.99917121 | 0.02169404 | 139.0518028 | 218.7762683 |
| YOR300W | YOR300W | 0.87991819 | 0.99917121 | 0.02172754 | 134.8199159 | 212.0334832 |
| YOR233W | KIN4 | 0.9038636 | 0.99917121 | 0.02190646 | 141.0325998 | 221.9045214 |
| YMR056C | AAC1 | 0.90047631 | 0.99917121 | 0.02217421 | 145.5525474 | 229.0692792 |
| YBR176W | ECM31 | 0.90127052 | 0.99917121 | 0.02242638 | 139.0988202 | 218.7618569 |
| YOL053W | YOL053W | 0.89148057 | 0.99917121 | 0.02257369 | 144.0369461 | 226.6071895 |
| YKR080W | MTD1 | 0.86985649 | 0.99917121 | 0.02277418 | 139.6933969 | 219.6662385 |
| YCR017C | CWH43 | 0.87379232 | 0.99917121 | 0.02284673 | 150.533891 | 236.9194 |
| YGR038W | ORM1 | 0.94530265 | 0.99917121 | 0.02288977 | 132.2401848 | 207.7839251 |
| YBR250W | YBR250W | 0.89564761 | 0.99917121 | 0.02308824 | 149.7774288 | 235.6853947 |
| YJR079W | YJR079W | 0.8821437 | 0.99917121 | 0.02348649 | 146.7878512 | 230.8763507 |
| YER139C | YER139C | 0.9707974 | 0.99917121 | 0.02352181 | 96.95923504 | 151.526779 |
| YGL198W | YIP4 | 0.92060974 | 0.99917121 | 0.02355651 | 138.8931627 | 218.2966013 |
| YBL071C | YBL071C | 0.84307693 | 0.99917121 | 0.02367921 | 130.4878785 | 204.8973752 |
| YGL021W | ALK1 | 0.88870533 | 0.99917121 | 0.02369192 | 143.3299275 | 225.3450343 |

| | | | | | | |
|---|---|---|---|---|---|---|
| YPL152W | RRD2 | 0.96610046 | 0.99917121 | 0.02377244 | 85.52849336 | 133.2943295 |
| YPR117W | YPR117W | 0.92075194 | 0.99917121 | 0.02394891 | 131.7461156 | 206.8680663 |
| YDR488C | PAC11 | 0.91019083 | 0.99917121 | 0.02410622 | 139.6060844 | 219.364816 |
| YCR073C | SSK22 | 0.8539998 | 0.99917121 | 0.02416675 | 145.9401026 | 229.4434948 |
| YOR085W | OST3 | 0.91395221 | 0.99917121 | 0.02425988 | 123.2526503 | 193.3054734 |
| YDR248C | YDR248C | 0.87320198 | 0.99917121 | 0.02426886 | 159.5638011 | 251.1249258 |
| YPL222W | FMP40 | 0.89665085 | 0.99917121 | 0.0243508 | 137.8106358 | 216.4759924 |
| YMR086C-A | YMR086C-A | 0.77525595 | 0.99917121 | 0.02441295 | 134.4554175 | 211.1256888 |
| YDL144C | YDL144C | 0.85779511 | 0.99917121 | 0.02472941 | 145.2440422 | 228.2665205 |
| YGR019W | UGA1 | 0.8780782 | 0.99917121 | 0.02474464 | 155.3895192 | 244.4199504 |
| YGL258W | VEL1 | 0.88009727 | 0.99917121 | 0.02482394 | 142.7131514 | 224.2248985 |
| YPL048W | CAM1 | 0.86490339 | 0.99917121 | 0.02490882 | 133.8138375 | 210.0436085 |
| YPL034W | YPL034W | 0.84338252 | 0.99917121 | 0.02497024 | 143.4694628 | 225.4113847 |
| YDR480W | DIG2 | 0.86587337 | 0.99917121 | 0.02502629 | 129.1505671 | 202.6036658 |
| YFR043C | IRC6 | 0.88581418 | 0.99917121 | 0.02506531 | 144.7208897 | 227.3925217 |
| YNL324W | YNL324W | 0.91692166 | 0.99917121 | 0.02513327 | 146.3800498 | 230.0262228 |
| YJL131C | YJL131C | 0.91698297 | 0.99917121 | 0.02514022 | 149.7623386 | 235.4112084 |
| YGL159W | YGL159W | 0.89158007 | 0.99917121 | 0.02518058 | 138.3186577 | 217.1837882 |
| YMR191W | SPG5 | 0.88414434 | 0.99917121 | 0.02522075 | 144.6457107 | 227.2538597 |
| YAL037W | YAL037W | 0.87590501 | 0.99917121 | 0.02522774 | 150.6633534 | 236.8352809 |
| YOR006C | YOR006C | 0.9058641 | 0.99917121 | 0.02533246 | 126.9749376 | 199.1019484 |
| YBR149W | ARA1 | 0.7891319 | 0.99917121 | 0.02534821 | 123.6290449 | 193.7721508 |
| YKL197C | PEX1 | 0.8757478 | 0.99917121 | 0.02541699 | 145.373614 | 228.3890226 |
| YPL058C | PDR12 | 0.8749264 | 0.99917121 | 0.02542776 | 135.0601371 | 211.9649064 |
| YPL184C | MRN1 | 0.87671013 | 0.99917121 | 0.02543013 | 140.8126825 | 221.1247601 |
| YMR279C | YMR279C | 0.89676686 | 0.99917121 | 0.02545427 | 135.0392514 | 211.9284159 |
| YPL079W | RPL21B | 0.91338299 | 0.99917121 | 0.02548172 | 123.9404062 | 194.2516753 |
| YFL019C | YFL019C | 0.88100118 | 0.99917121 | 0.02550429 | 139.9540009 | 219.7483855 |
| YER064C | YER064C | 0.90211106 | 0.99917121 | 0.02553672 | 138.0459737 | 216.7061596 |
| YPR125W | YLH47 | 0.89023337 | 0.99917121 | 0.02576496 | 138.4293602 | 217.2888251 |
| YOR304C-A | YOR304C-A | 0.87824401 | 0.99917121 | 0.0258136 | 142.857489 | 224.3340862 |
| YLR058C | SHM2 | 0.8919398 | 0.99917121 | 0.02587044 | 137.5915695 | 215.9418993 |
| YMR031C | YMR031C | 0.7994038 | 0.99917121 | 0.02592049 | 136.8998032 | 214.834254 |
| YJL067W | YJL067W | 0.87663134 | 0.99917121 | 0.02593366 | 141.3611039 | 221.9366598 |
| YBR074W | YBR074W | 0.87429647 | 0.99917121 | 0.0261413 | 157.4555409 | 247.5395398 |
| YNL169C | PSD1 | 0.96360497 | 0.99917121 | 0.02614218 | 91.98982732 | 143.2942251 |
| YBR262C | FMP51 | 0.89120724 | 0.99917121 | 0.02633931 | 148.0481596 | 232.5354302 |
| YBR084W | MIS1 | 0.86659288 | 0.99917121 | 0.02643704 | 150.4038156 | 236.2745768 |
| YJL088W | ARG3 | 0.89938937 | 0.99917121 | 0.02646273 | 135.9210117 | 213.209557 |
| YNL274C | YNL274C | 0.8890292 | 0.99917121 | 0.02647648 | 142.2317983 | 223.2569466 |
| YCL033C | YCL033C | 0.930019 | 0.99917121 | 0.02654058 | 148.4944969 | 233.2216243 |
| YER075C | PTP3 | 0.90939191 | 0.99917121 | 0.02655215 | 132.7320053 | 208.120599 |
| YDR338C | YDR338C | 0.84697334 | 0.99917121 | 0.02658714 | 147.7774413 | 232.0741353 |
| YOR005C | DNL4 | 0.91113065 | 0.99917121 | 0.02693294 | 139.031282 | 218.1049138 |
| YEL025C | YEL025C | 0.8639364 | 0.99917121 | 0.0269572 | 141.2681938 | 221.6639329 |
| YJR127C | RSF2 | 0.83313124 | 0.99917121 | 0.02709874 | 135.2096805 | 211.9993226 |
| YNL302C | RPS19B | 0.91692669 | 0.99917121 | 0.02712743 | 89.06370549 | 138.5146624 |
| YBR199W | KTR4 | 0.86902826 | 0.99917121 | 0.02721464 | 138.3615467 | 217.0041097 |
| YDL038C | YDL038C | 0.86219565 | 0.99917121 | 0.0272301 | 159.7118143 | 250.9996105 |
| YPL167C | REV3 | 0.83372138 | 0.99917121 | 0.02728645 | 136.8870511 | 214.6474229 |
| YMR262W | YMR262W | 0.88186281 | 0.99917121 | 0.0274047 | 124.9825345 | 195.6766906 |
| YJR031C | GEA1 | 0.86325993 | 0.99917121 | 0.02772557 | 143.6122313 | 225.3028205 |
| YPL114W | YPL114W | 0.85375803 | 0.99917121 | 0.0279737 | 145.4318338 | 228.1700393 |
| YDL162C | YDL162C | 0.87497018 | 0.99917121 | 0.028124 | 148.6596785 | 233.2916179 |
| YHR202W | YHR202W | 0.84948181 | 0.99917121 | 0.02821603 | 147.7157461 | 231.7773143 |
| YLR207W | HRD3 | 0.89043992 | 0.99917121 | 0.02831175 | 138.0418019 | 216.36121 |
| YER073W | ALD5 | 0.8779851 | 0.99917121 | 0.02842615 | 141.5361877 | 221.9115963 |
| YLL024C | SSA2 | 0.86205406 | 0.99917121 | 0.02845263 | 137.4054653 | 215.3307566 |
| YLR392C | YLR392C | 0.87516803 | 0.99917121 | 0.02849342 | 146.1070046 | 229.1817973 |
| YDR304C | CPR5 | 0.8390616 | 0.99917121 | 0.02855612 | 131.1619213 | 205.3761486 |
| YDL018C | ERP3 | 0.91180325 | 0.99917121 | 0.02866765 | 135.9949889 | 213.0585523 |
| YKL106W | AAT1 | 0.8104374 | 0.99917121 | 0.02867542 | 135.7945847 | 212.7384894 |
| YKR018C | YKR018C | 0.74861222 | 0.99917121 | 0.02885646 | 135.6043605 | 212.413512 |
| YPL026C | SKS1 | 0.85996835 | 0.99917121 | 0.02891944 | 140.5596778 | 220.2965005 |

| | | | | | | |
|---|---|---|---|---|---|---|
| YLR214W | FRE1 | 0.89763387 | 0.99917121 | 0.02918892 | 107.7220603 | 167.974227 |
| YOR226C | ISU2 | 0.84381054 | 0.99917121 | 0.02937767 | 140.2289045 | 219.7139254 |
| YNL008C | ASI3 | 0.87525405 | 0.99917121 | 0.02947321 | 136.0469413 | 213.0430738 |
| YBR215W | HPC2 | 0.87831964 | 0.99917121 | 0.02948863 | 150.5775596 | 236.179218 |
| YFL052W | YFL052W | 0.87015734 | 0.99917121 | 0.02988469 | 143.7562113 | 225.2688695 |
| YBR108W | YBR108W | 0.8707004 | 0.99917121 | 0.02995739 | 144.8483583 | 226.9991004 |
| YPL176C | TRE1 | 0.8831596 | 0.99917121 | 0.02997446 | 133.7987924 | 209.4020949 |
| YMR119W-A | YMR119W-A | 0.80046126 | 0.99917121 | 0.03002525 | 138.3842229 | 216.697575 |
| YKR048C | NAP1 | 0.84390315 | 0.99917121 | 0.03012568 | 134.3336001 | 210.235268 |
| YHR079C | IRE1 | 0.782547 | 0.99917121 | 0.03024417 | 162.9913684 | 255.8544048 |
| YPR039W | YPR039W | 0.8105341 | 0.99917121 | 0.03029035 | 138.679048 | 217.1347256 |
| YJL124C | LSM1 | 0.86309586 | 0.99917121 | 0.03038516 | 102.2392348 | 159.0977413 |
| YJL142C | YJL142C | 0.87944551 | 0.99917121 | 0.03069643 | 139.2354637 | 217.9712354 |
| YFR045W | YFR045W | 0.85441536 | 0.99917121 | 0.03075913 | 147.693145 | 231.4312946 |
| YJL012C | VTC4 | 0.84986777 | 0.99917121 | 0.0308479 | 144.9661152 | 227.07805 |
| YGR263C | SAY1 | 0.85710964 | 0.99917121 | 0.03118043 | 138.1560776 | 216.1934559 |
| YGR295C | COS6 | 0.83413778 | 0.99917121 | 0.03156057 | 140.9003491 | 220.5169905 |
| YBL072C | RPS8A | 0.88481628 | 0.99917121 | 0.03180124 | 127.6726662 | 199.424372 |
| YHR179W | OYE2 | 0.83294456 | 0.99917121 | 0.03207967 | 144.1034058 | 225.5541375 |
| YOR237W | HES1 | 0.8489579 | 0.99917121 | 0.03218356 | 146.5335905 | 229.4112087 |
| YAL068C | PAU8 | 0.79705957 | 0.99917121 | 0.03221277 | 142.3867878 | 222.8044314 |
| YFL011W | HXT10 | 0.84756876 | 0.99917121 | 0.03241671 | 148.2079586 | 232.0489887 |
| YDL180W | YDL180W | 0.87442718 | 0.99917121 | 0.03254951 | 150.6061691 | 235.8516211 |
| YGR142W | BTN2 | 0.81306887 | 0.99917121 | 0.03259716 | 149.4921758 | 234.0719298 |
| YER143W | DDI1 | 0.86377005 | 0.99917121 | 0.03263725 | 143.4216795 | 224.4006057 |
| YOR105W | YOR105W | 0.87090236 | 0.99917121 | 0.03269346 | 138.9727721 | 217.309476 |
| YIL157C | FMP35 | 0.87136587 | 0.99917121 | 0.03278387 | 146.7984352 | 229.7597541 |
| YGR210C | YGR210C | 0.70184756 | 0.99917121 | 0.03278508 | 132.6387459 | 207.2122348 |
| YOL044W | PEX15 | 0.87119597 | 0.99917121 | 0.0328854 | 136.1044306 | 212.7186345 |
| YJL183W | MNN11 | 0.7143557 | 0.99917121 | 0.0329704 | 128.8112241 | 201.0948369 |
| YML104C | MDM1 | 0.83486373 | 0.99917121 | 0.03300481 | 142.7689846 | 223.3164696 |
| YNR024W | YNR024W | 0.86039474 | 0.99917121 | 0.0330913 | 149.1093808 | 233.4021401 |
| YOR135C | YOR135C | 0.87286255 | 0.99917121 | 0.03312707 | 133.7249689 | 208.900205 |
| YAL034C | FUN19 | 0.82093594 | 0.99917121 | 0.03318019 | 154.3358831 | 241.7137945 |
| YOR049C | RSB1 | 0.8539705 | 0.99917121 | 0.0332238 | 134.8973603 | 210.7552848 |
| YDL167C | NRP1 | 0.83564731 | 0.99917121 | 0.03322492 | 154.1860184 | 241.4697018 |
| YOR334W | MRS2 | 0.90204163 | 0.99917121 | 0.03322673 | 123.1927393 | 192.1169182 |
| YIR013C | GAT4 | 0.86035781 | 0.99917121 | 0.03334468 | 141.1874324 | 220.7566291 |
| YLR096W | KIN2 | 0.85827657 | 0.99917121 | 0.03338296 | 137.8102723 | 215.3742963 |
| YHR006W | STP2 | 0.83838514 | 0.99917121 | 0.03342209 | 137.3476312 | 214.6328336 |
| YHR194W | MDM31 | 0.92865587 | 0.99917121 | 0.03348132 | 133.771774 | 208.9315484 |
| YJL201W | ECM25 | 0.79833721 | 0.99917121 | 0.03357758 | 158.0873421 | 247.6390341 |
| YLR063W | YLR063W | 0.8651638 | 0.99917121 | 0.03360702 | 143.2194171 | 223.9603057 |
| YLL063C | AYT1 | 0.85572226 | 0.99917121 | 0.03366289 | 141.6131354 | 221.3957106 |
| YJL149W | YJL149W | 0.84941742 | 0.99917121 | 0.03366648 | 142.8998532 | 223.4441951 |
| YCR059C | YIH1 | 0.85383164 | 0.99917121 | 0.03374179 | 146.5500755 | 229.2474945 |
| YGR034W | RPL26B | 0.87120495 | 0.99917121 | 0.03375667 | 117.7422067 | 183.3730834 |
| YIR014W | YIR014W | 0.88030562 | 0.99917121 | 0.03383125 | 145.3567559 | 227.3363895 |
| YGR007W | MUQ1 | 0.78765667 | 0.99917121 | 0.03399047 | 138.1495619 | 215.840507 |
| YBR188C | NTC20 | 0.82278557 | 0.99917121 | 0.03400001 | 145.6077543 | 227.7154964 |
| YIL024C | YIL024C | 0.87081889 | 0.99917121 | 0.0340163 | 145.6791691 | 227.8272291 |
| YHR057C | CPR2 | 0.81253231 | 0.99917121 | 0.03412245 | 150.3874736 | 235.3116197 |
| YLR345W | YLR345W | 0.85753174 | 0.99917121 | 0.03416533 | 136.6437436 | 213.4213794 |
| YJL181W | YJL181W | 0.88340269 | 0.99917121 | 0.03457166 | 142.59737 | 222.8521808 |
| YDL034W | YDL034W | 0.69999774 | 0.99917121 | 0.03465658 | 143.2262102 | 223.8431705 |
| YPL174C | NIP100 | 0.83919694 | 0.99917121 | 0.03468041 | 125.7011059 | 195.93393 |
| YPL019C | VTC3 | 0.79702083 | 0.99917121 | 0.03479968 | 145.8547039 | 228.0112417 |
| YGR026W | YGR026W | 0.8111151 | 0.99917121 | 0.0348721 | 150.1315899 | 234.8127697 |
| YNL314W | DAL82 | 0.90234897 | 0.99917121 | 0.03493619 | 142.6154918 | 222.8365968 |
| YPL207W | TYW1 | 0.76575305 | 0.99917121 | 0.03502171 | 140.5503533 | 219.5377203 |
| YDL023C | SRF4 | 0.81057229 | 0.99917121 | 0.03512396 | 147.1505321 | 230.0351383 |
| YDR316W | OMS1 | 0.81753161 | 0.99917121 | 0.03534212 | 137.0344798 | 213.9001105 |
| YML055W | SPC2 | 0.52664922 | 0.99917121 | 0.03535232 | 118.2502214 | 183.9875009 |
| YPL064C | CWC27 | 0.87620144 | 0.99917121 | 0.03546226 | 133.280734 | 207.9081371 |

| | | | | | | |
|---|---|---|---|---|---|---|
| YBR204C | YBR204C | 0.83149205 | 0.99917121 | 0.03565131 | 146.1505202 | 228.3784659 |
| YER101C | AST2 | 0.87359877 | 0.99917121 | 0.03567284 | 132.3122607 | 206.3403027 |
| YKL218C | SRY1 | 0.86325187 | 0.99917121 | 0.03570396 | 136.0653745 | 212.3128309 |
| YOR166C | SWT1 | 0.86427019 | 0.99917121 | 0.03570413 | 143.1370209 | 223.5734408 |
| YML099C | ARG81 | 0.86279821 | 0.99917121 | 0.03589661 | 139.9458448 | 218.4684628 |
| YOR038C | HIR2 | 0.889785 | 0.99917121 | 0.03594514 | 147.1059145 | 229.86398 |
| YDR284C | DPP1 | 0.71861569 | 0.99917121 | 0.03596851 | 139.1680515 | 217.221168 |
| YNL034W | YNL034W | 0.77551008 | 0.99917121 | 0.0362769 | 116.4377988 | 180.9887494 |
| YML075C | HMG1 | 0.73102887 | 0.99917121 | 0.03630278 | 134.2434538 | 209.3386682 |
| YPL074W | YTA6 | 0.82798029 | 0.99917121 | 0.0363406 | 146.1924945 | 228.361273 |
| YHR139C | SPS100 | 0.81859485 | 0.99917121 | 0.03644627 | 139.3525244 | 217.4566718 |
| YCR087W | YCR087W | 0.85138257 | 0.99917121 | 0.03650455 | 132.2012382 | 206.0621203 |
| YOR266W | PNT1 | 0.84156128 | 0.99917121 | 0.03651427 | 141.7308365 | 221.2355191 |
| YOR070C | GYP1 | 0.81127676 | 0.99917121 | 0.03660153 | 124.0264996 | 193.0331436 |
| YOR041C | SRF5 | 0.85746213 | 0.99917121 | 0.03663761 | 138.8254216 | 216.5940068 |
| YJR097W | JJJ3 | 0.84510159 | 0.99917121 | 0.03668672 | 137.8878555 | 215.0950732 |
| YML123C | PHO84 | 0.92978108 | 0.99917121 | 0.03673037 | 140.8926807 | 219.874526 |
| YDR049W | YDR049W | 0.86468435 | 0.99917121 | 0.03678035 | 116.7580976 | 181.4374054 |
| YPL245W | YPL245W | 0.80241644 | 0.99917121 | 0.03679408 | 143.3048147 | 223.7077532 |
| YPL155C | KIP2 | 0.78470427 | 0.99917121 | 0.03681501 | 148.9223361 | 232.6503363 |
| YOR142W | LSC1 | 0.88993976 | 0.99917121 | 0.03683773 | 127.9712779 | 199.2858679 |
| YDL169C | UGX2 | 0.79786334 | 0.99917121 | 0.03692782 | 153.203455 | 239.4536807 |
| YDR379W | RGA2 | 0.80870643 | 0.99917121 | 0.03696039 | 144.2101993 | 225.1291789 |
| YDR314C | RAD34 | 0.74231743 | 0.99917121 | 0.03696792 | 144.5493592 | 225.6683271 |
| YGR136W | LSB1 | 0.82131727 | 0.99917121 | 0.03718742 | 145.3326038 | 226.8887777 |
| YIL067C | YIL067C | 0.85141111 | 0.99917121 | 0.03726882 | 135.7373176 | 211.5996716 |
| YBL083C | YBL083C | 0.78219807 | 0.99917121 | 0.03728077 | 143.7860077 | 224.4146548 |
| YDR218C | SPR28 | 0.75405379 | 0.99917121 | 0.03733411 | 151.6984524 | 237.0076389 |
| YOR097C | YOR097C | 0.86853315 | 0.99917121 | 0.03733951 | 140.6755544 | 219.4545212 |
| YCR095C | OCA4 | 0.80902999 | 0.99917121 | 0.03736563 | 168.1110813 | 263.1386667 |
| YHR195W | NVJ1 | 0.85575996 | 0.99917121 | 0.03741134 | 143.5116771 | 223.9619022 |
| YML013W | SEL1 | 0.82516323 | 0.99917121 | 0.0374477 | 100.659039 | 155.7204966 |
| YHR014W | SPO13 | 0.82531271 | 0.99917121 | 0.03757988 | 145.2908246 | 226.7744046 |
| YMR297W | PRC1 | 0.82637362 | 0.99917121 | 0.03798408 | 141.9379959 | 221.3862067 |
| YPL067C | YPL067C | 0.79958948 | 0.99917121 | 0.03799614 | 134.4108039 | 209.398712 |
| YAL027W | YAL027W | 0.82623875 | 0.99917121 | 0.03805097 | 155.6931241 | 243.281215 |
| YLR118C | YLR118C | 0.85795322 | 0.99917121 | 0.03815384 | 137.8463989 | 214.8502019 |
| YOR307C | SLY41 | 0.8208205 | 0.99917121 | 0.03821 | 146.1534962 | 228.0712736 |
| YOR351C | MEK1 | 0.85270416 | 0.99917121 | 0.03838297 | 139.1621908 | 216.9174873 |
| YLR266C | PDR8 | 0.82203485 | 0.99917121 | 0.03854619 | 153.4342214 | 239.6238481 |
| YMR003W | YMR003W | 0.80854273 | 0.99917121 | 0.03855871 | 141.8697175 | 221.2074299 |
| YJR050W | ISY1 | 0.85017093 | 0.99917121 | 0.03874092 | 143.429508 | 223.668969 |
| YEL015W | EDC3 | 0.75301246 | 0.99917121 | 0.03898288 | 129.9318457 | 202.1462877 |
| YKR049C | FMP46 | 0.81588871 | 0.99917121 | 0.039143 | 143.1384873 | 223.156541 |
| YOR263C | YOR263C | 0.79271574 | 0.99917121 | 0.0392102 | 148.0662806 | 230.9951867 |
| YPL274W | SAM3 | 0.81123801 | 0.99917121 | 0.03923696 | 134.0288056 | 208.6391626 |
| YGR226C | YGR226C | 0.82200345 | 0.99917121 | 0.03925535 | 152.9425694 | 238.754506 |
| YPL256C | CLN2 | 0.81673354 | 0.99917121 | 0.03927514 | 138.5318521 | 215.8049939 |
| YHR138C | YHR138C | 0.85437574 | 0.99917121 | 0.03932928 | 134.1801961 | 208.8689767 |
| YKL142W | MRP8 | 0.73054227 | 0.99917121 | 0.03937077 | 137.0386299 | 213.4155835 |
| YDR452W | PPN1 | 0.82905304 | 0.99917121 | 0.0394987 | 150.1065081 | 234.2087983 |
| YCR089W | FIG2 | 0.83409106 | 0.99917121 | 0.03961005 | 160.3364071 | 250.4849407 |
| YIL013C | PDR11 | 0.75437962 | 0.99917121 | 0.03962192 | 149.5167917 | 233.2547341 |
| YDR261C | EXG2 | 0.81990082 | 0.99917121 | 0.03971901 | 142.9294826 | 222.7535076 |
| YKR035C | YKR035C | 0.46953364 | 0.99917121 | 0.03981916 | 116.3454885 | 180.4099189 |
| YIR020W-B | YIR020W-B | 0.82184443 | 0.99917121 | 0.03991158 | 146.6903975 | 228.7187751 |
| YOR352W | YOR352W | 0.84047001 | 0.99917121 | 0.04012314 | 140.8584055 | 219.4063322 |
| YLL041C | SDH2 | 0.62814412 | 0.99917121 | 0.04016678 | 109.7668851 | 169.8920129 |
| YJR133W | XPT1 | 0.84465628 | 0.99917121 | 0.04022165 | 128.764136 | 200.1358502 |
| YBR197C | YBR197C | 0.77947388 | 0.99917121 | 0.04025903 | 145.8628175 | 227.358608 |
| YOR315W | SFG1 | 0.7943615 | 0.99917121 | 0.04028673 | 148.2905416 | 231.2210502 |
| YDR139C | RUB1 | 0.77380709 | 0.99917121 | 0.04029063 | 152.2059429 | 237.4553169 |
| YJL188C | BUD19 | 0.83980658 | 0.99917121 | 0.04030383 | 143.7108546 | 223.9264396 |
| YPL168W | YPL168W | 0.75395459 | 0.99917121 | 0.04033256 | 127.4941981 | 198.1001271 |

| | | | | | | |
|---|---|---|---|---|---|---|
| YAL056W | GPB2 | 0.81679737 | 0.99917121 | 0.04045195 | 136.6175413 | 212.6132499 |
| YIL027C | KRE27 | 0.79742132 | 0.99917121 | 0.0405072 | 151.7470255 | 236.6981514 |
| YGL005C | COG7 | 0.80146652 | 0.99917121 | 0.04058957 | 142.8916103 | 222.5870708 |
| YHR075C | PPE1 | 0.81157273 | 0.99917121 | 0.0406554 | 139.9276057 | 217.859273 |
| YEL017W | GTT3 | 0.82139047 | 0.99917121 | 0.04069958 | 141.40289 | 220.2030749 |
| YBR260C | RGD1 | 0.81957783 | 0.99917121 | 0.04072319 | 146.8915975 | 228.9402139 |
| YDR368W | YPR1 | 0.82390262 | 0.99917121 | 0.04089617 | 147.3925419 | 229.7168117 |
| YMR088C | VBA1 | 0.81109523 | 0.99917121 | 0.04093375 | 154.5506069 | 241.1104711 |
| YDR059C | UBC5 | 0.77137614 | 0.99917121 | 0.04097781 | 136.2169225 | 211.9112115 |
| YDR538W | PAD1 | 0.84537336 | 0.99917121 | 0.0410534 | 139.3897013 | 216.9542125 |
| YAL007C | ERP2 | 0.74589105 | 0.99917121 | 0.04109761 | 143.9143219 | 224.1536634 |
| YLR023C | IZH3 | 0.86204374 | 0.99917121 | 0.04115973 | 133.2310967 | 207.1345137 |
| YLR239C | LIP2 | 0.93427339 | 0.99917121 | 0.04121263 | 46.37788166 | 68.82618228 |
| YML119W | YML119W | 0.81931671 | 0.99917121 | 0.04124264 | 143.6519001 | 223.7181116 |
| YDL146W | LDB17 | 0.64398554 | 0.99917121 | 0.0412871 | 143.6964563 | 223.7836413 |
| YDL114W | YDL114W | 0.81034277 | 0.99917121 | 0.04131726 | 150.0751475 | 233.9371588 |
| YPL259C | APM1 | 0.82568335 | 0.99917121 | 0.04153431 | 148.3053692 | 231.0925684 |
| YKL190W | CNB1 | 0.81390141 | 0.99917121 | 0.04163995 | 135.3412161 | 210.4360467 |
| YBR213W | MET8 | 0.7951723 | 0.99917121 | 0.0416706 | 161.0476784 | 251.3663394 |
| YIL165C | YIL165C | 0.79788236 | 0.99917121 | 0.04167383 | 143.6066985 | 223.5935676 |
| YHL002W | HSE1 | 0.8324848 | 0.99917121 | 0.04175968 | 140.7361087 | 219.0120799 |
| YKL206C | ADD66 | 0.76997156 | 0.99917121 | 0.04185356 | 140.5489085 | 218.7025445 |
| YLR012C | YLR012C | 0.87045342 | 0.99917121 | 0.04196593 | 130.6313843 | 202.8965422 |
| YOR314W | YOR314W | 0.76865298 | 0.99917121 | 0.04199425 | 135.520225 | 210.6779011 |
| YKR078W | YKR078W | 0.73019024 | 0.99917121 | 0.04206424 | 140.8873062 | 219.2157122 |
| YHR154W | RTT107 | 0.81730888 | 0.99917121 | 0.04224324 | 141.2140608 | 219.7142024 |
| YGR146C | YGR146C | 0.74788238 | 0.99917121 | 0.04224336 | 142.3250873 | 221.4833449 |
| YKL107W | YKL107W | 0.76601535 | 0.99917121 | 0.04228409 | 136.3678885 | 211.9923548 |
| YIR003W | YIR003W | 0.81129688 | 0.99917121 | 0.04232246 | 139.4443464 | 216.8865157 |
| YLR083C | EMP70 | 0.81034449 | 0.99917121 | 0.04235917 | 143.032013 | 222.5949098 |
| YML011C | RAD33 | 0.6108864 | 0.99917121 | 0.04236322 | 98.66836732 | 151.9513692 |
| YKL102C | YKL102C | 0.80207197 | 0.99917121 | 0.04240965 | 136.8224427 | 212.7008635 |
| YLL062C | MHT1 | 0.82187387 | 0.99917121 | 0.04259897 | 140.2109399 | 218.0735013 |
| YCR023C | YCR023C | 0.67565867 | 0.99917121 | 0.04260785 | 146.1924157 | 227.5971024 |
| YKL168C | KKQ8 | 0.79065351 | 0.99917121 | 0.04265467 | 143.591421 | 223.4496651 |
| YHL035C | VMR1 | 0.72607595 | 0.99917121 | 0.04272667 | 134.9279667 | 209.645521 |
| YDR319C | YDR319C | 0.81492024 | 0.99917121 | 0.04278544 | 145.37174 | 226.268638 |
| YIL105C | SLM1 | 0.74001554 | 0.99917121 | 0.04292506 | 148.7726987 | 231.6671788 |
| YLR311C | YLR311C | 0.75388491 | 0.99917121 | 0.0429293 | 158.4800083 | 247.1242277 |
| YOR280C | FSH3 | 0.82685651 | 0.99917121 | 0.04304521 | 140.0790706 | 217.8091169 |
| YBR128C | ATG14 | 0.75888472 | 0.99917121 | 0.04306746 | 142.7537358 | 222.0654428 |
| YNR072W | HXT17 | 0.80935239 | 0.99917121 | 0.04309374 | 138.1578087 | 214.7438526 |
| YLL002W | RTT109 | 0.91769863 | 0.99917121 | 0.04333403 | 60.48208133 | 91.02657253 |
| YNL001W | DOM34 | 0.62051432 | 0.99917121 | 0.04344505 | 103.4048249 | 159.3616447 |
| YMR255W | GFD1 | 0.77513358 | 0.99917121 | 0.04354152 | 132.3712469 | 205.474955 |
| YMR192W | GYL1 | 0.77771025 | 0.99917121 | 0.04357181 | 144.6215722 | 224.9782318 |
| YMR029C | FAR8 | 0.70605227 | 0.99917121 | 0.04362033 | 131.3872556 | 203.8984753 |
| YFL021W | GAT1 | 0.78477342 | 0.99917121 | 0.04383251 | 144.9836612 | 225.5230264 |
| YER183C | FAU1 | 0.82576746 | 0.99917121 | 0.04384363 | 137.0000447 | 212.8088521 |
| YLR287C-A | RPS30A | 0.80042523 | 0.99917121 | 0.043873 | 108.8791281 | 168.0265531 |
| YJR053W | BFA1 | 0.77100845 | 0.99917121 | 0.04394243 | 144.7254891 | 225.0985223 |
| YML012W | ERV25 | 0.83682157 | 0.99917121 | 0.04396359 | 138.5224115 | 215.2183892 |
| YER024W | YAT2 | 0.82252143 | 0.99917121 | 0.04399638 | 149.3051483 | 232.3844275 |
| YLR279W | YLR279W | 0.81352084 | 0.99917121 | 0.04400194 | 152.7309243 | 237.8388294 |
| YOL141W | PPM2 | 0.82934935 | 0.99917121 | 0.04414091 | 151.3037413 | 235.5492935 |
| YJR061W | YJR061W | 0.89848584 | 0.99917121 | 0.044381 | 158.568624 | 247.0883583 |
| YKL198C | PTK1 | 0.48375992 | 0.99917121 | 0.04446399 | 140.3937232 | 218.1371931 |
| YKL161C | YKL161C | 0.82373058 | 0.99917121 | 0.04459122 | 139.8211632 | 217.2099587 |
| YDR445C | YDR445C | 0.85060114 | 0.99917121 | 0.04459714 | 119.5434491 | 184.9197472 |
| YMR065W | KAR5 | 0.80569632 | 0.99917121 | 0.04464034 | 143.1823897 | 222.5562652 |
| YOR374W | ALD4 | 0.83412382 | 0.99917121 | 0.04467211 | 135.6408912 | 210.5435849 |
| YIL135C | VHS2 | 0.68401074 | 0.99917121 | 0.04481395 | 155.5316532 | 242.1996154 |
| YIL072W | HOP1 | 0.74978788 | 0.99917121 | 0.04500889 | 148.8002258 | 231.4569716 |
| YLL052C | AQY2 | 0.80919561 | 0.99917121 | 0.04511095 | 144.0195905 | 223.8320207 |

| | | | | | | |
|---|---|---|---|---|---|---|
| YGL227W | VID30 | 0.80318013 | 0.99917121 | 0.04534931 | 159.5127845 | 248.4737567 |
| YDL161W | ENT1 | 0.78037314 | 0.99917121 | 0.0454294 | 141.5002893 | 219.7815553 |
| YKL064W | MNR2 | 0.82471481 | 0.99917121 | 0.04550858 | 135.185507 | 209.7164738 |
| YNR010W | CSE2 | 0.65905316 | 0.99917121 | 0.0455925 | 10.53993357 | 11.22519173 |
| YKL033W-A | YKL033W-A | 0.78360798 | 0.99917121 | 0.04561236 | 125.8846015 | 194.8933996 |
| YGL105W | ARC1 | 0.9251385 | 0.99917121 | 0.04602872 | 59.03393083 | 88.39207869 |
| YPR095C | SYT1 | 0.78948323 | 0.99917121 | 0.04604584 | 149.256965 | 232.0578509 |
| YPL116W | HOS3 | 0.73093652 | 0.99917121 | 0.04627323 | 143.5893112 | 223.0051649 |
| YBR218C | PYC2 | 0.79813102 | 0.99917121 | 0.04629042 | 152.8779486 | 237.7939553 |
| YOR274W | MOD5 | 0.76400373 | 0.99917121 | 0.04639849 | 148.8294215 | 231.334055 |
| YKL128C | PMU1 | 0.71843221 | 0.99917121 | 0.04644145 | 131.8727361 | 204.327612 |
| YHR159W | YHR159W | 0.80581871 | 0.99917121 | 0.04645281 | 143.090474 | 222.1889418 |
| YKL140W | TGL1 | 0.73718977 | 0.99917121 | 0.04651556 | 134.8894587 | 209.122295 |
| YGL260W | YGL260W | 0.76593083 | 0.99917121 | 0.04655456 | 145.3660137 | 225.8000242 |
| YLR020C | YEH2 | 0.82137792 | 0.99917121 | 0.0465708 | 132.7074575 | 205.6410216 |
| YOR193W | PEX27 | 0.79165019 | 0.99917121 | 0.0466181 | 152.5113417 | 237.1702367 |
| YHR158C | KEL1 | 0.83316865 | 0.99917121 | 0.04663464 | 132.3073472 | 204.9961188 |
| YLR187W | SKG3 | 0.82223763 | 0.99917121 | 0.04666751 | 132.3555015 | 205.0687909 |
| YIL099W | SGA1 | 0.80017664 | 0.99917121 | 0.04684915 | 141.6784836 | 219.8922222 |
| YBR266C | YBR266C | 0.92739856 | 0.99917121 | 0.04685958 | 95.45063473 | 146.2794158 |
| YDR024W | FYV1 | 0.85517547 | 0.99917121 | 0.04687786 | 121.8041105 | 188.2414975 |
| YBR058C | UBP14 | 0.75957615 | 0.99917121 | 0.04698687 | 141.7994222 | 220.068011 |
| YMR126C | DLT1 | 0.74602996 | 0.99917121 | 0.04700585 | 113.1995747 | 174.5243464 |
| YDR515W | SLF1 | 0.8435602 | 0.99917121 | 0.04704157 | 126.0862341 | 195.0402361 |
| YFL046W | FMP32 | 0.70977297 | 0.99917121 | 0.04705709 | 144.2008554 | 223.8834047 |
| YER005W | YND1 | 0.81845551 | 0.99917121 | 0.04719384 | 143.1638986 | 222.2155215 |
| YOR161C | PNS1 | 0.75692398 | 0.99917121 | 0.04727005 | 129.8229083 | 200.9625251 |
| YCR079W | PTC6 | 0.79862816 | 0.99917121 | 0.04739511 | 158.9655804 | 247.3530049 |
| YHR163W | SOL3 | 0.70139033 | 0.99917121 | 0.04747722 | 131.4899682 | 203.5918344 |
| YLR423C | ATG17 | 0.77576182 | 0.99917121 | 0.04771654 | 143.7614135 | 223.1032591 |
| YER041W | YEN1 | 0.76465423 | 0.99917121 | 0.04781769 | 144.1114423 | 223.6483012 |
| YMR017W | SPO20 | 0.82850839 | 0.99917121 | 0.04791345 | 124.8797563 | 193.0127939 |
| YML058C-A | YML058C-A | 0.82507536 | 0.99917121 | 0.04817169 | 138.4328368 | 214.5627417 |
| YBR059C | AKL1 | 0.79426199 | 0.99917121 | 0.04818866 | 145.4497896 | 225.7342116 |
| YML019W | OST6 | 0.82213352 | 0.99917121 | 0.04824022 | 134.6116567 | 208.4696789 |
| YNL046W | YNL046W | 0.81051155 | 0.99917121 | 0.04828768 | 141.8770124 | 220.0329799 |
| YDL095W | PMT1 | 0.70984939 | 0.99917121 | 0.04831895 | 145.6399213 | 226.0210862 |
| YDR317W | HIM1 | 0.7249986 | 0.99917121 | 0.04849705 | 139.6562649 | 216.471219 |
| YHR095W | YHR095W | 0.79800064 | 0.99917121 | 0.04860855 | 151.916719 | 235.9807242 |
| YDR310C | SUM1 | 0.73459693 | 0.99917121 | 0.04861549 | 134.0495417 | 207.528838 |
| YCR105W | ADH7 | 0.69796116 | 0.99917121 | 0.04865486 | 160.0407629 | 248.911509 |
| YBR065C | ECM2 | 0.81062661 | 0.99917121 | 0.04869799 | 139.8491072 | 216.7537971 |
| YCR020C-A | MAK31 | 0.73231427 | 0.99917121 | 0.04870967 | 139.2145532 | 215.7419325 |
| YMR322C | SNO4 | 0.74580647 | 0.99917121 | 0.04884677 | 134.9668441 | 208.961322 |
| YDL048C | STP4 | 0.78631576 | 0.99917121 | 0.04912457 | 146.3319054 | 227.0247622 |
| YJL100W | LSB6 | 0.77988045 | 0.99917121 | 0.04922291 | 151.0773109 | 234.5691845 |
| YPR084W | YPR084W | 0.85146408 | 0.99917121 | 0.04925569 | 130.2520287 | 201.4037701 |
| YFR056C | YFR056C | 0.75832503 | 0.99917121 | 0.04936922 | 146.8046682 | 227.7477481 |
| YPL248C | GAL4 | 0.80077484 | 0.99917121 | 0.04959219 | 147.645116 | 229.0588629 |
| YJR036C | HUL4 | 0.79662141 | 0.99917121 | 0.04964708 | 140.9585993 | 218.404807 |
| YHR082C | KSP1 | 0.79070305 | 0.99917121 | 0.0496822 | 146.3963147 | 227.0593445 |
| YPL200W | CSM4 | 0.79803529 | 0.99917121 | 0.04974463 | 134.9915533 | 208.8912088 |
| YKL120W | OAC1 | 0.68126615 | 0.99917121 | 0.04985075 | 140.2720095 | 217.2866768 |
| YOR055W | YOR055W | 0.81684272 | 0.99917121 | 0.04991474 | 151.2087371 | 234.6941205 |
| YOR121C | YOR121C | 0.85194111 | 0.99917121 | 0.04992831 | 142.8760358 | 221.4237782 |
| YLR263W | RED1 | 0.81561987 | 0.99917121 | 0.05010047 | 133.0291766 | 205.7230115 |
| YML097C | VPS9 | 0.24819297 | 0.99917121 | 0.050304 | 87.78266637 | 133.6493114 |
| YML002W | YML002W | 0.61635408 | 0.99917121 | 0.05035293 | 125.799854 | 194.180525 |
| YEL011W | GLC3 | 0.75194395 | 0.99917121 | 0.05036725 | 140.0998668 | 216.9495963 |
| YER067W | YER067W | 0.78129265 | 0.99917121 | 0.05036915 | 139.8294062 | 216.5186933 |
| YOR227W | YOR227W | 0.79766749 | 0.99917121 | 0.05053984 | 140.9729264 | 218.3187838 |
| YLL021W | SPA2 | 0.74062933 | 0.99917121 | 0.05055304 | 146.9763207 | 227.8767605 |
| YMR194W | RPL36A | 0.75724777 | 0.99917121 | 0.05066167 | 113.460974 | 174.4949053 |
| YDR492W | IZH1 | 0.76475256 | 0.99917121 | 0.05102359 | 145.3474346 | 225.2256168 |

| | | | | | | |
|---|---|---|---|---|---|---|
| YER039C-A | YER039C-A | 0.77316797 | 0.99917121 | 0.05107759 | 140.0857508 | 216.8405213 |
| YNL024C | YNL024C | 0.74900483 | 0.99917121 | 0.05118742 | 138.3228996 | 214.0200316 |
| YKR032W | YKR032W | 0.74585097 | 0.99917121 | 0.05125096 | 152.1470009 | 236.0252784 |
| YER184C | YER184C | 0.81053905 | 0.99917121 | 0.0513222 | 140.2974807 | 217.1478507 |
| YKL103C | LAP4 | 0.78928302 | 0.99917121 | 0.0514517 | 133.4421196 | 206.2158376 |
| YGR261C | APL6 | 0.87390551 | 0.99917121 | 0.05145857 | 139.404448 | 215.7091935 |
| YBR157C | ICS2 | 0.72720252 | 0.99917121 | 0.05151896 | 155.9871051 | 242.1074478 |
| YNL004W | HRB1 | 0.76352796 | 0.99917121 | 0.05156325 | 137.7878928 | 213.122289 |
| YOL093W | TRM10 | 0.82474896 | 0.99917121 | 0.05167954 | 115.081546 | 176.9513551 |
| YGR232W | NAS6 | 0.63300283 | 0.99917121 | 0.05182409 | 146.558063 | 227.0557878 |
| YGR284C | ERV29 | 0.77540332 | 0.99917121 | 0.05197337 | 139.6154567 | 215.9824362 |
| YLR401C | DUS3 | 0.7826525 | 0.99917121 | 0.05202089 | 143.2670292 | 221.7912739 |
| YLR170C | APS1 | 0.81554156 | 0.99917121 | 0.05211988 | 134.2740445 | 207.4591057 |
| YOR355W | GDS1 | 0.78120865 | 0.99917121 | 0.05216072 | 142.3547355 | 220.3215235 |
| YIR019C | MUC1 | 0.68521684 | 0.99917121 | 0.05218977 | 139.4508848 | 215.6939962 |
| YDR451C | YHP1 | 0.82408845 | 0.99917121 | 0.05224587 | 151.97807 | 235.6349894 |
| YDL175C | AIR2 | 0.74473226 | 0.99917121 | 0.05232777 | 152.7365281 | 236.8327454 |
| YDR233C | RTN1 | 0.67022756 | 0.99917121 | 0.05241942 | 143.0165332 | 221.3438079 |
| YDR213W | UPC2 | 0.83602932 | 0.99917121 | 0.05276044 | 156.305805 | 242.4635838 |
| YKL076C | PSY1 | 0.75572762 | 0.99917121 | 0.05283464 | 130.2574058 | 200.9760209 |
| YJR149W | YJR149W | 0.73961734 | 0.99917121 | 0.05321926 | 143.9385018 | 222.7144084 |
| YIL014W | MNT3 | 0.7519133 | 0.99917121 | 0.05325047 | 154.799539 | 240.0053219 |
| YPL260W | YPL260W | 0.69703011 | 0.99917121 | 0.05339714 | 137.5536424 | 212.5257064 |
| YGL141W | HUL5 | 0.7361439 | 0.99917121 | 0.05346281 | 147.3332157 | 228.0903347 |
| YDR148C | KGD2 | 0.69186873 | 0.99917121 | 0.05374038 | 123.1895022 | 189.6109302 |
| YOL009C | MDM12 | 0.93838924 | 0.99917121 | 0.0537853 | 40.44541625 | 57.84679775 |
| YDR402C | DIT2 | 0.78116678 | 0.99917121 | 0.05381967 | 144.927825 | 224.2165736 |
| YGL153W | PEX14 | 0.73515059 | 0.99917121 | 0.05383153 | 151.7310385 | 235.0483157 |
| YNL122C | YNL122C | 0.85780096 | 0.99917121 | 0.05389765 | 118.9965828 | 182.9151061 |
| YJR094C | IME1 | 0.72549935 | 0.99917121 | 0.05392365 | 144.6959035 | 223.8345934 |
| YDL122W | UBP1 | 0.66164034 | 0.99917121 | 0.05398938 | 153.2594798 | 237.4629062 |
| YPL170W | DAP1 | 0.74258123 | 0.99917121 | 0.05412529 | 149.460074 | 231.3963024 |
| YPL053C | KTR6 | 0.77126369 | 0.99917121 | 0.05441017 | 146.3919168 | 226.4759521 |
| YGL019W | CKB1 | 0.76450138 | 0.99917121 | 0.05460353 | 111.0843251 | 170.229862 |
| YOR338W | YOR338W | 0.7377956 | 0.99917121 | 0.05460972 | 157.7416141 | 244.5244674 |
| YNL146W | YNL146W | 0.75948095 | 0.99917121 | 0.05483026 | 139.6287991 | 215.6553967 |
| YKL092C | BUD2 | 0.64391015 | 0.99917121 | 0.05487421 | 136.170237 | 210.1427507 |
| YLR082C | SRL2 | 0.80000325 | 0.99917121 | 0.05490744 | 136.0387009 | 209.9292465 |
| YBR138C | YBR138C | 0.75589073 | 0.99917121 | 0.05503699 | 150.6350029 | 233.1560704 |
| YMR233W | YMR233W | 0.76341014 | 0.99917121 | 0.05507804 | 146.4137424 | 226.4292857 |
| YMR238W | DFG5 | 0.80276154 | 0.99917121 | 0.05516304 | 140.4685278 | 216.951981 |
| YBL100C | YBL100C | 0.75758597 | 0.99917121 | 0.05535484 | 144.2403124 | 222.93465 |
| YML124C | TUB3 | 0.73320078 | 0.99917121 | 0.0554027 | 135.4952268 | 209.0034606 |
| YMR304C-A | YMR304C-A | 0.74401375 | 0.99917121 | 0.05549051 | 132.86944 | 204.8115493 |
| YDR532C | KRE28 | 0.76652033 | 0.99917121 | 0.05559222 | 20.8835325 | 26.47688776 |
| YBL088C | TEL1 | 0.76151983 | 0.99917121 | 0.05572722 | 151.9178382 | 235.1146646 |
| YLR280C | YLR280C | 0.79049732 | 0.99917121 | 0.05575317 | 140.0778943 | 216.258007 |
| YBL015W | ACH1 | 0.60648347 | 0.99917121 | 0.05598319 | 145.8128462 | 225.3620928 |
| YMR141C | YMR141C | 0.85000684 | 0.99917121 | 0.05609219 | 127.385806 | 196.0062603 |
| YBL070C | YBL070C | 0.70452314 | 0.99917121 | 0.05611687 | 147.7917022 | 228.4968536 |
| YNL297C | MON2 | 0.43509739 | 0.99917121 | 0.0561385 | 96.47545885 | 146.7801084 |
| YLR421C | RPN13 | 0.74230459 | 0.99917121 | 0.05615323 | 142.9277863 | 220.7472994 |
| YBL010C | YBL010C | 0.6443906 | 0.99917121 | 0.05617749 | 146.0636896 | 225.7378396 |
| YOR386W | PHR1 | 0.71905104 | 0.99917121 | 0.05617865 | 135.5566786 | 209.0067175 |
| YDR285W | ZIP1 | 0.55919186 | 0.99917121 | 0.05630912 | 145.2785594 | 224.4715802 |
| YDR242W | AMD2 | 0.84202747 | 0.99917121 | 0.05654753 | 130.2772031 | 200.5549044 |
| YOR202W | HIS3 | 0.43457356 | 0.99917121 | 0.05684277 | 129.2365162 | 198.8617596 |
| YPL134C | ODC1 | 0.71900549 | 0.99917121 | 0.05693843 | 137.7140298 | 212.3493793 |
| YOL014W | YOL014W | 0.68366018 | 0.99917121 | 0.05704055 | 138.4095713 | 213.4444849 |
| YMR153W | NUP53 | 0.51446424 | 0.99917121 | 0.05704632 | 123.679247 | 189.9877523 |
| YGR228W | YGR228W | 0.77837933 | 0.99917121 | 0.05722679 | 149.7861175 | 231.5373763 |
| YNL020C | ARK1 | 0.92355896 | 0.99917121 | 0.05742102 | 93.45203716 | 141.8093686 |
| YLR070C | XYL2 | 0.79826715 | 0.99917121 | 0.05744939 | 140.0811779 | 216.0564489 |
| YGR254W | ENO1 | 0.82306092 | 0.99917121 | 0.05760156 | 134.1804933 | 206.6418634 |

| | | | | | | |
|---|---|---|---|---|---|---|
| YDR135C | YCF1 | 0.63976325 | 0.99917121 | 0.05761809 | 139.5212901 | 215.1443374 |
| YGR259C | YGR259C | 0.7717861 | 0.99917121 | 0.05767859 | 140.6008774 | 216.8560566 |
| YMR063W | RIM9 | 0.56748554 | 0.99917121 | 0.05776244 | 105.2973439 | 160.6297796 |
| YDR034C | LYS14 | 0.36986364 | 0.99917121 | 0.05798192 | 122.0350172 | 187.2554815 |
| YHR133C | NSG1 | 0.76636843 | 0.99917121 | 0.05801573 | 150.5754481 | 232.6980971 |
| YKL177W | YKL177W | 0.74382316 | 0.99917121 | 0.05841001 | 146.9743887 | 226.9158353 |
| YHR039C | MSC7 | 0.7742633 | 0.99917121 | 0.058418 | 165.4980395 | 256.4112454 |
| YIL131C | FKH1 | 0.81729286 | 0.99917121 | 0.05856398 | 133.4139096 | 205.3038538 |
| YIL030C | SSM4 | 0.72986706 | 0.99917121 | 0.05857861 | 146.3307265 | 225.8703369 |
| YNR013C | PHO91 | 0.69531357 | 0.99917121 | 0.05858253 | 125.7916763 | 193.1642272 |
| YIL119C | RPI1 | 0.65967762 | 0.99917121 | 0.05864713 | 137.9654672 | 212.5414511 |
| YCR087C-A | LUG1 | 0.81308921 | 0.99917121 | 0.05878138 | 130.2173683 | 200.1872949 |
| YLL020C | YLL020C | 0.7188011 | 0.99917121 | 0.05880603 | 141.3173543 | 217.859501 |
| YKR100C | SKG1 | 0.69781467 | 0.99917121 | 0.05895605 | 145.1350217 | 223.9203258 |
| YLR445W | YLR445W | 0.77392121 | 0.99917121 | 0.05895984 | 115.1899044 | 176.2363553 |
| YIR005W | IST3 | 0.75914634 | 0.99917121 | 0.05896278 | 114.5569505 | 175.2281038 |
| YOR356W | YOR356W | 0.73373406 | 0.99917121 | 0.0589655 | 144.0938145 | 222.2611936 |
| YIL045W | PIG2 | 0.6854647 | 0.99917121 | 0.05916265 | 153.8470675 | 237.7678825 |
| YBR174C | :::SWD3 | 0.87849276 | 0.99917121 | 0.05916738 | 81.38218137 | 122.3768725 |
| YLR281C | YLR281C | 0.86317112 | 0.99917121 | 0.05916996 | 123.5807218 | 189.5719702 |
| YGL081W | YGL081W | 0.74204444 | 0.99917121 | 0.05923265 | 150.4674356 | 232.3777475 |
| YGL007W | YGL007W | 0.51016675 | 0.99917121 | 0.05926186 | 127.8987788 | 196.4366819 |
| YGR203W | YGR203W | 0.63524171 | 0.99917121 | 0.0592926 | 165.9502678 | 257.0247335 |
| YOL158C | ENB1 | 0.70771159 | 0.99917121 | 0.0593747 | 141.1181759 | 217.4730107 |
| YLR375W | STP3 | 0.7439391 | 0.99917121 | 0.0594925 | 140.1113614 | 215.8554349 |
| YJR034W | PET191 | 0.78056161 | 0.99917121 | 0.05962035 | 137.5437153 | 211.7512228 |
| YAL015C | NTG1 | 0.68119941 | 0.99917121 | 0.05965205 | 145.2091791 | 223.9535615 |
| YLR055C | SPT8 | 0.82223922 | 0.99917121 | 0.0598924 | 110.5272785 | 168.6980713 |
| YHR146W | CRP1 | 0.75297606 | 0.99917121 | 0.05993896 | 148.0062892 | 228.3726005 |
| YGL231C | YGL231C | 0.50589468 | 0.99917121 | 0.06001226 | 124.2346475 | 190.5105717 |
| YPL199C | YPL199C | 0.73156153 | 0.99917121 | 0.06004579 | 136.5792959 | 210.1636505 |
| YBR085W | AAC3 | 0.68511572 | 0.99917121 | 0.06018283 | 127.1158016 | 195.0776226 |
| YGL257C | MNT2 | 0.70052195 | 0.99917121 | 0.06041684 | 155.8636367 | 240.826095 |
| YBR115C | LYS2 | 0.5874118 | 0.99917121 | 0.06047814 | 112.3446061 | 171.5205092 |
| YDL121C | YDL121C | 0.69745235 | 0.99917121 | 0.06054749 | 162.3790217 | 251.185028 |
| YPR120C | CLB5 | 0.84003826 | 0.99917121 | 0.06056654 | 126.0244985 | 193.293092 |
| YKL051W | SFK1 | 0.7175982 | 0.99917121 | 0.06061348 | 135.3773447 | 208.1804997 |
| YDR169C | STB3 | 0.51359083 | 0.99917121 | 0.06067742 | 144.5308313 | 222.748382 |
| YFR018C | YFR018C | 0.73529529 | 0.99917121 | 0.06074021 | 151.3116651 | 233.5382793 |
| YJL083W | TAX4 | 0.75624695 | 0.99917121 | 0.06074035 | 149.3695308 | 230.4456791 |
| YKL009W | MRT4 | 0.61849335 | 0.99917121 | 0.06080159 | 92.54553915 | 139.9537669 |
| YBR235W | YBR235W | 0.54192916 | 0.99917121 | 0.06105241 | 143.7655988 | 221.4841393 |
| YOL112W | MSB4 | 0.79620848 | 0.99917121 | 0.06113192 | 131.0399048 | 201.2105166 |
| YGR096W | TPC1 | 0.72881468 | 0.99917121 | 0.06118849 | 140.1576355 | 215.7223608 |
| YOR007C | SGT2 | 0.71004866 | 0.99917121 | 0.06121161 | 141.0621177 | 217.1598065 |
| YOL047C | YOL047C | 0.73950432 | 0.99917121 | 0.06122958 | 144.9610703 | 223.3661645 |
| YML096W | YML096W | 0.48971425 | 0.99917121 | 0.06123691 | 128.6252255 | 197.3526704 |
| YHR017W | YSC83 | 0.57663896 | 0.99917121 | 0.06130681 | 153.9650861 | 237.6944147 |
| YKL121W | YKL121W | 0.6631473 | 0.99917121 | 0.06144122 | 124.4694868 | 190.7103153 |
| YDL109C | YDL109C | 0.41688676 | 0.99917121 | 0.06154677 | 134.5712217 | 206.7830808 |
| YOR009W | TIR4 | 0.75841296 | 0.99917121 | 0.06172947 | 148.4178813 | 228.8097226 |
| YGR059W | SPR3 | 0.69570511 | 0.99917121 | 0.06186897 | 142.3361131 | 219.1083303 |
| YDR426C | YDR426C | 0.75625906 | 0.99917121 | 0.06197335 | 140.2254409 | 215.7346482 |
| YDL237W | YDL237W | 0.51130525 | 0.99917121 | 0.06217139 | 140.4874128 | 216.1276593 |
| YKL097C | YKL097C | 0.66185616 | 0.99917121 | 0.06222677 | 134.9995348 | 207.3822125 |
| YFL027C | GYP8 | 0.72701477 | 0.99917121 | 0.06227662 | 141.4996963 | 217.726754 |
| YOR016C | ERP4 | 0.75613641 | 0.99917121 | 0.06231374 | 142.1246097 | 218.7173179 |
| YLR109W | AHP1 | 0.76342782 | 0.99917121 | 0.06245326 | 145.4164249 | 223.9420748 |
| YCL040W | GLK1 | 0.63732779 | 0.99917121 | 0.06252564 | 139.3669439 | 214.3002792 |
| YGL259W | YPS5 | 0.68837815 | 0.99917121 | 0.06263328 | 147.1683984 | 226.7099075 |
| YJR125C | ENT3 | 0.42451224 | 0.99917121 | 0.06263504 | 116.3749967 | 177.6754062 |
| YGL166W | CUP2 | 0.69826517 | 0.99917121 | 0.0627148 | 150.0936177 | 231.3579821 |
| YCR091W | KIN82 | 0.7304496 | 0.99917121 | 0.06283941 | 146.8687406 | 226.2076144 |
| YGL060W | YBP2 | 0.68606018 | 0.99917121 | 0.06307816 | 140.8997267 | 216.6736684 |

| | | | | | | |
|---|---|---|---|---|---|---|
| YBR286W | APE3 | 0.73479151 | 0.99917121 | 0.06310395 | 146.4801988 | 225.5566634 |
| YCL062W | YCL062W | 0.77304555 | 0.99917121 | 0.06315682 | 131.0101864 | 200.9163364 |
| YMR147W | YMR147W | 0.71896166 | 0.99917121 | 0.06319713 | 135.9685205 | 208.8068923 |
| YLR418C | CDC73 | 0.63626185 | 0.99917121 | 0.0633098 | 95.59440161 | 144.502887 |
| YMR054W | STV1 | 0.72578716 | 0.99917121 | 0.06334507 | 141.6731025 | 217.8726247 |
| YOR010C | TIR2 | 0.64011851 | 0.99917121 | 0.06354509 | 138.7465686 | 213.1881342 |
| YCL009C | ILV6 | 0.63933797 | 0.99917121 | 0.06357889 | 142.7979964 | 219.6353597 |
| YBR053C | YBR053C | 0.67674281 | 0.99917121 | 0.0636298 | 144.1520709 | 221.7853309 |
| YDL240W | LRG1 | 0.5542951 | 0.99917121 | 0.06373921 | 158.8653187 | 245.2008312 |
| YGL083W | SCY1 | 0.67732978 | 0.99917121 | 0.06374134 | 155.349035 | 239.6013701 |
| YDR031W | MIC14 | 0.75063884 | 0.99917121 | 0.06380142 | 139.5464423 | 214.4305747 |
| YBR249C | ARO4 | 0.73802389 | 0.99917121 | 0.06405963 | 145.77696 | 224.3203443 |
| YPL156C | PRM4 | 0.59925529 | 0.99917121 | 0.0641612 | 129.2148055 | 197.9349926 |
| YPR158W | YPR158W | 0.58516781 | 0.99917121 | 0.06416986 | 141.2424305 | 217.086287 |
| YBR274W | CHK1 | 0.45181724 | 0.99917121 | 0.06421925 | 148.6346118 | 228.8513051 |
| YPR152C | URN1 | 0.71749412 | 0.99917121 | 0.06430821 | 129.9263767 | 199.0501516 |
| YGR169C | PUS6 | 0.77149514 | 0.99917121 | 0.06466378 | 155.5424412 | 239.7968868 |
| YLR389C | STE23 | 0.75254117 | 0.99917121 | 0.0647105 | 139.1800512 | 213.7363208 |
| YBR184W | YBR184W | 0.71113724 | 0.99917121 | 0.06473039 | 151.1304957 | 232.7633456 |
| YPR200C | ARR2 | 0.74184101 | 0.99917121 | 0.06482368 | 144.1703587 | 221.6689057 |
| YNL125C | ESBP6 | 0.45654829 | 0.99917121 | 0.06514029 | 146.2005357 | 224.8630872 |
| YMR006C | PLB2 | 0.67822618 | 0.99917121 | 0.06514109 | 138.2007974 | 212.1244992 |
| YOR312C | RPL20B | 0.7628918 | 0.99917121 | 0.06521542 | 131.3088298 | 201.1409199 |
| YCL055W | KAR4 | 0.45860277 | 0.99917121 | 0.06524486 | 140.5970465 | 215.927547 |
| YPL149W | ATG5 | 0.7211332 | 0.99917121 | 0.06533856 | 164.2736429 | 253.6178704 |
| YLR455W | YLR455W | 0.6143174 | 0.99917121 | 0.06535415 | 120.1711045 | 183.388701 |
| YER187W | YER187W | 0.65785294 | 0.99917121 | 0.06544105 | 135.8903472 | 208.4088545 |
| YDL155W | CLB3 | 0.65418372 | 0.99917121 | 0.06561262 | 144.5874908 | 222.236951 |
| YMR106C | YKU80 | 0.70083666 | 0.99917121 | 0.06562608 | 141.706167 | 217.6471959 |
| YJR051W | OSM1 | 0.70741099 | 0.99917121 | 0.06569359 | 149.1300711 | 229.4605193 |
| YJL130C | URA2 | 0.69152142 | 0.99917121 | 0.06592756 | 143.8198622 | 220.9762136 |
| YMR004W | MVP1 | 0.75232838 | 0.99917121 | 0.06594403 | 134.4942356 | 206.1244188 |
| YMR109W | MYO5 | 0.73075951 | 0.99917121 | 0.06596367 | 141.386785 | 217.0974673 |
| YJL107C | YJL107C | 0.79707806 | 0.99917121 | 0.0659901 | 142.768505 | 219.294446 |
| YPL138C | SPP1 | 0.77843697 | 0.99917121 | 0.06604799 | 127.2244582 | 194.5356168 |
| YDR105C | TMS1 | 0.53710068 | 0.99917121 | 0.06632391 | 138.4341764 | 212.351925 |
| YNL166C | BNI5 | 0.68340344 | 0.99917121 | 0.06657938 | 141.1901324 | 216.7092632 |
| YER162C | RAD4 | 0.7503601 | 0.99917121 | 0.06668243 | 136.6719884 | 209.5021731 |
| YML037C | YML037C | 0.64563422 | 0.99917121 | 0.06685516 | 141.4711591 | 217.1231398 |
| YGL164C | YRB30 | 0.66604088 | 0.99917121 | 0.06712096 | 152.3645672 | 234.4370003 |
| YMR157C | FMP39 | 0.67770982 | 0.99917121 | 0.06720383 | 134.555391 | 206.0682168 |
| YMR272C | SCS7 | 0.7145401 | 0.99917121 | 0.06725499 | 141.0197548 | 216.3555966 |
| YLR097C | HRT3 | 0.73433862 | 0.99917121 | 0.06734134 | 133.8123767 | 204.8683047 |
| YOL092W | YOL092W | 0.76628219 | 0.99917121 | 0.06747156 | 136.2983408 | 208.8109868 |
| YLR257W | YLR257W | 0.70454358 | 0.99917121 | 0.06749941 | 155.6288724 | 239.5888235 |
| YGR051C | YGR051C | 0.70776952 | 0.99917121 | 0.06773939 | 149.5768833 | 229.922601 |
| YML117W | NAB6 | 0.56934888 | 0.99917121 | 0.06783301 | 135.1815776 | 206.9886292 |
| YHR081W | LRP1 | 0.72485915 | 0.99917121 | 0.06792359 | 93.40122002 | 140.4480741 |
| YBR229C | ROT2 | 0.61767695 | 0.99917121 | 0.06816929 | 151.0693581 | 232.2467544 |
| YOR089C | VPS21 | 0.60623139 | 0.99917121 | 0.06824914 | 121.2324007 | 184.7257402 |
| YNL242W | ATG2 | 0.70109864 | 0.99917121 | 0.06826003 | 117.2257384 | 178.3443504 |
| YNL239W | LAP3 | 0.7547283 | 0.99917121 | 0.06830235 | 156.1828893 | 240.3731329 |
| YBR161W | CSH1 | 0.65268824 | 0.99917121 | 0.06832917 | 145.6388324 | 223.5798933 |
| YOR094W | ARF3 | 0.65628182 | 0.99917121 | 0.0683498 | 140.9327337 | 216.0835581 |
| YLR043C | TRX1 | 0.71873184 | 0.99917121 | 0.06838783 | 139.7341035 | 214.1702674 |
| YJR150C | DAN1 | 0.53725889 | 0.99917121 | 0.06849622 | 115.9197597 | 176.2359636 |
| YLR262C | YPT6 | 0.59234864 | 0.99917121 | 0.06851633 | 95.70909365 | 144.050786 |
| YIL084C | SDS3 | 0.62116116 | 0.99917121 | 0.06856632 | 138.2872187 | 211.8445403 |
| YDL142C | CRD1 | 0.64565283 | 0.99917121 | 0.06858687 | 142.2614393 | 218.1704385 |
| YOR072W | YOR072W | 0.59128604 | 0.99917121 | 0.0687443 | 143.8454007 | 220.6734882 |
| YML017W | PSP2 | 0.73543321 | 0.99917121 | 0.06906298 | 141.2799822 | 216.5495594 |
| YDL052C | SLC1 | 0.87317988 | 0.99917121 | 0.06918883 | 41.41851029 | 57.51846215 |
| YOL007C | CSI2 | 0.68185055 | 0.99917121 | 0.06921726 | 141.0785748 | 216.210038 |
| YAR020C | PAU7 | 0.63271112 | 0.99917121 | 0.06928969 | 146.3850406 | 224.651029 |

| | | | | | | |
|---|---|---|---|---|---|---|
| YGR137W | YGR137W | 0.65165743 | 0.99917121 | 0.06948405 | 156.0084033 | 239.9512257 |
| YDR519W | FPR2 | 0.64414146 | 0.99917121 | 0.06963051 | 142.0557607 | 217.7156929 |
| YLR350W | ORM2 | 0.74028378 | 0.99917121 | 0.06978704 | 140.8519244 | 215.7796658 |
| YBR111C | YSA1 | 0.76976577 | 0.99917121 | 0.06981199 | 152.5052493 | 234.3329526 |
| YDL001W | RMD1 | 0.76750319 | 0.99917121 | 0.06981438 | 155.7678214 | 239.5278613 |
| YCR030C | SYP1 | 0.67572706 | 0.99917121 | 0.06983406 | 138.8471034 | 212.5815294 |
| YPR073C | LTP1 | 0.6917118 | 0.99917121 | 0.06988276 | 127.2742708 | 194.1474377 |
| YPR096C | YPR096C | 0.72888365 | 0.99917121 | 0.07002682 | 143.9312185 | 220.6537885 |
| YMR306C-A | YMR306C-A | 0.59969289 | 0.99917121 | 0.0700496 | 135.6793521 | 207.5110412 |
| YPR193C | HPA2 | 0.74855354 | 0.99917121 | 0.0700749 | 149.4930908 | 229.5044498 |
| YLR073C | YLR073C | 0.70977351 | 0.99917121 | 0.07025321 | 139.6496896 | 213.8084394 |
| YNL109W | YNL109W | 0.79153998 | 0.99917121 | 0.07029573 | 123.5443534 | 188.1577082 |
| YPL125W | KAP120 | 0.70322131 | 0.99917121 | 0.07033436 | 130.6991067 | 199.5459655 |
| YDL013W | HEX3 | 0.83747276 | 0.99917121 | 0.07036917 | 96.91650007 | 145.7475351 |
| YBR019C | GAL10 | 0.60352053 | 0.99917121 | 0.07053343 | 144.323261 | 221.2163015 |
| YPL136W | YPL136W | 0.74282223 | 0.99917121 | 0.07060963 | 141.4245229 | 216.5911675 |
| YPL150W | YPL150W | 0.7000974 | 0.99917121 | 0.07066395 | 145.2486143 | 222.6738895 |
| YHR115C | DMA1 | 0.72302302 | 0.99917121 | 0.07073935 | 153.4971436 | 235.7993535 |
| YNL111C | CYB5 | 0.68184739 | 0.99917121 | 0.07086723 | 133.9887556 | 204.7193197 |
| YBL046W | PSY4 | 0.60315374 | 0.99917121 | 0.07088528 | 145.9916476 | 223.8300859 |
| YLR309C | IMH1 | 0.69549336 | 0.99917121 | 0.07095728 | 133.2514986 | 203.5343602 |
| YCR019W | MAK32 | 0.73087722 | 0.99917121 | 0.07105965 | 148.015462 | 227.0314758 |
| YNL003C | PET8 | 0.9006457 | 0.99917121 | 0.07117086 | 57.1545677 | 82.33435343 |
| YKL204W | EAP1 | 0.73275139 | 0.99917121 | 0.07117958 | 120.7778334 | 183.6446516 |
| YGL032C | AGA2 | 0.60531068 | 0.99917121 | 0.07129351 | 150.5230283 | 230.9959222 |
| YKL050C | YKL050C | 0.66803366 | 0.99917121 | 0.07132109 | 137.3717643 | 210.0509686 |
| YEL004W | YEA4 | 0.67315808 | 0.99917121 | 0.07136142 | 147.3476699 | 225.931319 |
| YNL165W | YNL165W | 0.38419267 | 0.99917121 | 0.07137969 | 124.1832486 | 189.042915 |
| YGR197C | SNG1 | 0.5459963 | 0.99917121 | 0.07142541 | 139.5809184 | 213.5560266 |
| YDR306C | YDR306C | 0.60648048 | 0.99917121 | 0.07175818 | 145.9769211 | 223.7002195 |
| YLR210W | CLB4 | 0.71819817 | 0.99917121 | 0.0718878 | 141.3520571 | 216.3199534 |
| YOR030W | DFG16 | 0.71890115 | 0.99917121 | 0.07193333 | 151.9976292 | 233.266022 |
| YDL211C | YDL211C | 0.49720263 | 0.99917121 | 0.07211255 | 145.5810792 | 223.0266938 |
| YEL043W | YEL043W | 0.54939622 | 0.99917121 | 0.07228812 | 135.0450206 | 206.2280563 |
| YDL157C | YDL157C | 0.75628922 | 0.99917121 | 0.07256992 | 153.1134465 | 234.9652021 |
| YAR027W | UIP3 | 0.63910473 | 0.99917121 | 0.07269515 | 141.6420141 | 216.6832457 |
| YGR033C | TIM21 | 0.56728064 | 0.99917121 | 0.07274484 | 154.8752124 | 237.7492493 |
| YDR008C | YDR008C | 0.56395311 | 0.99917121 | 0.07281738 | 139.0082578 | 212.4744474 |
| YML009C | MRPL39 | 0.70348753 | 0.99917121 | 0.07282376 | 139.5057234 | 213.2658152 |
| YHR113W | YHR113W | 0.51433635 | 0.99917121 | 0.07287751 | 140.9185699 | 215.5090275 |
| YBR090C-A | YBR090C-A | 0.54332758 | 0.99917121 | 0.07305391 | 141.9697091 | 217.1613191 |
| YMR101C | SRT1 | 0.61020459 | 0.99917121 | 0.07307187 | 147.6614632 | 226.2224706 |
| YBL067C | UBP13 | 0.6112551 | 0.99917121 | 0.07308918 | 141.5431414 | 216.4777682 |
| YGL023C | PIB2 | 0.79879696 | 0.99917121 | 0.0732701 | 141.9739605 | 217.141732 |
| YPR065W | ROX1 | 0.64506244 | 0.99917121 | 0.07330662 | 138.1600317 | 211.06412 |
| YBR137W | YBR137W | 0.47108298 | 0.99917121 | 0.0733434 | 142.1227398 | 217.3697071 |
| YNL326C | PFA3 | 0.67845313 | 0.99917121 | 0.07342504 | 114.1942007 | 172.8873704 |
| YPL154C | PEP4 | 0.53336316 | 0.99917121 | 0.0734261 | 138.5763712 | 211.712518 |
| YPR029C | APL4 | 0.52793272 | 0.99917121 | 0.07352494 | 141.7037212 | 216.6803453 |
| YEL064C | AVT2 | 0.69988835 | 0.99917121 | 0.07355393 | 142.8152316 | 218.4467403 |
| YER087C-A | YER087C-A | 0.54991064 | 0.99917121 | 0.07358208 | 140.3588229 | 214.5318126 |
| YBR225W | YBR225W | 0.67938805 | 0.99917121 | 0.07366632 | 140.7088124 | 215.078853 |
| YNR042W | YNR042W | 0.74374891 | 0.99917121 | 0.07368267 | 141.1491555 | 215.7780461 |
| YIR018W | YAP5 | 0.70823444 | 0.99917121 | 0.0738 | 146.4310426 | 224.1744264 |
| YGR290W | YGR290W | 0.68765599 | 0.99917121 | 0.07388367 | 145.2522719 | 222.2871944 |
| YBR122C | MRPL36 | 0.90076942 | 0.99917121 | 0.07409851 | 33.50465276 | 44.31818219 |
| YGL146C | YGL146C | 0.68753153 | 0.99917121 | 0.07427096 | 142.7428467 | 218.2440629 |
| YPL056C | YPL056C | 0.67888422 | 0.99917121 | 0.0743483 | 152.5127136 | 233.7918136 |
| YCR044C | PER1 | 0.69249031 | 0.99917121 | 0.07438119 | 130.6704375 | 199.0069616 |
| YNL070W | TOM7 | 0.44299993 | 0.99917121 | 0.07443437 | 95.60595706 | 143.1650826 |
| YGR234W | YHB1 | 0.74114241 | 0.99917121 | 0.07446505 | 145.592282 | 222.7577386 |
| YKL184W | SPE1 | 0.9095935 | 0.99917121 | 0.07456233 | 36.77848137 | 49.47476343 |
| YOR208W | PTP2 | 0.77875263 | 0.99917121 | 0.07468321 | 134.3352944 | 204.8059272 |
| YDR124W | YDR124W | 0.56393189 | 0.99917121 | 0.07470147 | 148.7606495 | 227.774108 |

| | | | | | | |
|---|---|---|---|---|---|---|
| YPR042C | PUF2 | 0.78912121 | 0.99917121 | 0.07477491 | 131.4472606 | 200.1959485 |
| YFL049W | SWP82 | 0.63208553 | 0.99917121 | 0.07479319 | 139.4356281 | 212.9141034 |
| YOR029W | YOR029W | 0.50897488 | 0.99917121 | 0.0748375 | 130.9350855 | 199.372749 |
| YLR433C | CNA1 | 0.66078097 | 0.99917121 | 0.07508904 | 135.0816978 | 205.9449966 |
| YBR208C | DUR1 | 0.52422677 | 0.99917121 | 0.07516331 | 145.1100932 | 221.9047931 |
| YDL192W | ARF1 | 0.70640193 | 0.99917121 | 0.07551306 | 111.9127461 | 168.9999137 |
| YER044C-A | MEI4 | 0.34622967 | 0.99917121 | 0.07551722 | 135.0531036 | 205.8472657 |
| YGL163C | RAD54 | 0.64107179 | 0.99917121 | 0.07561361 | 121.2807489 | 183.9049201 |
| YMR171C | YMR171C | 0.64814986 | 0.99917121 | 0.07561376 | 139.6019049 | 213.078841 |
| YJR010W | MET3 | 0.58130694 | 0.99917121 | 0.07562543 | 166.9637814 | 256.6474697 |
| YDR441C | APT2 | 0.69075863 | 0.99917121 | 0.07566857 | 139.7193835 | 213.2592278 |
| YMR048W | CSM3 | 0.51322315 | 0.99917121 | 0.07580333 | 129.1221483 | 196.3681488 |
| YEL008W | YEL008W | 0.65695324 | 0.99917121 | 0.07584541 | 143.5282289 | 219.3027351 |
| YBL059W | YBL059W | 0.47621407 | 0.99917121 | 0.07587091 | 140.4713578 | 214.4319762 |
| YOR177C | MPC54 | 0.70754672 | 0.99917121 | 0.07603296 | 145.8156127 | 222.9222163 |
| YNL013C | YNL013C | 0.63260347 | 0.99917121 | 0.07604334 | 127.1802586 | 193.2466955 |
| YOL082W | ATG19 | 0.68708104 | 0.99917121 | 0.07615416 | 135.4083989 | 206.3353751 |
| YPR146C | YPR146C | 0.75228774 | 0.99917121 | 0.07624564 | 144.654615 | 221.047559 |
| YAL011W | SWC3 | 0.71442184 | 0.99917121 | 0.07630676 | 135.678074 | 206.7461924 |
| YGL241W | KAP114 | 0.66316388 | 0.99917121 | 0.07632443 | 143.2287886 | 218.7675193 |
| YLR179C | YLR179C | 0.76057934 | 0.99917121 | 0.07641071 | 125.7629431 | 190.9450277 |
| YDL096C | YDL096C | 0.54987785 | 0.99917121 | 0.07644374 | 147.4874757 | 225.5343523 |
| YPR063C | YPR063C | 0.73496278 | 0.99917121 | 0.07648416 | 141.9607782 | 216.7289134 |
| YJL148W | RPA34 | 0.61003034 | 0.99917121 | 0.07653031 | 117.3862815 | 177.5917569 |
| YER081W | SER3 | 0.62170595 | 0.99917121 | 0.07656429 | 131.3287821 | 199.7891428 |
| YPR130C | YPR130C | 0.69762545 | 0.99917121 | 0.07657592 | 141.6520839 | 216.2261735 |
| YFR009W | GCN20 | 0.66188159 | 0.99917121 | 0.07662556 | 144.6457353 | 220.9871025 |
| YLL023C | YLL023C | 0.42126681 | 0.99917121 | 0.07664295 | 143.6916805 | 219.4657806 |
| YPR148C | YPR148C | 0.54041872 | 0.99917121 | 0.07670342 | 141.1845087 | 215.4660801 |
| YLR367W | RPS22B | 0.73767803 | 0.99917121 | 0.07671627 | 127.5481254 | 193.7504356 |
| YOR175C | YOR175C | 0.78132403 | 0.99917121 | 0.07685235 | 132.9617037 | 202.3542298 |
| YCR062W | YCR062W | 0.61050836 | 0.99917121 | 0.07696938 | 145.2229875 | 221.8643821 |
| YIL058W | YIL058W | 0.7600492 | 0.99917121 | 0.07697387 | 130.7395776 | 198.8009826 |
| YDR276C | PMP3 | 0.57850354 | 0.99917121 | 0.07701647 | 144.6866568 | 221.0046085 |
| YBL075C | SSA3 | 0.60600373 | 0.99917121 | 0.0770909 | 156.6143753 | 239.9887973 |
| YDR380W | ARO10 | 0.50767685 | 0.99917121 | 0.07713695 | 142.7991797 | 217.9843708 |
| YJR145C | RPS4A | 0.67221567 | 0.99917121 | 0.07736138 | 141.5000128 | 215.8882645 |
| YLR296W | YLR296W | 0.67374213 | 0.99917121 | 0.07736636 | 153.4568773 | 234.9273308 |
| YPL162C | YPL162C | 0.67794208 | 0.99917121 | 0.07741289 | 154.6145892 | 236.7651565 |
| YGL063W | PUS2 | 0.67141182 | 0.99917121 | 0.07752389 | 127.7942621 | 194.0439165 |
| YGR236C | SPG1 | 0.63848337 | 0.99917121 | 0.07753605 | 146.0295657 | 223.0796649 |
| YOR297C | TIM18 | 0.75746389 | 0.99917121 | 0.07758878 | 125.8403285 | 190.9246333 |
| YKL043W | PHD1 | 0.71022517 | 0.99917121 | 0.07765049 | 136.8494716 | 208.4476673 |
| YGL196W | YGL196W | 0.48446474 | 0.99917121 | 0.07766561 | 137.6867133 | 209.7790173 |
| YGR227W | DIE2 | 0.72405473 | 0.99917121 | 0.0776782 | 153.055388 | 234.2499981 |
| YAL055W | PEX22 | 0.57232417 | 0.99917121 | 0.07777361 | 163.1897915 | 250.3760193 |
| YNL212W | VID27 | 0.68031438 | 0.99917121 | 0.07777872 | 142.2283609 | 216.9971819 |
| YGR131W | YGR131W | 0.66931617 | 0.99917121 | 0.07777996 | 149.6906406 | 228.8796911 |
| YOL029C | YOL029C | 0.62354834 | 0.99917121 | 0.07786512 | 148.8797154 | 227.578022 |
| YPL206C | YPL206C | 0.52653898 | 0.99917121 | 0.07790096 | 138.4983473 | 211.0427422 |
| YGL205W | POX1 | 0.67116241 | 0.99917121 | 0.07792089 | 140.8985568 | 214.8623185 |
| YLR319C | BUD6 | 0.62675949 | 0.99917121 | 0.07799331 | 138.5954496 | 211.1861053 |
| YDR428C | YDR428C | 0.69140968 | 0.99917121 | 0.07800101 | 142.5879129 | 217.542619 |
| YIL123W | SIM1 | 0.75800877 | 0.99917121 | 0.07810193 | 159.3077203 | 244.1543256 |
| YPL072W | UBP16 | 0.74455077 | 0.99917121 | 0.07811402 | 136.2286887 | 207.4026463 |
| YKL007W | CAP1 | 0.68674558 | 0.99917121 | 0.07813263 | 134.6962909 | 204.9602429 |
| YIL088C | AVT7 | 0.70591992 | 0.99917121 | 0.07815155 | 137.5571806 | 209.5135132 |
| YFL026W | STE2 | 0.68530899 | 0.99917121 | 0.07816678 | 145.8041304 | 222.6437969 |
| YJR106W | ECM27 | 0.69772473 | 0.99917121 | 0.07821286 | 148.1421157 | 226.361102 |
| YHR137W | ARO9 | 0.63508722 | 0.99917121 | 0.07821515 | 142.2053184 | 216.9072836 |
| YBR209W | YBR209W | 0.69481112 | 0.99917121 | 0.07839161 | 132.1324609 | 200.8461217 |
| YPR058W | YMC1 | 0.75119995 | 0.99917121 | 0.07839781 | 133.1137646 | 202.4079577 |
| YGL015C | YGL015C | 0.70065617 | 0.99917121 | 0.07843243 | 149.058748 | 227.7939452 |
| YHR200W | RPN10 | 0.68035668 | 0.99917121 | 0.07850888 | 128.6262257 | 195.2486247 |

| | | | | | | |
|---|---|---|---|---|---|---|
| YFR054C | YFR054C | 0.6736819 | 0.99917121 | 0.07869646 | 154.8676098 | 237.0115768 |
| YBR293W | VBA2 | 0.62973095 | 0.99917121 | 0.07875988 | 155.0926224 | 237.3621471 |
| YOL153C | YOL153C | 0.74591108 | 0.99917121 | 0.07888038 | 134.4890537 | 204.539087 |
| YLR108C | YLR108C | 0.74671393 | 0.99917121 | 0.07895341 | 138.8769696 | 211.5173418 |
| YPR083W | MDM36 | 0.77001493 | 0.99917121 | 0.07896024 | 136.3286301 | 207.458626 |
| YJL092W | HPR5 | 0.81602781 | 0.99917121 | 0.07899221 | 131.6471249 | 200.0000709 |
| YDR132C | YDR132C | 0.41090753 | 0.99917121 | 0.07902119 | 139.541561 | 212.5673486 |
| YBL032W | HEK2 | 0.59266477 | 0.99917121 | 0.07902253 | 150.6007103 | 230.17737 |
| YNR051C | BRE5 | 0.62635105 | 0.99917121 | 0.07906135 | 122.4034474 | 185.2723483 |
| YPR151C | SUE1 | 0.75272856 | 0.99917121 | 0.0790792 | 131.6595658 | 200.0092762 |
| YLR287C | YLR287C | 0.67892745 | 0.99917121 | 0.07918925 | 148.3439925 | 226.5635305 |
| YPL229W | YPL229W | 0.6975741 | 0.99917121 | 0.07944591 | 139.9521655 | 213.1694029 |
| YDR239C | YDR239C | 0.52121552 | 0.99917121 | 0.07949509 | 140.3349164 | 213.7728852 |
| YDR203W | YDR203W | 0.51782076 | 0.99917121 | 0.07967934 | 137.5815614 | 209.3660824 |
| YBR057C | MUM2 | 0.69892557 | 0.99917121 | 0.07986763 | 141.2704363 | 215.2171573 |
| YDL174C | DLD1 | 0.51010779 | 0.99917121 | 0.07989882 | 135.601476 | 206.1863091 |
| YBR217W | ATG12 | 0.58720046 | 0.99917121 | 0.0799092 | 146.3495445 | 223.2998754 |
| YHR029C | YHI9 | 0.6853624 | 0.99917121 | 0.07999505 | 156.4080296 | 239.3061728 |
| YFR046C | CNN1 | 0.60798189 | 0.99917121 | 0.08003657 | 147.3060142 | 224.8073949 |
| YER033C | ZRG8 | 0.68510353 | 0.99917121 | 0.08004907 | 147.6573918 | 225.3653922 |
| YNL031C | HHT2 | 0.63475798 | 0.99917121 | 0.08010632 | 134.9779472 | 205.1681287 |
| YCR101C | YCR101C | 0.38190136 | 0.99917121 | 0.08018421 | 141.2943968 | 215.2167163 |
| YLR091W | YLR091W | 0.69091976 | 0.99917121 | 0.08024172 | 141.2783002 | 215.1840738 |
| YKL031W | YKL031W | 0.57777109 | 0.99917121 | 0.08028486 | 141.2571571 | 215.1451472 |
| YBL003C | HTA2 | 0.53032486 | 0.99917121 | 0.08042832 | 146.8617835 | 224.0522597 |
| YDL053C | PBP4 | 0.60681959 | 0.99917121 | 0.08063218 | 153.37845 | 234.4043077 |
| YDL243C | AAD4 | 0.55234111 | 0.99917121 | 0.08073781 | 150.4677007 | 229.75646 |
| YBL068W | PRS4 | 0.57008582 | 0.99917121 | 0.08075863 | 150.2703054 | 229.4395967 |
| YBR151W | APD1 | 0.59109687 | 0.99917121 | 0.08077679 | 149.8728472 | 228.8044849 |
| YOR014W | RTS1 | 0.56522391 | 0.99917121 | 0.08078279 | 129.4441447 | 196.273835 |
| YPR020W | ATP20 | 0.74884159 | 0.99917121 | 0.08085592 | 118.7514836 | 179.2383173 |
| YBR180W | DTR1 | 0.43598273 | 0.99917121 | 0.08088712 | 149.0064622 | 227.4114347 |
| YLR348C | DIC1 | 0.68559073 | 0.99917121 | 0.08090534 | 139.6058656 | 212.4400468 |
| YMR159C | ATG16 | 0.60575396 | 0.99917121 | 0.08093469 | 132.8535868 | 201.6843877 |
| YAR002W | NUP60 | 0.76508851 | 0.99917121 | 0.08141507 | 142.1228105 | 216.385797 |
| YDR222W | YDR222W | 0.40859249 | 0.99917121 | 0.08161619 | 151.041236 | 230.5626525 |
| YLR225C | YLR225C | 0.72453546 | 0.99917121 | 0.08165873 | 139.5767703 | 212.3018711 |
| YCL047C | YCL047C | 0.50887896 | 0.99917121 | 0.08168582 | 153.9142746 | 235.1290852 |
| YDR094W | YDR094W | 0.61340816 | 0.99917121 | 0.08170533 | 153.9604657 | 235.2002604 |
| YLL032C | YLL032C | 0.60696126 | 0.99917121 | 0.08183209 | 143.8600216 | 219.1012296 |
| YHR049C-A | YHR049C-A | 0.67307925 | 0.99917121 | 0.08184953 | 148.2769359 | 226.1324353 |
| YER069W | ARG5 | 0.38204595 | 0.99917121 | 0.08186706 | 133.1188619 | 201.9931363 |
| YJL004C | SYS1 | 0.37673683 | 0.99917121 | 0.08187476 | 134.2106879 | 203.7307813 |
| YNR030W | ALG12 | 0.52298771 | 0.99917121 | 0.08190486 | 131.4923609 | 199.3985473 |
| YIL074C | SER33 | 0.66402884 | 0.99917121 | 0.08194939 | 166.4817974 | 255.1090175 |
| YER007W | PAC2 | 0.46491085 | 0.99917121 | 0.08237096 | 153.662475 | 234.6446028 |
| YJL029C | VPS53 | 0.76787422 | 0.99917121 | 0.08240943 | 113.5297383 | 170.7340114 |
| YBR061C | TRM7 | 0.51792504 | 0.99917121 | 0.08244714 | 142.3913424 | 216.6875766 |
| YOR235W | YOR235W | 0.66328313 | 0.99917121 | 0.08249802 | 120.5959478 | 181.9751846 |
| YBR027C | YBR027C | 0.5411679 | 0.99917121 | 0.08274299 | 145.4567077 | 221.532685 |
| YDR410C | STE14 | 0.43786688 | 0.99917121 | 0.0828466 | 152.2476648 | 232.3337254 |
| YMR294W | JNM1 | 0.61659 | 0.99917121 | 0.08292782 | 123.0493317 | 185.8294666 |
| YPR134W | MSS18 | 0.7489279 | 0.99917121 | 0.08308222 | 119.6378917 | 180.3783916 |
| YGL251C | HFM1 | 0.58662326 | 0.99917121 | 0.08308879 | 153.029054 | 233.5484552 |
| YCR001W | YCR001W | 0.63423293 | 0.99917121 | 0.08321117 | 153.0430391 | 233.5558052 |
| YLR351C | NIT3 | 0.71884645 | 0.99917121 | 0.08332751 | 135.593731 | 205.7559831 |
| YMR189W | GCV2 | 0.64376606 | 0.99917121 | 0.08333193 | 141.2533648 | 214.7676374 |
| YJL135W | YJL135W | 0.67828964 | 0.99917121 | 0.08338153 | 140.4691346 | 213.5128117 |
| YDL118W | YDL118W | 0.68645404 | 0.99917121 | 0.08340865 | 118.8442249 | 179.0747902 |
| YLR120C | YPS1 | 0.68257555 | 0.99917121 | 0.08349701 | 134.3582046 | 203.7679116 |
| YML068W | ITT1 | 0.45220474 | 0.99917121 | 0.0835348 | 126.3598151 | 191.0269613 |
| YLR361C | DCR2 | 0.60847632 | 0.99917121 | 0.08369555 | 123.0143017 | 185.6800916 |
| YBR244W | GPX2 | 0.6113906 | 0.99917121 | 0.08373868 | 149.7435828 | 228.2375635 |
| YOR308C | SNU66 | 0.59166027 | 0.99917121 | 0.08384337 | 132.6403457 | 200.9902306 |

| | | | | | | |
|---|---|---|---|---|---|---|
| YML071C | COG8 | 0.42841504 | 0.99917121 | 0.08385551 | 126.1076773 | 190.5863687 |
| YDL059C | RAD59 | 0.58328751 | 0.99917121 | 0.08387242 | 144.2076773 | 219.4060858 |
| YDR025W | RPS11A | 0.53883054 | 0.99917121 | 0.08388479 | 121.2481441 | 182.8446565 |
| YDL124W | YDL124W | 0.54143656 | 0.99917121 | 0.08392349 | 149.5993572 | 227.985373 |
| YGR023W | MTL1 | 0.64153074 | 0.99917121 | 0.08403513 | 149.3824609 | 227.626385 |
| YJL070C | YJL070C | 0.33886956 | 0.99917121 | 0.08406441 | 123.7760243 | 186.8480627 |
| YDR192C | NUP42 | 0.43600815 | 0.99917121 | 0.08407546 | 142.9202899 | 217.331344 |
| YLR267W | BOP2 | 0.62796153 | 0.99917121 | 0.08409192 | 142.9682054 | 217.4056359 |
| YBR146W | MRPS9 | 0.45414658 | 0.99917121 | 0.08416326 | 150.6947836 | 229.700459 |
| YKR097W | PCK1 | 0.73738114 | 0.99917121 | 0.08424894 | 110.1693401 | 165.1587807 |
| YAL031C | GIP4 | 0.55858437 | 0.99917121 | 0.08430262 | 146.0667507 | 222.3139591 |
| YMR316C-B | YMR316C-B | 0.66966628 | 0.99917121 | 0.08455655 | 146.89013 | 223.5941219 |
| YPR090W | YPR090W | 0.58030502 | 0.99917121 | 0.08456423 | 135.7187506 | 205.8042902 |
| YNL292W | PUS4 | 0.49109262 | 0.99917121 | 0.08460866 | 136.4008449 | 206.8850158 |
| YAL045C | YAL045C | 0.54495284 | 0.99917121 | 0.08461949 | 148.296018 | 225.8251339 |
| YKL160W | ELF1 | 0.46271022 | 0.99917121 | 0.08495973 | 134.1973884 | 203.333513 |
| YJL108C | PRM10 | 0.46465663 | 0.99917121 | 0.08504071 | 152.4517769 | 232.3912612 |
| YNL224C | SQS1 | 0.68968699 | 0.99917121 | 0.08505701 | 128.5937028 | 194.3985491 |
| YDR383C | NKP1 | 0.36473062 | 0.99917121 | 0.08512567 | 143.9571332 | 218.854343 |
| YOL080C | REX4 | 0.5581274 | 0.99917121 | 0.08515183 | 132.4767268 | 200.5701751 |
| YJL064W | YJL064W | 0.61985632 | 0.99917121 | 0.08539361 | 133.2304039 | 201.7408276 |
| YBL078C | ATG8 | 0.59695035 | 0.99917121 | 0.08553435 | 141.8495184 | 215.4484324 |
| YJR075W | HOC1 | 0.79142367 | 0.99917121 | 0.08557811 | 138.922073 | 210.7815403 |
| YNR066C | YNR066C | 0.64067374 | 0.99917121 | 0.08563454 | 156.3302054 | 238.4947344 |
| YGL228W | SHE10 | 0.62019493 | 0.99917121 | 0.08573116 | 149.4475607 | 227.5232841 |
| YFR040W | SAP155 | 0.4957908 | 0.99917121 | 0.08580102 | 124.6143152 | 187.9712154 |
| YDR340W | YDR340W | 0.54032809 | 0.99917121 | 0.08587427 | 144.8348861 | 220.1607825 |
| YLR341W | SPO77 | 0.67625688 | 0.99917121 | 0.08592424 | 137.4752643 | 208.4354982 |
| YLR182W | SWI6 | 0.78474401 | 0.99917121 | 0.08610502 | 43.15698552 | 58.22448181 |
| YFR055W | IRC7 | 0.58938291 | 0.99917121 | 0.08615385 | 145.4888108 | 221.1679842 |
| YMR138W | CIN4 | 0.38477585 | 0.99917121 | 0.08622469 | 125.4591309 | 189.264819 |
| YDR119W | YDR119W | 0.4834968 | 0.99917121 | 0.08629285 | 145.3850693 | 220.9858446 |
| YGR037C | ACB1 | 0.62613328 | 0.99917121 | 0.08643564 | 156.266074 | 238.2949507 |
| YER080W | FMP29 | 0.68868154 | 0.99917121 | 0.08645478 | 152.2979554 | 231.9739312 |
| YOR137C | SIA1 | 0.63574764 | 0.99917121 | 0.08650362 | 160.7281164 | 245.3918566 |
| YMR026C | PEX12 | 0.53464235 | 0.99917121 | 0.08660392 | 122.5776441 | 184.6302127 |
| YCL028W | RNQ1 | 0.64011253 | 0.99917121 | 0.08660758 | 156.0388007 | 237.9120887 |
| YER114C | BOI2 | 0.69834827 | 0.99917121 | 0.08662076 | 140.0001522 | 212.3711244 |
| YER079W | YER079W | 0.66586089 | 0.99917121 | 0.08664647 | 139.3290903 | 211.2994151 |
| YDR286C | YDR286C | 0.44558151 | 0.99917121 | 0.08665374 | 139.2063556 | 211.1030912 |
| YPL198W | RPL7B | 0.75339721 | 0.99917121 | 0.08690199 | 140.3685229 | 212.9234187 |
| YOL015W | IRC10 | 0.73579705 | 0.99917121 | 0.08706041 | 155.4732371 | 236.9563002 |
| YNL266W | YNL266W | 0.65506306 | 0.99917121 | 0.08710278 | 141.0277315 | 213.9486408 |
| YIL020C | HIS6 | 0.7550733 | 0.99917121 | 0.087239 | 123.7630147 | 186.4403297 |
| YGR201C | YGR201C | 0.52276519 | 0.99917121 | 0.08739555 | 153.7492461 | 234.1702228 |
| YLR353W | BUD8 | 0.66510738 | 0.99917121 | 0.08741456 | 141.89483 | 215.2913669 |
| YGL248W | PDE1 | 0.6055926 | 0.99917121 | 0.08749322 | 154.5367737 | 235.412346 |
| YLR297W | YLR297W | 0.66525715 | 0.99917121 | 0.08756295 | 144.5392445 | 219.4841459 |
| YGR135W | PRE9 | 0.59996264 | 0.99917121 | 0.08756387 | 114.8321933 | 172.1796124 |
| YLR017W | MEU1 | 0.61225601 | 0.99917121 | 0.08767607 | 148.0689973 | 225.0910042 |
| YLR327C | TMA10 | 0.65666295 | 0.99917121 | 0.08793117 | 141.0628136 | 213.9035144 |
| YBR130C | SHE3 | 0.61107162 | 0.99917121 | 0.08806589 | 145.9850049 | 221.7250074 |
| YBL037W | APL3 | 0.59448169 | 0.99917121 | 0.08818272 | 167.2683207 | 255.6015381 |
| YOR265W | RBL2 | 0.57093326 | 0.99917121 | 0.08842848 | 136.2552849 | 206.1875546 |
| YGR039W | YGR039W | 0.55692451 | 0.99917121 | 0.08848295 | 151.2651487 | 230.0820715 |
| YDR142C | PEX7 | 0.40457534 | 0.99917121 | 0.08861645 | 143.7002118 | 218.0196673 |
| YDL232W | OST4 | 0.79541269 | 0.99917121 | 0.08888709 | 116.4676057 | 174.6224677 |
| YBR158W | AMN1 | 0.55008106 | 0.99917121 | 0.08916994 | 149.8987729 | 227.8225536 |
| YNL318C | HXT14 | 0.6423386 | 0.99917121 | 0.0893343 | 139.7763869 | 211.6839991 |
| YKL027W | YKL027W | 0.35941584 | 0.99917121 | 0.08946821 | 133.1924863 | 201.1837113 |
| YGR109C | CLB6 | 0.6326134 | 0.99917121 | 0.08950281 | 151.6229873 | 230.5275484 |
| YDR391C | YDR391C | 0.3592909 | 0.99917121 | 0.08950344 | 147.3101626 | 223.6598883 |
| YGR152C | RSR1 | 0.57844425 | 0.99917121 | 0.08956517 | 159.8147308 | 243.5641791 |
| YOR120W | GCY1 | 0.57920945 | 0.99917121 | 0.08968575 | 138.7630139 | 210.0274956 |

| | | | | | | |
|---|---|---|---|---|---|---|
| YJR014W | TMA22 | 0.64089142 | 0.99917121 | 0.08976843 | 146.1098121 | 221.7161885 |
| YNL057W | YNL057W | 0.44813077 | 0.99917121 | 0.08984638 | 129.4136263 | 195.12029 |
| YDL138W | RGT2 | 0.66009646 | 0.99917121 | 0.08997187 | 151.5882473 | 230.4150466 |
| YMR070W | MOT3 | 0.51497366 | 0.99917121 | 0.09005826 | 142.2510548 | 215.5363105 |
| YJR146W | YJR146W | 0.55853076 | 0.99917121 | 0.09016935 | 148.7858337 | 225.9285114 |
| YOL024W | YOL024W | 0.62410001 | 0.99917121 | 0.09018146 | 145.0566444 | 219.9888093 |
| YBR077C | SLM4 | 0.60740527 | 0.99917121 | 0.09020071 | 154.4752005 | 234.9842278 |
| YJL023C | PET130 | 0.13725612 | 0.97853666 | 0.09034313 | 141.8654353 | 214.887536 |
| YHR025W | THR1 | 0.91180525 | 0.99917121 | 0.09050787 | 62.71167354 | 88.82589775 |
| YBR183W | YPC1 | 0.58163107 | 0.99917121 | 0.09052522 | 148.8526043 | 225.99145 |
| YMR280C | CAT8 | 0.64357714 | 0.99917121 | 0.09076699 | 137.702999 | 208.2077521 |
| YOL008W | COQ10 | 0.37920502 | 0.99917121 | 0.0907753 | 110.7180068 | 165.2368244 |
| YOL035C | YOL035C | 0.55834554 | 0.99917121 | 0.09087545 | 132.0030397 | 199.1181219 |
| YKR035W-A | DID2 | 0.20718801 | 0.99917121 | 0.09091855 | 124.9123497 | 187.8219129 |
| YGR275W | RTT102 | 0.6038836 | 0.99917121 | 0.09093074 | 155.4149608 | 236.3916686 |
| YOL114C | YOL114C | 0.44763702 | 0.99917121 | 0.09098359 | 131.0090226 | 197.5221032 |
| YDL125C | HNT1 | 0.49517126 | 0.99917121 | 0.09113246 | 147.3642994 | 223.5474977 |
| YLR254C | NDL1 | 0.63282028 | 0.99917121 | 0.09117523 | 137.1478479 | 207.2739802 |
| YML070W | DAK1 | 0.62521199 | 0.99917121 | 0.09131885 | 138.0740394 | 208.7313051 |
| YIR044C | YIR044C | 0.56312473 | 0.99917121 | 0.09146017 | 145.790993 | 221.0022716 |
| YDL085W | NDE2 | 0.53206831 | 0.99917121 | 0.09154146 | 157.2560448 | 239.2488907 |
| YDR111C | ALT2 | 0.55746869 | 0.99917121 | 0.09160223 | 145.5099661 | 220.5374555 |
| YDR089W | YDR089W | 0.46096733 | 0.99917121 | 0.09177942 | 141.3361803 | 213.8696704 |
| YLR405W | DUS4 | 0.61803878 | 0.99917121 | 0.09190769 | 145.2077624 | 220.0189982 |
| YAL067C | SEO1 | 0.59939772 | 0.99917121 | 0.09194477 | 146.6688019 | 222.340984 |
| YPR141C | KAR3 | 0.78532029 | 0.99917121 | 0.09208004 | 29.90095467 | 36.38764455 |
| YOL071W | EMI5 | 0.18875097 | 0.99917121 | 0.0921067 | 122.4871436 | 183.8152551 |
| YLR205C | HMX1 | 0.67290158 | 0.99917121 | 0.09213578 | 138.191089 | 208.8180977 |
| YGL051W | MST27 | 0.561006 | 0.99917121 | 0.09233415 | 157.709334 | 239.8740543 |
| YMR031W-A | YMR031W-A | 0.70916273 | 0.99917121 | 0.09234019 | 110.2363399 | 164.2790585 |
| YLL055W | YLL055W | 0.66870729 | 0.99917121 | 0.09237156 | 137.0454222 | 206.9650355 |
| YOR365C | YOR365C | 0.48172466 | 0.99917121 | 0.09251947 | 136.3564098 | 205.8498465 |
| YBR214W | SDS24 | 0.36335288 | 0.99917121 | 0.09301114 | 143.4204801 | 217.0384731 |
| YLR049C | YLR049C | 0.3517176 | 0.99917121 | 0.09304946 | 123.9511585 | 186.0315665 |
| YGL090W | LIF1 | 0.40548615 | 0.99917121 | 0.09320846 | 149.8646899 | 227.2759418 |
| YDR130C | FIN1 | 0.42383253 | 0.99917121 | 0.09335159 | 144.6288617 | 218.9211522 |
| YKR094C | RPL40B | 0.63201443 | 0.99917121 | 0.0934008 | 120.7360725 | 180.8691488 |
| YEL048C | YEL048C | 0.51908189 | 0.99917121 | 0.0934333 | 143.2314669 | 216.6860301 |
| YMR160W | YMR160W | 0.56532844 | 0.99917121 | 0.09367215 | 133.8875969 | 201.778075 |
| YGR166W | KRE11 | 0.57656408 | 0.99917121 | 0.09374585 | 148.4312474 | 224.9278673 |
| YNL136W | EAF7 | 0.70665022 | 0.99917121 | 0.09412483 | 104.1934179 | 154.438964 |
| YOR170W | YOR170W | 0.6224023 | 0.99917121 | 0.0942066 | 138.5722752 | 209.17263 |
| YGR138C | TPO2 | 0.48785801 | 0.99917121 | 0.09426454 | 159.3286724 | 242.2172936 |
| YPR024W | YME1 | 0.33891655 | 0.99917121 | 0.09439881 | 95.33515826 | 140.2999938 |
| YGL028C | SCW11 | 0.5939028 | 0.99917121 | 0.09474352 | 148.0348094 | 224.1749673 |
| YMR011W | HXT2 | 0.5042003 | 0.99917121 | 0.09513973 | 141.3824994 | 213.5337693 |
| YGL114W | YGL114W | 0.41921299 | 0.99917121 | 0.09519959 | 147.0118835 | 222.490497 |
| YGR199W | PMT6 | 0.63663875 | 0.99917121 | 0.09520738 | 147.2261894 | 222.8308009 |
| YOL103W | ITR2 | 0.65503697 | 0.99917121 | 0.0954105 | 134.1466251 | 201.9786182 |
| YBR300C | YBR300C | 0.62106263 | 0.99917121 | 0.09548489 | 151.7771941 | 230.0438223 |
| YAR002C-A | ERP1 | 0.46182216 | 0.99917121 | 0.09552058 | 144.9632105 | 219.1891337 |
| YBR162W-A | YSY6 | 0.73294462 | 0.99917121 | 0.09559663 | 156.4373576 | 237.4508742 |
| YNL042W | BOP3 | 0.53526321 | 0.99917121 | 0.09563908 | 132.675485 | 199.6081623 |
| YEL063C | CAN1 | 0.61434097 | 0.99917121 | 0.09568064 | 138.3408241 | 208.6243751 |
| YBR269C | FMP21 | 0.39743282 | 0.99917121 | 0.09588689 | 127.9118428 | 191.9925027 |
| YER115C | SPR6 | 0.6599345 | 0.99917121 | 0.09590814 | 133.5987751 | 201.0455745 |
| YBR139W | YBR139W | 0.55100238 | 0.99917121 | 0.09600226 | 153.0304997 | 231.9764678 |
| YDR374C | YDR374C | 0.78163035 | 0.99917121 | 0.09600489 | 144.7132883 | 218.7321233 |
| YOR380W | RDR1 | 0.52195178 | 0.99917121 | 0.09644468 | 144.3332364 | 218.0733277 |
| YOR144C | ELG1 | 0.58545088 | 0.99917121 | 0.09645649 | 133.3745264 | 200.6216387 |
| YDR465C | RMT2 | 0.61745687 | 0.99917121 | 0.09667047 | 143.2572142 | 216.3323831 |
| YAL023C | PMT2 | 0.61353211 | 0.99917121 | 0.09712798 | 145.5287052 | 219.893647 |
| YER167W | BCK2 | 0.45261377 | 0.99917121 | 0.0971889 | 153.1067289 | 231.9531878 |
| YOL020W | TAT2 | 0.58829296 | 0.99917121 | 0.09720813 | 134.8396838 | 202.8630695 |

| | | | | | | |
|---|---|---|---|---|---|---|
| YDR493W | FMP36 | 0.68066659 | 0.99917121 | 0.09727804 | 140.1891227 | 211.3727976 |
| YNL011C | YNL011C | 0.1679687 | 0.99636201 | 0.09728236 | 137.2439721 | 206.6825207 |
| YGL230C | YGL230C | 0.49579313 | 0.99917121 | 0.09762628 | 143.3896201 | 216.4266979 |
| YPR023C | EAF3 | 0.69997137 | 0.99917121 | 0.09763799 | 123.3818055 | 184.5655591 |
| YLR062C | BUD28 | 0.39596712 | 0.99917121 | 0.09781432 | 130.5483284 | 195.9557705 |
| YPR188C | MLC2 | 0.57854648 | 0.99917121 | 0.09790636 | 134.7692657 | 202.6658164 |
| YHR003C | YHR003C | 0.68043919 | 0.99917121 | 0.09809982 | 149.7773989 | 226.5406339 |
| YGL249W | ZIP2 | 0.58022239 | 0.99917121 | 0.09820756 | 153.9423945 | 233.1596861 |
| YLR318W | EST2 | 0.81901451 | 0.99917121 | 0.09840322 | 32.10999305 | 39.1343737 |
| YLR001C | YLR001C | 0.56938063 | 0.99917121 | 0.09849402 | 139.5203823 | 210.1596784 |
| YGR127W | YGR127W | 0.52647292 | 0.99917121 | 0.09850387 | 159.4692935 | 241.9243947 |
| YGL256W | ADH4 | 0.42464883 | 0.99917121 | 0.09855311 | 146.2094014 | 220.8038246 |
| YPL261C | YPL261C | 0.67411125 | 0.99917121 | 0.09856412 | 134.672001 | 202.4307482 |
| YMR251W-A | HOR7 | 0.57417699 | 0.99917121 | 0.09884492 | 147.3251555 | 222.5449357 |
| YKL041W | VPS24 | 0.33034558 | 0.99917121 | 0.09891531 | 100.2231051 | 147.5327727 |
| YJR100C | YJR100C | 0.76234751 | 0.99917121 | 0.09900157 | 141.0654544 | 212.5581186 |
| YML116W | ATR1 | 0.57198844 | 0.99917121 | 0.099122 | 135.9102536 | 204.3344845 |
| YJR154W | YJR154W | 0.09672564 | 0.94753174 | 0.09918571 | 137.9289129 | 207.5411566 |
| YLR011W | LOT6 | 0.57946503 | 0.99917121 | 0.09943918 | 143.495665 | 216.3745484 |
| YBR264C | YPT10 | 0.60196558 | 0.99917121 | 0.0995118 | 158.9904891 | 241.0390866 |
| YKL055C | OAR1 | 0.66120514 | 0.99917121 | 0.09994912 | 115.4851096 | 171.7093969 |
| YLR135W | SLX4 | 0.66515485 | 0.99917121 | 0.09996921 | 131.5543569 | 197.2950302 |
| YDR490C | PKH1 | 0.50687266 | 0.99917121 | 0.10006349 | 143.3819355 | 216.1173393 |
| YNL268W | LYP1 | 0.2226003 | 0.99917121 | 0.10010558 | 138.1508762 | 207.7824615 |
| YOR153W | PDR5 | 0.63604755 | 0.99917121 | 0.1002685 | 150.9535577 | 228.1491206 |
| YLR124W | YLR124W | 0.58701174 | 0.99917121 | 0.10030571 | 148.4327981 | 224.1306194 |
| YLL060C | GTT2 | 0.57568868 | 0.99917121 | 0.10052275 | 140.2361824 | 211.0521682 |
| YNR021W | YNR021W | 0.72433025 | 0.99917121 | 0.10060101 | 144.9732308 | 218.5857306 |
| YEL007W | YEL007W | 0.80395469 | 0.99917121 | 0.10060404 | 95.87805642 | 140.408002 |
| YGL108C | YGL108C | 0.56302793 | 0.99917121 | 0.10067979 | 156.8749886 | 237.5280506 |
| YKR012C | YKR012C | 0.73780589 | 0.99917121 | 0.10074769 | 129.4838803 | 193.9031737 |
| YLR151C | PCD1 | 0.61921385 | 0.99917121 | 0.10078082 | 141.6389232 | 213.2543801 |
| YBL095W | YBL095W | 0.43286885 | 0.99917121 | 0.1008017 | 144.0794117 | 217.1379799 |
| YGL229C | SAP4 | 0.49306204 | 0.99917121 | 0.1009101 | 152.0651601 | 229.8409785 |
| YDR229W | IVY1 | 0.46876314 | 0.99917121 | 0.10155349 | 146.0125687 | 220.1246161 |
| YEL028W | YEL028W | 0.40428719 | 0.99917121 | 0.10204316 | 135.2093048 | 202.8621983 |
| YGL180W | ATG1 | 0.4990687 | 0.99917121 | 0.10216933 | 156.8899974 | 237.3703595 |
| YFL028C | CAF16 | 0.61625266 | 0.99917121 | 0.10221635 | 137.906167 | 207.1354691 |
| YLR092W | SUL2 | 0.61395434 | 0.99917121 | 0.10229561 | 136.4223677 | 204.7630593 |
| YNL303W | YNL303W | 0.58639417 | 0.99917121 | 0.10235764 | 142.6071763 | 214.6039596 |
| YLR003C | YLR003C | 0.62629475 | 0.99917121 | 0.10239899 | 138.9363515 | 208.7536318 |
| YBR276C | PPS1 | 0.37600147 | 0.99917121 | 0.10267937 | 140.8253509 | 211.7274245 |
| YBL087C | RPL23A | 0.44697877 | 0.99917121 | 0.1028241 | 107.2678231 | 158.2740004 |
| YBR162C | TOS1 | 0.50995684 | 0.99917121 | 0.10288492 | 148.2393783 | 223.5081916 |
| YOL059W | GPD2 | 0.62535708 | 0.99917121 | 0.10289086 | 146.0185547 | 219.9711094 |
| YAR030C | YAR030C | 0.37009944 | 0.99917121 | 0.10293775 | 149.3812105 | 225.3199624 |
| YEL020C | YEL020C | 0.59692262 | 0.99917121 | 0.10299027 | 139.3515291 | 209.3426627 |
| YBR240C | THI2 | 0.38275544 | 0.99917121 | 0.10311127 | 146.922303 | 221.3833346 |
| YFL004W | VTC2 | 0.46972664 | 0.99917121 | 0.103164 | 149.3284242 | 225.2083249 |
| YAL002W | VPS8 | 0.70315043 | 0.99917121 | 0.10342989 | 113.1418501 | 167.5537333 |
| YJL094C | KHA1 | 0.15400893 | 0.98492771 | 0.10367392 | 134.4988759 | 201.5321303 |
| YOL045W | PSK2 | 0.48307879 | 0.99917121 | 0.1038064 | 136.7127906 | 205.0413371 |
| YLR391W | YLR391W | 0.59996038 | 0.99917121 | 0.10387897 | 137.8040256 | 206.7701324 |
| YCR045C | YCR045C | 0.43644305 | 0.99917121 | 0.10394473 | 129.2935762 | 193.210387 |
| YLR072W | YLR072W | 0.63799009 | 0.99917121 | 0.10399134 | 116.9850323 | 173.6050297 |
| YML010C-B | YML010C-B | 0.68328749 | 0.99917121 | 0.10425499 | 115.3742733 | 171.0079747 |
| YML100W | TSL1 | 0.27290881 | 0.99917121 | 0.10425529 | 135.2216913 | 202.6122395 |
| YDL233W | YDL233W | 0.41822083 | 0.99917121 | 0.10429271 | 153.2994772 | 231.3940836 |
| YER161C | SPT2 | 0.4712857 | 0.99917121 | 0.10436518 | 141.8449889 | 213.1455407 |
| YPR015C | YPR015C | 0.48811083 | 0.99917121 | 0.10442305 | 141.639744 | 212.8116604 |
| YOL042W | NGL1 | 0.49612555 | 0.99917121 | 0.1046207 | 143.2724056 | 215.3873557 |
| YLR036C | YLR036C | 0.66401698 | 0.99917121 | 0.10475447 | 133.1067921 | 199.1836965 |
| YCL037C | SRO9 | 0.49402528 | 0.99917121 | 0.10484309 | 116.9731583 | 173.4822845 |
| YLR152C | YLR152C | 0.55817283 | 0.99917121 | 0.10509395 | 144.3967952 | 217.1200976 |

| | | | | | | |
|---|---|---|---|---|---|---|
| YOL011W | PLB3 | 0.48362512 | 0.99917121 | 0.10525779 | 135.8267395 | 203.4534815 |
| YOL012C | HTZ1 | 0.51885125 | 0.99917121 | 0.10532288 | 128.531734 | 191.8292462 |
| YGL139W | FLC3 | 0.58950091 | 0.99917121 | 0.10541646 | 151.1228719 | 227.7911394 |
| YGL085W | YGL085W | 0.77171565 | 0.99917121 | 0.10552373 | 128.0852892 | 191.0938576 |
| YML020W | YML020W | 0.49147631 | 0.99917121 | 0.1055293 | 136.5513318 | 204.5741957 |
| YJL186W | MNN5 | 0.41988758 | 0.99917121 | 0.10566387 | 145.9650673 | 219.5478782 |
| YOR350C | MNE1 | 0.22704475 | 0.99917121 | 0.10567961 | 116.9054613 | 173.2725061 |
| YNL115C | YNL115C | 0.4981029 | 0.99917121 | 0.10578389 | 132.1711359 | 197.568295 |
| YKL130C | SHE2 | 0.56545218 | 0.99917121 | 0.10586072 | 145.9767393 | 219.5424657 |
| YOR045W | TOM6 | 0.4279827 | 0.99917121 | 0.10621287 | 133.2919642 | 199.3007635 |
| YBR241C | YBR241C | 0.4792941 | 0.99917121 | 0.10626878 | 154.3277039 | 232.7904893 |
| YLR408C | YLR408C | 0.59253086 | 0.99917121 | 0.10635092 | 139.5507488 | 209.2501928 |
| YGR173W | RBG2 | 0.53519675 | 0.99917121 | 0.10636775 | 153.3757175 | 231.2625155 |
| YOR053W | YOR053W | 0.53692725 | 0.99917121 | 0.1066754 | 131.1677599 | 195.8618703 |
| YOR034C | AKR2 | 0.40918681 | 0.99917121 | 0.10667592 | 135.446724 | 202.6754727 |
| YLR130C | ZRT2 | 0.62699498 | 0.99917121 | 0.10671964 | 142.4465294 | 213.8163773 |
| YMR130W | YMR130W | 0.39288569 | 0.99917121 | 0.10688042 | 151.0324792 | 227.4687284 |
| YBL028C | YBL028C | 0.50346125 | 0.99917121 | 0.107079 | 147.0292164 | 221.0698707 |
| YBR277C | YBR277C | 0.46887873 | 0.99917121 | 0.10725684 | 119.6182029 | 177.3998943 |
| YMR207C | HFA1 | 0.53345405 | 0.99917121 | 0.10727585 | 115.2953696 | 170.5140551 |
| YKL191W | DPH2 | 0.55502792 | 0.99917121 | 0.10741095 | 135.5871441 | 202.8094654 |
| YBL082C | ALG3 | 0.467946 | 0.99917121 | 0.10750862 | 142.4430357 | 213.7146285 |
| YAR014C | BUD14 | 0.46116663 | 0.99917121 | 0.10785209 | 123.8758028 | 184.1069744 |
| YOL136C | PFK27 | 0.65850074 | 0.99917121 | 0.10793478 | 145.2921162 | 218.1994475 |
| YDL168W | SFA1 | 0.36782277 | 0.99917121 | 0.10811474 | 146.8828496 | 220.7105332 |
| YBL101C | ECM21 | 0.4496347 | 0.99917121 | 0.10833722 | 148.0576245 | 222.5540797 |
| YER027C | GAL83 | 0.41645412 | 0.99917121 | 0.10850294 | 128.5515172 | 191.4730649 |
| YDR494W | RSM28 | 0.55711794 | 0.99917121 | 0.10859894 | 149.5472126 | 224.8941389 |
| YNL293W | MSB3 | 0.50656248 | 0.99917121 | 0.10864322 | 150.5547888 | 226.4931678 |
| YPR109W | YPR109W | 0.67784923 | 0.99917121 | 0.10873245 | 131.2681626 | 195.7709717 |
| YOR277C | YOR277C | 0.45435793 | 0.99917121 | 0.10880232 | 134.8667841 | 201.4927668 |
| YPL270W | MDL2 | 0.65764792 | 0.99917121 | 0.10880408 | 130.4984276 | 194.5365419 |
| YMR209C | YMR209C | 0.30106794 | 0.99917121 | 0.10880661 | 146.4056416 | 219.8662986 |
| YJL049W | YJL049W | 0.38621998 | 0.99917121 | 0.10883313 | 141.020921 | 211.2886342 |
| YLR169W | YLR169W | 0.60210982 | 0.99917121 | 0.10893374 | 145.2015249 | 217.9334094 |
| YDL081C | RPP1A | 0.40782981 | 0.99917121 | 0.10896087 | 91.52770313 | 132.4618708 |
| YGL034C | YGL034C | 0.37995456 | 0.99917121 | 0.1090062 | 149.9275485 | 225.4501229 |
| YMR199W | CLN1 | 0.54688108 | 0.99917121 | 0.1090488 | 136.4256353 | 203.944976 |
| YLR251W | SYM1 | 0.55961073 | 0.99917121 | 0.10906608 | 135.3486217 | 202.2278728 |
| YPR079W | MRL1 | 0.5489932 | 0.99917121 | 0.10921607 | 132.8985896 | 198.3082462 |
| YMR156C | TPP1 | 0.34505984 | 0.99917121 | 0.10929931 | 130.5832001 | 194.6111558 |
| YLR248W | RCK2 | 0.59011278 | 0.99917121 | 0.10940498 | 139.9002079 | 209.4343369 |
| YIL076W | SEC28 | 0.63182401 | 0.99917121 | 0.10947613 | 126.9592224 | 188.81891 |
| YPL144W | POC4 | 0.63979682 | 0.99917121 | 0.10964854 | 137.9789195 | 206.345255 |
| YOR173W | DCS2 | 0.87458814 | 0.99917121 | 0.10977172 | 89.46038452 | 129.0710972 |
| YOR023C | AHC1 | 0.40153985 | 0.99917121 | 0.10986526 | 147.6617482 | 221.7374163 |
| YMR110C | HFD1 | 0.24703022 | 0.99917121 | 0.1099449 | 139.3940941 | 208.5625968 |
| YMR023C | MSS1 | 0.58481351 | 0.99917121 | 0.11007435 | 147.963781 | 222.1928724 |
| YLR237W | THI7 | 0.48027939 | 0.99917121 | 0.11008144 | 152.4584161 | 229.3490994 |
| YCR021C | HSP30 | 0.32815192 | 0.99917121 | 0.11011071 | 143.3385629 | 214.8234114 |
| YLR041W | YLR041W | 0.59143432 | 0.99917121 | 0.11017855 | 141.4884797 | 211.8691354 |
| YLR356W | YLR356W | 0.62084461 | 0.99917121 | 0.11031592 | 137.52928 | 205.5479047 |
| YGL195W | GCN1 | 0.4465957 | 0.99917121 | 0.11033665 | 147.4800178 | 221.3905679 |
| YBR113W | YBR113W | 0.68108653 | 0.99917121 | 0.1104487 | 150.7937924 | 226.6536407 |
| YLR425W | TUS1 | 0.36758726 | 0.99917121 | 0.11060138 | 115.4075426 | 170.2872583 |
| YJL170C | ASG7 | 0.49660976 | 0.99917121 | 0.11078711 | 151.9896429 | 228.5166139 |
| YDL154W | MSH5 | 0.61373426 | 0.99917121 | 0.11093728 | 174.0544861 | 263.6335562 |
| YPR195C | YPR195C | 0.47468922 | 0.99917121 | 0.11094271 | 138.2125614 | 206.5595242 |
| YER163C | YER163C | 0.48835744 | 0.99917121 | 0.11100505 | 145.9627175 | 218.892989 |
| YGL262W | YGL262W | 0.42439262 | 0.99917121 | 0.11108558 | 147.5209225 | 221.3644007 |
| YGR281W | YOR1 | 0.51406173 | 0.99917121 | 0.11120274 | 139.2882856 | 208.2407673 |
| YLR016C | PML1 | 0.5903431 | 0.99917121 | 0.11157266 | 140.9414791 | 210.8281557 |
| YOL089C | HAL9 | 0.5206269 | 0.99917121 | 0.1116706 | 129.4104335 | 192.4546003 |
| YDL123W | SNA4 | 0.55424786 | 0.99917121 | 0.11172663 | 157.5535583 | 237.261852 |

| | | | | | | |
|---|---|---|---|---|---|---|
| YGL236C | MTO1 | 0.36943598 | 0.99917121 | 0.11242187 | 164.7641159 | 248.6589227 |
| YLR216C | CPR6 | 0.58347588 | 0.99917121 | 0.11244635 | 138.5258164 | 206.8750305 |
| YGR181W | TIM13 | 0.5758821 | 0.99917121 | 0.11276796 | 154.4957866 | 232.2658185 |
| YLL058W | YLL058W | 0.5406493 | 0.99917121 | 0.11289096 | 147.6324472 | 221.3218932 |
| YNR048W | YNR048W | 0.53190826 | 0.99917121 | 0.11305223 | 148.9045643 | 223.3279054 |
| YGL087C | MMS2 | 0.3736759 | 0.99917121 | 0.11305995 | 147.9292929 | 221.7739772 |
| YDL106C | PHO2 | 0.44110819 | 0.99917121 | 0.11309145 | 157.5130945 | 237.0310326 |
| YMR010W | YMR010W | 0.35359349 | 0.99917121 | 0.11314063 | 131.5056863 | 195.6117923 |
| YOR104W | PIN2 | 0.46539965 | 0.99917121 | 0.11325846 | 142.0945442 | 212.4587372 |
| YOL065C | INP54 | 0.51841177 | 0.99917121 | 0.11334937 | 138.5069439 | 206.7348903 |
| YLR283W | YLR283W | 0.48903953 | 0.99917121 | 0.11344733 | 149.0059895 | 223.4412448 |
| YFL030W | AGX1 | 0.29998501 | 0.99917121 | 0.11346383 | 133.0184048 | 197.9811878 |
| YKL072W | STB6 | 0.57769967 | 0.99917121 | 0.11366933 | 142.0970951 | 212.4127099 |
| YMR306W | FKS3 | 0.43188831 | 0.99917121 | 0.11369606 | 151.834961 | 227.9156719 |
| YDL177C | YDL177C | 0.3372984 | 0.99917121 | 0.11380211 | 144.6751883 | 216.5017834 |
| YGL031C | RPL24A | 0.57185428 | 0.99917121 | 0.11414867 | 107.9073725 | 157.911809 |
| YMR234W | RNH1 | 0.34473038 | 0.99917121 | 0.11420326 | 134.3242846 | 199.9704784 |
| YGL037C | PNC1 | 0.39874319 | 0.99917121 | 0.11443527 | 143.605827 | 214.7217826 |
| YHR076W | PTC7 | 0.20482194 | 0.99917121 | 0.11460011 | 139.4868419 | 208.1427656 |
| YJL169W | YJL169W | 0.5735953 | 0.99917121 | 0.11479839 | 144.8386891 | 216.6406794 |
| YPL201C | YIG1 | 0.22154792 | 0.99917121 | 0.11482399 | 146.2010286 | 218.8068972 |
| YDR156W | RPA14 | 0.50387446 | 0.99917121 | 0.11483206 | 133.9136335 | 199.2399153 |
| YOL098C | YOL098C | 0.37408334 | 0.99917121 | 0.11492667 | 136.8776395 | 203.9481559 |
| YBR141C | YBR141C | 0.44134301 | 0.99917121 | 0.11522324 | 151.4046624 | 227.0443011 |
| YGR018C | YGR018C | 0.42327945 | 0.99917121 | 0.11531761 | 148.6021891 | 222.57024 |
| YOL048C | YOL048C | 0.41719508 | 0.99917121 | 0.11535516 | 132.7806012 | 197.3719447 |
| YBR063C | YBR063C | 0.51811 | 0.99917121 | 0.11540052 | 143.8886174 | 215.0544136 |
| YKL148C | SDH1 | 0.36986186 | 0.99917121 | 0.11548048 | 124.0935014 | 183.5236467 |
| YDR066C | YDR066C | 0.40369272 | 0.99917121 | 0.11550486 | 145.1443857 | 217.0413333 |
| YPL182C | YPL182C | 0.52894981 | 0.99917121 | 0.11566182 | 144.8872201 | 216.6126964 |
| YBL053W | YBL053W | 0.54243969 | 0.99917121 | 0.11568405 | 145.5831839 | 217.7182139 |
| YIL111W | COX5B | 0.42178703 | 0.99917121 | 0.11575531 | 134.2137898 | 199.6053186 |
| YLL044W | YLL044W | 0.52636527 | 0.99917121 | 0.11576519 | 161.0964463 | 242.4110733 |
| YOR368W | RAD17 | 0.04997501 | 0.94753174 | 0.11587104 | 131.7217538 | 195.6229835 |
| YLR444C | YLR444C | 0.55850559 | 0.99917121 | 0.1159119 | 125.2955632 | 185.3851712 |
| YKL110C | KTI12 | 0.19529922 | 0.99917121 | 0.11617777 | 115.1599189 | 169.2131295 |
| YLR126C | YLR126C | 0.55000657 | 0.99917121 | 0.11619162 | 144.1813265 | 215.4240686 |
| YER177W | BMH1 | 0.54818466 | 0.99917121 | 0.11622005 | 136.9573651 | 203.917431 |
| YOR032C | HMS1 | 0.46902141 | 0.99917121 | 0.11630889 | 132.4118207 | 196.6684426 |
| YKL046C | DCW1 | 0.61324083 | 0.99917121 | 0.11632242 | 131.1918098 | 194.7240921 |
| YDR439W | LRS4 | 0.60068047 | 0.99917121 | 0.11646971 | 138.5513832 | 206.4252508 |
| YHR132C | ECM14 | 0.54223815 | 0.99917121 | 0.11668596 | 151.4965933 | 227.0123667 |
| YGL161C | YIP5 | 0.31150034 | 0.99917121 | 0.11676102 | 143.2897598 | 213.9349554 |
| YPL021W | ECM23 | 0.37135166 | 0.99917121 | 0.11679369 | 135.3979982 | 201.3644193 |
| YHR080C | YHR080C | 0.47030091 | 0.99917121 | 0.11680712 | 146.4044442 | 218.8890442 |
| YGR084C | MRP13 | 0.48859846 | 0.99917121 | 0.11732356 | 147.5153493 | 220.59505 |
| YKL109W | HAP4 | 0.17592446 | 0.99917121 | 0.11736471 | 110.7049156 | 161.9744452 |
| YHR009C | YHR009C | 0.4801141 | 0.99917121 | 0.11745246 | 130.7355102 | 193.8597332 |
| YKL077W | YKL077W | 0.3157338 | 0.99917121 | 0.11752683 | 161.0469637 | 242.1175166 |
| YMR303C | ADH2 | 0.45014851 | 0.99917121 | 0.11770529 | 140.4436324 | 209.2877686 |
| YCL025C | AGP1 | 0.44529735 | 0.99917121 | 0.11774807 | 153.2645392 | 229.698097 |
| YMR025W | CSI1 | 0.36355263 | 0.99917121 | 0.11780209 | 142.262452 | 212.1721906 |
| YLR211C | YLR211C | 0.56805368 | 0.99917121 | 0.11833654 | 138.0304385 | 205.3681316 |
| YGR244C | LSC2 | 0.50771771 | 0.99917121 | 0.11837713 | 147.0002788 | 219.6464288 |
| YFR033C | QCR6 | 0.52642965 | 0.99917121 | 0.11851895 | 155.3092574 | 232.8600528 |
| YPL258C | THI21 | 0.25276415 | 0.99917121 | 0.11857495 | 145.8317395 | 217.7615733 |
| YFL010C | WWM1 | 0.49050938 | 0.99917121 | 0.11870267 | 165.1620877 | 248.5269418 |
| YGR242W | YGR242W | 0.4608417 | 0.99917121 | 0.11881723 | 151.727234 | 227.119806 |
| YJR119C | JHD2 | 0.36449212 | 0.99917121 | 0.11899243 | 140.6667814 | 209.4861877 |
| YGR004W | PEX31 | 0.20042538 | 0.99917121 | 0.11902598 | 142.9924102 | 213.1853435 |
| YHR181W | SVP26 | 0.44194314 | 0.99917121 | 0.11905156 | 148.3158386 | 221.6590571 |
| YCL049C | YCL049C | 0.44228242 | 0.99917121 | 0.11909737 | 145.047235 | 216.4486687 |
| YBR185C | MBA1 | 0.48453037 | 0.99917121 | 0.11927219 | 129.5451387 | 191.7423849 |
| YDR036C | EHD3 | 0.46202172 | 0.99917121 | 0.11933659 | 140.1318885 | 208.5924863 |

| | | | | | | |
|---|---|---|---|---|---|---|
| YDR133C | YDR133C | 0.56379094 | 0.99917121 | 0.11941372 | 165.6329407 | 249.1900259 |
| YBR297W | MAL33 | 0.41867621 | 0.99917121 | 0.11952452 | 132.6331354 | 196.628836 |
| YKL151C | YKL151C | 0.4416873 | 0.99917121 | 0.11959218 | 140.7257643 | 209.5069943 |
| YOR047C | STD1 | 0.07881652 | 0.94753174 | 0.11963724 | 138.1082357 | 205.3334436 |
| YGR279C | SCW4 | 0.45481869 | 0.99917121 | 0.11970938 | 161.1699511 | 242.0472804 |
| YDR512C | EMI1 | 0.32190532 | 0.99917121 | 0.11985449 | 111.6912357 | 163.2414937 |
| YOL079W | YOL079W | 0.4844706 | 0.99917121 | 0.11986106 | 134.7595837 | 199.9738862 |
| YBR103W | SIF2 | 0.40489831 | 0.99917121 | 0.12004701 | 121.8467738 | 179.389331 |
| YOR286W | FMP31 | 0.6044291 | 0.99917121 | 0.12008349 | 132.8627449 | 196.9263133 |
| YDR479C | PEX29 | 0.52460156 | 0.99917121 | 0.12010139 | 143.5791134 | 213.9884842 |
| YGR141W | VPS62 | 0.33506815 | 0.99917121 | 0.12010918 | 151.0007387 | 225.8054594 |
| YBR047W | FMP23 | 0.43749867 | 0.99917121 | 0.12021103 | 152.6594884 | 228.4343746 |
| YDR179W-A | YDR179W-A | 0.225331 | 0.99917121 | 0.12021858 | 146.9110181 | 219.279801 |
| YLR362W | STE11 | 0.55406967 | 0.99917121 | 0.12031907 | 151.1893248 | 226.0801684 |
| YCL012W | YCL012W | 0.49094636 | 0.99917121 | 0.12033955 | 154.616076 | 231.5343058 |
| YMR311C | GLC8 | 0.50066103 | 0.99917121 | 0.12041034 | 152.3513101 | 227.9193446 |
| YBR195C | MSI1 | 0.37902425 | 0.99917121 | 0.1204221 | 139.5000654 | 207.4540592 |
| YBL005W | PDR3 | 0.45191448 | 0.99917121 | 0.1205042 | 147.1087799 | 219.5598892 |
| YGL079W | YGL079W | 0.42699816 | 0.99917121 | 0.12088426 | 147.7322486 | 220.5063443 |
| YDR219C | MFB1 | 0.39386616 | 0.99917121 | 0.12095337 | 137.2746662 | 203.8456472 |
| YGL214W | YGL214W | 0.17877704 | 0.99917121 | 0.12100143 | 134.1573175 | 198.8758366 |
| YHR139C-A | YHR139C-A | 0.62357597 | 0.99917121 | 0.12112512 | 156.6617632 | 234.696014 |
| YER135C | YER135C | 0.54305448 | 0.99917121 | 0.12115329 | 144.4088394 | 215.1814716 |
| YPR054W | SMK1 | 0.67407779 | 0.99917121 | 0.12115502 | 134.111306 | 198.7838453 |
| YOR059C | YOR059C | 0.51944167 | 0.99917121 | 0.12116132 | 156.3862406 | 234.2528682 |
| YPR184W | GDB1 | 0.53791214 | 0.99917121 | 0.12121108 | 132.4160125 | 196.0774877 |
| YMR068W | AVO2 | 0.43495488 | 0.99917121 | 0.12125203 | 137.6207066 | 204.36026 |
| YPR138C | MEP3 | 0.39717803 | 0.99917121 | 0.12127498 | 133.3978972 | 197.6332145 |
| YKR039W | GAP1 | 0.32181973 | 0.99917121 | 0.12132746 | 138.7635301 | 206.1708549 |
| YOL147C | PEX11 | 0.37730397 | 0.99917121 | 0.12152492 | 130.1764837 | 192.4730833 |
| YLR050C | YLR050C | 0.50579559 | 0.99917121 | 0.12164735 | 141.0288905 | 209.7391342 |
| YJR147W | HMS2 | 0.48032036 | 0.99917121 | 0.12176471 | 146.2394688 | 218.021961 |
| YCL027W | FUS1 | 0.51279632 | 0.99917121 | 0.12177597 | 157.3226933 | 235.6691091 |
| YMR021C | MAC1 | 0.31383921 | 0.99917121 | 0.12184259 | 116.2678221 | 170.2867121 |
| YLR021W | IRC25 | 0.3685497 | 0.99917121 | 0.12186902 | 126.5543918 | 186.6634478 |
| YCR085W | YCR085W | 0.32746227 | 0.99917121 | 0.12190172 | 134.8462157 | 199.8630577 |
| YOR066W | YOR066W | 0.35554861 | 0.99917121 | 0.12217521 | 138.5432212 | 205.7166923 |
| YCR051W | YCR051W | 0.57370857 | 0.99917121 | 0.12218113 | 148.7992811 | 222.0473455 |
| YFL001W | DEG1 | 0.32717426 | 0.99917121 | 0.12218125 | 107.0330177 | 155.5402608 |
| YGL104C | VPS73 | 0.11920453 | 0.96075443 | 0.12240098 | 138.2420944 | 205.2096661 |
| YMR099C | YMR099C | 0.01846069 | 0.87709187 | 0.12240837 | 118.1236679 | 173.1729189 |
| YGR283C | YGR283C | 0.52280501 | 0.99917121 | 0.12250724 | 154.5965945 | 231.2390188 |
| YMR172C-A | YMR172C-A | 0.44383527 | 0.99917121 | 0.12254915 | 140.744902 | 209.1769821 |
| YJR140C | HIR3 | 0.15437358 | 0.98492771 | 0.12268808 | 111.9443543 | 163.2991056 |
| YPR147C | YPR147C | 0.50152392 | 0.99917121 | 0.12274803 | 131.42573 | 194.3132277 |
| YBR116C | YBR116C | 0.46624225 | 0.99917121 | 0.12280466 | 148.0355129 | 220.7551343 |
| YOR251C | YOR251C | 0.50491666 | 0.99917121 | 0.12283488 | 136.2274312 | 201.9486927 |
| YLR262C-A | TMA7 | 0.5912761 | 0.99917121 | 0.12286027 | 141.1613671 | 209.8022159 |
| YCL074W | YCL074W | 0.49229699 | 0.99917121 | 0.12295214 | 141.8845278 | 210.9425516 |
| YGL109W | YGL109W | 0.46715771 | 0.99917121 | 0.12309479 | 160.0939394 | 239.9211615 |
| YHR094C | HXT1 | 0.44356926 | 0.99917121 | 0.12321397 | 148.0670151 | 220.7553979 |
| YKL061W | YKL061W | 0.25322585 | 0.99917121 | 0.12322924 | 129.1165662 | 190.5775348 |
| YNL180C | RHO5 | 0.80919662 | 0.99917121 | 0.12323752 | 102.138063 | 147.6169439 |
| YLR246W | ERF2 | 0.53938521 | 0.99917121 | 0.12331658 | 145.4820932 | 216.6267543 |
| YDR258C | HSP78 | 0.31570319 | 0.99917121 | 0.12333278 | 141.5114717 | 210.3021067 |
| YCL063W | VAC17 | 0.594391 | 0.99917121 | 0.12333564 | 134.7491616 | 199.5337022 |
| YMR299C | DYN3 | 0.28070995 | 0.99917121 | 0.12339934 | 126.032408 | 185.6456973 |
| YOR081C | TGL5 | 0.48137366 | 0.99917121 | 0.12403144 | 153.206272 | 228.8393039 |
| YDL010W | YDL010W | 0.26220809 | 0.99917121 | 0.12403452 | 151.5863149 | 226.2593688 |
| YOL083W | YOL083W | 0.43475418 | 0.99917121 | 0.12406415 | 134.9429801 | 199.753519 |
| YMR241W | YHM2 | 0.34061129 | 0.99917121 | 0.12428343 | 135.0000463 | 199.817656 |
| YLR149C | YLR149C | 0.6176907 | 0.99917121 | 0.12435743 | 135.3403238 | 200.3504799 |
| YLR220W | CCC1 | 0.27802579 | 0.99917121 | 0.12439898 | 134.9437332 | 199.7138981 |
| YPR066W | UBA3 | 0.64306677 | 0.99917121 | 0.12442601 | 123.7067509 | 181.8172436 |

| | | | | | | |
|---|---|---|---|---|---|---|
| YGR214W | RPS0A | 0.41901641 | 0.99917121 | 0.12461333 | 115.8200024 | 169.2358379 |
| YKL207W | YKL207W | 0.44796137 | 0.99917121 | 0.12464319 | 138.3315567 | 205.0787734 |
| YHR162W | YHR162W | 0.65186265 | 0.99917121 | 0.12495145 | 141.9678076 | 210.8314254 |
| YFR021W | ATG18 | 0.47716636 | 0.99917121 | 0.12496648 | 144.3793159 | 214.6695908 |
| YNL144C | YNL144C | 0.74691568 | 0.99917121 | 0.12505173 | 152.8713499 | 228.1816022 |
| YDR431W | YDR431W | 0.45824078 | 0.99917121 | 0.12522862 | 144.130996 | 214.2422183 |
| YLR313C | SPH1 | 0.53898175 | 0.99917121 | 0.12527421 | 141.1288185 | 209.4561018 |
| YOR042W | CUE5 | 0.49470708 | 0.99917121 | 0.125341 | 141.7293767 | 210.4042663 |
| YDL086W | YDL086W | 0.22723168 | 0.99917121 | 0.12548445 | 152.6831262 | 227.8291286 |
| YLR271W | YLR271W | 0.52090922 | 0.99917121 | 0.1257061 | 137.6568298 | 203.8747819 |
| YHR034C | PIH1 | 0.45669351 | 0.99917121 | 0.12574579 | 110.3885365 | 160.4489108 |
| YJL106W | IME2 | 0.42509549 | 0.99917121 | 0.12580722 | 135.3516654 | 200.1917949 |
| YJL123C | MTC1 | 0.51216957 | 0.99917121 | 0.12583681 | 153.5051095 | 229.0950686 |
| YER120W | SCS2 | 0.34711037 | 0.99917121 | 0.12602567 | 127.0365003 | 186.9243991 |
| YBR062C | YBR062C | 0.40039394 | 0.99917121 | 0.12604767 | 143.6581444 | 213.3894152 |
| YGR196C | FYV8 | 0.5936901 | 0.99917121 | 0.12607209 | 156.3707599 | 233.6295425 |
| YMR169C | ALD3 | 0.1019116 | 0.94753174 | 0.12620241 | 138.2078625 | 204.6917218 |
| YBL062W | YBL062W | 0.36094649 | 0.99917121 | 0.12623458 | 145.9458533 | 217.0094929 |
| YHR018C | ARG4 | 0.55384849 | 0.99917121 | 0.12649645 | 143.7441529 | 213.4716607 |
| YPL187W | MF(ALPHA)1 | 0.33980542 | 0.99917121 | 0.12650075 | 142.3521683 | 211.254591 |
| YPL120W | VPS30 | 0.51707229 | 0.99917121 | 0.1266149 | 129.065781 | 190.0839177 |
| YDR326C | YSP2 | 0.59207829 | 0.99917121 | 0.12662112 | 138.164231 | 204.5711991 |
| YMR181C | YMR181C | 0.40577436 | 0.99917121 | 0.12666608 | 139.2020085 | 206.218237 |
| YGR217W | CCH1 | 0.47385193 | 0.99917121 | 0.12667397 | 151.9335288 | 226.4904814 |
| YLR308W | CDA2 | 0.42834994 | 0.99917121 | 0.12669768 | 138.9820371 | 205.8641103 |
| YER118C | SHO1 | 0.524472 | 0.99917121 | 0.12680868 | 149.2891996 | 222.2633264 |
| YGL080W | FMP37 | 0.41724094 | 0.99917121 | 0.12681364 | 108.5397464 | 157.3747828 |
| YOR013W | YOR013W | 0.46166551 | 0.99917121 | 0.12682158 | 138.9927641 | 205.8660867 |
| YER072W | VTC1 | 0.55521028 | 0.99917121 | 0.12700596 | 149.0980868 | 221.9349545 |
| YJL119C | YJL119C | 0.65180611 | 0.99917121 | 0.12707698 | 126.8920655 | 186.5662402 |
| YER046W-A | YER046W-A | 0.22769001 | 0.99917121 | 0.12715432 | 141.1084088 | 209.1943966 |
| YLR252W | YLR252W | 0.62324112 | 0.99917121 | 0.12726984 | 133.4656579 | 197.0102773 |
| YBR075W | YBR075W | 0.3419994 | 0.99917121 | 0.12747623 | 144.5031991 | 214.5608926 |
| YKR053C | YSR3 | 0.46796337 | 0.99917121 | 0.12754256 | 136.0316763 | 201.0630629 |
| YLL016W | SDC25 | 0.25298066 | 0.99917121 | 0.12783453 | 152.470679 | 227.2043359 |
| YBR294W | SUL1 | 0.56442342 | 0.99917121 | 0.1281703 | 168.8635808 | 253.2668588 |
| YIR039C | YPS6 | 0.08187601 | 0.94753174 | 0.12824911 | 137.0042247 | 202.525578 |
| YAL043C-A | YAL043C-A | 0.47126764 | 0.99917121 | 0.12827013 | 138.6676475 | 205.1717884 |
| YOR276W | CAF20 | 0.14703432 | 0.98328895 | 0.12830379 | 137.5776435 | 203.4320028 |
| YOR111W | YOR111W | 0.47301432 | 0.99917121 | 0.12846047 | 147.2403038 | 218.7993681 |
| YDR486C | VPS60 | 0.54847061 | 0.99917121 | 0.12846433 | 145.5817108 | 216.1578151 |
| YMR044W | IOC4 | 0.35084986 | 0.99917121 | 0.12847254 | 133.8540285 | 197.4820824 |
| YGR188C | BUB1 | 0.59840426 | 0.99917121 | 0.12851131 | 106.0085791 | 153.1372804 |
| YHR096C | HXT5 | 0.20131447 | 0.99917121 | 0.12870909 | 145.3874073 | 215.818575 |
| YLR381W | CTF3 | 0.5726396 | 0.99917121 | 0.12879852 | 138.1915408 | 204.3492376 |
| YBR093C | PHO5 | 0.44985336 | 0.99917121 | 0.12884238 | 110.1560054 | 159.7011295 |
| YOL131W | YOL131W | 0.47287678 | 0.99917121 | 0.12897856 | 131.7049225 | 193.9982353 |
| YAL049C | YAL049C | 0.4141359 | 0.99917121 | 0.12907778 | 146.3292017 | 217.2733068 |
| YDL236W | PHO13 | 0.40103775 | 0.99917121 | 0.12918858 | 140.4507128 | 207.8991085 |
| YDL026W | YDL026W | 0.59368994 | 0.99917121 | 0.12947957 | 149.750985 | 222.6730463 |
| YBR015C | MNN2 | 0.53452864 | 0.99917121 | 0.12952529 | 141.970171 | 210.2775893 |
| YLR352W | YLR352W | 0.49118235 | 0.99917121 | 0.1295436 | 140.9481592 | 208.6479422 |
| YBR018C | GAL7 | 0.40440681 | 0.99917121 | 0.12974472 | 148.5213194 | 220.6826469 |
| YGL101W | YGL101W | 0.32290527 | 0.99917121 | 0.12975607 | 129.4950917 | 190.3845941 |
| YBR033W | EDS1 | 0.31369505 | 0.99917121 | 0.12984286 | 144.4287546 | 214.1538323 |
| YGR058W | YGR058W | 0.22451722 | 0.99917121 | 0.1299643 | 133.6800316 | 197.0231543 |
| YNL135C | FPR1 | 0.38235169 | 0.99917121 | 0.1299784 | 114.2689461 | 166.1119331 |
| YLR250W | SSP120 | 0.50556293 | 0.99917121 | 0.13043314 | 149.2951303 | 221.83091 |
| YNR063W | YNR063W | 0.52894904 | 0.99917121 | 0.13045091 | 160.8893152 | 240.2908986 |
| YBR156C | SLI15 | 0.29569753 | 0.99917121 | 0.13061909 | 145.6806968 | 216.0527489 |
| YMR074C | YMR074C | 0.62026454 | 0.99917121 | 0.13068381 | 145.8289257 | 216.2808931 |
| YAL022C | FUN26 | 0.36770361 | 0.99917121 | 0.13087413 | 145.1115098 | 215.1153039 |
| YER091C | MET6 | 0.29268944 | 0.99917121 | 0.13087678 | 120.1915718 | 175.4333833 |
| YPL214C | THI6 | 0.4302244 | 0.99917121 | 0.13103123 | 140.7478299 | 208.1475878 |

| | | | | | | |
|---|---|---|---|---|---|---|
| YPR027C | YPR027C | 0.40495401 | 0.99917121 | 0.13105907 | 137.148898 | 202.4133856 |
| YMR204C | INP1 | 0.19404329 | 0.99917121 | 0.13113321 | 134.3022945 | 197.8715201 |
| YMR170C | ALD2 | 0.31324145 | 0.99917121 | 0.13144113 | 131.9839335 | 194.1423083 |
| YLR189C | ATG26 | 0.59257529 | 0.99917121 | 0.13169062 | 143.2891457 | 212.1138993 |
| YGL202W | ARO8 | 0.4326493 | 0.99917121 | 0.13169193 | 151.9009 | 225.8267814 |
| YCL002C | YCL002C | 0.41896828 | 0.99917121 | 0.13171319 | 144.7434228 | 214.4268848 |
| YOR031W | CRS5 | 0.39768459 | 0.99917121 | 0.13179032 | 141.3005318 | 208.9351486 |
| YPL061W | ALD6 | 0.54001109 | 0.99917121 | 0.13183462 | 135.3473861 | 199.4501772 |
| YLR372W | SUR4 | 0.83021001 | 0.99917121 | 0.13198945 | 84.60524063 | 118.631365 |
| YFL051C | YFL051C | 0.37898775 | 0.99917121 | 0.13205407 | 138.4324736 | 204.3360032 |
| YNL218W | MGS1 | 0.45076258 | 0.99917121 | 0.13212184 | 127.8182285 | 187.4260059 |
| YOR379C | YOR379C | 0.45638685 | 0.99917121 | 0.1322822 | 143.4823408 | 212.3494164 |
| YDL093W | PMT5 | 0.32766174 | 0.99917121 | 0.13230512 | 140.6440663 | 207.8270574 |
| YKR072C | SIS2 | 0.0775384 | 0.94753174 | 0.13235805 | 137.3664764 | 202.6014909 |
| YKL139W | CTK1 | 0.80031402 | 0.99917121 | 0.13238532 | 71.82522479 | 98.23267408 |
| YKL176C | LST4 | 0.34652223 | 0.99917121 | 0.1326848 | 131.7052086 | 193.5468601 |
| YKL085W | MDH1 | 0.11368358 | 0.95531631 | 0.13273872 | 138.855836 | 204.9266848 |
| YER123W | YCK3 | 0.0400126 | 0.94753174 | 0.13274961 | 125.9512646 | 184.3765893 |
| YHR077C | NMD2 | 0.05767065 | 0.94753174 | 0.13312845 | 144.4586378 | 213.8008688 |
| YLR194C | YLR194C | 0.5361333 | 0.99917121 | 0.13336204 | 139.537775 | 205.93659 |
| YFL006W | YFL006W | 0.54617608 | 0.99917121 | 0.13338907 | 140.8089973 | 207.9575429 |
| YPL263C | KEL3 | 0.64339336 | 0.99917121 | 0.13345506 | 125.6015957 | 183.7337872 |
| YGL250W | YGL250W | 0.38653608 | 0.99917121 | 0.13357716 | 149.1273504 | 221.1804535 |
| YMR318C | ADH6 | 0.42726807 | 0.99917121 | 0.13364412 | 151.1460112 | 224.3867314 |
| YHR106W | TRR2 | 0.35475652 | 0.99917121 | 0.1337181 | 161.7202021 | 241.2156673 |
| YER186C | YER186C | 0.26111494 | 0.99917121 | 0.13374163 | 140.5165346 | 207.4488549 |
| YDL051W | LHP1 | 0.29033574 | 0.99917121 | 0.13410994 | 141.354179 | 208.7377876 |
| YLR387C | REH1 | 0.07449161 | 0.94753174 | 0.13412306 | 140.1665039 | 206.8449782 |
| YOL162W | YOL162W | 0.67212269 | 0.99917121 | 0.13415353 | 147.9495761 | 219.2347425 |
| YJR030C | YJR030C | 0.52260312 | 0.99917121 | 0.13428883 | 153.190872 | 227.5642964 |
| YLR123C | YLR123C | 0.3713791 | 0.99917121 | 0.13443235 | 142.489404 | 210.5061734 |
| YKL017C | HCS1 | 0.28867415 | 0.99917121 | 0.13452573 | 139.2934914 | 205.405735 |
| YKR031C | SPO14 | 0.51828795 | 0.99917121 | 0.13453217 | 143.9014032 | 212.7424199 |
| YOL058W | ARG1 | 0.29516325 | 0.99917121 | 0.1346117 | 141.7688969 | 209.3369994 |
| YOR003W | YSP3 | 0.50222706 | 0.99917121 | 0.1347667 | 158.1958597 | 235.4757979 |
| YDR349C | YPS7 | 0.85203454 | 0.99917121 | 0.13488439 | 118.7429189 | 172.6380312 |
| YMR176W | ECM5 | 0.0671342 | 0.94753174 | 0.13492855 | 143.5070963 | 212.0662179 |
| YGR151C | YGR151C | 0.4010738 | 0.99917121 | 0.13504735 | 148.0396976 | 219.2692832 |
| YGL006W | PMC1 | 0.39868606 | 0.99917121 | 0.13507633 | 150.5255705 | 223.2241635 |
| YER140W | YER140W | 0.36261871 | 0.99917121 | 0.13542686 | 148.6282434 | 220.1601956 |
| YKL113C | RAD27 | 0.2622187 | 0.99917121 | 0.13546285 | 107.0919055 | 154.0148626 |
| YMR085W | YMR085W | 0.4159646 | 0.99917121 | 0.13546377 | 143.1493991 | 211.4313847 |
| YLR200W | YKE2 | 0.60734581 | 0.99917121 | 0.13550959 | 120.0308272 | 174.61263 |
| YGL165C | YGL165C | 0.575661 | 0.99917121 | 0.13559219 | 135.4327228 | 199.1279758 |
| YLR303W | MET17 | 0.48174835 | 0.99917121 | 0.13559832 | 139.9690178 | 206.3506591 |
| YDR262W | YDR262W | 0.03073071 | 0.94753174 | 0.13561134 | 133.6141347 | 196.2297873 |
| YBR068C | BAP2 | 0.61375882 | 0.99917121 | 0.13576796 | 151.2190064 | 224.2440478 |
| YPR026W | ATH1 | 0.50038081 | 0.99917121 | 0.13584194 | 147.3663804 | 218.1002483 |
| YBR084C-A | RPL19A | 0.53098124 | 0.99917121 | 0.13584581 | 142.4123696 | 210.2111913 |
| YMR129W | POM152 | 0.17671184 | 0.99917121 | 0.13585988 | 133.6405396 | 196.2415349 |
| YNR015W | SMM1 | 0.15445859 | 0.98492771 | 0.13590023 | 129.064963 | 188.9506349 |
| YCR067C | SED4 | 0.44049741 | 0.99917121 | 0.13597206 | 148.8043049 | 220.3740834 |
| YPR150W | YPR150W | 0.51007889 | 0.99917121 | 0.13624379 | 147.973344 | 219.0177651 |
| YMR100W | MUB1 | 0.40281359 | 0.99917121 | 0.13624889 | 119.0545619 | 172.9679321 |
| YOR025W | HST3 | 0.52720205 | 0.99917121 | 0.13650369 | 151.8112735 | 225.0974594 |
| YBR299W | MAL32 | 0.41533864 | 0.99917121 | 0.13652698 | 140.9301775 | 207.76796 |
| YER054C | GIP2 | 0.18542489 | 0.99917121 | 0.13655903 | 141.848874 | 209.2269517 |
| YNL037C | IDH1 | 0.15004639 | 0.98328895 | 0.13668989 | 114.0029062 | 164.8700983 |
| YLL001W | DNM1 | 0.27390803 | 0.99917121 | 0.13672057 | 137.455395 | 202.2112435 |
| YGL154C | LYS5 | 0.13806982 | 0.97853666 | 0.1368032 | 122.1015431 | 177.7522579 |
| YGL094C | PAN2 | 0.44718689 | 0.99917121 | 0.13691323 | 153.7948213 | 228.2060607 |
| YFR044C | DUG1 | 0.28644895 | 0.99917121 | 0.13696735 | 140.7878622 | 207.487657 |
| YKL208W | CBT1 | 0.30610517 | 0.99917121 | 0.13699548 | 102.3479092 | 146.2738528 |
| YDR033W | MRH1 | 0.45710255 | 0.99917121 | 0.1372106 | 140.3486346 | 206.7585922 |

| | | | | | | |
|---|---|---|---|---|---|---|
| YKL158W | YKL158W | 0.23021602 | 0.99917121 | 0.1372605 | 144.4095997 | 213.2190412 |
| YPR196W | YPR196W | 0.34186306 | 0.99917121 | 0.13737816 | 140.5083964 | 206.9925637 |
| YER056C-A | RPL34A | 0.52790213 | 0.99917121 | 0.13744748 | 140.326984 | 206.6952387 |
| YER002W | NOP16 | 0.39371034 | 0.99917121 | 0.13750167 | 134.2230501 | 196.9689508 |
| YDR278C | YDR278C | 0.23216304 | 0.99917121 | 0.13763786 | 148.1788624 | 219.1750727 |
| YDR497C | ITR1 | 0.23714353 | 0.99917121 | 0.13796725 | 142.1254096 | 209.4956195 |
| YDL040C | NAT1 | 0.04222436 | 0.94753174 | 0.13806063 | 138.0658488 | 203.0199395 |
| YOR052C | YOR052C | 0.59888196 | 0.99917121 | 0.1384043 | 149.5970718 | 221.3399412 |
| YPL273W | SAM4 | 0.58613175 | 0.99917121 | 0.1384055 | 128.7641388 | 188.1661937 |
| YGR273C | YGR273C | 0.51193702 | 0.99917121 | 0.1384176 | 142.6856174 | 210.3327715 |
| YDL070W | BDF2 | 0.45848872 | 0.99917121 | 0.13860473 | 130.5008831 | 190.9074342 |
| YMR230W | RPS10B | 0.24641542 | 0.99917121 | 0.13860919 | 102.383912 | 146.134454 |
| YMR222C | FSH2 | 0.32707476 | 0.99917121 | 0.1386671 | 142.476479 | 209.969331 |
| YHR021W-A | ECM12 | 0.13281518 | 0.97853666 | 0.13876897 | 136.6088019 | 200.6134373 |
| YKR102W | FLO10 | 0.38127056 | 0.99917121 | 0.13882015 | 131.5205972 | 192.5049267 |
| YNR020C | YNR020C | 0.17008229 | 0.99636201 | 0.13887442 | 119.9348761 | 174.0496325 |
| YLR286C | CTS1 | 0.50138111 | 0.99917121 | 0.13888027 | 143.7156968 | 211.9166284 |
| YLR241W | YLR241W | 0.5638378 | 0.99917121 | 0.13929101 | 136.0138771 | 199.6024591 |
| YMR012W | CLU1 | 0.4450218 | 0.99917121 | 0.13935791 | 141.0825996 | 207.6655511 |
| YIL037C | PRM2 | 0.44647451 | 0.99917121 | 0.13961043 | 135.9489156 | 199.4600761 |
| YBL036C | YBL036C | 0.39798071 | 0.99917121 | 0.13966534 | 146.3003737 | 215.9366645 |
| YMR264W | CUE1 | 0.54283954 | 0.99917121 | 0.1396721 | 123.4165807 | 179.4965263 |
| YDR109C | YDR109C | 0.27323287 | 0.99917121 | 0.1396997 | 146.9517882 | 216.9697651 |
| YDL119C | YDL119C | 0.58426942 | 0.99917121 | 0.13974738 | 127.2141923 | 185.5345264 |
| YOR304W | ISW2 | 0.56968219 | 0.99917121 | 0.139782 | 142.9462093 | 210.5813939 |
| YMR148W | YMR148W | 0.17660787 | 0.99917121 | 0.13989375 | 135.5937318 | 198.8599548 |
| YML101C | CUE4 | 0.48721688 | 0.99917121 | 0.13996624 | 140.5162464 | 206.6895492 |
| YER001W | MNN1 | 0.56077918 | 0.99917121 | 0.14029455 | 150.8579512 | 223.1172775 |
| YLR377C | FBP1 | 0.53848297 | 0.99917121 | 0.14051313 | 138.9917195 | 204.1952764 |
| YGL058W | RAD6 | 0.45362078 | 0.99917121 | 0.14055748 | 155.9730075 | 231.230252 |
| YDL088C | ASM4 | 0.5192263 | 0.99917121 | 0.14068829 | 155.4291867 | 230.3483443 |
| YGL043W | DST1 | 0.39416835 | 0.99917121 | 0.14069768 | 137.1881146 | 201.300783 |
| YJL055W | YJL055W | 0.40235704 | 0.99917121 | 0.14075073 | 144.4759267 | 212.899161 |
| YKL201C | MNN4 | 0.23749272 | 0.99917121 | 0.14084902 | 138.0573111 | 202.6664107 |
| YCL064C | CHA1 | 0.2951306 | 0.99917121 | 0.14098316 | 146.2173011 | 215.6437273 |
| YLR112W | YLR112W | 0.494613 | 0.99917121 | 0.14108998 | 146.2408626 | 215.6682228 |
| YKL029C | MAE1 | 0.08134867 | 0.94753174 | 0.14113683 | 143.3282959 | 211.0246463 |
| YGL013C | PDR1 | 0.2394589 | 0.99917121 | 0.14114106 | 149.3554131 | 220.6214922 |
| YKL167C | MRP49 | 0.21792494 | 0.99917121 | 0.14124082 | 142.8210009 | 210.2041706 |
| YLR185W | RPL37A | 0.21447784 | 0.99917121 | 0.14151556 | 89.93027689 | 125.9494238 |
| YML102C-A | YML102C-A | 0.29843877 | 0.99917121 | 0.14163912 | 141.6988961 | 208.3688152 |
| YGL157W | YGL157W | 0.45258293 | 0.99917121 | 0.14165877 | 149.5744972 | 220.9072388 |
| YKR057W | RPS21A | 0.70621361 | 0.99917121 | 0.1418521 | 123.2353388 | 178.9421574 |
| YPL192C | PRM3 | 0.60266337 | 0.99917121 | 0.14196885 | 167.5610575 | 249.510578 |
| YPR093C | ASR1 | 0.40595811 | 0.99917121 | 0.14203038 | 140.4785483 | 206.3778788 |
| YBR206W | YBR206W | 0.4040476 | 0.99917121 | 0.1420602 | 161.3831636 | 239.6619893 |
| YLR398C | SKI2 | 0.46085748 | 0.99917121 | 0.1421454 | 134.7163143 | 197.1882871 |
| YBR105C | VID24 | 0.49199442 | 0.99917121 | 0.14240734 | 159.6877183 | 236.9199038 |
| YBR148W | YSW1 | 0.26217902 | 0.99917121 | 0.14243245 | 143.1066844 | 210.51381 |
| YDL078C | MDH3 | 0.32201259 | 0.99917121 | 0.1426854 | 149.4300873 | 220.5521291 |
| YNL140C | YNL140C | 0.42705299 | 0.99917121 | 0.14278701 | 127.7149682 | 185.9613801 |
| YPL047W | SGF11 | 0.54650287 | 0.99917121 | 0.14282138 | 127.9371206 | 186.3109378 |
| YNL336W | COS1 | 0.40538871 | 0.99917121 | 0.14298747 | 128.3416787 | 186.9348927 |
| YML113W | DAT1 | 0.45105883 | 0.99917121 | 0.1430587 | 146.4369736 | 215.7404947 |
| YPR038W | YPR038W | 0.33273547 | 0.99917121 | 0.14326906 | 144.5388737 | 212.6923843 |
| YNL319W | YNL319W | 0.35167584 | 0.99917121 | 0.14375913 | 138.3947681 | 202.8489913 |
| YCL001W-A | YCL001W-A | 0.3593651 | 0.99917121 | 0.14377629 | 140.7024845 | 206.5216218 |
| YCR043C | YCR043C | 0.29730635 | 0.99917121 | 0.14391223 | 146.9354443 | 216.4301863 |
| YDR408C | ADE8 | 0.29714715 | 0.99917121 | 0.14403178 | 148.9649807 | 219.6473724 |
| YPR149W | NCE102 | 0.5580171 | 0.99917121 | 0.14415059 | 127.985178 | 186.2254176 |
| YBR232C | YBR232C | 0.22615882 | 0.99917121 | 0.14427912 | 157.5848497 | 233.3431821 |
| YMR158C-B | YMR158C-B | 0.02855256 | 0.94311309 | 0.14433566 | 130.0946392 | 189.5618838 |
| YBL103C | RTG3 | 0.25661301 | 0.99917121 | 0.14443479 | 137.6343183 | 201.5557085 |
| YML108W | YML108W | 0.18158704 | 0.99917121 | 0.14457713 | 136.0603049 | 199.031954 |

| | | | | | | |
|---|---|---|---|---|---|---|
| YIL107C | PFK26 | 0.32255501 | 0.99917121 | 0.14460942 | 147.004425 | 216.4550337 |
| YFL035C-B | YFL035C-B | 0.3975331 | 0.99917121 | 0.14473848 | 142.8449894 | 209.8159671 |
| YHR021C | RPS27B | 0.43051032 | 0.99917121 | 0.14484847 | 90.23867433 | 126.034187 |
| YDR436W | PPZ2 | 0.28323058 | 0.99917121 | 0.145277 | 145.2048629 | 213.5080919 |
| YKL166C | TPK3 | 0.60043378 | 0.99917121 | 0.14544146 | 132.1900126 | 192.7636716 |
| YDR179C | CSN9 | 0.55209864 | 0.99917121 | 0.14556055 | 147.4141115 | 216.9914505 |
| YLR312C | YLR312C | 0.48531456 | 0.99917121 | 0.14564175 | 144.6464816 | 212.5744798 |
| YFR047C | BNA6 | 0.44372686 | 0.99917121 | 0.14581051 | 148.1043974 | 218.0601642 |
| YML094W | GIM5 | 0.25030578 | 0.99917121 | 0.1460318 | 136.1105837 | 198.934677 |
| YGR016W | YGR016W | 0.29098327 | 0.99917121 | 0.146101 | 155.4532841 | 229.7268488 |
| YNR032W | PPG1 | 0.37577225 | 0.99917121 | 0.14617653 | 148.314016 | 218.3493315 |
| YGL217C | YGL217C | 0.01531246 | 0.84351098 | 0.14619923 | 135.4174742 | 197.8105822 |
| YPR052C | NHP6A | 0.56592847 | 0.99917121 | 0.14625212 | 148.1493663 | 218.0779346 |
| YDR068W | DOS2 | 0.3512209 | 0.99917121 | 0.14657337 | 150.9535538 | 222.5040563 |
| YKL073W | LHS1 | 0.28578691 | 0.99917121 | 0.14659411 | 134.5851627 | 196.4371005 |
| YPR097W | YPR097W | 0.41335699 | 0.99917121 | 0.14663771 | 143.3650064 | 210.4124864 |
| YEL037C | RAD23 | 0.27908931 | 0.99917121 | 0.14668332 | 138.3315553 | 202.3918429 |
| YLR102C | APC9 | 0.38772516 | 0.99917121 | 0.14683348 | 155.233399 | 229.2874149 |
| YMR030W | RSF1 | 0.21638911 | 0.99917121 | 0.14718431 | 138.2372597 | 202.1806143 |
| YPR122W | AXL1 | 0.53877901 | 0.99917121 | 0.14719035 | 133.4964681 | 194.6308146 |
| YNL284C | MRPL10 | 0.35897991 | 0.99917121 | 0.14731055 | 150.1672822 | 221.1621559 |
| YOR127W | RGA1 | 0.52166452 | 0.99917121 | 0.14739124 | 149.2723296 | 219.727229 |
| YGL059W | YGL059W | 0.24233601 | 0.99917121 | 0.14746377 | 165.8363308 | 246.0942959 |
| YBR228W | SLX1 | 0.38583026 | 0.99917121 | 0.14751213 | 153.2914786 | 226.1124376 |
| YPL166W | ATG29 | 0.56543615 | 0.99917121 | 0.14751301 | 146.575494 | 215.418041 |
| YBL017C | PEP1 | 0.26472121 | 0.99917121 | 0.14762641 | 150.0130585 | 220.8780683 |
| YJR098C | YJR098C | 0.47802018 | 0.99917121 | 0.1476587 | 143.6925362 | 210.8095644 |
| YLR176C | RFX1 | 0.44711182 | 0.99917121 | 0.14796413 | 147.9128257 | 217.4925629 |
| YOR189W | IES4 | 0.41032881 | 0.99917121 | 0.14832867 | 143.7830204 | 210.8719709 |
| YGL035C | MIG1 | 0.64928095 | 0.99917121 | 0.14835491 | 136.445082 | 199.184107 |
| YER106W | MAM1 | 0.3984762 | 0.99917121 | 0.14860015 | 134.1637221 | 195.5214556 |
| YPR017C | DSS4 | 0.6023029 | 0.99917121 | 0.14883517 | 134.5803381 | 196.1562091 |
| YKR058W | GLG1 | 0.45684668 | 0.99917121 | 0.14902104 | 153.1222558 | 225.659021 |
| YML120C | NDI1 | 0.23823458 | 0.99917121 | 0.14909618 | 143.1978745 | 209.8466384 |
| YKL162C | YKL162C | 0.2385308 | 0.99917121 | 0.14930521 | 138.223538 | 201.9002035 |
| YIL103W | DPH1 | 0.4839414 | 0.99917121 | 0.14967108 | 147.3149206 | 216.332385 |
| YNR073C | YNR073C | 0.40210552 | 0.99917121 | 0.14990391 | 144.3515545 | 211.585245 |
| YPR154W | PIN3 | 0.37232087 | 0.99917121 | 0.15001557 | 139.5010262 | 203.8478287 |
| YMR008C | PLB1 | 0.03304439 | 0.94753174 | 0.15003686 | 133.4367608 | 194.1887185 |
| YLR144C | ACF2 | 0.21410495 | 0.99917121 | 0.15021382 | 132.2604995 | 192.2941109 |
| YLR079W | SIC1 | 0.60906509 | 0.99917121 | 0.15031858 | 115.3713479 | 165.387672 |
| YBR051W | YBR051W | 0.3567881 | 0.99917121 | 0.15044851 | 153.0352243 | 225.3464112 |
| YPR171W | BSP1 | 0.32567726 | 0.99917121 | 0.1505025 | 146.9085332 | 215.5839102 |
| YMR121C | RPL15B | 0.3395167 | 0.99917121 | 0.15062233 | 131.8244959 | 191.5500325 |
| YML118W | NGL3 | 0.15966189 | 0.99381655 | 0.15062759 | 139.0111402 | 202.9931409 |
| YNR027W | BUD17 | 0.3486005 | 0.99917121 | 0.15067564 | 143.3687328 | 209.9261532 |
| YFL013C | IES1 | 0.76047343 | 0.99917121 | 0.15086374 | 33.04357823 | 34.22548242 |
| YLR164W | YLR164W | 0.48156949 | 0.99917121 | 0.15119975 | 143.4754922 | 210.0322588 |
| YMR210W | YMR210W | 0.19565478 | 0.99917121 | 0.15122099 | 138.581248 | 202.2362548 |
| YBR296C | PHO89 | 0.47202375 | 0.99917121 | 0.15143456 | 159.9336286 | 236.210967 |
| YLR042C | YLR042C | 0.62026131 | 0.99917121 | 0.15145843 | 124.0806261 | 179.1170482 |
| YPR004C | YPR004C | 0.37929254 | 0.99917121 | 0.15147963 | 135.7774398 | 197.7400413 |
| YOR171C | LCB4 | 0.21070913 | 0.99917121 | 0.15155835 | 144.598495 | 211.7767709 |
| YDR459C | PFA5 | 0.3019173 | 0.99917121 | 0.1515662 | 139.133396 | 203.0733898 |
| YMR215W | GAS3 | 0.36143484 | 0.99917121 | 0.15162012 | 143.0247752 | 209.2633057 |
| YOR091W | TMA46 | 0.55838353 | 0.99917121 | 0.15162856 | 144.5789249 | 211.7370482 |
| YBR220C | YBR220C | 0.30724967 | 0.99917121 | 0.15173832 | 147.1518303 | 215.8206682 |
| YGL234W | ADE5 | 0.257664 | 0.99917121 | 0.15179501 | 142.159871 | 207.864743 |
| YPR060C | ARO7 | 0.13905038 | 0.97853666 | 0.15198775 | 93.34755248 | 130.1142964 |
| YBL052C | SAS3 | 0.38137231 | 0.99917121 | 0.15211596 | 145.6984915 | 213.4603859 |
| YIL009C-A | EST3 | 0.44607214 | 0.99917121 | 0.15213831 | 149.5806292 | 219.6394348 |
| YGR070W | ROM1 | 0.36387794 | 0.99917121 | 0.15222496 | 147.7563911 | 216.7240219 |
| YLR247C | IRC20 | 0.37342857 | 0.99917121 | 0.15225126 | 149.7013243 | 219.8178557 |
| YKL065C | YET1 | 0.16434836 | 0.99381655 | 0.15239943 | 132.1841724 | 191.9061204 |

| | | | | | | |
|---|---|---|---|---|---|---|
| YJL073W | JEM1 | 0.31666555 | 0.99917121 | 0.1526013 | 143.4466279 | 209.8154329 |
| YCR061W | YCR061W | 0.47141989 | 0.99917121 | 0.15261601 | 133.3549655 | 193.7440455 |
| YCR073W-A | SOL2 | 0.18469929 | 0.99917121 | 0.15262422 | 144.8170145 | 211.9947926 |
| YIL025C | YIL025C | 0.46550861 | 0.99917121 | 0.15275991 | 149.3769563 | 219.2393341 |
| YML004C | GLO1 | 0.36376342 | 0.99917121 | 0.15283257 | 135.4096267 | 196.9894112 |
| YHR112C | YHR112C | 0.39011191 | 0.99917121 | 0.15284252 | 158.4174065 | 233.6249458 |
| YLR236C | YLR236C | 0.46400007 | 0.99917121 | 0.15289117 | 157.5415841 | 232.2243875 |
| YDR481C | PHO8 | 0.31263679 | 0.99917121 | 0.15303629 | 142.4832475 | 208.2283507 |
| YIL089W | YIL089W | 0.36221421 | 0.99917121 | 0.15304821 | 145.6778513 | 213.3138684 |
| YGL071W | AFT1 | 0.41228946 | 0.99917121 | 0.15316413 | 147.6772068 | 216.4834373 |
| YDL136W | RPL35B | 0.55598501 | 0.99917121 | 0.15335835 | 134.0381554 | 194.741433 |
| YFL010W-A | AUA1 | 0.37463525 | 0.99917121 | 0.1533587 | 135.3450683 | 196.8224698 |
| YKL075C | YKL075C | 0.24780037 | 0.99917121 | 0.15352816 | 134.6286566 | 195.6610238 |
| YAR018C | KIN3 | 0.41096424 | 0.99917121 | 0.15357264 | 154.452036 | 227.221625 |
| YOL030W | GAS5 | 0.41664044 | 0.99917121 | 0.15373782 | 137.8816263 | 200.815374 |
| YFR007W | YFR007W | 0.23677305 | 0.99917121 | 0.15374245 | 143.4255021 | 209.6426742 |
| YOR132W | VPS17 | 0.49865437 | 0.99917121 | 0.15376033 | 149.3450529 | 219.0665705 |
| YGR291C | YGR291C | 0.39627524 | 0.99917121 | 0.15406388 | 135.8700257 | 197.5724243 |
| YMR095C | SNO1 | 0.2489137 | 0.99917121 | 0.15414594 | 135.8421561 | 197.5180423 |
| YKL015W | PUT3 | 0.13567246 | 0.97853666 | 0.15441941 | 143.8914185 | 210.3020536 |
| YLL061W | MMP1 | 0.48009742 | 0.99917121 | 0.15445335 | 141.5496891 | 206.5690324 |
| YDL218W | YDL218W | 0.58796071 | 0.99917121 | 0.15445684 | 164.3996427 | 242.9540372 |
| YPR098C | YPR098C | 0.25853382 | 0.99917121 | 0.15446895 | 133.407448 | 193.6017236 |
| YLR028C | ADE16 | 0.49561401 | 0.99917121 | 0.15487186 | 130.5433782 | 188.9919646 |
| YBL013W | FMT1 | 0.23448643 | 0.99917121 | 0.15488411 | 145.0230094 | 212.0473055 |
| YPR009W | SUT2 | 0.32479842 | 0.99917121 | 0.15493187 | 133.8756927 | 194.2909042 |
| YLR255C | YLR255C | 0.48118235 | 0.99917121 | 0.15500591 | 120.7678972 | 173.4095036 |
| YMR111C | YMR111C | 0.41752796 | 0.99917121 | 0.15522711 | 133.6916871 | 193.9619081 |
| YHR049W | FSH1 | 0.11590996 | 0.95714881 | 0.15545808 | 161.0706107 | 237.5309461 |
| YHR078W | YHR078W | 0.39693417 | 0.99917121 | 0.15548316 | 156.9383525 | 230.9478322 |
| YNL053W | MSG5 | 0.50636762 | 0.99917121 | 0.15584199 | 140.5134132 | 204.7496145 |
| YDL061C | RPS29B | 0.33984021 | 0.99917121 | 0.15594665 | 94.62357988 | 131.6635627 |
| YJR110W | YMR1 | 0.56725824 | 0.99917121 | 0.15596282 | 141.6588781 | 206.5588797 |
| YMR042W | ARG80 | 0.14108884 | 0.97853666 | 0.15601029 | 137.0953549 | 199.2863054 |
| YEL049W | PAU2 | 0.35457509 | 0.99917121 | 0.15609557 | 134.3604694 | 194.9209776 |
| YER066C-A | YER066C-A | 0.27954204 | 0.99917121 | 0.15624816 | 139.5951528 | 203.2378929 |
| YGR205W | YGR205W | 0.43828421 | 0.99917121 | 0.15641329 | 152.0093833 | 222.9857282 |
| YER049W | TPA1 | 0.2028539 | 0.99917121 | 0.15652185 | 144.2646159 | 210.64001 |
| YJR090C | GRR1 | 0.7998967 | 0.99917121 | 0.15653064 | 37.20925996 | 40.1679054 |
| YLR122C | YLR122C | 0.44135394 | 0.99917121 | 0.15653912 | 140.5607956 | 204.7400759 |
| YLR121C | YPS3 | 0.35752794 | 0.99917121 | 0.15654397 | 161.4216827 | 237.957599 |
| YER060W-A | FCY22 | 0.15952775 | 0.99381655 | 0.15669189 | 118.6656052 | 169.8563517 |
| YLR380W | CSR1 | 0.44545998 | 0.99917121 | 0.15680892 | 140.7825076 | 205.0602308 |
| YMR167W | MLH1 | 0.14830909 | 0.98328895 | 0.15715657 | 132.3732623 | 191.6272742 |
| YGL179C | TOS3 | 0.12736379 | 0.97616733 | 0.15740367 | 135.8014686 | 197.0561005 |
| YGL017W | ATE1 | 0.21821018 | 0.99917121 | 0.15741275 | 146.5325239 | 214.1427339 |
| YMR007W | YMR007W | 0.15167375 | 0.98381735 | 0.15762336 | 136.8201028 | 198.6513531 |
| YGL232W | TAN1 | 0.2190741 | 0.99917121 | 0.15770651 | 158.4624732 | 233.1037354 |
| YPR064W | YPR064W | 0.37149924 | 0.99917121 | 0.15779575 | 136.5673136 | 198.2278052 |
| YML122C | YML122C | 0.28235004 | 0.99917121 | 0.15785813 | 141.5233474 | 206.1120068 |
| YER111C | SWI4 | 0.3322473 | 0.99917121 | 0.15786581 | 137.2897757 | 199.3696867 |
| YLR031W | YLR031W | 0.22815602 | 0.99917121 | 0.15786749 | 142.7408866 | 208.0496311 |
| YMR237W | BCH1 | 0.21896333 | 0.99917121 | 0.15798488 | 138.4718053 | 201.2373915 |
| YLR315W | NKP2 | 0.25757391 | 0.99917121 | 0.15807427 | 131.8873337 | 190.7416219 |
| YBR005W | RCR1 | 0.27164324 | 0.99917121 | 0.15817564 | 149.8716107 | 219.3667684 |
| YER097W | YER097W | 0.12674931 | 0.97616733 | 0.1582501 | 140.2066658 | 203.9675859 |
| YMR105C | PGM2 | 0.04627365 | 0.94753174 | 0.15829328 | 136.5483678 | 198.1369821 |
| YKL159C | RCN1 | 0.35170999 | 0.99917121 | 0.15835128 | 143.3664236 | 208.986734 |
| YNR028W | CPR8 | 0.43174492 | 0.99917121 | 0.15856872 | 145.5377829 | 212.4178192 |
| YLR461W | PAU4 | 0.18225824 | 0.99917121 | 0.15863673 | 133.9463039 | 193.9516812 |
| YOR138C | RUP1 | 0.44052542 | 0.99917121 | 0.15879002 | 158.5149013 | 233.0551285 |
| YDR101C | ARX1 | 0.33728888 | 0.99917121 | 0.15890619 | 120.3790457 | 172.3148246 |
| YFR023W | PES4 | 0.36440708 | 0.99917121 | 0.15903075 | 151.7305515 | 222.2226313 |
| YLL054C | YLL054C | 0.13033098 | 0.97616733 | 0.15903911 | 131.3429728 | 189.7571776 |

| | | | | | | |
|---|---|---|---|---|---|---|
| YMR075C-A | YMR075C-A | 0.10513671 | 0.94753174 | 0.15906589 | 122.0115593 | 174.8949106 |
| YPR076W | YPR076W | 0.35157376 | 0.99917121 | 0.15923866 | 144.1119231 | 210.0656595 |
| YEL052W | AFG1 | 0.06313192 | 0.94753174 | 0.15944414 | 142.4658528 | 207.4194669 |
| YGL218W | YGL218W | 0.80261031 | 0.99917121 | 0.15969183 | 40.69761672 | 45.33725509 |
| YOR154W | SLP1 | 0.37472296 | 0.99917121 | 0.15972123 | 139.9434902 | 203.3691686 |
| YGR208W | SER2 | 0.5042228 | 0.99917121 | 0.15982376 | 140.8460892 | 204.7939343 |
| YMR178W | YMR178W | 0.1017541 | 0.94753174 | 0.15987793 | 140.0307138 | 203.4889574 |
| YDL231C | BRE4 | 0.49891578 | 0.99917121 | 0.15996208 | 147.5684476 | 215.4815103 |
| YPR068C | HOS1 | 0.38782063 | 0.99917121 | 0.16011268 | 138.748984 | 201.4193584 |
| YGR255C | COQ6 | 0.43437605 | 0.99917121 | 0.16030876 | 132.0272553 | 190.6920198 |
| YLR136C | TIS11 | 0.36949274 | 0.99917121 | 0.16039977 | 140.8584255 | 204.7433564 |
| YOR284W | HUA2 | 0.53454672 | 0.99917121 | 0.16043299 | 145.3174554 | 211.8397029 |
| YOR021C | YOR021C | 0.32991602 | 0.99917121 | 0.16047867 | 152.5468613 | 223.3459753 |
| YAR047C | YAR047C | 0.21884742 | 0.99917121 | 0.16070103 | 138.0238193 | 200.1929068 |
| YLR406C | RPL31B | 0.44672918 | 0.99917121 | 0.16070848 | 137.1552869 | 198.8089796 |
| YDR227W | SIR4 | 0.20469533 | 0.99917121 | 0.16072915 | 144.7495772 | 210.8993291 |
| YGL057C | YGL057C | 0.31424403 | 0.99917121 | 0.16081797 | 143.2837571 | 208.554383 |
| YPL111W | CAR1 | 0.41331762 | 0.99917121 | 0.1608543 | 146.4168604 | 213.538993 |
| YDR035W | ARO3 | 0.41428123 | 0.99917121 | 0.1608794 | 149.19877 | 217.9657448 |
| YNL219C | ALG9 | 0.25491864 | 0.99917121 | 0.16125412 | 128.013664 | 184.1856743 |
| YJL066C | MPM1 | 0.37465342 | 0.99917121 | 0.16147038 | 146.9281496 | 214.2780445 |
| YLR064W | YLR064W | 0.16232862 | 0.99381655 | 0.16148699 | 134.0778058 | 193.8136023 |
| YEL017C-A | PMP2 | 0.30075888 | 0.99917121 | 0.16149152 | 152.3420797 | 222.8964111 |
| YNR022C | MRPL50 | 0.54982555 | 0.99917121 | 0.16153209 | 145.2731441 | 211.6351518 |
| YLR013W | GAT3 | 0.42220393 | 0.99917121 | 0.16169746 | 150.0310463 | 219.1913007 |
| YML112W | CTK3 | 0.28843611 | 0.99917121 | 0.16181264 | 138.580317 | 200.9435369 |
| YKR033C | YKR033C | 0.24215481 | 0.99917121 | 0.16189091 | 141.8857257 | 206.1974065 |
| YIL034C | CAP2 | 0.16506268 | 0.99381655 | 0.16193434 | 140.3688054 | 203.7766237 |
| YOL004W | SIN3 | 0.34320251 | 0.99917121 | 0.16198813 | 109.7276494 | 154.9782096 |
| YER119C | AVT6 | 0.28351908 | 0.99917121 | 0.16213795 | 141.4524411 | 205.4773427 |
| YDL127W | PCL2 | 0.28800167 | 0.99917121 | 0.16245228 | 160.5263257 | 235.8115791 |
| YDR030C | RAD28 | 0.41372529 | 0.99917121 | 0.16249991 | 136.5470387 | 197.6220323 |
| YCR050C | YCR050C | 0.37286954 | 0.99917121 | 0.16251367 | 139.3573098 | 202.0953278 |
| YEL059W | YEL059W | 0.11486396 | 0.95531631 | 0.1625171 | 111.4464789 | 157.6507238 |
| YGR122C-A | YGR122C-A | 0.28088453 | 0.99917121 | 0.16273261 | 138.5934861 | 200.8523524 |
| YFL025C | BST1 | 0.37079892 | 0.99917121 | 0.16282463 | 139.5320372 | 202.3356481 |
| YER151C | UBP3 | 0.19438556 | 0.99917121 | 0.16292106 | 105.1039333 | 147.5018392 |
| YLR273C | PIG1 | 0.22061428 | 0.99917121 | 0.16301475 | 155.4906213 | 227.7243362 |
| YDR043C | NRG1 | 0.3716704 | 0.99917121 | 0.16304311 | 129.0151891 | 185.5623687 |
| YKL038W | RGT1 | 0.10017565 | 0.94753174 | 0.1635228 | 131.9373564 | 190.1570423 |
| YGR133W | PEX4 | 0.18470662 | 0.99917121 | 0.16364846 | 141.7921626 | 205.8341559 |
| YOL056W | GPM3 | 0.4972359 | 0.99917121 | 0.16369294 | 142.8470029 | 207.5084217 |
| YFL031W | HAC1 | 0.21410278 | 0.99917121 | 0.16372655 | 137.5894725 | 199.1324257 |
| YGL062W | PYC1 | 0.29394697 | 0.99917121 | 0.16373007 | 156.5985472 | 229.4013518 |
| YAL046C | YAL046C | 0.32607278 | 0.99917121 | 0.16401392 | 134.2072842 | 193.7117193 |
| YGR177C | ATF2 | 0.19848913 | 0.99917121 | 0.16425547 | 153.4187685 | 224.2739369 |
| YCL013W | YCL013W | 0.43528236 | 0.99917121 | 0.16428609 | 159.4689545 | 233.9042984 |
| YPL108W | YPL108W | 0.17662945 | 0.99917121 | 0.16440234 | 141.4139097 | 205.139933 |
| YPR071W | YPR071W | 0.23437231 | 0.99917121 | 0.16447508 | 148.4730197 | 216.3717341 |
| YPL001W | HAT1 | 0.00711783 | 0.84351098 | 0.16455975 | 136.6615402 | 197.5532446 |
| YML018C | YML018C | 0.37401454 | 0.99917121 | 0.16457779 | 132.9372988 | 191.6206993 |
| YOR044W | IRC23 | 0.4617399 | 0.99917121 | 0.16458837 | 157.1758705 | 230.2160241 |
| YBR200W | BEM1 | 0.63819607 | 0.99917121 | 0.16479671 | 118.7885915 | 169.0641268 |
| YOR186W | YOR186W | 0.29732884 | 0.99917121 | 0.16480585 | 139.5145555 | 202.0662787 |
| YKL044W | YKL044W | 0.18394508 | 0.99917121 | 0.16495617 | 143.0748351 | 207.7172122 |
| YOR100C | CRC1 | 0.41923685 | 0.99917121 | 0.16503385 | 141.1575281 | 204.6546927 |
| YIL060W | YIL060W | 0.29445458 | 0.99917121 | 0.16515458 | 146.8054962 | 213.6335931 |
| YCR069W | CPR4 | 0.40570057 | 0.99917121 | 0.16517582 | 155.0993172 | 226.8377807 |
| YGL027C | CWH41 | 0.34886135 | 0.99917121 | 0.16519414 | 148.449651 | 216.2468616 |
| YCL023C | YCL023C | 0.21292032 | 0.99917121 | 0.16520025 | 160.3873155 | 235.2552172 |
| YIL071C | PCI8 | 0.32963677 | 0.99917121 | 0.1652089 | 157.6385135 | 230.8770708 |
| YOR112W | YOR112W | 0.62098657 | 0.99917121 | 0.16525011 | 149.8219933 | 218.4253061 |
| YCR049C | YCR049C | 0.35156599 | 0.99917121 | 0.16536406 | 156.3827701 | 228.8585558 |
| YML128C | MSC1 | 0.483611 | 0.99917121 | 0.16540436 | 113.0069625 | 159.7835925 |

| | | | | | | |
|---|---|---|---|---|---|---|
| YKR047W | YKR047W | 0.14307247 | 0.98199783 | 0.16549744 | 130.2447021 | 187.2209921 |
| YDR453C | TSA2 | 0.39284729 | 0.99917121 | 0.1655804 | 154.530038 | 225.8819578 |
| YDL183C | YDL183C | 0.43273713 | 0.99917121 | 0.16595185 | 109.9988935 | 154.9269092 |
| YGR031W | YGR031W | 0.35413961 | 0.99917121 | 0.16630791 | 149.7860851 | 218.2391699 |
| YLR385C | SWC7 | 0.4568109 | 0.99917121 | 0.16646491 | 142.6335996 | 206.8306741 |
| YGR054W | YGR054W | 0.25065346 | 0.99917121 | 0.16660131 | 150.5695555 | 219.4509714 |
| YBR071W | YBR071W | 0.32541866 | 0.99917121 | 0.16687719 | 145.446803 | 211.2600549 |
| YLR046C | YLR046C | 0.38345281 | 0.99917121 | 0.16695342 | 149.984596 | 218.4765766 |
| YEL023C | YEL023C | 0.22641502 | 0.99917121 | 0.1669712 | 140.4250082 | 203.2520716 |
| YJL150W | YJL150W | 0.26346556 | 0.99917121 | 0.16805185 | 142.9434846 | 207.1306584 |
| YLR095C | IOC2 | 0.34330036 | 0.99917121 | 0.1683775 | 115.1444485 | 162.8247901 |
| YGR021W | YGR021W | 0.16896449 | 0.99636201 | 0.16854693 | 138.9227175 | 200.6677809 |
| YDL039C | PRM7 | 0.34388433 | 0.99917121 | 0.1686703 | 151.1484355 | 220.1205258 |
| YBL019W | APN2 | 0.27889832 | 0.99917121 | 0.16880417 | 145.5960037 | 211.2627169 |
| YKL156W | RPS27A | 0.13048907 | 0.97616733 | 0.16889083 | 124.914186 | 178.3191821 |
| YGR271W | SLH1 | 0.35543091 | 0.99917121 | 0.16899866 | 136.6998371 | 197.0730764 |
| YBR125C | PTC4 | 0.25584734 | 0.99917121 | 0.16901568 | 153.8825573 | 224.432137 |
| YPL057C | SUR1 | 0.18631803 | 0.99917121 | 0.16944233 | 132.2390215 | 189.915748 |
| YCL042W | YCL042W | 0.3114375 | 0.99917121 | 0.16947655 | 153.3617091 | 223.5465722 |
| YLR057W | YLR057W | 0.22029321 | 0.99917121 | 0.16993282 | 144.7203294 | 209.73073 |
| YPR011C | YPR011C | 0.35691646 | 0.99917121 | 0.17016392 | 151.0757847 | 219.8227516 |
| YNL012W | SPO1 | 0.32122658 | 0.99917121 | 0.17025113 | 162.8898981 | 238.6244823 |
| YLR180W | SAM1 | 0.21928791 | 0.99917121 | 0.17026619 | 132.3710332 | 190.0255219 |
| YPR140W | TAZ1 | 0.36490571 | 0.99917121 | 0.17027554 | 142.7016798 | 206.4745255 |
| YER035W | EDC2 | 0.10687926 | 0.9482222 | 0.17038839 | 144.2703909 | 208.9587265 |
| YLR436C | ECM30 | 0.14645195 | 0.98328895 | 0.17043499 | 128.4687792 | 183.7911372 |
| YLL005C | SPO75 | 0.617744 | 0.99917121 | 0.17081171 | 153.7306254 | 223.97125 |
| YMR075W | RCO1 | 0.28165627 | 0.99917121 | 0.17087392 | 126.3114548 | 180.3023821 |
| YHR135C | YCK1 | 0.30224151 | 0.99917121 | 0.17089576 | 154.1525433 | 224.6328499 |
| YML066C | SMA2 | 0.58541687 | 0.99917121 | 0.17092083 | 130.8469724 | 187.5188559 |
| YNL167C | SKO1 | 0.07420995 | 0.94753174 | 0.17100779 | 133.1616251 | 191.1940229 |
| YNL311C | SKP2 | 0.30065573 | 0.99917121 | 0.1712047 | 133.1554254 | 191.1601445 |
| YLL019C | KNS1 | 0.01123072 | 0.84351098 | 0.17142465 | 136.3466251 | 196.2148804 |
| YOR247W | SRL1 | 0.34358356 | 0.99917121 | 0.17146624 | 157.7160034 | 230.2376258 |
| YOR058C | ASE1 | 0.29595562 | 0.99917121 | 0.17148646 | 149.190885 | 216.6600742 |
| YLR087C | CSF1 | 0.24362469 | 0.99917121 | 0.17151799 | 127.454791 | 182.0444699 |
| YFR057W | YFR057W | 0.18586219 | 0.99917121 | 0.1716591 | 156.3850923 | 228.0948209 |
| YFR008W | FAR7 | 0.2066803 | 0.99917121 | 0.17181581 | 142.4893529 | 205.9486485 |
| YPL269W | KAR9 | 0.48667239 | 0.99917121 | 0.17196739 | 140.9398376 | 203.4627785 |
| YDR046C | BAP3 | 0.27483427 | 0.99917121 | 0.17199205 | 134.9314503 | 193.8922349 |
| YLR278C | YLR278C | 0.23360654 | 0.99917121 | 0.17219552 | 141.4244926 | 204.2067138 |
| YGL049C | TIF4632 | 0.41877291 | 0.99917121 | 0.17233476 | 139.8017964 | 201.6058162 |
| YBR245C | ISW1 | 0.64975896 | 0.99917121 | 0.17241875 | 167.4185911 | 245.5715513 |
| YOR079C | ATX2 | 0.24673543 | 0.99917121 | 0.17242047 | 153.9076105 | 224.0569505 |
| YMR180C | CTL1 | 0.24811031 | 0.99917121 | 0.17246275 | 139.0740236 | 200.4313346 |
| YDR309C | GIC2 | 0.19199083 | 0.99917121 | 0.17257557 | 150.5092199 | 218.6265685 |
| YBR159W | IFA38 | 0.19036768 | 0.99917121 | 0.17285954 | 146.5926644 | 212.3553702 |
| YFR024C-A | LSB3 | 0.22337173 | 0.99917121 | 0.17289442 | 152.8755666 | 222.3557814 |
| YLR268W | SEC22 | 0.33321577 | 0.99917121 | 0.1729554 | 122.0088362 | 173.1972951 |
| YPL267W | ACM1 | 0.28521056 | 0.99917121 | 0.17305871 | 144.3624079 | 208.7797107 |
| YGL176C | YGL176C | 0.20099455 | 0.99917121 | 0.17306685 | 153.5118149 | 223.3478987 |
| YJR117W | STE24 | 0.3859598 | 0.99917121 | 0.17322889 | 150.4343669 | 218.4277282 |
| YMR201C | RAD14 | 0.0328671 | 0.94753174 | 0.17340145 | 134.8970376 | 193.6656169 |
| YGR049W | SCM4 | 0.29298361 | 0.99917121 | 0.17345205 | 160.5252644 | 234.4688998 |
| YOR011W | AUS1 | 0.27669546 | 0.99917121 | 0.17345502 | 149.087948 | 216.2561729 |
| YLR238W | FAR10 | 0.39426099 | 0.99917121 | 0.17354802 | 145.0913299 | 209.8807664 |
| YBL096C | YBL096C | 0.25831572 | 0.99917121 | 0.17366412 | 154.0426457 | 224.1203608 |
| YML109W | ZDS2 | 0.1473954 | 0.98328895 | 0.17373983 | 144.2738199 | 208.5556103 |
| YAL014C | SYN8 | 0.10335116 | 0.94753174 | 0.1737561 | 157.3053964 | 229.3046321 |
| YIL085C | KTR7 | 0.35330453 | 0.99917121 | 0.17380806 | 143.7575979 | 207.7252792 |
| YMR153C-A | YMR153C-A | 0.27440354 | 0.99917121 | 0.17423869 | 134.3245089 | 192.651875 |
| YLR290C | YLR290C | 0.04457284 | 0.94753174 | 0.17466716 | 125.157464 | 178.0023735 |
| YMR119W | ASI1 | 0.11431843 | 0.95531631 | 0.17469433 | 148.5104141 | 215.1854442 |
| YPR002W | PDH1 | 0.42376284 | 0.99917121 | 0.17491452 | 137.7125442 | 197.9644671 |

| | | | | | | |
|---|---|---|---|---|---|---|
| YLR233C | EST1 | 0.01245952 | 0.84351098 | 0.17493991 | 137.1370656 | 197.0450012 |
| YOR067C | ALG8 | 0.41145836 | 0.99917121 | 0.17510919 | 136.8863221 | 196.6250896 |
| YIL158W | YIL158W | 0.44826867 | 0.99917121 | 0.17518616 | 128.4717231 | 183.2166061 |
| YHR193C | EGD2 | 0.4454881 | 0.99917121 | 0.17524349 | 177.4930653 | 261.2694089 |
| YPR132W | RPS23B | 0.5133825 | 0.99917121 | 0.17540697 | 135.4554168 | 194.3102647 |
| YKL116C | PRR1 | 0.0428054 | 0.94753174 | 0.17556589 | 143.1869042 | 206.6022285 |
| YDL130W-A | STF1 | 0.19674736 | 0.99917121 | 0.17587251 | 149.5283699 | 216.6627666 |
| YMR132C | JLP2 | 0.09083434 | 0.94753174 | 0.17611568 | 136.015226 | 195.1152857 |
| YGR207C | YGR207C | 0.19980689 | 0.99917121 | 0.17653159 | 153.5655674 | 223.0111038 |
| YNL191W | DUG3 | 0.11734868 | 0.9579757 | 0.17686061 | 126.441272 | 179.7792566 |
| YLL059C | YLL059C | 0.44933357 | 0.99917121 | 0.17727095 | 133.0921986 | 190.3199246 |
| YPR191W | QCR2 | 0.71295473 | 0.99917121 | 0.17730688 | 75.38172253 | 98.41949236 |
| YLL056C | YLL056C | 0.21145259 | 0.99917121 | 0.1774086 | 141.7002795 | 204.0103364 |
| YFL050C | ALR2 | 0.0841863 | 0.94753174 | 0.17753878 | 139.7435662 | 200.8786667 |
| YMR278W | YMR278W | 0.07398715 | 0.94753174 | 0.17779647 | 138.9251876 | 199.5440955 |
| YIL151C | YIL151C | 0.37093596 | 0.99917121 | 0.17826735 | 144.1098559 | 207.7425667 |
| YKR077W | YKR077W | 0.14154556 | 0.97853666 | 0.17842251 | 136.0617335 | 194.9081154 |
| YGL136C | MRM2 | 0.28847813 | 0.99917121 | 0.17887933 | 141.8867139 | 204.1279093 |
| YAL036C | RBG1 | 0.38227283 | 0.99917121 | 0.17891811 | 153.2818117 | 222.2683188 |
| YJL105W | SET4 | 0.32275074 | 0.99917121 | 0.17910266 | 138.8907966 | 199.3300949 |
| YGL131C | SNT2 | 0.23611908 | 0.99917121 | 0.17910529 | 155.2251036 | 225.3399263 |
| YLR328W | NMA1 | 0.50079434 | 0.99917121 | 0.17913374 | 140.3081019 | 201.5831704 |
| YKR016W | FMP13 | 0.39103151 | 0.99917121 | 0.17929891 | 142.1598863 | 204.5117477 |
| YER088C | DOT6 | 0.16842602 | 0.99636201 | 0.17944341 | 143.8863651 | 207.2433144 |
| YOR345C | YOR345C | 0.37055182 | 0.99917121 | 0.17952278 | 142.3098906 | 204.7233175 |
| YDR363W | ESC2 | 0.4145702 | 0.99917121 | 0.17952535 | 122.7652254 | 173.6007938 |
| YOR309C | YOR309C | 0.20723993 | 0.99917121 | 0.17983444 | 121.7731916 | 171.9834349 |
| YPR174C | YPR174C | 0.22726175 | 0.99917121 | 0.17992447 | 149.0352321 | 215.3835343 |
| YER010C | YER010C | 0.20987364 | 0.99917121 | 0.18000218 | 152.4800261 | 220.8594257 |
| YGR061C | ADE6 | 0.36884255 | 0.99917121 | 0.18028366 | 143.4024323 | 206.3702814 |
| YGR212W | SLI1 | 0.36585596 | 0.99917121 | 0.18052782 | 155.4262036 | 225.4867293 |
| YDL134C | PPH21 | 0.46065168 | 0.99917121 | 0.18069763 | 149.2437596 | 215.6213299 |
| YER065C | ICL1 | 0.21470315 | 0.99917121 | 0.1807247 | 135.8821035 | 194.3414179 |
| YNL299W | TRF5 | 0.14026243 | 0.97853666 | 0.18077981 | 121.3219588 | 171.1496568 |
| YCR005C | CIT2 | 0.07764008 | 0.94753174 | 0.18094432 | 141.0938299 | 202.6136065 |
| YLL025W | PAU17 | 0.11540494 | 0.95531631 | 0.18132433 | 148.2089555 | 213.8971449 |
| YAL064C-A | YAL064C-A | 0.13382659 | 0.97853666 | 0.18133445 | 151.4307169 | 219.0261257 |
| YPR111W | DBF20 | 0.26196461 | 0.99917121 | 0.18138487 | 142.1320474 | 204.2131177 |
| YJL065C | DLS1 | 0.2531261 | 0.99917121 | 0.18141849 | 151.5863438 | 219.2636943 |
| YDR174W | HMO1 | 0.10206377 | 0.94753174 | 0.18145754 | 90.6946101 | 122.2971656 |
| YBR066C | NRG2 | 0.15397214 | 0.98492771 | 0.18177602 | 149.2746265 | 215.539014 |
| YPR197C | YPR197C | 0.45182621 | 0.99917121 | 0.18189376 | 145.9615439 | 210.2490288 |
| YLR299W | ECM38 | 0.25225516 | 0.99917121 | 0.18201593 | 142.2992471 | 204.4024278 |
| YBR298C | MAL31 | 0.29320583 | 0.99917121 | 0.18214482 | 139.9108585 | 200.5835319 |
| YIL096C | YIL096C | 0.09846555 | 0.94753174 | 0.18214927 | 150.9556732 | 218.1703485 |
| YLR335W | NUP2 | 0.25327791 | 0.99917121 | 0.18243321 | 135.6898385 | 193.8269767 |
| YDL137W | ARF2 | 0.26372224 | 0.99917121 | 0.18257628 | 152.752111 | 220.9788732 |
| YMR223W | UBP8 | 0.00827021 | 0.84351098 | 0.18269406 | 117.551554 | 164.9124355 |
| YER174C | GRX4 | 0.38114339 | 0.99917121 | 0.1827053 | 173.0759148 | 253.3260262 |
| YGL252C | RTG2 | 0.33191347 | 0.99917121 | 0.18277344 | 127.8522532 | 181.3052148 |
| YDL041W | YDL041W | 0.18386347 | 0.99917121 | 0.18304868 | 145.210867 | 208.9128814 |
| YPR059C | YPR059C | 0.31433612 | 0.99917121 | 0.18313293 | 135.6074595 | 193.6104953 |
| YLL028W | TPO1 | 0.12023106 | 0.96279357 | 0.18332549 | 139.4068086 | 199.6369653 |
| YFR049W | YMR31 | 0.15060327 | 0.98328895 | 0.1835842 | 144.7253088 | 208.0744104 |
| YGR192C | TDH3 | 0.1195511 | 0.96096398 | 0.18361671 | 142.0175892 | 203.7587734 |
| YBR292C | YBR292C | 0.2276867 | 0.99917121 | 0.18375376 | 155.1499415 | 224.6535428 |
| YGL036W | YGL036W | 0.27851737 | 0.99917121 | 0.18378354 | 134.3105042 | 191.465955 |
| YBR126C | TPS1 | 0.45311492 | 0.99917121 | 0.18425214 | 128.9173084 | 182.8208997 |
| YOR191W | RIS1 | 0.31145745 | 0.99917121 | 0.18472165 | 134.9931267 | 192.4385724 |
| YER011W | TIR1 | 0.37034354 | 0.99917121 | 0.18484782 | 154.3690682 | 223.2767311 |
| YBR230C | OM14 | 0.29637854 | 0.99917121 | 0.1848628 | 152.4339911 | 220.1935589 |
| YMR194C-A | YMR194C-A | 0.46439404 | 0.99917121 | 0.18512693 | 139.2129311 | 199.1086263 |
| YGL242C | YGL242C | 0.23887064 | 0.99917121 | 0.18532139 | 164.2467992 | 238.9479352 |
| YKL053W | YKL053W | 0.1362743 | 0.97853666 | 0.18535595 | 127.6752087 | 180.7084589 |

| | | | | | | |
|---|---|---|---|---|---|---|
| YMR114C | YMR114C | 0.09252212 | 0.94753174 | 0.18536895 | 135.6231461 | 193.3628799 |
| YIL136W | OM45 | 0.33448907 | 0.99917121 | 0.1853826 | 143.0785066 | 205.2328586 |
| YOR090C | PTC5 | 0.09694297 | 0.94753174 | 0.18557814 | 137.1352706 | 195.745229 |
| YBR259W | YBR259W | 0.23526082 | 0.99917121 | 0.18561117 | 150.1697451 | 216.496823 |
| YML052W | SUR7 | 0.09645712 | 0.94753174 | 0.18569889 | 136.3822514 | 194.5314279 |
| YKL091C | YKL091C | 0.33047334 | 0.99917121 | 0.18586621 | 145.2014007 | 208.5543203 |
| YER144C | UBP5 | 0.17330043 | 0.99917121 | 0.1861092 | 140.9989379 | 201.8328491 |
| YPR003C | YPR003C | 0.3776037 | 0.99917121 | 0.18650142 | 136.4398316 | 194.5252797 |
| YER152C | YER152C | 0.16159959 | 0.99381655 | 0.1865419 | 144.9892853 | 208.1341822 |
| YOR024W | YOR024W | 0.39294138 | 0.99917121 | 0.18683242 | 160.5497385 | 232.8766609 |
| YDL076C | RXT3 | 0.22774513 | 0.99917121 | 0.18707128 | 174.94601 | 255.7716373 |
| YMR052W | FAR3 | 0.10525202 | 0.94753174 | 0.18724918 | 142.3257137 | 203.8065826 |
| YNL277W | MET2 | 0.31789773 | 0.99917121 | 0.18791508 | 145.848 | 209.3341618 |
| YEL060C | PRB1 | 0.14789281 | 0.98328895 | 0.18805463 | 137.8356784 | 196.5586222 |
| YOL063C | CRT10 | 0.23435219 | 0.99917121 | 0.18818746 | 138.103237 | 196.9684796 |
| YBR041W | FAT1 | 0.35045635 | 0.99917121 | 0.18853735 | 127.0767912 | 179.3677144 |
| YPL158C | YPL158C | 0.54669374 | 0.99917121 | 0.18854028 | 127.7128766 | 180.3802364 |
| YPR062W | FCY1 | 0.44375521 | 0.99917121 | 0.18860834 | 135.5130021 | 192.7925736 |
| YPR046W | MCM16 | 0.38367198 | 0.99917121 | 0.18874378 | 149.1256973 | 214.4524203 |
| YHR185C | PFS1 | 0.28775717 | 0.99917121 | 0.18892355 | 152.1778739 | 219.2906792 |
| YER051W | JHD1 | 0.30469249 | 0.99917121 | 0.1890805 | 154.7470701 | 223.3626392 |
| YDL181W | INH1 | 0.64049734 | 0.99917121 | 0.18908812 | 130.6580048 | 185.0031635 |
| YBR189W | RPS9B | 0.72638783 | 0.99917121 | 0.18912246 | 38.75385152 | 38.65416575 |
| YPL060W | LPE10 | 0.11712164 | 0.9579757 | 0.18917542 | 129.9002219 | 183.7858552 |
| YMR116C | ASC1 | 0.04557321 | 0.94753174 | 0.18933477 | 93.84591037 | 126.3548617 |
| YMR202W | ERG2 | 0.6217705 | 0.99917121 | 0.18960528 | 33.15593562 | 29.68138782 |
| YDL079C | MRK1 | 0.25765084 | 0.99917121 | 0.18971791 | 146.9268834 | 210.8323523 |
| YMR206W | YMR206W | 0.00638126 | 0.84351098 | 0.18988132 | 139.3910345 | 198.8126211 |
| YLR307W | CDA1 | 0.38957444 | 0.99917121 | 0.19025816 | 152.8916378 | 220.2645468 |
| YPL096W | PNG1 | 0.0007168 | 0.33960437 | 0.19055428 | 142.4966591 | 203.675863 |
| YIL094C | LYS12 | 0.50542548 | 0.99917121 | 0.19056234 | 127.3967825 | 179.6303897 |
| YER089C | PTC2 | 0.29171631 | 0.99917121 | 0.19058129 | 140.103555 | 199.8618787 |
| YGR206W | MVB12 | 0.06453965 | 0.94753174 | 0.19110317 | 138.2357436 | 196.8240218 |
| YPR128C | ANT1 | 0.38878825 | 0.99917121 | 0.19150805 | 135.8985879 | 193.0530607 |
| YPR126C | YPR126C | 0.36009211 | 0.99917121 | 0.19171685 | 160.5408849 | 232.2670981 |
| YGR032W | GSC2 | 0.18244014 | 0.99917121 | 0.19177009 | 145.1663396 | 207.7787441 |
| YMR120C | ADE17 | 0.08455712 | 0.94753174 | 0.19180547 | 142.4784077 | 203.4942662 |
| YCL030C | HIS4 | 0.12570301 | 0.97616733 | 0.19180863 | 151.9844322 | 218.6309272 |
| YPL066W | YPL066W | 0.45014211 | 0.99917121 | 0.19219412 | 143.2892649 | 204.738065 |
| YHR035W | YHR035W | 0.22931322 | 0.99917121 | 0.1923945 | 144.2100604 | 206.1798784 |
| YMR276W | DSK2 | 0.23329493 | 0.99917121 | 0.19254139 | 137.689994 | 195.7796552 |
| YCR090C | YCR090C | 0.51949583 | 0.99917121 | 0.19254374 | 172.3101798 | 250.9072868 |
| YDR123C | INO2 | 0.15068322 | 0.98328895 | 0.19268561 | 124.1372525 | 174.1811834 |
| YML059C | NTE1 | 0.17403652 | 0.99917121 | 0.1927335 | 142.4387874 | 203.3180394 |
| YLR364W | YLR364W | 0.4782366 | 0.99917121 | 0.19292851 | 122.3543431 | 171.3125321 |
| YDR505C | PSP1 | 0.24576415 | 0.99917121 | 0.1930187 | 143.6873723 | 205.2714723 |
| YER117W | RPL23B | 0.37483651 | 0.99917121 | 0.19318145 | 151.4354325 | 217.5893586 |
| YGL152C | YGL152C | 0.16643159 | 0.99381655 | 0.19345395 | 151.5340141 | 217.7131153 |
| YOL132W | GAS4 | 0.26000211 | 0.99917121 | 0.19372237 | 142.1914426 | 202.8036228 |
| YPR157W | YPR157W | 0.36015026 | 0.99917121 | 0.19378239 | 139.3627087 | 198.2919344 |
| YER039C | HVG1 | 0.07209268 | 0.94753174 | 0.19381256 | 140.7016622 | 200.4203562 |
| YHR207C | SET5 | 0.11546557 | 0.95531631 | 0.19406146 | 170.2810376 | 247.4911281 |
| YLR390W | ECM19 | 0.20204548 | 0.99917121 | 0.19446932 | 135.5635423 | 192.1585366 |
| YPR075C | OPY2 | 0.41232459 | 0.99917121 | 0.19454648 | 134.0346478 | 189.7145737 |
| YDR540C | IRC4 | 0.12409317 | 0.9759269 | 0.19499111 | 133.6001886 | 188.9685512 |
| YLR114C | AVL9 | 0.11034829 | 0.95531631 | 0.19507718 | 126.7383948 | 178.0315899 |
| YIR025W | MND2 | 0.11903884 | 0.96075443 | 0.19541084 | 144.0139534 | 205.4998798 |
| YDR513W | GRX2 | 0.20182525 | 0.99917121 | 0.19544028 | 142.8198749 | 203.5948847 |
| YLR413W | YLR413W | 0.1015529 | 0.94753174 | 0.19545402 | 151.7332763 | 217.7865839 |
| YNL090W | RHO2 | 0.108337 | 0.95523424 | 0.19551209 | 131.8514922 | 186.1204782 |
| YKR106W | YKR106W | 0.0940435 | 0.94753174 | 0.19558947 | 146.8303373 | 209.9628099 |
| YGL026C | TRP5 | 0.23316452 | 0.99917121 | 0.19568807 | 129.4091466 | 182.2099226 |
| YLR209C | PNP1 | 0.42395686 | 0.99917121 | 0.19583201 | 137.3322945 | 194.8089066 |
| YDR424C | DYN2 | 0.14071981 | 0.97853666 | 0.19589806 | 143.8353204 | 205.1560344 |

| | | | | | | |
|---|---|---|---|---|---|---|
| YBR271W | YBR271W | 0.17867699 | 0.99917121 | 0.19622651 | 151.1328686 | 216.7363415 |
| YDL159W | STE7 | 0.25996935 | 0.99917121 | 0.1964274 | 168.1694723 | 243.8403148 |
| YDR360W | YDR360W | 0.40338534 | 0.99917121 | 0.19653205 | 145.908452 | 208.3799232 |
| YPR091C | YPR091C | 0.38388544 | 0.99917121 | 0.19670488 | 144.9114382 | 206.7712453 |
| YBL057C | PTH2 | 0.25622167 | 0.99917121 | 0.19678744 | 152.3090994 | 218.5409452 |
| YER048C | CAJ1 | 0.28807912 | 0.99917121 | 0.19684785 | 154.885972 | 222.6368987 |
| YEL012W | UBC8 | 0.21984128 | 0.99917121 | 0.19686931 | 139.3223348 | 197.851315 |
| YGR182C | YGR182C | 0.20677709 | 0.99917121 | 0.19703392 | 147.8468505 | 211.4053747 |
| YJL103C | GSM1 | 0.385354 | 0.99917121 | 0.19725767 | 142.3639252 | 202.6472871 |
| YNL217W | YNL217W | 0.22785662 | 0.99917121 | 0.19736781 | 142.9053339 | 203.4959798 |
| YDR026C | YDR026C | 0.26791881 | 0.99917121 | 0.19744753 | 138.2993143 | 196.1518039 |
| YKR088C | TVP38 | 0.16341145 | 0.99381655 | 0.19754592 | 143.263307 | 204.0442897 |
| YKR060W | UTP30 | 0.16972136 | 0.99636201 | 0.19830913 | 140.4661543 | 199.497162 |
| YKL090W | CUE2 | 0.1932566 | 0.99917121 | 0.19831948 | 145.6750152 | 207.7903005 |
| YGL125W | MET13 | 0.11617019 | 0.95745643 | 0.19834465 | 136.8601179 | 193.7507118 |
| YBR034C | HMT1 | 0.16278406 | 0.99381655 | 0.19847486 | 135.5084112 | 191.5824289 |
| YBR045C | GIP1 | 0.20097695 | 0.99917121 | 0.19882543 | 150.1669268 | 214.8813749 |
| YBR165W | UBS1 | 0.07261921 | 0.94753174 | 0.19896049 | 140.8726388 | 200.0650256 |
| YBL039C | URA7 | 0.20395821 | 0.99917121 | 0.19917179 | 135.0073131 | 190.6995354 |
| YIL117C | PRM5 | 0.14197213 | 0.97853666 | 0.19918838 | 152.3599587 | 218.3292315 |
| YMR254C | YMR254C | 0.26510859 | 0.99917121 | 0.19919293 | 139.868577 | 198.4378571 |
| YLR416C | YLR416C | 0.16409321 | 0.99381655 | 0.19925897 | 143.0226926 | 203.452305 |
| YPR008W | HAA1 | 0.37437987 | 0.99917121 | 0.19931859 | 188.7651036 | 276.2835781 |
| YBR290W | BSD2 | 0.18948564 | 0.99917121 | 0.19943271 | 146.867126 | 209.5528589 |
| YJR082C | EAF6 | 0.15015757 | 0.98328895 | 0.19943413 | 134.9498421 | 190.5760388 |
| YML067C | ERV41 | 0.2144723 | 0.99917121 | 0.19971304 | 139.4929882 | 197.7763768 |
| YDR469W | SDC1 | 0.08252872 | 0.94753174 | 0.19993366 | 116.7386318 | 161.5162757 |
| YDR503C | LPP1 | 0.17824318 | 0.99917121 | 0.19995942 | 141.4096679 | 200.7983905 |
| YLR422W | YLR422W | 0.17585021 | 0.99917121 | 0.20009963 | 141.060761 | 200.225711 |
| YGL199C | YGL199C | 0.08353092 | 0.94753174 | 0.20024193 | 142.2601937 | 202.1182959 |
| YNL045W | YNL045W | 0.09518863 | 0.94753174 | 0.20033441 | 118.866224 | 164.8553204 |
| YCR107W | AAD3 | 0.31300435 | 0.99917121 | 0.20062669 | 141.7505603 | 201.2598683 |
| YPR012W | YPR012W | 0.42452531 | 0.99917121 | 0.20083889 | 144.2901328 | 205.2779207 |
| YOR012W | YOR012W | 0.52983876 | 0.99917121 | 0.20089463 | 158.0147069 | 227.1256359 |
| YHR079C-B | YHR079C-B | 0.08751241 | 0.94753174 | 0.20100667 | 151.9406821 | 217.4399219 |
| YGR015C | YGR015C | 0.16420823 | 0.99381655 | 0.20109862 | 148.2891702 | 211.6141784 |
| YGR129W | SYF2 | 0.23843698 | 0.99917121 | 0.20120258 | 152.2180219 | 217.8576634 |
| YMR187C | YMR187C | 0.07873063 | 0.94753174 | 0.20121108 | 129.7321429 | 182.0509364 |
| YBR031W | RPL4A | 0.2486449 | 0.99917121 | 0.20166528 | 129.4535783 | 181.5519884 |
| YLR018C | POM34 | 0.40508253 | 0.99917121 | 0.20168164 | 140.6412952 | 199.364905 |
| YPR199C | ARR1 | 0.31991735 | 0.99917121 | 0.20202181 | 142.5823757 | 202.414341 |
| YBR082C | UBC4 | 0.08278872 | 0.94753174 | 0.20203467 | 127.0920962 | 177.7466182 |
| YHR048W | YHR048W | 0.18027894 | 0.99917121 | 0.20211152 | 149.8477225 | 213.9724767 |
| YLR104W | YLR104W | 0.16192094 | 0.99381655 | 0.20212669 | 131.8913119 | 185.3774951 |
| YNL200C | YNL200C | 0.1726977 | 0.99917121 | 0.2021473 | 146.6484471 | 208.8737048 |
| YBR205W | KTR3 | 0.26392466 | 0.99917121 | 0.20237009 | 147.536881 | 210.2612547 |
| YGL211W | NCS6 | 0.41805861 | 0.99917121 | 0.20239996 | 120.4776329 | 167.1694566 |
| YPR022C | YPR022C | 0.30000894 | 0.99917121 | 0.20275455 | 138.4873102 | 195.8041787 |
| YEL030W | ECM10 | 0.20155079 | 0.99917121 | 0.2027982 | 144.4252269 | 205.2541792 |
| YPR006C | ICL2 | 0.22185228 | 0.99917121 | 0.20293778 | 143.9109154 | 204.418192 |
| YGR042W | YGR042W | 0.06305144 | 0.94753174 | 0.2029512 | 150.3979557 | 214.7462821 |
| YNL226W | YNL226W | 0.53908335 | 0.99917121 | 0.20305788 | 104.2661851 | 141.2747323 |
| YFL042C | YFL042C | 0.46147442 | 0.99917121 | 0.20319107 | 129.7595887 | 181.8532586 |
| YKL105C | YKL105C | 0.02660741 | 0.92955221 | 0.20355921 | 135.4740267 | 190.9078396 |
| YDR042C | YDR042C | 0.14985768 | 0.98328895 | 0.20358336 | 133.5053876 | 187.7701074 |
| YIR017C | MET28 | 0.28035621 | 0.99917121 | 0.20375882 | 166.2576135 | 239.9021632 |
| YKR074W | YKR074W | 0.01617885 | 0.84721907 | 0.20378979 | 112.7975648 | 154.7705617 |
| YLR437C | YLR437C | 0.17715822 | 0.99917121 | 0.20386458 | 152.1098377 | 217.3608685 |
| YER182W | FMP10 | 0.1590315 | 0.99381655 | 0.20403985 | 142.4673745 | 201.9851967 |
| YAL059W | ECM1 | 0.17417505 | 0.99917121 | 0.20408941 | 138.9345056 | 196.353543 |
| YLR232W | YLR232W | 0.17601573 | 0.99917121 | 0.20438389 | 137.9607103 | 194.767007 |
| YBR030W | YBR030W | 0.60124905 | 0.99917121 | 0.20444273 | 162.2902229 | 233.501259 |
| YER091C-A | YER091C-A | 0.23118756 | 0.99917121 | 0.2044741 | 147.4389239 | 209.8487702 |
| YMR198W | CIK1 | 0.45337527 | 0.99917121 | 0.20450853 | 115.8615473 | 159.5619133 |

| | | | | | | |
|---|---|---|---|---|---|---|
| YDR482C | CWC21 | 0.07851597 | 0.94753174 | 0.20459997 | 140.0513517 | 198.0697252 |
| YNR050C | LYS9 | 0.13370896 | 0.97853666 | 0.20469361 | 124.5149132 | 173.3186534 |
| YLR172C | DPH5 | 0.09495883 | 0.94753174 | 0.20471987 | 137.7062755 | 194.3208945 |
| YNL014W | HEF3 | 0.32740138 | 0.99917121 | 0.20474613 | 139.6502326 | 197.413179 |
| YDL035C | GPR1 | 0.29021757 | 0.99917121 | 0.20480611 | 155.7696688 | 223.0738672 |
| YOR358W | HAP5 | 0.08242311 | 0.94753174 | 0.20493295 | 113.0889295 | 155.0951568 |
| YGR272C | YGR272C | 0.21644324 | 0.99917121 | 0.20510358 | 141.2221432 | 199.8726551 |
| YJL176C | SWI3 | 0.06265662 | 0.94753174 | 0.20513974 | 144.4758718 | 205.0493657 |
| YPR119W | CLB2 | 0.00232427 | 0.52528514 | 0.2052803 | 144.9116283 | 205.7261129 |
| YML041C | VPS71 | 0.12900446 | 0.97616733 | 0.20534024 | 124.0757461 | 172.5405083 |
| YGR123C | PPT1 | 0.26604531 | 0.99917121 | 0.20567434 | 163.3510152 | 235.04028 |
| YIL148W | RPL40A | 0.09210481 | 0.94753174 | 0.20580089 | 141.9976294 | 201.0225017 |
| YDR466W | PKH3 | 0.23659022 | 0.99917121 | 0.20592982 | 144.6593953 | 205.2452822 |
| YDR524C | AGE1 | 0.13015524 | 0.97616733 | 0.2059878 | 131.4764493 | 184.2461732 |
| YLL017W | YLL017W | 0.51881662 | 0.99917121 | 0.20622739 | 157.518243 | 225.6849644 |
| YJL022W | YJL022W | 0.21817103 | 0.99917121 | 0.20639743 | 154.9035938 | 221.5007627 |
| YEL041W | YEF1 | 0.17764188 | 0.99917121 | 0.20663393 | 145.9698905 | 207.246228 |
| YAR015W | ADE1 | 0.13408584 | 0.97853666 | 0.20666107 | 149.068013 | 212.1762561 |
| YLR258W | GSY2 | 0.2527419 | 0.99917121 | 0.2067276 | 138.3155091 | 195.0462524 |
| YER132C | PMD1 | 0.27116931 | 0.99917121 | 0.20715773 | 149.4643158 | 212.7467669 |
| YKL094W | YJU3 | 0.12086359 | 0.96390893 | 0.20716432 | 144.1699278 | 204.3153736 |
| YEL062W | NPR2 | 0.05853287 | 0.94753174 | 0.20729995 | 129.6012415 | 181.1001955 |
| YFL047W | RGD2 | 0.29429065 | 0.99917121 | 0.20733477 | 150.9769108 | 215.133785 |
| YIL156W | UBP7 | 0.11533293 | 0.95531631 | 0.20739538 | 154.3625033 | 220.5174892 |
| YPR021C | AGC1 | 0.34220508 | 0.99917121 | 0.20750095 | 138.4840939 | 195.2204213 |
| YFR026C | YFR026C | 0.20636256 | 0.99917121 | 0.20756275 | 153.2804294 | 218.7740294 |
| YBR273C | UBX7 | 0.15903947 | 0.99381655 | 0.20791455 | 160.6402383 | 230.450632 |
| YLR395C | COX8 | 0.25463208 | 0.99917121 | 0.20806358 | 138.4665611 | 195.1239114 |
| YEL066W | HPA3 | 0.17986349 | 0.99917121 | 0.20819428 | 144.4904527 | 204.7002018 |
| YNL104C | LEU4 | 0.1777832 | 0.99917121 | 0.20839461 | 134.8524905 | 189.3286422 |
| YLL043W | FPS1 | 0.1075871 | 0.95057412 | 0.20863616 | 131.3730902 | 183.7587239 |
| YGL050W | TYW3 | 0.24145803 | 0.99917121 | 0.20891224 | 155.1282697 | 221.5519462 |
| YPR153W | YPR153W | 0.29825432 | 0.99917121 | 0.20907375 | 137.6670135 | 193.7275908 |
| YJR139C | HOM6 | 0.2999857 | 0.99917121 | 0.20921447 | 111.8656823 | 152.6253394 |
| YER128W | YER128W | 0.07077415 | 0.94753174 | 0.20934922 | 139.4521555 | 196.5366025 |
| YHR140W | YHR140W | 0.0980882 | 0.94753174 | 0.20973747 | 157.9674387 | 225.9723299 |
| YOL150C | YOL150C | 0.39513625 | 0.99917121 | 0.21001033 | 158.487946 | 226.7679024 |
| YHR136C | SPL2 | 0.4058038 | 0.99917121 | 0.21011118 | 179.3174933 | 259.9238174 |
| YLR253W | YLR253W | 0.32267447 | 0.99917121 | 0.21011144 | 156.1957263 | 223.1055299 |
| YPR078C | YPR078C | 0.1664541 | 0.99381655 | 0.21012051 | 130.795668 | 182.6583002 |
| YGL124C | MON1 | 0.21404508 | 0.99917121 | 0.21087653 | 103.5512383 | 139.1831003 |
| YER067C-A | YER067C-A | 0.12280547 | 0.97113569 | 0.21093114 | 139.4381994 | 196.3215267 |
| YKL185W | ASH1 | 0.47005554 | 0.99917121 | 0.21111833 | 150.4772086 | 213.8768199 |
| YIR020C | YIR020C | 0.09863812 | 0.94753174 | 0.21115645 | 151.4264023 | 215.3836341 |
| YMR009W | ADI1 | 0.251799 | 0.99917121 | 0.21199673 | 128.4414351 | 178.6807748 |
| YEL033W | MTC7 | 0.15715351 | 0.98919321 | 0.21200922 | 118.9855695 | 163.6220779 |
| YLR282C | YLR282C | 0.28462691 | 0.99917121 | 0.21213377 | 136.7353312 | 191.8709648 |
| YGR144W | THI4 | 0.19121467 | 0.99917121 | 0.21218236 | 157.706551 | 225.2588438 |
| YML026C | RPS18B | 0.46096144 | 0.99917121 | 0.21226344 | 113.8677907 | 155.4417215 |
| YMR177W | MMT1 | 0.18733312 | 0.99917121 | 0.21230245 | 140.5918532 | 197.9913849 |
| YMR275C | BUL1 | 0.14861822 | 0.98328895 | 0.21234011 | 141.849159 | 199.988882 |
| YHR203C | RPS4B | 0.45586642 | 0.99917121 | 0.21236173 | 120.7540037 | 166.3950922 |
| YFL013W-A | YFL013W-A | 0.45474257 | 0.99917121 | 0.21277237 | 159.1937301 | 227.5550451 |
| YBR028C | YBR028C | 0.00155594 | 0.43627345 | 0.21288684 | 144.4320238 | 204.0350894 |
| YJL199C | MBB1 | 0.25531014 | 0.99917121 | 0.21291962 | 156.5725569 | 223.3632338 |
| YLR039C | RIC1 | 0.36110797 | 0.99917121 | 0.21297632 | 130.0513313 | 181.1248919 |
| YER057C | HMF1 | 0.1663652 | 0.99381655 | 0.21407625 | 120.5953007 | 165.9333609 |
| YLR435W | TSR2 | 0.11786166 | 0.95851884 | 0.21416533 | 112.6901295 | 153.3345957 |
| YPR061C | JID1 | 0.23532925 | 0.99917121 | 0.21443605 | 137.0525071 | 192.0953508 |
| YBL025W | RRN10 | 0.35138494 | 0.99917121 | 0.21510567 | 160.0870861 | 228.6931379 |
| YDR458C | HEH2 | 0.18517198 | 0.99917121 | 0.21518983 | 146.1334498 | 206.4636184 |
| YBR169C | SSE2 | 0.12619973 | 0.97616733 | 0.21546232 | 155.1074016 | 220.720192 |
| YLR019W | PSR2 | 0.21549359 | 0.99917121 | 0.21561282 | 140.6112691 | 197.6187327 |
| YGL181W | GTS1 | 0.0960529 | 0.94753174 | 0.21612016 | 155.7188937 | 221.6137115 |

| | | | | | | |
|---|---|---|---|---|---|---|
| YEL067C | YEL067C | 0.16280202 | 0.99381655 | 0.21635473 | 142.3190787 | 200.2477395 |
| YHR152W | SPO12 | 0.0835372 | 0.94753174 | 0.21635696 | 152.142909 | 215.8905756 |
| YMR022W | QRI8 | 0.20486877 | 0.99917121 | 0.21635897 | 139.9148909 | 196.4188818 |
| YNL164C | IBD2 | 0.22266318 | 0.99917121 | 0.21652817 | 149.3219642 | 211.3777338 |
| YER108C | YER108C | 0.17680287 | 0.99917121 | 0.21697073 | 146.7222486 | 207.1840894 |
| YML034W | SRC1 | 0.11319577 | 0.95531631 | 0.2169775 | 132.3079944 | 184.2305334 |
| YLR178C | TFS1 | 0.1597622 | 0.99381655 | 0.21738841 | 144.3310057 | 203.3254415 |
| YPL239W | YAR1 | 0.50439313 | 0.99917121 | 0.21753752 | 95.5450436 | 125.6222831 |
| YKL175W | ZRT3 | 0.00053287 | 0.33960437 | 0.21808291 | 149.1176428 | 210.8628407 |
| YDR511W | ACN9 | 0.11925527 | 0.96075443 | 0.21838404 | 130.6780797 | 181.4636444 |
| YER047C | SAP1 | 0.23601581 | 0.99917121 | 0.21845369 | 153.594203 | 217.9459497 |
| YDR485C | VPS72 | 0.06053123 | 0.94753174 | 0.21902558 | 127.31725 | 176.0337719 |
| YMR118C | YMR118C | 0.20354888 | 0.99917121 | 0.21911358 | 145.962472 | 205.7130123 |
| YIL055C | YIL055C | 0.29219503 | 0.99917121 | 0.21917395 | 157.4185462 | 223.9478863 |
| YLR400W | YLR400W | 0.08342499 | 0.94753174 | 0.21942654 | 136.5549099 | 190.6946013 |
| YNR007C | ATG3 | 0.09231168 | 0.94753174 | 0.21952764 | 131.3460571 | 182.3878896 |
| YAL062W | GDH3 | 0.19320907 | 0.99917121 | 0.22007862 | 150.9261301 | 213.4993116 |
| YOR190W | SPR1 | 0.29814762 | 0.99917121 | 0.22045375 | 153.9864833 | 218.326774 |
| YNL296W | YNL296W | 0.37588795 | 0.99917121 | 0.22079234 | 132.9573617 | 184.7994918 |
| YLR191W | PEX13 | 0.27212474 | 0.99917121 | 0.22082378 | 144.0677872 | 202.487494 |
| YDR536W | STL1 | 0.06402857 | 0.94753174 | 0.22090448 | 145.9701727 | 205.5069445 |
| YNL097C | PHO23 | 0.42406165 | 0.99917121 | 0.22108529 | 140.1921326 | 196.2841631 |
| YFL063W | YFL063W | 0.21006629 | 0.99917121 | 0.22194325 | 145.2235378 | 204.1913942 |
| YLL047W | YLL047W | 0.08765175 | 0.94753174 | 0.22232603 | 140.6694738 | 196.8930045 |
| YPR045C | MNI2 | 0.35676643 | 0.99917121 | 0.22317832 | 112.0595617 | 151.2317237 |
| YLL057C | JLP1 | 0.19324715 | 0.99917121 | 0.22320991 | 137.5453432 | 191.8104986 |
| YJL038C | YJL038C | 0.11210212 | 0.95531631 | 0.22354545 | 152.3232777 | 215.3014349 |
| YKL070W | YKL070W | 0.1529964 | 0.98492771 | 0.2237332 | 144.8534137 | 203.3838078 |
| YEL040W | UTR2 | 0.19257356 | 0.99917121 | 0.22406895 | 149.9653516 | 211.4829389 |
| YMR052C-A | YMR052C-A | 0.07455027 | 0.94753174 | 0.22435354 | 145.2441382 | 203.9303565 |
| YLR119W | SRN2 | 0.22668822 | 0.99917121 | 0.22457069 | 123.1264487 | 168.684484 |
| YPR106W | ISR1 | 0.40189731 | 0.99917121 | 0.22472352 | 136.275368 | 189.6037108 |
| YNL076W | MKS1 | 0.07646864 | 0.94753174 | 0.22475119 | 121.673411 | 166.3487157 |
| YOR123C | LEO1 | 0.37943659 | 0.99917121 | 0.22504983 | 152.6803068 | 215.6865543 |
| YNR009W | NRM1 | 0.14338906 | 0.98199783 | 0.225054 | 139.6839982 | 194.9912 |
| YCL069W | VBA3 | 0.23225165 | 0.99917121 | 0.22510755 | 156.8777457 | 222.3633662 |
| YPL035C | YPL035C | 0.22127144 | 0.99917121 | 0.22524296 | 135.7735678 | 188.7413375 |
| YNL197C | WHI3 | 0.06464653 | 0.94753174 | 0.22537453 | 118.4743833 | 161.1787091 |
| YLR344W | RPL26A | 0.2714942 | 0.99917121 | 0.22552883 | 129.8119809 | 179.2134734 |
| YNL153C | GIM3 | 0.08874282 | 0.94753174 | 0.22589429 | 122.0791162 | 166.8553892 |
| YPL265W | DIP5 | 0.138479 | 0.97853666 | 0.22621167 | 146.9018458 | 206.3435046 |
| YGR178C | PBP1 | 0.05937302 | 0.94753174 | 0.22626126 | 145.6665514 | 204.3704213 |
| YGL226C-A | OST5 | 0.18186602 | 0.99917121 | 0.22638761 | 157.0085748 | 222.4156409 |
| YML030W | YML030W | 0.00010368 | 0.22259691 | 0.22663449 | 127.0131556 | 174.6219349 |
| YFL015C | YFL015C | 0.12608388 | 0.97616733 | 0.22676537 | 144.4016973 | 202.2948568 |
| YER030W | CHZ1 | 0.06085258 | 0.94753174 | 0.22686642 | 153.1437702 | 216.2030954 |
| YEL010W | YEL010W | 0.23533136 | 0.99917121 | 0.2269883 | 152.0495789 | 214.4458862 |
| YLR234W | TOP3 | 0.11294573 | 0.95531631 | 0.2272642 | 119.2341442 | 162.1581531 |
| YGL084C | GUP1 | 0.22055135 | 0.99917121 | 0.22730632 | 134.4040653 | 186.309046 |
| YFL056C | AAD6 | 0.14102929 | 0.97853666 | 0.22733062 | 148.4110865 | 208.6103514 |
| YOR243C | PUS7 | 0.23850845 | 0.99917121 | 0.22737676 | 144.4838097 | 202.351075 |
| YGL215W | CLG1 | 0.1105264 | 0.95531631 | 0.22743272 | 158.1302596 | 224.0743608 |
| YOR240W | YOR240W | 0.22019222 | 0.99917121 | 0.22751011 | 142.9487518 | 199.8904485 |
| YLR285W | NNT1 | 0.04905774 | 0.94753174 | 0.22831495 | 145.6131226 | 204.0349759 |
| YML063W | RPS1B | 0.57394511 | 0.99917121 | 0.22834535 | 103.840544 | 137.5141437 |
| YOR179C | SYC1 | 0.11200099 | 0.95531631 | 0.22835522 | 133.9097789 | 185.3940899 |
| YGL253W | HXK2 | 0.21378214 | 0.99917121 | 0.22860496 | 134.6657617 | 186.5674437 |
| YJL206C-A | YJL206C-A | 0.08235533 | 0.94753174 | 0.22872444 | 161.7825914 | 229.7327253 |
| YBR095C | RXT2 | 0.11443385 | 0.95531631 | 0.22897548 | 145.4498661 | 203.694487 |
| YMR080C | NAM7 | 0.11684456 | 0.9579757 | 0.22903651 | 138.9518476 | 193.3398397 |
| YPR001W | CIT3 | 0.19259959 | 0.99917121 | 0.22930218 | 152.5706175 | 214.9934826 |
| YDR446W | ECM11 | 0.12507651 | 0.97616733 | 0.22932248 | 151.8368678 | 213.8226117 |
| YDR501W | PLM2 | 0.23330468 | 0.99917121 | 0.22938007 | 152.7008117 | 215.1913033 |
| YGL126W | SCS3 | 0.07874568 | 0.94753174 | 0.22981558 | 154.7530617 | 218.406138 |

| | | | | | | |
|---|---|---|---|---|---|---|
| YGL222C | EDC1 | 0.23306565 | 0.99917121 | 0.23016589 | 152.8533855 | 215.3384562 |
| YER032W | FIR1 | 0.21258267 | 0.99917121 | 0.23049614 | 147.4143599 | 206.6372898 |
| YLR061W | RPL22A | 0.02028191 | 0.87709187 | 0.2305617 | 25.30098021 | 12.1804188 |
| YFL041W | FET5 | 0.29007604 | 0.99917121 | 0.23092342 | 154.2375742 | 217.4502366 |
| YHR116W | COX23 | 0.27355903 | 0.99917121 | 0.23122391 | 119.0674069 | 161.4099162 |
| YLR171W | YLR171W | 0.06279581 | 0.94753174 | 0.23139252 | 154.0290581 | 217.0610156 |
| YMR133W | REC114 | 0.10293229 | 0.94753174 | 0.23149977 | 137.6277081 | 190.9310309 |
| YLR393W | ATP10 | 0.05998898 | 0.94753174 | 0.23175325 | 103.1944839 | 136.0699218 |
| YEL065W | SIT1 | 0.06220924 | 0.94753174 | 0.23181705 | 153.786655 | 216.6232664 |
| YML106W | URA5 | 0.14104698 | 0.97853666 | 0.23189462 | 141.1925179 | 196.5593669 |
| YMR292W | GOT1 | 0.20494943 | 0.99917121 | 0.23206887 | 131.1335794 | 180.5206386 |
| YGR289C | MAL11 | 0.50816507 | 0.99917121 | 0.23218548 | 145.6812181 | 203.6715503 |
| YDR116C | MRPL1 | 0.37657441 | 0.99917121 | 0.23227881 | 150.7563828 | 211.7416781 |
| YPR028W | YOP1 | 0.16046975 | 0.99381655 | 0.23229048 | 141.2419086 | 196.5897554 |
| YOR114W | YOR114W | 0.22957909 | 0.99917121 | 0.23249266 | 148.8154639 | 208.6249598 |
| YNL246W | VPS75 | 0.13191015 | 0.97853666 | 0.23299182 | 137.6216057 | 190.7394161 |
| YMR143W | RPS16A | 0.05175569 | 0.94753174 | 0.23303216 | 93.97595714 | 121.2347645 |
| YEL016C | NPP2 | 0.06993656 | 0.94753174 | 0.23308187 | 138.1752657 | 191.6100661 |
| YER038W-A | FMP49 | 0.20554852 | 0.99917121 | 0.23313893 | 146.1580401 | 204.3145885 |
| YMR226C | TMA29 | 0.02278558 | 0.88145277 | 0.23316334 | 130.1936243 | 178.8904601 |
| YPR040W | TIP41 | 0.22427605 | 0.99917121 | 0.2331741 | 147.2476169 | 206.0453022 |
| YHR153C | SPO16 | 0.10569256 | 0.94753174 | 0.23332562 | 149.1759402 | 209.0974221 |
| YGR001C | YGR001C | 0.12146136 | 0.96405745 | 0.23346421 | 160.4791792 | 227.0793908 |
| YMR245W | YMR245W | 0.01484944 | 0.84351098 | 0.23361943 | 119.2303622 | 161.3773608 |
| YDR158W | HOM2 | 0.01079062 | 0.84351098 | 0.23404286 | 124.0889182 | 169.0623272 |
| YJR111C | YJR111C | 0.04091546 | 0.94753174 | 0.23439324 | 142.4512572 | 198.2591286 |
| YLR235C | YLR235C | 0.16293003 | 0.99381655 | 0.23449732 | 111.2941804 | 148.6330504 |
| YPL264C | YPL264C | 0.31002969 | 0.99917121 | 0.23456308 | 146.3800947 | 204.4945604 |
| YLR177W | YLR177W | 0.11657704 | 0.9579757 | 0.23477277 | 107.3182639 | 142.2683669 |
| YGR121C | MEP1 | 0.11506902 | 0.95531631 | 0.23528922 | 147.9088572 | 206.8403821 |
| YPL069C | BTS1 | 0.66454473 | 0.99917121 | 0.23558114 | 54.67499873 | 58.3426077 |
| YNR005C | YNR005C | 0.16623718 | 0.99381655 | 0.23566594 | 130.208008 | 178.6082708 |
| YMR310C | YMR310C | 0.13776507 | 0.97853666 | 0.23570925 | 136.2610196 | 188.2415846 |
| YGR043C | YGR043C | 0.08008851 | 0.94753174 | 0.23590254 | 151.9242782 | 213.1596215 |
| YER093C-A | YER093C-A | 0.14770082 | 0.98328895 | 0.23611506 | 129.4443298 | 177.3374654 |
| YMR073C | IRC21 | 0.05211461 | 0.94753174 | 0.23636426 | 131.5081623 | 180.5934567 |
| YPR198W | SGE1 | 0.18840643 | 0.99917121 | 0.23638281 | 145.8950182 | 203.5002981 |
| YFR038W | IRC5 | 0.06750247 | 0.94753174 | 0.23639278 | 150.8195404 | 211.340711 |
| YLR434C | YLR434C | 0.09625171 | 0.94753174 | 0.23656094 | 126.8587193 | 173.1658759 |
| YDR083W | RRP8 | 0.06195116 | 0.94753174 | 0.23699411 | 113.4536611 | 151.7673429 |
| YJR003C | YJR003C | 0.13244455 | 0.97853666 | 0.23707047 | 154.9205305 | 217.7883602 |
| YKR042W | UTH1 | 0.07581509 | 0.94753174 | 0.23720628 | 149.1686157 | 208.6126651 |
| YML005W | TRM12 | 0.27472019 | 0.99917121 | 0.23722769 | 153.9429668 | 216.2125569 |
| YGL086W | MAD1 | 0.12995784 | 0.97616733 | 0.237774 | 155.5974752 | 218.7805349 |
| YDR425W | SNX41 | 0.06489026 | 0.94753174 | 0.23778258 | 141.4530727 | 196.2564593 |
| YLR040C | YLR040C | 0.09735495 | 0.94753174 | 0.23842371 | 139.1493927 | 192.5100034 |
| YPR194C | OPT2 | 0.16516382 | 0.99381655 | 0.23848761 | 112.1391753 | 149.4921305 |
| YMR158W-A | YMR158W-A | 0.02268599 | 0.88145277 | 0.23898865 | 124.4748585 | 169.0739394 |
| YFL007W | BLM10 | 0.32636619 | 0.99917121 | 0.2392667 | 155.9180266 | 219.1089932 |
| YML107C | PML39 | 0.02085744 | 0.87709187 | 0.23961122 | 129.3389148 | 176.7433866 |
| YAL048C | GEM1 | 0.69954734 | 0.99917121 | 0.23995224 | 46.114211 | 44.17783901 |
| YEL068C | YEL068C | 0.13986248 | 0.97853666 | 0.24081013 | 144.597787 | 200.8948972 |
| YGR224W | AZR1 | 0.28274836 | 0.99917121 | 0.24145209 | 161.4489536 | 227.6498164 |
| YER170W | ADK2 | 0.17560827 | 0.99917121 | 0.24149655 | 155.2986949 | 217.8509495 |
| YLR165C | PUS5 | 0.08827994 | 0.94753174 | 0.24169521 | 131.4347427 | 179.8266461 |
| YMR190C | SGS1 | 0.21845535 | 0.99917121 | 0.24211275 | 123.876493 | 167.7402627 |
| YIL012W | YIL012W | 0.17498979 | 0.99917121 | 0.24250222 | 152.1903008 | 212.778654 |
| YDL083C | RPS16B | 0.25637347 | 0.99917121 | 0.24250947 | 106.6125417 | 140.2014137 |
| YFL055W | AGP3 | 0.2158217 | 0.99917121 | 0.24259143 | 152.5185025 | 213.290395 |
| YBL107C | YBL107C | 0.31533154 | 0.99917121 | 0.24277084 | 151.0820222 | 210.9811245 |
| YGR134W | CAF130 | 0.20747291 | 0.99917121 | 0.24277735 | 157.527277 | 221.2435181 |
| YKL149C | DBR1 | 0.03591458 | 0.94753174 | 0.24283323 | 132.0905259 | 180.7321538 |
| YNL215W | IES2 | 0.0217174 | 0.87709187 | 0.24288892 | 117.3428801 | 157.2417535 |
| YGL219C | MDM34 | 0.13825146 | 0.97853666 | 0.24302275 | 125.4168697 | 170.0821633 |

| | | | | | | |
|---|---|---|---|---|---|---|
| YDL182W | LYS20 | 0.16860353 | 0.99636201 | 0.24351329 | 120.4211104 | 162.0672969 |
| YOL039W | RPP2A | 0.32955353 | 0.99917121 | 0.24366725 | 139.7549001 | 192.8349468 |
| YLR035C | MLH2 | 0.22422747 | 0.99917121 | 0.24420297 | 147.7968187 | 205.5752943 |
| YDR467C | YDR467C | 0.07229825 | 0.94753174 | 0.24423295 | 153.2582379 | 214.268203 |
| YFR022W | ROG3 | 0.06900528 | 0.94753174 | 0.24426285 | 151.8936157 | 212.0915842 |
| YOR184W | SER1 | 0.13370843 | 0.97853666 | 0.24469318 | 138.5846313 | 190.8463821 |
| YNR045W | PET494 | 0.24266489 | 0.99917121 | 0.24474907 | 146.1138325 | 202.8287927 |
| YGR012W | YGR012W | 0.09816534 | 0.94753174 | 0.24503595 | 147.7612158 | 205.4170527 |
| YML056C | IMD4 | 0.26540103 | 0.99917121 | 0.2452886 | 151.3138452 | 211.0433292 |
| YDR474C | YDR474C | 0.21009482 | 0.99917121 | 0.24612411 | 151.7398982 | 211.6199027 |
| YER020W | GPA2 | 0.04377966 | 0.94753174 | 0.24634622 | 135.6413152 | 185.9580296 |
| YDL012C | YDL012C | 0.00670466 | 0.84351098 | 0.2469218 | 169.0770622 | 239.1297208 |
| YGL077C | HNM1 | 0.14332451 | 0.98199783 | 0.24729756 | 152.8030746 | 213.1698098 |
| YGR025W | YGR025W | 0.09191158 | 0.94753174 | 0.24758411 | 150.0161696 | 208.6971107 |
| YOR347C | PYK2 | 0.23786583 | 0.99917121 | 0.24813593 | 163.0806239 | 229.433197 |
| YLR265C | NEJ1 | 0.08162674 | 0.94753174 | 0.2482937 | 145.1913716 | 200.9277715 |
| YOR180C | DCI1 | 0.15423402 | 0.98492771 | 0.24847609 | 139.3129127 | 191.5448934 |
| YER180C | ISC10 | 0.08296156 | 0.94753174 | 0.24853454 | 145.6543743 | 201.6356796 |
| YNL147W | LSM7 | 0.40098349 | 0.99917121 | 0.24907763 | 110.8002206 | 146.0689921 |
| YLR184W | YLR184W | 0.1293962 | 0.97616733 | 0.2493689 | 121.8877969 | 163.688934 |
| YGL010W | YGL010W | 0.06543908 | 0.94753174 | 0.24941701 | 153.1797378 | 213.5112104 |
| YJR008W | YJR008W | 0.12962736 | 0.97616733 | 0.24954578 | 150.6488329 | 209.4653912 |
| YPR192W | AQY1 | 0.26410229 | 0.99917121 | 0.24955384 | 149.1052385 | 207.006446 |
| YML048W | GSF2 | 0.08486483 | 0.94753174 | 0.24979488 | 132.1513198 | 179.9802607 |
| YCR077C | PAT1 | 0.30987482 | 0.99917121 | 0.2499564 | 117.134155 | 156.0477859 |
| YIL102C | YIL102C | 0.28350731 | 0.99917121 | 0.2504252 | 141.5139878 | 194.8121882 |
| YLR107W | REX3 | 0.20006975 | 0.99917121 | 0.25042532 | 139.3385544 | 191.3480933 |
| YNL199C | GCR2 | 0.34950009 | 0.99917121 | 0.25088183 | 119.5562858 | 159.7918783 |
| YGL039W | YGL039W | 0.12865696 | 0.97616733 | 0.25112895 | 167.6903342 | 236.4086492 |
| YHR132W-A | IGO2 | 0.15610482 | 0.98575602 | 0.25122247 | 153.1331635 | 213.216942 |
| YNL086W | YNL086W | 0.36361068 | 0.99917121 | 0.25168018 | 145.8148316 | 201.5076989 |
| YDL080C | THI3 | 0.10931816 | 0.95531631 | 0.25226934 | 137.5018141 | 188.1985282 |
| YLR174W | IDP2 | 0.02592479 | 0.92955221 | 0.25255436 | 152.2121205 | 211.5879357 |
| YLL045C | RPL8B | 0.00185716 | 0.46909773 | 0.25276397 | 125.0096367 | 168.2461416 |
| YDR173C | ARG82 | 0.48081681 | 0.99917121 | 0.25281951 | 104.8792851 | 136.1845355 |
| YOR158W | PET123 | 0.18675794 | 0.99917121 | 0.25289886 | 142.613186 | 196.2609448 |
| YMR102C | YMR102C | 0.07489036 | 0.94753174 | 0.25303929 | 143.6576118 | 197.9069312 |
| YLR213C | CRR1 | 0.10409155 | 0.94753174 | 0.25312092 | 149.0852012 | 206.5396742 |
| YKL123W | YKL123W | 0.06536858 | 0.94753174 | 0.25367321 | 147.9023709 | 204.5888482 |
| YDR530C | APA2 | 0.10229806 | 0.94753174 | 0.25424774 | 151.1404633 | 209.6750266 |
| YER028C | MIG3 | 0.14562247 | 0.98328895 | 0.25484548 | 153.4191835 | 213.2307059 |
| YDL135C | RDI1 | 0.31937712 | 0.99917121 | 0.25500577 | 166.3022765 | 233.7257319 |
| YEL031W | SPF1 | 0.1534986 | 0.98492771 | 0.25508111 | 122.2420861 | 163.5567118 |
| YPL183W-A | RTC6 | 0.01181882 | 0.84351098 | 0.25523839 | 113.9327314 | 150.3060252 |
| YGR106C | YGR106C | 0.09246788 | 0.94753174 | 0.25527183 | 152.7978903 | 212.1894057 |
| YOR333C | YOR333C | 0.3462948 | 0.99917121 | 0.25594087 | 137.9150685 | 188.4089812 |
| YPR014C | YPR014C | 0.13874003 | 0.97853666 | 0.25596834 | 152.5913455 | 211.7755991 |
| YER119C-A | YER119C-A | 0.09796075 | 0.94753174 | 0.25598435 | 125.7642852 | 169.0552176 |
| YLR219W | MSC3 | 0.12262602 | 0.97113569 | 0.25647263 | 140.5205761 | 192.4930693 |
| YDR514C | YDR514C | 0.03283645 | 0.94753174 | 0.2564969 | 145.414785 | 200.2834699 |
| YHR189W | PTH1 | 0.05313529 | 0.94753174 | 0.25664699 | 129.2140861 | 174.4677721 |
| YGR008C | STF2 | 0.16261098 | 0.99381655 | 0.25676339 | 154.1262986 | 214.1228764 |
| YMR269W | TMA23 | 0.36906721 | 0.99917121 | 0.25683142 | 108.5563969 | 141.5507389 |
| YNL304W | YPT11 | 0.08735624 | 0.94753174 | 0.25699844 | 137.5908461 | 187.7637717 |
| YMR115W | FMP24 | 0.10310245 | 0.94753174 | 0.25712682 | 129.2292562 | 174.4334316 |
| YJR019C | TES1 | 0.10274709 | 0.94753174 | 0.25730266 | 151.619396 | 210.0652342 |
| YOR248W | YOR248W | 0.19131539 | 0.99917121 | 0.25739253 | 149.5961113 | 206.8324733 |
| YMR183C | SSO2 | 0.12383572 | 0.97578308 | 0.25766205 | 124.0109882 | 166.0588016 |
| YLL051C | FRE6 | 0.12958972 | 0.97616733 | 0.25773624 | 137.8092471 | 188.0216004 |
| YLL009C | COX17 | 0.01432191 | 0.84351098 | 0.25774986 | 104.7501648 | 135.3778663 |
| YFL034C-A | RPL22B | 0.16591107 | 0.99381655 | 0.25775639 | 148.9515543 | 205.7617465 |
| YJR077C | MIR1 | 0.16606178 | 0.99381655 | 0.25782358 | 118.0603367 | 156.5635103 |
| YER166W | DNF1 | 0.02978847 | 0.94753174 | 0.25837559 | 145.4703578 | 200.1429287 |
| YLR412W | YLR412W | 0.19676743 | 0.99917121 | 0.25872276 | 135.4343629 | 184.1196544 |

| | | | | | | |
|---|---|---|---|---|---|---|
| YBR168W | PEX32 | 0.18975674 | 0.99917121 | 0.25918442 | 138.2632264 | 188.5679511 |
| YGR118W | RPS23A | 0.10570233 | 0.94753174 | 0.25920651 | 116.3200614 | 153.6237659 |
| YGL173C | KEM1 | 0.52915633 | 0.99917121 | 0.25928228 | 68.51611937 | 77.4932799 |
| YPR053C | YPR053C | 0.14631058 | 0.98328895 | 0.26024199 | 144.6518528 | 198.6120379 |
| YMR316W | DIA1 | 0.08010625 | 0.94753174 | 0.26037629 | 137.4329993 | 187.1006263 |
| YLR173W | YLR173W | 0.06520661 | 0.94753174 | 0.26043461 | 140.0583495 | 191.2740289 |
| YIR034C | LYS1 | 0.21133687 | 0.99917121 | 0.2605242 | 128.506971 | 172.8691136 |
| YHR182W | YHR182W | 0.08632683 | 0.94753174 | 0.26062826 | 150.8108442 | 208.3723001 |
| YML062C | MFT1 | 0.13087144 | 0.97732514 | 0.26100614 | 123.8681209 | 165.4236257 |
| YHR045W | YHR045W | 0.13251711 | 0.97853666 | 0.26200364 | 153.0865934 | 211.8284464 |
| YDR471W | RPL27B | 0.19934882 | 0.99917121 | 0.2621134 | 128.7086582 | 172.9965331 |
| YJR074W | MOG1 | 0.56271449 | 0.99917121 | 0.26226205 | 143.8447183 | 197.0805187 |
| YLR188W | MDL1 | 0.28285498 | 0.99917121 | 0.26226263 | 144.6146546 | 198.3064664 |
| YLR138W | NHA1 | 0.01240761 | 0.84351098 | 0.26250359 | 109.7195084 | 142.7113372 |
| YDR438W | THI74 | 0.05668414 | 0.94753174 | 0.26285718 | 153.0818583 | 211.7168507 |
| YBR118W | TEF2 | 0.29250313 | 0.99917121 | 0.2636642 | 134.6630699 | 182.2890611 |
| YFL048C | EMP47 | 0.01351202 | 0.84351098 | 0.2638047 | 143.5907833 | 196.4880974 |
| YNL316C | PHA2 | 0.07588685 | 0.94753174 | 0.2642203 | 138.4625735 | 188.2714568 |
| YDR430C | CYM1 | 0.33653539 | 0.99917121 | 0.26451665 | 155.4802646 | 215.3336776 |
| YPR031W | NTO1 | 0.11376345 | 0.95531631 | 0.26466938 | 138.5957398 | 188.4287583 |
| YMR018W | YMR018W | 0.06216294 | 0.94753174 | 0.26473065 | 135.8598926 | 184.0648261 |
| YML103C | NUP188 | 0.22762577 | 0.99917121 | 0.26484563 | 123.153463 | 163.8175546 |
| YLR228C | ECM22 | 0.01092195 | 0.84351098 | 0.26506532 | 132.7320769 | 179.0434075 |
| YGR149W | YGR149W | 0.12099369 | 0.96390893 | 0.2656 | 155.7707756 | 215.6642044 |
| YMR243C | ZRC1 | 0.02846647 | 0.94311309 | 0.26710768 | 130.5665852 | 175.3461713 |
| YAL018C | YAL018C | 0.30142212 | 0.99917121 | 0.26730014 | 158.737929 | 220.1817257 |
| YLR037C | DAN2 | 0.0797953 | 0.94753174 | 0.26749969 | 147.2211276 | 201.8184656 |
| YER158C | YER158C | 0.12872147 | 0.97616733 | 0.26826289 | 142.207872 | 193.7424983 |
| YLR453C | RIF2 | 0.14464079 | 0.98328895 | 0.26829767 | 148.1459789 | 203.1938831 |
| YDR032C | PST2 | 0.26110254 | 0.99917121 | 0.26846381 | 144.5157837 | 197.3930388 |
| YOR182C | RPS30B | 0.17005586 | 0.99636201 | 0.26861811 | 108.017451 | 139.255618 |
| YPR018W | RLF2 | 0.26055402 | 0.99917121 | 0.26895975 | 140.0878137 | 190.2816406 |
| YPL106C | SSE1 | 0.39590468 | 0.99917121 | 0.26911944 | 111.1033165 | 144.108319 |
| YER052C | HOM3 | 0.23353651 | 0.99917121 | 0.26983575 | 125.8007184 | 167.424599 |
| YNL273W | TOF1 | 0.35517142 | 0.99917121 | 0.26993389 | 152.1064093 | 209.3008547 |
| YLR414C | YLR414C | 0.39833125 | 0.99917121 | 0.27013729 | 119.2970566 | 157.0316445 |
| YMR244C-A | YMR244C-A | 0.0262541 | 0.92955221 | 0.27074461 | 135.0075015 | 181.9743443 |
| YBR239C | YBR239C | 0.3629741 | 0.99917121 | 0.27098858 | 177.2460147 | 249.2036641 |
| YDR440W | DOT1 | 0.0835568 | 0.94753174 | 0.27103102 | 143.6058326 | 195.6310947 |
| YLR181C | VTA1 | 0.03192579 | 0.94753174 | 0.27138246 | 145.7518151 | 199.0054348 |
| YDR315C | IPK1 | 0.1943827 | 0.99917121 | 0.27163379 | 133.9412149 | 180.1680274 |
| YNR019W | ARE2 | 0.37624306 | 0.99917121 | 0.27169575 | 143.7890216 | 195.841761 |
| YER142C | MAG1 | 0.04110793 | 0.94753174 | 0.27196048 | 145.6950168 | 198.844525 |
| YGL014W | PUF4 | 0.14588085 | 0.98328895 | 0.27259034 | 148.8769072 | 203.834464 |
| YPR043W | RPL43A | 0.07355403 | 0.94753174 | 0.27270976 | 103.8551971 | 132.1289805 |
| YIL122W | POG1 | 0.23934427 | 0.99917121 | 0.27277916 | 145.9114384 | 199.0893409 |
| YLR125W | YLR125W | 0.35703511 | 0.99917121 | 0.2729042 | 148.0614 | 202.497617 |
| YML016C | PPZ1 | 0.11359389 | 0.95531631 | 0.27358448 | 140.905757 | 191.0202991 |
| YFR032C | YFR032C | 0.23456907 | 0.99917121 | 0.27480012 | 164.9926978 | 229.2272632 |
| YJR054W | YJR054W | 0.10371271 | 0.94753174 | 0.27486212 | 177.4788957 | 249.1022695 |
| YER156C | YER156C | 0.06821438 | 0.94753174 | 0.2750305 | 146.4062032 | 199.6027231 |
| YHR066W | SSF1 | 0.1513633 | 0.98328895 | 0.27539723 | 129.6330227 | 172.8490158 |
| YNL198C | YNL198C | 0.07639566 | 0.94753174 | 0.27595217 | 103.4789567 | 131.1345851 |
| YKL032C | IXR1 | 0.22710327 | 0.99917121 | 0.27617005 | 146.7896755 | 200.0744272 |
| YLR326W | YLR326W | 0.02619888 | 0.92955221 | 0.27621781 | 148.3867089 | 202.6116628 |
| YLR113W | HOG1 | 0.172787 | 0.99917121 | 0.27646561 | 149.0449536 | 203.6296177 |
| YLR131C | ACE2 | 0.10030317 | 0.94753174 | 0.27674333 | 138.1198819 | 186.1990759 |
| YKL205W | LOS1 | 0.0475915 | 0.94753174 | 0.27677781 | 123.1526645 | 162.361623 |
| YDL117W | CYK3 | 0.21817069 | 0.99917121 | 0.27697032 | 147.2784543 | 200.7551795 |
| YLR284C | ECI1 | 0.28797103 | 0.99917121 | 0.27746481 | 134.2694374 | 179.9798129 |
| YCL029C | BIK1 | 0.13643237 | 0.97853666 | 0.27822146 | 135.0288708 | 181.0968631 |
| YLR024C | UBR2 | 0.15046689 | 0.98328895 | 0.27878631 | 176.4384907 | 246.9671661 |
| YER060W | FCY21 | 0.06821597 | 0.94753174 | 0.27942807 | 145.7739786 | 198.0598812 |
| YFR014C | CMK1 | 0.04687906 | 0.94753174 | 0.27955072 | 180.4680255 | 253.2904607 |

| | | | | | | |
|---|---|---|---|---|---|---|
| YDR006C | SOK1 | 0.08986737 | 0.94753174 | 0.27955498 | 180.3225678 | 253.0583198 |
| YOL128C | YGK3 | 0.11066264 | 0.95531631 | 0.27965214 | 151.7081523 | 207.4819264 |
| YLR054C | OSW2 | 0.06711811 | 0.94753174 | 0.27966945 | 137.1448304 | 184.2897152 |
| YGL029W | CGR1 | 0.20750915 | 0.99917121 | 0.28205323 | 147.8559432 | 201.0550905 |
| YDR525W | API2 | 0.03430359 | 0.94753174 | 0.28221707 | 150.6859565 | 205.541526 |
| YOR008C | SLG1 | 0.01821584 | 0.87709187 | 0.28347804 | 115.8995058 | 149.9951287 |
| YEL047C | YEL047C | 0.07330386 | 0.94753174 | 0.28388249 | 144.9192888 | 196.1558634 |
| YMR035W | IMP2 | 0.00638157 | 0.84351098 | 0.28430029 | 144.457851 | 195.3701517 |
| YMR122C | YMR122C | 0.29099816 | 0.99917121 | 0.28478012 | 147.1885736 | 199.6599591 |
| YLR366W | YLR366W | 0.02257723 | 0.88145277 | 0.28585986 | 136.6631613 | 182.7680455 |
| YIL125W | KGD1 | 0.32906073 | 0.99917121 | 0.28616184 | 187.6085477 | 263.8548008 |
| YGR041W | BUD9 | 0.01504773 | 0.84351098 | 0.28627331 | 158.0971023 | 216.8482644 |
| YGR017W | YGR017W | 0.1744705 | 0.99917121 | 0.28698854 | 158.343697 | 217.1537395 |
| YEL056W | HAT2 | 0.0618031 | 0.94753174 | 0.28826375 | 149.1903526 | 202.4228265 |
| YLR289W | GUF1 | 0.14023234 | 0.97853666 | 0.2886109 | 153.56896 | 209.3528395 |
| YIL015W | BAR1 | 0.21021025 | 0.99917121 | 0.28862218 | 177.3994546 | 247.2982723 |
| YLR333C | RPS25B | 0.02662667 | 0.92955221 | 0.28928401 | 135.5084832 | 180.511937 |
| YLL049W | LDB18 | 0.00483699 | 0.84351098 | 0.29034455 | 134.5833832 | 178.9095512 |
| YFR017C | YFR017C | 0.05319859 | 0.94753174 | 0.29040314 | 163.4008731 | 224.7903251 |
| YLR407W | YLR407W | 0.02046728 | 0.87709187 | 0.29067825 | 131.7841992 | 174.4115509 |
| YMR294W-A | YMR294W-A | 0.15333919 | 0.98492771 | 0.29089927 | 141.4328609 | 189.7487821 |
| YML022W | APT1 | 0.49664389 | 0.99917121 | 0.29093561 | 107.4373062 | 135.6110743 |
| YOR069W | VPS5 | 0.20415334 | 0.99917121 | 0.29099168 | 135.1109075 | 179.6706693 |
| YLR324W | PEX30 | 0.14648473 | 0.98328895 | 0.29123499 | 144.1165087 | 193.9811975 |
| YLR085C | ARP6 | 0.07503309 | 0.94753174 | 0.29217869 | 129.4223349 | 170.4676848 |
| YKR009C | FOX2 | 0.02036014 | 0.87709187 | 0.29285196 | 146.7588203 | 197.9915917 |
| YGR168C | YGR168C | 0.13430946 | 0.97853666 | 0.29287167 | 125.7608206 | 164.5527424 |
| YJR120W | YJR120W | 0.16294615 | 0.99381655 | 0.2928767 | 125.7514472 | 164.5372026 |
| YMR015C | ERG5 | 0.05792281 | 0.94753174 | 0.29331525 | 126.2471665 | 165.2731047 |
| YNL192W | CHS1 | 0.47743016 | 0.99917121 | 0.29418393 | 150.8801706 | 204.3918975 |
| YMR136W | GAT2 | 0.02828304 | 0.94311309 | 0.29507885 | 142.3931374 | 190.7683555 |
| YGL024W | YGL024W | 0.33407626 | 0.99917121 | 0.29541762 | 115.2759623 | 147.5466591 |
| YDL173W | YDL173W | 0.42371059 | 0.99917121 | 0.29574776 | 158.2966397 | 216.010964 |
| YBR187W | GDT1 | 0.20451946 | 0.99917121 | 0.29624149 | 158.3050283 | 215.9641303 |
| YNR033W | ABZ1 | 0.26080923 | 0.99917121 | 0.29697584 | 152.8008638 | 207.1099757 |
| YMR124W | YMR124W | 0.14632058 | 0.98328895 | 0.29753622 | 143.1727412 | 191.7101894 |
| YDL242W | YDL242W | 0.20308845 | 0.99917121 | 0.29822651 | 145.0990128 | 194.6933602 |
| YKL137W | YKL137W | 0.05072066 | 0.94753174 | 0.29911986 | 133.0850919 | 175.4539225 |
| YLR292C | SEC72 | 0.11529971 | 0.95531631 | 0.2991327 | 136.3280273 | 180.616289 |
| YBR263W | SHM1 | 0.07622067 | 0.94753174 | 0.29963148 | 153.451675 | 207.8225524 |
| YFL054C | YFL054C | 0.0259811 | 0.92955221 | 0.30030901 | 153.7959439 | 208.288156 |
| YBR048W | RPS11B | 0.05785974 | 0.94753174 | 0.30082677 | 120.420274 | 155.0788399 |
| YLR294C | YLR294C | 0.0911483 | 0.94753174 | 0.30117612 | 144.3701761 | 193.173198 |
| YGL016W | KAP122 | 0.13131802 | 0.97853666 | 0.30144897 | 140.1131881 | 186.3612627 |
| YER053C | PIC2 | 0.03722773 | 0.94753174 | 0.30161362 | 153.9519851 | 208.377584 |
| YIL070C | MAM33 | 0.14082313 | 0.97853666 | 0.30200691 | 176.1684455 | 243.7063163 |
| YEL053C | MAK10 | 0.00982168 | 0.84351098 | 0.30432592 | 147.7493618 | 198.1700946 |
| YKL074C | MUD2 | 0.07149272 | 0.94753174 | 0.30582783 | 137.262897 | 181.2887338 |
| YER063W | THO1 | 0.07099007 | 0.94753174 | 0.30669911 | 154.6999443 | 208.9486308 |
| YLR006C | SSK1 | 0.23879133 | 0.99917121 | 0.30821638 | 143.9408298 | 191.63124 |
| YLR168C | YLR168C | 0.07707354 | 0.94753174 | 0.30838964 | 129.8522866 | 169.1760363 |
| YGL147C | RPL9A | 0.21820309 | 0.99917121 | 0.30844726 | 118.9047151 | 151.7364994 |
| YLR269C | YLR269C | 0.17753021 | 0.99917121 | 0.30848616 | 138.7432056 | 183.3218434 |
| YOL064C | MET22 | 0.10529787 | 0.94753174 | 0.30883706 | 135.0932614 | 177.4670278 |
| YFL036W | RPO41 | 0.10301298 | 0.94753174 | 0.30903467 | 123.7452724 | 159.3728144 |
| YCR031C | RPS14A | 0.20909576 | 0.99917121 | 0.3093647 | 141.0668684 | 186.9148555 |
| YNL171C | 0 | 0.09583229 | 0.94753174 | 0.30938537 | 117.8314098 | 149.913042 |
| YGR069W | YGR069W | 0.04712832 | 0.94753174 | 0.31009049 | 156.8840741 | 212.0131145 |
| YLR404W | YLR404W | 0.05284165 | 0.94753174 | 0.31018273 | 132.0809631 | 172.506303 |
| YNL329C | PEX6 | 0.03783005 | 0.94753174 | 0.31027896 | 135.0264978 | 177.1849332 |
| YMR123W | PKR1 | 0.47529803 | 0.99917121 | 0.3108734 | 131.1675808 | 170.9676653 |
| YFR034C | PHO4 | 0.03269932 | 0.94753174 | 0.31164271 | 161.3767629 | 218.9778757 |
| YLR044C | PDC1 | 0.31636988 | 0.99917121 | 0.31354457 | 124.0431036 | 159.2972649 |
| YPR121W | THI22 | 0.28699021 | 0.99917121 | 0.31356309 | 164.8164338 | 224.2209662 |

| | | | | | | |
|---|---|---|---|---|---|---|
| YOR183W | FYV12 | 0.10681071 | 0.9482222 | 0.31380037 | 115.1424259 | 145.0929661 |
| YML081C-A | ATP18 | 0.03230436 | 0.94753174 | 0.31405991 | 121.9930847 | 155.9700641 |
| YGR148C | RPL24B | 0.17605253 | 0.99917121 | 0.31489776 | 128.8371311 | 166.7661306 |
| YMR193C-A | YMR193C-A | 0.00885504 | 0.84351098 | 0.31520416 | 119.7581159 | 152.2716854 |
| YCR060W | TAH1 | 0.3186226 | 0.99917121 | 0.32044652 | 144.8208362 | 191.5415443 |
| YLR388W | RPS29A | 0.07297641 | 0.94753174 | 0.32107649 | 122.3082588 | 155.6165402 |
| YEL005C | VAB2 | 0.00305563 | 0.62480271 | 0.32135711 | 153.299831 | 204.932175 |
| YMR219W | ESC1 | 0.19062251 | 0.99917121 | 0.32227374 | 141.248564 | 185.6304314 |
| YMR020W | FMS1 | 0.09938499 | 0.94753174 | 0.32270564 | 140.7057606 | 184.713438 |
| YEL061C | CIN8 | 0.052548 | 0.94753174 | 0.32369942 | 125.8178651 | 160.8853452 |
| YEL042W | GDA1 | 0.02185581 | 0.87709187 | 0.32513306 | 146.6182141 | 193.8322835 |
| YML033W | YML033W | 0.03994085 | 0.94753174 | 0.32570992 | 146.1757247 | 193.0573542 |
| YML090W | YML090W | 0.03037796 | 0.94753174 | 0.32609914 | 129.041378 | 165.7257973 |
| YIL059C | YIL059C | 0.34515969 | 0.99917121 | 0.32660488 | 173.3253288 | 236.1802854 |
| YPR092W | YPR092W | 0.19809607 | 0.99917121 | 0.32673922 | 152.2753817 | 202.644743 |
| YNL236W | SIN4 | 0.50901265 | 0.99917121 | 0.32704169 | 33.32261283 | 13.19183114 |
| YOL115W | PAP2 | 0.212492 | 0.99917121 | 0.32744839 | 136.3083003 | 177.1328912 |
| YNR004W | YNR004W | 0.44672824 | 0.99917121 | 0.32760256 | 131.6534749 | 169.7019219 |
| YOL073C | YOL073C | 0.23167069 | 0.99917121 | 0.32817738 | 150.4795162 | 199.6097447 |
| YLR374C | YLR374C | 0.08314372 | 0.94753174 | 0.32836983 | 128.3490413 | 164.3465252 |
| YAL058W | CNE1 | 0.06951052 | 0.94753174 | 0.32895048 | 145.2089132 | 191.1227803 |
| YGR154C | GTO1 | 0.07152061 | 0.94753174 | 0.32931507 | 164.0801998 | 221.1282795 |
| YGL244W | RTF1 | 0.48132156 | 0.99917121 | 0.32948531 | 135.4238676 | 175.4762299 |
| YER074W | RPS24A | 0.28324389 | 0.99917121 | 0.33023257 | 132.1893123 | 170.2345442 |
| YER007C-A | TMA20 | 0.03389001 | 0.94753174 | 0.33025301 | 140.4948584 | 183.4574998 |
| YGL213C | SKI8 | 0.37470033 | 0.99917121 | 0.33101367 | 161.332658 | 216.5461171 |
| YLR150W | STM1 | 0.01576822 | 0.84351098 | 0.33127162 | 135.9604915 | 176.1129609 |
| YGL170C | SPO74 | 0.02816479 | 0.94311309 | 0.33189867 | 164.0855194 | 220.8217812 |
| YDR447C | RPS17B | 0.07656658 | 0.94753174 | 0.33195245 | 133.8846132 | 172.7244077 |
| YMR205C | PFK2 | 0.06467181 | 0.94753174 | 0.3327681 | 130.4664746 | 167.1820517 |
| YIL134W | FLX1 | 0.61199028 | 0.99917121 | 0.33289936 | 56.51783078 | 49.41293443 |
| YLL053C | YLL053C | 0.01370478 | 0.84351098 | 0.33295959 | 139.4099981 | 181.4000468 |
| YOL050C | :::GAL11 | 0.02097179 | 0.87709187 | 0.33302935 | 142.6414308 | 186.5371573 |
| YLR038C | COX12 | 0.03523011 | 0.94753174 | 0.33376003 | 102.6224983 | 122.723396 |
| YIL047C | SYG1 | 0.22195162 | 0.99917121 | 0.33392458 | 168.0226689 | 226.8441744 |
| YLR111W | YLR111W | 0.07023686 | 0.94753174 | 0.33403857 | 132.9542592 | 170.9886253 |
| YBR036C | CSG2 | 0.27934948 | 0.99917121 | 0.33454477 | 135.4873774 | 174.9605583 |
| YKL048C | ELM1 | 0.02074175 | 0.87709187 | 0.33466904 | 133.3329921 | 171.5148444 |
| YKL053C-A | MDM35 | 0.3217911 | 0.99917121 | 0.33524166 | 111.7607001 | 137.0941076 |
| YLR190W | MMR1 | 0.16376333 | 0.99381655 | 0.33569373 | 115.965601 | 143.7347256 |
| YGL237C | HAP2 | 0.06573833 | 0.94753174 | 0.33582192 | 117.844281 | 146.7106394 |
| YMR067C | UBX4 | 0.02800064 | 0.94311309 | 0.33683088 | 128.3642044 | 163.339177 |
| YMR256C | COX7 | 0.28921133 | 0.99917121 | 0.33685006 | 107.9675736 | 130.8579904 |
| YLR342W | FKS1 | 0.04710789 | 0.94753174 | 0.33719082 | 121.7974469 | 152.8386327 |
| YBL079W | NUP170 | 0.18992676 | 0.99917121 | 0.33749191 | 132.4691241 | 169.7951144 |
| YDR520C | YDR520C | 0.0159116 | 0.84351098 | 0.33935755 | 152.0752214 | 200.7877057 |
| YPL062W | YPL062W | 0.21223645 | 0.99917121 | 0.34000742 | 147.8006298 | 193.9017764 |
| YLR099C | ICT1 | 0.01392189 | 0.84351098 | 0.3470701 | 139.1907877 | 179.3307621 |
| YML102W | CAC2 | 0.00407176 | 0.79473315 | 0.34807256 | 126.8391865 | 159.5403138 |
| YER077C | YER077C | 0.12040525 | 0.96279357 | 0.34825186 | 144.9259142 | 188.3190986 |
| YML131W | YML131W | 0.04120863 | 0.94753174 | 0.34978493 | 155.6692682 | 205.2395249 |
| YMR265C | YMR265C | 0.11048031 | 0.95531631 | 0.35038399 | 148.8998763 | 194.3871608 |
| YDR435C | PPM1 | 0.02766338 | 0.94311309 | 0.35042131 | 130.6438879 | 165.3124436 |
| YLR110C | CCW12 | 0.0436685 | 0.94753174 | 0.35287217 | 136.1473917 | 173.7772359 |
| YER046W | SPO73 | 0.0436809 | 0.94753174 | 0.35520805 | 153.1473437 | 200.5625684 |
| YDL191W | RPL35A | 0.44695799 | 0.99917121 | 0.35535611 | 116.5024065 | 142.1924604 |
| YGR081C | SLX9 | 0.29434757 | 0.99917121 | 0.35556145 | 120.5800526 | 148.6605216 |
| YPR164W | MMS1 | 0.46262023 | 0.99917121 | 0.3561133 | 109.2671048 | 130.578922 |
| YGL149W | YGL149W | 0.0410298 | 0.94753174 | 0.35738093 | 137.5839276 | 175.5150568 |
| YLR410W | VIP1 | 0.01391155 | 0.84351098 | 0.35819632 | 127.3116709 | 159.0584862 |
| YMR263W | SAP30 | 0.0067831 | 0.84351098 | 0.35968189 | 132.4744598 | 167.0984159 |
| YML001W | YPT7 | 0.09883686 | 0.94753174 | 0.36337439 | 118.4538035 | 144.3222805 |
| YGL096W | TOS8 | 0.09540372 | 0.94753174 | 0.36745251 | 151.8337725 | 196.9781551 |
| YHL005C | YHL005C | 0.31281424 | 0.99917121 | 0.36809252 | 151.0362438 | 195.6301749 |

| | | | | | | |
|---|---|---|---|---|---|---|
| YEL006W | YEA6 | 0.07613205 | 0.94753174 | 0.36926483 | 166.4322427 | 220.0032836 |
| YGR078C | PAC10 | 0.01408947 | 0.84351098 | 0.37469265 | 137.1591398 | 172.7281566 |
| YMR319C | FET4 | 0.05995474 | 0.94753174 | 0.37477066 | 154.8625937 | 200.9089787 |
| YHR059W | FYV4 | 0.09630203 | 0.94753174 | 0.37483494 | 159.1897504 | 207.7915478 |
| YLR330W | CHS5 | 0.19132349 | 0.99917121 | 0.37525087 | 145.6534041 | 186.1860595 |
| YGL127C | SOH1 | 0.16948564 | 0.99636201 | 0.37579649 | 129.1304374 | 159.8089738 |
| YLR030W | YLR030W | 0.03752963 | 0.94753174 | 0.37730217 | 147.4702679 | 188.8290914 |
| YMR179W | SPT21 | 0.07568482 | 0.94753174 | 0.37787552 | 127.4129663 | 156.8206801 |
| YKL069W | YKL069W | 0.23752818 | 0.99917121 | 0.37925803 | 116.5038622 | 139.2808792 |
| YNL016W | PUB1 | 0.01991412 | 0.87709187 | 0.38060997 | 136.3698997 | 170.7500142 |
| YLR004C | THI73 | 0.12472837 | 0.97616733 | 0.38177001 | 148.9907911 | 190.7056395 |
| YPR167C | MET16 | 0.22821516 | 0.99917121 | 0.38365974 | 149.4653707 | 191.2309637 |
| YGL174W | BUD13 | 0.08392712 | 0.94753174 | 0.38784531 | 106.1625124 | 121.7668108 |
| YNL052W | COX5A | 0.13464098 | 0.97853666 | 0.39231792 | 155.2870654 | 199.4456929 |
| YLR065C | YLR065C | 0.09759082 | 0.94753174 | 0.39515531 | 131.7313468 | 161.5905196 |
| YOL041C | NOP12 | 0.11513796 | 0.95531631 | 0.39627714 | 122.4070705 | 146.6061204 |
| YDR108W | GSG1 | 0.20068046 | 0.99917121 | 0.39979459 | 152.0454239 | 193.372336 |
| YNL079C | TPM1 | 0.37830332 | 0.99917121 | 0.40146835 | 127.2824802 | 153.7366803 |
| YBL016W | FUS3 | 0.08107883 | 0.94753174 | 0.40224776 | 140.2462889 | 174.2847571 |
| YAL010C | MDM10 | 0.30209379 | 0.99917121 | 0.40339042 | 48.41241132 | 27.91254735 |
| YBR181C | RPS6B | 0.23171997 | 0.99917121 | 0.40491592 | 128.3736006 | 155.0538448 |
| YDR500C | RPL37B | 0.00857958 | 0.84351098 | 0.40494781 | 132.3660738 | 161.4074253 |
| YGR276C | RNH70 | 0.17928587 | 0.99917121 | 0.4062417 | 151.6075119 | 191.8890487 |
| YDR382W | RPP2B | 0.23779937 | 0.99917121 | 0.40753696 | 84.49413948 | 84.86226328 |
| YLR370C | ARC18 | 0.0676315 | 0.94753174 | 0.4095927 | 139.5193247 | 172.2317387 |
| YKL056C | TMA19 | 0.23422794 | 0.99917121 | 0.41018774 | 156.0368209 | 198.4610546 |
| YDR535C | YDR535C | 0.02124615 | 0.87709187 | 0.41518695 | 162.005449 | 207.355822 |
| YFR053C | HXK1 | 0.17655021 | 0.99917121 | 0.41808109 | 179.5712138 | 234.9740767 |
| YAL013W | DEP1 | 0.05027403 | 0.94753174 | 0.42487851 | 155.88536 | 196.4289116 |
| YKL006W | RPL14A | 0.44089307 | 0.99917121 | 0.42898438 | 36.97150945 | 6.57429619 |
| YPL157W | TGS1 | 0.0886099 | 0.94753174 | 0.42956056 | 99.89737012 | 106.7048918 |
| YGL066W | SGF73 | 0.06411692 | 0.94753174 | 0.43108515 | 133.6053426 | 160.1943705 |
| YLR357W | RSC2 | 0.21763067 | 0.99917121 | 0.43145654 | 126.5845491 | 148.9694401 |
| YLR360W | VPS38 | 0.11339884 | 0.95531631 | 0.43329101 | 139.7626955 | 169.7301969 |
| YMR162C | DNF3 | 0.04690923 | 0.94753174 | 0.44710818 | 154.4615826 | 191.4517057 |
| YOR065W | CYT1 | 0.18505299 | 0.99917121 | 0.44807009 | 54.10754079 | 31.53433357 |
| YNL325C | FIG4 | 0.18421352 | 0.99917121 | 0.44908477 | 160.701967 | 201.1476977 |
| YDR378C | LSM6 | 0.4951561 | 0.99917121 | 0.45305155 | 71.65451119 | 58.86819414 |
| YLR264W | RPS28B | 0.16917982 | 0.99636201 | 0.45702695 | 142.0926389 | 170.5466487 |
| YOR080W | DIA2 | 0.09537328 | 0.94753174 | 0.4578005 | 109.1174032 | 117.9437853 |
| YBR121C | GRS1 | 0.23913195 | 0.99917121 | 0.45911625 | 166.2841152 | 208.8135614 |
| YOR141C | ARP8 | 0.07330419 | 0.94753174 | 0.46030994 | 134.5020207 | 158.0593943 |
| YGL078C | DBP3 | 0.0087205 | 0.84351098 | 0.46127399 | 132.1806049 | 154.2453285 |
| YNL295W | YNL295W | 0.23665606 | 0.99917121 | 0.46264259 | 157.5255396 | 194.4368283 |
| YJL028W | YJL028W | 0.2054164 | 0.99917121 | 0.46680036 | 190.8559421 | 247.0040646 |
| YML035C-A | YML035C-A | 0.23263563 | 0.99917121 | 0.46766318 | 147.5510903 | 177.9418162 |
| YGL167C | PMR1 | 0.00202285 | 0.48256094 | 0.46829593 | 114.5409101 | 125.300474 |
| YER016W | BIM1 | 0.10961737 | 0.95531631 | 0.46845807 | 167.8651648 | 210.1923 |
| YGL203C | KEX1 | 0.09102012 | 0.94753174 | 0.47169825 | 170.0529894 | 213.2810988 |
| YDL065C | PEX19 | 0.44945788 | 0.99917121 | 0.47391724 | 159.2789173 | 195.8543424 |
| YLR386W | VAC14 | 0.102581 | 0.94753174 | 0.48102935 | 152.3892628 | 184.0164662 |
| YMR244W | YMR244W | 0.01589372 | 0.84351098 | 0.48246017 | 159.9691201 | 195.9119217 |
| YMR002W | MIC17 | 0.09514487 | 0.94753174 | 0.48397766 | 158.8416512 | 193.9315834 |
| YBR194W | SOY1 | 0.00739081 | 0.84351098 | 0.48513753 | 120.3161193 | 132.4435346 |
| YNL072W | RNH201 | 0.2308128 | 0.99917121 | 0.48802142 | 183.2037916 | 232.2319871 |
| YLR325C | RPL38 | 0.01050252 | 0.84351098 | 0.49486696 | 145.1544041 | 170.8089897 |
| YOR078W | BUD21 | 0.00889013 | 0.84351098 | 0.49681618 | 107.4904713 | 110.5966902 |
| YLR143W | YLR143W | 0.08810569 | 0.94753174 | 0.51655058 | 150.8946555 | 177.3060909 |
| YJR118C | ILM1 | 0.22913422 | 0.99917121 | 0.52242078 | 142.5766131 | 163.3451037 |
| YBR106W | PHO88 | 0.01456088 | 0.84351098 | 0.52958174 | 130.1888178 | 142.7462346 |
| YGR092W | DBF2 | 0.00075271 | 0.33960437 | 0.53038505 | 148.9071542 | 172.4546958 |
| YDL062W | YDL062W | 0.39100222 | 0.99917121 | 0.53113515 | 40.66348985 | 0 |
| YPL178W | CBC2 | 0.2573374 | 0.99917121 | 0.53502659 | 63.00240486 | 35.09726312 |
| YGL168W | HUR1 | 0.19608585 | 0.99917121 | 0.55097968 | 112.7254965 | 112.3296429 |

| | | | | | | |
|---|---|---|---|---|---|---|
| YOR026W | BUB3 | 0.05945089 | 0.94753174 | 0.55719672 | 139.7364267 | 154.5829357 |
| YLR373C | VID22 | 0.26574856 | 0.99917121 | 0.56179634 | 87.34825444 | 70.60118392 |
| YPR057W | BRR1 | 0.05476408 | 0.94753174 | 0.56880866 | 127.7083831 | 134.0142998 |
| YJR116W | YJR116W | 0.05475533 | 0.94753174 | 0.57379935 | 140.943097 | 154.4803553 |
| YNL081C | SWS2 | 0.45315864 | 0.99917121 | 0.5751171 | 119.7612493 | 120.5905089 |
| YKR092C | SRP40 | 0.15474199 | 0.98492771 | 0.59749661 | 117.2458804 | 113.8568254 |
| YPR069C | SPE3 | 0.34699331 | 0.99917121 | 0.60117221 | 92.80140437 | 74.48424095 |
| YOR054C | VHS3 | 0.20263157 | 0.99917121 | 0.6153476 | 158.3848621 | 177.1888085 |
| YGL054C | ERV14 | 0.03018727 | 0.94753174 | 0.63389999 | 165.9977393 | 187.0495403 |
| YML129C | COX14 | 0.16053053 | 0.99381655 | 0.64092543 | 120.9731307 | 114.4975249 |
| YIL011W | TIR3 | 0.02423829 | 0.90503648 | 0.64292609 | 226.2703901 | 281.9251267 |
| YLR048W | RPS0B | 0.10883526 | 0.95531631 | 0.64357668 | 86.19831889 | 58.80017081 |
| YLR371W | ROM2 | 0.01117961 | 0.84351098 | 0.65679603 | 127.8199168 | 123.4653003 |
| YLR089C | ALT1 | 0.17122915 | 0.99917121 | 0.71122601 | 99.19581383 | 71.24972949 |
| YNL315C | ATP11 | 0.04789698 | 0.94753174 | 0.72028964 | 106.7601477 | 82.18994455 |
| YOL052C | SPE2 | 0.06930681 | 0.94753174 | 0.74473242 | 84.13469567 | 43.18216694 |
| YPL172C | COX10 | 0.12744183 | 0.97616733 | 0.75458618 | 104.7991593 | 74.88622338 |
| YER153C | PET122 | 0.10525542 | 0.94753174 | 0.75486406 | 101.8230394 | 70.11328281 |
| YIL054W | YIL054W | 0.09248349 | 0.94753174 | 0.77279628 | 216.2169902 | 250.083896 |
| YGR125W | YGR125W | 0.01114742 | 0.84351098 | 0.799691 | 212.1474053 | 240.324884 |
| YOR293W | RPS10A | 0.01233389 | 0.84351098 | 0.98463931 | 75.38358319 | 0 |
| YIL036W | CST6 | 0.01327956 | 0.84351098 | 1.01606489 | 103.2255339 | 40.50338886 |
| YDR393W | SHE9 | 0.10678178 | 0.9482222 | 1.16431204 | 169.9503065 | 128.6805634 |

**Table S7 Fitness on RHPS4**

**Key**

| | |
|---|---|
| ORF: | Open reading frame Y-number for *yfg* deletion |
| Gene: | Standard human-readable gene name for *yfg* deletion |
| P: | p-value for the significance of difference between observed mean fitness of treated strains and predicted fitness estimated from mean fitness observed on CSM |
| Q: | False discovery rate corrected p-value (correcting for multiple testing) |
| FD: | Fitness differential (difference between observed mean fitness of treated strains and predicted fitness estimated from mean fitness observed on control media) |
| Mean_RHPS4: | Mean fitness for all replicate strains following treatment with 200μM RHPS4 |
| Mean_DMSO: | Mean fitness for all replicate strains grown on 1% DMSO |

**Supplementary Table 7: RHPS4 Screen Data**

| ORF | Gene | P | Q | FD | Mean_RHPS4 | Mean_DMSO |
|---|---|---|---|---|---|---|
| YOR275C | RIM20 | 0.01231341 | 0.59816436 | -0.9544396 | 19.36441641 | 199.6174198 |
| YPL164C | MLH3 | 0.03503923 | 0.82669471 | -0.8947531 | 24.26443142 | 198.5956206 |
| YML008C | ERG6 | 0.00244351 | 0.31772128 | -0.8831613 | 10.99046279 | 171.7164699 |
| YPR135W | CTF4 | 0.03702417 | 0.8554337 | -0.8316426 | 22.59697937 | 184.6704213 |
| YOR213C | SAS5 | 0.02687231 | 0.76802374 | -0.7929263 | 45.06101911 | 220.1785218 |
| YPL182C | YPL182C | 0.06371084 | 0.95675879 | -0.7773362 | 43.68967775 | 214.9391497 |
| YOR030W | DFG16 | 0.07362517 | 0.95675879 | -0.7741702 | 45.38886721 | 217.5843623 |
| YML035C | AMD1 | 0.00037518 | 0.10310418 | -0.7447744 | 6.633822064 | 139.8682763 |
| YGL042C | YGL042C | 0.09579998 | 0.95675879 | -0.7184702 | 44.45316745 | 206.2994985 |
| YOR054C | VHS3 | 0.13264889 | 0.95675879 | -0.6875286 | 51.08705749 | 213.447715 |
| YPL091W | GLR1 | 0.09824333 | 0.95675879 | -0.6747127 | 55.95329509 | 220.3818252 |
| YOR285W | YOR285W | 0.14248498 | 0.95675879 | -0.6737563 | 53.74784124 | 216.0817737 |
| YMR127C | SAS2 | 0.10285453 | 0.95675879 | -0.6724561 | 51.2813653 | 211.2333442 |
| YGR122W | YGR122W | 0.09252232 | 0.95675879 | -0.6673165 | 41.41574682 | 191.8506112 |
| YOR185C | GSP2 | 0.08747793 | 0.95675879 | -0.6659699 | 57.93006134 | 222.5934817 |
| YER113C | YER113C | 0.10180839 | 0.95675879 | -0.6639706 | 77.01246318 | 258.0412297 |
| YPL139C | UME1 | 0.18524371 | 0.95675879 | -0.6603261 | 59.94290265 | 225.4030375 |
| YOR268C | YOR268C | 0.11439121 | 0.95675879 | -0.6584973 | 47.77289527 | 202.2647964 |
| YDR181C | SAS4 | 0.06666703 | 0.95675879 | -0.6581432 | 60.6288301 | 226.3160398 |
| YOL103W | ITR2 | 0.12453047 | 0.95675879 | -0.6540927 | 49.17787547 | 204.1462919 |
| YLR193C | UPS1 | 0.01823323 | 0.64739045 | -0.6503841 | 14.57483989 | 138.6123795 |
| YPL105C | YPL105C | 0.06965869 | 0.95675879 | -0.6439652 | 60.84654267 | 224.2985944 |
| YOR028C | CIN5 | 0.12840049 | 0.95675879 | -0.6436773 | 66.29685783 | 234.4716289 |
| YOR129C | YOR129C | 0.12388582 | 0.95675879 | -0.6426744 | 61.64663661 | 225.578359 |
| YAL026C | DRS2 | 0.08076703 | 0.95675879 | -0.6387543 | 29.87700012 | 165.32241 |
| YOR364W | YOR364W | 0.14977044 | 0.95675879 | -0.6381596 | 50.86615834 | 204.5866737 |
| YNR055C | HOL1 | 0.11946914 | 0.95675879 | -0.6372638 | 61.733634 | 224.8158013 |
| YPL030W | TRM44 | 0.1018188 | 0.95675879 | -0.6306139 | 56.93998101 | 214.6873657 |
| YOR343C | YOR343C | 0.17092752 | 0.95675879 | -0.6273772 | 53.48188598 | 207.6477839 |
| YPR032W | SRO7 | 0.13200532 | 0.95675879 | -0.618864 | 47.91451769 | 195.7493936 |
| YOR113W | AZF1 | 0.16828243 | 0.95675879 | -0.6187673 | 49.76674112 | 199.2067777 |
| YPL041C | YPL041C | 0.11526089 | 0.95675879 | -0.6186659 | 54.31537148 | 207.7205744 |
| YNR034W | SOL1 | 0.12339371 | 0.95675879 | -0.6181378 | 59.9538509 | 218.2054071 |
| YOR175C | YOR175C | 0.17821838 | 0.95675879 | -0.6170546 | 53.37366651 | 205.6786769 |
| YNR042W | YNR042W | 0.14422172 | 0.95675879 | -0.6152169 | 65.04888735 | 227.2616138 |
| YDR074W | TPS2 | 0.20265099 | 0.95675879 | -0.6148544 | 39.95305571 | 180.1313442 |
| YOL119C | MCH4 | 0.07919878 | 0.95675879 | -0.6139574 | 52.94249518 | 204.3400919 |
| YOR298C-A | MBF1 | 0.21466774 | 0.95675879 | -0.6135761 | 55.69029025 | 209.4284454 |
| YNR021W | YNR021W | 0.11450929 | 0.95675879 | -0.6056816 | 58.44289263 | 213.2403617 |
| YPL087W | YDC1 | 0.08170179 | 0.95675879 | -0.604544 | 50.80988148 | 198.7297157 |
| YOR363C | PIP2 | 0.20246563 | 0.95675879 | -0.602898 | 58.39404004 | 212.6724932 |
| YOL155C | HPF1 | 0.09271093 | 0.95675879 | -0.600964 | 53.35724928 | 202.8948861 |
| YJR145C | RPS4A | 0.11687862 | 0.95675879 | -0.5996287 | 55.80975872 | 207.2662015 |
| YNR025C | YNR025C | 0.11784047 | 0.95675879 | -0.5992401 | 57.85631246 | 211.0381109 |
| YOL091W | SPO21 | 0.159112 | 0.95675879 | -0.5988134 | 50.86065585 | 197.8444753 |
| YPL141C | YPL141C | 0.10963393 | 0.95675879 | -0.5987581 | 55.19594382 | 205.9660144 |
| YOL101C | IZH4 | 0.21024569 | 0.95675879 | -0.5976152 | 73.64828405 | 240.3785887 |

| | | | | | | |
|---|---|---|---|---|---|---|
| YIL084C | SDS3 | 0.12568106 | 0.95675879 | -0.5970125 | 44.77291735 | 186.118544 |
| YPL145C | KES1 | 0.08539651 | 0.95675879 | -0.596612 | 50.26777775 | 196.3558603 |
| YOL113W | SKM1 | 0.17663845 | 0.95675879 | -0.5961275 | 65.47382424 | 224.7925199 |
| YOR297C | TIM18 | 0.241644 | 0.95675879 | -0.5958813 | 37.27068023 | 171.8542641 |
| YDR369C | XRS2 | 0.08343795 | 0.95675879 | -0.5955215 | 21.43061883 | 142.0840214 |
| YDR269C | YDR269C | 2.62E-07 | 0.00056262 | -0.5951814 | 46.39337777 | 188.8444962 |
| YOR071C | THI71 | 0.14842561 | 0.95675879 | -0.5916847 | 64.01306341 | 221.292673 |
| YOL099C | YOL099C | 0.17001745 | 0.95675879 | -0.5910893 | 69.01599384 | 230.5740036 |
| YOL137W | BSC6 | 0.12669497 | 0.95675879 | -0.5882131 | 58.51573254 | 210.3882335 |
| YKL150W | MCR1 | 0.09805735 | 0.95675879 | -0.5874103 | 49.66612604 | 193.6530831 |
| YOL032W | OPI10 | 0.1881686 | 0.95675879 | -0.5865224 | 71.78081935 | 234.9781681 |
| YPL170W | DAP1 | 0.24151127 | 0.95675879 | -0.5863304 | 63.07427899 | 218.6158589 |
| YPL219W | PCL8 | 0.27567479 | 0.95675879 | -0.5851131 | 63.94109396 | 220.0333273 |
| YOR197W | MCA1 | 0.2337858 | 0.95675879 | -0.5817233 | 61.45940375 | 214.798851 |
| YPL088W | YPL088W | 0.13722075 | 0.95675879 | -0.5810953 | 56.69769731 | 205.7606281 |
| YPL137C | GIP3 | 0.15270211 | 0.95675879 | -0.5798895 | 60.64447777 | 212.9566621 |
| YOR022C | YOR022C | 0.13531453 | 0.95675879 | -0.579635 | 60.97890793 | 213.5403622 |
| YPL253C | VIK1 | 0.21656601 | 0.95675879 | -0.578644 | 58.0379685 | 207.8549534 |
| YOL046C | YOL046C | 0.17143351 | 0.95675879 | -0.5781078 | 66.19963241 | 223.0707436 |
| YPR197C | YPR197C | 0.09656172 | 0.95675879 | -0.5749849 | 50.69780217 | 193.4621226 |
| YOR371C | GPB1 | 0.19180559 | 0.95675879 | -0.5741644 | 53.87393369 | 199.2787126 |
| YPL103C | FMP30 | 0.20653162 | 0.95675879 | -0.5722271 | 66.62637499 | 222.8649623 |
| YLR047C | FRE8 | 0.13690945 | 0.95675879 | -0.5662054 | 25.70830176 | 145.0911867 |
| YPR170C | YPR170C | 0.12010781 | 0.95675879 | -0.5642131 | 54.3431745 | 198.4561845 |
| YFL019C | YFL019C | 0.12265182 | 0.95675879 | -0.5631899 | 71.48615648 | 230.4334826 |
| YOL075C | YOL075C | 0.20513085 | 0.95675879 | -0.560989 | 72.00121153 | 231.022916 |
| YMR016C | SOK2 | 0.12428564 | 0.95675879 | -0.5569565 | 41.82809963 | 173.7420918 |
| YOR124C | UBP2 | 0.17012962 | 0.95675879 | -0.5542088 | 64.60645954 | 215.9937069 |
| YBR009C | HHF1 | 0.16772668 | 0.95675879 | -0.5529512 | 47.76060434 | 184.1834677 |
| YOL151W | GRE2 | 0.14382679 | 0.95675879 | -0.5522691 | 58.39673826 | 204.0152616 |
| YPL021W | ECM23 | 0.23093391 | 0.95675879 | -0.5515729 | 57.48049679 | 202.1776959 |
| YPL080C | YPL080C | 0.11587832 | 0.95675879 | -0.5505447 | 40.17750888 | 169.5493124 |
| YOR219C | STE13 | 0.14159185 | 0.95675879 | -0.5470543 | 59.0024308 | 204.2590477 |
| YFL003C | MSH4 | 0.21277438 | 0.95675879 | -0.5463562 | 52.17703362 | 191.3382894 |
| YOR344C | TYE7 | 0.19624468 | 0.95675879 | -0.5454237 | 48.90995196 | 185.0512063 |
| YOR273C | TPO4 | 0.22477673 | 0.95675879 | -0.5445347 | 52.38782768 | 191.422004 |
| YOL004W | SIN3 | 0.10316027 | 0.95675879 | -0.5442322 | 21.54103631 | 133.5158232 |
| YGL255W | ZRT1 | 0.15181733 | 0.95675879 | -0.5378913 | 74.65064556 | 232.0401671 |
| YPR141C | KAR3 | 0.05917699 | 0.95675879 | -0.537549 | 17.28378509 | 124.3877233 |
| YMR123W | PKR1 | 0.18497435 | 0.95675879 | -0.5362147 | 47.33258527 | 180.5171883 |
| YOL105C | WSC3 | 0.15447936 | 0.95675879 | -0.5347416 | 60.38578348 | 204.746955 |
| YPL135W | ISU1 | 0.16667401 | 0.95675879 | -0.5341104 | 78.60318118 | 238.8064202 |
| YPL127C | HHO1 | 0.18879762 | 0.95675879 | -0.5332063 | 61.81939328 | 207.1730672 |
| YJL028W | YJL028W | 0.18886948 | 0.95675879 | -0.5326316 | 84.05330192 | 248.7753278 |
| YNR049C | MSO1 | 0.12669652 | 0.95675879 | -0.5307571 | 55.58668017 | 195.0643184 |
| YDR455C | :::GUK1 | 0.17996119 | 0.95675879 | -0.5297419 | 43.9350395 | 173.0374915 |
| YOL054W | PSH1 | 0.14347547 | 0.95675879 | -0.5295198 | 58.28539489 | 199.9141682 |
| YPL183C | YPL183C | 0.15967873 | 0.95675879 | -0.5289119 | 29.87532694 | 146.5259198 |
| YGR050C | YGR050C | 0.10523883 | 0.95675879 | -0.5267459 | 85.1553042 | 249.8351697 |
| YPR013C | YPR013C | 0.14936802 | 0.95675879 | -0.5260215 | 16.63012754 | 121.189488 |
| YPL036W | PMA2 | 0.26593081 | 0.95675879 | -0.5251158 | 73.3414512 | 227.3989073 |
| YOL162W | YOL162W | 0.23180868 | 0.95675879 | -0.5245678 | 71.14391209 | 223.1835866 |
| YOR121C | YOR121C | 0.17208155 | 0.95675879 | -0.5211479 | 60.54078949 | 202.7118691 |
| YOR064C | YNG1 | 0.23570236 | 0.95675879 | -0.5206696 | 72.73974676 | 225.5096729 |
| YDL080C | THI3 | 0.25587904 | 0.95675879 | -0.5198454 | 50.95029379 | 184.5016629 |
| YOR100C | CRC1 | 0.22072197 | 0.95675879 | -0.5196614 | 66.20748519 | 213.0856608 |
| YOR049C | RSB1 | 0.19426652 | 0.95675879 | -0.5187759 | 65.61729399 | 211.8272206 |
| YOL160W | YOL160W | 0.21329885 | 0.95675879 | -0.5175789 | 68.5999054 | 217.2164395 |
| YPL100W | ATG21 | 0.27352872 | 0.95675879 | -0.5139031 | 60.28510614 | 200.9927807 |
| YPR089W | YPR089W | 0.13146097 | 0.95675879 | -0.5135092 | 38.62920908 | 160.30887 |
| YPR044C | YPR044C | 0.2596216 | 0.95675879 | -0.5129394 | 33.57276291 | 150.7278273 |
| YOL158C | ENB1 | 0.25745689 | 0.95675879 | -0.5118076 | 74.53362661 | 227.357939 |
| YOR311C | HSD1 | 0.20048063 | 0.95675879 | -0.5114632 | 63.10328934 | 205.8609469 |
| YOR156C | NFI1 | 0.2610496 | 0.95675879 | -0.510998 | 75.80322944 | 229.6006168 |

| | | | | | | |
|---|---|---|---|---|---|---|
| YNL032W | SIW14 | 0.1414097 | 0.95675879 | -0.5105843 | 67.12154869 | 213.2469882 |
| YBR086C | IST2 | 0.11893295 | 0.95675879 | -0.5099589 | 62.48620148 | 204.4462084 |
| YNR069C | BSC5 | 0.21906278 | 0.95675879 | -0.5090544 | 61.64060528 | 202.705497 |
| YOR212W | STE4 | 0.19626485 | 0.95675879 | -0.5081437 | 79.68543434 | 236.3934923 |
| YOR088W | YOR088W | 0.22380465 | 0.95675879 | -0.5081313 | 69.76759539 | 217.7900612 |
| YOR276W | CAF20 | 0.30536932 | 0.95675879 | -0.5062688 | 63.75884227 | 206.2017433 |
| YOR225W | YOR225W | 0.18316545 | 0.95675879 | -0.5057523 | 66.54430118 | 211.3376163 |
| YPR160W | GPH1 | 3.07E-05 | 0.03069366 | -0.5042978 | 38.06809774 | 157.680476 |
| YPR198W | SGE1 | 0.24255324 | 0.95675879 | -0.5039856 | 54.27384565 | 188.0216008 |
| YOR019W | YOR019W | 0.24322542 | 0.95675879 | -0.5032271 | 72.10207301 | 221.3293901 |
| YNR031C | SSK2 | 0.24603224 | 0.95675879 | -0.5022898 | 72.70455042 | 222.2989874 |
| YOR270C | VPH1 | 0.21585354 | 0.95675879 | -0.5022327 | 49.96039372 | 179.63163 |
| YOR277C | YOR277C | 0.18933184 | 0.95675879 | -0.5019423 | 59.71952934 | 197.8856058 |
| YGR237C | YGR237C | 0.17824213 | 0.95675879 | -0.4980743 | 75.34132085 | 226.5231246 |
| YPR145W | ASN1 | 0.24875539 | 0.95675879 | -0.4958489 | 66.51220923 | 209.5830155 |
| YLR324W | PEX30 | 0.15460623 | 0.95675879 | -0.4956435 | 63.36129406 | 203.6381998 |
| YOR271C | FSF1 | 0.21287481 | 0.95675879 | -0.4951761 | 60.3549512 | 197.9197182 |
| YOR339C | UBC11 | 0.1182885 | 0.95675879 | -0.492999 | 57.06260963 | 191.3723055 |
| YOL044W | PEX15 | 0.21812246 | 0.95675879 | -0.4927336 | 65.06265961 | 206.3313128 |
| YNR029C | YNR029C | 0.18093059 | 0.95675879 | -0.488283 | 57.83851269 | 192.0206565 |
| YOL104C | NDJ1 | 0.19695419 | 0.95675879 | -0.4855893 | 62.12491045 | 199.5990916 |
| YOL053W | YOL053W | 0.26255045 | 0.95675879 | -0.4851481 | 72.93292853 | 219.7944955 |
| YOR076C | SKI7 | 0.22317624 | 0.95675879 | -0.4838945 | 55.08658843 | 186.1084632 |
| YNL271C | BNI1 | 0.25576253 | 0.95675879 | -0.4838455 | 31.35313143 | 141.5870312 |
| YPL033C | YPL033C | 0.33061214 | 0.95675879 | -0.48361 | 64.25994831 | 203.2647991 |
| YBR187W | GDT1 | 0.20839875 | 0.95675879 | -0.4820076 | 79.87694457 | 232.2809486 |
| YOR015W | YOR015W | 0.21737425 | 0.95675879 | -0.4816831 | 78.39485029 | 229.4457111 |
| YPL035C | YPL035C | 0.3039574 | 0.95675879 | -0.4810458 | 59.29295377 | 193.5102783 |
| YNR062C | YNR062C | 0.21691789 | 0.95675879 | -0.4794785 | 62.32202364 | 198.9232751 |
| YOL001W | PHO80 | 0.17926767 | 0.95675879 | -0.4782514 | 32.84826635 | 143.4340927 |
| YPL054W | LEE1 | 0.26873729 | 0.95675879 | -0.4775542 | 66.90571385 | 207.1909374 |
| YER056C | FCY2 | 0.19472077 | 0.95675879 | -0.4773504 | 76.88521436 | 225.873015 |
| YOL068C | HST1 | 0.23794143 | 0.95675879 | -0.4772655 | 66.33814809 | 206.0770432 |
| YOL003C | PFA4 | 0.24330533 | 0.95675879 | -0.4765308 | 68.26996721 | 209.5745453 |
| YDL012C | YDL012C | 0.00036824 | 0.10310418 | -0.4759675 | 84.0579666 | 239.0892099 |
| YPL224C | MMT2 | 0.25680066 | 0.95675879 | -0.4757702 | 66.05545635 | 205.2910078 |
| YPL195W | APL5 | 0.29188678 | 0.95675879 | -0.4754725 | 57.81315441 | 189.7813035 |
| YOL060C | MAM3 | 0.24779805 | 0.95675879 | -0.4724804 | 81.97457573 | 234.5850991 |
| YOL088C | MPD2 | 0.14858478 | 0.95675879 | -0.4695303 | 53.54390042 | 180.7574691 |
| YOL061W | PRS5 | 0.24621398 | 0.95675879 | -0.4694393 | 64.39711844 | 201.0975551 |
| YOR317W | FAA1 | 0.25398955 | 0.95675879 | -0.4686835 | 63.62216653 | 199.5147885 |
| YOR041C | SRF5 | 0.22288485 | 0.95675879 | -0.4680786 | 78.08848286 | 226.5434505 |
| YNL096C | RPS7B | 0.12428371 | 0.95675879 | -0.4669873 | 48.80685694 | 171.4378674 |
| YPL072W | UBP16 | 0.28283569 | 0.95675879 | -0.4664234 | 67.10612864 | 205.6624127 |
| YLR056W | ERG3 | 0.20413123 | 0.95675879 | -0.4658572 | 56.46139232 | 185.6009041 |
| YOR252W | TMA16 | 0.17309121 | 0.95675879 | -0.4648169 | 48.43453458 | 170.3682192 |
| YPL062W | YPL062W | 0.29856956 | 0.95675879 | -0.4646393 | 57.03725123 | 186.4725645 |
| YOR011W | AUS1 | 0.30756445 | 0.95675879 | -0.4636343 | 75.84449803 | 221.57437 |
| YOR274W | MOD5 | 0.2435645 | 0.95675879 | -0.4623917 | 81.3722526 | 231.729305 |
| YPL008W | CHL1 | 0.14956039 | 0.95675879 | -0.4621536 | 57.30161442 | 186.5431128 |
| YJR078W | BNA2 | 0.30988508 | 0.95675879 | -0.4609489 | 85.80067766 | 239.7881446 |
| YOR018W | ROD1 | 0.19429692 | 0.95675879 | -0.4609447 | 68.58362253 | 207.4961382 |
| YOR186W | YOR186W | 0.22901206 | 0.95675879 | -0.4601272 | 78.5233985 | 225.9987269 |
| YMR063W | RIM9 | 0.20070404 | 0.95675879 | -0.457016 | 41.48837592 | 156.005727 |
| YOR342C | YOR342C | 0.29700418 | 0.95675879 | -0.4568665 | 71.13352104 | 211.5808287 |
| YNR019W | ARE2 | 0.2870761 | 0.95675879 | -0.4568566 | 54.03211409 | 179.5047416 |
| YNL029C | KTR5 | 0.14869347 | 0.95675879 | -0.4562065 | 35.83549073 | 145.2650167 |
| YOR378W | YOR378W | 0.2964171 | 0.95675879 | -0.455729 | 77.60731823 | 223.5280719 |
| YPL061W | ALD6 | 0.32534842 | 0.95675879 | -0.4550926 | 59.63342276 | 189.708417 |
| YOL110W | SHR5 | 0.19406477 | 0.95675879 | -0.4549589 | 70.18301487 | 209.4717336 |
| YOR299W | BUD7 | 0.26941921 | 0.95675879 | -0.4549071 | 63.93057747 | 197.7361648 |
| YOL128C | YGK3 | 0.23619083 | 0.95675879 | -0.4547532 | 79.24273808 | 226.4284161 |
| YPL099C | FMP14 | 0.35136764 | 0.95675879 | -0.4509873 | 54.04555942 | 178.5257669 |
| YOR066W | YOR066W | 0.26193901 | 0.95675879 | -0.4492746 | 81.29556594 | 229.3412167 |

| | | | | | | |
|---|---|---|---|---|---|---|
| YAL005C | SSA1 | 0.3032934 | 0.95675879 | -0.4489875 | 56.27826165 | 182.3711387 |
| YOR112W | YOR112W | 0.22050661 | 0.95675879 | -0.4481139 | 75.6108158 | 218.480658 |
| YDR183W | PLP1 | 0.24145208 | 0.95675879 | -0.4473046 | 82.14934167 | 230.6054551 |
| YNR060W | FRE4 | 0.23769944 | 0.95675879 | -0.4470705 | 80.60825454 | 227.6750284 |
| YNL325C | FIG4 | 0.1899282 | 0.95675879 | -0.4458575 | 67.79636084 | 203.4382673 |
| YOR108W | LEU9 | 0.23370196 | 0.95675879 | -0.4454609 | 76.93858128 | 220.5170219 |
| YOR083W | WHI5 | 0.2195539 | 0.95675879 | -0.4451864 | 74.5626567 | 216.0139153 |
| YEL031W | SPF1 | 0.20020673 | 0.95675879 | -0.4440234 | 39.58755379 | 150.2177112 |
| YOL147C | PEX11 | 0.26993171 | 0.95675879 | -0.4438239 | 68.21025081 | 203.8666058 |
| YOR104W | PIN2 | 0.30429949 | 0.95675879 | -0.4430143 | 72.51344509 | 211.798902 |
| YOL007C | CSI2 | 0.2366065 | 0.95675879 | -0.441384 | 76.86496294 | 219.6814196 |
| YPL004C | LSP1 | 0.24268551 | 0.95675879 | -0.4405855 | 76.1664206 | 218.2346485 |
| YOL093W | TRM10 | 0.2045051 | 0.95675879 | -0.4401117 | 56.29222348 | 180.8787165 |
| YLR072W | YLR072W | 0.18493423 | 0.95675879 | -0.4400611 | 50.40192845 | 169.8225831 |
| YDL219W | DTD1 | 0.27357757 | 0.95675879 | -0.4392357 | 86.52762557 | 237.4365607 |
| YOR352W | YOR352W | 0.25032937 | 0.95675879 | -0.43686 | 78.70126621 | 222.3514473 |
| YLR418C | CDC73 | 0.25687699 | 0.95675879 | -0.4359748 | 37.78754028 | 145.464646 |
| YOL092W | YOL092W | 0.33132231 | 0.95675879 | -0.4350132 | 74.25888357 | 213.7036063 |
| YPR179C | HDA3 | 0.05354422 | 0.95007794 | -0.4335278 | 43.45626605 | 155.6779061 |
| YOR109W | INP53 | 0.25903462 | 0.95675879 | -0.4312748 | 80.62622565 | 225.0061964 |
| YKR104W | YKR104W | 0.23802224 | 0.95675879 | -0.4304603 | 68.91289654 | 202.8980207 |
| YPL256C | CLN2 | 0.26634148 | 0.95675879 | -0.4284739 | 62.43108824 | 190.4012643 |
| YPL213W | LEA1 | 0.24185614 | 0.95675879 | -0.4269005 | 44.24850886 | 156.0298953 |
| YHL022C | SPO11 | 0.312251 | 0.95675879 | -0.4266917 | 54.65933693 | 175.5200974 |
| YPL181W | CTI6 | 0.28106376 | 0.95675879 | -0.426232 | 74.97594995 | 213.5460806 |
| YPL165C | SET6 | 0.22067985 | 0.95675879 | -0.4256797 | 71.40322933 | 206.7508095 |
| YPL152W | RRD2 | 0.31460332 | 0.95675879 | -0.4253103 | 25.56666675 | 120.7192612 |
| YGL046W | YGL046W | 0.22440464 | 0.95675879 | -0.4246827 | 65.25781836 | 195.054249 |
| YPL111W | CAR1 | 0.24801549 | 0.95675879 | -0.4246265 | 76.78399865 | 216.6624629 |
| YOL053C-A | YOL053C-A | 0.31121885 | 0.95675879 | -0.4212637 | 87.10383233 | 235.4423668 |
| YOL122C | SMF1 | 0.23837923 | 0.95675879 | -0.4207723 | 73.013815 | 208.9318986 |
| YOR091W | TMA46 | 0.24267843 | 0.95675879 | -0.4196191 | 75.38660799 | 213.1848516 |
| YNL315C | ATP11 | 0.13089881 | 0.95675879 | -0.4193401 | 23.58833764 | 115.9873623 |
| YPL090C | RPS6A | 0.35646739 | 0.95675879 | -0.4169666 | 57.0420032 | 178.3249744 |
| YGR080W | TWF1 | 0.17634865 | 0.95675879 | -0.4161938 | 82.79399273 | 226.4916545 |
| YJL082W | IML2 | 0.24633918 | 0.95675879 | -0.4147532 | 66.14867272 | 195.0262149 |
| YGL133W | ITC1 | 0.23340716 | 0.95675879 | -0.4134711 | 68.27577491 | 198.7963171 |
| YAL056W | GPB2 | 0.26053366 | 0.95675879 | -0.4127043 | 74.70868536 | 210.7303128 |
| YLR402W | YLR402W | 0.19390367 | 0.95675879 | -0.4117778 | 0 | 70.45261839 |
| YOR062C | YOR062C | 0.26869837 | 0.95675879 | -0.4099492 | 76.13017288 | 212.9249829 |
| YOL043C | NTG2 | 0.26900554 | 0.95675879 | -0.4099371 | 73.77572538 | 208.507059 |
| YPL230W | YPL230W | 0.2549654 | 0.95675879 | -0.4094555 | 74.22729068 | 209.2715785 |
| YPL221W | FLC1 | 0.32686356 | 0.95675879 | -0.4092399 | 90.91572464 | 240.5345242 |
| YPL179W | PPQ1 | 0.27127556 | 0.95675879 | -0.408674 | 59.16667487 | 180.8910708 |
| YOR008C-A | YOR008C-A | 0.27633196 | 0.95675879 | -0.4065498 | 82.24657122 | 223.81493 |
| YPL046C | ELC1 | 0.37039051 | 0.95675879 | -0.4065048 | 57.9859566 | 178.3054629 |
| YOL015W | IRC10 | 0.32171533 | 0.95675879 | -0.4063533 | 86.29693444 | 231.3779185 |
| YPL159C | PET20 | 0.24111584 | 0.95675879 | -0.4059026 | 55.42402674 | 173.397412 |
| YOR269W | PAC1 | 0.34488135 | 0.95675879 | -0.4053827 | 66.94817433 | 194.9224832 |
| YPL185W | YPL185W | 0.30998198 | 0.95675879 | -0.4041486 | 79.80805754 | 218.8305597 |
| YPL025C | YPL025C | 0.30010831 | 0.95675879 | -0.4034314 | 84.0575942 | 226.6780317 |
| YNR047W | YNR047W | 0.26143364 | 0.95675879 | -0.4029372 | 61.66467602 | 184.594645 |
| YOL079W | YOL079W | 0.26286172 | 0.95675879 | -0.4023947 | 63.20546074 | 187.3916345 |
| YOR016C | ERP4 | 0.28888253 | 0.95675879 | -0.4005497 | 77.13744309 | 213.2059697 |
| YOL031C | SIL1 | 0.43940462 | 0.95675879 | -0.4000305 | 82.81182499 | 223.7596697 |
| YOR138C | RUP1 | 0.36037526 | 0.95675879 | -0.3991261 | 90.43589493 | 237.9041907 |
| YIL125W | KGD1 | 0.03061698 | 0.78255549 | -0.398569 | 104.8178732 | 264.7828488 |
| YGL132W | YGL132W | 0.27205451 | 0.95675879 | -0.3980932 | 79.29499048 | 216.8322446 |
| YOL109W | ZEO1 | 0.27362555 | 0.95675879 | -0.3964067 | 62.39199896 | 184.8414461 |
| YNL097C | PHO23 | 0.36607589 | 0.95675879 | -0.3961972 | 55.69944975 | 172.2534529 |
| YOR003W | YSP3 | 0.36156159 | 0.95675879 | -0.3960987 | 90.11186072 | 236.7784666 |
| YOR184W | SER1 | 0.2932854 | 0.95675879 | -0.3953868 | 64.74138679 | 189.0733246 |
| YPL166W | ATG29 | 0.26765897 | 0.95675879 | -0.3945835 | 72.65801529 | 203.7838294 |
| YPR127W | YPR127W | 0.28717172 | 0.95675879 | -0.3935284 | 69.76959536 | 198.1859731 |

| | | | | | | |
|---|---|---|---|---|---|---|
| YOL098C | YOL098C | 0.29830414 | 0.95675879 | -0.3919703 | 81.39792345 | 219.7287808 |
| YBR270C | BIT2 | 0.30308479 | 0.95675879 | -0.3917506 | 83.96774708 | 224.511014 |
| YNR059W | MNT4 | 0.00332607 | 0.33544632 | -0.3912181 | 69.73124412 | 197.7187536 |
| YHR031C | RRM3 | 0.28619115 | 0.95675879 | -0.3892913 | 55.73085727 | 171.1308111 |
| YOR171C | LCB4 | 0.30233099 | 0.95675879 | -0.3876914 | 81.0616657 | 218.3660326 |
| YLL044W | YLL044W | 0.34368399 | 0.95675879 | -0.3874769 | 88.84734846 | 232.9316963 |
| YFR006W | YFR006W | 0.32547671 | 0.95675879 | -0.3860229 | 88.27758257 | 231.6142986 |
| YOR233W | KIN4 | 0.31346836 | 0.95675879 | -0.3854038 | 75.97934804 | 208.4425497 |
| YPL191C | YPL191C | 0.27331832 | 0.95675879 | -0.3838602 | 73.55259482 | 203.6269678 |
| YDR363W | ESC2 | 0.19334296 | 0.95675879 | -0.3831613 | 46.6528849 | 153.0558873 |
| YNR074C | AIF1 | 0.29761556 | 0.95675879 | -0.3831101 | 71.379041 | 199.4220469 |
| YGL232W | TAN1 | 0.37876259 | 0.95675879 | -0.3829713 | 100.50084 | 254.0174153 |
| YOR035C | SHE4 | 0.2114566 | 0.95675879 | -0.3827801 | 38.12651526 | 136.9991227 |
| YOL124C | TRM11 | 0.24587189 | 0.95675879 | -0.3823756 | 56.5102725 | 171.4093962 |
| YOR038C | HIR2 | 0.4743734 | 0.95675879 | -0.3815805 | 68.68062176 | 194.0993448 |
| YNR065C | YSN1 | 0.31600411 | 0.95675879 | -0.3811292 | 79.7153833 | 214.7182682 |
| YOR133W | EFT1 | 0.28870067 | 0.95675879 | -0.3806945 | 76.63885682 | 208.8737545 |
| YOR163W | DDP1 | 0.32472921 | 0.95675879 | -0.3806762 | 84.35117638 | 223.335391 |
| YDR220C | YDR220C | 0.37610755 | 0.95675879 | -0.3804832 | 90.62825384 | 235.0752705 |
| YBR283C | SSH1 | 0.22449842 | 0.95675879 | -0.3791666 | 66.56977064 | 189.7273506 |
| YJR083C | ACF4 | 0.28750548 | 0.95675879 | -0.3789362 | 77.02485036 | 209.2968518 |
| YOR070C | GYP1 | 0.31374867 | 0.95675879 | -0.3784148 | 65.07928787 | 186.8032613 |
| YGL210W | YPT32 | 0.28568711 | 0.95675879 | -0.3783978 | 77.45673111 | 210.0147455 |
| YDR399W | HPT1 | 0.37278226 | 0.95675879 | -0.3781232 | 97.63293191 | 247.8090586 |
| YJL196C | ELO1 | 0.27244879 | 0.95675879 | -0.377635 | 73.8457467 | 203.1116942 |
| YER178W | PDA1 | 0.25747493 | 0.95675879 | -0.3764641 | 65.7962924 | 187.8142768 |
| YOR360C | PDE2 | 0.31381652 | 0.95675879 | -0.3758726 | 66.94543342 | 189.8683382 |
| YOR013W | YOR013W | 0.30121721 | 0.95675879 | -0.3752385 | 77.59079425 | 209.7256595 |
| YOL152W | FRE7 | 0.3597631 | 0.95675879 | -0.3743945 | 81.15083004 | 216.258238 |
| YOR153W | PDR5 | 0.3655621 | 0.95675879 | -0.3741917 | 92.38330162 | 237.2905009 |
| YOL013W-A | YOL013W-A | 0.26677436 | 0.95675879 | -0.3734468 | 71.72936016 | 198.4257525 |
| YNL323W | LEM3 | 0.19096346 | 0.95675879 | -0.3731299 | 77.74251034 | 209.6494404 |
| YOR081C | TGL5 | 0.34198667 | 0.95675879 | -0.3730133 | 81.01388175 | 215.7650755 |
| YNR045W | PET494 | 0.32831846 | 0.95675879 | -0.3727994 | 79.1401727 | 212.2142686 |
| YDL188C | PPH22 | 0.32736845 | 0.95675879 | -0.3726333 | 83.96197422 | 221.2293333 |
| YER071C | YER071C | 0.30591313 | 0.95675879 | -0.372034 | 81.14169408 | 215.8372384 |
| YLR330W | CHS5 | 0.27601888 | 0.95675879 | -0.3714546 | 64.67180544 | 184.8481636 |
| YCL047C | YCL047C | 0.38346695 | 0.95675879 | -0.370348 | 97.34476171 | 245.9382948 |
| YKL214C | YRA2 | 0.29955414 | 0.95675879 | -0.3703346 | 79.43649667 | 212.348312 |
| YPR043W | RPL43A | 0.28123556 | 0.95675879 | -0.369999 | 40.98129734 | 140.1666058 |
| YFL014W | HSP12 | 0.33295835 | 0.95675879 | -0.3689225 | 85.80402159 | 224.0492631 |
| YNR044W | AGA1 | 0.49610647 | 0.95675879 | -0.3686225 | 70.01628256 | 194.3873937 |
| YPL272C | YPL272C | 0.27427093 | 0.95675879 | -0.3679796 | 67.38244858 | 189.3375308 |
| YPR185W | ATG13 | 0.12946556 | 0.95675879 | -0.3676077 | 55.11167208 | 166.2595736 |
| YBR006W | UGA2 | 0.15897015 | 0.95675879 | -0.3670662 | 77.85375861 | 208.8206336 |
| YOR042W | CUE5 | 0.31817925 | 0.95675879 | -0.3668495 | 78.3184994 | 209.6551908 |
| YOR014W | RTS1 | 0.28244654 | 0.95675879 | -0.3656086 | 55.76164762 | 167.1365867 |
| YBL066C | SEF1 | 0.19014998 | 0.95675879 | -0.3649801 | 86.4700419 | 224.6238954 |
| YBR100W | YBR100W | 0.11679779 | 0.95675879 | -0.3643647 | 71.67102458 | 196.7624435 |
| YIL094C | LYS12 | 0.32423277 | 0.95675879 | -0.3642251 | 60.93659481 | 176.6057078 |
| YHR073W | OSH3 | 0.34940136 | 0.95675879 | -0.3636155 | 84.40419868 | 220.5158533 |
| YOR381W | FRE3 | 0.29651373 | 0.95675879 | -0.3626622 | 73.49958155 | 199.900687 |
| YGL045W | RIM8 | 0.32435349 | 0.95675879 | -0.3619039 | 73.758072 | 200.2557683 |
| YPR155C | NCA2 | 0.31418393 | 0.95675879 | -0.3613704 | 73.13524337 | 198.9963361 |
| YOR040W | GLO4 | 0.37160158 | 0.95675879 | -0.3607231 | 86.38447518 | 223.7350647 |
| YER181C | YER181C | 0.30576345 | 0.95675879 | -0.3598731 | 78.16680528 | 208.1770563 |
| YPL003W | ULA1 | 0.32896537 | 0.95675879 | -0.3592057 | 75.78013743 | 203.5865853 |
| YOL063C | CRT10 | 0.33099792 | 0.95675879 | -0.359113 | 76.06908147 | 204.1126421 |
| YDR155C | CPR1 | 0.36825654 | 0.95675879 | -0.359042 | 90.15702734 | 230.5230208 |
| YDR205W | MSC2 | 0.2151241 | 0.95675879 | -0.3589299 | 84.0008032 | 218.9575907 |
| YPL174C | NIP100 | 0.37841297 | 0.95675879 | -0.3585191 | 54.25770999 | 163.1029259 |
| YIL006W | YIA6 | 0.33844751 | 0.95675879 | -0.3581075 | 79.92952593 | 211.1810358 |
| YDR524C | AGE1 | 0.2631656 | 0.95675879 | -0.3579346 | 68.46293122 | 189.6453814 |
| YBR043C | QDR3 | 0.16312339 | 0.95675879 | -0.3570478 | 78.75680543 | 208.8002435 |

| | | | | | | |
|---|---|---|---|---|---|---|
| YKL216W | URA1 | 0.42430788 | 0.95675879 | -0.35607 | 57.26158299 | 168.3177914 |
| YOR380W | RDR1 | 0.39808602 | 0.95675879 | -0.3554562 | 92.66613912 | 234.6154432 |
| YER088C | DOT6 | 0.35991993 | 0.95675879 | -0.3541147 | 86.69286884 | 223.1828111 |
| YFL056C | AAD6 | 0.3627661 | 0.95675879 | -0.3540516 | 88.1183087 | 225.845483 |
| YLR368W | MDM30 | 0.34529467 | 0.95675879 | -0.3536624 | 74.07233577 | 199.4351132 |
| YOL108C | INO4 | 0.28709076 | 0.95675879 | -0.3536302 | 42.65743625 | 140.5096778 |
| YDL203C | ACK1 | 0.3632813 | 0.95675879 | -0.3531771 | 88.13065976 | 225.7190262 |
| YPL241C | CIN2 | 0.34486517 | 0.95675879 | -0.35257 | 81.84703331 | 213.829961 |
| YPR153W | YPR153W | 0.29542419 | 0.95675879 | -0.3514519 | 71.92069862 | 195.0214145 |
| YJL177W | RPL17B | 0.40067032 | 0.95675879 | -0.3497793 | 74.29040133 | 199.179732 |
| YPR114W | YPR114W | 0.14554011 | 0.95675879 | -0.3489929 | 65.32034489 | 182.22147 |
| YGR055W | MUP1 | 0.4178742 | 0.95675879 | -0.3486101 | 29.08392566 | 114.1931095 |
| YOR325W | YOR325W | 0.42677395 | 0.95675879 | -0.3484605 | 69.46010916 | 189.8946851 |
| YNR071C | YNR071C | 0.39678758 | 0.95675879 | -0.3479868 | 86.1612623 | 221.137314 |
| YPR054W | SMK1 | 0.32202987 | 0.95675879 | -0.3479519 | 69.28508512 | 189.4794003 |
| YOR093C | YOR093C | 0.38725831 | 0.95675879 | -0.3476243 | 88.74072119 | 225.9131734 |
| YOL106W | YOL106W | 0.40793435 | 0.95675879 | -0.3476199 | 95.16302494 | 237.9577185 |
| YMR140W | SIP5 | 0.37029114 | 0.95675879 | -0.3473697 | 88.60923378 | 225.6230052 |
| YHR161C | YAP1801 | 0.35053492 | 0.95675879 | -0.3473186 | 79.21414546 | 207.9933892 |
| YOR043W | WHI2 | 0.49705556 | 0.95675879 | -0.3468904 | 82.31287184 | 213.7319124 |
| YAL051W | OAF1 | 0.33397453 | 0.95675879 | -0.3468179 | 101.93737 | 250.5260611 |
| YCL033C | YCL033C | 0.3985529 | 0.95675879 | -0.3460362 | 95.33754876 | 238.0140817 |
| YPL194W | DDC1 | 0.33613686 | 0.95675879 | -0.3454878 | 76.45787961 | 202.510667 |
| YEL061C | CIN8 | 0.11675551 | 0.95675879 | -0.3448275 | 67.63565697 | 185.851261 |
| YDR430C | CYM1 | 0.44807087 | 0.95675879 | -0.3426013 | 94.42123163 | 235.7077971 |
| YOR314W | YOR314W | 0.36227063 | 0.95675879 | -0.3423533 | 82.4095683 | 213.1370143 |
| YAL036C | RBG1 | 0.33463142 | 0.95675879 | -0.3419546 | 78.5668813 | 205.8616715 |
| YGL035C | MIG1 | 0.23006172 | 0.95675879 | -0.3417097 | 82.71911776 | 213.6074645 |
| YGL208W | SIP2 | 0.33723848 | 0.95675879 | -0.3415309 | 78.42372932 | 205.5206933 |
| YNR061C | YNR061C | 0.36459332 | 0.95675879 | -0.3412358 | 84.50730452 | 216.880199 |
| YNR030W | ALG12 | 0.37015619 | 0.95675879 | -0.3411764 | 83.14797535 | 214.3205595 |
| YDR445C | YDR445C | 0.33780011 | 0.95675879 | -0.3411327 | 69.36452585 | 188.4616614 |
| YOR170W | YOR170W | 0.38831761 | 0.95675879 | -0.3411263 | 87.24102217 | 221.9886695 |
| YDR496C | PUF6 | 0.00229133 | 0.31772128 | -0.3406323 | 55.54490181 | 162.4567832 |
| YNR056C | BIO5 | 0.4095835 | 0.95675879 | -0.3395231 | 88.22765167 | 223.5648363 |
| YDR058C | TGL2 | 0.31485438 | 0.95675879 | -0.3391688 | 71.27958665 | 191.7174214 |
| YOR374W | ALD4 | 0.35746077 | 0.95675879 | -0.338358 | 78.75661411 | 205.6021757 |
| YLR456W | YLR456W | 0.17183399 | 0.95675879 | -0.3375185 | 82.96845808 | 213.3580248 |
| YER117W | RPL23B | 0.34137762 | 0.95675879 | -0.3374381 | 78.99631678 | 205.8943501 |
| YFL020C | PAU5 | 0.28152363 | 0.95675879 | -0.3372013 | 67.70986644 | 184.6856527 |
| YOL116W | MSN1 | 0.3888669 | 0.95675879 | -0.3362735 | 85.03116 | 217.0137023 |
| YAL028W | FRT2 | 0.35915739 | 0.95675879 | -0.3358414 | 83.94577808 | 214.9040937 |
| YJL117W | PHO86 | 0.1643191 | 0.95675879 | -0.3357343 | 71.04646418 | 190.6925677 |
| YOR226C | ISU2 | 0.40897844 | 0.95675879 | -0.3356116 | 85.23360031 | 217.2801382 |
| YOR039W | CKB2 | 0.21774099 | 0.95675879 | -0.3341134 | 46.14156955 | 143.7051149 |
| YPR052C | NHP6A | 0.58544653 | 0.95959383 | -0.3333173 | 82.9578142 | 212.6192549 |
| YPL055C | LGE1 | 0.45243153 | 0.95675879 | -0.333135 | 60.22846663 | 169.9582543 |
| YBR278W | DPB3 | 0.30057831 | 0.95675879 | -0.3327033 | 69.83819257 | 187.9078325 |
| YPL270W | MDL2 | 0.46158147 | 0.95675879 | -0.3319919 | 59.16488024 | 167.7678826 |
| YOR289W | YOR289W | 0.36888092 | 0.95675879 | -0.3317456 | 82.71084075 | 211.8871488 |
| YFR024C-A | LSB3 | 0.38068826 | 0.95675879 | -0.3279689 | 85.63515787 | 216.7256464 |
| YPL073C | YPL073C | 0.43222049 | 0.95675879 | -0.3278731 | 90.07300724 | 225.032626 |
| YBR266C | YBR266C | 0.38829903 | 0.95675879 | -0.3278682 | 45.65281783 | 141.7199177 |
| YML016C | PPZ1 | 0.19664559 | 0.95675879 | -0.3272494 | 66.1226659 | 180.0060844 |
| YFL051C | YFL051C | 0.37414075 | 0.95675879 | -0.3268838 | 83.58062738 | 212.6866415 |
| YGL152C | YGL152C | 0.39477724 | 0.95675879 | -0.3266574 | 88.33685198 | 221.5683939 |
| YBR083W | TEC1 | 0.27541455 | 0.95675879 | -0.3249526 | 87.24079732 | 219.2210303 |
| YNL168C | FMP41 | 0.32732414 | 0.95675879 | -0.3248467 | 74.17884419 | 194.7046729 |
| YBL031W | SHE1 | 0.37366513 | 0.95675879 | -0.3246887 | 81.26043846 | 207.9594633 |
| YDR207C | UME6 | 0.16753038 | 0.95675879 | -0.3244185 | 76.37037869 | 198.7417272 |
| YOR288C | MPD1 | 0.59505191 | 0.95959383 | -0.3244105 | 84.50935139 | 214.0053425 |
| YER111C | SWI4 | 0.33711098 | 0.95675879 | -0.3240483 | 77.1230207 | 200.0900001 |
| YOR144C | ELG1 | 0.38460187 | 0.95675879 | -0.3237165 | 64.96129209 | 177.223426 |
| YPL086C | ELP3 | 0.47576075 | 0.95675879 | -0.3236582 | 61.44422337 | 170.6170368 |

| | | | | | | |
|---|---|---|---|---|---|---|
| YOR132W | VPS17 | 0.39302247 | 0.95675879 | -0.3232812 | 79.13920315 | 203.7401959 |
| YBR114W | RAD16 | 0.21076073 | 0.95675879 | -0.3229204 | 77.14091206 | 199.9305788 |
| YAL066W | YAL066W | 0.40497005 | 0.95675879 | -0.3225894 | 88.63995499 | 221.4408663 |
| YCL076W | YCL076W | 0.35035051 | 0.95675879 | -0.322254 | 94.22497021 | 231.8584057 |
| YDR092W | UBC13 | 0.34461314 | 0.95675879 | -0.3221262 | 76.66448974 | 198.9011417 |
| YBR042C | YBR042C | 0.36218389 | 0.95675879 | -0.3218764 | 81.14425551 | 207.2604002 |
| YOR123C | LEO1 | 0.4580094 | 0.95675879 | -0.3216372 | 70.74313166 | 187.7117379 |
| YJL029C | VPS53 | 0.38950637 | 0.95675879 | -0.3213714 | 46.81223161 | 142.7828942 |
| YBR212W | NGR1 | 0.43264133 | 0.95675879 | -0.3212704 | 91.86804627 | 227.2696094 |
| YNL128W | TEP1 | 0.38106417 | 0.95675879 | -0.3211856 | 80.14499601 | 205.2680511 |
| YBR200W | BEM1 | 0.29898152 | 0.95675879 | -0.3207335 | 65.57215842 | 177.8587584 |
| YOR312C | RPL20B | 0.38527496 | 0.95675879 | -0.3204919 | 80.3293888 | 205.4952033 |
| YOR284W | HUA2 | 0.41270893 | 0.95675879 | -0.3201486 | 79.55654155 | 203.9869489 |
| YDL186W | YDL186W | 0.4597217 | 0.95675879 | -0.3192457 | 56.98638536 | 161.5012354 |
| YKL023W | YKL023W | 0.27407978 | 0.95675879 | -0.3190468 | 63.95357281 | 174.5344383 |
| YDR313C | PIB1 | 0.21925018 | 0.95675879 | -0.3188579 | 74.38340025 | 194.0636854 |
| YDL194W | SNF3 | 0.39671128 | 0.95675879 | -0.3185792 | 83.78764498 | 211.654049 |
| YPL060W | LPE10 | 0.37542179 | 0.95675879 | -0.318496 | 73.4357853 | 192.2244817 |
| YOR365C | YOR365C | 0.21555528 | 0.95675879 | -0.317942 | 93.3292634 | 229.4407259 |
| YNL250W | RAD50 | 0.00567882 | 0.42780429 | -0.3164999 | 42.57031587 | 133.9935167 |
| YOR367W | SCP1 | 0.05791508 | 0.95675879 | -0.3159236 | 94.52682709 | 231.3414569 |
| YFR017C | YFR017C | 0.40808817 | 0.95675879 | -0.3159172 | 87.54473422 | 218.2451641 |
| YPR119W | CLB2 | 0.0073789 | 0.49536441 | -0.3155891 | 77.61980053 | 199.5744161 |
| YOL107W | YOL107W | 0.40151498 | 0.95675879 | -0.3154367 | 82.66245868 | 209.0060503 |
| YBR215W | HPC2 | 0.4209608 | 0.95675879 | -0.3151011 | 91.22520792 | 225.0084157 |
| YBR147W | RTC2 | 0.36027426 | 0.95675879 | -0.3150778 | 44.98585906 | 138.2806491 |
| YGL101W | YGL101W | 0.36385027 | 0.95675879 | -0.3150104 | 78.52403842 | 201.1713454 |
| YJL094C | KHA1 | 0.00370371 | 0.34020275 | -0.3149927 | 77.4795888 | 199.2094102 |
| YGL209W | MIG2 | 0.30526858 | 0.95675879 | -0.314538 | 87.27592053 | 217.5050218 |
| YOL081W | IRA2 | 0.36032755 | 0.95675879 | -0.314311 | 55.05789078 | 157.0399681 |
| YIR039C | YPS6 | 0.00751051 | 0.49536441 | -0.3142449 | 81.54562341 | 206.707467 |
| YDL006W | PTC1 | 0.39766447 | 0.95675879 | -0.3130941 | 48.55471921 | 144.6347964 |
| YHR117W | TOM71 | 0.40102652 | 0.95675879 | -0.3121733 | 80.33380615 | 204.0802223 |
| YJL046W | YJL046W | 0.24649657 | 0.95675879 | -0.3116937 | 23.90070073 | 98.1555847 |
| YJR120W | YJR120W | 0.38903257 | 0.95675879 | -0.311195 | 63.18507963 | 171.7497018 |
| YDR126W | SWF1 | 0.38071941 | 0.95675879 | -0.3103367 | 47.73612356 | 142.6277133 |
| YER173W | RAD24 | 0.00461751 | 0.39655204 | -0.3097557 | 79.56844053 | 202.2311113 |
| YOL014W | YOL014W | 0.43533498 | 0.95675879 | -0.3095844 | 82.71460879 | 208.1025629 |
| YDL113C | ATG20 | 0.43905357 | 0.95675879 | -0.3089567 | 85.49674769 | 213.2131943 |
| YDL222C | FMP45 | 0.26800214 | 0.95675879 | -0.3087774 | 83.03401476 | 208.5635534 |
| YGR018C | YGR018C | 0.44364271 | 0.95675879 | -0.3083531 | 92.75404078 | 226.7212692 |
| YOR304W | ISW2 | 0.34377806 | 0.95675879 | -0.3082859 | 77.7495494 | 198.56824 |
| YPL260W | YPL260W | 0.40245608 | 0.95675879 | -0.3079588 | 79.41642158 | 201.6385609 |
| YOR298W | MUM3 | 0.45583398 | 0.95675879 | -0.3075493 | 87.64414915 | 216.9999323 |
| YML050W | YML050W | 0.59668312 | 0.95959383 | -0.3071075 | 77.90911313 | 198.6658924 |
| YOR053W | YOR053W | 0.41893758 | 0.95675879 | -0.3067614 | 80.35852139 | 203.200636 |
| YOR084W | YOR084W | 0.46514929 | 0.95675879 | -0.3067303 | 84.12357507 | 210.2568284 |
| YPL024W | RMI1 | 0.38451726 | 0.95675879 | -0.3064095 | 54.10398501 | 153.8989761 |
| YOR255W | OSW1 | 0.43500728 | 0.95675879 | -0.3060383 | 86.52543919 | 214.6432271 |
| YPL257W | YPL257W | 0.09674738 | 0.95675879 | -0.3059286 | 90.16211103 | 221.4451854 |
| YDR271C | YDR271C | 0.308775 | 0.95675879 | -0.3058614 | 78.02308613 | 198.6664499 |
| YPL136W | YPL136W | 0.24244705 | 0.95675879 | -0.305645 | 79.41213297 | 201.2346328 |
| YBL051C | PIN4 | 0.30616824 | 0.95675879 | -0.3054977 | 65.1225289 | 174.4086998 |
| YFL034W | YFL034W | 0.37216793 | 0.95675879 | -0.3051992 | 73.86756823 | 190.759306 |
| YEL014C | YEL014C | 0.35601449 | 0.95675879 | -0.3048371 | 75.99981503 | 194.6964609 |
| YFL048C | EMP47 | 0.40501297 | 0.95675879 | -0.3046906 | 85.05406809 | 211.6530166 |
| YAR027W | UIP3 | 0.42256524 | 0.95675879 | -0.3042137 | 88.24673687 | 217.5594052 |
| YNL278W | CAF120 | 0.36290661 | 0.95675879 | -0.3036393 | 68.78329988 | 180.9566652 |
| YEL047C | YEL047C | 0.41664524 | 0.95675879 | -0.3027031 | 86.27483889 | 213.6025798 |
| YPR172W | YPR172W | 0.44906639 | 0.95675879 | -0.302291 | 68.16045955 | 179.5578171 |
| YLR451W | LEU3 | 0.33374728 | 0.95675879 | -0.3013423 | 69.46464532 | 181.8415543 |
| YMR173W | DDR48 | 0.28140068 | 0.95675879 | -0.3010095 | 77.0986758 | 196.1025443 |
| YFR013W | IOC3 | 0.37542076 | 0.95675879 | -0.3005813 | 78.39605796 | 198.4625816 |
| YOR050C | YOR050C | 0.54020896 | 0.95675879 | -0.3001088 | 97.18436039 | 233.619965 |

| | | | | | | |
|---|---|---|---|---|---|---|
| YER035W | EDC2 | 0.41702661 | 0.95675879 | -0.2997921 | 85.14611711 | 210.9875617 |
| YAL017W | PSK1 | 0.45913682 | 0.95675879 | -0.2989424 | 92.48046252 | 224.5980402 |
| YER145C | FTR1 | 0.09771189 | 0.95675879 | -0.2985811 | 81.87548961 | 204.6461634 |
| YOR283W | YOR283W | 0.39161722 | 0.95675879 | -0.2980926 | 78.50345563 | 198.2382059 |
| YGR100W | MDR1 | 0.44817176 | 0.95675879 | -0.2979965 | 92.4463627 | 224.3722477 |
| YLR346C | YLR346C | 0.40305824 | 0.95675879 | -0.2976992 | 79.41347523 | 199.8776768 |
| YJR137C | ECM17 | 0.34491812 | 0.95675879 | -0.297022 | 69.12841599 | 180.4717584 |
| YGR007W | MUQ1 | 0.42664783 | 0.95675879 | -0.2967033 | 86.59000701 | 213.1671557 |
| YOR032C | HMS1 | 0.47265091 | 0.95675879 | -0.296656 | 89.40920472 | 218.4465845 |
| YGR229C | SMI1 | 0.43331003 | 0.95675879 | -0.2964397 | 44.20262524 | 133.6228025 |
| YNL208W | YNL208W | 0.13446368 | 0.95675879 | -0.2960246 | 67.12392933 | 176.5416247 |
| YHL010C | YHL010C | 0.43576563 | 0.95675879 | -0.2959782 | 85.05117094 | 210.156955 |
| YKR055W | RHO4 | 0.34310645 | 0.95675879 | -0.295923 | 56.85420294 | 157.2629424 |
| YOR118W | RTC5 | 0.45829815 | 0.95675879 | -0.2940044 | 92.09891133 | 223.0375607 |
| YGR289C | MAL11 | 0.37425311 | 0.95675879 | -0.2937356 | 72.5006667 | 186.2342775 |
| YFL042C | YFL042C | 0.38355399 | 0.95675879 | -0.2937032 | 72.89386659 | 186.9661898 |
| YGL162W | SUT1 | 0.46095065 | 0.95675879 | -0.2932166 | 87.833576 | 214.9029668 |
| YAR028W | YAR028W | 0.43887031 | 0.95675879 | -0.2931741 | 81.66689138 | 203.3298292 |
| YNL294C | RIM21 | 0.19401045 | 0.95675879 | -0.2931603 | 74.23255723 | 189.3840821 |
| YGR092W | DBF2 | 0.37201235 | 0.95675879 | -0.2930318 | 70.69450692 | 182.7263406 |
| YHL008C | YHL008C | 0.42545337 | 0.95675879 | -0.293013 | 80.38128513 | 200.8910605 |
| YDR247W | VHS1 | 0.22914465 | 0.95675879 | -0.2929369 | 77.56607472 | 195.5979987 |
| YML032C | RAD52 | 0.0162779 | 0.6196335 | -0.2929132 | 51.35176059 | 146.4279436 |
| YCR027C | RHB1 | 0.35571092 | 0.95675879 | -0.2929025 | 71.86624165 | 184.901849 |
| YMR175W | SIP18 | 0.42750479 | 0.95675879 | -0.292202 | 81.81876211 | 203.4483583 |
| YOR328W | PDR10 | 0.09666519 | 0.95675879 | -0.2912192 | 97.62477428 | 232.9250138 |
| YJL092W | HPR5 | 0.42680702 | 0.95675879 | -0.2905082 | 81.16791595 | 201.9378691 |
| YJR040W | GEF1 | 0.35159343 | 0.95675879 | -0.2889562 | 93.02423586 | 223.9093423 |
| YGR052W | FMP48 | 0.43598043 | 0.95675879 | -0.288779 | 84.4929123 | 207.8781725 |
| YNL080C | EOS1 | 0.30113925 | 0.95675879 | -0.2885119 | 48.44172732 | 140.2170212 |
| YOR322C | LDB19 | 0.40329238 | 0.95675879 | -0.2884456 | 72.08483976 | 184.5492866 |
| YGR044C | RME1 | 0.44642373 | 0.95675879 | -0.288157 | 89.31321678 | 216.8124257 |
| YCR030C | SYP1 | 0.39576031 | 0.95675879 | -0.2879341 | 103.1913558 | 242.8033105 |
| YJR039W | YJR039W | 0.44342011 | 0.95675879 | -0.2878333 | 61.48168419 | 164.5578772 |
| YER041W | YEN1 | 0.21559942 | 0.95675879 | -0.2874365 | 81.96410805 | 202.9056151 |
| YEL001C | IRC22 | 0.40210842 | 0.95675879 | -0.2874348 | 78.88194482 | 197.1245871 |
| YDR202C | RAV2 | 0.49052846 | 0.95675879 | -0.2868325 | 90.01887442 | 217.9093129 |
| YHR130C | YHR130C | 0.45786781 | 0.95675879 | -0.2866879 | 85.67525087 | 209.7379227 |
| YGL254W | FZF1 | 0.42528755 | 0.95675879 | -0.2864594 | 81.18013624 | 201.2680581 |
| YGL263W | COS12 | 0.45207188 | 0.95675879 | -0.2862749 | 89.3735016 | 216.6034791 |
| YFR016C | YFR016C | 0.49888162 | 0.95675879 | -0.2858561 | 96.55869435 | 230.0079543 |
| YML028W | TSA1 | 0.01661311 | 0.62031921 | -0.2856694 | 67.37578272 | 175.2422648 |
| YOR346W | REV1 | 0.07449819 | 0.95675879 | -0.2854583 | 94.17630503 | 225.4716143 |
| YFR020W | YFR020W | 0.48095401 | 0.95675879 | -0.2852904 | 95.96411198 | 228.7959946 |
| YPR109W | YPR109W | 0.46454702 | 0.95675879 | -0.2851298 | 79.23519502 | 197.3927498 |
| YOR209C | NPT1 | 0.52427698 | 0.95675879 | -0.2850198 | 74.57038275 | 188.6248893 |
| YNL205C | YNL205C | 0.38191391 | 0.95675879 | -0.2833942 | 67.97321374 | 175.9735109 |
| YNR022C | MRPL50 | 0.46626876 | 0.95675879 | -0.283286 | 88.839677 | 215.0908877 |
| YCR024C-A | PMP1 | 0.45500365 | 0.95675879 | -0.2831707 | 75.13626674 | 189.3698655 |
| YPR120C | CLB5 | 0.45016281 | 0.95675879 | -0.2829325 | 65.05105767 | 170.4138955 |
| YNL042W | BOP3 | 0.42447 | 0.95675879 | -0.2821505 | 78.29884148 | 195.1268415 |
| YOR313C | SPS4 | 0.46421778 | 0.95675879 | -0.2816084 | 82.01858351 | 202.0106297 |
| YPR078C | YPR078C | 0.461944 | 0.95675879 | -0.2815072 | 82.03082181 | 202.0162694 |
| YCR020C | PET18 | 0.44130214 | 0.95675879 | -0.2813581 | 84.78142552 | 207.1496235 |
| YER066C-A | YER066C-A | 0.42731773 | 0.95675879 | -0.2810274 | 82.61398923 | 203.0279318 |
| YBL102W | SFT2 | 0.1684266 | 0.95675879 | -0.2808285 | 102.2054757 | 239.73853 |
| YOR008C | SLG1 | 0.39262872 | 0.95675879 | -0.2807128 | 58.98201454 | 158.6513765 |
| YOR106W | VAM3 | 0.24652542 | 0.95675879 | -0.2804828 | 29.31624823 | 102.9726725 |
| YHL027W | RIM101 | 0.5407844 | 0.95675879 | -0.2801946 | 96.01363664 | 228.0170297 |
| YBR301W | DAN3 | 0.49232582 | 0.95675879 | -0.280158 | 97.40839967 | 230.6266943 |
| YIL052C | RPL34B | 0.45731748 | 0.95675879 | -0.2800279 | 84.00298885 | 205.4620533 |
| YGL021W | ALK1 | 0.51132307 | 0.95675879 | -0.2798484 | 99.24103018 | 234.0108933 |
| YGR087C | PDC6 | 0.43901819 | 0.95675879 | -0.2795717 | 83.29072713 | 204.0481218 |
| YCR095C | OCA4 | 0.55516727 | 0.95959383 | -0.2784269 | 113.5609596 | 260.6253023 |

| | | | | | | |
|---|---|---|---|---|---|---|
| YJL053W | PEP8 | 0.46060911 | 0.95675879 | -0.2783456 | 86.75126673 | 210.3287195 |
| YDL171C | GLT1 | 0.42924999 | 0.95675879 | -0.2782061 | 82.71611993 | 202.7367744 |
| YOL085C | YOL085C | 0.47114034 | 0.95675879 | -0.2779519 | 88.79968776 | 214.1032665 |
| YGR017W | YGR017W | 0.46392195 | 0.95675879 | -0.2776739 | 97.7109162 | 230.7690595 |
| YOR044W | IRC23 | 0.52516682 | 0.95675879 | -0.277143 | 95.36724416 | 226.2825731 |
| YFR043C | IRC6 | 0.48138101 | 0.95675879 | -0.2771062 | 92.49209849 | 220.8838365 |
| YDR265W | PEX10 | 0.47692991 | 0.95675879 | -0.2766049 | 88.81053925 | 213.8931553 |
| YBL081W | YBL081W | 0.30925536 | 0.95675879 | -0.2755665 | 84.50462092 | 205.6395551 |
| YHR184W | SSP1 | 0.46240814 | 0.95675879 | -0.2750631 | 81.92814365 | 200.7211471 |
| YOR382W | FIT2 | 0.00970472 | 0.54423319 | -0.2749089 | 80.19517268 | 197.4445053 |
| YHR005C | GPA1 | 0.30316847 | 0.95675879 | -0.2739359 | 79.12384064 | 195.2686961 |
| YGR210C | YGR210C | 0.45018812 | 0.95675879 | -0.2737966 | 84.94023387 | 206.153753 |
| YDR360W | YDR360W | 0.39983454 | 0.95675879 | -0.2737565 | 88.00502948 | 211.8950355 |
| YGR045C | YGR045C | 0.25590176 | 0.95675879 | -0.2735453 | 80.57771445 | 197.92867 |
| YBR020W | GAL1 | 0.18256869 | 0.95675879 | -0.2732491 | 93.24087446 | 221.6282599 |
| YGL235W | YGL235W | 0.4301912 | 0.95675879 | -0.2730261 | 79.42263473 | 195.6734474 |
| YPL184C | MRN1 | 0.11295999 | 0.95675879 | -0.2728387 | 94.63202483 | 224.1672012 |
| YBR162W-A | YSY6 | 0.50629057 | 0.95675879 | -0.2723449 | 96.64008882 | 227.8489339 |
| YPL250C | ICY2 | 0.39769644 | 0.95675879 | -0.272299 | 71.46137004 | 180.6173578 |
| YNL190W | YNL190W | 0.1689224 | 0.95675879 | -0.2721334 | 56.15558251 | 151.8824135 |
| YPR201W | ARR3 | 0.5310687 | 0.95675879 | -0.272009 | 69.88482697 | 177.61087 |
| YIL088C | AVT7 | 0.1925227 | 0.95675879 | -0.271783 | 99.50585819 | 233.1276536 |
| YBR150C | TBS1 | 0.48059868 | 0.95675879 | -0.2712289 | 84.7970828 | 205.4459474 |
| YBR023C | CHS3 | 0.29336251 | 0.95675879 | -0.2712011 | 85.53987629 | 206.834333 |
| YOR047C | STD1 | 0.01630615 | 0.6196335 | -0.2704289 | 84.44101203 | 204.6412403 |
| YOR024W | YOR024W | 0.50397876 | 0.95675879 | -0.2699603 | 95.56275881 | 225.4203674 |
| YAR040C | YAR040C | 0.37428658 | 0.95675879 | -0.2697612 | 81.905087 | 199.7707843 |
| YJR033C | RAV1 | 0.49519886 | 0.95675879 | -0.2678465 | 74.26696096 | 185.1175613 |
| YBL009W | ALK2 | 0.54523325 | 0.95802746 | -0.267398 | 102.8762386 | 238.6986879 |
| YDR270W | CCC2 | 0.46666514 | 0.95675879 | -0.2671035 | 85.89388013 | 206.7972127 |
| YDR090C | YDR090C | 0.5747609 | 0.95959383 | -0.2671002 | 98.65272781 | 230.726371 |
| YCR048W | ARE1 | 0.49784266 | 0.95675879 | -0.2670025 | 94.0642788 | 222.103845 |
| YIR037W | HYR1 | 0.52052444 | 0.95675879 | -0.2668641 | 90.19609967 | 214.8252331 |
| YCR025C | YCR025C | 0.26342681 | 0.95675879 | -0.2660929 | 76.99775887 | 189.9392557 |
| YGR038W | ORM1 | 0.50476914 | 0.95675879 | -0.2660101 | 89.89629478 | 214.116821 |
| YLR414C | YLR414C | 0.19815833 | 0.95675879 | -0.2658312 | 60.28572795 | 158.550388 |
| YDR277C | MTH1 | 0.34666604 | 0.95675879 | -0.2656583 | 93.32421714 | 220.4858427 |
| YDR469W | SDC1 | 0.36005329 | 0.95675879 | -0.2651348 | 64.08696077 | 165.560617 |
| YGR067C | YGR067C | 0.41615358 | 0.95675879 | -0.2649913 | 67.3597293 | 171.6742631 |
| YCR068W | ATG15 | 0.44415533 | 0.95675879 | -0.2647224 | 72.65014407 | 181.5506511 |
| YDR505C | PSP1 | 0.42320654 | 0.95675879 | -0.2646342 | 76.38064768 | 188.532269 |
| YIL038C | NOT3 | 0.50025034 | 0.95675879 | -0.2644285 | 86.46958538 | 207.4192875 |
| YCR021C | HSP30 | 0.48445773 | 0.95675879 | -0.2642813 | 90.21244586 | 214.4139822 |
| YMR009W | ADI1 | 0.39772207 | 0.95675879 | -0.2642789 | 68.50947204 | 173.7087634 |
| YPR008W | HAA1 | 0.28388979 | 0.95675879 | -0.26365 | 118.7281168 | 267.7882669 |
| YDR253C | MET32 | 0.31938635 | 0.95675879 | -0.2635462 | 80.60944881 | 196.2774007 |
| YCR034W | FEN1 | 0.33040151 | 0.95675879 | -0.263515 | 23.74317877 | 89.61707504 |
| YPL265W | DIP5 | 0.64768832 | 0.96786115 | -0.2634304 | 81.55067117 | 198.0228989 |
| YIL166C | YIL166C | 0.3454216 | 0.95675879 | -0.2632734 | 90.83935494 | 215.4173366 |
| YNL183C | NPR1 | 0.46611443 | 0.95675879 | -0.2628294 | 81.853612 | 198.4882416 |
| YDR483W | KRE2 | 0.20957756 | 0.95675879 | -0.2627201 | 92.51166679 | 218.4591677 |
| YLR034C | SMF3 | 0.34833155 | 0.95675879 | -0.2623275 | 63.95032777 | 164.8240317 |
| YOL117W | RRI2 | 0.44062302 | 0.95675879 | -0.2622608 | 79.03384905 | 193.1023811 |
| YOR253W | NAT5 | 0.17143465 | 0.95675879 | -0.2619553 | 82.48771349 | 199.5279803 |
| YPL057C | SUR1 | 0.4603834 | 0.95675879 | -0.2618854 | 80.30264428 | 195.4178371 |
| YHR012W | VPS29 | 0.41596153 | 0.95675879 | -0.2618678 | 72.60150508 | 180.9710219 |
| YPL225W | YPL225W | 0.45779563 | 0.95675879 | -0.2616502 | 75.66459779 | 186.6787426 |
| YCL049C | YCL049C | 0.48555824 | 0.95675879 | -0.2614032 | 87.29448978 | 208.4488173 |
| YNR024W | YNR024W | 0.52671177 | 0.95675879 | -0.2611679 | 95.89464531 | 224.5384943 |
| YJR084W | CSN12 | 0.31352398 | 0.95675879 | -0.2610952 | 83.60712032 | 201.4803144 |
| YJL163C | YJL163C | 0.46371336 | 0.95675879 | -0.2604346 | 83.61034877 | 201.3733347 |
| YMR232W | FUS2 | 0.45374736 | 0.95675879 | -0.2600712 | 79.73814423 | 194.0486881 |
| YDR480W | DIG2 | 0.20917463 | 0.95675879 | -0.259106 | 88.48183286 | 210.2826939 |
| YER062C | HOR2 | 0.46233411 | 0.95675879 | -0.2587926 | 79.95399579 | 194.2347613 |

| | | | | | | |
|---|---|---|---|---|---|---|
| YMR119W-A | YMR119W-A | 0.46677238 | 0.95675879 | -0.2585832 | 84.92950792 | 203.5307202 |
| YHL024W | RIM4 | 0.35580484 | 0.95675879 | -0.2583573 | 74.68396369 | 184.2761224 |
| YHL032C | GUT1 | 0.57995347 | 0.95959383 | -0.2581306 | 91.00314415 | 214.8446233 |
| YHL020C | OPI1 | 0.39730634 | 0.95675879 | -0.2581146 | 27.55915602 | 95.85010994 |
| YML036W | CGI121 | 0.43822066 | 0.95675879 | -0.257611 | 76.19674762 | 186.9857353 |
| YDR067C | OCA6 | 0.37973934 | 0.95675879 | -0.2570731 | 65.62616343 | 167.0681371 |
| YDR051C | YDR051C | 0.5095514 | 0.95675879 | -0.2558161 | 90.38700153 | 213.2930271 |
| YBL089W | AVT5 | 0.41839649 | 0.95675879 | -0.2556971 | 73.60930454 | 181.8054198 |
| YGR286C | BIO2 | 0.50357039 | 0.95675879 | -0.2554308 | 86.19551439 | 205.3658043 |
| YLR228C | ECM22 | 0.40123986 | 0.95675879 | -0.2549756 | 68.61270796 | 172.3106647 |
| YER066W | YER066W | 0.49182561 | 0.95675879 | -0.2548827 | 85.52652619 | 204.0173119 |
| YPL018W | CTF19 | 0.4798661 | 0.95675879 | -0.2532254 | 83.17631715 | 199.3258529 |
| YDR255C | RMD5 | 0.5343996 | 0.95675879 | -0.2522816 | 91.75592138 | 215.2557631 |
| YBR076W | ECM8 | 0.4092082 | 0.95675879 | -0.2521568 | 67.46180267 | 169.6698108 |
| YNL162W | RPL42A | 0.44009909 | 0.95675879 | -0.2517435 | 68.60344789 | 171.7402891 |
| YBL094C | YBL094C | 0.40153447 | 0.95675879 | -0.2515683 | 85.58156498 | 203.5534757 |
| YMR105C | PGM2 | 0.02202035 | 0.71344081 | -0.2515156 | 79.38865099 | 191.9293985 |
| YBR201W | DER1 | 0.54243612 | 0.95734514 | -0.2511971 | 97.67423775 | 226.1702521 |
| YOR097C | YOR097C | 0.51596763 | 0.95675879 | -0.2511134 | 88.48106054 | 208.9137593 |
| YDL002C | NHP10 | 0.60819935 | 0.95959383 | -0.2505816 | 33.28105652 | 105.2929244 |
| YNL234W | YNL234W | 0.3573974 | 0.95675879 | -0.250445 | 58.86778542 | 153.2585073 |
| YDR266C | YDR266C | 0.48391662 | 0.95675879 | -0.2501726 | 84.87314902 | 201.986018 |
| YER182W | FMP10 | 0.06210792 | 0.95675879 | -0.2497779 | 98.83702268 | 228.1082847 |
| YEL039C | CYC7 | 0.48508556 | 0.95675879 | -0.2495107 | 84.01113113 | 200.2560135 |
| YGL140C | YGL140C | 0.5430965 | 0.95761397 | -0.2489354 | 97.59271773 | 225.6304022 |
| YGL013C | PDR1 | 0.52862146 | 0.95675879 | -0.2488905 | 93.5974647 | 218.1294674 |
| YBR231C | SWC5 | 0.27664762 | 0.95675879 | -0.2488635 | 72.74786602 | 179.0205698 |
| YMR154C | RIM13 | 0.53026524 | 0.95675879 | -0.248687 | 84.32475266 | 200.7032911 |
| YLR227C | ADY4 | 0.44799163 | 0.95675879 | -0.2486602 | 75.09892431 | 183.3952944 |
| YLR394W | CST9 | 0.41231 | 0.95675879 | -0.2483263 | 68.64000919 | 171.2242036 |
| YDR024W | FYV1 | 0.4866341 | 0.95675879 | -0.248012 | 76.08473277 | 185.1333092 |
| YPL102C | YPL102C | 0.51297513 | 0.95675879 | -0.2476918 | 63.80646348 | 162.0501349 |
| YGR058W | YGR058W | 0.32322092 | 0.95675879 | -0.2476097 | 84.40663369 | 200.6725389 |
| YJL024C | APS3 | 0.4968041 | 0.95675879 | -0.2471787 | 83.94991419 | 199.7422023 |
| YOR263C | YOR263C | 0.16883973 | 0.95675879 | -0.2468598 | 102.7490236 | 234.9461331 |
| YJL217W | YJL217W | 0.50124921 | 0.95675879 | -0.2462732 | 85.16592634 | 201.8679684 |
| YNR075W | COS10 | 0.34592191 | 0.95675879 | -0.2460164 | 86.20952057 | 203.7813348 |
| YGR097W | ASK10 | 0.38488861 | 0.95675879 | -0.2457851 | 88.84671045 | 208.6879196 |
| YMR057C | YMR057C | 0.47064146 | 0.95675879 | -0.2457776 | 80.65615882 | 193.3249192 |
| YDR225W | HTA1 | 0.20037539 | 0.95675879 | -0.2444903 | 101.7430905 | 232.6540678 |
| YPL263C | KEL3 | 0.45402317 | 0.95675879 | -0.2444838 | 73.66653373 | 179.9942342 |
| YGR011W | YGR011W | 0.44519949 | 0.95675879 | -0.2444573 | 95.60296854 | 221.1323648 |
| YOR220W | WSP1 | 0.44439205 | 0.95675879 | -0.2433463 | 84.87130273 | 200.8146184 |
| YKL175W | ZRT3 | 0.03258957 | 0.79965484 | -0.242877 | 90.16926357 | 210.6708576 |
| YIL011W | TIR3 | 0.13506313 | 0.95675879 | -0.2420769 | 121.3383647 | 268.9928822 |
| YDL204W | RTN2 | 0.49610345 | 0.95675879 | -0.2408017 | 101.8485267 | 232.2207154 |
| YOR292C | YOR292C | 0.20255871 | 0.95675879 | -0.2404462 | 104.2645654 | 236.6912759 |
| YOR368W | RAD17 | 0.02626559 | 0.76284641 | -0.2401652 | 81.8778874 | 194.6560755 |
| YBR255W | MTC4 | 0.52156541 | 0.95675879 | -0.2399109 | 90.18439331 | 210.1917429 |
| YKL217W | JEN1 | 0.40245753 | 0.95675879 | -0.239569 | 66.98465437 | 166.6211969 |
| YDR511W | ACN9 | 0.49050906 | 0.95675879 | -0.2393828 | 81.22527206 | 193.298201 |
| YPR023C | EAF3 | 0.53188168 | 0.95675879 | -0.2387338 | 73.23507232 | 178.2012163 |
| YOR161C | PNS1 | 0.39925364 | 0.95675879 | -0.2386716 | 96.93304455 | 222.6370791 |
| YBR121C | GRS1 | 0.38871404 | 0.95675879 | -0.2383784 | 86.56717711 | 203.1453204 |
| YDR029W | YDR029W | 0.49256471 | 0.95675879 | -0.2371589 | 81.045469 | 192.5804844 |
| YHR105W | YPT35 | 0.53199201 | 0.95675879 | -0.2368576 | 87.97806553 | 205.5312984 |
| YOL016C | CMK2 | 0.54118471 | 0.95675879 | -0.2367074 | 91.22159945 | 211.5889718 |
| YHR150W | PEX28 | 0.37297118 | 0.95675879 | -0.2366599 | 82.26725758 | 194.7866242 |
| YLR452C | SST2 | 0.44274987 | 0.95675879 | -0.2365977 | 72.00438138 | 175.5275278 |
| YBR288C | APM3 | 0.47841687 | 0.95675879 | -0.2364704 | 79.71578468 | 189.9688078 |
| YKR029C | SET3 | 0.42620054 | 0.95675879 | -0.2363698 | 69.23196154 | 170.2887527 |
| YJL043W | YJL043W | 0.56013819 | 0.95959383 | -0.2362456 | 94.81192656 | 218.2437763 |
| YJL192C | SOP4 | 0.35429722 | 0.95675879 | -0.2358467 | 79.70176503 | 189.8357888 |
| YBR216C | YBP1 | 0.06220889 | 0.95675879 | -0.2356978 | 96.91177225 | 222.0883952 |

| | | | | | | |
|---|---|---|---|---|---|---|
| YHL003C | LAG1 | 0.43705979 | 0.95675879 | -0.2355571 | 64.52460065 | 161.3208603 |
| YNR067C | DSE4 | 0.38710202 | 0.95675879 | -0.2353135 | 94.95921214 | 218.3605318 |
| YJR140C | HIR3 | 0.01087431 | 0.58995493 | -0.2346309 | 61.05415002 | 154.653415 |
| YJL212C | OPT1 | 0.52265746 | 0.95675879 | -0.2345093 | 82.14303938 | 194.1856787 |
| YPL158C | YPL158C | 0.46641764 | 0.95675879 | -0.2343344 | 70.5961059 | 172.4990241 |
| YHR171W | ATG7 | 0.38419837 | 0.95675879 | -0.2337548 | 76.74675236 | 183.9356389 |
| YLR093C | NYV1 | 0.428444 | 0.95675879 | -0.2335608 | 66.91116423 | 165.4554092 |
| YPL200W | CSM4 | 0.19932581 | 0.95675879 | -0.2333395 | 97.40794363 | 222.6154846 |
| YPL201C | YIG1 | 0.39178863 | 0.95675879 | -0.2331613 | 100.7289971 | 228.8137722 |
| YER064C | YER064C | 0.52844633 | 0.95675879 | -0.2326244 | 87.47535171 | 203.8641518 |
| YKR091W | SRL3 | 0.49957072 | 0.95675879 | -0.2323314 | 80.93946802 | 191.5557054 |
| YHR123W | EPT1 | 0.51171383 | 0.95675879 | -0.2319452 | 77.66449769 | 185.3472985 |
| YBL091C-A | SCS22 | 0.56918415 | 0.95959383 | -0.2319334 | 92.94075857 | 213.9965309 |
| YPL089C | RLM1 | 0.1941221 | 0.95675879 | -0.2318151 | 100.799151 | 228.7150246 |
| YHR001W-A | QCR10 | 0.5645248 | 0.95959383 | -0.2317494 | 89.19286481 | 206.9357235 |
| YNL004W | HRB1 | 0.51466797 | 0.95675879 | -0.2314758 | 84.00938698 | 197.1670912 |
| YPL212C | PUS1 | 0.63768998 | 0.9638299 | -0.2314713 | 104.0691173 | 234.7891522 |
| YER155C | BEM2 | 0.52914226 | 0.95675879 | -0.2314632 | 88.04491394 | 204.7337262 |
| YLR001C | YLR001C | 0.53682284 | 0.95675879 | -0.2312993 | 86.01399519 | 200.8966104 |
| YOL090W | MSH2 | 0.27526078 | 0.95675879 | -0.2310311 | 72.14078068 | 174.8309417 |
| YPL183W-A | RTC6 | 0.49245775 | 0.95675879 | -0.2308515 | 68.61017764 | 168.1784295 |
| YPL130W | SPO19 | 0.18436964 | 0.95675879 | -0.2307025 | 98.72639359 | 224.6371241 |
| YBR094W | PBY1 | 0.39043383 | 0.95675879 | -0.2303415 | 87.7540259 | 203.996238 |
| YMR253C | YMR253C | 0.4809114 | 0.95675879 | -0.230229 | 75.91873406 | 181.7794207 |
| YCR102W-A | YCR102W-A | 0.47981496 | 0.95675879 | -0.2298263 | 103.8602247 | 234.1159098 |
| YHR037W | PUT2 | 0.38884197 | 0.95675879 | -0.2295696 | 80.23387902 | 189.759828 |
| YDR497C | ITR1 | 0.06797553 | 0.95675879 | -0.2291068 | 97.54583377 | 222.1499148 |
| YGR059W | SPR3 | 0.23522715 | 0.95675879 | -0.2288249 | 100.6423311 | 227.909291 |
| YHL007C | STE20 | 0.53700993 | 0.95675879 | -0.2283897 | 82.07368971 | 193.0086001 |
| YNL305C | YNL305C | 0.25152838 | 0.95675879 | -0.2283637 | 73.63369383 | 177.174585 |
| YDR388W | RVS167 | 0.51385355 | 0.95675879 | -0.2278028 | 71.76253674 | 173.5691898 |
| YMR160W | YMR160W | 0.50883678 | 0.95675879 | -0.227775 | 79.85875925 | 188.7492245 |
| YGR040W | KSS1 | 0.54873765 | 0.95900765 | -0.2272544 | 87.15391984 | 202.3425219 |
| YBR092C | PHO3 | 0.61051615 | 0.9598703 | -0.2271801 | 106.5285059 | 238.6676332 |
| YBL096C | YBL096C | 0.58105597 | 0.95959383 | -0.2271381 | 99.07000184 | 224.6717219 |
| YOL048C | YOL048C | 0.57073354 | 0.95959383 | -0.2271157 | 92.68081461 | 212.684714 |
| YDR059C | UBC5 | 0.38268092 | 0.95675879 | -0.2271029 | 94.00905586 | 215.1736837 |
| YCL026C | YCL026C | 0.34235733 | 0.95675879 | -0.2269736 | 92.7353908 | 212.7627594 |
| YHL037C | YHL037C | 0.4950267 | 0.95675879 | -0.2266505 | 77.53355057 | 184.1958059 |
| YGR170W | PSD2 | 0.53485973 | 0.95675879 | -0.2259965 | 87.27304889 | 202.3507439 |
| YER162C | RAD4 | 0.51648076 | 0.95675879 | -0.2259765 | 77.69904298 | 184.3908728 |
| YNL065W | AQR1 | 0.27731643 | 0.95675879 | -0.2254765 | 76.17029947 | 181.4381148 |
| YDL190C | UFD2 | 0.56335738 | 0.95959383 | -0.2252512 | 93.09357073 | 213.1398484 |
| YML072C | TCB3 | 0.58394372 | 0.95959383 | -0.2251555 | 80.25068804 | 189.03612 |
| YBR280C | SAF1 | 0.29428337 | 0.95675879 | -0.2249036 | 107.4668811 | 240.0381028 |
| YDR151C | CTH1 | 0.52643604 | 0.95675879 | -0.2243988 | 83.89077364 | 195.7337863 |
| YGL216W | KIP3 | 0.43161827 | 0.95675879 | -0.2242913 | 88.00102716 | 203.4243474 |
| YDR049W | YDR049W | 0.49790321 | 0.95675879 | -0.2241265 | 74.73064187 | 178.5069975 |
| YOL018C | TLG2 | 0.52778034 | 0.95675879 | -0.2240526 | 66.29846244 | 162.6794691 |
| YEL012W | UBC8 | 0.55101526 | 0.95947263 | -0.2238851 | 89.32969482 | 205.8468108 |
| YKR098C | UBP11 | 0.45019561 | 0.95675879 | -0.2235053 | 67.90328091 | 165.5957281 |
| YOL062C | APM4 | 0.35930789 | 0.95675879 | -0.2231845 | 99.60865456 | 225.0055566 |
| YKR093W | PTR2 | 0.45846938 | 0.95675879 | -0.222942 | 67.53480791 | 164.8082609 |
| YHR157W | REC104 | 0.50846852 | 0.95675879 | -0.2229346 | 79.52648396 | 187.2978787 |
| YDR506C | YDR506C | 0.30722718 | 0.95675879 | -0.2228595 | 73.25774035 | 175.5277454 |
| YDR312W | SSF2 | 0.33651089 | 0.95675879 | -0.2226747 | 95.38999211 | 217.0060625 |
| YKR101W | SIR1 | 0.1645612 | 0.95675879 | -0.2225532 | 105.1452856 | 235.2817209 |
| YDR219C | MFB1 | 0.51508449 | 0.95675879 | -0.2225098 | 82.11596388 | 192.0818612 |
| YHR104W | GRE3 | 0.38761417 | 0.95675879 | -0.2216365 | 82.70086882 | 193.0294646 |
| YMR100W | MUB1 | 0.52045559 | 0.95675879 | -0.2213605 | 74.08597672 | 176.824655 |
| YIL152W | YIL152W | 0.37329691 | 0.95675879 | -0.2211188 | 83.04940384 | 193.5945757 |
| YCR092C | MSH3 | 0.30237991 | 0.95675879 | -0.2208411 | 114.2993401 | 252.1575837 |
| YGR194C | XKS1 | 0.55339255 | 0.95959383 | -0.2207482 | 83.33159098 | 194.0604239 |
| YDR034C | LYS14 | 0.02994729 | 0.78255549 | -0.2199762 | 78.65985945 | 185.1663131 |

| | | | | | | |
|---|---|---|---|---|---|---|
| YGR079W | YGR079W | 0.54207471 | 0.95710067 | -0.2188684 | 81.17369903 | 189.6915819 |
| YCR087W | YCR087W | 0.44896656 | 0.95675879 | -0.2188352 | 86.74506772 | 200.1352428 |
| YML021C | UNG1 | 0.42137033 | 0.95675879 | -0.2184747 | 62.8734342 | 155.3013362 |
| YOL041C | NOP12 | 0.48934961 | 0.95675879 | -0.2177201 | 58.27098237 | 146.5401471 |
| YAR029W | YAR029W | 0.61164514 | 0.9598703 | -0.2175661 | 102.887463 | 230.1938257 |
| YBL060W | YBL060W | 0.46252967 | 0.95675879 | -0.2169028 | 97.06218272 | 219.1547867 |
| YGL256W | ADH4 | 0.61440504 | 0.96098984 | -0.2168104 | 101.9894012 | 228.3801779 |
| YPR060C | ARO7 | 0.59909377 | 0.95959383 | -0.2166871 | 59.84452363 | 149.3146389 |
| YMR086C-A | YMR086C-A | 0.58236652 | 0.95959383 | -0.2164902 | 94.83965035 | 214.9157389 |
| YBR129C | OPY1 | 0.55920786 | 0.95959383 | -0.2162496 | 84.39710402 | 195.2891566 |
| YMR164C | MSS11 | 0.30036994 | 0.95675879 | -0.21611 | 69.62068484 | 167.5514973 |
| YLR454W | FMP27 | 0.53080277 | 0.95675879 | -0.2159608 | 73.13205192 | 174.1116836 |
| YNL333W | SNZ2 | 0.39744888 | 0.95675879 | -0.21585 | 88.7924826 | 203.4644938 |
| YDR537C | YDR537C | 0.52336607 | 0.95675879 | -0.2158128 | 79.01090627 | 185.1123844 |
| YPL092W | SSU1 | 0.17249668 | 0.95675879 | -0.2155946 | 101.1843253 | 226.6622011 |
| YMR169C | ALD3 | 0.08121675 | 0.95675879 | -0.2155559 | 84.85708122 | 196.0331712 |
| YMR138W | CIN4 | 0.5129913 | 0.95675879 | -0.2153024 | 78.34552453 | 183.777117 |
| YOR376W | YOR376W | 0.25217868 | 0.95675879 | -0.2152291 | 102.3992525 | 228.8783167 |
| YPL101W | ELP4 | 0.59216383 | 0.95959383 | -0.2152177 | 65.52841598 | 159.723621 |
| YJL122W | ALB1 | 0.17778222 | 0.95675879 | -0.2149886 | 106.1207783 | 235.8170292 |
| YNL011C | YNL011C | 0.05183039 | 0.93531943 | -0.2148022 | 86.53541228 | 199.0519976 |
| YLR389C | STE23 | 0.57597882 | 0.95959383 | -0.2143987 | 88.98040246 | 203.5686392 |
| YER055C | HIS1 | 0.39583867 | 0.95675879 | -0.2143278 | 82.21702298 | 190.8715171 |
| YDR217C | RAD9 | 0.05060517 | 0.9246749 | -0.2143194 | 82.93311247 | 192.2131335 |
| YBR105C | VID24 | 0.59435238 | 0.95959383 | -0.2142965 | 97.49943486 | 219.5289516 |
| YOL039W | RPP2A | 0.51110073 | 0.95675879 | -0.2141424 | 72.36904956 | 172.3695202 |
| YPR042C | PUF2 | 0.24021917 | 0.95675879 | -0.2140937 | 87.26513077 | 200.2993889 |
| YOR037W | CYC2 | 0.55369987 | 0.95959383 | -0.2140685 | 81.49688142 | 189.4764925 |
| YBR010W | HHT1 | 0.4169484 | 0.95675879 | -0.2138178 | 84.02072379 | 194.167171 |
| YGR093W | YGR093W | 0.30769161 | 0.95675879 | -0.213202 | 71.57065695 | 170.7111994 |
| YOL082W | ATG19 | 0.59297257 | 0.95959383 | -0.2128714 | 93.62659094 | 212.0214454 |
| YDL160C | DHH1 | 0.62709293 | 0.96098984 | -0.2125025 | 105.2952671 | 233.8434089 |
| YFR025C | HIS2 | 0.41458415 | 0.95675879 | -0.2121271 | 74.47537826 | 175.9752238 |
| YFL043C | YFL043C | 0.52780016 | 0.95675879 | -0.2120575 | 74.2240659 | 175.4919662 |
| YNL072W | RNH201 | 0.20547455 | 0.95675879 | -0.2117079 | 107.6285196 | 238.0835616 |
| YLR125W | YLR125W | 0.54090385 | 0.95675879 | -0.2116386 | 80.6684079 | 187.5069198 |
| YNL083W | SAL1 | 0.30267905 | 0.95675879 | -0.2115421 | 69.92927581 | 167.3487302 |
| YML038C | YMD8 | 0.4836307 | 0.95675879 | -0.2113335 | 64.69944037 | 157.5042696 |
| YCR006C | YCR006C | 0.56087128 | 0.95959383 | -0.2112189 | 86.18827801 | 197.7878559 |
| YPR117W | YPR117W | 0.53402558 | 0.95675879 | -0.2109104 | 78.29409997 | 182.9292164 |
| YOR051C | YOR051C | 0.60752261 | 0.95959383 | -0.2108676 | 97.27284091 | 218.5173006 |
| YKL215C | YKL215C | 0.51303322 | 0.95675879 | -0.2107945 | 77.27710654 | 181.0019781 |
| YPL199C | YPL199C | 0.59580643 | 0.95959383 | -0.2107281 | 88.66787176 | 202.3544653 |
| YOR061W | CKA2 | 0.2821187 | 0.95675879 | -0.2106212 | 76.13658945 | 178.8332361 |
| YJL077C | ICS3 | 0.29462958 | 0.95675879 | -0.2105742 | 109.6563334 | 241.6928454 |
| YBR115C | LYS2 | 0.33765184 | 0.95675879 | -0.2101024 | 69.94994201 | 167.1411696 |
| YNL227C | JJJ1 | 0.52758996 | 0.95675879 | -0.2100678 | 40.51116984 | 111.921644 |
| YOL055C | THI20 | 0.5928578 | 0.95959383 | -0.2096746 | 94.03973347 | 212.2493548 |
| YPL163C | SVS1 | 0.26103293 | 0.95675879 | -0.2093649 | 99.62459468 | 222.6710001 |
| YER078C | YER078C | 0.18147629 | 0.95675879 | -0.2090177 | 87.35843588 | 199.6059147 |
| YPR003C | YPR003C | 0.56998342 | 0.95959383 | -0.2088489 | 83.18420903 | 191.7481072 |
| YGR089W | NNF2 | 0.40701029 | 0.95675879 | -0.2086676 | 82.95709427 | 191.291129 |
| YGR107W | YGR107W | 0.48455713 | 0.95675879 | -0.208605 | 90.26462928 | 204.9859878 |
| YLR049C | YLR049C | 0.53210004 | 0.95675879 | -0.2084391 | 79.30982435 | 184.4114311 |
| YLR059C | REX2 | 0.53994082 | 0.95675879 | -0.2083453 | 78.75068891 | 183.3466932 |
| YDR004W | RAD57 | 0.47976043 | 0.95675879 | -0.2082845 | 93.10414793 | 210.256793 |
| YPL081W | RPS9A | 0.36651969 | 0.95675879 | -0.2080911 | 92.67384802 | 209.4166568 |
| YBR068C | BAP2 | 0.59271802 | 0.95959383 | -0.2076771 | 94.46023927 | 212.6962697 |
| YOR228C | YOR228C | 0.59449762 | 0.95959383 | -0.2075271 | 89.4455994 | 203.265454 |
| YGR088W | CTT1 | 0.23523957 | 0.95675879 | -0.2071673 | 103.4287964 | 229.4299351 |
| YJL133W | MRS3 | 0.24431379 | 0.95675879 | -0.2067135 | 104.0367235 | 230.4924909 |
| YJL182C | YJL182C | 0.48167421 | 0.95675879 | -0.2066159 | 90.32996537 | 204.7682134 |
| YFL041W | FET5 | 0.12225073 | 0.95675879 | -0.2065917 | 97.69460753 | 218.5767557 |
| YML027W | YOX1 | 0.54527692 | 0.95802746 | -0.2064075 | 69.84951123 | 166.3206231 |

| | | | | | | |
|---|---|---|---|---|---|---|
| YER087C-A | YER087C-A | 0.07170155 | 0.95675879 | -0.2063044 | 112.5833097 | 246.4519609 |
| YDL001W | RMD1 | 0.62998064 | 0.96098984 | -0.206151 | 104.4048529 | 231.0866974 |
| YKL202W | YKL202W | 0.51618767 | 0.95675879 | -0.205917 | 73.88942214 | 173.8137327 |
| YHR022C | YHR022C | 0.43395513 | 0.95675879 | -0.2056815 | 79.95602402 | 185.1515963 |
| YNL286W | CUS2 | 0.36804339 | 0.95675879 | -0.2056052 | 76.20768531 | 178.1083758 |
| YLR281C | YLR281C | 0.56377793 | 0.95959383 | -0.2055153 | 73.96484759 | 173.8864691 |
| YEL028W | YEL028W | 0.59539175 | 0.95959383 | -0.2049115 | 91.56860416 | 206.7997139 |
| YDL133C-A | RPL41B | 0.52127952 | 0.95675879 | -0.2047988 | 74.62842458 | 175.0084326 |
| YNL056W | OCA2 | 0.54361661 | 0.9578538 | -0.2043557 | 66.53969186 | 159.7618785 |
| YML041C | VPS71 | 0.34339127 | 0.95675879 | -0.2043519 | 67.95564465 | 162.4169088 |
| YGR008C | STF2 | 0.61636291 | 0.96098984 | -0.2038181 | 96.95814297 | 216.7209353 |
| YLR216C | CPR6 | 0.42507014 | 0.95675879 | -0.2038046 | 84.49867825 | 193.3503927 |
| YCR102C | YCR102C | 0.6668907 | 0.96992497 | -0.2037913 | 104.0298469 | 229.9796217 |
| YHR180W | YHR180W | 0.52942443 | 0.95675879 | -0.2034273 | 75.39831134 | 176.2177392 |
| YBR262C | FMP51 | 0.60129238 | 0.95959383 | -0.2033625 | 93.83397091 | 210.7834763 |
| YKR030W | GMH1 | 0.53491494 | 0.95675879 | -0.2032135 | 69.12461639 | 164.4145855 |
| YNL175C | NOP13 | 0.60580758 | 0.95959383 | -0.2026441 | 89.04918015 | 201.6864983 |
| YML047C | PRM6 | 0.36299926 | 0.95675879 | -0.2023031 | 75.3314346 | 175.8999565 |
| YLR332W | MID2 | 0.56846154 | 0.95959383 | -0.2022427 | 84.38625337 | 192.8723015 |
| YBL069W | AST1 | 0.47536691 | 0.95675879 | -0.2017467 | 93.107712 | 209.1448939 |
| YER139C | YER139C | 0.51234684 | 0.95675879 | -0.2016023 | 71.17860996 | 167.9912652 |
| YKL199C | YKT9 | 0.56901525 | 0.95959383 | -0.201217 | 84.66886796 | 193.2268748 |
| YOR069W | VPS5 | 0.54829812 | 0.95900765 | -0.201127 | 72.39342791 | 170.188389 |
| YML009C | MRPL39 | 0.57612596 | 0.95959383 | -0.1999272 | 85.5915667 | 194.7367522 |
| YOR300W | YOR300W | 0.47126177 | 0.95675879 | -0.1999123 | 81.66668804 | 187.3729388 |
| YNR020C | YNR020C | 0.58108989 | 0.95959383 | -0.1995815 | 71.6370773 | 168.5054009 |
| YNL046W | YNL046W | 0.20562716 | 0.95675879 | -0.1992339 | 108.9669717 | 238.4596525 |
| YDR293C | SSD1 | 0.67121306 | 0.96992497 | -0.1989197 | 56.94269836 | 140.8322608 |
| YLL046C | RNP1 | 0.39872376 | 0.95675879 | -0.1988758 | 82.09387733 | 187.996812 |
| YIR001C | SGN1 | 0.59419763 | 0.95959383 | -0.198856 | 86.59021146 | 196.4264727 |
| YOR238W | YOR238W | 0.4469068 | 0.95675879 | -0.1986053 | 96.1064178 | 214.2316215 |
| YDR305C | HNT2 | 0.51391522 | 0.95675879 | -0.1983622 | 81.60478852 | 186.9916372 |
| YFR040W | SAP155 | 0.32689751 | 0.95675879 | -0.1981714 | 85.92073544 | 195.0537175 |
| YLR150W | STM1 | 0.50708694 | 0.95675879 | -0.1981398 | 70.92035893 | 166.9144969 |
| YGL195W | GCN1 | 0.63037554 | 0.96098984 | -0.1981258 | 97.05176036 | 215.9226022 |
| YPL115C | BEM3 | 0.77164213 | 0.97903138 | -0.1978199 | 97.00278024 | 215.7784084 |
| YGR225W | AMA1 | 0.58166409 | 0.95959383 | -0.1977065 | 86.38618579 | 195.8471521 |
| YOL086C | ADH1 | 0.39425638 | 0.95675879 | -0.1976641 | 78.71669178 | 181.4554503 |
| YDR103W | STE5 | 0.35924491 | 0.95675879 | -0.1975595 | 102.4039654 | 225.8639917 |
| YDL227C | HO | 0.58039822 | 0.95959383 | -0.1971134 | 87.14806091 | 197.1746091 |
| YPL240C | HSP82 | 0.26568368 | 0.95675879 | -0.1968578 | 95.68718882 | 213.1463563 |
| YER057C | HMF1 | 0.53648467 | 0.95675879 | -0.1960634 | 75.07378327 | 174.3491611 |
| YDR334W | SWR1 | 0.49445833 | 0.95675879 | -0.1960396 | 90.04585399 | 202.4258055 |
| YDR461W | MFA1 | 0.38749531 | 0.95675879 | -0.1953885 | 109.1609445 | 238.1655454 |
| YLL048C | YBT1 | 0.58683491 | 0.95959383 | -0.195335 | 86.39377186 | 195.4556215 |
| YIL058W | YIL058W | 0.5698559 | 0.95959383 | -0.1943794 | 82.40286289 | 187.8070194 |
| YPL189W | GUP2 | 0.38221436 | 0.95675879 | -0.193705 | 117.9313079 | 254.3266667 |
| YNR040W | YNR040W | 0.31997698 | 0.95675879 | -0.1936652 | 105.0031312 | 230.0725491 |
| YPL112C | PEX25 | 0.3168897 | 0.95675879 | -0.1936325 | 101.0682638 | 222.6869399 |
| YPL096W | PNG1 | 0.0493508 | 0.92220427 | -0.1934573 | 93.83301149 | 209.0869611 |
| YJR154W | YJR154W | 0.07774278 | 0.95675879 | -0.1932771 | 89.78952547 | 201.4724127 |
| YER134C | YER134C | 0.2486046 | 0.95675879 | -0.1930064 | 100.2420656 | 221.0302606 |
| YDL074C | BRE1 | 0.36693164 | 0.95675879 | -0.1928505 | 64.87877477 | 154.6782887 |
| YJR069C | HAM1 | 0.51404449 | 0.95675879 | -0.1927391 | 88.06815633 | 198.1518537 |
| YMR158C-B | YMR158C-B | 0.59053763 | 0.95959383 | -0.1926398 | 87.3087788 | 196.7106272 |
| YOR384W | FRE5 | 0.35691785 | 0.95675879 | -0.1925552 | 110.7803637 | 240.7180622 |
| YNL068C | FKH2 | 0.38721248 | 0.95675879 | -0.1922038 | 75.24333795 | 174.0068079 |
| YOR202W | HIS3 | 0.00674145 | 0.47455374 | -0.1921502 | 86.020347 | 194.2103556 |
| YML076C | WAR1 | 0.41110598 | 0.95675879 | -0.1915953 | 81.22594109 | 185.1233056 |
| YLR252W | YLR252W | 0.58414939 | 0.95959383 | -0.190902 | 81.67349655 | 185.8441037 |
| YMR244W | YMR244W | 0.60659269 | 0.95959383 | -0.1908909 | 89.76604594 | 201.0200995 |
| YNL268W | LYP1 | 0.04636347 | 0.92220427 | -0.1907774 | 91.51618418 | 204.2831404 |
| YGR072W | UPF3 | 0.53392912 | 0.95675879 | -0.1904685 | 98.90945643 | 218.0966679 |
| YLR449W | FPR4 | 0.54139521 | 0.95675879 | -0.1904037 | 73.00473194 | 169.5002312 |

| | | | | | | |
|---|---|---|---|---|---|---|
| YDR420W | HKR1 | 0.54089279 | 0.95675879 | -0.1903528 | 93.71392956 | 208.3324622 |
| YLR042C | YLR042C | 0.55415684 | 0.95959383 | -0.1900314 | 75.58910726 | 174.2836335 |
| YPL262W | FUM1 | 0.54691295 | 0.95854866 | -0.1894696 | 72.55728113 | 168.5011894 |
| YPL022W | RAD1 | 0.24714031 | 0.95675879 | -0.1888844 | 113.4543534 | 245.1051834 |
| YDR400W | URH1 | 0.3511173 | 0.95675879 | -0.1886865 | 113.1090601 | 244.4237251 |
| YKL166C | TPK3 | 0.61008178 | 0.95959383 | -0.1885828 | 84.50328937 | 190.7546871 |
| YHL026C | YHL026C | 0.60660173 | 0.95959383 | -0.188474 | 81.36291437 | 184.846183 |
| YNL078W | NIS1 | 0.42279976 | 0.95675879 | -0.1884188 | 81.27505983 | 184.6719596 |
| YDR522C | SPS2 | 0.42528031 | 0.95675879 | -0.1883384 | 80.41594931 | 183.0469128 |
| YDL109C | YDL109C | 0.05766449 | 0.95675879 | -0.1882975 | 89.7814844 | 200.6053426 |
| YDR203W | YDR203W | 0.60469987 | 0.95959383 | -0.188161 | 104.9329382 | 228.9991556 |
| YDL100C | GET3 | 0.53525367 | 0.95675879 | -0.1881511 | 84.39740908 | 190.482248 |
| YOR351C | MEK1 | 0.29401104 | 0.95675879 | -0.1881424 | 97.22619582 | 214.5416694 |
| YOR383C | FIT3 | 0.2534183 | 0.95675879 | -0.1877223 | 103.9828599 | 227.1421935 |
| YDL054C | MCH1 | 0.60275987 | 0.95959383 | -0.1877152 | 88.22973751 | 197.5953482 |
| YKL220C | FRE2 | 0.56780721 | 0.95959383 | -0.187658 | 79.460436 | 181.1383811 |
| YGL041C | YGL041C | 0.2633356 | 0.95675879 | -0.1871417 | 114.0361256 | 245.8981576 |
| YLR154C | RNH203 | 0.53680262 | 0.95675879 | -0.185888 | 70.12808166 | 163.3323488 |
| YNL030W | HHF2 | 0.29253446 | 0.95675879 | -0.1858644 | 70.68708492 | 164.3767318 |
| YDR374C | YDR374C | 0.32783715 | 0.95675879 | -0.1856938 | 99.7582333 | 218.8716598 |
| YAL061W | YAL061W | 0.68300191 | 0.96992497 | -0.1856116 | 109.1202464 | 236.4164455 |
| YOR155C | ISN1 | 0.3053726 | 0.95675879 | -0.1855365 | 93.73498816 | 207.5479187 |
| YKL140W | TGL1 | 0.58178034 | 0.95959383 | -0.1852804 | 81.36449486 | 184.3027302 |
| YLR255C | YLR255C | 0.58824022 | 0.95959383 | -0.1850098 | 78.2607094 | 178.4351559 |
| YOL071W | EMI5 | 0.13653059 | 0.95675879 | -0.1849211 | 79.77338889 | 181.2570845 |
| YIL020C | HIS6 | 0.39544061 | 0.95675879 | -0.184849 | 88.53703733 | 197.6813168 |
| YOR090C | PTC5 | 0.65225408 | 0.96992497 | -0.1847299 | 95.59134039 | 210.8915826 |
| YLR343W | GAS2 | 0.43543794 | 0.95675879 | -0.1847099 | 81.59725699 | 184.6416859 |
| YBR118W | TEF2 | 0.60220502 | 0.95959383 | -0.1845051 | 77.19067319 | 176.3419158 |
| YER091C-A | YER091C-A | 0.18662412 | 0.95675879 | -0.1834145 | 102.2410602 | 223.1383338 |
| YPL119C | DBP1 | 0.22664137 | 0.95675879 | -0.1832307 | 95.93229691 | 211.274551 |
| YLR085C | ARP6 | 0.51331878 | 0.95675879 | -0.1828497 | 64.53994539 | 152.331728 |
| YOR280C | FSH3 | 0.29293714 | 0.95675879 | -0.1828097 | 90.16464453 | 200.3850475 |
| YLR390W-A | CCW14 | 0.38527808 | 0.95675879 | -0.1821366 | 74.77233092 | 171.4009876 |
| YKR064W | OAF3 | 0.56034898 | 0.95959383 | -0.1815772 | 74.70001789 | 171.1696409 |
| YNL095C | YNL095C | 0.40835482 | 0.95675879 | -0.1810372 | 76.24733051 | 173.9793083 |
| YLR393W | ATP10 | 0.56377962 | 0.95959383 | -0.1806604 | 69.98751066 | 162.1742838 |
| YBL052C | SAS3 | 0.62967152 | 0.96098984 | -0.1805916 | 91.13247302 | 201.820755 |
| YJL084C | ALY2 | 0.63065181 | 0.96098984 | -0.1805883 | 88.18100741 | 196.2845838 |
| YOR231W | MKK1 | 0.28893781 | 0.95675879 | -0.1804513 | 114.6129817 | 245.8353906 |
| YML060W | OGG1 | 0.60859165 | 0.95959383 | -0.1804208 | 75.70976029 | 172.8656098 |
| YDR146C | SWI5 | 0.60307251 | 0.95959383 | -0.1796289 | 84.08307567 | 188.434609 |
| YPL149W | ATG5 | 0.34126889 | 0.95675879 | -0.1795741 | 120.2930701 | 256.3385469 |
| YPR126C | YPR126C | 0.2294141 | 0.95675879 | -0.1795238 | 101.486543 | 221.0575287 |
| YLR328W | NMA1 | 0.60886472 | 0.95959383 | -0.1793169 | 79.68146779 | 180.1258256 |
| YGL241W | KAP114 | 0.64591905 | 0.96707685 | -0.178754 | 94.1839007 | 207.2294303 |
| YKL198C | PTK1 | 0.04624922 | 0.92220427 | -0.177457 | 91.96298261 | 202.842086 |
| YIR016W | YIR016W | 0.53152827 | 0.95675879 | -0.177374 | 97.08238896 | 212.4295512 |
| YLL059C | YLL059C | 0.6141294 | 0.96098984 | -0.1768251 | 81.26689611 | 182.6730309 |
| YFL055W | AGP3 | 0.35441207 | 0.95675879 | -0.1766906 | 115.2686222 | 246.4216387 |
| YDR320C | SWA2 | 0.42016895 | 0.95675879 | -0.1766042 | 100.3538268 | 218.4335502 |
| YBR209W | YBR209W | 0.65001811 | 0.96848639 | -0.1763308 | 90.45691648 | 199.824723 |
| YLR448W | RPL6B | 0.55860209 | 0.95959383 | -0.1763111 | 66.50050924 | 154.8901384 |
| YEL015W | EDC3 | 0.62651454 | 0.96098984 | -0.1760149 | 86.89598046 | 193.091988 |
| YLR455W | YLR455W | 0.23808453 | 0.95675879 | -0.1757109 | 80.2310986 | 180.539723 |
| YCL061C | MRC1 | 0.56657483 | 0.95959383 | -0.1756445 | 70.95955966 | 163.139216 |
| YER051W | JHD1 | 0.14482119 | 0.95675879 | -0.1756342 | 112.634966 | 241.3013717 |
| YDR504C | SPG3 | 0.28426561 | 0.95675879 | -0.1754893 | 112.2698366 | 240.5917661 |
| YBR078W | ECM33 | 0.3466513 | 0.95675879 | -0.1753143 | 90.34969457 | 199.4497103 |
| YBR261C | YBR261C | 0.62370882 | 0.96098984 | -0.1749375 | 114.5644672 | 244.8010199 |
| YLR121C | YPS3 | 0.18434382 | 0.95675879 | -0.1747833 | 109.9189458 | 236.0617747 |
| YPL187W | MF(ALPHA)1 | 0.29658332 | 0.95675879 | -0.1746313 | 97.39701326 | 212.5503845 |
| YMR224C | MRE11 | 0.21941623 | 0.95675879 | -0.1740682 | 59.23385951 | 140.8774864 |
| YGL221C | NIF3 | 0.62475163 | 0.96098984 | -0.1739967 | 83.4780307 | 186.3361902 |

| | | | | | | |
|---|---|---|---|---|---|---|
| YPR151C | SUE1 | 0.64381113 | 0.96617034 | -0.1739644 | 84.38953154 | 188.0402191 |
| YOL030W | GAS5 | 0.66509703 | 0.96992497 | -0.1735368 | 97.04127845 | 211.6959274 |
| YDR191W | HST4 | 0.3592765 | 0.95675879 | -0.1734818 | 117.1434697 | 249.3889861 |
| YNL119W | NCS2 | 0.40862067 | 0.95675879 | -0.1734698 | 64.59374252 | 150.8277893 |
| YBL007C | SLA1 | 0.58982758 | 0.95959383 | -0.1733037 | 77.8398443 | 175.6429756 |
| YOL080C | REX4 | 0.65112603 | 0.96912832 | -0.1732092 | 91.45601308 | 201.1644848 |
| YMR183C | SSO2 | 0.59333426 | 0.95959383 | -0.1731292 | 75.81661708 | 171.8184767 |
| YGL261C | PAU11 | 0.40990216 | 0.95675879 | -0.1723617 | 95.40004638 | 208.4166779 |
| YNL291C | MID1 | 0.62637556 | 0.96098984 | -0.1721585 | 76.8484653 | 173.5876618 |
| YPL140C | MKK2 | 0.23921928 | 0.95675879 | -0.171921 | 100.0665994 | 217.0935739 |
| YGR250C | YGR250C | 0.58153868 | 0.95959383 | -0.1714106 | 98.71515339 | 214.4715626 |
| YGL205W | POX1 | 0.65180667 | 0.96980521 | -0.1711435 | 89.7980057 | 197.7013929 |
| YDR485C | VPS72 | 0.39293318 | 0.95675879 | -0.1702511 | 68.52195215 | 157.6446089 |
| YMR304W | UBP15 | 0.6679211 | 0.96992497 | -0.1697937 | 94.10808099 | 205.5541746 |
| YLR015W | BRE2 | 0.59585484 | 0.95959383 | -0.1696279 | 76.70769985 | 172.8906791 |
| YOR251C | YOR251C | 0.45685927 | 0.95675879 | -0.1696084 | 90.77023158 | 199.2621989 |
| YKR065C | PAM17 | 0.60545241 | 0.95959383 | -0.1691054 | 77.94755593 | 175.1266942 |
| YBR274W | CHK1 | 0.08477949 | 0.95675879 | -0.1689522 | 102.3188999 | 220.8099221 |
| YDL200C | MGT1 | 0.33562945 | 0.95675879 | -0.1688443 | 108.4043516 | 232.2049762 |
| YER098W | UBP9 | 0.62923495 | 0.96098984 | -0.1688396 | 84.10862382 | 186.6365409 |
| YIL017C | VID28 | 0.56302718 | 0.95959383 | -0.1688294 | 75.48669828 | 170.4640252 |
| YER010C | YER010C | 0.15800172 | 0.95675879 | -0.1685638 | 116.2724277 | 246.9138703 |
| YPL009C | YPL009C | 0.26273237 | 0.95675879 | -0.1682002 | 95.3738448 | 207.6555262 |
| YDR335W | MSN5 | 0.25661243 | 0.95675879 | -0.1679976 | 101.7508817 | 219.5812533 |
| YDR372C | VPS74 | 0.69835091 | 0.97267176 | -0.1679407 | 105.2937885 | 226.2163885 |
| YER095W | RAD51 | 0.41642406 | 0.95675879 | -0.1676886 | 66.18114451 | 152.8159008 |
| YGR032W | GSC2 | 0.67324808 | 0.96992497 | -0.1675423 | 88.48193925 | 194.6169072 |
| YKL046C | DCW1 | 0.63209063 | 0.96098984 | -0.1674678 | 82.82757023 | 183.9991727 |
| YMR163C | INP2 | 0.40138759 | 0.95675879 | -0.1674008 | 75.34850835 | 169.9604246 |
| YNL143C | YNL143C | 0.60933751 | 0.95959383 | -0.1671351 | 73.75514824 | 166.9265485 |
| YGR053C | YGR053C | 0.62005772 | 0.96098984 | -0.166669 | 108.2817521 | 231.6028516 |
| YML070W | DAK1 | 0.64621044 | 0.96717589 | -0.1664527 | 88.06026423 | 193.6396163 |
| YPL150W | YPL150W | 0.34688323 | 0.95675879 | -0.1664269 | 101.6006982 | 219.0308315 |
| YLR014C | PPR1 | 0.61679923 | 0.96098984 | -0.1663398 | 77.29978116 | 173.4385861 |
| YAL011W | SWC3 | 0.46851863 | 0.95675879 | -0.1658061 | 79.47836909 | 177.4333032 |
| YBR095C | RXT2 | 0.48550613 | 0.95675879 | -0.164959 | 90.23769607 | 197.4679164 |
| YBR073W | RDH54 | 0.57680768 | 0.95959383 | -0.1649453 | 96.40615268 | 209.0347706 |
| YDR284C | DPP1 | 0.35758707 | 0.95675879 | -0.1648696 | 111.773731 | 237.8443249 |
| YNL237W | YTP1 | 0.65556264 | 0.96992497 | -0.1648476 | 90.0617253 | 197.1188205 |
| YIL077C | YIL077C | 0.64925088 | 0.96801503 | -0.1648199 | 83.99167542 | 185.7294571 |
| YJR150C | DAN1 | 0.64352491 | 0.96617034 | -0.1645021 | 79.82048989 | 177.8518491 |
| YDR153C | ENT5 | 0.36838335 | 0.95675879 | -0.1642188 | 106.2729837 | 227.4161003 |
| YPL107W | YPL107W | 0.75151158 | 0.97903138 | -0.1638412 | 95.43869366 | 207.0313464 |
| YJL165C | HAL5 | 0.63816135 | 0.96415344 | -0.1636514 | 78.91548045 | 176.0089177 |
| YPL162C | YPL162C | 0.43524775 | 0.95675879 | -0.1635968 | 114.7076275 | 243.129199 |
| YBR227C | MCX1 | 0.60982891 | 0.95959383 | -0.1632132 | 106.6007903 | 227.8588746 |
| YCR065W | HCM1 | 0.63667745 | 0.96331676 | -0.1632034 | 83.37406828 | 184.2945273 |
| YOR125C | CAT5 | 0.63297897 | 0.96098984 | -0.1630637 | 72.29515893 | 163.4916912 |
| YGR221C | TOS2 | 0.47401902 | 0.95675879 | -0.1628887 | 95.2386149 | 206.4931334 |
| YBL054W | YBL054W | 0.59844474 | 0.95959383 | -0.1627376 | 108.074726 | 230.5419315 |
| YDR003W | RCR2 | 0.37787448 | 0.95675879 | -0.162575 | 120.9290706 | 254.6229575 |
| YOR267C | HRK1 | 0.64809859 | 0.96786115 | -0.1620336 | 76.78541719 | 171.7371115 |
| YMR263W | SAP30 | 0.59071083 | 0.95959383 | -0.1619419 | 72.0184072 | 162.7806921 |
| YNL201C | PSY2 | 0.63314729 | 0.96098984 | -0.1618769 | 82.4879476 | 182.4056261 |
| YCL022C | YCL022C | 0.69956876 | 0.97267176 | -0.1617426 | 102.8857211 | 220.6394933 |
| YDR215C | YDR215C | 0.38610579 | 0.95675879 | -0.1616543 | 114.2098302 | 241.863221 |
| YDL184C | RPL41A | 0.66916273 | 0.96992497 | -0.1613772 | 91.8631909 | 199.9037737 |
| YDR104C | SPO71 | 0.38640376 | 0.95675879 | -0.1612256 | 111.4496014 | 236.612949 |
| YER167W | BCK2 | 0.12626626 | 0.95675879 | -0.1611634 | 102.1950898 | 219.2450899 |
| YPL001W | HAT1 | 0.06638853 | 0.95675879 | -0.160933 | 92.98190383 | 201.9259754 |
| YDR262W | YDR262W | 0.09770839 | 0.95675879 | -0.1608807 | 89.51349364 | 195.4118778 |
| YPL133C | RDS2 | 0.3472189 | 0.95675879 | -0.1606048 | 100.7365261 | 216.4139241 |
| YJL070C | YJL070C | 0.2679149 | 0.95675879 | -0.1605364 | 91.53314123 | 199.1409021 |
| YHR210C | YHR210C | 0.48608523 | 0.95675879 | -0.1602657 | 82.10165964 | 181.4054555 |

| | | | | | | |
|---|---|---|---|---|---|---|
| YHR043C | DOG2 | 0.48224243 | 0.95675879 | -0.1599455 | 103.7148178 | 221.8870212 |
| YLR377C | FBP1 | 0.65365729 | 0.96992497 | -0.159915 | 85.63417562 | 187.9708338 |
| YBL106C | SRO77 | 0.59472414 | 0.95959383 | -0.1598979 | 92.66446956 | 201.1535119 |
| YKR052C | MRS4 | 0.64091434 | 0.96479866 | -0.1592586 | 82.37863542 | 181.7526259 |
| YCR082W | AHC2 | 0.71941939 | 0.97530288 | -0.158688 | 105.8853317 | 225.7427737 |
| YJL193W | YJL193W | 0.6861411 | 0.96992497 | -0.1584725 | 93.28862158 | 202.0802455 |
| YJL210W | PEX2 | 0.6074296 | 0.95959383 | -0.1582388 | 93.43471423 | 202.314264 |
| YML066C | SMA2 | 0.52292251 | 0.95675879 | -0.1581657 | 106.217693 | 226.2767539 |
| YLR445W | YLR445W | 0.63401585 | 0.96098984 | -0.158132 | 77.42047638 | 172.2606422 |
| YOL056W | GPM3 | 0.66907583 | 0.96992497 | -0.1581293 | 89.68137913 | 195.2560031 |
| YHL006C | SHU1 | 0.65920716 | 0.96992497 | -0.1577862 | 86.60976174 | 189.4363614 |
| YJR127C | RSF2 | 0.65543347 | 0.96992497 | -0.1576279 | 86.01951906 | 188.3022449 |
| YCR026C | NPP1 | 0.10818933 | 0.95675879 | -0.157571 | 82.83877468 | 182.3269 |
| YKL185W | ASH1 | 0.64311501 | 0.96617034 | -0.1573765 | 81.8460579 | 180.4317507 |
| YDR513W | GRX2 | 0.28376298 | 0.95675879 | -0.1568244 | 112.7664995 | 238.3298183 |
| YIL005W | EPS1 | 0.7019596 | 0.97267176 | -0.1567461 | 87.65177818 | 191.212742 |
| YPL155C | KIP2 | 0.37708705 | 0.95675879 | -0.1567287 | 109.4246816 | 232.0457397 |
| YGL160W | YGL160W | 0.56001614 | 0.95959383 | -0.1566788 | 96.3124048 | 207.4445949 |
| YOR265W | RBL2 | 0.20753272 | 0.95675879 | -0.1556598 | 97.71315148 | 209.8973984 |
| YJL160C | YJL160C | 0.69200048 | 0.97074487 | -0.1555889 | 95.25101684 | 205.2674328 |
| YMR317W | YMR317W | 0.27474469 | 0.95675879 | -0.1554989 | 94.13943709 | 203.1672301 |
| YNL069C | RPL16B | 0.68402004 | 0.96992497 | -0.1547024 | 89.43676424 | 194.2108959 |
| YBR028C | YBR028C | 0.15042419 | 0.95675879 | -0.1545533 | 95.11963991 | 204.8438549 |
| YPL180W | TCO89 | 0.60822365 | 0.95959383 | -0.154319 | 60.2048725 | 139.3197048 |
| YGR109C | CLB6 | 0.25279039 | 0.95675879 | -0.1530999 | 116.6280562 | 244.9350898 |
| YDL155W | CLB3 | 0.4037063 | 0.95675879 | -0.1530083 | 112.6463789 | 237.4516303 |
| YIL145C | PAN6 | 0.58305492 | 0.95959383 | -0.1525219 | 90.3209116 | 195.4960901 |
| YKL211C | TRP3 | 0.63911453 | 0.96454138 | -0.1522979 | 77.09649731 | 170.6548404 |
| YJL115W | ASF1 | 0.55904156 | 0.95959383 | -0.152228 | 17.72366181 | 59.28671484 |
| YNL241C | ZWF1 | 0.42952868 | 0.95675879 | -0.1520149 | 70.7730073 | 158.7464483 |
| YDR446W | ECM11 | 0.11688833 | 0.95675879 | -0.151894 | 114.6653802 | 241.047694 |
| YOL159C | YOL159C | 0.52101854 | 0.95675879 | -0.1516516 | 88.53502423 | 191.997682 |
| YHR110W | ERP5 | 0.6642093 | 0.96992497 | -0.1515044 | 75.8627218 | 168.2050741 |
| YIL139C | REV7 | 0.46990111 | 0.95675879 | -0.1511169 | 103.8266757 | 220.586308 |
| YLR386W | VAC14 | 0.54404883 | 0.95802746 | -0.1509205 | 78.93870441 | 173.8743099 |
| YNL125C | ESBP6 | 0.13755949 | 0.95675879 | -0.1507937 | 100.8574588 | 214.9621176 |
| YGR282C | BGL2 | 0.36024912 | 0.95675879 | -0.1507581 | 110.987302 | 233.9549689 |
| YDR154C | YDR154C | 0.45418824 | 0.95675879 | -0.1504623 | 109.2865017 | 230.714437 |
| YGL217C | YGL217C | 0.15562222 | 0.95675879 | -0.1504425 | 89.17707355 | 192.9949987 |
| YOL002C | IZH2 | 0.29614005 | 0.95675879 | -0.1501794 | 108.6853956 | 229.5386262 |
| YBR025C | YBR025C | 0.669276 | 0.96992497 | -0.1500061 | 83.51436084 | 182.2996776 |
| YGR260W | TNA1 | 0.64917461 | 0.96801503 | -0.1497367 | 94.83363407 | 203.4833463 |
| YAR044W | OSH1 | 0.711536 | 0.97267176 | -0.1489132 | 97.42419723 | 208.2011633 |
| YOL059W | GPD2 | 0.5984456 | 0.95959383 | -0.148798 | 99.69563578 | 212.441619 |
| YNR048W | YNR048W | 0.34516722 | 0.95675879 | -0.1485581 | 111.3090065 | 234.1819318 |
| YDR014W | RAD61 | 0.59294036 | 0.95959383 | -0.1483312 | 89.17159233 | 192.6234864 |
| YAL008W | FUN14 | 0.4079599 | 0.95675879 | -0.1482643 | 113.8809181 | 238.9553735 |
| YOR286W | FMP31 | 0.37758795 | 0.95675879 | -0.1480503 | 95.48377457 | 204.4141838 |
| YER073W | ALD5 | 0.3713405 | 0.95675879 | -0.1471888 | 109.5749467 | 230.6953502 |
| YKR010C | TOF2 | 0.63143598 | 0.96098984 | -0.1466932 | 73.83644288 | 163.5815376 |
| YBR225W | YBR225W | 0.71021898 | 0.97267176 | -0.1465215 | 93.05512865 | 199.5975822 |
| YGL262W | YGL262W | 0.31724143 | 0.95675879 | -0.1464702 | 113.2921808 | 237.544215 |
| YPL192C | PRM3 | 0.49742627 | 0.95675879 | -0.1464566 | 118.1513358 | 246.6554367 |
| YGL121C | GPG1 | 0.56308268 | 0.95959383 | -0.1463463 | 88.66597356 | 191.3355728 |
| YOR025W | HST3 | 0.72931797 | 0.97683349 | -0.1462329 | 99.46026784 | 211.5612999 |
| YKL115C | YKL115C | 0.65695676 | 0.96992497 | -0.1460973 | 80.49468118 | 175.9673727 |
| YNL259C | ATX1 | 0.57072988 | 0.95959383 | -0.1454047 | 84.17498912 | 182.7514472 |
| YJL071W | ARG2 | 0.62572346 | 0.96098984 | -0.1451071 | 92.33030799 | 197.996161 |
| YPL110C | GDE1 | 0.41096175 | 0.95675879 | -0.1450524 | 108.844167 | 228.9592215 |
| YPL220W | RPL1A | 0.36939102 | 0.95675879 | -0.145022 | 102.5191153 | 217.0911181 |
| YBR291C | CTP1 | 0.58486849 | 0.95959383 | -0.1449709 | 91.10949691 | 195.6831725 |
| YPL232W | SSO1 | 0.34175952 | 0.95675879 | -0.1448455 | 99.29992114 | 211.0231935 |
| YDR193W | YDR193W | 0.4024981 | 0.95675879 | -0.1445482 | 117.8336188 | 245.73303 |
| YER034W | YER034W | 0.65254272 | 0.96992497 | -0.144375 | 82.37437526 | 179.198152 |

| | | | | | | |
|---|---|---|---|---|---|---|
| YHL040C | ARN1 | 0.6203101 | 0.96098984 | -0.14425 | 94.28382412 | 201.5134182 |
| YHR041C | SRB2 | 0.72584343 | 0.97673059 | -0.1441303 | 37.35912496 | 94.72835258 |
| YJL119C | YJL119C | 0.46142407 | 0.95675879 | -0.1441142 | 87.33358747 | 188.4547378 |
| YBR260C | RGD1 | 0.73476316 | 0.97731085 | -0.143484 | 103.1209089 | 217.9566686 |
| YOR208W | PTP2 | 0.45299758 | 0.95675879 | -0.1434213 | 94.70573141 | 202.1629357 |
| YJL095W | BCK1 | 0.62443095 | 0.96098984 | -0.1432037 | 94.81430667 | 202.3293446 |
| YDR452W | PPN1 | 0.39058355 | 0.95675879 | -0.1431422 | 115.533285 | 241.1780989 |
| YMR207C | HFA1 | 0.6451109 | 0.96620376 | -0.1430811 | 75.59820832 | 166.2677922 |
| YJL136C | RPS21B | 0.62503171 | 0.96098984 | -0.1429418 | 94.26379691 | 201.2520332 |
| YOR164C | YOR164C | 0.62454615 | 0.96098984 | -0.1428601 | 98.60515481 | 209.3804461 |
| YOR055W | YOR055W | 0.36893999 | 0.95675879 | -0.1427944 | 111.1766026 | 232.947455 |
| YJL154C | VPS35 | 0.69329291 | 0.97133302 | -0.1427646 | 85.96431662 | 185.6557039 |
| YOR026W | BUB3 | 0.51459089 | 0.95675879 | -0.1422 | 66.93002914 | 149.8595291 |
| YBR285W | YBR285W | 0.54031568 | 0.95675879 | -0.1417974 | 116.8807504 | 243.4752441 |
| YPL109C | YPL109C | 0.36204972 | 0.95675879 | -0.1417446 | 98.64193195 | 209.2585668 |
| YPR028W | YOP1 | 0.26774814 | 0.95675879 | -0.1417264 | 89.15626834 | 191.4647108 |
| YMR187C | YMR187C | 0.67983322 | 0.96992497 | -0.141591 | 82.00976974 | 178.037987 |
| YCR045C | YCR045C | 0.6168262 | 0.96098984 | -0.1411709 | 86.58207153 | 186.541646 |
| YJL188C | BUD19 | 0.42386682 | 0.95675879 | -0.1411561 | 110.9089495 | 232.1651632 |
| YMR176W | ECM5 | 0.10272279 | 0.95675879 | -0.1410225 | 98.07738724 | 208.0761957 |
| YLR434C | YLR434C | 0.67921416 | 0.96992497 | -0.1408228 | 81.14729327 | 176.2889505 |
| YDR001C | NTH1 | 0.39135841 | 0.95675879 | -0.140768 | 106.838259 | 224.4640188 |
| YKL068W | NUP100 | 0.67391579 | 0.96992497 | -0.1407603 | 81.42001941 | 176.7897569 |
| YPL019C | VTC3 | 0.4288702 | 0.95675879 | -0.1403225 | 110.5380001 | 231.3268051 |
| YKL097C | YKL097C | 0.68895551 | 0.96992497 | -0.140168 | 83.42293412 | 180.4449711 |
| YBR218C | PYC2 | 0.34494127 | 0.95675879 | -0.1397655 | 106.6183472 | 223.8800453 |
| YJR080C | FMP26 | 0.4367402 | 0.95675879 | -0.1396567 | 105.6777574 | 222.0973119 |
| YOL012C | HTZ1 | 0.68597981 | 0.96992497 | -0.1391535 | 84.50656837 | 182.3037969 |
| YIR032C | DAL3 | 0.55072336 | 0.95947263 | -0.1391031 | 114.707058 | 238.9374225 |
| YKR028W | SAP190 | 0.37586976 | 0.95675879 | -0.1390485 | 64.69511888 | 145.128657 |
| YBL098W | BNA4 | 0.59960965 | 0.95959383 | -0.1390311 | 90.37617395 | 193.2915446 |
| YDR326C | YSP2 | 0.37131957 | 0.95675879 | -0.1390194 | 92.46032149 | 197.1984449 |
| YHR028C | DAP2 | 0.56630781 | 0.95959383 | -0.1385765 | 79.21684057 | 172.2839705 |
| YOR266W | PNT1 | 0.7302683 | 0.97687603 | -0.1385462 | 98.84532374 | 209.092802 |
| YPL123C | RNY1 | 0.4639188 | 0.95675879 | -0.1383423 | 100.8238508 | 212.7687334 |
| YLR450W | HMG2 | 0.69242613 | 0.97102476 | -0.1380253 | 71.84677658 | 158.3668169 |
| YPL134C | ODC1 | 0.42067088 | 0.95675879 | -0.1374215 | 105.8641452 | 222.0644637 |
| YPR096C | YPR096C | 0.56713985 | 0.95959383 | -0.1372841 | 105.0874302 | 220.5841927 |
| YML117W-A | YML117W-A | 0.54143546 | 0.95675879 | -0.1371358 | 79.01717239 | 171.6629953 |
| YJR087W | YJR087W | 0.77103764 | 0.97903138 | -0.1366887 | 59.22808682 | 134.4712642 |
| YOR307C | SLY41 | 0.5496358 | 0.95947263 | -0.1364745 | 109.6510112 | 229.0048616 |
| YPR045C | MNI2 | 0.75635105 | 0.97903138 | -0.1362534 | 67.29888262 | 149.5338947 |
| YER060W | FCY21 | 0.34103128 | 0.95675879 | -0.1362003 | 106.0758965 | 222.2526742 |
| YDR025W | RPS11A | 0.56934116 | 0.95959383 | -0.1360773 | 79.91434337 | 173.1645655 |
| YIR002C | MPH1 | 0.68985789 | 0.96992497 | -0.1359972 | 84.05507355 | 180.916983 |
| YCR081W | SRB8 | 0.57835447 | 0.95959383 | -0.1359049 | 42.99707308 | 103.8952408 |
| YDR415C | YDR415C | 0.36633666 | 0.95675879 | -0.1356398 | 105.877866 | 221.7853602 |
| YIL054W | YIL054W | 0.47671029 | 0.95675879 | -0.1354685 | 117.9811648 | 244.456287 |
| YPL017C | IRC15 | 0.50828883 | 0.95675879 | -0.1353969 | 87.6857334 | 187.6237123 |
| YLR170C | APS1 | 0.41192419 | 0.95675879 | -0.1351807 | 91.10530782 | 194.0002744 |
| YIL070C | MAM33 | 0.44761487 | 0.95675879 | -0.1351317 | 117.4768679 | 243.4528214 |
| YOR324C | FRT1 | 0.35606826 | 0.95675879 | -0.1348777 | 110.5834439 | 230.4804711 |
| YDL201W | TRM8 | 0.34643031 | 0.95675879 | -0.1348302 | 116.6350834 | 241.8224435 |
| YDR244W | PEX5 | 0.67911212 | 0.96992497 | -0.1342772 | 101.5762116 | 213.4842961 |
| YAL058C-A | YAL058C-A | 0.77015477 | 0.97903138 | -0.1341068 | 106.655935 | 222.9823777 |
| YKL053W | YKL053W | 0.66890651 | 0.96992497 | -0.1340947 | 75.50552295 | 164.5564448 |
| YOR304C-A | YOR304C-A | 0.39815096 | 0.95675879 | -0.1339138 | 109.4826305 | 228.2509355 |
| YOL112W | MSB4 | 0.71133691 | 0.97267176 | -0.1335948 | 87.1181358 | 186.250838 |
| YMR155W | YMR155W | 0.54439252 | 0.95802746 | -0.133539 | 87.05800612 | 186.1285166 |
| YLR391W | YLR391W | 0.33470661 | 0.95675879 | -0.1334818 | 90.46268041 | 192.504327 |
| YOR214C | YOR214C | 0.43895694 | 0.95675879 | -0.133191 | 104.2442164 | 218.3024171 |
| YNR001C | CIT1 | 0.70974743 | 0.97267176 | -0.1331043 | 84.54458658 | 181.3401126 |
| YML042W | CAT2 | 0.59775227 | 0.95959383 | -0.1328536 | 85.56925314 | 183.2190255 |
| YGR261C | APL6 | 0.4824637 | 0.95675879 | -0.1327141 | 105.0795721 | 219.7875648 |

| | | | | | | |
|---|---|---|---|---|---|---|
| YPL051W | ARL3 | 0.31932041 | 0.95675879 | -0.1326481 | 98.88035651 | 208.1493836 |
| YKL222C | YKL222C | 0.71444264 | 0.97378831 | -0.1325606 | 85.66109125 | 183.3411544 |
| YPL038W | MET31 | 0.30367892 | 0.95675879 | -0.1324837 | 102.179592 | 214.309114 |
| YOR318C | YOR318C | 0.33735131 | 0.95675879 | -0.1324523 | 98.03366434 | 206.5278824 |
| YNL091W | NST1 | 0.65385349 | 0.96992497 | -0.1322326 | 27.40715828 | 74.02740732 |
| YMR246W | FAA4 | 0.57366057 | 0.95959383 | -0.1322282 | 79.43564841 | 171.6081973 |
| YLR095C | IOC2 | 0.56650546 | 0.95959383 | -0.1321032 | 75.03589705 | 163.3349105 |
| YDR466W | PKH3 | 0.25170478 | 0.95675879 | -0.1320857 | 111.9603854 | 232.585295 |
| YOR321W | PMT3 | 0.3967637 | 0.95675879 | -0.1317185 | 107.6415721 | 224.4223497 |
| YLR436C | ECM30 | 0.71122341 | 0.97267176 | -0.1314852 | 87.43339472 | 186.4811786 |
| YBR233W | PBP2 | 0.74420302 | 0.97903138 | -0.1313332 | 99.93492576 | 209.9022984 |
| YFR039C | YFR039C | 0.57963783 | 0.95959383 | -0.1313328 | 102.9064934 | 215.4755211 |
| YLR053C | YLR053C | 0.69388359 | 0.97175679 | -0.1312628 | 81.48252426 | 175.2820281 |
| YJL213W | YJL213W | 0.62453377 | 0.96098984 | -0.1312585 | 84.03372312 | 180.0661704 |
| YMR237W | BCH1 | 0.19851649 | 0.95675879 | -0.131003 | 103.578684 | 216.6798175 |
| YAL004W | YAL004W | 0.50348051 | 0.95675879 | -0.130859 | 98.98593792 | 208.0412976 |
| YDR162C | NBP2 | 0.67322739 | 0.96992497 | -0.1305761 | 95.38732227 | 201.2435525 |
| YMR124W | YMR124W | 0.73094801 | 0.97731085 | -0.130493 | 88.03339167 | 187.436735 |
| YDR488C | PAC11 | 0.3562756 | 0.95675879 | -0.1303437 | 99.74383027 | 209.3745994 |
| YPL106C | SSE1 | 0.73432151 | 0.97731085 | -0.1301387 | 60.07527047 | 134.9395278 |
| YFL015C | YFL015C | 0.42866106 | 0.95675879 | -0.1300148 | 115.0474726 | 238.0209348 |
| YJR088C | YJR088C | 0.77878105 | 0.97903138 | -0.1297083 | 113.456489 | 234.9845235 |
| YOR215C | YOR215C | 0.55540531 | 0.95959383 | -0.1295703 | 84.42450535 | 180.5102529 |
| YOR230W | WTM1 | 0.44724946 | 0.95675879 | -0.1293474 | 109.7121955 | 227.900205 |
| YMR172W | HOT1 | 0.39189783 | 0.95675879 | -0.1291891 | 103.3127987 | 215.8707932 |
| YIL146C | ECM37 | 0.73389245 | 0.97731085 | -0.1289325 | 93.33601252 | 197.1150285 |
| YDL241W | YDL241W | 0.76205482 | 0.97903138 | -0.1288559 | 103.3839366 | 215.9472042 |
| YGL179C | TOS3 | 0.20894321 | 0.95675879 | -0.1288211 | 95.03783919 | 200.2878052 |
| YPL064C | CWC27 | 0.45266987 | 0.95675879 | -0.1286354 | 95.25012673 | 200.6541971 |
| YIL064W | YIL064W | 0.73450478 | 0.97731085 | -0.1282912 | 88.25411502 | 187.474003 |
| YGL063W | PUS2 | 0.49546784 | 0.95675879 | -0.1272298 | 95.10117108 | 200.1343391 |
| YDR439W | LRS4 | 0.40643824 | 0.95675879 | -0.127226 | 103.9487798 | 216.7277302 |
| YMR095C | SNO1 | 0.72670333 | 0.97673059 | -0.1271561 | 89.86113548 | 190.2938225 |
| YDR453C | TSA2 | 0.40514922 | 0.95675879 | -0.1269156 | 118.2983253 | 243.5877819 |
| YDL176W | YDL176W | 0.5333648 | 0.95675879 | -0.1268155 | 96.01300529 | 201.7736244 |
| YBR151W | APD1 | 0.75335317 | 0.97903138 | -0.126792 | 98.853364 | 207.0968252 |
| YMR198W | CIK1 | 0.71264199 | 0.97267176 | -0.1266438 | 73.07443701 | 158.7220406 |
| YML006C | GIS4 | 0.55956194 | 0.95959383 | -0.1266295 | 76.43238181 | 165.0175495 |
| YDR436W | PPZ2 | 0.41550798 | 0.95675879 | -0.1264912 | 111.2098093 | 230.2203589 |
| YBR022W | POA1 | 0.74515508 | 0.97903138 | -0.1263413 | 96.10223625 | 201.8598619 |
| YGL212W | VAM7 | 0.5938678 | 0.95959383 | -0.1262108 | 26.40242123 | 71.11268845 |
| YOR386W | PHR1 | 0.52362068 | 0.95675879 | -0.1259424 | 100.6708333 | 210.3601928 |
| YOR094W | ARF3 | 0.64914619 | 0.96801503 | -0.125927 | 104.6533717 | 217.8269704 |
| YBR013C | YBR013C | 0.45402133 | 0.95675879 | -0.1259137 | 115.161173 | 237.5325003 |
| YER119C | AVT6 | 0.31694992 | 0.95675879 | -0.1258651 | 111.3046358 | 230.2910907 |
| YBR072W | HSP26 | 0.25955316 | 0.95675879 | -0.1256035 | 116.5545693 | 240.0928044 |
| YER007W | PAC2 | 0.2276868 | 0.95675879 | -0.1255664 | 110.5800518 | 228.881002 |
| YJL023C | PET130 | 0.12973678 | 0.95675879 | -0.1255012 | 99.74048988 | 208.5398118 |
| YEL057C | YEL057C | 0.73626644 | 0.97781627 | -0.1251668 | 31.57112042 | 80.62817599 |
| YPL244C | HUT1 | 0.5718159 | 0.95959383 | -0.125129 | 110.1085345 | 227.9218173 |
| YLR032W | RAD5 | 0.5499065 | 0.95947263 | -0.1250949 | 78.47107011 | 168.5786359 |
| YJL204C | RCY1 | 0.70670318 | 0.97267176 | -0.1249911 | 43.9185054 | 103.7561369 |
| YIL086C | YIL086C | 0.66121066 | 0.96992497 | -0.12494 | 85.93804713 | 182.5567464 |
| YIL032C | YIL032C | 0.54673449 | 0.95854866 | -0.1246956 | 116.0631836 | 239.0158472 |
| YML007W | YAP1 | 0.70815843 | 0.97267176 | -0.1246054 | 81.18011925 | 173.5758176 |
| YOL057W | YOL057W | 0.53853393 | 0.95675879 | -0.124487 | 110.8916873 | 229.2808071 |
| YDR422C | SIP1 | 0.45188492 | 0.95675879 | -0.1236706 | 114.4512302 | 235.8171992 |
| YNL013C | YNL013C | 0.72373027 | 0.97653633 | -0.1232086 | 82.43256424 | 175.6858484 |
| YDL040C | NAT1 | 0.1661082 | 0.95675879 | -0.1228583 | 97.78781088 | 204.425305 |
| YOR338W | YOR338W | 0.45813619 | 0.95675879 | -0.1225765 | 120.0698425 | 246.1679455 |
| YIL023C | YKE4 | 0.69912066 | 0.97267176 | -0.1225589 | 74.9465097 | 161.5342835 |
| YKR036C | CAF4 | 0.71147584 | 0.97267176 | -0.1224318 | 80.49301401 | 171.9152297 |
| YBR297W | MAL33 | 0.74643981 | 0.97903138 | -0.1221499 | 90.13950157 | 189.9593784 |
| YDL046W | NPC2 | 0.59335159 | 0.95959383 | -0.1221338 | 121.4447303 | 248.6708519 |

| | | | | | | |
|---|---|---|---|---|---|---|
| YNL111C | CYB5 | 0.78302964 | 0.97903138 | -0.1218042 | 89.58024233 | 188.8513255 |
| YFL010C | WWM1 | 0.48218681 | 0.95675879 | -0.1216003 | 115.4122476 | 237.2654152 |
| YER170W | ADK2 | 0.56467895 | 0.95959383 | -0.1215887 | 120.0663618 | 245.9923986 |
| YER179W | DMC1 | 0.27809472 | 0.95675879 | -0.1213556 | 79.66320466 | 170.1747535 |
| YLR262C-A | TMA7 | 0.74859219 | 0.97903138 | -0.1212227 | 91.76335516 | 192.8463449 |
| YLR012C | YLR012C | 0.51315798 | 0.95675879 | -0.1210626 | 91.76493286 | 192.8219135 |
| YDR148C | KGD2 | 0.7298246 | 0.97683349 | -0.1210131 | 86.83702239 | 183.570947 |
| YKR097W | PCK1 | 0.68825279 | 0.96992497 | -0.1208236 | 72.55577224 | 156.753461 |
| YIL167W | SDL1 | 0.49975272 | 0.95675879 | -0.1204723 | 96.58389074 | 201.759061 |
| YIR028W | DAL4 | 0.38637724 | 0.95675879 | -0.120108 | 121.8283366 | 249.0437204 |
| YMR161W | HLJ1 | 0.65364229 | 0.96992497 | -0.1197161 | 96.91398855 | 202.248791 |
| YHR015W | MIP6 | 0.63764995 | 0.9638299 | -0.1196104 | 84.69157436 | 179.3070835 |
| YPR012W | YPR012W | 0.4779818 | 0.95675879 | -0.1195293 | 96.30016616 | 201.0655938 |
| YDR383C | NKP1 | 0.46645802 | 0.95675879 | -0.1195208 | 110.1814764 | 227.0990883 |
| YNR064C | YNR064C | 0.62909702 | 0.96098984 | -0.1193911 | 100.4000912 | 208.7315174 |
| YPL236C | YPL236C | 0.5593398 | 0.95959383 | -0.1192631 | 120.0394203 | 245.5439804 |
| YOR128C | ADE2 | 0.59358196 | 0.95959383 | -0.1192014 | 85.63800379 | 181.0121631 |
| YOR234C | RPL33B | 0.7393152 | 0.97903138 | -0.1191608 | 87.03474844 | 183.624877 |
| YFL053W | DAK2 | 0.42063951 | 0.95675879 | -0.1190946 | 107.9646083 | 222.868356 |
| YPL246C | RBD2 | 0.5085443 | 0.95675879 | -0.118471 | 105.4107225 | 217.9717355 |
| YDR111C | ALT2 | 0.53069796 | 0.95675879 | -0.1184032 | 118.8984359 | 243.2568928 |
| YHL034C | SBP1 | 0.77124056 | 0.97903138 | -0.1182733 | 93.83511754 | 196.2273918 |
| YEL068C | YEL068C | 0.35483653 | 0.95675879 | -0.1181662 | 107.8860158 | 222.5621096 |
| YMR058W | FET3 | 0.76245174 | 0.97903138 | -0.117814 | 88.00987844 | 185.2233449 |
| YOR385W | YOR385W | 0.61569496 | 0.96098984 | -0.1177245 | 114.9255488 | 235.6894514 |
| YGL036W | YGL036W | 0.48400659 | 0.95675879 | -0.1177172 | 113.8231548 | 233.6206305 |
| YAR002W | NUP60 | 0.76012031 | 0.97903138 | -0.1174858 | 93.47486159 | 195.4169893 |
| YNL066W | SUN4 | 0.72757871 | 0.97683349 | -0.1174516 | 82.10728425 | 174.0907819 |
| YDL206W | YDL206W | 0.45337369 | 0.95675879 | -0.117417 | 108.5008911 | 223.5871345 |
| YEL010W | YEL010W | 0.43642249 | 0.95675879 | -0.1170158 | 118.7207164 | 242.6861889 |
| YGR071C | YGR071C | 0.70462266 | 0.97267176 | -0.1168853 | 91.93882582 | 192.4333453 |
| YML019W | OST6 | 0.74100135 | 0.97903138 | -0.1168142 | 85.05927973 | 179.518315 |
| YNL231C | PDR16 | 0.64406469 | 0.96617034 | -0.1167126 | 77.74008391 | 165.7734755 |
| YOR120W | GCY1 | 0.51873709 | 0.95675879 | -0.116499 | 108.516181 | 223.4587606 |
| YDR256C | CTA1 | 0.51735195 | 0.95675879 | -0.1162524 | 106.9202726 | 220.4233705 |
| YHR061C | GIC1 | 0.75459108 | 0.97903138 | -0.1159144 | 85.91211844 | 180.9638923 |
| YBR221C | PDB1 | 0.52211566 | 0.95675879 | -0.1157737 | 107.0133308 | 220.5160051 |
| YFL046W | FMP32 | 0.42891203 | 0.95675879 | -0.1156007 | 106.6417284 | 219.7894436 |
| YCR008W | SAT4 | 0.77040188 | 0.97903138 | -0.1154667 | 92.63738859 | 193.5008077 |
| YDL216C | RRI1 | 0.56425957 | 0.95959383 | -0.1152111 | 111.8121959 | 229.420206 |
| YBL075C | SSA3 | 0.43393385 | 0.95675879 | -0.115117 | 110.8211094 | 227.5452984 |
| YDR440W | DOT1 | 0.76126024 | 0.97903138 | -0.1148263 | 90.19247597 | 188.8057161 |
| YER031C | YPT31 | 0.36500503 | 0.95675879 | -0.1147619 | 103.5095181 | 213.7713476 |
| YBL027W | RPL19B | 0.72981752 | 0.97683349 | -0.1142769 | 77.90305056 | 165.6623976 |
| YLR361C | DCR2 | 0.75256761 | 0.97903138 | -0.1142129 | 83.04919332 | 175.3032495 |
| YDR475C | JIP4 | 0.25139718 | 0.95675879 | -0.1139337 | 99.95887629 | 206.9702802 |
| YBR050C | REG2 | 0.42977381 | 0.95675879 | -0.1138926 | 104.6245089 | 215.7138194 |
| YJR129C | YJR129C | 0.74721353 | 0.97903138 | -0.1138196 | 79.93462626 | 169.394467 |
| YDR127W | ARO1 | 0.72262898 | 0.97591869 | -0.1137067 | 67.09800782 | 145.2995442 |
| YJR001W | AVT1 | 0.63441526 | 0.96124881 | -0.1136218 | 115.2410209 | 235.5791919 |
| YML034W | SRC1 | 0.7429092 | 0.97903138 | -0.1136135 | 84.97295807 | 178.8088018 |
| YER166W | DNF1 | 0.46239697 | 0.95675879 | -0.1135891 | 107.7877892 | 221.594768 |
| YNL226W | YNL226W | 0.78513927 | 0.97903138 | -0.1135689 | 51.82464188 | 116.6301514 |
| YDR221W | GTB1 | 0.51865647 | 0.95675879 | -0.1133508 | 105.9392387 | 218.0869615 |
| YCL032W | STE50 | 0.77353388 | 0.97903138 | -0.1133323 | 115.7677359 | 236.5175319 |
| YKR023W | YKR023W | 0.73707595 | 0.97806061 | -0.113285 | 82.22157888 | 173.5922701 |
| YPR077C | YPR077C | 0.43009275 | 0.95675879 | -0.1128111 | 98.32304807 | 203.7101442 |
| YMR139W | RIM11 | 0.71928969 | 0.97530288 | -0.112343 | 73.69171388 | 157.4329909 |
| YJL021C | YJL021C | 0.52812063 | 0.95675879 | -0.1119543 | 116.8077024 | 238.2322755 |
| YLR233C | EST1 | 0.28555185 | 0.95675879 | -0.1117419 | 89.41490183 | 186.8196278 |
| YNL180C | RHO5 | 0.74502356 | 0.97903138 | -0.1116517 | 60.49022293 | 132.554777 |
| YNL320W | YNL320W | 0.61938616 | 0.96098984 | -0.1115026 | 86.2449471 | 180.8332994 |
| YOR347C | PYK2 | 0.58427875 | 0.95959383 | -0.1114501 | 116.9325974 | 238.3802504 |
| YJL178C | ATG27 | 0.61138584 | 0.9598703 | -0.1114084 | 76.99579068 | 163.4700191 |

| | | | | | | |
|---|---|---|---|---|---|---|
| YJL007C | YJL007C | 0.68706435 | 0.96992497 | -0.1112706 | 90.78082947 | 189.30083 |
| YMR015C | ERG5 | 0.62998158 | 0.96098984 | -0.1112345 | 84.89897196 | 178.2629916 |
| YDL036C | PUS9 | 0.53313668 | 0.95675879 | -0.1110157 | 110.5424702 | 226.3209833 |
| YPL186C | UIP4 | 0.50590825 | 0.95675879 | -0.110967 | 106.442609 | 218.6231922 |
| YER131W | RPS26B | 0.56714222 | 0.95959383 | -0.110653 | 69.13743704 | 148.6021099 |
| YMR285C | NGL2 | 0.45824585 | 0.95675879 | -0.1106085 | 109.0494622 | 223.4511255 |
| YGR022C | YGR022C | 0.69407966 | 0.97175679 | -0.1104054 | 88.29693762 | 184.4941702 |
| YDL229W | SSB1 | 0.55085095 | 0.95947263 | -0.109588 | 98.51439116 | 203.5175671 |
| YDR147W | EKI1 | 0.59227885 | 0.95959383 | -0.1093786 | 120.9992919 | 245.6530749 |
| YMR186W | HSC82 | 0.29488016 | 0.95675879 | -0.1093527 | 83.67611004 | 175.6475059 |
| YMR261C | TPS3 | 0.75626811 | 0.97903138 | -0.1093187 | 85.59039753 | 179.2320071 |
| YPR173C | VPS4 | 0.70563967 | 0.97267176 | -0.1092722 | 69.98448988 | 149.9545427 |
| YPL177C | CUP9 | 0.53090664 | 0.95675879 | -0.1091851 | 116.4094104 | 237.011463 |
| YBR165W | UBS1 | 0.56670788 | 0.95959383 | -0.1090556 | 97.08104987 | 200.7381885 |
| YJL207C | LAA1 | 0.50168967 | 0.95675879 | -0.1086748 | 99.64070561 | 205.4737615 |
| YNL224C | SQS1 | 0.7597605 | 0.97903138 | -0.1082952 | 79.78542434 | 168.169442 |
| YGL082W | YGL082W | 0.54458542 | 0.95802746 | -0.1082505 | 114.5324041 | 233.331159 |
| YPL198W | RPL7B | 0.57960214 | 0.95959383 | -0.1077279 | 97.3654877 | 201.0445024 |
| YDR066C | YDR066C | 0.44985145 | 0.95675879 | -0.1077171 | 108.7162659 | 222.3314975 |
| YKL055C | OAR1 | 0.75163455 | 0.97903138 | -0.1075609 | 80.56942036 | 169.5142151 |
| YBL013W | FMT1 | 0.79676876 | 0.9824616 | -0.1069217 | 102.8341851 | 211.1633242 |
| YOR105W | YOR105W | 0.60217141 | 0.95959383 | -0.1065353 | 111.5274542 | 227.4017967 |
| YHR167W | THP2 | 0.52147037 | 0.95675879 | -0.1063107 | 102.4150937 | 210.272766 |
| YIL007C | NAS2 | 0.65501163 | 0.96992497 | -0.1062577 | 76.3263864 | 161.3332687 |
| YDL110C | TMA17 | 0.73162422 | 0.97731085 | -0.1062488 | 101.4112852 | 208.3794813 |
| YML123C | PHO84 | 0.78441987 | 0.97903138 | -0.1059053 | 88.96845332 | 184.9836757 |
| YNL206C | RTT106 | 0.60043221 | 0.95959383 | -0.1058838 | 78.75874764 | 165.8312888 |
| YPR184W | GDB1 | 0.56102353 | 0.95959383 | -0.1057063 | 99.25520556 | 204.2428613 |
| YOR195W | SLK19 | 0.60387938 | 0.95959383 | -0.1054838 | 114.0170502 | 231.891233 |
| YGL031C | RPL24A | 0.63634384 | 0.96315137 | -0.1051558 | 72.96746688 | 154.8449513 |
| YJL215C | YJL215C | 0.72624028 | 0.97673059 | -0.1050191 | 86.33604787 | 179.8948802 |
| YLL019C | KNS1 | 0.22525259 | 0.95675879 | -0.1048333 | 91.27055087 | 189.1179535 |
| YJR061W | YJR061W | 0.57293399 | 0.95959383 | -0.1047917 | 114.1971061 | 232.1105135 |
| YKR019C | IRS4 | 0.75081478 | 0.97903138 | -0.1047757 | 79.37773338 | 166.8026379 |
| YDR438W | THI74 | 0.28250715 | 0.95675879 | -0.1044867 | 110.449846 | 225.030185 |
| YGR292W | MAL12 | 0.54873819 | 0.95900765 | -0.1042561 | 105.8952628 | 216.4484268 |
| YJL012C | VTC4 | 0.49290252 | 0.95675879 | -0.1041873 | 114.6545598 | 232.8650708 |
| YJL187C | SWE1 | 0.57396772 | 0.95959383 | -0.1040927 | 110.236189 | 224.5620584 |
| YNL031C | HHT2 | 0.77707029 | 0.97903138 | -0.1036023 | 88.04729666 | 182.8619852 |
| YKR080W | MTD1 | 0.47033939 | 0.95675879 | -0.1032858 | 101.5025684 | 208.0437517 |
| YDR306C | YDR306C | 0.60883382 | 0.95959383 | -0.1032251 | 119.4488797 | 241.6924008 |
| YIL140W | AXL2 | 0.65062807 | 0.96905894 | -0.1030708 | 119.7758917 | 242.2793123 |
| YGR270W | YTA7 | 0.61497572 | 0.96098984 | -0.10287 | 70.10589855 | 149.0868788 |
| YJL146W | IDS2 | 0.59754966 | 0.95959383 | -0.1028384 | 115.7760419 | 234.7376794 |
| YIR020W-B | YIR020W-B | 0.58319681 | 0.95959383 | -0.1026782 | 117.1566619 | 237.2996708 |
| YPL023C | MET12 | 0.58940246 | 0.95959383 | -0.1019384 | 108.1934583 | 220.3622551 |
| YER042W | MXR1 | 0.15851788 | 0.95675879 | -0.1016184 | 101.7569381 | 208.2355369 |
| YPR062W | FCY1 | 0.8065891 | 0.9824616 | -0.101411 | 87.75641778 | 181.9415183 |
| YPR030W | CSR2 | 0.62621452 | 0.96098984 | -0.1013773 | 98.58809088 | 202.2509984 |
| YBL086C | YBL086C | 0.81322682 | 0.9824616 | -0.1011649 | 106.9171665 | 217.8361679 |
| YPL121C | MEI5 | 0.45243235 | 0.95675879 | -0.1010722 | 103.8679883 | 212.1014507 |
| YDR279W | RNH202 | 0.59005453 | 0.95959383 | -0.1007252 | 109.9170485 | 223.3873358 |
| YCR010C | ADY2 | 0.80163087 | 0.9824616 | -0.1005116 | 99.32414284 | 203.4833653 |
| YMR247C | RKR1 | 0.78611633 | 0.97903138 | -0.1004942 | 91.67932954 | 189.1422268 |
| YML057W | CMP2 | 0.67772335 | 0.96992497 | -0.1004508 | 88.90432253 | 183.9301693 |
| YOR337W | TEA1 | 0.32102617 | 0.95675879 | -0.1004287 | 97.5677016 | 200.1749132 |
| YPL222W | FMP40 | 0.57735456 | 0.95959383 | -0.100236 | 104.6102662 | 213.3505471 |
| YER108C | YER108C | 0.57807673 | 0.95959383 | -0.1000593 | 109.6209484 | 222.718054 |
| YOL084W | PHM7 | 0.602452 | 0.95959383 | -0.0997309 | 113.2203868 | 229.412764 |
| YOR357C | SNX3 | 0.54996673 | 0.95947263 | -0.0996474 | 111.9072822 | 226.9356982 |
| YJL168C | SET2 | 0.79207689 | 0.98180632 | -0.0995163 | 85.5798415 | 177.5350769 |
| YML062C | MFT1 | 0.75770643 | 0.97903138 | -0.0995062 | 79.89872718 | 166.8782038 |
| YOL083W | YOL083W | 0.61249293 | 0.9598703 | -0.0994344 | 108.3070012 | 220.1467887 |
| YJL164C | TPK1 | 0.71185061 | 0.97267176 | -0.0992766 | 88.26932596 | 182.5383181 |

| | | | | | | |
|---|---|---|---|---|---|---|
| YFR009W | GCN20 | 0.49344455 | 0.95675879 | -0.0990291 | 104.3909002 | 212.7326397 |
| YHR039C | MSC7 | 0.59109997 | 0.95959383 | -0.0990006 | 115.6052996 | 233.7608179 |
| YJL139C | YUR1 | 0.80337608 | 0.9824616 | -0.0987338 | 94.90893601 | 194.8983015 |
| YHR126C | YHR126C | 0.71933196 | 0.97530288 | -0.0984529 | 92.86219604 | 191.0114995 |
| YJL044C | GYP6 | 0.49254157 | 0.95675879 | -0.0982538 | 115.7337635 | 233.8739762 |
| YLR254C | NDL1 | 0.56582452 | 0.95959383 | -0.0978047 | 100.235639 | 204.7297784 |
| YMR042W | ARG80 | 0.55566987 | 0.95959383 | -0.097727 | 110.4347667 | 223.8453641 |
| YLR079W | SIC1 | 0.72981939 | 0.97683349 | -0.0975745 | 62.84325249 | 134.5594655 |
| YLR432W | IMD3 | 0.67272065 | 0.96992497 | -0.0974613 | 80.6673041 | 167.9698171 |
| YPL259C | APM1 | 0.78866516 | 0.98007539 | -0.0970476 | 115.8570062 | 233.8987491 |
| YAL018C | YAL018C | 0.55186788 | 0.95959383 | -0.0969997 | 117.7137576 | 237.3729705 |
| YDL242W | YDL242W | 0.57117518 | 0.95959383 | -0.0966977 | 116.536029 | 235.1124188 |
| YFR041C | ERJ5 | 0.42389129 | 0.95675879 | -0.0966797 | 117.8955657 | 237.6592105 |
| YPL202C | AFT2 | 0.66157322 | 0.96992497 | -0.0962879 | 108.3196419 | 219.6321509 |
| YPL034W | YPL034W | 0.60501278 | 0.95959383 | -0.0962753 | 111.2679174 | 225.1595971 |
| YJL172W | CPS1 | 0.77730072 | 0.97903138 | -0.0961961 | 83.11165755 | 172.3378458 |
| YGR010W | NMA2 | 0.39439891 | 0.95675879 | -0.0960954 | 98.94211878 | 202.011287 |
| YJL101C | GSH1 | 0.82911785 | 0.9824616 | -0.0959971 | 33.63153129 | 79.50180914 |
| YKL075C | YKL075C | 0.77392515 | 0.97903138 | -0.0957719 | 81.49660703 | 169.2361772 |
| YNR013C | PHO91 | 0.67125701 | 0.96992497 | -0.0955725 | 87.29140029 | 180.0704219 |
| YEL004W | YEA4 | 0.51674485 | 0.95675879 | -0.095131 | 115.9380991 | 233.7229399 |
| YLR134W | PDC5 | 0.6204038 | 0.96098984 | -0.0950938 | 89.8769212 | 184.8377663 |
| YKL081W | TEF4 | 0.78272767 | 0.97903138 | -0.0948364 | 74.06223254 | 155.1326342 |
| YJR091C | JSN1 | 0.51174222 | 0.95675879 | -0.0943592 | 89.41866633 | 183.8526129 |
| YOR021C | YOR021C | 0.86681216 | 0.98696321 | -0.0943253 | 111.9086935 | 226.0277651 |
| YIL132C | CSM2 | 0.70623595 | 0.97267176 | -0.0940939 | 79.71485812 | 165.6073365 |
| YER069W | ARG5 | 0.43276719 | 0.95675879 | -0.0939703 | 103.2653123 | 209.7560093 |
| YBR111C | YSA1 | 0.52507114 | 0.95675879 | -0.0936745 | 111.8648798 | 225.8342499 |
| YOR291W | YOR291W | 0.55732235 | 0.95959383 | -0.0936537 | 105.3743869 | 213.6575122 |
| YPL223C | GRE1 | 0.5363825 | 0.95675879 | -0.0930313 | 105.1193477 | 213.0726872 |
| YEL006W | YEA6 | 0.62376251 | 0.96098984 | -0.0929437 | 120.2264274 | 241.3916249 |
| YOR012W | YOR012W | 0.6691246 | 0.96992497 | -0.0927827 | 113.9292757 | 229.5535292 |
| YIR003W | YIR003W | 0.5708958 | 0.95959383 | -0.0927269 | 113.6277532 | 228.9784499 |
| YGR230W | BNS1 | 0.62049249 | 0.96098984 | -0.0923551 | 117.7292807 | 236.6074198 |
| YIL137C | TMA108 | 0.76109594 | 0.97903138 | -0.0920812 | 89.65107627 | 183.8987603 |
| YOR222W | ODC2 | 0.60540658 | 0.95959383 | -0.0919679 | 105.7307225 | 214.0373994 |
| YGR149W | YGR149W | 0.53833053 | 0.95675879 | -0.0919029 | 117.0521573 | 235.2600887 |
| YJR079W | YJR079W | 0.57879328 | 0.95959383 | -0.091887 | 111.3707515 | 224.6016491 |
| YPL067C | YPL067C | 0.44047887 | 0.95675879 | -0.0917978 | 103.3764916 | 209.5928438 |
| YOR072W | YOR072W | 0.66350943 | 0.96992497 | -0.0917658 | 117.5990285 | 236.2623038 |
| YER101C | AST2 | 0.56652931 | 0.95959383 | -0.0917131 | 100.5706827 | 204.3159314 |
| YNR039C | ZRG17 | 0.61748332 | 0.96098984 | -0.0915819 | 113.008721 | 227.6215396 |
| YCR098C | GIT1 | 0.57239589 | 0.95959383 | -0.0911793 | 104.9317022 | 212.4038732 |
| YHL041W | YHL041W | 0.68526668 | 0.96992497 | -0.0911019 | 97.1822906 | 197.8562991 |
| YGL252C | RTG2 | 0.79049029 | 0.98074698 | -0.0909195 | 83.53673366 | 172.2322892 |
| YJL141C | YAK1 | 0.81903905 | 0.9824616 | -0.0907859 | 91.60613862 | 187.3439271 |
| YCL026C-A | FRM2 | 0.57094554 | 0.95959383 | -0.0907043 | 108.4748879 | 218.9679996 |
| YLR453C | RIF2 | 0.53716454 | 0.95675879 | -0.0904921 | 120.0059127 | 240.5585992 |
| YDR444W | YDR444W | 0.61708777 | 0.96098984 | -0.0902987 | 81.90158531 | 169.0592909 |
| YOL067C | RTG1 | 0.77808907 | 0.97903138 | -0.0902638 | 75.58148715 | 157.1997204 |
| YDR539W | YDR539W | 0.48007041 | 0.95675879 | -0.0893679 | 111.2760973 | 223.9931355 |
| YDR474C | YDR474C | 0.45512473 | 0.95675879 | -0.0893107 | 108.410301 | 218.6084256 |
| YER033C | ZRG8 | 0.57462808 | 0.95959383 | -0.0891183 | 115.6181628 | 232.0941468 |
| YER174C | GRX4 | 0.53774461 | 0.95675879 | -0.0888566 | 119.9519423 | 240.1775505 |
| YLR404W | YLR404W | 0.69020917 | 0.96992497 | -0.0887322 | 82.52376229 | 169.9581849 |
| YGR259C | YGR259C | 0.76012462 | 0.97903138 | -0.0886116 | 93.16865161 | 189.9024785 |
| YPR149W | NCE102 | 0.45142729 | 0.95675879 | -0.0884886 | 89.42519945 | 182.860437 |
| YAR035W | YAT1 | 0.61995952 | 0.96098984 | -0.088378 | 114.2734271 | 229.4453791 |
| YBR161W | CSH1 | 0.62058756 | 0.96098984 | -0.0883257 | 117.6579872 | 235.7842988 |
| YDR099W | BMH2 | 0.55460674 | 0.95959383 | -0.0882782 | 112.7840628 | 226.6349446 |
| YBL039C | URA7 | 0.80941986 | 0.9824616 | -0.0877977 | 88.60790993 | 181.2093779 |
| YDR332W | IRC3 | 0.60991893 | 0.95959383 | -0.0877623 | 111.432766 | 224.0122629 |
| YJR054W | YJR054W | 0.67058394 | 0.96992497 | -0.087686 | 119.4916872 | 239.1140357 |
| YJL042W | MHP1 | 0.80873935 | 0.9824616 | -0.0875044 | 81.02368569 | 166.9346669 |

| | | | | | | |
|---|---|---|---|---|---|---|
| YMR159C | ATG16 | 0.83255791 | 0.9824616 | -0.0872159 | 100.480172 | 203.3767401 |
| YPR024W | YME1 | 0.72432888 | 0.97653633 | -0.0872096 | 57.83559508 | 123.3940322 |
| YMR157C | FMP39 | 0.81277379 | 0.9824616 | -0.0871624 | 89.03285697 | 181.8976861 |
| YMR027W | YMR027W | 0.83348655 | 0.9824616 | -0.0871324 | 93.64882512 | 190.5499843 |
| YIL073C | SPO22 | 0.77011069 | 0.97903138 | -0.0871072 | 90.09040172 | 183.8717003 |
| YNL334C | SNO2 | 0.81222411 | 0.9824616 | -0.0868549 | 89.30827101 | 182.3616174 |
| YOL118C | YOL118C | 0.78624331 | 0.97903138 | -0.0868211 | 111.371842 | 223.7369569 |
| YHR103W | SBE22 | 0.83226436 | 0.9824616 | -0.0867291 | 87.40566164 | 178.7716668 |
| YNL106C | INP52 | 0.67305159 | 0.96992497 | -0.0866563 | 72.90218527 | 151.5573679 |
| YJL059W | YHC3 | 0.69018973 | 0.96992497 | -0.0866289 | 114.2509013 | 229.1038617 |
| YDL106C | PHO2 | 0.66765296 | 0.96992497 | -0.0864849 | 124.8974794 | 249.0473162 |
| YGR117C | YGR117C | 0.52338803 | 0.95675879 | -0.0863918 | 119.8825089 | 239.6256129 |
| YKL146W | AVT3 | 0.47148454 | 0.95675879 | -0.0859004 | 101.5993563 | 205.2507371 |
| YOL008W | COQ10 | 0.81226066 | 0.9824616 | -0.0856471 | 55.13271087 | 118.0573282 |
| YKL171W | YKL171W | 0.81952117 | 0.9824616 | -0.0853941 | 92.24379758 | 187.6173924 |
| YDR128W | MTC5 | 0.52405255 | 0.95675879 | -0.0849078 | 111.3794653 | 223.4239042 |
| YGL231C | YGL231C | 0.80608353 | 0.9824616 | -0.084788 | 85.79292149 | 175.4148075 |
| YNL077W | APJ1 | 0.74733766 | 0.97903138 | -0.084617 | 88.61653613 | 180.6813536 |
| YMR166C | YMR166C | 0.80292735 | 0.9824616 | -0.0843837 | 46.36626043 | 101.3993349 |
| YBL100C | YBL100C | 0.64030229 | 0.96479866 | -0.0841631 | 109.9688669 | 220.6508486 |
| YER183C | FAU1 | 0.48037261 | 0.95675879 | -0.0841248 | 113.218246 | 226.7386436 |
| YCR032W | BPH1 | 0.84299112 | 0.98519209 | -0.0840776 | 104.7876372 | 210.9186174 |
| YPL074W | YTA6 | 0.6254731 | 0.96098984 | -0.0838614 | 105.5652498 | 212.3400746 |
| YMR031W-A | YMR031W-A | 0.80516922 | 0.9824616 | -0.083825 | 72.50499762 | 150.3279954 |
| YHR079C | IRE1 | 0.84967065 | 0.98660791 | -0.0830071 | 109.29524 | 219.1896527 |
| YLR098C | CHA4 | 0.6212249 | 0.96098984 | -0.0829752 | 88.35325207 | 179.9066461 |
| YPR130C | YPR130C | 0.67519282 | 0.96992497 | -0.0829736 | 97.81449742 | 197.6513197 |
| YER114C | BOI2 | 0.45664599 | 0.95675879 | -0.0829483 | 105.0321382 | 211.1839807 |
| YCR007C | YCR007C | 0.74509105 | 0.97903138 | -0.0827633 | 111.6482999 | 223.5612029 |
| YGR026W | YGR026W | 0.56891033 | 0.95959383 | -0.0824257 | 121.455741 | 241.8976889 |
| YBR044C | TCM62 | 0.81049651 | 0.9824616 | -0.0824243 | 83.91443464 | 171.4872044 |
| YOR166C | SWT1 | 0.67699205 | 0.96992497 | -0.0818307 | 109.8639094 | 220.0549376 |
| YOR349W | CIN1 | 0.59173337 | 0.95959383 | -0.0816191 | 104.9794546 | 210.8577527 |
| YIR024C | YIR024C | 0.70400165 | 0.97267176 | -0.0810483 | 116.8568916 | 233.0366945 |
| YDR508C | GNP1 | 0.60968683 | 0.95959383 | -0.0805174 | 119.8906655 | 238.6358389 |
| YDR105C | TMS1 | 0.62666329 | 0.96098984 | -0.0804749 | 109.6898398 | 219.4965016 |
| YER048C | CAJ1 | 0.57871256 | 0.95959383 | -0.0802814 | 119.4056715 | 237.6858374 |
| YPR007C | REC8 | 0.50816028 | 0.95675879 | -0.0802678 | 92.00148008 | 186.2858266 |
| YBR176W | ECM31 | 0.52901478 | 0.95675879 | -0.0801388 | 108.8270166 | 217.820731 |
| YHR151C | MTC6 | 0.7791392 | 0.97903138 | -0.0800865 | 91.36005083 | 185.0517869 |
| YPR164W | MMS1 | 0.80989156 | 0.9824616 | -0.0800643 | 57.02350126 | 120.6483885 |
| YGL026C | TRP5 | 0.83973322 | 0.98484693 | -0.0796603 | 98.49629417 | 198.3631808 |
| YOR006C | YOR006C | 0.80206724 | 0.9824616 | -0.0792982 | 106.1231535 | 212.6057111 |
| YJR099W | YUH1 | 0.68808776 | 0.96992497 | -0.0791848 | 114.0173158 | 227.3921223 |
| YGL222C | EDC1 | 0.6030479 | 0.95959383 | -0.0791144 | 116.5620493 | 232.1528317 |
| YKR095W | MLP1 | 0.81940981 | 0.9824616 | -0.0788894 | 81.37083735 | 166.1117889 |
| YER128W | YER128W | 0.5047028 | 0.95675879 | -0.0787066 | 104.7181935 | 209.8694346 |
| YDR368W | YPR1 | 0.60703615 | 0.95959383 | -0.0785886 | 109.1651347 | 218.1896527 |
| YOL020W | TAT2 | 0.63928383 | 0.96454138 | -0.0785117 | 106.2715536 | 212.7494663 |
| YKL200C | YKL200C | 0.65936746 | 0.96992497 | -0.0785074 | 98.79968557 | 198.7349534 |
| YOL163W | YOL163W | 0.70148 | 0.97267176 | -0.0783409 | 116.1500341 | 231.2477459 |
| YER164W | CHD1 | 0.56044202 | 0.95959383 | -0.0782403 | 104.9120177 | 210.1531671 |
| YIL154C | IMP2' | 0.82143173 | 0.9824616 | -0.0781932 | 85.3975477 | 173.5449406 |
| YJL138C | TIF2 | 0.81205044 | 0.9824616 | -0.0780334 | 76.49095913 | 156.8129314 |
| YPR193C | HPA2 | 0.6890361 | 0.96992497 | -0.0775381 | 114.1657582 | 227.3888049 |
| YMR313C | TGL3 | 0.76940271 | 0.97903138 | -0.0773883 | 84.01726553 | 170.8184509 |
| YNL041C | COG6 | 0.79890981 | 0.9824616 | -0.0770668 | 75.03210566 | 153.9114058 |
| YPL095C | EEB1 | 0.67741756 | 0.96992497 | -0.0769066 | 114.4894592 | 227.8878651 |
| YPL156C | PRM4 | 0.34263691 | 0.95675879 | -0.0767288 | 99.82456492 | 200.3528367 |
| YGL156W | AMS1 | 0.5902942 | 0.95959383 | -0.0766109 | 119.571555 | 237.368947 |
| YMR018W | YMR018W | 0.83514018 | 0.98249094 | -0.076588 | 89.48709468 | 180.9404148 |
| YJR062C | NTA1 | 0.74473827 | 0.97903138 | -0.0764051 | 97.57005483 | 196.0690299 |
| YAR043C | YAR043C | 0.83874155 | 0.98484693 | -0.0762976 | 116.8694713 | 232.2474735 |
| YER045C | ACA1 | 0.57821451 | 0.95959383 | -0.0761886 | 105.8040547 | 211.4751851 |

| | | | | | | |
|---|---|---|---|---|---|---|
| YLL029W | YLL029W | 0.61127103 | 0.9598703 | -0.0759975 | 97.62285479 | 196.0983205 |
| YER046W-A | YER046W-A | 0.57303623 | 0.95959383 | -0.0758456 | 113.9200935 | 226.6384736 |
| YLR387C | REH1 | 0.44849676 | 0.95675879 | -0.0758364 | 100.139187 | 200.790253 |
| YNL144C | YNL144C | 0.85746237 | 0.98660791 | -0.0757983 | 99.07697671 | 198.791505 |
| YLR235C | YLR235C | 0.7712969 | 0.97903138 | -0.0756673 | 80.45271963 | 163.8385509 |
| YFR032C | YFR032C | 0.56390928 | 0.95959383 | -0.0756209 | 121.0927678 | 240.0526635 |
| YDR451C | YHP1 | 0.67186013 | 0.96992497 | -0.0754812 | 119.3734008 | 236.8040192 |
| YHL042W | YHL042W | 0.77726709 | 0.97903138 | -0.0750723 | 81.68511301 | 166.0481524 |
| YMR238W | DFG5 | 0.50731965 | 0.95675879 | -0.0749942 | 114.0248764 | 226.689326 |
| YJL137C | GLG2 | 0.62855007 | 0.96098984 | -0.0746586 | 120.8081603 | 239.3542285 |
| YPL056C | YPL056C | 0.67779674 | 0.96992497 | -0.0740006 | 114.8545973 | 228.0755049 |
| YGL104C | VPS73 | 0.67041566 | 0.96992497 | -0.0738538 | 113.6313986 | 225.7562248 |
| YDR344C | YDR344C | 0.69044009 | 0.96992497 | -0.0738104 | 115.4000676 | 229.0660028 |
| YLR407W | YLR407W | 0.85393004 | 0.98660791 | -0.0734402 | 87.58628136 | 176.8367844 |
| YJR124C | YJR124C | 0.83597175 | 0.98293064 | -0.0733534 | 86.49536157 | 174.7758692 |
| YGL154C | LYS5 | 0.82741395 | 0.9824616 | -0.0729855 | 83.58026332 | 169.2455329 |
| YNL257C | SIP3 | 0.77287464 | 0.97903138 | -0.0727953 | 88.40541457 | 178.262768 |
| YHR129C | ARP1 | 0.85353142 | 0.98660791 | -0.0726402 | 93.47548145 | 187.7453498 |
| YIL041W | GVP36 | 0.85588293 | 0.98660791 | -0.0726032 | 94.89632702 | 190.4038756 |
| YDR458C | HEH2 | 0.6542553 | 0.96992497 | -0.0722757 | 114.7731313 | 227.6275828 |
| YJL058C | BIT61 | 0.66022556 | 0.96992497 | -0.072269 | 113.8171644 | 225.8334871 |
| YDR063W | YDR063W | 0.66918108 | 0.96992497 | -0.0721832 | 111.3932663 | 221.2726946 |
| YNL197C | WHI3 | 0.83931019 | 0.98484693 | -0.0721432 | 79.65347608 | 161.7365721 |
| YBR077C | SLM4 | 0.47753753 | 0.95675879 | -0.0719584 | 114.9968094 | 227.9928189 |
| YJL067W | YJL067W | 0.74003452 | 0.97903138 | -0.0711822 | 118.4651572 | 234.3650331 |
| YBR277C | YBR277C | 0.8075742 | 0.9824616 | -0.071078 | 84.87328091 | 171.3442878 |
| YOR193W | PEX27 | 0.64682497 | 0.96742126 | -0.0709314 | 115.9488366 | 229.6026766 |
| YLR176C | RFX1 | 0.73255024 | 0.97731085 | -0.0708847 | 105.36026 | 209.7353707 |
| YPL207W | TYW1 | 0.63078141 | 0.96098984 | -0.0707306 | 109.497856 | 217.4692336 |
| YKL121W | YKL121W | 0.84239782 | 0.98509157 | -0.0706098 | 83.54576706 | 168.7743815 |
| YLR138W | NHA1 | 0.68718155 | 0.96992497 | -0.0705381 | 66.46668659 | 136.7296045 |
| YPL206C | YPL206C | 0.5462018 | 0.95854866 | -0.0703719 | 106.914847 | 212.5633291 |
| YHL036W | MUP3 | 0.87168814 | 0.98901687 | -0.0703458 | 105.8861871 | 210.629568 |
| YNL281W | HCH1 | 0.79098943 | 0.98108279 | -0.0703281 | 90.55016916 | 181.8632138 |
| YBR144C | YBR144C | 0.62796944 | 0.96098984 | -0.0702845 | 108.5240893 | 215.5665814 |
| YDR533C | HSP31 | 0.51195989 | 0.95675879 | -0.0702807 | 116.4792611 | 230.4861743 |
| YER065C | ICL1 | 0.4370052 | 0.95675879 | -0.0700711 | 101.301822 | 201.9844109 |
| YBR090C | YBR090C | 0.72667202 | 0.97673059 | -0.0700291 | 121.4479661 | 239.7621346 |
| YHR191C | CTF8 | 0.77301605 | 0.97903138 | -0.0699896 | 80.40337042 | 162.7745731 |
| YKR096W | YKR096W | 0.57374964 | 0.95959383 | -0.069652 | 106.9193016 | 212.4485084 |
| YPL070W | MUK1 | 0.66201858 | 0.96992497 | -0.0696436 | 100.429073 | 200.2743951 |
| YFL032W | YFL032W | 0.45776143 | 0.95675879 | -0.0695338 | 109.4393428 | 217.1547227 |
| YEL052W | AFG1 | 0.61242215 | 0.9598703 | -0.0690115 | 113.8741969 | 225.383116 |
| YBL003C | HTA2 | 0.69801933 | 0.97267176 | -0.0689568 | 123.4423241 | 243.319162 |
| YPR063C | YPR063C | 0.74479972 | 0.97903138 | -0.068858 | 101.4183474 | 201.995401 |
| YNR007C | ATG3 | 0.83994141 | 0.98484693 | -0.0687618 | 84.43448909 | 170.1250307 |
| YEL023C | YEL023C | 0.49664722 | 0.95675879 | -0.0686908 | 105.7751015 | 210.1380599 |
| YHL016C | DUR3 | 0.8617795 | 0.98660791 | -0.0686547 | 97.35146097 | 194.3329995 |
| YEL017W | GTT3 | 0.63160869 | 0.96098984 | -0.0684644 | 110.5775327 | 219.1064849 |
| YHR124W | NDT80 | 0.81163689 | 0.9824616 | -0.0682994 | 92.8448961 | 185.8199722 |
| YEL067C | YEL067C | 0.63075542 | 0.96098984 | -0.0682529 | 109.5002017 | 217.0497261 |
| YCR091W | KIN82 | 0.59683954 | 0.95959383 | -0.0681392 | 114.6400175 | 226.6702055 |
| YMR319C | FET4 | 0.66945614 | 0.96992497 | -0.0680926 | 97.45514791 | 194.4313069 |
| YJL206C | YJL206C | 0.8092351 | 0.9824616 | -0.0679653 | 95.35773076 | 190.4757407 |
| YCL057W | PRD1 | 0.4930202 | 0.95675879 | -0.0675182 | 125.3787779 | 246.704934 |
| YLR442C | SIR3 | 0.34102501 | 0.95675879 | -0.0674202 | 128.8584631 | 253.2144614 |
| YDR409W | SIZ1 | 0.70742694 | 0.97267176 | -0.0672237 | 116.3981274 | 229.8109595 |
| YDR386W | MUS81 | 0.6868537 | 0.96992497 | -0.0670493 | 118.9409475 | 234.5502953 |
| YDL144C | YDL144C | 0.68132466 | 0.96992497 | -0.0667236 | 120.7194641 | 237.8302422 |
| YOR237W | HES1 | 0.73885241 | 0.97903138 | -0.0666157 | 114.9430851 | 226.9779445 |
| YPL197C | YPL197C | 0.68910906 | 0.96992497 | -0.0666092 | 107.9439025 | 213.8495936 |
| YKR041W | YKR041W | 0.87495368 | 0.98901687 | -0.066578 | 104.8684351 | 208.0760914 |
| YCR088W | ABP1 | 0.64456811 | 0.96617034 | -0.0665487 | 131.6863204 | 258.3690974 |
| YMR202W | ERG2 | 0.66510319 | 0.96992497 | -0.0664116 | 5.695576991 | 22.04489606 |

| | | | | | | |
|---|---|---|---|---|---|---|
| YER185W | YER185W | 0.69137265 | 0.97049825 | -0.0663319 | 105.7939999 | 209.7699191 |
| YPL258C | THI21 | 0.75991369 | 0.97903138 | -0.0661818 | 117.8836387 | 232.4188397 |
| YGL067W | NPY1 | 0.64102715 | 0.96479866 | -0.0661626 | 113.6497176 | 224.4746678 |
| YDL013W | HEX3 | 0.88703354 | 0.98910891 | -0.065987 | 49.5229157 | 104.1722019 |
| YDR249C | YDR249C | 0.70791639 | 0.97267176 | -0.0658069 | 107.2334876 | 212.379907 |
| YDR525W | API2 | 0.48347283 | 0.95675879 | -0.0653255 | 108.3934907 | 214.4731799 |
| YER188W | YER188W | 0.47229362 | 0.95675879 | -0.0652861 | 107.7470355 | 213.2539846 |
| YOR010C | TIR2 | 0.71496884 | 0.97400895 | -0.0652481 | 111.0456039 | 219.4340725 |
| YGL196W | YGL196W | 0.70544311 | 0.97267176 | -0.0650985 | 111.8002367 | 220.8238325 |
| YER080W | FMP29 | 0.7361446 | 0.97781627 | -0.0650858 | 116.714343 | 230.0382634 |
| YAL022C | FUN26 | 0.69912549 | 0.97267176 | -0.0647503 | 113.2600922 | 223.5022754 |
| YJL185C | YJL185C | 0.6761816 | 0.96992497 | -0.0645887 | 119.4897446 | 235.1585912 |
| YJL176C | SWI3 | 0.77354444 | 0.97903138 | -0.0642146 | 120.6234968 | 237.220975 |
| YDL237W | YDL237W | 0.69961543 | 0.97267176 | -0.0641825 | 117.2854706 | 230.9548788 |
| YLR236C | YLR236C | 0.68561115 | 0.96992497 | -0.0639738 | 105.2164873 | 208.2833042 |
| YER005W | YND1 | 0.69292161 | 0.97133302 | -0.0634746 | 113.9221941 | 224.5258146 |
| YER016W | BIM1 | 0.84292847 | 0.98519209 | -0.0634647 | 98.90606802 | 196.360758 |
| YOR058C | ASE1 | 0.70229003 | 0.97267176 | -0.0634514 | 113.7170083 | 224.1369997 |
| YER059W | PCL6 | 0.63222865 | 0.96098984 | -0.0631756 | 101.779483 | 201.7005023 |
| YPL203W | TPK2 | 0.80493265 | 0.9824616 | -0.0630728 | 121.1227258 | 237.9619453 |
| YGR254W | ENO1 | 0.72213099 | 0.97591869 | -0.0629477 | 98.2820625 | 195.1019574 |
| YDR179C | CSN9 | 0.68158657 | 0.96992497 | -0.0627435 | 114.4623057 | 225.4137209 |
| YIR030C | DCG1 | 0.71120854 | 0.97267176 | -0.0625819 | 123.3841051 | 242.1192684 |
| YHR093W | AHT1 | 0.64989197 | 0.96848639 | -0.0625725 | 112.3868081 | 221.4917804 |
| YLR119W | SRN2 | 0.85124578 | 0.98660791 | -0.0622986 | 81.17984741 | 162.9149986 |
| YDR512C | EMI1 | 0.8486528 | 0.98660791 | -0.0621637 | 81.00743445 | 162.5685538 |
| YEL041W | YEF1 | 0.71935552 | 0.97530288 | -0.0620991 | 123.7180111 | 242.6629222 |
| YNL074C | MLF3 | 0.88042797 | 0.98910891 | -0.0620953 | 93.48919078 | 185.9668928 |
| YBR210W | ERV15 | 0.68838219 | 0.96992497 | -0.062042 | 120.0374126 | 235.7500441 |
| YCL074W | YCL074W | 0.88644192 | 0.98910891 | -0.062029 | 99.02271252 | 196.3338885 |
| YAL013W | DEP1 | 0.88141757 | 0.98910891 | -0.0618132 | 102.0511224 | 201.9768826 |
| YPL245W | YPL245W | 0.66181588 | 0.96992497 | -0.0617661 | 109.1506595 | 215.2842785 |
| YBR224W | YBR224W | 0.84681037 | 0.98660791 | -0.0613889 | 99.68882415 | 197.473695 |
| YLR097C | HRT3 | 0.73675123 | 0.97793193 | -0.061252 | 101.9189089 | 201.6328775 |
| YAL024C | LTE1 | 0.66516279 | 0.96992497 | -0.0610405 | 91.50960949 | 182.0736307 |
| YHR125W | YHR125W | 0.8178388 | 0.9824616 | -0.0609811 | 84.01041973 | 167.9984366 |
| YGL260W | YGL260W | 0.55659943 | 0.95959383 | -0.0608564 | 110.8403244 | 218.2976804 |
| YFR019W | FAB1 | 0.63586546 | 0.96276667 | -0.0606069 | 108.7442648 | 214.3237479 |
| YDR134C | YDR134C | 0.72202397 | 0.97591869 | -0.0605386 | 113.8974067 | 223.9769798 |
| YLR055C | SPT8 | 0.8675596 | 0.98696321 | -0.0604979 | 82.32597256 | 164.7565194 |
| YPL015C | HST2 | 0.76023027 | 0.97903138 | -0.0603407 | 101.0810617 | 199.9055533 |
| YGL211W | NCS6 | 0.85783382 | 0.98660791 | -0.0601491 | 81.01569001 | 162.2393555 |
| YPL229W | YPL229W | 0.68577512 | 0.96992497 | -0.0596721 | 111.5670399 | 219.4580398 |
| YGR021W | YGR021W | 0.6532865 | 0.96992497 | -0.0595591 | 106.7072941 | 210.3240528 |
| YGL009C | LEU1 | 0.73388869 | 0.97731085 | -0.0595031 | 118.7931634 | 232.9820036 |
| YER097W | YER097W | 0.63346599 | 0.96098984 | -0.0592472 | 112.7280306 | 221.5628277 |
| YPR071W | YPR071W | 0.63386752 | 0.96098984 | -0.0591546 | 101.8323828 | 201.111742 |
| YOR101W | RAS1 | 0.76053665 | 0.97903138 | -0.0589283 | 118.9359299 | 233.1514268 |
| YGL259W | YPS5 | 0.69469898 | 0.97230685 | -0.0588515 | 122.115731 | 239.1021324 |
| YDR490C | PKH1 | 0.5990041 | 0.95959383 | -0.0588298 | 121.3904502 | 237.7381255 |
| YOL121C | RPS19A | 0.71129619 | 0.97267176 | -0.0586437 | 112.5784177 | 221.1789682 |
| YBL001C | ECM15 | 0.65681926 | 0.96992497 | -0.0585967 | 118.7586397 | 232.7621786 |
| YOL019W | YOL019W | 0.73898282 | 0.97903138 | -0.0582921 | 108.895103 | 214.2106078 |
| YEL056W | HAT2 | 0.60292121 | 0.95959383 | -0.0580813 | 115.649385 | 226.8424589 |
| YPL161C | BEM4 | 0.84775923 | 0.98660791 | -0.0580634 | 90.07203271 | 178.8680384 |
| YPL066W | YPL066W | 0.70266867 | 0.97267176 | -0.0579938 | 97.38731982 | 192.5762429 |
| YGR235C | YGR235C | 0.87081314 | 0.98870218 | -0.0578997 | 95.89600316 | 189.7631272 |
| YJL055W | YJL055W | 0.78181802 | 0.97903138 | -0.0577768 | 123.2185235 | 240.9865857 |
| YPR073C | LTP1 | 0.8325021 | 0.9824616 | -0.0575386 | 103.0825522 | 203.1800021 |
| YIL173W | VTH1 | 0.73643638 | 0.97781627 | -0.0574879 | 112.3323081 | 220.5196311 |
| YER077C | YER077C | 0.82346882 | 0.9824616 | -0.0573741 | 87.74008876 | 174.3764556 |
| YDR149C | YDR149C | 0.76570026 | 0.97903138 | -0.0569373 | 101.0284406 | 199.2245659 |
| YDR171W | HSP42 | 0.7779734 | 0.97903138 | -0.0567836 | 111.4328956 | 218.712243 |
| YLR004C | THI73 | 0.76180352 | 0.97903138 | -0.0566608 | 100.5441905 | 198.2690193 |

| | | | | | | |
|---|---|---|---|---|---|---|
| YJR118C | ILM1 | 0.89127222 | 0.98910891 | -0.0566024 | 84.53254699 | 168.22854 |
| YDR479C | PEX29 | 0.89905653 | 0.98910891 | -0.0561652 | 105.5866619 | 207.6415826 |
| YBR157C | ICS2 | 0.74239458 | 0.97903138 | -0.056093 | 120.9571165 | 236.457146 |
| YLR169W | YLR169W | 0.77862429 | 0.97903138 | -0.0560927 | 108.8485786 | 213.747032 |
| YPL147W | PXA1 | 0.74193884 | 0.97903138 | -0.055769 | 108.0965449 | 212.2811913 |
| YBL046W | PSY4 | 0.75045261 | 0.97903138 | -0.0552393 | 114.1263743 | 223.4997468 |
| YPL058C | PDR12 | 0.79037526 | 0.98074698 | -0.0549089 | 106.9893895 | 210.0575057 |
| YKR027W | BCH2 | 0.84174977 | 0.98507962 | -0.0548744 | 65.84674557 | 132.8869129 |
| YNL141W | AAH1 | 0.78423997 | 0.97903138 | -0.0548277 | 69.49167089 | 139.7151222 |
| YBR107C | IML3 | 0.71837454 | 0.97530288 | -0.0545479 | 111.6352013 | 218.7091605 |
| YMR201C | RAD14 | 0.75304302 | 0.97903138 | -0.0542393 | 109.3438552 | 214.3588508 |
| YLL060C | GTT2 | 0.67487447 | 0.96992497 | -0.0539504 | 101.5300727 | 199.6543491 |
| YDR541C | YDR541C | 0.75237325 | 0.97903138 | -0.0538479 | 118.9317843 | 232.2744214 |
| YGR085C | RPL11B | 0.88171626 | 0.98910891 | -0.053756 | 82.96504433 | 164.8016214 |
| YBR149W | ARA1 | 0.60375607 | 0.95959383 | -0.0535972 | 98.57713822 | 194.0555734 |
| YDR387C | YDR387C | 0.75816107 | 0.97903138 | -0.0535245 | 108.9120327 | 213.4266537 |
| YPL052W | OAZ1 | 0.78186556 | 0.97903138 | -0.0534564 | 110.2927582 | 216.0045995 |
| YDR150W | NUM1 | 0.81010869 | 0.9824616 | -0.0531536 | 94.29763543 | 185.9532891 |
| YER019C-A | SBH2 | 0.666681 | 0.96992497 | -0.0529601 | 113.5555493 | 222.0391837 |
| YDR032C | PST2 | 0.78592844 | 0.97903138 | -0.0528874 | 101.085794 | 198.6392089 |
| YJR066W | TOR1 | 0.78040165 | 0.97903138 | -0.0528673 | 98.88056435 | 194.4997847 |
| YMR190C | SGS1 | 0.84415632 | 0.98543854 | -0.0527096 | 86.76546623 | 171.7504388 |
| YPL113C | YPL113C | 0.68357368 | 0.96992497 | -0.0526566 | 93.87890687 | 185.0829206 |
| YOR126C | IAH1 | 0.7337543 | 0.97731085 | -0.0525153 | 113.1192364 | 221.1447618 |
| YOR245C | DGA1 | 0.70602121 | 0.97267176 | -0.0524378 | 103.1576729 | 202.4481784 |
| YOL058W | ARG1 | 0.9213064 | 0.98998274 | -0.0523358 | 107.452074 | 210.4850609 |
| YLR217W | YLR217W | 0.72532488 | 0.97673059 | -0.0522701 | 105.5955661 | 206.9918631 |
| YKL161C | YKL161C | 0.70158943 | 0.97267176 | -0.0521722 | 113.5609983 | 221.9145919 |
| YAL037W | YAL037W | 0.71278042 | 0.97267176 | -0.0521453 | 122.9321442 | 239.4859687 |
| YGR023W | MTL1 | 0.65984002 | 0.96992497 | -0.0519281 | 112.5036422 | 219.889714 |
| YER085C | YER085C | 0.74400505 | 0.97903138 | -0.0514068 | 106.7933341 | 209.0906068 |
| YKL139W | CTK1 | 0.86952057 | 0.98801835 | -0.0510172 | 32.13792232 | 69.00470483 |
| YGL243W | TAD1 | 0.7176645 | 0.97530288 | -0.0508939 | 112.3620059 | 219.4471337 |
| YDL089W | YDL089W | 0.91712897 | 0.98910891 | -0.0504348 | 82.76449294 | 163.8572424 |
| YBR084C-A | RPL19A | 0.74892255 | 0.97903138 | -0.0500449 | 107.758919 | 210.6686018 |
| YIL042C | PKP1 | 0.91838744 | 0.98910891 | -0.0498879 | 100.5982163 | 197.2115463 |
| YJR133W | XPT1 | 0.89584867 | 0.98910891 | -0.049791 | 86.70893369 | 171.1450542 |
| YGR034W | RPL26B | 0.89654444 | 0.98910891 | -0.049763 | 90.05468332 | 177.4153581 |
| YDR117C | TMA64 | 0.81101017 | 0.9824616 | -0.0497494 | 126.0051557 | 244.839592 |
| YOL017W | ESC8 | 0.78469035 | 0.97903138 | -0.0496948 | 111.8440448 | 218.2705098 |
| YER096W | SHC1 | 0.74584328 | 0.97903138 | -0.049643 | 114.4224127 | 223.0974845 |
| YHL019C | APM2 | 0.82514757 | 0.9824616 | -0.0494283 | 99.47161031 | 195.0199081 |
| YPL032C | SVL3 | 0.81530812 | 0.9824616 | -0.0492827 | 115.8958867 | 225.7993996 |
| YJR115W | YJR115W | 0.80949328 | 0.9824616 | -0.0491594 | 89.48897031 | 176.2510611 |
| YDL218W | YDL218W | 0.80915218 | 0.9824616 | -0.0489337 | 129.5083489 | 251.2704132 |
| YHR199C | FMP34 | 0.87912331 | 0.98910891 | -0.0488382 | 99.39748478 | 194.7799256 |
| YLL055W | YLL055W | 0.75801403 | 0.97903138 | -0.0487854 | 103.7255401 | 202.8883383 |
| YER109C | FLO8 | 0.69915565 | 0.97267176 | -0.0485613 | 110.7489342 | 216.0226579 |
| YFR012W | YFR012W | 0.88546235 | 0.98910891 | -0.0483882 | 109.3897342 | 213.4438114 |
| YPR122W | AXL1 | 0.72260394 | 0.97591869 | -0.0483155 | 95.38163489 | 187.1586112 |
| YPL079W | RPL21B | 0.6887404 | 0.96992497 | -0.0482724 | 94.57961382 | 185.6470134 |
| YDR112W | YDR112W | 0.77003863 | 0.97903138 | -0.0481482 | 117.1835396 | 228.0203469 |
| YDL128W | VCX1 | 0.75274284 | 0.97903138 | -0.0480808 | 132.7168057 | 257.1420871 |
| YDR528W | HLR1 | 0.60256992 | 0.95959383 | -0.0479991 | 108.4925732 | 211.694573 |
| YFR053C | HXK1 | 0.71816067 | 0.97530288 | -0.047814 | 123.7176163 | 240.2180932 |
| YCL075W | YCL075W | 0.90305607 | 0.98910891 | -0.0474307 | 112.7238041 | 219.5331702 |
| YNL034W | YNL034W | 0.89632781 | 0.98910891 | -0.0469407 | 85.89568292 | 169.132098 |
| YDR210W | YDR210W | 0.83834258 | 0.98484693 | -0.0464778 | 128.2924006 | 248.5696644 |
| YLR420W | URA4 | 0.92168568 | 0.98998274 | -0.0462657 | 100.8849045 | 197.129505 |
| YER090W | TRP2 | 0.917826 | 0.98910891 | -0.0457976 | 92.57064846 | 181.4556884 |
| YNL159C | ASI2 | 0.8763476 | 0.98901687 | -0.0457191 | 92.23986499 | 180.8218651 |
| YGR178C | PBP1 | 0.60912467 | 0.95959383 | -0.0456331 | 107.7685778 | 209.931883 |
| YAR014C | BUD14 | 0.89967274 | 0.98910891 | -0.045484 | 86.91266582 | 170.7902683 |
| YCR022C | YCR022C | 0.72093465 | 0.97532873 | -0.0453673 | 126.0129998 | 244.1045598 |

| | | | | | | |
|---|---|---|---|---|---|---|
| YFL050C | ALR2 | 0.6271505 | 0.96098984 | -0.045332 | 103.5841885 | 202.0323743 |
| YGR004W | PEX31 | 0.92926444 | 0.99106129 | -0.0451878 | 124.0463699 | 240.3853476 |
| YGR070W | ROM1 | 0.77948397 | 0.97903138 | -0.0451358 | 122.1808191 | 236.8775424 |
| YAR023C | YAR023C | 0.75968973 | 0.97903138 | -0.0451095 | 122.2676998 | 237.0359881 |
| YDL231C | BRE4 | 0.77538273 | 0.97903138 | -0.0450638 | 110.4840402 | 214.9274423 |
| YPR050C | YPR050C | 0.9052908 | 0.98910891 | -0.0450076 | 80.8128944 | 159.2683895 |
| YGR241C | YAP1802 | 0.78383256 | 0.97903138 | -0.0449615 | 103.8125936 | 202.3973587 |
| YKL053C-A | MDM35 | 0.89799217 | 0.98910891 | -0.0449241 | 78.47753185 | 154.8740357 |
| YNL040W | YNL040W | 0.89988451 | 0.98910891 | -0.0446314 | 88.49889855 | 173.6194257 |
| YHR108W | GGA2 | 0.89696118 | 0.98910891 | -0.0445601 | 82.15635889 | 161.7115392 |
| YPR079W | MRL1 | 0.7796803 | 0.97903138 | -0.0440639 | 99.0429374 | 193.2981037 |
| YER049W | TPA1 | 0.74744767 | 0.97903138 | -0.0440536 | 116.01729 | 225.1324263 |
| YLR280C | YLR280C | 0.79256865 | 0.98180632 | -0.0439955 | 105.437381 | 205.2794361 |
| YPL026C | SKS1 | 0.7466495 | 0.97903138 | -0.0439853 | 108.4002636 | 210.834711 |
| YDR349C | YPS7 | 0.92029721 | 0.98998274 | -0.04398 | 93.33233846 | 182.573285 |
| YMR206W | YMR206W | 0.64486357 | 0.96617034 | -0.0437905 | 102.7671288 | 200.2361996 |
| YHL045W | YHL045W | 0.86544167 | 0.98677815 | -0.0436892 | 89.49852027 | 175.3330565 |
| YBR052C | RFS1 | 0.74534017 | 0.97903138 | -0.0435936 | 121.3280256 | 235.0142216 |
| YBR222C | PCS60 | 0.76485718 | 0.97903138 | -0.0433623 | 118.2432978 | 229.1891309 |
| YER187W | YER187W | 0.6445959 | 0.96617034 | -0.0433251 | 109.3771374 | 212.5539201 |
| YDR185C | YDR185C | 0.81145708 | 0.9824616 | -0.0433147 | 126.9082259 | 245.4324076 |
| YFL021W | GAT1 | 0.68471481 | 0.96992497 | -0.0432973 | 115.0231908 | 223.1385569 |
| YOR302W | YOR302W | 0.81996692 | 0.9824616 | -0.0431026 | 107.6539007 | 209.2838435 |
| YBR116C | YBR116C | 0.70265792 | 0.97267176 | -0.0429321 | 109.7430872 | 213.1730279 |
| YDR075W | PPH3 | 0.81872405 | 0.9824616 | -0.0428173 | 117.0286574 | 226.8177753 |
| YDR084C | TVP23 | 0.80557334 | 0.9824616 | -0.0424673 | 114.6109984 | 222.2234684 |
| YDL214C | PRR2 | 0.74106641 | 0.97903138 | -0.0424417 | 107.4456602 | 208.7802134 |
| YCR015C | YCR015C | 0.80611785 | 0.9824616 | -0.0421502 | 124.2190783 | 240.1895582 |
| YOR296W | YOR296W | 0.79654006 | 0.9824616 | -0.0420406 | 114.0372822 | 221.0744298 |
| YMR312W | ELP6 | 0.8232613 | 0.9824616 | -0.0419883 | 67.90553937 | 134.543524 |
| YIL114C | POR2 | 0.8409081 | 0.98489448 | -0.0417007 | 125.4629522 | 242.445594 |
| YIL092W | YIL092W | 0.80753703 | 0.9824616 | -0.0411629 | 104.1822649 | 202.4407744 |
| YLL061W | MMP1 | 0.81824888 | 0.9824616 | -0.0409671 | 105.4998236 | 204.8784144 |
| YOR320C | GNT1 | 0.78941488 | 0.98038716 | -0.0407517 | 100.7932117 | 196.0141238 |
| YPL053C | KTR6 | 0.82525598 | 0.9824616 | -0.0405028 | 119.0688525 | 230.2482358 |
| YDR077W | SED1 | 0.83954475 | 0.98484693 | -0.0404754 | 129.0919882 | 249.0423575 |
| YKL163W | PIR3 | 0.7993312 | 0.9824616 | -0.040271 | 99.89263957 | 194.2428134 |
| YFR022W | ROG3 | 0.8327607 | 0.9824616 | -0.0401634 | 117.3479768 | 226.9625941 |
| YER180C | ISC10 | 0.72732273 | 0.97683349 | -0.0401608 | 111.3420643 | 215.6978229 |
| YGR231C | PHB2 | 0.50208515 | 0.95675879 | -0.0400743 | 89.5815917 | 174.8703818 |
| YPL168W | YPL168W | 0.78974364 | 0.98038716 | -0.0399676 | 95.90366057 | 186.7094121 |
| YMR171C | YMR171C | 0.80028951 | 0.9824616 | -0.0399311 | 102.0236027 | 198.1813683 |
| YAR042W | SWH1 | 0.78376725 | 0.97903138 | -0.0398772 | 115.390057 | 223.2414821 |
| YEL071W | DLD3 | 0.80918371 | 0.9824616 | -0.039774 | 115.6064463 | 223.6296707 |
| YDR401W | YDR401W | 0.73259772 | 0.97731085 | -0.0397671 | 114.2133105 | 221.0156006 |
| YAL031C | GIP4 | 0.80467131 | 0.9824616 | -0.0395997 | 116.6859424 | 225.6244792 |
| YPR121W | THI22 | 0.77871949 | 0.97903138 | -0.0395492 | 103.524673 | 200.931343 |
| YNL047C | SLM2 | 0.86895025 | 0.98763165 | -0.0393437 | 103.6916325 | 201.2093212 |
| YOR115C | TRS33 | 0.83033105 | 0.9824616 | -0.0391728 | 119.0825398 | 230.0463564 |
| YGR110W | YGR110W | 0.90352767 | 0.98910891 | -0.0387996 | 113.7824299 | 220.0419292 |
| YBR106W | PHO88 | 0.88515587 | 0.98910891 | -0.0387289 | 66.91296274 | 132.1242585 |
| YOR301W | RAX1 | 0.77486841 | 0.97903138 | -0.038662 | 107.2733289 | 207.810311 |
| YFL010W-A | AUA1 | 0.73000901 | 0.97683349 | -0.038336 | 97.10513436 | 188.6836648 |
| YLR441C | RPS1A | 0.87623317 | 0.98901687 | -0.0381391 | 76.86386167 | 150.6866527 |
| YBR130C | SHE3 | 0.77357438 | 0.97903138 | -0.0379775 | 112.6134588 | 217.7088283 |
| YCR016W | YCR016W | 0.93826827 | 0.9913313 | -0.037641 | 107.4465241 | 207.9604553 |
| YOR031W | CRS5 | 0.8264508 | 0.9824616 | -0.0375945 | 111.2452441 | 215.0771546 |
| YPL116W | HOS3 | 0.76932316 | 0.97903138 | -0.0374978 | 110.5330632 | 213.724883 |
| YOL153C | YOL153C | 0.8245981 | 0.9824616 | -0.0374052 | 98.45451991 | 191.0552462 |
| YGR066C | YGR066C | 0.70499451 | 0.97267176 | -0.0373729 | 128.0858915 | 246.6245529 |
| YGR086C | PIL1 | 0.86078219 | 0.98660791 | -0.037248 | 103.7506926 | 200.9615409 |
| YDL236W | PHO13 | 0.83391076 | 0.9824616 | -0.0367712 | 104.2054033 | 201.7327885 |
| YHR121W | LSM12 | 0.90613579 | 0.98910891 | -0.0367642 | 93.71565777 | 182.0576482 |
| YOR239W | ABP140 | 0.83349997 | 0.9824616 | -0.0366716 | 113.8308003 | 219.7685669 |

| | | | | | | |
|---|---|---|---|---|---|---|
| YHL030W | ECM29 | 0.91835302 | 0.98910891 | -0.0366014 | 87.21206818 | 169.8320408 |
| YAL065C | YAL065C | 0.94404042 | 0.99161193 | -0.036351 | 92.14277324 | 179.0369436 |
| YER135C | YER135C | 0.79844174 | 0.9824616 | -0.0363266 | 109.5443071 | 211.6700405 |
| YHL047C | ARN2 | 0.92105014 | 0.98998274 | -0.0363155 | 110.3676638 | 213.2123905 |
| YOR264W | DSE3 | 0.83016668 | 0.9824616 | -0.0361818 | 115.9094727 | 223.5834 |
| YER143W | DDI1 | 0.82878174 | 0.9824616 | -0.0360551 | 114.6215111 | 221.1460952 |
| YJR026W | YJR026W | 0.85447629 | 0.98660791 | -0.0359005 | 120.0729515 | 231.3440463 |
| YLR152C | YLR152C | 0.88352688 | 0.98910891 | -0.0353125 | 107.4555527 | 207.5789984 |
| YIL098C | FMC1 | 0.80886785 | 0.9824616 | -0.0352748 | 92.05094107 | 178.6805815 |
| YAL019W | FUN30 | 0.86387033 | 0.98660791 | -0.035251 | 117.4902057 | 226.3888734 |
| YCR087C-A | LUG1 | 0.92670576 | 0.99106129 | -0.0352185 | 99.32225405 | 192.3085759 |
| YOR087W | YVC1 | 0.82884774 | 0.9824616 | -0.035117 | 111.3759729 | 214.8984603 |
| YMR316C-B | YMR316C-B | 0.80787564 | 0.9824616 | -0.0351142 | 120.8103848 | 232.5926021 |
| YJR153W | PGU1 | 0.8343318 | 0.9824616 | -0.034956 | 112.9643773 | 217.85003 |
| YLR024C | UBR2 | 0.80378657 | 0.9824616 | -0.034714 | 136.9095406 | 262.71874 |
| YDR198C | RKM2 | 0.80567946 | 0.9824616 | -0.0345355 | 116.3410364 | 224.1111491 |
| YIL123W | SIM1 | 0.88363946 | 0.98910891 | -0.0345199 | 122.279521 | 235.2463403 |
| YFR011C | YFR011C | 0.91628041 | 0.98910891 | -0.0344824 | 93.96789672 | 182.1403289 |
| YMR322C | SNO4 | 0.67282827 | 0.96992497 | -0.0342072 | 104.5315629 | 201.905826 |
| YMR054W | STV1 | 0.82605947 | 0.9824616 | -0.0335277 | 112.6605437 | 217.0358011 |
| YBL079W | NUP170 | 0.92740937 | 0.99106129 | -0.0334562 | 90.56089936 | 175.5747968 |
| YDR282C | YDR282C | 0.85452627 | 0.98660791 | -0.0334222 | 120.5536712 | 231.8216327 |
| YJR058C | APS2 | 0.92916222 | 0.99106129 | -0.0333746 | 92.5992155 | 179.383773 |
| YCR075C | ERS1 | 0.85028443 | 0.98660791 | -0.0331447 | 115.9124285 | 223.0693157 |
| YLR037C | DAN2 | 0.80453314 | 0.9824616 | -0.0329396 | 102.5138832 | 197.9047002 |
| YMR111C | YMR111C | 0.79689696 | 0.9824616 | -0.0328139 | 109.9725517 | 211.8722321 |
| YJR094C | IME1 | 0.86194487 | 0.98660791 | -0.0327713 | 114.135994 | 219.6736481 |
| YFL063W | YFL063W | 0.83427247 | 0.9824616 | -0.0327401 | 106.3798111 | 205.1212842 |
| YPR199C | ARR1 | 0.87954696 | 0.98910891 | -0.0326827 | 110.3061884 | 212.4755463 |
| YGL165C | YGL165C | 0.83315484 | 0.9824616 | -0.0325534 | 110.0210213 | 211.9185806 |
| YDL211C | YDL211C | 0.79341167 | 0.98181836 | -0.0323667 | 118.6278743 | 228.0291333 |
| YIL149C | MLP2 | 0.86756129 | 0.98696321 | -0.0321364 | 123.088262 | 236.3553617 |
| YGL050W | TYW3 | 0.82892961 | 0.9824616 | -0.031727 | 127.604368 | 244.7554557 |
| YIL015W | BAR1 | 0.87638779 | 0.98901687 | -0.0316175 | 125.1359372 | 240.1070888 |
| YPR200C | ARR2 | 0.86109852 | 0.98660791 | -0.0315019 | 103.3692219 | 199.2629443 |
| YDR503C | LPP1 | 0.81270393 | 0.9824616 | -0.0311075 | 109.682295 | 211.0358973 |
| YOR180C | DCI1 | 0.76572329 | 0.97903138 | -0.0310286 | 97.23258806 | 187.672458 |
| YOR279C | RFM1 | 0.89386318 | 0.98910891 | -0.0309196 | 123.3391682 | 236.6177628 |
| YGL175C | SAE2 | 0.83072942 | 0.9824616 | -0.030899 | 122.9686318 | 235.9192886 |
| YPR167C | MET16 | 0.91511765 | 0.98910891 | -0.0308037 | 89.52008136 | 173.1688585 |
| YIL164C | NIT1 | 0.89027051 | 0.98910891 | -0.0303918 | 119.1122095 | 228.5996365 |
| YDR519W | FPR2 | 0.78963543 | 0.98038716 | -0.0302711 | 110.289258 | 212.0311821 |
| YLR200W | YKE2 | 0.90642817 | 0.98910891 | -0.030247 | 74.47959262 | 144.8645711 |
| YJL208C | NUC1 | 0.88415915 | 0.98910891 | -0.0301151 | 122.9950322 | 235.8346767 |
| YNL104C | LEU4 | 0.93936482 | 0.9913313 | -0.0300942 | 97.45810336 | 187.9355533 |
| YLR124W | YLR124W | 0.85691095 | 0.98660791 | -0.0299406 | 113.7796158 | 218.5209423 |
| YOR140W | SFL1 | 0.39100222 | 0.95675879 | -0.0298993 | 0 | 5.115580282 |
| YIL097W | FYV10 | 0.86098482 | 0.98660791 | -0.0298194 | 94.11615699 | 181.6205815 |
| YER063W | THO1 | 0.84432817 | 0.98543854 | -0.0296481 | 121.6513891 | 233.2347217 |
| YPL154C | PEP4 | 0.87316742 | 0.98901687 | -0.0295659 | 112.5380737 | 216.1282587 |
| YJL078C | PRY3 | 0.86296495 | 0.98660791 | -0.029541 | 97.9985102 | 188.8544634 |
| YGR154C | GTO1 | 0.73164132 | 0.97731085 | -0.0294401 | 114.7073973 | 220.1753912 |
| YFL054C | YFL054C | 0.74906869 | 0.97903138 | -0.02916 | 116.1046754 | 222.7481215 |
| YHR075C | PPE1 | 0.78208517 | 0.97903138 | -0.0291025 | 100.8300712 | 194.0901525 |
| YHR209W | CRG1 | 0.92411847 | 0.99105012 | -0.0289712 | 91.31769613 | 176.2268326 |
| YDL019C | OSH2 | 0.88662777 | 0.98910891 | -0.0288856 | 123.1363244 | 235.8893264 |
| YFL052W | YFL052W | 0.84475996 | 0.98543854 | -0.0288831 | 117.0394294 | 224.4539248 |
| YNL010W | YNL010W | 0.93367535 | 0.99106129 | -0.0287705 | 92.31772538 | 178.0680906 |
| YDR481C | PHO8 | 0.86453019 | 0.98660791 | -0.0284724 | 112.5915137 | 216.0414064 |
| YNL012W | SPO1 | 0.90024694 | 0.98910891 | -0.0283276 | 125.188671 | 239.6431007 |
| YKR013W | PRY2 | 0.8301476 | 0.9824616 | -0.0282882 | 107.0806551 | 205.674052 |
| YKR078W | YKR078W | 0.82957179 | 0.9824616 | -0.0282818 | 108.5900398 | 208.5038737 |
| YBR146W | MRPS9 | 0.8421582 | 0.98507962 | -0.0282554 | 117.8959301 | 225.952932 |
| YEL011W | GLC3 | 0.83369065 | 0.9824616 | -0.0282086 | 106.3707911 | 204.3290572 |

| | | | | | | |
|---|---|---|---|---|---|---|
| YDR096W | GIS1 | 0.85896224 | 0.98660791 | -0.0280649 | 106.3885765 | 204.3378233 |
| YFL006W | YFL006W | 0.86991596 | 0.98820612 | -0.0279349 | 112.653387 | 216.0654838 |
| YIL015C-A | YIL015C-A | 0.94225337 | 0.9913313 | -0.0278314 | 92.47749818 | 178.2070868 |
| YGL054C | ERV14 | 0.85419708 | 0.98660791 | -0.0277798 | 104.3344573 | 200.4364632 |
| YDR097C | MSH6 | 0.86118635 | 0.98660791 | -0.0277065 | 101.6583207 | 195.4047074 |
| YDR186C | YDR186C | 0.84062826 | 0.98489448 | -0.0276346 | 115.2256527 | 220.8385009 |
| YJR128W | YJR128W | 0.86200019 | 0.98660791 | -0.0273411 | 90.49452796 | 174.4040452 |
| YDR056C | YDR056C | 0.87537749 | 0.98901687 | -0.0270814 | 118.4030578 | 226.7031984 |
| YMR158W-A | YMR158W-A | 0.9423084 | 0.9913313 | -0.0269544 | 86.8952099 | 167.5872198 |
| YOR142W | LSC1 | 0.90516856 | 0.98910891 | -0.0269004 | 83.07277005 | 160.4088375 |
| YJR010W | MET3 | 0.84326403 | 0.98519209 | -0.0268491 | 126.4203827 | 241.7002725 |
| YIL099W | SGA1 | 0.88893169 | 0.98910891 | -0.0266899 | 113.0660362 | 216.6264208 |
| YNL223W | ATG4 | 0.90957094 | 0.98910891 | -0.0266303 | 87.19332099 | 168.0908956 |
| YPR083W | MDM36 | 0.86400685 | 0.98660791 | -0.0265903 | 97.04011386 | 186.5521004 |
| YOR229W | WTM2 | 0.88289954 | 0.98910891 | -0.0264962 | 112.4044534 | 215.3524505 |
| YDL187C | YDL187C | 0.89407139 | 0.98910891 | -0.0264133 | 116.1011786 | 222.2716251 |
| YEL065W | SIT1 | 0.80961997 | 0.9824616 | -0.0262687 | 126.0674096 | 240.938958 |
| YDL230W | PTP1 | 0.90047826 | 0.98910891 | -0.0261909 | 102.3638989 | 196.4687534 |
| YGL016W | KAP122 | 0.9488306 | 0.9925075 | -0.0261893 | 92.8359247 | 178.5983684 |
| YPR046W | MCM16 | 0.8005504 | 0.9824616 | -0.0261676 | 102.1674443 | 196.0962993 |
| YIR007W | YIR007W | 0.89997486 | 0.98910891 | -0.0260743 | 102.9360074 | 197.5218117 |
| YNL107W | YAF9 | 0.93026334 | 0.99106129 | -0.0259694 | 93.7995172 | 180.3680069 |
| YPR128C | ANT1 | 0.89549565 | 0.98910891 | -0.0259244 | 100.0388636 | 192.0624532 |
| YMR174C | PAI3 | 0.94283639 | 0.9913313 | -0.0258865 | 90.62846151 | 174.4063806 |
| YDR109C | YDR109C | 0.86394282 | 0.98660791 | -0.0258575 | 118.1331708 | 225.9876187 |
| YGR077C | PEX8 | 0.93960166 | 0.9913313 | -0.0257565 | 101.0658916 | 193.9599494 |
| YGR014W | MSB2 | 0.85825269 | 0.98660791 | -0.025403 | 115.9482074 | 221.8118555 |
| YFR054C | YFR054C | 0.86325293 | 0.98660791 | -0.0253056 | 130.6538553 | 249.3762356 |
| YBL056W | PTC3 | 0.87893419 | 0.98910891 | -0.0249504 | 117.6429199 | 224.9129217 |
| YLR334C | YLR334C | 0.92885823 | 0.99106129 | -0.0248934 | 76.77244598 | 148.2489418 |
| YPR196W | YPR196W | 0.85978961 | 0.98660791 | -0.0248289 | 98.9005329 | 189.7400386 |
| YEL008W | YEL008W | 0.83102738 | 0.9824616 | -0.0247867 | 107.070172 | 205.0553014 |
| YIR004W | DJP1 | 0.89069159 | 0.98910891 | -0.0247559 | 100.3682399 | 192.4802854 |
| YDR393W | SHE9 | 0.94142309 | 0.9913313 | -0.0245736 | 107.3180034 | 205.4836565 |
| YDR209C | YDR209C | 0.86767259 | 0.98696321 | -0.0244595 | 114.5122402 | 218.9572177 |
| YCR085W | YCR085W | 0.92920509 | 0.99106129 | -0.0244097 | 106.6700194 | 204.2403029 |
| YBR054W | YRO2 | 0.90569079 | 0.98910891 | -0.0242832 | 124.2515713 | 237.1935753 |
| YLR126C | YLR126C | 0.850024 | 0.98660791 | -0.0242788 | 105.247581 | 201.5500645 |
| YER004W | FMP52 | 0.87957752 | 0.98910891 | -0.0242661 | 105.5093432 | 202.0388393 |
| YER130C | YER130C | 0.84701518 | 0.98660791 | -0.0242527 | 110.4104604 | 211.228788 |
| YER028C | MIG3 | 0.89497867 | 0.98910891 | -0.0241186 | 127.2275752 | 242.7470217 |
| YHL021C | FMP12 | 0.93484153 | 0.9913313 | -0.0239982 | 110.2930227 | 210.9649897 |
| YDL240W | LRG1 | 0.9093776 | 0.98910891 | -0.0238452 | 128.601869 | 245.2777957 |
| YNL229C | URE2 | 0.84749186 | 0.98660791 | -0.0235566 | 107.2389114 | 205.1613128 |
| YHR008C | SOD2 | 0.85307146 | 0.98660791 | -0.0233986 | 91.07998324 | 174.8275564 |
| YOR303W | CPA1 | 0.9061245 | 0.98910891 | -0.0233013 | 113.9987725 | 217.796034 |
| YPL248C | GAL4 | 0.91170606 | 0.98910891 | -0.0229554 | 118.9972489 | 227.1116966 |
| YHL014C | YLF2 | 0.95622343 | 0.99380889 | -0.022562 | 85.92828285 | 165.0221989 |
| YER158C | YER158C | 0.81176185 | 0.9824616 | -0.0224598 | 107.5202147 | 205.5012559 |
| YGL177W | YGL177W | 0.85183979 | 0.98660791 | -0.0224412 | 116.2810068 | 221.9292928 |
| YCR099C | YCR099C | 0.89395565 | 0.98910891 | -0.0224194 | 127.6137097 | 243.1805156 |
| YAL046C | YAL046C | 0.88297493 | 0.98910891 | -0.0224124 | 102.2460378 | 195.6012081 |
| YHR109W | CTM1 | 0.88764837 | 0.98910891 | -0.0223691 | 124.3241635 | 237.0022293 |
| YPR065W | ROX1 | 0.8748018 | 0.98901687 | -0.022312 | 99.24337567 | 189.9524258 |
| YOR223W | YOR223W | 0.91579987 | 0.98910891 | -0.0222077 | 110.0805467 | 210.2601337 |
| YIL148W | RPL40A | 0.9092004 | 0.98910891 | -0.0221471 | 112.5713078 | 214.9212844 |
| YER039C-A | YER039C-A | 0.83958302 | 0.98484693 | -0.0218411 | 109.3380357 | 208.8047926 |
| YDR135C | YCF1 | 0.88870947 | 0.98910891 | -0.0217078 | 112.2685062 | 214.278214 |
| YBR219C | YBR219C | 0.86451991 | 0.98660791 | -0.0216319 | 120.3810675 | 229.4806623 |
| YJR119C | JHD2 | 0.87989896 | 0.98910891 | -0.0216076 | 103.2832095 | 197.4087845 |
| YBR016W | YBR016W | 0.90217617 | 0.98910891 | -0.0215039 | 119.4080875 | 227.6338964 |
| YLR096W | KIN2 | 0.85988406 | 0.98660791 | -0.0214763 | 101.126422 | 193.3411638 |
| YGL056C | SDS23 | 0.78547078 | 0.97903138 | -0.0213429 | 121.7907944 | 232.075218 |
| YBR108W | YBR108W | 0.86739383 | 0.98696321 | -0.021291 | 111.3137421 | 212.4162024 |

| | | | | | | |
|---|---|---|---|---|---|---|
| YDR019C | GCV1 | 0.91786682 | 0.98910891 | -0.0211584 | 109.4505514 | 208.8990146 |
| YJR008W | YJR008W | 0.89722658 | 0.98910891 | -0.0211458 | 115.7530988 | 220.7175448 |
| YDR275W | BSC2 | 0.9089146 | 0.98910891 | -0.0211216 | 109.9701166 | 209.8671917 |
| YIR027C | DAL1 | 0.90543766 | 0.98910891 | -0.0210119 | 113.8303371 | 217.0884271 |
| YER081W | SER3 | 0.86409776 | 0.98660791 | -0.020795 | 102.4710702 | 195.7465527 |
| YGR248W | SOL4 | 0.91641125 | 0.98910891 | -0.0207851 | 92.59816784 | 177.2278216 |
| YPR115W | YPR115W | 0.91279502 | 0.98910891 | -0.0207688 | 119.9152507 | 228.4593357 |
| YMR179W | SPT21 | 0.95301975 | 0.99380889 | -0.0207151 | 80.79118912 | 155.0713812 |
| YER163C | YER163C | 0.915252 | 0.98910891 | -0.0206638 | 119.8758322 | 228.367434 |
| YOL036W | YOL036W | 0.88781332 | 0.98910891 | -0.0202573 | 113.1571327 | 215.6966998 |
| YML012W | ERV25 | 0.90217887 | 0.98910891 | -0.0201068 | 117.4353425 | 223.694892 |
| YJR092W | BUD4 | 0.89246 | 0.98910891 | -0.0197581 | 105.8679167 | 201.9400601 |
| YDR008C | YDR008C | 0.91032698 | 0.98910891 | -0.019754 | 115.6430591 | 220.2730419 |
| YLR265C | NEJ1 | 0.88133816 | 0.98910891 | -0.0196653 | 102.2576119 | 195.1529086 |
| YDR317W | HIM1 | 0.9118803 | 0.98910891 | -0.0196327 | 116.2062251 | 221.3085155 |
| YLR303W | MET17 | 0.90105548 | 0.98910891 | -0.0194696 | 107.3589106 | 204.6871244 |
| YDR095C | YDR095C | 0.90613522 | 0.98910891 | -0.019366 | 130.1939638 | 247.4974592 |
| YNL053W | MSG5 | 0.88699582 | 0.98910891 | -0.0192127 | 98.77228121 | 188.5385892 |
| YBR298C | MAL31 | 0.88033642 | 0.98910891 | -0.0190399 | 104.3221635 | 198.9180653 |
| YLR023C | IZH3 | 0.91632213 | 0.98910891 | -0.018941 | 101.9727142 | 194.4946522 |
| YER001W | MNN1 | 0.86668274 | 0.98696321 | -0.0187995 | 113.1523388 | 215.4382731 |
| YJL066C | MPM1 | 0.9224068 | 0.98998274 | -0.0187767 | 119.3489545 | 227.0563908 |
| YNL147W | LSM7 | 0.92704646 | 0.99106129 | -0.018642 | 69.19034146 | 132.9588171 |
| YEL048C | YEL048C | 0.88862755 | 0.98910891 | -0.0185466 | 109.818789 | 209.1428049 |
| YLR035C | MLH2 | 0.9156589 | 0.98910891 | -0.0185427 | 104.4078318 | 198.9936695 |
| YNL283C | WSC2 | 0.89613493 | 0.98910891 | -0.0185393 | 107.5800052 | 204.9426323 |
| YDR514C | YDR514C | 0.8494261 | 0.98660791 | -0.0184785 | 115.7519845 | 220.2591051 |
| YCL045C | YCL045C | 0.89808474 | 0.98910891 | -0.0184147 | 121.8699925 | 231.722759 |
| YBR246W | YBR246W | 0.96082998 | 0.99488882 | -0.018385 | 91.43058908 | 174.6273349 |
| YBR062C | YBR062C | 0.90441934 | 0.98910891 | -0.0182339 | 114.542757 | 217.9492965 |
| YOL132W | GAS4 | 0.92020621 | 0.98998274 | -0.0180301 | 113.8319634 | 216.5813014 |
| YLR058C | SHM2 | 0.92063433 | 0.98998274 | -0.0180111 | 109.2444609 | 207.9740143 |
| YGR084C | MRP13 | 0.89487719 | 0.98910891 | -0.0179362 | 118.2487624 | 224.8491232 |
| YGL136C | MRM2 | 0.96715602 | 0.99631757 | -0.0178825 | 104.5757242 | 199.1955987 |
| YBR036C | CSG2 | 0.96015121 | 0.99466569 | -0.0178105 | 86.23846563 | 164.7910142 |
| YPR091C | YPR091C | 0.90851419 | 0.98910891 | -0.0175929 | 99.30967873 | 189.2693714 |
| YPR118W | YPR118W | 0.92112008 | 0.98998274 | -0.0175514 | 104.3549326 | 198.724854 |
| YPL264C | YPL264C | 0.95320748 | 0.99380889 | -0.0173727 | 103.9718863 | 197.9758519 |
| YDR509W | YDR509W | 0.89576355 | 0.98910891 | -0.0171369 | 112.8515708 | 214.5897121 |
| YPL039W | YPL039W | 0.93242193 | 0.99106129 | -0.0171267 | 112.6250781 | 214.163184 |
| YOR027W | STI1 | 0.91319218 | 0.98910891 | -0.0171117 | 119.2583422 | 226.6015636 |
| YLL053C | YLL053C | 0.88844147 | 0.98910891 | -0.0166501 | 90.46049412 | 172.5110632 |
| YGR025W | YGR025W | 0.92458165 | 0.99105182 | -0.0164674 | 113.5614023 | 215.8064815 |
| YOL065C | INP54 | 0.93356986 | 0.99106129 | -0.0163826 | 117.6228955 | 223.4094734 |
| YHR077C | NMD2 | 0.85021566 | 0.98660791 | -0.016056 | 104.5467997 | 198.82884 |
| YOR356W | YOR356W | 0.91021589 | 0.98910891 | -0.0160138 | 109.0509534 | 207.269349 |
| YGR027C | RPS25A | 0.95034742 | 0.99265187 | -0.0158498 | 90.54741886 | 172.5371603 |
| YBR134W | YBR134W | 0.92226295 | 0.98998274 | -0.0155816 | 115.5218785 | 219.3318814 |
| YOL131W | YOL131W | 0.94630213 | 0.99208511 | -0.0153137 | 114.3735727 | 217.1323474 |
| YOR137C | SIA1 | 0.9259143 | 0.99106129 | -0.0153047 | 125.1976185 | 237.4317487 |
| YLR214W | FRE1 | 0.92551759 | 0.99106129 | -0.0149708 | 87.42151038 | 166.5240002 |
| YJL150W | YJL150W | 0.92514054 | 0.99106129 | -0.0148679 | 112.404826 | 213.3636119 |
| YPR004C | YPR004C | 0.92333807 | 0.99064476 | -0.0147933 | 101.9405654 | 193.7247147 |
| YPL273W | SAM4 | 0.92654872 | 0.99106129 | -0.0147776 | 98.02745891 | 186.3828401 |
| YHR116W | COX23 | 0.9640233 | 0.9955546 | -0.0146477 | 77.98296171 | 148.7663347 |
| YBR172C | SMY2 | 0.9738315 | 0.99677322 | -0.0144144 | 103.1669487 | 195.9600168 |
| YER118C | SHO1 | 0.91686144 | 0.98910891 | -0.0142048 | 108.3830709 | 205.7072049 |
| YIR013C | GAT4 | 0.93286876 | 0.99106129 | -0.0142025 | 115.4998417 | 219.0546035 |
| YJL186W | MNN5 | 0.94601814 | 0.99208511 | -0.0140845 | 128.0186582 | 242.513954 |
| YGR003W | CUL3 | 0.91446502 | 0.98910891 | -0.0139801 | 120.7682758 | 228.8977016 |
| YLR108C | YLR108C | 0.92958345 | 0.99106129 | -0.0139728 | 110.4566516 | 209.5565994 |
| YCR083W | TRX3 | 0.92689613 | 0.99106129 | -0.0138015 | 117.7256526 | 223.1605918 |
| YBR033W | EDS1 | 0.90929982 | 0.98910891 | -0.013781 | 110.3272967 | 209.2811662 |
| YJL116C | NCA3 | 0.94501229 | 0.99201598 | -0.0137669 | 132.5435746 | 250.9462898 |

| | | | | | | |
|---|---|---|---|---|---|---|
| YBR208C | DUR1 | 0.9417709 | 0.9913313 | -0.0135854 | 128.115582 | 242.6103558 |
| YIL168W | YIL168W | 0.94110577 | 0.9913313 | -0.0134668 | 96.6495281 | 183.5741961 |
| YHL046C | PAU13 | 0.94313319 | 0.9913313 | -0.0133566 | 93.83624849 | 178.278921 |
| YLR290C | YLR290C | 0.9695975 | 0.99631757 | -0.0131576 | 86.27256057 | 164.058867 |
| YER072W | VTC1 | 0.87931085 | 0.98910891 | -0.0131194 | 115.157657 | 218.2275098 |
| YLR351C | NIT3 | 0.93622983 | 0.9913313 | -0.0130867 | 103.9876672 | 197.2721491 |
| YJR117W | STE24 | 0.93153256 | 0.99106129 | -0.0130824 | 110.6975992 | 209.8561511 |
| YJL216C | YJL216C | 0.93106139 | 0.99106129 | -0.0130711 | 110.5050743 | 209.4931367 |
| YEL066W | HPA3 | 0.93138132 | 0.99106129 | -0.0126998 | 117.0167047 | 221.6424316 |
| YGR153W | YGR153W | 0.94974693 | 0.9925075 | -0.0126888 | 119.3958782 | 226.1027849 |
| YGR255C | COQ6 | 0.95611499 | 0.99380889 | -0.0123852 | 99.34578242 | 188.4460811 |
| YIL035C | CKA1 | 0.97799578 | 0.99677322 | -0.0123093 | 77.77235442 | 147.9712448 |
| YKR017C | YKR017C | 0.91460776 | 0.98910891 | -0.0122335 | 98.42654308 | 186.6960536 |
| YBR131W | CCZ1 | 0.98211087 | 0.99677322 | -0.0121812 | 39.47972276 | 76.12994522 |
| YHR139C | SPS100 | 0.93222592 | 0.99106129 | -0.0120725 | 113.3666497 | 214.6892798 |
| YER161C | SPT2 | 0.88530986 | 0.98910891 | -0.0120351 | 100.9083637 | 191.3168648 |
| YNL045W | YNL045W | 0.9713748 | 0.9966974 | -0.0120032 | 82.49962748 | 156.785071 |
| YGL144C | ROG1 | 0.89697254 | 0.98910891 | -0.0119432 | 135.786124 | 256.7157972 |
| YOR315W | SFG1 | 0.94684657 | 0.99213255 | -0.0118101 | 121.1153119 | 229.1773194 |
| YLR348C | DIC1 | 0.9334872 | 0.99106129 | -0.0116533 | 109.4023512 | 207.1823617 |
| YDR340W | YDR340W | 0.95125858 | 0.99287903 | -0.0116307 | 120.4950384 | 227.9832704 |
| YPL027W | SMA1 | 0.95687291 | 0.99380889 | -0.0115726 | 125.5537181 | 237.4610846 |
| YEL064C | AVT2 | 0.88968448 | 0.98910891 | -0.0114864 | 112.5250181 | 213.0104963 |
| YLR312C | YLR312C | 0.94844999 | 0.9925075 | -0.0113904 | 107.2886144 | 203.1729839 |
| YEL030W | ECM10 | 0.93155662 | 0.99106129 | -0.0113236 | 112.0451808 | 212.0826888 |
| YBL048W | YBL048W | 0.93944543 | 0.9913313 | -0.011307 | 128.672265 | 243.264619 |
| YBR064W | YBR064W | 0.93659697 | 0.9913313 | -0.0111774 | 118.2078085 | 223.6159189 |
| YNR033W | ABZ1 | 0.9319431 | 0.99106129 | -0.0111665 | 101.3152966 | 191.9314641 |
| YLR306W | UBC12 | 0.93247486 | 0.99106129 | -0.0110744 | 99.37691706 | 188.2801946 |
| YDR352W | YDR352W | 0.94331153 | 0.9913313 | -0.0110101 | 114.3964243 | 216.4388993 |
| YDR516C | EMI2 | 0.9413333 | 0.9913313 | -0.0105184 | 108.1071114 | 204.5589128 |
| YBL095W | YBL095W | 0.94317727 | 0.9913313 | -0.0103166 | 115.3416098 | 218.09297 |
| YDL197C | ASF2 | 0.94390503 | 0.99161193 | -0.0102638 | 115.3603603 | 218.1191169 |
| YDR336W | YDR336W | 0.95954832 | 0.99466569 | -0.0101877 | 117.9205621 | 222.9078527 |
| YLR320W | MMS22 | 0.9558302 | 0.99380889 | -0.010127 | 64.83338891 | 123.3303111 |
| YFL027C | GYP8 | 0.94511816 | 0.99201598 | -0.0099471 | 104.9508262 | 198.5414265 |
| YDR482C | CWC21 | 0.90010565 | 0.98910891 | -0.0098524 | 114.5278709 | 216.4873523 |
| YNL134C | YNL134C | 0.97344146 | 0.99677322 | -0.0095665 | 94.33152619 | 178.559375 |
| YGR203W | YGR203W | 0.94083861 | 0.9913313 | -0.009532 | 126.2539013 | 238.4251724 |
| YPL261C | YPL261C | 0.92950506 | 0.99106129 | -0.0094841 | 92.57112967 | 175.2435779 |
| YJR015W | YJR015W | 0.96964811 | 0.99631757 | -0.0092564 | 115.029478 | 217.3261597 |
| YLR446W | YLR446W | 0.97893027 | 0.99677322 | -0.0091448 | 89.07832805 | 168.6346422 |
| YJL089W | SIP4 | 0.96456477 | 0.99583115 | -0.0091169 | 126.133809 | 238.1289256 |
| YDL213C | NOP6 | 0.95918317 | 0.99466569 | -0.0090876 | 120.1336754 | 226.8704223 |
| YOL070C | NBA1 | 0.95387889 | 0.99380889 | -0.0089626 | 112.4604703 | 212.4576248 |
| YPR075C | OPY2 | 0.93546126 | 0.9913313 | -0.0088802 | 95.9608095 | 181.4977359 |
| YMR292W | GOT1 | 0.94115828 | 0.9913313 | -0.0088629 | 94.46773971 | 178.6944615 |
| YLR114C | AVL9 | 0.9813463 | 0.99677322 | -0.0086401 | 90.74296627 | 171.6703876 |
| YML112W | CTK3 | 0.94139077 | 0.9913313 | -0.0086166 | 103.3019887 | 195.2213242 |
| YML090W | YML090W | 0.98158755 | 0.99677322 | -0.0084152 | 89.56624928 | 169.4249229 |
| YER152C | YER152C | 0.95415503 | 0.99380889 | -0.0084119 | 120.9383087 | 228.2639364 |
| YBL037W | APL3 | 0.95021674 | 0.99265187 | -0.0083182 | 132.7957324 | 250.4869772 |
| YPR005C | HAL1 | 0.9636361 | 0.9955546 | -0.0082912 | 103.646611 | 195.8120051 |
| YDR260C | SWM1 | 0.96043603 | 0.99472077 | -0.0081768 | 117.8749441 | 222.4782379 |
| YOR375C | GDH1 | 0.96543624 | 0.99605556 | -0.0080156 | 110.098135 | 207.8649451 |
| YMR103C | YMR103C | 0.98644929 | 0.99677322 | -0.0079407 | 98.94219877 | 186.9287071 |
| YNL314W | DAL82 | 0.95363649 | 0.99380889 | -0.0079264 | 109.3468676 | 206.4406402 |
| YEL060C | PRB1 | 0.92438943 | 0.99105182 | -0.0076581 | 106.3292335 | 200.7350481 |
| YIL163C | YIL163C | 0.97351304 | 0.99677322 | -0.0075825 | 126.0857759 | 237.7763019 |
| YPR074C | TKL1 | 0.97047365 | 0.99646434 | -0.0075213 | 99.56200758 | 188.0194283 |
| YER044C-A | MEI4 | 0.94917871 | 0.9925075 | -0.0069437 | 106.7870457 | 201.4714575 |
| YLR030W | YLR030W | 0.95684299 | 0.99380889 | -0.006889 | 101.4025163 | 191.3631918 |
| YOL011W | PLB3 | 0.96009629 | 0.99466569 | -0.0067196 | 108.6895405 | 205.0013333 |
| YLR110C | CCW12 | 0.93817062 | 0.9913313 | -0.0066456 | 100.0793595 | 188.8399183 |

| | | | | | | |
|---|---|---|---|---|---|---|
| YOR045W | TOM6 | 0.95440552 | 0.99380889 | -0.0065414 | 104.2679247 | 196.6779121 |
| YER054C | GIP2 | 0.95114476 | 0.99287903 | -0.0065168 | 124.4342972 | 234.4965662 |
| YGR142W | BTN2 | 0.96594443 | 0.9961012 | -0.0064139 | 121.2259126 | 228.4614967 |
| YHR047C | AAP1 | 0.95629998 | 0.99380889 | -0.0063395 | 107.8470471 | 203.3561556 |
| YDR242W | AMD2 | 0.96625725 | 0.99618455 | -0.0061777 | 97.36455375 | 183.6681367 |
| YLR206W | ENT2 | 0.96346433 | 0.9955546 | -0.0060113 | 96.19668238 | 181.4492836 |
| YPL208W | RKM1 | 0.9778262 | 0.99677322 | -0.0059618 | 92.35309214 | 174.2320004 |
| YIL124W | AYR1 | 0.97649029 | 0.99677322 | -0.0058513 | 104.2929728 | 196.6068219 |
| YLR342W | FKS1 | 0.98571592 | 0.99677322 | -0.0057417 | 75.75602151 | 143.0658564 |
| YBR245C | ISW1 | 0.96118642 | 0.99501796 | -0.005727 | 125.8460594 | 237.0092392 |
| YLR241W | YLR241W | 0.96879234 | 0.99631757 | -0.0056316 | 102.985586 | 194.117175 |
| YGR284C | ERV29 | 0.97203479 | 0.99677322 | -0.0055217 | 106.2476575 | 200.2165247 |
| YIL153W | RRD1 | 0.98855799 | 0.9971501 | -0.0054941 | 93.07158362 | 175.4995387 |
| YDR161W | YDR161W | 0.97680003 | 0.99677322 | -0.0054071 | 119.9769725 | 225.9468057 |
| YBR284W | YBR284W | 0.98889701 | 0.99725781 | -0.0053361 | 106.0689992 | 199.8496772 |
| YNL214W | PEX17 | 0.9703381 | 0.99646434 | -0.0052569 | 95.61339189 | 180.2262163 |
| YBR281C | DUG2 | 0.98095512 | 0.99677322 | -0.0052036 | 100.0733389 | 188.5819048 |
| YER140W | YER140W | 0.9600918 | 0.99466569 | -0.0052032 | 112.6044509 | 212.0844456 |
| YLL002W | RTT109 | 0.9814736 | 0.99677322 | -0.0051128 | 22.57356978 | 43.21241875 |
| YNR041C | COQ2 | 0.98535821 | 0.99677322 | -0.0049293 | 67.45366067 | 127.355455 |
| YPR037C | ERV2 | 0.97480954 | 0.99677322 | -0.0045863 | 105.1748691 | 198.0444278 |
| YML010C-B | YML010C-B | 0.98692378 | 0.99677322 | -0.0043134 | 83.3448932 | 157.05472 |
| YJL169W | YJL169W | 0.98116633 | 0.99677322 | -0.0040644 | 103.9461937 | 195.6507011 |
| YBR258C | SHG1 | 0.9839106 | 0.99677322 | -0.0040192 | 132.2221939 | 248.6757508 |
| YER144C | UBP5 | 0.97257425 | 0.99677322 | -0.0038347 | 112.6093206 | 211.8594398 |
| YML099C | ARG81 | 0.97497857 | 0.99677322 | -0.0037903 | 112.5527944 | 211.745827 |
| YGR202C | PCT1 | 0.98361862 | 0.99677322 | -0.0037749 | 112.6442999 | 211.9148201 |
| YLR313C | SPH1 | 0.98225712 | 0.99677322 | -0.0037355 | 104.6821351 | 196.974709 |
| YIL044C | AGE2 | 0.98965999 | 0.99755868 | -0.0034678 | 89.56740233 | 168.5806188 |
| YDR389W | SAC7 | 0.98029402 | 0.99677322 | -0.0033227 | 105.6827243 | 198.7807396 |
| YDR535C | YDR535C | 0.98094068 | 0.99677322 | -0.0032575 | 116.647268 | 219.3340245 |
| YDR046C | BAP3 | 0.97894177 | 0.99677322 | -0.0032361 | 95.67789398 | 180.0014426 |
| YGR139W | YGR139W | 0.9823347 | 0.99677322 | -0.0032275 | 120.3971604 | 226.361963 |
| YKL073W | LHS1 | 0.98157648 | 0.99677322 | -0.0032144 | 99.67643466 | 187.4971664 |
| YLR043C | TRX1 | 0.9846664 | 0.99677322 | -0.0031511 | 111.0736806 | 208.8623288 |
| YOL136C | PFK27 | 0.98677304 | 0.99677322 | -0.0030977 | 119.0122339 | 223.7422751 |
| YNR073C | YNR073C | 0.98738524 | 0.99677322 | -0.0027707 | 117.9445792 | 221.6838957 |
| YFL044C | OTU1 | 0.9820454 | 0.99677322 | -0.0025021 | 109.6919475 | 206.1598005 |
| YDR494W | RSM28 | 0.9746177 | 0.99677322 | -0.0024119 | 113.7051311 | 213.6712434 |
| YPR188C | MLC2 | 0.98738492 | 0.99677322 | -0.0022299 | 98.19594059 | 184.5519979 |
| YHR155W | YSP1 | 0.99349519 | 0.99927115 | -0.0020736 | 119.4021425 | 224.2983478 |
| YDR165W | TRM82 | 0.99099161 | 0.99819798 | -0.0018665 | 114.0204635 | 214.1693458 |
| YGL197W | MDS3 | 0.99074078 | 0.99819798 | -0.0017928 | 125.0982347 | 234.9335546 |
| YOR154W | SLP1 | 0.98623201 | 0.99677322 | -0.0017368 | 110.8092273 | 208.1243601 |
| YER176W | ECM32 | 0.9962841 | 0.99957465 | -0.0015134 | 101.7202926 | 191.0394753 |
| YGL081W | YGL081W | 0.99174581 | 0.99872338 | -0.0012767 | 128.5550314 | 241.3286114 |
| YGL028C | SCW11 | 0.99436148 | 0.99927115 | -0.0009024 | 126.5040916 | 237.4179512 |
| YIL108W | YIL108W | 0.99717118 | 0.99957465 | -0.0005819 | 119.308107 | 223.866751 |
| YJL045W | YJL045W | 0.99755092 | 0.99957465 | -0.0005392 | 113.1452045 | 212.3006697 |
| YER186C | YER186C | 0.99706111 | 0.99957465 | -0.000451 | 107.8793312 | 202.4092266 |
| YDR379W | RGA2 | 0.99887079 | 0.99987123 | -0.0002697 | 118.8067409 | 222.8730128 |
| YML020W | YML020W | 0.99786468 | 0.99957465 | -0.000266 | 102.2129611 | 191.7500746 |
| YDR010C | YDR010C | 0.99925756 | 0.99987123 | -0.0001634 | 117.0289022 | 219.5204181 |
| YFR055W | IRC7 | 0.99931514 | 0.99987123 | -9.45E-05 | 117.4473032 | 220.2933545 |
| YNL236W | SIN4 | 1 | 1 | 0 | 0 | 0 |
| YOR139C | YOR139C | 1 | 1 | 0 | 0 | 0 |
| YCR050C | YCR050C | 0.99940553 | 0.99987123 | 9.71E-05 | 108.6597385 | 203.7791424 |
| YDR144C | MKC7 | 0.99817795 | 0.99957465 | 0.00033368 | 115.5881946 | 216.7332588 |
| YPL071C | YPL071C | 0.99798576 | 0.99957465 | 0.00035025 | 108.8798333 | 204.1486198 |
| YNR014W | YNR014W | 0.99801371 | 0.99957465 | 0.00045298 | 101.5077248 | 190.3043546 |
| YGR122C-A | YGR122C-A | 0.99564152 | 0.99941197 | 0.00060034 | 104.9201365 | 196.6792587 |
| YML048W | GSF2 | 0.99376333 | 0.99927115 | 0.00074058 | 104.1687314 | 195.2459739 |
| YER002W | NOP16 | 0.99399901 | 0.99927115 | 0.00083353 | 101.9080452 | 190.990063 |
| YLR294C | YLR294C | 0.99568803 | 0.99941197 | 0.00086036 | 102.1038275 | 191.352669 |

| | | | | | | |
|---|---|---|---|---|---|---|
| YGR041W | BUD9 | 0.99550643 | 0.99941197 | 0.0010255 | 125.1934613 | 234.6299596 |
| YPR156C | TPO3 | 0.99501271 | 0.99941197 | 0.00103488 | 98.37337923 | 184.3262033 |
| YDR273W | DON1 | 0.99790407 | 0.99957465 | 0.00107644 | 118.5307757 | 222.1251073 |
| YOR173W | DCS2 | 0.9976365 | 0.99957465 | 0.00111635 | 75.9667366 | 142.2876965 |
| YPR125W | YLH47 | 0.99468265 | 0.99933723 | 0.00112636 | 110.5945372 | 207.2318285 |
| YDR338C | YDR338C | 0.99395571 | 0.99927115 | 0.00127877 | 119.0599169 | 223.0829154 |
| YBR223C | TDP1 | 0.99438417 | 0.99927115 | 0.00132542 | 120.153796 | 225.1265495 |
| YOL028C | YAP7 | 0.9933608 | 0.99927115 | 0.00161881 | 123.9057488 | 232.1132914 |
| YAL030W | SNC1 | 0.99403373 | 0.99927115 | 0.00173651 | 125.873936 | 235.7845693 |
| YHR046C | INM1 | 0.99079658 | 0.99819798 | 0.00190174 | 118.8834619 | 222.6453804 |
| YIL095W | PRK1 | 0.98703301 | 0.99677322 | 0.00232845 | 116.7414892 | 218.5550166 |
| YPR092W | YPR092W | 0.98659647 | 0.99677322 | 0.0025452 | 109.5281851 | 204.9890887 |
| YER067W | YER067W | 0.98332789 | 0.99677322 | 0.00260322 | 106.5059703 | 199.3108737 |
| YDR501W | PLM2 | 0.98677103 | 0.99677322 | 0.00262238 | 126.6144251 | 237.021819 |
| YDR229W | IVY1 | 0.98342826 | 0.99677322 | 0.00266515 | 118.7813984 | 222.3233421 |
| YOL037C | YOL037C | 0.98834593 | 0.9971501 | 0.00278314 | 116.0670009 | 217.2121918 |
| YFL011W | HXT10 | 0.9816036 | 0.99677322 | 0.00281403 | 112.265822 | 210.0776414 |
| YCR073C | SSK22 | 0.98349806 | 0.99677322 | 0.0028246 | 123.5443684 | 231.2292051 |
| YLR327C | TMA10 | 0.98799034 | 0.99704595 | 0.00297797 | 105.7344678 | 197.7997732 |
| YPR031W | NTO1 | 0.98298837 | 0.99677322 | 0.00310457 | 97.20320824 | 181.7773883 |
| YLR431C | ATG23 | 0.99291038 | 0.99927115 | 0.003152 | 95.37545261 | 178.3412449 |
| YFR015C | GSY1 | 0.97979576 | 0.99677322 | 0.00319536 | 120.1153023 | 224.7344174 |
| YCR061W | YCR061W | 0.98960209 | 0.99755868 | 0.00322318 | 94.77172112 | 177.1967434 |
| YDR157W | YDR157W | 0.98709888 | 0.99677322 | 0.0032262 | 115.1691023 | 215.4523418 |
| YBR188C | NTC20 | 0.98130041 | 0.99677322 | 0.00342312 | 118.732165 | 222.1013185 |
| YOR086C | TCB1 | 0.9861904 | 0.99677322 | 0.00345047 | 119.2053841 | 222.9841806 |
| YOL073C | YOL073C | 0.97799952 | 0.99677322 | 0.00362259 | 101.8871601 | 190.473701 |
| YOR135C | YOR135C | 0.98304698 | 0.99677322 | 0.00364659 | 113.9945671 | 213.1775283 |
| YOR134W | BAG7 | 0.98458804 | 0.99677322 | 0.00383721 | 115.1337993 | 215.2815893 |
| YKR043C | YKR043C | 0.97522692 | 0.99677322 | 0.00390711 | 101.4310172 | 189.569507 |
| YIR035C | YIR035C | 0.96984111 | 0.99631757 | 0.00444201 | 104.4605903 | 195.1600758 |
| YGL027C | CWH41 | 0.96986666 | 0.99631757 | 0.00455182 | 113.2419414 | 211.6110682 |
| YKL092C | BUD2 | 0.97439438 | 0.99677322 | 0.00462592 | 99.27845078 | 185.4092961 |
| YHR021C | RPS27B | 0.98674957 | 0.99677322 | 0.00475386 | 67.82043364 | 126.3866187 |
| YOL141W | PPM2 | 0.97548878 | 0.99677322 | 0.00502542 | 124.4631478 | 232.575866 |
| YOR188W | MSB1 | 0.98121468 | 0.99677322 | 0.00513451 | 125.3935393 | 234.3021883 |
| YBR067C | TIP1 | 0.97945395 | 0.99677322 | 0.00514204 | 122.5590525 | 228.9847047 |
| YGR182C | YGR182C | 0.97648771 | 0.99677322 | 0.00521648 | 115.6975178 | 216.1028828 |
| YDR276C | PMP3 | 0.95514789 | 0.99380889 | 0.00525644 | 107.6793401 | 201.0576271 |
| YOR327C | SNC2 | 0.97399326 | 0.99677322 | 0.00536059 | 101.7005157 | 189.8262809 |
| YDR351W | SBE2 | 0.97457763 | 0.99677322 | 0.00540116 | 120.8805476 | 225.792267 |
| YFR026C | YFR026C | 0.97101138 | 0.9966974 | 0.00542888 | 121.494323 | 226.9386858 |
| YLR349W | YLR349W | 0.97478797 | 0.99677322 | 0.0054322 | 111.1208962 | 207.4823339 |
| YLR094C | GIS3 | 0.97139698 | 0.9966974 | 0.00543367 | 113.9459667 | 212.7806176 |
| YJL128C | PBS2 | 0.98257776 | 0.99677322 | 0.00551314 | 128.128748 | 239.3674024 |
| YNL187W | YNL187W | 0.96911245 | 0.99631757 | 0.005562 | 107.0841517 | 199.8890475 |
| YPL138C | SPP1 | 0.96838507 | 0.99631757 | 0.00564314 | 93.45483912 | 174.3128363 |
| YPR174C | YPR174C | 0.96231577 | 0.99517478 | 0.00584205 | 110.8226754 | 206.8528868 |
| YDR304C | CPR5 | 0.97530124 | 0.99677322 | 0.00590898 | 119.7869944 | 223.6543795 |
| YDR525W-A | SNA2 | 0.94957939 | 0.9925075 | 0.00594499 | 112.6612605 | 210.2836146 |
| YNL218W | MGS1 | 0.98748795 | 0.99677322 | 0.00614886 | 92.63125729 | 172.6816509 |
| YBR098W | MMS4 | 0.96483885 | 0.99583115 | 0.00623937 | 114.4549982 | 213.5974763 |
| YGL166W | CUP2 | 0.96950419 | 0.99631757 | 0.00627014 | 129.1736509 | 241.197642 |
| YLR064W | YLR064W | 0.96808126 | 0.99631757 | 0.00629282 | 107.1253152 | 199.8412135 |
| YMR279C | YMR279C | 0.95895711 | 0.99466569 | 0.00641168 | 109.3504169 | 203.994146 |
| YER137C | YER137C | 0.9332714 | 0.99106129 | 0.00654295 | 104.67129 | 195.1957935 |
| YDR538W | PAD1 | 0.94734236 | 0.99214232 | 0.00684173 | 106.2676294 | 198.1386741 |
| YHR204W | MNL1 | 0.9593055 | 0.99466569 | 0.00705952 | 117.3811603 | 218.945289 |
| YGL002W | ERP6 | 0.96249671 | 0.99517478 | 0.00734239 | 108.8298538 | 202.8585703 |
| YMR306W | FKS3 | 0.96955036 | 0.99631757 | 0.00764055 | 125.582876 | 234.2285287 |
| YIL089W | YIL089W | 0.9862759 | 0.99677322 | 0.00770073 | 104.4643843 | 194.6096442 |
| YNL230C | ELA1 | 0.88061138 | 0.98910891 | 0.00777263 | 106.1817685 | 197.8183666 |
| YGR146C | YGR146C | 0.94754906 | 0.99214232 | 0.00782973 | 124.3099275 | 231.8086961 |
| YOR165W | SEY1 | 0.96145316 | 0.9950542 | 0.00794511 | 116.6566175 | 217.4348619 |

| | | | | | | |
|---|---|---|---|---|---|---|
| YJL153C | INO1 | 0.97808837 | 0.99677322 | 0.00889224 | 101.2788841 | 188.4312506 |
| YNL079C | TPM1 | 0.96814279 | 0.99631757 | 0.00912473 | 75.92988748 | 140.8484012 |
| YDR259C | YAP6 | 0.926595 | 0.99106129 | 0.00932889 | 116.2675949 | 216.4684766 |
| YJR009C | TDH2 | 0.95983566 | 0.99466569 | 0.00934384 | 109.7437551 | 204.2301927 |
| YER047C | SAP1 | 0.94712612 | 0.99214232 | 0.00936988 | 119.9549666 | 223.3772786 |
| YGR161C | RTS3 | 0.94149332 | 0.9913313 | 0.00943384 | 122.8716079 | 228.8366145 |
| YIL100W | YIL100W | 0.96329766 | 0.9955546 | 0.00959095 | 128.6077872 | 239.5681716 |
| YJL181W | YJL181W | 0.96399828 | 0.9955546 | 0.00962639 | 126.9443993 | 236.4423569 |
| YOR377W | ATF1 | 0.9563495 | 0.99380889 | 0.00976184 | 106.1104946 | 197.3443479 |
| YER115C | SPR6 | 0.87320308 | 0.98901687 | 0.00976262 | 105.7486588 | 196.6655768 |
| YLR136C | TIS11 | 0.95083465 | 0.99287903 | 0.00980969 | 107.4798125 | 199.9043724 |
| YDR435C | PPM1 | 0.91441142 | 0.98910891 | 0.00982343 | 94.03522601 | 174.6861544 |
| YLR142W | PUT1 | 0.96205478 | 0.99517478 | 0.01002301 | 110.3464969 | 205.2444588 |
| YBR027C | YBR027C | 0.93645357 | 0.9913313 | 0.01018115 | 121.326294 | 225.8104567 |
| YKR105C | YKR105C | 0.94633922 | 0.99208511 | 0.01019056 | 108.169728 | 201.133173 |
| YJL093C | TOK1 | 0.96652255 | 0.99621888 | 0.01053675 | 113.151123 | 210.4167512 |
| YEL016C | NPP2 | 0.90740324 | 0.98910891 | 0.01068222 | 103.7801078 | 192.8161426 |
| YDR107C | YDR107C | 0.94031998 | 0.9913313 | 0.01079493 | 116.2511592 | 216.1868206 |
| YHR095W | YHR095W | 0.95920181 | 0.99466569 | 0.0108675 | 124.7351313 | 232.0864395 |
| YCL064C | CHA1 | 0.93973678 | 0.9913313 | 0.01094386 | 122.0251342 | 226.9906641 |
| YLR261C | YLR261C | 0.9674172 | 0.99631757 | 0.01122737 | 67.24408368 | 124.1980743 |
| YPR192W | AQY1 | 0.91635403 | 0.98910891 | 0.01160513 | 105.7797025 | 196.408559 |
| YBR206W | YBR206W | 0.93328951 | 0.99106129 | 0.01180409 | 132.4411013 | 246.3790536 |
| YEL049W | PAU2 | 0.94141764 | 0.9913313 | 0.01181017 | 110.4034284 | 205.0454636 |
| YPR154W | PIN3 | 0.91504029 | 0.98910891 | 0.01183808 | 104.2088858 | 193.4225722 |
| YLR443W | ECM7 | 0.97546789 | 0.99677322 | 0.01191893 | 95.5698508 | 177.2058794 |
| YER184C | YER184C | 0.91927052 | 0.98955819 | 0.01211379 | 108.1594178 | 200.7847827 |
| YOR227W | YOR227W | 0.94587423 | 0.99208511 | 0.0125214 | 116.6759345 | 216.6881163 |
| YDR465C | RMT2 | 0.93159161 | 0.99106129 | 0.01252599 | 111.9527975 | 207.828896 |
| YBR040W | FIG1 | 0.93879053 | 0.9913313 | 0.01261813 | 127.7345096 | 237.4123723 |
| YDR130C | FIN1 | 0.93602765 | 0.9913313 | 0.0126608 | 115.8248181 | 215.0679626 |
| YMR194C-A | YMR194C-A | 0.94904183 | 0.9925075 | 0.01268834 | 104.194744 | 193.2505741 |
| YCR037C | PHO87 | 0.90036532 | 0.98910891 | 0.01275739 | 129.2848224 | 240.296221 |
| YIR043C | YIR043C | 0.94323416 | 0.9913313 | 0.0128204 | 118.172748 | 219.4442933 |
| YPL171C | OYE3 | 0.95700972 | 0.99380889 | 0.01312476 | 114.9600949 | 213.3667588 |
| YPL048W | CAM1 | 0.91349011 | 0.98910891 | 0.01322527 | 102.6478346 | 190.2574177 |
| YDR055W | PST1 | 0.93711219 | 0.9913313 | 0.01335327 | 120.8434426 | 224.3621184 |
| YBR007C | DSF2 | 0.92350978 | 0.99064476 | 0.01345712 | 110.0455259 | 204.0924203 |
| YOL126C | MDH2 | 0.93615171 | 0.9913313 | 0.01386253 | 119.7261019 | 222.1793694 |
| YLR011W | LOT6 | 0.93567877 | 0.9913313 | 0.01387659 | 108.0417248 | 200.2624415 |
| YPR020W | ATP20 | 0.91235064 | 0.98910891 | 0.01420949 | 96.72062795 | 178.9723067 |
| YNR057C | BIO4 | 0.9311734 | 0.99106129 | 0.01428004 | 121.4907872 | 225.4176761 |
| YBR026C | ETR1 | 0.96183212 | 0.99517478 | 0.01440508 | 96.2419031 | 178.0409744 |
| YHL012W | YHL012W | 0.97303911 | 0.99677322 | 0.01454966 | 96.42854141 | 178.366286 |
| YDR278C | YDR278C | 0.91400842 | 0.98910891 | 0.01467696 | 122.3687237 | 226.9963699 |
| YOR316C | COT1 | 0.94664536 | 0.99213255 | 0.01486729 | 130.3050542 | 241.8487165 |
| YJL123C | MTC1 | 0.9392418 | 0.9913313 | 0.01540016 | 126.8511387 | 235.2795874 |
| YBR047W | FMP23 | 0.94201556 | 0.9913313 | 0.01555973 | 130.3638664 | 241.8405486 |
| YDR031W | MIC14 | 0.93356851 | 0.99106129 | 0.0156196 | 111.6353163 | 206.7041486 |
| YOR162C | YRR1 | 0.93666107 | 0.9913313 | 0.01568508 | 120.4675804 | 223.2582166 |
| YIR019C | MUC1 | 0.7854698 | 0.97903138 | 0.01570228 | 106.3302018 | 196.7400465 |
| YIL071C | PCI8 | 0.92133823 | 0.98998274 | 0.01577841 | 130.9774267 | 242.9538902 |
| YGL114W | YGL114W | 0.89692376 | 0.98910891 | 0.01579419 | 125.2302227 | 232.1720766 |
| YBR058C | UBP14 | 0.89379857 | 0.98910891 | 0.01601812 | 118.4224568 | 219.3655232 |
| YML109W | ZDS2 | 0.91066618 | 0.98910891 | 0.01612691 | 121.8874353 | 225.8456165 |
| YKL136W | YKL136W | 0.87457464 | 0.98901687 | 0.01625474 | 104.7055386 | 193.5984016 |
| YER024W | YAT2 | 0.9283193 | 0.99106129 | 0.01629203 | 127.0795062 | 235.5553053 |
| YLR063W | YLR063W | 0.91848548 | 0.98910891 | 0.01668526 | 114.5023828 | 211.8991213 |
| YML054C | CYB2 | 0.86223366 | 0.98660791 | 0.0167531 | 117.8795243 | 218.2214801 |
| YOR005C | DNL4 | 0.95687842 | 0.99380889 | 0.01684051 | 111.5567497 | 206.3479049 |
| YMR170C | ALD2 | 0.89709082 | 0.98910891 | 0.01711774 | 107.8147633 | 199.282225 |
| YGR127W | YGR127W | 0.90977496 | 0.98910891 | 0.01716969 | 135.7999707 | 251.7607283 |
| YKR026C | GCN3 | 0.92812248 | 0.99106129 | 0.01721134 | 103.0327932 | 190.2974306 |
| YGR295C | COS6 | 0.83894483 | 0.98484693 | 0.01727651 | 110.5020619 | 204.2951985 |

| | | | | | | |
|---|---|---|---|---|---|---|
| YDR009W | GAL3 | 0.91835679 | 0.98910891 | 0.01733777 | 117.9824292 | 218.3144483 |
| YDR370C | YDR370C | 0.93805295 | 0.9913313 | 0.01736812 | 120.7425983 | 223.4860655 |
| YGR273C | YGR273C | 0.91509735 | 0.98910891 | 0.01766636 | 106.93483 | 197.5380079 |
| YOL006C | TOP1 | 0.95184797 | 0.99325277 | 0.01769739 | 53.02789373 | 96.42805239 |
| YOR052C | YOR052C | 0.92804391 | 0.99106129 | 0.01780838 | 126.676272 | 234.5395859 |
| YGL202W | ARO8 | 0.90190064 | 0.98910891 | 0.01785758 | 119.9571677 | 221.9292147 |
| YML100W-A | YML100W-A | 0.88691737 | 0.98910891 | 0.01788301 | 115.5418108 | 213.6436827 |
| YKL204W | EAP1 | 0.9545033 | 0.99380889 | 0.01840877 | 79.96166782 | 146.8217261 |
| YDR216W | ADR1 | 0.91809543 | 0.98910891 | 0.01858119 | 116.8743286 | 216.0234201 |
| YPR129W | SCD6 | 0.91862281 | 0.98910891 | 0.01869877 | 107.4248685 | 198.280457 |
| YJR036C | HUL4 | 0.93820824 | 0.9913313 | 0.01871582 | 120.9703921 | 223.6827187 |
| YCR105W | ADH7 | 0.8964486 | 0.98910891 | 0.01873877 | 120.4018521 | 222.612473 |
| YNR028W | CPR8 | 0.92238875 | 0.98998274 | 0.01874374 | 120.3311638 | 222.4790427 |
| YBR205W | KTR3 | 0.89559525 | 0.98910891 | 0.01897361 | 116.1995022 | 214.6906145 |
| YPR038W | YPR038W | 0.84035378 | 0.98484693 | 0.01901023 | 107.0217104 | 197.471029 |
| YDR089W | YDR089W | 0.91474364 | 0.98910891 | 0.01910558 | 120.8117088 | 223.318416 |
| YLR353W | BUD8 | 0.89796167 | 0.98910891 | 0.01911084 | 111.0392113 | 204.9888016 |
| YNL196C | YNL196C | 0.95502936 | 0.99380889 | 0.01920384 | 112.6652478 | 208.0225864 |
| YFL026W | STE2 | 0.85482767 | 0.98660791 | 0.01928115 | 108.3140194 | 199.8484542 |
| YBR099C | YBR099C | 0.9658291 | 0.9961012 | 0.01929529 | 110.6332366 | 204.1958201 |
| YEL025C | YEL025C | 0.87291782 | 0.98901687 | 0.01939414 | 128.2166528 | 237.1573181 |
| YKL109W | HAP4 | 0.8517456 | 0.98660791 | 0.01951351 | 79.92045056 | 146.5554085 |
| YHR134W | WSS1 | 0.94098502 | 0.9913313 | 0.01965582 | 91.60189299 | 168.4400782 |
| YDR459C | PFA5 | 0.88626993 | 0.98910891 | 0.01971262 | 108.1692977 | 199.5032 |
| YGL079W | YGL079W | 0.89242454 | 0.98910891 | 0.01975057 | 127.3350222 | 235.4428013 |
| YPR138C | MEP3 | 0.88518693 | 0.98910891 | 0.01985195 | 102.1235552 | 188.1403269 |
| YCL014W | BUD3 | 0.96852747 | 0.99631757 | 0.02021185 | 98.41319256 | 181.1198143 |
| YNL275W | BOR1 | 0.85841041 | 0.98660791 | 0.02032392 | 108.7133602 | 200.4190231 |
| YGL161C | YIP5 | 0.81925492 | 0.9824616 | 0.02034227 | 110.7306523 | 204.1993968 |
| YEL033W | MTC7 | 0.94817705 | 0.9925075 | 0.02050089 | 78.26185089 | 143.2757018 |
| YNL307C | MCK1 | 0.94974075 | 0.9925075 | 0.02068941 | 75.44699772 | 137.9640758 |
| YDR245W | MNN10 | 0.92589186 | 0.99106129 | 0.02102658 | 80.00670277 | 146.4583005 |
| YMR278W | YMR278W | 0.88195243 | 0.98910891 | 0.02114411 | 118.0555663 | 217.8003798 |
| YLR253W | YLR253W | 0.88378807 | 0.98910891 | 0.02118334 | 117.7631913 | 217.2453059 |
| YNL285W | YNL285W | 0.89525206 | 0.98910891 | 0.02147281 | 120.4987347 | 222.3264016 |
| YJL027C | YJL027C | 0.9113114 | 0.98910891 | 0.0216956 | 127.2786951 | 235.0043751 |
| YPR006C | ICL2 | 0.87069412 | 0.98870218 | 0.02175089 | 109.2465251 | 201.1748492 |
| YGR188C | BUB1 | 0.95656122 | 0.99380889 | 0.02196166 | 39.4332802 | 70.20121424 |
| YJR020W | YJR020W | 0.91043424 | 0.98910891 | 0.02203905 | 113.0675354 | 208.2920069 |
| YGL146C | YGL146C | 0.88950743 | 0.98910891 | 0.02211092 | 123.3670921 | 227.5969471 |
| YJL047C | RTT101 | 0.86647456 | 0.98696321 | 0.02234913 | 89.22826013 | 163.5274256 |
| YNL014W | HEF3 | 0.85151757 | 0.98660791 | 0.02235127 | 101.5458247 | 186.6291508 |
| YKL066W | YKL066W | 0.86522203 | 0.98677815 | 0.02245436 | 111.5666341 | 205.4059485 |
| YBL065W | YBL065W | 0.9043802 | 0.98910891 | 0.02249347 | 120.8100683 | 222.7356924 |
| YNL070W | TOM7 | 0.94449903 | 0.99185102 | 0.0226561 | 77.04009827 | 140.6155134 |
| YPR095C | SYT1 | 0.91068562 | 0.98910891 | 0.02266658 | 113.673579 | 209.3212993 |
| YGR269W | YGR269W | 0.90180095 | 0.98910891 | 0.02268935 | 106.0340238 | 194.989107 |
| YDR274C | YDR274C | 0.85713309 | 0.98660791 | 0.02302421 | 99.01033074 | 181.7585935 |
| YER038W-A | FMP49 | 0.87547501 | 0.98901687 | 0.02311264 | 109.7233835 | 201.8362287 |
| YOR059C | YOR059C | 0.89130553 | 0.98910891 | 0.02317737 | 126.0062133 | 232.3642619 |
| YFR049W | YMR31 | 0.85891381 | 0.98660791 | 0.0232332 | 118.3515826 | 217.9981389 |
| YBR032W | YBR032W | 0.96498682 | 0.99583115 | 0.02323579 | 110.5071059 | 203.2850617 |
| YBL028C | YBL028C | 0.90566167 | 0.98910891 | 0.02325969 | 127.427689 | 235.016214 |
| YBR183W | YPC1 | 0.9108327 | 0.98910891 | 0.02331905 | 126.5704802 | 233.3983279 |
| YLR050C | YLR050C | 0.87245371 | 0.98901687 | 0.02342851 | 104.5427222 | 192.0656453 |
| YLL058W | YLL058W | 0.8327044 | 0.9824616 | 0.02349687 | 105.9045055 | 194.6080287 |
| YJL064W | YJL064W | 0.91420751 | 0.98910891 | 0.02369905 | 116.7020027 | 214.8245816 |
| YBR203W | COS111 | 0.89239697 | 0.98910891 | 0.02370657 | 124.9425889 | 230.2788487 |
| YER142C | MAG1 | 0.77908717 | 0.97903138 | 0.02395054 | 114.9832401 | 211.5579434 |
| YOL035C | YOL035C | 0.87932901 | 0.98910891 | 0.02396419 | 105.0402982 | 192.9072183 |
| YKR007W | MEH1 | 0.86051415 | 0.98660791 | 0.02408637 | 97.65151658 | 179.0283538 |
| YOL087C | YOL087C | 0.89600947 | 0.98910891 | 0.02423423 | 121.060802 | 222.9081198 |
| YIR038C | GTT1 | 0.90264498 | 0.98910891 | 0.02441376 | 109.123496 | 200.488502 |
| YMR119W | ASI1 | 0.7377183 | 0.97861056 | 0.02443959 | 112.3004654 | 206.4426188 |

| | | | | | | |
|---|---|---|---|---|---|---|
| YNL108C | YNL108C | 0.954723 | 0.99380889 | 0.02444639 | 101.1200382 | 185.4721097 |
| YGR197C | SNG1 | 0.9072597 | 0.98910891 | 0.0245592 | 112.3376878 | 206.491965 |
| YGR151C | YGR151C | 0.86108546 | 0.98660791 | 0.02473708 | 117.5975323 | 216.3265826 |
| YBR074W | YBR074W | 0.89730517 | 0.98910891 | 0.024766 | 138.6739167 | 255.8512493 |
| YER132C | PMD1 | 0.86035446 | 0.98660791 | 0.02486884 | 119.2453167 | 219.3945265 |
| YDR121W | DPB4 | 0.8403094 | 0.98484693 | 0.02494295 | 108.5705923 | 199.3609676 |
| YBL008W | HIR1 | 0.95819998 | 0.99466569 | 0.02498546 | 109.3404655 | 200.7976236 |
| YIL087C | YIL087C | 0.90511561 | 0.98910891 | 0.0250727 | 121.5147272 | 223.6160192 |
| YPR022C | YPR022C | 0.88320823 | 0.98910891 | 0.0251519 | 104.3615113 | 191.4309163 |
| YLR246W | ERF2 | 0.91151808 | 0.98910891 | 0.02517686 | 112.273236 | 206.2654057 |
| YPL125W | KAP120 | 0.87418088 | 0.98901687 | 0.02533658 | 106.5687267 | 195.5390405 |
| YAR015W | ADE1 | 0.90056977 | 0.98910891 | 0.02550051 | 128.9626514 | 237.5117075 |
| YER039C | HVG1 | 0.84108246 | 0.98489448 | 0.02562046 | 107.4818232 | 197.2030201 |
| YNL212W | VID27 | 0.8754515 | 0.98901687 | 0.02578661 | 115.0450224 | 211.3596803 |
| YAR047C | YAR047C | 0.80926236 | 0.9824616 | 0.02583414 | 106.8501033 | 195.9816439 |
| YEL059W | YEL059W | 0.90063796 | 0.98910891 | 0.0261248 | 75.59353859 | 137.3089587 |
| YGL053W | PRM8 | 0.86240392 | 0.98660791 | 0.02622685 | 125.6353878 | 231.1470174 |
| YMR315W | YMR315W | 0.83591779 | 0.98293064 | 0.02624007 | 103.0776466 | 188.836796 |
| YNL067W | RPL9B | 0.91707189 | 0.98910891 | 0.02634242 | 83.32837965 | 151.7787325 |
| YDR263C | DIN7 | 0.86306529 | 0.98660791 | 0.02647558 | 118.7724691 | 218.2327785 |
| YBR024W | SCO2 | 0.87504947 | 0.98901687 | 0.02649276 | 125.1766599 | 230.2411581 |
| YPR026W | ATH1 | 0.83946727 | 0.98484693 | 0.02649899 | 111.8255901 | 205.1996203 |
| YBR204C | YBR204C | 0.8243815 | 0.9824616 | 0.02653104 | 118.5665235 | 217.8370309 |
| YGR061C | ADE6 | 0.86399451 | 0.98660791 | 0.02661228 | 104.8805543 | 192.1545404 |
| YEL017C-A | PMP2 | 0.8339089 | 0.9824616 | 0.02667651 | 124.4601969 | 228.865965 |
| YDR026C | YDR026C | 0.84340152 | 0.98519209 | 0.02668334 | 104.204505 | 190.8744247 |
| YOR114W | YOR114W | 0.87578226 | 0.98901687 | 0.02692166 | 122.2634049 | 224.7038494 |
| YBL064C | PRX1 | 0.83293966 | 0.9824616 | 0.02705235 | 113.8847678 | 208.9670154 |
| YLR224W | YLR224W | 0.86292588 | 0.98660791 | 0.02706471 | 117.4484518 | 215.6487343 |
| YDR515W | SLF1 | 0.82606275 | 0.9824616 | 0.0270968 | 96.36000484 | 176.0910062 |
| YPL247C | YPL247C | 0.89316582 | 0.98910891 | 0.02709987 | 125.5054199 | 230.7538895 |
| YPR064W | YPR064W | 0.86174627 | 0.98660791 | 0.02711147 | 100.5905666 | 184.0230849 |
| YIL067C | YIL067C | 0.82414308 | 0.9824616 | 0.02716213 | 110.23951 | 202.1114022 |
| YMR144W | YMR144W | 0.8732931 | 0.98901687 | 0.0271905 | 123.3633836 | 226.7209056 |
| YOR017W | PET127 | 0.87292275 | 0.98901687 | 0.02752752 | 118.414921 | 217.3822014 |
| YNR027W | BUD17 | 0.8445377 | 0.98543854 | 0.02752852 | 111.1216073 | 203.7031245 |
| YDL199C | YDL199C | 0.89105768 | 0.98910891 | 0.02772189 | 135.2975364 | 249.0129761 |
| YMR102C | YMR102C | 0.85676834 | 0.98660791 | 0.02787893 | 108.0616171 | 197.904036 |
| YPR002W | PDH1 | 0.85116847 | 0.98660791 | 0.02796463 | 102.2736574 | 187.0338198 |
| YBR014C | YBR014C | 0.88386268 | 0.98910891 | 0.02805795 | 124.0238678 | 227.8112564 |
| YFL031W | HAC1 | 0.82281601 | 0.9824616 | 0.02806039 | 107.2473813 | 196.3458575 |
| YMR251W-A | HOR7 | 0.86734261 | 0.98696321 | 0.02806377 | 120.0347016 | 220.3284175 |
| YLR264W | RPS28B | 0.86017019 | 0.98660791 | 0.0280753 | 105.3045328 | 192.6994149 |
| YNL166C | BNI5 | 0.70261776 | 0.97267176 | 0.02821314 | 113.9030261 | 208.8026547 |
| YKL086W | SRX1 | 0.86092075 | 0.98660791 | 0.0282797 | 115.3356474 | 211.4782062 |
| YIL170W | HXT12 | 0.87333079 | 0.98901687 | 0.0284883 | 116.0152369 | 212.7171136 |
| YJL079C | PRY1 | 0.76548448 | 0.97903138 | 0.02851495 | 97.66023436 | 178.287002 |
| YGR130C | YGR130C | 0.82248352 | 0.9824616 | 0.02886492 | 115.6537987 | 211.9747839 |
| YLR194C | YLR194C | 0.83506869 | 0.98249094 | 0.02931513 | 104.1551545 | 190.331583 |
| YDR131C | YDR131C | 0.83199943 | 0.9824616 | 0.02935927 | 116.2467459 | 213.0022999 |
| YDR234W | LYS4 | 0.86543699 | 0.98677815 | 0.02952029 | 104.7725352 | 191.4544029 |
| YBR066C | NRG2 | 0.82838425 | 0.9824616 | 0.02960639 | 125.6221307 | 230.5439342 |
| YDL223C | HBT1 | 0.88511691 | 0.98910891 | 0.02973653 | 119.6979594 | 219.4106455 |
| YEL020C | YEL020C | 0.87981411 | 0.98910891 | 0.02976348 | 111.4153009 | 203.8715711 |
| YIL130W | ASG1 | 0.87457073 | 0.98901687 | 0.02995464 | 121.9925992 | 223.6770177 |
| YLR380W | CSR1 | 0.86422641 | 0.98660791 | 0.03011592 | 106.7498434 | 195.0610163 |
| YLR385C | SWC7 | 0.83128475 | 0.9824616 | 0.03023768 | 106.6629329 | 194.8771804 |
| YHR086W | NAM8 | 0.90810739 | 0.98910891 | 0.03029734 | 99.16060636 | 180.796055 |
| YGL060W | YBP2 | 0.83442884 | 0.9824616 | 0.03034043 | 116.0334749 | 212.4344327 |
| YMR099C | YMR099C | 0.4029933 | 0.95675879 | 0.03049249 | 90.9780134 | 165.415881 |
| YDR358W | GGA1 | 0.8820859 | 0.98910891 | 0.03090327 | 115.0340857 | 210.4637377 |
| YGR288W | MAL13 | 0.76232002 | 0.97903138 | 0.03106224 | 111.6403414 | 204.0714346 |
| YFL028C | CAF16 | 0.79478165 | 0.98238124 | 0.03108564 | 102.7372416 | 187.3693062 |
| YDR428C | YDR428C | 0.81617668 | 0.9824616 | 0.03119356 | 112.0319495 | 204.7834436 |

| | | | | | | |
|---|---|---|---|---|---|---|
| YGR176W | YGR176W | 0.89354437 | 0.98910891 | 0.03151861 | 110.9328429 | 202.6664096 |
| YLR284C | ECI1 | 0.75529717 | 0.97903138 | 0.03194573 | 95.51219544 | 173.6712835 |
| YGR136W | LSB1 | 0.80790223 | 0.9824616 | 0.03231799 | 121.079013 | 221.5591954 |
| YHR159W | YHR159W | 0.84722312 | 0.98660791 | 0.03232577 | 120.6061964 | 220.6710767 |
| YNL049C | SFB2 | 0.8075857 | 0.9824616 | 0.03244715 | 108.8925682 | 198.6809249 |
| YFL035C-B | YFL035C-B | 0.76705891 | 0.97903138 | 0.03272977 | 110.8067494 | 202.2226947 |
| YLR367W | RPS22B | 0.8168868 | 0.9824616 | 0.03274944 | 99.66988499 | 181.331688 |
| YJL013C | MAD3 | 0.8233048 | 0.9824616 | 0.03298446 | 110.684237 | 201.9493414 |
| YPL014W | YPL014W | 0.87581129 | 0.98901687 | 0.03311936 | 114.1024931 | 208.3373396 |
| YGR043C | YGR043C | 0.67438026 | 0.96992497 | 0.03316037 | 111.6199867 | 203.6742812 |
| YAR003W | SWD1 | 0.94560767 | 0.99208511 | 0.0332987 | 58.87584462 | 104.7268356 |
| YDR072C | IPT1 | 0.88550325 | 0.98910891 | 0.03331229 | 113.2282287 | 206.6646117 |
| YPL176C | TRE1 | 0.81002686 | 0.9824616 | 0.03333216 | 107.8396548 | 196.5547239 |
| YGL228W | SHE10 | 0.81216647 | 0.9824616 | 0.03350179 | 128.7126546 | 235.6738587 |
| YIR031C | DAL7 | 0.86183075 | 0.98660791 | 0.03363708 | 115.3355646 | 210.5614362 |
| YJL151C | SNA3 | 0.88206364 | 0.98910891 | 0.0337093 | 108.4627095 | 197.6587611 |
| YPR059C | YPR059C | 0.8231657 | 0.9824616 | 0.03377357 | 106.0888397 | 193.1954768 |
| YNL155W | YNL155W | 0.91554629 | 0.98910891 | 0.03387832 | 93.91631939 | 170.3474989 |
| YOR034C | AKR2 | 0.83228598 | 0.9824616 | 0.03388929 | 113.2156886 | 206.542372 |
| YDR251W | PAM1 | 0.84883821 | 0.98660791 | 0.03391806 | 130.6612475 | 239.257302 |
| YMR118C | YMR118C | 0.8106066 | 0.9824616 | 0.03416459 | 109.3830694 | 199.3070362 |
| YDR425W | SNX41 | 0.75433273 | 0.97903138 | 0.03433176 | 109.4941148 | 199.4867044 |
| YDL185W | TFP1 | 0.82736098 | 0.9824616 | 0.03479247 | 124.0774044 | 226.7594312 |
| YCL046W | YCL046W | 0.6832072 | 0.96992497 | 0.03489708 | 118.0395958 | 215.4173792 |
| YBR018C | GAL7 | 0.81567055 | 0.9824616 | 0.0349959 | 120.1037576 | 219.2718895 |
| YER056C-A | RPL34A | 0.81030647 | 0.9824616 | 0.0350653 | 108.4920902 | 197.4818627 |
| YNL280C | ERG24 | 0.75818323 | 0.97903138 | 0.03513797 | 101.3554854 | 184.084437 |
| YNL054W | VAC7 | 0.79740017 | 0.9824616 | 0.03538959 | 112.0088856 | 204.0222708 |
| YDR318W | MCM21 | 0.86452648 | 0.98660791 | 0.03540964 | 116.7389798 | 212.8903246 |
| YLR016C | PML1 | 0.79322433 | 0.98181836 | 0.03541641 | 109.6181356 | 199.5337337 |
| YIL138C | TPM2 | 0.84645327 | 0.98660791 | 0.0354178 | 120.4110883 | 219.7761174 |
| YLL056C | YLL056C | 0.77999314 | 0.97903138 | 0.0354845 | 112.0410577 | 204.066373 |
| YCR107W | AAD3 | 0.79814418 | 0.9824616 | 0.03552884 | 104.8732458 | 190.6152643 |
| YBL053W | YBL053W | 0.79881193 | 0.9824616 | 0.0355666 | 112.7888288 | 205.4548015 |
| YBR276C | PPS1 | 0.83381906 | 0.9824616 | 0.03559996 | 132.847312 | 243.0695936 |
| YPL167C | REV3 | 0.85680912 | 0.98660791 | 0.03587426 | 114.1030357 | 207.8670105 |
| YGR015C | YGR015C | 0.78855823 | 0.98007539 | 0.03590926 | 119.3237132 | 217.6526157 |
| YJR095W | SFC1 | 0.81207033 | 0.9824616 | 0.03599892 | 92.13690044 | 166.6473051 |
| YJL126W | NIT2 | 0.79424768 | 0.98228673 | 0.0360641 | 115.3560787 | 210.1846627 |
| YNL058C | YNL058C | 0.90265505 | 0.98910891 | 0.03607355 | 89.78842482 | 162.2298751 |
| YJL088W | ARG3 | 0.82559329 | 0.9824616 | 0.03608709 | 119.3672579 | 217.7038602 |
| YJL068C | YJL068C | 0.83351095 | 0.9824616 | 0.03610498 | 120.846991 | 220.4760976 |
| YMR262W | YMR262W | 0.62602348 | 0.96098984 | 0.03616085 | 100.0455814 | 181.4526519 |
| YNL140C | YNL140C | 0.78088763 | 0.97903138 | 0.03635392 | 97.42610732 | 176.5066879 |
| YDR534C | FIT1 | 0.72829748 | 0.97683349 | 0.036505 | 108.9312839 | 198.059264 |
| YGL110C | CUE3 | 0.76111326 | 0.97903138 | 0.0368973 | 121.5872812 | 221.7289807 |
| YBL091C | MAP2 | 0.82239181 | 0.9824616 | 0.03705661 | 135.6583069 | 248.0925035 |
| YBR104W | YMC2 | 0.85067817 | 0.98660791 | 0.03716796 | 100.7631704 | 182.6262092 |
| YNR032C-A | HUB1 | 0.80407057 | 0.9824616 | 0.03745037 | 115.0142855 | 209.3064347 |
| YDR248C | YDR248C | 0.86460307 | 0.98660791 | 0.03749592 | 133.4296948 | 243.8374885 |
| YBR008C | FLR1 | 0.89744084 | 0.98910891 | 0.03757904 | 129.9516994 | 237.3001452 |
| YDR213W | UPC2 | 0.86405867 | 0.98660791 | 0.03766473 | 131.4887056 | 240.1682017 |
| YGR126W | YGR126W | 0.7145801 | 0.97378831 | 0.03776077 | 120.727522 | 219.968733 |
| YLR135W | SLX4 | 0.8218074 | 0.9824616 | 0.03789252 | 105.7539442 | 191.8626402 |
| YLR177W | YLR177W | 0.92759685 | 0.99106129 | 0.038065 | 86.63878727 | 155.9818773 |
| YDR132C | YDR132C | 0.80315635 | 0.9824616 | 0.03831485 | 114.6446648 | 208.4652889 |
| YAL067C | SEO1 | 0.77215001 | 0.97903138 | 0.03834952 | 115.0660422 | 209.2496674 |
| YML081C-A | ATP18 | 0.85846406 | 0.98660791 | 0.03846685 | 85.85594659 | 154.4448733 |
| YLR130C | ZRT2 | 0.83112523 | 0.9824616 | 0.03849439 | 113.5623954 | 206.4047295 |
| YJL152W | YJL152W | 0.85215351 | 0.98660791 | 0.03849665 | 124.8557335 | 227.5854574 |
| YBR184W | YBR184W | 0.80630134 | 0.9824616 | 0.03850838 | 129.5169321 | 236.3257172 |
| YER175C | TMT1 | 0.77203267 | 0.97903138 | 0.03855327 | 106.6490721 | 193.4284381 |
| YOR178C | GAC1 | 0.83157645 | 0.9824616 | 0.0386099 | 119.958605 | 218.3813177 |
| YBR030W | YBR030W | 0.75173367 | 0.97903138 | 0.03900444 | 119.9035109 | 218.2104829 |

| | | | | | | |
|---|---|---|---|---|---|---|
| YJR126C | VPS70 | 0.92717171 | 0.99106129 | 0.03900629 | 95.04121122 | 171.5799151 |
| YER121W | YER121W | 0.75897691 | 0.97903138 | 0.0390893 | 113.7495861 | 206.6540278 |
| YHR092C | HXT4 | 0.86187129 | 0.98660791 | 0.03912679 | 120.8202501 | 219.9089313 |
| YNL336W | COS1 | 0.85042027 | 0.98660791 | 0.03914283 | 109.0352018 | 197.8028503 |
| YBR263W | SHM1 | 0.78394529 | 0.97903138 | 0.03916782 | 129.8411335 | 236.820944 |
| YBL011W | SCT1 | 0.93787411 | 0.9913313 | 0.03967888 | 118.8132187 | 216.0502037 |
| YDR476C | YDR476C | 0.77849157 | 0.97903138 | 0.03982004 | 112.9129427 | 204.9598452 |
| YDR536W | STL1 | 0.7472682 | 0.97903138 | 0.03986005 | 120.8671473 | 219.8714328 |
| YGR290W | YGR290W | 0.83222346 | 0.9824616 | 0.03986245 | 117.2963588 | 213.1738631 |
| YER053C | PIC2 | 0.6383531 | 0.96415344 | 0.03991591 | 122.9381182 | 223.7460664 |
| YNL173C | MDG1 | 0.70008019 | 0.97267176 | 0.04010656 | 107.6262615 | 194.9954379 |
| YDR467C | YDR467C | 0.83755555 | 0.98452327 | 0.04024819 | 122.557058 | 222.9745208 |
| YDR348C | YDR348C | 0.75317521 | 0.97903138 | 0.04026506 | 116.1952842 | 211.0398702 |
| YLR173W | YLR173W | 0.78880776 | 0.98007539 | 0.04031642 | 108.5804427 | 196.7491384 |
| YIL079C | AIR1 | 0.85000134 | 0.98660791 | 0.04035611 | 126.1548497 | 229.7038599 |
| YLR211C | YLR211C | 0.78297391 | 0.97903138 | 0.04051899 | 110.0276199 | 199.4287185 |
| YIL102C | YIL102C | 0.78749599 | 0.97986316 | 0.04057158 | 103.4631754 | 187.1078387 |
| YLR401C | DUS3 | 0.81847185 | 0.9824616 | 0.04071387 | 110.0895376 | 199.5115047 |
| YLR297W | YLR297W | 0.81869705 | 0.9824616 | 0.04078536 | 114.5908715 | 207.9417088 |
| YMR251W | GTO3 | 0.68717002 | 0.96992497 | 0.04079744 | 104.1189641 | 188.2991544 |
| YJL083W | TAX4 | 0.83112482 | 0.9824616 | 0.04083535 | 124.7169701 | 226.9250641 |
| YMR300C | ADE4 | 0.74351151 | 0.97903138 | 0.0410137 | 101.1042549 | 182.607944 |
| YDL091C | UBX3 | 0.75473843 | 0.97903138 | 0.04103974 | 129.4025241 | 235.6780404 |
| YNL050C | YNL050C | 0.93382254 | 0.99106129 | 0.04108232 | 97.66360941 | 176.1431336 |
| YDR030C | RAD28 | 0.79741181 | 0.9824616 | 0.0410933 | 107.6814248 | 194.9300731 |
| YDR254W | CHL4 | 0.89566877 | 0.98910891 | 0.04118704 | 129.7965997 | 236.3919439 |
| YOR243C | PUS7 | 0.76371363 | 0.97903138 | 0.04137893 | 104.6181869 | 189.1359776 |
| YLR225C | YLR225C | 0.78536301 | 0.97903138 | 0.04143894 | 107.5211544 | 194.5703438 |
| YEL003W | GIM4 | 0.89876093 | 0.98910891 | 0.04152614 | 74.83099801 | 133.2437093 |
| YBL057C | PTH2 | 0.8332922 | 0.9824616 | 0.04205166 | 122.6468223 | 222.8343141 |
| YDR517W | GRH1 | 0.79533599 | 0.9824616 | 0.04220137 | 123.7480313 | 224.8740626 |
| YCR086W | CSM1 | 0.9160327 | 0.98910891 | 0.04220758 | 89.47018672 | 160.5835113 |
| YGR291C | YGR291C | 0.78603425 | 0.97903138 | 0.04225145 | 104.3001916 | 188.390281 |
| YIL047C | SYG1 | 0.792716 | 0.98180632 | 0.04245921 | 122.0304631 | 221.608578 |
| YJL211C | YJL211C | 0.84184873 | 0.98507962 | 0.04246294 | 107.5774101 | 194.5006527 |
| YJL100W | LSB6 | 0.79502237 | 0.98239599 | 0.0425583 | 131.9418666 | 240.1808641 |
| YMR320W | YMR320W | 0.74234356 | 0.97903138 | 0.04296133 | 114.5852647 | 207.5588982 |
| YHR021W-A | ECM12 | 0.80099602 | 0.9824616 | 0.04308163 | 113.2983639 | 205.1246803 |
| YGR121C | MEP1 | 0.75625524 | 0.97903138 | 0.04321754 | 121.648496 | 220.7624383 |
| YMR284W | YKU70 | 0.76864828 | 0.97903138 | 0.04322081 | 118.0699534 | 214.0501765 |
| YDR192C | NUP42 | 0.81590649 | 0.9824616 | 0.04332239 | 123.0304171 | 223.3363479 |
| YGL096W | TOS8 | 0.75913831 | 0.97903138 | 0.04334801 | 103.4614795 | 186.6296288 |
| YMR147W | YMR147W | 0.75683839 | 0.97903138 | 0.04354627 | 106.7520335 | 192.7672748 |
| YNL253W | TEX1 | 0.68756375 | 0.96992497 | 0.0435609 | 101.3693142 | 182.6692632 |
| YOL111C | MDY2 | 0.76564193 | 0.97903138 | 0.04380052 | 122.9854394 | 223.1701858 |
| YBR169C | SSE2 | 0.77981649 | 0.97903138 | 0.04391851 | 127.3086237 | 231.2583057 |
| YJR043C | POL32 | 0.90967439 | 0.98910891 | 0.04400264 | 84.8217941 | 151.5581395 |
| YBR139W | YBR139W | 0.81567155 | 0.9824616 | 0.04416801 | 134.3290125 | 244.3826426 |
| YBR065C | ECM2 | 0.82004979 | 0.9824616 | 0.04419973 | 130.4957023 | 237.187686 |
| YHR136C | SPL2 | 0.78356993 | 0.97903138 | 0.04422411 | 135.6892057 | 246.9241407 |
| YJL105W | SET4 | 0.91167472 | 0.98910891 | 0.04441804 | 102.7469702 | 185.1064614 |
| YJR109C | CPA2 | 0.80593945 | 0.9824616 | 0.04447749 | 98.14598407 | 176.4669547 |
| YPR097W | YPR097W | 0.75045326 | 0.97903138 | 0.04463297 | 108.7911864 | 196.4058607 |
| YPR076W | YPR076W | 0.73160536 | 0.97731085 | 0.04464117 | 110.5442323 | 199.692367 |
| YJR021C | REC107 | 0.8059386 | 0.9824616 | 0.04466807 | 115.6842033 | 209.3279888 |
| YBL063W | KIP1 | 0.70926889 | 0.97267176 | 0.04481714 | 127.4502926 | 231.3702622 |
| YCR101C | YCR101C | 0.81734558 | 0.9824616 | 0.04485375 | 119.4446333 | 216.3490589 |
| YMR199W | CLN1 | 0.71111128 | 0.97267176 | 0.04518874 | 104.8378889 | 188.8962027 |
| YPL227C | ALG5 | 0.86358069 | 0.98660791 | 0.04544474 | 136.7142403 | 248.6377934 |
| YJR116W | YJR116W | 0.79930403 | 0.9824616 | 0.04552516 | 92.91108109 | 166.469432 |
| YPR039W | YPR039W | 0.78570761 | 0.97903138 | 0.04556144 | 107.8600665 | 194.500653 |
| YDR142C | PEX7 | 0.7518592 | 0.97903138 | 0.04580462 | 113.6237502 | 205.2690687 |
| YFL040W | YFL040W | 0.47465177 | 0.95675879 | 0.04585979 | 94.61129359 | 169.6009958 |
| YDR333C | YDR333C | 0.65964305 | 0.96992497 | 0.04598064 | 113.6213332 | 205.2344195 |

| | | | | | | |
|---|---|---|---|---|---|---|
| YDR414C | ERD1 | 0.80244558 | 0.9824616 | 0.04602799 | 117.3729557 | 212.2626383 |
| YLR128W | DCN1 | 0.72041311 | 0.97530288 | 0.04606327 | 97.3096695 | 174.6270952 |
| YMR302C | YME2 | 0.73604105 | 0.97781627 | 0.04608818 | 120.6803226 | 218.4554408 |
| YIL093C | RSM25 | 0.80605294 | 0.9824616 | 0.04621443 | 114.454035 | 206.756186 |
| YDR222W | YDR222W | 0.8093436 | 0.9824616 | 0.04637781 | 130.2088322 | 236.2769944 |
| YBR220C | YBR220C | 0.81331607 | 0.9824616 | 0.04656276 | 124.2737953 | 225.113947 |
| YFR014C | CMK1 | 0.72869091 | 0.97683349 | 0.0466486 | 130.9555651 | 237.6311909 |
| YDL065C | PEX19 | 0.69002598 | 0.96992497 | 0.04670692 | 134.9528617 | 245.1183044 |
| YJR094W-A | RPL43B | 0.90183254 | 0.98910891 | 0.046856 | 86.46370694 | 154.1494202 |
| YDR057W | YOS9 | 0.78776239 | 0.97987767 | 0.04708859 | 136.6740633 | 248.2811876 |
| YLR057W | YLR057W | 0.81295812 | 0.9824616 | 0.04710437 | 117.2153336 | 211.7828504 |
| YBR248C | HIS7 | 0.68765478 | 0.96992497 | 0.047241 | 119.2264766 | 215.5314545 |
| YNL093W | YPT53 | 0.90499601 | 0.98910891 | 0.04733341 | 94.71979008 | 169.5523574 |
| YDR123C | INO2 | 0.88744651 | 0.98910891 | 0.04794232 | 83.53596444 | 148.4724588 |
| YGR033C | TIM21 | 0.72124386 | 0.97543973 | 0.0482482 | 125.0411007 | 226.2646908 |
| YJR107W | YJR107W | 0.73879867 | 0.97903138 | 0.04829552 | 107.8496079 | 194.0132532 |
| YDR314C | RAD34 | 0.77691732 | 0.97903138 | 0.04833969 | 128.1550078 | 232.0892966 |
| YBR294W | SUL1 | 0.77390178 | 0.97903138 | 0.04868013 | 131.9069784 | 239.0680226 |
| YBR199W | KTR4 | 0.78296972 | 0.97903138 | 0.04878302 | 112.7002462 | 203.0274132 |
| YOR192C | THI72 | 0.83362089 | 0.9824616 | 0.04887221 | 133.1197949 | 241.3098458 |
| YBL107C | YBL107C | 0.73514533 | 0.97731085 | 0.04895215 | 115.2964532 | 207.8677676 |
| YDL210W | UGA4 | 0.6620941 | 0.96992497 | 0.0490388 | 109.5963632 | 197.1621932 |
| YCR106W | RDS1 | 0.74201446 | 0.97903138 | 0.04910399 | 113.4333771 | 204.3475143 |
| YLR400W | YLR400W | 0.77585743 | 0.97903138 | 0.04917917 | 110.9243833 | 199.628932 |
| YBL010C | YBL010C | 0.7737225 | 0.97903138 | 0.04934475 | 133.5448254 | 242.0261594 |
| YBL019W | APN2 | 0.77810328 | 0.97903138 | 0.04935432 | 122.962873 | 222.1776409 |
| YHR045W | YHR045W | 0.92243152 | 0.98998274 | 0.04950901 | 112.9328746 | 203.3395049 |
| YBL101C | ECM21 | 0.80528737 | 0.9824616 | 0.04964406 | 123.680154 | 223.4733582 |
| YIL111W | COX5B | 0.78539576 | 0.97903138 | 0.04975137 | 110.2242358 | 198.2178767 |
| YGR137W | YGR137W | 0.67129822 | 0.96992497 | 0.04978261 | 122.8219861 | 221.8401245 |
| YOL009C | MDM12 | 0.91548972 | 0.98910891 | 0.0498661 | 33.1851059 | 53.708233 |
| YMR258C | YMR258C | 0.72046128 | 0.97530288 | 0.04991465 | 106.6033142 | 191.398756 |
| YBR063C | YBR063C | 0.71190132 | 0.97267176 | 0.05018655 | 125.9709231 | 227.6769708 |
| YOL042W | NGL1 | 0.8136313 | 0.9824616 | 0.05020449 | 127.2449321 | 230.063358 |
| YLR362W | STE11 | 0.75516969 | 0.97903138 | 0.05027048 | 125.2392495 | 226.2903278 |
| YML095C | RAD10 | 0.78796404 | 0.97987767 | 0.05036128 | 125.9020183 | 227.5178427 |
| YIL016W | SNL1 | 0.75007041 | 0.97903138 | 0.05036996 | 119.2552419 | 215.0500591 |
| YNL242W | ATG2 | 0.90027091 | 0.98910891 | 0.05039547 | 89.64027474 | 159.501622 |
| YGL198W | YIP4 | 0.78135914 | 0.97903138 | 0.05043156 | 126.2170767 | 228.0967222 |
| YDR491C | YDR491C | 0.70723228 | 0.97267176 | 0.05052304 | 119.873238 | 216.1829445 |
| YJL171C | YJL171C | 0.80014054 | 0.9824616 | 0.05057329 | 122.6793902 | 221.4373998 |
| YLR425W | TUS1 | 0.89828704 | 0.98910891 | 0.05072484 | 96.22657748 | 171.7981456 |
| YGR134W | CAF130 | 0.72804944 | 0.97683349 | 0.05077526 | 125.5265563 | 226.7428189 |
| YNL120C | YNL120C | 0.86890787 | 0.98763165 | 0.05097021 | 81.63166743 | 144.3828195 |
| YJR052W | RAD7 | 0.70640051 | 0.97267176 | 0.05123734 | 120.8252675 | 217.8463017 |
| YIL049W | DFG10 | 0.87959708 | 0.98910891 | 0.05137992 | 96.52536479 | 172.2464534 |
| YKR005C | YKR005C | 0.74057084 | 0.97903138 | 0.05138521 | 116.2536287 | 209.2467078 |
| YGR213C | RTA1 | 0.70134568 | 0.97267176 | 0.05159416 | 107.2865242 | 192.3927895 |
| YNL288W | CAF40 | 0.71870712 | 0.97530288 | 0.05176778 | 120.5261949 | 217.1946252 |
| YKR050W | TRK2 | 0.77926608 | 0.97903138 | 0.05179201 | 103.369705 | 185.0127861 |
| YJL048C | UBX6 | 0.71222089 | 0.97267176 | 0.05203655 | 119.2051634 | 214.6709913 |
| YOR079C | ATX2 | 0.76397137 | 0.97903138 | 0.05213431 | 128.9641644 | 232.9576679 |
| YCL035C | GRX1 | 0.75358256 | 0.97903138 | 0.05244564 | 131.183951 | 237.0677015 |
| YIL133C | RPL16A | 0.85776538 | 0.98660791 | 0.05258826 | 85.37263046 | 151.1223087 |
| YIL066C | RNR3 | 0.75573572 | 0.97903138 | 0.05262832 | 125.1156413 | 225.6550848 |
| YML053C | YML053C | 0.68314411 | 0.96992497 | 0.05270013 | 121.3435451 | 218.5680783 |
| YJR035W | RAD26 | 0.70904847 | 0.97267176 | 0.05286503 | 108.3973902 | 194.2588263 |
| YOR092W | ECM3 | 0.76258632 | 0.97903138 | 0.05298372 | 122.7804001 | 221.2144371 |
| YDR441C | APT2 | 0.69725721 | 0.97267176 | 0.05302419 | 112.2942103 | 201.5402393 |
| YDR330W | UBX5 | 0.79302348 | 0.98181836 | 0.05306403 | 112.3745732 | 201.6841467 |
| YAL027W | YAL027W | 0.79181023 | 0.98180632 | 0.05322473 | 136.7627653 | 247.3976962 |
| YJL145W | SFH5 | 0.77922333 | 0.97903138 | 0.05334128 | 116.3723146 | 209.1346378 |
| YER149C | PEA2 | 0.67348512 | 0.96992497 | 0.05362075 | 116.8682637 | 210.0169937 |
| YPR152C | URN1 | 0.74653733 | 0.97903138 | 0.05368908 | 104.1026163 | 186.0628132 |

| | | | | | | |
|---|---|---|---|---|---|---|
| YBR229C | ROT2 | 0.61086668 | 0.9598703 | 0.0539333 | 130.5530487 | 235.6298887 |
| YOR190W | SPR1 | 0.74340831 | 0.97903138 | 0.0542285 | 117.0484891 | 210.2510314 |
| YLR006C | SSK1 | 0.68318108 | 0.96992497 | 0.05430645 | 93.87767818 | 166.7798986 |
| YMR181C | YMR181C | 0.71355321 | 0.9730065 | 0.05432249 | 109.555492 | 196.1815317 |
| YGL141W | HUL5 | 0.69974975 | 0.97267176 | 0.05449356 | 124.0236498 | 223.2878795 |
| YPL114W | YPL114W | 0.84195004 | 0.98507962 | 0.05449732 | 131.5728271 | 237.4460233 |
| YLR390W | ECM19 | 0.66817143 | 0.96992497 | 0.05451562 | 104.6194374 | 186.8907173 |
| YOR152C | YOR152C | 0.74884991 | 0.97903138 | 0.05458211 | 119.0195099 | 213.8872608 |
| YKL084W | HOT13 | 0.71084227 | 0.97267176 | 0.05461259 | 114.0013651 | 204.4703113 |
| YGL226W | MTC3 | 0.87473524 | 0.98901687 | 0.05469565 | 108.0026574 | 193.2052812 |
| YMR017W | SPO20 | 0.84456718 | 0.98543854 | 0.05484311 | 96.21008731 | 171.0626086 |
| YKR058W | GLG1 | 0.77605918 | 0.97903138 | 0.05500622 | 129.3436255 | 233.1779963 |
| YGR212W | SLI1 | 0.77119678 | 0.97903138 | 0.05512078 | 126.6263642 | 228.0620625 |
| YER124C | DSE1 | 0.67528811 | 0.96992497 | 0.05529963 | 114.5076841 | 205.3023859 |
| YPL144W | POC4 | 0.7863715 | 0.97903138 | 0.05533037 | 111.1284122 | 198.9591639 |
| YLR092W | SUL2 | 0.72593401 | 0.97673059 | 0.05552848 | 105.3981539 | 188.1779369 |
| YLR286C | CTS1 | 0.68582 | 0.96992497 | 0.05560683 | 117.8804069 | 211.5755034 |
| YMR182C | RGM1 | 0.50725958 | 0.95675879 | 0.05572835 | 112.5502192 | 201.5577287 |
| YNL086W | YNL086W | 0.71138361 | 0.97267176 | 0.05583231 | 102.4963662 | 182.6835332 |
| YIL151C | YIL151C | 0.62915542 | 0.96098984 | 0.0558523 | 116.1147854 | 208.2220099 |
| YNL085W | MKT1 | 0.86874115 | 0.98763165 | 0.05613404 | 96.65746652 | 171.6808162 |
| YGR181W | TIM13 | 0.53747936 | 0.95675879 | 0.05625029 | 115.4350491 | 206.8790444 |
| YML055W | SPC2 | 0.68001989 | 0.96992497 | 0.05632551 | 98.87364143 | 175.804583 |
| YNR072W | HXT17 | 0.7191675 | 0.97530288 | 0.05635855 | 118.4967739 | 212.6029108 |
| YDR424C | DYN2 | 0.76216085 | 0.97903138 | 0.05639187 | 114.4795126 | 205.0626737 |
| YNL167C | SKO1 | 0.58999971 | 0.95959383 | 0.05640725 | 103.7297654 | 184.8984549 |
| YJL198W | PHO90 | 0.76910273 | 0.97903138 | 0.05654068 | 132.0610452 | 238.0120898 |
| YDR179W-A | YDR179W-A | 0.73975893 | 0.97903138 | 0.05659057 | 123.1345367 | 221.2615265 |
| YMR107W | SPG4 | 0.77885178 | 0.97903138 | 0.05662625 | 119.6825246 | 214.7810321 |
| YOL129W | VPS68 | 0.78694252 | 0.97945831 | 0.05672151 | 127.7927615 | 229.9758121 |
| YGR123C | PPT1 | 0.70862582 | 0.97267176 | 0.05681765 | 136.3104882 | 245.9347063 |
| YGR141W | VPS62 | 0.68026957 | 0.96992497 | 0.05709265 | 128.7413762 | 231.6914789 |
| YDL239C | ADY3 | 0.75951786 | 0.97903138 | 0.0572632 | 124.5012211 | 223.7097144 |
| YNL009W | IDP3 | 0.77386767 | 0.97903138 | 0.05748457 | 133.7489346 | 241.0163019 |
| YLR040C | YLR040C | 0.71131664 | 0.97267176 | 0.05760301 | 104.1770519 | 185.5327718 |
| YDR403W | DIT1 | 0.70648307 | 0.97267176 | 0.05772926 | 126.2338662 | 226.879621 |
| YDR285W | ZIP1 | 0.71960698 | 0.97530288 | 0.05777109 | 116.1329756 | 207.9278348 |
| YPR066W | UBA3 | 0.67126527 | 0.96992497 | 0.05778122 | 93.89642547 | 166.2205493 |
| YLL015W | BPT1 | 0.66253262 | 0.96992497 | 0.05781779 | 111.9203253 | 200.0188477 |
| YBR069C | TAT1 | 0.7447819 | 0.97903138 | 0.05782308 | 118.2571576 | 211.9029293 |
| YDR015C | YDR015C | 0.75867227 | 0.97903138 | 0.05790378 | 116.1292878 | 207.8982151 |
| YKR003W | OSH6 | 0.8871994 | 0.98910891 | 0.05792039 | 98.16718632 | 174.2067232 |
| YLL063C | AYT1 | 0.72700917 | 0.97677641 | 0.05797814 | 115.4899439 | 206.6863765 |
| YDR426C | YDR426C | 0.62049328 | 0.96098984 | 0.05799767 | 110.4944788 | 197.3138387 |
| YKL067W | YNK1 | 0.61160271 | 0.9598703 | 0.05805491 | 106.1637714 | 189.1816276 |
| YJR152W | DAL5 | 0.49393396 | 0.95675879 | 0.05806587 | 92.48014611 | 163.5155577 |
| YDR530C | APA2 | 0.63206983 | 0.96098984 | 0.0581011 | 120.779615 | 216.5863319 |
| YPL196W | OXR1 | 0.6577411 | 0.96992497 | 0.05827679 | 120.5567149 | 216.1382139 |
| YBR045C | GIP1 | 0.65851961 | 0.96992497 | 0.0582944 | 125.9845027 | 226.3152374 |
| YOR099W | KTR1 | 0.76455947 | 0.97903138 | 0.05838794 | 120.3733181 | 215.7752298 |
| YBR041W | FAT1 | 0.88600495 | 0.98910891 | 0.05867081 | 99.80895796 | 177.1575402 |
| YLR081W | GAL2 | 0.68709813 | 0.96992497 | 0.05881863 | 119.1624933 | 213.4305904 |
| YDR287W | YDR287W | 0.67802377 | 0.96992497 | 0.05911077 | 115.9697489 | 207.3924854 |
| YJL049W | YJL049W | 0.77339577 | 0.97903138 | 0.05934439 | 125.0426946 | 224.369192 |
| YLR406C | RPL31B | 0.654538 | 0.96992497 | 0.05934452 | 100.3799843 | 178.1132553 |
| YOR158W | PET123 | 0.7123848 | 0.97267176 | 0.05960774 | 107.207235 | 190.8730069 |
| YBR214W | SDS24 | 0.7761201 | 0.97903138 | 0.05993698 | 123.2793406 | 220.9605616 |
| YDR110W | FOB1 | 0.6811115 | 0.96992497 | 0.06001226 | 123.718341 | 221.771045 |
| YNL309W | STB1 | 0.57410086 | 0.95959383 | 0.06008545 | 103.925229 | 184.6357381 |
| YKL094W | YJU3 | 0.72674761 | 0.97673059 | 0.06031588 | 102.6306391 | 182.1682556 |
| YNL304W | YPT11 | 0.42536406 | 0.95675879 | 0.06036391 | 100.7041398 | 178.546811 |
| YBL025W | RRN10 | 0.6609554 | 0.96992497 | 0.06076644 | 124.4951637 | 223.0989715 |
| YKL101W | HSL1 | 0.39100222 | 0.95675879 | 0.06076773 | 5.543457813 | 0 |
| YLR345W | YLR345W | 0.71103346 | 0.97267176 | 0.06077315 | 109.546628 | 195.061238 |

| | | | | | | |
|---|---|---|---|---|---|---|
| YML017W | PSP2 | 0.52984044 | 0.95675879 | 0.06106901 | 114.3951082 | 204.1041397 |
| YOR247W | SRL1 | 0.80917564 | 0.9824616 | 0.06108242 | 124.2622308 | 222.6080351 |
| YHR014W | SPO13 | 0.69998332 | 0.97267176 | 0.06114404 | 123.31754 | 220.8256863 |
| YDR309C | GIC2 | 0.68468995 | 0.96992497 | 0.06121596 | 124.8278002 | 223.6459346 |
| YML059C | NTE1 | 0.63470394 | 0.96134699 | 0.06132652 | 121.6956378 | 217.752521 |
| YJL051W | IRC8 | 0.61186259 | 0.9598703 | 0.06137531 | 118.593479 | 211.925948 |
| YLR210W | CLB4 | 0.63964882 | 0.96463263 | 0.0614701 | 113.5166531 | 202.3879381 |
| YOR002W | ALG6 | 0.67558994 | 0.96992497 | 0.06147764 | 111.1040382 | 197.8616901 |
| YHR033W | YHR033W | 0.90527995 | 0.98910891 | 0.06159602 | 113.6155926 | 202.5519586 |
| YOL089C | HAL9 | 0.70813078 | 0.97267176 | 0.06177698 | 113.2103718 | 201.7609895 |
| YBR296C | PHO89 | 0.75267125 | 0.97903138 | 0.06177836 | 138.2273875 | 248.6811817 |
| YDR239C | YDR239C | 0.71664761 | 0.97530288 | 0.06177907 | 115.0394463 | 205.1911351 |
| YMR135W-A | YMR135W-A | 0.86636679 | 0.98696321 | 0.06185144 | 65.97954739 | 113.1649222 |
| YJL159W | HSP150 | 0.71597111 | 0.97475585 | 0.06185971 | 110.2437236 | 196.182765 |
| YBR185C | MBA1 | 0.88294663 | 0.98910891 | 0.06189575 | 105.5788806 | 187.4274969 |
| YCL060C | :::MRC1 | 0.85448309 | 0.98660791 | 0.06191509 | 109.3655695 | 194.5262766 |
| YCR067C | SED4 | 0.66840042 | 0.96992497 | 0.06192504 | 126.3743276 | 226.4251901 |
| YDR540C | IRC4 | 0.52968447 | 0.95675879 | 0.0623328 | 106.5267776 | 189.1305393 |
| YCL001W-A | YCL001W-A | 0.61240669 | 0.9598703 | 0.06300316 | 108.8838294 | 193.4365909 |
| YNL063W | MTQ1 | 0.69531099 | 0.97267176 | 0.0632125 | 124.8597469 | 223.364256 |
| YPR148C | YPR148C | 0.62472001 | 0.96098984 | 0.06343448 | 111.042382 | 197.4112484 |
| YNL254C | RTC4 | 0.47890995 | 0.95675879 | 0.06354592 | 115.4996043 | 205.7518833 |
| YOR221C | MCT1 | 0.72400183 | 0.97653633 | 0.06357401 | 100.8274005 | 178.228764 |
| YBR051W | YBR051W | 0.67735129 | 0.96992497 | 0.06358364 | 127.8055431 | 228.8257165 |
| YHR049C-A | YHR049C-A | 0.7202522 | 0.97530288 | 0.06373539 | 126.8572441 | 227.021181 |
| YJL161W | FMP33 | 0.78370954 | 0.97903138 | 0.06390278 | 115.1216707 | 204.9819964 |
| YHR032W | YHR032W | 0.76358919 | 0.97903138 | 0.06402692 | 124.5737987 | 222.6886071 |
| YCL036W | GFD2 | 0.79376169 | 0.98196851 | 0.06407767 | 136.8287408 | 245.664565 |
| YJL175W | YJL175W | 0.72386194 | 0.97653633 | 0.06436723 | 66.74387274 | 114.1680096 |
| YNL028W | YNL028W | 0.62624317 | 0.96098984 | 0.06441099 | 114.9111658 | 204.5002353 |
| YOR179C | SYC1 | 0.69111687 | 0.97045646 | 0.0645704 | 104.2111436 | 184.4046344 |
| YLR046C | YLR046C | 0.70335761 | 0.97267176 | 0.0647291 | 119.7821287 | 213.5814971 |
| YJL132W | YJL132W | 0.66279477 | 0.96992497 | 0.06475341 | 120.877777 | 215.6322709 |
| YBL029W | YBL029W | 0.77977435 | 0.97903138 | 0.06521456 | 129.292474 | 231.3354769 |
| YBR012C | YBR012C | 0.88270675 | 0.98910891 | 0.06534566 | 105.7718001 | 187.199068 |
| YPL239W | YAR1 | 0.79462917 | 0.98238124 | 0.06565802 | 82.77065861 | 144.0060495 |
| YML103C | NUP188 | 0.8605487 | 0.98660791 | 0.06581836 | 89.68018433 | 156.9377132 |
| YDR163W | CWC15 | 0.52739855 | 0.95675879 | 0.06581961 | 119.4085855 | 212.6943219 |
| YJR103W | URA8 | 0.65088686 | 0.96910824 | 0.06582695 | 114.1707726 | 202.8693356 |
| YOR354C | MSC6 | 0.61362122 | 0.96098984 | 0.06592388 | 117.0159455 | 208.1889883 |
| YLR165C | PUS5 | 0.58665986 | 0.95959383 | 0.06596657 | 96.60073978 | 169.892138 |
| YDR423C | CAD1 | 0.63172164 | 0.96098984 | 0.06628156 | 113.63922 | 201.794606 |
| YLR338W | YLR338W | 0.606799 | 0.95959383 | 0.0663368 | 86.70710755 | 151.2728851 |
| YPR195C | YPR195C | 0.69663722 | 0.97267176 | 0.06638221 | 108.3901218 | 191.9324886 |
| YGR239C | PEX21 | 0.8102024 | 0.9824616 | 0.06659496 | 114.6712022 | 203.676509 |
| YLR250W | SSP120 | 0.68338719 | 0.96992497 | 0.06660217 | 125.0503782 | 223.1418418 |
| YLR149C | YLR149C | 0.62314357 | 0.96098984 | 0.06668265 | 108.418 | 191.9333716 |
| YOR067C | ALG8 | 0.66346381 | 0.96992497 | 0.06679093 | 110.1656723 | 195.192677 |
| YPR146C | YPR146C | 0.69168249 | 0.97061589 | 0.06702058 | 114.9620007 | 204.1490934 |
| YMR096W | SNZ1 | 0.62739327 | 0.96098984 | 0.06716816 | 115.3636281 | 204.8771114 |
| YPR017C | DSS4 | 0.6707016 | 0.96992497 | 0.06719669 | 104.5643504 | 184.6177473 |
| YBR207W | FTH1 | 0.46739609 | 0.95675879 | 0.06727147 | 122.4842864 | 218.2145199 |
| YKL006W | RPL14A | 0.53649259 | 0.95675879 | 0.06742797 | 13.81746981 | 14.37871888 |
| YEL040W | UTR2 | 0.75126659 | 0.97903138 | 0.06742826 | 128.307831 | 229.1099873 |
| YDR070C | FMP16 | 0.75645233 | 0.97903138 | 0.06743293 | 132.4628822 | 236.9021554 |
| YPR147C | YPR147C | 0.58836884 | 0.95959383 | 0.06748955 | 103.872341 | 183.2697477 |
| YML013C-A | YML013C-A | 0.71545419 | 0.97436102 | 0.06753759 | 71.27758422 | 122.1287399 |
| YER075C | PTP3 | 0.49912355 | 0.95675879 | 0.06755333 | 106.1887694 | 187.6033915 |
| YJL112W | MDV1 | 0.47377889 | 0.95675879 | 0.06801432 | 114.3332816 | 202.7998831 |
| YIL039W | YIL039W | 0.5400493 | 0.95675879 | 0.06807831 | 98.21931473 | 172.5665354 |
| YDR101C | ARX1 | 0.5372923 | 0.95675879 | 0.06835683 | 94.04642176 | 164.6924523 |
| YDL156W | YDL156W | 0.60424665 | 0.95959383 | 0.06837763 | 126.9634795 | 226.4261709 |
| YJR019C | TES1 | 0.72091889 | 0.97532873 | 0.06838584 | 133.0457717 | 237.8323518 |
| YHR127W | YHR127W | 0.61420445 | 0.96098984 | 0.06847892 | 110.5547796 | 195.633659 |

| | | | | | | |
|---|---|---|---|---|---|---|
| YBR286W | APE3 | 0.89718779 | 0.98910891 | 0.06856924 | 124.9206803 | 222.5620349 |
| YDL224C | WHI4 | 0.74651678 | 0.97903138 | 0.06859519 | 132.3885643 | 236.5639144 |
| YNR015W | SMM1 | 0.56706565 | 0.95959383 | 0.06862448 | 93.93710463 | 164.4416295 |
| YNL105W | YNL105W | 0.64458937 | 0.96617034 | 0.06870067 | 96.53941081 | 169.3093258 |
| YLR028C | ADE16 | 0.59764789 | 0.95959383 | 0.06890408 | 99.83144032 | 175.4488575 |
| YIL157C | FMP35 | 0.76009097 | 0.97903138 | 0.06898505 | 134.1447124 | 239.7909394 |
| YMR120C | ADE17 | 0.67583378 | 0.96992497 | 0.06903325 | 124.3995062 | 221.5051623 |
| YLR278C | YLR278C | 0.60558536 | 0.95959383 | 0.0690654 | 111.2110548 | 196.7641864 |
| YKL179C | COY1 | 0.65792085 | 0.96992497 | 0.06908278 | 105.5588817 | 186.1603331 |
| YGL015C | YGL015C | 0.45935764 | 0.95675879 | 0.06911263 | 134.4660147 | 240.3717259 |
| YAR030C | YAR030C | 0.69034261 | 0.96992497 | 0.06915518 | 126.75025 | 225.8932159 |
| YNL191W | DUG3 | 0.44082826 | 0.95675879 | 0.06918135 | 100.4128728 | 176.4919192 |
| YGL224C | SDT1 | 0.74727196 | 0.97903138 | 0.06920742 | 126.5331681 | 225.4771319 |
| YLL054C | YLL054C | 0.56019607 | 0.95959383 | 0.06921315 | 105.089778 | 185.2582044 |
| YDR250C | YDR250C | 0.70868733 | 0.97267176 | 0.06926742 | 127.8544871 | 227.9450538 |
| YIR017C | MET28 | 0.70134862 | 0.97267176 | 0.06928851 | 127.7021665 | 227.655762 |
| YGR266W | YGR266W | 0.76605712 | 0.97903138 | 0.0693272 | 133.0729427 | 237.722251 |
| YDR011W | SNQ2 | 0.72033861 | 0.97530288 | 0.06942695 | 128.2875539 | 228.729994 |
| YPL047W | SGF11 | 0.62311634 | 0.96098984 | 0.06972837 | 96.38253086 | 168.8392579 |
| YGR233C | PHO81 | 0.6179098 | 0.96098984 | 0.06988439 | 117.3928816 | 208.2183285 |
| YKR012C | YKR012C | 0.70677262 | 0.97267176 | 0.07008502 | 98.27389322 | 172.3255631 |
| YMR011W | HXT2 | 0.57820475 | 0.95959383 | 0.070111 | 121.8210567 | 216.4847797 |
| YDR502C | SAM2 | 0.70589895 | 0.97267176 | 0.07014712 | 109.2253489 | 192.8548388 |
| YAL060W | BDH1 | 0.70212421 | 0.97267176 | 0.07026878 | 125.6518653 | 223.6426202 |
| YLR247C | IRC20 | 0.67074975 | 0.96992497 | 0.07028057 | 116.6465235 | 206.7507201 |
| YPR070W | MED1 | 0.39100222 | 0.95675879 | 0.07031937 | 6.414794158 | 0 |
| YER120W | SCS2 | 0.46812461 | 0.95675879 | 0.07066343 | 103.97108 | 182.9119058 |
| YLR422W | YLR422W | 0.22888398 | 0.95675879 | 0.0707396 | 113.6693017 | 201.0882824 |
| YER011W | TIR1 | 0.66254691 | 0.96992497 | 0.07084903 | 131.9319102 | 235.3218224 |
| YLR311C | YLR311C | 0.69051249 | 0.96992497 | 0.07093197 | 123.6558712 | 219.7855857 |
| YGL164C | YRB30 | 0.57008512 | 0.95959383 | 0.07097059 | 129.7734922 | 231.2528252 |
| YFL047W | RGD2 | 0.77790079 | 0.97903138 | 0.07097616 | 133.4357677 | 238.1206183 |
| YBR019C | GAL10 | 0.64397133 | 0.96617034 | 0.07119705 | 122.108622 | 216.838304 |
| YDR477W | SNF1 | 0.78625519 | 0.97903138 | 0.07133031 | 101.3592227 | 177.8991637 |
| YJL120W | YJL120W | 0.80867998 | 0.9824616 | 0.07140819 | 96.71994753 | 169.1846899 |
| YNL087W | TCB2 | 0.65516551 | 0.96992497 | 0.07142687 | 114.0220608 | 201.6323085 |
| YBR287W | ZSP1 | 0.50240139 | 0.95675879 | 0.07145677 | 140.0540323 | 250.4512112 |
| YLR054C | OSW2 | 0.67910434 | 0.96992497 | 0.07145796 | 106.4445624 | 187.4150846 |
| YOR355W | GDS1 | 0.68405258 | 0.96992497 | 0.07160248 | 111.7111121 | 197.2679848 |
| YBL061C | SKT5 | 0.60150183 | 0.95959383 | 0.07170968 | 125.9550378 | 223.9647044 |
| YEL005C | VAB2 | 0.60211435 | 0.95959383 | 0.0717694 | 131.4517383 | 234.2637709 |
| YLR273C | PIG1 | 0.6175722 | 0.96098984 | 0.07233055 | 121.8947641 | 216.243269 |
| YDL123W | SNA4 | 0.57894186 | 0.95959383 | 0.07236978 | 130.8844634 | 233.0971039 |
| YLR120C | YPS1 | 0.59669976 | 0.95959383 | 0.07240114 | 106.8240394 | 187.9654368 |
| YJL144W | YJL144W | 0.89357521 | 0.98910891 | 0.07243045 | 96.13351735 | 167.9099132 |
| YMR162C | DNF3 | 0.60151947 | 0.95959383 | 0.07243231 | 109.471784 | 192.9260563 |
| YJL103C | GSM1 | 0.70348653 | 0.97267176 | 0.07245959 | 108.400929 | 190.9129556 |
| YLR112W | YLR112W | 0.69822381 | 0.97267176 | 0.07248287 | 119.2736319 | 211.3011691 |
| YJL106W | IME2 | 0.53558276 | 0.95675879 | 0.07333984 | 106.2524942 | 186.7328744 |
| YBL103C | RTG3 | 0.70339872 | 0.97267176 | 0.07335088 | 103.5940545 | 181.744973 |
| YGL229C | SAP4 | 0.46058125 | 0.95675879 | 0.07340206 | 118.6263372 | 209.9298729 |
| YJR037W | YJR037W | 0.7807974 | 0.97903138 | 0.07343724 | 138.1880819 | 246.612701 |
| YJL073W | JEM1 | 0.64662934 | 0.96742126 | 0.07353737 | 120.2088699 | 212.8748265 |
| YJR047C | ANB1 | 0.70342156 | 0.97267176 | 0.07359587 | 127.1113193 | 225.8106419 |
| YIR005W | IST3 | 0.84509401 | 0.98556047 | 0.0737382 | 86.1874998 | 149.0320068 |
| YBL068W | PRS4 | 0.63086677 | 0.96098984 | 0.07377432 | 126.6477631 | 224.9106922 |
| YKL160W | ELF1 | 0.49017396 | 0.95675879 | 0.07380615 | 102.6141489 | 179.829227 |
| YPR069C | SPE3 | 0.8228181 | 0.9824616 | 0.07380672 | 35.49065353 | 53.93629026 |
| YJR100C | YJR100C | 0.5324651 | 0.95675879 | 0.07384648 | 100.9388275 | 176.6801943 |
| YNL327W | EGT2 | 0.65834479 | 0.96992497 | 0.07387879 | 119.1806483 | 210.8879416 |
| YJR051W | OSM1 | 0.6771717 | 0.96992497 | 0.07390453 | 132.8275309 | 236.4788181 |
| YDR184C | ATC1 | 0.68969558 | 0.96992497 | 0.07403209 | 115.0597429 | 203.1327874 |
| YNL204C | SPS18 | 0.49579515 | 0.95675879 | 0.0741855 | 104.4218995 | 183.1548321 |
| YPR015C | YPR015C | 0.56468876 | 0.95959383 | 0.07431702 | 112.588927 | 198.4499215 |

| | | | | | | |
|---|---|---|---|---|---|---|
| YCL039W | GID7 | 0.47135853 | 0.95675879 | 0.07442107 | 118.8089855 | 210.0980909 |
| YGR016W | YGR016W | 0.58953953 | 0.95959383 | 0.07453752 | 128.1757289 | 227.6458745 |
| YDL066W | IDP1 | 0.59483522 | 0.95959383 | 0.07457171 | 132.0347532 | 234.877782 |
| YBL055C | YBL055C | 0.68897839 | 0.96992497 | 0.07459338 | 125.6144476 | 222.8325303 |
| YJR096W | YJR096W | 0.63402054 | 0.96098984 | 0.07481684 | 116.9200104 | 206.4875279 |
| YJR031C | GEA1 | 0.59718548 | 0.95959383 | 0.07497664 | 118.686451 | 209.7732182 |
| YNL123W | NMA111 | 0.55652907 | 0.95959383 | 0.07504919 | 100.6321803 | 175.8992883 |
| YJL020C | BBC1 | 0.5731171 | 0.95959383 | 0.07524197 | 119.3934567 | 211.0538392 |
| YLR073C | YLR073C | 0.57535657 | 0.95959383 | 0.07540824 | 111.150321 | 195.5650571 |
| YFL012W | YFL012W | 0.73254452 | 0.97731085 | 0.07543439 | 116.1445082 | 204.9273838 |
| YDL226C | GCS1 | 0.82991995 | 0.9824616 | 0.07550949 | 85.77980362 | 147.9642983 |
| YDL215C | GDH2 | 0.62782858 | 0.96098984 | 0.07560416 | 117.2718371 | 207.0126885 |
| YHR137W | ARO9 | 0.46882617 | 0.95675879 | 0.07582264 | 113.3847843 | 199.6849825 |
| YLR118C | YLR118C | 0.38252237 | 0.95675879 | 0.07619465 | 105.2178326 | 184.3038845 |
| YPL274W | SAM3 | 0.48822048 | 0.95675879 | 0.07649294 | 104.6104394 | 183.1136586 |
| YAR018C | KIN3 | 0.6094234 | 0.95959383 | 0.07656929 | 128.2885818 | 227.5099125 |
| YBR164C | ARL1 | 0.62469868 | 0.96098984 | 0.07665246 | 117.7733334 | 207.7739078 |
| YBR299W | MAL32 | 0.6167683 | 0.96098984 | 0.07700636 | 111.9393869 | 196.7715536 |
| YOR007C | SGT2 | 0.55483496 | 0.95959383 | 0.07704198 | 123.6770189 | 218.7798639 |
| YLR205C | HMX1 | 0.59587997 | 0.95959383 | 0.07705606 | 112.1348282 | 197.1296086 |
| YOL114C | YOL114C | 0.64785083 | 0.96786115 | 0.07715598 | 119.564283 | 211.0467562 |
| YDL079C | MRK1 | 0.64857619 | 0.96801503 | 0.07729233 | 134.5317818 | 239.0955797 |
| YPR157W | YPR157W | 0.64265201 | 0.96617034 | 0.07740167 | 107.2636551 | 187.9343939 |
| YLR018C | POM34 | 0.62213806 | 0.96098984 | 0.07780088 | 107.9519676 | 189.1570493 |
| YKR040C | YKR040C | 0.63764128 | 0.9638299 | 0.07781186 | 102.0392136 | 178.0655614 |
| YIL025C | YIL025C | 0.62662027 | 0.96098984 | 0.07787969 | 124.5659246 | 220.3037162 |
| YCR011C | ADP1 | 0.62391043 | 0.96098984 | 0.07806024 | 135.4466987 | 240.6801574 |
| YGL058W | RAD6 | 0.53157118 | 0.95675879 | 0.07815943 | 125.268256 | 221.5731046 |
| YHR006W | STP2 | 0.58587907 | 0.95959383 | 0.07817714 | 120.5294217 | 212.6821994 |
| YMR303C | ADH2 | 0.44834338 | 0.95675879 | 0.07821627 | 107.6669733 | 188.5514609 |
| YBR186W | PCH2 | 0.73340485 | 0.97731085 | 0.07851447 | 134.6654818 | 239.1372379 |
| YLR084C | RAX2 | 0.58162687 | 0.95959383 | 0.07869674 | 121.0257508 | 213.524185 |
| YDL124W | YDL124W | 0.56220127 | 0.95959383 | 0.07885309 | 124.9053718 | 220.7738205 |
| YGL043W | DST1 | 0.85986456 | 0.98660791 | 0.07899558 | 108.1362694 | 189.298309 |
| YGR271W | SLH1 | 0.69038409 | 0.96992497 | 0.07900177 | 115.2242767 | 202.5910951 |
| YBL047C | EDE1 | 0.5756962 | 0.95959383 | 0.07902485 | 120.1107324 | 211.751892 |
| YML001W | YPT7 | 0.81962069 | 0.9824616 | 0.07920899 | 78.82247623 | 134.2825872 |
| YKR089C | TGL4 | 0.6325417 | 0.96098984 | 0.07941246 | 108.0406701 | 189.0476834 |
| YHR131C | YHR131C | 0.64473404 | 0.96617034 | 0.07942994 | 117.7489722 | 207.2530068 |
| YKL221W | MCH2 | 0.56960747 | 0.95959383 | 0.07948836 | 96.31738519 | 167.0472005 |
| YNL266W | YNL266W | 0.64301043 | 0.96617034 | 0.079531 | 123.7157286 | 218.4266094 |
| YLL047W | YLL047W | 0.5532634 | 0.95959383 | 0.08006416 | 112.8893003 | 198.0299836 |
| YIL043C | CBR1 | 0.70115408 | 0.97267176 | 0.0800877 | 122.5897732 | 216.2195878 |
| YKL114C | APN1 | 0.60656318 | 0.95959383 | 0.08018473 | 108.9330827 | 190.5893079 |
| YEL038W | UTR4 | 0.37707257 | 0.95675879 | 0.08024938 | 114.7647325 | 201.515743 |
| YMR136W | GAT2 | 0.47351882 | 0.95675879 | 0.08026252 | 115.3665535 | 202.6422343 |
| YIR025W | MND2 | 0.76859757 | 0.97903138 | 0.08041777 | 131.3312292 | 232.5580696 |
| YGL087C | MMS2 | 0.62546784 | 0.96098984 | 0.08050304 | 127.4842161 | 225.328251 |
| YDR020C | YDR020C | 0.4096323 | 0.95675879 | 0.08062144 | 118.0378857 | 207.5910183 |
| YBL104C | YBL104C | 0.68012599 | 0.96992497 | 0.08067512 | 141.3516062 | 251.3076625 |
| YBL070C | YBL070C | 0.55574345 | 0.95959383 | 0.08077169 | 124.4657036 | 219.6209445 |
| YPL214C | THI6 | 0.65495895 | 0.96992497 | 0.08093274 | 110.1594669 | 192.7614615 |
| YIL116W | HIS5 | 0.60395677 | 0.95959383 | 0.08097871 | 124.8039116 | 220.2198471 |
| YHR106W | TRR2 | 0.77396125 | 0.97903138 | 0.08119196 | 135.4148452 | 240.0845979 |
| YLR151C | PCD1 | 0.65700509 | 0.96992497 | 0.08121012 | 116.4290431 | 204.4728481 |
| YOL025W | LAG2 | 0.69578053 | 0.97267176 | 0.08141566 | 128.8396172 | 227.7142178 |
| YLR292C | SEC72 | 0.54081768 | 0.95675879 | 0.08151712 | 101.5335868 | 176.4832896 |
| YNL192W | CHS1 | 0.63310238 | 0.96098984 | 0.08168631 | 109.6706386 | 191.7157142 |
| YHR156C | LIN1 | 0.72644647 | 0.97673059 | 0.08194711 | 107.4090948 | 187.4294756 |
| YGR068C | YGR068C | 0.57323189 | 0.95959383 | 0.08197689 | 109.3288905 | 191.0250346 |
| YLL014W | YLL014W | 0.55139478 | 0.95959383 | 0.08200531 | 103.698882 | 180.4608623 |
| YJR030C | YJR030C | 0.66887443 | 0.96992497 | 0.08202583 | 130.6170204 | 230.9434134 |
| YGR054W | YGR054W | 0.55777774 | 0.95959383 | 0.08204875 | 123.7794193 | 218.1152938 |
| YIL029C | YIL029C | 0.81053271 | 0.9824616 | 0.08209888 | 103.195156 | 179.5000959 |

| | | | | | | |
|---|---|---|---|---|---|---|
| YFL025C | BST1 | 0.39211112 | 0.95675879 | 0.08221349 | 103.2395702 | 179.5637858 |
| YIL120W | QDR1 | 0.63219796 | 0.96098984 | 0.08227069 | 118.002118 | 207.2417573 |
| YGR200C | ELP2 | 0.82955391 | 0.9824616 | 0.08239535 | 93.44380599 | 161.1603188 |
| YAL007C | ERP2 | 0.53851572 | 0.95675879 | 0.08251172 | 122.436502 | 215.5173861 |
| YLR020C | YEH2 | 0.52167761 | 0.95675879 | 0.08251928 | 105.1321708 | 183.0611176 |
| YDR116C | MRPL1 | 0.67286751 | 0.96992497 | 0.08258893 | 112.2745046 | 196.4449365 |
| YCL037C | SRO9 | 0.82603161 | 0.9824616 | 0.08275906 | 93.59510053 | 161.3818486 |
| YNL326C | PFA3 | 0.38751043 | 0.95675879 | 0.08276433 | 92.02370218 | 158.4337261 |
| YER089C | PTC2 | 0.68128695 | 0.96992497 | 0.0830255 | 109.0668002 | 190.3540631 |
| YIR018W | YAP5 | 0.6397936 | 0.96463263 | 0.08302644 | 113.9452364 | 199.5036075 |
| YLR209C | PNP1 | 0.58205697 | 0.95959383 | 0.08315249 | 105.204614 | 183.0886486 |
| YBR177C | EHT1 | 0.71953654 | 0.97530288 | 0.08315444 | 133.9178995 | 236.9412482 |
| YOR009W | TIR4 | 0.67360775 | 0.96992497 | 0.08326588 | 128.1475723 | 226.0996983 |
| YFL030W | AGX1 | 0.55203632 | 0.95959383 | 0.08340081 | 111.06958 | 194.0461453 |
| YMR310C | YMR310C | 0.53177702 | 0.95675879 | 0.08340971 | 113.5758381 | 198.7452101 |
| YOL029C | YOL029C | 0.68036705 | 0.96992497 | 0.08364256 | 130.3368967 | 230.1414178 |
| YGL236C | MTO1 | 0.24962869 | 0.95675879 | 0.0837736 | 133.4856872 | 236.0246815 |
| YNL284C | MRPL10 | 0.55086905 | 0.95947263 | 0.08378239 | 115.286042 | 201.8890036 |
| YKL026C | GPX1 | 0.56525666 | 0.95959383 | 0.08383779 | 115.086151 | 201.5046224 |
| YML131W | YML131W | 0.56937353 | 0.95959383 | 0.0838436 | 116.1811156 | 203.557278 |
| YKL001C | MET14 | 0.58426504 | 0.95959383 | 0.0839341 | 111.8483856 | 195.4155843 |
| YDL173W | YDL173W | 0.76796918 | 0.97903138 | 0.08398235 | 115.8782686 | 202.965538 |
| YKR061W | KTR2 | 0.59365862 | 0.95959383 | 0.08401751 | 105.3872961 | 183.283277 |
| YGL039W | YGL039W | 0.5078624 | 0.95675879 | 0.08421604 | 138.4940969 | 245.3424589 |
| YIL103W | DPH1 | 0.65573628 | 0.96992497 | 0.08423628 | 118.4217737 | 207.6925388 |
| YLR031W | YLR031W | 0.5900411 | 0.95959383 | 0.08439049 | 113.5032162 | 198.4412007 |
| YMR135C | GID8 | 0.60775103 | 0.95959383 | 0.0844249 | 106.3930942 | 185.0999911 |
| YLR356W | YLR356W | 0.47354957 | 0.95675879 | 0.08446487 | 106.20571 | 184.7417054 |
| YOR235W | YOR235W | 0.67356454 | 0.96992497 | 0.08449567 | 94.00221902 | 161.8482921 |
| YFR047C | BNA6 | 0.43116126 | 0.95675879 | 0.08455995 | 135.6817511 | 240.0089489 |
| YGL159W | YGL159W | 0.67050787 | 0.96992497 | 0.08459362 | 135.950376 | 240.5070056 |
| YLR287C | YLR287C | 0.51615396 | 0.95675879 | 0.0847304 | 115.3822851 | 201.9073133 |
| YKL178C | STE3 | 0.54128269 | 0.95675879 | 0.08498732 | 108.5946178 | 189.1328115 |
| YBR103W | SIF2 | 0.33176954 | 0.95675879 | 0.08499107 | 96.96638956 | 167.3229547 |
| YPL037C | EGD1 | 0.68233386 | 0.96992497 | 0.08502953 | 121.6624117 | 213.6347671 |
| YGL158W | RCK1 | 0.35320592 | 0.95675879 | 0.08516852 | 115.6531629 | 202.3403963 |
| YBL015W | ACH1 | 0.60106076 | 0.95959383 | 0.08537029 | 130.4319407 | 230.0240725 |
| YIL030C | SSM4 | 0.62551777 | 0.96098984 | 0.08554916 | 122.2901908 | 214.7232854 |
| YKL093W | MBR1 | 0.59487766 | 0.95959383 | 0.08557315 | 101.3315382 | 175.410378 |
| YGR157W | CHO2 | 0.66629462 | 0.96992497 | 0.08560203 | 15.94943429 | 15.26783634 |
| YJR034W | PET191 | 0.59491966 | 0.95959383 | 0.0856293 | 114.2378779 | 199.6071361 |
| YDR178W | SDH4 | 0.73423394 | 0.97731085 | 0.08572457 | 113.386919 | 197.994827 |
| YLR251W | SYM1 | 0.51876179 | 0.95675879 | 0.08587677 | 105.7676495 | 183.6785383 |
| YBR126C | TPS1 | 0.82366227 | 0.9824616 | 0.08603909 | 96.20341322 | 165.7126522 |
| YNR006W | VPS27 | 0.69896915 | 0.97267176 | 0.08611302 | 79.87448141 | 135.0744292 |
| YGR268C | HUA1 | 0.68366605 | 0.96992497 | 0.08623875 | 130.6968326 | 230.372299 |
| YGL080W | FMP37 | 0.81634751 | 0.9824616 | 0.08664094 | 96.70021844 | 166.5414586 |
| YIL110W | MNI1 | 0.74148729 | 0.97903138 | 0.08670088 | 79.95370256 | 135.1224323 |
| YGR287C | YGR287C | 0.70877324 | 0.97267176 | 0.08678708 | 119.8865615 | 210.003381 |
| YJR049C | UTR1 | 0.75543562 | 0.97903138 | 0.08700143 | 116.8043554 | 204.1859054 |
| YMR204C | INP1 | 0.49700051 | 0.95675879 | 0.08713545 | 107.0833384 | 185.9308135 |
| YDL041W | YDL041W | 0.56879526 | 0.95959383 | 0.08720502 | 132.6289577 | 233.8307563 |
| YGR105W | VMA21 | 0.8347728 | 0.98249094 | 0.08758279 | 43.43922722 | 66.48716248 |
| YGR164W | YGR164W | 0.5690486 | 0.95959383 | 0.08762031 | 120.6449511 | 211.283211 |
| YAL045C | YAL045C | 0.59476893 | 0.95959383 | 0.08787488 | 128.0190399 | 225.0700595 |
| YKL037W | YKL037W | 0.22689223 | 0.95675879 | 0.08789307 | 98.19290912 | 169.1268285 |
| YBL067C | UBP13 | 0.53817305 | 0.95675879 | 0.08790135 | 125.3894215 | 220.1335741 |
| YLR242C | ARV1 | 0.48794217 | 0.95675879 | 0.088327 | 81.1564378 | 137.0999918 |
| YGR168C | YGR168C | 0.81551049 | 0.9824616 | 0.08834742 | 91.37703136 | 156.2656361 |
| YHL013C | OTU2 | 0.75608358 | 0.97903138 | 0.08851316 | 103.9365071 | 179.7930857 |
| YGR236C | SPG1 | 0.65956998 | 0.96992497 | 0.08864927 | 123.2274275 | 215.9507016 |
| YMR003W | YMR003W | 0.5667354 | 0.95959383 | 0.08871491 | 127.6641744 | 224.2607697 |
| YDL119C | YDL119C | 0.6755398 | 0.96992497 | 0.08875198 | 101.5524374 | 175.2808064 |
| YML030W | YML030W | 0.26359375 | 0.95675879 | 0.08878498 | 100.7775151 | 173.821761 |

| | | | | | | |
|---|---|---|---|---|---|---|
| YMR153C-A | YMR153C-A | 0.67122368 | 0.96992497 | 0.08909889 | 110.8507208 | 192.6607586 |
| YML106W | URA5 | 0.37435149 | 0.95675879 | 0.08917178 | 119.5179423 | 208.9040135 |
| YGL118C | YGL118C | 0.67601164 | 0.96992497 | 0.08920445 | 127.8497186 | 224.5250093 |
| YGR223C | HSV2 | 0.59064777 | 0.95959383 | 0.08924894 | 92.07379975 | 157.4182101 |
| YPR106W | ISR1 | 0.3973745 | 0.95675879 | 0.08934822 | 102.248559 | 176.4843969 |
| YPR158W | YPR158W | 0.54198149 | 0.95710067 | 0.08937511 | 111.1987142 | 193.2661749 |
| YNL008C | ASI3 | 0.53315553 | 0.95675879 | 0.08942101 | 110.8244884 | 192.5564474 |
| YAL029C | MYO4 | 0.50714208 | 0.95675879 | 0.08943566 | 121.3421112 | 212.2801681 |
| YIR014W | YIR014W | 0.68850656 | 0.96992497 | 0.08946696 | 128.402453 | 225.5167703 |
| YPR171W | BSP1 | 0.53825995 | 0.95675879 | 0.08960245 | 112.5548598 | 195.770785 |
| YGR272C | YGR272C | 0.46280678 | 0.95675879 | 0.08971489 | 108.7496375 | 188.6146979 |
| YMR289W | ABZ2 | 0.45062152 | 0.95675879 | 0.08972243 | 104.2360799 | 180.1480484 |
| YLR354C | TAL1 | 0.51370216 | 0.95675879 | 0.08981266 | 103.5750181 | 178.8927619 |
| YPR093C | ASR1 | 0.58834663 | 0.95959383 | 0.09012493 | 113.5159651 | 197.4839849 |
| YLR384C | IKI3 | 0.81517908 | 0.9824616 | 0.09038779 | 93.56328692 | 160.0169536 |
| YJL183W | MNN11 | 0.85968139 | 0.98660791 | 0.09039955 | 119.8852062 | 209.3827677 |
| YGL258W | VEL1 | 0.72540329 | 0.97673059 | 0.09055947 | 132.175423 | 232.4062082 |
| YDR371W | CTS2 | 0.67534757 | 0.96992497 | 0.09124092 | 123.2667516 | 215.5810403 |
| YPR051W | MAK3 | 0.76728298 | 0.97903138 | 0.09134436 | 109.1403781 | 189.0687566 |
| YIL024C | YIL024C | 0.6542251 | 0.96992497 | 0.09135096 | 120.3088618 | 210.0145714 |
| YJR038C | YJR038C | 0.51706342 | 0.95675879 | 0.09146234 | 113.4054743 | 197.0479311 |
| YIR009W | MSL1 | 0.59563085 | 0.95959383 | 0.0915808 | 121.9763051 | 213.1026053 |
| YCR076C | YCR076C | 0.51778135 | 0.95675879 | 0.09163995 | 138.7332355 | 244.5207879 |
| YNL300W | YNL300W | 0.44602278 | 0.95675879 | 0.09166066 | 96.44439349 | 165.202805 |
| YGL004C | RPN14 | 0.41387358 | 0.95675879 | 0.09171502 | 119.5806288 | 208.5864529 |
| YBR057C | MUM2 | 0.70952125 | 0.97267176 | 0.09178513 | 115.5582101 | 201.0302467 |
| YLR412W | YLR412W | 0.50204596 | 0.95675879 | 0.0917991 | 101.5405991 | 174.7372597 |
| YHR206W | SKN7 | 0.69332457 | 0.97133302 | 0.09185222 | 127.3734442 | 223.1787196 |
| YNL311C | SKP2 | 0.52675441 | 0.95675879 | 0.09231365 | 102.7660949 | 176.9476895 |
| YGR228W | YGR228W | 0.55328067 | 0.95959383 | 0.09248563 | 118.9129668 | 207.2023793 |
| YLR190W | MMR1 | 0.66413597 | 0.96992497 | 0.09259196 | 79.13794965 | 132.5845275 |
| YFR038W | IRC5 | 0.50554366 | 0.95675879 | 0.09303176 | 126.3185546 | 220.9984193 |
| YPR098C | YPR098C | 0.54663032 | 0.95854866 | 0.09309141 | 107.6938496 | 186.0568232 |
| YLR221C | RSA3 | 0.54325748 | 0.95761397 | 0.09310866 | 104.2921115 | 179.6737747 |
| YLR408C | YLR408C | 0.48048062 | 0.95675879 | 0.09311945 | 105.080145 | 181.1499168 |
| YGL180W | ATG1 | 0.42512041 | 0.95675879 | 0.09319679 | 131.4459543 | 230.5868311 |
| YGL115W | SNF4 | 0.80480113 | 0.9824616 | 0.0932435 | 71.48263563 | 118.1152015 |
| YGR238C | KEL2 | 0.81606638 | 0.9824616 | 0.09331556 | 117.7845688 | 204.9440273 |
| YLR461W | PAU4 | 0.47714526 | 0.95675879 | 0.09344939 | 112.5141601 | 195.0362636 |
| YGR143W | SKN1 | 0.5756173 | 0.95959383 | 0.0934866 | 131.3148762 | 230.2914033 |
| YBR145W | ADH5 | 0.56722753 | 0.95959383 | 0.09352555 | 127.6019639 | 223.3210221 |
| YLR146C | SPE4 | 0.76575284 | 0.97903138 | 0.09372623 | 100.0464136 | 171.6051329 |
| YDR233C | RTN1 | 0.5475308 | 0.9588488 | 0.09376445 | 129.2399854 | 226.3523224 |
| YBR031W | RPL4A | 0.75288809 | 0.97903138 | 0.09382798 | 103.0271351 | 177.1781892 |
| YNL296W | YNL296W | 0.62108738 | 0.96098984 | 0.09421305 | 97.96582492 | 167.619613 |
| YKL079W | SMY1 | 0.46485608 | 0.95675879 | 0.09427234 | 110.1742359 | 190.5068393 |
| YML052W | SUR7 | 0.51058596 | 0.95675879 | 0.09448224 | 104.8833943 | 180.5477386 |
| YPR111W | DBF20 | 0.49852772 | 0.95675879 | 0.09454278 | 109.5673148 | 189.3222641 |
| YLR370C | ARC18 | 0.50180448 | 0.95675879 | 0.09461904 | 106.8785206 | 184.2662741 |
| YBR250W | YBR250W | 0.49818133 | 0.95675879 | 0.09470801 | 129.2370673 | 226.1854128 |
| YCL027W | FUS1 | 0.49390099 | 0.95675879 | 0.09474436 | 138.4607697 | 243.478622 |
| YLL038C | ENT4 | 0.43719897 | 0.95675879 | 0.09475349 | 104.1096942 | 179.0502232 |
| YBR125C | PTC4 | 0.58762896 | 0.95959383 | 0.09475956 | 135.1557054 | 237.2772392 |
| YJR070C | LIA1 | 0.19199623 | 0.95675879 | 0.09501332 | 105.3908772 | 181.4086787 |
| YLR325C | RPL38 | 0.48156112 | 0.95675879 | 0.09522103 | 101.4728019 | 174.0246316 |
| YML111W | BUL2 | 0.49990928 | 0.95675879 | 0.09536059 | 11.22515219 | 4.737625159 |
| YPL120W | VPS30 | 0.55750461 | 0.95959383 | 0.09542675 | 102.581348 | 176.0685574 |
| YOR131C | YOR131C | 0.7120628 | 0.97267176 | 0.09573908 | 133.4751798 | 233.9577546 |
| YKR034W | DAL80 | 0.64824608 | 0.96786115 | 0.09618203 | 99.77028641 | 170.667073 |
| YLR104W | YLR104W | 0.55634468 | 0.95959383 | 0.09626497 | 102.082221 | 174.9890112 |
| YKR106W | YKR106W | 0.49125937 | 0.95675879 | 0.09650627 | 126.1429123 | 220.0745286 |
| YBR271W | YBR271W | 0.54514693 | 0.95802746 | 0.09654664 | 140.131114 | 246.3030622 |
| YLR077W | FMP25 | 0.60278402 | 0.95959383 | 0.09666218 | 117.3371121 | 203.5322178 |
| YJR060W | CBF1 | 0.57344748 | 0.95959383 | 0.09704246 | 103.6915335 | 177.8743186 |

| | | | | | | |
|---|---|---|---|---|---|---|
| YDR152W | GIR2 | 0.50111208 | 0.95675879 | 0.09713274 | 118.8955472 | 206.3746156 |
| YLR352W | YLR352W | 0.4599499 | 0.95675879 | 0.09715595 | 105.0985275 | 180.4937746 |
| YKL076C | PSY1 | 0.34048342 | 0.95675879 | 0.09718198 | 101.5011003 | 173.742201 |
| YOR191W | RIS1 | 0.54496289 | 0.95802746 | 0.09727537 | 113.8080113 | 196.8083333 |
| YLL043W | FPS1 | 0.48321606 | 0.95675879 | 0.09759099 | 99.28613249 | 169.5179588 |
| YIL065C | FIS1 | 0.58088946 | 0.95959383 | 0.09769461 | 92.15475498 | 156.1250419 |
| YNL099C | OCA1 | 0.61877424 | 0.96098984 | 0.09775874 | 102.6090484 | 175.7215213 |
| YFL013W-A | YFL013W-A | 0.17568433 | 0.95675879 | 0.09778454 | 118.7000363 | 205.8964077 |
| YFR045W | YFR045W | 0.50441506 | 0.95675879 | 0.09782985 | 127.7364165 | 222.8367541 |
| YLR090W | XDJ1 | 0.42651556 | 0.95675879 | 0.09786508 | 118.0846255 | 204.7283997 |
| YLL007C | YLL007C | 0.49163925 | 0.95675879 | 0.09789348 | 105.8115727 | 181.7049324 |
| YMR025W | CSI1 | 0.38798781 | 0.95675879 | 0.09796 | 116.8213212 | 202.342782 |
| YNL202W | SPS19 | 0.59811121 | 0.95959383 | 0.09842909 | 136.8478413 | 239.8230747 |
| YGL037C | PNC1 | 0.52292733 | 0.95675879 | 0.09846446 | 117.0158511 | 202.6213199 |
| YMR088C | VBA1 | 0.55410828 | 0.95959383 | 0.09855996 | 134.3027024 | 235.0271729 |
| YGR028W | MSP1 | 0.83144184 | 0.9824616 | 0.09886194 | 126.6606354 | 220.6424989 |
| YER020W | GPA2 | 0.33999522 | 0.95675879 | 0.0992516 | 107.9793315 | 185.5382872 |
| YJR130C | STR2 | 0.26911374 | 0.95675879 | 0.09939489 | 105.3266586 | 180.5385756 |
| YBL088C | TEL1 | 0.53060733 | 0.95675879 | 0.09944337 | 130.7821367 | 228.2730627 |
| YJL004C | SYS1 | 0.42904652 | 0.95675879 | 0.09980954 | 107.3234343 | 184.2126649 |
| YER060W-A | FCY22 | 0.79225903 | 0.98180632 | 0.09996563 | 109.7603402 | 188.7564743 |
| YOR306C | MCH5 | 0.72417462 | 0.97653633 | 0.10009335 | 83.43770002 | 139.3654422 |
| YMR294W-A | YMR294W-A | 0.25078844 | 0.95675879 | 0.10021355 | 101.717516 | 173.6294137 |
| YLR083C | EMP70 | 0.53825377 | 0.95675879 | 0.10027903 | 120.4946551 | 208.8354978 |
| YMR189W | GCV2 | 0.52949646 | 0.95675879 | 0.10062711 | 120.6921091 | 209.1462766 |
| YDR085C | AFR1 | 0.71992621 | 0.97530288 | 0.10081817 | 131.4673767 | 229.3230384 |
| YLR091W | YLR091W | 0.40641856 | 0.95675879 | 0.10104838 | 109.382536 | 187.8626353 |
| YGR111W | YGR111W | 0.82039461 | 0.9824616 | 0.10106595 | 105.4318676 | 180.4499904 |
| YPR084W | YPR084W | 0.56951227 | 0.95959383 | 0.10112357 | 114.834232 | 198.0746481 |
| YML119W | YML119W | 0.60557884 | 0.95959383 | 0.10113232 | 131.4713782 | 229.2767932 |
| YBR075W | YBR075W | 0.45555736 | 0.95675879 | 0.10146739 | 118.1835932 | 204.2976851 |
| YPR001W | CIT3 | 0.62479655 | 0.96098984 | 0.10154923 | 115.7872152 | 199.7891784 |
| YLR376C | PSY3 | 0.66357247 | 0.96992497 | 0.10165247 | 118.3619905 | 204.6006107 |
| YMR234W | RNH1 | 0.33964218 | 0.95675879 | 0.10168576 | 109.2692042 | 187.5410255 |
| YGR232W | NAS6 | 0.58329235 | 0.95959383 | 0.10179191 | 122.586152 | 212.4993392 |
| YPR029C | APL4 | 0.60076391 | 0.95959383 | 0.10201577 | 115.7333393 | 199.6083108 |
| YNL195C | YNL195C | 0.33934804 | 0.95675879 | 0.10203181 | 112.1540251 | 192.8924168 |
| YLR232W | YLR232W | 0.34471783 | 0.95675879 | 0.10203779 | 109.7434285 | 188.3702226 |
| YDL134C-A | YDL134C-A | 0.49366899 | 0.95675879 | 0.10207651 | 143.9689374 | 252.5549286 |
| YGL049C | TIF4632 | 0.6171981 | 0.96098984 | 0.10217953 | 119.1082401 | 205.9100564 |
| YBL042C | FUI1 | 0.4843821 | 0.95675879 | 0.10224277 | 131.7361483 | 229.5833894 |
| YDR227W | SIR4 | 0.51021751 | 0.95675879 | 0.10230186 | 120.1962473 | 207.9297282 |
| YGR108W | CLB1 | 0.60112886 | 0.95959383 | 0.10231558 | 136.3364383 | 238.198964 |
| YLR405W | DUS4 | 0.51705194 | 0.95675879 | 0.10263517 | 120.848099 | 209.0952746 |
| YNL179C | SRF6 | 0.45369917 | 0.95675879 | 0.10281843 | 109.952959 | 188.6296426 |
| YBR138C | YBR138C | 0.4898778 | 0.95675879 | 0.10317618 | 123.4956385 | 213.9682783 |
| YLR258W | GSY2 | 0.40856978 | 0.95675879 | 0.10326274 | 106.7391746 | 182.5260403 |
| YJR048W | CYC1 | 0.70749661 | 0.97267176 | 0.10337783 | 125.8738199 | 218.3941535 |
| YLR102C | APC9 | 0.68228786 | 0.96992497 | 0.1034137 | 139.8018078 | 244.510523 |
| YKL128C | PMU1 | 0.53156737 | 0.95675879 | 0.10346909 | 106.7730838 | 182.5543345 |
| YLR041W | YLR041W | 0.52315257 | 0.95675879 | 0.1036365 | 115.2994396 | 198.5172167 |
| YLR021W | IRC25 | 0.80031267 | 0.9824616 | 0.10372887 | 102.0755703 | 173.6995103 |
| YLL005C | SPO75 | 0.43727619 | 0.95675879 | 0.10373104 | 106.0454797 | 181.1448643 |
| YPR021C | AGC1 | 0.44701235 | 0.95675879 | 0.10396853 | 109.0945487 | 186.8228837 |
| YOL024W | YOL024W | 0.63346446 | 0.96098984 | 0.10409347 | 124.4387052 | 215.5800966 |
| YLL017W | YLL017W | 0.40738809 | 0.95675879 | 0.10412612 | 107.8721201 | 184.5032069 |
| YKR100C | SKG1 | 0.53134328 | 0.95675879 | 0.10422112 | 121.4963926 | 210.039829 |
| YKL077W | YKL077W | 0.53302662 | 0.95675879 | 0.1044022 | 134.1451094 | 233.7320287 |
| YKL033W-A | YKL033W-A | 0.41197235 | 0.95675879 | 0.10483502 | 101.0663896 | 171.6174952 |
| YGL109W | YGL109W | 0.51962149 | 0.95675879 | 0.10494658 | 132.1111083 | 229.8240374 |
| YDR291W | HRQ1 | 0.64077041 | 0.96479866 | 0.10496039 | 140.6443129 | 245.8260465 |
| YDR402C | DIT2 | 0.77523143 | 0.97903138 | 0.10507866 | 120.1429651 | 207.3547022 |
| YKL047W | YKL047W | 0.46374145 | 0.95675879 | 0.10528001 | 109.2935802 | 186.9717893 |
| YDR310C | SUM1 | 0.44526813 | 0.95675879 | 0.10540024 | 115.9802742 | 199.4923848 |

| | | | | | | |
|---|---|---|---|---|---|---|
| YCL029C | BIK1 | 0.50760021 | 0.95675879 | 0.10567813 | 99.7160543 | 168.9406355 |
| YJR139C | HOM6 | 0.76214612 | 0.97903138 | 0.10578944 | 84.33453426 | 140.0729259 |
| YDR297W | SUR2 | 0.63314601 | 0.96098984 | 0.10580185 | 139.0870612 | 242.7613895 |
| YLR231C | BNA5 | 0.460894 | 0.95675879 | 0.10583997 | 123.4442197 | 213.4160836 |
| YIL101C | XBP1 | 0.70187835 | 0.97267176 | 0.10606317 | 137.7003506 | 240.1158475 |
| YIR042C | YIR042C | 0.63088944 | 0.96098984 | 0.10607643 | 131.7548032 | 228.9624624 |
| YJR098C | YJR098C | 0.60490802 | 0.95959383 | 0.10611144 | 120.2224343 | 207.3270464 |
| YLR220W | CCC1 | 0.37037218 | 0.95675879 | 0.10622979 | 110.2390685 | 188.5825896 |
| YCL048W | SPS22 | 0.42970823 | 0.95675879 | 0.10659841 | 140.1852234 | 244.6847505 |
| YOR240W | YOR240W | 0.62883007 | 0.96098984 | 0.10661349 | 119.0061643 | 204.9599858 |
| YGL029W | CGR1 | 0.52328804 | 0.95675879 | 0.1069188 | 112.2025746 | 192.1473398 |
| YDR214W | AHA1 | 0.48988185 | 0.95675879 | 0.1069196 | 118.5476549 | 204.0476584 |
| YDL238C | GUD1 | 0.54291542 | 0.95761397 | 0.10695924 | 135.465933 | 235.7717948 |
| YER091C | MET6 | 0.3219924 | 0.95675879 | 0.10710282 | 98.96830371 | 167.2944439 |
| YFR046C | CNN1 | 0.33837476 | 0.95675879 | 0.10731093 | 126.6558153 | 219.1878891 |
| YIL040W | APQ12 | 0.81490084 | 0.9824616 | 0.10762832 | 37.35052365 | 51.63788771 |
| YPR027C | YPR027C | 0.37542172 | 0.95675879 | 0.10785283 | 107.8197835 | 183.7674304 |
| YIL059C | YIL059C | 0.60313594 | 0.95959383 | 0.10792384 | 128.8307269 | 223.1621585 |
| YGR081C | SLX9 | 0.83160503 | 0.9824616 | 0.10809606 | 99.03672738 | 167.2528379 |
| YCL024W | KCC4 | 0.83133075 | 0.9824616 | 0.10809754 | 125.3170035 | 216.5423079 |
| YPR090W | YPR090W | 0.54748777 | 0.9588488 | 0.10810641 | 117.399611 | 201.6914002 |
| YIL131C | FKH1 | 0.52351506 | 0.95675879 | 0.10829529 | 108.9291667 | 185.7724219 |
| YJR003C | YJR003C | 0.53930619 | 0.95675879 | 0.1084858 | 129.0105835 | 223.4033399 |
| YBL032W | HEK2 | 0.5323038 | 0.95675879 | 0.1085138 | 135.0916344 | 234.8038061 |
| YNL170W | YNL170W | 0.74104775 | 0.97903138 | 0.10873816 | 59.7427415 | 93.44551414 |
| YDL177C | YDL177C | 0.4001198 | 0.95675879 | 0.10883818 | 131.3197174 | 227.6739239 |
| YML002W | YML002W | 0.22736994 | 0.95675879 | 0.10890438 | 114.254764 | 195.6565838 |
| YDR354W | TRP4 | 0.42737217 | 0.95675879 | 0.10896627 | 122.4007784 | 210.9241759 |
| YBR249C | ARO4 | 0.49855557 | 0.95675879 | 0.10921238 | 128.7135288 | 222.7218868 |
| YDR036C | EHD3 | 0.42900816 | 0.95675879 | 0.10940417 | 110.3959433 | 188.3336992 |
| YBR267W | REI1 | 0.81738068 | 0.9824616 | 0.10945727 | 44.11060202 | 64.00376689 |
| YNL076W | MKS1 | 0.66983292 | 0.96992497 | 0.10946788 | 94.21613504 | 157.976912 |
| YCL013W | YCL013W | 0.45706799 | 0.95675879 | 0.10948906 | 134.8971064 | 234.2720997 |
| YOR111W | YOR111W | 0.59468044 | 0.95959383 | 0.10987361 | 128.1466406 | 221.5455339 |
| YOR107W | RGS2 | 0.59034452 | 0.95959383 | 0.10992526 | 123.6496562 | 213.1024197 |
| YHR111W | UBA4 | 0.72950024 | 0.97683349 | 0.11009193 | 90.90072077 | 151.6519462 |
| YGR148C | RPL24B | 0.59361803 | 0.95959383 | 0.11011462 | 100.6144172 | 169.8664965 |
| YER046W | SPO73 | 0.22437037 | 0.95675879 | 0.11013261 | 125.0819344 | 215.7532396 |
| YLR143W | YLR143W | 0.38043516 | 0.95675879 | 0.11019611 | 103.9113034 | 176.035997 |
| YML018C | YML018C | 0.16336493 | 0.95675879 | 0.11054076 | 107.9735022 | 183.595849 |
| YOR136W | IDH2 | 0.55351637 | 0.95959383 | 0.11064944 | 129.5081923 | 223.9664386 |
| YIL112W | HOS4 | 0.52782401 | 0.95675879 | 0.11071569 | 107.09774 | 181.923392 |
| YIL056W | VHR1 | 0.51808327 | 0.95675879 | 0.11074741 | 126.3555776 | 218.0368211 |
| YML113W | DAT1 | 0.4174925 | 0.95675879 | 0.11082141 | 111.2118883 | 189.621556 |
| YOR183W | FYV12 | 0.7422614 | 0.97903138 | 0.11097403 | 80.21175137 | 131.4534282 |
| YGR037C | ACB1 | 0.48129078 | 0.95675879 | 0.11114879 | 140.5201409 | 244.5343604 |
| YNL289W | PCL1 | 0.53259854 | 0.95675879 | 0.11121591 | 122.8940724 | 211.464469 |
| YLR107W | REX3 | 0.50708801 | 0.95675879 | 0.1113675 | 107.0957844 | 181.8082029 |
| YJL017W | YJL017W | 0.49202943 | 0.95675879 | 0.11141417 | 114.17468 | 195.0769736 |
| YAL040C | CLN3 | 0.49509449 | 0.95675879 | 0.11150888 | 121.7078577 | 209.1895506 |
| YGL138C | YGL138C | 0.02589797 | 0.76284641 | 0.1115486 | 122.3237266 | 210.3378408 |
| YIL008W | URM1 | 0.78161859 | 0.97903138 | 0.11170982 | 93.33405292 | 155.9389484 |
| YDR357C | YDR357C | 0.56163691 | 0.95959383 | 0.11177641 | 111.5879682 | 190.1635149 |
| YDR281C | PHM6 | 0.59071144 | 0.95959383 | 0.1118318 | 120.8669213 | 207.5570902 |
| YOR177C | MPC54 | 0.52292077 | 0.95675879 | 0.11197593 | 126.5016246 | 218.1005458 |
| YNL101W | AVT4 | 0.61380793 | 0.96098984 | 0.11197727 | 133.4720856 | 231.1736981 |
| YGR106C | YGR106C | 0.35933388 | 0.95675879 | 0.11211433 | 120.2957593 | 206.4375137 |
| YNR002C | ATO2 | 0.75624749 | 0.97903138 | 0.11215922 | 103.3040682 | 174.5612272 |
| YLR109W | AHP1 | 0.46887252 | 0.95675879 | 0.11217142 | 117.3788491 | 200.9569618 |
| YBL016W | FUS3 | 0.75756305 | 0.97903138 | 0.11241938 | 90.55630649 | 150.6077716 |
| YLR271W | YLR271W | 0.50489329 | 0.95675879 | 0.11242 | 110.3960609 | 187.817929 |
| YDL112W | TRM3 | 0.28557453 | 0.95675879 | 0.11288362 | 141.0689475 | 245.2668504 |
| YJR044C | VPS55 | 0.52544642 | 0.95675879 | 0.11290522 | 107.707366 | 182.6921543 |
| YLR319C | BUD6 | 0.48097496 | 0.95675879 | 0.11292102 | 113.1488867 | 192.895244 |

| | | | | | | |
|---|---|---|---|---|---|---|
| YBR084W | MIS1 | 0.49565647 | 0.95675879 | 0.11305565 | 132.6848735 | 229.5127455 |
| YPR132W | RPS23B | 0.55511059 | 0.95959383 | 0.11309754 | 105.909943 | 179.2881105 |
| YAL043C-A | YAL043C-A | 0.45023746 | 0.95675879 | 0.11336247 | 122.6064105 | 210.5576853 |
| YJR108W | ABM1 | 0.36549656 | 0.95675879 | 0.11341754 | 109.4887904 | 185.9456333 |
| YCR036W | RBK1 | 0.81675007 | 0.9824616 | 0.11356807 | 114.3358392 | 195.0107161 |
| YAR020C | PAU7 | 0.49108532 | 0.95675879 | 0.11382178 | 124.6291154 | 214.2727656 |
| YDR261C | EXG2 | 0.49953833 | 0.95675879 | 0.11408874 | 123.634793 | 212.3621983 |
| YER084W | YER084W | 0.44412976 | 0.95675879 | 0.11409215 | 129.9477025 | 224.2017315 |
| YPL216W | YPL216W | 0.75117148 | 0.97903138 | 0.1142938 | 139.0471474 | 241.2336095 |
| YLR191W | PEX13 | 0.48646237 | 0.95675879 | 0.1143723 | 106.3359561 | 179.8690123 |
| YPL249C | GYP5 | 0.62336564 | 0.96098984 | 0.11440546 | 127.9388214 | 220.3803886 |
| YLR174W | IDP2 | 0.4824941 | 0.95675879 | 0.11447753 | 126.200362 | 217.1075061 |
| YNL217W | YNL217W | 0.4199013 | 0.95675879 | 0.11504485 | 127.2307741 | 218.943021 |
| YMR106C | YKU80 | 0.52295712 | 0.95675879 | 0.11535052 | 126.3904905 | 217.3147375 |
| YGR199W | PMT6 | 0.40268585 | 0.95675879 | 0.1156587 | 119.206612 | 203.7883535 |
| YMR110C | HFD1 | 0.31350097 | 0.95675879 | 0.11583874 | 119.2428999 | 203.8256101 |
| YER032W | FIR1 | 0.26914238 | 0.95675879 | 0.11598823 | 123.2583846 | 211.3312379 |
| YLL010C | PSR1 | 0.57069721 | 0.95959383 | 0.11603006 | 116.0116807 | 197.732594 |
| YFL034C-A | RPL22B | 0.68876737 | 0.96992497 | 0.11605312 | 139.6685879 | 242.0981383 |
| YKR044W | UIP5 | 0.46183633 | 0.95675879 | 0.11606701 | 111.643265 | 189.5331303 |
| YKL105C | YKL105C | 0.11118095 | 0.95675879 | 0.11618068 | 107.903621 | 182.4998289 |
| YLR296W | YLR296W | 0.54864684 | 0.95900765 | 0.11625269 | 125.211253 | 214.9486733 |
| YKR103W | NFT1 | 0.56460633 | 0.95959383 | 0.11664468 | 111.3623378 | 188.9074053 |
| YDR223W | CRF1 | 0.68675474 | 0.96992497 | 0.11671081 | 129.7140092 | 223.3153944 |
| YHR048W | YHR048W | 0.30968771 | 0.95675879 | 0.1168607 | 123.1507291 | 210.9800516 |
| YHR087W | RTC3 | 0.46453382 | 0.95675879 | 0.11728472 | 118.9311555 | 202.9935223 |
| YJR149W | YJR149W | 0.41550968 | 0.95675879 | 0.11767497 | 116.6800309 | 198.7046779 |
| YHL033C | RPL8A | 0.58525071 | 0.95959383 | 0.11775516 | 88.93007865 | 146.6447967 |
| YER092W | IES5 | 0.39100222 | 0.95675879 | 0.1178242 | 10.74836139 | 0 |
| YER093C-A | YER093C-A | 0.40870945 | 0.95675879 | 0.11786394 | 108.439911 | 183.2176672 |
| YLR285W | NNT1 | 0.4880793 | 0.95675879 | 0.11795055 | 124.5696984 | 213.4549181 |
| YKR077W | YKR077W | 0.3089791 | 0.95675879 | 0.11800719 | 105.1692665 | 177.0589316 |
| YJR135C | MCM22 | 0.41939843 | 0.95675879 | 0.11822468 | 111.193494 | 188.3204035 |
| YBR090C-A | YBR090C-A | 0.40735182 | 0.95675879 | 0.11836331 | 121.3965844 | 207.4329938 |
| YDL162C | YDL162C | 0.40520701 | 0.95675879 | 0.11849039 | 134.6578058 | 232.28321 |
| YKL015W | PUT3 | 0.36143998 | 0.95675879 | 0.11849647 | 124.2264435 | 212.7177264 |
| YAR037W | YAR037W | 0.49055758 | 0.95675879 | 0.11891108 | 129.8985888 | 223.2851287 |
| YHL009C | YAP3 | 0.7317545 | 0.97731085 | 0.11909282 | 111.695167 | 189.1127783 |
| YCL051W | LRE1 | 0.53862144 | 0.95675879 | 0.11918873 | 140.4823463 | 243.0878905 |
| YMR215W | GAS3 | 0.3498385 | 0.95675879 | 0.11936002 | 118.2817254 | 201.4204191 |
| YMR191W | SPG5 | 0.32035519 | 0.95675879 | 0.11963725 | 133.4609684 | 229.842273 |
| YPR053C | YPR053C | 0.39889893 | 0.95675879 | 0.11970108 | 113.6571041 | 192.6884014 |
| YNL273W | TOF1 | 0.24104367 | 0.95675879 | 0.11983519 | 105.1649759 | 176.7381237 |
| YKL007W | CAP1 | 0.30812184 | 0.95675879 | 0.11993614 | 107.7066447 | 181.4878559 |
| YNL117W | MLS1 | 0.23144386 | 0.95675879 | 0.12011663 | 110.9494123 | 187.5389167 |
| YIL141W | YIL141W | 0.23996863 | 0.95675879 | 0.12017274 | 114.5682152 | 194.3165279 |
| YKL010C | UFD4 | 0.4406474 | 0.95675879 | 0.12022311 | 112.1724509 | 189.8145571 |
| YLR427W | MAG2 | 0.32404641 | 0.95675879 | 0.12024771 | 113.3846288 | 192.0838373 |
| YLL012W | YEH1 | 0.48339891 | 0.95675879 | 0.12034255 | 109.0585851 | 183.9539406 |
| YIL085C | KTR7 | 0.35071244 | 0.95675879 | 0.12038125 | 111.6933378 | 188.8889048 |
| YBR295W | PCA1 | 0.53412348 | 0.95675879 | 0.12062459 | 106.5188396 | 179.1422897 |
| YMR299C | DYN3 | 0.36798682 | 0.95675879 | 0.1206369 | 106.1984258 | 178.5392345 |
| YNL328C | MDJ2 | 0.39627882 | 0.95675879 | 0.12072323 | 114.0733337 | 193.2941726 |
| YDL234C | GYP7 | 0.5463512 | 0.95854866 | 0.12098919 | 139.207914 | 240.3895945 |
| YMR086W | YMR086W | 0.38053866 | 0.95675879 | 0.12109435 | 120.8169461 | 205.8785954 |
| YLR122C | YLR122C | 0.49560391 | 0.95675879 | 0.12110342 | 118.0835786 | 200.7505012 |
| YER150W | SPI1 | 0.68918447 | 0.96992497 | 0.12130946 | 119.6132344 | 203.5841814 |
| YLR070C | XYL2 | 0.3829946 | 0.95675879 | 0.12146725 | 116.4786344 | 197.6781144 |
| YKL197C | PEX1 | 0.40057504 | 0.95675879 | 0.12154221 | 116.8865936 | 198.4304334 |
| YOR345C | YOR345C | 0.39386734 | 0.95675879 | 0.12160506 | 116.6603254 | 197.9953045 |
| YCL034W | LSB5 | 0.36915135 | 0.95675879 | 0.12167263 | 134.2192436 | 230.9162072 |
| YKR090W | PXL1 | 0.35384634 | 0.95675879 | 0.12172131 | 121.4979242 | 207.0485277 |
| YGL248W | PDE1 | 0.50519344 | 0.95675879 | 0.1217607 | 130.7595717 | 224.4123853 |
| YAL042W | ERV46 | 0.41346242 | 0.95675879 | 0.12176204 | 128.8272523 | 220.7880123 |

| | | | | | | |
|---|---|---|---|---|---|---|
| YER079W | YER079W | 0.3937446 | 0.95675879 | 0.12182464 | 110.2132555 | 185.8659959 |
| YHR082C | KSP1 | 0.48369751 | 0.95675879 | 0.12193016 | 115.3040882 | 195.3960044 |
| YGL250W | YGL250W | 0.20437663 | 0.95675879 | 0.12205741 | 137.8921281 | 237.7390186 |
| YGL034C | YGL034C | 0.47311011 | 0.95675879 | 0.12206698 | 135.6834174 | 233.5948549 |
| YLR248W | RCK2 | 0.32305252 | 0.95675879 | 0.12235534 | 114.6743295 | 194.1421209 |
| YDL179W | PCL9 | 0.73349812 | 0.97731085 | 0.1225819 | 144.1849436 | 249.4517121 |
| YLR257W | YLR257W | 0.56799288 | 0.95959383 | 0.12260197 | 133.2119188 | 228.8679247 |
| YKR031C | SPO14 | 0.44772916 | 0.95675879 | 0.12282996 | 110.1101795 | 185.5006677 |
| YKR021W | ALY1 | 0.38405858 | 0.95675879 | 0.12301265 | 108.9334833 | 183.2624697 |
| YMR021C | MAC1 | 0.78462085 | 0.97903138 | 0.12334969 | 98.67838883 | 163.9709582 |
| YOR379C | YOR379C | 0.43109871 | 0.95675879 | 0.12374414 | 118.4762976 | 201.035251 |
| YHR097C | YHR097C | 0.38263163 | 0.95675879 | 0.12378305 | 118.3405204 | 200.7739389 |
| YBR119W | MUD1 | 0.47176444 | 0.95675879 | 0.12396989 | 134.7894243 | 231.5925586 |
| YDR076W | RAD55 | 0.78127232 | 0.97903138 | 0.12416157 | 105.4009543 | 176.4404944 |
| YLR341W | SPO77 | 0.17395758 | 0.95675879 | 0.12417971 | 109.1753897 | 183.5164983 |
| YFR044C | DUG1 | 0.42235624 | 0.95675879 | 0.12422939 | 130.7835556 | 224.0349902 |
| YEL007W | YEL007W | 0.76522961 | 0.97903138 | 0.12428386 | 51.89878215 | 76.07407607 |
| YKR070W | YKR070W | 0.38420279 | 0.95675879 | 0.12429476 | 119.1577297 | 202.219097 |
| YDL011C | YDL011C | 0.50694705 | 0.95675879 | 0.12435349 | 139.6199868 | 240.5868428 |
| YKL190W | CNB1 | 0.20570669 | 0.95675879 | 0.12461672 | 102.0633413 | 170.1027928 |
| YKL151C | YKL151C | 0.40426047 | 0.95675879 | 0.12466496 | 113.2692504 | 191.1116764 |
| YDL026W | YDL026W | 0.31837979 | 0.95675879 | 0.12487169 | 124.8128796 | 212.726851 |
| YMR273C | ZDS1 | 0.65877194 | 0.96992497 | 0.1251093 | 124.8007767 | 212.6634991 |
| YIL072W | HOP1 | 0.51808688 | 0.95675879 | 0.12520865 | 133.1781491 | 228.3586019 |
| YKL117W | SBA1 | 0.33943918 | 0.95675879 | 0.12522046 | 111.9942789 | 188.6253735 |
| YKL120W | OAC1 | 0.11094288 | 0.95675879 | 0.12522075 | 113.2713977 | 191.0206117 |
| YDR384C | ATO3 | 0.59356755 | 0.95959383 | 0.12552946 | 119.3982645 | 202.4589801 |
| YKR039W | GAP1 | 0.40182477 | 0.95675879 | 0.12581641 | 111.3475154 | 187.310378 |
| YNL338W | YNL338W | 0.31647795 | 0.95675879 | 0.12585988 | 107.916895 | 180.8686739 |
| YNR004W | YNR004W | 0.44032779 | 0.95675879 | 0.12586356 | 99.69060839 | 165.4393087 |
| YML058C-A | YML058C-A | 0.25122747 | 0.95675879 | 0.12617001 | 122.0525844 | 207.3276718 |
| YNL233W | BNI4 | 0.42905051 | 0.95675879 | 0.1263958 | 106.795231 | 178.6732543 |
| YNL200C | YNL200C | 0.43641168 | 0.95675879 | 0.12640068 | 119.2221013 | 201.9795195 |
| YEL013W | VAC8 | 0.73496292 | 0.97731085 | 0.12665162 | 82.85945851 | 133.7369734 |
| YIR044C | YIR044C | 0.59560055 | 0.95959383 | 0.12669901 | 126.4624642 | 215.5080708 |
| YKR045C | YKR045C | 0.47292672 | 0.95675879 | 0.12700711 | 113.3722501 | 190.9041285 |
| YLR337C | VRP1 | 0.68319449 | 0.96992497 | 0.12711207 | 74.73067744 | 118.4123333 |
| YNL129W | NRK1 | 0.27571714 | 0.95675879 | 0.12721355 | 113.5298939 | 191.1644753 |
| YGL226C-A | OST5 | 0.32280528 | 0.95675879 | 0.12721471 | 132.6398258 | 227.0057306 |
| YLL020C | YLL020C | 0.40082587 | 0.95675879 | 0.12723992 | 110.6465569 | 185.752148 |
| YKR084C | HBS1 | 0.46539593 | 0.95675879 | 0.12728128 | 102.9885594 | 171.3821871 |
| YDR120C | TRM1 | 0.39217715 | 0.95675879 | 0.12731367 | 115.7774465 | 195.3627213 |
| YDL039C | PRM7 | 0.45871491 | 0.95675879 | 0.12732812 | 125.0834069 | 212.8139567 |
| YNL071W | LAT1 | 0.28290589 | 0.95675879 | 0.12757129 | 106.289188 | 177.5230309 |
| YOR216C | RUD3 | 0.50808858 | 0.95675879 | 0.12759441 | 122.6074522 | 208.1246419 |
| YGR275W | RTT102 | 0.50779432 | 0.95675879 | 0.12767802 | 133.3030087 | 228.1702877 |
| YNL321W | YNL321W | 0.45569915 | 0.95675879 | 0.12768711 | 118.695796 | 200.7723116 |
| YLR282C | YLR282C | 0.47455826 | 0.95675879 | 0.1277262 | 112.3268787 | 188.8204617 |
| YGL234W | ADE5 | 0.43514833 | 0.95675879 | 0.12778851 | 117.2774722 | 198.0948398 |
| YKL156W | RPS27A | 0.35875744 | 0.95675879 | 0.12798183 | 99.24168936 | 164.2349208 |
| YBL043W | ECM13 | 0.39466864 | 0.95675879 | 0.12806698 | 141.3394193 | 243.1763532 |
| YIL155C | GUT2 | 0.3992356 | 0.95675879 | 0.12836078 | 114.8998523 | 193.5376053 |
| YNR012W | URK1 | 0.27085306 | 0.95675879 | 0.1283656 | 115.35074 | 194.3824383 |
| YKR088C | TVP38 | 0.39944614 | 0.95675879 | 0.12892192 | 123.1202438 | 208.8592756 |
| YDR329C | PEX3 | 0.40179353 | 0.95675879 | 0.12897782 | 121.9163257 | 206.5917135 |
| YCL011C | GBP2 | 0.30569973 | 0.95675879 | 0.1290035 | 132.3098364 | 226.0807711 |
| YDR346C | SVF1 | 0.43770249 | 0.95675879 | 0.12901003 | 120.7945597 | 204.4822844 |
| YMR255W | GFD1 | 0.38390748 | 0.95675879 | 0.12902344 | 107.2334058 | 179.0454954 |
| YMR044W | IOC4 | 0.35205057 | 0.95675879 | 0.12936743 | 107.571768 | 179.6212529 |
| YBR300C | YBR300C | 0.29620392 | 0.95675879 | 0.12955536 | 133.7504007 | 228.6881885 |
| YPR189W | SKI3 | 0.49100743 | 0.95675879 | 0.12958763 | 112.3945569 | 188.6289156 |
| YLL052C | AQY2 | 0.41834913 | 0.95675879 | 0.12960613 | 120.4818352 | 203.7937684 |
| YFL007W | BLM10 | 0.39693514 | 0.95675879 | 0.12973432 | 119.6776612 | 202.2635757 |
| YNL057W | YNL057W | 0.20081607 | 0.95675879 | 0.12983471 | 107.2005533 | 178.8450765 |

| | | | | | | |
|---|---|---|---|---|---|---|
| YLR269C | YLR269C | 0.46809572 | 0.95675879 | 0.12985179 | 117.0653341 | 197.343952 |
| YMR074C | YMR074C | 0.38420958 | 0.95675879 | 0.12989349 | 118.9210479 | 200.8172837 |
| YLR350W | ORM2 | 0.41062098 | 0.95675879 | 0.12991468 | 125.8718136 | 213.8501009 |
| YAL049C | YAL049C | 0.37708876 | 0.95675879 | 0.12999696 | 123.3735129 | 209.1503588 |
| YLR113W | HOG1 | 0.38979064 | 0.95675879 | 0.13027518 | 107.4551211 | 179.2471677 |
| YLR036C | YLR036C | 0.28624545 | 0.95675879 | 0.13035038 | 108.2156058 | 180.6606215 |
| YMR259C | YMR259C | 0.24075564 | 0.95675879 | 0.13037076 | 111.4951158 | 186.8079883 |
| YNL154C | YCK2 | 0.713269 | 0.97292791 | 0.13046883 | 98.10791152 | 161.6829647 |
| YDR395W | SXM1 | 0.58393142 | 0.95959383 | 0.13049246 | 129.8216584 | 221.1593412 |
| YKL008C | LAC1 | 0.38498377 | 0.95675879 | 0.13059725 | 109.3540058 | 182.7534991 |
| YKR056W | TRM2 | 0.32983732 | 0.95675879 | 0.13069853 | 113.2310149 | 190.0076585 |
| YGR019W | UGA1 | 0.32935591 | 0.95675879 | 0.13074197 | 136.6501037 | 233.9236763 |
| YHR195W | NVJ1 | 0.47224933 | 0.95675879 | 0.13095565 | 125.4083455 | 212.8027442 |
| YDL146W | LDB17 | 0.4513914 | 0.95675879 | 0.13110226 | 134.6234521 | 230.0609662 |
| YIL158W | YIL158W | 0.38936402 | 0.95675879 | 0.1311423 | 104.6263747 | 173.7933795 |
| YAL054C | ACS1 | 0.54113193 | 0.95675879 | 0.13115339 | 150.666005 | 260.1406772 |
| YGL251C | HFM1 | 0.34873501 | 0.95675879 | 0.13130642 | 133.053425 | 227.0813864 |
| YGR276C | RNH70 | 0.37029359 | 0.95675879 | 0.1315592 | 109.8203797 | 183.4636177 |
| YHR193C | EGD2 | 0.37805211 | 0.95675879 | 0.13170404 | 144.1583805 | 247.8411498 |
| YLR087C | CSF1 | 0.41045123 | 0.95675879 | 0.13174145 | 111.3553139 | 186.3112681 |
| YMR318C | ADH6 | 0.33953559 | 0.95675879 | 0.13195008 | 120.2565522 | 202.970206 |
| YMR304C-A | YMR304C-A | 0.20067347 | 0.95675879 | 0.13211533 | 105.4552856 | 175.1815557 |
| YDL048C | STP4 | 0.37351658 | 0.95675879 | 0.1323315 | 134.965463 | 230.4921063 |
| YGR243W | FMP43 | 0.37871251 | 0.95675879 | 0.13239662 | 113.2969492 | 189.840788 |
| YDR048C | YDR048C | 0.52001526 | 0.95675879 | 0.13241613 | 117.3336726 | 197.4084889 |
| YDL038C | YDL038C | 0.64196168 | 0.96586667 | 0.13246688 | 147.8248929 | 254.5873265 |
| YOL047C | YOL047C | 0.32246291 | 0.95675879 | 0.13251224 | 128.3358021 | 218.0269855 |
| YNL027W | CRZ1 | 0.39131055 | 0.95675879 | 0.13294056 | 114.9376039 | 192.8248378 |
| YFL004W | VTC2 | 0.50366518 | 0.95675879 | 0.13303393 | 118.5526845 | 199.5890923 |
| YPR061C | JID1 | 0.33702075 | 0.95675879 | 0.13309402 | 111.8352138 | 186.9799224 |
| YLR364W | YLR364W | 0.3418903 | 0.95675879 | 0.13314532 | 96.32777089 | 157.8863067 |
| YHR004C | NEM1 | 0.3770061 | 0.95675879 | 0.13322849 | 106.5454027 | 177.0356607 |
| YJL142C | YJL142C | 0.33860545 | 0.95675879 | 0.13333204 | 116.3920516 | 195.4857329 |
| YLL051C | FRE6 | 0.43714288 | 0.95675879 | 0.13354715 | 109.3629217 | 182.2655118 |
| YJL190C | RPS22A | 0.371655 | 0.95675879 | 0.13362057 | 121.5339276 | 205.0801641 |
| YHR114W | BZZ1 | 0.56412976 | 0.95959383 | 0.13394745 | 125.2713968 | 212.0340136 |
| YML022W | APT1 | 0.57913834 | 0.95959383 | 0.1345688 | 78.28421672 | 123.8013404 |
| YNL169C | PSD1 | 0.64087374 | 0.96479866 | 0.13465954 | 83.58148251 | 133.721052 |
| YDL134C | PPH21 | 0.44756883 | 0.95675879 | 0.13476127 | 123.1711685 | 207.9557099 |
| YMR188C | MRPS17 | 0.28221783 | 0.95675879 | 0.13486028 | 111.244861 | 185.570496 |
| YOL049W | GSH2 | 0.72273438 | 0.97591869 | 0.13493947 | 70.94567093 | 109.9741812 |
| YMR109W | MYO5 | 0.31121735 | 0.95675879 | 0.13494482 | 119.7651675 | 201.536213 |
| YKR046C | PET10 | 0.42147802 | 0.95675879 | 0.13515608 | 111.8413336 | 186.6385953 |
| YCR100C | YCR100C | 0.12206762 | 0.95675879 | 0.13518658 | 122.0758337 | 205.8285968 |
| YKL183W | LOT5 | 0.40512313 | 0.95675879 | 0.13538987 | 106.0173751 | 175.6755245 |
| YNL339C | YRF1-6 | 0.38341042 | 0.95675879 | 0.13542925 | 112.3965708 | 187.633228 |
| YCL012W | YCL012W | 0.31824358 | 0.95675879 | 0.1356793 | 129.8234252 | 220.2752185 |
| YDR098C | GRX3 | 0.5481184 | 0.95900765 | 0.13598771 | 125.2713374 | 211.684825 |
| YNL130C | CPT1 | 0.26819193 | 0.95675879 | 0.13607013 | 112.2171408 | 187.187048 |
| YNL043C | YNL043C | 0.61700963 | 0.96098984 | 0.13629729 | 123.9786227 | 209.2073194 |
| YHR158C | KEL1 | 0.29426414 | 0.95675879 | 0.1364205 | 109.5793042 | 182.1797334 |
| YPL098C | MGR2 | 0.68152092 | 0.96992497 | 0.13648495 | 112.7932633 | 188.1966174 |
| YLL049W | LDB18 | 0.77076882 | 0.97903138 | 0.13652014 | 108.6701679 | 180.4575629 |
| YMR266W | RSN1 | 0.37642691 | 0.95675879 | 0.13663457 | 116.7086962 | 195.5145713 |
| YDR286C | YDR286C | 0.49567168 | 0.95675879 | 0.13673281 | 128.4564177 | 217.5310903 |
| YNL303W | YNL303W | 0.35441342 | 0.95675879 | 0.13681178 | 116.3214282 | 194.7579138 |
| YAL053W | FLC2 | 0.4453392 | 0.95675879 | 0.13681484 | 128.3215332 | 217.2640755 |
| YBR085W | AAC3 | 0.39521872 | 0.95675879 | 0.13751632 | 113.8371269 | 189.9779637 |
| YNR032W | PPG1 | 0.51755229 | 0.95675879 | 0.13754938 | 132.7329344 | 225.4121617 |
| YNL156C | NSG2 | 0.35772052 | 0.95675879 | 0.13774706 | 114.4605347 | 191.107712 |
| YBR178W | YBR178W | 0.42115714 | 0.95675879 | 0.13785814 | 133.3800754 | 226.5730745 |
| YMR026C | PEX12 | 0.17004738 | 0.95675879 | 0.13797984 | 107.4410605 | 177.9025772 |
| YKL043W | PHD1 | 0.21601562 | 0.95675879 | 0.13801266 | 113.3841626 | 189.0434908 |
| YGR133W | PEX4 | 0.48447192 | 0.95675879 | 0.13803812 | 127.6192089 | 215.7375407 |

| | | | | | | |
|---|---|---|---|---|---|---|
| YLL021W | SPA2 | 0.36117475 | 0.95675879 | 0.13830273 | 126.4419074 | 213.484191 |
| YMR245W | YMR245W | 0.0775016 | 0.95675879 | 0.13836281 | 93.83731688 | 152.3226802 |
| YNR050C | LYS9 | 0.72927583 | 0.97683349 | 0.13850755 | 92.01718127 | 148.884177 |
| YJR111C | YJR111C | 0.36636216 | 0.95675879 | 0.13855683 | 111.9007252 | 186.1681396 |
| YNL024C | YNL024C | 0.38387877 | 0.95675879 | 0.13866075 | 113.9082042 | 189.915467 |
| YLR187W | SKG3 | 0.40019032 | 0.95675879 | 0.13878222 | 115.4415594 | 192.770555 |
| YLR308W | CDA2 | 0.30351841 | 0.95675879 | 0.13882764 | 116.4493256 | 194.6528893 |
| YKR059W | TIF1 | 0.43695708 | 0.95675879 | 0.13892043 | 113.0393187 | 188.2414088 |
| YMR316C-A | YMR316C-A | 0.44510872 | 0.95675879 | 0.13903876 | 130.3722656 | 220.729807 |
| YLR307W | CDA1 | 0.25977562 | 0.95675879 | 0.13908353 | 120.9628691 | 203.0744424 |
| YGL094C | PAN2 | 0.53346115 | 0.95675879 | 0.13928034 | 127.5390787 | 215.3747186 |
| YGR209C | TRX2 | 0.31724083 | 0.95675879 | 0.13935036 | 118.2709329 | 197.979955 |
| YDR252W | BTT1 | 0.25256999 | 0.95675879 | 0.13947176 | 124.3016884 | 209.2701098 |
| YGR256W | GND2 | 0.42891006 | 0.95675879 | 0.1395615 | 116.3763365 | 194.3904378 |
| YDR033W | MRH1 | 0.51916978 | 0.95675879 | 0.1396471 | 116.1957761 | 194.037143 |
| YPR009W | SUT2 | 0.13299965 | 0.95675879 | 0.13969096 | 109.6234125 | 181.7029044 |
| YHR202W | YHR202W | 0.40895462 | 0.95675879 | 0.13994613 | 121.5781952 | 204.0809259 |
| YDR493W | FMP36 | 0.44583664 | 0.95675879 | 0.14025437 | 125.8133756 | 211.9714411 |
| YGL242C | YGL242C | 0.18203402 | 0.95675879 | 0.14085592 | 140.9146138 | 240.1915053 |
| YDR068W | DOS2 | 0.44655368 | 0.95675879 | 0.14087906 | 126.4706928 | 213.0973855 |
| YIR029W | DAL2 | 0.51514493 | 0.95675879 | 0.14088706 | 128.781507 | 217.4300428 |
| YDL243C | AAD4 | 0.44081341 | 0.95675879 | 0.14092168 | 136.929566 | 232.7061343 |
| YMR254C | YMR254C | 0.42145271 | 0.95675879 | 0.14106258 | 113.5253818 | 188.7865305 |
| YHR016C | YSC84 | 0.34245596 | 0.95675879 | 0.14110538 | 119.4064972 | 199.8094788 |
| YDL129W | YDL129W | 0.27760533 | 0.95675879 | 0.14113004 | 134.4839343 | 228.0836037 |
| YMR137C | PSO2 | 0.25925348 | 0.95675879 | 0.14150749 | 122.6085004 | 205.7461671 |
| YOL150C | YOL150C | 0.22630724 | 0.95675879 | 0.14156882 | 130.8488669 | 221.1908161 |
| YLR019W | PSR2 | 0.2219159 | 0.95675879 | 0.14201448 | 111.1846253 | 184.2334832 |
| YDR380W | ARO10 | 0.42457966 | 0.95675879 | 0.14206215 | 126.6913531 | 213.3088232 |
| YKL191W | DPH2 | 0.36848883 | 0.95675879 | 0.14209558 | 108.3163891 | 178.8401136 |
| YGR131W | YGR131W | 0.17734746 | 0.95675879 | 0.14236783 | 124.3727877 | 208.907961 |
| YNL153C | GIM3 | 0.201689 | 0.95675879 | 0.14256427 | 100.2155721 | 163.5665119 |
| YGL157W | YGL157W | 0.39716557 | 0.95675879 | 0.14270214 | 139.7910761 | 237.7683874 |
| YOR196C | LIP5 | 0.66442347 | 0.96992497 | 0.14326816 | 63.05808195 | 93.75570058 |
| YDR139C | RUB1 | 0.47422137 | 0.95675879 | 0.14327786 | 139.2286915 | 236.6151107 |
| YLR426W | YLR426W | 0.04571849 | 0.92220427 | 0.14365574 | 108.8679934 | 179.6077359 |
| YGL032C | AGA2 | 0.39395203 | 0.95675879 | 0.14390183 | 135.5419545 | 229.5937276 |
| YKL123W | YKL123W | 0.40403559 | 0.95675879 | 0.1439547 | 119.1577784 | 198.8554953 |
| YLR178C | TFS1 | 0.49370151 | 0.95675879 | 0.14397124 | 116.1570524 | 193.2246818 |
| YHL044W | YHL044W | 0.72464842 | 0.97666049 | 0.14406495 | 124.0760689 | 208.0610855 |
| YNL022C | YNL022C | 0.48870771 | 0.95675879 | 0.14415201 | 130.5200716 | 220.1321787 |
| YPR040W | TIP41 | 0.43387928 | 0.95675879 | 0.14417686 | 121.7973199 | 203.7680515 |
| YKL025C | PAN3 | 0.27291389 | 0.95675879 | 0.14495607 | 111.3889328 | 184.1133825 |
| YKL174C | TPO5 | 0.33612721 | 0.95675879 | 0.14501401 | 112.7641565 | 186.6827559 |
| YKL017C | HCS1 | 0.34002051 | 0.95675879 | 0.14520815 | 111.3410272 | 183.980403 |
| YBR158W | AMN1 | 0.48053785 | 0.95675879 | 0.14523001 | 135.4150582 | 229.1284863 |
| YBR241C | YBR241C | 0.45040897 | 0.95675879 | 0.14525052 | 146.1585062 | 249.2747494 |
| YOL045W | PSK2 | 0.3785686 | 0.95675879 | 0.14532513 | 121.2393433 | 202.5250829 |
| YDR119W | YDR119W | 0.35459573 | 0.95675879 | 0.14543622 | 123.7616109 | 207.2366916 |
| YAL064C-A | YAL064C-A | 0.45035318 | 0.95675879 | 0.14566747 | 132.4603588 | 223.5119794 |
| YDL053C | PBP4 | 0.25959288 | 0.95675879 | 0.14569841 | 135.3300093 | 228.8888319 |
| YOR359W | VTS1 | 0.37559878 | 0.95675879 | 0.14580527 | 114.6857635 | 190.1514293 |
| YLR199C | YLR199C | 0.37922886 | 0.95675879 | 0.14581818 | 124.2605605 | 208.1071397 |
| YKR015C | YKR015C | 0.24474301 | 0.95675879 | 0.14591907 | 104.34044 | 170.7288852 |
| YLL042C | ATG10 | 0.75981491 | 0.97903138 | 0.14600284 | 110.3585525 | 182.0017664 |
| YKL164C | PIR1 | 0.34801191 | 0.95675879 | 0.14602455 | 110.786981 | 182.8015869 |
| YKR054C | DYN1 | 0.35425353 | 0.95675879 | 0.14619651 | 107.1435879 | 175.9388331 |
| YKL096W-A | CWP2 | 0.370581 | 0.95675879 | 0.14631826 | 116.740451 | 193.9173091 |
| YLR289W | GUF1 | 0.48709944 | 0.95675879 | 0.14632997 | 119.7609921 | 199.5804524 |
| YGR205W | YGR205W | 0.31186901 | 0.95675879 | 0.14642678 | 122.6653926 | 205.0112112 |
| YFR032C-A | RPL29 | 0.23805505 | 0.95675879 | 0.14677508 | 116.7697527 | 193.8941066 |
| YLL057C | JLP1 | 0.24625019 | 0.95675879 | 0.14697037 | 108.8394567 | 178.987101 |
| YKL133C | YKL133C | 0.15076303 | 0.95675879 | 0.14702264 | 113.6131599 | 187.9314322 |
| YKL051W | SFK1 | 0.3065763 | 0.95675879 | 0.14718084 | 107.8656227 | 177.1246268 |

| | | | | | | |
|---|---|---|---|---|---|---|
| YJL057C | IKS1 | 0.44853705 | 0.95675879 | 0.14726012 | 118.6611124 | 197.3584416 |
| YMR153W | NUP53 | 0.01337303 | 0.59816436 | 0.14732866 | 109.7640095 | 180.6598372 |
| YGL139W | FLC3 | 0.29377267 | 0.95675879 | 0.14738656 | 130.1234935 | 218.8349694 |
| YBL049W | MOH1 | 0.30775893 | 0.95675879 | 0.14738991 | 122.8374993 | 205.1692189 |
| YFR007W | YFR007W | 0.34254819 | 0.95675879 | 0.14740784 | 115.998708 | 192.3397192 |
| YKR047W | YKR047W | 0.2736488 | 0.95675879 | 0.14751874 | 104.4044188 | 170.5751863 |
| YBR015C | MNN2 | 0.35436885 | 0.95675879 | 0.14754128 | 128.4280342 | 215.6285953 |
| YKL065C | YET1 | 0.29932211 | 0.95675879 | 0.14766402 | 108.6154785 | 178.4483419 |
| YPL069C | BTS1 | 0.74527208 | 0.97903138 | 0.14785961 | 49.42227333 | 67.3956198 |
| YKR033C | YKR033C | 0.38037278 | 0.95675879 | 0.14798379 | 116.0610077 | 192.358024 |
| YBR180W | DTR1 | 0.50406977 | 0.95675879 | 0.14818894 | 137.4013301 | 232.347564 |
| YBR034C | HMT1 | 0.16857823 | 0.95675879 | 0.14828126 | 109.0151881 | 179.092408 |
| YDL024C | DIA3 | 0.25044493 | 0.95675879 | 0.14836511 | 136.973638 | 231.5152683 |
| YJL121C | RPE1 | 0.19239105 | 0.95675879 | 0.14842834 | 107.4684863 | 176.1663407 |
| YGL176C | YGL176C | 0.23150331 | 0.95675879 | 0.14853697 | 129.3958804 | 217.2734726 |
| YKL061W | YKL061W | 0.34319695 | 0.95675879 | 0.14854037 | 107.0144602 | 175.2956289 |
| YGL108C | YGL108C | 0.38847665 | 0.95675879 | 0.14861645 | 144.3014891 | 245.2159478 |
| YDL035C | GPR1 | 0.33405328 | 0.95675879 | 0.14864748 | 137.081452 | 231.6691669 |
| YMR178W | YMR178W | 0.47049009 | 0.95675879 | 0.14867596 | 130.8693884 | 220.0133175 |
| YBR170C | NPL4 | 0.30399822 | 0.95675879 | 0.14907454 | 120.8650092 | 201.1815028 |
| YFR023W | PES4 | 0.38606865 | 0.95675879 | 0.14917996 | 139.0567429 | 235.282801 |
| YAR002C-A | ERP1 | 0.35487709 | 0.95675879 | 0.14924357 | 125.8978316 | 210.5918456 |
| YLR164W | YLR164W | 0.33211649 | 0.95675879 | 0.14929969 | 112.375406 | 185.2203853 |
| YLR268W | SEC22 | 0.40010344 | 0.95675879 | 0.14933429 | 105.2497437 | 171.849997 |
| YHR096C | HXT5 | 0.29262428 | 0.95675879 | 0.1493389 | 130.6960667 | 219.5748195 |
| YHL023C | RMD11 | 0.2891684 | 0.95675879 | 0.14943988 | 94.95593286 | 152.5254722 |
| YDR410C | STE14 | 0.46865374 | 0.95675879 | 0.14954947 | 130.9042827 | 219.9293107 |
| YKR066C | CCP1 | 0.37498302 | 0.95675879 | 0.14981602 | 112.6146126 | 185.580686 |
| YGR096W | TPC1 | 0.01618552 | 0.6196335 | 0.14991486 | 111.4906257 | 183.4556933 |
| YNL092W | YNL092W | 0.39105672 | 0.95675879 | 0.14998625 | 124.3342572 | 207.5322299 |
| YGL062W | PYC1 | 0.11267489 | 0.95675879 | 0.15013583 | 125.4721967 | 209.6408909 |
| YGL005C | COG7 | 0.22310979 | 0.95675879 | 0.15016655 | 126.3394691 | 211.2622381 |
| YGR247W | CPD1 | 0.31921234 | 0.95675879 | 0.15017834 | 123.0621502 | 205.1134768 |
| YLL062C | MHT1 | 0.23458087 | 0.95675879 | 0.15041744 | 119.4068696 | 198.2169404 |
| YAL062W | GDH3 | 0.38356059 | 0.95675879 | 0.15044134 | 129.4007344 | 216.9567512 |
| YDL071C | YDL071C | 0.33276061 | 0.95675879 | 0.1505703 | 123.6385703 | 206.1275135 |
| YMR165C | PAH1 | 0.69962716 | 0.97267176 | 0.15060774 | 39.61560388 | 48.53262529 |
| YDL034W | YDL034W | 0.35003501 | 0.95675879 | 0.15064684 | 146.8692704 | 249.6845387 |
| YBR292C | YBR292C | 0.39103597 | 0.95675879 | 0.15120663 | 140.4761049 | 237.5981215 |
| YLR438W | CAR2 | 0.08406791 | 0.95675879 | 0.15120709 | 118.1390384 | 195.7039685 |
| YGR001C | YGR001C | 0.37566278 | 0.95675879 | 0.15128979 | 136.5831248 | 230.2824514 |
| YER106W | MAM1 | 0.60251804 | 0.95959383 | 0.15145385 | 129.031836 | 216.0916329 |
| YDR083W | RRP8 | 0.15843304 | 0.95675879 | 0.15149164 | 89.03525511 | 141.0699569 |
| YML124C | TUB3 | 0.61538507 | 0.96098984 | 0.15164103 | 124.6703189 | 207.879406 |
| YKL064W | MNR2 | 0.29720573 | 0.95675879 | 0.15167732 | 109.5018476 | 179.4241137 |
| YBR197C | YBR197C | 0.36207351 | 0.95675879 | 0.15178654 | 132.6594976 | 222.8385388 |
| YHL039W | YHL039W | 0.28467622 | 0.95675879 | 0.15199164 | 127.362932 | 212.8695231 |
| YMR291W | YMR291W | 0.39819572 | 0.95675879 | 0.15208393 | 120.9848973 | 200.891471 |
| YGL089C | MF(ALPHA)2 | 0.29172684 | 0.95675879 | 0.15208921 | 139.7385648 | 236.0638311 |
| YKR016W | FMP13 | 0.47122994 | 0.95675879 | 0.15210085 | 102.4595085 | 166.1434571 |
| YKL158W | YKL158W | 0.33075638 | 0.95675879 | 0.15221238 | 120.1131302 | 199.2344591 |
| YPL108W | YPL108W | 0.49159144 | 0.95675879 | 0.15253844 | 126.0072222 | 210.2332812 |
| YMR173W-A | YMR173W-A | 0.23132851 | 0.95675879 | 0.15255893 | 113.6430411 | 187.0402522 |
| YFR008W | FAR7 | 0.11451587 | 0.95675879 | 0.15261478 | 132.2926239 | 222.0087455 |
| YDR159W | SAC3 | 0.56008097 | 0.95959383 | 0.1527562 | 21.09702047 | 13.43268318 |
| YMR276W | DSK2 | 0.37186855 | 0.95675879 | 0.15305488 | 122.1141916 | 202.8433848 |
| YCR005C | CIT2 | 0.20176249 | 0.95675879 | 0.15320472 | 118.6000122 | 196.226761 |
| YDL168W | SFA1 | 0.38103714 | 0.95675879 | 0.15355843 | 128.7883685 | 215.2749191 |
| YPL269W | KAR9 | 0.54110805 | 0.95675879 | 0.15363806 | 116.7480078 | 192.6791105 |
| YMR019W | STB4 | 0.05892308 | 0.95675879 | 0.15365793 | 129.6842127 | 216.9380873 |
| YIL161W | YIL161W | 0.58630531 | 0.95959383 | 0.15395541 | 142.7110839 | 241.3196157 |
| YNR008W | LRO1 | 0.17135849 | 0.95675879 | 0.15416172 | 129.419303 | 216.3550427 |
| YDR035W | ARO3 | 0.43510045 | 0.95675879 | 0.15417337 | 125.2713017 | 208.573306 |
| YGR138C | TPO2 | 0.32645956 | 0.95675879 | 0.15420462 | 135.3369557 | 227.4465016 |

| | | | | | | |
|---|---|---|---|---|---|---|
| YMR177W | MMT1 | 0.30229057 | 0.95675879 | 0.15426909 | 114.023498 | 187.4612154 |
| YHR160C | PEX18 | 0.42394528 | 0.95675879 | 0.15450739 | 126.2174747 | 210.2907418 |
| YLR003C | YLR003C | 0.32878821 | 0.95675879 | 0.1546871 | 111.7993486 | 183.2182148 |
| YBR101C | FES1 | 0.32043556 | 0.95675879 | 0.15484089 | 124.271938 | 206.5847504 |
| YGR039W | YGR039W | 0.30925589 | 0.95675879 | 0.15500088 | 125.796111 | 209.4160255 |
| YMR052C-A | YMR052C-A | 0.26680524 | 0.95675879 | 0.15507202 | 140.7918831 | 237.529032 |
| YMR214W | SCJ1 | 0.17837025 | 0.95675879 | 0.15507671 | 121.0935297 | 200.5831679 |
| YBR259W | YBR259W | 0.31985056 | 0.95675879 | 0.1551979 | 131.7771428 | 220.5999832 |
| YMR306C-A | YMR306C-A | 0.26926945 | 0.95675879 | 0.15543871 | 112.0199344 | 183.5033372 |
| YKR094C | RPL40B | 0.36108693 | 0.95675879 | 0.15561901 | 105.7609477 | 171.7335048 |
| YIL162W | SUC2 | 0.42461763 | 0.95675879 | 0.15570701 | 122.3027287 | 202.7432297 |
| YDR094W | YDR094W | 0.43994794 | 0.95675879 | 0.15572862 | 131.4904386 | 219.9714554 |
| YCR060W | TAH1 | 0.23096479 | 0.95675879 | 0.15593873 | 116.2583919 | 191.3671844 |
| YMR075C-A | YMR075C-A | 0.22903318 | 0.95675879 | 0.15629891 | 106.8593345 | 173.6772473 |
| YKR009C | FOX2 | 0.41283554 | 0.95675879 | 0.15642527 | 114.263761 | 187.5429307 |
| YLR123C | YLR123C | 0.21268501 | 0.95675879 | 0.1565645 | 128.1011478 | 213.471689 |
| YIL119C | RPI1 | 0.46293662 | 0.95675879 | 0.15660078 | 121.3364772 | 200.7780684 |
| YBR226C | YBR226C | 0.24834864 | 0.95675879 | 0.15662325 | 128.9366269 | 215.0286124 |
| YAR050W | FLO1 | 0.4552092 | 0.95675879 | 0.15678478 | 142.1779602 | 239.8356334 |
| YJR142W | YJR142W | 0.42514056 | 0.95675879 | 0.15702895 | 128.3810304 | 213.9171564 |
| YNL052W | COX5A | 0.26007522 | 0.95675879 | 0.15706417 | 109.6005724 | 178.6876183 |
| YGL085W | YGL085W | 0.3599025 | 0.95675879 | 0.15727665 | 113.5818924 | 186.1183918 |
| YBL021C | HAP3 | 0.21463037 | 0.95675879 | 0.15775949 | 102.5066655 | 165.2637436 |
| YMR122C | YMR122C | 0.04902175 | 0.92220427 | 0.15786667 | 115.0265541 | 188.726964 |
| YNL082W | PMS1 | 0.71285469 | 0.97267176 | 0.15789289 | 107.7711415 | 175.1146586 |
| YGR201C | YGR201C | 0.30281388 | 0.95675879 | 0.15789612 | 131.8678235 | 220.3084097 |
| YLR263W | RED1 | 0.1890171 | 0.95675879 | 0.15808086 | 108.2858093 | 176.0477779 |
| YLL025W | PAU17 | 0.31163234 | 0.95675879 | 0.15836574 | 132.6832323 | 221.7573938 |
| YMR078C | CTF18 | 0.64789359 | 0.96786115 | 0.15925786 | 91.22285631 | 143.84414 |
| YMR053C | STB2 | 0.29866039 | 0.95675879 | 0.15929775 | 120.2714997 | 198.3192246 |
| YKL142W | MRP8 | 0.35073947 | 0.95675879 | 0.15939842 | 111.7477625 | 182.3153851 |
| YLR283W | YLR283W | 0.33793475 | 0.95675879 | 0.15941104 | 122.9346556 | 203.2946987 |
| YJL191W | RPS14B | 0.31267113 | 0.95675879 | 0.15946586 | 114.7961397 | 188.0212025 |
| YLR326W | YLR326W | 0.30414126 | 0.95675879 | 0.16003082 | 124.8054887 | 206.6974813 |
| YGR184C | UBR1 | 0.32488023 | 0.95675879 | 0.16005024 | 121.7252597 | 200.9170638 |
| YLR219W | MSC3 | 0.11797521 | 0.95675879 | 0.16039236 | 107.2720497 | 173.7509481 |
| YDR411C | DFM1 | 0.45411961 | 0.95675879 | 0.16041716 | 130.5681623 | 217.4395093 |
| YNL142W | MEP2 | 0.44618006 | 0.95675879 | 0.16061462 | 104.775105 | 169.0297997 |
| YKR092C | SRP40 | 0.57313222 | 0.95959383 | 0.16070593 | 76.10338454 | 115.2392014 |
| YIR036C | IRC24 | 0.42737321 | 0.95675879 | 0.16075339 | 128.0938148 | 212.7412421 |
| YKL102C | YKL102C | 0.30100191 | 0.95675879 | 0.160828 | 114.0418407 | 186.3734306 |
| YMR265C | YMR265C | 0.32780243 | 0.95675879 | 0.16104989 | 118.2443221 | 194.2173907 |
| YGR224W | AZR1 | 0.33724856 | 0.95675879 | 0.16108432 | 128.7923182 | 213.9946948 |
| YFL018C | LPD1 | 0.06895788 | 0.95675879 | 0.16109047 | 110.1733421 | 179.0729958 |
| YOR029W | YOR029W | 0.16273077 | 0.95675879 | 0.16131209 | 123.3962849 | 203.8352444 |
| YBL083C | YBL083C | 0.27779115 | 0.95675879 | 0.16134033 | 139.141745 | 233.3616615 |
| YGR189C | CRH1 | 0.40674578 | 0.95675879 | 0.16232977 | 127.9827045 | 212.2631421 |
| YCR001W | YCR001W | 0.2448276 | 0.95675879 | 0.16251385 | 137.6722123 | 230.4047126 |
| YBR093C | PHO5 | 0.3278059 | 0.95675879 | 0.16265636 | 108.6369205 | 175.9234612 |
| YGR163W | GTR2 | 0.66025737 | 0.96992497 | 0.16302861 | 83.49872364 | 128.7120634 |
| YGL203C | KEX1 | 0.1951858 | 0.95675879 | 0.16328659 | 128.495137 | 213.0605243 |
| YLR171W | YLR171W | 0.45810073 | 0.95675879 | 0.16368768 | 129.8957034 | 215.6187189 |
| YAL068C | PAU8 | 0.26314392 | 0.95675879 | 0.1637257 | 128.3919021 | 212.7917738 |
| YLR065C | YLR065C | 0.27917491 | 0.95675879 | 0.16408115 | 99.86797901 | 159.2331834 |
| YGL230C | YGL230C | 0.24387653 | 0.95675879 | 0.1641819 | 134.2203315 | 223.6451774 |
| YDR108W | GSG1 | 0.29185438 | 0.95675879 | 0.16432949 | 111.8010618 | 181.5716742 |
| YKL103C | LAP4 | 0.25185671 | 0.95675879 | 0.16442803 | 112.7691997 | 183.3705972 |
| YDL086W | YDL086W | 0.19075494 | 0.95675879 | 0.16466703 | 131.8182377 | 219.0569501 |
| YNL037C | IDH1 | 0.05059714 | 0.9246749 | 0.1647364 | 99.80761066 | 159.0078514 |
| YOR366W | YOR366W | 0.43836858 | 0.95675879 | 0.16482012 | 119.0467544 | 195.0773218 |
| YCL010C | SGF29 | 0.47093567 | 0.95675879 | 0.16497908 | 18.16534564 | 5.842948761 |
| YDR124W | YDR124W | 0.31454959 | 0.95675879 | 0.16521212 | 132.5437827 | 220.3244771 |
| YNL322C | KRE1 | 0.07061958 | 0.95675879 | 0.16526648 | 119.0178497 | 194.9467398 |
| YBR213W | MET8 | 0.40240439 | 0.95675879 | 0.1653785 | 143.2639329 | 240.4020871 |

| | | | | | | |
|---|---|---|---|---|---|---|
| YLR429W | CRN1 | 0.73449667 | 0.97731085 | 0.16545698 | 116.8180908 | 190.7884103 |
| YLR082C | SRL2 | 0.39218977 | 0.95675879 | 0.16553665 | 115.5919184 | 188.4750423 |
| YHL005C | YHL005C | 0.66529342 | 0.96992497 | 0.16581227 | 94.54550736 | 148.9544883 |
| YLR144C | ACF2 | 0.18632309 | 0.95675879 | 0.16602953 | 110.2221765 | 178.3195449 |
| YBR056W | YBR056W | 0.25219782 | 0.95675879 | 0.1662117 | 133.2731886 | 221.5214849 |
| YML104C | MDM1 | 0.37856795 | 0.95675879 | 0.16624508 | 129.1697462 | 213.8196025 |
| YLR237W | THI7 | 0.4583198 | 0.95675879 | 0.16656951 | 129.5583885 | 214.4930084 |
| YNL255C | GIS2 | 0.32037806 | 0.95675879 | 0.16688742 | 116.3230659 | 189.6152303 |
| YLR335W | NUP2 | 0.37418799 | 0.95675879 | 0.1669889 | 111.6437826 | 180.8216833 |
| YKL187C | YKL187C | 0.12498001 | 0.95675879 | 0.16723755 | 116.6914414 | 190.2462297 |
| YLR133W | CKI1 | 0.30360382 | 0.95675879 | 0.16731981 | 117.3713882 | 191.5074222 |
| YHL043W | ECM34 | 0.75550145 | 0.97903138 | 0.1673306 | 120.2123527 | 196.8339212 |
| YER030W | CHZ1 | 0.13933235 | 0.95675879 | 0.16772255 | 144.0923534 | 241.5547703 |
| YKL159C | RCN1 | 0.38900135 | 0.95675879 | 0.16787313 | 111.3661541 | 180.1496923 |
| YGL059W | YGL059W | 0.38664188 | 0.95675879 | 0.16833984 | 142.2204581 | 237.9383404 |
| YBR182C | SMP1 | 0.58480788 | 0.95959383 | 0.16834046 | 129.6259513 | 214.3167263 |
| YDL178W | DLD2 | 0.17763422 | 0.95675879 | 0.16861491 | 141.0158238 | 235.6319379 |
| YJL036W | SNX4 | 0.34746466 | 0.95675879 | 0.1688976 | 137.0592561 | 228.1628679 |
| YJL030W | MAD2 | 0.34861196 | 0.95675879 | 0.16895118 | 120.1616396 | 196.4615362 |
| YGR144W | THI4 | 0.17457603 | 0.95675879 | 0.1690234 | 130.9089132 | 216.6061263 |
| YGL163C | RAD54 | 0.70013679 | 0.97267176 | 0.16907975 | 108.6992735 | 174.9414044 |
| YLR366W | YLR366W | 0.27306889 | 0.95675879 | 0.16911144 | 118.7067066 | 193.7053297 |
| YBR061C | TRM7 | 0.11317625 | 0.95675879 | 0.16953322 | 130.3259531 | 215.4255341 |
| YMR132C | JLP2 | 0.2142341 | 0.95675879 | 0.16960707 | 128.6222141 | 212.2174678 |
| YOR242C | SSP2 | 0.36638343 | 0.95675879 | 0.17005791 | 131.3827102 | 217.3177532 |
| YDL095W | PMT1 | 0.31465712 | 0.95675879 | 0.17020059 | 135.0164236 | 224.1085198 |
| YML029W | USA1 | 0.06673511 | 0.95675879 | 0.17022907 | 122.0948794 | 199.8687666 |
| YML118W | NGL3 | 0.02024568 | 0.67882086 | 0.17024203 | 120.6254892 | 197.110648 |
| YGR152C | RSR1 | 0.42374623 | 0.95675879 | 0.17043017 | 141.7332008 | 236.6668284 |
| YOR172W | YRM1 | 0.42628639 | 0.95675879 | 0.17050684 | 125.750963 | 206.6783738 |
| YKR035C | YKR035C | 0.16964333 | 0.95675879 | 0.17060359 | 101.1907587 | 160.5981616 |
| YMR156C | TPP1 | 0.06813128 | 0.95675879 | 0.17086942 | 119.5039256 | 194.8997678 |
| YBR053C | YBR053C | 0.11251811 | 0.95675879 | 0.17107129 | 128.1057375 | 210.9982761 |
| YDL180W | YDL180W | 0.15726807 | 0.95675879 | 0.17118431 | 134.5888197 | 223.1382216 |
| YGR169C | PUS6 | 0.39068365 | 0.95675879 | 0.1712301 | 130.9927673 | 216.3858466 |
| YIL113W | SDP1 | 0.38628727 | 0.95675879 | 0.17126362 | 118.6819015 | 193.2905823 |
| YOR308C | SNU66 | 0.23327339 | 0.95675879 | 0.17154879 | 109.792784 | 176.569891 |
| YAR031W | PRM9 | 0.35835783 | 0.95675879 | 0.17164153 | 135.8306121 | 225.3890263 |
| YER067C-A | YER067C-A | 0.55339281 | 0.95959383 | 0.17194267 | 130.210193 | 214.7961791 |
| YNL116W | DMA2 | 0.19710032 | 0.95675879 | 0.17201206 | 117.2166811 | 190.4144482 |
| YLR189C | ATG26 | 0.22415228 | 0.95675879 | 0.1722247 | 115.5772697 | 187.3032843 |
| YGR051C | YGR051C | 0.36790525 | 0.95675879 | 0.17227607 | 145.5151307 | 243.4441685 |
| YGR234W | YHB1 | 0.34917453 | 0.95675879 | 0.17242732 | 130.2002291 | 214.6945714 |
| YLR329W | REC102 | 0.20448265 | 0.95675879 | 0.17290901 | 123.7213773 | 202.4608073 |
| YML056C | IMD4 | 0.06338601 | 0.95675879 | 0.17355319 | 121.1866794 | 197.5966639 |
| YHR142W | CHS7 | 0.33930492 | 0.95675879 | 0.17396126 | 117.3818783 | 190.3907871 |
| YDR419W | RAD30 | 0.40314138 | 0.95675879 | 0.17399677 | 128.71083 | 211.63262 |
| YIL165C | YIL165C | 0.30402195 | 0.95675879 | 0.17419243 | 115.9399343 | 187.646811 |
| YPR150W | YPR150W | 0.44907331 | 0.95675879 | 0.1742628 | 124.541103 | 203.7666123 |
| YPR014C | YPR014C | 0.30503501 | 0.95675879 | 0.17433571 | 121.5093922 | 198.0680408 |
| YAL014C | SYN8 | 0.2911839 | 0.95675879 | 0.17457357 | 136.3425511 | 225.8475355 |
| YOR082C | YOR082C | 0.17705824 | 0.95675879 | 0.17480974 | 124.4950855 | 203.5867265 |
| YDR486C | VPS60 | 0.29008216 | 0.95675879 | 0.1748803 | 124.4124526 | 203.4196722 |
| YJL214W | HXT8 | 0.35657619 | 0.95675879 | 0.17501824 | 128.4205415 | 210.9134053 |
| YMR004W | MVP1 | 0.21716578 | 0.95675879 | 0.17502562 | 123.0215852 | 200.7861811 |
| YHR080C | YHR080C | 0.32887532 | 0.95675879 | 0.17518451 | 119.9008951 | 194.906014 |
| YHR076W | PTC7 | 0.28964916 | 0.95675879 | 0.17598419 | 128.378981 | 210.6701883 |
| YML101C | CUE4 | 0.23954232 | 0.95675879 | 0.17617835 | 122.1529786 | 198.9598485 |
| YOR085W | OST3 | 0.22417235 | 0.95675879 | 0.17627775 | 119.3700402 | 193.723328 |
| YLR309C | IMH1 | 0.07351443 | 0.95675879 | 0.17651208 | 112.8270842 | 181.4116567 |
| YKL091C | YKL091C | 0.42182702 | 0.95675879 | 0.17698919 | 110.2108963 | 176.4232588 |
| YGL090W | LIF1 | 0.41845099 | 0.95675879 | 0.17738766 | 138.9762568 | 230.3056839 |
| YNL165W | YNL165W | 0.26100104 | 0.95675879 | 0.17754814 | 122.7166766 | 199.7827252 |
| YDL037C | BSC1 | 0.11828418 | 0.95675879 | 0.177679 | 135.0700177 | 222.9295276 |

| | | | | | | |
|---|---|---|---|---|---|---|
| YGL227W | VID30 | 0.41729751 | 0.95675879 | 0.17776149 | 148.5770295 | 248.2483632 |
| YLR089C | ALT1 | 0.65586697 | 0.96992497 | 0.17819016 | 71.01022605 | 102.6953331 |
| YCR023C | YCR023C | 0.36473454 | 0.95675879 | 0.17819145 | 124.0828638 | 202.2349973 |
| YNL324W | YNL324W | 0.56221363 | 0.95959383 | 0.17857138 | 129.3665535 | 212.0797688 |
| YBR244W | GPX2 | 0.25252501 | 0.95675879 | 0.17869912 | 143.0223688 | 237.6699485 |
| YGL170C | SPO74 | 0.28438318 | 0.95675879 | 0.17880156 | 133.2808432 | 219.3817954 |
| YMR087W | YMR087W | 0.72967491 | 0.97683349 | 0.17930731 | 115.410507 | 185.7787245 |
| YAL015C | NTG1 | 0.27498803 | 0.95675879 | 0.17971022 | 128.5457519 | 210.345474 |
| YJR097W | JJJ3 | 0.46088255 | 0.95675879 | 0.17983386 | 128.9946431 | 211.1662335 |
| YIL034C | CAP2 | 0.10692515 | 0.95675879 | 0.1801318 | 119.7079678 | 193.6977212 |
| YKR102W | FLO10 | 0.21609765 | 0.95675879 | 0.18022003 | 108.0401539 | 171.7991668 |
| YDR406W | PDR15 | 0.48251136 | 0.95675879 | 0.18049462 | 146.9490139 | 244.7273321 |
| YIL001W | YIL001W | 0.27520052 | 0.95675879 | 0.18062763 | 127.2821593 | 207.8185918 |
| YJL218W | YJL218W | 0.35580959 | 0.95675879 | 0.18071943 | 132.6422953 | 217.8560375 |
| YMR271C | URA10 | 0.29535689 | 0.95675879 | 0.18110606 | 116.7469589 | 187.9775382 |
| YMR008C | PLB1 | 0.32218121 | 0.95675879 | 0.18133471 | 131.1715104 | 214.9922504 |
| YKL090W | CUE2 | 0.25566454 | 0.95675879 | 0.18153579 | 120.2690898 | 194.5099134 |
| YHR029C | YHI9 | 0.35290091 | 0.95675879 | 0.18156927 | 131.7258549 | 215.9918146 |
| YLR381W | CTF3 | 0.1296075 | 0.95675879 | 0.18178232 | 113.6412985 | 182.0370421 |
| YNL127W | FAR11 | 0.21240242 | 0.95675879 | 0.1819382 | 124.182364 | 201.7805684 |
| YKL062W | MSN4 | 0.21152146 | 0.95675879 | 0.18210539 | 115.8421677 | 186.1095867 |
| YNL228W | YNL228W | 0.60118562 | 0.95959383 | 0.18212914 | 72.1130415 | 104.0897728 |
| YGL020C | GET1 | 0.14712177 | 0.95675879 | 0.18223631 | 143.5031728 | 237.9665254 |
| YDL023C | SRF4 | 0.09669747 | 0.95675879 | 0.18237782 | 137.3236551 | 226.3523772 |
| YDR321W | ASP1 | 0.23660589 | 0.95675879 | 0.18250745 | 134.6701875 | 221.3535123 |
| YDL027C | YDL027C | 0.12792174 | 0.95675879 | 0.18254184 | 134.851169 | 221.687066 |
| YMR294W | JNM1 | 0.18159623 | 0.95675879 | 0.18273696 | 108.7093504 | 172.6236411 |
| YDR043C | NRG1 | 0.13856893 | 0.95675879 | 0.18274528 | 107.8776045 | 171.0622441 |
| YHR138C | YHR138C | 0.18573539 | 0.95675879 | 0.18279926 | 116.5715563 | 187.3588684 |
| YLL041C | SDH2 | 0.15092782 | 0.95675879 | 0.18287743 | 100.0659606 | 156.38858 |
| YPR011C | YPR011C | 0.28089304 | 0.95675879 | 0.1830564 | 126.4491795 | 205.8407555 |
| YMR209C | YMR209C | 0.16258838 | 0.95675879 | 0.1837683 | 134.6049465 | 221.0154274 |
| YLR181C | VTA1 | 0.20906123 | 0.95675879 | 0.18404622 | 113.9037988 | 182.142033 |
| YHR050W | SMF2 | 0.41025984 | 0.95675879 | 0.18410812 | 124.8289423 | 202.6219937 |
| YPL068C | YPL068C | 0.30365395 | 0.95675879 | 0.18421909 | 118.127935 | 190.0349953 |
| YFR033C | QCR6 | 0.09094356 | 0.95675879 | 0.18422957 | 143.162259 | 236.9860935 |
| YER119C-A | YER119C-A | 0.09115004 | 0.95675879 | 0.184307 | 105.8759304 | 167.0408235 |
| YDL130W | RPP1B | 0.43088145 | 0.95675879 | 0.18452752 | 89.79032951 | 136.8338952 |
| YKL162C | YKL162C | 0.28651201 | 0.95675879 | 0.18491633 | 116.8364434 | 187.4934571 |
| YHR025W | THR1 | 0.75660863 | 0.97903138 | 0.18506927 | 56.95732614 | 75.16157603 |
| YHR207C | SET5 | 0.36711367 | 0.95675879 | 0.1850932 | 144.3134107 | 238.9973633 |
| YDR272W | GLO2 | 0.32739123 | 0.95675879 | 0.18523225 | 131.5603196 | 215.0546333 |
| YKR076W | ECM4 | 0.38670545 | 0.95675879 | 0.18541128 | 124.399386 | 201.5933797 |
| YKR060W | UTP30 | 0.30940057 | 0.95675879 | 0.18558067 | 108.5280232 | 171.797014 |
| YDR206W | EBS1 | 0.32215745 | 0.95675879 | 0.18558816 | 130.130858 | 212.3127253 |
| YMR037C | MSN2 | 0.24291089 | 0.95675879 | 0.18575048 | 142.3132575 | 235.1335376 |
| YNR066C | YNR066C | 0.51488175 | 0.95675879 | 0.18577755 | 137.2844604 | 225.6971928 |
| YGL084C | GUP1 | 0.10972885 | 0.95675879 | 0.18597594 | 118.5954855 | 190.6113188 |
| YGR031W | YGR031W | 0.26653653 | 0.95675879 | 0.18612492 | 132.2306677 | 216.1591677 |
| YBR156C | SLI15 | 0.19688753 | 0.95675879 | 0.18630998 | 127.705672 | 207.6406908 |
| YKL205W | LOS1 | 0.3613115 | 0.95675879 | 0.18669822 | 105.1508933 | 165.2718633 |
| YJL107C | YJL107C | 0.43586485 | 0.95675879 | 0.1867422 | 131.9991642 | 215.6193599 |
| YIL128W | MET18 | 0.62079382 | 0.96098984 | 0.1869017 | 89.38335625 | 135.664394 |
| YIL050W | PCL7 | 0.717898 | 0.97530288 | 0.18705457 | 126.257439 | 204.7970771 |
| YJL149W | YJL149W | 0.07860489 | 0.95675879 | 0.18708991 | 128.7909704 | 209.5427709 |
| YER027C | GAL83 | 0.13524458 | 0.95675879 | 0.18710461 | 115.7778199 | 185.133565 |
| YNL279W | PRM1 | 0.37416641 | 0.95675879 | 0.18759192 | 113.1805417 | 180.1788886 |
| YIL010W | DOT5 | 0.32890047 | 0.95675879 | 0.18763111 | 115.9942045 | 185.4493223 |
| YKL071W | YKL071W | 0.19462512 | 0.95675879 | 0.18771151 | 121.9341435 | 196.5761629 |
| YJL201W | ECM25 | 0.48560993 | 0.95675879 | 0.1878909 | 151.3944013 | 251.7993789 |
| YJR110W | YMR1 | 0.286214 | 0.95675879 | 0.18802347 | 110.6287794 | 175.3191195 |
| YNL239W | LAP3 | 0.33046172 | 0.95675879 | 0.18808105 | 137.0585081 | 224.8792966 |
| YJL124C | LSM1 | 0.16331742 | 0.95675879 | 0.18826338 | 89.77724146 | 136.1701668 |
| YJL062W | LAS21 | 0.20394094 | 0.95675879 | 0.18839357 | 112.5299776 | 178.8215724 |

| | | | | | | |
|---|---|---|---|---|---|---|
| YDR022C | CIS1 | 0.30936487 | 0.95675879 | 0.18847757 | 121.4005957 | 195.4444042 |
| YKR051W | YKR051W | 0.28896917 | 0.95675879 | 0.18858778 | 116.291177 | 185.8426266 |
| YLR360W | VPS38 | 0.20597104 | 0.95675879 | 0.18895465 | 102.6187639 | 160.1366908 |
| YHR113W | YHR113W | 0.22514764 | 0.95675879 | 0.18917358 | 126.6355855 | 205.1437568 |
| YKL148C | SDH1 | 0.2159737 | 0.95675879 | 0.18928713 | 104.5531827 | 163.7078861 |
| YIL028W | YIL028W | 0.63917407 | 0.96454138 | 0.18986056 | 122.2639028 | 196.8269511 |
| YJL199C | MBB1 | 0.41818054 | 0.95675879 | 0.18988681 | 150.3243412 | 249.4509499 |
| YHR198C | FMP22 | 0.74208805 | 0.97903138 | 0.18989744 | 128.4789036 | 208.4771267 |
| YKL107W | YKL107W | 0.3310073 | 0.95675879 | 0.18998258 | 110.375524 | 174.5089372 |
| YKL070W | YKL070W | 0.18984028 | 0.95675879 | 0.19062598 | 119.296677 | 191.1308402 |
| YML048W-A | YML048W-A | 0.07093167 | 0.95675879 | 0.19167309 | 120.2629286 | 192.7639297 |
| YOR023C | AHC1 | 0.45428303 | 0.95675879 | 0.19175355 | 138.9811514 | 227.8569499 |
| YJL098W | SAP185 | 0.36414163 | 0.95675879 | 0.1917976 | 149.0610322 | 246.7546391 |
| YLR262C | YPT6 | 0.45252791 | 0.95675879 | 0.1919154 | 80.82841271 | 118.7614366 |
| YLL024C | SSA2 | 0.19535361 | 0.95675879 | 0.19220441 | 116.7649758 | 186.1124716 |
| YCR069W | CPR4 | 0.19780084 | 0.95675879 | 0.19227249 | 135.0891975 | 220.4686448 |
| YDL167C | NRP1 | 0.24240916 | 0.95675879 | 0.19235178 | 146.3868278 | 241.6442437 |
| YOR167C | RPS28A | 0.4385407 | 0.95675879 | 0.19289102 | 128.9355584 | 208.8214186 |
| YBR290W | BSD2 | 0.08172657 | 0.95675879 | 0.19289195 | 127.9604859 | 206.9924721 |
| YBR175W | SWD3 | 0.59250029 | 0.95959383 | 0.19296933 | 69.63699397 | 97.59115618 |
| YKL176C | LST4 | 0.14831441 | 0.95675879 | 0.19330957 | 112.8418617 | 178.5654253 |
| YGR125W | YGR125W | 0.18500773 | 0.95675879 | 0.19338698 | 145.865067 | 240.4885434 |
| YKL207W | YKL207W | 0.26588163 | 0.95675879 | 0.19339182 | 113.7030249 | 180.1665002 |
| YMR250W | GAD1 | 0.27532365 | 0.95675879 | 0.19367751 | 119.9229997 | 191.7834361 |
| YKR014C | YPT52 | 0.23264418 | 0.95675879 | 0.193747 | 113.5072954 | 179.7386321 |
| YLR017W | MEU1 | 0.36469218 | 0.95675879 | 0.19386094 | 132.8339548 | 215.9670714 |
| YKL050C | YKL050C | 0.26215549 | 0.95675879 | 0.19390762 | 112.2674721 | 177.3858117 |
| YCR089W | FIG2 | 0.30931631 | 0.95675879 | 0.19405097 | 154.4689902 | 256.5119462 |
| YDR319C | YDR319C | 0.2056886 | 0.95675879 | 0.19427667 | 129.6525875 | 209.9291591 |
| YJR005W | APL1 | 0.55048957 | 0.95947263 | 0.19429738 | 132.8037163 | 215.8356864 |
| YKL157W | APE2 | 0.22238058 | 0.95675879 | 0.19452245 | 123.7906488 | 198.8928034 |
| YGR244C | LSC2 | 0.29226287 | 0.95675879 | 0.19471858 | 128.8108527 | 208.2748434 |
| YDL183C | YDL183C | 0.60762623 | 0.95959383 | 0.19480871 | 87.99059342 | 131.6993706 |
| YDL175C | AIR2 | 0.34211633 | 0.95675879 | 0.19496261 | 144.3893703 | 237.4512334 |
| YBL005W | PDR3 | 0.48936438 | 0.95675879 | 0.19575283 | 137.6085805 | 224.5983846 |
| YKL124W | SSH4 | 0.14584111 | 0.95675879 | 0.19578312 | 114.1031406 | 180.5077974 |
| YGL194C | HOS2 | 0.00975919 | 0.54423319 | 0.19592998 | 112.700697 | 177.8523301 |
| YLR183C | TOS4 | 0.28498677 | 0.95675879 | 0.19611725 | 119.2436461 | 190.0918559 |
| YBR128C | ATG14 | 0.28396786 | 0.95675879 | 0.1961309 | 122.3728562 | 195.9584802 |
| YLR168C | YLR168C | 0.20853812 | 0.95675879 | 0.19631207 | 109.8046788 | 172.3553574 |
| YDR005C | MAF1 | 0.20914608 | 0.95675879 | 0.19644507 | 124.3265291 | 199.5689207 |
| YLR413W | YLR413W | 0.34535081 | 0.95675879 | 0.19654485 | 131.0532221 | 212.1680348 |
| YDR408C | ADE8 | 0.38301919 | 0.95675879 | 0.19696206 | 131.3324767 | 212.6204058 |
| YBL017C | PEP1 | 0.28836802 | 0.95675879 | 0.19723465 | 133.236095 | 216.1440815 |
| YDL191W | RPL35A | 0.17271248 | 0.95675879 | 0.19738845 | 110.2998546 | 173.0999179 |
| YLR344W | RPL26A | 0.07403611 | 0.95675879 | 0.19747547 | 107.0014064 | 166.8986559 |
| YGL019W | CKB1 | 0.2665148 | 0.95675879 | 0.19762892 | 105.9360922 | 164.8743609 |
| YIL090W | ICE2 | 0.50752933 | 0.95675879 | 0.19769995 | 88.84717241 | 132.8112456 |
| YNL302C | RPS19B | 0.17876477 | 0.95675879 | 0.1977062 | 93.00199874 | 140.6027215 |
| YMR060C | SAM37 | 0.08134835 | 0.95675879 | 0.19818781 | 97.51998566 | 148.9939899 |
| YJR121W | ATP2 | 0.05496356 | 0.95675879 | 0.19831997 | 119.4604378 | 190.1215863 |
| YBR162C | TOS1 | 0.18199644 | 0.95675879 | 0.19860634 | 132.1534736 | 213.878893 |
| YLL013C | PUF3 | 0.16670574 | 0.95675879 | 0.19945583 | 121.3777204 | 193.5231883 |
| YDL131W | LYS21 | 0.18650011 | 0.95675879 | 0.19952671 | 127.7036824 | 205.3756602 |
| YDL170W | UGA3 | 0.31549035 | 0.95675879 | 0.1995561 | 140.767229 | 229.8718427 |
| YLR179C | YLR179C | 0.15647665 | 0.95675879 | 0.19958103 | 109.1213992 | 170.5145396 |
| YNL089C | YNL089C | 0.42153799 | 0.95675879 | 0.19977194 | 114.0168429 | 179.6634797 |
| YMR225C | MRPL44 | 0.11277595 | 0.95675879 | 0.19995944 | 134.6756992 | 218.377923 |
| YIL135C | VHS2 | 0.39854995 | 0.95675879 | 0.2001265 | 140.8972279 | 230.018069 |
| YKR087C | OMA1 | 0.36859222 | 0.95675879 | 0.20034053 | 120.4832718 | 191.6942471 |
| YHR181W | SVP26 | 0.10784203 | 0.95675879 | 0.20051521 | 129.9475846 | 209.4150626 |
| YOR246C | YOR246C | 0.38943379 | 0.95675879 | 0.20094708 | 135.5714965 | 219.8890476 |
| YHR179W | OYE2 | 0.3226219 | 0.95675879 | 0.20111792 | 125.3712833 | 200.7289041 |
| YLR392C | YLR392C | 0.31458796 | 0.95675879 | 0.20120588 | 131.1260449 | 211.5071447 |

| | | | | | | |
|---|---|---|---|---|---|---|
| YIL037C | PRM2 | 0.1498207 | 0.95675879 | 0.20133657 | 126.0687498 | 201.9996211 |
| YIL136W | OM45 | 0.15248355 | 0.95675879 | 0.20146715 | 122.1385753 | 194.6060785 |
| YBR240C | THI2 | 0.35159172 | 0.95675879 | 0.20148712 | 138.2943397 | 224.903454 |
| YJL162C | JJJ2 | 0.37356943 | 0.95675879 | 0.20154802 | 135.029816 | 218.7702873 |
| YNL035C | YNL035C | 0.20376818 | 0.95675879 | 0.20172852 | 120.5743301 | 191.6275538 |
| YLR213C | CRR1 | 0.28976336 | 0.95675879 | 0.20203628 | 130.9722146 | 211.0765522 |
| YBR048W | RPS11B | 0.29166494 | 0.95675879 | 0.20234376 | 111.4985391 | 174.5002772 |
| YDL052C | SLC1 | 0.40003043 | 0.95675879 | 0.20244868 | 37.24868011 | 35.22370188 |
| YDR391C | YDR391C | 0.3746617 | 0.95675879 | 0.20246433 | 129.6842705 | 208.5877242 |
| YMR101C | SRT1 | 0.11992017 | 0.95675879 | 0.20262641 | 132.996308 | 214.771855 |
| YGL117W | YGL117W | 0.43709092 | 0.95675879 | 0.20291907 | 142.6675747 | 232.8606348 |
| YBR217W | ATG12 | 0.34452482 | 0.95675879 | 0.20313863 | 136.0102837 | 220.3370498 |
| YBR001C | NTH2 | 0.26868106 | 0.95675879 | 0.20322889 | 124.7649215 | 199.2304752 |
| YDL114W | YDL114W | 0.26855943 | 0.95675879 | 0.2035352 | 141.5284024 | 230.6186547 |
| YDL174C | DLD1 | 0.2461947 | 0.95675879 | 0.20361784 | 128.765169 | 206.6665542 |
| YKL127W | PGM1 | 0.27195312 | 0.95675879 | 0.20374243 | 119.5336486 | 189.3311458 |
| YLR180W | SAM1 | 0.23468237 | 0.95675879 | 0.20374665 | 113.4620252 | 177.9428489 |
| YDL076C | RXT3 | 0.11445749 | 0.95675879 | 0.20387611 | 145.2001446 | 237.4468293 |
| YKL208W | CBT1 | 0.58393385 | 0.95959383 | 0.20388424 | 90.6092767 | 135.0580498 |
| YDR173C | ARG82 | 0.58563962 | 0.95959383 | 0.20398206 | 83.5372872 | 121.7775102 |
| YMR075W | RCO1 | 0.09113163 | 0.95675879 | 0.20406914 | 116.1373606 | 182.9053721 |
| YML005W | TRM12 | 0.17698018 | 0.95675879 | 0.20413366 | 130.6608111 | 210.1336542 |
| YMR056C | AAC1 | 0.47626993 | 0.95675879 | 0.20432446 | 146.6385465 | 240.0679009 |
| YGL126W | SCS3 | 0.16055598 | 0.95675879 | 0.20438415 | 140.4057886 | 228.3678975 |
| YDR018C | YDR018C | 0.35145288 | 0.95675879 | 0.20454364 | 132.1398963 | 212.8375943 |
| YDR471W | RPL27B | 0.29795181 | 0.95675879 | 0.20495957 | 112.2216399 | 175.4089319 |
| YDR122W | KIN1 | 0.25713886 | 0.95675879 | 0.20496496 | 136.8236399 | 221.5500595 |
| YKL044W | YKL044W | 0.20116128 | 0.95675879 | 0.20520396 | 117.5211435 | 185.3065526 |
| YDL130W-A | STF1 | 0.15481159 | 0.95675879 | 0.20556197 | 134.8651863 | 217.7747556 |
| YNR005C | YNR005C | 0.18173101 | 0.95675879 | 0.20627367 | 111.7881097 | 174.3709946 |
| YMR041C | YMR041C | 0.04786716 | 0.92220427 | 0.20634332 | 145.037956 | 236.7205139 |
| YHR115C | DMA1 | 0.26439433 | 0.95675879 | 0.20651667 | 138.3028888 | 224.0589626 |
| YER019W | ISC1 | 0.26010669 | 0.95675879 | 0.20672604 | 132.6756629 | 213.4690501 |
| YBR272C | HSM3 | 0.08218497 | 0.95675879 | 0.20756559 | 130.9719534 | 210.1300323 |
| YKR057W | RPS21A | 0.35233434 | 0.95675879 | 0.20772966 | 100.7044357 | 153.3340027 |
| YPR068C | HOS1 | 0.31263548 | 0.95675879 | 0.20780217 | 117.9194239 | 185.6090057 |
| YLR299W | ECM38 | 0.14516358 | 0.95675879 | 0.20809176 | 122.3152369 | 193.8039847 |
| YLR080W | EMP46 | 0.25649316 | 0.95675879 | 0.20844987 | 119.5766319 | 188.606349 |
| YML004C | GLO1 | 0.10598686 | 0.95675879 | 0.20848839 | 122.132113 | 193.3926675 |
| YLR131C | ACE2 | 0.02447418 | 0.750658 | 0.20935842 | 113.3300415 | 176.7351692 |
| YPL267W | ACM1 | 0.43400592 | 0.95675879 | 0.209546 | 124.2621869 | 197.2067567 |
| YDR307W | YDR307W | 0.35796355 | 0.95675879 | 0.20962353 | 128.6148532 | 205.3570941 |
| YKR069W | MET1 | 0.31047648 | 0.95675879 | 0.21050662 | 122.766532 | 194.2372404 |
| YDL189W | RBS1 | 0.19155681 | 0.95675879 | 0.2107766 | 132.2356388 | 211.950741 |
| YDR385W | EFT2 | 0.32882787 | 0.95675879 | 0.21087719 | 127.4421192 | 202.9430903 |
| YKL188C | PXA2 | 0.09532423 | 0.95675879 | 0.21096358 | 115.2621871 | 180.0843539 |
| YHR035W | YHR035W | 0.08449068 | 0.95675879 | 0.21103503 | 127.499559 | 203.0238162 |
| YEL053C | MAK10 | 0.13169586 | 0.95675879 | 0.21116358 | 136.2001717 | 219.3201736 |
| YKL218C | SRY1 | 0.13100444 | 0.95675879 | 0.21139327 | 118.5071434 | 186.0968835 |
| YDL077C | VAM6 | 0.03975175 | 0.89368591 | 0.21140804 | 98.02211296 | 147.6738498 |
| YMR326C | YMR326C | 0.11363437 | 0.95675879 | 0.21143307 | 113.0541179 | 175.8627038 |
| YBL036C | YBL036C | 0.24168556 | 0.95675879 | 0.21179676 | 132.5037168 | 212.2789906 |
| YKL177W | YKL177W | 0.1166086 | 0.95675879 | 0.2119907 | 130.1477953 | 207.827181 |
| YBR166C | TYR1 | 0.31459137 | 0.95675879 | 0.21215846 | 140.6174722 | 227.4347821 |
| YLR374C | YLR374C | 0.21382061 | 0.95675879 | 0.21219285 | 106.2862724 | 163.0393409 |
| YMR055C | BUB2 | 0.04137173 | 0.91069949 | 0.21248037 | 124.815767 | 197.7429669 |
| YNL176C | YNL176C | 0.31046075 | 0.95675879 | 0.21267365 | 121.9271867 | 192.2922486 |
| YMR233W | YMR233W | 0.12519252 | 0.95675879 | 0.21277764 | 137.6917179 | 221.8414743 |
| YBR137W | YBR137W | 0.17934404 | 0.95675879 | 0.21351428 | 131.6982974 | 210.4745359 |
| YKR053C | YSR3 | 0.34549545 | 0.95675879 | 0.21361652 | 114.636158 | 178.4563087 |
| YKL048C | ELM1 | 0.07928887 | 0.95675879 | 0.21371821 | 94.39097017 | 140.4682386 |
| YHR146W | CRP1 | 0.35438364 | 0.95675879 | 0.21394342 | 132.6150315 | 212.1204841 |
| YLR363C | NMD4 | 0.26013261 | 0.95675879 | 0.21395888 | 129.3959512 | 206.0803241 |
| YKR049C | FMP46 | 0.10461396 | 0.95675879 | 0.21468242 | 121.8439233 | 191.7923966 |

| | | | | | | |
|---|---|---|---|---|---|---|
| YHR163W | SOL3 | 0.17320087 | 0.95675879 | 0.2150126 | 126.9876918 | 201.3832518 |
| YJL022W | YJL022W | 0.40197825 | 0.95675879 | 0.2151537 | 147.5584645 | 239.9404293 |
| YLR395C | COX8 | 0.19329523 | 0.95675879 | 0.21531978 | 117.9152825 | 184.3150227 |
| YJL157C | FAR1 | 0.31153257 | 0.95675879 | 0.21560733 | 137.6907222 | 221.3554633 |
| YFR056C | YFR056C | 0.18151567 | 0.95675879 | 0.21604982 | 136.6754913 | 219.3756507 |
| YKR067W | GPT2 | 0.22197607 | 0.95675879 | 0.21618832 | 122.2723588 | 192.3382944 |
| YNL335W | DDI3 | 0.31536373 | 0.95675879 | 0.21642488 | 129.7249177 | 206.2753974 |
| YKL149C | DBR1 | 0.11424089 | 0.95675879 | 0.21680763 | 106.9872858 | 163.5645595 |
| YML094W | GIM5 | 0.052059 | 0.93531943 | 0.21727059 | 119.6946299 | 187.3184898 |
| YJL060W | BNA3 | 0.41887886 | 0.95675879 | 0.21754035 | 134.5269208 | 215.0908982 |
| YDR006C | SOK1 | 0.36594193 | 0.95675879 | 0.21757499 | 140.7256988 | 226.7110319 |
| YDR257C | SET7 | 0.17641067 | 0.95675879 | 0.21776283 | 128.283806 | 203.3436192 |
| YLR333C | RPS25B | 0.15956573 | 0.95675879 | 0.21826086 | 113.2223952 | 175.0101208 |
| YGL051W | MST27 | 0.20362786 | 0.95675879 | 0.21828485 | 135.6560704 | 217.0812847 |
| YML003W | YML003W | 0.01143769 | 0.59540465 | 0.21830378 | 118.2852055 | 184.4982848 |
| YOR127W | RGA1 | 0.21566562 | 0.95675879 | 0.21831552 | 127.8084901 | 202.3575832 |
| YPR140W | TAZ1 | 0.10873647 | 0.95675879 | 0.21835901 | 124.3008865 | 195.7714881 |
| YBR071W | YBR071W | 0.20691018 | 0.95675879 | 0.21846413 | 127.5416385 | 201.8316657 |
| YDR218C | SPR28 | 0.36995477 | 0.95675879 | 0.21853029 | 140.7899436 | 226.6680794 |
| YCR059C | YIH1 | 0.13850157 | 0.95675879 | 0.21871031 | 139.4523924 | 224.1286478 |
| YLR099C | ICT1 | 0.16554895 | 0.95675879 | 0.21879243 | 109.483805 | 167.9072956 |
| YMR121C | RPL15B | 0.03248125 | 0.79965484 | 0.21891648 | 111.94086 | 172.4943777 |
| YKL147C | YKL147C | 0.21241283 | 0.95675879 | 0.21904181 | 127.0686351 | 200.8456908 |
| YNL094W | APP1 | 0.46003165 | 0.95675879 | 0.2192397 | 136.06912 | 217.6926084 |
| YGR226C | YGR226C | 0.21206387 | 0.95675879 | 0.21925512 | 137.2442975 | 219.8940632 |
| YDL117W | CYK3 | 0.28795972 | 0.95675879 | 0.21973034 | 119.4968069 | 186.5266168 |
| YJL099W | CHS6 | 0.16876992 | 0.95675879 | 0.21982539 | 120.5120088 | 188.4144064 |
| YMR007W | YMR007W | 0.35593487 | 0.95675879 | 0.22036257 | 125.8663371 | 198.3647579 |
| YDL070W | BDF2 | 0.23686833 | 0.95675879 | 0.22057664 | 114.1148838 | 176.2878039 |
| YNL145W | MFA2 | 0.13058934 | 0.95675879 | 0.22072583 | 119.3237909 | 186.0317963 |
| YLR416C | YLR416C | 0.04401495 | 0.92220427 | 0.2208238 | 137.7093086 | 220.4978177 |
| YHR176W | FMO1 | 0.24831452 | 0.95675879 | 0.22089902 | 124.5135597 | 195.7357853 |
| YNL203C | YNL203C | 0.16767349 | 0.95675879 | 0.22113039 | 122.1324898 | 191.2304074 |
| YDL135C | RDI1 | 0.07852871 | 0.95675879 | 0.22116602 | 138.5449193 | 222.0064879 |
| YHR030C | SLT2 | 0.67510771 | 0.96992497 | 0.22136262 | 128.8412485 | 203.7732231 |
| YDR102C | YDR102C | 0.42802024 | 0.95675879 | 0.2215081 | 133.2014141 | 211.9260002 |
| YLR300W | EXG1 | 0.39237991 | 0.95675879 | 0.22154719 | 137.3689688 | 219.7357291 |
| YCR014C | POL4 | 0.09983468 | 0.95675879 | 0.22165278 | 137.3255309 | 219.6361941 |
| YNL164C | IBD2 | 0.1590904 | 0.95675879 | 0.22223195 | 132.5379028 | 210.557711 |
| YGR069W | YGR069W | 0.10040281 | 0.95675879 | 0.2223055 | 131.5569679 | 208.7053442 |
| YGL124C | MON1 | 0.15092941 | 0.95675879 | 0.22231529 | 101.2124528 | 151.7912994 |
| YJR025C | BNA1 | 0.17115201 | 0.95675879 | 0.22245736 | 112.7269533 | 173.3629057 |
| YML107C | PML39 | 0.0666658 | 0.95675879 | 0.22267101 | 112.7103052 | 173.2951264 |
| YLR279W | YLR279W | 0.25642019 | 0.95675879 | 0.22268212 | 134.2981355 | 213.7820783 |
| YMR126C | DLT1 | 0.32761075 | 0.95675879 | 0.22278448 | 113.5737527 | 174.8951442 |
| YDR143C | SAN1 | 0.48184468 | 0.95675879 | 0.22297899 | 147.9560596 | 239.3472752 |
| YGL023C | PIB2 | 0.24276525 | 0.95675879 | 0.22328183 | 138.8486435 | 222.2141337 |
| YMR172C-A | YMR172C-A | 0.09519165 | 0.95675879 | 0.223652 | 127.8611081 | 201.5432316 |
| YGL249W | ZIP2 | 0.04550555 | 0.92220427 | 0.22370934 | 142.4248837 | 228.8483731 |
| YHR057C | CPR2 | 0.18168064 | 0.95675879 | 0.22399267 | 127.1425461 | 200.1372528 |
| YLR044C | PDC1 | 0.33594002 | 0.95675879 | 0.22422552 | 101.8672431 | 152.6925559 |
| YGR281W | YOR1 | 0.03177028 | 0.79314877 | 0.22451163 | 126.2567581 | 198.38713 |
| YPR123C | YPR123C | 0.10018521 | 0.95675879 | 0.22452685 | 119.0027776 | 184.7793922 |
| YER061C | CEM1 | 0.03050825 | 0.78255549 | 0.22461688 | 94.42137806 | 138.6605753 |
| YKL040C | NFU1 | 0.1921168 | 0.95675879 | 0.2246212 | 121.6738773 | 189.7730044 |
| YJR053W | BFA1 | 0.20586182 | 0.95675879 | 0.22497986 | 118.1445877 | 183.0923139 |
| YHR133C | NSG1 | 0.21008195 | 0.95675879 | 0.22517499 | 134.6422444 | 214.0009534 |
| YMR092C | AIP1 | 0.4751504 | 0.95675879 | 0.22537394 | 116.9201673 | 180.7284399 |
| YKL098W | MTC2 | 0.20172801 | 0.95675879 | 0.2254224 | 115.794014 | 178.6080019 |
| YDL142C | CRD1 | 0.18472389 | 0.95675879 | 0.22572938 | 143.2992457 | 230.1426586 |
| YFR010W | UBP6 | 0.09978925 | 0.95675879 | 0.22586399 | 107.1227562 | 162.2691529 |
| YDR289C | RTT103 | 0.26498 | 0.95675879 | 0.22697402 | 132.7899823 | 210.2191577 |
| YKL038W | RGT1 | 0.02003105 | 0.67882086 | 0.22735897 | 114.2288566 | 175.3411499 |
| YDL093W | PMT5 | 0.25706982 | 0.95675879 | 0.22777188 | 141.1483015 | 225.7590156 |

| | | | | | | |
|---|---|---|---|---|---|---|
| YKR020W | VPS51 | 0.12879017 | 0.95675879 | 0.22783884 | 99.47907885 | 147.5952416 |
| YJL130C | URA2 | 0.36414895 | 0.95675879 | 0.22802181 | 136.7024963 | 217.3779658 |
| YMR272C | SCS7 | 0.115451 | 0.95675879 | 0.22804311 | 118.6939063 | 183.5984805 |
| YGL010W | YGL010W | 0.17409063 | 0.95675879 | 0.22811109 | 131.7597253 | 208.0923232 |
| YDL192W | ARF1 | 0.16485487 | 0.95675879 | 0.22830411 | 101.8098139 | 151.8870254 |
| YHR182W | YHR182W | 0.14668267 | 0.95675879 | 0.22832883 | 132.8475262 | 210.0952846 |
| YDR492W | IZH1 | 0.06172891 | 0.95675879 | 0.22836918 | 129.5534733 | 203.9102512 |
| YML100W | TSL1 | 0.00175471 | 0.27255016 | 0.228453 | 130.9822954 | 206.5757237 |
| YGR217W | CCH1 | 0.27218701 | 0.95675879 | 0.22851176 | 134.8691733 | 213.8556667 |
| YBR171W | SEC66 | 0.14231594 | 0.95675879 | 0.22866541 | 140.4687642 | 224.3316387 |
| YIL002C | INP51 | 0.43069309 | 0.95675879 | 0.22872302 | 130.5637779 | 205.7445785 |
| YKL100C | YKL100C | 0.20008359 | 0.95675879 | 0.22928627 | 128.002984 | 200.8453363 |
| YHR081W | LRP1 | 0.55872524 | 0.95959383 | 0.22959559 | 89.08906123 | 127.8077721 |
| YAL002W | VPS8 | 0.11577322 | 0.95675879 | 0.23006259 | 101.2423325 | 150.5218249 |
| YDR125C | ECM18 | 0.2900499 | 0.95675879 | 0.23040473 | 138.7084304 | 220.7324747 |
| YJR106W | ECM27 | 0.08863382 | 0.95675879 | 0.23041983 | 129.6481686 | 203.7370022 |
| YLL026W | HSP104 | 0.09648699 | 0.95675879 | 0.23061485 | 124.5412824 | 194.1254632 |
| YHR194W | MDM31 | 0.62008157 | 0.96098984 | 0.23076627 | 72.05714168 | 95.66342048 |
| YFL049W | SWP82 | 0.16425388 | 0.95675879 | 0.23112387 | 135.1742166 | 213.9808719 |
| YLL032C | YLL032C | 0.29807528 | 0.95675879 | 0.23136837 | 126.7895446 | 198.2132468 |
| YCR044C | PER1 | 0.04721708 | 0.92220427 | 0.2314309 | 119.3620945 | 184.2720649 |
| YMR068W | AVO2 | 0.03957129 | 0.89368591 | 0.23150515 | 127.6597875 | 199.8220203 |
| YDR258C | HSP78 | 0.04526755 | 0.92220427 | 0.23150998 | 125.6489311 | 196.0497511 |
| YBL062W | YBL062W | 0.24703366 | 0.95675879 | 0.23165353 | 140.8686181 | 224.5703322 |
| YDR359C | VID21 | 0.57638695 | 0.95959383 | 0.23181341 | 48.62142016 | 51.52961495 |
| YFR035C | YFR035C | 0.16747244 | 0.95675879 | 0.23206779 | 125.7029091 | 196.0555513 |
| YNL318C | HXT14 | 0.33722676 | 0.95675879 | 0.23227854 | 120.3865244 | 186.048398 |
| YMR295C | YMR295C | 0.38732285 | 0.95675879 | 0.2324606 | 134.9215553 | 213.2782902 |
| YKL132C | RMA1 | 0.07256222 | 0.95675879 | 0.23246123 | 122.0521679 | 189.1411229 |
| YLR218C | YLR218C | 0.45752187 | 0.95675879 | 0.23272003 | 38.79680892 | 32.94803982 |
| YOR350C | MNE1 | 0.25525319 | 0.95675879 | 0.23300908 | 118.8608344 | 183.0619141 |
| YDL125C | HNT1 | 0.11184132 | 0.95675879 | 0.23320165 | 150.1731337 | 241.756455 |
| YGL173C | KEM1 | 0.46658269 | 0.95675879 | 0.23353083 | 64.96319095 | 81.88542942 |
| YNL044W | YIP3 | 0.45678422 | 0.95675879 | 0.23385785 | 124.0578404 | 192.6638913 |
| YER129W | SAK1 | 0.11764698 | 0.95675879 | 0.23388235 | 127.9046247 | 199.8744991 |
| YHR009C | YHR009C | 0.54663905 | 0.95854866 | 0.23390387 | 107.1190723 | 160.8866697 |
| YKL031W | YKL031W | 0.19198038 | 0.95675879 | 0.23398558 | 122.1331863 | 189.0322692 |
| YKL072W | STB6 | 0.22620721 | 0.95675879 | 0.23403323 | 124.232832 | 192.9620886 |
| YLL040C | VPS13 | 0.18304833 | 0.95675879 | 0.23471842 | 113.1262632 | 172.0140351 |
| YHR044C | DOG1 | 0.66430398 | 0.96992497 | 0.23476224 | 131.7715798 | 206.9765856 |
| YKL069W | YKL069W | 0.24913035 | 0.95675879 | 0.23498735 | 98.78575245 | 145.0718136 |
| YBR228W | SLX1 | 0.19103225 | 0.95675879 | 0.23506534 | 138.0953585 | 218.7852308 |
| YGL006W | PMC1 | 0.19309578 | 0.95675879 | 0.23508175 | 126.3321651 | 196.720077 |
| YIL117C | PRM5 | 0.32425134 | 0.95675879 | 0.23514539 | 144.9701292 | 231.6654477 |
| YMR030W | RSF1 | 0.00516341 | 0.41833367 | 0.23515031 | 128.9482349 | 201.6148919 |
| YBR232C | YBR232C | 0.02986559 | 0.78255549 | 0.23519527 | 141.6085989 | 225.3522257 |
| YNL274C | YNL274C | 0.15878527 | 0.95675879 | 0.235248 | 137.4742229 | 217.5890142 |
| YKR011C | YKR011C | 0.27530901 | 0.95675879 | 0.23527428 | 126.3543063 | 196.7286638 |
| YGR049W | SCM4 | 0.11095284 | 0.95675879 | 0.23561132 | 141.0582587 | 224.248858 |
| YNL292W | PUS4 | 0.28692283 | 0.95675879 | 0.23583775 | 121.3698135 | 187.2836385 |
| YLR062C | BUD28 | 0.1493467 | 0.95675879 | 0.23584668 | 124.6859924 | 193.5017383 |
| YIL060W | YIL060W | 0.21539028 | 0.95675879 | 0.23619206 | 131.0101835 | 205.3039232 |
| YNL015W | PBI2 | 0.38651596 | 0.95675879 | 0.23622894 | 125.8911001 | 195.6965656 |
| YFR024C | YFR024C | 0.6020342 | 0.95959383 | 0.23629292 | 101.6262466 | 150.1759014 |
| YML120C | NDI1 | 0.32574486 | 0.95675879 | 0.23640992 | 135.1251945 | 212.984519 |
| YLL001W | DNM1 | 0.28061096 | 0.95675879 | 0.23646398 | 116.2083509 | 177.4959615 |
| YMR048W | CSM3 | 0.65749696 | 0.96992497 | 0.23654741 | 131.493967 | 206.1504806 |
| YGL257C | MNT2 | 0.30162543 | 0.95675879 | 0.23664462 | 149.1318823 | 239.214474 |
| YFR057W | YFR057W | 0.17940711 | 0.95675879 | 0.23678069 | 140.2870431 | 222.6023397 |
| YCL050C | APA1 | 0.2709909 | 0.95675879 | 0.23686808 | 147.5364975 | 236.1840336 |
| YHL002W | HSE1 | 0.10465592 | 0.95675879 | 0.2370209 | 122.5956799 | 189.3803712 |
| YIL045W | PIG2 | 0.12058982 | 0.95675879 | 0.23743702 | 132.9798045 | 208.785022 |
| YFR030W | MET10 | 0.1906491 | 0.95675879 | 0.23749531 | 123.9898092 | 191.9139487 |
| YJL158C | CIS3 | 0.43929298 | 0.95675879 | 0.23753655 | 146.8198102 | 234.7254862 |

| | | | | | | |
|---|---|---|---|---|---|---|
| YJL197W | UBP12 | 0.31562854 | 0.95675879 | 0.23778186 | 153.5245544 | 247.2585356 |
| YMR152W | YIM1 | 0.08153139 | 0.95675879 | 0.23787075 | 126.3918245 | 196.3547912 |
| YDL059C | RAD59 | 0.05827769 | 0.95675879 | 0.23806129 | 151.8207432 | 244.0151597 |
| YBR264C | YPT10 | 0.30115874 | 0.95675879 | 0.23816567 | 156.6189381 | 252.9965098 |
| YNL265C | IST1 | 0.33020138 | 0.95675879 | 0.23832904 | 129.7626151 | 202.5984338 |
| YNL064C | YDJ1 | 0.38096472 | 0.95675879 | 0.23837704 | 79.65208204 | 108.6058839 |
| YLR172C | DPH5 | 0.07401436 | 0.95675879 | 0.23848491 | 113.6495114 | 172.3509856 |
| YGL057C | YGL057C | 0.12853018 | 0.95675879 | 0.23877437 | 128.2640626 | 199.7116459 |
| YGL083W | SCY1 | 0.33707896 | 0.95675879 | 0.23904532 | 144.8589659 | 230.7897014 |
| YJL206C-A | YJL206C-A | 0.32245212 | 0.95675879 | 0.23915671 | 145.4877975 | 231.9500425 |
| YJL135W | YJL135W | 0.19679754 | 0.95675879 | 0.23933659 | 126.8258573 | 196.9180399 |
| YBL085W | BOI1 | 0.2868175 | 0.95675879 | 0.2402381 | 134.421505 | 211.0097434 |
| YKL056C | TMA19 | 0.21894856 | 0.95675879 | 0.24032061 | 110.5580924 | 166.238827 |
| YCL062W | YCL062W | 0.00228601 | 0.31772128 | 0.2403569 | 112.0513395 | 169.0332635 |
| YOR248W | YOR248W | 0.15202789 | 0.95675879 | 0.24073795 | 128.8562022 | 200.4862707 |
| YDR294C | DPL1 | 0.19308139 | 0.95675879 | 0.24115344 | 129.4489086 | 201.5268276 |
| YCL056C | YCL056C | 0.35570036 | 0.95675879 | 0.24123484 | 142.5054442 | 226.0009636 |
| YJR010C-A | SPC1 | 0.13469871 | 0.95675879 | 0.24195098 | 133.7108732 | 209.3838608 |
| YDR169C | STB3 | 0.34031677 | 0.95675879 | 0.24230122 | 133.4027333 | 208.7460093 |
| YNL297C | MON2 | 0.06846503 | 0.95675879 | 0.24255709 | 98.92841566 | 144.0442482 |
| YJR125C | ENT3 | 0.13879629 | 0.95675879 | 0.24259097 | 111.6214173 | 167.8446916 |
| YNL277W | MET2 | 0.24129917 | 0.95675879 | 0.24274871 | 127.8912365 | 198.3324104 |
| YGR263C | SAY1 | 0.11595623 | 0.95675879 | 0.24300432 | 127.6416157 | 197.820503 |
| YDR363W-A | SEM1 | 0.24671533 | 0.95675879 | 0.24303345 | 125.2555051 | 193.3402721 |
| YDL122W | UBP1 | 0.18251381 | 0.95675879 | 0.24361295 | 142.9773572 | 226.4791748 |
| YMR244C-A | YMR244C-A | 0.09514061 | 0.95675879 | 0.24373704 | 116.4660251 | 176.734865 |
| YHL017W | YHL017W | 0.65292983 | 0.96992497 | 0.24404036 | 125.7505581 | 194.0964869 |
| YOR334W | MRS2 | 0.25448383 | 0.95675879 | 0.2441604 | 113.6798332 | 171.4368153 |
| YMR114C | YMR114C | 0.04437504 | 0.92220427 | 0.24464718 | 126.373485 | 195.1609891 |
| YML033W | YML033W | 0.21919252 | 0.95675879 | 0.24487583 | 117.4166155 | 178.3228963 |
| YDL020C | RPN4 | 0.52165708 | 0.95675879 | 0.24496472 | 89.8481819 | 126.601972 |
| YNL330C | RPD3 | 0.25725794 | 0.95675879 | 0.24569165 | 120.3855164 | 183.751608 |
| YPR018W | RLF2 | 0.04868416 | 0.92220427 | 0.2460209 | 115.6582716 | 174.8291369 |
| YNL121C | TOM70 | 0.56612271 | 0.95959383 | 0.24669587 | 116.1133668 | 175.5672021 |
| YKL129C | MYO3 | 0.48312053 | 0.95675879 | 0.24676774 | 121.1129219 | 184.9317753 |
| YKL020C | SPT23 | 0.17806756 | 0.95675879 | 0.24681787 | 123.5619345 | 189.5164195 |
| YLR239C | LIP2 | 0.47819196 | 0.95675879 | 0.24702502 | 62.07817536 | 74.16569247 |
| YHR034C | PIH1 | 0.06057719 | 0.95675879 | 0.2470565 | 97.28327402 | 140.1888982 |
| YHR185C | PFS1 | 0.11255398 | 0.95675879 | 0.24721236 | 136.6978677 | 214.085901 |
| YJL147C | YJL147C | 0.23852034 | 0.95675879 | 0.24753314 | 129.8153169 | 201.122514 |
| YMR034C | YMR034C | 0.14490909 | 0.95675879 | 0.24753783 | 134.0051521 | 208.9799186 |
| YLR185W | RPL37A | 0.31358114 | 0.95675879 | 0.24808107 | 82.07003682 | 111.4805569 |
| YJR148W | BAT2 | 0.21227261 | 0.95675879 | 0.2482283 | 146.7564916 | 232.777438 |
| YIL122W | POG1 | 0.52549435 | 0.95675879 | 0.24851472 | 126.2605927 | 194.2875424 |
| YER123W | YCK3 | 0.16775782 | 0.95675879 | 0.24884866 | 124.0562679 | 190.0961064 |
| YMR274C | RCE1 | 0.22099161 | 0.95675879 | 0.24923769 | 129.0652591 | 199.4241123 |
| YNL298W | CLA4 | 0.50592867 | 0.95675879 | 0.2492378 | 82.67486843 | 112.417033 |
| YKL213C | DOA1 | 0.26475126 | 0.95675879 | 0.24942419 | 103.2355031 | 150.9474476 |
| YHR135C | YCK1 | 0.20316603 | 0.95675879 | 0.24951926 | 135.7049068 | 211.8288665 |
| YMR145C | NDE1 | 0.11483346 | 0.95675879 | 0.24953853 | 101.2129376 | 147.1344816 |
| YLR137W | YLR137W | 0.11096535 | 0.95675879 | 0.24959381 | 121.8024034 | 185.7414015 |
| YDR093W | DNF2 | 0.13382327 | 0.95675879 | 0.2497023 | 138.2228207 | 216.5199992 |
| YMR133W | REC114 | 0.03526682 | 0.8275175 | 0.24971243 | 124.8739911 | 191.4819947 |
| YJR147W | HMS2 | 0.17465541 | 0.95675879 | 0.25035856 | 119.4832078 | 181.2608136 |
| YDR378C | LSM6 | 0.60888378 | 0.95959383 | 0.25063232 | 52.86353537 | 56.26607505 |
| YMR104C | YPK2 | 0.04637971 | 0.92220427 | 0.2506475 | 128.0787158 | 197.3326004 |
| YKL030W | YKL030W | 0.2429238 | 0.95675879 | 0.25065222 | 144.2133537 | 227.5929623 |
| YGL077C | HNM1 | 0.04939613 | 0.92220427 | 0.25075407 | 141.4983621 | 222.4834585 |
| YGL153W | PEX14 | 0.27057997 | 0.95675879 | 0.25080258 | 144.3281645 | 227.7825689 |
| YMR073C | IRC21 | 0.20329743 | 0.95675879 | 0.25129172 | 112.0541339 | 167.1676258 |
| YDL154W | MSH5 | 0.26126661 | 0.95675879 | 0.25133123 | 155.8045473 | 249.2165404 |
| YMR085W | YMR085W | 0.07273934 | 0.95675879 | 0.25190418 | 132.4568824 | 205.3290201 |
| YCR062W | YCR062W | 0.08115473 | 0.95675879 | 0.2520286 | 136.9079668 | 213.6559214 |
| YNR063W | YNR063W | 0.50502663 | 0.95675879 | 0.25227108 | 144.0669782 | 227.0414518 |

| | | | | | | |
|---|---|---|---|---|---|---|
| YPR058W | YMC1 | 0.02111124 | 0.69199756 | 0.25262913 | 121.4130359 | 184.4918026 |
| YCL028W | RNQ1 | 0.21242764 | 0.95675879 | 0.25276987 | 154.4529804 | 246.4354802 |
| YHR094C | HXT1 | 0.08790173 | 0.95675879 | 0.25285099 | 128.4341629 | 197.6222538 |
| YEL037C | RAD23 | 0.03409659 | 0.82054633 | 0.25326759 | 132.8291578 | 205.7939665 |
| YMR080C | NAM7 | 0.12042375 | 0.95675879 | 0.25463814 | 125.6425092 | 192.0806224 |
| YEL043W | YEL043W | 0.1522451 | 0.95675879 | 0.25524884 | 143.8620481 | 226.1476201 |
| YNL098C | RAS2 | 0.13414264 | 0.95675879 | 0.25562206 | 127.4947739 | 195.3862776 |
| YMR052W | FAR3 | 0.1726142 | 0.95675879 | 0.25571049 | 139.9581593 | 218.7467335 |
| YJL037W | IRC18 | 0.28327438 | 0.95675879 | 0.25670313 | 139.2083225 | 217.1705494 |
| YLR398C | SKI2 | 0.1353587 | 0.95675879 | 0.25673919 | 115.7961379 | 173.2538779 |
| YMR221C | FMP42 | 0.0628795 | 0.95675879 | 0.25682607 | 130.5114916 | 200.8382564 |
| YBR005W | RCR1 | 0.17580603 | 0.95675879 | 0.25707358 | 136.3288888 | 211.7066737 |
| YIL009C-A | EST3 | 0.26224298 | 0.95675879 | 0.25750832 | 124.8874711 | 190.1734484 |
| YMR316W | DIA1 | 0.15425773 | 0.95675879 | 0.25771653 | 115.6608795 | 172.8329786 |
| YHL035C | VMR1 | 0.06167213 | 0.95675879 | 0.25772627 | 127.1630595 | 194.4041165 |
| YIR034C | LYS1 | 0.16121921 | 0.95675879 | 0.25824196 | 113.1562023 | 168.0454571 |
| YGR283C | YGR283C | 0.20360006 | 0.95675879 | 0.25878753 | 140.1725235 | 218.6223203 |
| YNL021W | HDA1 | 0.51353149 | 0.95675879 | 0.25940044 | 113.3625897 | 168.2343367 |
| YNL246W | VPS75 | 0.07627777 | 0.95675879 | 0.25964593 | 115.3303752 | 171.8829964 |
| YMR297W | PRC1 | 0.16377734 | 0.95675879 | 0.25982918 | 123.3082603 | 186.8144895 |
| YCL055W | KAR4 | 0.05860981 | 0.95675879 | 0.26010108 | 146.2077055 | 229.716808 |
| YML102W | CAC2 | 0.00534542 | 0.42413462 | 0.26058966 | 107.47771 | 156.9935362 |
| YNL332W | THI12 | 0.28483057 | 0.95675879 | 0.2609188 | 136.3711369 | 211.1280169 |
| YLR315W | NKP2 | 0.06336894 | 0.95675879 | 0.26111055 | 117.1384508 | 175.0235268 |
| YKR074W | YKR074W | 0.07921602 | 0.95675879 | 0.26124034 | 97.96022488 | 139.0317801 |
| YOR333C | YOR333C | 0.18630028 | 0.95675879 | 0.26140367 | 114.1995569 | 169.4613618 |
| YKL005C | BYE1 | 0.28066214 | 0.95675879 | 0.26169797 | 124.3728231 | 188.4913822 |
| YOR358W | HAP5 | 0.41539251 | 0.95675879 | 0.26171828 | 105.271627 | 152.6628389 |
| YER007C-A | TMA20 | 0.10610342 | 0.95675879 | 0.26178937 | 118.7222434 | 177.8778535 |
| YKL116C | PRR1 | 0.21770026 | 0.95675879 | 0.26179232 | 138.0188671 | 214.0689495 |
| YER177W | BMH1 | 0.03867663 | 0.87871674 | 0.2623097 | 114.9780516 | 170.766444 |
| YIL105C | SLM1 | 0.32246722 | 0.95675879 | 0.26273535 | 145.331887 | 227.6234674 |
| YMR130W | YMR130W | 0.07315772 | 0.95675879 | 0.26348985 | 149.5015936 | 235.3148318 |
| YER116C | SLX8 | 0.10465068 | 0.95675879 | 0.26353229 | 116.7490692 | 173.8788823 |
| YML067C | ERV41 | 0.0444235 | 0.92220427 | 0.26373236 | 128.6483876 | 196.1623055 |
| YKR099W | BAS1 | 0.1970004 | 0.95675879 | 0.26407068 | 125.7529748 | 190.6739576 |
| YNR058W | BIO3 | 0.01302665 | 0.59816436 | 0.26486992 | 134.2351129 | 206.4458052 |
| YIL159W | BNR1 | 0.25265366 | 0.95675879 | 0.26547743 | 141.3165365 | 219.6233624 |
| YMR020W | FMS1 | 0.0412211 | 0.91069949 | 0.26556139 | 121.5567995 | 182.5488087 |
| YML058W | SML1 | 0.14437218 | 0.95675879 | 0.26619102 | 130.0426669 | 198.3566716 |
| YML013W | SEL1 | 0.08677961 | 0.95675879 | 0.26632668 | 110.5341279 | 161.7444052 |
| YKL106W | AAT1 | 0.08177457 | 0.95675879 | 0.26638378 | 124.3838665 | 187.710381 |
| YNL115C | YNL115C | 0.02856141 | 0.77288087 | 0.26639108 | 123.610244 | 186.2581729 |
| YGR078C | PAC10 | 0.06067046 | 0.95675879 | 0.26665052 | 115.4892248 | 170.9824831 |
| YBR113W | YBR113W | 0.41811507 | 0.95675879 | 0.26715899 | 139.4679039 | 215.8684722 |
| YCR019W | MAK32 | 0.0975246 | 0.95675879 | 0.26720805 | 152.6639004 | 240.6097051 |
| YGR174C | CBP4 | 0.10061093 | 0.95675879 | 0.26761701 | 131.1751316 | 200.2366765 |
| YNL301C | RPL18B | 0.6839625 | 0.96992497 | 0.26776917 | 119.7509405 | 178.7841089 |
| YMR219W | ESC1 | 0.04351345 | 0.92220427 | 0.26809898 | 120.4783549 | 180.0919768 |
| YGR208W | SER2 | 0.08230151 | 0.95675879 | 0.26812267 | 119.6501651 | 178.534619 |
| YBL082C | ALG3 | 0.06762058 | 0.95675879 | 0.26813842 | 135.2130075 | 207.7206667 |
| YGL181W | GTS1 | 0.21408587 | 0.95675879 | 0.26831525 | 140.1703836 | 216.9881727 |
| YML037C | YML037C | 0.01164741 | 0.59540465 | 0.26872949 | 127.3430781 | 192.8591674 |
| YAL048C | GEM1 | 0.5503738 | 0.95947263 | 0.26883145 | 51.78079936 | 51.12160145 |
| YLR444C | YLR444C | 0.17596317 | 0.95675879 | 0.26887164 | 116.2193298 | 171.9718043 |
| YJR050W | ISY1 | 0.11776464 | 0.95675879 | 0.26901205 | 128.0000719 | 194.0430417 |
| YKR024C | DBP7 | 0.48281536 | 0.95675879 | 0.26910549 | 30.63772386 | 11.42001918 |
| YJR131W | MNS1 | 0.28599883 | 0.95675879 | 0.26938267 | 131.3885926 | 200.3349382 |
| YCL040W | GLK1 | 0.11684824 | 0.95675879 | 0.2698184 | 140.4901383 | 217.3307061 |
| YKL131W | YKL131W | 0.39096107 | 0.95675879 | 0.27000661 | 132.271348 | 201.8838297 |
| YML096W | YML096W | 0.00308685 | 0.33137349 | 0.27043792 | 126.2265337 | 190.4727404 |
| YMR195W | ICY1 | 0.36092589 | 0.95675879 | 0.27080427 | 130.5822875 | 198.5794539 |
| YJR075W | HOC1 | 0.28147876 | 0.95675879 | 0.27132624 | 118.9897771 | 176.7479231 |
| YGL086W | MAD1 | 0.23031321 | 0.95675879 | 0.27150899 | 147.6562258 | 230.4817445 |

| | | | | | | |
|---|---|---|---|---|---|---|
| YLR039C | RIC1 | 0.06952229 | 0.95675879 | 0.27169236 | 108.0580523 | 156.1823901 |
| YKL201C | MNN4 | 0.24673846 | 0.95675879 | 0.27203238 | 123.9155313 | 185.8655591 |
| YDL233W | YDL233W | 0.15924288 | 0.95675879 | 0.27292758 | 139.0717051 | 214.1384158 |
| YJL134W | LCB3 | 0.20361076 | 0.95675879 | 0.27309418 | 135.082427 | 206.627858 |
| YMR036C | MIH1 | 0.19778747 | 0.95675879 | 0.27322174 | 135.487809 | 207.3663439 |
| YFR018C | YFR018C | 0.21679931 | 0.95675879 | 0.27352686 | 142.0170456 | 219.5599882 |
| YGR042W | YGR042W | 0.09081112 | 0.95675879 | 0.2735367 | 148.6373689 | 231.9749898 |
| YMR283C | RIT1 | 0.24988575 | 0.95675879 | 0.27370196 | 135.0443787 | 206.452509 |
| YDL121C | YDL121C | 0.16981283 | 0.95675879 | 0.27395983 | 156.3991578 | 246.4601441 |
| YAL023C | PMT2 | 0.19340349 | 0.95675879 | 0.27426372 | 134.9044406 | 206.0939367 |
| YDR042C | YDR042C | 0.16168131 | 0.95675879 | 0.2745919 | 125.2033298 | 187.8429594 |
| YLR287C-A | RPS30A | 0.20637956 | 0.95675879 | 0.27551942 | 111.158837 | 161.3432509 |
| YNL249C | MPA43 | 0.27857521 | 0.95675879 | 0.27610768 | 124.6819395 | 186.6057306 |
| YDL078C | MDH3 | 0.1228222 | 0.95675879 | 0.2767535 | 139.6336191 | 214.5377173 |
| YDR061W | YDR061W | 0.28939456 | 0.95675879 | 0.27748514 | 135.7020697 | 207.0387571 |
| YML116W | ATR1 | 0.0139386 | 0.59852326 | 0.27769116 | 121.367661 | 180.118743 |
| YNL023C | FAP1 | 0.67406547 | 0.96992497 | 0.27771769 | 120.6782588 | 178.8212024 |
| YHR153C | SPO16 | 0.12648839 | 0.95675879 | 0.27955708 | 139.0332942 | 212.9321085 |
| YJR146W | YJR146W | 0.12078811 | 0.95675879 | 0.27970739 | 132.2932976 | 200.2652545 |
| YBR141C | YBR141C | 0.18092015 | 0.95675879 | 0.280166 | 133.972415 | 203.3360416 |
| YKR048C | NAP1 | 0.12968821 | 0.95675879 | 0.28024593 | 118.891178 | 175.0368938 |
| YMR222C | FSH2 | 0.02101534 | 0.69199756 | 0.28032138 | 138.9535184 | 212.6517176 |
| YKL027W | YKL027W | 0.02861855 | 0.77288087 | 0.28035807 | 111.9139864 | 161.9317009 |
| YMR230W | RPS10B | 0.03426543 | 0.82054633 | 0.28040069 | 104.1839062 | 147.4263298 |
| YGR206W | MVB12 | 0.11073771 | 0.95675879 | 0.28076715 | 125.5140911 | 187.3692592 |
| YIL096C | YIL096C | 0.30537626 | 0.95675879 | 0.28145918 | 145.8524164 | 225.3962115 |
| YGR242W | YGR242W | 0.14115177 | 0.95675879 | 0.28224934 | 143.0358867 | 219.9785032 |
| YOR068C | VAM10 | 0.35662152 | 0.95675879 | 0.2825613 | 113.9611166 | 165.3942188 |
| YNL122C | YNL122C | 0.48750901 | 0.95675879 | 0.28338853 | 119.2855859 | 175.2389431 |
| YMR243C | ZRC1 | 0.04220522 | 0.91069949 | 0.28447806 | 122.7238041 | 181.5010486 |
| YML068W | ITT1 | 0.04560421 | 0.92220427 | 0.2844833 | 120.8854651 | 178.0522728 |
| YMR129W | POM152 | 0.06269909 | 0.95675879 | 0.28449892 | 128.3566341 | 192.0620816 |
| YGR279C | SCW4 | 0.14889483 | 0.95675879 | 0.28457189 | 141.3691821 | 216.4551583 |
| YHR079C-B | YHR079C-B | 0.07847382 | 0.95675879 | 0.28509152 | 138.755326 | 211.4638587 |
| YLR184W | YLR184W | 0.06438325 | 0.95675879 | 0.2852659 | 103.1430964 | 144.6418415 |
| YIL160C | POT1 | 0.18230066 | 0.95675879 | 0.28547965 | 122.701418 | 181.2876964 |
| YGR132C | PHB1 | 0.0574223 | 0.95675879 | 0.28563186 | 140.9605444 | 215.5073868 |
| YGR124W | ASN2 | 0.23035085 | 0.95675879 | 0.28591824 | 146.284451 | 225.4435928 |
| YMR252C | YMR252C | 0.32874554 | 0.95675879 | 0.28613595 | 134.3008747 | 202.93066 |
| YDR290W | :::RTT103 | 0.23323259 | 0.95675879 | 0.28626514 | 122.5102445 | 180.7947499 |
| YGR166W | KRE11 | 0.09694244 | 0.95675879 | 0.28626702 | 131.7625915 | 198.1475819 |
| YFR021W | ATG18 | 0.1653132 | 0.95675879 | 0.28680519 | 128.2104251 | 191.393271 |
| YML035C-A | YML035C-A | 0.06889014 | 0.95675879 | 0.28691814 | 117.0195295 | 170.3849666 |
| YHR162W | YHR162W | 0.06772931 | 0.95675879 | 0.28704616 | 119.6964616 | 175.3837589 |
| YNL001W | DOM34 | 0.06160058 | 0.95675879 | 0.28710696 | 104.4500378 | 146.7780708 |
| YLR433C | CNA1 | 0.06429797 | 0.95675879 | 0.28720292 | 127.8744132 | 190.6950186 |
| YPL178W | CBC2 | 0.46823142 | 0.95675879 | 0.28743183 | 59.99607383 | 63.3472727 |
| YCR017C | CWH43 | 0.13219596 | 0.95675879 | 0.28785652 | 153.8550849 | 239.3109959 |
| YCR090C | YCR090C | 0.03429543 | 0.82054633 | 0.28810588 | 149.5490108 | 231.1921149 |
| YEL063C | CAN1 | 0.17906171 | 0.95675879 | 0.28816247 | 125.444542 | 185.9735237 |
| YKR082W | NUP133 | 0.08982559 | 0.95675879 | 0.2891658 | 94.68899525 | 128.1185831 |
| YHR018C | ARG4 | 0.19870018 | 0.95675879 | 0.28960437 | 130.8812873 | 195.9236597 |
| YDL018C | ERP3 | 0.04648423 | 0.92220427 | 0.28998605 | 141.3739912 | 215.5378478 |
| YDR007W | TRP1 | 0.24700022 | 0.95675879 | 0.29021018 | 129.6699309 | 193.5480605 |
| YHR139C-A | YHR139C-A | 0.26636114 | 0.95675879 | 0.29042194 | 146.8531786 | 225.7397094 |
| YCL030C | HIS4 | 0.0491021 | 0.92220427 | 0.29048799 | 142.0643914 | 216.7468436 |
| YGR035C | YGR035C | 0.53786877 | 0.95675879 | 0.29084118 | 136.1203625 | 205.5381477 |
| YML051W | GAL80 | 0.07427948 | 0.95675879 | 0.29110728 | 129.2305012 | 192.5704054 |
| YNL199C | GCR2 | 0.14463038 | 0.95675879 | 0.29230102 | 127.7920779 | 189.6683416 |
| YIL156W | UBP7 | 0.15758832 | 0.95675879 | 0.29247565 | 138.4901551 | 209.7031418 |
| YOR080W | DIA2 | 0.29766943 | 0.95675879 | 0.29257434 | 37.20800862 | 19.72748151 |
| YLR415C | YLR415C | 0.05675569 | 0.95675879 | 0.2926171 | 138.8490453 | 210.3520531 |
| YAL058W | CNE1 | 0.32742547 | 0.95675879 | 0.29302549 | 131.3981543 | 196.3077327 |
| YLR437C | YLR437C | 0.09246958 | 0.95675879 | 0.29320622 | 158.1172172 | 246.3894965 |

| | | | | | | |
|---|---|---|---|---|---|---|
| YDL099W | BUG1 | 0.34669447 | 0.95675879 | 0.293359 | 128.4089277 | 190.6442559 |
| YJL065C | DLS1 | 0.2971887 | 0.95675879 | 0.29344902 | 152.2088175 | 235.2665114 |
| YPR194C | OPT2 | 0.18656257 | 0.95675879 | 0.29346348 | 106.7283837 | 149.9636398 |
| YDR447C | RPS17B | 0.01540504 | 0.61680755 | 0.29352019 | 115.9511197 | 167.2515526 |
| YKL063C | YKL063C | 0.1579771 | 0.95675879 | 0.29355841 | 127.3225063 | 188.5725104 |
| YCR043C | YCR043C | 0.09241887 | 0.95675879 | 0.29410981 | 148.9486661 | 229.0389095 |
| YCR033W | SNT1 | 0.49257694 | 0.95675879 | 0.29449836 | 110.0405411 | 155.9986625 |
| YNL193W | YNL193W | 0.01480636 | 0.61680755 | 0.29471828 | 121.7786977 | 177.9764257 |
| YPL205C | YPL205C | 0.38458123 | 0.95675879 | 0.29505254 | 60.93876956 | 63.8114811 |
| YDR241W | BUD26 | 0.12126157 | 0.95675879 | 0.29527816 | 104.5853982 | 145.6339025 |
| YKL074C | MUD2 | 0.05718903 | 0.95675879 | 0.29658871 | 114.9847351 | 164.9140541 |
| YMR141C | YMR141C | 0.17905005 | 0.95675879 | 0.29689817 | 126.3491543 | 186.1755373 |
| YBL087C | RPL23A | 0.00112105 | 0.22922861 | 0.29693237 | 115.0281841 | 164.9367457 |
| YGL174W | BUD13 | 0.31535706 | 0.95675879 | 0.29726236 | 89.04948739 | 116.1561877 |
| YLR188W | MDL1 | 0.06955129 | 0.95675879 | 0.29737167 | 120.6873011 | 175.4754878 |
| YML128C | MSC1 | 0.05285301 | 0.94562837 | 0.29740013 | 112.8296729 | 160.7333187 |
| YHR140W | YHR140W | 0.21232986 | 0.95675879 | 0.29781334 | 151.7798042 | 233.715173 |
| YJR032W | CPR7 | 0.26075948 | 0.95675879 | 0.29793458 | 87.11755105 | 112.4177486 |
| YNR018W | YNR018W | 0.1304227 | 0.95675879 | 0.29826705 | 126.4535428 | 186.1371151 |
| YNL293W | MSB3 | 0.10720581 | 0.95675879 | 0.29887912 | 135.402899 | 202.8172738 |
| YLR013W | GAT3 | 0.19166983 | 0.95675879 | 0.29904442 | 141.5177634 | 214.2576682 |
| YHR152W | SPO12 | 0.09076515 | 0.95675879 | 0.29954344 | 138.9104381 | 209.2821449 |
| YMR180C | CTL1 | 0.02857152 | 0.77288087 | 0.29982596 | 128.282791 | 189.3012235 |
| YBR159W | IFA38 | 0.18108792 | 0.95675879 | 0.30124982 | 138.4909615 | 208.2034484 |
| YJL170C | ASG7 | 0.23601059 | 0.95675879 | 0.30137643 | 145.8022471 | 221.8943992 |
| YDL051W | LHP1 | 0.099539 | 0.95675879 | 0.30157274 | 146.11471 | 222.446849 |
| YLR238W | FAR10 | 0.03096467 | 0.78675907 | 0.30239447 | 132.5637622 | 196.8909034 |
| YGL168W | HUR1 | 0.41514849 | 0.95675879 | 0.30253348 | 85.54874533 | 108.6885481 |
| YHR017W | YSC83 | 0.20537967 | 0.95675879 | 0.30306952 | 149.8223206 | 229.1445323 |
| YEL042W | GDA1 | 0.02782684 | 0.76802374 | 0.30311189 | 118.8520333 | 171.0512525 |
| YLR111W | YLR111W | 0.0061458 | 0.4472889 | 0.30321298 | 108.1407876 | 150.9445803 |
| YPR057W | BRR1 | 0.14209711 | 0.95675879 | 0.30330305 | 105.4850882 | 145.948299 |
| YGR249W | MGA1 | 0.35396251 | 0.95675879 | 0.30367604 | 138.4520731 | 207.7153998 |
| YBR168W | PEX32 | 0.13288795 | 0.95675879 | 0.30551047 | 107.3145851 | 149.0019188 |
| YNL020C | ARK1 | 0.30317308 | 0.95675879 | 0.30559798 | 93.74734004 | 123.5410282 |
| YAL034C | FUN19 | 0.15133785 | 0.95675879 | 0.30582474 | 142.394773 | 214.7424641 |
| YHR203C | RPS4B | 0.14056856 | 0.95675879 | 0.30598502 | 105.8016593 | 146.0831719 |
| YCL009C | ILV6 | 0.02489006 | 0.7579993 | 0.30609098 | 143.9145746 | 217.5473632 |
| YFR031C-A | RPL2A | 0.10241036 | 0.95675879 | 0.30726671 | 114.0577272 | 161.3484734 |
| YBR148W | YSW1 | 0.1931025 | 0.95675879 | 0.30738109 | 138.9241189 | 207.9668304 |
| YJL155C | FBP26 | 0.25749187 | 0.95675879 | 0.30818745 | 154.3108105 | 236.6872322 |
| YMR115W | FMP24 | 0.02209772 | 0.71344081 | 0.30879555 | 114.6421629 | 162.1830319 |
| YLR423C | ATG17 | 0.14775112 | 0.95675879 | 0.30899846 | 142.350855 | 214.1170908 |
| YOR189W | IES4 | 0.13499 | 0.95675879 | 0.30999988 | 148.4522075 | 225.3890884 |
| YCR073W-A | SOL2 | 0.04021865 | 0.89947345 | 0.31013472 | 153.8853792 | 235.5561521 |
| YMR210W | YMR210W | 0.02520316 | 0.76212928 | 0.31050652 | 134.5543967 | 199.2364968 |
| YKL168C | KKQ8 | 0.04653958 | 0.92220427 | 0.31059234 | 134.1167695 | 198.4010269 |
| YFL033C | RIM15 | 0.12822902 | 0.95675879 | 0.31061586 | 118.9327109 | 169.9186838 |
| YMR012W | CLU1 | 0.08286847 | 0.95675879 | 0.31087104 | 140.2107941 | 209.7829326 |
| YMR065W | KAR5 | 0.12843498 | 0.95675879 | 0.31165068 | 131.2026796 | 192.7544571 |
| YNL295W | YNL295W | 0.08498317 | 0.95675879 | 0.3121399 | 130.5866073 | 191.5152854 |
| YDL050C | YDL050C | 0.08933233 | 0.95675879 | 0.31265081 | 154.0562506 | 235.4461402 |
| YKL110C | KTI12 | 0.06835484 | 0.95675879 | 0.31366003 | 117.447537 | 166.6123411 |
| YMR196W | YMR196W | 0.21747445 | 0.95675879 | 0.31369553 | 134.2913637 | 198.1975484 |
| YHR178W | STB5 | 0.23467621 | 0.95675879 | 0.31526533 | 94.0654694 | 122.4836684 |
| YDL085W | NDE2 | 0.12124513 | 0.95675879 | 0.31635264 | 159.6357731 | 245.2774018 |
| YGL147C | RPL9A | 0.02771561 | 0.76802374 | 0.31635667 | 120.3537258 | 171.6016384 |
| YIL012W | YIL012W | 0.11885264 | 0.95675879 | 0.31650661 | 136.6664677 | 202.1711946 |
| YOR089C | VPS21 | 0.00931894 | 0.54423319 | 0.31662599 | 127.2876008 | 184.560324 |
| YLR435W | TSR2 | 0.02964401 | 0.78255549 | 0.3167038 | 118.7901588 | 168.609713 |
| YHR132C | ECM14 | 0.29588235 | 0.95675879 | 0.31754078 | 140.8085308 | 209.7628608 |
| YCR049C | YCR049C | 0.22843449 | 0.95675879 | 0.31787667 | 155.4827297 | 237.227448 |
| YDR316W | OMS1 | 0.10591988 | 0.95675879 | 0.31855999 | 130.5660728 | 190.378335 |
| YNL219C | ALG9 | 0.00022568 | 0.10310418 | 0.31889078 | 115.6929548 | 162.4266039 |

| | | | | | | |
|---|---|---|---|---|---|---|
| YBR046C | ZTA1 | 0.04779145 | 0.92220427 | 0.32025765 | 143.6841456 | 214.691355 |
| YDR431W | YDR431W | 0.13443284 | 0.95675879 | 0.32047532 | 151.0779053 | 228.5214082 |
| YNR009W | NRM1 | 0.18975508 | 0.95675879 | 0.3205721 | 126.9879414 | 183.3231451 |
| YDL025C | YDL025C | 0.023456 | 0.74058858 | 0.32126645 | 136.2350203 | 200.5476179 |
| YPR191W | QCR2 | 0.05200917 | 0.93531943 | 0.32192627 | 97.6432 | 128.0542017 |
| YGR227W | DIE2 | 0.07813534 | 0.95675879 | 0.32266392 | 138.747231 | 205.0202729 |
| YLR372W | SUR4 | 0.48781628 | 0.95675879 | 0.32329946 | 105.538587 | 142.6273764 |
| YDR100W | TVP15 | 0.1922827 | 0.95675879 | 0.32364586 | 143.7343963 | 214.2059001 |
| YCL001W | RER1 | 0.0172194 | 0.63741456 | 0.32391608 | 139.6764215 | 206.5487699 |
| YMR006C | PLB2 | 0.04313614 | 0.92220427 | 0.32452195 | 132.3085527 | 192.6263732 |
| YMR223W | UBP8 | 0.02605645 | 0.76284641 | 0.32513056 | 117.4331601 | 164.6228412 |
| YFL013C | IES1 | 0.43673896 | 0.95675879 | 0.32711472 | 33.26714295 | 6.426583646 |
| YKL032C | IXR1 | 0.20964063 | 0.95675879 | 0.32726666 | 124.7950659 | 178.0649209 |
| YMR194W | RPL36A | 0.02790212 | 0.76802374 | 0.32731691 | 112.1694638 | 154.3764944 |
| YMR023C | MSS1 | 0.04909909 | 0.92220427 | 0.32742821 | 137.61987 | 202.0907218 |
| YIL013C | PDR11 | 0.3801307 | 0.95675879 | 0.3275512 | 145.3150069 | 216.5022207 |
| YLR048W | RPS0B | 0.24177928 | 0.95675879 | 0.32759948 | 57.99660361 | 52.72475026 |
| YJR059W | PTK2 | 0.05545358 | 0.95675879 | 0.32797394 | 132.6770426 | 192.7268766 |
| YGR173W | RBG2 | 0.15046807 | 0.95675879 | 0.32823605 | 138.2360556 | 203.1081851 |
| YNR010W | CSE2 | 0.21690168 | 0.95675879 | 0.32846413 | 32.53740507 | 4.827055142 |
| YMR305C | SCW10 | 0.03439645 | 0.82054633 | 0.32851639 | 129.0042564 | 185.745608 |
| YKR072C | SIS2 | 0.02915995 | 0.78255549 | 0.32926348 | 127.571999 | 182.9315284 |
| YKL029C | MAE1 | 0.20764896 | 0.95675879 | 0.32971992 | 135.3820002 | 197.5014086 |
| YIR023W | DAL81 | 0.49601773 | 0.95675879 | 0.32988125 | 133.7672099 | 194.4452009 |
| YCL006C | YCL006C | 0.38359391 | 0.95675879 | 0.33002903 | 151.3495172 | 227.3962471 |
| YML011C | RAD33 | 0.01355414 | 0.59822632 | 0.33033933 | 114.1519739 | 157.5776557 |
| YDL042C | SIR2 | 0.00273167 | 0.31772128 | 0.33116475 | 142.5693134 | 210.7343044 |
| YKR018C | YKR018C | 0.23936108 | 0.95675879 | 0.33117198 | 127.3974498 | 182.2776222 |
| YPL172C | COX10 | 0.13513406 | 0.95675879 | 0.33128635 | 106.3193405 | 142.7252038 |
| YDL133W | YDL133W | 0.05656453 | 0.95675879 | 0.33156804 | 160.5889818 | 244.4619234 |
| YOL013C | HRD1 | 0.11311314 | 0.95675879 | 0.33158713 | 149.044904 | 222.8072712 |
| YGL017W | ATE1 | 0.03002017 | 0.78255549 | 0.33180129 | 138.2516932 | 202.5275243 |
| YCR077C | PAT1 | 0.09575112 | 0.95675879 | 0.33237062 | 100.1336703 | 130.9382167 |
| YCL025C | AGP1 | 0.13186825 | 0.95675879 | 0.33243918 | 160.073675 | 243.3463974 |
| YGR193C | PDX1 | 0.17740403 | 0.95675879 | 0.33275174 | 145.498193 | 215.956012 |
| YML026C | RPS18B | 0.09274025 | 0.95675879 | 0.33281133 | 111.5233333 | 152.2245887 |
| YKL206C | ADD66 | 0.01330864 | 0.59816436 | 0.33399179 | 125.6020756 | 178.4278725 |
| YMR031C | YMR031C | 0.01217967 | 0.59816436 | 0.33434051 | 133.4941848 | 193.1701791 |
| YCL023C | YCL023C | 0.38455188 | 0.95675879 | 0.33450454 | 158.1486503 | 239.3825662 |
| YBL078C | ATG8 | 0.06120474 | 0.95675879 | 0.33496155 | 138.0003777 | 201.5154717 |
| YNL109W | YNL109W | 0.08758144 | 0.95675879 | 0.33501812 | 118.2751721 | 164.5103685 |
| YDL096C | YDL096C | 0.00972875 | 0.54423319 | 0.33512007 | 145.6787661 | 215.8894779 |
| YBR275C | RIF1 | 0.05795611 | 0.95675879 | 0.33621386 | 126.5025524 | 179.7365713 |
| YLR375W | STP3 | 0.10484372 | 0.95675879 | 0.33622007 | 140.6690678 | 206.3053828 |
| YOR078W | BUD21 | 0.13722944 | 0.95675879 | 0.33646448 | 83.15333 | 98.39046505 |
| YDL172C | YDL172C | 0.03130084 | 0.79062247 | 0.33680717 | 147.5841662 | 219.1744806 |
| YIL027C | KRE27 | 0.04903645 | 0.92220427 | 0.33691194 | 147.6310596 | 219.2445068 |
| YCR079W | PTC6 | 0.14501067 | 0.95675879 | 0.33734718 | 170.5721229 | 262.1969351 |
| YGR129W | SYF2 | 0.11225088 | 0.95675879 | 0.33761213 | 143.3961902 | 211.1820395 |
| YJR082C | EAF6 | 0.24757027 | 0.95675879 | 0.33767624 | 126.5682211 | 179.6095318 |
| YDL010W | YDL010W | 0.20182981 | 0.95675879 | 0.33779988 | 160.150151 | 242.5726497 |
| YER074W | RPS24A | 0.00458468 | 0.39655204 | 0.33784196 | 111.9242545 | 152.1158235 |
| YDR392W | SPT3 | 0.09844655 | 0.95675879 | 0.33990463 | 114.7544691 | 157.071097 |
| YKL085W | MDH1 | 0.34882739 | 0.95675879 | 0.3417214 | 124.9175621 | 175.8215506 |
| YGL131C | SNT2 | 0.29990279 | 0.95675879 | 0.34172709 | 151.8790491 | 226.3879415 |
| YDR500C | RPL37B | 0.09316673 | 0.95675879 | 0.34223809 | 116.5226276 | 159.9881082 |
| YMR010W | YMR010W | 0.05649902 | 0.95675879 | 0.34301129 | 129.6485839 | 184.474083 |
| YBR195C | MSI1 | 0.05813924 | 0.95675879 | 0.34520135 | 136.5198865 | 196.9867838 |
| YHR059W | FYV4 | 0.44536778 | 0.95675879 | 0.34665375 | 119.7765577 | 165.3354945 |
| YFR034C | PHO4 | 0.09055991 | 0.95675879 | 0.34741885 | 146.4290809 | 215.1924799 |
| YLL009C | COX17 | 0.42950419 | 0.95675879 | 0.34750763 | 95.11961553 | 118.9443059 |
| YNL157W | IGO1 | 0.05562374 | 0.95675879 | 0.34796553 | 140.9917251 | 204.9009638 |
| YKL087C | CYT2 | 0.00288569 | 0.31772128 | 0.34904156 | 121.0535327 | 167.321974 |
| YMR226C | TMA29 | 0.07527256 | 0.95675879 | 0.35036089 | 124.2048834 | 173.0067302 |

| | | | | | | |
|---|---|---|---|---|---|---|
| YOR001W | RRP6 | 0.08979406 | 0.95675879 | 0.35121738 | 125.5783784 | 175.4362353 |
| YMR067C | UBX4 | 0.03400624 | 0.82054633 | 0.3512496 | 117.0247916 | 159.3881245 |
| YNL051W | COG5 | 0.03170941 | 0.79314877 | 0.35128933 | 123.9786429 | 172.4235573 |
| YLL016W | SDC25 | 0.09706227 | 0.95675879 | 0.35152597 | 145.1180883 | 212.0309574 |
| YGL078C | DBP3 | 0.02316992 | 0.73697491 | 0.35285539 | 122.6955705 | 169.7491591 |
| YAL020C | ATS1 | 0.07029555 | 0.95675879 | 0.3530512 | 130.3222476 | 184.0197995 |
| YLR460C | YLR460C | 0.4055191 | 0.95675879 | 0.35576526 | 129.3648307 | 181.7597675 |
| YHR049W | FSH1 | 0.2194792 | 0.95675879 | 0.35587617 | 157.0411911 | 233.6489279 |
| YDL159W | STE7 | 0.06918308 | 0.95675879 | 0.35613344 | 163.212044 | 245.1785966 |
| YML117W | NAB6 | 0.0122559 | 0.59816436 | 0.35725339 | 130.9838704 | 184.5417327 |
| YGR196C | FYV8 | 0.0534494 | 0.95007794 | 0.35854929 | 142.7908457 | 206.4644731 |
| YMR148W | YMR148W | 0.06453167 | 0.95675879 | 0.35904193 | 128.129589 | 178.8824026 |
| YDL149W | ATG9 | 0.10793456 | 0.95675879 | 0.35998379 | 139.6253494 | 200.2820218 |
| YML108W | YML108W | 0.1252875 | 0.95675879 | 0.36165382 | 143.4754707 | 207.2173486 |
| YNL215W | IES2 | 0.02773877 | 0.76802374 | 0.36169685 | 119.3062982 | 161.8797223 |
| YJR011C | YJR011C | 0.10327852 | 0.95675879 | 0.36176449 | 130.8174808 | 183.4578395 |
| YML129C | COX14 | 0.04737772 | 0.92220427 | 0.3621093 | 110.1378361 | 144.6133327 |
| YNL081C | SWS2 | 0.03705419 | 0.8554337 | 0.36309505 | 134.4003149 | 189.9499407 |
| YLR428C | YLR428C | 0.17593091 | 0.95675879 | 0.36338889 | 125.7982018 | 173.7660541 |
| YGR135W | PRE9 | 0.06193261 | 0.95675879 | 0.36391381 | 119.3659344 | 161.6122655 |
| YDL094C | YDL094C | 0.03809974 | 0.87021425 | 0.36563713 | 159.5031112 | 236.5963192 |
| YMR039C | SUB1 | 0.22327053 | 0.95675879 | 0.36624218 | 127.3198271 | 176.1317431 |
| YOR348C | PUT4 | 0.52097393 | 0.95675879 | 0.36682635 | 141.0799112 | 201.8393916 |
| YMR192W | GYL1 | 0.02016242 | 0.67882086 | 0.36850821 | 154.2404681 | 226.2347948 |
| YGL127C | SOH1 | 0.43158866 | 0.95675879 | 0.36880582 | 119.7827922 | 161.5571064 |
| YDL088C | ASM4 | 0.09591242 | 0.95675879 | 0.36956681 | 143.8588548 | 206.5825379 |
| YGR012W | YGR012W | 0.01596886 | 0.6196335 | 0.37048207 | 139.3901572 | 198.0447199 |
| YHL025W | SNF6 | 0.34652954 | 0.95675879 | 0.37110731 | 90.24086967 | 105.7562619 |
| YJL148W | RPA34 | 0.04994904 | 0.92448791 | 0.37123964 | 114.5639511 | 151.3525475 |
| YGL213C | SKI8 | 0.07806706 | 0.95675879 | 0.37144195 | 140.150393 | 199.306343 |
| YMR040W | YET2 | 0.00369295 | 0.34020275 | 0.37173427 | 145.3906699 | 209.0846813 |
| YBR235W | YBR235W | 0.02658642 | 0.76618851 | 0.37256609 | 155.9075965 | 228.6672836 |
| YNL090W | RHO2 | 0.07382425 | 0.95675879 | 0.37297491 | 134.3672289 | 188.1975036 |
| YBR239C | YBR239C | 0.00135501 | 0.25067939 | 0.3735553 | 164.6737235 | 244.9392636 |
| YML102C-A | YML102C-A | 0.00480388 | 0.39992349 | 0.37387473 | 142.3146328 | 202.949228 |
| YDL056W | MBP1 | 0.06498049 | 0.95675879 | 0.37504956 | 141.7918598 | 201.7677402 |
| YML122C | YML122C | 0.01834031 | 0.64739045 | 0.37572138 | 140.5192093 | 199.2658893 |
| YAL059W | ECM1 | 0.51084562 | 0.95675879 | 0.37639531 | 133.9778779 | 186.8820509 |
| YDR133C | YDR133C | 0.23684642 | 0.95675879 | 0.37655512 | 154.5298537 | 225.4007734 |
| YNL270C | ALP1 | 0.00656459 | 0.46980564 | 0.37717055 | 142.0347179 | 201.8603422 |
| YJL131C | YJL131C | 0.1607545 | 0.95675879 | 0.37739061 | 143.7290442 | 205.0004698 |
| YNL316C | PHA2 | 0.23905896 | 0.95675879 | 0.37767588 | 139.5383535 | 197.0918514 |
| YCL002C | YCL002C | 0.01332806 | 0.59816436 | 0.37801375 | 134.8083399 | 188.1627118 |
| YCL016C | DCC1 | 0.26223193 | 0.95675879 | 0.38119264 | 117.591365 | 155.3276877 |
| YKR035W-A | DID2 | 0.01335403 | 0.59816436 | 0.38157299 | 121.9581988 | 163.4527864 |
| YGR214W | RPS0A | 0.00945739 | 0.54423319 | 0.38286229 | 117.153317 | 154.2204436 |
| YIL057C | YIL057C | 0.22143823 | 0.95675879 | 0.38327205 | 161.7899566 | 237.8681664 |
| YIL076W | SEC28 | 0.05084888 | 0.92519106 | 0.38353332 | 124.9865923 | 168.797261 |
| YER083C | GET2 | 0.01389942 | 0.59852326 | 0.38404604 | 117.7818926 | 155.1968315 |
| YGL014W | PUF4 | 0.00833783 | 0.52650955 | 0.38614064 | 148.0109117 | 211.5342095 |
| YML097C | VPS9 | 0.36702917 | 0.95675879 | 0.38626047 | 104.5804431 | 130.0581018 |
| YPR134W | MSS18 | 0.01594462 | 0.6196335 | 0.38759667 | 119.1415492 | 157.1394308 |
| YHR189W | PTH1 | 0.50957661 | 0.95675879 | 0.38770476 | 127.6393092 | 173.0588322 |
| YNL211C | YNL211C | 0.2757152 | 0.95675879 | 0.38803089 | 124.3550967 | 166.8433598 |
| YDR158W | HOM2 | 0.13394361 | 0.95675879 | 0.38851487 | 123.700454 | 165.5327442 |
| YKR001C | VPS1 | 0.10687418 | 0.95675879 | 0.38873229 | 115.8349702 | 150.7435114 |
| YLR038C | COX12 | 0.04569659 | 0.92220427 | 0.38920739 | 103.3415542 | 127.2303152 |
| YLL028W | TPO1 | 0.15465672 | 0.95675879 | 0.38964363 | 129.3008622 | 175.8434134 |
| YGR187C | HGH1 | 0.11173569 | 0.95675879 | 0.39035655 | 143.2885352 | 201.9558847 |
| YGL214W | YGL214W | 0.0164525 | 0.61971068 | 0.39056783 | 139.2300087 | 194.3078049 |
| YDL082W | RPL13A | 0.00280774 | 0.31772128 | 0.39334238 | 116.2267683 | 150.6895871 |
| YDR345C | HXT3 | 0.16027757 | 0.95675879 | 0.39495516 | 147.9121075 | 209.8407899 |
| YJR134C | SGM1 | 0.06488272 | 0.95675879 | 0.39687316 | 145.2732507 | 204.5633483 |
| YMR035W | IMP2 | 0.01159812 | 0.59540465 | 0.39726593 | 139.3440786 | 193.3757445 |

| | | | | | | |
|---|---|---|---|---|---|---|
| YNL100W | YNL100W | 0.41645061 | 0.95675879 | 0.39821084 | 137.1820697 | 189.1591414 |
| YBR059C | AKL1 | 0.14505006 | 0.95675879 | 0.39943747 | 149.1134685 | 211.3270956 |
| YJR074W | MOG1 | 0.12966672 | 0.95675879 | 0.39952592 | 116.2906429 | 149.7514213 |
| YGR183C | QCR9 | 0.02039308 | 0.67882086 | 0.39990247 | 124.1721427 | 164.4690688 |
| YIR033W | MGA2 | 0.16233975 | 0.95675879 | 0.40008914 | 134.3508931 | 183.5277887 |
| YBL024W | NCL1 | 0.09314016 | 0.95675879 | 0.40100363 | 145.7057061 | 204.6677382 |
| YHR200W | RPN10 | 0.04215423 | 0.91069949 | 0.40151051 | 123.2234393 | 162.414611 |
| YBL059W | YBL059W | 0.0411312 | 0.91069949 | 0.40319183 | 143.3190046 | 199.8169945 |
| YML063W | RPS1B | 0.1361466 | 0.95675879 | 0.40489125 | 109.52132 | 136.1373057 |
| YLL023C | YLL023C | 0.09910236 | 0.95675879 | 0.40541001 | 144.1949192 | 201.0802923 |
| YBR242W | YBR242W | 0.01551356 | 0.61680755 | 0.4054716 | 174.530466 | 257.9653025 |
| YDL137W | ARF2 | 0.14665293 | 0.95675879 | 0.40622902 | 155.6591786 | 222.4418484 |
| YCL042W | YCL042W | 0.01839349 | 0.64739045 | 0.40713957 | 163.5403146 | 237.0674483 |
| YMR002W | MIC17 | 0.01534314 | 0.61680755 | 0.40759198 | 137.0420753 | 187.2915217 |
| YHR067W | HTD2 | 0.39521621 | 0.95675879 | 0.40789687 | 93.16194519 | 104.9403918 |
| YKL041W | VPS24 | 0.00975617 | 0.54423319 | 0.40801068 | 110.3966264 | 137.2452646 |
| YNL135C | FPR1 | 0.06546949 | 0.95675879 | 0.40981074 | 122.0531694 | 158.7996042 |
| YAL055W | PEX22 | 0.09976321 | 0.95675879 | 0.41029103 | 174.0201937 | 256.1836924 |
| YDL161W | ENT1 | 0.00059257 | 0.14967674 | 0.41080526 | 143.7765347 | 199.3725011 |
| YMR070W | MOT3 | 0.15282349 | 0.95675879 | 0.41205009 | 128.3992407 | 170.3187805 |
| YLR371W | ROM2 | 0.21629533 | 0.95675879 | 0.41380136 | 68.8860724 | 58.39978665 |
| YPL226W | NEW1 | 0.1613215 | 0.95675879 | 0.41399657 | 91.81370043 | 101.3680837 |
| YIR020C | YIR020C | 0.203707 | 0.95675879 | 0.41421654 | 161.8274029 | 232.643988 |
| YKL009W | MRT4 | 0.04910526 | 0.92220427 | 0.41480216 | 103.1766201 | 122.5418686 |
| YMR280C | CAT8 | 0.06596704 | 0.95675879 | 0.41584995 | 131.6872798 | 175.8354971 |
| YBR230C | OM14 | 0.02776549 | 0.76802374 | 0.41691699 | 151.3441551 | 212.5202017 |
| YGL237C | HAP2 | 0.11047094 | 0.95675879 | 0.41717627 | 120.7763262 | 155.1446363 |
| YLR192C | HCR1 | 0.18067838 | 0.95675879 | 0.41739592 | 84.51946065 | 87.10583297 |
| YNL016W | PUB1 | 0.02782629 | 0.76802374 | 0.41818791 | 130.3283552 | 172.88677 |
| YDR421W | ARO80 | 0.2326587 | 0.95675879 | 0.41890448 | 156.6679182 | 222.1650878 |
| YCL063W | VAC17 | 0.02246429 | 0.719863 | 0.42078788 | 137.8140255 | 186.4816088 |
| YHR003C | YHR003C | 0.1741476 | 0.95675879 | 0.42183363 | 139.0433264 | 188.608292 |
| YDR071C | PAA1 | 0.41063772 | 0.95675879 | 0.42343112 | 147.6776677 | 204.5290279 |
| YNL146W | YNL146W | 0.39248362 | 0.95675879 | 0.42442685 | 143.6274975 | 196.7624068 |
| YOR293W | RPS10A | 0.09395003 | 0.95675879 | 0.42614437 | 66.32564425 | 51.48578756 |
| YBL071C | YBL071C | 0.03478477 | 0.82522551 | 0.426235 | 146.7963573 | 202.3963692 |
| YHR143W | DSE2 | 0.28467112 | 0.95675879 | 0.42676706 | 154.814289 | 217.3432928 |
| YMR275C | BUL1 | 0.04991701 | 0.92448791 | 0.42747054 | 131.1347872 | 172.8110656 |
| YKL130C | SHE2 | 0.1153859 | 0.95675879 | 0.42766358 | 142.0357099 | 193.2231595 |
| YLR388W | RPS29A | 0.06090358 | 0.95675879 | 0.42793814 | 116.0561607 | 144.4504861 |
| YGL215W | CLG1 | 0.05047455 | 0.9246749 | 0.42808331 | 155.9403665 | 219.2300942 |
| YCR051W | YCR051W | 0.21731072 | 0.95675879 | 0.42816021 | 145.7631653 | 200.1291836 |
| YOL064C | MET22 | 0.13715226 | 0.95675879 | 0.42822325 | 121.9256556 | 155.4101816 |
| YGL167C | PMR1 | 0.01743328 | 0.63955627 | 0.42979776 | 116.951134 | 145.8108751 |
| YLR410W | VIP1 | 0.01275926 | 0.59816436 | 0.42992106 | 116.4456854 | 144.8417904 |
| YDR315C | IPK1 | 0.01099124 | 0.58995493 | 0.42994943 | 132.4777023 | 174.9056335 |
| YLR421C | RPN13 | 0.02404495 | 0.74977299 | 0.4300271 | 138.566999 | 186.3130677 |
| YPL157W | TGS1 | 0.23420379 | 0.95675879 | 0.43019106 | 53.11767109 | 26.02133475 |
| YGL199C | YGL199C | 0.10091095 | 0.95675879 | 0.43188161 | 148.061437 | 203.8029773 |
| YJR014W | TMA22 | 0.07865862 | 0.95675879 | 0.43220504 | 151.1641919 | 209.566982 |
| YIL107C | PFK26 | 0.16649803 | 0.95675879 | 0.43299772 | 152.0031891 | 211.0049337 |
| YML075C | HMG1 | 0.21161336 | 0.95675879 | 0.4337305 | 150.499657 | 208.0596234 |
| YHR066W | SSF1 | 0.07151167 | 0.95675879 | 0.4349646 | 117.7761573 | 146.4742254 |
| YGR207C | YGR207C | 0.09894334 | 0.95675879 | 0.43512203 | 152.0762787 | 210.7785606 |
| YDL169C | UGX2 | 0.10264112 | 0.95675879 | 0.43797702 | 170.7199049 | 245.2569668 |
| YEL062W | NPR2 | 0.05766054 | 0.95675879 | 0.43809965 | 134.1228812 | 176.5967811 |
| YMR205C | PFK2 | 0.00860544 | 0.52898871 | 0.44054534 | 128.0427618 | 164.7748296 |
| YGL066W | SGF73 | 0.04163227 | 0.91069949 | 0.44298407 | 135.118404 | 177.6282309 |
| YLL006W | MMM1 | 0.33170848 | 0.95675879 | 0.44299313 | 73.35959582 | 61.79553127 |
| YKL039W | PTM1 | 0.07638636 | 0.95675879 | 0.44421789 | 145.6051933 | 197.0855322 |
| YGL071W | AFT1 | 0.20928954 | 0.95675879 | 0.44472989 | 144.5857307 | 195.0858873 |
| YMR022W | QRI8 | 0.03778533 | 0.86764809 | 0.44473145 | 150.3696648 | 205.9336246 |
| YER052C | HOM3 | 0.20977912 | 0.95675879 | 0.4461634 | 107.9660381 | 126.1589032 |
| YKL034W | TUL1 | 0.15011677 | 0.95675879 | 0.44651466 | 149.1675435 | 203.3739 |

| | | | | | | |
|---|---|---|---|---|---|---|
| YDR073W | SNF11 | 0.19339222 | 0.95675879 | 0.44758987 | 161.7310571 | 226.7533178 |
| YBR293W | VBA2 | 0.14695671 | 0.95675879 | 0.45248878 | 162.3982594 | 227.1665107 |
| YBR273C | UBX7 | 0.10181373 | 0.95675879 | 0.4530132 | 169.7065025 | 240.7836915 |
| YDL138W | RGT2 | 0.10300953 | 0.95675879 | 0.45339073 | 153.0659679 | 209.5091008 |
| YCL069W | VBA3 | 0.06669344 | 0.95675879 | 0.45364157 | 152.814688 | 208.9948983 |
| YGL149W | YGL149W | 0.06380879 | 0.95675879 | 0.45447853 | 140.5043208 | 185.7631068 |
| YFL001W | DEG1 | 0.21304602 | 0.95675879 | 0.45510559 | 128.0953343 | 162.3822624 |
| YDL118W | YDL118W | 0.01443308 | 0.60760455 | 0.45525013 | 134.3120907 | 174.0173109 |
| YKL212W | SAC1 | 0.30345022 | 0.95675879 | 0.45857742 | 97.47833087 | 104.3648214 |
| YGL253W | HXK2 | 0.00550296 | 0.42413462 | 0.46011218 | 142.7619489 | 189.0334975 |
| YOL115W | PAP2 | 0.01757514 | 0.63955627 | 0.46195877 | 127.6312374 | 160.339294 |
| YDR463W | STP1 | 0.06987717 | 0.95675879 | 0.46689077 | 142.3738043 | 187.1457423 |
| YLR267W | BOP2 | 0.18340652 | 0.95675879 | 0.46744773 | 152.550568 | 206.1373819 |
| YJR090C | GRR1 | 0.10546824 | 0.95675879 | 0.46828319 | 42.71853164 | 0 |
| YNL171C | 0 | 0.22773819 | 0.95675879 | 0.46997203 | 103.7536543 | 114.1848979 |
| YLR365W | YLR365W | 0.15843258 | 0.95675879 | 0.47007576 | 159.4034929 | 218.5406811 |
| YOR309C | YOR309C | 0.00100575 | 0.215934 | 0.47220794 | 125.271127 | 154.1592414 |
| YMR311C | GLC8 | 0.18750138 | 0.95675879 | 0.47522259 | 153.6223823 | 206.8173843 |
| YJL038C | YJL038C | 0.15073963 | 0.95675879 | 0.47531432 | 159.5653727 | 217.9480084 |
| YKL167C | MRP49 | 0.13985667 | 0.95675879 | 0.47909048 | 140.9400806 | 182.3694404 |
| YDR156W | RPA14 | 0.04433051 | 0.92220427 | 0.4809503 | 134.2943848 | 169.586965 |
| YLR266C | PDR8 | 0.18787791 | 0.95675879 | 0.4814881 | 162.4862031 | 222.3698499 |
| YMR307W | GAS1 | 0.23848114 | 0.95675879 | 0.48172015 | 94.71469926 | 95.22194127 |
| YGL244W | RTF1 | 0.40425443 | 0.95675879 | 0.48228152 | 134.6894595 | 170.1001799 |
| YMR241W | YHM2 | 0.00484304 | 0.39992349 | 0.48236773 | 147.5714316 | 194.2460911 |
| YHR154W | RTT107 | 0.23137383 | 0.95675879 | 0.48284878 | 153.5287725 | 205.3370211 |
| YKR042W | UTH1 | 0.00690983 | 0.4785614 | 0.48351225 | 145.3577305 | 189.8983851 |
| YDR080W | VPS41 | 0.28650204 | 0.95675879 | 0.48379588 | 111.2298556 | 125.8416423 |
| YNL136W | EAF7 | 0.09370912 | 0.95675879 | 0.48487499 | 104.2110006 | 112.4928659 |
| YMR193C-A | YMR193C-A | 0.06037171 | 0.95675879 | 0.48667784 | 123.8776021 | 149.0699196 |
| YKR032W | YKR032W | 0.12159201 | 0.95675879 | 0.48721318 | 162.9739194 | 222.3050554 |
| YOL138C | RTC1 | 0.1830932 | 0.95675879 | 0.48998311 | 136.7312336 | 172.6119158 |
| YCL038C | ATG22 | 0.0897676 | 0.95675879 | 0.49080705 | 165.1881456 | 225.8430381 |
| YGL125W | MET13 | 0.01365305 | 0.59822632 | 0.49283215 | 142.1239015 | 182.2386308 |
| YBR194W | SOY1 | 0.4036455 | 0.95675879 | 0.49439584 | 119.5225389 | 139.5813198 |
| YCR031C | RPS14A | 0.01506723 | 0.61680755 | 0.49498233 | 150.3868338 | 197.3682119 |
| YBR289W | SNF5 | 0.3094088 | 0.95675879 | 0.49812095 | 107.7637571 | 116.8899033 |
| YJR077C | MIR1 | 0.03046638 | 0.78255549 | 0.49845825 | 121.589656 | 142.7632292 |
| YGL024W | YGL024W | 0.00759846 | 0.49536441 | 0.49886746 | 114.0757822 | 128.6006394 |
| YHR078W | YHR078W | 0.12528373 | 0.95675879 | 0.50107512 | 164.6800356 | 223.1332547 |
| YOL027C | MDM38 | 0.04814013 | 0.92220427 | 0.50346276 | 156.9290389 | 208.1874348 |
| YMR029C | FAR8 | 0.0240961 | 0.74977299 | 0.50447979 | 147.082828 | 189.5464585 |
| YNL329C | PEX6 | 0.03020421 | 0.78255549 | 0.50464194 | 138.2236295 | 172.9029301 |
| YIL055C | YIL055C | 0.12562453 | 0.95675879 | 0.50497666 | 163.2956914 | 219.8693312 |
| YLR207W | HRD3 | 0.00372369 | 0.34020275 | 0.50498763 | 150.4033186 | 195.6872865 |
| YBR181C | RPS6B | 0.01932555 | 0.66387116 | 0.50547193 | 122.0831768 | 142.4888498 |
| YJL110C | GZF3 | 0.11529044 | 0.95675879 | 0.50566547 | 154.0672686 | 202.4431978 |
| YBR122C | MRPL36 | 0.26304527 | 0.95675879 | 0.50721004 | 84.66955108 | 72.02069653 |
| YKL137W | YKL137W | 0.13110501 | 0.95675879 | 0.50722988 | 137.0823775 | 170.3196844 |
| YFR048W | RMD8 | 0.00347736 | 0.3393585 | 0.50822261 | 167.3515575 | 226.9209102 |
| YJL016W | YJL016W | 0.13528105 | 0.95675879 | 0.51136708 | 166.2032326 | 224.2291821 |
| YLL045C | RPL8B | 0.08897397 | 0.95675879 | 0.51348913 | 132.8471444 | 161.3054153 |
| YGL219C | MDM34 | 0.01243869 | 0.59816436 | 0.51572365 | 136.6076951 | 167.976168 |
| YHL029C | OCA5 | 0.34130374 | 0.95675879 | 0.51809766 | 147.0173736 | 187.0937639 |
| YDL033C | SLM3 | 0.17160735 | 0.95675879 | 0.52027798 | 66.96311518 | 36.57571479 |
| YFL036W | RPO41 | 0.06262326 | 0.95675879 | 0.52252786 | 135.1074921 | 163.9983179 |
| YDL136W | RPL35B | 0.00761389 | 0.49536441 | 0.52257027 | 135.6583105 | 165.0241445 |
| YIL036W | CST6 | 0.14125967 | 0.95675879 | 0.5237655 | 63.99296501 | 30.40838378 |
| YER156C | YER156C | 0.16690677 | 0.95675879 | 0.52396541 | 152.0019747 | 195.4386504 |
| YOR065W | CYT1 | 0.18269255 | 0.95675879 | 0.52402884 | 77.39186293 | 55.49350505 |
| YGL148W | ARO2 | 0.0255749 | 0.76284641 | 0.5251503 | 123.8317493 | 142.4015214 |
| YJL108C | PRM10 | 0.10787281 | 0.95675879 | 0.52656263 | 159.5917979 | 209.2293026 |
| YDR199W | YDR199W | 0.05587108 | 0.95675879 | 0.52810379 | 157.8212607 | 205.6449065 |
| YCR063W | BUD31 | 0.18923224 | 0.95675879 | 0.52852967 | 48.21443962 | 0 |

| | | | | | | |
|---|---|---|---|---|---|---|
| YMR269W | TMA23 | 0.0262928 | 0.76284641 | 0.52937638 | 109.9689723 | 115.678266 |
| YBR174C | :::SWD3 | 0.09939072 | 0.95675879 | 0.53036348 | 108.9446299 | 113.588183 |
| YMR264W | CUE1 | 0.01146747 | 0.59540465 | 0.53070158 | 139.1684768 | 170.2163874 |
| YOR182C | RPS30B | 0.00034575 | 0.10310418 | 0.53338585 | 113.4802291 | 121.5777748 |
| YDR520C | YDR520C | 0.14511614 | 0.95675879 | 0.53541789 | 161.6155961 | 211.5099385 |
| YNR051C | BRE5 | 0.04561551 | 0.92220427 | 0.54118956 | 140.579931 | 171.0691991 |
| YKL113C | RAD27 | 0.11414603 | 0.95675879 | 0.54502185 | 122.0319849 | 135.6260922 |
| YIL053W | RHR2 | 0.14581488 | 0.95675879 | 0.54612339 | 148.5812548 | 185.2318591 |
| YLR061W | RPL22A | 0.23466773 | 0.95675879 | 0.54614863 | 56.22513409 | 12.00988814 |
| YNL299W | TRF5 | 0.30941244 | 0.95675879 | 0.54622944 | 139.094539 | 167.4209933 |
| YDR174W | HMO1 | 0.0078144 | 0.50082146 | 0.55292165 | 114.0277274 | 119.2621766 |
| YCR071C | IMG2 | 0.0002333 | 0.10310418 | 0.55353967 | 120.0265489 | 130.4074692 |
| YDL157C | YDL157C | 0.00253731 | 0.31772128 | 0.5539763 | 176.7169742 | 236.657958 |
| YJR073C | OPI3 | 0.38910339 | 0.95675879 | 0.5578378 | 129.0325909 | 146.5632832 |
| YHL031C | GOS1 | 0.00318896 | 0.33398467 | 0.56043388 | 155.0325607 | 194.8831087 |
| YMR216C | SKY1 | 0.00862348 | 0.52898871 | 0.56083071 | 140.0541855 | 166.7226635 |
| YDL127W | PCL2 | 0.00286245 | 0.31772128 | 0.56090396 | 171.5196212 | 225.7248319 |
| YMR038C | CCS1 | 0.00425415 | 0.38056956 | 0.56305207 | 121.1045693 | 130.8018283 |
| YHL028W | WSC4 | 0.35007186 | 0.95675879 | 0.56538547 | 166.1711171 | 214.9267364 |
| YBL072C | RPS8A | 0.00246825 | 0.31772128 | 0.57100225 | 139.7723303 | 164.4537447 |
| YGR118W | RPS23A | 0.00097193 | 0.215934 | 0.572889 | 139.5849691 | 163.7795302 |
| YOR141C | ARP8 | 0.0014011 | 0.25067939 | 0.58269258 | 133.5077364 | 150.7041031 |
| YMR256C | COX7 | 0.0126884 | 0.59816436 | 0.58604708 | 116.8285509 | 118.8476788 |
| YDL090C | RAM1 | 0.00176178 | 0.27255016 | 0.58983769 | 144.0672686 | 169.2864488 |
| YER151C | UBP3 | 0.03224766 | 0.79965484 | 0.59010307 | 128.9160059 | 140.8242358 |
| YLR373C | VID22 | 0.10887675 | 0.95675879 | 0.59343184 | 92.73856113 | 72.40243984 |
| YOL050C | :::GAL11 | 0.06962001 | 0.95675879 | 0.59388164 | 144.3048364 | 169.0401222 |
| YMR282C | AEP2 | 0.00038418 | 0.10310418 | 0.59423558 | 127.192053 | 136.8838465 |
| YBR269C | FMP21 | 0.28792527 | 0.95675879 | 0.60206729 | 155.7506954 | 189.1067814 |
| YCL044C | MGR1 | 0.01290347 | 0.59816436 | 0.60308522 | 158.1179199 | 193.3724468 |
| YCR009C | RVS161 | 0.30012806 | 0.95675879 | 0.60706777 | 113.0650954 | 108.1926555 |
| YFL023W | BUD27 | 0.01890493 | 0.65465938 | 0.60987223 | 138.4750788 | 155.3702862 |
| YCR020C-A | MAK31 | 0.00285994 | 0.31772128 | 0.61052497 | 166.5336748 | 207.8836406 |
| YHR132W-A | IGO2 | 0.11136173 | 0.95675879 | 0.61062166 | 167.7383264 | 210.1264716 |
| YDR532C | KRE28 | 0.13808068 | 0.95675879 | 0.61185013 | 67.77471861 | 22.43048408 |
| YJR024C | YJR024C | 0.19487474 | 0.95675879 | 0.61332211 | 184.1852593 | 240.5113309 |
| YNL194C | YNL194C | 0.28157585 | 0.95675879 | 0.61406955 | 155.764877 | 187.0798674 |
| YDR375C | BCS1 | 0.0016069 | 0.27255016 | 0.61557733 | 144.1469946 | 165.0320859 |
| YHR112C | YHR112C | 0.12736934 | 0.95675879 | 0.62355673 | 175.5747898 | 222.610966 |
| YML121W | GTR1 | 0.10967163 | 0.95675879 | 0.62835539 | 160.9941379 | 194.4433416 |
| YGR101W | PCP1 | 0.21583805 | 0.95675879 | 0.62871722 | 85.91644079 | 53.57016594 |
| YML074C | FPR3 | 0.06447819 | 0.95675879 | 0.63087586 | 176.0171424 | 222.1883582 |
| YIL074C | SER33 | 0.15413605 | 0.95675879 | 0.63213885 | 178.8389343 | 227.2646541 |
| YNL319W | YNL319W | 0.01416178 | 0.60208591 | 0.63469531 | 156.2536009 | 184.4675511 |
| YGR192C | TDH3 | 0.02430004 | 0.750658 | 0.6354147 | 163.2760176 | 197.5152956 |
| YNL003C | PET8 | 0.0708607 | 0.95675879 | 0.63806071 | 116.955663 | 110.1868745 |
| YKL096W | CWP1 | 0.11843883 | 0.95675879 | 0.63811242 | 166.3762504 | 202.8683444 |
| YNL198C | YNL198C | 0.00335915 | 0.33544632 | 0.63948669 | 130.8654159 | 136.0312037 |
| YBR238C | YBR238C | 0.04190958 | 0.91069949 | 0.63964733 | 178.0836585 | 224.5634499 |
| YMR116C | ASC1 | 0.00036716 | 0.10310418 | 0.64118455 | 120.1505739 | 115.6445891 |
| YLR234W | TOP3 | 0.21518122 | 0.95675879 | 0.64204162 | 142.0214567 | 156.5176795 |
| YLR357W | RSC2 | 0.12545087 | 0.95675879 | 0.64213979 | 83.98723079 | 47.65533656 |
| YGR177C | ATF2 | 0.11572637 | 0.95675879 | 0.64257765 | 171.4810118 | 211.6785577 |
| YOR033C | EXO1 | 1.00E-08 | 4.31E-05 | 0.64841019 | 120.0657383 | 114.2492147 |
| YMR167W | MLH1 | 0.01855476 | 0.6477572 | 0.652891 | 158.2204372 | 185.0432622 |
| YDR382W | RPP2B | 0.24525595 | 0.95675879 | 0.65401352 | 108.4554155 | 91.51488927 |
| YNL235C | :::SIN4 | 0.25227398 | 0.95675879 | 0.66605263 | 71.97374467 | 21.032218 |
| YNL238W | KEX2 | 0.21205858 | 0.95675879 | 0.67065476 | 122.5154634 | 115.0378657 |
| YDL061C | RPS29B | 0.000338 | 0.10310418 | 0.67466376 | 121.545712 | 112.5331441 |
| YBR189W | RPS9B | 0.18412569 | 0.95675879 | 0.67719776 | 87.80749581 | 48.82219806 |
| YKL184W | SPE1 | 0.06490401 | 0.95675879 | 0.68100731 | 62.12401598 | 0 |
| YDL181W | INH1 | 0.00087555 | 0.20886827 | 0.68150266 | 133.5248158 | 133.8303451 |
| YML071C | COG8 | 0.02621514 | 0.76284641 | 0.68461025 | 149.4068281 | 163.0860148 |
| YMR143W | RPS16A | 0.00583236 | 0.43179579 | 0.68657842 | 127.4706893 | 121.6071546 |

| | | | | | | |
|---|---|---|---|---|---|---|
| YCR053W | THR4 | 0.07083502 | 0.95675879 | 0.69270378 | 78.20555732 | 28.16038996 |
| YER153C | PET122 | 0.03565461 | 0.83206998 | 0.70337363 | 116.9164102 | 98.93862019 |
| YDL081C | RPP1A | 0.00177723 | 0.27255016 | 0.7115293 | 131.5592675 | 125.0065087 |
| YDL104C | QRI7 | 0.00254367 | 0.31772128 | 0.71658695 | 147.9034992 | 154.795446 |
| YLR182W | SWI6 | 0.07392042 | 0.95675879 | 0.72078421 | 92.66136659 | 50.46845943 |
| YLL039C | UBI4 | 0.0840002 | 0.95675879 | 0.72806641 | 157.7243004 | 171.2506932 |
| YDL232W | OST4 | 0.00032141 | 0.10310418 | 0.75879764 | 150.0415989 | 151.5835522 |
| YDL182W | LYS20 | 3.57E-05 | 0.03069366 | 0.77194902 | 144.2119041 | 138.3996038 |
| YDL083C | RPS16B | 0.00028401 | 0.10310418 | 0.77341705 | 138.4765982 | 127.3916333 |
| YLR318W | EST2 | 0.06558076 | 0.95675879 | 0.77492369 | 94.18108147 | 44.05581831 |
| YCR028C-A | RIM1 | 5.87E-05 | 0.04200147 | 0.7787206 | 152.7865913 | 153.3232029 |
| YIL014W | MNT3 | 0.10197981 | 0.95675879 | 0.78154144 | 192.6269207 | 227.5627285 |
| YBR082C | UBC4 | 1.82E-05 | 0.02609829 | 0.7968767 | 155.8662701 | 155.9928698 |
| YGL218W | YGL218W | 0.04664307 | 0.92220427 | 0.81647791 | 92.19114823 | 33.21394989 |
| YAL010C | MDM10 | 0.00911178 | 0.54423319 | 0.83059844 | 102.1080333 | 49.3975343 |
| YGL007W | YGL007W | 0.01820209 | 0.64739045 | 0.84687351 | 171.933605 | 177.5736647 |
| YIL134W | FLX1 | 0.00553133 | 0.42413462 | 0.84849949 | 143.0899009 | 123.197933 |
| YGL105W | ARC1 | 0.16469721 | 0.95675879 | 0.89574846 | 132.3774106 | 95.02221642 |
| YOL052C | SPE2 | 0.09993753 | 0.95675879 | 0.89666025 | 81.79667806 | 0 |
| YOL095C | HMI1 | 0.01519178 | 0.61680755 | 0.8974265 | 139.3745043 | 107.8584488 |
| YBR132C | AGP2 | 0.00121361 | 0.23687528 | 0.95746539 | 103.1805511 | 29.70293898 |
| YGL151W | NUT1 | 0.07271386 | 0.95675879 | 0.96607488 | 90.16409265 | 3.817013942 |
| YDL062W | YDL062W | 0.0101605 | 0.55934867 | 1.01850548 | 123.4461436 | 57.26830061 |

**Table S8  Fitness on HU**

**Key**

| | |
|---|---|
| ORF: | Open reading frame Y-number for *yfg* deletion |
| Gene: | Standard human-readable gene name for *yfg* deletion |
| P: | p-value for the significance of difference between observed mean fitness of treated strains and predicted fitness estimated from mean fitness observed on CSM |
| Q: | False discovery rate corrected p-value (correcting for multiple testing) |
| FD: | Fitness differential (difference between observed mean fitness of treated strains and predicted fitness estimated from mean fitness observed on control media) |
| Mean_HU: | Mean fitness for all replicate strains following treatment with 100mM HU |
| Mean_CSM: | Mean fitness for all replicate strains grown on CSM |

**Supplementary Table 8: HU Screen Data**

| ORF | Gene | P | Q | FD | Mean_HU | Mean_CSM |
|---|---|---|---|---|---|---|
| YJL027C | YJL027C | 0.00111158 | 0.08349939 | -1.3618674 | 0 | 243.8728124 |
| YFR041C | ERJ5 | 0.0004733 | 0.04956904 | -1.3082216 | 0 | 234.2663362 |
| YDR386W | MUS81 | 0.00044155 | 0.04746692 | -1.3080045 | 0 | 234.2274532 |
| YBR171W | SEC66 | 0.00028148 | 0.03774117 | -1.2566354 | 0 | 225.0286735 |
| YDL059C | RAD59 | 7.48E-05 | 0.01888307 | -1.225237 | 0 | 219.4060858 |
| YDR004W | RAD57 | 0.00022847 | 0.03774117 | -1.1928893 | 0 | 213.6135108 |
| YBL094C | YBL094C | 0.00271858 | 0.16918248 | -1.1864228 | 0 | 212.4555358 |
| YHR059W | FYV4 | 7.34E-05 | 0.01888307 | -1.1603776 | 0 | 207.7915478 |
| YBR098W | MMS4 | 0.00022201 | 0.03774117 | -1.141468 | 11.37387874 | 225.6248206 |
| YBR099C | YBR099C | 2.18E-05 | 0.00935957 | -1.1295856 | 5.620571345 | 212.7634574 |
| YFR039C | YFR039C | 3.55E-05 | 0.01089207 | -1.1154709 | 12.01370204 | 222.1631235 |
| YHR154W | RTT107 | 4.48E-05 | 0.01282041 | -1.09383 | 12.77825087 | 219.7142024 |
| YDR076W | RAD55 | 1.95E-05 | 0.00935957 | -1.0438954 | 5.76896038 | 197.6955499 |
| YLR056W | ERG3 | 0.0003576 | 0.04516271 | -1.0339986 | 0 | 185.1605721 |
| YDR207C | UME6 | 3.54E-05 | 0.01089207 | -1.033385 | 0 | 185.0506958 |
| YIL128W | MET18 | 0.00156009 | 0.10467248 | -1.0294415 | 0 | 184.3445149 |
| YKL204W | EAP1 | 0.00054438 | 0.05565634 | -1.0255332 | 0 | 183.6446516 |
| YLR032W | RAD5 | 0.00126956 | 0.09040142 | -1.0223185 | 0 | 183.0689917 |
| YKL032C | IXR1 | 0.00130528 | 0.09040142 | -0.9920891 | 12.01668897 | 200.0744272 |
| YBR100W | YBR100W | 2.78E-05 | 0.01086646 | -0.9845909 | 4.867694442 | 185.3943319 |
| YDR266C | YDR266C | 0.0077304 | 0.42556841 | -0.9544936 | 25.24426775 | 218.0198717 |
| YNL111C | CYB5 | 0.00042807 | 0.04746692 | -0.9414964 | 19.36254667 | 204.7193197 |
| YML008C | ERG6 | 0.00022941 | 0.03774117 | -0.9375299 | 0 | 167.8856885 |
| YCL060C | :::MRC1 | 0.0564829 | 0.96815259 | -0.9282638 | 29.9852292 | 222.167725 |
| YJL092W | HPR5 | 0.00143151 | 0.09756999 | -0.924237 | 18.4896149 | 200.0000709 |
| YMR015C | ERG5 | 0.0001392 | 0.0291563 | -0.9229403 | 0 | 165.2731047 |
| YGL163C | RAD54 | 0.00084918 | 0.07441579 | -0.9203284 | 10.23758729 | 183.9049201 |
| YJL047C | RTT101 | 0.00016944 | 0.03163327 | -0.9101058 | 12.32909542 | 185.9763125 |
| YGL127C | SOH1 | 0.00027311 | 0.03774117 | -0.8924268 | 0 | 159.8089738 |
| YOR141C | ARP8 | 0.00177578 | 0.11553326 | -0.8826566 | 0 | 158.0593943 |
| YNL153C | GIM3 | 2.04E-05 | 0.00935957 | -0.8824702 | 4.732649323 | 166.8553892 |
| YMR038C | CCS1 | 0.00097936 | 0.08087239 | -0.8815672 | 0 | 157.8643125 |
| YDR369C | XRS2 | 0.00084279 | 0.07441579 | -0.8777213 | 5.575503927 | 167.5774479 |
| YPL194W | DDC1 | 0.03329548 | 0.93883273 | -0.861325 | 31.84010034 | 213.6413317 |
| YLR234W | TOP3 | 0.00015015 | 0.02930666 | -0.8523725 | 5.103798677 | 162.1581531 |
| YDL074C | BRE1 | 2.59E-07 | 0.00018504 | -0.8425735 | 12.6636649 | 174.5073329 |
| YER095W | RAD51 | 0.00023987 | 0.03774117 | -0.8409815 | 6.554620808 | 162.8250341 |
| YNL171C | 0 | 0.00260483 | 0.16448723 | -0.8371646 | 0 | 149.913042 |
| YNL215W | IES2 | 1.37E-05 | 0.00843101 | -0.8308272 | 4.536572245 | 157.2417535 |
| YGL175C | SAE2 | 0.05276312 | 0.96815259 | -0.8287006 | 51.84647574 | 245.1236819 |
| YJR074W | MOG1 | 0.01340576 | 0.63257512 | -0.8224889 | 26.69098856 | 197.0805187 |
| YGR078C | PAC10 | 0.00075252 | 0.06875145 | -0.8224782 | 13.63885283 | 172.7281566 |
| YBR094W | PBY1 | 0.09681751 | 0.96815259 | -0.8159716 | 33.3806059 | 208.3938109 |
| YNL319W | YNL319W | 0.00627124 | 0.36390143 | -0.8119377 | 30.79571759 | 202.8489913 |
| YBR231C | SWC5 | 0.07542237 | 0.96815259 | -0.8079177 | 39.05029497 | 217.5291136 |
| YEL059W | YEL059W | 0.00044217 | 0.04746692 | -0.8038092 | 7.349111699 | 157.6507238 |
| YER173W | RAD24 | 2.60E-13 | 2.79E-10 | -0.8019755 | 31.6390377 | 202.6383605 |

| | | | | | | |
|---|---|---|---|---|---|---|
| YKR082W | NUP133 | 0.00073647 | 0.06874788 | -0.7956066 | 0 | 142.4711549 |
| YER083C | GET2 | 0.00039645 | 0.04736515 | -0.7833159 | 5.340969931 | 150.2344908 |
| YNL250W | RAD50 | 4.69E-15 | 2.01E-11 | -0.7665382 | 7.249573775 | 150.7908179 |
| YLR235C | YLR235C | 0.00059858 | 0.05841598 | -0.7657239 | 6.17114441 | 148.6330504 |
| YPL008W | CHL1 | 0.01888185 | 0.76489316 | -0.7638776 | 31.61440186 | 195.7701169 |
| YOR368W | RAD17 | 1.41E-11 | 1.21E-08 | -0.7557082 | 32.31967535 | 195.6229835 |
| YLR320W | MMS22 | 0.00103122 | 0.0820012 | -0.7548481 | 5.6649998 | 145.7412263 |
| YHR194W | MDM31 | 0.04520072 | 0.96815259 | -0.7539547 | 39.62153594 | 208.9315484 |
| YAR002W | NUP60 | 0.13950895 | 0.96815259 | -0.752268 | 43.77899679 | 216.385797 |
| YOR312C | RPL20B | 0.07457147 | 0.96815259 | -0.7511616 | 35.71375269 | 201.1409199 |
| YML032C | RAD52 | 2.36E-13 | 2.79E-10 | -0.7485659 | 12.51578477 | 157.3972881 |
| YNL147W | LSM7 | 0.01640615 | 0.72492154 | -0.7472959 | 6.565581868 | 146.0689921 |
| YPR179C | HDA3 | 0.0003971 | 0.04736515 | -0.745676 | 6.728768523 | 146.0833526 |
| YDR092W | UBC13 | 0.01780336 | 0.73573115 | -0.7340187 | 46.89861505 | 218.9378995 |
| YBR076W | ECM8 | 0.10359804 | 0.96815259 | -0.7337852 | 30.8275907 | 188.9135122 |
| YPR164W | MMS1 | 0.06872873 | 0.96815259 | -0.7291964 | 0 | 130.578922 |
| YLR292C | SEC72 | 0.0874764 | 0.96815259 | -0.7194335 | 27.7577275 | 180.616289 |
| YNL298W | CLA4 | 0.0009128 | 0.0773828 | -0.7150153 | 24.68481951 | 174.0922115 |
| YKL113C | RAD27 | 0.00091908 | 0.0773828 | -0.7143699 | 13.98506891 | 154.0148626 |
| YDL077C | VAM6 | 0.02278602 | 0.81908097 | -0.7140441 | 17.85194697 | 161.170681 |
| YGL023C | PIB2 | 0.12238351 | 0.96815259 | -0.7139208 | 47.86494209 | 217.141732 |
| YER116C | SLX8 | 0.0927136 | 0.96815259 | -0.7098149 | 37.54782843 | 197.1585671 |
| YER111C | SWI4 | 0.1084845 | 0.96815259 | -0.7086991 | 38.8401192 | 199.3696867 |
| YNL068C | FKH2 | 0.0115686 | 0.57098356 | -0.7070153 | 33.69774797 | 189.4744116 |
| YMR116C | ASC1 | 3.25E-05 | 0.01089207 | -0.7056078 | 0 | 126.3548617 |
| YNL307C | MCK1 | 0.00025962 | 0.03774117 | -0.7054793 | 18.43686735 | 160.728206 |
| YBL024W | NCL1 | 0.08038207 | 0.96815259 | -0.7027498 | 51.05120322 | 221.0857038 |
| YMR224C | MRE11 | 4.47E-14 | 9.59E-11 | -0.7016243 | 13.13832554 | 150.1527692 |
| YGL167C | PMR1 | 0.00026521 | 0.03774117 | -0.6997198 | 0 | 125.300474 |
| YNL242W | ATG2 | 0.09861082 | 0.96815259 | -0.6995435 | 28.44908178 | 178.3443504 |
| YPL024W | RMI1 | 0.07759253 | 0.96815259 | -0.6990505 | 25.83802535 | 173.3848034 |
| YCL016C | DCC1 | 0.00751326 | 0.41898615 | -0.6937409 | 27.00018747 | 174.6021648 |
| YOR273C | TPO4 | 0.0630285 | 0.96815259 | -0.6928381 | 40.13742977 | 198.9497271 |
| YGR184C | UBR1 | 0.02906094 | 0.91703766 | -0.6886339 | 46.89271539 | 210.7997248 |
| YHL025W | SNF6 | 0.00042143 | 0.04746692 | -0.6882712 | 11.28415434 | 144.3024045 |
| YIL090W | ICE2 | 0.13658169 | 0.96815259 | -0.6685562 | 31.9718406 | 179.3675502 |
| YLR268W | SEC22 | 0.1209139 | 0.96815259 | -0.6594463 | 29.53892882 | 173.1972951 |
| YMR060C | SAM37 | 0.03411814 | 0.94810002 | -0.6538721 | 17.32409871 | 149.4107614 |
| YMR078C | CTF18 | 0.00058194 | 0.0581128 | -0.6525504 | 10.89212555 | 137.1744126 |
| YNL064C | YDJ1 | 0.0011034 | 0.08349939 | -0.651425 | 0 | 116.6522057 |
| YNL099C | OCA1 | 0.10223402 | 0.96815259 | -0.6486597 | 36.99293704 | 185.1721453 |
| YKR055W | RHO4 | 0.10290744 | 0.96815259 | -0.6438017 | 37.868965 | 185.9365424 |
| YFR010W | UBP6 | 0.04387348 | 0.96815259 | -0.6436896 | 37.46115107 | 185.1556454 |
| YPR120C | CLB5 | 0.06908864 | 0.96815259 | -0.6400608 | 42.17122974 | 193.293092 |
| YMR190C | SGS1 | 0.1198267 | 0.96815259 | -0.6385383 | 28.62074149 | 167.7402627 |
| YCL037C | SRO9 | 0.01780624 | 0.73573115 | -0.6277443 | 32.73460159 | 173.4822845 |
| YBR289W | SNF5 | 0.00107127 | 0.08349939 | -0.6271003 | 12.08500464 | 134.8424664 |
| YNL183C | NPR1 | 0.00613193 | 0.36069186 | -0.6253199 | 67.83000655 | 238.5231632 |
| YPL055C | LGE1 | 0.09660018 | 0.96815259 | -0.6244767 | 31.90589847 | 171.3511029 |
| YJR043C | POL32 | 0.14443402 | 0.96815259 | -0.6226252 | 36.61874593 | 179.8119771 |
| YKL212W | SAC1 | 0.00065998 | 0.06297637 | -0.6193079 | 0 | 110.9009355 |
| YDR049W | YDR049W | 0.13068943 | 0.96815259 | -0.6177283 | 37.9600223 | 181.4374054 |
| YHL022C | SPO11 | 0.18202354 | 0.96815259 | -0.6162378 | 53.1435281 | 209.4972943 |
| YOR113W | AZF1 | 0.14370268 | 0.96815259 | -0.614851 | 51.71447483 | 206.5828744 |
| YBL031W | SHE1 | 0.06189138 | 0.96815259 | -0.6032534 | 55.74720803 | 212.0296497 |
| YDL119C | YDL119C | 0.17052119 | 0.96815259 | -0.6016752 | 41.69698219 | 185.5345264 |
| YHR191C | CTF8 | 0.08006392 | 0.96815259 | -0.5972697 | 39.00949267 | 179.7317636 |
| YDL118W | YDL118W | 0.14801268 | 0.96815259 | -0.5969746 | 38.68568096 | 179.0747902 |
| YJL124C | LSM1 | 0.08816747 | 0.96815259 | -0.5942878 | 28.23562655 | 159.0977413 |
| YPL080C | YPL080C | 0.06803996 | 0.96815259 | -0.5941311 | 36.33260247 | 174.1756345 |
| YBL089W | AVT5 | 0.10287823 | 0.96815259 | -0.5904643 | 32.87183821 | 167.0625028 |
| YBR227C | MCX1 | 0.01781929 | 0.73573115 | -0.5873207 | 72.56294001 | 240.5484623 |
| YKL213C | DOA1 | 0.03135329 | 0.9284317 | -0.5866461 | 30.00996684 | 161.0395873 |
| YHR134W | WSS1 | 0.08160925 | 0.96815259 | -0.5864808 | 49.02860809 | 196.4917268 |

| | | | | | | |
|---|---|---|---|---|---|---|
| YCL061C | MRC1 | 0.07050261 | 0.96815259 | -0.5862714 | 37.65339873 | 175.2322903 |
| YPR135W | CTF4 | 0.15737222 | 0.96815259 | -0.5862369 | 29.85950824 | 160.6856063 |
| YKL048C | ELM1 | 0.12460143 | 0.96815259 | -0.5847299 | 35.80875789 | 171.5148444 |
| YHR041C | SRB2 | 0.1257622 | 0.96815259 | -0.5831796 | 26.83435026 | 154.4943007 |
| YAR003W | SWD1 | 0.06493477 | 0.96815259 | -0.579231 | 17.64255222 | 136.6387239 |
| YDR463W | STP1 | 0.18100041 | 0.96815259 | -0.578005 | 52.24512668 | 200.9747633 |
| YGL136C | MRM2 | 0.1811578 | 0.96815259 | -0.5777998 | 53.95494794 | 204.1279093 |
| YCR077C | PAT1 | 0.14401127 | 0.96815259 | -0.5765068 | 28.30752817 | 156.0477859 |
| YLR218C | YLR218C | 0.07505711 | 0.96815259 | -0.5749653 | 0 | 102.9604009 |
| YHR178W | STB5 | 0.15681861 | 0.96815259 | -0.5699513 | 39.27493175 | 175.3349887 |
| YDR126W | SWF1 | 0.14506516 | 0.96815259 | -0.5698639 | 28.07076006 | 154.4165037 |
| YJL115W | ASF1 | 0.06002708 | 0.96815259 | -0.566548 | 0 | 101.4530798 |
| YLR357W | RSC2 | 0.12378041 | 0.96815259 | -0.5635016 | 25.76174603 | 148.9694401 |
| YOR026W | BUB3 | 0.07319748 | 0.96815259 | -0.5595397 | 29.15093316 | 154.5829357 |
| YDR392W | SPT3 | 0.0876561 | 0.96815259 | -0.5566761 | 37.82070992 | 170.2447284 |
| YPL226W | NEW1 | 0.09093652 | 0.96815259 | -0.5550674 | 30.91053146 | 157.0648307 |
| YLR337C | VRP1 | 0.0002581 | 0.03774117 | -0.5530232 | 4.570946147 | 107.5588653 |
| YBR200W | BEM1 | 0.18250622 | 0.96815259 | -0.5457963 | 38.23218888 | 169.0641268 |
| YMR031W-A | YMR031W-A | 0.14896605 | 0.96815259 | -0.5442988 | 35.81107011 | 164.2790585 |
| YBR057C | MUM2 | 0.09056393 | 0.96815259 | -0.5405873 | 63.47075639 | 215.2171573 |
| YMR312W | ELP6 | 0.10220772 | 0.96815259 | -0.5403447 | 29.60095995 | 151.9852263 |
| YLR192C | HCR1 | 0.02310788 | 0.82004318 | -0.5372474 | 29.7958183 | 151.794114 |
| YMR198W | CIK1 | 0.14307838 | 0.96815259 | -0.5283749 | 34.81107774 | 159.5619133 |
| YOL081W | IRA2 | 0.14505691 | 0.96815259 | -0.5280054 | 32.31571253 | 154.8403262 |
| YDR074W | TPS2 | 0.21905976 | 0.96815259 | -0.525614 | 52.37821079 | 191.8412722 |
| YDL006W | PTC1 | 0.1280374 | 0.96815259 | -0.5199665 | 32.86561257 | 154.4266829 |
| YEL033W | MTC7 | 0.10856725 | 0.96815259 | -0.5162775 | 38.14854139 | 163.6220779 |
| YFL023W | BUD27 | 0.1964174 | 0.96815259 | -0.5158577 | 44.65127569 | 175.6785999 |
| YBR023C | CHS3 | 0.16589065 | 0.96815259 | -0.5117971 | 76.61174826 | 234.5778542 |
| YLL002W | RTT109 | 0.00029005 | 0.03774117 | -0.5083229 | 0 | 91.02657253 |
| YNL106C | INP52 | 0.14616218 | 0.96815259 | -0.5051116 | 40.30441894 | 165.6446363 |
| YDR244W | PEX5 | 0.20488476 | 0.96815259 | -0.5045853 | 74.26899475 | 228.9157077 |
| YPL079W | RPL21B | 0.06915212 | 0.96815259 | -0.5038885 | 55.75553602 | 194.2516753 |
| YBL027W | RPL19B | 0.19843544 | 0.96815259 | -0.5022792 | 45.91106924 | 175.5973661 |
| YNL297C | MON2 | 0.0358379 | 0.96815259 | -0.5015472 | 30.5349292 | 146.7801084 |
| YKL041W | VPS24 | 0.0772466 | 0.96815259 | -0.4978957 | 31.28885338 | 147.5327727 |
| YML038C | YMD8 | 0.13306129 | 0.96815259 | -0.4971676 | 36.24961518 | 156.65732 |
| YPL213W | LEA1 | 0.07519593 | 0.96815259 | -0.496154 | 41.82944164 | 166.8857072 |
| YLR418C | CDC73 | 0.11175098 | 0.96815259 | -0.4938559 | 30.05256215 | 144.502887 |
| YJR145C | RPS4A | 0.16309699 | 0.96815259 | -0.491767 | 68.51649276 | 215.8882645 |
| YJL175W | YJL175W | 0.00462464 | 0.28368843 | -0.4897186 | 11.75440615 | 109.6244284 |
| YNL170W | YNL170W | 0.21069617 | 0.96815259 | -0.489409 | 23.10295973 | 130.7411869 |
| YBR147W | RTC2 | 0.193481 | 0.96815259 | -0.4883993 | 27.91535073 | 139.538511 |
| YPL170W | DAP1 | 0.01850682 | 0.7568406 | -0.4877174 | 77.21769143 | 231.3963024 |
| YPL157W | TGS1 | 0.00112785 | 0.08349939 | -0.486874 | 10.46253538 | 106.7048918 |
| YLR414C | YLR414C | 0.11109799 | 0.96815259 | -0.4857746 | 37.54380683 | 157.0316445 |
| YDL013W | HEX3 | 0.24083368 | 0.96815259 | -0.484281 | 31.63875816 | 145.7475351 |
| YML097C | VPS9 | 0.01627621 | 0.72492154 | -0.479613 | 25.6020153 | 133.6493114 |
| YNL199C | GCR2 | 0.14646798 | 0.96815259 | -0.47829 | 39.74173572 | 159.7918783 |
| YOL049W | GSH2 | 0.27503182 | 0.96815259 | -0.4758383 | 30.96151076 | 142.9722038 |
| YGR188C | BUB1 | 0.10793305 | 0.96815259 | -0.4757263 | 36.4208678 | 153.1372804 |
| YOL006C | TOP1 | 0.06048941 | 0.96815259 | -0.4736128 | 16.22384002 | 115.0786348 |
| YBR175W | SWD3 | 0.01728475 | 0.73573115 | -0.4675012 | 22.0357712 | 124.8271311 |
| YBR215W | HPC2 | 0.06466773 | 0.96815259 | -0.4666616 | 81.80242687 | 236.179218 |
| YOR035C | SHE4 | 0.11900207 | 0.96815259 | -0.4650592 | 35.75126929 | 149.977871 |
| YMR166C | YMR166C | 0.12540217 | 0.96815259 | -0.4626128 | 11.088353 | 103.5279235 |
| YDR455C | :::GUK1 | 0.17254898 | 0.96815259 | -0.4621445 | 47.22279281 | 170.8575468 |
| YLR177W | YLR177W | 0.10136548 | 0.96815259 | -0.4590784 | 32.1929462 | 142.2683669 |
| YKL069W | YKL069W | 0.25936716 | 0.96815259 | -0.456551 | 30.83421333 | 139.2808792 |
| YOR231W | MKK1 | 0.03444425 | 0.94810002 | -0.4565227 | 90.58175737 | 250.7425969 |
| YDR080W | VPS41 | 0.08081328 | 0.96815259 | -0.4562623 | 26.20632371 | 130.595251 |
| YPL180W | TCO89 | 0.09859691 | 0.96815259 | -0.4543329 | 32.0733111 | 141.1953791 |
| YPL090C | RPS6A | 0.04411837 | 0.96815259 | -0.4498658 | 49.06803338 | 172.1013013 |
| YHL005C | YHL005C | 0.1343112 | 0.96815259 | -0.4443614 | 62.20813067 | 195.6301749 |

| | | | | | | |
|---|---|---|---|---|---|---|
| YIL040W | APQ12 | 0.24519498 | 0.96815259 | -0.4415113 | 28.15115764 | 131.5820951 |
| YBR036C | CSG2 | 0.16254135 | 0.96815259 | -0.4391201 | 51.63205515 | 174.9605583 |
| YLR055C | SPT8 | 0.10246355 | 0.96815259 | -0.438496 | 48.33518879 | 168.6980713 |
| YHL003C | LAG1 | 0.24089598 | 0.96815259 | -0.4375229 | 48.12791905 | 168.1371226 |
| YOR106W | VAM3 | 0.18063797 | 0.96815259 | -0.4338955 | 21.56326969 | 117.9277664 |
| YER178W | PDA1 | 0.23071958 | 0.96815259 | -0.4326895 | 70.19154046 | 208.4341443 |
| YNL271C | BNI1 | 0.18827884 | 0.96815259 | -0.428536 | 51.88008528 | 173.5279702 |
| YBR174C | :::SWD3 | 0.17208116 | 0.96815259 | -0.4244343 | 24.85617895 | 122.3768725 |
| YGL024W | YGL024W | 0.27622509 | 0.96815259 | -0.4218052 | 38.59984308 | 147.5466591 |
| YDR025W | RPS11A | 0.17245922 | 0.96815259 | -0.4201568 | 57.67821461 | 182.8446565 |
| YHL033C | RPL8A | 0.19514448 | 0.96815259 | -0.4197632 | 64.64524506 | 195.7720722 |
| YPR013C | YPR013C | 0.34980346 | 0.96815259 | -0.41899 | 32.35898937 | 135.3994106 |
| YDR173C | ARG82 | 0.32879264 | 0.96815259 | -0.4159189 | 33.07460555 | 136.1845355 |
| YML022W | APT1 | 0.28125277 | 0.96815259 | -0.4128324 | 33.06347508 | 135.6110743 |
| YDL100C | GET3 | 0.00173985 | 0.11493681 | -0.4112558 | 76.27532647 | 215.9460503 |
| YDL020C | RPN4 | 0.21921438 | 0.96815259 | -0.407562 | 54.36011223 | 174.3989141 |
| YCR020C | PET18 | 0.22599112 | 0.96815259 | -0.4074741 | 75.13466521 | 213.1407963 |
| YLR200W | YKE2 | 0.24340959 | 0.96815259 | -0.4074415 | 54.48623335 | 174.61263 |
| YGR223C | HSV2 | 0.11748837 | 0.96815259 | -0.4070673 | 59.44160713 | 183.7905203 |
| YJR039W | YJR039W | 0.1984262 | 0.96815259 | -0.4046721 | 54.62760203 | 174.380458 |
| YOL061W | PRS5 | 0.24789839 | 0.96815259 | -0.4042064 | 76.23221763 | 214.6032767 |
| YGL105W | ARC1 | 0.02289001 | 0.81908097 | -0.4022488 | 8.769391935 | 88.39207869 |
| YDR024W | FYV1 | 0.31137698 | 0.96815259 | -0.401094 | 62.40073617 | 188.2414975 |
| YPR024W | YME1 | 0.16871499 | 0.96815259 | -0.4002926 | 36.78043302 | 140.2999938 |
| YDR162C | NBP2 | 0.30137337 | 0.96815259 | -0.3992706 | 68.15434799 | 198.6490839 |
| YCL022C | YCL022C | 0.02081525 | 0.78250728 | -0.3971318 | 95.5638834 | 249.4021273 |
| YOL108C | INO4 | 0.27197469 | 0.96815259 | -0.3938848 | 45.75830366 | 155.9019288 |
| YHR004C | NEM1 | 0.03188026 | 0.9284317 | -0.3917848 | 100.4006108 | 257.4681781 |
| YJL211C | YJL211C | 0.296432 | 0.96815259 | -0.3842425 | 80.78593792 | 219.5238421 |
| YNL141W | AAH1 | 0.242492 | 0.96815259 | -0.3793201 | 41.69130772 | 145.7062835 |
| YGR259C | YGR259C | 0.28338875 | 0.96815259 | -0.3772248 | 80.02956338 | 216.8560566 |
| YPL144W | POC4 | 0.07574908 | 0.96815259 | -0.3711552 | 74.97824328 | 206.345255 |
| YGR135W | PRE9 | 0.38395877 | 0.96815259 | -0.369039 | 56.86816811 | 172.1796124 |
| YBR082C | UBC4 | 0.03110431 | 0.9284317 | -0.3672804 | 60.02094588 | 177.7466182 |
| YHR180W | YHR180W | 0.02261994 | 0.81908097 | -0.3666711 | 93.96197312 | 240.9588957 |
| YJL082W | IML2 | 0.27004884 | 0.96815259 | -0.3612613 | 66.05748663 | 187.930707 |
| YGR105W | VMA21 | 0.38263271 | 0.96815259 | -0.3542686 | 25.97950048 | 111.9078371 |
| YGL168W | HUR1 | 0.35192359 | 0.96815259 | -0.3512767 | 26.49277428 | 112.3296429 |
| YLR334C | YLR334C | 0.2244151 | 0.96815259 | -0.350474 | 49.08759217 | 154.3394698 |
| YPL106C | SSE1 | 0.31208256 | 0.96815259 | -0.3482744 | 43.81469782 | 144.108319 |
| YHR116W | COX23 | 0.24596036 | 0.96815259 | -0.3460053 | 53.30636548 | 161.4099162 |
| YDR217C | RAD9 | 8.27E-05 | 0.0197365 | -0.3423625 | 74.04653272 | 199.4510594 |
| YCR081W | SRB8 | 0.13910918 | 0.96815259 | -0.3423405 | 32.11682488 | 121.2218141 |
| YPL047W | SGF11 | 0.27623077 | 0.96815259 | -0.3409502 | 67.13883196 | 186.3109378 |
| YBR111C | YSA1 | 0.29978034 | 0.96815259 | -0.339748 | 92.99459886 | 234.3329526 |
| YOR322C | LDB19 | 0.11144411 | 0.96815259 | -0.3393662 | 71.55880611 | 194.2733348 |
| YPR160W | GPH1 | 0.00129039 | 0.09040142 | -0.3372214 | 56.88164101 | 166.5071052 |
| YHR086W | NAM8 | 0.39054665 | 0.96815259 | -0.3365775 | 81.34135634 | 212.0245617 |
| YOL001W | PHO80 | 0.35807558 | 0.96815259 | -0.3339946 | 50.7325266 | 154.4573056 |
| YLR402W | YLR402W | 0.32006193 | 0.96815259 | -0.3338051 | 13.54930631 | 85.05325942 |
| YPL022W | RAD1 | 0.02119532 | 0.78459243 | -0.3324061 | 102.0980234 | 250.0018244 |
| YJR073C | OPI3 | 0.21024244 | 0.96815259 | -0.3314434 | 57.91558914 | 167.4013776 |
| YIL116W | HIS5 | 0.05153992 | 0.96815259 | -0.3295844 | 93.9123602 | 234.2251279 |
| YML035C | AMD1 | 0.20671472 | 0.96815259 | -0.3260843 | 56.42802023 | 163.666462 |
| YEL007W | YEL007W | 0.35157265 | 0.96815259 | -0.3231778 | 44.24018265 | 140.408002 |
| YKL109W | HAP4 | 0.00119602 | 0.0870461 | -0.3195811 | 56.14529096 | 161.9744452 |
| YFL034W | YFL034W | 0.38441633 | 0.96815259 | -0.3175859 | 83.55518307 | 212.7538743 |
| YPL105C | YPL105C | 0.10575672 | 0.96815259 | -0.3148707 | 89.67280036 | 223.6808633 |
| YJL176C | SWI3 | 0.34953311 | 0.96815259 | -0.3145434 | 79.71750908 | 205.0493657 |
| YCL035C | GRX1 | 0.31377479 | 0.96815259 | -0.3127653 | 96.92100178 | 236.8263011 |
| YOR040W | GLO4 | 0.06149769 | 0.96815259 | -0.3105177 | 97.08822768 | 236.7357914 |
| YLL038C | ENT4 | 0.04495412 | 0.96815259 | -0.3101913 | 86.34188262 | 216.6286545 |
| YOR360C | PDE2 | 0.04635404 | 0.96815259 | -0.3100287 | 75.51219027 | 196.395347 |
| YNL091W | NST1 | 0.03032472 | 0.92350606 | -0.3093388 | 31.50655211 | 114.1735788 |

| | | | | | | |
|---|---|---|---|---|---|---|
| YDR469W | SDC1 | 0.26945869 | 0.96815259 | -0.3077679 | 57.03358932 | 161.5162757 |
| YAL002W | VPS8 | 0.34153261 | 0.96815259 | -0.3062001 | 60.42023171 | 167.5537333 |
| YGR229C | SMI1 | 0.34446525 | 0.96815259 | -0.3056597 | 38.87429816 | 127.2602473 |
| YGR292W | MAL12 | 0.04154297 | 0.96815259 | -0.3040498 | 96.08306219 | 233.7023153 |
| YMR107W | SPG4 | 0.38247759 | 0.96815259 | -0.3037905 | 80.37490824 | 204.3502881 |
| YBR267W | REI1 | 0.42692675 | 0.96815259 | -0.3023556 | 35.03809757 | 119.5116461 |
| YNL032W | SIW14 | 0.10473591 | 0.96815259 | -0.3016983 | 95.65244965 | 232.4778545 |
| YOR267C | HRK1 | 0.36311301 | 0.96815259 | -0.2997334 | 69.01464677 | 182.4297278 |
| YNL198C | YNL198C | 0.20533969 | 0.96815259 | -0.2987429 | 41.61490267 | 131.1345851 |
| YMR214W | SCJ1 | 0.13343655 | 0.96815259 | -0.2985685 | 98.00894478 | 236.3137396 |
| YOR080W | DIA2 | 0.30448272 | 0.96815259 | -0.2979469 | 34.62087785 | 117.9437853 |
| YGL212W | VAM7 | 0.13922632 | 0.96815259 | -0.2970136 | 11.5476107 | 74.73049039 |
| YHL032C | GUT1 | 0.04137517 | 0.96815259 | -0.2969717 | 105.4186973 | 249.8516486 |
| YKR091W | SRL3 | 0.02095676 | 0.78250728 | -0.2968869 | 93.19467724 | 227.0309806 |
| YDR348C | YDR348C | 0.07833072 | 0.96815259 | -0.2949143 | 87.52541435 | 216.1009923 |
| YCR086W | CSM1 | 0.27047415 | 0.96815259 | -0.2919478 | 77.43960532 | 196.7533904 |
| YDR382W | RPP2B | 0.30300931 | 0.96815259 | -0.290208 | 17.63159964 | 84.86226328 |
| YJR091C | JSN1 | 0.0585501 | 0.96815259 | -0.2888513 | 95.85738901 | 230.5596636 |
| YEL003W | GIM4 | 0.34967488 | 0.96815259 | -0.2871901 | 50.00217647 | 144.7133506 |
| YDR260C | SWM1 | 0.03332001 | 0.93883273 | -0.2870572 | 97.49868213 | 233.3004218 |
| YHR021C | RPS27B | 0.19186523 | 0.96815259 | -0.2855509 | 40.14726036 | 126.034187 |
| YEL013W | VAC8 | 0.26829108 | 0.96815259 | -0.2831047 | 74.07869021 | 188.8996222 |
| YKR023W | YKR023W | 0.01011499 | 0.51706848 | -0.2827808 | 75.5408032 | 191.5693685 |
| YOR008C-A | YOR008C-A | 0.01328116 | 0.63257512 | -0.2824663 | 98.87922696 | 235.0539146 |
| YBR092C | PHO3 | 0.07671826 | 0.96815259 | -0.2820007 | 106.1550852 | 248.5445992 |
| YJL206C | YJL206C | 0.07503243 | 0.96815259 | -0.2806391 | 101.1527965 | 238.9683481 |
| YDR477W | SNF1 | 0.16074348 | 0.96815259 | -0.2800958 | 74.46711692 | 189.0854624 |
| YJR140C | HIR3 | 0.33814362 | 0.96815259 | -0.2785534 | 60.79335839 | 163.2991056 |
| YDR245W | MNN10 | 0.49891889 | 0.96815259 | -0.2782221 | 30.47541014 | 106.6777144 |
| YDR389W | SAC7 | 0.01697733 | 0.73573115 | -0.278165 | 95.30078892 | 227.6076203 |
| YJL062W | LAS21 | 0.45250983 | 0.96815259 | -0.2775706 | 84.7522858 | 207.8215832 |
| YGL066W | SGF73 | 0.4525475 | 0.96815259 | -0.2772895 | 59.25050075 | 160.1943705 |
| YFL001W | DEG1 | 0.02141715 | 0.78602775 | -0.276664 | 56.81588601 | 155.5402608 |
| YKL184W | SPE1 | 0.39100222 | 0.96815259 | -0.2762836 | 0 | 49.47476343 |
| YML063W | RPS1B | 0.43272897 | 0.96815259 | -0.2762122 | 47.19703283 | 137.5141437 |
| YPL183C | YPL183C | 0.43908981 | 0.96815259 | -0.2755496 | 67.79324848 | 175.8204014 |
| YHR130C | YHR130C | 0.05838252 | 0.96815259 | -0.2747048 | 106.3595858 | 247.6196209 |
| YGL009C | LEU1 | 0.08506817 | 0.96815259 | -0.274701 | 106.628432 | 248.1205089 |
| YOR363C | PIP2 | 0.50801604 | 0.96815259 | -0.2744206 | 98.88554047 | 233.6249273 |
| YOR131C | YOR131C | 0.0887941 | 0.96815259 | -0.2732737 | 97.87050829 | 231.5258682 |
| YDR108W | GSG1 | 0.09995884 | 0.96815259 | -0.2727693 | 77.46816645 | 193.372336 |
| YNL056W | OCA2 | 0.0936505 | 0.96815259 | -0.2716469 | 71.78260634 | 182.5641922 |
| YKL009W | MRT4 | 0.21774968 | 0.96815259 | -0.2711285 | 48.99265836 | 139.9537669 |
| YGL020C | GET1 | 0.1243896 | 0.96815259 | -0.2704805 | 100.221504 | 235.4117725 |
| YHR067W | HTD2 | 0.56152035 | 0.96815259 | -0.2701964 | 62.55828573 | 165.0952836 |
| YDL046W | NPC2 | 0.03740042 | 0.96815259 | -0.2699632 | 106.8598014 | 247.7037466 |
| YDR277C | MTH1 | 0.05245363 | 0.96815259 | -0.2694961 | 99.91478373 | 234.6632735 |
| YJL029C | VPS53 | 0.36723119 | 0.96815259 | -0.269198 | 65.67654338 | 170.7340114 |
| YGR163W | GTR2 | 0.60422391 | 0.96815259 | -0.2688376 | 58.87616424 | 157.9824998 |
| YDR253C | MET32 | 0.04671259 | 0.96815259 | -0.2674061 | 95.00130083 | 225.1222781 |
| YKL214C | YRA2 | 0.07176241 | 0.96815259 | -0.2673647 | 103.4907087 | 240.9529454 |
| YCL075W | YCL075W | 0.06529631 | 0.96815259 | -0.267019 | 102.3020645 | 238.6734734 |
| YCR090C | YCR090C | 0.04743547 | 0.96815259 | -0.2667492 | 108.8854355 | 250.9072868 |
| YHR016C | YSC84 | 0.19097826 | 0.96815259 | -0.2637842 | 99.15343144 | 232.2200321 |
| YNL329C | PEX6 | 0.3854895 | 0.96815259 | -0.2634392 | 69.68706952 | 177.1849332 |
| YDL052C | SLC1 | 0.25653934 | 0.96815259 | -0.2628369 | 5.602200565 | 57.51846215 |
| YLR182W | SWI6 | 0.15949178 | 0.96815259 | -0.2628219 | 5.982076702 | 58.22448181 |
| YPL091W | GLR1 | 0.17935705 | 0.96815259 | -0.2616128 | 92.6478251 | 219.6941392 |
| YBR084C-A | RPL19A | 0.10900578 | 0.96815259 | -0.2613393 | 87.59110059 | 210.2111913 |
| YGR055W | MUP1 | 0.47129263 | 0.96815259 | -0.2611294 | 32.61009814 | 107.5994141 |
| YHR138C | YHR138C | 0.4968429 | 0.96815259 | -0.25976 | 87.02324706 | 208.8689767 |
| YHR103W | SBE22 | 0.54794944 | 0.96815259 | -0.2597347 | 88.92184382 | 212.4065127 |
| YDR181C | SAS4 | 0.02764627 | 0.89818524 | -0.258369 | 114.0702779 | 259.0796282 |
| YDL222C | FMP45 | 0.02684681 | 0.88677081 | -0.2577803 | 98.23300324 | 229.4277074 |

| | | | | | | |
|---|---|---|---|---|---|---|
| YBL021C | HAP3 | 0.11465408 | 0.96815259 | -0.2556044 | 79.77785201 | 194.6076069 |
| YGR097W | ASK10 | 0.13074373 | 0.96815259 | -0.2535579 | 101.5392452 | 234.8398303 |
| YDL038C | YDL038C | 0.02432405 | 0.84153481 | -0.2531918 | 110.2362314 | 250.9996105 |
| YGR077C | PEX8 | 0.33466928 | 0.96815259 | -0.2529819 | 77.81698055 | 190.4797197 |
| YNL238W | KEX2 | 0.52126276 | 0.96815259 | -0.2529482 | 44.71474705 | 128.7171768 |
| YDL110C | TMA17 | 0.09647753 | 0.96815259 | -0.2516513 | 102.4089018 | 236.1208686 |
| YDR090C | YDR090C | 0.0309222 | 0.9284317 | -0.2510192 | 103.0912115 | 237.2806122 |
| YML007W | YAP1 | 0.29266672 | 0.96815259 | -0.2507116 | 85.30112595 | 204.035818 |
| YLR444C | YLR444C | 0.4281654 | 0.96815259 | -0.2497786 | 75.39370412 | 185.3851712 |
| YNR001C | CIT1 | 0.09049193 | 0.96815259 | -0.2489311 | 90.98004591 | 214.3117387 |
| YCL026C | YCL026C | 0.12234914 | 0.96815259 | -0.2486342 | 92.85925436 | 217.76447 |
| YBR164C | ARL1 | 0.03261768 | 0.93373536 | -0.2472342 | 102.8604788 | 236.1723597 |
| YJL007C | YJL007C | 0.10095252 | 0.96815259 | -0.2469515 | 94.72555538 | 220.9449863 |
| YKR012C | YKR012C | 0.48144032 | 0.96815259 | -0.2468527 | 80.24030545 | 193.9031737 |
| YDL206W | YDL206W | 0.09289084 | 0.96815259 | -0.2467505 | 105.0579033 | 240.185324 |
| YBR287W | ZSP1 | 0.08078128 | 0.96815259 | -0.2467039 | 112.3844211 | 253.8455421 |
| YDL019C | OSH2 | 0.15193707 | 0.96815259 | -0.2459036 | 107.3600235 | 244.328567 |
| YCL026C-A | FRM2 | 0.4034323 | 0.96815259 | -0.2439819 | 85.70204384 | 203.5786682 |
| YCR006C | YCR006C | 0.05374099 | 0.96815259 | -0.2432896 | 104.3933769 | 238.3258084 |
| YDL194W | SNF3 | 0.09485069 | 0.96815259 | -0.2430093 | 103.4142214 | 236.4488632 |
| YNL015W | PBI2 | 0.10621666 | 0.96815259 | -0.242339 | 100.9605376 | 231.7511784 |
| YMR294W | JNM1 | 0.46622274 | 0.96815259 | -0.241656 | 76.41150563 | 185.8294666 |
| YLL044W | YLL044W | 0.1067383 | 0.96815259 | -0.241551 | 106.7500087 | 242.4110733 |
| YIL123W | SIM1 | 0.12830691 | 0.96815259 | -0.2412175 | 107.7164234 | 244.1543256 |
| YDL219W | DTD1 | 0.07795979 | 0.96815259 | -0.2406201 | 108.4561447 | 245.4273851 |
| YDR265W | PEX10 | 0.1034872 | 0.96815259 | -0.2387751 | 99.8618618 | 229.0632511 |
| YDR400W | URH1 | 0.05619416 | 0.96815259 | -0.2371888 | 111.6788521 | 250.8253168 |
| YOR038C | HIR2 | 0.22786263 | 0.96815259 | -0.2368225 | 100.4784841 | 229.86398 |
| YCR048W | ARE1 | 0.10915475 | 0.96815259 | -0.2365283 | 103.474409 | 235.4005986 |
| YIL152W | YIL152W | 0.15385247 | 0.96815259 | -0.2364766 | 96.5316197 | 222.4386685 |
| YDR014W | RAD61 | 0.04350622 | 0.96815259 | -0.2351478 | 87.97824931 | 206.2432968 |
| YGR235C | YGR235C | 0.50271346 | 0.96815259 | -0.2350719 | 95.12052221 | 219.5545395 |
| YGR202C | PCT1 | 0.17917184 | 0.96815259 | -0.2350195 | 101.7710327 | 231.9525311 |
| YDL186W | YDL186W | 0.57251468 | 0.96815259 | -0.2348929 | 75.77341642 | 183.4279518 |
| YJL079C | PRY1 | 0.15118429 | 0.96815259 | -0.2342471 | 92.56881875 | 214.6463031 |
| YOR196C | LIP5 | 0.29195916 | 0.96815259 | -0.2333315 | 31.92733548 | 101.347806 |
| YDL133W | YDL133W | 0.04436625 | 0.96815259 | -0.232647 | 113.9116015 | 254.177488 |
| YHR039C | MSC7 | 0.1205343 | 0.96815259 | -0.231403 | 115.2283224 | 256.4112454 |
| YER162C | RAD4 | 0.16314306 | 0.96815259 | -0.2304925 | 90.17188794 | 209.5021731 |
| YCL006C | YCL006C | 0.09224252 | 0.96815259 | -0.2298642 | 109.1292703 | 244.7571199 |
| YMR017W | SPO20 | 0.51203098 | 0.96815259 | -0.2281566 | 81.55759766 | 193.0127939 |
| YDR007W | TRP1 | 0.29347213 | 0.96815259 | -0.2267628 | 93.6437386 | 215.311477 |
| YPL166W | ATG29 | 0.20348485 | 0.96815259 | -0.2255788 | 93.81450581 | 215.418041 |
| YDL081C | RPP1A | 0.11436734 | 0.96815259 | -0.2251301 | 49.39206384 | 132.4618708 |
| YDR205W | MSC2 | 0.02851479 | 0.91375017 | -0.2249117 | 103.6073365 | 233.5683753 |
| YJR090C | GRR1 | 0.39100222 | 0.96815259 | -0.224311 | 0 | 40.1679054 |
| YKL163W | PIR3 | 0.07999482 | 0.96815259 | -0.2240806 | 102.6432559 | 231.6209248 |
| YNL094W | APP1 | 0.11135585 | 0.96815259 | -0.2240419 | 109.0600903 | 243.5854338 |
| YGR282C | BGL2 | 0.22687656 | 0.96815259 | -0.2236453 | 112.6457715 | 250.2039743 |
| YCL056C | YCL056C | 0.0293156 | 0.91703766 | -0.2223278 | 106.9777255 | 239.3935638 |
| YNR004W | YNR004W | 0.45703718 | 0.96815259 | -0.2219995 | 69.65367092 | 169.7019219 |
| YBR255W | MTC4 | 0.0618156 | 0.96815259 | -0.2215832 | 105.0767385 | 235.7136849 |
| YKR024C | DBP7 | 0.04349601 | 0.96815259 | -0.2214798 | 0 | 39.66090747 |
| YDL089W | YDL089W | 0.61725087 | 0.96815259 | -0.2211121 | 73.20158925 | 176.1621058 |
| YML017W | PSP2 | 0.15821965 | 0.96815259 | -0.2210209 | 94.85850694 | 216.5495594 |
| YJL128C | PBS2 | 0.20425905 | 0.96815259 | -0.2207318 | 113.5813234 | 251.4276362 |
| YIL079C | AIR1 | 0.20284597 | 0.96815259 | -0.2196809 | 105.6157835 | 236.3787026 |
| YEL031W | SPF1 | 0.45123966 | 0.96815259 | -0.2193721 | 66.61196087 | 163.5567118 |
| YIL032C | YIL032C | 0.27203585 | 0.96815259 | -0.2191697 | 112.1122123 | 248.4070804 |
| YLR006C | SSK1 | 0.25687164 | 0.96815259 | -0.218956 | 81.70018202 | 191.63124 |
| YJL028W | YJL028W | 0.24139283 | 0.96815259 | -0.2178263 | 111.4891169 | 247.0040646 |
| YDR121W | DPB4 | 0.1138471 | 0.96815259 | -0.2177948 | 107.8037394 | 240.1228585 |
| YKR043C | YKR043C | 0.09823743 | 0.96815259 | -0.2175093 | 99.62652776 | 224.816085 |
| YBR270C | BIT2 | 0.1962931 | 0.96815259 | -0.2169587 | 109.3845177 | 242.9222988 |

| | | | | | | |
|---|---|---|---|---|---|---|
| YBL066C | SEF1 | 0.08587327 | 0.96815259 | -0.2168054 | 97.94380513 | 221.5507011 |
| YKR026C | GCN3 | 0.10518321 | 0.96815259 | -0.2167701 | 106.5332268 | 237.5690688 |
| YCR016W | YCR016W | 0.09917667 | 0.96815259 | -0.216207 | 102.7871967 | 230.4795273 |
| YIL073C | SPO22 | 0.07812075 | 0.96815259 | -0.2151117 | 98.83130894 | 222.9031744 |
| YCR008W | SAT4 | 0.08167716 | 0.96815259 | -0.2150271 | 96.62759754 | 218.7766997 |
| YOR078W | BUD21 | 0.25557449 | 0.96815259 | -0.2148718 | 38.65670953 | 110.5966902 |
| YFL014W | HSP12 | 0.23617184 | 0.96815259 | -0.2126086 | 105.6611802 | 235.1969357 |
| YNL234W | YNL234W | 0.34972146 | 0.96815259 | -0.2125702 | 78.65422993 | 184.8050989 |
| YJR075W | HOC1 | 0.15954368 | 0.96815259 | -0.2121764 | 92.6157128 | 210.7815403 |
| YBR280C | SAF1 | 0.12106341 | 0.96815259 | -0.2118079 | 108.400285 | 240.163716 |
| YDL173W | YDL173W | 0.15504998 | 0.96815259 | -0.2116083 | 95.473283 | 216.010964 |
| YCR009C | RVS161 | 0.50202734 | 0.96815259 | -0.2113288 | 44.61161394 | 121.0718966 |
| YKL216W | URA1 | 0.26614376 | 0.96815259 | -0.2109695 | 81.02800387 | 188.9470438 |
| YFL007W | BLM10 | 0.13195658 | 0.96815259 | -0.2101801 | 97.27094144 | 219.1089932 |
| YIL006W | YIA6 | 0.17987666 | 0.96815259 | -0.2092537 | 100.959963 | 225.8254358 |
| YIL043C | CBR1 | 0.30712012 | 0.96815259 | -0.2088462 | 113.8777094 | 249.8522046 |
| YBR283C | SSH1 | 0.22808045 | 0.96815259 | -0.2078599 | 99.93432159 | 223.662384 |
| YCR017C | CWH43 | 0.06192478 | 0.96815259 | -0.207859 | 107.0403297 | 236.9194 |
| YKL075C | YKL075C | 0.26238797 | 0.96815259 | -0.2074572 | 84.96390676 | 195.6610238 |
| YBR162W-A | YSY6 | 0.05510413 | 0.96815259 | -0.2074428 | 107.3651567 | 237.4508742 |
| YDL024C | DIA3 | 0.01990476 | 0.78054746 | -0.2070591 | 110.0981145 | 242.480846 |
| YCR032W | BPH1 | 0.12750881 | 0.96815259 | -0.2065352 | 106.3298933 | 235.3569405 |
| YDR057W | YOS9 | 0.08992848 | 0.96815259 | -0.2054392 | 117.2073104 | 255.4538961 |
| YDL190C | UFD2 | 0.14560009 | 0.96815259 | -0.2050439 | 102.9351877 | 228.7566169 |
| YDR270W | CCC2 | 0.14725511 | 0.96815259 | -0.2039785 | 113.1321749 | 247.5896281 |
| YOR216C | RUD3 | 0.22967331 | 0.96815259 | -0.203839 | 95.3090822 | 214.3133642 |
| YJL204C | RCY1 | 0.54450848 | 0.96815259 | -0.2036636 | 55.63379382 | 140.2625705 |
| YBR258C | SHG1 | 0.08824928 | 0.96815259 | -0.2033335 | 117.602616 | 255.8143256 |
| YPR141C | KAR3 | 0.19677357 | 0.96815259 | -0.2032008 | 0 | 36.38764455 |
| YLR428C | YLR428C | 0.6079603 | 0.96815259 | -0.202797 | 87.89635103 | 200.2973626 |
| YDR058C | TGL2 | 0.10624309 | 0.96815259 | -0.2027417 | 92.95697289 | 209.728715 |
| YOR125C | CAT5 | 0.52204938 | 0.96815259 | -0.202345 | 62.11343992 | 152.11507 |
| YHL008C | YHL008C | 0.14195918 | 0.96815259 | -0.2023282 | 95.97441574 | 215.2840975 |
| YBR272C | HSM3 | 0.12652265 | 0.96815259 | -0.2022422 | 111.7046863 | 244.6155466 |
| YHR025W | THR1 | 0.67130278 | 0.97060928 | -0.2019458 | 28.22799557 | 88.82589775 |
| YOR298C-A | MBF1 | 0.26884639 | 0.96815259 | -0.200166 | 100.4761588 | 223.2954784 |
| YCR106W | RDS1 | 0.09519534 | 0.96815259 | -0.2001641 | 103.5311151 | 228.9945652 |
| YJL178C | ATG27 | 0.11122318 | 0.96815259 | -0.19889 | 85.87790408 | 195.8320458 |
| YPL250C | ICY2 | 0.57857296 | 0.96815259 | -0.1981522 | 83.46196471 | 191.1926827 |
| YOR275C | RIM20 | 0.3089768 | 0.96815259 | -0.1977894 | 94.57116164 | 211.8533637 |
| YMR016C | SOK2 | 0.46250496 | 0.96815259 | -0.1967769 | 81.37161691 | 187.046602 |
| YJL078C | PRY3 | 0.07964084 | 0.96815259 | -0.1965419 | 103.467074 | 228.2264437 |
| YJL190C | RPS22A | 0.29704175 | 0.96815259 | -0.1957556 | 112.4048065 | 244.7601394 |
| YCR102W-A | YCR102W-A | 0.15452192 | 0.96815259 | -0.1943009 | 105.4764737 | 231.5739417 |
| YDL142C | CRD1 | 0.01747752 | 0.73573115 | -0.1941649 | 98.30508776 | 218.1704385 |
| YKL056C | TMA19 | 0.24517163 | 0.96815259 | -0.1941284 | 87.74411848 | 198.4610546 |
| YMR304W | UBP15 | 0.18973064 | 0.96815259 | -0.1939464 | 94.94672128 | 211.865836 |
| YNL069C | RPL16B | 0.42912604 | 0.96815259 | -0.1935879 | 85.8471271 | 194.8251763 |
| YKL124W | SSH4 | 0.15892279 | 0.96815259 | -0.1934862 | 100.1398278 | 221.4718485 |
| YDR305C | HNT2 | 0.49298864 | 0.96815259 | -0.1934454 | 90.1493007 | 202.825915 |
| YDR358W | GGA1 | 0.13355601 | 0.96815259 | -0.1933069 | 111.0974983 | 241.882699 |
| YOR252W | TMA16 | 0.33118012 | 0.96815259 | -0.1924178 | 80.46656348 | 184.5775072 |
| YDR095C | YDR095C | 0.05986453 | 0.96815259 | -0.1921964 | 119.8517199 | 258.0159771 |
| YKL071W | YKL071W | 0.23577463 | 0.96815259 | -0.1921084 | 107.1813679 | 234.3620344 |
| YJL095W | BCK1 | 0.10915344 | 0.96815259 | -0.1914615 | 105.2600198 | 230.6616545 |
| YOR364W | YOR364W | 0.30569168 | 0.96815259 | -0.1910373 | 93.5523777 | 208.7435765 |
| YNL180C | RHO5 | 0.59873634 | 0.96815259 | -0.1910058 | 60.79078334 | 147.6169439 |
| YDR248C | YDR248C | 0.32383556 | 0.96815259 | -0.1901753 | 116.3520372 | 251.1249258 |
| YLR372W | SUR4 | 0.6583275 | 0.96815259 | -0.1899541 | 45.35511245 | 118.631365 |
| YLR415C | YLR415C | 0.08883683 | 0.96815259 | -0.1896935 | 107.7478063 | 234.9863547 |
| YIL023C | YKE4 | 0.22131896 | 0.96815259 | -0.1895416 | 81.95286601 | 186.8353429 |
| YBL037W | APL3 | 0.08022148 | 0.96815259 | -0.1894727 | 118.8189901 | 255.6015381 |
| YDR297W | SUR2 | 0.16552373 | 0.96815259 | -0.1894367 | 116.7740758 | 251.7800311 |
| YGL045W | RIM8 | 0.12338595 | 0.96815259 | -0.1892452 | 98.68856909 | 218.0048988 |

| | | | | | | |
|---|---|---|---|---|---|---|
| YER055C | HIS1 | 0.26284974 | 0.96815259 | -0.1890229 | 92.73836793 | 206.8642086 |
| YOL018C | TLG2 | 0.24668433 | 0.96815259 | -0.1887547 | 81.1229473 | 185.1461155 |
| YPL141C | YPL141C | 0.32351091 | 0.96815259 | -0.1885156 | 99.95525722 | 220.2374196 |
| YCR082W | AHC2 | 0.12572007 | 0.96815259 | -0.1884937 | 117.0882111 | 252.1972289 |
| YDR019C | GCV1 | 0.13526824 | 0.96815259 | -0.1879294 | 102.2314831 | 224.3790336 |
| YHR179W | OYE2 | 0.22111873 | 0.96815259 | -0.1878809 | 102.8660121 | 225.5541375 |
| YKL150W | MCR1 | 0.16295208 | 0.96815259 | -0.1878268 | 109.816384 | 238.5112789 |
| YLR287C | YLR287C | 0.20480677 | 0.96815259 | -0.187538 | 103.4399669 | 226.5635305 |
| YPR051W | MAK3 | 0.23825887 | 0.96815259 | -0.1874713 | 92.4442306 | 206.0376132 |
| YLR390W | ECM19 | 0.46267344 | 0.96815259 | -0.1871531 | 85.03541587 | 192.1585366 |
| YBR050C | REG2 | 0.63044882 | 0.96815259 | -0.1871508 | 91.0030202 | 203.2914562 |
| YDR512C | EMI1 | 0.50810735 | 0.96815259 | -0.1870501 | 69.54542093 | 163.2414937 |
| YOR037W | CYC2 | 0.23018484 | 0.96815259 | -0.1858171 | 92.57027758 | 205.9765445 |
| YKR069W | MET1 | 0.0907437 | 0.96815259 | -0.185253 | 112.0757547 | 242.2655351 |
| YDR071C | PAA1 | 0.12961449 | 0.96815259 | -0.1846074 | 101.0361109 | 221.5540361 |
| YPL086C | ELP3 | 0.32974528 | 0.96815259 | -0.1846 | 83.24311093 | 188.3575692 |
| YLR427W | MAG2 | 0.17163917 | 0.96815259 | -0.1842327 | 102.8629214 | 224.8950843 |
| YLR015W | BRE2 | 0.02948581 | 0.91703766 | -0.1840808 | 79.98738251 | 182.1906117 |
| YKL160W | ELF1 | 0.20472754 | 0.96815259 | -0.1837923 | 91.34792976 | 203.333513 |
| YOR343C | YOR343C | 0.36132207 | 0.96815259 | -0.1837269 | 104.6445174 | 228.1283015 |
| YML029W | USA1 | 0.24605266 | 0.96815259 | -0.1830774 | 108.4846261 | 235.1762225 |
| YJR147W | HMS2 | 0.15156927 | 0.96815259 | -0.1825185 | 99.34338183 | 218.021961 |
| YJL212C | OPT1 | 0.08204828 | 0.96815259 | -0.1821199 | 98.29705185 | 215.9985121 |
| YKL136W | YKL136W | 0.18976857 | 0.96815259 | -0.1819648 | 107.1029476 | 232.3992828 |
| YLR456W | YLR456W | 0.12622325 | 0.96815259 | -0.1817076 | 105.1661817 | 228.7399341 |
| YDL230W | PTP1 | 0.50717923 | 0.96815259 | -0.1812691 | 87.17812592 | 195.1023649 |
| YDL218W | YDL218W | 0.20479742 | 0.96815259 | -0.1805783 | 112.893504 | 242.9540372 |
| YOR302W | YOR302W | 0.21405163 | 0.96815259 | -0.1797 | 101.6952827 | 221.9050285 |
| YNL325C | FIG4 | 0.17706309 | 0.96815259 | -0.1796875 | 90.57030685 | 201.1476977 |
| YPL046C | ELC1 | 0.39914713 | 0.96815259 | -0.179564 | 89.37852905 | 198.9021646 |
| YOR271C | FSF1 | 0.23078078 | 0.96815259 | -0.1795184 | 90.33596455 | 200.6802274 |
| YMR020W | FMS1 | 0.57810323 | 0.96815259 | -0.1793431 | 81.79440161 | 184.713438 |
| YER016W | BIM1 | 0.32598677 | 0.96815259 | -0.1793401 | 95.45166479 | 210.1923 |
| YIL098C | FMC1 | 0.18419495 | 0.96815259 | -0.1791988 | 91.14817171 | 202.1382678 |
| YOL151W | GRE2 | 0.24310497 | 0.96815259 | -0.1788937 | 94.78870541 | 208.8755188 |
| YJL196C | ELO1 | 0.21119038 | 0.96815259 | -0.1786499 | 111.9383088 | 240.8266753 |
| YDL172C | YDL172C | 0.13820516 | 0.96815259 | -0.1785558 | 110.1357797 | 237.4469716 |
| YDR147W | EKI1 | 0.15521994 | 0.96815259 | -0.1785393 | 112.191777 | 241.2797384 |
| YKR028W | SAP190 | 0.47871495 | 0.96815259 | -0.1781588 | 62.3672018 | 148.2574023 |
| YDL204W | RTN2 | 0.14332034 | 0.96815259 | -0.1777763 | 109.6625047 | 236.4244334 |
| YBL060W | YBL060W | 0.10940699 | 0.96815259 | -0.1777026 | 104.9990817 | 227.7110063 |
| YEL057C | YEL057C | 0.57032171 | 0.96815259 | -0.1775875 | 19.17214203 | 67.56911803 |
| YBR221C | PDB1 | 0.1506748 | 0.96815259 | -0.177579 | 104.4912034 | 226.741355 |
| YCL076W | YCL076W | 0.13112935 | 0.96815259 | -0.177207 | 113.1058739 | 242.7465322 |
| YLL014W | YLL014W | 0.25774791 | 0.96815259 | -0.1770324 | 100.3635767 | 218.9428613 |
| YIL064W | YIL064W | 0.28623335 | 0.96815259 | -0.1767881 | 101.5936115 | 221.1939079 |
| YKL121W | YKL121W | 0.13340913 | 0.96815259 | -0.1767703 | 85.25574434 | 190.7103153 |
| YOL104C | NDJ1 | 0.30492703 | 0.96815259 | -0.1765717 | 96.35358479 | 211.3792011 |
| YCL033C | YCL033C | 0.12290947 | 0.96815259 | -0.1757833 | 108.1370637 | 233.2216243 |
| YDR072C | IPT1 | 0.24752235 | 0.96815259 | -0.1754536 | 100.1569105 | 218.2745782 |
| YGL213C | SKI8 | 0.32111006 | 0.96815259 | -0.1754011 | 99.23547615 | 216.5461171 |
| YBR245C | ISW1 | 0.15428052 | 0.96815259 | -0.1753886 | 114.7946583 | 245.5715513 |
| YMR221C | FMP42 | 0.24453777 | 0.96815259 | -0.1751798 | 105.7943871 | 228.7429928 |
| YCL034W | LSB5 | 0.16480354 | 0.96815259 | -0.1750253 | 116.9539018 | 249.5348522 |
| YIL042C | PKP1 | 0.28570961 | 0.96815259 | -0.1748893 | 100.8324765 | 219.4338756 |
| YPL179W | PPQ1 | 0.13594457 | 0.96815259 | -0.1747969 | 79.82923676 | 180.2330756 |
| YIL155C | GUT2 | 0.23637966 | 0.96815259 | -0.1746839 | 99.01189065 | 216.0005713 |
| YKL008C | LAC1 | 0.19357826 | 0.96815259 | -0.1746293 | 105.4944802 | 228.0848918 |
| YOL027C | MDM38 | 0.20912642 | 0.96815259 | -0.1741255 | 104.4184 | 225.987112 |
| YDL171C | GLT1 | 0.13269101 | 0.96815259 | -0.1740564 | 101.4936698 | 220.5182787 |
| YJL133W | MRS3 | 0.13949122 | 0.96815259 | -0.1733937 | 111.9715668 | 239.947486 |
| YKL197C | PEX1 | 0.4110401 | 0.96815259 | -0.1731643 | 105.7981167 | 228.3890226 |
| YMR137C | PSO2 | 0.23507626 | 0.96815259 | -0.1730776 | 107.1155277 | 230.8313034 |
| YJL057C | IKS1 | 0.24901929 | 0.96815259 | -0.1730349 | 110.4442913 | 237.0339055 |

| | | | | | | |
|---|---|---|---|---|---|---|
| YDR247W | VHS1 | 0.30702634 | 0.96815259 | -0.1723369 | 101.3270798 | 219.8995716 |
| YKL096W-A | CWP2 | 0.15850381 | 0.96815259 | -0.1722545 | 111.4797807 | 238.8259838 |
| YKL025C | PAN3 | 0.21143252 | 0.96815259 | -0.1720575 | 103.0413791 | 223.0477774 |
| YDL091C | UBX3 | 0.09935556 | 0.96815259 | -0.172041 | 113.3765934 | 242.3265017 |
| YDR414C | ERD1 | 0.14781022 | 0.96815259 | -0.1713321 | 109.4465819 | 234.8676154 |
| YJR049C | UTR1 | 0.21483598 | 0.96815259 | -0.1712946 | 108.4630328 | 233.0259714 |
| YCR088W | ABP1 | 0.08836335 | 0.96815259 | -0.1711721 | 123.3817192 | 260.8367698 |
| YIL161W | YIL161W | 0.29629023 | 0.96815259 | -0.1710017 | 115.3901021 | 245.8968531 |
| YOR306C | MCH5 | 0.51172898 | 0.96815259 | -0.1709569 | 68.12617251 | 157.7118522 |
| YKR020W | VPS51 | 0.0573398 | 0.96815259 | -0.1709361 | 75.64284871 | 171.7314594 |
| YDL133C-A | RPL41B | 0.20077567 | 0.96815259 | -0.1708754 | 92.10021609 | 202.4239439 |
| YNL078W | NIS1 | 0.16422999 | 0.96815259 | -0.1708737 | 88.06549795 | 194.8963445 |
| YKL074C | MUD2 | 0.01629535 | 0.72492154 | -0.1706268 | 80.79535254 | 181.2887338 |
| YDL157C | YDL157C | 0.21840853 | 0.96815259 | -0.1704881 | 109.5798978 | 234.9652021 |
| YDR213W | UPC2 | 0.1839391 | 0.96815259 | -0.1702453 | 113.6224271 | 242.4635838 |
| YGL139W | FLC3 | 0.11632426 | 0.96815259 | -0.1701935 | 105.762792 | 227.7911394 |
| YCL045C | YCL045C | 0.29953417 | 0.96815259 | -0.1699887 | 114.3157221 | 243.7110661 |
| YIL077C | YIL077C | 0.21052503 | 0.96815259 | -0.1699592 | 96.05948316 | 209.6463982 |
| YCL047C | YCL047C | 0.17622111 | 0.96815259 | -0.1698798 | 109.7261342 | 235.1290852 |
| YDL170W | UGA3 | 0.36161707 | 0.96815259 | -0.1694182 | 99.16880436 | 215.3503703 |
| YDR374C | YDR374C | 0.30040434 | 0.96815259 | -0.1690655 | 101.0153182 | 218.7321233 |
| YER078C | YER078C | 0.28288607 | 0.96815259 | -0.1689638 | 100.3052989 | 217.389276 |
| YDR067C | OCA6 | 0.33144367 | 0.96815259 | -0.1689069 | 93.36421117 | 204.4295799 |
| YIR035C | YIR035C | 0.35234057 | 0.96815259 | -0.1682736 | 103.8511858 | 223.8809829 |
| YKL188C | PXA2 | 0.27526337 | 0.96815259 | -0.1678008 | 104.047079 | 224.1617832 |
| YNL052W | COX5A | 0.33637523 | 0.96815259 | -0.1677361 | 90.80516195 | 199.4456929 |
| YKR101W | SIR1 | 0.21063358 | 0.96815259 | -0.1676633 | 112.5749223 | 240.0469594 |
| YKL040C | NFU1 | 0.15800181 | 0.96815259 | -0.1669573 | 104.7332266 | 225.2908386 |
| YLR036C | YLR036C | 0.59572984 | 0.96815259 | -0.1669541 | 90.73978972 | 199.1836965 |
| YIL153W | RRD1 | 0.17354175 | 0.96815259 | -0.1668632 | 95.80653053 | 208.6200725 |
| YPR065W | ROX1 | 0.10686595 | 0.96815259 | -0.1666788 | 97.1342646 | 211.06412 |
| YCL001W | RER1 | 0.20638035 | 0.96815259 | -0.1660676 | 107.4687187 | 230.2349357 |
| YNL296W | YNL296W | 0.51273689 | 0.96815259 | -0.1660565 | 83.11583975 | 184.7994918 |
| YCR034W | FEN1 | 0.33948641 | 0.96815259 | -0.1659665 | 24.71784242 | 75.83435503 |
| YOR268C | YOR268C | 0.2131701 | 0.96815259 | -0.1659128 | 100.5569348 | 217.3123902 |
| YHR001W-A | QCR10 | 0.2934625 | 0.96815259 | -0.1657684 | 100.3779885 | 216.9526736 |
| YMR202W | ERG2 | 0.26723314 | 0.96815259 | -0.1657508 | 0 | 29.68138782 |
| YBL051C | PIN4 | 0.19468349 | 0.96815259 | -0.1655247 | 81.68425076 | 182.0334413 |
| YBR084W | MIS1 | 0.18004171 | 0.96815259 | -0.1652408 | 110.7854037 | 236.2745768 |
| YCL014W | BUD3 | 0.71325951 | 0.97135148 | -0.165073 | 77.13150648 | 173.458818 |
| YLL013C | PUF3 | 0.13710408 | 0.96815259 | -0.1647485 | 110.0312052 | 234.7793717 |
| YKL001C | MET14 | 0.30705494 | 0.96815259 | -0.1645676 | 103.9324497 | 223.3689604 |
| YKL066W | YKL066W | 0.21710613 | 0.96815259 | -0.1645611 | 107.0944665 | 229.2669397 |
| YKL132C | RMA1 | 0.23879499 | 0.96815259 | -0.1645489 | 107.6370045 | 230.2769292 |
| YMR160W | YMR160W | 0.34326978 | 0.96815259 | -0.1643312 | 92.38216686 | 201.778075 |
| YDL160C | DHH1 | 0.15343673 | 0.96815259 | -0.1642286 | 121.5133439 | 256.1076823 |
| YCL039W | GID7 | 0.22592852 | 0.96815259 | -0.1639482 | 109.3550279 | 233.3745536 |
| YDL187C | YDL187C | 0.24309409 | 0.96815259 | -0.1636777 | 110.6698833 | 235.7791515 |
| YMR140W | SIP5 | 0.30888322 | 0.96815259 | -0.1634803 | 108.2879048 | 231.2999194 |
| YJL185C | YJL185C | 0.29562942 | 0.96815259 | -0.1633661 | 116.808573 | 247.1758649 |
| YOR344C | TYE7 | 0.30053058 | 0.96815259 | -0.1629879 | 89.56017748 | 196.2727435 |
| YDR332W | IRC3 | 0.17635878 | 0.96815259 | -0.1623443 | 106.6361053 | 228.0148358 |
| YKL222C | YKL222C | 0.15807777 | 0.96815259 | -0.1623324 | 99.40430876 | 214.5208592 |
| YKL047W | YKL047W | 0.2315271 | 0.96815259 | -0.1622824 | 109.9050388 | 234.1023736 |
| YIL135C | VHS2 | 0.1838907 | 0.96815259 | -0.1621863 | 114.2544791 | 242.1996154 |
| YJR035W | RAD26 | 0.30798508 | 0.96815259 | -0.1618494 | 105.4238527 | 225.6645977 |
| YDL226C | GCS1 | 0.33042998 | 0.96815259 | -0.1616082 | 84.57459637 | 186.7244203 |
| YJL192C | SOP4 | 0.16976713 | 0.96815259 | -0.1603811 | 99.07848675 | 213.5635632 |
| YJL210W | PEX2 | 0.35631832 | 0.96815259 | -0.1598232 | 101.1035095 | 217.2416058 |
| YNL214W | PEX17 | 0.45373017 | 0.96815259 | -0.1597863 | 103.4085217 | 221.5353036 |
| YDR371W | CTS2 | 0.18472645 | 0.96815259 | -0.1596433 | 115.5044688 | 244.0762551 |
| YML013C-A | YML013C-A | 0.49294323 | 0.96815259 | -0.1595679 | 57.04196194 | 134.9933595 |
| YDR422C | SIP1 | 0.12884516 | 0.96815259 | -0.1591337 | 114.2763561 | 241.6937899 |
| YLL005C | SPO75 | 0.23174491 | 0.96815259 | -0.1587688 | 104.811887 | 223.97125 |

| | | | | | | |
|---|---|---|---|---|---|---|
| YBR131W | CCZ1 | 0.72804792 | 0.97135148 | -0.1580688 | 30.86393082 | 85.88640787 |
| YML048W-A | YML048W-A | 0.4303521 | 0.96815259 | -0.1579582 | 104.7524164 | 223.7151406 |
| YKR013W | PRY2 | 0.23790603 | 0.96815259 | -0.1578835 | 104.9738292 | 224.1148387 |
| YNL230C | ELA1 | 0.09079779 | 0.96815259 | -0.1576286 | 101.7853806 | 218.120735 |
| YPR050C | YPR050C | 0.37510133 | 0.96815259 | -0.1575197 | 77.22494101 | 172.2805476 |
| YJL099W | CHS6 | 0.20814156 | 0.96815259 | -0.1574277 | 102.2864123 | 219.0195036 |
| YNL196C | YNL196C | 0.34359215 | 0.96815259 | -0.1573582 | 108.4638519 | 230.5318721 |
| YDR206W | EBS1 | 0.12477152 | 0.96815259 | -0.157081 | 115.3942152 | 243.4117128 |
| YNL201C | PSY2 | 0.2499067 | 0.96815259 | -0.1570229 | 90.57898765 | 197.1052931 |
| YCR011C | ADP1 | 0.12238596 | 0.96815259 | -0.1565822 | 114.798238 | 242.2105242 |
| YNL127W | FAR11 | 0.31272716 | 0.96815259 | -0.1558909 | 111.6492616 | 236.2119033 |
| YCR076C | YCR076C | 0.14284241 | 0.96815259 | -0.1558749 | 117.9098082 | 247.8889063 |
| YDL200C | MGT1 | 0.18725862 | 0.96815259 | -0.1556344 | 118.085356 | 248.1733436 |
| YDR085C | AFR1 | 0.16932331 | 0.96815259 | -0.1550379 | 118.0221903 | 247.94869 |
| YLL015W | BPT1 | 0.24465227 | 0.96815259 | -0.1550037 | 110.972819 | 234.7910515 |
| YOR185C | GSP2 | 0.41875108 | 0.96815259 | -0.1548362 | 102.5012192 | 218.9561823 |
| YGR045C | YGR045C | 0.13983687 | 0.96815259 | -0.1548032 | 92.91931448 | 201.0739914 |
| YIL124W | AYR1 | 0.249842 | 0.96815259 | -0.1547407 | 105.1761548 | 223.9295217 |
| YJL112W | MDV1 | 0.31803067 | 0.96815259 | -0.1541739 | 109.6183065 | 232.1154273 |
| YIR032C | DAL3 | 0.26865392 | 0.96815259 | -0.1541404 | 115.6407179 | 243.3450225 |
| YDL065C | PEX19 | 0.70730899 | 0.97135148 | -0.1538794 | 90.21019248 | 195.8543424 |
| YKL020C | SPT23 | 0.19284463 | 0.96815259 | -0.1538736 | 110.0140794 | 232.8000168 |
| YDR148C | KGD2 | 0.00937598 | 0.49704272 | -0.1538282 | 86.86855733 | 189.6109302 |
| YHR150W | PEX28 | 0.33410646 | 0.96815259 | -0.1538013 | 99.23453334 | 212.6764369 |
| YOL050C | :::GAL11 | 0.11217501 | 0.96815259 | -0.1537354 | 85.22989121 | 186.5371573 |
| YKR093W | PTR2 | 0.04036349 | 0.96815259 | -0.153302 | 91.03301005 | 197.286017 |
| YCR073C | SSK22 | 0.08166951 | 0.96815259 | -0.1524685 | 108.3498053 | 229.4434948 |
| YDR312W | SSF2 | 0.29471167 | 0.96815259 | -0.1523771 | 113.37538 | 238.8029817 |
| YDR313C | PIB1 | 0.27737559 | 0.96815259 | -0.152139 | 94.70828904 | 203.9344611 |
| YKR103W | NFT1 | 0.25932321 | 0.96815259 | -0.1511082 | 111.9806911 | 235.9737855 |
| YKL084W | HOT13 | 0.32675578 | 0.96815259 | -0.1508444 | 111.83016 | 235.6457131 |
| YNL093W | YPT53 | 0.26075967 | 0.96815259 | -0.1506322 | 93.60822479 | 201.6123357 |
| YLR364W | YLR364W | 0.65460978 | 0.96815259 | -0.1500504 | 77.42300919 | 171.3125321 |
| YDR149C | YDR149C | 0.26799479 | 0.96815259 | -0.1500314 | 97.99150625 | 209.6823301 |
| YDR006C | SOK1 | 0.27374768 | 0.96815259 | -0.1497696 | 121.2666907 | 253.0583198 |
| YPR070W | MED1 | 0.53528574 | 0.96815259 | -0.1495081 | 5.747367361 | 37.49522565 |
| YHR046C | INM1 | 0.31503795 | 0.96815259 | -0.1489895 | 116.602485 | 244.2169363 |
| YDR022C | CIS1 | 0.20053368 | 0.96815259 | -0.1489423 | 109.4586884 | 230.8808094 |
| YOL155C | HPF1 | 0.35783214 | 0.96815259 | -0.1489076 | 94.57574847 | 203.108539 |
| YKL183W | LOT5 | 0.24073952 | 0.96815259 | -0.1489051 | 101.5635624 | 216.1447744 |
| YJL207C | LAA1 | 0.25298137 | 0.96815259 | -0.148905 | 106.6513238 | 225.63662 |
| YGR027C | RPS25A | 0.63726818 | 0.96815259 | -0.1488357 | 78.1168357 | 172.3894386 |
| YKL187C | YKL187C | 0.25446431 | 0.96815259 | -0.1487312 | 108.2854671 | 228.654205 |
| YDR387C | YDR387C | 0.21573267 | 0.96815259 | -0.1486808 | 114.3142868 | 239.8927336 |
| YGL236C | MTO1 | 0.6175093 | 0.96815259 | -0.1486375 | 119.0172246 | 248.6589227 |
| YIL138C | TPM2 | 0.23625138 | 0.96815259 | -0.1486133 | 115.5231995 | 242.1360305 |
| YJR135C | MCM22 | 0.18141188 | 0.96815259 | -0.1485024 | 108.8838051 | 229.7295133 |
| YHR097C | YHR097C | 0.40079915 | 0.96815259 | -0.1484279 | 115.7471485 | 242.5206302 |
| YOR349W | CIN1 | 0.46664953 | 0.96815259 | -0.1482974 | 106.5033198 | 225.2516989 |
| YKR080W | MTD1 | 0.19577493 | 0.96815259 | -0.1479035 | 103.5472532 | 219.6662385 |
| YJR087W | YJR087W | 0.69034462 | 0.97135148 | -0.1478454 | 74.82921777 | 166.0786254 |
| YJL131C | YJL131C | 0.24501521 | 0.96815259 | -0.1474899 | 112.0264425 | 235.4112084 |
| YDR221W | GTB1 | 0.23340609 | 0.96815259 | -0.1474721 | 113.5229162 | 238.1998781 |
| YKL220C | FRE2 | 0.24958392 | 0.96815259 | -0.1470264 | 97.25848349 | 207.776665 |
| YHL040C | ARN1 | 0.2994267 | 0.96815259 | -0.1467636 | 109.4733676 | 230.5180572 |
| YLR191W | PEX13 | 0.40235131 | 0.96815259 | -0.1466974 | 94.4550073 | 202.487494 |
| YKR067W | GPT2 | 0.22750663 | 0.96815259 | -0.146552 | 108.6738881 | 228.9886292 |
| YKR059W | TIF1 | 0.19861434 | 0.96815259 | -0.1465433 | 109.6377394 | 230.7852644 |
| YDR415C | YDR415C | 0.21258139 | 0.96815259 | -0.1464013 | 114.993147 | 240.7510365 |
| YIL137C | TMA108 | 0.38445021 | 0.96815259 | -0.1461989 | 100.3018097 | 213.3061963 |
| YFR033C | QCR6 | 0.34249251 | 0.96815259 | -0.1461934 | 110.78344 | 232.8600528 |
| YDR273W | DON1 | 0.17530903 | 0.96815259 | -0.1461139 | 107.6098813 | 226.9251263 |
| YNL320W | YNL320W | 0.26370448 | 0.96815259 | -0.146056 | 103.0917358 | 218.4855831 |
| YIL159W | BNR1 | 0.35819893 | 0.96815259 | -0.1457793 | 119.3312015 | 248.7328459 |

| | | | | | | |
|---|---|---|---|---|---|---|
| YDL076C | RXT3 | 0.17399479 | 0.96815259 | -0.1453792 | 123.1424741 | 255.7716373 |
| YDR003W | RCR2 | 0.18047747 | 0.96815259 | -0.1452431 | 121.0273329 | 251.801196 |
| YHL031C | GOS1 | 0.33410362 | 0.96815259 | -0.1451967 | 119.9481248 | 249.7794726 |
| YGR143W | SKN1 | 0.47954351 | 0.96815259 | -0.1450559 | 111.2050108 | 233.4428501 |
| YKL114C | APN1 | 0.3616894 | 0.96815259 | -0.1448706 | 105.7341783 | 223.2031232 |
| YKL067W | YNK1 | 0.26644082 | 0.96815259 | -0.1447347 | 108.674496 | 228.6643247 |
| YDR256C | CTA1 | 0.36988222 | 0.96815259 | -0.1444547 | 105.3412658 | 222.3956194 |
| YKL140W | TGL1 | 0.14431266 | 0.96815259 | -0.1443876 | 98.23304716 | 209.122295 |
| YPR201W | ARR3 | 0.42331785 | 0.96815259 | -0.1442023 | 93.3208616 | 199.9248032 |
| YHR032W | YHR032W | 0.33429839 | 0.96815259 | -0.1441908 | 119.5919971 | 248.9349519 |
| YJR100C | YJR100C | 0.38133455 | 0.96815259 | -0.1440018 | 100.1117222 | 212.5581186 |
| YKL098W | MTC2 | 0.38103502 | 0.96815259 | -0.1438472 | 94.33979026 | 201.7621622 |
| YBR126C | TPS1 | 0.67326736 | 0.97060928 | -0.143326 | 84.23708043 | 182.8208997 |
| YCR100C | YCR100C | 0.29102555 | 0.96815259 | -0.1426634 | 110.8709569 | 232.3912088 |
| YGR227W | DIE2 | 0.26448681 | 0.96815259 | -0.1423997 | 111.8926089 | 234.2499981 |
| YER124C | DSE1 | 0.46508102 | 0.96815259 | -0.1423598 | 106.0529272 | 223.3481692 |
| YBR301W | DAN3 | 0.3201568 | 0.96815259 | -0.1423464 | 117.0910897 | 243.9388895 |
| YLR090W | XDJ1 | 0.31586408 | 0.96815259 | -0.1417925 | 111.728115 | 233.8343892 |
| YBR250W | YBR250W | 0.16283577 | 0.96815259 | -0.1417296 | 112.7263127 | 235.6853947 |
| YOR225W | YOR225W | 0.29085778 | 0.96815259 | -0.1415932 | 103.0540951 | 217.6161871 |
| YKR066C | CCP1 | 0.26588885 | 0.96815259 | -0.1415016 | 106.0044889 | 223.1041355 |
| YDR001C | NTH1 | 0.17093178 | 0.96815259 | -0.1414989 | 118.2993691 | 246.0413412 |
| YGR268C | HUA1 | 0.42876811 | 0.96815259 | -0.141291 | 128.3739865 | 264.7996044 |
| YCR007C | YCR007C | 0.25035043 | 0.96815259 | -0.14128 | 111.5521923 | 233.4144108 |
| YJR107W | YJR107W | 0.2832019 | 0.96815259 | -0.1409297 | 113.4287165 | 236.8525762 |
| YJL138C | TIF2 | 0.24015125 | 0.96815259 | -0.1405387 | 91.13619198 | 195.1929604 |
| YML057W | CMP2 | 0.08390812 | 0.96815259 | -0.1404293 | 98.08815368 | 208.1431666 |
| YKL179C | COY1 | 0.2053419 | 0.96815259 | -0.1399985 | 108.1062922 | 226.7561453 |
| YLR361C | DCR2 | 0.16590387 | 0.96815259 | -0.1396843 | 86.11918315 | 185.6800916 |
| YDL112W | TRM3 | 0.24083687 | 0.96815259 | -0.1393067 | 115.9772189 | 241.3164947 |
| YJR121W | ATP2 | 0.1467637 | 0.96815259 | -0.1392222 | 107.9270618 | 226.2827666 |
| YBL009W | ALK2 | 0.26962001 | 0.96815259 | -0.1390954 | 112.6802239 | 235.1276919 |
| YLR384C | IKI3 | 0.40294567 | 0.96815259 | -0.138953 | 86.31988675 | 185.9235752 |
| YHR050W | SMF2 | 0.36366137 | 0.96815259 | -0.1388607 | 116.5029997 | 242.2175451 |
| YBL088C | TEL1 | 0.30320551 | 0.96815259 | -0.1387566 | 112.7057562 | 235.1146646 |
| YML055W | SPC2 | 0.70534842 | 0.97135148 | -0.1382554 | 85.34909428 | 183.9875009 |
| YKR045C | YKR045C | 0.36166773 | 0.96815259 | -0.1381135 | 110.8612649 | 231.5583535 |
| YHR092C | HXT4 | 0.36517495 | 0.96815259 | -0.1379723 | 120.4881119 | 249.4932003 |
| YDL054C | MCH1 | 0.24221107 | 0.96815259 | -0.1377057 | 105.9143606 | 222.2562371 |
| YHR005C | GPA1 | 0.29810043 | 0.96815259 | -0.1371347 | 100.9227152 | 212.8414368 |
| YOL090W | MSH2 | 0.34385543 | 0.96815259 | -0.1368914 | 85.06731614 | 183.2175549 |
| YGL046W | YGL046W | 0.08051214 | 0.96815259 | -0.1368906 | 98.56667412 | 208.4022249 |
| YHL037C | YHL037C | 0.27503259 | 0.96815259 | -0.1368556 | 100.7073042 | 212.3895779 |
| YJR061W | YJR061W | 0.41098453 | 0.96815259 | -0.1361222 | 119.3766651 | 247.0883583 |
| YKL200C | YKL200C | 0.39889938 | 0.96815259 | -0.1360545 | 107.6189229 | 225.1406338 |
| YJR060W | CBF1 | 0.28676566 | 0.96815259 | -0.1359984 | 109.060981 | 227.8209351 |
| YKR050W | TRK2 | 0.18222663 | 0.96815259 | -0.1355886 | 104.7598165 | 219.7231811 |
| YDR134C | YDR134C | 0.26129574 | 0.96815259 | -0.1354218 | 113.1587707 | 235.3626412 |
| YKL146W | AVT3 | 0.14802693 | 0.96815259 | -0.1353991 | 105.0005039 | 220.138277 |
| YDR293C | SSD1 | 0.71552034 | 0.97135148 | -0.1348808 | 73.8767692 | 161.9801117 |
| YJR084W | CSN12 | 0.3615763 | 0.96815259 | -0.134675 | 102.3552844 | 215.0736048 |
| YBR275C | RIF1 | 0.31909431 | 0.96815259 | -0.134433 | 97.2379881 | 205.4832966 |
| YDL099W | BUG1 | 0.22713938 | 0.96815259 | -0.1344296 | 107.494393 | 224.6173408 |
| YJL077C | ICS3 | 0.40308071 | 0.96815259 | -0.1342956 | 117.1354894 | 242.5800472 |
| YFL013C | IES1 | 0.33249908 | 0.96815259 | -0.1341389 | 5.469958535 | 34.22548242 |
| YBR262C | FMP51 | 0.31421418 | 0.96815259 | -0.1340129 | 111.7785834 | 232.5354302 |
| YPR069C | SPE3 | 0.74170962 | 0.97135148 | -0.1338886 | 27.07316725 | 74.48424095 |
| YNL162W | RPL42A | 0.14865824 | 0.96815259 | -0.1335324 | 91.84564039 | 195.261903 |
| YIL050W | PCL7 | 0.29629118 | 0.96815259 | -0.1333137 | 111.9012846 | 232.6391388 |
| YIL145C | PAN6 | 0.3726465 | 0.96815259 | -0.1332073 | 101.8634891 | 213.8932713 |
| YIL157C | FMP35 | 0.22309134 | 0.96815259 | -0.1331167 | 110.3768083 | 229.7597541 |
| YKR051W | YKR051W | 0.25466814 | 0.96815259 | -0.1328012 | 109.2485444 | 227.5983362 |
| YPL161C | BEM4 | 0.36990388 | 0.96815259 | -0.1327576 | 85.25885961 | 182.8346629 |
| YCR022C | YCR022C | 0.29884222 | 0.96815259 | -0.1326729 | 120.6660962 | 248.8762829 |

| | | | | | | |
|---|---|---|---|---|---|---|
| YKR046C | PET10 | 0.32029178 | 0.96815259 | -0.1325614 | 110.2716314 | 229.4640934 |
| YIR027C | DAL1 | 0.39552678 | 0.96815259 | -0.1323851 | 111.4533406 | 231.6371509 |
| YIL154C | IMP2' | 0.32235098 | 0.96815259 | -0.1321986 | 92.63754106 | 196.5004507 |
| YHL030W | ECM29 | 0.44811297 | 0.96815259 | -0.1317422 | 104.1308416 | 217.8609518 |
| YJR069C | HAM1 | 0.36499504 | 0.96815259 | -0.1316591 | 104.878708 | 219.2413265 |
| YNL229C | URE2 | 0.39922873 | 0.96815259 | -0.1316434 | 113.6439357 | 235.5911779 |
| YCR095C | OCA4 | 0.32626871 | 0.96815259 | -0.131609 | 128.4130284 | 263.1386667 |
| YGL152C | YGL152C | 0.23349455 | 0.96815259 | -0.1315525 | 104.0698025 | 217.7131153 |
| YLL026W | HSP104 | 0.30074413 | 0.96815259 | -0.1315524 | 112.6849941 | 233.785841 |
| YGR068C | YGR068C | 0.37166826 | 0.96815259 | -0.131443 | 99.27699831 | 208.7519012 |
| YBL061C | SKT5 | 0.23599651 | 0.96815259 | -0.1314011 | 104.5934591 | 218.6629527 |
| YNL028W | YNL028W | 0.40465379 | 0.96815259 | -0.1305448 | 118.2459208 | 243.9800521 |
| YLL012W | YEH1 | 0.2858207 | 0.96815259 | -0.1302935 | 106.4663157 | 221.9586696 |
| YJR110W | YMR1 | 0.178361 | 0.96815259 | -0.1302457 | 98.216429 | 206.5588797 |
| YHR104W | GRE3 | 0.37052824 | 0.96815259 | -0.1298791 | 100.1028766 | 210.0126324 |
| YGL208W | SIP2 | 0.00230933 | 0.14800383 | -0.1298772 | 91.46114269 | 193.8900188 |
| YKR061W | KTR2 | 0.25444529 | 0.96815259 | -0.1298618 | 110.2295465 | 228.9021551 |
| YKL159C | RCN1 | 0.21028056 | 0.96815259 | -0.1294786 | 99.59141846 | 208.986734 |
| YOR339C | UBC11 | 0.49770743 | 0.96815259 | -0.1294089 | 92.26110718 | 195.2986081 |
| YOR297C | TIM18 | 0.49857559 | 0.96815259 | -0.1293137 | 89.92574392 | 190.9246333 |
| YOL052C | SPE2 | 0.53656002 | 0.96815259 | -0.1292358 | 10.74146788 | 43.18216694 |
| YDL241W | YDL241W | 0.28073709 | 0.96815259 | -0.1290601 | 105.5402479 | 220.0100958 |
| YKR030W | GMH1 | 0.12069689 | 0.96815259 | -0.1290454 | 90.08486914 | 191.1734597 |
| YDR191W | HST4 | 0.22695651 | 0.96815259 | -0.1288281 | 124.9576766 | 256.194277 |
| YCR037C | PHO87 | 0.30192623 | 0.96815259 | -0.1287204 | 122.9623578 | 252.4524639 |
| YIR007W | YIR007W | 0.34501482 | 0.96815259 | -0.1286738 | 105.8024399 | 220.4300752 |
| YDL023C | SRF4 | 0.13306036 | 0.96815259 | -0.1285296 | 110.9647134 | 230.0351383 |
| YKR018C | YKR018C | 0.10431429 | 0.96815259 | -0.1284254 | 101.5293066 | 212.413512 |
| YMR103C | YMR103C | 0.2516503 | 0.96815259 | -0.1283938 | 97.59534475 | 205.0685337 |
| YDR420W | HKR1 | 0.19029758 | 0.96815259 | -0.1283766 | 107.6422157 | 223.8091869 |
| YGR260W | TNA1 | 0.5149676 | 0.96815259 | -0.1280276 | 115.8228224 | 239.008687 |
| YJL214W | HXT8 | 0.29965282 | 0.96815259 | -0.1278508 | 116.6929474 | 240.6003419 |
| YKL079W | SMY1 | 0.46048768 | 0.96815259 | -0.1273576 | 106.603236 | 221.6883762 |
| YHR155W | YSP1 | 0.55199969 | 0.96815259 | -0.1271798 | 118.836946 | 244.4800937 |
| YHR095W | YHR095W | 0.30972901 | 0.96815259 | -0.1271732 | 114.2818133 | 235.9807242 |
| YOL137W | BSC6 | 0.42682994 | 0.96815259 | -0.1268209 | 100.0913113 | 209.4434174 |
| YBR086C | IST2 | 0.50405513 | 0.96815259 | -0.1266988 | 89.49921471 | 189.660634 |
| YPL101W | ELP4 | 0.55265291 | 0.96815259 | -0.1264973 | 81.84335852 | 175.3415576 |
| YOR342C | YOR342C | 0.38879973 | 0.96815259 | -0.1264134 | 110.0127255 | 227.880126 |
| YKL115C | YKL115C | 0.12462911 | 0.96815259 | -0.126121 | 94.56523745 | 199.0084882 |
| YLL017W | YLL017W | 0.27636219 | 0.96815259 | -0.1260851 | 108.8676043 | 225.6849644 |
| YDL240W | LRG1 | 0.27732814 | 0.96815259 | -0.1258962 | 119.3464702 | 245.2008312 |
| YBR213W | MET8 | 0.17355026 | 0.96815259 | -0.1257587 | 122.6644579 | 251.3663394 |
| YLR368W | MDM30 | 0.7233186 | 0.97135148 | -0.1257088 | 100.2515402 | 209.5432042 |
| YPL087W | YDC1 | 0.4080106 | 0.96815259 | -0.1256621 | 96.06576777 | 201.725741 |
| YKR054C | DYN1 | 0.37536294 | 0.96815259 | -0.1256112 | 101.3125394 | 211.505159 |
| YKR007W | MEH1 | 0.2504951 | 0.96815259 | -0.125603 | 94.62996174 | 199.0364816 |
| YKR052C | MRS4 | 0.04130681 | 0.96815259 | -0.1255076 | 97.29510065 | 203.9915448 |
| YDR018C | YDR018C | 0.1997642 | 0.96815259 | -0.1254835 | 115.7959296 | 238.5029211 |
| YJL217W | YJL217W | 0.40730976 | 0.96815259 | -0.1254097 | 111.5176416 | 230.5080076 |
| YDL053C | PBP4 | 0.17269099 | 0.96815259 | -0.1252729 | 113.6192329 | 234.4043077 |
| YKR095W | MLP1 | 0.27843841 | 0.96815259 | -0.1251913 | 88.58928027 | 187.6930853 |
| YHR079C | IRE1 | 0.5610576 | 0.96815259 | -0.1251622 | 125.127369 | 255.8544048 |
| YNR018W | YNR018W | 0.3696515 | 0.96815259 | -0.1251327 | 100.9577871 | 210.7576397 |
| YJR103W | URA8 | 0.35927864 | 0.96815259 | -0.1250283 | 115.8659318 | 238.552014 |
| YDR363W-A | SEM1 | 0.29769145 | 0.96815259 | -0.1249228 | 89.46750854 | 189.2834511 |
| YCL057W | PRD1 | 0.29669443 | 0.96815259 | -0.1245402 | 116.3204669 | 239.3126094 |
| YJL093C | TOK1 | 0.50402655 | 0.96815259 | -0.1244308 | 109.481215 | 226.5335019 |
| YDR216W | ADR1 | 0.3370894 | 0.96815259 | -0.1242734 | 111.9955085 | 231.1960604 |
| YMR065W | KAR5 | 0.32764489 | 0.96815259 | -0.1240109 | 107.3896714 | 222.5562652 |
| YOL124C | TRM11 | 0.46586783 | 0.96815259 | -0.1238035 | 91.30195363 | 192.5054116 |
| YNL009W | IDP3 | 0.2297079 | 0.96815259 | -0.1237424 | 123.6198885 | 252.7877585 |
| YOR355W | GDS1 | 0.38741668 | 0.96815259 | -0.1235943 | 106.2318076 | 220.3215235 |
| YHL021C | FMP12 | 0.22333836 | 0.96815259 | -0.1233552 | 104.1164583 | 216.3322417 |

| | | | | | | |
|---|---|---|---|---|---|---|
| YIL084C | SDS3 | 0.47056174 | 0.96815259 | -0.122993 | 101.7457616 | 211.8445403 |
| YDR351W | SBE2 | 0.27357142 | 0.96815259 | -0.1228392 | 115.5852434 | 237.6363452 |
| YKR035C | YKR035C | 0.01461938 | 0.68234381 | -0.1227194 | 84.92268733 | 180.4099189 |
| YGL209W | MIG2 | 0.04047143 | 0.96815259 | -0.1225862 | 97.13962335 | 203.1783539 |
| YPR089W | YPR089W | 0.73115664 | 0.97135148 | -0.1224596 | 74.85041554 | 161.5722655 |
| YKR094C | RPL40B | 0.35274431 | 0.96815259 | -0.1224406 | 85.19559342 | 180.8691488 |
| YDL056W | MBP1 | 0.40355898 | 0.96815259 | -0.1223804 | 109.7065745 | 226.5867764 |
| YDL027C | YDL027C | 0.24004548 | 0.96815259 | -0.1223104 | 115.9121814 | 238.1516065 |
| YDL122W | UBP1 | 0.11277465 | 0.96815259 | -0.1221755 | 115.5559814 | 237.4629062 |
| YNR069C | BSC5 | 0.29903897 | 0.96815259 | -0.1221061 | 93.73997047 | 196.7498799 |
| YDL096C | YDL096C | 0.18594457 | 0.96815259 | -0.1220976 | 109.1696104 | 225.5343523 |
| YOR243C | PUS7 | 0.41894043 | 0.96815259 | -0.1219584 | 96.75645836 | 202.351075 |
| YDR223W | CRF1 | 0.2272749 | 0.96815259 | -0.1219265 | 115.3681466 | 237.0678785 |
| YCR020C-A | MAK31 | 0.20490134 | 0.96815259 | -0.1216533 | 103.9634052 | 215.7419325 |
| YMR144W | YMR144W | 0.38921756 | 0.96815259 | -0.1215648 | 109.4805917 | 226.0191095 |
| YOR065W | CYT1 | 0.40185913 | 0.96815259 | -0.1214059 | 5.249640912 | 31.53433357 |
| YMR251W | GTO3 | 0.41567509 | 0.96815259 | -0.1213882 | 109.3811697 | 225.8020046 |
| YKL037W | YKL037W | 0.2333324 | 0.96815259 | -0.1212066 | 104.5945046 | 216.8393455 |
| YPL102C | YPL102C | 0.46254705 | 0.96815259 | -0.1211403 | 84.74125115 | 179.7886572 |
| YDR254W | CHL4 | 0.33537767 | 0.96815259 | -0.1207665 | 111.7846262 | 230.1746444 |
| YML095C | RAD10 | 0.33326767 | 0.96815259 | -0.1207595 | 109.0220522 | 225.0194402 |
| YPL127C | HHO1 | 0.35122115 | 0.96815259 | -0.1206602 | 104.2654659 | 216.1276448 |
| YDL001W | RMD1 | 0.3969939 | 0.96815259 | -0.1203862 | 116.8345702 | 239.5278613 |
| YOR238W | YOR238W | 0.42150577 | 0.96815259 | -0.1203334 | 113.4038716 | 233.1180034 |
| YKL178C | STE3 | 0.33557872 | 0.96815259 | -0.1201566 | 111.662549 | 229.8376693 |
| YCR105W | ADH7 | 0.4146207 | 0.96815259 | -0.1197019 | 121.9300018 | 248.911509 |
| YKL026C | GPX1 | 0.29766958 | 0.96815259 | -0.1196994 | 114.5336759 | 235.1122617 |
| YLR311C | YLR311C | 0.35654905 | 0.96815259 | -0.1193539 | 121.0053983 | 247.1242277 |
| YLR113W | HOG1 | 0.27742946 | 0.96815259 | -0.1192906 | 97.69784167 | 203.6296177 |
| YGR237C | YGR237C | 0.52955662 | 0.96815259 | -0.1192662 | 120.1454274 | 245.5041385 |
| YBL069W | AST1 | 0.29366016 | 0.96815259 | -0.1191526 | 110.0977383 | 226.738522 |
| YNL339C | YRF1-6 | 0.31809765 | 0.96815259 | -0.1189192 | 117.5781722 | 240.6524567 |
| YOR220W | WSP1 | 0.49644931 | 0.96815259 | -0.1187376 | 97.10555981 | 202.4256185 |
| YMR086W | YMR086W | 0.27130692 | 0.96815259 | -0.1186542 | 109.9332025 | 226.3423122 |
| YDL149W | ATG9 | 0.52199565 | 0.96815259 | -0.1185848 | 109.1348417 | 224.8404431 |
| YKL127W | PGM1 | 0.39649138 | 0.96815259 | -0.1184883 | 111.9795182 | 230.1302701 |
| YDR255C | RMD5 | 0.25644894 | 0.96815259 | -0.1184679 | 114.060788 | 234.0095009 |
| YDL239C | ADY3 | 0.23247154 | 0.96815259 | -0.1184176 | 115.4139185 | 236.5249319 |
| YDL128W | VCX1 | 0.27656826 | 0.96815259 | -0.1184152 | 123.0489577 | 250.7686542 |
| YNR021W | YNR021W | 0.3617555 | 0.96815259 | -0.1183662 | 105.8032264 | 218.5857306 |
| YGR230W | BNS1 | 0.56613799 | 0.96815259 | -0.1183358 | 119.3281931 | 243.8128639 |
| YER077C | YER077C | 0.25781337 | 0.96815259 | -0.1182772 | 89.58848881 | 188.3190986 |
| YNL273W | TOF1 | 0.48334234 | 0.96815259 | -0.1182518 | 100.8374007 | 209.3008547 |
| YDL011C | YDL011C | 0.34149023 | 0.96815259 | -0.118161 | 113.4206781 | 232.7603394 |
| YCR023C | YCR023C | 0.18970325 | 0.96815259 | -0.1181398 | 110.6551581 | 227.5971024 |
| YMR172W | HOT1 | 0.5618817 | 0.96815259 | -0.1179922 | 110.04965 | 226.4410085 |
| YDR493W | FMP36 | 0.47734134 | 0.96815259 | -0.1179018 | 101.9815766 | 211.3727976 |
| YKL086W | SRX1 | 0.43078492 | 0.96815259 | -0.1176853 | 114.6732273 | 235.0119394 |
| YHR143W | DSE2 | 0.33328325 | 0.96815259 | -0.1176413 | 125.8987814 | 255.9467839 |
| YHR082C | KSP1 | 0.49346612 | 0.96815259 | -0.117453 | 110.4328364 | 227.0593445 |
| YHL019C | APM2 | 0.39557071 | 0.96815259 | -0.1173831 | 104.0490496 | 215.1370399 |
| YDR307W | YDR307W | 0.29488673 | 0.96815259 | -0.1171025 | 115.6705027 | 236.7681201 |
| YGR100W | MDR1 | 0.46501095 | 0.96815259 | -0.1170947 | 118.5457881 | 242.1309487 |
| YDL188C | PPH22 | 0.4366436 | 0.96815259 | -0.116811 | 111.2335446 | 228.4382019 |
| YHR105W | YPT35 | 0.30340292 | 0.96815259 | -0.1167323 | 110.2681347 | 226.6230109 |
| YJR153W | PGU1 | 0.39307484 | 0.96815259 | -0.1167302 | 113.1022438 | 231.9100335 |
| YNR044W | AGA1 | 0.52949912 | 0.96815259 | -0.1166471 | 94.81363877 | 197.7753861 |
| YDL002C | NHP10 | 0.8135302 | 0.97633618 | -0.1163324 | 40.11759133 | 95.67647629 |
| YOR124C | UBP2 | 0.48266901 | 0.96815259 | -0.1162959 | 96.12244482 | 200.1542425 |
| YGR170W | PSD2 | 0.55026904 | 0.96815259 | -0.116272 | 107.2589386 | 220.9265359 |
| YDR061W | YDR061W | 0.34217115 | 0.96815259 | -0.1162578 | 116.6289503 | 238.404968 |
| YOR226C | ISU2 | 0.30081273 | 0.96815259 | -0.1162431 | 106.6117339 | 219.7139254 |
| YCL051W | LRE1 | 0.19935391 | 0.96815259 | -0.1159287 | 118.066494 | 241.0279543 |
| YMR138W | CIN4 | 0.07078795 | 0.96815259 | -0.1157449 | 90.33846144 | 189.264819 |

| | | | | | | |
|---|---|---|---|---|---|---|
| YDR279W | RNH202 | 0.35839455 | 0.96815259 | -0.115726 | 109.4342483 | 224.8870964 |
| YKR064W | OAF3 | 0.25829638 | 0.96815259 | -0.1156696 | 93.95047537 | 195.9900131 |
| YKR084C | HBS1 | 0.50840121 | 0.96815259 | -0.1155566 | 96.16102758 | 200.0938418 |
| YER007W | PAC2 | 0.24504853 | 0.96815259 | -0.1153088 | 114.7044403 | 234.6446028 |
| YEL053C | MAK10 | 0.06384011 | 0.96815259 | -0.1149143 | 95.19152405 | 198.1700946 |
| YGL035C | MIG1 | 0.42352612 | 0.96815259 | -0.1148227 | 95.74383878 | 199.184107 |
| YMR284W | YKU70 | 0.38042565 | 0.96815259 | -0.1147032 | 118.6137823 | 241.8295405 |
| YPL205C | YPL205C | 0.62621217 | 0.96815259 | -0.114676 | 8.939732121 | 37.21353359 |
| YKL010C | UFD4 | 0.20905321 | 0.96815259 | -0.1144619 | 101.5321921 | 209.9184221 |
| YCR098C | GIT1 | 0.38727775 | 0.96815259 | -0.114207 | 112.5512539 | 230.4302488 |
| YDR199W | YDR199W | 0.31046481 | 0.96815259 | -0.1142011 | 117.0451296 | 238.8131027 |
| YOR253W | NAT5 | 0.47910492 | 0.96815259 | -0.1137765 | 98.02353975 | 203.2498273 |
| YGR174C | CBP4 | 0.27098837 | 0.96815259 | -0.1134496 | 108.7485489 | 223.2001824 |
| YBR041W | FAT1 | 0.40639975 | 0.96815259 | -0.1134041 | 85.25817493 | 179.3677144 |
| YFR030W | MET10 | 0.42346818 | 0.96815259 | -0.1133544 | 100.7147506 | 208.1950449 |
| YGL244W | RTF1 | 0.74235179 | 0.97135148 | -0.1132997 | 83.18231706 | 175.4762299 |
| YAL065C | YAL065C | 0.82496879 | 0.97873587 | -0.1130429 | 92.440115 | 192.7018587 |
| YNR075W | COS10 | 0.47629176 | 0.96815259 | -0.1128972 | 96.63464732 | 200.501215 |
| YER167W | BCK2 | 0.31798699 | 0.96815259 | -0.1127437 | 113.5080146 | 231.9531878 |
| YHR184W | SSP1 | 0.60676919 | 0.96815259 | -0.1127403 | 110.2705996 | 225.9127645 |
| YJL058C | BIT61 | 0.30405873 | 0.96815259 | -0.112712 | 114.5529349 | 233.896946 |
| YCR014C | POL4 | 0.14146389 | 0.96815259 | -0.1126914 | 117.3176283 | 239.0511461 |
| YNL259C | ATX1 | 0.21533246 | 0.96815259 | -0.1123743 | 98.69354325 | 204.2487162 |
| YLR398C | SKI2 | 0.45836221 | 0.96815259 | -0.1123155 | 94.91471292 | 197.1882871 |
| YNL322C | KRE1 | 0.35464184 | 0.96815259 | -0.112143 | 108.246888 | 222.0303085 |
| YCR068W | ATG15 | 0.46122019 | 0.96815259 | -0.1120979 | 105.2396493 | 216.4118262 |
| YIR031C | DAL7 | 0.51302004 | 0.96815259 | -0.1120854 | 115.0017918 | 234.622136 |
| YMR023C | MSS1 | 0.33483595 | 0.96815259 | -0.1119543 | 108.3521371 | 222.1928724 |
| YBR194W | SOY1 | 0.72437183 | 0.97135148 | -0.1119057 | 60.25007617 | 132.4435346 |
| YDL216C | RRI1 | 0.33031475 | 0.96815259 | -0.1118593 | 117.0790185 | 238.4569762 |
| YJR117W | STE24 | 0.42267907 | 0.96815259 | -0.1116756 | 106.3607284 | 218.4277282 |
| YDR214W | AHA1 | 0.27663055 | 0.96815259 | -0.1116388 | 114.0901994 | 232.8414607 |
| YDL066W | IDP1 | 0.28245119 | 0.96815259 | -0.1113204 | 123.7187784 | 250.747807 |
| YJL149W | YJL149W | 0.36189292 | 0.96815259 | -0.1111512 | 109.0999459 | 223.4441951 |
| YJR133W | XPT1 | 0.25893996 | 0.96815259 | -0.1110251 | 96.61849628 | 200.1358502 |
| YJR080C | FMP26 | 0.61459212 | 0.96815259 | -0.1109069 | 107.4792621 | 220.3768348 |
| YBR224W | YBR224W | 0.27979664 | 0.96815259 | -0.1108958 | 106.4940763 | 218.5368622 |
| YIL066C | RNR3 | 0.52112954 | 0.96815259 | -0.1108728 | 114.8753742 | 234.1691481 |
| YDR102C | YDR102C | 0.36557979 | 0.96815259 | -0.1106619 | 115.8135991 | 235.8817515 |
| YDL156W | YDL156W | 0.3229283 | 0.96815259 | -0.1105813 | 108.4517824 | 222.1328989 |
| YJR053W | BFA1 | 0.36803704 | 0.96815259 | -0.1104732 | 110.0517664 | 225.0985223 |
| YDR491C | YDR491C | 0.32836281 | 0.96815259 | -0.1103401 | 115.2315005 | 234.7381463 |
| YDR329C | PEX3 | 0.43753459 | 0.96815259 | -0.1103022 | 111.2837827 | 227.3663892 |
| YGR221C | TOS2 | 0.50638275 | 0.96815259 | -0.1099289 | 104.2290328 | 214.1379944 |
| YJR148W | BAT2 | 0.28006155 | 0.96815259 | -0.1099256 | 124.3886371 | 251.7477501 |
| YDR333C | YDR333C | 0.30103867 | 0.96815259 | -0.1098043 | 115.0185577 | 234.2449389 |
| YDR282C | YDR282C | 0.3727318 | 0.96815259 | -0.1096548 | 116.1275987 | 236.287219 |
| YGL255W | ZRT1 | 0.33433662 | 0.96815259 | -0.1094499 | 102.9907365 | 211.7420121 |
| YDR354W | TRP4 | 0.32448614 | 0.96815259 | -0.1091412 | 107.3819021 | 219.8790162 |
| YKL171W | YKL171W | 0.35873326 | 0.96815259 | -0.1090039 | 107.5905701 | 220.2437221 |
| YDR330W | UBX5 | 0.30747415 | 0.96815259 | -0.1088952 | 112.3683399 | 229.1378138 |
| YBR232C | YBR232C | 0.29839504 | 0.96815259 | -0.1087385 | 114.6375111 | 233.3431821 |
| YKR014C | YPT52 | 0.34013396 | 0.96815259 | -0.1087212 | 109.56857 | 223.883311 |
| YKL100C | YKL100C | 0.42702114 | 0.96815259 | -0.108233 | 119.0062441 | 241.4030981 |
| YKL190W | CNB1 | 0.38314388 | 0.96815259 | -0.1079179 | 102.4377751 | 210.4360467 |
| YJR033C | RAV1 | 0.40460709 | 0.96815259 | -0.1075045 | 107.9870862 | 220.714976 |
| YNL085W | MKT1 | 0.49878 | 0.96815259 | -0.1074901 | 104.2157523 | 213.6764835 |
| YPL152W | RRD2 | 0.7635212 | 0.97135148 | -0.1074026 | 61.13834078 | 133.2943295 |
| YJL160C | YJL160C | 0.40154009 | 0.96815259 | -0.1073094 | 110.0535397 | 224.5352803 |
| YKR078W | YKR078W | 0.28723748 | 0.96815259 | -0.1072354 | 107.2092913 | 219.2157122 |
| YML028W | TSA1 | 0.04445094 | 0.96815259 | -0.107174 | 91.04782084 | 189.0534012 |
| YIR009W | MSL1 | 0.45777032 | 0.96815259 | -0.1070862 | 117.445272 | 238.285551 |
| YDL050C | YDL050C | 0.3447642 | 0.96815259 | -0.1070173 | 118.9066589 | 240.999612 |
| YMR040W | YET2 | 0.33581807 | 0.96815259 | -0.1070155 | 108.783511 | 222.1132399 |

| | | | | | | |
|---|---|---|---|---|---|---|
| YHR167W | THP2 | 0.46198128 | 0.96815259 | -0.1068925 | 114.0925575 | 231.9959416 |
| YCL046W | YCL046W | 0.55757444 | 0.96815259 | -0.1068692 | 120.4366459 | 243.827476 |
| YBR294W | SUL1 | 0.43995032 | 0.96815259 | -0.1066787 | 125.5145528 | 253.2668588 |
| YDR276C | PMP3 | 0.33223163 | 0.96815259 | -0.1066305 | 108.22622 | 221.0046085 |
| YDR183W | PLP1 | 0.42923248 | 0.96815259 | -0.1066111 | 115.2508915 | 234.1065719 |
| YOL084W | PHM7 | 0.59915953 | 0.96815259 | -0.1064889 | 115.3141826 | 234.2027524 |
| YHL007C | STE20 | 0.44867101 | 0.96815259 | -0.1064245 | 100.4826111 | 206.5210038 |
| YMR034C | YMR034C | 0.36223988 | 0.96815259 | -0.1064166 | 104.7782003 | 214.53357 |
| YOR269W | PAC1 | 0.32570938 | 0.96815259 | -0.106293 | 97.15451913 | 200.2884686 |
| YDR209C | YDR209C | 0.48372587 | 0.96815259 | -0.1061896 | 117.9980348 | 239.1562442 |
| YJL154C | VPS35 | 0.52484058 | 0.96815259 | -0.1060928 | 104.9315682 | 214.761723 |
| YBR187W | GDT1 | 0.43062314 | 0.96815259 | -0.1059364 | 105.591084 | 215.9641303 |
| YDL071C | YDL071C | 0.37986264 | 0.96815259 | -0.1057669 | 110.6384021 | 225.3502017 |
| YLR410W | VIP1 | 0.73142559 | 0.97135148 | -0.1057337 | 75.1084341 | 159.0584862 |
| YKR105C | YKR105C | 0.58072862 | 0.96815259 | -0.1056168 | 108.7710853 | 221.8395949 |
| YDL135C | RDI1 | 0.2413711 | 0.96815259 | -0.1055656 | 115.1471125 | 233.7257319 |
| YGR194C | XKS1 | 0.45519643 | 0.96815259 | -0.1055611 | 105.5736037 | 215.8643066 |
| YFL010C | WWM1 | 0.32231086 | 0.96815259 | -0.1054339 | 123.0933852 | 248.5269418 |
| YJR015W | YJR015W | 0.36459905 | 0.96815259 | -0.1054237 | 109.225474 | 222.6527332 |
| YHR022C | YHR022C | 0.5405311 | 0.96815259 | -0.105238 | 100.4721327 | 206.2889972 |
| YMR036C | MIH1 | 0.51154172 | 0.96815259 | -0.105216 | 115.6804433 | 234.6581287 |
| YBL015W | ACH1 | 0.0244974 | 0.84153481 | -0.1051433 | 110.7046388 | 225.3620928 |
| YDR048C | YDR048C | 0.32723148 | 0.96815259 | -0.1051411 | 109.4371085 | 222.9969722 |
| YDR011W | SNQ2 | 0.34814397 | 0.96815259 | -0.1051001 | 113.9720916 | 231.4502176 |
| YDR186C | YDR186C | 0.2909193 | 0.96815259 | -0.1048651 | 112.6777916 | 228.9934579 |
| YKR001C | VPS1 | 0.33642805 | 0.96815259 | -0.1048628 | 88.81129921 | 184.4670096 |
| YKL206C | ADD66 | 0.31458548 | 0.96815259 | -0.1048486 | 107.1633222 | 218.7025445 |
| YNL021W | HDA1 | 0.53972111 | 0.96815259 | -0.1040374 | 85.31319974 | 177.7930487 |
| YDR349C | YPS7 | 0.80937538 | 0.9762419 | -0.1038405 | 82.5689526 | 172.6380312 |
| YJL177W | RPL17B | 0.40329957 | 0.96815259 | -0.1036294 | 110.3626873 | 224.4530465 |
| YKR031C | SPO14 | 0.38535241 | 0.96815259 | -0.1034437 | 104.1034703 | 212.7424199 |
| YKL164C | PIR1 | 0.30551266 | 0.96815259 | -0.1034139 | 108.8698352 | 221.6293453 |
| YMR135C | GID8 | 0.51405256 | 0.96815259 | -0.1031118 | 104.419134 | 213.2718902 |
| YCL012W | YCL012W | 0.4013521 | 0.96815259 | -0.1028263 | 114.2354175 | 231.5343058 |
| YBR300C | YBR300C | 0.34646222 | 0.96815259 | -0.1026733 | 113.4511811 | 230.0438223 |
| YDR318W | MCM21 | 0.36324488 | 0.96815259 | -0.1026337 | 114.9461433 | 232.8257748 |
| YPR066W | UBA3 | 0.57526827 | 0.96815259 | -0.102466 | 87.62104645 | 181.8172436 |
| YDL123W | SNA4 | 0.39595372 | 0.96815259 | -0.1024235 | 117.344111 | 237.261852 |
| YBR150C | TBS1 | 0.46243551 | 0.96815259 | -0.102366 | 103.2751307 | 211.0040552 |
| YDR178W | SDH4 | 0.50021651 | 0.96815259 | -0.1023145 | 102.593157 | 209.7225156 |
| YBR218C | PYC2 | 0.59286232 | 0.96815259 | -0.1020701 | 117.663242 | 237.7939553 |
| YIL057C | YIL057C | 0.63219443 | 0.96815259 | -0.1019613 | 132.8861848 | 266.1748403 |
| YOR123C | LEO1 | 0.48972762 | 0.96815259 | -0.1018997 | 105.8297709 | 215.6865543 |
| YKL031W | YKL031W | 0.64825227 | 0.96815259 | -0.1018707 | 105.5423508 | 215.1451472 |
| YKR041W | YKR041W | 0.3550641 | 0.96815259 | -0.1018659 | 113.524411 | 230.0358633 |
| YDL203C | ACK1 | 0.40919841 | 0.96815259 | -0.10185 | 116.1378036 | 234.9086422 |
| YMR191W | SPG5 | 0.34377011 | 0.96815259 | -0.1017693 | 112.0424953 | 227.2538597 |
| YIL010W | DOT5 | 0.5955606 | 0.96815259 | -0.1017168 | 115.1609411 | 233.0623188 |
| YCR015C | YCR015C | 0.24091219 | 0.96815259 | -0.1016262 | 117.2983112 | 237.0336268 |
| YJR129C | YJR129C | 0.15125369 | 0.96815259 | -0.1014905 | 93.33562707 | 192.3038356 |
| YDR198C | RKM2 | 0.32402652 | 0.96815259 | -0.1014689 | 116.1546831 | 234.8718834 |
| YJL053W | PEP8 | 0.44640482 | 0.96815259 | -0.1013323 | 111.935152 | 226.9753433 |
| YJR126C | VPS70 | 0.19197182 | 0.96815259 | -0.1013072 | 94.83442581 | 195.0672217 |
| YKL202W | YKL202W | 0.22674327 | 0.96815259 | -0.1011576 | 97.45518972 | 199.9298152 |
| YGL002W | ERP6 | 0.48859502 | 0.96815259 | -0.1010415 | 115.1140443 | 232.8538932 |
| YJL020C | BBC1 | 0.47634145 | 0.96815259 | -0.1008679 | 119.600426 | 241.1927391 |
| YFR035C | YFR035C | 0.40832914 | 0.96815259 | -0.1008441 | 113.665835 | 230.1167257 |
| YDR287W | YDR287W | 0.3540631 | 0.96815259 | -0.1003294 | 114.6841134 | 231.9242802 |
| YOR327C | SNC2 | 0.68390248 | 0.97080037 | -0.100284 | 104.7083758 | 213.305124 |
| YMR039C | SUB1 | 0.43987932 | 0.96815259 | -0.1000576 | 93.16650063 | 191.7317225 |
| YDR359C | VID21 | 0.82205414 | 0.97873587 | -0.0999148 | 61.71945342 | 133.0376179 |
| YFR025C | HIS2 | 0.38562452 | 0.96815259 | -0.0997707 | 83.80120299 | 174.2081764 |
| YCR099C | YCR099C | 0.36630062 | 0.96815259 | -0.0996386 | 116.5474646 | 235.2769028 |
| YDR310C | SUM1 | 0.29663601 | 0.96815259 | -0.0994621 | 101.6911009 | 207.528838 |

| | | | | | | |
|---|---|---|---|---|---|---|
| YMR109W | MYO5 | 0.41589302 | 0.96815259 | -0.0993759 | 106.8282754 | 217.0974673 |
| YDL215C | GDH2 | 0.45675657 | 0.96815259 | -0.0992839 | 114.683245 | 231.7354466 |
| YCR019W | MAK32 | 0.23012999 | 0.96815259 | -0.0992288 | 112.1671513 | 227.0314758 |
| YOR055W | YOR055W | 0.59526326 | 0.96815259 | -0.0986856 | 116.3265596 | 234.6941205 |
| YLR077W | FMP25 | 0.45054836 | 0.96815259 | -0.0986151 | 105.9599637 | 215.3412656 |
| YML123C | PHO84 | 0.66991501 | 0.97060928 | -0.098592 | 108.3920583 | 219.874526 |
| YGR181W | TIM13 | 0.47085721 | 0.96815259 | -0.0982748 | 115.0643845 | 232.2658185 |
| YDL184C | RPL41A | 0.42440752 | 0.96815259 | -0.0982321 | 103.555719 | 210.7872512 |
| YHR123W | EPT1 | 0.51610611 | 0.96815259 | -0.0979201 | 111.1937351 | 224.9810947 |
| YDR215C | YDR215C | 0.40392783 | 0.96815259 | -0.0978865 | 114.4676006 | 231.0829052 |
| YMR316C-A | YMR316C-A | 0.40786733 | 0.96815259 | -0.0969148 | 118.5353757 | 238.4978624 |
| YJL187C | SWE1 | 0.60306272 | 0.96815259 | -0.0968984 | 119.3797232 | 240.0701529 |
| YJR001W | AVT1 | 0.50570475 | 0.96815259 | -0.0965739 | 124.1270094 | 248.8687298 |
| YDL238C | GUD1 | 0.3994139 | 0.96815259 | -0.0964194 | 123.4206379 | 247.5232291 |
| YJR079W | YJR079W | 0.5685133 | 0.96815259 | -0.0963755 | 114.501917 | 230.8763507 |
| YNR020C | YNR020C | 0.15955756 | 0.96815259 | -0.0959731 | 84.08073258 | 174.0496325 |
| YKR021W | ALY1 | 0.44149358 | 0.96815259 | -0.0959662 | 107.6012413 | 217.9289456 |
| YPL174C | NIP100 | 0.54524879 | 0.96815259 | -0.095887 | 95.81924832 | 195.93393 |
| YJL216C | YJL216C | 0.56162059 | 0.96815259 | -0.0958212 | 115.9327196 | 233.4464251 |
| YCR026C | NPP1 | 0.49498723 | 0.96815259 | -0.0957457 | 104.8139561 | 212.6894152 |
| YLR021W | IRC25 | 0.45978005 | 0.96815259 | -0.0953154 | 90.90502541 | 186.6634478 |
| YDR352W | YDR352W | 0.40900967 | 0.96815259 | -0.0949556 | 112.3139329 | 226.5401077 |
| YDR165W | TRM82 | 0.37900708 | 0.96815259 | -0.0949332 | 114.1594835 | 229.9792096 |
| YGR169C | PUS6 | 0.51329018 | 0.96815259 | -0.0947891 | 119.4357023 | 239.7968868 |
| YDR370C | YDR370C | 0.36672218 | 0.96815259 | -0.0945882 | 117.6529746 | 236.4349878 |
| YPL033C | YPL033C | 0.52030797 | 0.96815259 | -0.0945345 | 106.1980844 | 215.0547974 |
| YDL134C-A | YDL134C-A | 0.41834265 | 0.96815259 | -0.0944626 | 126.9372615 | 253.7335397 |
| YNL237W | YTP1 | 0.58640719 | 0.96815259 | -0.0944616 | 114.7622233 | 231.0192463 |
| YJL165C | HAL5 | 0.5037229 | 0.96815259 | -0.0941869 | 100.3085728 | 204.00491 |
| YIR017C | MET28 | 0.57674834 | 0.96815259 | -0.0941092 | 119.5573967 | 239.9021632 |
| YNL003C | PET8 | 0.81226553 | 0.97633618 | -0.0940152 | 35.1081681 | 82.33435343 |
| YBR274W | CHK1 | 0.11564033 | 0.96815259 | -0.0938995 | 113.6541318 | 228.8513051 |
| YMR188C | MRPS17 | 0.57124063 | 0.96815259 | -0.0938283 | 107.3806287 | 217.1345282 |
| YER019W | ISC1 | 0.47971687 | 0.96815259 | -0.0936794 | 111.2570002 | 224.3397393 |
| YKR011C | YKR011C | 0.51562941 | 0.96815259 | -0.093403 | 121.4859307 | 243.3736276 |
| YHL044W | YHL044W | 0.53818533 | 0.96815259 | -0.0933303 | 123.3698668 | 246.8753335 |
| YCR059C | YIH1 | 0.47397217 | 0.96815259 | -0.0931742 | 113.9361075 | 229.2474945 |
| YGL016W | KAP122 | 0.36376707 | 0.96815259 | -0.0930015 | 90.96514541 | 186.3612627 |
| YDR146C | SWI5 | 0.35319292 | 0.96815259 | -0.0930005 | 108.0720372 | 218.2761929 |
| YKL157W | APE2 | 0.53134618 | 0.96815259 | -0.0929254 | 119.4468124 | 239.4838679 |
| YGL218W | YGL218W | 0.68675459 | 0.97133463 | -0.0928858 | 15.38568049 | 45.33725509 |
| YOR291W | YOR291W | 0.5672526 | 0.96815259 | -0.0927164 | 110.1480704 | 222.0984323 |
| YPL019C | VTC3 | 0.35355323 | 0.96815259 | -0.0925083 | 113.3373828 | 228.0112417 |
| YCR091W | KIN82 | 0.47676344 | 0.96815259 | -0.0924618 | 112.3750811 | 226.2076144 |
| YBR225W | YBR225W | 0.48084985 | 0.96815259 | -0.0922481 | 106.4304382 | 215.078853 |
| YML016C | PPZ1 | 0.32242975 | 0.96815259 | -0.0918932 | 93.5688271 | 191.0202991 |
| YIL139C | REV7 | 0.57965713 | 0.96815259 | -0.0915146 | 114.3565982 | 229.7347745 |
| YDR334W | SWR1 | 0.51332997 | 0.96815259 | -0.0911553 | 110.6199818 | 222.699295 |
| YCL069W | VBA3 | 0.23769417 | 0.96815259 | -0.0910072 | 110.4541365 | 222.3633662 |
| YLR281C | YLR281C | 0.77844331 | 0.97246744 | -0.0908863 | 92.88915284 | 189.5719702 |
| YKR056W | TRM2 | 0.43131377 | 0.96815259 | -0.0908609 | 111.5342764 | 224.3523085 |
| YNL301C | RPL18B | 0.26688891 | 0.96815259 | -0.090799 | 100.8796952 | 204.4637328 |
| YLR207W | HRD3 | 0.52271201 | 0.96815259 | -0.0907035 | 107.2660583 | 216.36121 |
| YBL075C | SSA3 | 0.58530153 | 0.96815259 | -0.0906005 | 119.9406203 | 239.9887973 |
| YDR015C | YDR015C | 0.4520533 | 0.96815259 | -0.0905823 | 115.4642388 | 231.6342827 |
| YDR225W | HTA1 | 0.4884521 | 0.96815259 | -0.0903182 | 118.9431395 | 238.0773272 |
| YLR450W | HMG2 | 0.08486672 | 0.96815259 | -0.0902884 | 83.56276415 | 172.0653117 |
| YMR006C | PLB2 | 0.35093612 | 0.96815259 | -0.0902319 | 105.0403928 | 212.1244992 |
| YMR305C | SCW10 | 0.49787239 | 0.96815259 | -0.0901737 | 109.2419882 | 219.9526913 |
| YBR108W | YBR108W | 0.54417091 | 0.96815259 | -0.0901141 | 113.0246672 | 226.9991004 |
| YIR004W | DJP1 | 0.82511035 | 0.97873587 | -0.0900253 | 95.80500656 | 194.8576862 |
| YMR283C | RIT1 | 0.57790256 | 0.96815259 | -0.0899316 | 118.7938306 | 237.7295449 |
| YPR046W | MCM16 | 0.50701301 | 0.96815259 | -0.089884 | 106.321579 | 214.4524203 |
| YDR084C | TVP23 | 0.4545546 | 0.96815259 | -0.0896472 | 117.5771361 | 235.4087054 |

| | | | | | | |
|---|---|---|---|---|---|---|
| YMR054W | STV1 | 0.41334125 | 0.96815259 | -0.0895648 | 108.1854865 | 217.8726247 |
| YMR274C | RCE1 | 0.45303682 | 0.96815259 | -0.0895059 | 114.7628718 | 230.1330255 |
| YPL123C | RNY1 | 0.65921981 | 0.96815259 | -0.0894562 | 114.754862 | 230.1091924 |
| YER131W | RPS26B | 0.78720532 | 0.97260548 | -0.089347 | 75.63765716 | 157.111428 |
| YMR055C | BUB2 | 0.52665235 | 0.96815259 | -0.0892643 | 111.3706945 | 223.7612191 |
| YBL008W | HIR1 | 0.74980019 | 0.97135148 | -0.0892428 | 103.1902898 | 208.4957697 |
| YIL173W | VTH1 | 0.5161087 | 0.96815259 | -0.0888326 | 109.455891 | 220.1115988 |
| YPR185W | ATG13 | 0.66291294 | 0.96927465 | -0.088667 | 90.2066897 | 184.1700662 |
| YIR043C | YIR043C | 0.62582742 | 0.96815259 | -0.0886336 | 117.6005854 | 235.2709589 |
| YML036W | CGI121 | 0.36588027 | 0.96815259 | -0.0885718 | 94.79433713 | 192.71187 |
| YKL091C | YKL091C | 0.27409521 | 0.96815259 | -0.0885173 | 103.2913126 | 208.5543203 |
| YLR124W | YLR124W | 0.44389588 | 0.96815259 | -0.0885089 | 111.6412013 | 224.1306194 |
| YOR313C | SPS4 | 0.40415346 | 0.96815259 | -0.0884972 | 109.8644892 | 220.8138274 |
| YDR281C | PHM6 | 0.38981769 | 0.96815259 | -0.088462 | 114.8029658 | 230.0209009 |
| YDL138W | RGT2 | 0.42407925 | 0.96815259 | -0.0880429 | 115.0544559 | 230.4150466 |
| YKL102C | YKL102C | 0.50844643 | 0.96815259 | -0.0880288 | 105.5607981 | 212.7008635 |
| YDR010C | YDR010C | 0.42631499 | 0.96815259 | -0.0879862 | 115.7051264 | 231.6187966 |
| YOR142W | LSC1 | 0.72231603 | 0.97135148 | -0.0879838 | 98.37452037 | 199.2858679 |
| YDR153C | ENT5 | 0.31355731 | 0.96815259 | -0.087904 | 118.3101344 | 236.4640597 |
| YKL168C | KKQ8 | 0.40227989 | 0.96815259 | -0.087873 | 111.3372443 | 223.4496651 |
| YCL036W | GFD2 | 0.46664667 | 0.96815259 | -0.0877558 | 109.7697307 | 220.5042897 |
| YDR070C | FMP16 | 0.44909742 | 0.96815259 | -0.0874081 | 123.4323581 | 245.9314327 |
| YHR206W | SKN7 | 0.67866194 | 0.97060928 | -0.0873321 | 125.3110533 | 249.422762 |
| YDR116C | MRPL1 | 0.47626568 | 0.96815259 | -0.0872272 | 105.1236006 | 211.7416781 |
| YKR104W | YKR104W | 0.58302997 | 0.96815259 | -0.0869977 | 101.8631474 | 205.6177731 |
| YIL140W | AXL2 | 0.52389408 | 0.96815259 | -0.0869054 | 125.7461757 | 250.1581294 |
| YIR024C | YIR024C | 0.50163168 | 0.96815259 | -0.0868521 | 121.9163298 | 243.0035211 |
| YHR121W | LSM12 | 0.54255914 | 0.96815259 | -0.0863394 | 105.0804998 | 211.5022753 |
| YJL021C | YJL021C | 0.52327755 | 0.96815259 | -0.0862946 | 123.8753118 | 246.5584136 |
| YHR209W | CRG1 | 0.50539149 | 0.96815259 | -0.085819 | 99.77949514 | 201.5193861 |
| YDL036C | PUS9 | 0.47098553 | 0.96815259 | -0.0857525 | 110.3174824 | 221.1674483 |
| YGR110W | YGR110W | 0.19048988 | 0.96815259 | -0.0856899 | 103.6196992 | 208.6606636 |
| YKR090W | PXL1 | 0.45907722 | 0.96815259 | -0.0856698 | 115.0012023 | 229.8907321 |
| YCR075C | ERS1 | 0.39219471 | 0.96815259 | -0.0855544 | 110.1992287 | 220.9113618 |
| YKR015C | YKR015C | 0.48976794 | 0.96815259 | -0.0854975 | 101.2400211 | 204.1866138 |
| YDL037C | BSC1 | 0.43968643 | 0.96815259 | -0.0849916 | 115.2418208 | 230.218179 |
| YNR008W | LRO1 | 0.31049293 | 0.96815259 | -0.0848986 | 119.0188678 | 237.2481096 |
| YJL197W | UBP12 | 0.47849118 | 0.96815259 | -0.0847815 | 127.1543409 | 252.4049215 |
| YML119W | YML119W | 0.47582309 | 0.96815259 | -0.0847078 | 111.7849442 | 223.7181116 |
| YDR063W | YDR063W | 0.44723146 | 0.96815259 | -0.0842557 | 117.8550758 | 234.9617828 |
| YNL279W | PRM1 | 0.73647679 | 0.97135148 | -0.0841778 | 103.3592003 | 207.9038929 |
| YHR200W | RPN10 | 0.70252747 | 0.97135148 | -0.0841092 | 96.5824047 | 195.2486247 |
| YOL044W | PEX15 | 0.6206828 | 0.96815259 | -0.0839573 | 105.9611287 | 212.7186345 |
| YDR406W | PDR15 | 0.49066798 | 0.96815259 | -0.0838011 | 128.2429223 | 254.2602441 |
| YKL139W | CTK1 | 0.80298971 | 0.9762419 | -0.0837708 | 44.61316176 | 98.23267408 |
| YKL133C | YKL133C | 0.45945557 | 0.96815259 | -0.0837063 | 107.9546045 | 216.3927753 |
| YJL152W | YJL152W | 0.55870593 | 0.96815259 | -0.0836908 | 118.7500625 | 236.5303214 |
| YBR278W | DPB3 | 0.58970693 | 0.96815259 | -0.0836743 | 110.5143021 | 221.162505 |
| YOR228C | YOR228C | 0.67452492 | 0.97060928 | -0.0836395 | 110.3685971 | 220.8844283 |
| YGL121C | GPG1 | 0.42278206 | 0.96815259 | -0.0834484 | 97.14300126 | 196.1761479 |
| YJR078W | BNA2 | 0.49124866 | 0.96815259 | -0.0834469 | 123.3583335 | 245.0839827 |
| YGL133W | ITC1 | 0.6427287 | 0.96815259 | -0.0833792 | 95.18399937 | 192.5089831 |
| YJL122W | ALB1 | 0.57363081 | 0.96815259 | -0.0833742 | 119.8310788 | 238.4904175 |
| YOR166C | SWT1 | 0.57116471 | 0.96815259 | -0.0832308 | 111.8491676 | 223.5734408 |
| YDR220C | YDR220C | 0.55317601 | 0.96815259 | -0.0830973 | 115.0799893 | 229.5770567 |
| YIL168W | YIL168W | 0.56083164 | 0.96815259 | -0.0830656 | 101.5447045 | 204.3195421 |
| YGR107W | YGR107W | 0.3255868 | 0.96815259 | -0.0830379 | 98.07300658 | 197.8376902 |
| YJL145W | SFH5 | 0.47246692 | 0.96815259 | -0.0828486 | 113.6053287 | 226.7813542 |
| YCR102C | YCR102C | 0.66043007 | 0.96815259 | -0.0827884 | 121.7718977 | 242.0063566 |
| YOL101C | IZH4 | 0.59914375 | 0.96815259 | -0.0827356 | 115.1852052 | 229.7085704 |
| YPR037C | ERV2 | 0.57283858 | 0.96815259 | -0.0826752 | 120.2815626 | 239.2056759 |
| YPR064W | YPR064W | 0.4159596 | 0.96815259 | -0.0826704 | 98.31738793 | 198.2278052 |
| YOR286W | FMP31 | 0.60802139 | 0.96815259 | -0.0826249 | 97.62414187 | 196.9263133 |
| YHL045W | YHL045W | 0.4905375 | 0.96815259 | -0.0825578 | 100.856384 | 202.9444606 |

| | | | | | | |
|---|---|---|---|---|---|---|
| YPL225W | YPL225W | 0.71671603 | 0.97135148 | -0.0823595 | 97.137428 | 195.9707621 |
| YKL039W | PTM1 | 0.43058167 | 0.96815259 | -0.0823325 | 111.4942792 | 222.7504946 |
| YDL234C | GYP7 | 0.44422752 | 0.96815259 | -0.0822261 | 120.0449837 | 238.6838925 |
| YLR150W | STM1 | 0.24574317 | 0.96815259 | -0.0821391 | 86.51456528 | 176.1129609 |
| YIL087C | YIL087C | 0.42554914 | 0.96815259 | -0.0821253 | 113.6373303 | 226.7115344 |
| YJR021C | REC107 | 0.56075291 | 0.96815259 | -0.0820152 | 113.9125573 | 227.2052778 |
| YOR209C | NPT1 | 0.53041855 | 0.96815259 | -0.0819963 | 113.0502646 | 225.5931848 |
| YPL224C | MMT2 | 0.584867 | 0.96815259 | -0.0817957 | 100.5146808 | 202.1705006 |
| YDR154C | YDR154C | 0.47721142 | 0.96815259 | -0.0817308 | 115.953952 | 230.9628332 |
| YDL113C | ATG20 | 0.55395763 | 0.96815259 | -0.0817199 | 112.7913991 | 225.0607332 |
| YDR104C | SPO71 | 0.25111452 | 0.96815259 | -0.0816695 | 112.3365336 | 224.2030996 |
| YBR012C | YBR012C | 0.81890127 | 0.97821875 | -0.0815966 | 104.0144321 | 208.6640871 |
| YIL160C | POT1 | 0.54286957 | 0.96815259 | -0.0811902 | 103.8936427 | 208.3659709 |
| YJL051W | IRC8 | 0.62106934 | 0.96815259 | -0.0810245 | 121.2150047 | 240.6515413 |
| YGR226C | YGR226C | 0.57936751 | 0.96815259 | -0.0808201 | 120.2177894 | 238.754506 |
| YHR126C | YHR126C | 0.34822008 | 0.96815259 | -0.0807852 | 107.0389729 | 214.1614561 |
| YMR295C | YMR295C | 0.5029209 | 0.96815259 | -0.0807406 | 119.1250072 | 236.7015409 |
| YCR089W | FIG2 | 0.4389102 | 0.96815259 | -0.0805962 | 126.5269348 | 250.4849407 |
| YCL050C | APA1 | 0.54976515 | 0.96815259 | -0.0805452 | 119.0698691 | 236.5636852 |
| YIL163C | YIL163C | 0.57501385 | 0.96815259 | -0.0804568 | 121.3668826 | 240.8332315 |
| YHR106W | TRR2 | 0.6528914 | 0.96815259 | -0.0803278 | 121.5842572 | 241.2156673 |
| YDL178W | DLD2 | 0.59256797 | 0.96815259 | -0.0803248 | 116.9012219 | 232.478329 |
| YCR024C-A | PMP1 | 0.62661024 | 0.96815259 | -0.0802749 | 109.2449429 | 218.1856066 |
| YNL040W | YNL040W | 0.41874462 | 0.96815259 | -0.0801325 | 96.49207432 | 194.3679856 |
| YBL025W | RRN10 | 0.44300946 | 0.96815259 | -0.0800316 | 114.9004647 | 228.6931379 |
| YIL132C | CSM2 | 0.51991435 | 0.96815259 | -0.0799864 | 105.2987519 | 210.7718209 |
| YKL072W | STB6 | 0.5387244 | 0.96815259 | -0.0796948 | 106.2062809 | 212.4127099 |
| YJR128W | YJR128W | 0.35345054 | 0.96815259 | -0.0796461 | 97.46954428 | 196.1044733 |
| YGR093W | YGR093W | 0.48583797 | 0.96815259 | -0.0796019 | 95.20037215 | 191.8631237 |
| YDR193W | YDR193W | 0.46635246 | 0.96815259 | -0.079513 | 118.1728813 | 234.7054025 |
| YOR357C | SNX3 | 0.59998805 | 0.96815259 | -0.0793693 | 118.8611282 | 235.9636819 |
| YJR099W | YUH1 | 0.65362497 | 0.96815259 | -0.0793413 | 124.0021943 | 245.549988 |
| YPR114W | YPR114W | 0.56125573 | 0.96815259 | -0.0791189 | 94.09255182 | 189.7098501 |
| YNL077W | APJ1 | 0.27865869 | 0.96815259 | -0.0790919 | 97.07667076 | 195.272274 |
| YKR049C | FMP46 | 0.49139675 | 0.96815259 | -0.0790488 | 112.027114 | 223.156541 |
| YIR037W | HYR1 | 0.58347291 | 0.96815259 | -0.0790376 | 116.5446608 | 231.5826031 |
| YML050W | YML050W | 0.67705602 | 0.97060928 | -0.0789474 | 101.6353552 | 203.7512186 |
| YNL233W | BNI4 | 0.58628703 | 0.96815259 | -0.0787914 | 106.1366607 | 212.1210491 |
| YDR234W | LYS4 | 0.51410778 | 0.96815259 | -0.0786116 | 102.8502879 | 205.9577044 |
| YOR139C | YOR139C | 0.18710166 | 0.96815259 | -0.0786082 | 0 | 14.07654881 |
| YGR286C | BIO2 | 0.47663945 | 0.96815259 | -0.0785886 | 108.4183914 | 216.3416039 |
| YBR014C | YBR014C | 0.56683692 | 0.96815259 | -0.0785068 | 121.0244673 | 239.8452143 |
| YPR054W | SMK1 | 0.66871772 | 0.9703088 | -0.0784751 | 99.01811461 | 198.7838453 |
| YOR118W | RTC5 | 0.64713106 | 0.96815259 | -0.0781015 | 111.8868474 | 222.7252164 |
| YDL201W | TRM8 | 0.53370661 | 0.96815259 | -0.0780044 | 122.3099362 | 242.1534659 |
| YJL163C | YJL163C | 0.54813239 | 0.96815259 | -0.0778201 | 107.708282 | 214.8791775 |
| YDL095W | PMT1 | 0.29089721 | 0.96815259 | -0.0776757 | 113.6943362 | 226.0210862 |
| YGR244C | LSC2 | 0.43421188 | 0.96815259 | -0.0776556 | 110.279374 | 219.6464288 |
| YDR338C | YDR338C | 0.48785784 | 0.96815259 | -0.0776364 | 116.9426231 | 232.0741353 |
| YDR107C | YDR107C | 0.46386133 | 0.96815259 | -0.0775819 | 117.8981194 | 233.8469803 |
| YIL165C | YIL165C | 0.55841485 | 0.96815259 | -0.077524 | 112.4077218 | 223.5935676 |
| YJR124C | YJR124C | 0.59072882 | 0.96815259 | -0.0774557 | 107.8279401 | 215.0371531 |
| YOR058C | ASE1 | 0.54884489 | 0.96815259 | -0.0773786 | 108.7052399 | 216.6600742 |
| YNR042W | YNR042W | 0.54005842 | 0.96815259 | -0.07734 | 108.2361648 | 215.7780461 |
| YDL094C | YDL094C | 0.57243371 | 0.96815259 | -0.0770989 | 120.4197713 | 238.4649606 |
| YDR056C | YDR056C | 0.47990848 | 0.96815259 | -0.0765649 | 123.8391198 | 244.7485772 |
| YOL092W | YOL092W | 0.71173923 | 0.97135148 | -0.0764692 | 104.585325 | 208.8109868 |
| YKL176C | LST4 | 0.64985199 | 0.96815259 | -0.0761243 | 96.4366701 | 193.5468601 |
| YKL064W | MNR2 | 0.58232078 | 0.96815259 | -0.0760173 | 105.1140563 | 209.7164738 |
| YIL065C | FIS1 | 0.53754082 | 0.96815259 | -0.0760132 | 94.22394041 | 189.3988212 |
| YJR108W | ABM1 | 0.49895858 | 0.96815259 | -0.0759962 | 110.6577265 | 220.0551462 |
| YDR316W | OMS1 | 0.48093727 | 0.96815259 | -0.0757668 | 107.3805748 | 213.9001105 |
| YDR401W | YDR401W | 0.59812695 | 0.96815259 | -0.0753319 | 127.9963174 | 252.2835694 |
| YDR294C | DPL1 | 0.54954757 | 0.96815259 | -0.0752796 | 111.8657334 | 222.1805031 |

| | | | | | | |
|---|---|---|---|---|---|---|
| YLR190W | MMR1 | 0.14459718 | 0.96815259 | -0.0751627 | 69.82906449 | 143.7347256 |
| YPL030W | TRM44 | 0.65859873 | 0.96815259 | -0.0751587 | 108.4899862 | 215.8609685 |
| YDR372C | VPS74 | 0.5259473 | 0.96815259 | -0.0749618 | 114.2613132 | 226.5928593 |
| YPL159C | PET20 | 0.57891948 | 0.96815259 | -0.0748923 | 85.98325366 | 173.8240383 |
| YNL157W | IGO1 | 0.59764834 | 0.96815259 | -0.0748554 | 126.3336909 | 249.0963953 |
| YDL185W | TFP1 | 0.47423536 | 0.96815259 | -0.0747736 | 120.6845464 | 238.5425327 |
| YGR270W | YTA7 | 0.63126869 | 0.96815259 | -0.0746975 | 86.56399308 | 174.8726008 |
| YGL115W | SNF4 | 0.81070931 | 0.9762419 | -0.0745531 | 61.95340499 | 128.9324919 |
| YKR087C | OMA1 | 0.53780348 | 0.96815259 | -0.0744832 | 115.4687049 | 228.7597094 |
| YMR233W | YMR233W | 0.34601817 | 0.96815259 | -0.0744161 | 114.2260157 | 226.4292857 |
| YJR134C | SGM1 | 0.60486354 | 0.96815259 | -0.0741579 | 120.170421 | 237.4731088 |
| YMR271C | URA10 | 0.56993924 | 0.96815259 | -0.0741205 | 116.5859769 | 230.7791734 |
| YDR269C | YDR269C | 0.16836992 | 0.96815259 | -0.0740691 | 100.5219485 | 200.8004379 |
| YML074C | FPR3 | 0.39481739 | 0.96815259 | -0.0739392 | 113.6913269 | 225.346353 |
| YFR011C | YFR011C | 0.61656653 | 0.96815259 | -0.073806 | 101.761546 | 203.0659489 |
| YNL236W | SIN4 | 0.1817059 | 0.96815259 | -0.0736676 | 0 | 13.19183114 |
| YIL045W | PIG2 | 0.58644315 | 0.96815259 | -0.0736251 | 120.3795661 | 237.7678825 |
| YDR093W | DNF2 | 0.52740567 | 0.96815259 | -0.0736077 | 123.8459291 | 244.231732 |
| YJR059W | PTK2 | 0.5335721 | 0.96815259 | -0.073467 | 107.3047131 | 213.3467565 |
| YPR077C | YPR077C | 0.53381049 | 0.96815259 | -0.0734455 | 117.4305329 | 232.233918 |
| YLR423C | ATG17 | 0.46482661 | 0.96815259 | -0.0734317 | 112.5377121 | 223.1032591 |
| YDR144C | MKC7 | 0.53833486 | 0.96815259 | -0.0733917 | 118.2459042 | 233.7454813 |
| YPL145C | KES1 | 0.67493488 | 0.97060928 | -0.0732048 | 102.6356933 | 204.5891284 |
| YIL070C | MAM33 | 0.55143936 | 0.96815259 | -0.0731406 | 123.6091409 | 243.7063163 |
| YDL129W | YDL129W | 0.4614898 | 0.96815259 | -0.0730299 | 120.2587378 | 237.4358808 |
| YGL254W | FZF1 | 0.51966954 | 0.96815259 | -0.0730191 | 101.5088301 | 202.4535626 |
| YMR161W | HLJ1 | 0.58510907 | 0.96815259 | -0.0729888 | 114.0733722 | 225.8889211 |
| YDR051C | YDR051C | 0.38851717 | 0.96815259 | -0.0729714 | 109.2023679 | 216.7983258 |
| YBR006W | UGA2 | 0.49619118 | 0.96815259 | -0.0728261 | 98.32390449 | 196.4771105 |
| YPR170C | YPR170C | 0.66398273 | 0.96977183 | -0.0725266 | 96.81189909 | 193.6026521 |
| YIL053W | RHR2 | 0.65388716 | 0.96815259 | -0.0725031 | 108.3771207 | 215.1748536 |
| YDL167C | NRP1 | 0.37469651 | 0.96815259 | -0.0724934 | 122.472405 | 241.4697018 |
| YDR344C | YDR344C | 0.58143688 | 0.96815259 | -0.0722704 | 120.3545637 | 237.4786568 |
| YJR119C | JHD2 | 0.35772782 | 0.96815259 | -0.0721627 | 105.3605957 | 209.4861877 |
| YKL174C | TPO5 | 0.54736348 | 0.96815259 | -0.0721244 | 112.0515901 | 221.9622402 |
| YNL010W | YNL010W | 0.41533801 | 0.96815259 | -0.0720127 | 97.45480315 | 194.7100451 |
| YBR248C | HIS7 | 0.04361066 | 0.96815259 | -0.0719482 | 97.61572719 | 194.9987121 |
| YDR388W | RVS167 | 0.61767087 | 0.96815259 | -0.0718675 | 93.71543621 | 187.7077669 |
| YGL004C | RPN14 | 0.52659205 | 0.96815259 | -0.0718625 | 116.3445876 | 229.9244883 |
| YKL131W | YKL131W | 0.67479024 | 0.97060928 | -0.0718062 | 115.1517646 | 227.6890419 |
| YOR265W | RBL2 | 0.55785354 | 0.96815259 | -0.0717873 | 103.6285273 | 206.1875546 |
| YJL089W | SIP4 | 0.62785318 | 0.96815259 | -0.0713187 | 127.6108664 | 250.8458045 |
| YGL110C | CUE3 | 0.6024135 | 0.96815259 | -0.07126 | 113.6224813 | 224.7381507 |
| YJL137C | GLG2 | 0.44899927 | 0.96815259 | -0.0712506 | 119.7133285 | 236.09973 |
| YDR020C | YDR020C | 0.60044413 | 0.96815259 | -0.0709778 | 113.9208387 | 225.2442369 |
| YPR194C | OPT2 | 0.41784998 | 0.96815259 | -0.0707715 | 73.33659031 | 149.4921305 |
| YKL117W | SBA1 | 0.56089883 | 0.96815259 | -0.0707136 | 110.7560034 | 219.2925166 |
| YNL314W | DAL82 | 0.65996216 | 0.96815259 | -0.0706578 | 112.6610254 | 222.8365968 |
| YDL035C | GPR1 | 0.77017406 | 0.97246744 | -0.0705721 | 112.7964309 | 223.0738672 |
| YDR075W | PPH3 | 0.70427501 | 0.97135148 | -0.0702595 | 122.9989593 | 242.0520153 |
| YMR101C | SRT1 | 0.48799036 | 0.96815259 | -0.0702505 | 114.5149926 | 226.2224706 |
| YCR083W | TRX3 | 0.50503492 | 0.96815259 | -0.0701921 | 111.3291455 | 220.268412 |
| YGR044C | RME1 | 0.62719514 | 0.96815259 | -0.0699256 | 116.7394475 | 230.3142969 |
| YNL129W | NRK1 | 0.65894742 | 0.96815259 | -0.0698744 | 117.9851334 | 232.6291129 |
| YHR161C | YAP1801 | 0.72779824 | 0.97135148 | -0.0698301 | 115.1883056 | 227.4033389 |
| YDR163W | CWC15 | 0.5585624 | 0.96815259 | -0.069494 | 115.3246153 | 227.5974645 |
| YDR009W | GAL3 | 0.55366312 | 0.96815259 | -0.0694346 | 116.8030179 | 230.3449663 |
| YNL130C | CPT1 | 0.76704673 | 0.97246744 | -0.0693841 | 119.1382398 | 234.6925879 |
| YBR090C | YBR090C | 0.55365603 | 0.96815259 | -0.0693395 | 123.0500691 | 241.9826336 |
| YLR446W | YLR446W | 0.43682447 | 0.96815259 | -0.0692734 | 95.13873077 | 189.8985732 |
| YJR149W | YJR149W | 0.4853671 | 0.96815259 | -0.0691666 | 112.7386684 | 222.7144084 |
| YHR017W | YSC83 | 0.59987327 | 0.96815259 | -0.0691623 | 120.7685436 | 237.6944147 |
| YDR005C | MAF1 | 0.60226972 | 0.96815259 | -0.0690893 | 109.9148114 | 217.4322924 |
| YDR274C | YDR274C | 0.68228101 | 0.97060928 | -0.0690605 | 108.2123349 | 214.2509529 |

| | | | | | | |
|---|---|---|---|---|---|---|
| YML122C | YML122C | 0.30333342 | 0.96815259 | -0.0688666 | 103.8683695 | 206.1120068 |
| YLR239C | LIP2 | 0.82730779 | 0.97923902 | -0.0688054 | 30.28738206 | 68.82618228 |
| YIL120W | QDR1 | 0.6226414 | 0.96815259 | -0.0686081 | 113.7531053 | 224.5069568 |
| YJL119C | YJL119C | 0.86316787 | 0.98248371 | -0.067958 | 93.47882137 | 186.5662402 |
| YGR287C | YGR287C | 0.65050978 | 0.96815259 | -0.0679441 | 117.0507729 | 230.5402914 |
| YHR136C | SPL2 | 0.69726157 | 0.97135148 | -0.0679082 | 132.8041509 | 259.9238174 |
| YDR077W | SED1 | 0.54620637 | 0.96815259 | -0.0678787 | 131.6357713 | 257.7387833 |
| YMR280C | CAT8 | 0.58905262 | 0.96815259 | -0.0677468 | 105.0992038 | 208.2077521 |
| YDL114W | YDL114W | 0.49442971 | 0.96815259 | -0.0677259 | 118.8924863 | 233.9371588 |
| YDL176W | YDL176W | 0.68435322 | 0.97080037 | -0.0677183 | 117.4968282 | 231.3320255 |
| YJL201W | ECM25 | 0.58200204 | 0.96815259 | -0.0676906 | 126.2402457 | 247.6390341 |
| YOR126C | IAH1 | 0.49465019 | 0.96815259 | -0.067498 | 106.6675306 | 211.0891245 |
| YML096W | YML096W | 0.22747279 | 0.96815259 | -0.0674795 | 99.30640672 | 197.3526704 |
| YFL011W | HXT10 | 0.55682878 | 0.96815259 | -0.0674563 | 117.9062789 | 232.0489887 |
| YBR043C | QDR3 | 0.59094924 | 0.96815259 | -0.067401 | 104.0510219 | 206.190311 |
| YKL062W | MSN4 | 0.61053768 | 0.96815259 | -0.0673949 | 107.5825631 | 212.7777657 |
| YDL155W | CLB3 | 0.48870281 | 0.96815259 | -0.0672826 | 112.6635752 | 222.236951 |
| YJL157C | FAR1 | 0.56175998 | 0.96815259 | -0.067239 | 117.6783282 | 231.5848107 |
| YBR223C | TDP1 | 0.37078301 | 0.96815259 | -0.0672276 | 128.354115 | 251.4998285 |
| YJR031C | GEA1 | 0.46556806 | 0.96815259 | -0.0671645 | 114.3182541 | 225.3028205 |
| YHR043C | DOG2 | 0.78034796 | 0.97246744 | -0.0670813 | 124.9528451 | 245.1281227 |
| YKL120W | OAC1 | 0.28556633 | 0.96815259 | -0.0669818 | 110.0390467 | 217.2866768 |
| YML058C-A | YML058C-A | 0.63968719 | 0.96815259 | -0.0669486 | 108.5821702 | 214.5627417 |
| YJR005W | APL1 | 0.75350353 | 0.97135148 | -0.0668904 | 121.730054 | 239.0814056 |
| YER130C | YER130C | 0.67328367 | 0.97060928 | -0.0668656 | 121.0758939 | 237.8565366 |
| YDL131W | LYS21 | 0.66028601 | 0.96815259 | -0.0667338 | 112.4162825 | 221.6773046 |
| YDL018C | ERP3 | 0.68857536 | 0.97133463 | -0.0665367 | 107.8154438 | 213.0585523 |
| YNL063W | MTQ1 | 0.65137829 | 0.96815259 | -0.0664934 | 125.2655921 | 245.6063147 |
| YIL095W | PRK1 | 0.68751231 | 0.97133463 | -0.0664647 | 119.5914346 | 235.0153039 |
| YGL132W | YGL132W | 0.38681477 | 0.96815259 | -0.0664561 | 106.3603994 | 210.3295469 |
| YKR076W | ECM4 | 0.54900967 | 0.96815259 | -0.0663659 | 115.5396788 | 227.438538 |
| YCL024W | KCC4 | 0.49354881 | 0.96815259 | -0.066139 | 107.1334248 | 211.7149499 |
| YKR036C | CAF4 | 0.2825152 | 0.96815259 | -0.0660205 | 96.82815247 | 192.4679092 |
| YKR096W | YKR096W | 0.56255783 | 0.96815259 | -0.0658968 | 116.3662985 | 228.8967033 |
| YGR084C | MRP13 | 0.57039322 | 0.96815259 | -0.0654988 | 111.95472 | 220.59505 |
| YML012W | ERV25 | 0.6380654 | 0.96815259 | -0.0654923 | 109.0733875 | 215.2183892 |
| YAL005C | SSA1 | 0.76459686 | 0.9713547 | -0.0653768 | 99.73845625 | 197.7821762 |
| YKL218C | SRY1 | 0.64967385 | 0.96815259 | -0.0652374 | 107.5404359 | 212.3128309 |
| YPL066W | YPL066W | 0.53719694 | 0.96815259 | -0.0651758 | 103.4861893 | 204.738065 |
| YBR238C | YBR238C | 0.63802851 | 0.96815259 | -0.0651201 | 117.7690719 | 231.3746597 |
| YJR038C | YJR038C | 0.62198792 | 0.96815259 | -0.0650537 | 109.8115456 | 216.5169797 |
| YBR290W | BSD2 | 0.29035263 | 0.96815259 | -0.0650516 | 106.078891 | 209.5528589 |
| YCR010C | ADY2 | 0.58255665 | 0.96815259 | -0.0650152 | 115.2048393 | 226.5719811 |
| YJR127C | RSF2 | 0.45551674 | 0.96815259 | -0.0649866 | 107.3964656 | 211.9993226 |
| YFR019W | FAB1 | 0.64102634 | 0.96815259 | -0.064956 | 123.2258769 | 241.5256634 |
| YIL076W | SEC28 | 0.64394216 | 0.96815259 | -0.064899 | 94.97989526 | 188.81891 |
| YDR251W | PAM1 | 0.6048508 | 0.96815259 | -0.0648092 | 125.9526678 | 246.5865414 |
| YBR260C | RGD1 | 0.49786207 | 0.96815259 | -0.064698 | 116.5046873 | 228.9402139 |
| YPR173C | VPS4 | 0.53219742 | 0.96815259 | -0.0645702 | 82.40026135 | 165.2911037 |
| YDL224C | WHI4 | 0.59469707 | 0.96815259 | -0.0645181 | 123.5267877 | 242.0086222 |
| YMR252C | YMR252C | 0.66665251 | 0.9703088 | -0.0645082 | 122.8546423 | 240.7528803 |
| YPL262W | FUM1 | 0.53205785 | 0.96815259 | -0.0643253 | 90.47473402 | 180.3112198 |
| YOL093W | TRM10 | 0.62830563 | 0.96815259 | -0.0642958 | 88.67664221 | 176.9513551 |
| YHL026C | YHL026C | 0.63488472 | 0.96815259 | -0.064237 | 104.1879189 | 205.8791215 |
| YGL235W | YGL235W | 0.40935641 | 0.96815259 | -0.0641461 | 92.93005583 | 184.859847 |
| YPR095C | SYT1 | 0.7094375 | 0.97135148 | -0.0641309 | 118.230211 | 232.0578509 |
| YJR083C | ACF4 | 0.74405559 | 0.97135148 | -0.0640801 | 122.6190498 | 240.2366964 |
| YGR203W | YGR203W | 0.72531462 | 0.97135148 | -0.0639493 | 131.6301969 | 257.0247335 |
| YDR345C | HXT3 | 0.57112202 | 0.96815259 | -0.0639445 | 124.3438441 | 243.4302321 |
| YMR182C | RGM1 | 0.76900926 | 0.97246744 | -0.0638312 | 118.090647 | 231.7438076 |
| YFR047C | BNA6 | 0.64558218 | 0.96815259 | -0.0638154 | 110.7575695 | 218.0601642 |
| YER096W | SHC1 | 0.72701866 | 0.97135148 | -0.063522 | 125.4358228 | 245.3918069 |
| YJR052W | RAD7 | 0.65708384 | 0.96815259 | -0.0633978 | 113.7939683 | 223.6501816 |
| YOR374W | ALD4 | 0.66823895 | 0.9703088 | -0.0633921 | 106.7692253 | 210.5435849 |

| | | | | | | |
|---|---|---|---|---|---|---|
| YJL191W | RPS14B | 0.6320225 | 0.96815259 | -0.0632416 | 116.7385817 | 229.1157725 |
| YPR145W | ASN1 | 0.76064349 | 0.97135148 | -0.0632102 | 96.77722902 | 191.8696618 |
| YMR207C | HFA1 | 0.18943045 | 0.96815259 | -0.063134 | 85.33768613 | 170.5140551 |
| YKL046C | DCW1 | 0.37576893 | 0.96815259 | -0.06309 | 98.31877585 | 194.7240921 |
| YJR142W | YJR142W | 0.68257396 | 0.97060928 | -0.0630425 | 129.1583645 | 252.2508284 |
| YPL071C | YPL071C | 0.6815392 | 0.97060928 | -0.0630341 | 111.7699028 | 219.808889 |
| YDR321W | ASP1 | 0.62376501 | 0.96815259 | -0.0630156 | 120.9994603 | 237.0245079 |
| YGR079W | YGR079W | 0.40670536 | 0.96815259 | -0.0629596 | 89.05892949 | 177.42529 |
| YDL214C | PRR2 | 0.57017875 | 0.96815259 | -0.062717 | 118.582642 | 232.4621547 |
| YJL193W | YJL193W | 0.67729651 | 0.97060928 | -0.0627151 | 114.0059528 | 223.923414 |
| YDR210W | YDR210W | 0.64569059 | 0.96815259 | -0.0625173 | 126.6391246 | 247.4568053 |
| YDL223C | HBT1 | 0.57135217 | 0.96815259 | -0.0624495 | 119.5506973 | 234.2202818 |
| YIL101C | XBP1 | 0.67444436 | 0.97060928 | -0.062448 | 120.7857485 | 236.5241637 |
| YDL088C | ASM4 | 0.62555988 | 0.96815259 | -0.0622638 | 117.4931162 | 230.3483443 |
| YKL217W | JEN1 | 0.46706336 | 0.96815259 | -0.0617401 | 95.29676406 | 188.8444001 |
| YOR121C | YOR121C | 0.71175344 | 0.97135148 | -0.0617315 | 112.7605341 | 221.4237782 |
| YHR131C | YHR131C | 0.77580285 | 0.97246744 | -0.0616797 | 120.5290971 | 235.9077736 |
| YNL338W | YNL338W | 0.6794856 | 0.97060928 | -0.0616515 | 114.8649543 | 225.3355338 |
| YJR025C | BNA1 | 0.5963207 | 0.96815259 | -0.0616467 | 104.5254884 | 206.045054 |
| YKL007W | CAP1 | 0.64577552 | 0.96815259 | -0.061642 | 103.9444688 | 204.9602429 |
| YKL128C | PMU1 | 0.54272837 | 0.96815259 | -0.0613854 | 103.6300005 | 204.327612 |
| YGR133W | PEX4 | 0.64700663 | 0.96815259 | -0.0612569 | 104.4498567 | 205.8341559 |
| YIL011W | TIR3 | 0.80404328 | 0.9762419 | -0.0612433 | 145.2368449 | 281.9251267 |
| YDL197C | ASF2 | 0.52818351 | 0.96815259 | -0.0612062 | 114.4236217 | 224.432415 |
| YOR223W | YOR223W | 0.6140038 | 0.96815259 | -0.0611971 | 109.4198205 | 215.0955573 |
| YHR195W | NVJ1 | 0.67667084 | 0.97060928 | -0.060799 | 114.2105016 | 223.9619022 |
| YMR088C | VBA1 | 0.77160028 | 0.97246744 | -0.0607831 | 123.4038699 | 241.1104711 |
| YJL083W | TAX4 | 0.65414411 | 0.96815259 | -0.0606968 | 117.6956955 | 230.4456791 |
| YMR266W | RSN1 | 0.62949209 | 0.96815259 | -0.0600545 | 120.5725286 | 235.6977711 |
| YPR167C | MET16 | 0.88221203 | 0.98248371 | -0.0599842 | 96.7445311 | 191.2309637 |
| YOR359W | VTS1 | 0.77567297 | 0.97246744 | -0.0596648 | 108.8116798 | 213.686593 |
| YMR003W | YMR003W | 0.55904205 | 0.96815259 | -0.059594 | 112.8497331 | 221.2074299 |
| YHR171W | ATG7 | 0.72671817 | 0.97135148 | -0.0594674 | 99.80021434 | 196.8391944 |
| YGL042C | YGL042C | 0.7531879 | 0.97135148 | -0.0594331 | 96.17937002 | 190.0778968 |
| YKL177W | YKL177W | 0.63001405 | 0.96815259 | -0.0592297 | 115.9444702 | 226.9158353 |
| YDL180W | YDL180W | 0.72944396 | 0.97135148 | -0.0590104 | 120.7552153 | 235.8516211 |
| YOR325W | YOR325W | 0.74774211 | 0.97135148 | -0.0589531 | 96.56053587 | 190.7030604 |
| YKL185W | ASH1 | 0.62429161 | 0.96815259 | -0.0588892 | 108.9880913 | 213.8768199 |
| YOR299W | BUD7 | 0.60492211 | 0.96815259 | -0.0587773 | 102.1230843 | 201.0492302 |
| YNR019W | ARE2 | 0.62423296 | 0.96815259 | -0.0587055 | 99.33871404 | 195.841761 |
| YIL100W | YIL100W | 0.70999863 | 0.97135148 | -0.0586357 | 125.1758698 | 244.0318208 |
| YHL024W | RIM4 | 0.75730284 | 0.97135148 | -0.0585992 | 107.1911516 | 210.472474 |
| YML003W | YML003W | 0.80684164 | 0.9762419 | -0.058389 | 112.2323931 | 219.8399132 |
| YKR089C | TGL4 | 0.6782455 | 0.97060928 | -0.0582968 | 110.8545056 | 217.2527754 |
| YHR193C | EGD2 | 0.79928118 | 0.9762419 | -0.0581172 | 134.4651868 | 261.2694089 |
| YDR259C | YAP6 | 0.65246684 | 0.96815259 | -0.0581085 | 117.7497828 | 230.0830977 |
| YIR013C | GAT4 | 0.62688492 | 0.96815259 | -0.0579874 | 112.7623066 | 220.7566291 |
| YDR379W | RGA2 | 0.57025788 | 0.96815259 | -0.0578686 | 115.117448 | 225.1291789 |
| YER115C | SPR6 | 0.75228101 | 0.97135148 | -0.0578534 | 102.2098026 | 201.0455745 |
| YNR049C | MSO1 | 0.764008 | 0.97135148 | -0.0575869 | 104.7252504 | 205.6907423 |
| YHR045W | YHR045W | 0.84229544 | 0.98035418 | -0.0574691 | 108.0264389 | 211.8284464 |
| YBL065W | YBL065W | 0.49287785 | 0.96815259 | -0.0574571 | 116.2571536 | 227.1817635 |
| YJL030W | MAD2 | 0.63180289 | 0.96815259 | -0.0574515 | 113.780222 | 222.5597188 |
| YDL199C | YDL199C | 0.65137208 | 0.96815259 | -0.0573848 | 128.036184 | 249.1441216 |
| YJR130C | STR2 | 0.73279917 | 0.97135148 | -0.0572353 | 105.4884665 | 207.0516621 |
| YKL051W | SFK1 | 0.58516387 | 0.96815259 | -0.0571842 | 106.0984433 | 208.1804997 |
| YBR296C | PHO89 | 0.54178909 | 0.96815259 | -0.0570824 | 121.1328843 | 236.210967 |
| YJL042W | MHP1 | 0.66275099 | 0.96927465 | -0.0569909 | 106.2339465 | 208.3986826 |
| YCL010C | SGF29 | 0.39100222 | 0.96815259 | -0.0569833 | 0 | 10.20413957 |
| YDR151C | CTH1 | 0.6688668 | 0.9703088 | -0.0568271 | 116.1908979 | 226.9453247 |
| YGR250C | YGR250C | 0.74394639 | 0.97135148 | -0.056788 | 116.0832258 | 226.737453 |
| YER113C | YER113C | 0.7282214 | 0.97135148 | -0.0564949 | 118.3158717 | 230.8502539 |
| YPR189W | SKI3 | 0.73765523 | 0.97135148 | -0.056428 | 101.295273 | 199.0841417 |
| YGR241C | YAP1802 | 0.80881237 | 0.9762419 | -0.0563806 | 117.7181481 | 229.7146573 |

| | | | | | | |
|---|---|---|---|---|---|---|
| YIR036C | IRC24 | 0.76244427 | 0.97135148 | -0.0563152 | 119.491243 | 233.0108851 |
| YPL017C | IRC15 | 0.77554441 | 0.97246744 | -0.0562672 | 106.7229256 | 209.1813457 |
| YCL049C | YCL049C | 0.51688341 | 0.96815259 | -0.0562581 | 110.6191726 | 216.4486687 |
| YOR301W | RAX1 | 0.70868287 | 0.97135148 | -0.0560706 | 115.2732959 | 225.0979494 |
| YKR040C | YKR040C | 0.52808053 | 0.96815259 | -0.0559625 | 99.83923626 | 196.2843566 |
| YDR152W | GIR2 | 0.65140381 | 0.96815259 | -0.0557713 | 113.8153353 | 222.3243503 |
| YML100W-A | YML100W-A | 0.73818558 | 0.97135148 | -0.0557468 | 115.8402018 | 226.0976151 |
| YDR096W | GIS1 | 0.66972191 | 0.97060928 | -0.0556778 | 117.7561384 | 229.6596889 |
| YGL140C | YGL140C | 0.65365941 | 0.96815259 | -0.0556297 | 122.1008505 | 237.7566885 |
| YPL135W | ISU1 | 0.78931795 | 0.97272554 | -0.0555988 | 119.9071443 | 233.6585114 |
| YMR041C | YMR041C | 0.48938209 | 0.96815259 | -0.0555635 | 119.6966477 | 233.2594774 |
| YLR332W | MID2 | 0.64580325 | 0.96815259 | -0.0555341 | 111.1181282 | 217.2498896 |
| YGR271W | SLH1 | 0.69945352 | 0.97135148 | -0.0554809 | 100.3082191 | 197.0730764 |
| YBR026C | ETR1 | 0.64065265 | 0.96815259 | -0.055453 | 98.73916087 | 194.1407974 |
| YMR155W | YMR155W | 0.53582063 | 0.96815259 | -0.0553209 | 96.79484679 | 190.4897606 |
| YIL052C | RPL34B | 0.47320911 | 0.96815259 | -0.0552336 | 104.0982518 | 204.0995747 |
| YLR080W | EMP46 | 0.72556259 | 0.97135148 | -0.0551228 | 105.2242395 | 206.1804183 |
| YOL116W | MSN1 | 0.76565325 | 0.97212154 | -0.0550534 | 112.3261593 | 219.4175439 |
| YCR101C | YCR101C | 0.42910752 | 0.96815259 | -0.0548934 | 110.0898232 | 215.2167163 |
| YKR099W | BAS1 | 0.71672091 | 0.97135148 | -0.0545343 | 120.2975095 | 234.1961754 |
| YJL141C | YAK1 | 0.73945734 | 0.97135148 | -0.054515 | 116.5982598 | 227.2912752 |
| YDL179W | PCL9 | 0.7459619 | 0.97135148 | -0.0544534 | 122.8928263 | 239.0235753 |
| YDL231C | BRE4 | 0.44854771 | 0.96815259 | -0.0543061 | 110.2881263 | 215.4815103 |
| YGR275W | RTT102 | 0.72486781 | 0.97135148 | -0.0542772 | 121.4990008 | 236.3916686 |
| YDR335W | MSN5 | 0.77525179 | 0.97246744 | -0.0542643 | 117.9520948 | 229.7721514 |
| YJL116C | NCA3 | 0.56237493 | 0.96815259 | -0.0540912 | 128.1446564 | 248.7566891 |
| YBL049W | MOH1 | 0.63297209 | 0.96815259 | -0.0535009 | 123.9047144 | 240.7408269 |
| YKL097C | YKL097C | 0.69118441 | 0.97135148 | -0.0530624 | 106.0661876 | 207.3822125 |
| YIL170W | HXT12 | 0.74619321 | 0.97135148 | -0.0529929 | 125.2291231 | 243.1207023 |
| YMR010W | YMR010W | 0.47010357 | 0.96815259 | -0.0528125 | 99.78108518 | 195.6117923 |
| YJL171C | YJL171C | 0.70240898 | 0.97135148 | -0.0526763 | 123.3057759 | 239.4757716 |
| YMR165C | PAH1 | 0.86970783 | 0.98248371 | -0.0526657 | 28.58114894 | 62.75280377 |
| YNL050C | YNL050C | 0.58286383 | 0.96815259 | -0.0525383 | 98.34862046 | 192.8902514 |
| YDR131C | YDR131C | 0.65257032 | 0.96815259 | -0.052347 | 119.8629345 | 232.993728 |
| YNL212W | VID27 | 0.67602687 | 0.97060928 | -0.0522733 | 111.2956654 | 216.9971819 |
| YBR151W | APD1 | 0.77860522 | 0.97246744 | -0.0522421 | 117.6275163 | 228.8044849 |
| YKL050C | YKL050C | 0.66983412 | 0.97060928 | -0.0520668 | 107.592235 | 210.0509686 |
| YER145C | FTR1 | 0.75585242 | 0.97135148 | -0.0519868 | 112.1578112 | 218.5543238 |
| YLL020C | YLL020C | 0.56673762 | 0.96815259 | -0.051934 | 111.7904455 | 217.859501 |
| YJL123C | MTC1 | 0.67440485 | 0.97060928 | -0.0518618 | 117.8197822 | 229.0950686 |
| YLR146C | SPE4 | 0.70591613 | 0.97135148 | -0.0518342 | 98.21494366 | 192.5147619 |
| YLR118C | YLR118C | 0.69501806 | 0.97135148 | -0.0517808 | 110.1921295 | 214.8502019 |
| YNL065W | AQR1 | 0.64244861 | 0.96815259 | -0.0515592 | 99.98188793 | 195.7619891 |
| YDR368W | YPR1 | 0.61248484 | 0.96815259 | -0.0515514 | 118.1828349 | 229.7168117 |
| YKL107W | YKL107W | 0.58419312 | 0.96815259 | -0.0514692 | 108.6902036 | 211.9923548 |
| YIR001C | SGN1 | 0.747654 | 0.97135148 | -0.051429 | 110.2079456 | 214.8167143 |
| YIL044C | AGE2 | 0.63339677 | 0.96815259 | -0.0513814 | 100.384854 | 196.4819301 |
| YIL097W | FYV10 | 0.62076355 | 0.96815259 | -0.0513676 | 97.31662503 | 190.7552774 |
| YCL027W | FUS1 | 0.50816861 | 0.96815259 | -0.0513514 | 121.3925362 | 235.6691091 |
| YDL174C | DLD1 | 0.55499166 | 0.96815259 | -0.0513042 | 105.5939236 | 206.1863091 |
| YDR272W | GLO2 | 0.68819459 | 0.97133463 | -0.0512861 | 124.2303174 | 240.9516684 |
| YHL009C | YAP3 | 0.78875412 | 0.97272554 | -0.0512604 | 118.1987499 | 229.6943932 |
| YMR297W | PRC1 | 0.6244037 | 0.96815259 | -0.0512412 | 113.7472996 | 221.3862067 |
| YOR101W | RAS1 | 0.74083392 | 0.97135148 | -0.0511856 | 118.4446173 | 230.1397001 |
| YNL041C | COG6 | 0.7001876 | 0.97135148 | -0.0511448 | 99.45294902 | 194.7009809 |
| YDR403W | DIT1 | 0.62225757 | 0.96815259 | -0.0511074 | 120.9788775 | 234.853686 |
| YHR129C | ARP1 | 0.78316101 | 0.97260548 | -0.0509838 | 111.013866 | 216.24054 |
| YJL139C | YUR1 | 0.72940124 | 0.97135148 | -0.0509502 | 114.9016065 | 223.4876052 |
| YBR281C | DUG2 | 0.74929009 | 0.97135148 | -0.0508722 | 105.4310387 | 205.8050699 |
| YPR039W | YPR039W | 0.436897 | 0.96815259 | -0.0508498 | 111.5060284 | 217.1347256 |
| YLR338W | YLR338W | 0.25496312 | 0.96815259 | -0.0508071 | 67.87089148 | 135.7200875 |
| YIR018W | YAP5 | 0.61072295 | 0.96815259 | -0.0507359 | 115.2903268 | 224.1744264 |
| YIL072W | HOP1 | 0.66141732 | 0.96866506 | -0.0507319 | 119.1942359 | 231.4569716 |
| YHL036W | MUP3 | 0.80306388 | 0.9762419 | -0.0505782 | 122.8295898 | 238.2116707 |

| | | | | | | |
|---|---|---|---|---|---|---|
| YHL047C | ARN2 | 0.66813034 | 0.9703088 | -0.050494 | 112.7347739 | 219.3634067 |
| YLR451W | LEU3 | 0.16758791 | 0.96815259 | -0.0502262 | 94.17525487 | 184.6902547 |
| YCL028W | RNQ1 | 0.69429513 | 0.97135148 | -0.0502136 | 122.7040072 | 237.9120887 |
| YDR444W | YDR444W | 0.90940429 | 0.98520231 | -0.0501213 | 81.85825996 | 161.6925197 |
| YDL161W | ENT1 | 0.63054236 | 0.96815259 | -0.0500055 | 113.0057984 | 219.7815553 |
| YPR193C | HPA2 | 0.73425384 | 0.97135148 | -0.0498851 | 118.2289461 | 229.5044498 |
| YNL074C | MLF3 | 0.77860107 | 0.97246744 | -0.04984 | 104.9880615 | 204.7937964 |
| YLL006W | MMM1 | 0.91604912 | 0.98520231 | -0.0497982 | 31.4697917 | 67.62845704 |
| YDL048C | STP4 | 0.60346856 | 0.96815259 | -0.0497667 | 116.9111678 | 227.0247622 |
| YDR263C | DIN7 | 0.73955176 | 0.97135148 | -0.0497633 | 122.7731076 | 237.9603577 |
| YJL101C | GSH1 | 0.91744274 | 0.98520231 | -0.0495042 | 43.76787992 | 90.51948601 |
| YOL103W | ITR2 | 0.59786151 | 0.96815259 | -0.0493401 | 103.5270747 | 201.9786182 |
| YLR093C | NYV1 | 0.60155722 | 0.96815259 | -0.0492736 | 88.75205207 | 174.4019877 |
| YIL016W | SNL1 | 0.83946095 | 0.98035418 | -0.0487666 | 121.0719942 | 234.6082434 |
| YNR025C | YNR025C | 0.82852641 | 0.97952308 | -0.0487067 | 109.9886357 | 213.9200616 |
| YBL081W | YBL081W | 0.69751962 | 0.97135148 | -0.0486565 | 105.3417445 | 205.2416926 |
| YMR232W | FUS2 | 0.80860306 | 0.9762419 | -0.0486338 | 112.8602892 | 219.264451 |
| YPL081W | RPS9A | 0.77860915 | 0.97246744 | -0.0483556 | 116.3210463 | 225.6711191 |
| YKR010C | TOF2 | 0.31124169 | 0.96815259 | -0.0483297 | 90.02775284 | 176.6129455 |
| YLL007C | YLL007C | 0.70609004 | 0.97135148 | -0.0482769 | 108.8552238 | 211.728579 |
| YPL150W | YPL150W | 0.71629229 | 0.97135148 | -0.048269 | 114.7228059 | 222.6738895 |
| YOL091W | SPO21 | 0.69407318 | 0.97135148 | -0.048144 | 101.7603749 | 198.4684173 |
| YNL155W | YNL155W | 0.20698869 | 0.96815259 | -0.0480773 | 88.63214177 | 173.9640476 |
| YNL173C | MDG1 | 0.86995792 | 0.98248371 | -0.0478275 | 116.3896748 | 225.7045888 |
| YKL085W | MDH1 | 0.46921461 | 0.96815259 | -0.0476883 | 105.265821 | 204.9266848 |
| YJL208C | NUC1 | 0.63976455 | 0.96815259 | -0.0475857 | 116.5882095 | 226.0316854 |
| YKR027W | BCH2 | 0.57365895 | 0.96815259 | -0.0474064 | 73.57001556 | 145.743576 |
| YGR092W | DBF2 | 0.66341365 | 0.96927465 | -0.0473686 | 87.89113479 | 172.4546958 |
| YCR092C | MSH3 | 0.7184754 | 0.97135148 | -0.0472138 | 129.467628 | 249.9933111 |
| YKR077W | YKR077W | 0.60036424 | 0.96815259 | -0.0471547 | 99.94696808 | 194.9081154 |
| YHR075C | PPE1 | 0.81860247 | 0.97821875 | -0.0471366 | 112.2507998 | 217.859273 |
| YGL019W | CKB1 | 0.45743293 | 0.96815259 | -0.0470768 | 86.72660149 | 170.229862 |
| YLR431C | ATG23 | 0.52939868 | 0.96815259 | -0.0469649 | 99.51087043 | 194.0605337 |
| YMR163C | INP2 | 0.5066603 | 0.96815259 | -0.0469215 | 93.30062109 | 182.4667405 |
| YCR063W | BUD31 | 0.39100222 | 0.96815259 | -0.0469048 | 0 | 8.399353583 |
| YPL107W | YPL107W | 0.79778411 | 0.97570065 | -0.0468387 | 110.0472581 | 213.6949232 |
| YCR053W | THR4 | 0.78541589 | 0.97260548 | -0.0463398 | 15.06019207 | 36.3949173 |
| YKR048C | NAP1 | 0.65596682 | 0.96815259 | -0.0462905 | 108.2454586 | 210.235268 |
| YKL063C | YKL063C | 0.52526486 | 0.96815259 | -0.0460671 | 110.4948063 | 214.3917115 |
| YNL092W | YNL092W | 0.73568361 | 0.97135148 | -0.0459138 | 126.647592 | 244.4993785 |
| YPL181W | CTI6 | 0.69468951 | 0.97135148 | -0.0459 | 117.2492461 | 226.9630676 |
| YDR252W | BTT1 | 0.7137577 | 0.97135148 | -0.045898 | 121.4648553 | 234.8274739 |
| YJR051W | OSM1 | 0.69708878 | 0.97135148 | -0.045785 | 118.5989403 | 229.4605193 |
| YGR247W | CPD1 | 0.83360313 | 0.98013789 | -0.0457359 | 123.4401217 | 238.4835682 |
| YDR098C | GRX3 | 0.75067364 | 0.97135148 | -0.0454566 | 124.0121453 | 239.5007333 |
| YGL210W | YPT32 | 0.33784007 | 0.96815259 | -0.0453556 | 97.62476781 | 190.2535888 |
| YLR217W | YLR217W | 0.78548454 | 0.97260548 | -0.045216 | 113.8139742 | 220.4316372 |
| YBR288C | APM3 | 0.68767627 | 0.97133463 | -0.0451964 | 95.22762963 | 185.7528987 |
| YDL210W | UGA4 | 0.7507648 | 0.97135148 | -0.0451922 | 117.9858926 | 228.2106399 |
| YFR012W | YFR012W | 0.56081369 | 0.96815259 | -0.0450164 | 111.4070235 | 215.9054195 |
| YJR010W | MET3 | 0.69813697 | 0.97135148 | -0.044995 | 133.2473076 | 256.6474697 |
| YLR263W | RED1 | 0.7641419 | 0.97135148 | -0.0447326 | 105.9763647 | 205.7230115 |
| YPL167C | REV3 | 0.61377582 | 0.96815259 | -0.0446559 | 110.7673193 | 214.6474229 |
| YHL002W | HSE1 | 0.80219462 | 0.9762419 | -0.0445032 | 113.121493 | 219.0120799 |
| YNL335W | DDI3 | 0.75290769 | 0.97135148 | -0.0443896 | 126.1560004 | 243.3093071 |
| YFL020C | PAU5 | 0.7294564 | 0.97135148 | -0.0443714 | 102.3954411 | 198.977653 |
| YDL175C | AIR2 | 0.63208805 | 0.96815259 | -0.0441728 | 122.7052905 | 236.8327454 |
| YOR371C | GPB1 | 0.81981598 | 0.97867384 | -0.0441598 | 112.2197938 | 217.2683649 |
| YKL027W | YKL027W | 0.5786994 | 0.96815259 | -0.0441353 | 103.6005788 | 201.1837113 |
| YLL010C | PSR1 | 0.74908812 | 0.97135148 | -0.0441314 | 120.7545516 | 233.1859763 |
| YBR067C | TIP1 | 0.71830071 | 0.97135148 | -0.0441279 | 122.7405772 | 236.890526 |
| YLR081W | GAL2 | 0.76008469 | 0.97135148 | -0.0440806 | 121.6112472 | 234.7751541 |
| YDR132C | YDR132C | 0.65015824 | 0.96815259 | -0.0439854 | 109.7167307 | 212.5673486 |
| YCL009C | ILV6 | 0.57576519 | 0.96815259 | -0.0437292 | 113.5298674 | 219.6353597 |

| | | | | | | |
|---|---|---|---|---|---|---|
| YKL161C | YKL161C | 0.76064486 | 0.97135148 | -0.0435227 | 112.2496475 | 217.2099587 |
| YDR402C | DIT2 | 0.70900958 | 0.97135148 | -0.0433414 | 116.0226725 | 224.2165736 |
| YMR056C | AAC1 | 0.71050753 | 0.97135148 | -0.0433024 | 118.6275315 | 229.0692792 |
| YKL199C | YKT9 | 0.75508887 | 0.97135148 | -0.0432806 | 115.145727 | 222.5696332 |
| YDR488C | PAC11 | 0.74514665 | 0.97135148 | -0.0431695 | 113.4385751 | 219.364816 |
| YMR173W-A | YMR173W-A | 0.79977747 | 0.9762419 | -0.0431353 | 109.7270259 | 212.4343216 |
| YJR050W | ISY1 | 0.7358279 | 0.97135148 | -0.0430283 | 115.7592087 | 223.668969 |
| YKL077W | YKL077W | 0.65565042 | 0.96815259 | -0.0428522 | 125.6647489 | 242.1175166 |
| YDL025C | YDL025C | 0.8349564 | 0.98035418 | -0.0427451 | 122.8460812 | 236.8397356 |
| YLR448W | RPL6B | 0.79920127 | 0.9762419 | -0.0426576 | 88.08186449 | 171.9669283 |
| YPL103C | FMP30 | 0.77714572 | 0.97246744 | -0.042601 | 112.531931 | 217.5715588 |
| YJL153C | INO1 | 0.7977007 | 0.97570065 | -0.0425728 | 113.4594303 | 219.2968714 |
| YMR025W | CSI1 | 0.54871595 | 0.96815259 | -0.0425639 | 109.6413659 | 212.1721906 |
| YKL103C | LAP4 | 0.74583524 | 0.97135148 | -0.0425004 | 106.4547881 | 206.2158376 |
| YNL208W | YNL208W | 0.60084981 | 0.96815259 | -0.0424308 | 94.41693359 | 183.7452008 |
| YBR013C | YBR013C | 0.66225589 | 0.96927465 | -0.0424243 | 123.2099659 | 237.4611703 |
| YDR155C | CPR1 | 0.75302588 | 0.97135148 | -0.042184 | 120.2816572 | 231.9550042 |
| YOR317W | FAA1 | 0.73903257 | 0.97135148 | -0.0419562 | 112.0724793 | 216.598914 |
| YMR172C-A | YMR172C-A | 0.72646999 | 0.97135148 | -0.0417418 | 108.1148066 | 209.1769821 |
| YPL064C | CWC27 | 0.810808 | 0.9762419 | -0.0415163 | 107.4563427 | 207.9081371 |
| YIR030C | DCG1 | 0.75593091 | 0.97135148 | -0.041475 | 129.2628523 | 248.583616 |
| YGL101W | YGL101W | 0.39125113 | 0.96815259 | -0.0412789 | 98.08629496 | 190.3845941 |
| YGL063W | PUS2 | 0.75006375 | 0.97135148 | -0.0412333 | 100.0521092 | 194.0439165 |
| YPL196W | OXR1 | 0.69730368 | 0.97135148 | -0.0410078 | 119.0606747 | 229.4664676 |
| YHR049C-A | YHR049C-A | 0.76452727 | 0.9713547 | -0.0409853 | 117.2757478 | 226.1324353 |
| YKL148C | SDH1 | 0.59451159 | 0.96815259 | -0.0408409 | 94.45078272 | 183.5236467 |
| YML053C | YML053C | 0.77865314 | 0.97246744 | -0.0407507 | 121.5093965 | 233.9888401 |
| YAL060W | BDH1 | 0.77276741 | 0.97246744 | -0.0407206 | 121.7860854 | 234.4996571 |
| YBL091C-A | SCS22 | 0.72810408 | 0.97135148 | -0.0407161 | 117.2246636 | 225.9889185 |
| YGR243W | FMP43 | 0.86487233 | 0.98248371 | -0.0406688 | 121.813058 | 234.5406993 |
| YKR047W | YKR047W | 0.58432142 | 0.96815259 | -0.0405746 | 96.45816426 | 187.2209921 |
| YGR239C | PEX21 | 0.617168 | 0.96815259 | -0.0405463 | 111.6973234 | 215.6465529 |
| YGL162W | SUT1 | 0.53811371 | 0.96815259 | -0.040104 | 99.01386866 | 191.9047079 |
| YLR236C | YLR236C | 0.7361431 | 0.97135148 | -0.0400067 | 120.6350385 | 232.2243875 |
| YDL085W | NDE2 | 0.72101601 | 0.97135148 | -0.0399982 | 124.4010722 | 239.2488907 |
| YKR003W | OSH6 | 0.69966974 | 0.97135148 | -0.0399213 | 105.4363744 | 203.8540091 |
| YML068W | ITT1 | 0.65711951 | 0.96815259 | -0.0398326 | 98.56943192 | 191.0269613 |
| YNL107W | YAF9 | 0.62194388 | 0.96815259 | -0.0398145 | 98.8254492 | 191.5013596 |
| YDR320C | SWA2 | 0.81782082 | 0.97821552 | -0.0397937 | 123.9246155 | 238.3233705 |
| YHR151C | MTC6 | 0.83927076 | 0.98035418 | -0.0397802 | 114.880343 | 221.4476886 |
| YOR367W | SCP1 | 0.85787441 | 0.98248371 | -0.0397633 | 115.1944541 | 222.030683 |
| YPR127W | YPR127W | 0.8261363 | 0.97887121 | -0.0397149 | 104.094242 | 201.3131394 |
| YKL106W | AAT1 | 0.68939615 | 0.97135148 | -0.0396921 | 110.2205567 | 212.7384894 |
| YOR247W | SRL1 | 0.66774039 | 0.9703088 | -0.0396832 | 119.6011614 | 230.2376258 |
| YIL164C | NIT1 | 0.82806932 | 0.97933188 | -0.039565 | 123.1457905 | 236.8294168 |
| YML071C | COG8 | 0.68672545 | 0.97133463 | -0.0394494 | 98.37004904 | 190.5863687 |
| YDR128W | MTC5 | 0.80648429 | 0.9762419 | -0.0393891 | 117.9979763 | 227.1940085 |
| YOL009C | MDM12 | 0.91430326 | 0.98520231 | -0.0391762 | 27.24626039 | 57.84679775 |
| YLR134W | PDC5 | 0.81912087 | 0.97821875 | -0.0391741 | 105.9443554 | 204.6679073 |
| YOR309C | YOR309C | 0.78656741 | 0.97260548 | -0.0391001 | 88.43217989 | 171.9834349 |
| YJL126W | NIT2 | 0.81136038 | 0.9762419 | -0.0390559 | 122.034696 | 234.6653655 |
| YIR016W | YIR016W | 0.7919247 | 0.97340714 | -0.0390354 | 117.0024019 | 225.2732964 |
| YOL062C | APM4 | 0.92669483 | 0.98677548 | -0.0390188 | 97.82513276 | 189.4926395 |
| YNL303W | YNL303W | 0.74145332 | 0.97135148 | -0.0389873 | 111.2881294 | 214.6039596 |
| YNL087W | TCB2 | 0.78905168 | 0.97272554 | -0.0388431 | 123.3858273 | 237.1479683 |
| YLR031W | YLR031W | 0.62426947 | 0.96815259 | -0.0385562 | 107.8163047 | 208.0496311 |
| YGL089C | MF(ALPHA)2 | 0.82611808 | 0.97887121 | -0.0384851 | 124.5139762 | 239.1885659 |
| YLL021W | SPA2 | 0.7764141 | 0.97246744 | -0.0384684 | 118.4523171 | 227.8767605 |
| YJR092W | BUD4 | 0.81695524 | 0.97770507 | -0.0384589 | 124.6817435 | 239.4968618 |
| YJL181W | YJL181W | 0.8122241 | 0.97633618 | -0.0383949 | 115.766134 | 222.8521808 |
| YER005W | YND1 | 0.79749876 | 0.97570065 | -0.0383693 | 115.4273376 | 222.2155215 |
| YOR195W | SLK19 | 0.83025926 | 0.97970137 | -0.0383158 | 118.8398002 | 228.5723407 |
| YBR141C | YBR141C | 0.52156047 | 0.96815259 | -0.038183 | 118.0334984 | 227.0443011 |
| YOR027W | STI1 | 0.78711222 | 0.97260548 | -0.0378849 | 124.7729575 | 239.56425 |

| | | | | | | |
|---|---|---|---|---|---|---|
| YFR015C | GSY1 | 0.78854764 | 0.97272554 | -0.0378186 | 119.8080485 | 230.2897007 |
| YKR017C | YKR017C | 0.80818412 | 0.9762419 | -0.0377696 | 107.0828328 | 206.5403906 |
| YLR393W | ATP10 | 0.40820015 | 0.96815259 | -0.0377647 | 69.31027933 | 136.0699218 |
| YMR096W | SNZ1 | 0.76818376 | 0.97246744 | -0.0375959 | 115.6020408 | 222.4029569 |
| YLR319C | BUD6 | 0.73664136 | 0.97135148 | -0.0375394 | 109.5950959 | 211.1861053 |
| YHR031C | RRM3 | 0.82426464 | 0.97873587 | -0.0374924 | 106.4170881 | 205.2487131 |
| YPR032W | SRO7 | 0.84928401 | 0.98085678 | -0.0374028 | 105.0525242 | 202.6869011 |
| YIL039W | YIL039W | 0.79752543 | 0.97570065 | -0.0373104 | 101.0509054 | 195.204808 |
| YLR224W | YLR224W | 0.80994648 | 0.9762419 | -0.0371148 | 125.7844823 | 241.313483 |
| YDR384C | ATO3 | 0.77370546 | 0.97246744 | -0.037072 | 121.6792786 | 233.6470238 |
| YFL013W-A | YFL013W-A | 0.78141142 | 0.97246744 | -0.0370547 | 118.4155663 | 227.5550451 |
| YOR115C | TRS33 | 0.80232993 | 0.9762419 | -0.0368598 | 118.251868 | 227.2147386 |
| YNR034W | SOL1 | 0.75551031 | 0.97135148 | -0.036857 | 99.47789139 | 192.1889583 |
| YDR486C | VPS60 | 0.77505296 | 0.97246744 | -0.0367201 | 112.3386277 | 216.1578151 |
| YNR061C | YNR061C | 0.73193745 | 0.97135148 | -0.0366825 | 107.6108225 | 207.3307462 |
| YMR209C | YMR209C | 0.72008141 | 0.97135148 | -0.0366421 | 114.3339036 | 219.8662986 |
| YJL060W | BNA3 | 0.75943555 | 0.97135148 | -0.0365699 | 119.824359 | 230.0965195 |
| YIL111W | COX5B | 0.68354039 | 0.97060928 | -0.0365488 | 103.4827295 | 199.6053186 |
| YML005W | TRM12 | 0.83397283 | 0.98013789 | -0.0365202 | 112.3871614 | 216.2125569 |
| YER056C-A | RPL34A | 0.83400814 | 0.98013789 | -0.0364899 | 107.288668 | 206.6952387 |
| YLR376C | PSY3 | 0.77055835 | 0.97246744 | -0.0364268 | 114.9796877 | 221.0325357 |
| YOR318C | YOR318C | 0.79275797 | 0.97369374 | -0.036381 | 118.5561706 | 227.6967265 |
| YLR346C | YLR346C | 0.78641521 | 0.97260548 | -0.0363792 | 109.8016768 | 211.3637633 |
| YNL257C | SIP3 | 0.72584332 | 0.97135148 | -0.0362999 | 103.1491791 | 198.9384676 |
| YDR169C | STB3 | 0.65962732 | 0.96815259 | -0.0362748 | 115.9139972 | 222.748382 |
| YML104C | MDM1 | 0.74565019 | 0.97135148 | -0.0361684 | 116.228711 | 223.3164696 |
| YGL081W | YGL081W | 0.71499556 | 0.97135148 | -0.0359913 | 121.1026622 | 232.3777475 |
| YPL264C | YPL264C | 0.75587153 | 0.97135148 | -0.0359874 | 106.1573089 | 204.4945604 |
| YOL036W | YOL036W | 0.78951989 | 0.97272554 | -0.0358958 | 116.9357513 | 224.5867356 |
| YOR378W | YOR378W | 0.8011717 | 0.9762419 | -0.0358461 | 117.9480089 | 226.466336 |
| YMR164C | MSS11 | 0.51007397 | 0.96815259 | -0.0358305 | 86.35461565 | 167.5219662 |
| YDL229W | SSB1 | 0.78052778 | 0.97246744 | -0.0357917 | 113.7505719 | 218.6257256 |
| YBR044C | TCM62 | 0.77940069 | 0.97246744 | -0.03571 | 91.70315751 | 177.4787858 |
| YDL042C | SIR2 | 0.77129047 | 0.97246744 | -0.0357012 | 111.8793999 | 215.1186009 |
| YDL227C | HO | 0.78048673 | 0.97246744 | -0.0356164 | 116.282424 | 223.3178433 |
| YDR055W | PST1 | 0.76362049 | 0.97135148 | -0.035561 | 125.9942934 | 241.4266635 |
| YDR112W | YDR112W | 0.75611235 | 0.97135148 | -0.035539 | 123.8927943 | 237.5021047 |
| YDR157W | YDR157W | 0.83429425 | 0.98015308 | -0.0355084 | 125.444713 | 240.3919327 |
| YIL136W | OM45 | 0.73802363 | 0.97135148 | -0.035337 | 106.6154755 | 205.2328586 |
| YBL007C | SLA1 | 0.69351008 | 0.97135148 | -0.0352246 | 93.31268703 | 180.3946531 |
| YHR006W | STP2 | 0.78082139 | 0.97246744 | -0.0351188 | 111.6749206 | 214.6328336 |
| YLR373C | VID22 | 0.92958725 | 0.98677548 | -0.0351008 | 34.47393797 | 70.60118392 |
| YOR175C | YOR175C | 0.79220667 | 0.97340714 | -0.034994 | 105.1054163 | 202.3542298 |
| YBR007C | DSF2 | 0.70010025 | 0.97135148 | -0.0349391 | 114.1292448 | 219.179513 |
| YOR213C | SAS5 | 0.72786586 | 0.97135148 | -0.0348097 | 121.246669 | 232.4348147 |
| YJR009C | TDH2 | 0.83405306 | 0.98013789 | -0.0347241 | 119.4559189 | 229.0786182 |
| YIR028W | DAL4 | 0.77431568 | 0.97246744 | -0.034673 | 127.2312629 | 243.5753789 |
| YBR010W | HHT1 | 0.79117103 | 0.97320687 | -0.0345783 | 102.7004643 | 197.7930362 |
| YPL165C | SET6 | 0.79562666 | 0.97556279 | -0.0345049 | 115.4442274 | 221.5550305 |
| YDR275W | BSC2 | 0.77514819 | 0.97246744 | -0.0345037 | 115.1536615 | 221.0127246 |
| YDL154W | MSH5 | 0.783117 | 0.97260548 | -0.034435 | 138.0055309 | 263.6335562 |
| YCL048W | SPS22 | 0.76485179 | 0.97139118 | -0.0343887 | 129.012035 | 246.8467183 |
| YHR199C | FMP34 | 0.83192751 | 0.98013789 | -0.0341804 | 115.746094 | 222.0600957 |
| YMR086C-A | YMR086C-A | 0.73435779 | 0.97135148 | -0.0341795 | 109.8852074 | 211.1256888 |
| YJR020W | YJR020W | 0.88096751 | 0.98248371 | -0.0341309 | 116.9689632 | 224.3326448 |
| YGL108C | YGL108C | 0.76838822 | 0.97246744 | -0.0341262 | 124.0423098 | 237.5280506 |
| YKL142W | MRP8 | 0.71950324 | 0.97135148 | -0.0341139 | 111.1189186 | 213.4155835 |
| YMR027W | YMR027W | 0.57945712 | 0.96815259 | -0.0340396 | 100.2757897 | 193.1730206 |
| YCR021C | HSP30 | 0.6766016 | 0.97060928 | -0.0339611 | 111.8881959 | 214.8234114 |
| YBR249C | ARO4 | 0.78031467 | 0.97246744 | -0.0339289 | 116.9817531 | 224.3203443 |
| YPL164C | MLH3 | 0.82424707 | 0.97873587 | -0.0339277 | 101.9143648 | 196.2099557 |
| YLR324W | PEX30 | 0.72525805 | 0.97135148 | -0.0338654 | 100.7256971 | 193.9811975 |
| YDR506C | YDR506C | 0.5235402 | 0.96815259 | -0.0338373 | 90.61017619 | 175.1043456 |
| YGL256W | ADH4 | 0.65013743 | 0.96815259 | -0.0336824 | 115.1205217 | 220.8038246 |

| | | | | | | |
|---|---|---|---|---|---|---|
| YHR204W | MNL1 | 0.88599329 | 0.98262107 | -0.0336747 | 125.0646786 | 239.3545551 |
| YGR249W | MGA1 | 0.89559562 | 0.98335646 | -0.0334774 | 131.8818016 | 252.0374491 |
| YNL280C | ERG24 | 0.84140965 | 0.98035418 | -0.033395 | 111.0263941 | 213.1142398 |
| YKL221W | MCH2 | 0.78521278 | 0.97260548 | -0.0333881 | 99.11282518 | 190.8866896 |
| YML076C | WAR1 | 0.67563182 | 0.97060928 | -0.0332178 | 96.87857299 | 186.6879072 |
| YNL095C | YNL095C | 0.61598803 | 0.96815259 | -0.0332068 | 93.46633915 | 180.3199856 |
| YKR098C | UBP11 | 0.54949564 | 0.96815259 | -0.0331074 | 96.42034407 | 185.8132566 |
| YJL121C | RPE1 | 0.84532356 | 0.98035418 | -0.0328891 | 111.4163143 | 213.7510919 |
| YJR036C | HUL4 | 0.79001291 | 0.97272554 | -0.0328047 | 113.9188661 | 218.404807 |
| YML052W | SUR7 | 0.46754561 | 0.96815259 | -0.0327351 | 101.1291217 | 194.5314279 |
| YGL263W | COS12 | 0.86673673 | 0.98248371 | -0.0326776 | 119.2330824 | 228.2964194 |
| YDR122W | KIN1 | 0.77091559 | 0.97246744 | -0.032607 | 119.9757242 | 229.66926 |
| YGR189C | CRH1 | 0.88365311 | 0.98248371 | -0.032458 | 126.9363356 | 242.6284969 |
| YNL218W | MGS1 | 0.73802104 | 0.97135148 | -0.0323669 | 97.3558687 | 187.4260059 |
| YGR153W | YGR153W | 0.83303569 | 0.98013789 | -0.0322861 | 115.5628742 | 221.3790547 |
| YNL105W | YNL105W | 0.40737432 | 0.96815259 | -0.0322291 | 91.61054291 | 176.6826685 |
| YCL030C | HIS4 | 0.50564921 | 0.96815259 | -0.0320433 | 114.113145 | 218.6309272 |
| YJR096W | YJR096W | 0.78819926 | 0.97272554 | -0.0317776 | 118.9341633 | 227.5775743 |
| YKL068W | NUP100 | 0.70993198 | 0.97135148 | -0.0317481 | 101.281771 | 194.6394761 |
| YCL062W | YCL062W | 0.838508 | 0.98035418 | -0.0317128 | 104.6496323 | 200.9163364 |
| YJL012C | VTC4 | 0.73921832 | 0.97135148 | -0.0313994 | 118.7027128 | 227.07805 |
| YMR087W | YMR087W | 0.4232978 | 0.96815259 | -0.031374 | 98.92744651 | 190.180177 |
| YNL083W | SAL1 | 0.66516622 | 0.96985526 | -0.0312918 | 91.96413411 | 177.1745037 |
| YMR030W | RSF1 | 0.47405471 | 0.96815259 | -0.0312193 | 105.3746774 | 202.1806143 |
| YMR092C | AIP1 | 0.59336444 | 0.96815259 | -0.0311344 | 99.50918909 | 191.2225889 |
| YMR272C | SCS7 | 0.84060172 | 0.98035418 | -0.030855 | 113.0075986 | 216.3555966 |
| YDR008C | YDR008C | 0.61748111 | 0.96815259 | -0.0307957 | 110.9329566 | 212.4744474 |
| YCL044C | MGR1 | 0.84650971 | 0.98035418 | -0.0305361 | 111.1237376 | 212.7838938 |
| YGL205W | POX1 | 0.7873087 | 0.97260548 | -0.0305301 | 112.2383757 | 214.8623185 |
| YPL073C | YPL073C | 0.8413232 | 0.98035418 | -0.030343 | 120.3477675 | 229.9579324 |
| YJL110C | GZF3 | 0.8687853 | 0.98248371 | -0.0302103 | 120.1353856 | 229.5379499 |
| YCR079W | PTC6 | 0.80134183 | 0.9762419 | -0.0301507 | 129.6901955 | 247.3530049 |
| YMR085W | YMR085W | 0.81633962 | 0.97741538 | -0.0301279 | 110.4379557 | 211.4313847 |
| YGR295C | COS6 | 0.80977452 | 0.9762419 | -0.0300996 | 115.3106629 | 220.5169905 |
| YHR162W | YHR162W | 0.86215564 | 0.98248371 | -0.0300033 | 110.1283295 | 210.8314254 |
| YDR233C | RTN1 | 0.73373225 | 0.97135148 | -0.029931 | 115.7700389 | 221.3438079 |
| YMR127C | SAS2 | 0.89671114 | 0.98357026 | -0.0299193 | 114.7894833 | 219.5123593 |
| YLR391W | YLR391W | 0.81612771 | 0.97741538 | -0.0297981 | 107.9711282 | 206.7701324 |
| YER041W | YEN1 | 0.85111161 | 0.98105658 | -0.0296858 | 117.0288103 | 223.6483012 |
| YGR122W | YGR122W | 0.86742098 | 0.98248371 | -0.0296623 | 102.6963752 | 196.9050813 |
| YGR261C | APL6 | 0.86092058 | 0.98248371 | -0.0295542 | 112.7859767 | 215.7091935 |
| YDR059C | UBC5 | 0.73099043 | 0.97135148 | -0.0294689 | 110.7584081 | 211.9112115 |
| YHR158C | KEL1 | 0.88903523 | 0.98278659 | -0.0293769 | 107.0606641 | 204.9961188 |
| YNL117W | MLS1 | 0.85439307 | 0.98200317 | -0.0293519 | 123.6350555 | 235.9133219 |
| YJL045W | YJL045W | 0.80940619 | 0.9762419 | -0.0292923 | 122.5631209 | 233.9028121 |
| YGR224W | AZR1 | 0.83520781 | 0.98035418 | -0.02924 | 119.2164536 | 227.6498164 |
| YPR008W | HAA1 | 0.89996667 | 0.98385994 | -0.0290494 | 145.3030334 | 276.2835781 |
| YGL041C | YGL041C | 0.86931462 | 0.98248371 | -0.0289891 | 124.3576545 | 237.196448 |
| YMR196W | YMR196W | 0.86124524 | 0.98248371 | -0.0289032 | 114.0768474 | 218.0009024 |
| YDR097C | MSH6 | 0.86492345 | 0.98248371 | -0.0287953 | 103.8602108 | 198.9211094 |
| YOR246C | YOR246C | 0.87714043 | 0.98248371 | -0.0286437 | 121.4207526 | 231.6554253 |
| YHL020C | OPI1 | 0.91736409 | 0.98520231 | -0.02862 | 28.24829952 | 57.82591536 |
| YOR076C | SKI7 | 0.93214613 | 0.98690564 | -0.0284188 | 96.29543277 | 184.7406259 |
| YLR183C | TOS4 | 0.83978546 | 0.98035418 | -0.0283521 | 113.2956084 | 216.444699 |
| YEL014C | YEL014C | 0.85848891 | 0.98248371 | -0.0283443 | 110.8114366 | 211.808771 |
| YBR242W | YBR242W | 0.80694585 | 0.9762419 | -0.0281234 | 135.5566959 | 257.9346966 |
| YHR157W | REC104 | 0.87527795 | 0.98248371 | -0.0279893 | 109.3427057 | 209.0050838 |
| YGR200C | ELP2 | 0.86410527 | 0.98248371 | -0.0277201 | 98.62343259 | 188.9586984 |
| YOR162C | YRR1 | 0.84243307 | 0.98035418 | -0.0277001 | 117.4598077 | 224.0968055 |
| YLR242C | ARV1 | 0.80583247 | 0.9762419 | -0.0276909 | 86.50192713 | 166.3392248 |
| YKR100C | SKG1 | 0.8052201 | 0.9762419 | -0.0275141 | 117.3830696 | 223.9203258 |
| YBR189W | RPS9B | 0.91021245 | 0.98520231 | -0.0273397 | 18.09490206 | 38.65416575 |
| YDL134C | PPH21 | 0.91146475 | 0.98520231 | -0.027178 | 112.9669633 | 215.6213299 |
| YLR187W | SKG3 | 0.87798504 | 0.98248371 | -0.0271298 | 107.315304 | 205.0687909 |

| | | | | | | |
|---|---|---|---|---|---|---|
| YMR153W | NUP53 | 0.37923946 | 0.96815259 | -0.0270414 | 99.24016753 | 189.9877523 |
| YDL144C | YDL144C | 0.76910261 | 0.97246744 | -0.0270117 | 119.760903 | 228.2665205 |
| YJL213W | YJL213W | 0.89010309 | 0.98278659 | -0.0269866 | 111.7782664 | 213.3693804 |
| YPL136W | YPL136W | 0.86803885 | 0.98248371 | -0.0269427 | 113.5093958 | 216.5911675 |
| YDR461W | MFA1 | 0.89739072 | 0.98357026 | -0.0265867 | 135.8968064 | 258.2940382 |
| YGR088W | CTT1 | 0.86623259 | 0.98248371 | -0.0265814 | 116.3381265 | 221.8038332 |
| YDR150W | NUM1 | 0.74221351 | 0.97135148 | -0.0265053 | 104.5678108 | 199.8311588 |
| YIL149C | MLP2 | 0.87028685 | 0.98248371 | -0.0264858 | 120.8242446 | 230.1561547 |
| YIL093C | RSM25 | 0.88962758 | 0.98278659 | -0.0264288 | 117.060451 | 223.1241032 |
| YCL074W | YCL074W | 0.90544879 | 0.98454853 | -0.0264012 | 110.5336455 | 210.9425516 |
| YLR170C | APS1 | 0.87389669 | 0.98248371 | -0.0260981 | 108.6955646 | 207.4591057 |
| YNL029C | KTR5 | 0.93580382 | 0.98727159 | -0.0259876 | 80.31325064 | 154.4884352 |
| YKR072C | SIS2 | 0.67676283 | 0.97060928 | -0.0258871 | 106.1120799 | 202.6014909 |
| YPL187W | MF(ALPHA)1 | 0.77157845 | 0.97246744 | -0.0258263 | 110.7560788 | 211.254591 |
| YIL028W | YIL028W | 0.88720835 | 0.98278659 | -0.0255977 | 120.7986509 | 229.9493701 |
| YJL155C | FBP26 | 0.82384253 | 0.97873587 | -0.0255574 | 121.6537251 | 231.5373956 |
| YML033W | YML033W | 0.62463562 | 0.96815259 | -0.025207 | 101.061582 | 193.0573542 |
| YHR210C | YHR210C | 0.89619252 | 0.98357026 | -0.0251621 | 109.7614785 | 209.2800885 |
| YCR067C | SED4 | 0.7680812 | 0.97246744 | -0.025047 | 115.7190413 | 220.3740834 |
| YFL019C | YFL019C | 0.84387975 | 0.98035418 | -0.0249719 | 115.3908677 | 219.7483855 |
| YOR346W | REV1 | 0.81015369 | 0.9762419 | -0.0248963 | 116.5371795 | 221.8734446 |
| YPL241C | CIN2 | 0.88123025 | 0.98248371 | -0.024631 | 115.9295352 | 220.6922902 |
| YIR014W | YIR014W | 0.8576735 | 0.98248371 | -0.0245029 | 119.5031443 | 227.3363895 |
| YOR280C | FSH3 | 0.88303177 | 0.98248371 | -0.024386 | 114.4076364 | 217.8091169 |
| YIL089W | YIL089W | 0.87566643 | 0.98248371 | -0.0241949 | 112.0164658 | 213.3138684 |
| YDR317W | HIM1 | 0.69034432 | 0.97135148 | -0.024113 | 113.7167128 | 216.471219 |
| YJL046W | YJL046W | 0.92491072 | 0.98623457 | -0.0240019 | 42.79551301 | 84.13864617 |
| YPL232W | SSO1 | 0.90570744 | 0.98454853 | -0.0238879 | 117.293173 | 223.1032604 |
| YMR147W | YMR147W | 0.85032476 | 0.98105658 | -0.0238747 | 109.6314087 | 208.8068923 |
| YMR074C | YMR074C | 0.88362296 | 0.98248371 | -0.0237166 | 113.6527393 | 216.2808931 |
| YLR308W | CDA2 | 0.79456136 | 0.97509188 | -0.0235305 | 108.0870806 | 205.8641103 |
| YOL111C | MDY2 | 0.90574882 | 0.98454853 | -0.0235198 | 126.0839782 | 239.4377312 |
| YGR248W | SOL4 | 0.88766744 | 0.98278659 | -0.0234768 | 112.1289251 | 213.3950821 |
| YDL033C | SLM3 | 0.88251928 | 0.98248371 | -0.0234737 | 13.95700755 | 30.24209359 |
| YBR291C | CTP1 | 0.8984717 | 0.98385994 | -0.023402 | 118.9154374 | 226.0428092 |
| YGR283C | YGR283C | 0.82584386 | 0.97887121 | -0.0233741 | 121.7033543 | 231.2390188 |
| YKL043W | PHD1 | 0.87394883 | 0.98248371 | -0.0233103 | 109.4930384 | 208.4476673 |
| YKL033W-A | YKL033W-A | 0.84688822 | 0.98035418 | -0.023213 | 102.2371191 | 194.8933996 |
| YCL013W | YCL013W | 0.86017536 | 0.98248371 | -0.0231164 | 123.1567109 | 233.9042984 |
| YKR070W | YKR070W | 0.8817626 | 0.98248371 | -0.0230614 | 117.3141965 | 222.9944855 |
| YBR072W | HSP26 | 0.84833062 | 0.98085678 | -0.0230571 | 131.5254191 | 249.5065995 |
| YPL068C | YPL068C | 0.89711889 | 0.98357026 | -0.0230542 | 114.6266053 | 217.979147 |
| YMR253C | YMR253C | 0.75317361 | 0.97135148 | -0.0229081 | 101.9946267 | 194.3863962 |
| YDL121C | YDL121C | 0.84615596 | 0.98035418 | -0.0225831 | 132.4705764 | 251.185028 |
| YHR028C | DAP2 | 0.87726718 | 0.98248371 | -0.0225787 | 103.2301682 | 196.632465 |
| YLR121C | YPS3 | 0.8572034 | 0.98248371 | -0.0224187 | 125.3962954 | 237.957599 |
| YMR215W | GAS3 | 0.87306092 | 0.98248371 | -0.022364 | 110.0210532 | 209.2633057 |
| YOR347C | PYK2 | 0.88213846 | 0.98248371 | -0.0223212 | 120.8364757 | 229.433197 |
| YLL052C | AQY2 | 0.85134083 | 0.98105658 | -0.0222818 | 117.8379513 | 223.8320207 |
| YDL146W | LDB17 | 0.72950224 | 0.97135148 | -0.0219069 | 117.8480042 | 223.7836413 |
| YDR408C | ADE8 | 0.78392022 | 0.97260548 | -0.0215534 | 115.6648482 | 219.6473724 |
| YKR035W-A | DID2 | 0.67648725 | 0.97060928 | -0.0213701 | 98.62361142 | 187.8219129 |
| YDR239C | YDR239C | 0.83590415 | 0.98035418 | -0.0212728 | 112.5429844 | 213.7728852 |
| YLR014C | PPR1 | 0.82951458 | 0.97952308 | -0.0212589 | 96.45007183 | 183.7469884 |
| YNL330C | RPD3 | 0.88331355 | 0.98248371 | -0.0211552 | 117.9260148 | 223.7945586 |
| YDR103W | STE5 | 0.8991835 | 0.98385994 | -0.0211309 | 126.9590481 | 240.6425051 |
| YML002W | YML002W | 0.76208137 | 0.97135148 | -0.0209943 | 102.067974 | 194.180525 |
| YHR207C | SET5 | 0.90000043 | 0.98385994 | -0.0207528 | 130.6662866 | 247.4911281 |
| YHR057C | CPR2 | 0.91734999 | 0.98520231 | -0.0206121 | 124.1514205 | 235.3116197 |
| YGL138C | YGL138C | 0.86983601 | 0.98248371 | -0.0205796 | 122.3759628 | 231.9934452 |
| YIL113W | SDP1 | 0.91750778 | 0.98520231 | -0.0204987 | 125.0663939 | 236.9983119 |
| YML099C | ARG81 | 0.90113618 | 0.98408827 | -0.0204904 | 115.1349684 | 218.4684628 |
| YDL026W | YDL026W | 0.87917837 | 0.98248371 | -0.0204665 | 117.390968 | 222.6730463 |
| YMR152W | YIM1 | 0.88383668 | 0.98248371 | -0.0203002 | 118.2039847 | 224.16004 |

| | | | | | | |
|---|---|---|---|---|---|---|
| YOR002W | ALG6 | 0.9177126 | 0.98520231 | -0.0202836 | 116.766482 | 221.4752242 |
| YJL158C | CIS3 | 0.88475977 | 0.98248371 | -0.0202737 | 124.155307 | 235.2582691 |
| YGR263C | SAY1 | 0.87344509 | 0.98248371 | -0.0201387 | 113.9492948 | 216.1934559 |
| YJR062C | NTA1 | 0.89187642 | 0.98278659 | -0.0200913 | 113.1754298 | 214.7412147 |
| YIL103W | DPH1 | 0.8767726 | 0.98248371 | -0.020076 | 114.0297829 | 216.332385 |
| YMR170C | ALD2 | 0.90783772 | 0.98487962 | -0.0199648 | 102.1463105 | 194.1423083 |
| YML117W-A | YML117W-A | 0.73999759 | 0.97135148 | -0.0199323 | 90.39495818 | 172.2128327 |
| YLR348C | DIC1 | 0.88037473 | 0.98248371 | -0.0199007 | 111.9602645 | 212.4400468 |
| YDR319C | YDR319C | 0.8595099 | 0.98248371 | -0.0198321 | 119.3791465 | 226.268638 |
| YLR454W | FMP27 | 0.77417712 | 0.97246744 | -0.0197019 | 95.55862057 | 181.805047 |
| YML120C | NDI1 | 0.68160814 | 0.97060928 | -0.0196335 | 110.5958175 | 209.8466384 |
| YPL222W | FMP40 | 0.90887202 | 0.98520231 | -0.0196011 | 114.1523451 | 216.4759924 |
| YGR129W | SYF2 | 0.80415442 | 0.9762419 | -0.019298 | 114.9220317 | 217.8576634 |
| YER140W | YER140W | 0.85336058 | 0.98107907 | -0.0192394 | 116.1618369 | 220.1601956 |
| YOR014W | RTS1 | 0.87853007 | 0.98248371 | -0.0192247 | 103.3598729 | 196.273835 |
| YDR357C | YDR357C | 0.91691965 | 0.98520231 | -0.0189076 | 110.4600185 | 209.4633029 |
| YGR053C | YGR053C | 0.89674511 | 0.98357026 | -0.0187761 | 119.4114272 | 226.1397661 |
| YOR012W | YOR012W | 0.91892029 | 0.98534698 | -0.0186413 | 119.9528071 | 227.1256359 |
| YOR140W | SFL1 | 0.91137168 | 0.98520231 | -0.0185214 | 11.69962538 | 25.1438304 |
| YDL233W | YDL233W | 0.84235023 | 0.98035418 | -0.0183482 | 122.2688745 | 231.3940836 |
| YPL067C | YPL067C | 0.87667089 | 0.98248371 | -0.018307 | 110.4830439 | 209.398712 |
| YGL049C | TIF4632 | 0.87978385 | 0.98248371 | -0.0182116 | 106.3151151 | 201.6058162 |
| YMR216C | SKY1 | 0.57034329 | 0.96815259 | -0.0180808 | 88.47165042 | 168.2930992 |
| YJL150W | YJL150W | 0.84162706 | 0.98035418 | -0.0179858 | 109.29817 | 207.1306584 |
| YDR261C | EXG2 | 0.84766138 | 0.98082942 | -0.0179789 | 117.6728747 | 222.7535076 |
| YPL115C | BEM3 | 0.92080509 | 0.98571422 | -0.0179225 | 119.2277362 | 225.6442055 |
| YBR095C | RXT2 | 0.87847248 | 0.98248371 | -0.0177673 | 107.4773208 | 203.694487 |
| YJR152W | DAL5 | 0.79766544 | 0.97570065 | -0.0177574 | 94.14467963 | 178.8189522 |
| YHR133C | NSG1 | 0.88825738 | 0.98278659 | -0.017699 | 123.0301594 | 232.6980971 |
| YIL035C | CKA1 | 0.96729503 | 0.99415147 | -0.0175682 | 80.59670538 | 153.5095753 |
| YBR078W | ECM33 | 0.93024386 | 0.98677548 | -0.0175466 | 110.2915135 | 208.9052182 |
| YGL005C | COG7 | 0.87802064 | 0.98248371 | -0.0175061 | 117.6290416 | 222.5870708 |
| YBR261C | YBR261C | 0.92723918 | 0.98677548 | -0.0174146 | 129.1811663 | 244.1226635 |
| YCL002C | YCL002C | 0.88524207 | 0.98248371 | -0.0173216 | 113.2727932 | 214.4268848 |
| YPL212C | PUS1 | 0.89429715 | 0.98335646 | -0.0169841 | 120.3781915 | 227.6224899 |
| YJR095W | SFC1 | 0.84448608 | 0.98035418 | -0.016825 | 94.63714619 | 179.5707466 |
| YKR005C | YKR005C | 0.89556763 | 0.98335646 | -0.0167813 | 125.2728159 | 236.717736 |
| YDR516C | EMI2 | 0.92715232 | 0.98677548 | -0.0167209 | 123.5570512 | 233.5059377 |
| YLR345W | YLR345W | 0.89489079 | 0.98335646 | -0.016636 | 112.7996332 | 213.4213794 |
| YIL119C | RPI1 | 0.85092331 | 0.98105658 | -0.0165766 | 112.3336855 | 212.5414511 |
| YBL047C | EDE1 | 0.8689763 | 0.98248371 | -0.0165174 | 112.8914488 | 213.571432 |
| YIL099W | SGA1 | 0.88890372 | 0.98278659 | -0.0165123 | 116.2799559 | 219.8922222 |
| YOR172W | YRM1 | 0.91307944 | 0.98520231 | -0.0163783 | 121.4130411 | 229.4446589 |
| YNR059W | MNT4 | 0.83178953 | 0.98013789 | -0.0163614 | 109.2746372 | 206.7958564 |
| YFL006W | YFL006W | 0.92097938 | 0.98571422 | -0.0163612 | 109.8973321 | 207.9575429 |
| YIL112W | HOS4 | 0.92352265 | 0.98623457 | -0.0163409 | 123.5505265 | 233.4257155 |
| YLL058W | YLL058W | 0.8988911 | 0.98385994 | -0.0163229 | 117.0644579 | 221.3218932 |
| YLR354C | TAL1 | 0.92335887 | 0.98623457 | -0.0163053 | 104.615393 | 198.0933962 |
| YHR077C | NMD2 | 0.80361237 | 0.9762419 | -0.0162327 | 113.0417571 | 213.8008688 |
| YML006C | GIS4 | 0.63008537 | 0.96815259 | -0.0162281 | 88.36892524 | 167.7696808 |
| YDL162C | YDL162C | 0.86100745 | 0.98248371 | -0.0160583 | 123.5057759 | 233.2916179 |
| YNL123W | NMA111 | 0.96088074 | 0.99240066 | -0.0159141 | 105.9694431 | 200.549492 |
| YLR343W | GAS2 | 0.85106406 | 0.98105658 | -0.0158957 | 96.91972996 | 183.6627828 |
| YOR043W | WHI2 | 0.93333685 | 0.98690564 | -0.0158926 | 119.2778271 | 225.3741588 |
| YDR284C | DPP1 | 0.84965264 | 0.98101867 | -0.0157685 | 114.9196366 | 217.221168 |
| YLR089C | ALT1 | 0.96949622 | 0.99474714 | -0.0157667 | 36.67735118 | 71.24972949 |
| YPR172W | YPR172W | 0.91908012 | 0.98534698 | -0.0156759 | 100.0476158 | 189.4589017 |
| YLR429W | CRN1 | 0.7496465 | 0.97135148 | -0.0156614 | 102.9604166 | 194.8905109 |
| YDR411C | DFM1 | 0.90492715 | 0.98454853 | -0.015659 | 127.8294816 | 241.2865409 |
| YJL188C | BUD19 | 0.91843615 | 0.98520231 | -0.0155256 | 118.5370583 | 223.9264396 |
| YMR053C | STB2 | 0.9129035 | 0.98520231 | -0.0154369 | 111.4583039 | 210.7042308 |
| YNL321W | YNL321W | 0.90372011 | 0.98432086 | -0.0153951 | 119.3207238 | 225.3650949 |
| YLR227C | ADY4 | 0.92712451 | 0.98677548 | -0.0153515 | 111.2482871 | 210.2971162 |
| YJR115W | YJR115W | 0.86843521 | 0.98248371 | -0.015084 | 102.0892628 | 193.1618701 |

| | | | | | | |
|---|---|---|---|---|---|---|
| YDR171W | HSP42 | 0.90147822 | 0.98408827 | -0.015081 | 123.182403 | 232.5133121 |
| YLR109W | AHP1 | 0.90408977 | 0.98432086 | -0.0148682 | 118.6085372 | 223.9420748 |
| YBR230C | OM14 | 0.89100589 | 0.98278659 | -0.0148369 | 116.6022923 | 220.1935589 |
| YDR100W | TVP15 | 0.90654206 | 0.98454853 | -0.0147139 | 133.4628279 | 251.6270408 |
| YBL042C | FUI1 | 0.89940844 | 0.98385994 | -0.0146524 | 123.4139592 | 232.8685662 |
| YMR167W | MLH1 | 0.70709957 | 0.97135148 | -0.0146249 | 101.3107668 | 191.6272742 |
| YCR069W | CPR4 | 0.88926011 | 0.98278659 | -0.0145811 | 120.1882335 | 226.8377807 |
| YNL004W | HRB1 | 0.91325255 | 0.98520231 | -0.0144965 | 112.8446768 | 213.122289 |
| YHR159W | YHR159W | 0.93341362 | 0.98690564 | -0.0143339 | 117.7201246 | 222.1889418 |
| YMR048W | CSM3 | 0.92438348 | 0.98623457 | -0.0143079 | 103.8823626 | 196.3681488 |
| YDR143C | SAN1 | 0.92403537 | 0.98623457 | -0.0141658 | 136.0431703 | 256.3428493 |
| YLR108C | YLR108C | 0.94582737 | 0.99015457 | -0.0139888 | 112.033145 | 211.5173418 |
| YKL076C | PSY1 | 0.94579368 | 0.99015457 | -0.0139884 | 106.3829058 | 200.9760209 |
| YPR011C | YPR011C | 0.88980304 | 0.98278659 | -0.0138478 | 116.4984769 | 219.8227516 |
| YPL018W | CTF19 | 0.94811357 | 0.99015457 | -0.0138455 | 117.1602326 | 221.0569406 |
| YGL053W | PRM8 | 0.88286098 | 0.98248371 | -0.0136648 | 121.8508197 | 229.7754793 |
| YLR133W | CKI1 | 0.91553681 | 0.98520231 | -0.0136336 | 117.3510963 | 221.3750619 |
| YMR159C | ATG16 | 0.88023233 | 0.98248371 | -0.0136139 | 106.7985394 | 201.6843877 |
| YBL068W | PRS4 | 0.93821289 | 0.98742308 | -0.0135676 | 121.6801113 | 229.4395967 |
| YCL011C | GBP2 | 0.93332083 | 0.98690564 | -0.0134227 | 126.8285675 | 239.0187518 |
| YEL001C | IRC22 | 0.93738021 | 0.98742308 | -0.0133822 | 117.6180677 | 221.8281219 |
| YPL121C | MEI5 | 0.9389185 | 0.98768154 | -0.0133118 | 114.6899852 | 216.3527995 |
| YLR352W | YLR352W | 0.92078099 | 0.98571422 | -0.0132695 | 110.5641479 | 208.6479422 |
| YNL023C | FAP1 | 0.78085825 | 0.97246744 | -0.0131762 | 96.92053875 | 183.1773029 |
| YOR085W | OST3 | 0.93012283 | 0.98677548 | -0.0131005 | 102.3566186 | 193.3054734 |
| YML051W | GAL80 | 0.94478724 | 0.99015457 | -0.0128901 | 113.2467961 | 213.58483 |
| YPL088W | YPL088W | 0.94218955 | 0.98930183 | -0.0126488 | 111.6453647 | 210.5539384 |
| YDR119W | YDR119W | 0.86761649 | 0.98248371 | -0.0125322 | 117.248185 | 220.9858446 |
| YMR201C | RAD14 | 0.91637392 | 0.98520231 | -0.0123396 | 102.6226963 | 193.6656169 |
| YCL064C | CHA1 | 0.87580182 | 0.98248371 | -0.0122622 | 114.4106651 | 215.6437273 |
| YNL146W | YNL146W | 0.91448245 | 0.98520231 | -0.0120124 | 114.4408891 | 215.6553967 |
| YOR153W | PDR5 | 0.93441635 | 0.98705629 | -0.011905 | 121.1479935 | 228.1491206 |
| YJL164C | TPK1 | 0.93590774 | 0.98727159 | -0.0118443 | 108.2782249 | 204.1280385 |
| YOL085C | YOL085C | 0.94660822 | 0.99015457 | -0.0116487 | 115.8278828 | 218.1778828 |
| YJL146W | IDS2 | 0.94865311 | 0.99015457 | -0.0116274 | 130.0896841 | 244.7813072 |
| YNL176C | YNL176C | 0.93720186 | 0.98742308 | -0.0115701 | 120.3867133 | 226.6688985 |
| YJR118C | ILM1 | 0.94503982 | 0.99015457 | -0.011444 | 86.4564969 | 163.3451037 |
| YBR244W | GPX2 | 0.92891994 | 0.98677548 | -0.0114405 | 121.23998 | 228.2375635 |
| YKR016W | FMP13 | 0.91692746 | 0.98520231 | -0.01132 | 108.534218 | 204.5117477 |
| YDR110W | FOB1 | 0.91356275 | 0.98520231 | -0.0109198 | 120.3649709 | 226.5118806 |
| YIL038C | NOT3 | 0.92977084 | 0.98677548 | -0.0107428 | 110.2385779 | 207.5880801 |
| YMR225C | MRPL44 | 0.93142518 | 0.98690564 | -0.0107275 | 114.4259185 | 215.3973693 |
| YIR002C | MPH1 | 0.94941001 | 0.99015457 | -0.0106234 | 103.2573437 | 194.5423024 |
| YGL021W | ALK1 | 0.92074003 | 0.98571422 | -0.0105565 | 119.7744043 | 225.3450343 |
| YDR528W | HLR1 | 0.96170589 | 0.9925284 | -0.0104831 | 124.9730612 | 235.0306596 |
| YOR307C | SLY41 | 0.94643642 | 0.99015457 | -0.0104625 | 121.2447243 | 228.0712736 |
| YML054C | CYB2 | 0.92646146 | 0.98677548 | -0.0103659 | 114.3154795 | 215.1265845 |
| YLR044C | PDC1 | 0.97755851 | 0.99579575 | -0.0103503 | 84.39178523 | 159.2972649 |
| YJL182C | YJL182C | 0.95315021 | 0.99015457 | -0.010324 | 113.4471599 | 213.4991129 |
| YER149C | PEA2 | 0.95481309 | 0.99015457 | -0.0103136 | 130.1363811 | 244.6331458 |
| YIL088C | AVT7 | 0.94230193 | 0.98930183 | -0.0103104 | 111.3121333 | 209.5135132 |
| YNR010W | CSE2 | 0.87946618 | 0.98248371 | -0.010199 | 5.037884563 | 11.22519173 |
| YMR095C | SNO1 | 0.84594528 | 0.98035418 | -0.0101919 | 104.8937929 | 197.5180423 |
| YBR024W | SCO2 | 0.93757661 | 0.98742308 | -0.0101824 | 125.0493677 | 235.1191745 |
| YBL086C | YBL086C | 0.93919636 | 0.98773186 | -0.0099305 | 127.8724115 | 240.3408309 |
| YBL062W | YBL062W | 0.91792649 | 0.98520231 | -0.009688 | 115.3898162 | 217.0094929 |
| YDL159W | STE7 | 0.90392124 | 0.98432086 | -0.0092288 | 129.8155373 | 243.8403148 |
| YOR144C | ELG1 | 0.9541922 | 0.99015457 | -0.0091706 | 106.655388 | 200.6216387 |
| YNL058C | YNL058C | 0.89143652 | 0.98278659 | -0.0091325 | 89.78977767 | 169.1498369 |
| YLR433C | CNA1 | 0.93782873 | 0.98742308 | -0.0090986 | 109.5156811 | 205.9449966 |
| YCR050C | YCR050C | 0.94800685 | 0.99015457 | -0.0089371 | 107.4677126 | 202.0953278 |
| YMR174C | PAI3 | 0.92300897 | 0.98623457 | -0.0088149 | 92.31597598 | 173.8059193 |
| YDR537C | YDR537C | 0.96120036 | 0.99240066 | -0.0086918 | 114.6482785 | 215.4476642 |
| YMR285C | NGL2 | 0.95618563 | 0.99065822 | -0.0085945 | 127.8385503 | 240.0384038 |

| | | | | | | |
|---|---|---|---|---|---|---|
| YAL027W | YAL027W | 0.96069582 | 0.99240066 | -0.0084542 | 129.5901985 | 243.281215 |
| YBR138C | YBR138C | 0.94687505 | 0.99015457 | -0.008451 | 124.1633094 | 233.1560704 |
| YBL001C | ECM15 | 0.94451492 | 0.99015457 | -0.0084338 | 125.3245831 | 235.3195087 |
| YMR258C | YMR258C | 0.92158986 | 0.98587615 | -0.008301 | 114.78555 | 215.6337917 |
| YDR399W | HPT1 | 0.93220997 | 0.98690564 | -0.0081334 | 126.0444346 | 236.6086845 |
| YHR003C | YHR003C | 0.96518092 | 0.99316723 | -0.0080944 | 120.6515839 | 226.5406339 |
| YOR197W | MCA1 | 0.96410369 | 0.99296447 | -0.007829 | 118.712847 | 222.8761459 |
| YNL169C | PSD1 | 0.93245503 | 0.98690564 | -0.0077935 | 76.05938032 | 143.2942251 |
| YHR160C | PEX18 | 0.95782737 | 0.99100821 | -0.007643 | 122.844632 | 230.5512241 |
| YDR250C | YDR250C | 0.95707224 | 0.99065822 | -0.0075773 | 130.7160829 | 245.2246727 |
| YJR048W | CYC1 | 0.9494484 | 0.99015457 | -0.0075348 | 121.6982092 | 228.3930474 |
| YPL198W | RPL7B | 0.96786162 | 0.99449577 | -0.0075218 | 113.4075511 | 212.9234187 |
| YOL110W | SHR5 | 0.97393814 | 0.99555698 | -0.0074132 | 116.3973623 | 218.4818577 |
| YDR346C | SVF1 | 0.93636529 | 0.98727159 | -0.0073883 | 117.6379257 | 220.7918266 |
| YNL223W | ATG4 | 0.90804341 | 0.98487962 | -0.007328 | 95.92838517 | 180.2790676 |
| YMR316C-B | YMR316C-B | 0.95665561 | 0.99065822 | -0.0071892 | 119.1590968 | 223.5941219 |
| YDR227W | SIR4 | 0.93574206 | 0.98727159 | -0.0071729 | 112.3561042 | 210.8993291 |
| YLL040C | VPS13 | 0.95358147 | 0.99015457 | -0.0071427 | 108.4665158 | 203.6373968 |
| YIR042C | YIR042C | 0.95480326 | 0.99015457 | -0.0069796 | 120.3866739 | 225.8467877 |
| YMR029C | FAR8 | 0.93015674 | 0.98677548 | -0.0069222 | 108.6276228 | 203.8984753 |
| YLR408C | YLR408C | 0.95890525 | 0.99125879 | -0.006807 | 111.5072709 | 209.2501928 |
| YBR114W | RAD16 | 0.95718602 | 0.99065822 | -0.00669 | 104.8897436 | 196.8833887 |
| YDL125C | HNT1 | 0.93643233 | 0.98727159 | -0.0066794 | 119.1830422 | 223.5474977 |
| YMR070W | MOT3 | 0.97237629 | 0.9950867 | -0.0066215 | 114.8945098 | 215.5363105 |
| YJL037W | IRC18 | 0.95428227 | 0.99015457 | -0.006593 | 123.434154 | 231.463029 |
| YBL098W | BNA4 | 0.97065236 | 0.9950867 | -0.0065617 | 108.3729132 | 203.3587284 |
| YOR018W | ROD1 | 0.9881942 | 0.99620136 | -0.0065138 | 105.9539644 | 198.8372931 |
| YKL162C | YKL162C | 0.9287183 | 0.98677548 | -0.0064623 | 107.6006725 | 201.9002035 |
| YNL159C | ASI2 | 0.95312983 | 0.99015457 | -0.0064098 | 102.8448822 | 193.01827 |
| YNR055C | HOL1 | 0.96691734 | 0.99415147 | -0.0063994 | 114.1075865 | 214.0284413 |
| YMR311C | GLC8 | 0.95013985 | 0.99015457 | -0.0061566 | 121.576591 | 227.9193446 |
| YPL099C | FMP14 | 0.99038668 | 0.99646895 | -0.0060585 | 93.53689109 | 175.5901017 |
| YKL093W | MBR1 | 0.94820316 | 0.99015457 | -0.0060451 | 100.2214679 | 188.0586466 |
| YNL211C | YNL211C | 0.97117571 | 0.9950867 | -0.0059832 | 109.8638122 | 206.0366042 |
| YNR057C | BIO4 | 0.96835529 | 0.99452706 | -0.0059567 | 130.4325846 | 244.4055569 |
| YLR389C | STE23 | 0.96505472 | 0.99316723 | -0.0057578 | 114.0125889 | 213.7363208 |
| YIR029W | DAL2 | 0.9624798 | 0.9925284 | -0.0056866 | 121.2212531 | 227.1722652 |
| YMR021C | MAC1 | 0.83968429 | 0.98035418 | -0.0056081 | 90.73744655 | 170.2867121 |
| YDR306C | YDR306C | 0.94019178 | 0.98829461 | -0.0055876 | 119.3696997 | 223.7002195 |
| YLR445W | YLR445W | 0.93742385 | 0.98742308 | -0.0054716 | 93.9396239 | 176.2363553 |
| YBR132C | AGP2 | 0.97406829 | 0.99555698 | -0.0054007 | 12.70258661 | 24.66542803 |
| YIL024C | YIL024C | 0.98128316 | 0.99596289 | -0.0053994 | 121.5998904 | 227.8272291 |
| YJL043W | YJL043W | 0.97176321 | 0.9950867 | -0.005376 | 116.8106117 | 218.8880227 |
| YER185W | YER185W | 0.97224545 | 0.9950867 | -0.0053692 | 119.1085292 | 223.1738591 |
| YMR132C | JLP2 | 0.91576283 | 0.98520231 | -0.0052211 | 104.0830103 | 195.1152857 |
| YPR028W | YOP1 | 0.95104875 | 0.99015457 | -0.0051336 | 104.8817366 | 196.5897554 |
| YOL075C | YOL075C | 0.97197202 | 0.9950867 | -0.0050846 | 124.4076011 | 233.0090069 |
| YBL104C | YBL104C | 0.96351069 | 0.99296447 | -0.0050662 | 133.2421406 | 249.4876865 |
| YCR030C | SYP1 | 0.95295474 | 0.99015457 | -0.004917 | 113.4743116 | 212.5815294 |
| YER188W | YER188W | 0.96699261 | 0.99415147 | -0.0047625 | 123.088711 | 230.4907702 |
| YBR229C | ROT2 | 0.95568405 | 0.99065822 | -0.0047415 | 124.0319608 | 232.2467544 |
| YFR023W | PES4 | 0.98203577 | 0.99596289 | -0.0047405 | 118.6590052 | 222.2226313 |
| YOR009W | TIR4 | 0.97115743 | 0.9950867 | -0.0046283 | 122.2005304 | 228.8097226 |
| YDL237W | YDL237W | 0.93137482 | 0.98690564 | -0.0045957 | 115.4059257 | 216.1276593 |
| YOR219C | STE13 | 0.98128037 | 0.99596289 | -0.0045765 | 111.0202595 | 207.9421969 |
| YHR047C | AAP1 | 0.98482963 | 0.99596289 | -0.0043945 | 123.3620013 | 230.9347237 |
| YPL041C | YPL041C | 0.9842157 | 0.99596289 | -0.0043583 | 118.9456165 | 222.688908 |
| YJL064W | YJL064W | 0.98024139 | 0.99596289 | -0.0042393 | 107.7286157 | 201.7408276 |
| YML018C | YML018C | 0.97184077 | 0.9950867 | -0.0042209 | 102.3058673 | 191.6206993 |
| YGR054W | YGR054W | 0.96920963 | 0.99474714 | -0.0042076 | 117.2245129 | 219.4509714 |
| YGL259W | YPS5 | 0.959171 | 0.99125879 | -0.0041631 | 121.1196557 | 226.7099075 |
| YLR188W | MDL1 | 0.98012016 | 0.99596289 | -0.0040798 | 105.9030678 | 198.3064664 |
| YJL144W | YJL144W | 0.99262402 | 0.99680251 | -0.0038827 | 101.7344368 | 190.4940444 |
| YJR010C-A | SPC1 | 0.97713354 | 0.99579575 | -0.0038566 | 133.1742563 | 249.1444295 |

| | | | | | | |
|---|---|---|---|---|---|---|
| YPL199C | YPL199C | 0.97398797 | 0.99555698 | -0.0037552 | 112.2898215 | 210.1636505 |
| YLR050C | YLR050C | 0.98472676 | 0.99596289 | -0.0033369 | 112.102423 | 209.7391342 |
| YGL216W | KIP3 | 0.98659643 | 0.99620136 | -0.0032623 | 107.0062728 | 200.2182503 |
| YML027W | YOX1 | 0.97429417 | 0.99555698 | -0.0032233 | 89.24207754 | 167.0698532 |
| YPL139C | UME1 | 0.98375881 | 0.99596289 | -0.0031953 | 124.573319 | 232.9798456 |
| YOR350C | MNE1 | 0.95422097 | 0.99015457 | -0.003178 | 92.57112303 | 173.2725061 |
| YGR228W | YGR228W | 0.98938501 | 0.99632462 | -0.0025568 | 123.861419 | 231.5373763 |
| YNL192W | CHS1 | 0.98814689 | 0.99620136 | -0.0025219 | 109.3144651 | 204.3918975 |
| YDR409W | SIZ1 | 0.98837986 | 0.99620136 | -0.0024932 | 122.4780861 | 228.945189 |
| YOL037C | YOL037C | 0.98707518 | 0.99620136 | -0.0024711 | 122.4387551 | 228.8678672 |
| YIL125W | KGD1 | 0.9912904 | 0.99649166 | -0.0024179 | 141.1972823 | 263.8548008 |
| YAR028W | YAR028W | 0.99116661 | 0.99649166 | -0.002292 | 111.7360756 | 208.8685583 |
| YDR419W | RAD30 | 0.99126767 | 0.99649166 | -0.0020907 | 118.3172953 | 221.1106214 |
| YER085C | YER085C | 0.98824685 | 0.99620136 | -0.0020846 | 126.4124764 | 236.2121448 |
| YMR318C | ADH6 | 0.98396498 | 0.99596289 | -0.0020835 | 120.0740216 | 224.3867314 |
| YJL161W | FMP33 | 0.99043537 | 0.99646895 | -0.001699 | 121.5669178 | 227.1030712 |
| YDR509W | YDR509W | 0.99318534 | 0.99704144 | -0.0016767 | 127.1615506 | 237.5365831 |
| YPL009C | YPL009C | 0.9920824 | 0.99649166 | -0.0013624 | 117.3573008 | 219.1892036 |
| YDR380W | ARO10 | 0.985553 | 0.99620136 | -0.0010346 | 116.7429576 | 217.9843708 |
| YER073W | ALD5 | 0.99607488 | 0.99770131 | -0.0008061 | 118.8699319 | 221.9115963 |
| YMR011W | HXT2 | 0.99361804 | 0.99710117 | -0.000776 | 114.3822005 | 213.5337693 |
| YIL108W | YIL108W | 0.99643501 | 0.99782927 | -0.0005744 | 115.8736555 | 216.2801658 |
| YDR534C | FIT1 | 0.99900345 | 0.99946897 | -0.0002453 | 125.2721324 | 233.7553206 |
| YKL151C | YKL151C | 0.99802226 | 0.99895282 | -0.0002413 | 112.2751206 | 209.5069943 |
| YER117W | RPL23B | 0.99945832 | 0.99969113 | -0.0001245 | 116.6185802 | 217.5893586 |
| YKL101W | HSL1 | 1 | 1 | 0 | 0 | 0 |
| YJL147C | YJL147C | 0.99863955 | 0.99933774 | 0.00024398 | 114.9848751 | 214.475487 |
| YKL215C | YKL215C | 0.99792454 | 0.99895282 | 0.00031522 | 113.5324951 | 211.7531257 |
| YML011C | RAD33 | 0.99186052 | 0.99649166 | 0.00058793 | 81.50420524 | 151.9513692 |
| YGR067C | YGR067C | 0.9957605 | 0.99768406 | 0.00083439 | 105.332816 | 196.3625935 |
| YLR306W | UBC12 | 0.99571341 | 0.99768406 | 0.00090259 | 110.4191624 | 205.8396191 |
| YJR070C | LIA1 | 0.99582531 | 0.99768406 | 0.00090287 | 112.8301306 | 210.3375432 |
| YKL081W | TEF4 | 0.99175992 | 0.99649166 | 0.0011561 | 93.61244369 | 174.4391112 |
| YJL162C | JJJ2 | 0.9945419 | 0.99732903 | 0.00120381 | 126.7922533 | 236.331801 |
| YDR161W | YDR161W | 0.99170124 | 0.99649166 | 0.0012174 | 120.0427217 | 223.7372409 |
| YOL046C | YOL046C | 0.99450585 | 0.99732903 | 0.00123219 | 115.4380898 | 215.1440567 |
| YLR273C | PIG1 | 0.98786368 | 0.99620136 | 0.0013747 | 122.1949511 | 227.7243362 |
| YOR381W | FRE3 | 0.99481813 | 0.99737311 | 0.00144847 | 115.3473174 | 214.9359779 |
| YJL148W | RPA34 | 0.98920706 | 0.99632462 | 0.00148745 | 95.33410461 | 177.5917569 |
| YGR038W | ORM1 | 0.99332633 | 0.99704144 | 0.00155921 | 111.5243599 | 207.7839251 |
| YOR028C | CIN5 | 0.99391971 | 0.99717085 | 0.00170834 | 124.3208099 | 231.6306556 |
| YMR119W | ASI1 | 0.98668502 | 0.99620136 | 0.0018546 | 115.5200169 | 215.1854442 |
| YNL266W | YNL266W | 0.98785537 | 0.99620136 | 0.00190538 | 114.8619492 | 213.9486408 |
| YER175C | TMT1 | 0.98922947 | 0.99632462 | 0.00196976 | 120.9532684 | 225.3012573 |
| YBR228W | SLX1 | 0.98410332 | 0.99596289 | 0.00205234 | 121.3959968 | 226.1124376 |
| YBR233W | PBP2 | 0.98659982 | 0.99620136 | 0.00238185 | 121.1294096 | 225.5560775 |
| YNR031C | SSK2 | 0.99034631 | 0.99646895 | 0.00245831 | 125.0020819 | 232.7673577 |
| YBL063W | KIP1 | 0.97539138 | 0.99579575 | 0.00256995 | 127.793355 | 237.9548467 |
| YDR179C | CSN9 | 0.98611252 | 0.99620136 | 0.00269708 | 116.5689233 | 216.9914505 |
| YHR176W | FMO1 | 0.98854541 | 0.99620136 | 0.00275887 | 117.3792781 | 218.4922088 |
| YOR233W | KIN4 | 0.98959584 | 0.99632462 | 0.00275939 | 119.208369 | 221.9045214 |
| YOR311C | HSD1 | 0.98673366 | 0.99620136 | 0.0028251 | 112.0101695 | 208.4635808 |
| YGL039W | YGL039W | 0.98149965 | 0.99596289 | 0.00291187 | 126.9973933 | 236.4086492 |
| YOL002C | IZH2 | 0.98235918 | 0.99596289 | 0.00291843 | 118.6209032 | 220.7800476 |
| YBR235W | YBR235W | 0.96224857 | 0.9925284 | 0.00292175 | 118.998624 | 221.4841393 |
| YAL028W | FRT2 | 0.97539805 | 0.99579575 | 0.00299288 | 119.0039585 | 221.4813547 |
| YAL046C | YAL046C | 0.97098229 | 0.9950867 | 0.00307107 | 104.1266027 | 193.7117193 |
| YJL135W | YJL135W | 0.98136661 | 0.99596289 | 0.00309789 | 114.742802 | 213.5128117 |
| YFL044C | OTU1 | 0.97852449 | 0.99579575 | 0.00311336 | 127.4143913 | 237.1505317 |
| YPL111W | CAR1 | 0.98423457 | 0.99596289 | 0.00329171 | 114.7754401 | 213.538993 |
| YOL126C | MDH2 | 0.98447344 | 0.99596289 | 0.0033554 | 124.2282988 | 231.163121 |
| YCL032W | STE50 | 0.98402609 | 0.99596289 | 0.00342498 | 127.9185016 | 238.0352125 |
| YOR354C | MSC6 | 0.98066904 | 0.99596289 | 0.00343449 | 121.0968654 | 225.3068627 |
| YJL132W | YJL132W | 0.97590677 | 0.99579575 | 0.00346092 | 114.8284507 | 213.6075905 |

| | | | | | | |
|---|---|---|---|---|---|---|
| YOR152C | YOR152C | 0.98291929 | 0.99596289 | 0.00359017 | 118.3657654 | 220.1837642 |
| YDR139C | RUB1 | 0.97766697 | 0.99579575 | 0.00359766 | 127.6242458 | 237.4553169 |
| YMR157C | FMP39 | 0.986281 | 0.99620136 | 0.00382302 | 110.8220112 | 206.0682168 |
| YOR167C | RPS28A | 0.98267513 | 0.99596289 | 0.00391057 | 122.7751115 | 228.352597 |
| YNL195C | YNL195C | 0.97934728 | 0.99596289 | 0.00393112 | 126.1726073 | 234.6873844 |
| YNL265C | IST1 | 0.98264472 | 0.99596289 | 0.00393696 | 122.5479544 | 227.9240804 |
| YOL118C | YOL118C | 0.98044972 | 0.99596289 | 0.00403343 | 123.1153339 | 228.9653238 |
| YJR106W | ECM27 | 0.97758531 | 0.99579575 | 0.00403471 | 121.7195623 | 226.361102 |
| YMR177W | MMT1 | 0.95317041 | 0.99015457 | 0.00407258 | 106.5166854 | 197.9913849 |
| YAL011W | SWC3 | 0.97983132 | 0.99596289 | 0.00411719 | 111.2136499 | 206.7461924 |
| YAL029C | MYO4 | 0.97863486 | 0.99579575 | 0.00424781 | 129.3197525 | 240.5020814 |
| YDR309C | GIC2 | 0.95632193 | 0.99065822 | 0.0043915 | 117.6080052 | 218.6265685 |
| YHR117W | TOM71 | 0.98169151 | 0.99596289 | 0.00439893 | 118.9360578 | 221.1028932 |
| YPR119W | CLB2 | 0.95047794 | 0.99015457 | 0.00443227 | 110.6971175 | 205.7261129 |
| YDL168W | SFA1 | 0.97325689 | 0.99555698 | 0.00459175 | 118.7442558 | 220.7105332 |
| YOL122C | SMF1 | 0.97776456 | 0.99579575 | 0.0046621 | 111.6195104 | 207.4057997 |
| YOR222W | ODC2 | 0.97826348 | 0.99579575 | 0.00467574 | 119.9526064 | 222.9498269 |
| YMR275C | BUL1 | 0.97515198 | 0.99579575 | 0.00489712 | 107.6665113 | 199.988882 |
| YDL010W | YDL010W | 0.94492198 | 0.99015457 | 0.00508437 | 121.7657834 | 226.2593688 |
| YKL094W | YJU3 | 0.96319895 | 0.99296447 | 0.0050907 | 110.0041439 | 204.3153736 |
| YPR096C | YPR096C | 0.97032701 | 0.9950867 | 0.00510869 | 118.763459 | 220.6537885 |
| YNL202W | SPS19 | 0.96232157 | 0.9925284 | 0.00527206 | 135.3218824 | 251.5164165 |
| YKL065C | YET1 | 0.94069345 | 0.98857995 | 0.00527385 | 103.3702133 | 191.9061204 |
| YOR304W | ISW2 | 0.97850665 | 0.99579575 | 0.00533891 | 113.3866313 | 210.5813939 |
| YPL034W | YPL034W | 0.96707324 | 0.99415147 | 0.00540141 | 121.3416843 | 225.4113847 |
| YNR058W | BIO3 | 0.96183281 | 0.9925284 | 0.00548224 | 125.221714 | 232.6356068 |
| YPL100W | ATG21 | 0.97645662 | 0.99579575 | 0.00550946 | 122.7688164 | 228.054535 |
| YGR050C | YGR050C | 0.97733232 | 0.99579575 | 0.005562 | 135.4550768 | 251.7129881 |
| YNL288W | CAF40 | 0.97197751 | 0.9950867 | 0.00570136 | 113.4998836 | 210.7277767 |
| YDR036C | EHD3 | 0.96369196 | 0.99296447 | 0.00576732 | 112.3616731 | 208.5924863 |
| YGR284C | ERV29 | 0.97850979 | 0.99579575 | 0.00576858 | 116.3228966 | 215.9824362 |
| YHL027W | RIM101 | 0.97427742 | 0.99555698 | 0.0058134 | 121.8804891 | 226.3428173 |
| YML060W | OGG1 | 0.95091609 | 0.99015457 | 0.00590186 | 95.72882982 | 177.5376706 |
| YGL228W | SHE10 | 0.94828872 | 0.99015457 | 0.00623074 | 122.553292 | 227.5232841 |
| YGL177W | YGL177W | 0.96820714 | 0.99452706 | 0.00633425 | 123.777086 | 229.7878945 |
| YNL051W | COG5 | 0.97445878 | 0.99555698 | 0.0063489 | 121.3370626 | 225.2330927 |
| YLR432W | IMD3 | 0.83753603 | 0.98035418 | 0.00635683 | 94.69650409 | 175.5302601 |
| YLR251W | SYM1 | 0.95623386 | 0.99065822 | 0.00646946 | 109.0175584 | 202.2278728 |
| YMR195W | ICY1 | 0.98169241 | 0.99596289 | 0.00647078 | 124.9331141 | 231.9201672 |
| YBR129C | OPY1 | 0.96420084 | 0.99296447 | 0.00648465 | 108.3455687 | 200.9714689 |
| YPR073C | LTP1 | 0.95305171 | 0.99015457 | 0.00660298 | 104.6991629 | 194.1474377 |
| YLR300W | EXG1 | 0.95457107 | 0.99015457 | 0.0066996 | 127.8212898 | 237.2674578 |
| YNL206C | RTT106 | 0.96719321 | 0.99415147 | 0.0067372 | 112.4709001 | 208.6225849 |
| YKL055C | OAR1 | 0.95069849 | 0.99015457 | 0.00707742 | 92.71764075 | 171.7093969 |
| YGR183C | QCR9 | 0.96894507 | 0.99474714 | 0.00714356 | 107.8678431 | 199.9622164 |
| YJR109C | CPA2 | 0.95259741 | 0.99015457 | 0.0073435 | 117.2258007 | 217.3848949 |
| YNL096C | RPS7B | 0.96112672 | 0.99240066 | 0.00735608 | 101.0589541 | 187.2212957 |
| YOR221C | MCT1 | 0.93367673 | 0.98690564 | 0.00737 | 95.37652224 | 176.6174915 |
| YLL029W | YLL029W | 0.95306261 | 0.99015457 | 0.00746072 | 119.5837895 | 221.7630373 |
| YGR238C | KEL2 | 0.96930462 | 0.99474714 | 0.00754342 | 129.5584681 | 240.3572843 |
| YDR117C | TMA64 | 0.95909944 | 0.99125879 | 0.00776311 | 129.2365377 | 239.71734 |
| YGL109W | YGL109W | 0.94927759 | 0.99015457 | 0.00789796 | 129.3587316 | 239.9211615 |
| YER129W | SAK1 | 0.95900043 | 0.99125879 | 0.00792004 | 120.2710191 | 222.9629028 |
| YBR241C | YBR241C | 0.91410653 | 0.98520231 | 0.00796215 | 125.5427665 | 232.7904893 |
| YPR138C | MEP3 | 0.90139188 | 0.98408827 | 0.00811038 | 106.7122695 | 197.6332145 |
| YLR460C | YLR460C | 0.8568406 | 0.98248371 | 0.00814016 | 97.000901 | 179.5100663 |
| YNR024W | YNR024W | 0.96429014 | 0.99296447 | 0.00836859 | 125.9096312 | 233.4021401 |
| YPL221W | FLC1 | 0.94654451 | 0.99015457 | 0.00862495 | 126.3525842 | 234.1826205 |
| YLR421C | RPN13 | 0.96481382 | 0.99316723 | 0.00887647 | 119.1752323 | 220.7472994 |
| YDL236W | PHO13 | 0.94308449 | 0.98959703 | 0.00889333 | 112.2900648 | 207.8991085 |
| YML100W | TSL1 | 0.84491535 | 0.98035418 | 0.0091755 | 109.4833228 | 202.6122395 |
| YDR111C | ALT2 | 0.91417077 | 0.98520231 | 0.00919362 | 119.0931948 | 220.5374555 |
| YOL099C | YOL099C | 0.95800537 | 0.99100821 | 0.00919548 | 124.2834661 | 230.2202457 |
| YOL004W | SIN3 | 0.94120449 | 0.98887498 | 0.00923211 | 83.95634002 | 154.9782096 |

| | | | | | | |
|---|---|---|---|---|---|---|
| YJR026W | YJR026W | 0.94173487 | 0.9891902 | 0.00951521 | 121.6392197 | 225.2298064 |
| YDR089W | YDR089W | 0.88356208 | 0.98248371 | 0.00999768 | 115.5963585 | 213.8696704 |
| YOR193W | PEX27 | 0.94893955 | 0.99015457 | 0.01005978 | 128.0917051 | 237.1702367 |
| YIR044C | YIR044C | 0.92469897 | 0.98623457 | 0.01018107 | 119.4371224 | 221.0022716 |
| YGR256W | GND2 | 0.95023296 | 0.99015457 | 0.0101958 | 121.2242766 | 224.3338013 |
| YDL117W | CYK3 | 0.92861839 | 0.98677548 | 0.01044883 | 108.6101371 | 200.7551795 |
| YPL069C | BTS1 | 0.97717773 | 0.99579575 | 0.01053617 | 32.28365784 | 58.3426077 |
| YPL265W | DIP5 | 0.89498191 | 0.98335646 | 0.01059856 | 111.6199184 | 206.3435046 |
| YJL159W | HSP150 | 0.95487426 | 0.99015457 | 0.01061994 | 119.0788188 | 220.2552199 |
| YBR271W | YBR271W | 0.87793072 | 0.98248371 | 0.01069753 | 117.2001047 | 216.7363415 |
| YGL157W | YGL157W | 0.91445682 | 0.98520231 | 0.01072341 | 119.4382401 | 220.9072388 |
| YGR217W | CCH1 | 0.89191 | 0.98278659 | 0.0107712 | 122.4355124 | 226.4904814 |
| YML001W | YPT7 | 0.92758522 | 0.98677548 | 0.01105286 | 78.41939751 | 144.3222805 |
| YGR142W | BTN2 | 0.92008052 | 0.98571422 | 0.01116869 | 126.537414 | 234.0719298 |
| YFL053W | DAK2 | 0.95478288 | 0.99015457 | 0.01130405 | 127.9036523 | 236.5965858 |
| YNR039C | ZRG17 | 0.94593633 | 0.99015457 | 0.01133714 | 125.9525054 | 232.9505426 |
| YKL017C | HCS1 | 0.88501034 | 0.98248371 | 0.0113877 | 111.1930082 | 205.405735 |
| YCR065W | HCM1 | 0.9482304 | 0.99015457 | 0.01140533 | 112.0616334 | 207.0231114 |
| YPR040W | TIP41 | 0.91339611 | 0.98520231 | 0.01144378 | 111.541207 | 206.0453022 |
| YER059W | PCL6 | 0.9340439 | 0.98690564 | 0.01146788 | 121.2654151 | 224.1827567 |
| YNL134C | YNL134C | 0.90686641 | 0.98454853 | 0.01149166 | 101.5443651 | 187.3863257 |
| YML009C | MRPL39 | 0.95720562 | 0.99065822 | 0.01162247 | 115.4286408 | 213.2658152 |
| YLR395C | COX8 | 0.89810151 | 0.98385994 | 0.01166365 | 105.7083128 | 195.1239114 |
| YOL013C | HRD1 | 0.93946578 | 0.98777328 | 0.01173368 | 126.9812589 | 234.7988068 |
| YKR042W | UTH1 | 0.87962137 | 0.98248371 | 0.01178337 | 112.9499396 | 208.6126651 |
| YKR053C | YSR3 | 0.87480143 | 0.98248371 | 0.01183948 | 108.9086472 | 201.0630629 |
| YLL041C | SDH2 | 0.79296857 | 0.97369374 | 0.01187576 | 92.20407002 | 169.8920129 |
| YLL001W | DNM1 | 0.86745564 | 0.98248371 | 0.01205208 | 109.544492 | 202.2112435 |
| YDR222W | YDR222W | 0.90803048 | 0.98487962 | 0.01206543 | 124.7424721 | 230.5626525 |
| YCR051W | YCR051W | 0.93645952 | 0.98727159 | 0.0121399 | 120.1853122 | 222.0473455 |
| YGL060W | YBP2 | 0.91261805 | 0.98520231 | 0.01230246 | 117.3205604 | 216.6736684 |
| YGR197C | SNG1 | 0.94327448 | 0.98959703 | 0.01234387 | 115.6534415 | 213.5560266 |
| YLR082C | SRL2 | 0.93802051 | 0.98742308 | 0.01245906 | 113.7204995 | 209.9292465 |
| YDR385W | EFT2 | 0.93008293 | 0.98677548 | 0.01252031 | 118.5544215 | 218.9365863 |
| YOR284W | HUA2 | 0.87764776 | 0.98248371 | 0.012554 | 114.7536398 | 211.8397029 |
| YEL063C | CAN1 | 0.93519163 | 0.98727159 | 0.01278595 | 113.0524493 | 208.6243751 |
| YMR012W | CLU1 | 0.93388133 | 0.98690564 | 0.01291736 | 112.5511213 | 207.6655511 |
| YPL092W | SSU1 | 0.95330107 | 0.99015457 | 0.01299637 | 127.8653622 | 236.222102 |
| YER109C | FLO8 | 0.93248122 | 0.98690564 | 0.01329508 | 119.7178842 | 220.9684369 |
| YMR199W | CLN1 | 0.92797031 | 0.98677548 | 0.01332168 | 110.5956567 | 203.944976 |
| YLL061W | MMP1 | 0.92474884 | 0.98623457 | 0.01334522 | 112.0044422 | 206.5690324 |
| YIR034C | LYS1 | 0.931562 | 0.98690564 | 0.01335417 | 93.9417364 | 172.8691136 |
| YOR274W | MOD5 | 0.91081329 | 0.98520231 | 0.01343159 | 125.2870846 | 231.334055 |
| YPL003W | ULA1 | 0.94799229 | 0.99015457 | 0.01347367 | 117.6606487 | 217.0984172 |
| YPL176C | TRE1 | 0.95151589 | 0.99015457 | 0.01352185 | 113.5399516 | 209.4020949 |
| YDR125C | ECM18 | 0.89222154 | 0.98278659 | 0.01359931 | 124.4906597 | 229.8181881 |
| YCL042W | YCL042W | 0.86572571 | 0.98248371 | 0.01373241 | 121.141773 | 223.5465722 |
| YMR068W | AVO2 | 0.91108972 | 0.98520231 | 0.0138625 | 110.870164 | 204.36026 |
| YBR210W | ERV15 | 0.94255544 | 0.9893261 | 0.01387049 | 132.6493332 | 244.9906884 |
| YEL065W | SIT1 | 0.82988006 | 0.97952308 | 0.01391221 | 117.4480559 | 216.6232664 |
| YAL058C-A | YAL058C-A | 0.91925943 | 0.98534698 | 0.0139725 | 116.0639481 | 214.0302383 |
| YKL061W | YKL061W | 0.8091369 | 0.9762419 | 0.01402032 | 103.4976051 | 190.5775348 |
| YBR298C | MAL31 | 0.90058883 | 0.98408827 | 0.01404614 | 108.8634195 | 200.5835319 |
| YPL177C | CUP9 | 0.93375888 | 0.98690564 | 0.01426593 | 125.9498196 | 232.4210672 |
| YKR060W | UTP30 | 0.86658267 | 0.98248371 | 0.01444473 | 108.3193704 | 199.497162 |
| YJR011C | YJR011C | 0.94759796 | 0.99015457 | 0.01454022 | 118.8296008 | 219.0882579 |
| YBR239C | YBR239C | 0.92853659 | 0.98677548 | 0.0145781 | 134.9754583 | 249.2036641 |
| YKL191W | DPH2 | 0.87957661 | 0.98248371 | 0.01462327 | 110.1119417 | 202.8094654 |
| YBL003C | HTA2 | 0.82068855 | 0.97873587 | 0.0146534 | 121.5012292 | 224.0522597 |
| YLR135W | SLX4 | 0.93859993 | 0.98758836 | 0.01472387 | 107.1657939 | 197.2950302 |
| YOR334W | MRS2 | 0.92464442 | 0.98623457 | 0.01492966 | 104.4100166 | 192.1169182 |
| YLL062C | MHT1 | 0.91234819 | 0.98520231 | 0.01495258 | 118.3252591 | 218.0735013 |
| YNL274C | YNL274C | 0.89263087 | 0.98278659 | 0.01501373 | 121.1095177 | 223.2569466 |
| YAL034C | FUN19 | 0.90549133 | 0.98454853 | 0.01530163 | 131.0302457 | 241.7137945 |

| | | | | | | |
|---|---|---|---|---|---|---|
| YLR018C | POM34 | 0.92714283 | 0.98677548 | 0.01548244 | 108.3480835 | 199.364905 |
| YLR042C | YLR042C | 0.86690916 | 0.98248371 | 0.01548819 | 97.49553943 | 179.1170482 |
| YGR033C | TIM21 | 0.90293972 | 0.98421339 | 0.01552171 | 128.9263265 | 237.7492493 |
| YJL218W | YJL218W | 0.92953058 | 0.98677548 | 0.01558207 | 133.3366828 | 245.966532 |
| YOR320C | GNT1 | 0.87670763 | 0.98248371 | 0.01558791 | 113.7850268 | 209.4893401 |
| YPL130W | SPO19 | 0.89150232 | 0.98278659 | 0.01567001 | 121.7720416 | 224.3754496 |
| YML030W | YML030W | 0.61897893 | 0.96815259 | 0.01568164 | 95.10467192 | 174.6219349 |
| YCR001W | YCR001W | 0.90871562 | 0.98520231 | 0.01570553 | 126.6962335 | 233.5558052 |
| YNL255C | GIS2 | 0.92456051 | 0.98623457 | 0.01600648 | 120.3706321 | 221.7006844 |
| YJL134W | LCB3 | 0.92220534 | 0.98623457 | 0.01602205 | 126.802265 | 233.6969407 |
| YDR159W | SAC3 | 0.82800195 | 0.97933188 | 0.01617438 | 6.150134599 | 8.577488451 |
| YPR121W | THI22 | 0.90973382 | 0.98520231 | 0.01618624 | 121.7387869 | 224.2209662 |
| YGR288W | MAL13 | 0.94527563 | 0.99015457 | 0.01618626 | 130.1664975 | 239.9439467 |
| YKR033C | YKR033C | 0.82504435 | 0.97873587 | 0.01619268 | 112.0785594 | 206.1974065 |
| YOL019W | YOL019W | 0.92873428 | 0.98677548 | 0.0163873 | 123.0300813 | 226.5940378 |
| YKL092C | BUD2 | 0.86829139 | 0.98248371 | 0.01649139 | 114.2219835 | 210.1427507 |
| YMR222C | FSH2 | 0.77019446 | 0.97246744 | 0.01654264 | 114.1339477 | 209.969331 |
| YKR009C | FOX2 | 0.85963505 | 0.98248371 | 0.01654853 | 107.7142992 | 197.9915917 |
| YDR314C | RAD34 | 0.88101145 | 0.98248371 | 0.01657327 | 122.5517399 | 225.6683271 |
| YIR033W | MGA2 | 0.89744487 | 0.98357026 | 0.01657654 | 114.6628047 | 210.9499117 |
| YJL044C | GYP6 | 0.9359573 | 0.98727159 | 0.0165805 | 133.3734801 | 245.856391 |
| YHR008C | SOD2 | 0.83333869 | 0.98013789 | 0.01688642 | 104.77482 | 192.4471055 |
| YNR012W | URK1 | 0.9182307 | 0.98520231 | 0.01690321 | 123.2255778 | 226.8663772 |
| YBL070C | YBL070C | 0.86756907 | 0.98248371 | 0.01696226 | 124.105201 | 228.4968536 |
| YDL213C | NOP6 | 0.90338083 | 0.98429772 | 0.0171281 | 123.2474945 | 226.8669941 |
| YDR285W | ZIP1 | 0.88324864 | 0.98248371 | 0.01725887 | 121.9760755 | 224.4715802 |
| YLR184W | YLR184W | 0.87826465 | 0.98248371 | 0.0176628 | 89.43461288 | 163.688934 |
| YOR165W | SEY1 | 0.92408712 | 0.98623457 | 0.01777664 | 125.8324387 | 231.5734056 |
| YGR010W | NMA2 | 0.92723893 | 0.98677548 | 0.01780638 | 113.4621967 | 208.4897915 |
| YNL327W | EGT2 | 0.8709747 | 0.98248371 | 0.01784829 | 122.2811789 | 224.9352401 |
| YJL100W | LSB6 | 0.90224644 | 0.98421339 | 0.01786508 | 127.4467015 | 234.5691845 |
| YBR197C | YBR197C | 0.78316687 | 0.97260548 | 0.01809987 | 123.6042812 | 227.358608 |
| YDR094W | YDR094W | 0.87890608 | 0.98248371 | 0.0181017 | 127.8076774 | 235.2002604 |
| YML106W | URA5 | 0.84059235 | 0.98035418 | 0.01829212 | 107.1139677 | 196.5593669 |
| YFR017C | YFR017C | 0.88572304 | 0.98262107 | 0.01842427 | 122.2587883 | 224.7903251 |
| YIL030C | SSM4 | 0.92394022 | 0.98623457 | 0.01847234 | 122.842301 | 225.8703369 |
| YLL042C | ATG10 | 0.9165613 | 0.98520231 | 0.01853701 | 121.2600383 | 222.9068398 |
| YOR376W | YOR376W | 0.91552072 | 0.98520231 | 0.01855843 | 125.9787411 | 231.7063551 |
| YLR401C | DUS3 | 0.90191478 | 0.98419875 | 0.01861759 | 120.6698163 | 221.7912739 |
| YML111W | BUL2 | 0.72932224 | 0.97135148 | 0.01864612 | 4.226040153 | 4.545219816 |
| YNL166C | BNI5 | 0.87771884 | 0.98248371 | 0.01869601 | 117.9533241 | 216.7092632 |
| YPL026C | SKS1 | 0.87656799 | 0.98248371 | 0.01876498 | 119.8827464 | 220.2965005 |
| YDL189W | RBS1 | 0.8517447 | 0.98105658 | 0.01877569 | 121.4172241 | 223.1573493 |
| YFL046W | FMP32 | 0.87164947 | 0.98248371 | 0.0188056 | 121.8092699 | 223.8834047 |
| YLR063W | YLR063W | 0.89401996 | 0.98333036 | 0.01885296 | 121.8550351 | 223.9603057 |
| YHR142W | CHS7 | 0.93215887 | 0.98690564 | 0.01894548 | 122.8809807 | 225.8577718 |
| YBR246W | YBR246W | 0.89992539 | 0.98385994 | 0.0190547 | 109.2619065 | 200.430067 |
| YPR078C | YPR078C | 0.75892401 | 0.97135148 | 0.01908679 | 99.73910417 | 182.6583002 |
| YNL289W | PCL1 | 0.87561294 | 0.98248371 | 0.01924984 | 127.0853819 | 233.6471243 |
| YJL049W | YJL049W | 0.87361685 | 0.98248371 | 0.01927962 | 115.1038192 | 211.2886342 |
| YDR291W | HRQ1 | 0.89371847 | 0.98325061 | 0.01938592 | 135.5950062 | 249.4985625 |
| YNL071W | LAT1 | 0.90681017 | 0.98454853 | 0.01941378 | 115.98931 | 212.9166078 |
| YLR091W | YLR091W | 0.89005353 | 0.98278659 | 0.01949493 | 117.212488 | 215.1840738 |
| YER135C | YER135C | 0.91272184 | 0.98520231 | 0.01955095 | 117.2164704 | 215.1814716 |
| YGR279C | SCW4 | 0.87436013 | 0.98248371 | 0.01963144 | 131.6245954 | 242.0472804 |
| YOR137C | SIA1 | 0.88930562 | 0.98278659 | 0.01973075 | 133.4268603 | 245.3918566 |
| YDR068W | DOS2 | 0.8062086 | 0.9762419 | 0.01980476 | 121.1658265 | 222.5040563 |
| YJR094C | IME1 | 0.88492095 | 0.98248371 | 0.01989231 | 121.8874142 | 223.8345934 |
| YBR128C | ATG14 | 0.80870281 | 0.9762419 | 0.02011632 | 120.9606295 | 222.0654428 |
| YPL060W | LPE10 | 0.6601269 | 0.96815259 | 0.02011974 | 100.4426356 | 183.7858552 |
| YIL067C | YIL067C | 0.87777364 | 0.98248371 | 0.02014097 | 115.3532154 | 211.5996716 |
| YJR120W | YJR120W | 0.85546165 | 0.98244245 | 0.02014562 | 90.12760902 | 164.5372026 |
| YOR081C | TGL5 | 0.87545057 | 0.98248371 | 0.02025311 | 124.6046311 | 228.8393039 |
| YBR040W | FIG1 | 0.90478326 | 0.98454853 | 0.02033015 | 136.527579 | 251.0693115 |

| | | | | | | |
|---|---|---|---|---|---|---|
| YFL040W | YFL040W | 0.90620669 | 0.98454853 | 0.02038198 | 106.4401499 | 194.9280347 |
| YBR170C | NPL4 | 0.90148518 | 0.98408827 | 0.02045548 | 114.404047 | 209.7725555 |
| YFR016C | YFR016C | 0.88897396 | 0.98278659 | 0.02069272 | 121.8434388 | 223.6092216 |
| YCR005C | CIT2 | 0.78255237 | 0.97260548 | 0.02071709 | 110.5918746 | 202.6136065 |
| YBR119W | MUD1 | 0.85791686 | 0.98248371 | 0.02072958 | 131.7690978 | 242.1202234 |
| YBR118W | TEF2 | 0.88931543 | 0.98278659 | 0.02093319 | 99.71841435 | 182.2890611 |
| YLR001C | YLR001C | 0.92149845 | 0.98587615 | 0.02099297 | 114.6631405 | 210.1596784 |
| YOR263C | YOR263C | 0.8946237 | 0.98335646 | 0.02101067 | 125.832924 | 230.9951867 |
| YDL090C | RAM1 | 0.89733451 | 0.98357026 | 0.02101991 | 107.691812 | 197.148937 |
| YPR038W | YPR038W | 0.80093522 | 0.9762419 | 0.02108409 | 116.0294476 | 212.6923843 |
| YML116W | ATR1 | 0.90158436 | 0.98408827 | 0.02112448 | 111.5533888 | 204.3344845 |
| YNL067W | RPL9B | 0.88963434 | 0.98278659 | 0.02121649 | 98.66016781 | 180.2640336 |
| YPL048W | CAM1 | 0.89283896 | 0.98278659 | 0.02131518 | 114.6318528 | 210.0436085 |
| YLR178C | TFS1 | 0.80340515 | 0.9762419 | 0.02137662 | 111.036732 | 203.3254415 |
| YKR044W | UIP5 | 0.93162373 | 0.98690564 | 0.0215027 | 122.3961751 | 224.495377 |
| YHR037W | PUT2 | 0.89353471 | 0.98325061 | 0.02155356 | 114.1973011 | 209.1902084 |
| YAL019W | FUN30 | 0.88449468 | 0.98248371 | 0.02158435 | 121.2790417 | 222.3965993 |
| YLR216C | CPR6 | 0.91413483 | 0.98520231 | 0.02158704 | 112.9595512 | 206.8750305 |
| YGR208W | SER2 | 0.91988383 | 0.98571422 | 0.02160796 | 111.8460671 | 204.7939343 |
| YKR034W | DAL80 | 0.61392609 | 0.96815259 | 0.02163366 | 99.96510053 | 182.6238505 |
| YLR377C | FBP1 | 0.87593512 | 0.98248371 | 0.02186378 | 111.5497339 | 204.1952764 |
| YPL207W | TYW1 | 0.79498061 | 0.97532764 | 0.02203506 | 119.7899093 | 219.5377203 |
| YDL086W | YDL086W | 0.81221069 | 0.97633618 | 0.02211688 | 124.2420586 | 227.8291286 |
| YOR289W | YOR289W | 0.84917302 | 0.98085678 | 0.02219864 | 122.4195198 | 224.4143057 |
| YBR064W | YBR064W | 0.84602805 | 0.98035418 | 0.02220801 | 124.3238667 | 227.9654336 |
| YDR202C | RAV2 | 0.83987746 | 0.98035418 | 0.0223148 | 128.1271354 | 235.0418006 |
| YHL023C | RMD11 | 0.82190754 | 0.97873587 | 0.02235722 | 86.2141522 | 156.8401066 |
| YPL149W | ATG5 | 0.87860174 | 0.98248371 | 0.02238166 | 138.0905508 | 253.6178704 |
| YHL046C | PAU13 | 0.90249282 | 0.98421339 | 0.0224277 | 114.1914877 | 209.0228282 |
| YNR062C | YNR062C | 0.90307424 | 0.98421339 | 0.02260836 | 117.7672915 | 215.6616014 |
| YHR125W | YHR125W | 0.89895435 | 0.98385994 | 0.0232152 | 107.2244263 | 195.8838537 |
| YDR156W | RPA14 | 0.85132019 | 0.98105658 | 0.02326102 | 109.0277142 | 199.2399153 |
| YCR062W | YCR062W | 0.89253606 | 0.98278659 | 0.02350474 | 121.1780956 | 221.8643821 |
| YIL008W | URM1 | 0.86645735 | 0.98248371 | 0.02369405 | 104.2693907 | 190.2851029 |
| YOL106W | YOL106W | 0.89564674 | 0.98335646 | 0.02373762 | 126.8314405 | 232.3697265 |
| YNL156C | NSG2 | 0.85533244 | 0.98244245 | 0.02388493 | 120.3261201 | 220.2068305 |
| YNL253W | TEX1 | 0.91821189 | 0.98520231 | 0.0239541 | 118.2061037 | 216.2392782 |
| YPR157W | YPR157W | 0.82962932 | 0.97952308 | 0.02398116 | 108.5887076 | 198.2919344 |
| YEL037C | RAD23 | 0.82079494 | 0.97873587 | 0.02427114 | 110.8141417 | 202.3918429 |
| YBL085W | BOI1 | 0.87499288 | 0.98248371 | 0.02428224 | 131.3388894 | 240.6814297 |
| YAR040C | YAR040C | 0.84868047 | 0.98085678 | 0.02443335 | 110.1364974 | 201.0985629 |
| YJR098C | YJR098C | 0.78650745 | 0.97260548 | 0.02449372 | 115.3475068 | 210.8095644 |
| YKL030W | YKL030W | 0.87981593 | 0.98248371 | 0.02453679 | 128.064309 | 234.5266942 |
| YNL012W | SPO1 | 0.86180002 | 0.98248371 | 0.02453771 | 130.2608622 | 238.6244823 |
| YJR146W | YJR146W | 0.85329474 | 0.98107907 | 0.02455023 | 123.4568693 | 225.9285114 |
| YDR109C | YDR109C | 0.78565737 | 0.97260548 | 0.02467129 | 118.6664932 | 216.9697651 |
| YGR117C | YGR117C | 0.84666234 | 0.98035418 | 0.02467515 | 124.7935563 | 228.3999043 |
| YNL144C | YNL144C | 0.91766977 | 0.98520231 | 0.02474189 | 124.6829497 | 228.1816022 |
| YOR189W | IES4 | 0.87136637 | 0.98248371 | 0.02483062 | 115.4132947 | 210.8719709 |
| YHR114W | BZZ1 | 0.91066383 | 0.98520231 | 0.02487383 | 126.8915043 | 232.2783199 |
| YJL017W | YJL017W | 0.88709073 | 0.98278659 | 0.0249164 | 119.9470376 | 219.3148937 |
| YLR128W | DCN1 | 0.87531563 | 0.98248371 | 0.02494397 | 108.607307 | 198.1542217 |
| YLR405W | DUS4 | 0.83833311 | 0.98035418 | 0.02503363 | 120.3356983 | 220.0189982 |
| YNR032W | PPG1 | 0.8140252 | 0.97642791 | 0.02512696 | 119.4496945 | 218.3493315 |
| YPL070W | MUK1 | 0.86950551 | 0.98248371 | 0.02519426 | 121.7373134 | 222.6051286 |
| YKL105C | YKL105C | 0.69407226 | 0.97135148 | 0.02527035 | 104.7544863 | 190.9078396 |
| YNL318C | HXT14 | 0.81406891 | 0.97642791 | 0.02530801 | 115.8943742 | 211.6839991 |
| YER045C | ACA1 | 0.88894709 | 0.98278659 | 0.02548628 | 120.1493311 | 219.5902496 |
| YGR201C | YGR201C | 0.90292937 | 0.98421339 | 0.02550232 | 127.9659127 | 234.1702228 |
| YDR203W | YDR203W | 0.77209649 | 0.97246744 | 0.02550593 | 114.6709396 | 209.3660824 |
| YIL086C | YIL086C | 0.88605235 | 0.98262107 | 0.02555127 | 114.6692307 | 209.3547751 |
| YGR072W | UPF3 | 0.9070503 | 0.98454853 | 0.02570027 | 105.9502835 | 193.0617674 |
| YLL043W | FPS1 | 0.76735931 | 0.97246744 | 0.02585907 | 100.9789821 | 183.7587239 |
| YLR154C | RNH203 | 0.62335423 | 0.96815259 | 0.02613273 | 95.34389216 | 173.1967287 |

| | | | | | | |
|---|---|---|---|---|---|---|
| YHR061C | GIC1 | 0.86926028 | 0.98248371 | 0.02616865 | 115.9798182 | 211.6892891 |
| YMR124W | YMR124W | 0.85265506 | 0.98105658 | 0.02647289 | 105.2999816 | 191.7101894 |
| YOR215C | YOR215C | 0.89961864 | 0.98385994 | 0.02658417 | 107.3772852 | 195.5657414 |
| YPR111W | DBF20 | 0.80423686 | 0.9762419 | 0.0266472 | 112.0184336 | 204.2131177 |
| YMR026C | PEX12 | 0.82969318 | 0.97952308 | 0.02676268 | 101.5328433 | 184.6302127 |
| YLR247C | IRC20 | 0.82812158 | 0.97933188 | 0.02691493 | 120.4084597 | 219.8178557 |
| YPL162C | YPL162C | 0.84217234 | 0.98035418 | 0.02699727 | 129.5003217 | 236.7651565 |
| YGR213C | RTA1 | 0.91053443 | 0.98520231 | 0.02715291 | 119.7251733 | 218.5004816 |
| YNL034W | YNL034W | 0.68587533 | 0.97133463 | 0.02727624 | 99.63026863 | 180.9887494 |
| YGR131W | YGR131W | 0.83210414 | 0.98013789 | 0.0273141 | 125.3040274 | 228.8796911 |
| YBR144C | YBR144C | 0.85245408 | 0.98105658 | 0.02747729 | 121.6886284 | 222.1054729 |
| YGL056C | SDS23 | 0.91005377 | 0.98520231 | 0.02752023 | 126.1273975 | 230.3788821 |
| YGR034W | RPL26B | 0.86804824 | 0.98248371 | 0.02770524 | 100.9494787 | 183.3730834 |
| YLR283W | YLR283W | 0.80924205 | 0.9762419 | 0.02777186 | 122.4328921 | 223.4412448 |
| YJL088W | ARG3 | 0.83382958 | 0.98013789 | 0.02778199 | 116.9495562 | 213.209557 |
| YHR096C | HXT5 | 0.73476058 | 0.97135148 | 0.02781069 | 118.3507767 | 215.818575 |
| YOL054W | PSH1 | 0.88387647 | 0.98248371 | 0.02786451 | 111.5136939 | 203.0534752 |
| YMR210W | YMR210W | 0.70300288 | 0.97135148 | 0.02799378 | 111.0880626 | 202.2362548 |
| YML021C | UNG1 | 0.71767128 | 0.97135148 | 0.02820555 | 86.56197121 | 156.4417333 |
| YJL215C | YJL215C | 0.87621937 | 0.98248371 | 0.02823402 | 113.4517562 | 206.6030111 |
| YGR269W | YGR269W | 0.89205388 | 0.98278659 | 0.02835296 | 118.3640372 | 215.7462096 |
| YOR242C | SSP2 | 0.75770299 | 0.97135148 | 0.02841023 | 123.4494157 | 225.2233867 |
| YBR263W | SHM1 | 0.67200904 | 0.97060928 | 0.02841401 | 114.1227212 | 207.8225524 |
| YIL029C | YIL029C | 0.88144476 | 0.98248371 | 0.02845452 | 117.4088027 | 213.9459097 |
| YOR358W | HAP5 | 0.69787324 | 0.97135148 | 0.02848071 | 85.86660089 | 155.0951568 |
| YBR297W | MAL33 | 0.78095603 | 0.97246744 | 0.02855134 | 108.1359356 | 196.628836 |
| YOL060C | MAM3 | 0.80982003 | 0.9762419 | 0.02862592 | 114.0089244 | 207.5723022 |
| YGR164W | YGR164W | 0.89034935 | 0.98278659 | 0.02864722 | 133.0060147 | 243.0100207 |
| YLR265C | NEJ1 | 0.80586672 | 0.9762419 | 0.02900104 | 110.4833813 | 200.9277715 |
| YLR309C | IMH1 | 0.83797545 | 0.98035418 | 0.02909739 | 111.8897923 | 203.5343602 |
| YNL249C | MPA43 | 0.76302562 | 0.97135148 | 0.02912232 | 117.4266872 | 213.8596897 |
| YPR118W | YPR118W | 0.86038869 | 0.98248371 | 0.02917569 | 116.9226644 | 212.9098139 |
| YOL163W | YOL163W | 0.85519333 | 0.98244245 | 0.02918625 | 123.7013486 | 225.5544363 |
| YKL149C | DBR1 | 0.61669771 | 0.96815259 | 0.02918938 | 99.67636379 | 180.7321538 |
| YCR085W | YCR085W | 0.75073277 | 0.97135148 | 0.02929036 | 109.9404519 | 199.8630577 |
| YGL241W | KAP114 | 0.83614752 | 0.98035418 | 0.02930964 | 120.0753228 | 218.7675193 |
| YIR003W | YIR003W | 0.81353769 | 0.97633618 | 0.02936936 | 119.0728144 | 216.8865157 |
| YJR040W | GEF1 | 0.86829415 | 0.98248371 | 0.02953214 | 121.5619227 | 221.5011208 |
| YDL177C | YDL177C | 0.72975656 | 0.97135148 | 0.02966366 | 118.8948413 | 216.5017834 |
| YAR020C | PAU7 | 0.79737172 | 0.97570065 | 0.02976153 | 123.2723294 | 224.651029 |
| YCR045C | YCR045C | 0.80281826 | 0.9762419 | 0.02977889 | 106.4214311 | 193.210387 |
| YBR177C | EHT1 | 0.82975866 | 0.97952308 | 0.0298895 | 127.4672955 | 232.4543643 |
| YNL082W | PMS1 | 0.81353405 | 0.97633618 | 0.03000867 | 99.26578782 | 179.8194595 |
| YMR120C | ADE17 | 0.2641037 | 0.96815259 | 0.0301463 | 111.9689812 | 203.4942662 |
| YLR437C | YLR437C | 0.4200711 | 0.96815259 | 0.03017819 | 119.4047084 | 217.3608685 |
| YJL070C | YJL070C | 0.72781382 | 0.97135148 | 0.03033835 | 103.064848 | 186.8480627 |
| YDR502C | SAM2 | 0.88640622 | 0.9827597 | 0.03051287 | 128.7833696 | 234.7980425 |
| YDR391C | YDR391C | 0.70073305 | 0.97135148 | 0.03055112 | 122.8168556 | 223.6598883 |
| YHL042W | YHL042W | 0.84531762 | 0.98035418 | 0.03058258 | 111.8319991 | 203.1605827 |
| YER179W | DMC1 | 0.87764231 | 0.98248371 | 0.03063223 | 108.6587676 | 197.2316175 |
| YMR153C-A | YMR153C-A | 0.77203857 | 0.97246744 | 0.03079381 | 106.2194793 | 192.651875 |
| YPL054W | LEE1 | 0.72111934 | 0.97135148 | 0.03096633 | 109.3213224 | 198.4078716 |
| YLR241W | YLR241W | 0.84698236 | 0.98035418 | 0.03107543 | 109.9721075 | 199.6024591 |
| YDL106C | PHO2 | 0.88523411 | 0.98248371 | 0.03121824 | 130.047984 | 237.0310326 |
| YMR178W | YMR178W | 0.63969891 | 0.96815259 | 0.03125465 | 112.0725202 | 203.4889574 |
| YNR048W | YNR048W | 0.83570641 | 0.98035418 | 0.03127674 | 122.7085567 | 223.3279054 |
| YKR058W | GLG1 | 0.83959469 | 0.98035418 | 0.03155615 | 123.9848824 | 225.659021 |
| YKR102W | FLO10 | 0.74370672 | 0.97135148 | 0.03166519 | 106.2243526 | 192.5049267 |
| YPL058C | PDR12 | 0.80655774 | 0.9762419 | 0.03170096 | 116.6585705 | 211.9649064 |
| YML070W | DAK1 | 0.8499685 | 0.98105658 | 0.03174722 | 114.9297611 | 208.7313051 |
| YHR135C | YCK1 | 0.88325147 | 0.98248371 | 0.03182459 | 123.4606082 | 224.6328499 |
| YBR219C | YBR219C | 0.77518176 | 0.97246744 | 0.03186965 | 129.8699893 | 236.5823104 |
| YGL222C | EDC1 | 0.84465727 | 0.98035418 | 0.03191519 | 118.4873968 | 215.3384562 |
| YNL235C | :::SIN4 | 0.67038463 | 0.97060928 | 0.03215516 | 6.077887367 | 5.580986059 |

| | | | | | | |
|---|---|---|---|---|---|---|
| YJL016W | YJL016W | 0.8129518 | 0.97633618 | 0.03218285 | 130.1357322 | 237.0220033 |
| YLR059C | REX2 | 0.64553093 | 0.96815259 | 0.03225376 | 100.5199838 | 181.757291 |
| YKL038W | RGT1 | 0.58660717 | 0.96815259 | 0.0322798 | 105.0248519 | 190.1570423 |
| YLR034C | SMF3 | 0.37511397 | 0.96815259 | 0.03231327 | 92.79487438 | 167.3344418 |
| YOR348C | PUT4 | 0.89112835 | 0.98278659 | 0.03233945 | 116.205118 | 211.004596 |
| YJL098W | SAP185 | 0.8524129 | 0.98105658 | 0.03245466 | 132.6008389 | 241.5723054 |
| YDL051W | LHP1 | 0.78549737 | 0.97260548 | 0.03248647 | 115.0041929 | 208.7377876 |
| YOR192C | THI72 | 0.84702236 | 0.98035418 | 0.0326337 | 131.6142969 | 239.6997231 |
| YOR296W | YOR296W | 0.82654091 | 0.97908046 | 0.03267335 | 124.7167744 | 226.8244002 |
| YMR052C-A | YMR052C-A | 0.40065412 | 0.96815259 | 0.03274082 | 112.4517651 | 203.9303565 |
| YKL147C | YKL147C | 0.80931372 | 0.9762419 | 0.03299385 | 126.5936764 | 230.2686127 |
| YDL079C | MRK1 | 0.62476426 | 0.96815259 | 0.03302219 | 116.178326 | 210.8323523 |
| YNL219C | ALG9 | 0.81920695 | 0.97821875 | 0.03315803 | 101.9084228 | 184.1856743 |
| YPL186C | UIP4 | 0.78340814 | 0.97260548 | 0.03316439 | 128.2512112 | 233.3304191 |
| YBR285W | YBR285W | 0.84569192 | 0.98035418 | 0.03317319 | 132.0970267 | 240.5037094 |
| YFR020W | YFR020W | 0.86755621 | 0.98248371 | 0.03319605 | 133.9722156 | 243.9980238 |
| YHR124W | NDT80 | 0.86691345 | 0.98248371 | 0.03320919 | 119.5507039 | 217.0904734 |
| YMR037C | MSN2 | 0.87445536 | 0.98248371 | 0.03321473 | 121.7555311 | 221.2028725 |
| YDR142C | PEX7 | 0.70188783 | 0.97135148 | 0.03327737 | 120.0553079 | 218.0196673 |
| YCL063W | VAC17 | 0.85797894 | 0.98248371 | 0.03328721 | 110.1475513 | 199.5337022 |
| YOR300W | YOR300W | 0.84665185 | 0.98035418 | 0.03333858 | 116.8525155 | 212.0334832 |
| YLR252W | YLR252W | 0.84194979 | 0.98035418 | 0.03349871 | 108.8152653 | 197.0102773 |
| YPL259C | APM1 | 0.80584124 | 0.9762419 | 0.03350908 | 127.0847813 | 231.0925684 |
| YOL136C | PFK27 | 0.8404251 | 0.98035418 | 0.03361579 | 120.1841549 | 218.1994475 |
| YDL039C | PRM7 | 0.64178583 | 0.96815259 | 0.03362758 | 121.2150081 | 220.1205258 |
| YML101C | CUE4 | 0.82398658 | 0.97873587 | 0.0336382 | 114.0168609 | 206.6895492 |
| YPR001W | CIT3 | 0.74845838 | 0.97135148 | 0.03369939 | 118.4737436 | 214.9934826 |
| YKL087C | CYT2 | 0.65737672 | 0.96815259 | 0.03379079 | 97.05205533 | 175.0121817 |
| YIR038C | GTT1 | 0.82596078 | 0.97887121 | 0.03379893 | 119.8341635 | 217.5136985 |
| YDR135C | YCF1 | 0.73863066 | 0.97135148 | 0.03385417 | 118.5694596 | 215.1443374 |
| YLR350W | ORM2 | 0.7991712 | 0.9762419 | 0.03387302 | 118.9118129 | 215.7796658 |
| YOL029C | YOL029C | 0.76116413 | 0.97135148 | 0.03390769 | 125.2392021 | 227.578022 |
| YKL211C | TRP3 | 0.57243099 | 0.96815259 | 0.03397838 | 99.55382539 | 179.6459655 |
| YOR158W | PET123 | 0.7552054 | 0.97135148 | 0.03409481 | 108.4708312 | 196.2609448 |
| YLR016C | PML1 | 0.79936553 | 0.9762419 | 0.03423456 | 116.2924464 | 210.8281557 |
| YDR383C | NKP1 | 0.69521862 | 0.97135148 | 0.03424163 | 120.5952583 | 218.854343 |
| YAL008W | FUN14 | 0.77368133 | 0.97246744 | 0.03425093 | 127.0991372 | 230.9865068 |
| YNR013C | PHO91 | 0.74848651 | 0.97135148 | 0.03437262 | 106.8376179 | 193.1642272 |
| YOR105W | YOR105W | 0.82975823 | 0.97952308 | 0.03458929 | 119.8005606 | 217.309476 |
| YNL302C | RPS19B | 0.82070493 | 0.97873587 | 0.03476053 | 77.58202328 | 138.5146624 |
| YOR277C | YOR277C | 0.78576766 | 0.97260548 | 0.03489761 | 111.3522075 | 201.4927668 |
| YMR052W | FAR3 | 0.48159587 | 0.96815259 | 0.03493754 | 112.596273 | 203.8065826 |
| YKR065C | PAM17 | 0.42765179 | 0.96815259 | 0.0349442 | 106.3959415 | 192.2378687 |
| YPR134W | MSS18 | 0.84056339 | 0.98035418 | 0.03496413 | 100.0410319 | 180.3783916 |
| YFR014C | CMK1 | 0.85288407 | 0.98105658 | 0.03507823 | 139.1337353 | 253.2904607 |
| YDR218C | SPR28 | 0.82493195 | 0.97873587 | 0.03509352 | 130.407413 | 237.0076389 |
| YLR125W | YLR125W | 0.84050776 | 0.98035418 | 0.03516328 | 111.9163192 | 202.497617 |
| YFR040W | SAP155 | 0.72431573 | 0.97135148 | 0.03519489 | 104.1330263 | 187.9712154 |
| YPL032C | SVL3 | 0.81108188 | 0.9762419 | 0.03531562 | 123.5884909 | 224.2462847 |
| YER075C | PTP3 | 0.88348791 | 0.98248371 | 0.03545615 | 114.9584171 | 208.120599 |
| YKL123W | YKL123W | 0.70728161 | 0.97135148 | 0.0354885 | 113.0684615 | 204.5888482 |
| YGR089W | NNF2 | 0.81648555 | 0.97741538 | 0.0358637 | 98.53798215 | 177.4131694 |
| YPR155C | NCA2 | 0.84882385 | 0.98085678 | 0.0358709 | 114.18605 | 206.6053771 |
| YLR406C | RPL31B | 0.83299139 | 0.98013789 | 0.03587661 | 110.0076345 | 198.8089796 |
| YPL138C | SPP1 | 0.85863706 | 0.98248371 | 0.03588354 | 107.7177255 | 194.5356168 |
| YMR004W | MVP1 | 0.84465149 | 0.98035418 | 0.03589167 | 113.930244 | 206.1244188 |
| YLR286C | CTS1 | 0.82263628 | 0.97873587 | 0.03641418 | 117.0850909 | 211.9166284 |
| YIL009C-A | EST3 | 0.8520223 | 0.98105658 | 0.03647219 | 121.2301772 | 219.6394348 |
| YOR377W | ATF1 | 0.83960425 | 0.98035418 | 0.03648913 | 121.5387349 | 220.212055 |
| YIL059C | YIL059C | 0.85627373 | 0.98248371 | 0.03649081 | 130.0980601 | 236.1802854 |
| YOR155C | ISN1 | 0.7932919 | 0.9738123 | 0.03650412 | 117.374318 | 212.4401132 |
| YNL109W | YNL109W | 0.83339461 | 0.98013789 | 0.03654548 | 104.3626249 | 188.1577082 |
| YNL277W | MET2 | 0.65130758 | 0.96815259 | 0.03661917 | 115.7205341 | 209.3341618 |
| YOR100C | CRC1 | 0.76662169 | 0.97246744 | 0.03665973 | 113.216175 | 204.6546927 |

| | | | | | | |
|---|---|---|---|---|---|---|
| YCL038C | ATG22 | 0.80115268 | 0.9762419 | 0.03688483 | 131.9257774 | 239.5195704 |
| YBR101C | FES1 | 0.81036807 | 0.9762419 | 0.03707537 | 128.9377159 | 233.9108341 |
| YNL332W | THI12 | 0.77340346 | 0.97246744 | 0.03715121 | 125.2662302 | 227.0476201 |
| YFL043C | YFL043C | 0.86088763 | 0.98248371 | 0.03715552 | 114.7278517 | 207.3861397 |
| YCR043C | YCR043C | 0.62924635 | 0.96815259 | 0.03721389 | 119.5811734 | 216.4301863 |
| YBR077C | SLM4 | 0.82006922 | 0.97870407 | 0.0373161 | 129.5361745 | 234.9842278 |
| YMR154C | RIM13 | 0.82225178 | 0.97873587 | 0.03735001 | 105.4034597 | 189.9554512 |
| YLR253W | YLR253W | 0.75958551 | 0.97135148 | 0.03736595 | 123.1738336 | 223.1055299 |
| YGL124C | MON1 | 0.72975824 | 0.97135148 | 0.03737236 | 78.19101112 | 139.1831003 |
| YKL044W | YKL044W | 0.55651058 | 0.96815259 | 0.03752384 | 114.940664 | 207.7172122 |
| YOR086C | TCB1 | 0.85737254 | 0.98248371 | 0.03772108 | 130.1774959 | 236.1081758 |
| YER081W | SER3 | 0.8246218 | 0.97873587 | 0.03775285 | 110.7131043 | 199.7891428 |
| YCR087W | YCR087W | 0.79687812 | 0.97570065 | 0.0378274 | 114.0826523 | 206.0621203 |
| YLR189C | ATG26 | 0.80480322 | 0.9762419 | 0.03793427 | 117.3367365 | 212.1138993 |
| YFL025C | BST1 | 0.78732572 | 0.97260548 | 0.03793668 | 112.0957066 | 202.3356481 |
| YBL102W | SFT2 | 0.77101848 | 0.97246744 | 0.03801903 | 129.3576597 | 234.5253098 |
| YGR193C | PDX1 | 0.88195543 | 0.98248371 | 0.03805848 | 139.696008 | 253.8057729 |
| YLR420W | URA4 | 0.86254797 | 0.98248371 | 0.03807489 | 116.6816718 | 210.8666104 |
| YPL236C | YPL236C | 0.8363977 | 0.98035418 | 0.03818364 | 135.4240773 | 245.8135206 |
| YLR380W | CSR1 | 0.78198033 | 0.97260548 | 0.03842938 | 113.6034077 | 205.0602308 |
| YPR117W | YPR117W | 0.84830311 | 0.98085678 | 0.03869599 | 114.5980202 | 206.8680663 |
| YHL014C | YLF2 | 0.78602966 | 0.97260548 | 0.03870006 | 108.1834701 | 194.9001626 |
| YJR137C | ECM17 | 0.83786143 | 0.98035418 | 0.0387225 | 105.2485107 | 189.4205985 |
| YLR152C | YLR152C | 0.74407699 | 0.97135148 | 0.03879469 | 120.1027064 | 217.1200976 |
| YMR175W | SIP18 | 0.86148285 | 0.98248371 | 0.03889674 | 121.581735 | 219.8611426 |
| YNR074C | AIF1 | 0.84069192 | 0.98035418 | 0.03902602 | 119.4697597 | 215.897828 |
| YDR179W-A | YDR179W-A | 0.67638081 | 0.97060928 | 0.03906084 | 121.2858804 | 219.279801 |
| YLR392C | YLR392C | 0.77478144 | 0.97246744 | 0.03912491 | 126.5996202 | 229.1817973 |
| YJL066C | MPM1 | 0.66272165 | 0.96927465 | 0.03922493 | 118.620629 | 214.2780445 |
| YAL017W | PSK1 | 0.72061127 | 0.97135148 | 0.03930574 | 128.2599993 | 232.2470679 |
| YJR111C | YJR111C | 0.67603599 | 0.97060928 | 0.0393607 | 110.0473279 | 198.2591286 |
| YFL012W | YFL012W | 0.88074312 | 0.98248371 | 0.03943297 | 119.8038708 | 216.4482819 |
| YGR037C | ACB1 | 0.80376147 | 0.9762419 | 0.03943394 | 131.5140424 | 238.2949507 |
| YLL009C | COX17 | 0.46405759 | 0.96815259 | 0.03947887 | 76.35355276 | 135.3778663 |
| YDL093W | PMT5 | 0.67903718 | 0.97060928 | 0.03955659 | 115.1946562 | 207.8270574 |
| YIL167W | SDL1 | 0.84407845 | 0.98035418 | 0.03960755 | 118.3352531 | 213.6771222 |
| YNL200C | YNL200C | 0.65001803 | 0.96815259 | 0.0396868 | 115.7681693 | 208.8737048 |
| YDR026C | YDR026C | 0.69660029 | 0.97135148 | 0.03970533 | 108.9508551 | 196.1518039 |
| YMR189W | GCV2 | 0.7017077 | 0.97135148 | 0.03979243 | 118.9375277 | 214.7676374 |
| YGR052W | FMP48 | 0.84672027 | 0.98035418 | 0.0398082 | 113.0234394 | 203.7313161 |
| YIL054W | YIL054W | 0.87386727 | 0.98248371 | 0.03983802 | 137.8718458 | 250.083896 |
| YOR292C | YOR292C | 0.76036104 | 0.97135148 | 0.0399959 | 125.2965228 | 226.5947299 |
| YLR122C | YLR122C | 0.75843952 | 0.97135148 | 0.04000023 | 113.5825791 | 204.7400759 |
| YPL052W | OAZ1 | 0.7815522 | 0.97246744 | 0.04005633 | 124.7475225 | 225.5596776 |
| YKL015W | PUT3 | 0.63293137 | 0.96815259 | 0.04013831 | 116.57712 | 210.3020536 |
| YIL133C | RPL16A | 0.7279871 | 0.97135148 | 0.04019068 | 100.882383 | 181.0121113 |
| YBR276C | PPS1 | 0.59222881 | 0.96815259 | 0.04028063 | 117.3547967 | 211.7274245 |
| YCL055W | KAR4 | 0.55639484 | 0.96815259 | 0.04038484 | 119.6161163 | 215.927547 |
| YJL048C | UBX6 | 0.82682008 | 0.97914105 | 0.04049211 | 126.4931062 | 228.7382535 |
| YHR127W | YHR127W | 0.8489787 | 0.98085678 | 0.04049224 | 124.3807639 | 224.7973826 |
| YMR102C | YMR102C | 0.41668986 | 0.96815259 | 0.04064008 | 109.9813466 | 197.9069312 |
| YLR137W | YLR137W | 0.79048781 | 0.97287322 | 0.04065852 | 121.2730271 | 218.9697218 |
| YML103C | NUP188 | 0.74536505 | 0.97135148 | 0.04092743 | 91.73660999 | 163.8175546 |
| YAL042W | ERV46 | 0.71125866 | 0.97135148 | 0.0409723 | 134.972838 | 244.4723034 |
| YER071C | YER071C | 0.82399796 | 0.97873587 | 0.04103791 | 129.3358375 | 233.943999 |
| YBL083C | YBL083C | 0.66496436 | 0.96985526 | 0.04104383 | 124.2285614 | 224.4146548 |
| YGL037C | PNC1 | 0.57292369 | 0.96815259 | 0.04115342 | 119.0435841 | 214.7217826 |
| YCL023C | YCL023C | 0.7510195 | 0.97135148 | 0.04121182 | 130.0553586 | 235.2552172 |
| YMR306C-A | YMR306C-A | 0.62944257 | 0.96815259 | 0.04127097 | 115.1898222 | 207.5110412 |
| YFR013W | IOC3 | 0.81080623 | 0.9762419 | 0.04157304 | 110.923726 | 199.497994 |
| YIL085C | KTR7 | 0.70756556 | 0.97135148 | 0.04159213 | 115.3354825 | 207.7252792 |
| YOR059C | YOR059C | 0.76350885 | 0.97135148 | 0.04169101 | 129.5640826 | 234.2528682 |
| YNR027W | BUD17 | 0.76801435 | 0.97246744 | 0.04173702 | 116.529085 | 209.9261532 |
| YLL016W | SDC25 | 0.71880715 | 0.97135148 | 0.04192968 | 125.8088929 | 227.2043359 |

| | | | | | | |
|---|---|---|---|---|---|---|
| YMR057C | YMR057C | 0.79014129 | 0.97272554 | 0.04192972 | 109.6819815 | 197.1174869 |
| YMR122C | YMR122C | 0.818293 | 0.97821552 | 0.04196623 | 111.0482814 | 199.6599591 |
| YOR087W | YVC1 | 0.82453083 | 0.97873587 | 0.04207974 | 121.4759307 | 219.0937632 |
| YPL095C | EEB1 | 0.73418057 | 0.97135148 | 0.04211973 | 126.8857277 | 229.1792783 |
| YBR240C | THI2 | 0.57143274 | 0.96815259 | 0.04221688 | 122.7163318 | 221.3833346 |
| YPL113C | YPL113C | 0.86984029 | 0.98248371 | 0.04228242 | 111.2316029 | 199.9453483 |
| YML056C | IMD4 | 0.79650906 | 0.97570065 | 0.04236596 | 117.188274 | 211.0433292 |
| YMR171C | YMR171C | 0.78474172 | 0.97260548 | 0.04250209 | 118.2923991 | 213.078841 |
| YKR106W | YKR106W | 0.51773508 | 0.96815259 | 0.04257834 | 116.629488 | 209.9628099 |
| YGL230C | YGL230C | 0.68334707 | 0.97060928 | 0.04277047 | 120.1126516 | 216.4266979 |
| YBR222C | PCS60 | 0.69109374 | 0.97135148 | 0.04279124 | 132.5663765 | 239.657015 |
| YPR052C | NHP6A | 0.82318995 | 0.97873587 | 0.04293181 | 121.0132202 | 218.0779346 |
| YER062C | HOR2 | 0.59515242 | 0.96815259 | 0.04293611 | 106.4501436 | 190.9078583 |
| YJL084C | ALY2 | 0.73397296 | 0.97135148 | 0.04318095 | 113.654109 | 204.303946 |
| YFR018C | YFR018C | 0.77726171 | 0.97246744 | 0.0432119 | 129.3270366 | 233.5382793 |
| YLR390W-A | CCW14 | 0.44875595 | 0.96815259 | 0.04342205 | 97.98293539 | 175.0241673 |
| YER054C | GIP2 | 0.75110212 | 0.97135148 | 0.04344418 | 116.3181658 | 209.2269517 |
| YJR077C | MIR1 | 0.6945846 | 0.97135148 | 0.04347294 | 88.09268509 | 156.5635103 |
| YDR105C | TMS1 | 0.6724355 | 0.97060928 | 0.04347799 | 117.9964347 | 212.351925 |
| YAR042W | SWH1 | 0.71150787 | 0.97135148 | 0.04368569 | 122.4944071 | 220.7062817 |
| YLR349W | YLR349W | 0.79228177 | 0.97340714 | 0.04368787 | 124.6963242 | 224.8138518 |
| YPL062W | YPL062W | 0.82402492 | 0.97873587 | 0.04376034 | 108.1340332 | 193.9017764 |
| YOR308C | SNU66 | 0.68353804 | 0.97060928 | 0.04388388 | 111.9453883 | 200.9902306 |
| YJL169W | YJL169W | 0.81786543 | 0.97821552 | 0.04391009 | 120.3367339 | 216.6406794 |
| YLR181C | VTA1 | 0.59958436 | 0.96815259 | 0.04400803 | 110.8934302 | 199.0054348 |
| YBR259W | YBR259W | 0.70049622 | 0.97135148 | 0.04407567 | 120.2755184 | 216.496823 |
| YLR221C | RSA3 | 0.78767203 | 0.97272554 | 0.04415837 | 121.7506572 | 219.234075 |
| YPR130C | YPR130C | 0.83071126 | 0.97994418 | 0.04446134 | 120.1674656 | 216.2261735 |
| YKL006W | RPL14A | 0.64271216 | 0.96815259 | 0.04450487 | 7.795700176 | 6.57429619 |
| YOR279C | RFM1 | 0.84700227 | 0.98035418 | 0.04451572 | 129.8027543 | 234.1923147 |
| YBR178W | YBR178W | 0.75918127 | 0.97135148 | 0.04454415 | 137.3928647 | 248.3475562 |
| YPL256C | CLN2 | 0.81528231 | 0.97741538 | 0.04461788 | 119.9567337 | 215.8049939 |
| YOL071W | EMI5 | 0.14015461 | 0.96815259 | 0.04473259 | 102.8208571 | 183.8152551 |
| YPL197C | YPL197C | 0.75210313 | 0.97135148 | 0.04497001 | 124.5542479 | 224.3191942 |
| YDR532C | KRE28 | 0.72658235 | 0.97135148 | 0.04537476 | 18.54722627 | 26.47688776 |
| YPL219W | PCL8 | 0.85266885 | 0.98105658 | 0.04548348 | 125.6618522 | 226.2936264 |
| YLR164W | YLR164W | 0.71886518 | 0.97135148 | 0.0455902 | 116.9558059 | 210.0322588 |
| YCL040W | GLK1 | 0.66864811 | 0.9703088 | 0.04566772 | 119.2509578 | 214.3002792 |
| YOL162W | YOL162W | 0.80620943 | 0.9762419 | 0.04575019 | 121.9038057 | 219.2347425 |
| YGR007W | MUQ1 | 0.78993063 | 0.97272554 | 0.0457872 | 120.0880063 | 215.840507 |
| YCR049C | YCR049C | 0.64629043 | 0.96815259 | 0.04587452 | 127.0742197 | 228.8585558 |
| YHR044C | DOG1 | 0.82160613 | 0.97873587 | 0.04597496 | 126.2715198 | 227.3430295 |
| YLR438W | CAR2 | 0.7809599 | 0.97246744 | 0.04601976 | 118.1081965 | 212.1052704 |
| YHR202W | YHR202W | 0.85670708 | 0.98248371 | 0.04613797 | 128.6639969 | 231.7773143 |
| YDR483W | KRE2 | 0.65725246 | 0.96815259 | 0.04618282 | 112.2445709 | 201.1367165 |
| YMR156C | TPP1 | 0.59372336 | 0.96815259 | 0.04622199 | 108.7505504 | 194.6111558 |
| YKL198C | PTK1 | 0.40961911 | 0.96815259 | 0.0462271 | 121.3612816 | 218.1371931 |
| YML113W | DAT1 | 0.83154532 | 0.98013789 | 0.0462381 | 120.0776788 | 215.7404947 |
| YBR264C | YPT10 | 0.68925069 | 0.97135148 | 0.04650337 | 133.6634917 | 241.0390866 |
| YMR130W | YMR130W | 0.74924036 | 0.97135148 | 0.04682164 | 126.4201648 | 227.4687284 |
| YPL109C | YPL109C | 0.74047715 | 0.97135148 | 0.04690314 | 115.4451538 | 206.9788293 |
| YOR129C | YOR129C | 0.84386999 | 0.98035418 | 0.04714341 | 126.9240253 | 228.3511256 |
| YGL094C | PAN2 | 0.81601262 | 0.97741538 | 0.04728789 | 126.8601369 | 228.2060607 |
| YLR289W | GUF1 | 0.75817885 | 0.97135148 | 0.04759486 | 116.7840461 | 209.3528395 |
| YDR130C | FIN1 | 0.57660113 | 0.96815259 | 0.04764733 | 121.9178137 | 218.9211522 |
| YIL034C | CAP2 | 0.81563489 | 0.97741538 | 0.04767274 | 113.8026028 | 203.7766237 |
| YJL120W | YJL120W | 0.81828211 | 0.97821552 | 0.04796557 | 93.77201925 | 166.354538 |
| YPL261C | YPL261C | 0.77765047 | 0.97246744 | 0.0479867 | 113.1113328 | 202.4307482 |
| YGR066C | YGR066C | 0.65851389 | 0.96815259 | 0.0479978 | 134.3340218 | 242.0224354 |
| YGR016W | YGR016W | 0.73162019 | 0.97135148 | 0.04850464 | 127.7920868 | 229.7268488 |
| YIL056W | VHR1 | 0.81275527 | 0.97633618 | 0.04867628 | 124.0333422 | 222.6836873 |
| YPL089C | RLM1 | 0.80424596 | 0.9762419 | 0.04870757 | 126.4136267 | 227.1188131 |
| YGL080W | FMP37 | 0.50420943 | 0.96815259 | 0.04889828 | 89.04828721 | 157.3747828 |
| YLR330W | CHS5 | 0.62151118 | 0.96815259 | 0.04909612 | 104.5104702 | 186.1860595 |

| | | | | | | |
|---|---|---|---|---|---|---|
| YLR329W | REC102 | 0.76064742 | 0.97135148 | 0.04943885 | 126.8950787 | 227.8860725 |
| YDL243C | AAD4 | 0.62985319 | 0.96815259 | 0.04955026 | 127.9083227 | 229.75646 |
| YJL036W | SNX4 | 0.77912365 | 0.97246744 | 0.04966511 | 127.2629597 | 228.5318849 |
| YJL013C | MAD3 | 0.80992987 | 0.9762419 | 0.04967528 | 124.1020182 | 222.6329171 |
| YML058W | SML1 | 0.77904122 | 0.97246744 | 0.04986594 | 113.2221904 | 202.3010484 |
| YDL127W | PCL2 | 0.66624943 | 0.9703088 | 0.0499127 | 131.1887288 | 235.8115791 |
| YLR238W | FAR10 | 0.7104516 | 0.97135148 | 0.05002596 | 117.3003702 | 209.8807664 |
| YGL146C | YGL146C | 0.77917949 | 0.97246744 | 0.05004765 | 121.7852805 | 218.2440629 |
| YOR179C | SYC1 | 0.57312041 | 0.96815259 | 0.05005643 | 104.17814 | 185.3940899 |
| YMR105C | PGM2 | 0.54101533 | 0.96815259 | 0.05023397 | 111.0255257 | 198.1369821 |
| YIL114C | POR2 | 0.75946448 | 0.97135148 | 0.05057426 | 134.1352833 | 241.1902908 |
| YLR053C | YLR053C | 0.45710601 | 0.96815259 | 0.05059196 | 103.6777151 | 184.3645825 |
| YPL230W | YPL230W | 0.75779974 | 0.97135148 | 0.05060322 | 118.8302909 | 212.6316602 |
| YLR351C | NIT3 | 0.75553218 | 0.97135148 | 0.05062617 | 115.1470481 | 205.7559831 |
| YBR284W | YBR284W | 0.79532444 | 0.97547077 | 0.05074569 | 118.4783809 | 211.9496131 |
| YDR423C | CAD1 | 0.75548748 | 0.97135148 | 0.05079853 | 126.9497614 | 227.7446094 |
| YEL004W | YEA4 | 0.73389706 | 0.97135148 | 0.05081545 | 125.9794402 | 225.931319 |
| YOR067C | ALG8 | 0.76671288 | 0.97246744 | 0.05085346 | 110.2745948 | 196.6250896 |
| YOL105C | WSC3 | 0.80848728 | 0.9762419 | 0.05093253 | 119.0946921 | 213.0659634 |
| YOR163W | DDP1 | 0.81021991 | 0.9762419 | 0.05105741 | 125.839762 | 225.6274031 |
| YLR353W | BUD8 | 0.74549132 | 0.97135148 | 0.05109159 | 120.3028029 | 215.2913669 |
| YGR014W | MSB2 | 0.70203842 | 0.97135148 | 0.05110573 | 124.3264593 | 222.7954871 |
| YHR146W | CRP1 | 0.76020382 | 0.97135148 | 0.05119296 | 127.3242319 | 228.3726005 |
| YML019W | OST6 | 0.82735891 | 0.97923902 | 0.05123644 | 116.6601986 | 208.4696789 |
| YNR033W | ABZ1 | 0.75019025 | 0.97135148 | 0.05126935 | 115.9345405 | 207.1099757 |
| YGL153W | PEX14 | 0.75826181 | 0.97135148 | 0.05127412 | 130.9102866 | 235.0483157 |
| YER177W | BMH1 | 0.73253387 | 0.97135148 | 0.05145276 | 114.2409018 | 203.917431 |
| YMR262W | YMR262W | 0.75298251 | 0.97135148 | 0.0515306 | 109.8312366 | 195.6766906 |
| YGR146C | YGR146C | 0.6144943 | 0.96815259 | 0.0516946 | 123.6796577 | 221.4833449 |
| YAL007C | ERP2 | 0.65762983 | 0.96815259 | 0.05175478 | 125.1167572 | 224.1536634 |
| YHR113W | YHR113W | 0.78639556 | 0.97260548 | 0.05177357 | 120.4849314 | 215.5090275 |
| YDR378C | LSM6 | 0.90651347 | 0.98454853 | 0.0519807 | 36.54342947 | 58.86819414 |
| YLR296W | YLR296W | 0.71762039 | 0.97135148 | 0.05228009 | 130.9419945 | 234.9273308 |
| YGR233C | PHO81 | 0.80879009 | 0.9762419 | 0.05232584 | 126.6943304 | 226.9945708 |
| YOR384W | FRE5 | 0.70704747 | 0.97135148 | 0.05239896 | 132.2338043 | 237.3160829 |
| YBL067C | UBP13 | 0.49023862 | 0.96815259 | 0.05240685 | 121.0649733 | 216.4777682 |
| YMR073C | IRC21 | 0.77275902 | 0.97246744 | 0.05245809 | 101.8354657 | 180.5934567 |
| YHR093W | AHT1 | 0.81137588 | 0.9762419 | 0.0525779 | 136.6770263 | 245.5734448 |
| YIL105C | SLM1 | 0.66677637 | 0.9703088 | 0.05259697 | 129.2249295 | 231.6671788 |
| YAL031C | GIP4 | 0.56352335 | 0.96815259 | 0.05265009 | 124.2165894 | 222.3139591 |
| YLR211C | YLR211C | 0.75780105 | 0.97135148 | 0.0526584 | 115.1342187 | 205.3681316 |
| YPR097W | YPR097W | 0.68269746 | 0.97060928 | 0.05269191 | 117.8412701 | 210.4124864 |
| YNL024C | YNL024C | 0.650189 | 0.96815259 | 0.05271132 | 119.7768206 | 214.0200316 |
| YMR316W | DIA1 | 0.59170908 | 0.96815259 | 0.05275137 | 105.351538 | 187.1006263 |
| YOR092W | ECM3 | 0.77887555 | 0.97246744 | 0.05295627 | 126.8456754 | 227.1640316 |
| YPL004C | LSP1 | 0.77523547 | 0.97246744 | 0.0530497 | 123.3574567 | 220.6395772 |
| YOL131W | YOL131W | 0.68296068 | 0.97060928 | 0.05317722 | 109.0896152 | 193.9982353 |
| YDR192C | NUP42 | 0.4665673 | 0.96815259 | 0.0531878 | 121.5974597 | 217.331344 |
| YOR191W | RIS1 | 0.70075326 | 0.97135148 | 0.05331333 | 108.2666815 | 192.4385724 |
| YOR366W | YOR366W | 0.78113266 | 0.97246744 | 0.05335199 | 126.9062564 | 227.2061895 |
| YMR002W | MIC17 | 0.71437567 | 0.97135148 | 0.05336442 | 109.0718574 | 193.9315834 |
| YFL041W | FET5 | 0.744787 | 0.97135148 | 0.05338277 | 121.6799012 | 217.4502366 |
| YIL005W | EPS1 | 0.77688511 | 0.97246744 | 0.053416 | 120.9839992 | 216.1459906 |
| YPL051W | ARL3 | 0.67838432 | 0.97060928 | 0.05343853 | 123.5399609 | 220.9104335 |
| YLR279W | YLR279W | 0.74896988 | 0.97135148 | 0.05365485 | 132.6345497 | 237.8388294 |
| YMR035W | IMP2 | 0.73645247 | 0.97135148 | 0.05368641 | 109.8738538 | 195.3701517 |
| YHL041W | YHL041W | 0.78528426 | 0.97260548 | 0.05385111 | 120.3344453 | 214.8562479 |
| YLR404W | YLR404W | 0.70674689 | 0.97135148 | 0.05393207 | 97.64213383 | 172.506303 |
| YLR085C | ARP6 | 0.55295794 | 0.96815259 | 0.053998 | 96.55573838 | 170.4676848 |
| YAL062W | GDH3 | 0.60070011 | 0.96815259 | 0.05401804 | 119.6231339 | 213.4993116 |
| YHR110W | ERP5 | 0.76851107 | 0.97246744 | 0.0540925 | 110.9169208 | 197.2434086 |
| YLR412W | YLR412W | 0.77490891 | 0.97246744 | 0.05409313 | 103.8824894 | 184.1196544 |
| YER164W | CHD1 | 0.69464956 | 0.97135148 | 0.05409969 | 121.213155 | 216.4510795 |
| YPL061W | ALD6 | 0.74520419 | 0.97135148 | 0.05444347 | 112.1334631 | 199.4501772 |

| | | | | | | |
|---|---|---|---|---|---|---|
| YNL309W | STB1 | 0.82424811 | 0.97873587 | 0.05461776 | 122.3239345 | 218.4306116 |
| YML037C | YML037C | 0.77124914 | 0.97246744 | 0.05476476 | 121.6372238 | 217.1231398 |
| YGR157W | CHO2 | 0.71830736 | 0.97135148 | 0.05480177 | 17.5381774 | 22.90625845 |
| YIR020W-B | YIR020W-B | 0.76183806 | 0.97135148 | 0.05480204 | 127.8562032 | 228.7187751 |
| YAR027W | UIP3 | 0.64213255 | 0.96815259 | 0.05480355 | 121.4051585 | 216.6832457 |
| YLR119W | SRN2 | 0.58333962 | 0.96815259 | 0.05495385 | 95.69166787 | 168.684484 |
| YGL234W | ADE5 | 0.49102737 | 0.96815259 | 0.05508414 | 116.7052664 | 207.864743 |
| YGL160W | YGL160W | 0.70953513 | 0.97135148 | 0.05510767 | 121.6183752 | 217.026569 |
| YMR244C-A | YMR244C-A | 0.31728794 | 0.96815259 | 0.05518739 | 102.83761 | 181.9743443 |
| YLR449W | FPR4 | 0.48634986 | 0.96815259 | 0.05520281 | 97.31838437 | 171.6747526 |
| YLR098C | CHA4 | 0.57598066 | 0.96815259 | 0.05533295 | 99.26123495 | 175.2760879 |
| YNL295W | YNL295W | 0.2459188 | 0.96815259 | 0.05536284 | 109.5344928 | 194.4368283 |
| YGL196W | YGL196W | 0.73717062 | 0.97135148 | 0.05551227 | 117.772435 | 209.7790173 |
| YMR273C | ZDS1 | 0.76210504 | 0.97135148 | 0.05552623 | 127.9781004 | 228.8165074 |
| YML067C | ERV41 | 0.58525769 | 0.96815259 | 0.05555724 | 111.3431906 | 197.7763768 |
| YKL090W | CUE2 | 0.53479289 | 0.96815259 | 0.05563485 | 116.7182243 | 207.7903005 |
| YJR154W | YJR154W | 0.44798796 | 0.96815259 | 0.05566246 | 116.5873304 | 207.5411566 |
| YAL055W | PEX22 | 0.53748827 | 0.96815259 | 0.05566923 | 139.5479858 | 250.3760193 |
| YGL151W | NUT1 | 0.39100222 | 0.96815259 | 0.05580255 | 5.356204738 | 0 |
| YBL013W | FMT1 | 0.7044642 | 0.97135148 | 0.05582644 | 119.018421 | 212.0473055 |
| YFL033C | RIM15 | 0.73220219 | 0.97135148 | 0.05586429 | 104.1934408 | 184.3826077 |
| YPL035C | YPL035C | 0.61489975 | 0.96815259 | 0.05588878 | 106.5321236 | 188.7413375 |
| YGL262W | YGL262W | 0.64222719 | 0.96815259 | 0.05617808 | 124.0462485 | 221.3644007 |
| YPL119C | DBP1 | 0.72368127 | 0.97135148 | 0.05619637 | 122.8166873 | 219.0672199 |
| YJL105W | SET4 | 0.74999324 | 0.97135148 | 0.05624443 | 112.241963 | 199.3300949 |
| YLR092W | SUL2 | 0.70672027 | 0.97135148 | 0.05628103 | 115.15761 | 204.7630593 |
| YKL070W | YKL070W | 0.50675933 | 0.96815259 | 0.05634238 | 114.4242034 | 203.3838078 |
| YDR229W | IVY1 | 0.61345254 | 0.96815259 | 0.05634426 | 123.39766 | 220.1246161 |
| YDR249C | YDR249C | 0.48970487 | 0.96815259 | 0.05667522 | 127.4902673 | 227.7006394 |
| YBR107C | IML3 | 0.75410285 | 0.97135148 | 0.05668055 | 126.1898914 | 225.2736664 |
| YBL056W | PTC3 | 0.75639062 | 0.97135148 | 0.05671277 | 128.3407128 | 229.2805315 |
| YER057C | HMF1 | 0.60938446 | 0.96815259 | 0.05674025 | 94.3884997 | 165.9333609 |
| YOR283W | YOR283W | 0.7780888 | 0.97246744 | 0.05682136 | 124.6746353 | 222.421544 |
| YLR248W | RCK2 | 0.69559776 | 0.97135148 | 0.05683698 | 117.7148342 | 209.4343369 |
| YMR110C | HFD1 | 0.4631976 | 0.96815259 | 0.05686353 | 117.2501192 | 208.5625968 |
| YLR455W | YLR455W | 0.54635346 | 0.96815259 | 0.05712841 | 103.7820314 | 183.388701 |
| YPR061C | JID1 | 0.55200747 | 0.96815259 | 0.05716828 | 108.4527277 | 192.0953508 |
| YOL086C | ADH1 | 0.6767358 | 0.97060928 | 0.05717866 | 105.9648353 | 187.4520063 |
| YML020W | YML020W | 0.64850488 | 0.96815259 | 0.05720471 | 115.1450361 | 204.5741957 |
| YDR426C | YDR426C | 0.63441483 | 0.96815259 | 0.05720932 | 121.1276166 | 215.7346482 |
| YKL005C | BYE1 | 0.51095653 | 0.96815259 | 0.05733805 | 115.2808843 | 204.8037594 |
| YLR070C | XYL2 | 0.7276514 | 0.97135148 | 0.05737265 | 121.3157827 | 216.0564489 |
| YAR035W | YAT1 | 0.60237773 | 0.96815259 | 0.05741732 | 129.1613946 | 230.6854539 |
| YER004W | FMP52 | 0.75541165 | 0.97135148 | 0.05745848 | 121.3537604 | 216.1119302 |
| YNR014W | YNR014W | 0.85844708 | 0.98248371 | 0.05746657 | 120.5457796 | 214.6030891 |
| YDR286C | YDR286C | 0.51886287 | 0.96815259 | 0.05760328 | 118.6828603 | 211.1030912 |
| YEL020C | YEL020C | 0.75808628 | 0.97135148 | 0.05768138 | 117.7467456 | 209.3426627 |
| YLR039C | RIC1 | 0.63015396 | 0.96815259 | 0.057714 | 102.6248104 | 181.1248919 |
| YLR104W | YLR104W | 0.64248459 | 0.96815259 | 0.05771597 | 104.9044461 | 185.3774951 |
| YNL030W | HHF2 | 0.32305514 | 0.96815259 | 0.05773454 | 98.44870742 | 173.3301531 |
| YLR228C | ECM22 | 0.55009726 | 0.96815259 | 0.05779514 | 101.5168971 | 179.0434075 |
| YMR139W | RIM11 | 0.3429414 | 0.96815259 | 0.05781246 | 88.98307017 | 155.6568282 |
| YPR062W | FCY1 | 0.81141298 | 0.9762419 | 0.05788273 | 108.8950243 | 192.7925736 |
| YDR184C | ATC1 | 0.64264494 | 0.96815259 | 0.05799485 | 116.7606081 | 207.4467618 |
| YLR011W | LOT6 | 0.6827608 | 0.97060928 | 0.05801247 | 121.5477009 | 216.3745484 |
| YLR206W | ENT2 | 0.7765878 | 0.97246744 | 0.05805705 | 108.5032731 | 192.0304958 |
| YPR152C | URN1 | 0.6482617 | 0.96815259 | 0.05807443 | 112.2675616 | 199.0501516 |
| YPL260W | YPL260W | 0.58892398 | 0.96815259 | 0.05809243 | 119.49235 | 212.5257064 |
| YOR017W | PET127 | 0.79130662 | 0.97320687 | 0.05839433 | 127.0241847 | 226.5232571 |
| YGL250W | YGL250W | 0.71730251 | 0.97135148 | 0.05842253 | 124.1630844 | 221.1804535 |
| YFL032W | YFL032W | 0.78874088 | 0.97272554 | 0.05846973 | 126.2865284 | 225.133561 |
| YDR278C | YDR278C | 0.64983969 | 0.96815259 | 0.05847706 | 123.0934095 | 219.1750727 |
| YJL142C | YJL142C | 0.73557057 | 0.97135148 | 0.05851922 | 122.4521851 | 217.9712354 |
| YGL165C | YGL165C | 0.71825791 | 0.97135148 | 0.05852871 | 112.3528812 | 199.1279758 |

| | | | | | | |
|---|---|---|---|---|---|---|
| YLL060C | GTT2 | 0.63597314 | 0.96815259 | 0.05853085 | 118.7445976 | 211.0521682 |
| YBR209W | YBR209W | 0.58236101 | 0.96815259 | 0.05854536 | 113.2754262 | 200.8461217 |
| YCR027C | RHB1 | 0.72998633 | 0.97135148 | 0.05865431 | 117.6847507 | 209.0527776 |
| YKR088C | TVP38 | 0.37795195 | 0.96815259 | 0.05866063 | 115.0007471 | 204.0442897 |
| YNR060W | FRE4 | 0.71892312 | 0.97135148 | 0.05892256 | 130.4165604 | 232.7575821 |
| YPR197C | YPR197C | 0.75326259 | 0.97135148 | 0.05896583 | 118.3558563 | 210.2490288 |
| YMR031C | YMR031C | 0.67546567 | 0.97060928 | 0.05902844 | 120.8196028 | 214.834254 |
| YCR025C | YCR025C | 0.76157415 | 0.97135148 | 0.05917234 | 118.6946315 | 210.8440759 |
| YGR003W | CUL3 | 0.74373252 | 0.97135148 | 0.05921567 | 130.1633819 | 232.2327563 |
| YLR219W | MSC3 | 0.61256146 | 0.96815259 | 0.05957818 | 108.8972239 | 192.4930693 |
| YLR381W | CTF3 | 0.70697145 | 0.97135148 | 0.05977214 | 115.2708911 | 204.3492376 |
| YOR109W | INP53 | 0.76301089 | 0.97135148 | 0.05982378 | 124.4107796 | 221.3916372 |
| YDL124W | YDL124W | 0.72464468 | 0.97135148 | 0.05983206 | 127.9458974 | 227.985373 |
| YOR190W | SPR1 | 0.70600828 | 0.97135148 | 0.05986555 | 122.7719858 | 218.326774 |
| YBR009C | HHF1 | 0.65754732 | 0.96815259 | 0.05994324 | 110.2406189 | 194.9339753 |
| YBR295W | PCA1 | 0.77572552 | 0.97246744 | 0.06010751 | 115.52788 | 204.7686295 |
| YGL217C | YGL217C | 0.31117222 | 0.96815259 | 0.06018224 | 111.8054563 | 197.8105822 |
| YJL059W | YHC3 | 0.72867214 | 0.97135148 | 0.06023668 | 129.8038556 | 231.379179 |
| YLR142W | PUT1 | 0.69289855 | 0.97135148 | 0.06027421 | 124.5571901 | 221.5841243 |
| YLR041W | YLR041W | 0.70585145 | 0.97135148 | 0.06029557 | 119.3518884 | 211.8691354 |
| YLR114C | AVL9 | 0.35960124 | 0.96815259 | 0.06043028 | 101.2274843 | 178.0315899 |
| YKL166C | TPK3 | 0.75069191 | 0.97135148 | 0.06061366 | 109.1416601 | 192.7636716 |
| YJR150C | DAN1 | 0.55638417 | 0.96815259 | 0.06063485 | 100.2846432 | 176.2359636 |
| YER155C | BEM2 | 0.7223242 | 0.97135148 | 0.06071763 | 120.8684401 | 214.6228789 |
| YLR341W | SPO77 | 0.69294348 | 0.97135148 | 0.0608655 | 117.5661225 | 208.4354982 |
| YDL012C | YDL012C | 0.37653659 | 0.96815259 | 0.060908 | 134.022676 | 239.1297208 |
| YNL203C | YNL203C | 0.76285803 | 0.97135148 | 0.06106829 | 124.3937595 | 221.1370252 |
| YOR112W | YOR112W | 0.79627493 | 0.97570065 | 0.06145611 | 122.9774693 | 218.4253061 |
| YMR291W | YMR291W | 0.81260552 | 0.97633618 | 0.06145776 | 127.963357 | 227.7268286 |
| YML107C | PML39 | 0.40169276 | 0.96815259 | 0.06148826 | 100.6385423 | 176.7433866 |
| YBL078C | ATG8 | 0.60245576 | 0.96815259 | 0.06158587 | 121.3942841 | 215.4484324 |
| YJR082C | EAF6 | 0.49892649 | 0.96815259 | 0.06186927 | 108.0895826 | 190.5760388 |
| YGR232W | NAS6 | 0.75696082 | 0.97135148 | 0.06188602 | 127.644777 | 227.0557878 |
| YLR123C | YLR123C | 0.51403527 | 0.96815259 | 0.06203124 | 118.7879229 | 210.5061734 |
| YOL158C | ENB1 | 0.65570087 | 0.96815259 | 0.06220901 | 122.5392949 | 217.4730107 |
| YIL027C | KRE27 | 0.745818 | 0.97135148 | 0.06225548 | 132.8486638 | 236.6981514 |
| YDL192W | ARF1 | 0.62947155 | 0.96815259 | 0.06225649 | 96.56168529 | 168.9999137 |
| YLR312C | YLR312C | 0.69386605 | 0.97135148 | 0.06244168 | 119.9359556 | 212.5744798 |
| YAL024C | LTE1 | 0.7297069 | 0.97135148 | 0.06247756 | 108.8597894 | 191.9040307 |
| YLR269C | YLR269C | 0.51879622 | 0.96815259 | 0.06256981 | 104.2684871 | 183.3218434 |
| YNL154C | YCK2 | 0.5456902 | 0.96815259 | 0.06264378 | 93.8207197 | 163.8169344 |
| YPL156C | PRM4 | 0.38904814 | 0.96815259 | 0.06277909 | 112.1214 | 197.9349926 |
| YKL129C | MYO3 | 0.63184962 | 0.96815259 | 0.06287584 | 110.4277166 | 194.7578818 |
| YMR234W | RNH1 | 0.74366748 | 0.97135148 | 0.06293818 | 113.2277149 | 199.9704784 |
| YDR219C | MFB1 | 0.55438699 | 0.96815259 | 0.06322962 | 115.3328262 | 203.8456472 |
| YNR071C | YNR071C | 0.78244977 | 0.97260548 | 0.06334753 | 131.3969623 | 233.794259 |
| YNL125C | ESBP6 | 0.33722755 | 0.96815259 | 0.06335972 | 126.6109165 | 224.8630872 |
| YBR052C | RFS1 | 0.67846946 | 0.97060928 | 0.06355617 | 132.0926729 | 235.054835 |
| YMR158W-A | YMR158W-A | 0.01999539 | 0.78054746 | 0.06367208 | 96.73723931 | 169.0739394 |
| YLR442C | SIR3 | 0.72777252 | 0.97135148 | 0.06376872 | 134.0550989 | 238.6779327 |
| YJR058C | APS2 | 0.73907479 | 0.97135148 | 0.06380068 | 117.8791075 | 208.4938031 |
| YLR020C | YEH2 | 0.72503718 | 0.97135148 | 0.06387548 | 116.3571612 | 205.6410216 |
| YNL022C | YNL022C | 0.55469749 | 0.96815259 | 0.0639811 | 131.658676 | 234.1690641 |
| YIL141W | YIL141W | 0.72487312 | 0.97135148 | 0.0641691 | 128.2419436 | 227.7610423 |
| YOR054C | VHS3 | 0.79005628 | 0.97272554 | 0.06429389 | 101.1465921 | 177.1888085 |
| YIL162W | SUC2 | 0.73122573 | 0.97135148 | 0.06467145 | 126.9844252 | 225.3250223 |
| YGR032W | GSC2 | 0.63564755 | 0.96815259 | 0.06482471 | 117.5941182 | 207.7787441 |
| YNL294C | RIM21 | 0.76406201 | 0.97135148 | 0.06483054 | 126.3204964 | 224.0578875 |
| YER150W | SPI1 | 0.68292209 | 0.97060928 | 0.06496531 | 110.6036836 | 194.7120047 |
| YBL055C | YBL055C | 0.62471654 | 0.96815259 | 0.06503059 | 130.3268758 | 231.4964841 |
| YKR019C | IRS4 | 0.69796103 | 0.97135148 | 0.06524044 | 109.7889949 | 193.14283 |
| YMR176W | ECM5 | 0.32781031 | 0.96815259 | 0.06528206 | 119.9361551 | 212.0662179 |
| YPR158W | YPR158W | 0.67292441 | 0.97060928 | 0.06535719 | 122.6341839 | 217.086287 |
| YLR387C | REH1 | 0.32727683 | 0.96815259 | 0.06537228 | 117.1461676 | 206.8449782 |

| | | | | | | |
|---|---|---|---|---|---|---|
| YHR181W | SVP26 | 0.66716367 | 0.9703088 | 0.06546401 | 125.0954973 | 221.6590571 |
| YOL089C | HAL9 | 0.67271241 | 0.97060928 | 0.06569624 | 109.4638454 | 192.4546003 |
| YMR259C | YMR259C | 0.75934053 | 0.97135148 | 0.06570472 | 127.1874652 | 225.5187898 |
| YBR042C | YBR042C | 0.68690767 | 0.97133463 | 0.06579591 | 122.0324255 | 215.8850655 |
| YGR281W | YOR1 | 0.67402878 | 0.97060928 | 0.0658034 | 117.9357081 | 208.2407673 |
| YER121W | YER121W | 0.7255267 | 0.97135148 | 0.06583637 | 124.5172756 | 220.5136299 |
| YJL067W | YJL067W | 0.66084193 | 0.96815259 | 0.06597449 | 125.2932942 | 221.9366598 |
| YGL173C | KEM1 | 0.75618705 | 0.97135148 | 0.06600865 | 47.87317301 | 77.4932799 |
| YMR106C | YKU80 | 0.73943923 | 0.97135148 | 0.06607922 | 123.0041422 | 217.6471959 |
| YKR097W | PCK1 | 0.5422015 | 0.96815259 | 0.06612107 | 94.87373302 | 165.1587807 |
| YPL108W | YPL108W | 0.33573403 | 0.96815259 | 0.0664365 | 116.3343911 | 205.139933 |
| YIL058W | YIL058W | 0.57675293 | 0.96815259 | 0.06648487 | 112.9412801 | 198.8009826 |
| YDR539W | YDR539W | 0.71162076 | 0.97135148 | 0.06654881 | 128.5089344 | 227.8330083 |
| YGL082W | YGL082W | 0.80216681 | 0.9762419 | 0.0665578 | 132.1365566 | 234.5991965 |
| YJR054W | YJR054W | 0.77509008 | 0.97246744 | 0.0665675 | 139.9113095 | 249.1022695 |
| YLR102C | APC9 | 0.66070065 | 0.96815259 | 0.06658679 | 129.2921596 | 229.2874149 |
| YJL024C | APS3 | 0.71960042 | 0.97135148 | 0.06678408 | 109.6149153 | 192.5416379 |
| YCR087C-A | LUG1 | 0.75054257 | 0.97135148 | 0.06690387 | 113.7245782 | 200.1872949 |
| YGR017W | YGR017W | 0.69259046 | 0.97135148 | 0.0669517 | 122.8233883 | 217.1537395 |
| YNL136W | EAF7 | 0.81641482 | 0.97741538 | 0.06702049 | 89.21411302 | 154.438964 |
| YLR023C | IZH3 | 0.70822381 | 0.97135148 | 0.06712079 | 117.4691927 | 207.1345137 |
| YLL024C | SSA2 | 0.59354636 | 0.96815259 | 0.06723963 | 121.8738843 | 215.3307566 |
| YLR407W | YLR407W | 0.51456581 | 0.96815259 | 0.0672922 | 99.94574068 | 174.4115509 |
| YFR024C | YFR024C | 0.88181464 | 0.98248371 | 0.06734582 | 90.50145215 | 156.7824054 |
| YOL016C | CMK2 | 0.73959693 | 0.97135148 | 0.06741032 | 127.8362334 | 226.4237253 |
| YOR108W | LEU9 | 0.75356447 | 0.97135148 | 0.06744461 | 123.7403954 | 218.7762683 |
| YLR205C | HMX1 | 0.65673654 | 0.96815259 | 0.06749406 | 118.4074424 | 208.8180977 |
| YJL183W | MNN11 | 0.64432225 | 0.96815259 | 0.06755557 | 114.2735845 | 201.0948369 |
| YHL010C | YHL010C | 0.62335379 | 0.96815259 | 0.06761401 | 125.5724855 | 222.1639352 |
| YMR226C | TMA29 | 0.20703165 | 0.96815259 | 0.06762779 | 102.3787021 | 178.8904601 |
| YIL041W | GVP36 | 0.7451735 | 0.97135148 | 0.06776206 | 122.3231489 | 216.0753647 |
| YOR314W | YOR314W | 0.63388877 | 0.96815259 | 0.06777936 | 119.431704 | 210.6779011 |
| YLL055W | YLL055W | 0.70427127 | 0.97135148 | 0.06780499 | 117.4440225 | 206.9650355 |
| YMR136W | GAT2 | 0.15416524 | 0.96815259 | 0.06786553 | 108.7682177 | 190.7683555 |
| YOL028C | YAP7 | 0.74213101 | 0.97135148 | 0.06790734 | 130.6232318 | 231.5342277 |
| YHL017W | YHL017W | 0.75199562 | 0.97135148 | 0.06792359 | 127.8691418 | 226.3932068 |
| YPL195W | APL5 | 0.73026241 | 0.97135148 | 0.0680592 | 114.8785259 | 202.1332464 |
| YNL285W | YNL285W | 0.74055755 | 0.97135148 | 0.06810487 | 133.4889272 | 236.8451829 |
| YPL125W | KAP120 | 0.71505814 | 0.97135148 | 0.06811609 | 113.4971725 | 199.5459655 |
| YMR080C | NAM7 | 0.67907885 | 0.97060928 | 0.06828253 | 110.1865899 | 193.3398397 |
| YOR041C | SRF5 | 0.6960324 | 0.97135148 | 0.06829797 | 122.6525866 | 216.5940068 |
| YPL178W | CBC2 | 0.8426615 | 0.98035418 | 0.0684742 | 25.38505019 | 35.09726312 |
| YCR036W | RBK1 | 0.70991277 | 0.97135148 | 0.06862718 | 112.1064043 | 196.8597853 |
| YJL206C-A | YJL206C-A | 0.54328598 | 0.96815259 | 0.06863072 | 129.7270382 | 229.7327253 |
| YOR008C | SLG1 | 0.27286352 | 0.96815259 | 0.06866998 | 86.99048923 | 149.9951287 |
| YPL206C | YPL206C | 0.63290034 | 0.96815259 | 0.06868069 | 119.7137772 | 211.0427422 |
| YMR300C | ADE4 | 0.75828289 | 0.97135148 | 0.06879251 | 126.0950977 | 222.9278978 |
| YMR192W | GYL1 | 0.70699691 | 0.97135148 | 0.06886338 | 127.2009041 | 224.9782318 |
| YLR335W | NUP2 | 0.53596681 | 0.96815259 | 0.06919351 | 110.5351409 | 193.8269767 |
| YGL079W | YGL079W | 0.49645933 | 0.96815259 | 0.06924772 | 124.8408089 | 220.5063443 |
| YLR214W | FRE1 | 0.40469837 | 0.96815259 | 0.06938345 | 96.69598633 | 167.974227 |
| YCL025C | AGP1 | 0.52384893 | 0.96815259 | 0.06941767 | 129.7840123 | 229.698097 |
| YER120W | SCS2 | 0.60705597 | 0.96815259 | 0.06948672 | 106.86342 | 186.9243991 |
| YPL191C | YPL191C | 0.72849002 | 0.97135148 | 0.06982156 | 119.9126632 | 211.2094923 |
| YAL066W | YAL066W | 0.58145519 | 0.96815259 | 0.06988331 | 126.9509338 | 224.3292391 |
| YLR231C | BNA5 | 0.68646134 | 0.97133463 | 0.06996975 | 133.461597 | 236.4602452 |
| YLR065C | YLR065C | 0.61926587 | 0.96815259 | 0.07000579 | 93.33397687 | 161.5905196 |
| YLR225C | YLR225C | 0.71130335 | 0.97135148 | 0.07004005 | 120.5191635 | 212.3018711 |
| YOL159C | YOL159C | 0.71107881 | 0.97135148 | 0.0700779 | 118.3480586 | 208.2446159 |
| YGR178C | PBP1 | 0.50615492 | 0.96815259 | 0.07021511 | 116.2846139 | 204.3704213 |
| YPR076W | YPR076W | 0.5366398 | 0.96815259 | 0.07056774 | 119.3711775 | 210.0656595 |
| YCL001W-A | YCL001W-A | 0.66320082 | 0.96927465 | 0.07065729 | 117.4801258 | 206.5216218 |
| YKL207W | YKL207W | 0.7227072 | 0.97135148 | 0.07084716 | 116.7249663 | 205.0787734 |
| YDR124W | YDR124W | 0.51406182 | 0.96815259 | 0.07086236 | 128.8913989 | 227.774108 |

| | | | | | | |
|---|---|---|---|---|---|---|
| YBR053C | YBR053C | 0.59275759 | 0.96815259 | 0.07086862 | 125.6819431 | 221.7853309 |
| YMR119W-A | YMR119W-A | 0.74081463 | 0.97135148 | 0.07089357 | 122.9572391 | 216.697575 |
| YDR257C | SET7 | 0.68524631 | 0.97133463 | 0.07094484 | 126.0130632 | 222.3894303 |
| YOR138C | RUP1 | 0.60122341 | 0.96815259 | 0.07098466 | 131.7338287 | 233.0551285 |
| YBR122C | MRPL36 | 0.83745249 | 0.98035418 | 0.07099588 | 30.56961867 | 44.31818219 |
| YER061C | CEM1 | 0.55362072 | 0.96815259 | 0.0714994 | 84.09256687 | 144.0820086 |
| YLR237W | THI7 | 0.59304863 | 0.96815259 | 0.07171341 | 129.817302 | 229.3490994 |
| YPL155C | KIP2 | 0.5336154 | 0.96815259 | 0.07172718 | 131.5881267 | 232.6503363 |
| YNR065C | YSN1 | 0.73925418 | 0.97135148 | 0.07178442 | 125.2282681 | 220.7749473 |
| YIL055C | YIL055C | 0.71525252 | 0.97135148 | 0.07189016 | 126.939151 | 223.9478863 |
| YOL020W | TAT2 | 0.60521647 | 0.96815259 | 0.07207083 | 115.6547755 | 202.8630695 |
| YNR047W | YNR047W | 0.42705831 | 0.96815259 | 0.07212603 | 103.3280521 | 179.8560849 |
| YHL043W | ECM34 | 0.69752045 | 0.97135148 | 0.07217143 | 125.7696753 | 221.7157103 |
| YOL045W | PSK2 | 0.5083287 | 0.96815259 | 0.07217475 | 116.8323287 | 205.0413371 |
| YNL168C | FMP41 | 0.63156522 | 0.96815259 | 0.07232685 | 124.2181887 | 218.793379 |
| YLR250W | SSP120 | 0.64057602 | 0.96815259 | 0.07247859 | 125.8609073 | 221.83091 |
| YLR149C | YLR149C | 0.68710232 | 0.97133463 | 0.07249418 | 114.3486324 | 200.3504799 |
| YMR187C | YMR187C | 0.63627733 | 0.96815259 | 0.07251033 | 104.5414065 | 182.0509364 |
| YHR137W | ARO9 | 0.73562703 | 0.97135148 | 0.07252792 | 123.2265185 | 216.9072836 |
| YMR129W | POM152 | 0.5847486 | 0.96815259 | 0.07276643 | 112.1723208 | 196.2415349 |
| YDR480W | DIG2 | 0.50393156 | 0.96815259 | 0.07280057 | 115.585776 | 202.6036658 |
| YMR067C | UBX4 | 0.17765447 | 0.96815259 | 0.07282253 | 94.54164296 | 163.339177 |
| YDR511W | ACN9 | 0.51484014 | 0.96815259 | 0.07293016 | 104.2669078 | 181.4636444 |
| YIL094C | LYS12 | 0.70962975 | 0.97135148 | 0.07293699 | 103.2849167 | 179.6303897 |
| YPL200W | CSM4 | 0.68087136 | 0.97060928 | 0.07294714 | 118.9700444 | 208.8912088 |
| YJL068C | YJL068C | 0.76346537 | 0.97135148 | 0.07301614 | 136.0791782 | 240.7981582 |
| YMR250W | GAD1 | 0.71739463 | 0.97135148 | 0.0730912 | 130.3816652 | 230.1552688 |
| YOL041C | NOP12 | 0.58229933 | 0.96815259 | 0.07314968 | 85.6039239 | 146.6061204 |
| YPR014C | YPR014C | 0.47875401 | 0.96815259 | 0.07325049 | 120.5452292 | 211.7755991 |
| YNL281W | HCH1 | 0.2232907 | 0.96815259 | 0.07328898 | 107.4849954 | 187.4031487 |
| YIL130W | ASG1 | 0.67219412 | 0.97060928 | 0.07331715 | 125.9976964 | 221.935945 |
| YOR030W | DFG16 | 0.70264823 | 0.97135148 | 0.07333582 | 132.0725461 | 233.266022 |
| YPL202C | AFT2 | 0.60868583 | 0.96815259 | 0.07336984 | 127.080917 | 223.9473987 |
| YBR293W | VBA2 | 0.61318701 | 0.96815259 | 0.07353611 | 134.2873436 | 237.3621471 |
| YLL019C | KNS1 | 0.39995051 | 0.96815259 | 0.07371603 | 112.2491811 | 196.2148804 |
| YLR307W | CDA1 | 0.61347094 | 0.96815259 | 0.07374828 | 125.1431886 | 220.2645468 |
| YNL270C | ALP1 | 0.62130067 | 0.96815259 | 0.07375873 | 132.1990533 | 233.4263049 |
| YPL014W | YPL014W | 0.70107513 | 0.97135148 | 0.0737643 | 121.2201306 | 212.9427064 |
| YHL006C | SHU1 | 0.68298567 | 0.97060928 | 0.07393376 | 120.8768413 | 212.2719086 |
| YPR060C | ARO7 | 0.29761159 | 0.96815259 | 0.07400494 | 76.84619816 | 130.1142964 |
| YOR270C | VPH1 | 0.57274025 | 0.96815259 | 0.07404697 | 100.8642764 | 174.9156045 |
| YDR360W | YDR360W | 0.68073041 | 0.97060928 | 0.07407665 | 118.8044051 | 208.3799232 |
| YPR126C | YPR126C | 0.74636846 | 0.97135148 | 0.07415965 | 131.6161866 | 232.2670981 |
| YOR127W | RGA1 | 0.70076454 | 0.97135148 | 0.07446544 | 124.9240158 | 219.727229 |
| YDR508C | GNP1 | 0.64166833 | 0.96815259 | 0.07462663 | 140.2279347 | 248.2498059 |
| YBR016W | YBR016W | 0.47314451 | 0.96815259 | 0.07473027 | 124.7430561 | 219.3422005 |
| YLL059C | YLL059C | 0.69177532 | 0.97135148 | 0.07492 | 109.2049756 | 190.3199246 |
| YGL125W | MET13 | 0.15627205 | 0.96815259 | 0.07495753 | 111.0475219 | 193.7507118 |
| YFL003C | MSH4 | 0.7252588 | 0.97135148 | 0.07498538 | 114.5485517 | 200.2773502 |
| YBR030W | YBR030W | 0.51855643 | 0.96815259 | 0.07502143 | 132.3604295 | 233.501259 |
| YER161C | SPT2 | 0.65549176 | 0.96815259 | 0.07502372 | 121.4497377 | 213.1455407 |
| YLL028W | TPO1 | 0.38556359 | 0.96815259 | 0.07509977 | 114.2162782 | 199.6369653 |
| YLR058C | SHM2 | 0.62522454 | 0.96815259 | 0.07514409 | 122.9601741 | 215.9418993 |
| YDR185C | YDR185C | 0.58102025 | 0.96815259 | 0.0751634 | 135.9738711 | 240.2171787 |
| YJL172W | CPS1 | 0.15240528 | 0.96815259 | 0.07519018 | 100.4050151 | 173.8540769 |
| YBR183W | YPC1 | 0.51949236 | 0.96815259 | 0.07525376 | 128.3573813 | 225.99145 |
| YOL024W | YOL024W | 0.6598725 | 0.96815259 | 0.07539131 | 125.1530958 | 219.9888093 |
| YJL073W | JEM1 | 0.50691772 | 0.96815259 | 0.07564058 | 119.7239695 | 209.8154329 |
| YHR080C | YHR080C | 0.71749976 | 0.97135148 | 0.07571005 | 124.5942026 | 218.8890442 |
| YKL201C | MNN4 | 0.4005101 | 0.96815259 | 0.07576212 | 115.9036731 | 202.6664107 |
| YGR166W | KRE11 | 0.75593334 | 0.97135148 | 0.07594388 | 127.8535289 | 224.9278673 |
| YGR176W | YGR176W | 0.72556977 | 0.97135148 | 0.07615767 | 118.5416017 | 207.516977 |
| YBL048W | YBL048W | 0.55697358 | 0.96815259 | 0.07619826 | 138.3726365 | 244.5070713 |
| YLR422W | YLR422W | 0.68309195 | 0.97060928 | 0.07644487 | 114.6609616 | 200.225711 |

| | | | | | | |
|---|---|---|---|---|---|---|
| YPL227C | ALG5 | 0.61098355 | 0.96815259 | 0.07650683 | 138.8496166 | 245.341684 |
| YMR326C | YMR326C | 0.71441993 | 0.97135148 | 0.07661111 | 118.7838528 | 207.8877292 |
| YGR205W | YGR205W | 0.69869603 | 0.97135148 | 0.07664293 | 126.879617 | 222.9857282 |
| YIL107C | PFK26 | 0.69926993 | 0.97135148 | 0.07667528 | 123.3821915 | 216.4550337 |
| YNL027W | CRZ1 | 0.60992778 | 0.96815259 | 0.0767004 | 130.194046 | 229.1589322 |
| YOR091W | TMA46 | 0.69808771 | 0.97135148 | 0.07671681 | 120.8572797 | 211.7370482 |
| YOR097C | YOR097C | 0.74722318 | 0.97135148 | 0.07672439 | 124.9946664 | 219.4545212 |
| YIL096C | YIL096C | 0.35850401 | 0.96815259 | 0.07691957 | 124.3250689 | 218.1703485 |
| YKL158W | YKL158W | 0.57086227 | 0.96815259 | 0.07703348 | 121.6820417 | 213.2190412 |
| YBL028C | YBL028C | 0.33656861 | 0.96815259 | 0.0770579 | 125.8925259 | 221.0698707 |
| YDL070W | BDF2 | 0.44937682 | 0.96815259 | 0.07734252 | 109.7524133 | 190.9074342 |
| YLR084C | RAX2 | 0.65767091 | 0.96815259 | 0.07737531 | 135.1518119 | 238.2874301 |
| YPR128C | ANT1 | 0.6260222 | 0.96815259 | 0.0776721 | 110.9341304 | 193.0530607 |
| YGL227W | VID30 | 0.67862814 | 0.97060928 | 0.07786011 | 140.6583405 | 248.4737567 |
| YLR120C | YPS1 | 0.59443967 | 0.96815259 | 0.07815928 | 116.7241819 | 203.7679116 |
| YLL046C | RNP1 | 0.20609437 | 0.96815259 | 0.07846586 | 105.3484528 | 182.490115 |
| YKR029C | SET3 | 0.32611049 | 0.96815259 | 0.07855842 | 103.6264421 | 179.2609067 |
| YPR063C | YPR063C | 0.73623263 | 0.97135148 | 0.07862541 | 123.7161771 | 216.7289134 |
| YOR072W | YOR072W | 0.57386899 | 0.96815259 | 0.07873155 | 125.8407045 | 220.6734882 |
| YHR111W | UBA4 | 0.43665461 | 0.96815259 | 0.07873693 | 105.5975741 | 182.9063424 |
| YLR037C | DAN2 | 0.47649277 | 0.96815259 | 0.07891234 | 115.7515376 | 201.8184656 |
| YDL130W-A | STF1 | 0.46232385 | 0.96815259 | 0.07906191 | 123.7226193 | 216.6627666 |
| YDL136W | RPL35B | 0.68261597 | 0.97060928 | 0.07912195 | 111.9782815 | 194.741433 |
| YHR073W | OSH3 | 0.73592235 | 0.97135148 | 0.0791522 | 128.0999588 | 224.8130929 |
| YBR085W | AAC3 | 0.31567104 | 0.96815259 | 0.07928037 | 112.1736898 | 195.0776226 |
| YNL047C | SLM2 | 0.75295705 | 0.97135148 | 0.07932884 | 117.7057739 | 205.3897633 |
| YNR072W | HXT17 | 0.65985773 | 0.96815259 | 0.07961231 | 122.7468881 | 214.7438526 |
| YIL146C | ECM37 | 0.63837838 | 0.96815259 | 0.07962544 | 120.5275499 | 210.6010383 |
| YGL144C | ROG1 | 0.63179162 | 0.96815259 | 0.0798203 | 149.1892053 | 264.0381747 |
| YPR146C | YPR146C | 0.69123781 | 0.97135148 | 0.07992298 | 126.1555709 | 221.047559 |
| YOR089C | VPS21 | 0.6507564 | 0.96815259 | 0.07995823 | 106.6900194 | 184.7257402 |
| YPL249C | GYP5 | 0.56320223 | 0.96815259 | 0.08035898 | 128.9917746 | 226.2607892 |
| YBR051W | YBR051W | 0.37940282 | 0.96815259 | 0.08057908 | 128.5227834 | 225.3464112 |
| YOR019W | YOR019W | 0.71056846 | 0.97135148 | 0.08059761 | 134.8299295 | 237.109891 |
| YJR014W | TMA22 | 0.61024817 | 0.96815259 | 0.08064137 | 126.5829184 | 221.7161885 |
| YHR030C | SLT2 | 0.70032726 | 0.97135148 | 0.08079092 | 124.7171944 | 218.2086575 |
| YGR173W | RBG2 | 0.59348756 | 0.96815259 | 0.08079881 | 131.7149773 | 231.2625155 |
| YOL087C | YOL087C | 0.73118678 | 0.97135148 | 0.08102785 | 122.6956944 | 214.3948598 |
| YPL185W | YPL185W | 0.60064266 | 0.96815259 | 0.08107957 | 125.8031926 | 220.1830392 |
| YLR096W | KIN2 | 0.67363503 | 0.97060928 | 0.08115963 | 123.2333333 | 215.3742963 |
| YPL025C | YPL025C | 0.73575203 | 0.97135148 | 0.08123989 | 133.6495772 | 234.7927767 |
| YKL175W | ZRT3 | 0.15441718 | 0.96815259 | 0.08175501 | 120.8722858 | 210.8628407 |
| YMR007W | YMR007W | 0.64566376 | 0.96815259 | 0.08175986 | 114.3272463 | 198.6513531 |
| YAL018C | YAL018C | 0.41897496 | 0.96815259 | 0.08182308 | 125.8738548 | 220.1817257 |
| YPR122W | AXL1 | 0.60326388 | 0.96815259 | 0.08186126 | 112.1819216 | 194.6308146 |
| YOL117W | RRI2 | 0.7194404 | 0.97135148 | 0.08190815 | 119.6103865 | 208.4811816 |
| YIL092W | YIL092W | 0.64952665 | 0.96815259 | 0.08210205 | 125.6916148 | 219.7917788 |
| YNL054W | VAC7 | 0.63170123 | 0.96815259 | 0.08214857 | 133.6042858 | 234.54556 |
| YLR313C | SPH1 | 0.58633421 | 0.96815259 | 0.082244 | 120.1651925 | 209.4561018 |
| YFR006W | YFR006W | 0.66466501 | 0.96985526 | 0.08226137 | 135.6245441 | 238.2944134 |
| YAL026C | DRS2 | 0.58473667 | 0.96815259 | 0.08229189 | 103.6366606 | 178.6114093 |
| YOR375C | GDH1 | 0.54245663 | 0.96815259 | 0.08230905 | 126.8182408 | 221.856577 |
| YMR118C | YMR118C | 0.44088318 | 0.96815259 | 0.08237383 | 118.1713124 | 205.7130123 |
| YHR079C-B | YHR079C-B | 0.40299382 | 0.96815259 | 0.08241753 | 124.4612729 | 217.4399219 |
| YBR226C | YBR226C | 0.72646871 | 0.97135148 | 0.08247704 | 130.967036 | 229.5666085 |
| YKL137W | YKL137W | 0.32878278 | 0.96815259 | 0.08249936 | 101.9641233 | 175.4539225 |
| YMR279C | YMR279C | 0.62584716 | 0.96815259 | 0.08288826 | 121.5522219 | 211.9284159 |
| YOR324C | FRT1 | 0.65542111 | 0.96815259 | 0.08323502 | 131.1389048 | 229.7515198 |
| YHL016C | DUR3 | 0.58945171 | 0.96815259 | 0.08324296 | 120.3345948 | 209.5932571 |
| YOR248W | YOR248W | 0.45239209 | 0.96815259 | 0.08329394 | 118.8596745 | 206.8324733 |
| YFL050C | ALR2 | 0.51299601 | 0.96815259 | 0.083407 | 115.6792139 | 200.8786667 |
| YML081C-A | ATP18 | 0.24597708 | 0.96815259 | 0.08359946 | 91.62613258 | 155.9700641 |
| YPL154C | PEP4 | 0.33861763 | 0.96815259 | 0.08388624 | 121.532289 | 211.712518 |
| YMR238W | DFG5 | 0.68170165 | 0.97060928 | 0.0840693 | 124.3582761 | 216.951981 |

| | | | | | | |
|---|---|---|---|---|---|---|
| YLR057W | YLR057W | 0.28214728 | 0.96815259 | 0.08417169 | 120.4974255 | 209.73073 |
| YHR109W | CTM1 | 0.69864323 | 0.97135148 | 0.08419511 | 130.0521385 | 227.5520899 |
| YPL134C | ODC1 | 0.50536199 | 0.96815259 | 0.08424292 | 121.9078902 | 212.3493793 |
| YPR148C | YPR148C | 0.60562015 | 0.96815259 | 0.08425396 | 123.5795393 | 215.4660801 |
| YKL073W | LHS1 | 0.60557094 | 0.96815259 | 0.08437585 | 113.3914758 | 196.4371005 |
| YPL216W | YPL216W | 0.59352334 | 0.96815259 | 0.08446531 | 139.0243713 | 244.2425679 |
| YNR028W | CPR8 | 0.54604117 | 0.96815259 | 0.08455724 | 121.9747447 | 212.4178192 |
| YNL011C | YNL011C | 0.0934734 | 0.96815259 | 0.08460403 | 118.9050464 | 206.6825207 |
| YOR385W | YOR385W | 0.53189561 | 0.96815259 | 0.08460996 | 133.8521708 | 234.567255 |
| YGL243W | TAD1 | 0.63186514 | 0.96815259 | 0.0846228 | 130.6295022 | 228.5526502 |
| YOR104W | PIN2 | 0.5099586 | 0.96815259 | 0.08472923 | 122.0131855 | 212.4587372 |
| YDR174W | HMO1 | 0.43012898 | 0.96815259 | 0.08496646 | 73.70826304 | 122.2971656 |
| YER067W | YER067W | 0.56312711 | 0.96815259 | 0.08561254 | 124.2741563 | 216.5186933 |
| YGR149W | YGR149W | 0.5261419 | 0.96815259 | 0.08564362 | 123.8191228 | 215.6642044 |
| YLR049C | YLR049C | 0.50714802 | 0.96815259 | 0.08574555 | 107.9454537 | 186.0315665 |
| YLR315W | NKP2 | 0.28224331 | 0.96815259 | 0.08605579 | 110.4998788 | 190.7416219 |
| YIR019C | MUC1 | 0.64889 | 0.96815259 | 0.08620552 | 123.8890255 | 215.6939962 |
| YPL001W | HAT1 | 0.23700695 | 0.96815259 | 0.08627238 | 114.1717811 | 197.5532446 |
| YAL067C | SEO1 | 0.53745197 | 0.96815259 | 0.08630532 | 127.4614706 | 222.340984 |
| YLR254C | NDL1 | 0.57771496 | 0.96815259 | 0.08630548 | 119.38539 | 207.2739802 |
| YML059C | NTE1 | 0.17100036 | 0.96815259 | 0.08645036 | 117.278864 | 203.3180394 |
| YHL039W | YHL039W | 0.68771992 | 0.97133463 | 0.08663249 | 128.3798091 | 223.9956754 |
| YOR050C | YOR050C | 0.62646731 | 0.96815259 | 0.08688032 | 134.7120272 | 235.7648699 |
| YDR073W | SNF11 | 0.69034195 | 0.97135148 | 0.08703506 | 151.0056966 | 266.135111 |
| YHR185C | PFS1 | 0.51381282 | 0.96815259 | 0.08715502 | 125.9080286 | 219.2906792 |
| YOR070C | GYP1 | 0.45026684 | 0.96815259 | 0.08715838 | 111.8339943 | 193.0331436 |
| YLR004C | THI73 | 0.44905267 | 0.96815259 | 0.0872277 | 110.5930774 | 190.7056395 |
| YEL011W | GLC3 | 0.56841408 | 0.96815259 | 0.08727432 | 124.6646312 | 216.9495963 |
| YOL007C | CSI2 | 0.68169872 | 0.97060928 | 0.08746554 | 124.2865731 | 216.210038 |
| YER080W | FMP29 | 0.68838179 | 0.97133463 | 0.08769293 | 132.7580367 | 231.9739312 |
| YDR066C | YDR066C | 0.46815826 | 0.96815259 | 0.0878109 | 124.7653064 | 217.0413333 |
| YBR201W | DER1 | 0.57507784 | 0.96815259 | 0.08782398 | 133.4878132 | 233.3119572 |
| YMR042W | ARG80 | 0.46889912 | 0.96815259 | 0.08815211 | 115.2811477 | 199.2863054 |
| YHR012W | VPS29 | 0.61928511 | 0.96815259 | 0.08853349 | 111.8664597 | 192.8474675 |
| YLR165C | PUS5 | 0.22308646 | 0.96815259 | 0.08856022 | 104.8897071 | 179.8266461 |
| YJR131W | MNS1 | 0.44252118 | 0.96815259 | 0.08877269 | 124.527661 | 216.4257437 |
| YOL017W | ESC8 | 0.69118722 | 0.97135148 | 0.0887878 | 130.8274859 | 228.1761774 |
| YLR199C | YLR199C | 0.54042417 | 0.96815259 | 0.08879758 | 123.4062539 | 214.3291566 |
| YDR475C | JIP4 | 0.66605468 | 0.9703088 | 0.08893993 | 134.7637839 | 235.4926094 |
| YOL121C | RPS19A | 0.6228444 | 0.96815259 | 0.08900816 | 125.0031561 | 217.2706761 |
| YDR438W | THI74 | 0.23277811 | 0.96815259 | 0.08912894 | 122.037832 | 211.7168507 |
| YNL164C | IBD2 | 0.06884018 | 0.96815259 | 0.08917594 | 121.8605718 | 211.3777338 |
| YLR126C | YLR126C | 0.58344718 | 0.96815259 | 0.08930995 | 124.0423199 | 215.4240686 |
| YNL128W | TEP1 | 0.58965036 | 0.96815259 | 0.08939988 | 121.5857584 | 210.8249317 |
| YOR024W | YOR024W | 0.63211186 | 0.96815259 | 0.0894845 | 133.4138741 | 232.8766609 |
| YDR485C | VPS72 | 0.14608867 | 0.96815259 | 0.08951458 | 102.9482855 | 176.0337719 |
| YPR059C | YPR059C | 0.48915916 | 0.96815259 | 0.08989489 | 112.4061259 | 193.6104953 |
| YPL056C | YPL056C | 0.52493075 | 0.96815259 | 0.08990186 | 133.9444679 | 233.7918136 |
| YGR071C | YGR071C | 0.61200928 | 0.96815259 | 0.08991983 | 110.7078952 | 190.4377595 |
| YPL183W-A | RTC6 | 0.07886017 | 0.96815259 | 0.08994936 | 89.19963414 | 150.3060252 |
| YLR110C | CCW12 | 0.45166855 | 0.96815259 | 0.08997764 | 101.7832015 | 173.7772359 |
| YBL010C | YBL010C | 0.28367823 | 0.96815259 | 0.09011979 | 129.6483585 | 225.7378396 |
| YPL240C | HSP82 | 0.63583584 | 0.96815259 | 0.09017795 | 122.1922526 | 211.8170939 |
| YIL015W | BAR1 | 0.73226793 | 0.97135148 | 0.09020711 | 141.2133924 | 247.2982723 |
| YER019C-A | SBH2 | 0.51838679 | 0.96815259 | 0.09040055 | 130.8531124 | 227.9351883 |
| YFL004W | VTC2 | 0.61319754 | 0.96815259 | 0.09054413 | 129.4052618 | 225.2083249 |
| YGR236C | SPG1 | 0.69717084 | 0.97135148 | 0.09056855 | 128.2666188 | 223.0796649 |
| YFR053C | HXK1 | 0.5968869 | 0.96815259 | 0.09075731 | 134.6602852 | 234.9740767 |
| YFR045W | YFR045W | 0.60830282 | 0.96815259 | 0.09084443 | 132.7696737 | 231.4312946 |
| YDR120C | TRM1 | 0.66029389 | 0.96815259 | 0.09111701 | 129.0779423 | 224.4950811 |
| YLR367W | RPS22B | 0.62227408 | 0.96815259 | 0.09117804 | 112.6042989 | 193.7504356 |
| YER051W | JHD1 | 0.48438951 | 0.96815259 | 0.09120858 | 128.479729 | 223.3626392 |
| YGL226W | MTC3 | 0.49791603 | 0.96815259 | 0.09121049 | 113.369101 | 195.1714625 |
| YHR087W | RTC3 | 0.6508238 | 0.96815259 | 0.09150361 | 122.1888064 | 211.5732758 |

| | | | | | | |
|---|---|---|---|---|---|---|
| YFR009W | GCN20 | 0.64578277 | 0.96815259 | 0.09163764 | 127.2475965 | 220.9871025 |
| YHR132C | ECM14 | 0.62262107 | 0.96815259 | 0.09164828 | 130.4782319 | 227.0123667 |
| YGR266W | YGR266W | 0.69260537 | 0.97135148 | 0.09186804 | 136.5688599 | 238.3358692 |
| YMR247C | RKR1 | 0.64559018 | 0.96815259 | 0.09192472 | 129.2162159 | 224.6084091 |
| YDR336W | YDR336W | 0.59103437 | 0.96815259 | 0.09204612 | 127.4804785 | 221.348426 |
| YLR003C | YLR003C | 0.55684293 | 0.96815259 | 0.09207681 | 120.7324617 | 208.7536318 |
| YLL023C | YLL023C | 0.59185993 | 0.96815259 | 0.09227698 | 126.4935168 | 219.4657806 |
| YGR242W | YGR242W | 0.56027595 | 0.96815259 | 0.09253925 | 130.6213404 | 227.119806 |
| YML131W | YML131W | 0.10403976 | 0.96815259 | 0.09260504 | 118.8995603 | 205.2395249 |
| YLR079W | SIC1 | 0.65206842 | 0.96815259 | 0.09263044 | 97.54092279 | 165.387672 |
| YFL030W | AGX1 | 0.59170763 | 0.96815259 | 0.09264333 | 115.0126786 | 197.9811878 |
| YBR054W | YRO2 | 0.56406782 | 0.96815259 | 0.09265245 | 134.8286435 | 234.9488027 |
| YPL272C | YPL272C | 0.668184 | 0.9703088 | 0.09267262 | 112.8599472 | 193.9597432 |
| YLR220W | CCC1 | 0.27182437 | 0.96815259 | 0.09270648 | 115.9474939 | 199.7138981 |
| YMR111C | YMR111C | 0.67845937 | 0.97060928 | 0.09271348 | 112.8650299 | 193.9619081 |
| YPR129W | SCD6 | 0.69128429 | 0.97135148 | 0.09275199 | 117.2903472 | 202.2110152 |
| YJR125C | ENT3 | 0.08996272 | 0.96815259 | 0.09289952 | 104.1531248 | 177.6754062 |
| YOR052C | YOR052C | 0.68556037 | 0.97133463 | 0.09299184 | 127.5667054 | 221.3399412 |
| YOR051C | YOR051C | 0.65720816 | 0.96815259 | 0.09324344 | 127.3588938 | 220.9071885 |
| YLL025W | PAU17 | 0.44785497 | 0.96815259 | 0.09331274 | 123.6080779 | 213.8971449 |
| YMR169C | ALD3 | 0.1442089 | 0.96815259 | 0.09339051 | 118.6813244 | 204.6917218 |
| YOR015W | YOR015W | 0.61252031 | 0.96815259 | 0.09359865 | 128.8586332 | 223.6415377 |
| YBR059C | AKL1 | 0.46397399 | 0.96815259 | 0.09376723 | 129.9965125 | 225.7342116 |
| YOL119C | MCH4 | 0.66420562 | 0.96977183 | 0.09387376 | 117.8247628 | 203.0071579 |
| YKL156W | RPS27A | 0.21532364 | 0.96815259 | 0.09396515 | 104.6004806 | 178.3191821 |
| YBR005W | RCR1 | 0.36339594 | 0.96815259 | 0.09402129 | 126.607872 | 219.3667684 |
| YDR042C | YDR042C | 0.32421608 | 0.96815259 | 0.09413153 | 109.6822608 | 187.7701074 |
| YPR150W | YPR150W | 0.52644066 | 0.96815259 | 0.09436751 | 126.4540338 | 219.0177651 |
| YBR063C | YBR063C | 0.40022953 | 0.96815259 | 0.09473119 | 124.364538 | 215.0544136 |
| YMR241W | YHM2 | 0.05438447 | 0.96815259 | 0.09497357 | 116.2207159 | 199.817656 |
| YOR128C | ADE2 | 0.7464768 | 0.97135148 | 0.09502415 | 112.7993541 | 193.4256054 |
| YER074W | RPS24A | 0.42262507 | 0.96815259 | 0.0950668 | 100.3727589 | 170.2345442 |
| YML034W | SRC1 | 0.49900551 | 0.96815259 | 0.09510624 | 107.8785641 | 184.2305334 |
| YBR068C | BAP2 | 0.64180358 | 0.96815259 | 0.09515443 | 129.3309174 | 224.2440478 |
| YLR172C | DPH5 | 0.26782234 | 0.96815259 | 0.09530435 | 113.3061352 | 194.3208945 |
| YLR136C | TIS11 | 0.39580012 | 0.96815259 | 0.09549116 | 118.9106322 | 204.7433564 |
| YAL013W | DEP1 | 0.42156141 | 0.96815259 | 0.09599661 | 114.5025046 | 196.4289116 |
| YOL013W-A | YOL013W-A | 0.62643689 | 0.96815259 | 0.09600135 | 122.7356997 | 211.7881553 |
| YMR320W | YMR320W | 0.68838767 | 0.97133463 | 0.09600159 | 135.3039826 | 235.2358709 |
| YFR057W | YFR057W | 0.48001978 | 0.96815259 | 0.09605079 | 131.481015 | 228.0948209 |
| YIL166C | YIL166C | 0.55033797 | 0.96815259 | 0.09605867 | 127.3823416 | 220.4468031 |
| YNL066W | SUN4 | 0.38813728 | 0.96815259 | 0.09606185 | 108.8335149 | 185.8409952 |
| YDL137W | ARF2 | 0.38111052 | 0.96815259 | 0.09606713 | 127.6683499 | 220.9788732 |
| YPL182C | YPL182C | 0.63812286 | 0.96815259 | 0.09610757 | 125.3319077 | 216.6126964 |
| YJR066W | TOR1 | 0.61159734 | 0.96815259 | 0.09633791 | 120.4205322 | 207.4086416 |
| YLR095C | IOC2 | 0.52029754 | 0.96815259 | 0.0963898 | 96.5280287 | 162.8247901 |
| YKR039W | GAP1 | 0.411141 | 0.96815259 | 0.09639758 | 119.7627906 | 206.1708549 |
| YOR111W | YOR111W | 0.48822098 | 0.96815259 | 0.09640697 | 126.5327285 | 218.7993681 |
| YOL132W | GAS4 | 0.45902899 | 0.96815259 | 0.09643136 | 117.9611563 | 202.8036228 |
| YBR188C | NTC20 | 0.52682911 | 0.96815259 | 0.09651191 | 131.3219536 | 227.7154964 |
| YLR194C | YLR194C | 0.59344825 | 0.96815259 | 0.09654747 | 119.6516089 | 205.93659 |
| YGR018C | YGR018C | 0.41846334 | 0.96815259 | 0.09658176 | 128.5707383 | 222.57024 |
| YOR161C | PNS1 | 0.52933572 | 0.96815259 | 0.09663477 | 116.9938297 | 200.9625251 |
| YER002W | NOP16 | 0.5898444 | 0.96815259 | 0.09679769 | 114.8688635 | 196.9689508 |
| YBL064C | PRX1 | 0.55373761 | 0.96815259 | 0.09680943 | 135.2264853 | 234.9466278 |
| YBR130C | SHE3 | 0.51522203 | 0.96815259 | 0.09689116 | 128.1473813 | 221.7250074 |
| YGR225W | AMA1 | 0.52283136 | 0.96815259 | 0.09691287 | 114.1289853 | 195.5679875 |
| YPR090W | YPR090W | 0.55819473 | 0.96815259 | 0.09692275 | 119.6167162 | 205.8042902 |
| YBR292C | YBR292C | 0.49585367 | 0.96815259 | 0.09693984 | 129.7217846 | 224.6535428 |
| YDL041W | YDL041W | 0.51039065 | 0.96815259 | 0.0971268 | 121.3025448 | 208.9128814 |
| YLR179C | YLR179C | 0.60886978 | 0.96815259 | 0.09732127 | 111.690224 | 190.9450277 |
| YOR202W | HIS3 | 0.08637691 | 0.96815259 | 0.09732992 | 115.9345175 | 198.8617596 |
| YMR302C | YME2 | 0.62297875 | 0.96815259 | 0.09746808 | 140.7646885 | 245.1609171 |
| YLR255C | YLR255C | 0.32864317 | 0.96815259 | 0.09812148 | 102.3677782 | 173.4095036 |

| | | | | | | |
|---|---|---|---|---|---|---|
| YCR033W | SNT1 | 0.73867093 | 0.97135148 | 0.0982567 | 110.5269264 | 188.6072369 |
| YER034W | YER034W | 0.83092146 | 0.97994418 | 0.09861289 | 99.01228108 | 167.061391 |
| YGR096W | TPC1 | 0.64859049 | 0.96815259 | 0.09882726 | 125.1157263 | 215.7223608 |
| YIL025C | YIL025C | 0.69903529 | 0.97135148 | 0.09889001 | 127.0068898 | 219.2393341 |
| YBR185C | MBA1 | 0.1114029 | 0.96815259 | 0.09898512 | 112.2773212 | 191.7423849 |
| YGR255C | COQ6 | 0.61717644 | 0.96815259 | 0.09909245 | 111.7246153 | 190.6920198 |
| YBL053W | YBL053W | 0.47619305 | 0.96815259 | 0.09925462 | 126.2265479 | 217.7182139 |
| YER072W | VTC1 | 0.4202242 | 0.96815259 | 0.09937641 | 128.4984623 | 221.9349545 |
| YBR166C | TYR1 | 0.38302751 | 0.96815259 | 0.09946685 | 137.0264846 | 237.8288897 |
| YJL071W | ARG2 | 0.52968081 | 0.96815259 | 0.09948946 | 130.0963965 | 224.8958641 |
| YGL156W | AMS1 | 0.647816 | 0.96815259 | 0.09953112 | 135.1527853 | 234.3217529 |
| YPL168W | YPL168W | 0.53011373 | 0.96815259 | 0.09962053 | 115.7461379 | 198.1001271 |
| YLR290C | YLR290C | 0.13183472 | 0.96815259 | 0.09986033 | 104.9965162 | 178.0023735 |
| YER001W | MNN1 | 0.51327042 | 0.96815259 | 0.09991663 | 129.184054 | 223.1172775 |
| YHL012W | YHL012W | 0.51011778 | 0.96815259 | 0.09994346 | 124.7747245 | 214.8862972 |
| YFR032C | YFR032C | 0.64791594 | 0.96815259 | 0.09995742 | 132.462996 | 229.2272632 |
| YDR099W | BMH2 | 0.50435547 | 0.96815259 | 0.10005369 | 129.058563 | 222.8586124 |
| YKL023W | YKL023W | 0.55393603 | 0.96815259 | 0.10023137 | 116.0435667 | 198.5456341 |
| YLR426W | YLR426W | 0.65429272 | 0.96815259 | 0.10029689 | 110.6701997 | 188.5091904 |
| YBR203W | COS111 | 0.50500956 | 0.96815259 | 0.10044183 | 137.8305909 | 239.154462 |
| YDR492W | IZH1 | 0.61323304 | 0.96815259 | 0.10049274 | 130.3694472 | 225.2256168 |
| YBL091C | MAP2 | 0.29017621 | 0.96815259 | 0.10056867 | 139.8520329 | 242.9030115 |
| YOR382W | FIT2 | 0.08624454 | 0.96815259 | 0.10071481 | 118.0600801 | 202.2211315 |
| YOL043C | NTG2 | 0.73026298 | 0.97135148 | 0.10078242 | 122.0668556 | 209.6841825 |
| YAL048C | GEM1 | 0.79143884 | 0.97320687 | 0.10084564 | 33.35951998 | 44.17783901 |
| YNR006W | VPS27 | 0.42328218 | 0.96815259 | 0.10103244 | 87.63301866 | 145.3986288 |
| YOR351C | MEK1 | 0.64050388 | 0.96815259 | 0.10107682 | 125.9722524 | 216.9174873 |
| YHR115C | DMA1 | 0.67981711 | 0.97060928 | 0.10108956 | 136.0943842 | 235.7993535 |
| YOR156C | NFI1 | 0.65704893 | 0.96815259 | 0.10128693 | 134.5033919 | 232.795808 |
| YPR200C | ARR2 | 0.58684973 | 0.96815259 | 0.10138592 | 128.5487395 | 221.6689057 |
| YLR425W | TUS1 | 0.14840189 | 0.96815259 | 0.10142744 | 101.0115396 | 170.2872583 |
| YNL284C | MRPL10 | 0.52597341 | 0.96815259 | 0.10146964 | 128.2851511 | 221.1621559 |
| YOR093C | YOR093C | 0.66761095 | 0.9703088 | 0.10157797 | 130.0875607 | 224.5053855 |
| YPL239W | YAR1 | 0.62549917 | 0.96815259 | 0.10176072 | 77.10256215 | 125.6222831 |
| YLR097C | HRT3 | 0.53730805 | 0.96815259 | 0.10177114 | 119.5803895 | 204.8683047 |
| YOR235W | YOR235W | 0.4647294 | 0.96815259 | 0.10207728 | 107.3387845 | 181.9751846 |
| YBR156C | SLI15 | 0.07553136 | 0.96815259 | 0.10210867 | 125.6077848 | 216.0527489 |
| YPR027C | YPR027C | 0.62719419 | 0.96815259 | 0.10217135 | 118.3029374 | 202.4133856 |
| YAR023C | YAR023C | 0.5311866 | 0.96815259 | 0.10217479 | 137.1990829 | 237.6659779 |
| YML112W | CTK3 | 0.35560788 | 0.96815259 | 0.1025715 | 117.5534886 | 200.9435369 |
| YKL110C | KTI12 | 0.25052528 | 0.96815259 | 0.10261032 | 100.5493319 | 169.2131295 |
| YNL293W | MSB3 | 0.51979132 | 0.96815259 | 0.10262785 | 131.2538089 | 226.4931678 |
| YBR180W | DTR1 | 0.30916818 | 0.96815259 | 0.10264951 | 131.7480902 | 227.4114347 |
| YGL179C | TOS3 | 0.13094051 | 0.96815259 | 0.10289262 | 115.5005984 | 197.0561005 |
| YOR084W | YOR084W | 0.63014541 | 0.96815259 | 0.10299831 | 128.0851028 | 220.5151977 |
| YGL071W | AFT1 | 0.59273041 | 0.96815259 | 0.10307132 | 125.9310389 | 216.4834373 |
| YMR180C | CTL1 | 0.53191568 | 0.96815259 | 0.10309348 | 117.3290443 | 200.4313346 |
| YKL167C | MRP49 | 0.7023669 | 0.97135148 | 0.1031219 | 122.5701308 | 210.2041706 |
| YLR232W | YLR232W | 0.359444 | 0.96815259 | 0.1031376 | 114.2971309 | 194.767007 |
| YOR029W | YOR029W | 0.18427254 | 0.96815259 | 0.10331357 | 116.7827553 | 199.372749 |
| YOL152W | FRE7 | 0.64729724 | 0.96815259 | 0.10331736 | 129.2390038 | 222.6108165 |
| YGR210C | YGR210C | 0.59501664 | 0.96815259 | 0.10334921 | 120.9882354 | 207.2122348 |
| YDR522C | SPS2 | 0.46642032 | 0.96815259 | 0.10383753 | 107.4100281 | 181.7928857 |
| YAL015C | NTG1 | 0.43477268 | 0.96815259 | 0.10423706 | 130.0470094 | 223.9535615 |
| YKR074W | YKR074W | 0.1998409 | 0.96815259 | 0.10428158 | 92.96835741 | 154.7705617 |
| YOL031C | SIL1 | 0.64991955 | 0.96815259 | 0.10466149 | 133.3520774 | 230.043591 |
| YIL156W | UBP7 | 0.44589488 | 0.96815259 | 0.10480699 | 128.2599377 | 220.5174892 |
| YLR356W | YLR356W | 0.60517513 | 0.96815259 | 0.10480755 | 120.2361123 | 205.5479047 |
| YBR020W | GAL1 | 0.41163449 | 0.96815259 | 0.10520752 | 123.5473086 | 211.6537456 |
| YBR206W | YBR206W | 0.3774493 | 0.96815259 | 0.10546401 | 138.5846849 | 239.6619893 |
| YPR115W | YPR115W | 0.65076396 | 0.96815259 | 0.10549392 | 139.6993619 | 241.7362073 |
| YMR263W | SAP30 | 0.37262804 | 0.96815259 | 0.10563073 | 99.70573422 | 167.0984159 |
| YOL053C-A | YOL053C-A | 0.62307778 | 0.96815259 | 0.10564017 | 129.2803748 | 222.272048 |
| YOL107W | YOL107W | 0.59661878 | 0.96815259 | 0.10578151 | 128.0807962 | 220.0087686 |

| | | | | | | |
|---|---|---|---|---|---|---|
| YFL021W | GAT1 | 0.58796541 | 0.96815259 | 0.10614457 | 131.0713531 | 225.5230264 |
| YMR075W | RCO1 | 0.43860869 | 0.96815259 | 0.1064279 | 106.8597361 | 180.3023821 |
| YCR073W-A | SOL2 | 0.25715854 | 0.96815259 | 0.10643342 | 123.8477819 | 211.9947926 |
| YOL011W | PLB3 | 0.54001894 | 0.96815259 | 0.10653257 | 119.2790524 | 203.4534815 |
| YGR199W | PMT6 | 0.68432797 | 0.97080037 | 0.10664856 | 129.6766639 | 222.8308009 |
| YOR285W | YOR285W | 0.67080276 | 0.97060928 | 0.10673492 | 131.8671319 | 226.9019379 |
| YDR538W | PAD1 | 0.61510087 | 0.96815259 | 0.10675215 | 126.536684 | 216.9542125 |
| YLR284C | ECI1 | 0.56728037 | 0.96815259 | 0.10687015 | 106.7292847 | 179.9798129 |
| YPR092W | YPR092W | 0.40057464 | 0.96815259 | 0.10691636 | 118.8823965 | 202.644743 |
| YAL053W | FLC2 | 0.47136371 | 0.96815259 | 0.10710902 | 131.4874253 | 226.1265567 |
| YPR023C | EAF3 | 0.61555366 | 0.96815259 | 0.10728949 | 109.2275503 | 184.5655591 |
| YBR212W | NGR1 | 0.64966709 | 0.96815259 | 0.10730955 | 123.7617849 | 211.677464 |
| YLR144C | ACF2 | 0.40614313 | 0.96815259 | 0.10731872 | 113.3729531 | 192.2941109 |
| YLR280C | YLR280C | 0.57410327 | 0.96815259 | 0.10745888 | 126.2313445 | 216.258007 |
| YDR029W | YDR029W | 0.63835251 | 0.96815259 | 0.10756761 | 128.0123262 | 219.5611884 |
| YNL283C | WSC2 | 0.54019924 | 0.96815259 | 0.10762049 | 126.9583263 | 217.5853444 |
| YHR029C | YHI9 | 0.68031141 | 0.97060928 | 0.10777543 | 138.6158251 | 239.3061728 |
| YNL049C | SFB2 | 0.63364851 | 0.96815259 | 0.10790324 | 133.0681352 | 228.933352 |
| YLR210W | CLB4 | 0.44971645 | 0.96815259 | 0.10796292 | 126.3129288 | 216.3199534 |
| YFL028C | CAF16 | 0.61264457 | 0.96815259 | 0.10802062 | 121.3954723 | 207.1354691 |
| YFL026W | STE2 | 0.58520569 | 0.96815259 | 0.10829681 | 129.7346341 | 222.6437969 |
| YHR140W | YHR140W | 0.4536408 | 0.96815259 | 0.10836002 | 131.5248357 | 225.9723299 |
| YGL258W | VEL1 | 0.61544327 | 0.96815259 | 0.10865026 | 130.6160493 | 224.2248985 |
| YMR141C | YMR141C | 0.70193426 | 0.97135148 | 0.1087603 | 115.5010805 | 196.0062603 |
| YBR034C | HMT1 | 0.35855299 | 0.96815259 | 0.10877645 | 113.1314032 | 191.5824289 |
| YLR171W | YLR171W | 0.30155756 | 0.96815259 | 0.10881147 | 126.7915952 | 217.0610156 |
| YNL179C | SRF6 | 0.52590129 | 0.96815259 | 0.10894325 | 129.2914576 | 221.7012334 |
| YKR032W | YKR032W | 0.68701891 | 0.97133463 | 0.10902955 | 136.9776024 | 236.0252784 |
| YML124C | TUB3 | 0.68076965 | 0.97060928 | 0.10907964 | 122.498389 | 209.0034606 |
| YDL109C | YDL109C | 0.10306879 | 0.96815259 | 0.10921904 | 121.3216188 | 206.7830808 |
| YMR256C | COX7 | 0.24608343 | 0.96815259 | 0.10922712 | 80.62562682 | 130.8579904 |
| YPL039W | YPL039W | 0.61699502 | 0.96815259 | 0.10925399 | 133.6347441 | 229.7485532 |
| YER118C | SHO1 | 0.36293767 | 0.96815259 | 0.10934967 | 129.631756 | 222.2633264 |
| YPR188C | MLC2 | 0.5847442 | 0.96815259 | 0.10940697 | 119.1327538 | 202.6658164 |
| YHR014W | SPO13 | 0.66491213 | 0.96985526 | 0.10964066 | 132.0776792 | 226.7744046 |
| YPR125W | YLH47 | 0.55817602 | 0.96815259 | 0.10974224 | 127.0030444 | 217.2888251 |
| YGR137W | YGR137W | 0.4666737 | 0.96815259 | 0.10993253 | 139.1686302 | 239.9512257 |
| YPL274W | SAM3 | 0.57527331 | 0.96815259 | 0.11005071 | 122.3963284 | 208.6391626 |
| YGL231C | YGL231C | 0.42743035 | 0.96815259 | 0.11015198 | 112.688905 | 190.5105717 |
| YIL047C | SYG1 | 0.64249348 | 0.96815259 | 0.11027848 | 132.1762978 | 226.8441744 |
| YOR062C | YOR062C | 0.7066526 | 0.97135148 | 0.11030395 | 127.3373649 | 217.8119579 |
| YLR385C | SWC7 | 0.51868543 | 0.96815259 | 0.11032612 | 121.453392 | 206.8306741 |
| YOR132W | VPS17 | 0.54175592 | 0.96815259 | 0.11065047 | 128.0431135 | 219.0665705 |
| YOR321W | PMT3 | 0.66869748 | 0.9703088 | 0.11083443 | 135.0341439 | 232.0763011 |
| YMR306W | FKS3 | 0.37034184 | 0.96815259 | 0.1110291 | 132.8226819 | 227.9156719 |
| YPL112C | PEX25 | 0.65915323 | 0.96815259 | 0.11126611 | 128.434398 | 219.6863187 |
| YBL019W | APN2 | 0.65083337 | 0.96815259 | 0.11129268 | 123.9217963 | 211.2627169 |
| YIL158W | YIL158W | 0.45897009 | 0.96815259 | 0.1112954 | 108.8890024 | 183.2166061 |
| YOR337W | TEA1 | 0.62502874 | 0.96815259 | 0.11140324 | 117.2706275 | 198.8343026 |
| YLR130C | ZRT2 | 0.50242597 | 0.96815259 | 0.1114307 | 125.3038364 | 213.8163773 |
| YML004C | GLO1 | 0.60711451 | 0.96815259 | 0.11151732 | 116.2926933 | 196.9894112 |
| YBL100C | YBL100C | 0.3644618 | 0.96815259 | 0.11157246 | 130.2049477 | 222.93465 |
| YFL027C | GYP8 | 0.5597226 | 0.96815259 | 0.1118442 | 127.4395361 | 217.726754 |
| YGR041W | BUD9 | 0.47920754 | 0.96815259 | 0.11185391 | 126.9695872 | 216.8482644 |
| YPL208W | RKM1 | 0.67406597 | 0.97060928 | 0.11197157 | 115.2529971 | 194.9683801 |
| YLR038C | COX12 | 0.02390314 | 0.83447212 | 0.11197807 | 76.52943578 | 122.723396 |
| YGL096W | TOS8 | 0.53808919 | 0.96815259 | 0.11200225 | 116.3332056 | 196.9781551 |
| YHR139C | SPS100 | 0.63805459 | 0.96815259 | 0.11208223 | 127.3176156 | 217.4566718 |
| YMR121C | RPL15B | 0.63606142 | 0.96815259 | 0.11209236 | 113.432316 | 191.5500325 |
| YGL029W | CGR1 | 0.48234586 | 0.96815259 | 0.11217094 | 118.5346846 | 201.0550905 |
| YOL057W | YOL057W | 0.66307407 | 0.96927465 | 0.11223905 | 131.9637843 | 226.0966189 |
| YIL013C | PDR11 | 0.6008304 | 0.96815259 | 0.11264258 | 135.8393533 | 233.2547341 |
| YHR021W-A | ECM12 | 0.55175975 | 0.96815259 | 0.11271863 | 118.3505233 | 200.6134373 |
| YDL211C | YDL211C | 0.54619292 | 0.96815259 | 0.11296959 | 130.3883878 | 223.0266938 |

| | | | | | | |
|---|---|---|---|---|---|---|
| YGR061C | ADE6 | 0.4832996 | 0.96815259 | 0.11308007 | 121.4709539 | 206.3702814 |
| YBR273C | UBX7 | 0.37238006 | 0.96815259 | 0.11311696 | 134.3818544 | 230.450632 |
| YGL006W | PMC1 | 0.59714993 | 0.96815259 | 0.11317346 | 130.5138027 | 223.2241635 |
| YOL015W | IRC10 | 0.68803817 | 0.97133463 | 0.11324134 | 137.88091 | 236.9563002 |
| YKL208W | CBT1 | 0.11369836 | 0.96815259 | 0.1132832 | 89.27803822 | 146.2738528 |
| YNL239W | LAP3 | 0.495946 | 0.96815259 | 0.11372697 | 139.7589867 | 240.3731329 |
| YBR217W | ATG12 | 0.48348913 | 0.96815259 | 0.11376794 | 130.6114462 | 223.2998754 |
| YDR525W-A | SNA2 | 0.49570745 | 0.96815259 | 0.11382229 | 130.2197873 | 222.5594528 |
| YDR410C | STE14 | 0.2963668 | 0.96815259 | 0.11391129 | 135.467459 | 232.3337254 |
| YMR261C | TPS3 | 0.50442629 | 0.96815259 | 0.11392138 | 115.7591501 | 195.5635161 |
| YOR010C | TIR2 | 0.46516724 | 0.96815259 | 0.11392915 | 125.2069044 | 213.1881342 |
| YFR021W | ATG18 | 0.33570334 | 0.96815259 | 0.11402039 | 126.0097412 | 214.6695908 |
| YGL195W | GCN1 | 0.50918204 | 0.96815259 | 0.11404217 | 129.6143563 | 221.3905679 |
| YDL242W | YDL242W | 0.3930857 | 0.96815259 | 0.11449624 | 115.3479141 | 194.6933602 |
| YOL012C | HTZ1 | 0.3905925 | 0.96815259 | 0.11450504 | 113.8135587 | 191.8292462 |
| YML010C-B | YML010C-B | 0.5361359 | 0.96815259 | 0.11452263 | 102.6547934 | 171.0079747 |
| YLR209C | PNP1 | 0.5577584 | 0.96815259 | 0.11474525 | 115.4337489 | 194.8089066 |
| YOL129W | VPS68 | 0.55732489 | 0.96815259 | 0.11491385 | 129.885812 | 221.7409101 |
| YDR242W | AMD2 | 0.64209795 | 0.96815259 | 0.11507422 | 118.5452502 | 200.5549044 |
| YMR204C | INP1 | 0.50348086 | 0.96815259 | 0.11524542 | 117.1233564 | 197.8715201 |
| YLR267W | BOP2 | 0.45672677 | 0.96815259 | 0.11548647 | 127.617016 | 217.4056359 |
| YHR076W | PTC7 | 0.39006313 | 0.96815259 | 0.11554057 | 122.6571982 | 208.1427656 |
| YDR326C | YSP2 | 0.58417788 | 0.96815259 | 0.11555828 | 120.7444947 | 204.5711991 |
| YLR297W | YLR297W | 0.53165189 | 0.96815259 | 0.11570048 | 128.7516635 | 219.4841459 |
| YMR181C | YMR181C | 0.47013317 | 0.96815259 | 0.11573082 | 121.6438883 | 206.218237 |
| YPL015C | HST2 | 0.66627754 | 0.9703088 | 0.1157841 | 124.7457492 | 211.9956183 |
| YLL057C | JLP1 | 0.29595389 | 0.96815259 | 0.11599817 | 113.9468278 | 191.8104986 |
| YKL205W | LOS1 | 0.25896228 | 0.96815259 | 0.1161949 | 98.18075693 | 162.361623 |
| YPL245W | YPL245W | 0.46410059 | 0.96815259 | 0.11619546 | 131.063079 | 223.7077532 |
| YDR290W | :::RTT103 | 0.61058946 | 0.96815259 | 0.11622005 | 112.2147724 | 188.5393917 |
| YPL120W | VPS30 | 0.29617914 | 0.96815259 | 0.11624321 | 113.0448797 | 190.0839177 |
| YEL071W | DLD3 | 0.57593655 | 0.96815259 | 0.11627978 | 137.0327579 | 234.8298619 |
| YDR034C | LYS14 | 0.05271376 | 0.96815259 | 0.116312 | 111.5354062 | 187.2554815 |
| YNL268W | LYP1 | 0.02767595 | 0.89818524 | 0.11649366 | 122.5555525 | 207.7824615 |
| YMR148W | YMR148W | 0.63798498 | 0.96815259 | 0.11650362 | 117.7739372 | 198.8599548 |
| YLR094C | GIS3 | 0.54299684 | 0.96815259 | 0.1165586 | 131.1067584 | 223.7242132 |
| YOR365C | YOR365C | 0.45012005 | 0.96815259 | 0.1166413 | 121.5338191 | 205.8498465 |
| YHR049W | FSH1 | 0.60194949 | 0.96815259 | 0.11678765 | 138.5293195 | 237.5309461 |
| YOL113W | SKM1 | 0.63733221 | 0.96815259 | 0.11704683 | 137.280331 | 235.1543849 |
| YIL007C | NAS2 | 0.6401393 | 0.96815259 | 0.11731156 | 107.9661462 | 180.4175701 |
| YMR246W | FAA4 | 0.58047181 | 0.96815259 | 0.11731229 | 121.5630315 | 205.7841897 |
| YMR219W | ESC1 | 0.600063 | 0.96815259 | 0.11733221 | 110.7622852 | 185.6304314 |
| YIR005W | IST3 | 0.37309343 | 0.96815259 | 0.1173834 | 105.191425 | 175.2281038 |
| YPR084W | YPR084W | 0.5756881 | 0.96815259 | 0.11739821 | 119.2233202 | 201.4037701 |
| YLL053C | YLL053C | 0.36444065 | 0.96815259 | 0.11744726 | 108.5057906 | 181.4000468 |
| YCR044C | PER1 | 0.54585091 | 0.96815259 | 0.11752643 | 117.9509089 | 199.0069616 |
| YOL025W | LAG2 | 0.66018778 | 0.96815259 | 0.11761841 | 140.1094205 | 240.330063 |
| YNL328C | MDJ2 | 0.53593483 | 0.96815259 | 0.11770922 | 126.1654361 | 214.2994933 |
| YMR104C | YPK2 | 0.22477093 | 0.96815259 | 0.11806447 | 121.4876807 | 205.50892 |
| YDR395W | SXM1 | 0.61584407 | 0.96815259 | 0.11807714 | 137.2918615 | 234.9913962 |
| YDR363W | ESC2 | 0.38753815 | 0.96815259 | 0.11818768 | 104.3963655 | 173.6007938 |
| YIL060W | YIL060W | 0.33837215 | 0.96815259 | 0.1182143 | 125.8569857 | 213.6335931 |
| YNL013C | YNL013C | 0.50680609 | 0.96815259 | 0.11823804 | 114.9316405 | 193.2466955 |
| YDR304C | CPR5 | 0.16879739 | 0.96815259 | 0.11834518 | 121.4434578 | 205.3761486 |
| YJL004C | SYS1 | 0.35936133 | 0.96815259 | 0.11861719 | 120.5876298 | 203.7307813 |
| YPL267W | ACM1 | 0.48065776 | 0.96815259 | 0.11876548 | 123.308151 | 208.7797107 |
| YGR234W | YHB1 | 0.62895691 | 0.96815259 | 0.11879011 | 130.8029069 | 222.7577386 |
| YOL042W | NGL1 | 0.488538 | 0.96815259 | 0.11885521 | 126.8585411 | 215.3873557 |
| YPR004C | YPR004C | 0.42715614 | 0.96815259 | 0.11894433 | 117.4079211 | 197.7400413 |
| YKL034W | TUL1 | 0.65571308 | 0.96815259 | 0.11918463 | 140.7597789 | 241.2629237 |
| YFR032C-A | RPL29 | 0.47842191 | 0.96815259 | 0.11920452 | 115.7398898 | 194.5815186 |
| YGR254W | ENO1 | 0.58809586 | 0.96815259 | 0.11922073 | 122.2059366 | 206.6418634 |
| YGR087C | PDC6 | 0.51071055 | 0.96815259 | 0.11932138 | 124.896164 | 211.6428089 |
| YLR328W | NMA1 | 0.59274971 | 0.96815259 | 0.1193237 | 119.5042987 | 201.5831704 |

| | | | | | | |
|---|---|---|---|---|---|---|
| YER048C | CAJ1 | 0.13831228 | 0.96815259 | 0.11954246 | 130.8103499 | 222.6368987 |
| YPR174C | YPR174C | 0.58171197 | 0.96815259 | 0.11970627 | 126.9381824 | 215.3835343 |
| YOL160W | YOL160W | 0.56555818 | 0.96815259 | 0.11994581 | 136.1916514 | 232.6041845 |
| YJL023C | PET130 | 0.06470314 | 0.96815259 | 0.11997629 | 126.6982388 | 214.887536 |
| YNL175C | NOP13 | 0.55121451 | 0.96815259 | 0.11998674 | 126.9602599 | 215.3744997 |
| YOR005C | DNL4 | 0.49644174 | 0.96815259 | 0.12017608 | 128.441969 | 218.1049138 |
| YNL324W | YNL324W | 0.59593583 | 0.96815259 | 0.12021048 | 134.8352369 | 230.0262228 |
| YOL065C | INP54 | 0.50592532 | 0.96815259 | 0.12027423 | 122.3569194 | 206.7348903 |
| YOR066W | YOR066W | 0.45360522 | 0.96815259 | 0.12030298 | 121.8139126 | 205.7166923 |
| YJR030C | YJR030C | 0.53384617 | 0.96815259 | 0.120441 | 133.5377406 | 227.5642964 |
| YOR177C | MPC54 | 0.5020105 | 0.96815259 | 0.12050705 | 131.0558693 | 222.9222163 |
| YOR298W | MUM3 | 0.60455924 | 0.96815259 | 0.12091224 | 132.5429897 | 225.6240727 |
| YMR251W-A | HOR7 | 0.53470083 | 0.96815259 | 0.12118868 | 130.9190691 | 222.5449357 |
| YNL194C | YNL194C | 0.51286452 | 0.96815259 | 0.12124332 | 120.9734824 | 203.9803708 |
| YHR015W | MIP6 | 0.44181091 | 0.96815259 | 0.12139476 | 121.1219174 | 204.2301782 |
| YBR046C | ZTA1 | 0.4431771 | 0.96815259 | 0.12164654 | 138.4281338 | 236.472079 |
| YER101C | AST2 | 0.58766182 | 0.96815259 | 0.12165091 | 122.2775562 | 206.3403027 |
| YBR058C | UBP14 | 0.50294092 | 0.96815259 | 0.12168732 | 129.639269 | 220.068011 |
| YER064C | YER064C | 0.48877538 | 0.96815259 | 0.12179181 | 127.8473059 | 216.7061596 |
| YML094W | GIM5 | 0.54283813 | 0.96815259 | 0.12199909 | 118.3414721 | 198.934677 |
| YNL086W | YNL086W | 0.58838019 | 0.96815259 | 0.12208223 | 119.7286226 | 201.5076989 |
| YIL122W | POG1 | 0.57700156 | 0.96815259 | 0.12220402 | 118.4440436 | 199.0893409 |
| YMR315W | YMR315W | 0.39097329 | 0.96815259 | 0.12232055 | 119.5059128 | 201.0495285 |
| YDR340W | YDR340W | 0.39675704 | 0.96815259 | 0.12232673 | 129.7503695 | 220.1607825 |
| YAL023C | PMT2 | 0.35705411 | 0.96815259 | 0.12235335 | 129.6097367 | 219.893647 |
| YPL057C | SUR1 | 0.29293718 | 0.96815259 | 0.12237368 | 113.5431715 | 189.915748 |
| YBR146W | MRPS9 | 0.4305846 | 0.96815259 | 0.12241387 | 134.8721157 | 229.700459 |
| YML108W | YML108W | 0.4147344 | 0.96815259 | 0.12251503 | 118.4431361 | 199.031954 |
| YLR436C | ECM30 | 0.51928578 | 0.96815259 | 0.12257679 | 110.2798012 | 183.7911372 |
| YGR059W | SPR3 | 0.61712076 | 0.96815259 | 0.12267483 | 129.2196549 | 219.1083303 |
| YNR056C | BIO5 | 0.6545788 | 0.96815259 | 0.12270721 | 141.275208 | 241.5937282 |
| YOR229W | WTM2 | 0.58989531 | 0.96815259 | 0.12279784 | 135.7824022 | 231.3299575 |
| YEL040W | UTR2 | 0.09082488 | 0.96815259 | 0.12292701 | 125.1565584 | 211.4829389 |
| YNR002C | ATO2 | 0.38510353 | 0.96815259 | 0.1229363 | 116.640765 | 195.5939605 |
| YPL192C | PRM3 | 0.56690906 | 0.96815259 | 0.12295991 | 145.5429912 | 249.510578 |
| YIL117C | PRM5 | 0.31717437 | 0.96815259 | 0.12296672 | 128.830066 | 218.3292315 |
| YDL034W | YDL034W | 0.34597203 | 0.96815259 | 0.12298928 | 131.7877694 | 223.8431705 |
| YLR072W | YLR072W | 0.33337808 | 0.96815259 | 0.12307414 | 104.8676626 | 173.6050297 |
| YJL151C | SNA3 | 0.5434344 | 0.96815259 | 0.12311917 | 132.2489044 | 224.6802192 |
| YEL064C | AVT2 | 0.51442541 | 0.96815259 | 0.12313262 | 128.9089755 | 218.4467403 |
| YNL116W | DMA2 | 0.57594913 | 0.96815259 | 0.12321673 | 125.1471632 | 211.4135294 |
| YML035C-A | YML035C-A | 0.54256142 | 0.96815259 | 0.12327151 | 107.2111769 | 177.9418162 |
| YBR286W | APE3 | 0.59107377 | 0.96815259 | 0.12328905 | 132.734995 | 225.5566634 |
| YBR158W | AMN1 | 0.19664228 | 0.96815259 | 0.12332322 | 133.9528201 | 227.8225536 |
| YGR212W | SLI1 | 0.60851276 | 0.96815259 | 0.12343165 | 132.7111971 | 225.4867293 |
| YBR028C | YBR028C | 0.12372356 | 0.96815259 | 0.12351514 | 121.2208725 | 204.0350894 |
| YKL053C-A | MDM35 | 0.56958425 | 0.96815259 | 0.12356331 | 85.34431919 | 137.0941076 |
| YPR018W | RLF2 | 0.47611117 | 0.96815259 | 0.12361545 | 113.8584856 | 190.2816406 |
| YDR032C | PST2 | 0.53338137 | 0.96815259 | 0.1237947 | 117.6874865 | 197.3930388 |
| YML075C | HMG1 | 0.36788455 | 0.96815259 | 0.12393397 | 124.1038564 | 209.3386682 |
| YPL246C | RBD2 | 0.62310262 | 0.96815259 | 0.12402782 | 126.1546562 | 213.1478946 |
| YDR541C | YDR541C | 0.61078354 | 0.96815259 | 0.1240958 | 133.1249144 | 226.1396403 |
| YOR023C | AHC1 | 0.2774251 | 0.96815259 | 0.12425001 | 130.7800706 | 221.7374163 |
| YGL015C | YGL015C | 0.53110343 | 0.96815259 | 0.12439403 | 134.040267 | 227.7939452 |
| YHR139C-A | YHR139C-A | 0.60119738 | 0.96815259 | 0.12453505 | 137.7533951 | 234.696014 |
| YGL221C | NIF3 | 0.46407771 | 0.96815259 | 0.12466394 | 110.3260202 | 183.5036124 |
| YOR007C | SGT2 | 0.50424121 | 0.96815259 | 0.12482304 | 128.3814187 | 217.1598065 |
| YGR196C | FYV8 | 0.63177302 | 0.96815259 | 0.12487116 | 137.2140149 | 233.6295425 |
| YOR164C | YOR164C | 0.59219156 | 0.96815259 | 0.12490722 | 129.581023 | 219.3827505 |
| YOL067C | RTG1 | 0.6026079 | 0.96815259 | 0.12496783 | 110.1630202 | 183.1450981 |
| YGL013C | PDR1 | 0.60456572 | 0.96815259 | 0.12499686 | 130.253608 | 220.6214922 |
| YBR083W | TEC1 | 0.39935951 | 0.96815259 | 0.12512171 | 128.1900902 | 216.7493755 |
| YDR524C | AGE1 | 0.48474352 | 0.96815259 | 0.12518068 | 110.7736409 | 184.2461732 |
| YOR214C | YOR214C | 0.61308946 | 0.96815259 | 0.12529198 | 134.8395444 | 229.1243025 |

| | | | | | | |
|---|---|---|---|---|---|---|
| YDR258C | HSP78 | 0.31692672 | 0.96815259 | 0.12532353 | 124.7536483 | 210.3021067 |
| YOR212W | STE4 | 0.63458133 | 0.96815259 | 0.12535084 | 139.6335677 | 238.0576343 |
| YNL205C | YNL205C | 0.41584423 | 0.96815259 | 0.12539283 | 109.7693473 | 182.3345432 |
| YER143W | DDI1 | 0.56291926 | 0.96815259 | 0.1254631 | 132.3240107 | 224.4006057 |
| YNL191W | DUG3 | 0.03887898 | 0.96815259 | 0.12558727 | 108.4183458 | 179.7792566 |
| YPR030W | CSR2 | 0.55018026 | 0.96815259 | 0.12567306 | 123.9106998 | 208.6668865 |
| YOR266W | PNT1 | 0.37477083 | 0.96815259 | 0.12573689 | 130.6537653 | 221.2355191 |
| YGR022C | YGR022C | 0.49734534 | 0.96815259 | 0.12580346 | 109.7357957 | 182.1984156 |
| YBL106C | SRO77 | 0.54096189 | 0.96815259 | 0.12586333 | 126.744169 | 213.9190183 |
| YCR060W | TAH1 | 0.49923825 | 0.96815259 | 0.12588844 | 114.7519819 | 191.5415443 |
| YPR044C | YPR044C | 0.72011991 | 0.97135148 | 0.12590831 | 80.08224193 | 126.8570965 |
| YLR344W | RPL26A | 0.38074292 | 0.96815259 | 0.12591053 | 108.146107 | 179.2134734 |
| YOL088C | MPD2 | 0.54520532 | 0.96815259 | 0.12593954 | 113.1884027 | 188.6153338 |
| YOR082C | YOR082C | 0.53469464 | 0.96815259 | 0.12602138 | 123.9189264 | 208.6198592 |
| YEL030W | ECM10 | 0.08016097 | 0.96815259 | 0.12620396 | 122.1324057 | 205.2541792 |
| YKL029C | MAE1 | 0.28258168 | 0.96815259 | 0.12621974 | 125.2269605 | 211.0246463 |
| YML117W | NAB6 | 0.57627395 | 0.96815259 | 0.12630461 | 123.0717523 | 206.9886292 |
| YKR057W | RPS21A | 0.5640873 | 0.96815259 | 0.12632796 | 108.0407452 | 178.9421574 |
| YGR144W | THI4 | 0.32836251 | 0.96815259 | 0.12644335 | 132.8781256 | 225.2588438 |
| YOR227W | YOR227W | 0.44757278 | 0.96815259 | 0.12648489 | 129.1621572 | 218.3187838 |
| YER035W | EDC2 | 0.3593418 | 0.96815259 | 0.12662494 | 124.1584951 | 208.9587265 |
| YOR114W | YOR114W | 0.31478184 | 0.96815259 | 0.12672658 | 123.9893481 | 208.6249598 |
| YGR290W | YGR290W | 0.64646643 | 0.96815259 | 0.12684376 | 131.323719 | 222.2871944 |
| YML102C-A | YML102C-A | 0.54247959 | 0.96815259 | 0.12699364 | 123.8776853 | 208.3688152 |
| YER044C-A | MEI4 | 0.48612462 | 0.96815259 | 0.12705142 | 122.5316506 | 205.8472657 |
| YBR073W | RDH54 | 0.46666147 | 0.96815259 | 0.12731941 | 121.4383773 | 203.7596337 |
| YNL304W | YPT11 | 0.25673903 | 0.96815259 | 0.12765685 | 112.8967911 | 187.7637717 |
| YOL112W | MSB4 | 0.54766505 | 0.96815259 | 0.12791451 | 120.129141 | 201.2105166 |
| YLR443W | ECM7 | 0.32330345 | 0.96815259 | 0.12802804 | 113.9753664 | 189.7095242 |
| YBR277C | YBR277C | 0.41983493 | 0.96815259 | 0.12804519 | 107.3789023 | 177.3998943 |
| YMR075C-A | YMR075C-A | 0.16851586 | 0.96815259 | 0.12836743 | 106.0671303 | 174.8949106 |
| YMR009W | ADI1 | 0.22158954 | 0.96815259 | 0.12838961 | 108.0985287 | 178.6807748 |
| YNL142W | MEP2 | 0.53672367 | 0.96815259 | 0.12839422 | 118.0590323 | 197.2625581 |
| YOR071C | THI71 | 0.60364473 | 0.96815259 | 0.12841266 | 129.6930755 | 218.964071 |
| YNL315C | ATP11 | 0.6273885 | 0.96815259 | 0.12859526 | 56.39801518 | 82.18994455 |
| YLL054C | YLL054C | 0.25605751 | 0.96815259 | 0.1285958 | 114.0554056 | 189.7571776 |
| YEL017C-A | PMP2 | 0.42294194 | 0.96815259 | 0.12867594 | 131.8261284 | 222.8964111 |
| YGR126W | YGR126W | 0.54896246 | 0.96815259 | 0.12871344 | 137.0335933 | 232.6048963 |
| YLR271W | YLR271W | 0.40868031 | 0.96815259 | 0.12878259 | 121.640542 | 203.8747819 |
| YMR126C | DLT1 | 0.33939222 | 0.96815259 | 0.12883979 | 105.9138435 | 174.5243464 |
| YLR452C | SST2 | 0.50757466 | 0.96815259 | 0.1289764 | 107.1039288 | 176.7201417 |
| YLR365W | YLR365W | 0.67441834 | 0.97060928 | 0.1290977 | 141.4498829 | 240.775246 |
| YNR040W | YNR040W | 0.59348916 | 0.96815259 | 0.12910518 | 133.9301529 | 226.7448766 |
| YGL104C | VPS73 | 0.36256638 | 0.96815259 | 0.12924444 | 122.4003861 | 205.2096661 |
| YHR108W | GGA2 | 0.48952262 | 0.96815259 | 0.12927039 | 111.414301 | 184.7090546 |
| YGR011W | YGR011W | 0.50824386 | 0.96815259 | 0.12946128 | 138.5685657 | 235.3346681 |
| YDR439W | LRS4 | 0.523364 | 0.96815259 | 0.12968901 | 123.0946264 | 206.4252508 |
| YNL140C | YNL140C | 0.36151598 | 0.96815259 | 0.12994663 | 112.1504716 | 185.9613801 |
| YJR003C | YJR003C | 0.27922195 | 0.96815259 | 0.12995556 | 129.2109753 | 217.7883602 |
| YOR021C | YOR021C | 0.29351539 | 0.96815259 | 0.13000084 | 132.194271 | 223.3459753 |
| YIL049W | DFG10 | 0.46703964 | 0.96815259 | 0.13004683 | 127.5031367 | 214.5858213 |
| YKL116C | PRR1 | 0.45438921 | 0.96815259 | 0.13024489 | 123.2428444 | 206.6022285 |
| YMR145C | NDE1 | 0.26839481 | 0.96815259 | 0.13026306 | 88.29461027 | 141.3985169 |
| YNR066C | YNR066C | 0.60348845 | 0.96815259 | 0.13043095 | 140.3554725 | 238.4947344 |
| YBL072C | RPS8A | 0.43442696 | 0.96815259 | 0.13068995 | 119.4381458 | 199.424372 |
| YBR207W | FTH1 | 0.44094951 | 0.96815259 | 0.13075581 | 138.0055828 | 234.0525367 |
| YLR040C | YLR040C | 0.36636791 | 0.96815259 | 0.13096658 | 115.7585134 | 192.5100034 |
| YMR019W | STB4 | 0.61406232 | 0.96815259 | 0.13101087 | 131.870676 | 222.5613997 |
| YPR005C | HAL1 | 0.59410446 | 0.96815259 | 0.13106197 | 140.3474998 | 238.366861 |
| YGL202W | ARO8 | 0.46932636 | 0.96815259 | 0.13108021 | 133.6276158 | 225.8267814 |
| YHL013C | OTU2 | 0.49687057 | 0.96815259 | 0.1311849 | 115.5457686 | 192.0740068 |
| YBR061C | TRM7 | 0.29701974 | 0.96815259 | 0.13130567 | 128.750532 | 216.6875766 |
| YBR161W | CSH1 | 0.41993789 | 0.96815259 | 0.13143502 | 132.4573135 | 223.5798933 |
| YLR362W | STE11 | 0.50420835 | 0.96815259 | 0.13201589 | 133.8532458 | 226.0801684 |

| | | | | | | |
|---|---|---|---|---|---|---|
| YLR294C | YLR294C | 0.34201415 | 0.96815259 | 0.13224549 | 116.2367498 | 193.173198 |
| YOR154W | SLP1 | 0.38963026 | 0.96815259 | 0.13238462 | 121.7152673 | 203.3691686 |
| YBR149W | ARA1 | 0.07460319 | 0.96815259 | 0.13247493 | 116.5798179 | 193.7721508 |
| YHR009C | YHR009C | 0.5120514 | 0.96815259 | 0.13251038 | 116.6301664 | 193.8597332 |
| YPR053C | YPR053C | 0.40178998 | 0.96815259 | 0.13255339 | 119.1815874 | 198.6120379 |
| YLR327C | TMA10 | 0.47987182 | 0.96815259 | 0.13287594 | 127.4089635 | 213.9035144 |
| YMR115W | FMP24 | 0.31838034 | 0.96815259 | 0.13291827 | 106.2565843 | 174.4334316 |
| YEL010W | YEL010W | 0.10989495 | 0.96815259 | 0.13301877 | 127.7133908 | 214.4458862 |
| YNL204C | SPS18 | 0.43116453 | 0.96815259 | 0.13313662 | 123.681034 | 206.9018976 |
| YFR024C-A | LSB3 | 0.54995671 | 0.96815259 | 0.1332776 | 131.9780339 | 222.3557814 |
| YER031C | YPT31 | 0.46597507 | 0.96815259 | 0.13339102 | 133.1770304 | 224.5723534 |
| YMR294W-A | YMR294W-A | 0.11019397 | 0.96815259 | 0.13343888 | 114.5157686 | 189.7487821 |
| YNR063W | YNR063W | 0.55094657 | 0.96815259 | 0.13360527 | 141.6229257 | 240.2908986 |
| YGL118C | YGL118C | 0.59393937 | 0.96815259 | 0.13361544 | 133.591256 | 225.3049582 |
| YFL010W-A | AUA1 | 0.49159199 | 0.96815259 | 0.13361739 | 118.3244851 | 196.8224698 |
| YOL053W | YOL053W | 0.50376648 | 0.96815259 | 0.1336174 | 134.2894568 | 226.6071895 |
| YGR207C | YGR207C | 0.32853477 | 0.96815259 | 0.13373509 | 132.3732072 | 223.0111038 |
| YLR019W | PSR2 | 0.44567325 | 0.96815259 | 0.13414715 | 118.8021401 | 197.6187327 |
| YJL108C | PRM10 | 0.48068632 | 0.96815259 | 0.1341481 | 137.4407279 | 232.3912612 |
| YGL154C | LYS5 | 0.03601005 | 0.96815259 | 0.13433459 | 108.1714614 | 177.7522579 |
| YNL121C | TOM70 | 0.51349773 | 0.96815259 | 0.13451419 | 132.905883 | 223.8653631 |
| YMR135W-A | YMR135W-A | 0.13347115 | 0.96815259 | 0.13457915 | 86.64206194 | 137.5425816 |
| YPR006C | ICL2 | 0.32890342 | 0.96815259 | 0.13478929 | 122.5083689 | 204.418192 |
| YLR012C | YLR012C | 0.54449554 | 0.96815259 | 0.13483229 | 121.6968739 | 202.8965422 |
| YPR042C | PUF2 | 0.58153653 | 0.96815259 | 0.13489404 | 120.2552494 | 200.1959485 |
| YGR289C | MAL11 | 0.56064016 | 0.96815259 | 0.13493484 | 122.1221301 | 203.6715503 |
| YOL032W | OPI10 | 0.41474021 | 0.96815259 | 0.13495473 | 128.6155657 | 215.7823333 |
| YDR519W | FPR2 | 0.50527625 | 0.96815259 | 0.13536943 | 129.6916754 | 217.7156929 |
| YPR083W | MDM36 | 0.52815267 | 0.96815259 | 0.13536949 | 124.1937688 | 207.458626 |
| YNR032C-A | HUB1 | 0.55154955 | 0.96815259 | 0.13560456 | 133.9865395 | 225.6862127 |
| YGR019W | UGA1 | 0.53587096 | 0.96815259 | 0.13570917 | 144.0380906 | 244.4199504 |
| YLL063C | AYT1 | 0.55408804 | 0.96815259 | 0.136053 | 131.7298212 | 221.3957106 |
| YOL059W | GPD2 | 0.49941805 | 0.96815259 | 0.13606736 | 130.9675968 | 219.9711094 |
| YOL141W | PPM2 | 0.55303725 | 0.96815259 | 0.13619022 | 139.3294845 | 235.5492935 |
| YGR040W | KSS1 | 0.48297655 | 0.96815259 | 0.13624184 | 117.5606912 | 194.9275473 |
| YEL042W | GDA1 | 0.42584789 | 0.96815259 | 0.13630435 | 116.9796164 | 193.8322835 |
| YMR058W | FET3 | 0.3974236 | 0.96815259 | 0.13638832 | 120.4318415 | 200.2578195 |
| YGR125W | YGR125W | 0.5119247 | 0.96815259 | 0.1364047 | 141.9098458 | 240.324884 |
| YMR022W | QRI8 | 0.42077192 | 0.96815259 | 0.1366124 | 118.3956326 | 196.4188818 |
| YPL096W | PNG1 | 0.00986662 | 0.51667384 | 0.13661627 | 122.285834 | 203.675863 |
| YMR307W | GAS1 | 0.47505302 | 0.96815259 | 0.13672666 | 84.92569577 | 133.9559192 |
| YLL048C | YBT1 | 0.14720184 | 0.96815259 | 0.13691308 | 119.530958 | 198.4831338 |
| YBL043W | ECM13 | 0.44985088 | 0.96815259 | 0.13692167 | 147.7162306 | 251.0648719 |
| YOR016C | ERP4 | 0.60983643 | 0.96815259 | 0.13696707 | 130.3819071 | 218.7173179 |
| YGR291C | YGR291C | 0.30692509 | 0.96815259 | 0.13710032 | 119.0607789 | 197.5724243 |
| YPR199C | ARR1 | 0.38127695 | 0.96815259 | 0.13732551 | 121.6777192 | 202.414341 |
| YEL008W | YEL008W | 0.43805578 | 0.96815259 | 0.13743853 | 130.7409516 | 219.3027351 |
| YBL103C | RTG3 | 0.28957142 | 0.96815259 | 0.13753626 | 121.2377107 | 201.5557085 |
| YGL224C | SDT1 | 0.5214541 | 0.96815259 | 0.13758913 | 129.7166307 | 217.364764 |
| YDR315C | IPK1 | 0.30134144 | 0.96815259 | 0.13769249 | 109.7886508 | 180.1680274 |
| YBR208C | DUR1 | 0.09434336 | 0.96815259 | 0.1377184 | 132.1625493 | 221.9047931 |
| YBR062C | YBR062C | 0.15275395 | 0.96815259 | 0.13780066 | 127.606099 | 213.3894152 |
| YMR133W | REC114 | 0.2640885 | 0.96815259 | 0.13780839 | 115.5688755 | 190.9310309 |
| YEL017W | GTT3 | 0.48549522 | 0.96815259 | 0.13788464 | 131.2663638 | 220.2030749 |
| YER027C | GAL83 | 0.35957737 | 0.96815259 | 0.13790581 | 115.8687627 | 191.4730649 |
| YDR452W | PPN1 | 0.31674481 | 0.96815259 | 0.13796498 | 138.7813125 | 234.2087983 |
| YHR048W | YHR048W | 0.47815035 | 0.96815259 | 0.13806553 | 127.9440505 | 213.9724767 |
| YER069W | ARG5 | 0.30002174 | 0.96815259 | 0.13809527 | 121.5258343 | 201.9931363 |
| YLR262C-A | TMA7 | 0.48120954 | 0.96815259 | 0.13817946 | 125.7196761 | 209.8022159 |
| YLL049W | LDB18 | 0.2732907 | 0.96815259 | 0.13825805 | 109.1683779 | 178.9095512 |
| YGR086C | PIL1 | 0.46937189 | 0.96815259 | 0.13826735 | 114.0412306 | 187.9988242 |
| YLR374C | YLR374C | 0.05448822 | 0.96815259 | 0.13829786 | 101.3662408 | 164.3465252 |
| YIL001W | YIL001W | 0.57814607 | 0.96815259 | 0.13831691 | 136.440259 | 229.7782389 |
| YGL062W | PYC1 | 0.51421288 | 0.96815259 | 0.13835993 | 136.2423722 | 229.4013518 |

| | | | | | | |
|---|---|---|---|---|---|---|
| YFR008W | FAR7 | 0.04515832 | 0.96815259 | 0.13850228 | 123.6851033 | 205.9486485 |
| YBR047W | FMP23 | 0.14878972 | 0.96815259 | 0.13857165 | 135.7443833 | 228.4343746 |
| YGR132C | PHB1 | 0.49217716 | 0.96815259 | 0.13861412 | 133.8557808 | 224.9033369 |
| YPL189W | GUP2 | 0.54991784 | 0.96815259 | 0.13879592 | 149.2972162 | 253.6787789 |
| YLR043C | TRX1 | 0.3825948 | 0.96815259 | 0.13888858 | 128.1290697 | 214.1702674 |
| YLR360W | VPS38 | 0.2308382 | 0.96815259 | 0.13898423 | 104.3178352 | 169.7301969 |
| YLR363C | NMD4 | 0.57370491 | 0.96815259 | 0.13936483 | 135.4407289 | 227.7258323 |
| YLR107W | REX3 | 0.335253 | 0.96815259 | 0.13941577 | 115.9467103 | 191.3480933 |
| YDR289C | RTT103 | 0.55037366 | 0.96815259 | 0.13977419 | 134.363125 | 225.6421164 |
| YMR254C | YMR254C | 0.16508477 | 0.96815259 | 0.13982973 | 119.7866435 | 198.4378571 |
| YER092W | IES5 | 0.40977157 | 0.96815259 | 0.14021111 | 20.5286402 | 13.19090227 |
| YIL015C-A | YIL015C-A | 0.46568086 | 0.96815259 | 0.14036744 | 120.8718835 | 200.366225 |
| YPL147W | PXA1 | 0.51282268 | 0.96815259 | 0.14042909 | 133.9536215 | 224.7608598 |
| YNL336W | COS1 | 0.11959183 | 0.96815259 | 0.14051309 | 113.6865073 | 186.9348927 |
| YPR154W | PIN3 | 0.4581788 | 0.96815259 | 0.1405694 | 122.7574503 | 203.8478287 |
| YBR019C | GAL10 | 0.33712072 | 0.96815259 | 0.14063122 | 132.0730959 | 221.2163015 |
| YEL043W | YEL043W | 0.39769065 | 0.96815259 | 0.14066238 | 124.0422059 | 206.2280563 |
| YBR001C | NTH2 | 0.39016917 | 0.96815259 | 0.14077504 | 134.6732125 | 226.0414018 |
| YFL018C | LPD1 | 0.60743086 | 0.96815259 | 0.14090474 | 108.4364425 | 177.0700821 |
| YPL248C | GAL4 | 0.56032427 | 0.96815259 | 0.14102643 | 136.3147386 | 229.0588629 |
| YHR153C | SPO16 | 0.1757967 | 0.96815259 | 0.14105401 | 125.617812 | 209.0974221 |
| YJL107C | YJL107C | 0.58258697 | 0.96815259 | 0.14117766 | 131.0954085 | 219.294446 |
| YER090W | TRP2 | 0.37647342 | 0.96815259 | 0.14131991 | 112.3781226 | 184.3494526 |
| YDR031W | MIC14 | 0.551729 | 0.96815259 | 0.14138252 | 128.5079781 | 214.4305747 |
| YOR064C | YNG1 | 0.53421005 | 0.96815259 | 0.14147858 | 140.5432769 | 236.866781 |
| YNL098C | RAS2 | 0.52631992 | 0.96815259 | 0.14153017 | 133.6961996 | 224.0834341 |
| YNL044W | YIP3 | 0.21183042 | 0.96815259 | 0.14156065 | 124.2238673 | 206.4061129 |
| YNR064C | YNR064C | 0.61893095 | 0.96815259 | 0.14158496 | 116.2628793 | 191.5495064 |
| YER137C | YER137C | 0.51397846 | 0.96815259 | 0.14170345 | 128.6119411 | 214.5670608 |
| YLR461W | PAU4 | 0.5001719 | 0.96815259 | 0.14184269 | 117.5752124 | 193.9516812 |
| YER065C | ICL1 | 0.20461991 | 0.96815259 | 0.14209287 | 117.8081297 | 194.3414179 |
| YNR041C | COQ2 | 0.026148 | 0.87038384 | 0.14220384 | 81.92114178 | 127.3697228 |
| YDL040C | NAT1 | 0.03829805 | 0.96815259 | 0.14225845 | 122.4758154 | 203.0199395 |
| YOR107W | RGS2 | 0.59725777 | 0.96815259 | 0.1422658 | 122.7321624 | 203.4968714 |
| YLR257W | YLR257W | 0.55603494 | 0.96815259 | 0.14228521 | 142.0797484 | 239.5888235 |
| YOR338W | YOR338W | 0.50342805 | 0.96815259 | 0.14257204 | 144.7528444 | 244.5244674 |
| YNL187W | YNL187W | 0.42555168 | 0.96815259 | 0.14261836 | 129.1991779 | 215.498792 |
| YNL145W | MFA2 | 0.38870659 | 0.96815259 | 0.14266301 | 125.4717968 | 208.5368846 |
| YDL082W | RPL13A | 0.04856981 | 0.96815259 | 0.14287663 | 105.4669187 | 171.1769399 |
| YPR007C | REC8 | 0.59758103 | 0.96815259 | 0.14291439 | 121.2502514 | 200.6160295 |
| YPL110C | GDE1 | 0.58950434 | 0.96815259 | 0.14292789 | 137.0163308 | 230.0272755 |
| YMR282C | AEP2 | 0.03895002 | 0.96815259 | 0.14295713 | 88.24617349 | 139.0349941 |
| YAR029W | YAR029W | 0.48418697 | 0.96815259 | 0.14297127 | 141.9699352 | 239.2610978 |
| YML090W | YML090W | 0.01214207 | 0.58582063 | 0.14314731 | 102.5710211 | 165.7257973 |
| YDR046C | BAP3 | 0.53530023 | 0.96815259 | 0.14316267 | 117.6700468 | 193.8922349 |
| YMR264W | CUE1 | 0.39390741 | 0.96815259 | 0.14348277 | 109.9844977 | 179.4965263 |
| YML062C | MFT1 | 0.26725942 | 0.96815259 | 0.14349892 | 102.4428025 | 165.4236257 |
| YPL223C | GRE1 | 0.51966586 | 0.96815259 | 0.14363782 | 129.3912417 | 215.6745555 |
| YML047C | PRM6 | 0.46748566 | 0.96815259 | 0.14371468 | 101.2514003 | 163.1622729 |
| YBR199W | KTR4 | 0.2829531 | 0.96815259 | 0.14373929 | 130.1136389 | 217.0041097 |
| YLR394W | CST9 | 0.55439527 | 0.96815259 | 0.14404719 | 106.3294215 | 172.576437 |
| YNL254C | RTC4 | 0.4704847 | 0.96815259 | 0.1440852 | 127.8784101 | 212.7720592 |
| YNL101W | AVT4 | 0.46460387 | 0.96815259 | 0.14415633 | 139.2917741 | 234.0524304 |
| YGR130C | YGR130C | 0.50314676 | 0.96815259 | 0.14425198 | 133.1571374 | 222.5903429 |
| YJL198W | PHO90 | 0.56885346 | 0.96815259 | 0.1443811 | 143.2217063 | 241.3439772 |
| YOL114C | YOL114C | 0.08545313 | 0.96815259 | 0.14449481 | 119.7435653 | 197.5221032 |
| YGL090W | LIF1 | 0.53377783 | 0.96815259 | 0.14459187 | 135.7012995 | 227.2759418 |
| YJR034W | PET191 | 0.49052375 | 0.96815259 | 0.1446063 | 127.3812469 | 211.7512228 |
| YPL270W | MDL2 | 0.464131 | 0.96815259 | 0.14487648 | 118.1799034 | 194.5365419 |
| YDR504C | SPG3 | 0.43699101 | 0.96815259 | 0.14496958 | 134.0482598 | 224.1243437 |
| YGR008C | STF2 | 0.44599457 | 0.96815259 | 0.14498461 | 128.6887943 | 214.1228764 |
| YNL305C | YNL305C | 0.22226728 | 0.96815259 | 0.14511021 | 97.90353549 | 156.6664979 |
| YDR271C | YDR271C | 0.54016542 | 0.96815259 | 0.1451485 | 118.8165442 | 195.6755662 |
| YBL046W | PSY4 | 0.4439904 | 0.96815259 | 0.1452813 | 133.9204542 | 223.8300859 |

| | | | | | | |
|---|---|---|---|---|---|---|
| YBR216C | YBP1 | 0.49549875 | 0.96815259 | 0.14532583 | 132.2240483 | 220.6572474 |
| YGL237C | HAP2 | 0.2090831 | 0.96815259 | 0.14541493 | 92.59632361 | 146.7106394 |
| YJR024C | YJR024C | 0.61079592 | 0.96815259 | 0.14570691 | 148.1604164 | 250.3203647 |
| YGR031W | YGR031W | 0.48408937 | 0.96815259 | 0.14574216 | 130.9678906 | 218.2391699 |
| YJL055W | YJL055W | 0.30198161 | 0.96815259 | 0.1461191 | 128.1417618 | 212.899161 |
| YER119C-A | YER119C-A | 0.29025555 | 0.96815259 | 0.14619002 | 104.6476847 | 169.0552176 |
| YMR243C | ZRC1 | 0.27852914 | 0.96815259 | 0.14623189 | 108.0237309 | 175.3461713 |
| YBL017C | PEP1 | 0.35093713 | 0.96815259 | 0.14628571 | 132.4345454 | 220.8780683 |
| YFL051C | YFL051C | 0.39357481 | 0.96815259 | 0.14633323 | 123.5723596 | 204.3360032 |
| YHR018C | ARG4 | 0.53692215 | 0.96815259 | 0.14658054 | 128.4929198 | 213.4716607 |
| YLR028C | ADE16 | 0.45085427 | 0.96815259 | 0.14661585 | 115.3748956 | 188.9919646 |
| YNL008C | ASI3 | 0.52359703 | 0.96815259 | 0.14684134 | 128.2882255 | 213.0430738 |
| YOR022C | YOR022C | 0.56411376 | 0.96815259 | 0.14706148 | 129.3674089 | 215.0170091 |
| YDR490C | PKH1 | 0.11440622 | 0.96815259 | 0.14707949 | 129.9589274 | 216.1173393 |
| YGL260W | YGL260W | 0.49187158 | 0.96815259 | 0.14729849 | 135.1699846 | 225.8000242 |
| YNL323W | LEM3 | 0.35980955 | 0.96815259 | 0.14730353 | 129.4113993 | 215.055735 |
| YGL161C | YIP5 | 0.37943282 | 0.96815259 | 0.14738759 | 128.8187169 | 213.9349554 |
| YML066C | SMA2 | 0.58614359 | 0.96815259 | 0.1480668 | 114.7245614 | 187.5188559 |
| YPL253C | VIK1 | 0.50226736 | 0.96815259 | 0.14812238 | 135.7339853 | 226.7047043 |
| YPL137C | GIP3 | 0.40583733 | 0.96815259 | 0.14817171 | 127.7937942 | 211.8824159 |
| YAR044W | OSH1 | 0.31338325 | 0.96815259 | 0.1486196 | 134.61109 | 224.5207596 |
| YJL103C | GSM1 | 0.53960286 | 0.96815259 | 0.14899087 | 122.9222801 | 202.6472871 |
| YOR345C | YOR345C | 0.3694478 | 0.96815259 | 0.14904119 | 124.0398877 | 204.7233175 |
| YGL059W | YGL059W | 0.5227045 | 0.96815259 | 0.14926279 | 146.236503 | 246.0942959 |
| YGR049W | SCM4 | 0.47413019 | 0.96815259 | 0.14931825 | 140.0104736 | 234.4688998 |
| YNR050C | LYS9 | 0.04534058 | 0.96815259 | 0.14934432 | 107.2357036 | 173.3186534 |
| YGL085W | YGL085W | 0.58009156 | 0.96815259 | 0.14940414 | 116.7691697 | 191.0938576 |
| YDR101C | ARX1 | 0.23056618 | 0.96815259 | 0.14946405 | 106.7091317 | 172.3148246 |
| YOR208W | PTP2 | 0.60740739 | 0.96815259 | 0.1496186 | 124.1395907 | 204.8059272 |
| YGR001C | YGR001C | 0.33555867 | 0.96815259 | 0.14971641 | 136.0878251 | 227.0793908 |
| YIL151C | YIL151C | 0.3411383 | 0.96815259 | 0.14973806 | 125.7251311 | 207.7425667 |
| YIL148W | RPL40A | 0.33688582 | 0.96815259 | 0.15044219 | 122.190681 | 201.0225017 |
| YDR133C | YDR133C | 0.56672883 | 0.96815259 | 0.15046282 | 148.0110369 | 249.1900259 |
| YNR022C | MRPL50 | 0.43175981 | 0.96815259 | 0.15051888 | 127.8865505 | 211.6351518 |
| YFL047W | RGD2 | 0.39749807 | 0.96815259 | 0.1506129 | 129.7708851 | 215.133785 |
| YLR083C | EMP70 | 0.41203362 | 0.96815259 | 0.15074179 | 133.78251 | 222.5949098 |
| YCR061W | YCR061W | 0.43244007 | 0.96815259 | 0.15083216 | 118.3267712 | 193.7440455 |
| YER184C | YER184C | 0.49059468 | 0.96815259 | 0.15086789 | 130.8749237 | 217.1478507 |
| YPR147C | YPR147C | 0.38356352 | 0.96815259 | 0.15102751 | 118.6506107 | 194.3132277 |
| YLR176C | RFX1 | 0.25745581 | 0.96815259 | 0.15112901 | 131.0847575 | 217.4925629 |
| YIL102C | YIL102C | 0.42747508 | 0.96815259 | 0.15132766 | 118.9468695 | 194.8121882 |
| YAL037W | YAL037W | 0.33671996 | 0.96815259 | 0.1513949 | 141.4782092 | 236.8352809 |
| YBL005W | PDR3 | 0.1794849 | 0.96815259 | 0.15148871 | 132.2273947 | 219.5598892 |
| YOL082W | ATG19 | 0.41041867 | 0.96815259 | 0.15166377 | 125.1556987 | 206.3353751 |
| YLR173W | YLR173W | 0.22663999 | 0.96815259 | 0.1517128 | 117.0873406 | 191.2740289 |
| YER176W | ECM32 | 0.07352258 | 0.96815259 | 0.15172235 | 115.8979232 | 189.0533074 |
| YLR233C | EST1 | 0.02041227 | 0.78250728 | 0.15173268 | 120.1825594 | 197.0450012 |
| YJR037W | YJR037W | 0.55092524 | 0.96815259 | 0.15182584 | 139.7387512 | 233.5129272 |
| YHL034C | SBP1 | 0.52070449 | 0.96815259 | 0.15222748 | 127.0907815 | 209.8445777 |
| YIR023W | DAL81 | 0.60067851 | 0.96815259 | 0.15239884 | 128.9897715 | 213.3567053 |
| YML109W | ZDS2 | 0.47494849 | 0.96815259 | 0.15246892 | 126.4230531 | 208.5556103 |
| YDL104C | QRI7 | 0.62882014 | 0.96815259 | 0.15294116 | 81.01906405 | 123.7640231 |
| YLR064W | YLR064W | 0.34192054 | 0.96815259 | 0.15308836 | 118.5806159 | 193.8136023 |
| YOL064C | MET22 | 0.38074197 | 0.96815259 | 0.15312702 | 109.8223645 | 177.4670278 |
| YBR045C | GIP1 | 0.08798407 | 0.96815259 | 0.15316864 | 129.8809029 | 214.8813749 |
| YER084W | YER084W | 0.34030947 | 0.96815259 | 0.15320648 | 135.3062915 | 224.9963641 |
| YBR182C | SMP1 | 0.34352271 | 0.96815259 | 0.15358817 | 134.1831725 | 222.8326907 |
| YBR018C | GAL7 | 0.14223358 | 0.96815259 | 0.15378366 | 133.0494868 | 220.6826469 |
| YOL068C | HST1 | 0.5409826 | 0.96815259 | 0.15392561 | 125.465678 | 206.5086491 |
| YJL065C | DLS1 | 0.50617829 | 0.96815259 | 0.15392815 | 132.30278 | 219.2636943 |
| YGR101W | PCP1 | 0.76088373 | 0.97135148 | 0.15409735 | 48.96238102 | 63.75108729 |
| YMR223W | UBP8 | 0.2695585 | 0.96815259 | 0.15424411 | 103.2001749 | 164.9124355 |
| YAL059W | ECM1 | 0.46335675 | 0.96815259 | 0.15446838 | 120.0745162 | 196.353543 |
| YNL072W | RNH201 | 0.51339875 | 0.96815259 | 0.15451718 | 139.3104801 | 232.2319871 |

| | | | | | | |
|---|---|---|---|---|---|---|
| YPR091C | YPR091C | 0.48144967 | 0.96815259 | 0.15458298 | 125.6695303 | 206.7712453 |
| YLR287C-A | RPS30A | 0.29205257 | 0.96815259 | 0.15467884 | 104.9111078 | 168.0265531 |
| YPL163C | SVS1 | 0.54939093 | 0.96815259 | 0.15488587 | 131.8377171 | 218.2245572 |
| YMR194W | RPL36A | 0.29503701 | 0.96815259 | 0.15547898 | 108.4550236 | 174.4949053 |
| YNR015W | SMM1 | 0.2933085 | 0.96815259 | 0.15555256 | 116.210532 | 188.9506349 |
| YPR017C | DSS4 | 0.50298005 | 0.96815259 | 0.15560303 | 120.0776519 | 196.1562091 |
| YOR039W | CKB2 | 0.37277106 | 0.96815259 | 0.15595601 | 101.8148425 | 162.021365 |
| YGR023W | MTL1 | 0.4784659 | 0.96815259 | 0.15650818 | 137.032928 | 227.626385 |
| YBR220C | YBR220C | 0.22509586 | 0.96815259 | 0.15653915 | 130.7078931 | 215.8206682 |
| YNR007C | ATG3 | 0.21157081 | 0.96815259 | 0.15654818 | 112.7883863 | 182.3878896 |
| YOR240W | YOR240W | 0.27902191 | 0.96815259 | 0.15656237 | 122.1713314 | 199.8904485 |
| YNL053W | MSG5 | 0.52775135 | 0.96815259 | 0.15667383 | 124.7866018 | 204.7496145 |
| YMR289W | ABZ2 | 0.50687383 | 0.96815259 | 0.15682231 | 128.20505 | 211.100584 |
| YOL153C | YOL153C | 0.49423531 | 0.96815259 | 0.15685392 | 124.6910427 | 204.539087 |
| YOL039W | RPP2A | 0.45129546 | 0.96815259 | 0.15691606 | 118.4234458 | 192.8349468 |
| YPR022C | YPR022C | 0.39071462 | 0.96815259 | 0.15698051 | 120.0211764 | 195.8041787 |
| YJL136C | RPS21B | 0.44493781 | 0.96815259 | 0.15713927 | 125.9555462 | 206.847084 |
| YBR008C | FLR1 | 0.47352427 | 0.96815259 | 0.15715021 | 137.272787 | 227.9589043 |
| YNR073C | YNR073C | 0.25517368 | 0.96815259 | 0.15731518 | 128.512142 | 211.585245 |
| YEL049W | PAU2 | 0.04509828 | 0.96815259 | 0.15756614 | 119.6039819 | 194.9209776 |
| YPR058W | YMC1 | 0.42582797 | 0.96815259 | 0.15765645 | 123.6257624 | 202.4079577 |
| YDR421W | ARO80 | 0.5350215 | 0.96815259 | 0.15770409 | 145.961941 | 244.0704611 |
| YDR445C | YDR445C | 0.31555782 | 0.96815259 | 0.15785532 | 114.2709578 | 184.9197472 |
| YIL074C | SER33 | 0.55099454 | 0.96815259 | 0.15810594 | 151.9173121 | 255.1090175 |
| YBR172C | SMY2 | 0.48277395 | 0.96815259 | 0.15864463 | 113.8529653 | 183.998584 |
| YDR533C | HSP31 | 0.4784951 | 0.96815259 | 0.15872123 | 138.0929765 | 229.2077469 |
| YDL181W | INH1 | 0.46991529 | 0.96815259 | 0.15896168 | 114.421864 | 185.0031635 |
| YGL170C | SPO74 | 0.15378016 | 0.96815259 | 0.15896188 | 133.6210964 | 220.8217812 |
| YMR299C | DYN3 | 0.23785752 | 0.96815259 | 0.15908728 | 114.7783261 | 185.6456973 |
| YGL051W | MST27 | 0.48924363 | 0.96815259 | 0.15911082 | 143.8476414 | 239.8740543 |
| YOR188W | MSB1 | 0.48832978 | 0.96815259 | 0.1591684 | 138.6959679 | 230.25263 |
| YEL028W | YEL028W | 0.25146041 | 0.96815259 | 0.15917265 | 124.0147726 | 202.8621983 |
| YOL056W | GPM3 | 0.41892373 | 0.96815259 | 0.15923512 | 126.5112013 | 207.5084217 |
| YGR108W | CLB1 | 0.46681123 | 0.96815259 | 0.15944821 | 136.592317 | 226.2778912 |
| YLR169W | YLR169W | 0.36305271 | 0.96815259 | 0.15956643 | 132.1309219 | 217.9334094 |
| YGL141W | HUL5 | 0.53284727 | 0.96815259 | 0.15971623 | 137.5895346 | 228.0903347 |
| YDL232W | OST4 | 0.36802047 | 0.96815259 | 0.15991015 | 108.9487246 | 174.6224677 |
| YOR356W | YOR356W | 0.49688272 | 0.96815259 | 0.1603309 | 134.5240436 | 222.2611936 |
| YLR375W | STP3 | 0.46879419 | 0.96815259 | 0.16065068 | 131.1211736 | 215.8554349 |
| YMR099C | YMR099C | 0.46285798 | 0.96815259 | 0.16075848 | 108.2531759 | 173.1729189 |
| YNL300W | YNL300W | 0.58079473 | 0.96815259 | 0.16095666 | 117.4402302 | 190.2770698 |
| YLR143W | YLR143W | 0.24527505 | 0.96815259 | 0.16132356 | 110.522845 | 177.3060909 |
| YLL032C | YLL032C | 0.45449382 | 0.96815259 | 0.16134797 | 132.9278879 | 219.1012296 |
| YMR018W | YMR018W | 0.3368008 | 0.96815259 | 0.16137827 | 114.1508606 | 184.0648261 |
| YGL214W | YGL214W | 0.16095904 | 0.96815259 | 0.16166412 | 122.1171784 | 198.8758366 |
| YGL180W | ATG1 | 0.34920177 | 0.96815259 | 0.16203595 | 142.7863987 | 237.3703595 |
| YOR032C | HMS1 | 0.44549991 | 0.96815259 | 0.16224051 | 120.9893136 | 196.6684426 |
| YLR112W | YLR112W | 0.3650705 | 0.96815259 | 0.16238027 | 131.1868404 | 215.6682228 |
| YKL053W | YKL053W | 0.38292723 | 0.96815259 | 0.16249406 | 112.4589064 | 180.7084589 |
| YOR288C | MPD1 | 0.59076391 | 0.96815259 | 0.16253635 | 134.1864566 | 221.2364449 |
| YMR255W | GFD1 | 0.36095345 | 0.96815259 | 0.16257314 | 125.741638 | 205.474955 |
| YGR042W | YGR042W | 0.3285747 | 0.96815259 | 0.16265899 | 130.7194224 | 214.7462821 |
| YIL002C | INP51 | 0.3223591 | 0.96815259 | 0.16269181 | 138.5243329 | 229.3014775 |
| YGL050W | TYW3 | 0.40780679 | 0.96815259 | 0.16274612 | 134.3757034 | 221.5519462 |
| YAL049C | YAL049C | 0.12391098 | 0.96815259 | 0.16276156 | 132.0837832 | 217.2733068 |
| YOL003C | PFA4 | 0.43689094 | 0.96815259 | 0.16282841 | 131.8425195 | 216.8112273 |
| YOR034C | AKR2 | 0.38256594 | 0.96815259 | 0.16298962 | 124.2810574 | 202.6754727 |
| YJR116W | YJR116W | 0.25471271 | 0.96815259 | 0.16303117 | 98.45187929 | 154.4803553 |
| YAL045C | YAL045C | 0.15042376 | 0.96815259 | 0.16306621 | 136.6969074 | 225.8251339 |
| YMR322C | SNO4 | 0.31083374 | 0.96815259 | 0.16336296 | 127.6861837 | 208.961322 |
| YGL158W | RCK1 | 0.44348366 | 0.96815259 | 0.16338519 | 136.5197219 | 225.4374501 |
| YGR015C | YGR015C | 0.36236409 | 0.96815259 | 0.16342871 | 129.1144579 | 211.6141784 |
| YBL057C | PTH2 | 0.35366428 | 0.96815259 | 0.16352856 | 132.8368733 | 218.5409452 |
| YOR383C | FIT3 | 0.51614876 | 0.96815259 | 0.16380288 | 136.1304898 | 224.6364905 |

| | | | | | | |
|---|---|---|---|---|---|---|
| YDL078C | MDH3 | 0.36579249 | 0.96815259 | 0.16402718 | 133.9627523 | 220.5521291 |
| YER039C-A | YER039C-A | 0.3424087 | 0.96815259 | 0.16414833 | 131.9849133 | 216.8405213 |
| YOR047C | STD1 | 0.03221619 | 0.9284317 | 0.16464321 | 125.8644816 | 205.3334436 |
| YDR465C | RMT2 | 0.24027314 | 0.96815259 | 0.16497391 | 131.7917885 | 216.3323831 |
| YNL227C | JJJ1 | 0.34102029 | 0.96815259 | 0.16524309 | 101.2739324 | 159.3491671 |
| YNL334C | SNO2 | 0.43456625 | 0.96815259 | 0.16546905 | 111.7986937 | 178.9440091 |
| YEL038W | UTR4 | 0.43453113 | 0.96815259 | 0.16554428 | 136.9746915 | 225.8996215 |
| YBR022W | POA1 | 0.23084867 | 0.96815259 | 0.16558848 | 125.8034501 | 205.0503095 |
| YDR123C | INO2 | 0.07752628 | 0.96815259 | 0.1657518 | 109.2729016 | 174.1811834 |
| YGR151C | YGR151C | 0.47507976 | 0.96815259 | 0.16580806 | 133.4460682 | 219.2692832 |
| YJR094W-A | RPL43B | 0.19976816 | 0.96815259 | 0.16626837 | 113.9366023 | 182.789419 |
| YGR123C | PPT1 | 0.45000827 | 0.96815259 | 0.16642244 | 141.9584853 | 235.04028 |
| YMR319C | FET4 | 0.28644696 | 0.96815259 | 0.16642565 | 123.6640024 | 200.9089787 |
| YMR206W | YMR206W | 0.00644253 | 0.36400266 | 0.16681091 | 122.5773089 | 198.8126211 |
| YLR054C | OSW2 | 0.24166711 | 0.96815259 | 0.16685218 | 114.7968163 | 184.2897152 |
| YER010C | YER010C | 0.23943526 | 0.96815259 | 0.16709364 | 134.4218005 | 220.8594257 |
| YNL020C | ARK1 | 0.71367484 | 0.97135148 | 0.16734962 | 92.07458323 | 141.8093686 |
| YLR262C | YPT6 | 0.13640459 | 0.96815259 | 0.16742868 | 93.28359931 | 144.050786 |
| YGL249W | ZIP2 | 0.45114133 | 0.96815259 | 0.1676594 | 141.0691937 | 233.1596861 |
| YPL203W | TPK2 | 0.43128459 | 0.96815259 | 0.16777457 | 144.7536808 | 240.0129505 |
| YIL037C | PRM2 | 0.41181956 | 0.96815259 | 0.16801594 | 123.040017 | 199.4600761 |
| YDR525W | API2 | 0.27049701 | 0.96815259 | 0.16823011 | 126.3203052 | 205.541526 |
| YNL119W | NCS2 | 0.46052457 | 0.96815259 | 0.16838475 | 100.7618727 | 157.831269 |
| YNR051C | BRE5 | 0.45715823 | 0.96815259 | 0.1684429 | 115.4762046 | 185.2723483 |
| YGR209C | TRX2 | 0.44086123 | 0.96815259 | 0.16872926 | 124.8222616 | 202.6573487 |
| YLR303W | MET17 | 0.3081835 | 0.96815259 | 0.16898597 | 126.8265609 | 206.3506591 |
| YNL241C | ZWF1 | 0.26130573 | 0.96815259 | 0.16900007 | 108.4127264 | 171.9947411 |
| YLR325C | RPL38 | 0.07813897 | 0.96815259 | 0.1692936 | 107.8053244 | 170.8089897 |
| YGR154C | GTO1 | 0.35292515 | 0.96815259 | 0.16952645 | 134.7994226 | 221.1282795 |
| YLR131C | ACE2 | 0.1128425 | 0.96815259 | 0.16954083 | 116.0783271 | 186.1990759 |
| YER114C | BOI2 | 0.41664615 | 0.96815259 | 0.16958751 | 130.1113427 | 212.3711244 |
| YLR168C | YLR168C | 0.13565623 | 0.96815259 | 0.16967072 | 106.966239 | 169.1760363 |
| YEL048C | YEL048C | 0.41732236 | 0.96815259 | 0.16969287 | 132.4342969 | 216.6860301 |
| YFR056C | YFR056C | 0.45948334 | 0.96815259 | 0.16979937 | 138.3737346 | 227.7477481 |
| YLR413W | YLR413W | 0.29348545 | 0.96815259 | 0.17000409 | 133.0540794 | 217.7865839 |
| YOR003W | YSP3 | 0.52411322 | 0.96815259 | 0.17030803 | 142.5648864 | 235.4757979 |
| YGL251C | HFM1 | 0.44178787 | 0.96815259 | 0.17078404 | 141.5774967 | 233.5484552 |
| YNL001W | DOM34 | 0.22010166 | 0.96815259 | 0.17096873 | 101.8301962 | 159.3616447 |
| YHR035W | YHR035W | 0.4554115 | 0.96815259 | 0.1709747 | 126.9259088 | 206.1798784 |
| YLR111W | YLR111W | 0.41531019 | 0.96815259 | 0.17124613 | 108.0890245 | 170.9886253 |
| YLR258W | GSY2 | 0.26447076 | 0.96815259 | 0.17130564 | 120.9899161 | 195.0462524 |
| YOR049C | RSB1 | 0.38664035 | 0.96815259 | 0.17147941 | 129.4268269 | 210.7552848 |
| YBR148W | YSW1 | 0.17089643 | 0.96815259 | 0.17155431 | 129.3045823 | 210.51381 |
| YDR479C | PEX29 | 0.33733222 | 0.96815259 | 0.17185562 | 131.1959712 | 213.9884842 |
| YIR039C | YPS6 | 0.00864494 | 0.46401722 | 0.17191329 | 125.0572503 | 202.525578 |
| YOR328W | PDR10 | 0.41883562 | 0.96815259 | 0.17223363 | 140.8745952 | 231.9775203 |
| YBL029W | YBL029W | 0.42044838 | 0.96815259 | 0.17262626 | 141.2008256 | 232.5158356 |
| YBR104W | YMC2 | 0.2377381 | 0.96815259 | 0.1730791 | 122.8970993 | 198.2867724 |
| YOR234C | RPL33B | 0.27649759 | 0.96815259 | 0.17333466 | 124.3474216 | 200.9467732 |
| YPR029C | APL4 | 0.50399793 | 0.96815259 | 0.17379064 | 132.824574 | 216.6803453 |
| YPL116W | HOS3 | 0.29735979 | 0.96815259 | 0.17383645 | 136.2191514 | 223.0051649 |
| YPR196W | YPR196W | 0.40154644 | 0.96815259 | 0.17406931 | 127.6585541 | 206.9925637 |
| YPR074C | TKL1 | 0.42995962 | 0.96815259 | 0.1740884 | 118.1202555 | 189.1942143 |
| YCL029C | BIK1 | 0.35435542 | 0.96815259 | 0.17434828 | 113.8049223 | 181.0968631 |
| YER033C | ZRG8 | 0.28910462 | 0.96815259 | 0.17437776 | 137.5362195 | 225.3653922 |
| YBR125C | PTC4 | 0.20796512 | 0.96815259 | 0.17451758 | 137.0494036 | 224.432137 |
| YKL096W | CWP1 | 0.54768322 | 0.96815259 | 0.1746209 | 146.1459384 | 241.3843992 |
| YGL166W | CUP2 | 0.45452331 | 0.96815259 | 0.17494547 | 140.8028111 | 231.3579821 |
| YOR136W | IDH2 | 0.54909089 | 0.96815259 | 0.17496758 | 139.8158107 | 229.5126446 |
| YNL043C | YNL043C | 0.4295018 | 0.96815259 | 0.17528914 | 137.9915529 | 226.0516727 |
| YBR139W | YBR139W | 0.1492987 | 0.96815259 | 0.17551879 | 141.1893572 | 231.9764678 |
| YLR035C | MLH2 | 0.42159848 | 0.96815259 | 0.17563128 | 127.0488063 | 205.5752943 |
| YMR162C | DNF3 | 0.22677799 | 0.96815259 | 0.1756813 | 119.4831932 | 191.4517057 |
| YBR113W | YBR113W | 0.38033775 | 0.96815259 | 0.17571294 | 138.3548931 | 226.6536407 |

| | | | | | | |
|---|---|---|---|---|---|---|
| YBR066C | NRG2 | 0.1385985 | 0.96815259 | 0.17574362 | 132.4002639 | 215.539014 |
| YOL150C | YOL150C | 0.40533393 | 0.96815259 | 0.17580219 | 138.4247057 | 226.7679024 |
| YBR071W | YBR071W | 0.19956097 | 0.96815259 | 0.17636568 | 130.166398 | 211.2600549 |
| YPL036W | PMA2 | 0.4701538 | 0.96815259 | 0.1763979 | 138.8875074 | 227.5246446 |
| YPR068C | HOS1 | 0.29963929 | 0.96815259 | 0.17661081 | 124.9151943 | 201.4193584 |
| YER163C | YER163C | 0.34185696 | 0.96815259 | 0.17689487 | 134.3085378 | 218.892989 |
| YMR114C | YMR114C | 0.28968856 | 0.96815259 | 0.17692279 | 120.6267697 | 193.3628799 |
| YOR380W | RDR1 | 0.42293051 | 0.96815259 | 0.17713753 | 133.8924811 | 218.0733277 |
| YDR262W | YDR262W | 0.00592311 | 0.35324765 | 0.17744469 | 122.2135618 | 196.2297873 |
| YPR093C | ASR1 | 0.41821684 | 0.96815259 | 0.1776486 | 127.6726334 | 206.3778788 |
| YOL035C | YOL035C | 0.37740934 | 0.96815259 | 0.17794393 | 123.8096636 | 199.1181219 |
| YLL039C | UBI4 | 0.51696139 | 0.96815259 | 0.17813837 | 118.6499816 | 189.4572484 |
| YOR178C | GAC1 | 0.4409038 | 0.96815259 | 0.17840701 | 136.8940587 | 223.445831 |
| YEL052W | AFG1 | 0.04418148 | 0.96815259 | 0.17863245 | 128.325372 | 207.4194669 |
| YDR441C | APT2 | 0.27632405 | 0.96815259 | 0.17866541 | 131.4587182 | 213.2592278 |
| YGR004W | PEX31 | 0.47736023 | 0.96815259 | 0.17867276 | 131.4198207 | 213.1853435 |
| YBL082C | ALG3 | 0.19126995 | 0.96815259 | 0.17871326 | 131.7074117 | 213.7146285 |
| YGL010W | YGL010W | 0.19301369 | 0.96815259 | 0.17874374 | 131.6013026 | 213.5112104 |
| YJR097W | JJJ3 | 0.44309599 | 0.96815259 | 0.17917129 | 132.4913107 | 215.0950732 |
| YNR009W | NRM1 | 0.04440693 | 0.96815259 | 0.17923573 | 121.7215764 | 194.9912 |
| YJL130C | URA2 | 0.29088232 | 0.96815259 | 0.17929729 | 135.655767 | 220.9762136 |
| YGL229C | SAP4 | 0.45283846 | 0.96815259 | 0.17931564 | 140.4091501 | 229.8409785 |
| YDR430C | CYM1 | 0.30257435 | 0.96815259 | 0.17942992 | 132.6440302 | 215.3336776 |
| YGR012W | YGR012W | 0.29302105 | 0.96815259 | 0.1794676 | 127.3322155 | 205.4170527 |
| YBR105C | VID24 | 0.3505118 | 0.96815259 | 0.17956846 | 144.2278058 | 236.9199038 |
| YGL197W | MDS3 | 0.41460936 | 0.96815259 | 0.17960491 | 129.3221717 | 209.1049844 |
| YBR015C | MNN2 | 0.2807618 | 0.96815259 | 0.17968814 | 129.9586904 | 210.2775893 |
| YLR180W | SAM1 | 0.23851674 | 0.96815259 | 0.17968954 | 119.1034722 | 190.0255219 |
| YOR304C-A | YOR304C-A | 0.42286025 | 0.96815259 | 0.1800733 | 137.5301127 | 224.3340862 |
| YAL056W | GPB2 | 0.27209545 | 0.96815259 | 0.18008028 | 131.2482728 | 212.6132499 |
| YNL275W | BOR1 | 0.41803415 | 0.96815259 | 0.18010426 | 129.6644054 | 209.6540459 |
| YGL159W | YGL159W | 0.52740861 | 0.96815259 | 0.18018146 | 133.7078481 | 217.1837882 |
| YFL063W | YFL063W | 0.26857971 | 0.96815259 | 0.18044741 | 126.7692949 | 204.1913942 |
| YNL089C | YNL089C | 0.39613855 | 0.96815259 | 0.18078281 | 118.990744 | 189.6194399 |
| YOR239W | ABP140 | 0.43910012 | 0.96815259 | 0.18081605 | 138.458687 | 225.9334552 |
| YDR496C | PUF6 | 0.00101235 | 0.0820012 | 0.18083353 | 107.3877455 | 167.9634593 |
| YML118W | NGL3 | 0.43748832 | 0.96815259 | 0.18092118 | 126.1724916 | 202.9931409 |
| YNL197C | WHI3 | 0.17219665 | 0.96815259 | 0.18103064 | 103.7699565 | 161.1787091 |
| YHR094C | HXT1 | 0.43781437 | 0.96815259 | 0.18108878 | 135.7093633 | 220.7553979 |
| YMR276W | DSK2 | 0.21642146 | 0.96815259 | 0.18123036 | 122.3356527 | 195.7796552 |
| YOR031W | CRS5 | 0.38369525 | 0.96815259 | 0.18161604 | 129.4241755 | 208.9351486 |
| YPR079W | MRL1 | 0.39271071 | 0.96815259 | 0.18162209 | 123.7286082 | 198.3082462 |
| YFR054C | YFR054C | 0.42964455 | 0.96815259 | 0.18163122 | 144.4749378 | 237.0115768 |
| YOR120W | GCY1 | 0.38891674 | 0.96815259 | 0.18167834 | 130.0156667 | 210.0274956 |
| YOL079W | YOL079W | 0.39243385 | 0.96815259 | 0.18181951 | 124.6403602 | 199.9738862 |
| YGR134W | CAF130 | 0.27975854 | 0.96815259 | 0.18210355 | 136.0684044 | 221.2435181 |
| YOR069W | VPS5 | 0.31444377 | 0.96815259 | 0.18215551 | 113.7898417 | 179.6706693 |
| YAL068C | PAU8 | 0.27648854 | 0.96815259 | 0.18233268 | 136.9270658 | 222.8044314 |
| YFL048C | EMP47 | 0.27122476 | 0.96815259 | 0.18245365 | 122.8328036 | 196.4880974 |
| YFR022W | ROG3 | 0.36771505 | 0.96815259 | 0.18251209 | 131.2020709 | 212.0915842 |
| YDL080C | THI3 | 0.16562664 | 0.96815259 | 0.18271259 | 118.4143479 | 188.1985282 |
| YGL199C | YGL199C | 0.1284662 | 0.96815259 | 0.18280227 | 125.8841205 | 202.1182959 |
| YEL068C | YEL068C | 0.06797753 | 0.96815259 | 0.18280663 | 125.2287823 | 200.8948972 |
| YGL028C | SCW11 | 0.4478894 | 0.96815259 | 0.18334295 | 137.75866 | 224.1749673 |
| YPL072W | UBP16 | 0.43415806 | 0.96815259 | 0.18335895 | 128.7700294 | 207.4026463 |
| YOR025W | HST3 | 0.4491384 | 0.96815259 | 0.18386583 | 138.3033161 | 225.0974594 |
| YLR047C | FRE8 | 0.21271736 | 0.96815259 | 0.18386628 | 103.9089254 | 160.9301886 |
| YHR112C | YHR112C | 0.3693275 | 0.96815259 | 0.18389969 | 142.8774023 | 233.6249458 |
| YPL133C | RDS2 | 0.4553741 | 0.96815259 | 0.18396072 | 139.8553734 | 227.9760318 |
| YOR386W | PHR1 | 0.4086804 | 0.96815259 | 0.18404988 | 129.6961493 | 209.0067175 |
| YGL211W | NCS6 | 0.16233355 | 0.96815259 | 0.1842179 | 107.2869981 | 167.1694566 |
| YJR044C | VPS55 | 0.52520893 | 0.96815259 | 0.18424439 | 121.574714 | 193.820296 |
| YLL047W | YLL047W | 0.17603331 | 0.96815259 | 0.18425186 | 123.2224396 | 196.8930045 |
| YGR187C | HGH1 | 0.47822569 | 0.96815259 | 0.18439785 | 142.8960809 | 233.5705871 |

| | | | | | | |
|---|---|---|---|---|---|---|
| YFR055W | IRC7 | 0.44001605 | 0.96815259 | 0.18466914 | 136.2741754 | 221.1679842 |
| YBR214W | SDS24 | 0.2545427 | 0.96815259 | 0.18470285 | 134.0639426 | 217.0384731 |
| YJR088C | YJR088C | 0.54405181 | 0.96815259 | 0.18477559 | 145.799023 | 238.9187583 |
| YER052C | HOM3 | 0.16415301 | 0.96815259 | 0.18477994 | 107.4777055 | 167.424599 |
| YER144C | UBP5 | 0.10119642 | 0.96815259 | 0.1848321 | 125.9259508 | 201.8328491 |
| YER134C | YER134C | 0.41150224 | 0.96815259 | 0.18499817 | 138.7770502 | 225.7785022 |
| YLR416C | YLR416C | 0.22581672 | 0.96815259 | 0.18500738 | 126.8108223 | 203.452305 |
| YHR198C | FMP22 | 0.43600558 | 0.96815259 | 0.18517931 | 130.8308574 | 210.9214121 |
| YAL036C | RBG1 | 0.23997417 | 0.96815259 | 0.18529463 | 136.9240056 | 222.2683188 |
| YFR049W | YMR31 | 0.38792034 | 0.96815259 | 0.18546948 | 129.332682 | 208.0744104 |
| YPR153W | YPR153W | 0.35316185 | 0.96815259 | 0.18552428 | 121.6478732 | 193.7275908 |
| YOR303W | CPA1 | 0.33339608 | 0.96815259 | 0.18568858 | 136.7122419 | 221.8027003 |
| YKL130C | SHE2 | 0.39054676 | 0.96815259 | 0.18583655 | 135.5149316 | 219.5424657 |
| YPL172C | COX10 | 0.58633051 | 0.96815259 | 0.18586486 | 57.98015214 | 74.88622338 |
| YNL035C | YNL035C | 0.35322277 | 0.96815259 | 0.18591313 | 130.3240129 | 209.8444217 |
| YOR099W | KTR1 | 0.41997918 | 0.96815259 | 0.18608457 | 139.4032382 | 226.7521914 |
| YPR195C | YPR195C | 0.43984725 | 0.96815259 | 0.18612918 | 128.584006 | 206.5595242 |
| YFL015C | YFL015C | 0.32019985 | 0.96815259 | 0.18633647 | 126.3179893 | 202.2948568 |
| YGR109C | CLB6 | 0.41604513 | 0.96815259 | 0.18634955 | 141.4523089 | 230.5275484 |
| YLR062C | BUD28 | 0.08884959 | 0.96815259 | 0.18668025 | 122.9531599 | 195.9557705 |
| YOR237W | HES1 | 0.42440789 | 0.96815259 | 0.18691192 | 140.9079165 | 229.4112087 |
| YOR170W | YOR170W | 0.4384399 | 0.96815259 | 0.18704854 | 130.0729069 | 209.17263 |
| YCR031C | RPS14A | 0.26660043 | 0.96815259 | 0.18707808 | 118.1453058 | 186.9148555 |
| YMR313C | TGL3 | 0.36391534 | 0.96815259 | 0.18720814 | 115.9821067 | 182.8558375 |
| YFR043C | IRC6 | 0.39637944 | 0.96815259 | 0.18798911 | 139.9292697 | 227.3925217 |
| YGL067W | NPY1 | 0.41912419 | 0.96815259 | 0.18804338 | 139.9803443 | 227.4780898 |
| YPR009W | SUT2 | 0.4092164 | 0.96815259 | 0.18811097 | 122.1980987 | 194.2909042 |
| YLR185W | RPL37A | 0.04642751 | 0.96815259 | 0.18814457 | 85.56946336 | 125.9494238 |
| YHR033W | YHR033W | 0.3373346 | 0.96815259 | 0.18831645 | 140.7305743 | 228.8288417 |
| YOR013W | YOR013W | 0.43815934 | 0.96815259 | 0.18834741 | 128.4252317 | 205.8660867 |
| YOL115W | PAP2 | 0.36968072 | 0.96815259 | 0.1884878 | 113.0373664 | 177.1328912 |
| YFL031W | HAC1 | 0.35471467 | 0.96815259 | 0.18859115 | 124.8393028 | 199.1324257 |
| YGR138C | TPO2 | 0.44833209 | 0.96815259 | 0.18893882 | 147.9666847 | 242.2172936 |
| YDR440W | DOT1 | 0.2441191 | 0.96815259 | 0.18906363 | 123.0078982 | 195.6310947 |
| YER079W | YER079W | 0.35706941 | 0.96815259 | 0.18909331 | 131.4091564 | 211.2994151 |
| YMR292W | GOT1 | 0.24371015 | 0.96815259 | 0.18919017 | 114.9206566 | 180.5206386 |
| YPR171W | BSP1 | 0.48062592 | 0.96815259 | 0.18952199 | 133.7468447 | 215.5839102 |
| YBR269C | FMP21 | 0.44501634 | 0.96815259 | 0.18957783 | 121.1069242 | 191.9925027 |
| YDR459C | PFA5 | 0.33984747 | 0.96815259 | 0.18964475 | 127.0528372 | 203.0733898 |
| YPL244C | HUT1 | 0.36120549 | 0.96815259 | 0.18995365 | 138.7449855 | 224.8312908 |
| YDL169C | UGX2 | 0.43210786 | 0.96815259 | 0.19015649 | 146.6022332 | 239.4536807 |
| YPR021C | AGC1 | 0.41257832 | 0.96815259 | 0.19033739 | 122.9100337 | 195.2204213 |
| YGR021W | YGR021W | 0.30403827 | 0.96815259 | 0.19051607 | 125.8470355 | 200.6677809 |
| YGL226C-A | OST5 | 0.41611317 | 0.96815259 | 0.19060545 | 137.5127303 | 222.4156409 |
| YOR094W | ARF3 | 0.29591188 | 0.96815259 | 0.19070936 | 134.1286318 | 216.0835581 |
| YNL079C | TPM1 | 0.16717301 | 0.96815259 | 0.19082278 | 100.7208373 | 153.7366803 |
| YNL228W | YNL228W | 0.05168336 | 0.96815259 | 0.19091022 | 86.97942834 | 128.0846454 |
| YDR476C | YDR476C | 0.36198268 | 0.96815259 | 0.19131747 | 140.341228 | 227.5650664 |
| YML121W | GTR1 | 0.17695347 | 0.96815259 | 0.19141454 | 125.3336885 | 199.5491747 |
| YOR333C | YOR333C | 0.40143963 | 0.96815259 | 0.19191216 | 119.4101742 | 188.4089812 |
| YER187W | YER187W | 0.37095163 | 0.96815259 | 0.19195326 | 130.1342933 | 208.4088545 |
| YER098W | UBP9 | 0.22549197 | 0.96815259 | 0.19233818 | 116.4273916 | 182.7679253 |
| YJL094C | KHA1 | 0.00574844 | 0.34765911 | 0.19240489 | 126.4916354 | 201.5321303 |
| YGR124W | ASN2 | 0.41958821 | 0.96815259 | 0.19243398 | 142.7828371 | 231.9202682 |
| YJL106W | IME2 | 0.43979298 | 0.96815259 | 0.19252112 | 125.7843555 | 200.1917949 |
| YER030W | CHZ1 | 0.02949578 | 0.91703766 | 0.19258543 | 134.3727797 | 216.2030954 |
| YKR092C | SRP40 | 0.50276317 | 0.96815259 | 0.1926715 | 79.52220118 | 113.8568254 |
| YPR198W | SGE1 | 0.41646274 | 0.96815259 | 0.19313313 | 127.6164969 | 203.5002981 |
| YAR015W | ADE1 | 0.10593886 | 0.96815259 | 0.19319168 | 132.2725353 | 212.1762561 |
| YEL005C | VAB2 | 0.03430469 | 0.94810002 | 0.19326109 | 128.3962829 | 204.932175 |
| YOR134W | BAG7 | 0.46021662 | 0.96815259 | 0.19332994 | 130.2286676 | 208.3383966 |
| YPL158C | YPL158C | 0.29340902 | 0.96815259 | 0.19333071 | 115.2428293 | 180.3802364 |
| YDR540C | IRC4 | 0.31491197 | 0.96815259 | 0.19373256 | 119.8848415 | 188.9685512 |
| YOR011W | AUS1 | 0.37602683 | 0.96815259 | 0.19379277 | 134.5171161 | 216.2561729 |

| | | | | | | |
|---|---|---|---|---|---|---|
| YBR165W | UBS1 | 0.12156362 | 0.96815259 | 0.1939805 | 125.8564846 | 200.0650256 |
| YBR075W | YBR075W | 0.12527166 | 0.96815259 | 0.1940334 | 133.6315221 | 214.5608926 |
| YBR134W | YBR134W | 0.35845571 | 0.96815259 | 0.19476083 | 144.3975752 | 234.5160953 |
| YER020W | GPA2 | 0.13010829 | 0.96815259 | 0.19523264 | 118.4151504 | 185.9580296 |
| YAR014C | BUD14 | 0.23734913 | 0.96815259 | 0.19532731 | 117.4320498 | 184.1069744 |
| YFL042C | YFL042C | 0.19698135 | 0.96815259 | 0.19534954 | 116.2261637 | 181.8532586 |
| YOR255W | OSW1 | 0.45801236 | 0.96815259 | 0.19540802 | 141.6236998 | 229.2251791 |
| YDR431W | YDR431W | 0.17294496 | 0.96815259 | 0.19542512 | 133.5942932 | 214.2422183 |
| YPL257W | YPL257W | 0.48197095 | 0.96815259 | 0.19556791 | 138.8533147 | 224.0280347 |
| YNL326C | PFA3 | 0.47217437 | 0.96815259 | 0.19563338 | 111.447584 | 172.8873704 |
| YML128C | MSC1 | 0.2932508 | 0.96815259 | 0.19594169 | 104.4533937 | 159.7835925 |
| YJL170C | ASG7 | 0.42853667 | 0.96815259 | 0.19649575 | 141.3483063 | 228.5166139 |
| YIL110W | MNI1 | 0.35224795 | 0.96815259 | 0.19650683 | 108.484889 | 167.2036692 |
| YPR012W | YPR012W | 0.44495497 | 0.96815259 | 0.19696304 | 128.936938 | 205.2779207 |
| YNL031C | HHT2 | 0.2552457 | 0.96815259 | 0.19724121 | 128.9047889 | 205.1681287 |
| YER053C | PIC2 | 0.05324083 | 0.96815259 | 0.19730904 | 130.6316061 | 208.377584 |
| YLR441C | RPS1A | 0.3004978 | 0.96815259 | 0.19752518 | 111.228988 | 172.1407821 |
| YBR299W | MAL32 | 0.42362874 | 0.96815259 | 0.19760034 | 130.3328004 | 207.76796 |
| YDR428C | YDR428C | 0.34690151 | 0.96815259 | 0.1977257 | 135.5841685 | 217.542619 |
| YMR123W | PKR1 | 0.25172646 | 0.96815259 | 0.19774647 | 110.621424 | 170.9676653 |
| YOL048C | YOL048C | 0.33025116 | 0.96815259 | 0.19775951 | 124.7756886 | 197.3719447 |
| YMR269W | TMA23 | 0.17208891 | 0.96815259 | 0.19785859 | 94.8643565 | 141.5507389 |
| YNL224C | SQS1 | 0.3735856 | 0.96815259 | 0.1979681 | 123.2019336 | 194.3985491 |
| YHL028W | WSC4 | 0.55777372 | 0.96815259 | 0.19808373 | 148.0450608 | 240.7259138 |
| YLL051C | FRE6 | 0.18531139 | 0.96815259 | 0.19829121 | 119.814826 | 188.0216004 |
| YMR205C | PFK2 | 0.1748694 | 0.96815259 | 0.19835499 | 108.6506974 | 167.1820517 |
| YOR245C | DGA1 | 0.42334437 | 0.96815259 | 0.19843068 | 132.1243321 | 210.9616028 |
| YBR033W | EDS1 | 0.20942511 | 0.96815259 | 0.19850078 | 133.8421339 | 214.1538323 |
| YGL027C | CWH41 | 0.44925314 | 0.96815259 | 0.19854817 | 134.9685724 | 216.2468616 |
| YAR031W | PRM9 | 0.19076854 | 0.96815259 | 0.19881008 | 144.7413836 | 234.4324066 |
| YLL056C | YLL056C | 0.31617641 | 0.96815259 | 0.19887254 | 128.4407812 | 204.0103364 |
| YGL242C | YGL242C | 0.12702894 | 0.96815259 | 0.19896504 | 147.1766353 | 238.9479352 |
| YPR020W | ATP20 | 0.34492094 | 0.96815259 | 0.19898498 | 115.1734711 | 179.2383173 |
| YMR245W | YMR245W | 0.30737543 | 0.96815259 | 0.19906849 | 105.6077985 | 161.3773608 |
| YCR107W | AAD3 | 0.42084426 | 0.96815259 | 0.19908208 | 126.9866097 | 201.2598683 |
| YBL036C | YBL036C | 0.19978286 | 0.96815259 | 0.19910118 | 134.8553835 | 215.9366645 |
| YHR182W | YHR182W | 0.23508065 | 0.96815259 | 0.19936166 | 130.8257947 | 208.3723001 |
| YOL128C | YGK3 | 0.2503133 | 0.96815259 | 0.1993841 | 130.350697 | 207.4819264 |
| YBR121C | GRS1 | 0.28051069 | 0.96815259 | 0.19978652 | 131.1030959 | 208.8135614 |
| YNL316C | PHA2 | 0.01131645 | 0.56503293 | 0.2000796 | 120.1204107 | 188.2714568 |
| YER088C | DOT6 | 0.07670992 | 0.96815259 | 0.20033713 | 130.3142747 | 207.2433144 |
| YLR282C | YLR282C | 0.29753658 | 0.96815259 | 0.20075784 | 122.1148917 | 191.8709648 |
| YGR161C | RTS3 | 0.38654515 | 0.96815259 | 0.20076068 | 141.2445494 | 227.5593117 |
| YJR008W | YJR008W | 0.29457943 | 0.96815259 | 0.20116711 | 131.5850001 | 209.4653912 |
| YPR149W | NCE102 | 0.39891164 | 0.96815259 | 0.20209329 | 119.2169928 | 186.2254176 |
| YPL023C | MET12 | 0.42890612 | 0.96815259 | 0.20242103 | 140.2101854 | 225.3322475 |
| YCR071C | IMG2 | 0.35638053 | 0.96815259 | 0.20249767 | 71.4820006 | 97.09719338 |
| YMR237W | BCH1 | 0.45035656 | 0.96815259 | 0.20250605 | 127.3032115 | 201.2373915 |
| YBR186W | PCH2 | 0.19020689 | 0.96815259 | 0.2025775 | 144.2259629 | 232.7961816 |
| YLR030W | YLR030W | 0.15523334 | 0.96815259 | 0.20263962 | 120.6650329 | 188.8290914 |
| YGL117W | YGL117W | 0.43045983 | 0.96815259 | 0.20283279 | 139.5038387 | 223.9407328 |
| YOL014W | YOL014W | 0.37461812 | 0.96815259 | 0.20293857 | 133.8878763 | 213.4444849 |
| YOR230W | WTM1 | 0.5017118 | 0.96815259 | 0.20303854 | 139.9838716 | 224.7994507 |
| YNL115C | YNL115C | 0.14985869 | 0.96815259 | 0.20308948 | 125.3925315 | 197.568295 |
| YPL074W | YTA6 | 0.43114775 | 0.96815259 | 0.20314458 | 141.9032297 | 228.361273 |
| YLR370C | ARC18 | 0.06062942 | 0.96815259 | 0.20334505 | 111.8363621 | 172.2317387 |
| YNL333W | SNZ2 | 0.33096698 | 0.96815259 | 0.20344415 | 130.1665479 | 206.4113287 |
| YER151C | UBP3 | 0.10664263 | 0.96815259 | 0.20348442 | 98.59421378 | 147.5018392 |
| YER056C | FCY2 | 0.31636727 | 0.96815259 | 0.20351299 | 140.2793363 | 225.2657189 |
| YNL057W | YNL057W | 0.24894857 | 0.96815259 | 0.20370348 | 124.1393063 | 195.12029 |
| YBL059W | YBL059W | 0.06072999 | 0.96815259 | 0.203855 | 134.5051474 | 214.4319762 |
| YBL095W | YBL095W | 0.23123971 | 0.96815259 | 0.20407764 | 135.9769677 | 217.1379799 |
| YGR182C | YGR182C | 0.41266308 | 0.96815259 | 0.20441378 | 132.9364867 | 211.4053747 |
| YLR299W | ECM38 | 0.27206177 | 0.96815259 | 0.20455668 | 129.1965388 | 204.4024278 |

| | | | | | | |
|---|---|---|---|---|---|---|
| YBL032W | HEK2 | 0.2241373 | 0.96815259 | 0.2045681 | 143.0133159 | 230.17737 |
| YLR264W | RPS28B | 0.31657946 | 0.96815259 | 0.204607 | 111.0542616 | 170.5466487 |
| YOL073C | YOL073C | 0.41627875 | 0.96815259 | 0.20461487 | 126.6331881 | 199.6097447 |
| YAR047C | YAR047C | 0.25579469 | 0.96815259 | 0.20495888 | 126.9787896 | 200.1929068 |
| YHR066W | SSF1 | 0.31196188 | 0.96815259 | 0.20500904 | 112.3269473 | 172.8490158 |
| YMR008C | PLB1 | 0.29615188 | 0.96815259 | 0.205181 | 123.7817918 | 194.1887185 |
| YNL167C | SKO1 | 0.28668017 | 0.96815259 | 0.20523516 | 122.1817977 | 191.1940229 |
| YNR029C | YNR029C | 0.4650621 | 0.96815259 | 0.20523947 | 119.5684449 | 186.3177035 |
| YLR333C | RPS25B | 0.24795928 | 0.96815259 | 0.20563776 | 116.4947139 | 180.511937 |
| YPL038W | MET31 | 0.39014368 | 0.96815259 | 0.20612173 | 134.7008523 | 214.3911814 |
| YDR494W | RSM28 | 0.35391998 | 0.96815259 | 0.20627507 | 140.3452837 | 224.8941389 |
| YBR181C | RPS6B | 0.26969014 | 0.96815259 | 0.20658436 | 102.9397273 | 155.0538448 |
| YGL017W | ATE1 | 0.32938614 | 0.96815259 | 0.20671505 | 134.6246315 | 214.1427339 |
| YGR122C-A | YGR122C-A | 0.28713734 | 0.96815259 | 0.20713435 | 127.5410724 | 200.8523524 |
| YLR046C | YLR046C | 0.28955871 | 0.96815259 | 0.2071911 | 136.9933178 | 218.4765766 |
| YOR186W | YOR186W | 0.22807698 | 0.96815259 | 0.207419 | 128.219074 | 202.0662787 |
| YNL190W | YNL190W | 0.11017586 | 0.96815259 | 0.20759316 | 103.3597841 | 155.6568658 |
| YGL026C | TRP5 | 0.23857025 | 0.96815259 | 0.20784089 | 117.6163225 | 182.2099226 |
| YGR026W | YGR026W | 0.40928087 | 0.96815259 | 0.20798664 | 145.8260747 | 234.8127697 |
| YIR025W | MND2 | 0.29402135 | 0.96815259 | 0.20802046 | 130.117257 | 205.4998798 |
| YIL017C | VID28 | 0.31342056 | 0.96815259 | 0.20802414 | 122.6749962 | 191.6147198 |
| YPL220W | RPL1A | 0.45333761 | 0.96815259 | 0.20835208 | 140.3903345 | 224.6062519 |
| YPR075C | OPY2 | 0.38479773 | 0.96815259 | 0.20866658 | 121.7181599 | 189.7145737 |
| YPL247C | YPL247C | 0.41145807 | 0.96815259 | 0.20868028 | 146.4979367 | 235.9420031 |
| YBL052C | SAS3 | 0.29119341 | 0.96815259 | 0.20889446 | 134.4680756 | 213.4603859 |
| YOR133W | EFT1 | 0.3944121 | 0.96815259 | 0.20903142 | 136.0182059 | 216.3278294 |
| YER186C | YER186C | 0.14280158 | 0.96815259 | 0.21037145 | 131.387591 | 207.4488549 |
| YAL004W | YAL004W | 0.26729071 | 0.96815259 | 0.21049621 | 131.8205997 | 208.2343485 |
| YDR481C | PHO8 | 0.27481552 | 0.96815259 | 0.21052465 | 131.8201145 | 208.2283507 |
| YML041C | VPS71 | 0.24282394 | 0.96815259 | 0.21069898 | 112.7077325 | 172.5405083 |
| YDR375C | BCS1 | 0.04793053 | 0.96815259 | 0.21097033 | 103.2185295 | 154.7885794 |
| YNR067C | DSE4 | 0.37918557 | 0.96815259 | 0.21103836 | 136.3527903 | 216.592652 |
| YIL012W | YIL012W | 0.36285747 | 0.96815259 | 0.21110267 | 134.3146136 | 212.778654 |
| YGL083W | SCY1 | 0.33299565 | 0.96815259 | 0.21137945 | 148.7184806 | 239.6013701 |
| YFR034C | PHO4 | 0.370069 | 0.96815259 | 0.2114452 | 137.670349 | 218.9778757 |
| YLR151C | PCD1 | 0.36562808 | 0.96815259 | 0.21166207 | 134.6233027 | 213.2543801 |
| YGL248W | PDE1 | 0.36776086 | 0.96815259 | 0.21187781 | 146.520948 | 235.412346 |
| YHR081W | LRP1 | 0.14585556 | 0.96815259 | 0.21215322 | 95.64538401 | 140.4480741 |
| YJL038C | YJL038C | 0.36311548 | 0.96815259 | 0.21219217 | 135.7714305 | 215.3014349 |
| YBR090C-A | YBR090C-A | 0.23672815 | 0.96815259 | 0.21225534 | 136.7744146 | 217.1613191 |
| YPR140W | TAZ1 | 0.41961502 | 0.96815259 | 0.21238615 | 131.0587191 | 206.4745255 |
| YDR505C | PSP1 | 0.17470515 | 0.96815259 | 0.21242699 | 130.4177887 | 205.2714723 |
| YGL164C | YRB30 | 0.34688253 | 0.96815259 | 0.21289929 | 146.0961981 | 234.4370003 |
| YOR316C | COT1 | 0.39518326 | 0.96815259 | 0.21294366 | 147.5581184 | 237.1564572 |
| YML042W | CAT2 | 0.07651234 | 0.96815259 | 0.21296762 | 117.3425334 | 180.7810785 |
| YJL168C | SET2 | 0.45817543 | 0.96815259 | 0.21302572 | 130.2017645 | 204.7612355 |
| YOL138C | RTC1 | 0.28615113 | 0.96815259 | 0.21367362 | 120.4285699 | 186.4120543 |
| YPL140C | MKK2 | 0.45110262 | 0.96815259 | 0.21368945 | 140.0358543 | 222.9891473 |
| YFR048W | RMD8 | 0.44831261 | 0.96815259 | 0.21383458 | 144.1829877 | 230.7001735 |
| YBR137W | YBR137W | 0.33343461 | 0.96815259 | 0.21390365 | 137.0443262 | 217.3697071 |
| YER007C-A | TMA20 | 0.15293549 | 0.96815259 | 0.21397162 | 118.8734962 | 183.4574998 |
| YGL203C | KEX1 | 0.28585302 | 0.96815259 | 0.21399223 | 134.8612842 | 213.2810988 |
| YNL299W | TRF5 | 0.22067634 | 0.96815259 | 0.21403573 | 112.2824972 | 171.1496568 |
| YNL076W | MKS1 | 0.29806899 | 0.96815259 | 0.21407965 | 109.7133503 | 166.3487157 |
| YLR246W | ERF2 | 0.35494914 | 0.96815259 | 0.21418224 | 136.6728341 | 216.6267543 |
| YGR206W | MVB12 | 0.37717034 | 0.96815259 | 0.21435431 | 126.0748466 | 196.8240218 |
| YPL114W | YPL114W | 0.41842352 | 0.96815259 | 0.21493482 | 142.9324115 | 228.1700393 |
| YBR184W | YBR184W | 0.15389091 | 0.96815259 | 0.21563718 | 145.4618954 | 232.7633456 |
| YNR030W | ALG12 | 0.27802085 | 0.96815259 | 0.2157509 | 127.5888746 | 199.3985473 |
| YML013W | SEL1 | 0.20188999 | 0.96815259 | 0.21626037 | 104.2258122 | 155.7204966 |
| YML048W | GSF2 | 0.22740911 | 0.96815259 | 0.21655747 | 117.2578571 | 179.9802607 |
| YGR139W | YGR139W | 0.43248958 | 0.96815259 | 0.21693327 | 143.4785532 | 228.8310704 |
| YPR109W | YPR109W | 0.39545033 | 0.96815259 | 0.21702148 | 125.7664068 | 195.7709717 |
| YPL037C | EGD1 | 0.38137133 | 0.96815259 | 0.21702611 | 137.6381043 | 217.9183318 |

| | | | | | | |
|---|---|---|---|---|---|---|
| YOL047C | YOL047C | 0.41915742 | 0.96815259 | 0.21707188 | 140.5626021 | 223.3661645 |
| YDR035W | ARO3 | 0.41651469 | 0.96815259 | 0.21709425 | 137.670059 | 217.9657448 |
| YEL060C | PRB1 | 0.18942939 | 0.96815259 | 0.21756862 | 126.2411145 | 196.5586222 |
| YJR047C | ANB1 | 0.44309259 | 0.96815259 | 0.2185204 | 145.6316476 | 232.5637356 |
| YLR388W | RPS29A | 0.12375075 | 0.96815259 | 0.21877563 | 104.4115179 | 155.6165402 |
| YPR031W | NTO1 | 0.17585533 | 0.96815259 | 0.21921128 | 122.0410796 | 188.4287583 |
| YJR032W | CPR7 | 0.1095926 | 0.96815259 | 0.21937937 | 110.3473583 | 166.5825059 |
| YFR026C | YFR026C | 0.26876331 | 0.96815259 | 0.21938061 | 138.3227656 | 218.7740294 |
| YPL229W | YPL229W | 0.45468339 | 0.96815259 | 0.21983061 | 135.3618111 | 213.1694029 |
| YDR453C | TSA2 | 0.18324782 | 0.96815259 | 0.2200324 | 142.1952629 | 225.8819578 |
| YBR115C | LYS2 | 0.10373514 | 0.96815259 | 0.22006313 | 113.0598179 | 171.5205092 |
| YER170W | ADK2 | 0.23417952 | 0.96815259 | 0.22016341 | 137.9031203 | 217.8509495 |
| YPR192W | AQY1 | 0.33404838 | 0.96815259 | 0.22020286 | 132.0941223 | 207.006446 |
| YOL147C | PEX11 | 0.14678962 | 0.96815259 | 0.22026108 | 124.3096517 | 192.4730833 |
| YDR451C | YHP1 | 0.25258956 | 0.96815259 | 0.22052152 | 147.4699545 | 235.6349894 |
| YFL035C-B | YFL035C-B | 0.13814553 | 0.96815259 | 0.22065343 | 133.643307 | 209.8159671 |
| YNL014W | HEF3 | 0.37305053 | 0.96815259 | 0.22072133 | 127.00178 | 197.413179 |
| YNL143C | YNL143C | 0.27160456 | 0.96815259 | 0.22074778 | 116.7615703 | 178.3040051 |
| YPR123C | YPR123C | 0.3750257 | 0.96815259 | 0.220876 | 127.7663948 | 198.81197 |
| YHL035C | VMR1 | 0.42178166 | 0.96815259 | 0.22088298 | 133.5739787 | 209.645521 |
| YMR193C-A | YMR193C-A | 0.00011744 | 0.02654215 | 0.22107323 | 102.8391702 | 152.2716854 |
| YDR030C | RAD28 | 0.19893327 | 0.96815259 | 0.22108971 | 127.1490866 | 197.6220323 |
| YGR272C | YGR272C | 0.35341786 | 0.96815259 | 0.2210937 | 128.3558306 | 199.8726551 |
| YBR266C | YBR266C | 0.56853914 | 0.96815259 | 0.22175523 | 99.69270239 | 146.2794158 |
| YNL193W | YNL193W | 0.27244196 | 0.96815259 | 0.22177482 | 128.4725225 | 199.9683884 |
| YLR024C | UBR2 | 0.25719678 | 0.96815259 | 0.22234186 | 153.718863 | 246.9671661 |
| YMR244W | YMR244W | 0.19647219 | 0.96815259 | 0.22234867 | 126.3532882 | 195.9119217 |
| YOR079C | ATX2 | 0.33454342 | 0.96815259 | 0.22239311 | 141.4436299 | 224.0569505 |
| YOR006C | YOR006C | 0.42554026 | 0.96815259 | 0.22246503 | 128.0743495 | 199.1019484 |
| YPR026W | ATH1 | 0.37825819 | 0.96815259 | 0.22288636 | 138.2981098 | 218.1002483 |
| YFL049W | SWP82 | 0.04180077 | 0.96815259 | 0.22313947 | 135.5425677 | 212.9141034 |
| YGR080W | TWF1 | 0.17417939 | 0.96815259 | 0.2235278 | 138.2723201 | 217.93727 |
| YHR163W | SOL3 | 0.43182662 | 0.96815259 | 0.22361589 | 130.5914481 | 203.5918344 |
| YOR352W | YOR352W | 0.39561957 | 0.96815259 | 0.22363949 | 139.0704755 | 219.4063322 |
| YDR503C | LPP1 | 0.28246059 | 0.96815259 | 0.22379403 | 129.1112267 | 200.7983905 |
| YER108C | YER108C | 0.27254896 | 0.96815259 | 0.22424735 | 132.577551 | 207.1840894 |
| YMR194C-A | YMR194C-A | 0.26472371 | 0.96815259 | 0.22432033 | 128.2560101 | 199.1086263 |
| YNL037C | IDH1 | 0.27028843 | 0.96815259 | 0.22440586 | 109.9119536 | 164.8700983 |
| YEL023C | YEL023C | 0.23368793 | 0.96815259 | 0.22456742 | 130.5006637 | 203.2520716 |
| YGR043C | YGR043C | 0.27657019 | 0.96815259 | 0.22463045 | 135.8172803 | 213.1596215 |
| YNL246W | VPS75 | 0.18098376 | 0.96815259 | 0.22474184 | 123.8104706 | 190.7394161 |
| YER183C | FAU1 | 0.31721617 | 0.96815259 | 0.22492293 | 135.6573373 | 212.8088521 |
| YEL016C | NPP2 | 0.22945653 | 0.96815259 | 0.22534554 | 124.3350953 | 191.6100661 |
| YGL087C | MMS2 | 0.37494572 | 0.96815259 | 0.225472 | 140.5154556 | 221.7739772 |
| YJL186W | MNN5 | 0.30698846 | 0.96815259 | 0.22551792 | 139.3266474 | 219.5478782 |
| YER174C | GRX4 | 0.33497243 | 0.96815259 | 0.22554064 | 157.4343239 | 253.3260262 |
| YNL291C | MID1 | 0.21160524 | 0.96815259 | 0.22609676 | 123.3283017 | 189.5972397 |
| YBR069C | TAT1 | 0.23973421 | 0.96815259 | 0.2269627 | 139.8340213 | 220.2357306 |
| YFR038W | IRC5 | 0.26537255 | 0.96815259 | 0.22706424 | 135.0759299 | 211.340711 |
| YGR051C | YGR051C | 0.36292218 | 0.96815259 | 0.22726719 | 145.0555274 | 229.922601 |
| YOR264W | DSE3 | 0.40238057 | 0.96815259 | 0.22731302 | 141.8604351 | 223.9535346 |
| YOL098C | YOL098C | 0.26489331 | 0.96815259 | 0.22811608 | 131.2143915 | 203.9481559 |
| YOR088W | YOR088W | 0.33064668 | 0.96815259 | 0.22812671 | 127.427724 | 196.8817349 |
| YGR028W | MSP1 | 0.25260134 | 0.96815259 | 0.22829487 | 135.1021133 | 211.1691883 |
| YDR393W | SHE9 | 0.55369798 | 0.96815259 | 0.22850422 | 90.90730672 | 128.6805634 |
| YPR043W | RPL43A | 0.18588144 | 0.96815259 | 0.22863222 | 92.76798564 | 132.1289805 |
| YOR033C | EXO1 | 0.0208773 | 0.78250728 | 0.22904892 | 84.07745006 | 115.841041 |
| YPR071W | YPR071W | 0.28412952 | 0.96815259 | 0.22910806 | 137.9687945 | 216.3717341 |
| YPR184W | GDB1 | 0.30711611 | 0.96815259 | 0.22957163 | 127.1353285 | 196.0774877 |
| YNL292W | PUS4 | 0.28219187 | 0.96815259 | 0.22961145 | 132.9321167 | 206.8850158 |
| YML072C | TCB3 | 0.19545122 | 0.96815259 | 0.23037439 | 120.7593635 | 184.0385466 |
| YDR482C | CWC21 | 0.23699528 | 0.96815259 | 0.23045711 | 128.288185 | 198.0697252 |
| YOR251C | YOR251C | 0.28584048 | 0.96815259 | 0.23088451 | 130.4083823 | 201.9486927 |
| YFL052W | YFL052W | 0.31447517 | 0.96815259 | 0.23137393 | 142.9552561 | 225.2688695 |

| | | | | | | |
|---|---|---|---|---|---|---|
| YGL054C | ERV14 | 0.16426342 | 0.96815259 | 0.23148936 | 122.4803132 | 187.0495403 |
| YDR466W | PKH3 | 0.12514142 | 0.96815259 | 0.23180799 | 132.2640343 | 205.2452822 |
| YNL097C | PHO23 | 0.35852657 | 0.96815259 | 0.23190954 | 127.4705134 | 196.2841631 |
| YDR517W | GRH1 | 0.28711687 | 0.96815259 | 0.23210186 | 136.4095587 | 212.9266696 |
| YLR073C | YLR073C | 0.2613295 | 0.96815259 | 0.23230833 | 136.9020155 | 213.8084394 |
| YOL055C | THI20 | 0.27532917 | 0.96815259 | 0.23233395 | 141.1170645 | 221.6675711 |
| YGL131C | SNT2 | 0.33821121 | 0.96815259 | 0.23240857 | 143.0926535 | 225.3399263 |
| YER067C-A | YER067C-A | 0.03765237 | 0.96815259 | 0.23265415 | 127.5620125 | 196.3215267 |
| YNL231C | PDR16 | 0.33712252 | 0.96815259 | 0.2327353 | 116.7529073 | 176.1412099 |
| YLR285W | NNT1 | 0.1888183 | 0.96815259 | 0.23281122 | 131.7115905 | 204.0349759 |
| YLL045C | RPL8B | 0.03188633 | 0.9284317 | 0.23282476 | 112.5296422 | 168.2461416 |
| YLR193C | UPS1 | 0.13586043 | 0.96815259 | 0.23311356 | 105.9800098 | 155.9752384 |
| YJL022W | YJL022W | 0.30071533 | 0.96815259 | 0.23314176 | 141.105191 | 221.5007627 |
| YGR106C | YGR106C | 0.29030172 | 0.96815259 | 0.23334886 | 136.134069 | 212.1894057 |
| YGL014W | PUF4 | 0.19913509 | 0.96815259 | 0.2333787 | 131.658583 | 203.834464 |
| YMR173W | DDR48 | 0.25305234 | 0.96815259 | 0.23359061 | 127.9847922 | 196.9425832 |
| YGL058W | RAD6 | 0.36679166 | 0.96815259 | 0.23398425 | 146.4011814 | 231.230252 |
| YER039C | HVG1 | 0.15451093 | 0.96815259 | 0.23424283 | 129.9115235 | 200.4203562 |
| YEL015W | EDC3 | 0.12935437 | 0.96815259 | 0.23436067 | 130.8479552 | 202.1462877 |
| YOR042W | CUE5 | 0.32292062 | 0.96815259 | 0.23474897 | 135.3116018 | 210.4042663 |
| YOR184W | SER1 | 0.30981196 | 0.96815259 | 0.23480743 | 124.8339507 | 190.8463821 |
| YNL311C | SKP2 | 0.23069439 | 0.96815259 | 0.23486164 | 125.0073342 | 191.1601445 |
| YIL020C | HIS6 | 0.34971791 | 0.96815259 | 0.23496337 | 122.4872217 | 186.4403297 |
| YDR425W | SNX41 | 0.18372205 | 0.96815259 | 0.2350474 | 127.756851 | 196.2564593 |
| YDR158W | HOM2 | 0.01041969 | 0.52637819 | 0.23510202 | 113.1857096 | 169.0623272 |
| YDR043C | NRG1 | 0.24805365 | 0.96815259 | 0.23512767 | 122.0323941 | 185.5623687 |
| YGR085C | RPL11B | 0.24378172 | 0.96815259 | 0.23526521 | 101.9963953 | 148.1579887 |
| YBL101C | ECM21 | 0.12092614 | 0.96815259 | 0.2357144 | 141.9167163 | 222.5540797 |
| YFR007W | YFR007W | 0.26041333 | 0.96815259 | 0.23573161 | 134.9976987 | 209.6426742 |
| YBL011W | SCT1 | 0.32155265 | 0.96815259 | 0.23585609 | 130.3846606 | 201.0141639 |
| YBL054W | YBL054W | 0.30158327 | 0.96815259 | 0.23615878 | 150.7883789 | 239.0257389 |
| YNL100W | YNL100W | 0.22028682 | 0.96815259 | 0.23621253 | 123.622771 | 188.3351553 |
| YAL022C | FUN26 | 0.13591358 | 0.96815259 | 0.2363149 | 137.9870812 | 215.1153039 |
| YPR151C | SUE1 | 0.27320925 | 0.96815259 | 0.23633551 | 129.8920461 | 200.0092762 |
| YMR100W | MUB1 | 0.16914241 | 0.96815259 | 0.23658256 | 115.4212708 | 172.9679321 |
| YOL080C | REX4 | 0.27012323 | 0.96815259 | 0.23660727 | 130.2187793 | 200.5701751 |
| YNR005C | YNR005C | 0.27470939 | 0.96815259 | 0.23684772 | 118.4700123 | 178.6082708 |
| YPL021W | ECM23 | 0.30988201 | 0.96815259 | 0.23717071 | 130.6985854 | 201.3644193 |
| YAR037W | YAR037W | 0.1973206 | 0.96815259 | 0.23736448 | 150.9597641 | 239.129574 |
| YBR074W | YBR074W | 0.2706443 | 0.96815259 | 0.23768867 | 155.4987257 | 247.5395398 |
| YOR053W | YOR053W | 0.23794285 | 0.96815259 | 0.23787159 | 127.8164268 | 195.8618703 |
| YBR169C | SSE2 | 0.05912945 | 0.96815259 | 0.237892 | 141.1427466 | 220.720192 |
| YGR192C | TDH3 | 0.31000661 | 0.96815259 | 0.23802254 | 132.0637509 | 203.7587734 |
| YIL071C | PCI8 | 0.31360901 | 0.96815259 | 0.23829601 | 146.6257358 | 230.8770708 |
| YER046W-A | YER046W-A | 0.29353316 | 0.96815259 | 0.23883205 | 135.0550117 | 209.1943966 |
| YMR310C | YMR310C | 0.21863266 | 0.96815259 | 0.2389134 | 123.8318591 | 188.2415846 |
| YLR266C | PDR8 | 0.45597417 | 0.96815259 | 0.23899948 | 151.3816369 | 239.6238481 |
| YPR003C | YPR003C | 0.30309819 | 0.96815259 | 0.23938095 | 127.2448739 | 194.5252797 |
| YBR145W | ADH5 | 0.27121372 | 0.96815259 | 0.23951067 | 151.8562731 | 240.4178031 |
| YBR032W | YBR032W | 0.25224502 | 0.96815259 | 0.23952548 | 135.3586329 | 209.6366667 |
| YGR231C | PHB2 | 0.00014259 | 0.0291563 | 0.24015274 | 120.5690604 | 181.9324794 |
| YLR386W | VAC14 | 0.14057189 | 0.96815259 | 0.24036286 | 121.706271 | 184.0164662 |
| YBL016W | FUS3 | 0.09300868 | 0.96815259 | 0.24063767 | 116.5163347 | 174.2847571 |
| YER038W-A | FMP49 | 0.21457827 | 0.96815259 | 0.24088609 | 132.6365326 | 204.3145885 |
| YFL034C-A | RPL22B | 0.04630606 | 0.96815259 | 0.24126693 | 133.4487816 | 205.7617465 |
| YNL042W | BOP3 | 0.36664478 | 0.96815259 | 0.24166626 | 130.1887156 | 199.6081623 |
| YNL286W | CUS2 | 0.23770678 | 0.96815259 | 0.24181464 | 119.0406094 | 178.7833554 |
| YNL046W | YNL046W | 0.35766128 | 0.96815259 | 0.241988 | 141.167547 | 220.0329799 |
| YMR317W | YMR317W | 0.24899196 | 0.96815259 | 0.24200286 | 129.1915808 | 197.687602 |
| YGR168C | YGR168C | 0.2461555 | 0.96815259 | 0.24326525 | 111.5520652 | 164.5527424 |
| YNL104C | LEU4 | 0.0575145 | 0.96815259 | 0.24343012 | 124.848072 | 189.3286422 |
| YPL098C | MGR2 | 0.28455954 | 0.96815259 | 0.24356056 | 129.0816237 | 197.2035217 |
| YMR304C-A | YMR304C-A | 0.28431365 | 0.96815259 | 0.24403586 | 133.2052395 | 204.8115493 |
| YDR424C | DYN2 | 0.23657376 | 0.96815259 | 0.2441095 | 133.3969566 | 205.1560344 |

| | | | | | | |
|---|---|---|---|---|---|---|
| YGL149W | YGL149W | 0.12641405 | 0.96815259 | 0.24416735 | 117.5145866 | 175.5150568 |
| YEL012W | UBC8 | 0.20711403 | 0.96815259 | 0.24428292 | 129.4981837 | 197.851315 |
| YMR063W | RIM9 | 0.29704883 | 0.96815259 | 0.24437738 | 109.5560573 | 160.6297796 |
| YFL056C | AAD6 | 0.25148132 | 0.96815259 | 0.24454379 | 135.2901977 | 208.6103514 |
| YGL148W | ARO2 | 0.03170335 | 0.9284317 | 0.24497858 | 101.818023 | 146.0858164 |
| YGL232W | TAN1 | 0.3488388 | 0.96815259 | 0.24511864 | 148.4741246 | 233.1037354 |
| YER091C | MET6 | 0.13195772 | 0.96815259 | 0.24513294 | 117.5634903 | 175.4333833 |
| YDR033W | MRH1 | 0.3828616 | 0.96815259 | 0.24528134 | 134.368426 | 206.7585922 |
| YNL135C | FPR1 | 0.11886137 | 0.96815259 | 0.24534934 | 112.587852 | 166.1119331 |
| YLR453C | RIF2 | 0.38924589 | 0.96815259 | 0.24589858 | 132.5169445 | 203.1938831 |
| YPL171C | OYE3 | 0.29789797 | 0.96815259 | 0.24642654 | 143.9877135 | 224.4995461 |
| YOR044W | IRC23 | 0.31356939 | 0.96815259 | 0.24657728 | 147.0662841 | 230.2160241 |
| YPL269W | KAR9 | 0.33422218 | 0.96815259 | 0.24672562 | 132.7404586 | 203.4627785 |
| YER180C | ISC10 | 0.18565867 | 0.96815259 | 0.24733452 | 131.8195572 | 201.6356796 |
| YER042W | MXR1 | 0.27440863 | 0.96815259 | 0.24771287 | 131.4942778 | 200.9610777 |
| YMR303C | ADH2 | 0.24776668 | 0.96815259 | 0.24807902 | 135.9926304 | 209.2877686 |
| YER066W | YER066W | 0.23941863 | 0.96815259 | 0.24830132 | 130.048936 | 198.15923 |
| YDR520C | YDR520C | 0.26519511 | 0.96815259 | 0.24838868 | 131.4662161 | 200.7877057 |
| YAL058W | CNE1 | 0.05835467 | 0.96815259 | 0.24839417 | 126.2862261 | 191.1227803 |
| YGR070W | ROM1 | 0.24228705 | 0.96815259 | 0.25000161 | 140.1630919 | 216.7240219 |
| YER139C | YER139C | 0.48387239 | 0.96815259 | 0.25022564 | 105.238084 | 151.526779 |
| YBR027C | YBR027C | 0.15856906 | 0.96815259 | 0.2503946 | 142.7783144 | 221.532685 |
| YMR158C-B | YMR158C-B | 0.36764149 | 0.96815259 | 0.25065534 | 125.6666053 | 189.5618838 |
| YGL077C | HNM1 | 0.30353251 | 0.96815259 | 0.25071754 | 138.3267094 | 213.1698098 |
| YER106W | MAM1 | 0.20035166 | 0.96815259 | 0.25083519 | 128.8782703 | 195.5214556 |
| YJR139C | HOM6 | 0.22889241 | 0.96815259 | 0.25090421 | 105.8920577 | 152.6253394 |
| YER066C-A | YER066C-A | 0.13113523 | 0.96815259 | 0.25131396 | 133.0603295 | 203.2378929 |
| YLR326W | YLR326W | 0.19408874 | 0.96815259 | 0.25141789 | 132.7346377 | 202.6116628 |
| YOR135C | YOR135C | 0.35687272 | 0.96815259 | 0.2517385 | 136.1361464 | 208.900205 |
| YDR446W | ECM11 | 0.08148482 | 0.96815259 | 0.25256634 | 138.8540766 | 213.8226117 |
| YPL263C | KEL3 | 0.31246155 | 0.96815259 | 0.25264994 | 122.7341264 | 183.7337872 |
| YLR174W | IDP2 | 0.25248026 | 0.96815259 | 0.25316082 | 137.7133241 | 211.5879357 |
| YER152C | YER152C | 0.1247447 | 0.96815259 | 0.25317281 | 135.8632212 | 208.1341822 |
| YAL051W | OAF1 | 0.1467986 | 0.96815259 | 0.25326288 | 159.0836812 | 251.4388296 |
| YNL108C | YNL108C | 0.2447252 | 0.96815259 | 0.2535341 | 131.888074 | 200.6533328 |
| YGL252C | RTG2 | 0.40164401 | 0.96815259 | 0.25353689 | 121.5175161 | 181.3052148 |
| YPL201C | YIG1 | 0.32921634 | 0.96815259 | 0.25368842 | 141.633416 | 218.8068972 |
| YEL066W | HPA3 | 0.17754307 | 0.96815259 | 0.25369765 | 134.0729427 | 204.7002018 |
| YBR025C | YBR025C | 0.08230262 | 0.96815259 | 0.25386463 | 118.062736 | 174.8011859 |
| YER091C-A | YER091C-A | 0.06289439 | 0.96815259 | 0.25389959 | 136.8520206 | 209.8487702 |
| YMR186W | HSC82 | 0.27450219 | 0.96815259 | 0.25402825 | 115.557016 | 170.0971403 |
| YNL120C | YNL120C | 0.15888801 | 0.96815259 | 0.25445978 | 107.426157 | 154.8506962 |
| YDR513W | GRX2 | 0.12412933 | 0.96815259 | 0.25584316 | 133.6864165 | 203.5948847 |
| YPR045C | MNI2 | 0.13898148 | 0.96815259 | 0.2559407 | 105.6284907 | 151.2317237 |
| YOR061W | CKA2 | 0.24443118 | 0.96815259 | 0.25608314 | 119.45038 | 176.9927421 |
| YER087C-A | YER087C-A | 0.17485843 | 0.96815259 | 0.25626217 | 139.5889605 | 214.5318126 |
| YER089C | PTC2 | 0.25975848 | 0.96815259 | 0.25679381 | 131.7767277 | 199.8618787 |
| YML129C | COX14 | 0.37258076 | 0.96815259 | 0.25763186 | 86.10084143 | 114.4975249 |
| YLR435W | TSR2 | 0.05526937 | 0.96815259 | 0.25818233 | 106.9708178 | 153.3345957 |
| YPR132W | RPS23B | 0.34668417 | 0.96815259 | 0.25833825 | 128.9492382 | 194.3102647 |
| YMR044W | IOC4 | 0.171877 | 0.96815259 | 0.2587978 | 130.6934809 | 197.4820824 |
| YAL040C | CLN3 | 0.18732896 | 0.96815259 | 0.25918216 | 148.1431567 | 229.9678845 |
| YER028C | MIG3 | 0.0390285 | 0.96815259 | 0.25947612 | 139.2000423 | 213.2307059 |
| YNR045W | PET494 | 0.28292134 | 0.96815259 | 0.25949124 | 133.6259427 | 202.8287927 |
| YDR435C | PPM1 | 0.18154049 | 0.96815259 | 0.25976552 | 113.5430518 | 165.3124436 |
| YJR019C | TES1 | 0.25818007 | 0.96815259 | 0.25984362 | 137.5385858 | 210.0652342 |
| YGR127W | YGR127W | 0.31828305 | 0.96815259 | 0.2603294 | 154.662109 | 241.9243947 |
| YBR195C | MSI1 | 0.22460041 | 0.96815259 | 0.26117805 | 136.2670494 | 207.4540592 |
| YOL109W | ZEO1 | 0.25702201 | 0.96815259 | 0.26126912 | 122.3062018 | 181.3919817 |
| YGL036W | YGL036W | 0.1896125 | 0.96815259 | 0.26151587 | 127.7296575 | 191.465955 |
| YPR002W | PDH1 | 0.30571057 | 0.96815259 | 0.26154007 | 131.2152615 | 197.9644671 |
| YOR180C | DCI1 | 0.31144286 | 0.96815259 | 0.26205951 | 127.8241511 | 191.5448934 |
| YAL064C-A | YAL064C-A | 0.07133931 | 0.96815259 | 0.26210233 | 142.5585344 | 219.0261257 |
| YGL147C | RPL9A | 0.15979405 | 0.96815259 | 0.26253722 | 106.5322228 | 151.7364994 |

| | | | | | | |
|---|---|---|---|---|---|---|
| YOR379C | YOR379C | 0.31278245 | 0.96815259 | 0.26394527 | 139.1566321 | 212.3494164 |
| YMR265C | YMR265C | 0.20433265 | 0.96815259 | 0.26455048 | 129.5867364 | 194.3871608 |
| YBL039C | URA7 | 0.04418179 | 0.96815259 | 0.26465257 | 127.6199239 | 190.6995354 |
| YBR205W | KTR3 | 0.06639796 | 0.96815259 | 0.2649798 | 138.1366508 | 210.2612547 |
| YLR017W | MEU1 | 0.24488984 | 0.96815259 | 0.26500579 | 146.0880711 | 225.0910042 |
| YEL039C | CYC7 | 0.22960398 | 0.96815259 | 0.2652203 | 123.7766857 | 183.4278123 |
| YOL063C | CRT10 | 0.22087141 | 0.96815259 | 0.26594661 | 131.104362 | 196.9684796 |
| YGR273C | YGR273C | 0.33416608 | 0.96815259 | 0.2660711 | 138.2797329 | 210.3327715 |
| YEL041W | YEF1 | 0.0847851 | 0.96815259 | 0.2665682 | 136.6730228 | 207.246228 |
| YOR090C | PTC5 | 0.23801833 | 0.96815259 | 0.26685822 | 130.5361861 | 195.745229 |
| YEL056W | HAT2 | 0.14225985 | 0.96815259 | 0.26783186 | 134.208914 | 202.4228265 |
| YNL070W | TOM7 | 0.15195164 | 0.96815259 | 0.26804437 | 102.4664435 | 143.1650826 |
| YEL025C | YEL025C | 0.12414646 | 0.96815259 | 0.26826671 | 144.564119 | 221.6639329 |
| YPR106W | ISR1 | 0.26580379 | 0.96815259 | 0.26899205 | 127.4490731 | 189.6037108 |
| YPL027W | SMA1 | 0.3152392 | 0.96815259 | 0.2690007 | 151.8748905 | 235.1716878 |
| YER060W | FCY21 | 0.09892701 | 0.96815259 | 0.26906142 | 131.9883413 | 198.0598812 |
| YOR083W | WHI5 | 0.33509852 | 0.96815259 | 0.2690828 | 133.8488766 | 201.5271223 |
| YLR261C | YLR261C | 0.02594905 | 0.87038384 | 0.26936281 | 106.6295846 | 150.6958658 |
| YER132C | PMD1 | 0.13324787 | 0.96815259 | 0.26937329 | 139.8906246 | 212.7467669 |
| YAL054C | ACS1 | 0.17022588 | 0.96815259 | 0.26948758 | 167.1067494 | 263.5015065 |
| YER166W | DNF1 | 0.14771371 | 0.96815259 | 0.26955544 | 133.1522982 | 200.1429287 |
| YDL061C | RPS29B | 0.06569946 | 0.96815259 | 0.26991965 | 96.48148835 | 131.6635627 |
| YNL217W | YNL217W | 0.10476863 | 0.96815259 | 0.27007432 | 134.9993788 | 203.4959798 |
| YPR098C | YPR098C | 0.29449656 | 0.96815259 | 0.27187291 | 129.8685754 | 193.6017236 |
| YOL070C | NBA1 | 0.22876651 | 0.96815259 | 0.27229003 | 143.2808606 | 218.549384 |
| YGL032C | AGA2 | 0.30023663 | 0.96815259 | 0.27366296 | 150.0841364 | 230.9959222 |
| YOL083W | YOL083W | 0.25018972 | 0.96815259 | 0.27416852 | 133.3863567 | 199.753519 |
| YLR342W | FKS1 | 0.12669471 | 0.96815259 | 0.27448267 | 108.2695628 | 152.8386327 |
| YBR116C | YBR116C | 0.19196245 | 0.96815259 | 0.27502275 | 144.72547 | 220.7551343 |
| YER123W | YCK3 | 0.17021675 | 0.96815259 | 0.27506583 | 125.2302641 | 184.3765893 |
| YFL036W | RPO41 | 0.07037204 | 0.96815259 | 0.27571931 | 111.8906624 | 159.3728144 |
| YPL184C | MRN1 | 0.30854479 | 0.96815259 | 0.27578769 | 144.997016 | 221.1247601 |
| YPR015C | YPR015C | 0.32772434 | 0.96815259 | 0.27580434 | 140.5426928 | 212.8116604 |
| YAL030W | SNC1 | 0.27055434 | 0.96815259 | 0.2761019 | 149.8463951 | 230.1156386 |
| YGL114W | YGL114W | 0.23566715 | 0.96815259 | 0.27615769 | 145.7645827 | 222.490497 |
| YPL053C | KTR6 | 0.30033816 | 0.96815259 | 0.27621704 | 147.9065311 | 226.4759521 |
| YGR141W | VPS62 | 0.27459265 | 0.96815259 | 0.27669243 | 147.5927689 | 225.8054594 |
| YER158C | YER158C | 0.1349898 | 0.96815259 | 0.27841022 | 130.5715162 | 193.7424983 |
| YGL057C | YGL057C | 0.18910841 | 0.96815259 | 0.27882923 | 138.5510838 | 208.554383 |
| YNL278W | CAF120 | 0.20318984 | 0.96815259 | 0.27957963 | 121.2908221 | 176.2187526 |
| YBR168W | PEX32 | 0.15336908 | 0.96815259 | 0.27990466 | 127.9413402 | 188.5679511 |
| YDL183C | YDL183C | 0.15989701 | 0.96815259 | 0.28006458 | 109.9246836 | 154.9269092 |
| YHR152W | SPO12 | 0.30623527 | 0.96815259 | 0.28033261 | 142.6276737 | 215.8905756 |
| YNL045W | YNL045W | 0.15269582 | 0.96815259 | 0.28089596 | 115.3262327 | 164.8553204 |
| YER060W-A | FCY22 | 0.11626541 | 0.96815259 | 0.28113953 | 118.0302246 | 169.8563517 |
| YNL016W | PUB1 | 0.17017965 | 0.96815259 | 0.28115217 | 118.5104519 | 170.7500142 |
| YGR121C | MEP1 | 0.23240881 | 0.96815259 | 0.28232782 | 137.9681711 | 206.8403821 |
| YAR030C | YAR030C | 0.16576983 | 0.96815259 | 0.28434139 | 148.0667217 | 225.3199624 |
| YEL067C | YEL067C | 0.09987529 | 0.96815259 | 0.28543873 | 134.7330352 | 200.2477395 |
| YDR514C | YDR514C | 0.05879008 | 0.96815259 | 0.28555477 | 134.7633249 | 200.2834699 |
| YER024W | YAT2 | 0.16477452 | 0.96815259 | 0.28577312 | 151.9907857 | 232.3844275 |
| YER128W | YER128W | 0.16306641 | 0.96815259 | 0.28589625 | 132.787736 | 196.5366025 |
| YPL273W | SAM4 | 0.18608505 | 0.96815259 | 0.28598605 | 128.309715 | 188.1661937 |
| YDR241W | BUD26 | 0.07606244 | 0.96815259 | 0.28604782 | 118.7398589 | 170.3013262 |
| YBR093C | PHO5 | 0.02989876 | 0.91703766 | 0.28616164 | 113.0689497 | 159.7011295 |
| YPL214C | THI6 | 0.31570111 | 0.96815259 | 0.28653271 | 139.0724547 | 208.1475878 |
| YER142C | MAG1 | 0.13792173 | 0.96815259 | 0.28654409 | 134.086993 | 198.844525 |
| YAL014C | SYN8 | 0.10878144 | 0.96815259 | 0.28707069 | 150.4645253 | 229.3046321 |
| YER063W | THO1 | 0.20904905 | 0.96815259 | 0.28742072 | 139.5870598 | 208.9486308 |
| YGR039W | YGR039W | 0.30188891 | 0.96815259 | 0.28742101 | 150.9148669 | 230.0820715 |
| YMR179W | SPT21 | 0.18967625 | 0.96815259 | 0.28767375 | 111.6701341 | 156.8206801 |
| YAL061W | YAL061W | 0.29636193 | 0.96815259 | 0.2876796 | 154.1311207 | 236.0361029 |
| YLR138W | NHA1 | 0.16668791 | 0.96815259 | 0.28771624 | 104.1114333 | 142.7113372 |
| YDR436W | PPZ2 | 0.02559252 | 0.87038384 | 0.28811973 | 142.0980797 | 213.5080919 |

| | | | | | | |
|---|---|---|---|---|---|---|
| YNL226W | YNL226W | 0.13922635 | 0.96815259 | 0.28861741 | 103.427895 | 141.2747323 |
| YBR157C | ICS2 | 0.12717531 | 0.96815259 | 0.29044986 | 157.6513387 | 242.1074478 |
| YFL054C | YFL054C | 0.15075272 | 0.96815259 | 0.29159659 | 139.6338579 | 208.288156 |
| YOR171C | LCB4 | 0.24285491 | 0.96815259 | 0.29190066 | 141.5329834 | 211.7767709 |
| YAL010C | MDM10 | 0.39369332 | 0.96815259 | 0.29198623 | 42.98774726 | 27.91254735 |
| YGR025W | YGR025W | 0.25983683 | 0.96815259 | 0.29252159 | 139.9418484 | 208.6971107 |
| YPR057W | BRR1 | 0.08585385 | 0.96815259 | 0.29279279 | 99.93698919 | 134.0142998 |
| YOR276W | CAF20 | 0.2117622 | 0.96815259 | 0.2930357 | 137.1690336 | 203.4320028 |
| YBR159W | IFA38 | 0.09167841 | 0.96815259 | 0.29376252 | 142.02183 | 212.3553702 |
| YOR068C | VAM10 | 0.10086431 | 0.96815259 | 0.29530151 | 107.4694031 | 147.617751 |
| YLR087C | CSF1 | 0.10408299 | 0.96815259 | 0.29608774 | 125.9980073 | 182.0444699 |
| YER181C | YER181C | 0.15102142 | 0.96815259 | 0.29733576 | 129.3550643 | 188.0840095 |
| YOL058W | ARG1 | 0.21827152 | 0.96815259 | 0.29861883 | 140.8700801 | 209.3369994 |
| YLR400W | YLR400W | 0.20128576 | 0.96815259 | 0.29943573 | 130.9559391 | 190.6946013 |
| YIL131C | FKH1 | 0.25122227 | 0.96815259 | 0.29944307 | 138.7873795 | 205.3038538 |
| YHR203C | RPS4B | 0.10392582 | 0.96815259 | 0.29963151 | 117.9498993 | 166.3950922 |
| YJL199C | MBB1 | 0.23727607 | 0.96815259 | 0.29992505 | 148.5136891 | 223.3632338 |
| YLR278C | YLR278C | 0.14023251 | 0.96815259 | 0.30045833 | 138.2967484 | 204.2067138 |
| YGL198W | YIP4 | 0.24190398 | 0.96815259 | 0.30146648 | 145.9458663 | 218.2966013 |
| YBR162C | TOS1 | 0.24766528 | 0.96815259 | 0.30150172 | 148.7427243 | 223.5081916 |
| YDR497C | ITR1 | 0.20425114 | 0.96815259 | 0.30169567 | 141.2504327 | 209.4956195 |
| YIL014W | MNT3 | 0.3844074 | 0.96815259 | 0.30190193 | 157.6238005 | 240.0053219 |
| YLR366W | YLR366W | 0.08005117 | 0.96815259 | 0.3020022 | 126.9535516 | 182.7680455 |
| YOR001W | RRP6 | 0.2229766 | 0.96815259 | 0.30306439 | 132.0937813 | 192.1676017 |
| YDR515W | SLF1 | 0.21551811 | 0.96815259 | 0.30358942 | 133.6839434 | 195.0402361 |
| YBL079W | NUP170 | 0.07182898 | 0.96815259 | 0.3039899 | 120.1906929 | 169.7951144 |
| YHR156C | LIN1 | 0.14651506 | 0.96815259 | 0.30463332 | 136.4796438 | 200.0690437 |
| YGL257C | MNT2 | 0.29073513 | 0.96815259 | 0.3047389 | 158.3360504 | 240.826095 |
| YMR230W | RPS10B | 0.12929651 | 0.96815259 | 0.30817818 | 107.9103043 | 146.134454 |
| YML026C | RPS18B | 0.07284351 | 0.96815259 | 0.30834128 | 112.9147671 | 155.4417215 |
| YER047C | SAP1 | 0.13627201 | 0.96815259 | 0.30855812 | 146.4386033 | 217.9459497 |
| YHR078W | YHR078W | 0.32912643 | 0.96815259 | 0.30966459 | 153.5139744 | 230.9478322 |
| YEL006W | YEA6 | 0.14435107 | 0.96815259 | 0.31046008 | 147.723919 | 220.0032836 |
| YIR020C | YIR020C | 0.23955742 | 0.96815259 | 0.31055434 | 145.2567786 | 215.3836341 |
| YFR044C | DUG1 | 0.19512083 | 0.96815259 | 0.31088926 | 141.0565864 | 207.487657 |
| YER049W | TPA1 | 0.15436519 | 0.96815259 | 0.31140061 | 142.7953676 | 210.64001 |
| YER032W | FIR1 | 0.12141509 | 0.96815259 | 0.31162684 | 140.6715762 | 206.6372898 |
| YGR136W | LSB1 | 0.14340159 | 0.96815259 | 0.31188295 | 151.5512014 | 226.8887777 |
| YHL029C | OCA5 | 0.27428285 | 0.96815259 | 0.31315195 | 152.6955754 | 228.7965133 |
| YOR045W | TOM6 | 0.17203464 | 0.96815259 | 0.31437896 | 137.0032714 | 199.3007635 |
| YFR031C-A | RPL2A | 0.15961385 | 0.96815259 | 0.31446702 | 122.5561145 | 172.3319518 |
| YAR018C | KIN3 | 0.0493192 | 0.96815259 | 0.31473671 | 152.0035291 | 227.221625 |
| YGL194C | HOS2 | 0.03921692 | 0.96815259 | 0.31474311 | 138.3934071 | 201.8290337 |
| YNL165W | YNL165W | 0.17747379 | 0.96815259 | 0.31563427 | 131.6254311 | 189.042915 |
| YFL055W | AGP3 | 0.12829982 | 0.96815259 | 0.31603507 | 144.6608448 | 213.290395 |
| YHR189W | PTH1 | 0.17859255 | 0.96815259 | 0.31658156 | 123.9039036 | 174.4677721 |
| YGR276C | RNH70 | 0.16582891 | 0.96815259 | 0.31704578 | 133.2864765 | 191.8890487 |
| YDR458C | HEH2 | 0.1466045 | 0.96815259 | 0.31860306 | 141.2480981 | 206.4636184 |
| YJL117W | PHO86 | 0.24106906 | 0.96815259 | 0.31967742 | 134.330439 | 193.3654414 |
| YPR156C | TPO3 | 0.22112746 | 0.96815259 | 0.31973162 | 137.8631545 | 199.9464739 |
| YML102W | CAC2 | 0.13612285 | 0.96815259 | 0.32081358 | 116.3088151 | 159.5403138 |
| YBL107C | YBL107C | 0.09858611 | 0.96815259 | 0.32117115 | 143.9160338 | 210.9811245 |
| YMR278W | YMR278W | 0.15792401 | 0.96815259 | 0.32220006 | 137.8844083 | 199.5440955 |
| YEL061C | CIN8 | 0.05722926 | 0.96815259 | 0.32238669 | 117.1807638 | 160.8853452 |
| YDR501W | PLM2 | 0.06834404 | 0.96815259 | 0.32337714 | 146.3844829 | 215.1913033 |
| YER097W | YER097W | 0.16768589 | 0.96815259 | 0.32344673 | 140.3751139 | 203.9675859 |
| YAR043C | YAR043C | 0.19424138 | 0.96815259 | 0.32428828 | 157.4703433 | 235.7102519 |
| YHR034C | PIH1 | 0.18185286 | 0.96815259 | 0.3250356 | 117.2010853 | 160.4489108 |
| YEL047C | YEL047C | 0.16828175 | 0.96815259 | 0.32651656 | 136.4825938 | 196.1558634 |
| YLR434C | YLR434C | 0.28571073 | 0.96815259 | 0.32673224 | 124.1803836 | 173.1658759 |
| YDL130W | RPP1B | 0.09217704 | 0.96815259 | 0.32767919 | 116.8855941 | 159.3869284 |
| YGR214W | RPS0A | 0.11157167 | 0.96815259 | 0.3278491 | 122.181038 | 169.2358379 |
| YNL090W | RHO2 | 0.06326476 | 0.96815259 | 0.32798628 | 131.2445763 | 186.1204782 |
| YOL030W | GAS5 | 0.15782092 | 0.96815259 | 0.32826091 | 139.147579 | 200.815374 |

| | | | | | | |
|---|---|---|---|---|---|---|
| YGL174W | BUD13 | 0.04685266 | 0.96815259 | 0.32827841 | 96.77827221 | 121.7668108 |
| YDL083C | RPS16B | 0.085125 | 0.96815259 | 0.32829007 | 106.6605614 | 140.2014137 |
| YBR031W | RPL4A | 0.05505607 | 0.96815259 | 0.32846005 | 128.8412858 | 181.5519884 |
| YLR371W | ROM2 | 0.01654455 | 0.72492154 | 0.33020047 | 97.8731719 | 123.4653003 |
| YDR535C | YDR535C | 0.08548805 | 0.96815259 | 0.3306696 | 142.8845366 | 207.355822 |
| YER182W | FMP10 | 0.08530781 | 0.96815259 | 0.3308502 | 140.0231515 | 201.9851967 |
| YNL081C | SWS2 | 0.28755034 | 0.96815259 | 0.33161409 | 96.46793518 | 120.5905089 |
| YGL034C | YGL034C | 0.24291966 | 0.96815259 | 0.3321913 | 152.729361 | 225.4501229 |
| YNL122C | YNL122C | 0.1496815 | 0.96815259 | 0.33233303 | 129.9436815 | 182.9151061 |
| YFR046C | CNN1 | 0.19030157 | 0.96815259 | 0.33285107 | 152.4481788 | 224.8073949 |
| YBL087C | RPL23A | 0.01985519 | 0.78054746 | 0.33312702 | 116.8119616 | 158.2740004 |
| YDR467C | YDR467C | 0.13065343 | 0.96815259 | 0.33407974 | 146.9169779 | 214.268203 |
| YPR191W | QCR2 | 0.20911614 | 0.96815259 | 0.33437934 | 84.8494242 | 98.41949236 |
| YPL258C | THI21 | 0.25693468 | 0.96815259 | 0.33700369 | 149.0701218 | 217.7615733 |
| YGR069W | YGR069W | 0.19896159 | 0.96815259 | 0.33987821 | 146.2647896 | 212.0131145 |
| YLR213C | CRR1 | 0.15517407 | 0.96815259 | 0.34430915 | 143.7562628 | 206.5396742 |
| YBR204C | YBR204C | 0.11171893 | 0.96815259 | 0.34477036 | 155.5063885 | 228.3784659 |
| YBR056W | YBR056W | 0.19050547 | 0.96815259 | 0.34506243 | 169.2694381 | 254.0029149 |
| YMR183C | SSO2 | 0.12973459 | 0.96815259 | 0.34538773 | 122.161553 | 166.0588016 |
| YDR474C | YDR474C | 0.11114306 | 0.96815259 | 0.34577656 | 146.6201763 | 211.6199027 |
| YGR152C | RSR1 | 0.2503147 | 0.96815259 | 0.34600311 | 163.7644398 | 243.5641791 |
| YOR315W | SFG1 | 0.23442361 | 0.96815259 | 0.3483949 | 157.3779493 | 231.2210502 |
| YAL043C-A | YAL043C-A | 0.07393726 | 0.96815259 | 0.34946344 | 143.5177941 | 205.1717884 |
| YGL261C | PAU11 | 0.23836185 | 0.96815259 | 0.35028701 | 138.0740082 | 194.8682234 |
| YDR083W | RRP8 | 0.00641601 | 0.36400266 | 0.35272056 | 115.2049993 | 151.7673429 |
| YGL181W | GTS1 | 0.20684442 | 0.96815259 | 0.35289245 | 152.6599971 | 221.6137115 |
| YAR050W | FLO1 | 0.0974275 | 0.96815259 | 0.35326477 | 165.4410963 | 245.3918349 |
| YGR081C | SLX9 | 0.11001054 | 0.96815259 | 0.35437599 | 113.6986015 | 148.6605216 |
| YDR471W | RPL27B | 0.12881743 | 0.96815259 | 0.35574289 | 126.8742003 | 172.9965331 |
| YHR132W-A | IGO2 | 0.24246737 | 0.96815259 | 0.35658487 | 148.5136434 | 213.216942 |
| YGR035C | YGR035C | 0.15157637 | 0.96815259 | 0.35701336 | 149.5946907 | 215.1570451 |
| YOR173W | DCS2 | 0.47573036 | 0.96815259 | 0.35713018 | 103.4627941 | 129.0710972 |
| YNL080C | EOS1 | 0.07509262 | 0.96815259 | 0.35785175 | 113.1971378 | 147.1025634 |
| YLR099C | ICT1 | 0.10705556 | 0.96815259 | 0.35947218 | 130.6273793 | 179.3307621 |
| YBR065C | ECM2 | 0.12777647 | 0.96815259 | 0.36136992 | 150.8687339 | 216.7537971 |
| YER011W | TIR1 | 0.17026805 | 0.96815259 | 0.3623429 | 154.4584963 | 223.2767311 |
| YDR536W | STL1 | 0.14170328 | 0.96815259 | 0.3644159 | 145.132653 | 205.5069445 |
| YDL182W | LYS20 | 0.05775453 | 0.96815259 | 0.36593486 | 121.9942732 | 162.0672969 |
| YMR143W | RPS16A | 0.01177487 | 0.57456009 | 0.36761472 | 100.2687824 | 121.2347645 |
| YOL095C | HMI1 | 0.16233477 | 0.96815259 | 0.37045744 | 93.35036152 | 107.8185013 |
| YGL215W | CLG1 | 0.20475502 | 0.96815259 | 0.37634291 | 156.2298256 | 224.0743608 |
| YLR318W | EST2 | 0.28228475 | 0.96815259 | 0.37658634 | 57.12311988 | 39.1343737 |
| YLR013W | GAT3 | 0.17232655 | 0.96815259 | 0.38032931 | 153.9950814 | 219.1913007 |
| YDR530C | APA2 | 0.12180976 | 0.96815259 | 0.38058598 | 148.9188796 | 209.6750266 |
| YGR177C | ATF2 | 0.28268755 | 0.96815259 | 0.38686533 | 157.3467952 | 224.2739369 |
| YGL031C | RPL24A | 0.07009256 | 0.96815259 | 0.38976296 | 122.0540197 | 157.911809 |
| YER093C-A | YER093C-A | 0.09783541 | 0.96815259 | 0.39417893 | 132.8902738 | 177.3374654 |
| YLR048W | RPS0B | 0.13341849 | 0.96815259 | 0.39612335 | 69.53948406 | 58.80017081 |
| YAL020C | ATS1 | 0.05369233 | 0.96815259 | 0.40048676 | 148.0248047 | 204.4433341 |
| YBR176W | ECM31 | 0.12984641 | 0.96815259 | 0.40131325 | 155.7790368 | 218.7618569 |
| YDR447C | RPS17B | 0.05197602 | 0.96815259 | 0.40486978 | 131.4437798 | 172.7244077 |
| YBL071C | YBL071C | 0.03217732 | 0.9284317 | 0.40529235 | 148.7294399 | 204.8973752 |
| YDR127W | ARO1 | 0.09067803 | 0.96815259 | 0.40606515 | 122.7182723 | 156.2317848 |
| YER156C | YER156C | 0.06608314 | 0.96815259 | 0.40710336 | 146.0652725 | 199.6027231 |
| YER046W | SPO73 | 0.07819858 | 0.96815259 | 0.41137491 | 146.9897657 | 200.5625684 |
| YBR106W | PHO88 | 0.00836517 | 0.45468389 | 0.41203463 | 116.0628337 | 142.7462346 |
| YBR103W | SIF2 | 0.04856238 | 0.96815259 | 0.41622924 | 136.1065968 | 179.389331 |
| YCR028C-A | RIM1 | 0.15231119 | 0.96815259 | 0.41653687 | 100.2765098 | 112.4885759 |
| YBR048W | RPS11B | 0.02059033 | 0.78250728 | 0.42396135 | 123.8180457 | 155.0788399 |
| YER119C | AVT6 | 0.04187511 | 0.96815259 | 0.42423267 | 150.8582954 | 205.4773427 |
| YOL008W | COQ10 | 0.13818205 | 0.96815259 | 0.42482938 | 129.3461659 | 165.2368244 |
| YAR002C-A | ERP1 | 0.08027524 | 0.96815259 | 0.4315476 | 158.9101049 | 219.1891337 |
| YLR061W | RPL22A | 0.01633491 | 0.72492154 | 0.4350624 | 48.28829571 | 12.1804188 |
| YGR058W | YGR058W | 0.02584341 | 0.87038384 | 0.43776272 | 147.6254298 | 197.0231543 |

| | | | | | | |
|---|---|---|---|---|---|---|
| YGL126W | SCS3 | 0.16976314 | 0.96815259 | 0.440484 | 159.3481699 | 218.406138 |
| YEL062W | NPR2 | 0.04064967 | 0.96815259 | 0.4407381 | 139.3761223 | 181.1001955 |
| YGL086W | MAD1 | 0.15628751 | 0.96815259 | 0.442314 | 159.7245031 | 218.7805349 |
| YIL134W | FLX1 | 0.55477426 | 0.96815259 | 0.44727232 | 69.41734349 | 49.41293443 |
| YBL096C | YBL096C | 0.09489121 | 0.96815259 | 0.45375025 | 163.6844224 | 224.1203608 |
| YGL176C | YGL176C | 0.17189436 | 0.96815259 | 0.45430911 | 163.3240159 | 223.3478987 |
| YGR111W | YGR111W | 0.01972611 | 0.78054746 | 0.4558409 | 133.6805452 | 167.7698641 |
| YOR182C | RPS30B | 0.10024731 | 0.96815259 | 0.46338134 | 119.1203324 | 139.255618 |
| YGL043W | DST1 | 0.09195429 | 0.96815259 | 0.47038802 | 153.0498284 | 201.300783 |
| YER153C | PET122 | 0.12627206 | 0.96815259 | 0.4723641 | 82.92141434 | 70.11328281 |
| YGL084C | GUP1 | 0.04769603 | 0.96815259 | 0.47926995 | 145.866607 | 186.309046 |
| YGL078C | DBP3 | 0.05340443 | 0.96815259 | 0.4933548 | 130.0319999 | 154.2453285 |
| YOR293W | RPS10A | 0.01011036 | 0.51706848 | 0.50832846 | 48.79188263 | 0 |
| YOR183W | FYV12 | 0.04827496 | 0.96815259 | 0.50967802 | 126.6930066 | 145.0929661 |
| YGL007W | YGL007W | 0.06991947 | 0.96815259 | 0.50981169 | 154.2266896 | 196.4366819 |
| YGR148C | RPL24B | 0.02333727 | 0.82139523 | 0.53630672 | 140.8660394 | 166.7661306 |
| YDL191W | RPL35A | 0.1248389 | 0.96815259 | 0.55689924 | 129.6708274 | 142.1924604 |
| YGL253W | HXK2 | 0.05217243 | 0.96815259 | 0.60254623 | 157.8377791 | 186.5674437 |
| YGR118W | RPS23A | 0.02977811 | 0.91703766 | 0.62246328 | 142.0913061 | 153.6237659 |
| YIL036W | CST6 | 0.06274219 | 0.96815259 | 0.62901793 | 82.08656279 | 40.50338886 |
| YGL219C | MDM34 | 0.03345166 | 0.93883273 | 0.67687782 | 156.1361841 | 170.0821633 |
| YDR500C | RPL37B | 0.01494315 | 0.68995594 | 0.69173007 | 152.9120115 | 161.4074253 |
| YDL062W | YDL062W | 0.0278199 | 0.89818524 | 1.04016232 | 99.83993078 | 0 |
